# Proceedings of the 25th European Paediatric Rheumatology Congress (PReS 2018): Lisbon, Portugal. 5-8 September 2018

**DOI:** 10.1186/s12969-018-0265-6

**Published:** 2018-08-22

## Updates in JIA management

### O01 PREDICTING WHICH CHILDREN WITH JUVENILE IDIOPATHIC ARTHRITIS WILL NOT ATTAIN EARLY REMISSION WITH CONVENTIONAL TREATMENT: RESULTS FROM A CANADIAN INCEPTION COHORT

#### Jaime Guzman Ramirez^1^, Andrew Henrey^2^, Thomas Loughin^2^, Roberta Berard^3^, Natalie J. Shiff^4^, Roman Jurencak^5^, Adam M. Huber^6^, Kiem Oen^7^, Kerstin Gerhold^7^, Brian M. Feldman^8^, Rosie Scuccimarri^9^, Kristin Houghton^10^, Gaelle Chedeville^9^, Kimberley Morishita^10^, Bianca Lang^6^, Paul Dancey^11^, Alan M. Rosenberg^12^, Julie Barsalou^13^, Alessandra Bruns^14^, Karen Watanabe Duffy^5^, Susanne Benseler^15^, Ciaran Duffy^5^, Lori B. Tucker^10^ and ReACCh-Out Investigators

##### ^1^Pediatrics, University of British Columbia, Vancouver; ^2^Statistics, Simon Fraser University, Burnaby; ^3^Pediatrics, Western University, London, Canada; ^4^Pediatrics, University of Florida, Gainesville, USA; ^5^Pediatrics, University of Ottawa, Ottawa; ^6^Pediatrics, Dalhousie University, Halifax; ^7^Pediatrics, University of Manitoba, Winnipeg; ^8^Pediatrics, University of Toronto, Toronto; ^9^Pediatrics, McGill University, Montreal; ^10^Pediatrics, UBC and BC Children's Hospital, Vancouver; ^11^Pediatrics, Memorial University, St. John's; ^12^Pediatrics, University of Saskatchewan, Saskatoon; ^13^Pediatrics, Universite de Montreal, Montreal; ^14^Pediatrics, Universite de Sherbrooke, Sherbrooke; ^15^Pediatrics, University of Calgary, Calgary, Canada

###### **Correspondence:** Jaime Guzman Ramirez

**Introduction:** Accurate estimates of the likelihood of attaining early remission with conventional treatment escalation may help target aggressive treatment to children with juvenile idiopathic arthritis (JIA) who have a low chance of remission.

**Objectives:** To develop a clinical prediction tool to estimate the chance of early clinical remission with conventional treatment for each child at the time of JIA diagnosis.

**Methods:** Prediction models were developed using data from 1074 subjects in the Research in Arthritis in Canadian Children emphasizing Outcomes (ReACCh-Out) cohort diagnosed 2005-2010. Treatment was compatible with the 2011 recommendations of the American College of Rheumatology. The predicted outcome was clinical inactive disease for 6 or more months starting within one year of the diagnosis in patients who did not receive early biologic agents or triple DMARD therapy. Models were developed in 200 random splits of 75% of the cohort, and tested on the remaining 25% of subjects, calculating expected and observed frequencies of remission and c-index values.

**Results:** A Cox-logistic model combining 18 clinical variables at a median of 2 days after diagnosis had a c-index of 0.69 (95%CI: 0.67-0.71) and performed better than JIA category alone (0.59, 0.56-0.63). Table 1 lists the top 8 variables included in the final model. Using the model, an online calculator (https://andrew-j-henrey.shinyapps.io/JIA_Remission_Calc/) produces estimates of the chance of remission for each child with JIA at diagnosis. Children in the lowest decile of probability of remission according to the model had a 20% chance of remission and 21% of them remitted; children in the highest decile had a 69% chance of remission and 73% remitted. Compared to 5% of subjects identified by JIA category alone, the model identified 14% of the cohort as having a low chance of remission (<0.25 probability of remission), of whom 77% failed to attain remission within one year of diagnosis.

**Conclusion:** Although the model did not meet our *a priori* performance threshold (c-index>0.70), it identified three times more subjects with low chance of remission than JIA category alone, and it may serve as a benchmark for judging the value added by future biomarkers.

**Disclosure of Interest**

None Declared

**Table 1 (abstract O01).** The top 8 variables in the final prediction model (full model uses 18 variables)

Variable	Adjusted Hazard Ratio for time to remission on medications (95% CI)	P-value
Physician global (0-10)	0.933 (0.888-0.981)	0.005
Weeks from onset to diagnosis	0.997 (0.995-0.999)	0.003
RF+ Polyarthritis	0.383 (0.150-0.978)	0.041
Systemic arthritis	0.550 (0.308-0.983)	0.039
Subtalar joint involved	0.670 (0.482-0.931)	0.015
Symmetric joints involved	0.777 (0.616-0.979)	0.029
Pain intensity (0-10)	0.927 (0.874-0.983)	0.010
No. enthesitis sites	0.960 (0.921-1.001)	0.050

## New tools and some omics

### O02 TREG SPECIFIC CONSTITUTIVE NRF2 ACTIVATION INDUCES AUTOIMMUNITY

#### Klaus Tenbrock^1^, Patricia Klemm^1^, Kim Ohl^1^

##### ^1^Pediatrics, RWTH AACHEN UNIVERSITY, Aachen, Germany

###### **Correspondence:** Klaus Tenbrock

**Introduction:** Immune cells are constantly confronted with intracellular and extracellular radical oxygen species (ROS) under steady-state and even more under inflammatory and pathogenic conditions. To investigate the effects of oxidative stress and ROS molecules in regulatory T cells (T_regs_), we deciphered the role of Nuclear factor (erythroid-derived 2)-like 2 (Nrf2) in this context. T_regs_ were already found to be more resistant to ROS than effector T cells and activated T_reg_ cells show higher expression of genes, which belong to the Nrf2-mediated oxidative stress response compared to activated effector T cells.

**Objectives:** To analyze the specific role of Nrf2 in T_regs._

**Methods:** To define the role of Nrf2 in Tregs, we generated mice with a knock out of Keap in all hematopoietic cells (VAV^CRE^-Keap^fl/fl^) with a Treg specific knockout of Keap 1, which is the constitutive binding partner of Nrf2 (Foxp3^CRE^-Keap1^fl/fl^) and characterized the mice using flow cytometry, T cell suppression assays and metabolomics.

**Results:** While mice bearing a constitutive activation of Nrf2 activation in all immune cells (Vav^cre^Keap^fl/fl^) accumulate high percentages of Foxp3-positive T_regs_ in the spleen, lymph nodes and thymus, their suppressive capacity seems to be defective. Interestingly, a T_reg_ specific activation of Nrf2 (Foxp3^cre^Keap^fl/fl^) results in an auto-inflammatory phenotype with immune cell infiltrates in the lungs, enhanced effector T cell activation and high percentages of IFN-γ producing effector T cells. Moreover, the constitutive Nrf2 activation in T_regs_ increases their *in-vitro* proliferation, glucose uptake and mTOR activity, while the differentiation and Foxp3 expression in T_regs_ declines

**Conclusion:** We demonstrate for the first time that constitutive Nrf2 activation specific to T_regs_ affects T_reg_ lineage stability and metabolism and might thereby induce an auto-inflammatory phenotype.

Our results therefore may have implications for diseases associated with oxidative stress and dysregulated T_reg_ responses.

**Disclosure of Interest**

None Declared

### O03 EXTENDED PHENOTYPIC IMMUNOME CHARACTERISATION (EPIC): A WEB-BASED IMMUNE REFERENCE ATLAS

#### Joo Guan Yeo^1,2^, Pan Lu^1^, Thaschawee Arkachaisri^1,2^, Su Li Poh^1^, Fauziah Ally^1^, Jingyao Leong^1^, Kee Thai Yeo^1,2^, Loshinidevi D/O Thana Bathi^1^, Angela Yun June Tan^2^, Liyun Lai^1^, Elene Seck Choon Lee^2^, Salvatore Albani^1,2^

##### ^1^Translational Immunology Institute, SingHealth Duke-NUS Academic Medical Centre; ^2^KK Women’s and Children’s Hospital, Singapore, Singapore

###### **Correspondence:** Joo Guan Yeo

**Introduction:** An atlas of the developing immune system is key to understanding its normal maturation and identifying disease-associated cell subsets. The availability of high dimensional mass cytometry, in comparison to traditional oligo-dimensional approach such as fluorescence-based flow cytometry, provides an opportunity for the creation of this reference. However to date, the power available from these big data has not been fully harnessed due to the absence of clinically relevant and standardised datasets. This results in issues of fragmentation by focusing on individual cell subsets and lack the ability to transverse the whole developmental gradient from neonatal to adult age. There is a critical unmet need for standardised datasets depicting at single cell level and with high dimensionality the entire human immune system. These limitations hamper translational and clinical research.

**Objectives:** To address this need, we aim to construct a mass cytometry based immune atlas from healthy peripheral blood mononuclear cells (PBMC) samples ranging from cord blood to adult age and make this dataset available to the research community via an interactive web portal to enable its mining and comparison with diseased dataset.

**Methods:** The mass cytometry data from 113 healthy individuals (cord blood, newborn to adult) using 63 immune markers encompassing the major immune lineages were obtained. Quality control was performed before dimensional reduction and clustering to identify the cell subsets using our in-house analysis and visualisation pipeline. Their frequencies across the ages were presented as 3-D frequency histograms to create the immune landscape. This database and the analytic pipeline were incorporated into a web-based portal allowing users to interact and upload their own data for comparison.

**Results:** Here, we described EPIC with examples of its potential by exploring the evolution of some representative immune subsets over the full gradient of ages. Three developmental trajectories made up the healthy immune landscape with the most distinct being an increase or decrease in the cell populations with age. The last trajectory constituted an increase in population size in early childhood followed by a region of levelling. There was a distinct segregation of the naive T cell subset enriched in the cord blood/newborn period with the memory T cell subset enriched in adulthood. The naive IL8+ and TNFα+ CD4+ T cells were prominent peaks in the landscape and were found in higher frequency in the cord blood/newborn period. In contrast, the memory IFNɣ+ and TNFα+ CD4+ T cells were enriched in adulthood.

For specific cell subsets, transition developmental milestones were observed in the TNFα+ CD4+ T cells where the size of its memory subset would exceed its naive subset at 8 year old. There was a significant reduction and increase with age in the frequency of the naive and memory TNFα+ CD4+ T cells with a Spearman’s correlation coefficient, rho, of -0.4662 and 0.4164 respectively (p<0.0001). A similar intersection was present for the naive and memory regulatory T cell (CD4+, CD25+, Foxp3+, CD152+) subsets at 14 year old with a rho of -0.537 and 0.5034 respectively (p<0.0001).

**Conclusion:** A holistic description of the developing immunome was obtained with key developmental milestones in the T cell compartment identified. This atlas has the translational potential of helping us define the stage of immune maturity and identify the pathological cell subset in both paediatric and adult immune mediated diseases by the direct comparison with this reference atlas using the web-based portal that will be made freely available.

**Disclosure of Interest**

None Declared

## Oral presentation 1

### O04 VALIDATION OF THE RECENTLY DEVELOPED EVIDENCE-BASED EUROFEVER CLASSIFICATION CRITERIA FOR HEREDITARY RECURRENT FEVERS (HRF) AND PFAPA

#### Silvia Federici^1,2^, Federica Vanoni^3,4^, Francesca Bovis^5^, Nicola Ruperto^1^, Michael Hofer^6^, Marco Gattorno^1^ and on behalf of the Expert Committee for the Classification Criteria in periodic fever

##### ^1^Division of Rheumatology, Istituto Giannina Gaslini, Genova; ^2^Division of Rheumatology, IRCCS Ospedale Pediatrico Bambino Gesù, Rome, Italy; ^3^Pediatric Rheumatology Unit CHUV, University of Lausanne, Lausanne; ^4^Departement of Pediatric of Southern Switzerland, Bellinzona, Switzerland; ^5^Biostatistic Unit, Department of Health Sciences, University of Genoa, genoa, Italy; ^6^Pediatric Rheumatology Unit, CHUV, Lausanne, Switzerland

###### **Correspondence:** Silvia Federici

**Introduction:** Provisional Eurofever evidence-based classification criteria for the 4 inherited recurrent fever (FMF, CAPS, TRAPS, MKD) have been published and other diagnostic criteria are available in the literature for FMF, CAPS and PFAPA. Despite a significant increase of accuracy of the recent Eurofever classification criteria in respect to the previous ones, none of them combine the clinical criteria with the results of molecular analysis. This is a major limitation considering the fact that the genetic analysis might be considered per se pathognomonic, and therefore diagnostic, at least in the presence of a confirmatory genotype. Recently new evidence-based classification criteria for hereditary recurrent fevers (HRF) and PFAPA have been identified during a Consensus Conference held in Genoa in March 2017. For each of the monogenic recurrent fever new classification criteria based on genetic and clinical variables were developed. For PFAPA novel classification criteria based on positive and negative clinical variables were also approved by experts (Gattorno et al. PRES 2017, submitted).

**Objectives:** To evaluate the performance of the final set of classification criteria, in discriminating patients with the different HRF and PFAPA in a separate set of real patients coming from the Eurofever Registry and to compare their accuracy with respect to existing criteria.

**Methods:** We selected those patients with recurrent HPF coming from the Eurofever Registry excluding patients belonging to the original dataset of 360 patients used for the development of the criteria themselves. Patients with inherited periodic fever (TRAPS, FMF, MKD and CAPS), PFAPA and undefined periodic fevers were considered and classified according to the indication of each center, without any process of validation by expert. Sensitivity, specificity, accuracy, negative and positive predictive values and AUC-ROC of the new criteria were calculated.

**Results:** A total of 1018 new patients coming from the Eurofever Registry were included. The performance of the criteria coming from the Consensus conference in comparison with the criteria of the literature is listed in Table 1. Overall, their performance was superior (accuracy ranging from 0.81 to 0.98) to the already published literature’s criteria (accuracy 0.56-0.94) with a very high specificity and a variable sensitivity. Most of the patients not classified with the new criteria were negative for genetic analysis or carriers of low-penetrance mutations with an inconsistent clinical phenotype.

**Conclusion:** The validation of the new Eurofever classification criteria in a large group of unselected patients coming from the registry confirms their high specificity and overall better performance in comparison to other criteria available in the literature. It is recommended to use them as classification rather that diagnostic criteria, for clinical trials and pathogenic studies.

**Disclosure of Interest**

None Declared

**Table 1 (abstract O04).** See text for description

	Sensitivity	Specificity	Accuracy	AUC
New CAPS criteria (clinical + genetics)	0.72	**1**	**0.96**	**0.86**
Kuemmerle CAPS criteria	**0.88**	**0.82**	**0.83**	**0.85**
New FMF criteria (clinical + genetics)	**0.89**	**1**	**0.96**	**0.94**
Livneh criteria	0,9	0,58	0,7	0,74
Tel Hashomer	0,59	0,95	0,64	0,77
Pediatric FMF criteria	0,61	0,94	0,83	0,77
New MKD criteria (clinical + genetics)	**0.74**	**1**	**0.98**	**0.87**
Clinical guideline	**0.77**	**0.53**	**0.56**	**0.65**
New TRAPS criteria (clinical + genetics)	**0.74**	**1**	**0.97**	**0.87**
New PFAPA clinical criteria	**0.66**	**0.97**	**0.9**	**0.82**
Modified Marshall criteria	**0.46**	**0.91**	**0.81**	**0.69**

### O05 PERFORMANCE OF THE AUTOINFLAMMATORY DISEASE ACTIVITY INDEX (AIDAI) IN PATIENTS WITH COLCHICINE-RESISTANT FMF, HIDS/MKD AND TRAPS: RESULTS FROM A PIVOTAL, PHASE 3 TRIAL OF CANAKINUMAB

#### Isabelle Kone-Paut^1^, Maryam Piram^1^, S Benseler^2^, J. B Kuemmerle-Deschner^3^, A. F Jansson^4^, I Rosner^5^, A Tomassini^6^, S Murias^7^, O Karadag^8^, J Levy^9^, S Smeets^10^, F De Benedetti^11^

##### ^1^APHP, CHU de Bicêtre, Univ Paris Sud, Le Kremlin Bicêtre, France; ^2^Alberta Children's Hospital, Calgary, Canada; ^3^University Hospital Tuebingen, Tuebingen; ^4^Ludwig Maximilian University, Munich, Germany; ^5^Bnai-Zion Medical Center, Rheumatology, Haifa, Israel; ^6^Department of Internal Medicine, Università Cattolica Sacro Cuore, Rome, Italy; ^7^Hospital La Paz, Madrid, Spain; ^8^Hacettepe University Faculty of Medicine, Ankara, Turkey; ^9^BIOP, Reinach, Switzerland; ^10^Novartis Pharma B.V., Arnhem, Netherlands; ^11^IRCCS Ospedale Bambino Gesú, Rome, Italy

###### **Correspondence:** Isabelle Kone-Paut

**Introduction:** AIDAI is a novel and unique, validated patient (pt)-reported assessment tool to evaluate disease activity in familial Mediterranean fever (FMF), hyper-IgD syndrome/mevalonate kinase deficiency (HIDS/MKD) and TNF receptor-associated periodic syndrome (TRAPS).^1^

**Objectives:** To perform an external validation of AIDAI, we calculated scores over 40 weeks (wks) of canakinumab (CAN) treatment in pts enrolled into the CLUSTER trial and assessed correlation between AIDAI and disease/response characteristics.

**Methods:** CLUSTER consisted of one cohort per disease (crFMF, HIDS/MKD and TRAPS).^2^ AIDAI was calculated as the sum of 12 items (Yes=1; No=0)^1^ for 30 consecutive days. AIDAI score was calculated if the first score was recorded before ≥29 days. Missing items beyond last evaluable measurement were imputed by last observation carried forward (LOCF). Inactive disease (ID) was defined as AIDAI score <9. Correlation analysis of AIDAI with Sheehan disability score (SDS), child health questionnaire–psychological/physical (CHQ–PsCS/PCS), physician global assessment (PGA), short form 12–physical/mental component summaries (SF12–PCS/MCS), C-reactive protein (CRP) and serum amyloid A (SAA) were performed. Significance was set at p<0.05.

**Results:** Overall, 167 (crFMF: N=59; HIDS/MKD: N=66; TRAPS: N=42) pts were randomized to CAN 150 mg or placebo every 4 wks. Median AIDAI scores in all 3 cohorts decreased from baseline (BL) to Wk 16 (crFMF: 22.5 to 5.0; HIDS/MKD: 41.5 to 12.0; TRAPS: 89.0 to 13.0) and through Wk 40 (crFMF: 1.0; HIDS/MKD: 5.0; TRAPS: 20.5). In all 3 cohorts, the proportion of pts with ID (AIDAI score <9) was higher at Wk 40 versus BL (crFMF: 69.5% vs 5.1%; HIDS/MKD: 56.1% vs 6.1%; TRAPS: 42.9% vs 2.4%). AIDAI at Wk 40 correlated significantly with: SDS in all 3 cohorts; CHQ-PsCS in crFMF and HIDS/MKD; CHQ-PCS in crFMF; PGA in TRAPS; SF12–PCS in crFMF and TRAPS. SF12-MCS, CRP and SAA did not correlate with AIDAI (Table 1).

**Conclusion:** AIDAI scores decreased markedly over 40 weeks of treatment with canakinumab in crFMF, HIDS/MKD and TRAPS, with a relevant percentage of patients having inactive disease score. AIDAI improvements at Week 40 correlated with patient- and physician-driven evaluations. AIDAI is a validated patient-reported tool to assess disease activity and appears to have good sensitivity to change to be used in comparative trials. Patient’s experience on disease activity does not strictly correlate with CRP and SAA, as these reflect more closely biological inflammation than clinical symptoms.

^1^Piram M, et al. Ann Rheum Dis. 2014;73:2168-73. ^2^De Benedetti F, et al. N Engl J Med. 2018 (in press).

**Trial registration identifying number:** NCT02059291

**Disclosure of Interest**

I. Kone-Paut Grant / Research Support from: Novartis, SOBI, Roche, Consultant for: Novartis, SOBI, Pfizer, AbbVie, Roche, M. Piram Consultant for: Novartis, Pfizer, Abbvie, Speaker Bureau of: Novartis, S. Benseler Consultant for: Novartis, SOBI, AbbVie, J. B. Kuemmerle-Deschner Grant / Research Support from: Novartis, Consultant for: Novartis, SOBI, A. F. Jansson Grant / Research Support from: Novartis, I. Rosner: None Declared, A. Tomassini: None Declared, S. Murias: None Declared, O. Karadag: None Declared, J. Levy Consultant for: Novartis, S. Smeets Employee of: Novartis, F. De Benedetti Grant/Research Support from: Novartis, Roche, Pfizer, SOBI, AbbVie, Novimmune, BMS, Sanofi

**Table 1 (abstract O05).** Correlation between AIDAI and disease activity variables at Week 40

	Correlation coefficient (95% CI)							
	SDS	CHQ-PsCS	CHQ PCS	PGA	SF12-PCS	SF12-MCS	SAA	CRP
crFMF	0.54* (0.20; 0.76)	-0.83* (-0.95; -0.49)	-0.67* (-0.90; -0.16)	0.24 (-0.08; 0.51)	-0.47* (-0.77; 0.0)	-0.43 (-0.75; 0.05)	-0.07 (-0.38; 0.25)	-0.07 (-0.38; 0.24)
HIDS/MKD	0.39* (0.05; 0.65)	-0.47* (-0.76; -0.03)	-0.32 (-0.67; 0.14)	0.24 (-0.03; 0.47)	-0.09 (-0.71; 0.61)	-0.19 (-0.76; 0.54)	0.06 (-0.22; 0.32)	0.08 (-0.19; 0.34)
TRAPS	0.41* (0.02; 0.69)	-0.07 (-0.62; 0.53)	-0.02 (-0.59; 0.56)	0.37* (0.02; 0.63)	-0.60* (-0.86; -0.11)	-0.13 (-0.62; 0.43)	-0.17 (-0.50; 0.20)	-0.18 (-0.51; 0.18)

*p<0.05

### O06 INTERRATOR RELIABILITY OF A STANDARDIZED SCORING TOOL TO REPORT MRI FINDINGS IN CHRONIC NONBACTERIAL OSTEOMYELITIS

#### Yongdong (Dan) Zhao^1,2^, T. S. Sato^3^, Meinrad Beer^4^, Mingqian Huang^5^, Ramesh S. Iyer^6^, Michael McGuire^7^, Anh-Vu Ngo^6^, Jeffrey P. Otjen^6^, Jyoti Panwar^8^, Jennifer Stimec^9^, Mahesh Thapa^6^, Paolo Toma^10^, Angela Taneja^11^, Nancy E. Gove^2^, Polly J. Ferguson^12^

##### ^1^Seattle Children’s Hospital, Department of Pediatrics, University of Washington; ^2^Center for Clinical and Translational Research, Seattle Children’s Research Institute, Seattle; ^3^Radiology, University of Iowa Carver College of Medicine, Iowa City, USA; ^4^Department of Diagnostic and Interventional Radiology, University Hospital of Ulm, Ulm, Germany; ^5^Department of Radiology, University of Stony Brook, Stony Brook; ^6^Department of Radiology, Seattle Children’s Hospital, University of Washington, Seattle; ^7^Department of Radiology, Hackensack University Medical Center, Hackensack, USA; ^8^Joint Department of Medical Imaging, University Heath Network, University of Toronto; ^9^Hospital for Sick Children, Department of Medical Imaging, Toronto, Canada; ^10^Department of Imaging, Bambino Gesù Children Hospital, IRCCS, Rome, Italy; ^11^Pediatric Rheumatology, Children’s Healthcare of Atlanta, Emory University, Atlanta; ^12^Department of Pediatrics, University of Iowa Carver College of Medicine, Iowa City, USA

###### **Correspondence:** Yongdong (Dan) Zhao

**Introduction:** CNO is an inflammatory bone disease that can result in bone destruction, persistent bone pain and pathological fractures. For clinical and research purposes, serial MRI exams are needed to determine the objective response to treatment. A previously developed MRI scoring tool for CNO in a single center showed sensitivity to detect imaging change after aggressive treatment.

**Objectives:** To use a nominal group technique to develop a practical and consensus-based MRI scoring tool for clinical and research use in CNO. Furthermore, inter-rater reliability were evaluated using whole body (WB) MRI from children with CNO.

**Methods:** Eleven pediatric radiologists, each with at least 5 years of experience reading musculoskeletal MRI from seven different pediatric hospitals in North America and Europe, discussed definitions and grading of signal intensity, extent of signal abnormality within bone marrow and surrounding tissue, physis damage and vertebral fracture on MRI through monthly conference calls and an in-person meeting (Seattle, July 2017). Nine sets of whole body MRIs were used as a pilot reading session at the conference that demonstrated greater than 70% of agreement among the radiologist attendees. Fifty sets of pre-existing WB MRI scans between Jan 2013 and August 2016 from children with CNO at the University of Iowa Children’s Hospital were anonymized and used for an inter-rater reliability study. Inter-rater agreement of presence of abnormal signal and severity were assessed using Fleiss kappa analysis.

**Results:** Signal intensity was rated as absent, < fluid signal or similar to fluid signal within bone marrow. Extent of abnormal signal intensity within bone or surrounding tissues were scored according to the relative affected proportion. Long bones were divided into proximal epiphysis, proximal metaphysis, diaphysis, distal metaphysis and distal epiphysis. Complex bony regions such as the pelvis were divided into easily identifiable anatomical subareas. The agreement among 11 radiologists in readings of femur and tibia were shown in Table 1. There was moderate to substantial agreement (0.44-0.63) in all parameters except the sizes of femoral proximal epiphyseal and femoral proximal metaphyseal lesions (0.28-0.34). There were 38 tibias and 30 femurs identified as active inflammation by at least 9 of 11 radiologists. Data from other bones are being analyzed.

**Conclusion:** A comprehensive standardized scoring tool for MRI in children with CNO was developed. There was moderate to substantial agreements among radiologists in majority of parameters of femur and tibia. If proven reproducible, this tool can be validated in a prospective study and will become a key element of disease activity assessment in CNO.

This study was funded by CARRA-AF small grant.

**Disclosure of Interest**

Y. D. Zhao Grant / Research Support from: Bristol-Myers Squibbs, CARRA, Clinical Research Scholar Program from Seattle Children's Research Institute, T. Sato: None Declared, M. Beer: None Declared, M. Huang: None Declared, R. Iyer: None Declared, M. McGuire: None Declared, A.-V. Ngo: None Declared, J. Otjen: None Declared, J. Panwar: None Declared, J. Stimec: None Declared, M. Thapa: None Declared, P. Toma: None Declared, A. Taneja: None Declared, N. Gove: None Declared, P. Ferguson Grant/Research Support from: R01AR059703 from NIH/NIAMS

**Table 1 (abstract O06).** See text for description

MRI characteristics	Kappa values (95% CI) of tibial lesions (n=100)	Kappa values (95% CI) of femoral lesions (n=100)
Max signal intensity	0.46 (0.44,0.48)	0.48 (0.46,0.5)
Size of proximal epiphyseal lesion	0.46 (0.44,0.49)	0.34 (0.32,0.36)
Size of proximal metaphyseal lesion	0.54 (0.52,0.55)	0.28 (0.25,0.3)
Size of diaphyseal lesion	0.46 (0.44,0.48)	0.44 (0.42,0.46)
Size of distal metaphyseal lesion	0.58 (0.57,0.6)	0.47 (0.46,0.49)
Size of distal epiphyseal lesion	0.48 (0.45,0.5)	0.49 (0.46,0.51)
Soft tissue inflammation	0.6 (0.57,0.63)	0.63 (0.6,0.65)

### O07 IMPACT OF PEDIATRIC RHEUMATIC DISEASES ON CAREGIVERS MULTIASSESSMENT QUESTIONNAIRE: CAREGIVERS QUESTIONNAIRE

#### Marcia D. Torres-Made^1^, Nadina E. Rubio Pérez^1^, Ingris Peláez Ballestas^2^, Fernando García Rodriguez^1^, Ana V. Villarreal Treviño^1^, Brenda D. J. Fortuna Reyna^1^, Manuel E. De la O Cavazos^1^

##### ^1^Pediatría, Hospital Universitario "Dr. José Eleuterio Gónzalez", Monterrey, Nuevo León; ^2^Reumatología, Hospital General Dr. Eduardo Liceaga, Mexico, DF. , Mexico

###### **Correspondence:** Marcia D. Torres-Made

**Introduction:** Most common pediatric rheumatic disease (PRD) is juvenile idiopathic arthritis (JIA), this influence affects quality of life (QoL) of children and families. Caregiver is a cornerstone to achieve disease control however, there are no information on how this conditions affects them; neither a tool to assess this impact was reported.

**Objectives:** To develop and validate psycho-social impact and financial burden questionnaire in caregivers of JIA patients (CAREGIVERS Questionnaire).

**Methods:** We development and validate the CAREGIVERS Questionnaire in two phases. Phase 1.Design the questionnaire based in non-systematic review and qualitative study: First version (1stQ), exploratory and open questions, was constructed based on literature review by an expert group (pediatric rheumatologists and methodologist). A qualitative study of pragmatic type was conducted on 15 caregivers to identify areas affected after JIA diagnosis. Interviews were reviewed using thematic analysis with Atlas-ti software. Questionnaire was adapted based on qualitative study and second version (2ndQ) was tested on 10 participants to evaluate comprehensibility. Changes in structure were made after pediatric rheumatologists, anthropologist/methodologist, psychologist, and pedagogue analyzed the answers (3rdQ).

Phase 2. Validation of questionnaire. 3rdQ was evaluated for face, content, reliability, internal consistency, convergent, and divergent validity. Construct validity was tested for anxiety/depression (Beck), coping strategies (Reyes-Lagunes), QoL (EuroQol), and social and economic factors (Socioeconomic burden). Correlation matrix to assess the construct validity using Cohen’s kappa, and internal consistency tests using Cronbach’s alpha were performed.

**Results:** Qualitative study (1stQ) identified topics like socioeconomic burden, family interactions, alternative medicine, religion, information access, and prognosis. 2ndQ was conformed by 75 items. After pilot study, six items were confuse and modified, 11 were eliminated for being redundant. Items were organized in five domains to develop 3rdQ: disease impact (social, economic, family and relationship), knowledge, future, alternative medicine, and religion (13, 27, 2, 2, and 2 items, respectively). Validity analysis included 32 participants (93% women). Mean age 39 (SD 8.5) years, mean EuroQol VAS 82, minimal depression in 90%. Socioeconomic tool demonstrated family income above 14 minimum wages (65 USD) in 68% and 59% have remunerated job. Reyes-Lagunes describes “life coping” with emotional-negative strategies in 31 caregivers and straight strategies in “children´s health coping” in 100%. Finally, in 3rdQ, 22% felt sadness at diagnosis, 84% change initial feeling and currently 43% are relief. Sixty two percent mention disease has influenced in their financial situation and 72% feel anxiety about their children future. Statistical analysis show adequate inter-item reliability in all domains except for religion, consequently items were adapted to create new dimensions (Table 1). Correlation matrix shown good results in all domains, except for social and economic due to redundant items. After changes, CAREGIVERS Questionnaire was created.

**Conclusion:** We design CAREGIVERS Questionnaire, a specific multidimensional tool to assess burden in caregivers of patients with PRD, and validated in JIA. Further efforts will be performed to validated in other PRD.

**Disclosure of Interest**

None Declared

**Table 1 (abstract O07).** See text for description

Domains	Cronbach’s	items
Disease impact on:		
- Social	0.42	13
- Economic	0.43	13
- Familiar	0.19	4
- Relationship	0.26	2
II. Disease knowledge	0.28	8
III. Discrimination	0.68	4
IV. Use of social networks	0.55	10
V. Future	0.31	2
VI. Religion	0.004	2

### O08 TREAT-TO-TARGET USING FIRST-LINE TREATMENT WITH RECOMBINANT IL-1 RECEPTOR ANTAGONIST IS HIGHLY EFFECTIVE FOR SYSTEMIC JIA: A 5 YEAR FOLLOW-UP STUDY

#### Nienke M. ter Haar^1,2^, Evert H. P. van Dijkhuizen^2^, Joost F. Swart^2^, Wilco de Jager^1^, Dirk Holzinger^3,4^, Arjen P. Leek^2^, Jorg van Loosdregt^1^, Annet Van Royen-Kerkhof^2^, Nico M. Wulffraat^2^, Sytze de Roock^1,2^, Sebastiaan J. Vastert^1,2^

##### ^1^Laboratory for Translational Immunology; ^2^Paediatric Rheumatology and Immunology, University Medical Center Utrecht, Utrecht, Netherlands; ^3^Paediatric Rheumatology and Immunology, University of Muenster, Muenster; ^4^Paediatric Hematology-Oncology, University of Duisburg-Essen, Essen, Germany

###### **Correspondence:** Sebastiaan J. Vastert

**Introduction:** Systemic onset JIA (sJIA) is a multifactorial autoinflammatory disease. Historically, the prognosis of sJIA was very poor. The use of interleukin (IL)-1 and IL-6 blockade improved outcome, but still most studies report inactive disease in only 30-50% of patients within one year. It is hypothesized that innate immune activation is most prominent in the early phase of sJIA. To make use of this ‘window of opportunity’, we started a prospective cohort with recombinant IL-1 receptor antagonist (rIL-1RA) as first-line monotherapy in new-onset sJIA patients (Vastert *et al.* AR 2014).

**Objectives:** To analyse the long-term efficacy of this therapeutic strategy and to separately analyse a subset of sJIA patients without arthritis at disease onset.

**Methods:** In this single centre, prospective cohort study, new-onset sJIA patients with an unsatisfactory response to NSAIDs received rIL-1RA 2mg/kg. Inactive disease (ID) was defined according to the Wallace criteria. In patients reaching ID, rIL-1RA was tapered after 3 months and subsequently stopped. In patients with an incomplete response to rIL-1RA, our protocol directed to increase rIL-1RA dose, add prednisolone or switch to alternative therapy. Clinical data including damage (JADI) and patient reported outcomes (CHAQ and/or JAMAR) were completed in a standardized way. Cytokine profiling was performed using Luminex multiplex immunoassay.

**Results:** Forty-two patients were analysed, with a median follow-up of 5.8 years. At 3 months, 30/42 patients (71%) had ID with rIL-1RA monotherapy and another 6 had ID with rIL-1RA and prednisone. At one year, 32 patients (76%) had ID, of which 22 (52%) were off medication.

Twelve patients had no arthritis at onset; 4 of them developed arthritis during flares. The clinical phenotype, inflammatory parameters and cytokine profile of patients without arthritis at onset was similar to patients with arthritis. Hence, after extensive ancillary investigations to exclude other diseases, these non-arthritic patients also received rIL-1RA as first-line therapy. After 3 months, 11/12 non-arthritic patients had ID (10 on rIL-1RA monotherapy), at 1 year all 12 had ID (8/12 off medication).

Long-term outcome reflected the high efficacy of first-line rIL-1RA: 95% of all patients had ID and 72% had ID off medication at 3 and 5 years follow-up, only 5% reported articular damage and 5% extra-articular damage, no patient developed growth failure and only one developed obesity during follow-up. Moreover, around 60% of patients reported complete absence of pain and disease, no limitations in daily life and the highest possible score on well-being. One patient died due to MAS. In total, 24 patients (57%) never used other medication besides rIL-1RA and NSAIDs and only 14 patients (33%) used glucocorticoids.

Patients who achieved ID at one year were younger and had a significantly shorter disease duration, less active joints and higher neutrophil and leukocyte count before start of rIL-1RA. Multivariate analysis confirmed high neutrophil count as a significant predictor for ID at one year. Furthermore, ID at one year was highly associated with a good response at 1 month.

**Conclusion:** First-line, short course monotherapy with rIL-1RA is a highly efficacious strategy to induce and sustain inactive disease and to prevent disease- and glucocorticoid-related damage. Our data on sJIA patients without arthritis plea for leaving out arthritis as a prerequisite criterion in future disease classification criteria.

**Disclosure of Interest**

None Declared

### O09 CLINICAL OUTCOMES FOLLOWING MYCOPHENOLATE MOFETIL VS. CYCLOPHOSPHAMIDE INDUCTION TREATMENT FOR PROLIFERATIVE JUVENILE-ONSET LUPUS NEPHRITIS

#### Eve M. Smith^1^, Michael W. Beresford^1^, Christian M. Hedrich^2^

##### ^1^Institute of Translational Medicine; ^2^Institue of Translational Medicine, University of Liverpool, Liverpool, UK

###### **Correspondence:** Eve M. Smith

**Introduction:** Treatment of proliferative class III/IV lupus nephritis (LN) in children is largely based upon adult studies. The largest study demonstrated a similar renal response rate with mycophenolate mofetil (MMF) or cyclophosphamide (CYC) induction treatment in adult patients with LN [1]. A further smaller study showed MMF to be more effective than CYC [2]. In both, MMF had a better safety profile [1,2].

**Objectives:** Within a predominantly Caucasian JSLE cohort, to compare efficacy of MMF vs. CYC in patients with LN, monitoring 1) response to treatment, 2) damage accrual, 3) time to achievement of inactive LN, and 4) time to subsequent LN flare, following MMF or CYC LN induction treatments.

**Methods:** Participants of the UK JSLE Cohort Study (2006-2018), <18 years at the time of diagnosis, with ³4 ACR criteria for SLE, were eligible for inclusion if they had ISN/RPS class III or IV LN. Median values and interquartile ranges quoted. Mann-Whitney U tests for continuous data and Fishers exact test for categorical data.

**Results:** 69/411 UK JSLE Cohort Study patients met the inclusion criteria; 18/61 were excluded due to insufficient follow-up. 34/51 (67%) received MMF (13/34 (38%) class III, 21/34 (62%) class IV LN), and 17/51 (33%) received CYC (8/17 (47%) class III, 9/17 (53%) class IV LN). No statistically significant differences were identified between treatment groups at 4-8, 10-14 months post renal biopsy, and last follow-up, in terms of the renal-BILAG score, urine albumin/creatinine ratio, serum creatinine, ESR, anti-dsDNA antibody, C3 levels and patient/physician global scores (all *p>0.05,* see Table 1). Standardised Damage Index (SLICC-SDI) scores did not differ between treatment groups at a median of 13 months, or last follow-up (all *p>0.05*, see Table 1). Inactive LN, following the definition of renal BILAG, was attained at 262 days [141–390] after MMF treatment, and 151 days [117-305] following CYC (*p=0.17*). Time to subsequent renal flare did not differ; 451 days [157–1266] for MMF, and 343 days [198–635] for CYC (*p=0.47*)).

**Conclusion:** This is the largest study to date investigating induction treatments for proliferative LN in children. In Caucasian JSLE populations, MMF and CYC may be comparably efficacious in regards to treatment response, damage accrual, and time to next flare. Remission may be reached quicker in patients treated with CYC. Future prospective comparison is warranted to inform LN treatment, given CYC’s poor safety profile.

**References**

1. Appel G, et al. *JASN* 2009, 20:1103-12.

2. Ginzler E, et al. *NEJM* 2005, 353:2219-28.

**Disclosure of Interest**

None Declared

**Table 1 (abstract O09).** Comparison of clinical parameters and SLICC-SDI scores following MMF vs CYC treatment

Time post biopsy*	Outcome parameter	MMF induction	CYC induction	*p*
10-14	Renal BILAG score	A=1, B=5, C=3, D=18	A=1, B=3, C=2, D=8	*1.00*
	UACR**	13[4-42]	21[3–56]	*0.99*
	Serum creatinine	59[51-69]	62[50-73]	*0.32*
	ESR	12[5–21]	20[4–49]	*0.62*
	dsDNA	44[24-95]	20[6-440]	*0.84*
	C3	0.98[0.81-1.18]	1.04[0.92–1.34]	*0.20*
	Patient global	4.5[0–9.3]	3.0[0.5–52.0]	*0.98*
	Physician global	7.0[1.3–14.0]	9.9[3.0–24.0]	*0.66*
13***	SLICC SDI	0[0-1]	0[0–2.5]	0.67

*Months. **Urinary albumin/creatinine ratio. ***SLICC SDI score after a median of 13 months post renal biopsy (range 10-18)

### O10 WORLDWIDE COHERENCE OF ASSOCIATION OF KAWASAKI DISEASE TO MASSIVE AGRICULTURAL CROPLAND BYPRODUCTS

#### Xavier Rodó^1,2^, Joseph Boyard-Micheau^1^, Silvia Borràs^1^, Dan Cayan^3^, Jane Burns^4^on behalf of International Kawasaki Disease Consortium, Yosikazu Nakamura^5^, Judith Sanchez Manubens^6^, Jordi Anton^6^, Josep-Anton Morguí^7^, Roger Curcoll^7^, Joan Ballester^1^ and International Kawasaki Disease Consortium

##### ^1^Climate and Health Program, ISGlobal (Barcelona Institute of Global Health); ^2^ICREA, Barcelona, Spain; ^3^Climate, Atmospheric Sciences, and Physical Oceanography, Scripps Institution of Oceanography, UCSD; ^4^ Kawasaki Disease Research Center, Dept. of Pediatrics University of California San Diego, La Jolla, CA, USA; ^5^Jichi Medical Hospital, Tokyo University, Tokyo, Japan; ^6^Pediatric Rheumatology Unit, Hospital Sant Joan de Déu; ^7^Land, Atmosphere and Oceans Lab, ICTA, Universitat Autònoma de Barcelona, Barcelona, Spain

###### **Correspondence:** Xavier Rodó

**Introduction:** Long-standing debate continues on the factors and potential etiological agents of vasculitides and rheumatic diseases, such as Kawasaki disease (KD). Together with the genetic predisposition to suffer the condition, hitherto undefined environmental factors have been suggested to contribute to the onset of autoimmune vasculitis in susceptible individuals. A combined approach integrating research on atmospheric circulation and wind dynamics with a time-series study on Kawasaki disease epidemiology in Japan and the US led to a publication firmly pointing to the role of winds and large-scale circulation in the propagation of the potential aetiologic agent of this disease (Rodó et al., 2011).

**Objectives:** The hypothesis of a windborne role in the generation of KD etiology is further tested in this study for a series of other world locations where the disease is also prevalent and along the suspicion that the interaction between bioaerosols, the air chemistry and predisposed or genetically susceptible patients might similarly be associated with KD incidence there. Recovery of land source regions is attempted with the aim of exploring similarities/differences with those found for Japan. Exploration of the phenological status of vegetation in each of those region is performed to seek further mechanistic explanations on the epidemiological links to KD.

**Methods:** In this study, a retrospective time series analysis of historical daily hospital admissions of KD and reconstruction of air movements and source regions is presented for several locations in all continents (US and Canada, Chile, France, the United Kingdom, Catalonia, South Korea, India and Thailand). The FLEXPART v9 atmospheric particle dispersion model is used and land-surface cover and high-resolution vegetation data employed to explore relationships to stages of vegetation development.

**Results:** Source regions and their land-cover typology is reconstructed for all major KD world sites in terms of disease incidence. Similar strong relationships emerge to cereal croplands in all the different continents. The phenological cycle of vegetation in those areas shows a link to the stages where vegetation is decaying, indicating that a relationship between the major KD season and some kind of agricultural byproducts or remnants might play a role in the onset or exacerbation of this disease. An application of a statistical model integrating the air physics ssociated to the movement of winds from these sources further demonstrates predictive capacity to anticipate peaks in KD incidence.

**Conclusion:** Long-standing debate continues on the factors and potential etiological agents of vasculitides and rheumatic diseases, such as Kawasaki disease (KD) in susceptible children. Together with the genetic predisposition to suffer the condition, hitherto undefined environmental factors are now suggested to contribute to the onset of autoimmune vasculitis in susceptible individuals. The role of bioaerosols and air chemistry and physics is presented pointing to a much clearer combination of factors ultimately appearing to be crucial to the manifestation of epidemiological clusters of this pediatric vasculitis. A computational model integrating all this new environmental information in combination with epidemiological dynamics proves successful in predicting KD incidence in Japan.

**Disclosure of Interest**

None Declared

## HPPR/PRES Joint Session JMD

### O11 THE INTERACTION BETWEEN GENETIC RISK FACTORS AND AGE OF DISEASE ONSET IN JUVENILE DERMATOMYOSITIS

#### Claire T. Deakin^1,2,3^, John Bowes^4^, Meredyth Wilkinson^1^, Lucy R. Marshall^1^, Cerise R. Johnson-Moore^1^, Gulnara Mamyrova^5^, Rudolfo Curiel^5^, Kelly Rouster-Stevens^6^, Heinrike Schmeling^7^, Helga Sanner^8^, Adam M. Huber^9^, Brian M. Feldman^10^, Ann M. Reed^11^, Lauren M. Pachman^12^, Stephen Eyre^4^, Soumya Raychaudhuri^13,14^, Lucy R. Wedderburn^1,2,3,15^, JDCBS and MYOGEN

##### ^1^UCL Great Ormond Street Institute of Child Health; ^2^ARUK Centre for Adolescent Rheumatology, University College London; ^3^NIHR Biomedical Research Centre, Great Ormond Street Hospital, London; ^4^ARUK Centre for Genetics and Genomics, University of Manchester, Manchester, UK; ^5^George Washington University, Washington; ^6^Emory University, Atlanta, USA; ^7^Department of Pediatrics, Alberta Children's Hospital, Calgary, Canada; ^8^Oslo University Hospital, Oslo, Norway; ^9^IWK Health Centre, Halifax; ^10^The Hospital for Sick Children, Toronto, Canada; ^11^Duke University, Durham; ^12^Northwestern University, Chicago; ^13^Brigham and Women's Hospital and Harvard Medical School, Boston; ^14^Broad Institute of MIT and Harvard, Cambridge, USA; ^15^Great Ormond Street Hospital for Children NHS Trust, London, UK

###### **Correspondence:** Claire T. Deakin

**Introduction:** Juvenile dermatomyositis (JDM) is a rare, severe autoimmune disease characterised by proximal muscle weakness and skin rash. While JDM and adult-onset dermatomyositis (DM) share similar clinical and biological features, there are differences in prevalence of these features, including cancer, calcinosis and myositis-specific autoantibodies, suggesting a possible influence of age on pathogenesis.

**Objectives:** To investigate genetic risk factors for JDM and age of disease onset.

**Methods:** Caucasian JDM cases from the UK (n=312) were genotyped (Illumina HumanCoreExome). Caucasian control data (n=2808) were obtained from the Wellcome Trust Case Control Consortium (Affymetrix 500K). Following quality control, classical human leukocyte antigen (HLA) alleles and HLA amino acids were imputed using SNP2HLA. Logistic, linear and Cox regression were performed using PLINK and R package GenABEL, with adjustment for the first two principal components. Genome-wide association was set at P<5×10^-8^ and suggestive association at P<1×10^-5^. We subsequently built an international consortium to create a replication cohort of Caucasian cases (n=479). Genotyping of the replication cohort is ongoing using the HumanCoreExome array.

**Results:** Case-control analyses confirmed involvement of HLA including multiple loci within *HLA-C* (p=1.35×10^‑8^, OR=2.49, 95% CI = 1.82-3.42) and *HLA-DRB1* (p=2.73×10^-8^, OR=0.56, 95% CI=0.46-0.69). Outside the HLA region there was suggestive evidence of association at *ZNF337* (p=7.49×10^-6^, OR=1.81, 95% CI=1.40-2.34). Analyses of association with age of disease onset did not implicate HLA involvement, suggesting age does not influence the association between HLA and JDM/DM. Analysis of age of onset as a quantitative trait revealed suggestive associations at *PDE1A* (p=1.56×10^‑6^, β=-1.61, 95% CI = -2.26--0.97) and *AGPAT3* (p=2.26×10^-6^, β=1.63, 95% CI = 0.97-2.30), genes involved in regulating intracellular cyclic nucleic acid concentrations and phospholipid biosynthesis/Golgi-to-endoplasmic reticulum retrograde transport, respectively. In addition, we now have a replication cohort via our international consortium to validate these findings. Genotyping of the replication cohort is ongoing using the HumanCoreExome array.

**Conclusion:** This study confirms previous findings regarding HLA involvement. Analyses of associations with age of JDM onset identified novel loci, *PDE1A* and *AGPAT3*, which if validated in an independent replication cohort could suggest novel processes involved in pathogenesis. Together with the replication cohort, this study will be the largest GWAS of JDM to date.

**Disclosure of Interest**

None Declared

## Novel therapeutic targets: from lab bench to bed side

### O12 CORRELATIONS OF TYPE I INTERFERON SCORE AND INTERFERON INDUCED CHEMOKINES (CXCL10 AND CXCL9) WITH CUTANEOUS AND MUSCULAR DISEASE ACTIVITY IN JUVENILE DERMATOMYOSITIS

#### Gian Marco Moneta^1^, Ivan Caiello^1^, Lucilla Rava’^2^, Silvia Rosina^3^, Luisa Bracci-Laudiero^1,4^, Angelo Ravelli^3^, Fabrizio De Benedetti^1^, Rebecca Nicolai^1^

##### ^1^Division of Rheumatology; ^2^Epidemiology Unit, Ospedale Pediatrico Bambino Gesù, Rome; ^3^Rheumatology, Giannina Gaslini Institute, Genoa; ^4^Institute of Translational Pharmacology, CNR, Rome, Italy

###### **Correspondence:** Gian Marco Moneta

**Introduction:** Interferons (IFNs) seem to play an important role in the pathogenesis of juvenile dermatomyositis (JDM). We previously reported that expression of both type I and type II IFN related genes is increased in muscle biopsies of JDM patients and correlates with histological and clinical features of the disease. Interferon regulated genes (IRGs) have also been reported to be upregulated in peripheral blood of JDM patients and could represent valuable biomarkers of disease activity.

**Objectives:** The aim of this study was to investigate expression of IRGs (measured as type I IFN score), as well as serum levels of two type I and type II IFN induced chemokines (CXCL9, CXCL10) in peripheral blood of JDM patients and to assess their correlations with clinical and laboratory findings.

**Methods:** We collected 125 blood samples from 28 JDM patients at different time points during follow-up. We measured expression of IRGs (IFI27, IFI44L, IFIT1, ISG15, RSAD2, SIGLEC1) by quantitative PCR (qPCR) and calculated the type I IFN score; serum levels of CXCL9 and CXCL10 were analyzed by ELISA. At each visit, the following clinical data were recorded: physician’s global assessment of disease activity VAS (Visual Analogue Scale), cutaneous VAS, Cutaneous Assessment Tool (CAT) activity score, Childhood Myositis Assessment Score (CMAS), serum levels of creatine phosphokinase (CK, IU/l), antinuclear antibody (ANA) status, presence of myositis specific or myositis associated antibodies (MSA/MAA), prednisone (or equivalent) dose (mg/kg/daily), ongoing immunosuppressive medications.

**Results:** Type I IFN score was significantly higher in patients with features of active disease (physician’s global VAS >0.2, CAT activity score≥1, CK>150 IU/l). CXCL10 levels were significantly higher in patients with features of active muscle disease (CMAS<46, CK>150 IU/l) whereas CXCL9 levels were significantly higher only in patients with abnormal CK levels. In a multilevel mixed effect approach, type I IFN score was significantly associated with physician’s global VAS, cutaneous VAS, CAT activity score, CMAS and CK levels; CXCL9 showed no significant association with the evaluated clinical features; CXCL10 levels were significantly associated with CK levels and CMAS. Including time from disease onset to sampling did not change the results. Immunosuppressive medications negatively modulated expression of IRGs and IFN induced chemokines.

**Conclusion:** Our findings indicate that expression of IRGs, measured as type I IFN score, and serum levels of CXCL10 reflect specific features of disease activity in JDM, further supporting their role as valuable disease biomarkers.

**Disclosure of Interest**

G. M. Moneta: None Declared, I. Caiello: None Declared, L. Rava’: None Declared, S. Rosina: None Declared, L. Bracci-Laudiero: None Declared, A. Ravelli: None Declared, F. De Benedetti Grant/Research Support from: BMS, Pfizer, Abbvie, Novartis, Novimmune, Roche, SOBI, Sanofi, UBC, Consultant for: Roche, Novartis, Novimmune, SOBI, R. Nicolai: None Declared

## Oral presentation 2

### O13 EARLY AGGRESSIVE TREATMENT DECREASES PAIN IN PATIENTS WITH JUVENILE IDIOPATHIC ARTHRITIS

#### Maarit Tarkiainen^1,2^, Pirjo Tynjälä^3^, Paula Vähäsalo^4^, Liisa Kröger^5^, Kristiina Aalto^6^, Pekka Lahdenne^7^ and ACUTE-JIA working group

##### ^1^Department of Pediatric Rheumatology, Children’s Hospital, Helsinki University Central Hospital; ^2^University of Helsinki; ^3^Poison Information Centre, , Helsinki University Central Hospital, Helsinki; ^4^Department of Pediatrics, Oulu University Hospital, Oulu; ^5^Department of Pediatrics, Kuopio University Hospital, Kuopio; ^6^Department of Pediatric Rheumatology; ^7^Helsinki University Central Hospital, Helsinki, Finland

###### **Correspondence:** Maarit Tarkiainen

**Introduction:** Patients with Juvenile Idiopathic Arthritis (JIA) experience more pain than normal population. Pain has become an important outcome measure in JIA.

**Objectives:** To measure the level of pain during the first year of early aggressive treatment in JIA and at three years from disease onset.

**Methods:** This study was performed along with Acute-JIA study, in which 60 new-onset patients with polyarticular JIA were randomized to receive either infliximab plus methotrexate (IFX), combination of hydroxychloroquine, sulphasalazine, and methotrexate (Triple) or methotrexate monotherapy (MTX). Disease activity and quality of life were measured at 7 study visits during the first year from onset, and at 3 years’ follow-up visit during open-label extension. Pain VAS (0-100) was utilized. Patients continued on their originally randomized treatment until they failed the strategy or achieved inactive disease (ID) on medication for at least 6 months.

**Results:** All 60 patients in the original trial completed the 3-year follow-up and 56 pain surveys. 50 (83%) switched treatment strategies between 1 to 3 years. Pain-VAS decreased from 37.0 (SD 24.3) at onset to 8.5 (15.6) at 1 year (Wilk’s Lambda p<0.005). During the open-label extension, the level of pain increased to 10.1 (15.4) at 3 years (p=0.068, compared with 1 year). No differences between treatment groups were detected (p=0.21). CHAQ and JADAS at each time point associated with level of pain. Cumulative time spent in inactive disease (ID) associated with lower level of pain at 3 years (coefficient beta -0.63, p=0.004)

**Conclusion:** During one-year early aggressive treatment, pain decreased as disease activity diminished. Small, although not statistically different increase in pain was noted during open-label extension. The treatment strategy seemed to have no influence on pain.

**Trial registration identifying number:** Trial registration: ClinicalTrials.gov NCT01015547

**Disclosure of Interest**

None Declared

### O14 PHARMACOVIGILANCE IN JUVENILE IDIOPATHIC ARTHRITIS PATIENTS TREATED WITH BIOLOGIC AGENTS AND/OR METHOTREXATE. COMBINED DATA OF MORE THAN 15,000 PATIENTS FROM PHARMACHILD AND NATIONAL REGISTRIES

#### Gabriella Giancane, Joost Swart, Gerd Horneff, Bo Magnusson, Michael Hofer, Ekaterina Alexeeva, Violeta Panaviene, Brigitte Bader-Meunier, Jordi Anton, Susan Nielsen, Fabrizio De Benedetti, Sylvia Kamphuis, Valda Stanevicha, Maria Trachana, Laura M. Ailioaie, Elena Tsitsami, Ariane Klein, Kirsten Minden, Ivan Foeldvari, Johannes-Peter Haas, Jens Klotsche, Alessandro Consolaro, Francesca Bovis, Francesca Bagnasco, Angela Pistorio, Alberto Martini, Nico Wulffraat, Nicolino Ruperto and for the Paediatric Rheumatology International Trials Organisation (PRINTO), BiKeR and the board of the Swedish Registry.

##### Clinica Pediatrica e Reumatologia - PRINTO, Istituto Giannina Gaslini, GENOA, Italy

###### **Correspondence:** Gabriella Giancane

**Introduction:** The availability of methotrexate (MTX) and the introduction of multiple biological agents have revolutionized the treatment of juvenile idiopathic arthritis (JIA). Several international and national drug registries have been implemented to monitor accurately the long-term safety/efficacy of these agents.

**Objectives:** This study aims to present the combined data coming from “Pharmacovigilance in JIA patients treated with biologic agents and/or MTX” (Pharmachild)/Pediatric Rheumatology International Organization (PRINTO) registry and the national registries from Germany (BiKeR) and Sweden.

**Methods:** Descriptive statistics for demographic, clinical data, drug exposure, adverse events (AE) and events of special interest (ESI) were reported. For the Swedish register, AE data were not available.

**Results:** A total of 15,284 patient’s data were reported, 8,274 (54%) from the Pharmachild registry, 3,990 (26%) and 3,020 (20%) from the Germany and the Swedish registries, respectively. Pharmachild children showed a younger age (median of 5.4 years versus 7.6) at JIA onset and shorter disease duration (5.3 versus 6.1-6.8) when compared to the other registries. The most frequent JIA category was the rheumatoid factor negative polyarthritis (range 24.6-29.9%). Methotrexate (61-84%) and etanercept (24%>61.8%) were the most frequently used synthetic and biologic disease-modifying anti-rheumatic drug (DMARD), respectively. There was a wide variability in glucocorticoid use (16.7-42.1%). Serious AE were present in 572 (6.9%) patients in Pharmachild versus 297 (7.4%) in BiKeR. Infection and infestations were the most frequent AE (29.4-30.1%) followed by gastrointestinal disorders (11.5-19.6 %). The most frequent ESI were infections (75.3-89%),(Table 1).

**Conclusion:** Sharing of data from national and international registries represents the most powerful tool for future analysis of safety and effectiveness of immunosuppressive therapies in JIA.

**Trial registration identifying number:** NCT01399281

**Disclosure of Interest**

G. Giancane: None Declared, J. Swart: None Declared, G. Horneff: None Declared, B. Magnusson: None Declared, M. Hofer: None Declared, E. Alexeeva: None Declared, V. Panaviene: None Declared, B. Bader-Meunier: None Declared, J. Anton: None Declared, S. Nielsen: None Declared, F. De Benedetti: None Declared, S. Kamphuis: None Declared, V. Stanevicha: None Declared, M. Trachana: None Declared, L. Ailioaie: None Declared, E. Tsitsami: None Declared, A. Klein: None Declared, K. Minden: None Declared, I. Foeldvari: None Declared, J.-P. Haas: None Declared, J. Klotsche: None Declared, A. Consolaro: None Declared, F. Bovis: None Declared, F. Bagnasco: None Declared, A. Pistorio: None Declared, A. Martini: None Declared, N. Wulffraat: None Declared, N. Ruperto Grant/Research Support from: The G. Gaslini Hospital, which is the public Hospital where I work as full time public employee, has received contributions from the following industries for the coordination activity of the PRINTO network: BMS, GlaxoSmithKline (GSK), Hoffman-La Roche, Novartis, Pfizer, Sanofi Aventis, Schwarz Biosciences, Abbott, Francesco Angelini S.P.A., Sobi, Merck Serono. This money has been reinvested for the research activities of the hospital in fully independent manners without any commitment with third parties., Speaker Bureau of: I received speaker’s bureaus and consulting fees from the following pharmaceutical companies: AbbVie, Amgen, Biogenidec, Alter, AstraZeneca, Baxalta Biosimilars, Biogenidec, Boehringer, BMS, Celgene, CrescendoBio, EMD Serono, Hoffman-La Roche, Italfarmaco, Janssen, MedImmune, Medac, Novartis, Novo Nordisk, Pfizer, Sanofi Aventis, Servier, Takeda, UCB Biosciences GmbH.

**Table 1 (abstract O14).** Most frequent events of special interest ordered by decreasing frequencies. Data are absolute numbers and frequencies (%)

	Pharmachild N = 2,022 (%)	BiKeR N=1,697 (%)
Infections:	1523 (75.3)	1509 (89)
Serious/targeted infections (Epstein‐Barr virus, cytomegalovirus, papilloma virus, herpes zoster primary and reactivation, and opportunistic infections)	674 (33.3)	171 (10.1)
Tuberculosis	27 (1,3)	0
Other infections	822 (40.6)	1338 (78.8)
Infusion/injection related reactions:	218 (10.8)	24 (1.4)
Infusion related reaction	144 (7.1)	11 (0.6)
Injection related reaction	74 (3.7)	13 (0.8)
Blood cells related ESI:	188 (9.3)	90 (5.3)
Pancytopenia	6 (0.3)	65 (3.8)
Neutropenia	107 (5.3)	14 (0.8)
Macrophage activation syndrome	75 (3.7)	11 (0.6)
Aplastic anemia	0	0
Autoimmune ESI:	50 (2.5)	50 (2.9)
Inflammatory Bowel Disease (IBD)	21 (1.1)	23 (1.3)
Other autoimmune diseases excluding IBD, uveitis and demyelinisation disorders	18 (0.9)	24 (1.4)
Lupus erythematosus systemic/lupus-like syndrome	4 (0.2)	1 (0.1)
Optic neuritis	4 (0.2)	0
Multiple sclerosis	2 (0.1)	0
Demyelination	1 (0.05)	2 (0.2)
Malignancies:	16 (0.8)	13 (0.8)
Leukaemias	3 (0.1)	2 (0.2)
Lymphomas	2 (0.1)	5 (0.3)
Haematopoietic neoplasms (excluding leukaemias and lymphomas)	1 (0.05)	2 (0.2)
Neoplasm (other)	10 (0.5)	4 (0.2)

### O15 REMISSION STATUS AFTER 18 YEARS OF FOLLOW-UP IN THE POPULATION-BASED NORDIC JUVENILE IDIOPATHIC ARTHRITIS (JIA) COHORT.

#### Mia Glerup^1^, Veronika Rypdal^2^, Ellen Arnstad^3,4^, Maria Ekelund^5,6^, Suvi Peltoniemi^6,7^, Kristiina Aalto^8^, Marite Rygg^4,9^, Peter Toftedal^10^, Susan Nielsen^10^, Anders Fasth^11^, Lillemor Berntson^6^, Ellen Nordal^2^, Troels Herlin^12^

##### ^1^Pediatrics, Aarhus University Hospital, Aarhus N, Denmark; ^2^University Hospital of North Norway, Tromsø; ^3^Levanger Hospital, Nord-Trøndelag; ^4^Fac. of Med. and Health Sci., NTNU, Norway; ^5^Ryhov County Hospital, Jonkoping; ^6^Uppsala University, Uppsala, Sweden; ^7^University of Helsinki; ^8^Hospital for Children and Adolescents, Helsinki, Finland; ^9^St. Olavs Hospital, Trondheim, Norway; ^10^Rigshospitalet, Copenhagen, Denmark; ^11^Sahlgrenska Academy, Gothenburg, Sweden; ^12^Aarhus University Hospital, Aarhus N, Denmark

###### **Correspondence:** Mia Glerup

**Introduction:** Innovative changes towards targeted treatment have improved the outcome dramatically for juvenile idiopathic arthritis (JIA) during the last two decades. The question remains how well these children perform during long-term follow-up.

**Objectives:** To study the disease activity, remission rate and damage 18 years after JIA onset.

**Methods:** 510 consecutive cases of JIA with disease onset in 1997 to 2000 from Denmark, Norway, Sweden and Finland were prospectively included in a longitudinal, close to population-based 18-year (mean 17.5 years; range 14.2-20.2) follow-up study. The last follow-up visit included an update on the family history, medication and clinical data such as a clinical joint examination and remission status according to the preliminary Wallace criteria.

**Results:** 434 (85%) out of 510 eligible JIA participants with a mean age (S.D) of 24.0 (± 4.4) years were included. 76 patients (15%) were lost to follow-up. Out of the included patients 329 (76%) attended a follow-up visit, and 105 patients (24%) were evaluated by a telephone interview.

The distribution of the JIA categories was as follows: 3% systemic, 27% persistent oligoarticular, 20% extended oligoarticular, 16% polyarticular RF negative, 1% polyarticular RF positive, 7% psoriatic, 10% enthesitis-related arthritis (ERA) and 15% undifferentiated JIA.

For all 329 patients attending a clinical visit the median active joint count was 0 (range 0-15), however during the 18 years of disease course the median cumulative joint count was 8 (range 1-59). 66 participants (15 %) received disease-modifying anti-rheumatic drugs (DMARDS) and 84 patients (19%) were treated with biologics at the last follow-up.

At the final study visit the median composite juvenile arthritis disease activity score JADAS71 was median 1.5 (range 0-31.6) with the ERA category having the highest median score of 4.5 (range 0-16.5) (p=0.003). In the follow-up cohort 48% had a JADAS71 score <1 indicating inactive disease.

Articular damage (JADI-A) was found in 20% of the patients who had a follow-up visit and 12.5% had developed extra-articular damage (JADI-E), most commonly ocular damage, found in 26/41 (63%). The highest JADI-A and JADI-E scores were found in the polyarticular RF negative and psoriatic categories.

Less than half (44%) of the participants were in remission off medication 18 years after disease onset, but 39.8% had active disease. Achievement of remission off medication was observed in the following order: persistent oligoarticular 39/72 (54%), systemic 7/13 (54%), psoriatic 9/22 (41%), undifferentiated 21/55 (38%), extended oligoarticular 19/66 (29%), RFneg poly 16/59 (27%), RFpos poly 1/5 (20%), and ERA 4/37 (11%), the latter being significantly different from the other categories (p<0.001).

**Conclusion:** A significant proportion of the JIA cohort do not reach remission despite new treatment options and notably more than one third receive systemic treatment even 18 years after disease onset. The worst outcome was evident in the ERA category and in general the JIA disease burden in adulthood remains extensive.

**Disclosure of Interest**

None Declared

### O16 RESULTS AFTER 24 MONTHS OF DRUG-FREE INACTIVE DISEASE AND FLARE FREQUENCY IN JUVENILE IDIOPATHIC ARTHRITIS PATIENTS IN A TREATMENT-TO-TARGET STRATEGY STUDY

#### Petra Hissink Muller^1,2^, Danielle M. Brinkman^1,3^, Dieneke Schonenberg-Meinema^4^, Wytse B. van den Bosch^1^, Yvonne Koopman-Keemink^5^, Peter Bekkering^6^, Taco Kuijpers^4^, Marion van Rossum^7^, Lisette van Suijlekom-Smit^2^, J M. van den Berg^8^, Stefan Boehringer^9^, Cornelia F. Allaart^10^, Rebecca ten Cate^1^

##### ^1^Pediatric Rheumatology, LUMC, Leiden; ^2^Pediatric Rheumatology, Erasmus MC, Sophia Children's Hospital , Rotterdam; ^3^Pediatrics , Alrijne, Leiderdorp; ^4^Pediatric Hematology, Immunology, Rheumatology and Infectious Diseases , Emma Children's Hospital AMC, Amsterdam; ^5^Pediatrics , Juliana Children's Hospital, The Hague; ^6^Pediatric Oncology, Princess Maxima Center of Pediatric Oncology, Utrecht; ^7^Pediatrics; ^8^Hematology, Immunology, Rheumatology and Infectious Diseases, Emma Children's Hospital AMC, Amsterdam; ^9^Medical Statistics and Bioinformatics; ^10^Rheumatology, LUMC, Leiden, Netherlands

###### **Correspondence:** Petra Hissink Muller

**Introduction:** Reaching early inactive disease is the goal in Juvenile Idiopathic Arthritis (JIA) patients. How to proceed after reaching inactive disease is subject of ongoing research. Tapering and stopping disease modyfying anti-rheumatic drug (DMARD) therapy, including observation of subsequent flares, was applied in all three arms of the BeSt for Kids study.

**Objectives:** The aim of the BeSt for Kids study was to investigate, which of three treatment strategies was most effective and safe, by comparing them directly. The therapeutic target, in all arms, was inactive disease. If inactive disease was reached and maintained for a specific period of time, medication was tapered and stopped.

**Methods:** We conducted a randomized, single-blinded, multicenter, treatment strategy study with 24 months of follow-up. DMARD-naive JIA (oligoarticular, rheumatoid factor negative polyarticular, and juvenile psoriatic arthritis patients) were randomized to 1) sequential DMARD monotherapy, 2) combination therapy: MTX and prednisolone-bridging, 3) combination therapy MTX with etanercept. For all arms, the treatment protocol described a number of subsequent treatment steps in case medication failed. After reaching inactive disease for 3 (oligoarticular) or 6 (polyarticular) months, medication was tapered and stopped according to protocol. Flare frequency was observed.

Missing data were imputed. In case of drug-free clinically inactive disease often intentionally no blood was drawn causing non-random missing ESR, and here ‘0’ was imputed for the analysis of inactive disease.

Primary outcome was time to inactive disease and time to flare after tapering and stopping DMARD therapy calculated using Kaplan Meier curve with log rank test. Secondary outcomes are adjusted ACRPedi 30/50/70/90 scores, toxicity, functional ability and quality of life. Generalised Estimated Equations were used for longitudinal data analyses.

In this abstract we share time-to-flare, flare frequencies and flare characteristics in all three arms.

**Results:** During 24 months 59% (19 (3 oligo)/31 (61%) of patients in arm 1, 16 (1 oligo)/32 (50%) in arm 2 and 19 (1 oligo)/29 (65%) in arm 3) had tapered and stopped all DMARDs (drug-free inactive disease) after median 15.0 (IQR 12.0-18.0) months (arm 1), 19.5 (12.0-24.0) months (arm 2) and 18.0 (12.0-21.0) months (arm 3) of therapy. However, 26% (6 (1 oligo) patients in arm 1, 3 in arm 2 and 5 in arm 3) subsequently had to restart treatment before end of study, after median time-to-flare 18.0 (15.0-21.0) months (p=0.7).

Flares were characterised by JADAS-10 of 9.7 (8.1-11.3), which improved 3 months after restart of treatment to JADAS-10 of 3.9 (1.8-6.0).

**Conclusion:** During 24 months of  treatment to target inactive disease, including tapering and stop-strategies, flare frequency was 26% after median 18 months. After 24 months, 71% of patients had inactive disease and 39% were in drug-free inactive disease.

It is feasible to incorporate strategies for tapering and stopping DMARD-therapy in juvenile idiopathic arthritis patients, when tight control is maintained.

**Trial registration identifying number:** Trial registration number: Dutch trial register 1574

**Disclosure of Interest**

None Declared

### O17 NEW JADAS10- AND CJADAS10-BASED CUTOFFS FOR JUVENILE IDIOPATHIC ARTHRITIS DISEASE ACTIVITY STATES: VALIDATION IN A MULTINATIONAL DATASET OF 4830 PATIENTS

#### Chiara Trincianti^1^, Giedre Janukeviciute^1^, Pieter Van Dijkhuizen^2^, Carolina Montobbio^1^, Gabriella Giancane^1^, Alessandra Alongi^1^, Nicola Ruperto^1^, Joost Swart^2^, Angelo Ravelli^1,3^, Alessandro Consolaro^1,3^, on behalf of EPOCA Study Group

##### ^1^ISTITUTO GIANNINA GASLINI, Genova, Italy; ^2^University Medical Center, Utrecht, Netherlands; ^3^University of Genova, Genova, Italy

###### **Correspondence:** Chiara Trincianti

**Introduction:** The measurement of disease activity plays a pivotal role in the care of patients with juvenile idiopathic arthritis (JIA). To serve this purpose, the Juvenile Arthritis Disease Activity Score (JADAS) and its clinical version excluding the acute phase reactant (cJADAS) were developed.

Cutoffs for the state of remission, low disease activity (LDA), moderate disease activity (MDA) and high disease activity (HDA) are necessary to interpret the scores and are ideally suited for pursuing tight disease control in a treat-to-target strategy.

Aiming to obtain cutoffs suitable for any clinical setting, new values were recently developed for JADAS10 and cJADAS10 in oligoarthritis and polyarthritis, based on a large multinational dataset of JIA patients (Consolaro A, et al. Arthritis Rheumatol. 2017;69s10).

**Objectives:** To externally validate the new cutoffs for JADAS10 and cJADAS10 disease activity states.

**Methods:** Four not selected for cutoffs development JIA patients dataset were considered: 1) 4397 oligoarthritis and polyarthritis patients from the EPOCA study; 2) 148 oligoarthritis patients from the TRIMECA trial; 3) 172 polyarthritis patients from the Abatacept trial, 4) 113 oligoarthritis and polyarthritis patients first starting methotrexate from a monocentric retrospective cohort (Swart et al, Ann Rheum Dis. 2018;77:336-342).

Face validity was assessed in dataset 1) by plotting the proportion of patients in remission and LDA against the values of the 6 parameters in the ACR JIA core set.

Discriminative ability was assessed in datasets 2) and 3) by comparing the percentage of patients below the cutoff values in the different ACR pediatric categories of response. In dataset 1) we compared in each disease activity state, the level of pain (0-10 VAS), functional ability impairment, and number of restricted joints and the frequency of patients satisfied with disease outcome, starting a new medication for JIA, and having morning stiffness.

Predictive ability was assessed in dataset 4) by calculating the sensitivity and specificity of the cutoffs for remission and LDA after 3 months for treatment response after 12 months.

**Results:** Only most relevant results are described. JADAS10 and cJADAS10 cutoffs for remission allowed up to 1 active joint for polyarthritis and 0 for oligoarthritis.

In dataset 2), 42% and 63% of patients achieving an ACRp70 response met the JADAS10 cutoffs for remission and LDA, respectively. In dataset 3), these percentages were 48% and 82%, respectively. In dataset 1), the median level of pain was 0, 1.5, 3, and 5.5 for polyarthritis patients in cJADAS10 remission, LDA, MDA, and HDA, respectively (Kruskal-Wallis p<0.001). The frequency of satisfaction with disease outcome was 94%, 76%, 56%, and 24% for oligoarthritis patients in cJADAS10 remission, LDA, MDA, and HDA, respectively (Chi2 test p <0.001).

In dataset 4), 100% and 71% of patients with oligoarthritis classified as non-responders after 12 months had JADAS levels after 3 months above the cutoffs for remission and LDA, respectively. For polyarthritis, 90% and 80% of non-responders had JADAS levels after 3 months above the cutoffs for remission and LDA, respectively.

**Conclusion:** New JADAS cutoffs showed good face and content validity; achievement of remission and LDA defined by the cutoffs predicted the response to therapy. Cutoffs were developed and validated in a large multinational dataset and they are ready for use in clinical trials and routine practice.

**Disclosure of Interest**

None Declared

### O18 JUVENILE INFLAMMATORY MYOPATHY WITH POSITIVE MDA5 AUTO-ANTIBODIES: A SPECIFIC SUBGROUP WITH SYSTEMIC UPREGULATION OF INTERFERON-ALPHA PATHWAY.

#### Isabelle Melki^1^, Vincent Bondet^2^, Cyril Gitiaux^1^, Hervé Devilliers^3^, Marie-Louise Fremond^4^, Darragh Duffy^2^, Yanick Crow^4^, Mathieu Rodero^4^, Brigitte Bader-Meunier^1^

##### ^1^APHP; ^2^Pasteur Institute, Paris; ^3^Médecine interne 2 CHU Dijon, Dijon; ^4^Imagine Institute, Paris, France

###### **Correspondence:** Isabelle Melki

**Introduction:** Juvenile dermatomyositis (JDM) and overlap myositis (OM) represent heterogeneous subtypes of juvenile inflammatory myopathy (JIM). Chronic evolution may occur in 60% of cases, and morbidity/mortality is substantial (gastro-intestinal; pulmonary; organ failure).

**Objectives:** Clinico-biological description of JDM and OM with positive MDA5 auto-antibodies in comparison to other JIM.

**Methods:** Retrospective and prospective study of patients fulfilling Bohan and Peter criteria for JDM and Troyanov for OM, aged less than 16 years at diagnosis, ascertained from 3 French paediatric rheumatology reference centres between 2013 to 2018. Severe forms were defined as requiring ICU management, and/or more than 3 treatment lines and/or with fatal outcome. Muscle biopsies were reviewed. Type I interferon pathway activity was assessed by dosage of interferon alpha (IFNa) through digital elisa (Simoa) with a Pan-IFNa antibody in patient sera and myoblast lysate, mRNA expression of interferon stimulated genes (ISGs) by RTqPCR and phosphorylation of STAT by flow cytometry on whole blood.

**Results:** 67 patients were included, 12 (group 1) and 54 (group 2) with and without anti-MDA5 antibodies respectively. Group 1 demonstrated more arthritis (100% vs 24%), cutaneous ulcerations (42% vs 26%), interstitial lung disease (ILD) (33% vs 14%) and a milder muscular involvement (median of CK: 96 vs 149 UI/l), with better preserved muscular function (MMT and C-MAS, respectively 79/80 vs 70/80 and 50/52 vs 38/52). Total muscle biopsy scores were lower for group 1 than group 2 (respectively 5.5 vs 20). Group 1 included more severe forms (36% vs 19%). 133 IFN signatures (ISGs) and 210 IFNa Simoa measurements were assessed. Correlation between ISG expression and protein at 79 simultaneous data points was high (Pearson: r = 0,74 ****). ISGs and IFNa (signature and dosage) were higher in group 1 than 2 (median of ISGs 21 vs 7.4 and median of Simoa 3807.5 vs 49.6 fg/ml). In contrast, IFNa levels in patient myoblasts were similar to controls for group 1 (1 patient), whilst those in group 2 were higher (10 patients). A constitutive phosphorylation of STAT1 and STAT3 was observed in T and B lymphocytes and monocytes of 5 patients, including one from group 1. Treatment with steroids, tacrolimus, rituximab and immunoadsorption in one group 1 patient correlated with the disappearance of MDA5 auto-antibodies, the normalisation of IFNa levels and the induction of complete remission (normalisation of clinical, laboratory and radiological parameters).

**Conclusion:** This study indicates a higher frequency of cutaneous ulcerations, arthritis and ILD, and milder muscular involvement in JIM with positive MDA5 auto-antibodies compared to other JIM. These data suggest an important role of systemic IFNa in the pathology of the disease. In severe and refractory forms, IFNa may be a potential therapeutic target.

**Disclosure of Interest**

None Declared

### O19 PREDICTING NON-RESPONSE TO BIOLOGICALS IN JUVENILE IDIOPATHIC ARTHRITIS PATIENTS; A BIOMARKER HUNT IN PERIPHERAL BLOOD SERUM

#### Koen Willemsen^1^, Sytze de Roock^1^, Wilco de Jager^1^, Nienke ter Haar^1^, Pieter van Dijkhuizen^1^, Rae Yeung^2^, Trang Duong^2^, Nico Wulffraat^1^, Joost Swart^1^, Sebastiaan Vastert^1^

##### ^1^Department of Pediatric Rheumatology and Immunology, Wilhelmina Children’s Hospital, University Medical Centre Utrecht, Utrecht, Netherlands; ^2^Hospital for Sick Children, Toronto, Canada

###### **Correspondence:** Koen Willemsen

**Introduction:** Patients with Juvenile Idiopathic Arthritis (JIA) who fail to reach a state of clinically inactive disease might start biological treatment (TNFa-blockers) [3]. Currently little is known in relation to which patients will respond or not to specific biologicals, potentially leading to a delay in achieving acceptable clinical state [4].

**Objectives:** To investigate the possible predictive value of serum proteins on therapeutic (non-)response prior to start of biological therapy.

**Methods:** This study presents a retrospective analysis of a prospectively collected and followed cohort of non-systemic JIA patients from a single pediatric rheumatology center. Non-response to biological was defined as a necessity to switch biological or when criteria for clinically relevant improvement (a change of ≤-5.5 in JADAS27 [5] while not achieving clinically inactive disease i.e. a JADAS27 ≤1 [6]) were not met and was evaluated 6 months after start of biological therapy. Serum protein concentration was analysed by Luminex® multiplex assay (44-plex including cytokines, chemokines and growth factors). We performed basic statistics, including univariate analysis (Mann-Whitney U test) to assess whether protein concentrations associate with response to therapy. Proteins associating with a p<0.1 were included for further analysis later on whereas proteins with a p<0.05 were considered significant. If proteins correlated with a Spearman’s rho >0.6 the more significant protein was included.

**Results:** 67 Patients were included (Table 1). 36 patients received etanercept, 30 adalimumab and 1 golimumab. Of the oligo articular JIA patients 14 (60.9%) were persistent oligo articular and 13 (56.5%) were ANA+. At t=6 months 15 patients were non-responsive (34.1% of cases with JADAS27 available). We found a significant association with non-response for TNF-ligand-1a (TL1a) and haptoglobin. With a p<0.1 interleukine-8 (IL-8) and vascular endothelial growth factor (VEGF) were of interest for further analysis.

**Conclusion:** This preliminary analysis yielded various proteins of which the serum concentration relates to non-response to biological therapy. These proteins might serve as biomarkers predicting therapeutic response. Further research and analyses, including a planned validation in a separate prospective cohort, is required to predict non-response.

**Disclosure of Interest**

None Declared

**Table 1 (abstract O19).** See text for description

Total *n*	67	42
Female *n (%)*	42	32.7%
Age at sampling *years median (1*^*st*^*;3*^*rd*^ *quartile)*	12.7	(8.52;15.6)
JIA subtype		
Oligo articular JIA *n (%)*	23	34.3%
Poly articular JIA RF- *n (%)*	25	37.3%
Poly articular JIA RF+ *n (%)*	3	4.5%
Enthesitis related JIA *n (%)*	12	17.9%
Psoriatic JIA *n (%)*	4	6.0%
JADAS27 t=0 *median (1*^*st*^*;3*^*rd*^ *quartile)*	10.5	(6.1;15.4)

## Clinical Trials and drug development

### O20 PEDIATRIC OPEN-LABEL CLINICAL STUDY OF RITUXIMAB FOR THE TREATMENT OF GRANULOMATOSIS WITH POLYANGIITIS (GPA) AND MICROSCOPIC POLYANGIITIS (MPA)

#### Paul Brogan^1^, Gavin Cleary^2^, Ozgur Kasapcopur^3^, Satyapal Rangaraj^4^, Rae Yeung^5^, Paul Brunetta^6^, Jennifer Cooper^6^, Pooneh Pordeli^7^, Patricia B. Lehane^8^

##### ^1^UCL Institute of Child Health and Great Ormond Street Hospital NHS Foundation Trust, London; ^2^Alder Hey Children's Hospital, Liverpool, UK; ^3^Cerrahpasa Medical School, Istanbul University, Istanbul, Turkey; ^4^Nottingham University Hospitals NHS Trust, Nottingham, UK; ^5^Hospital for Sick Children and University of Toronto, Toronto, Canada; ^6^Genentech, Inc., South San Francisco, USA; ^7^Hoffmann-La Roche Ltd, Mississauga, Canada; ^8^Roche Products Ltd, Welwyn Garden City, UK

###### **Correspondence:** Paul Brogan

**Introduction:** Rituximab in combination with glucocorticoids (GC) is approved to treat adult patients with GPA or MPA; however, limited data exist on the safety and efficacy of rituximab in pediatric patients with these potentially life- and organ-threatening diseases.

**Objectives:** To report the interim safety, pharmacokinetics (PK) and exploratory efficacy data from the 6-month remission induction phase of a Phase IIa international, open-label, 18-month clinical study of rituximab in pediatric patients with GPA or MPA.

**Methods:** Patients aged ≥2 to ≤18 years with newly diagnosed or relapsing GPA/MPA received 4 intravenous (IV) rituximab infusions of 375 mg/m^2^ body surface area (BSA) on Days 1, 8, 15 and 22 with concomitant GC 1 mg/kg/day (max 60 mg/day) tapered to 0.2 mg/kg/day minimum (max 10 mg/day) by Month 6. All patients received 3 doses of pulse IV methylprednisolone (30 mg/kg/day, max 1g/day) prior to first rituximab infusion and mandatory prophylaxis for *Pneumocystis jiroveci* infection. Patients were also pre-medicated with acetaminophen and an antihistamine, 1 hour before each rituximab infusion. Adverse events (AEs) and laboratory data were measured at each study visit (1, 2, 4 and 6 months). Plasma samples for PK analysis were collected throughout the study; clearance and area under the curve (AUC) were calculated using population PK modeling from the RAVE study of rituximab in adult patients with GPA/MPA (Stone et al. NEJM. 2010;363:221-32). For exploratory efficacy assessment, the Pediatric Vasculitis Activity Score (PVAS) was measured at each study visit.

**Results:** Of the 25 patients enrolled, 19 (76%) had GPA and 6 (25%) had MPA (median [range] age 14 [6-17] years; 80% female). Median (range) disease duration was 0.5 (0.2-0.7) months; 2 patients had received prior cyclophosphamide therapy. All received 4/4 rituximab infusions and completed the 6-month induction phase. By Month 6, all patients had experienced ≥1 AE. The most common AEs by system organ class were infections and infestations in 16 patients (64%). Eleven serious AEs occurred in 7 patients (28%), including 3 serious infections (viral gastroenteritis, one lower and one upper respiratory tract infection). 32% of patients had ≥1 infusion related reaction (IRR). No serious IRRs or deaths were reported. The relationship between AUC and BSA was flat and comparable to adult patients. A total of 13 patients (52%) achieved remission by 6 months, defined as PVAS of 0 and GC dose 0.2 mg/kg/day (max 10 mg/day) or PVAS of 0 on 2 consecutive readings ≥4 weeks apart irrespective of GC dose.

**Conclusion:** In the initial 6 months of this first global clinical trial of rituximab in pediatric patients with GPA/MPA, rituximab was generally safe and well tolerated. The overall safety profile and PK parameters were comparable to rituximab-treated adults with GPA/MPA. No new safety signals were observed. However, the study size and interim nature of the analysis limit firm conclusions. The clinical trial and additional efficacy, PK and safety analyses are ongoing.

**Trial registration identifying number:** NCT01750697

**Disclosure of Interest**

P. Brogan Grant / Research Support from: Roche, SOBI, Novartis, Chemocentryx, Consultant for: Roche, SOBI, UCB, Speaker Bureau of: SOBI, Novartis, G. Cleary Speaker Bureau of: AbbVie, O. Kasapcopur: None Declared, S. Rangaraj: None Declared, R. Yeung Consultant for: Novartis, Eli Lilly, P. Brunetta Shareholder of: Roche, Employee of: Genentech, Inc., J. Cooper Employee of: Genentech, Inc., P. Pordeli Employee of: Roche, P. Lehane Employee of: Roche

## Immune defensee and inflammation

### O21 JUVENILE SYSTEMIC LUPUS ERYTHEMATOSUS WITH A BASELINE HIGH INTERFERON SIGNATURE ASSOCIATES WITH INCREASED IMMUNE CELL TLR7 EXPRESSION AND ENHANCED TLR7 DEPENDENT INTERFERON ALPHA PRODUCTION

#### Kate Webb^1^, Gary Butler^2^, Coziana Ciurtin^1^, Hannah Peckham^1^, Anna Radziszewska^1^, Fraser Simpson^3^, Paola Oliveri^3^, Lucy Wedderburn^1,4^, Yiannis Ioannou^1^

##### ^1^ARUK Centre for Adolescent Rheumatology; ^2^Paediatric Endocrinology; ^3^Department of Genetics, Evolution & Environment, Nanostring Facility, UCL; ^4^NIHR Biomedical Research Centre, UCL, GOSH, London, UK

###### **Correspondence:** Kate Webb

**Introduction:** Juvenile onset systemic lupus erythematosus (jSLE) is characterised by a type 1 interferon (IFN) transcription signature. Up to 80% of patients with jSLE of varying severity express a predominant IFN signature which correlates with disease activity, and is abrogated by steroids. Investigating the IFN signature outside of a flare may provide insight into the true prevalence of high IFN signature disease, and underlying differences in disease pathogenesis, that may be masked by steroid or disease activity.

**Objectives:** To investigate the prevalence of high IFN signature in low disease activity jSLE, and to assess whether toll like receptor (TLR) 7 or 9 dependent IFNα production pathways differ between high and low IFN signature patients.

**Methods:** Blood was collected, with consent, from healthy volunteers (n=24:10 female; 14 male, age=12-19) and young people with jSLE (n=29:20 female; 9 male, age=14-21). Clinical data were recorded. Peripheral blood mononuclear cells (PBMC) were separated by Ficoll gradient centrifugation. Ex vivo RNA was assessed by Nanostring Plex Set. IFN score was calculated from counts of 5 IFN inducible genes (MX1+BST2+MCP1+ISG15+ IFIT1/5). Samples were classed as IFN positive (IFN+) if >2SD from the healthy mean. Separately, PBMC were stimulated with TLR7 agonist, R848, or TLR9 agonist, CPGODN2216, before assessing for secretion of IFNα and TNFα by luminex. Statistical analysis was performed using SPSS with linear regression.

**Results:** There was a significantly higher IFN score in jSLE than healthy controls (p=0.001). 14/29 (48.27%) jSLE patients were IFN+ vs 1/23 (0.04%) healthy controls (p=0.005). Patients with jSLE had a mean SLEDAI of 3.0 (range=0-12); 15 (52%) were on prednisone (mean= 6.9mg). In patients with jSLE, the only autoantibody that significantly predicted IFN score was towards RNP (p=0.018). There was no association between any specific organ involvement and IFN score. Four patients with jSLE were B cell depleted (<2%), with no effect of B cell depletion on IFN score. There were no significant differences in markers of disease activity (SLEDAI, CRP, ESR, C3, C4 and double stranded DNA antibodies) between IFN+ and IFN- jSLE patients. Only jSLE patients who were IFN+ produced significantly more IFNα (p=0.046) and TNFα (p=0.031) than healthy controls after TLR7 stimulation. After TLR9 stimulation, however, IFNα production was decreased in jSLE compared to healthy, regardless of IFN score. IFN+ patients with jSLE had significantly higher PBMC RNA expression of TLR7 than those who were IFN- (p=0.001), and healthy controls (p=0.04). IFN+ patients with jSLE showed a significant decrease in TLR9 expression when compared to healthy controls (p=0.023). We confirmed that TLR7 was not an IFN inducible marker, by assessing expression in healthy controls with and without IFNα pre-stimulation, which showed no significant difference.

**Conclusion:** We present novel data, showing that, in non-flaring patients with jSLE on low doses of steroid, half are IFN signature positive. This is lower than reported in studies that include flaring patients and may better represent prevalence of high IFN signature disease at baseline. In jSLE, IFN signature score associates with autoantibodies to RNP. PBMC from IFN+ patients produce more IFNα after TLR7 stimulation, and have a significantly higher expression of TLR7, along with a decrease in TLR9 expression compared to samples from patients with a negative IFN signature, and healthy controls.

Disclosure of Interest

None Declared

### O22 STERILE INFLAMMATORY ACTIVATION OF HUMAN CORONARY ARTERY ENDOTHELIAL CELLS DEPENDS STRICTLY ON INTERLEUKIN 1Β: IMPLICATIONS FOR KAWASAKI DISEASE PATHOLOGY

#### Giulia Armaroli^1^, Emely Verweyen^1^, Carolin Pretzer^1^, Katharina Kessel^1^, Keiichi Hirono^2^, Fukiko Ichida^2^, Mako Okabe^2^, David Cabral^3^, Dirk Foell^1^, Kelly Brown^3^, Christoph Kessel^1^

##### ^1^Pediatric Rheumatology & Immunology, University Children’s Hospital, Muenster, Germany; ^2^Department of Pediatrics, University of Toyama, Toyama, Japan; ^3^Department of Pediatrics, University of British Columbia, Vancouver, Canada

###### **Correspondence:** Christoph Kessel

**Introduction:** The granulocytic protein S100A12 is expressed at high levels in Kawasaki Disease (KD) but it is unclear whether S100A12 does actively participate in KD pathology or is a bystander of neutrophil activation.

**Objectives:** Kawasaki disease (KD) is an acute vasculitis of childhood, predominantly affecting coronary arteries. S100A12 is an endogenous pattern recognition receptor (PRR) ligand, which is strongly upregulated in KD and S100A12 serum levels track with KD disease activity and response to intravenous immunoglobulin (IVIG) treatment. While S100A12 was originally described as agonist of the multi-ligand receptor for glycation endproducts (RAGE), we described human monocytes to respond to S100A12-stimulation in an exclusively TLR4-dependent manner. Here, we aimed to investigate whether and how serum S100A12 might be actively involved in inflammatory processes in KD.

**Methods:** Serum samples (n=75) from patients with KD at different stages pre- and post-intravenous immunoglobulin (IVIG) treatment were analyzed for S100A12, cytokines, chemokines and soluble markers of endothelial activation. Primary human coronary artery endothelial cells (HCAECs) were analyzed for responsiveness following direct stimulation with S100A12 or lipopolysaccharide (LPS). Alternatively, HCAEC were cultured in conditioned medium obtained from primary human monocytes stimulated with LPS or S100A12 in the absence or presence of IVIG or cytokine antagonists.

**Results:** Multiple correlation analyses revealed, that in KD patients’ sera in course of IVIG-therapy soluble vascular cell adhesion molecule-1 (sVCAM-1) titers as indicator of endothelial activation exclusively associated with pre-treatment S100A12 levels. Yet, in contrast to stimulations with LPS, HCAECs were not responsive to direct treatment with S100A12, despite the presence of appropriate receptors (RAGE, TLR4). Unresponsiveness of HCAECs to S100A12 was due to lack of membrane CD14 expression. Addition of recombinant soluble CD14 or human serum benefitted LPS- but could not establish response to S100A12. Instead, HCAECs strongly responded to supernatants obtained from S100A12-stimulated primary human monocytes, as evidenced by expression of inflammatory cytokines and adhesion molecules. Inflammatory activation of HCAECs by S100A12-stimulated monocytes required monocyte-co-stimulation with ATP. Endothelial cell stimulation was completely abrogated upon IL-1β blockade but remained unaffected by treatment with IVIG or anti-TNFa and IL-6 receptor blocking antibodies.

**Conclusion:** We identified IL-1β-signaling in coronary artery endothelium as particularly relevant in course of sterile inflammation as induced by damage associated molecular pattern (DAMP) molecules such as S100A12. These data are in support of a potential key role for IL-1 in KD pathology but also highlight the coronary artery endothelium, which is critically involved in early KD pathology and aneurysm formation as particularly sensitive to IL-1β blockade during DAMP-induced sterile inflammation.

**Disclosure of Interest**

None Declared

## Autoinflammatory mixture

### O23 LONG-TERM EFFICACY AND SAFETY OF CANAKINUMAB-TREATED PATIENTS WITH COLCHICINE-RESISTANT FMF, TRAPS AND HIDS/MKD: RESULTS FROM THE PHASE 3 CLUSTER TRIAL

#### F. De Benedetti^1^, J. Frenkel^2^, A. Simon^3^, J. Anton^4^, H. Lachmann^5^, M. Gattorno^6^, S. Ozen^7^, I. Kone-Paut^8^, E. Ben-Chetrit^9^, M. B. Wozniak^10^, J. G. Wang^11^, E. Vritzali^11^

##### ^1^IRCCS Ospedale Bambino Gesú, Rome, Italy; ^2^University Medical Center Utrecht, Utrecht; ^3^Radboud University Medical Centre, Nijmegen, Netherlands; ^4^Hospital Sant Joan de Déu, Barcelona, Spain; ^5^UK National Amyloidosis Centre, University College London Medical School, London, UK; ^6^Pediatric Rheumatology, G. Gaslini Institute, Genoa, Italy; ^7^Hacettepe University, Ankara, Turkey; ^8^Hôpital Kremlin Bicetre, University of Paris SUD, Paris, France; ^9^Hadassah—Hebrew University Medical Center, Jerusalem, Israel; ^10^Novartis Ireland Ltd, Dublin, Ireland; ^11^Novartis Pharma AG, Basel, Switzerland

###### **Correspondence:** F. De Benedetti

**Introduction:** Canakinumab (CAN) has demonstrated efficacy and safety in patients with colchicine-resistant familial Mediterranean fever (crFMF), TNF receptor-associated periodic syndrome (TRAPS), and hyper-IgD syndrome (HIDS)/mevalonate kinase deficiency (MKD) in epoch 2 and 3 (E2 and E3 up to week 40) of the pivotal, randomised CLUSTER study.^1^

**Objectives:** To assess long-term maintenance of optimal control of disease activity (median of no or 1 flare, and no uptitration) and safety of CAN dosing regimens of every 4 weeks (q4w) and every 8 weeks (q8w) in patients with crFMF, TRAPS or HIDS/MKD from the epoch 4 (E4 up to week 112) of CLUSTER.

**Methods:** CLUSTER comprised 4 epochs (E1-E4). After lead-in E1, efficacy of CAN 150/300 mg q4w to induce complete response was assessed in E2, a 16-week randomised, double-blind, placebo (PBO)-controlled epoch. E3, a 24-week randomised withdrawal epoch, evaluated whether responders to CAN 150/300 mg q4w in E2 could maintain clinical efficacy on 150/300 mg q8w or PBO. In E4, a 72-week, open-label epoch, the long-term maintenance of efficacy and safety of CAN 150/300 mg q4w or q8w in patients with crFMF, TRAPS or HIDS/MKD was evaluated. Patients who did not maintain clinical response on q8w could be uptitrated to 150/300 mg q4w. Safety assessments included incidence of adverse events (AEs) and serious AEs.

**Results:** Five patients discontinued from the study in E4 (one TRAPS patient discontinued due to AE). A substantial proportion of patients maintained optimal control of disease activity following treatment with 150/300 mg q4w or q8w in all 3 cohorts at week 112 (Table 1). HIDS/MKD patients more often required uptitration to 300 mg q4w. Majority of patients had 1 or no new flare (crFMF: 96.6%, TRAPS: 94.3%, HIDS/MKD: 83.3%). In all 3 cohorts, the median SAA levels decreased rapidly from baseline and remained suppressed through E4 (crFMF: 618 to 21 mg/L, TRAPS: 243 to 12 mg/L and HIDS/MKD: 2061 to 16 mg/L). No new safety findings were reported in CAN-treated patients through week 112.

**Conclusion:** Long-term treatment with canakinumab 150/300 mg q4w in epoch 4 of the CLUSTER study showed that optimal control of disease activity can be maintained in patients with crFMF, TRAPS or HIDS/MKD. There were no new or unexpected safety issues reported over 112 weeks of treatment.

**References**

1. De Benedetti F, et al. *NEJM* 2018, in press.

**Trial registration identifying number:** NCT02059291

**Disclosure of Interest**

F. De Benedetti Grant / Research Support from: Novartis, Roche, Pfizer, SOBI, AbbVie, Novimmune, BMS, Sanofi, J. Frenkel Grant / Research Support from: Novartis and SOBI, A. Simon Grant / Research Support from: Novartis, Xoma/Servier, CSL Behring, Consultant for: Novartis, Takeda, SOBI, Xoma, J. Anton Grant / Research Support from: Novartis, Pfizer, Abbvie, GSK, BMS, Roche, Sanofi, Consultant for: Novartis, Sobi, Roche, Pfizer, Abbvie, H. Lachmann Consultant for: Novartis, SOBI, Takeda and GSK, Speaker Bureau of: Novartis and SOBI, M. Gattorno Grant/Research Support from: Novartis and SOBI, Consultant for: Novartis and SOBI, Speaker Bureau of: Novartis and SOBI, S. Ozen Speaker Bureau of: Novartis and SOBI, I. Kone-Paut Grant/Research Support from: Novartis, SOBI and Roche, Consultant for: Novartis, SOBI, Pfizer, AbbVie and Roche, E. Ben-Chetrit Consultant for: Novartis, M. B. Wozniak Employee of: Novartis, J. G. Wang Employee of: Novartis, E. Vritzali Employee of: Novartis

**Table 1 (abstract O23).** Proportion of responders who maintained optimal control of disease activity* at the end of epoch 4, following treatment with canakinumab (Week 112 analysis)

Cohort	150 mg q8w n (%)	150 mg q4w n (%)	300 mg q8w n (%)	300 mg q4w n (%)
crFMF (N=57)	23 (40.4)	20 (35.1)	5 (8.8)	9 (15.8)
TRAPS (N=50)	25 (50.0)	5 (10.0)	9 (18.0)	11 (22.0)
HIDS/MKD (N=64)	14 (21.9)	7 (10.9)	11 (17.2)	32 (50.0)

*Optimal control of disease activity was defined as median of no or 1 flare, and no uptitration

## Epigenetic in inflammation and autoimmunity

### O24 EXPRESSION OF THE INTERLEUKIN 1 FAMILY MEMBERS IL-1B AND IL-18 BY HUMAN MONOCYTES IS CONTROLLED BY DIFFERENT REGULATORY PATHWAYS

#### Emely Verweyen^1^, Melissa van Dülmen^1^, Dirk Holzinger^2^, Dirk Foell^1^, Christoph Kessel^1^

##### ^1^Pediatric Rheumatology and Immunology, University Children's Hospital, Münster; ^2^Department of Child and Adolescent Medicine, University Hospital, Essen, Germany

###### **Correspondence:** Emely Verweyen

**Introduction:** Regulation of IL-18 gene expression by primary human monocytes is poorly understood but may provide insights into the cytokine’s still unclear role in autoinflammation.

**Objectives:** The interleukin (IL) 1 family cytokine IL-18 is overabundant in plasma and serum of familial mediterranean fever (FMF) and systemic juvenile idiopathic arthritis (sJIA) patients and its levels coincide with hypersecretion of the damage associated pattern (DAMP) proteins S100A8/A9 and S100A12. Comparable to IL-1b it is thought to require caspase-dependent processing to render an immature pro-form into bioactive mature IL-18. Yet, in stark contrast to IL-1b, it seems largely unknown whether and how IL-18 gene expression is regulated.

**Methods:** Primary human monocytes isolated from healthy donors were analyzed for kinetics of cytokine expression on gene and protein level following different stimulations and drug treatments.

**Results:** Initially, we performed endotoxin desensitization experiments on primary human monocytes, which suggested TNFa, IL-6 and IL-1b but not IL-18 gene expression to be subjected to LPS-tolerance. However, when studying time kinetic gene expression of cytokines, we found that IL-1b and IL-18 followed identical secretion but completely different gene expression patterns. In contrast to IL-1b, we observed human monocytes to maintain a cytoplasmic store of mature, bioactive IL-18 from which it could be readily secreted upon LPS stimulation and, following the observed attenuated IL-18 gene expression, this intracellular bioactive IL-18 store replenished. Importantly, colchicine as the mainstay of todays FMF-therapy, as well as the the microtubule polymerisation inhibitor nocodazole abrogated IL-18 expression, while IL-1b gene expression was massively enhanced.

**Conclusion:** We propose monocytic IL-18 transcription to be subjected to negative feedback regulation by an intracellular store of IL-18 protein. The cellular efforts to maintain such a store may suggest IL-18 to rather operate as DAMP than classical cytokine. This may explain the orchestrated hypersecretion of IL-18, S100A8/A9 and A12 in diseases such as FMF or sJIA.

**Disclosure of Interest**

None Declared

### O25 SUPPRESSION OF T-CELL ACTIVATION VIA SIRT1 INHIBITION USING NICOTINAMIDE (VITAMIN B3)

#### Lotte Nijhuis^1^, Isabelle Houtzager^1^, Arief Lalmohamed^2^, Bas Vastert^3^, Jorg Van Loosdregt^1^

##### ^1^Laboratory of Translational Immunology; ^2^Department of Clinical pharmacy; ^3^Department of Pediatric Rheumatology, UMC UTRECHT, Utrecht, Netherlands

###### **Correspondence:** Lotte Nijhuis

**Introduction:** Juvenile Idiopathic Arthritis is hallmarked by a disturbed immunological balance between regulatory T-cells (Treg) and effector T-cells (Teff). Restoring this balance by either enhancing the suppressive function of Treg or inhibiting activation and proliferation of pro-inflammatory T-eff is therefore a promising therapeutic strategy. Previously we demonstrated that inhibition of the lysine deacetylase SIRT1 resulted in an increase in FOXP3 positive cells *in vitro*. Since the transcription factor FOXP3 is essential for Treg differentiation and function, this approach could positively influence immunological tolerance. Interestingly, SIRT1 can be inhibited by the well known food additive, Vitamin B3, also known as Nicotinamide (NAM). We therefore aim to translate these laboratory findings into clinical practice and envision a role for NAM as a novel therapeutic strategy in maintaining the immunological balance in patients with JIA. We demonstrated a distinctive effect of NAM on Treg, however the effect of NAM on the other side of the balance; Teff cells is still unexplored.

**Objectives:** To investigate the effect of SIRT1 inhibition on proliferation, activation and cytokine production of Teff cells.

**Methods:** T-cells were isolated from the blood of healthy controls and JIA patients as well as the synovial fluid from JIA patients. Cells were stimulated with aCD3/aCD28 and cultured in the presence of increasing concentrations of NAM (0-9mM) for 1-4 days. Proliferation and expression of activation makers were determined using flow cytometry. Cytokine production was determined by qPCR, luminex and flow cytometry. The specific SIRT1 inhibitor EX-527 was used as a control to determine the pathway involved.

**Results:** *In vitro* NAM treatment of human CD4+ T-cells significantly decreased the production of the pro-inflammatory cytokines IL-2 and IFNy measured both on mRNA and protein level. In line with this, surface activation markers were downregulated after NAM incubation. Furthermore proliferation of both CD4+ and CD8+ T-cells was inhibited with NAM treatment in a dose dependent manner in both PBMC from HC and SFMC from JIA patients. Results were verified by using another SIRT 1 inhibitor; EX-527.

**Conclusion:** In addition to the previously demonstrated increase of Treg numbers and function, these data demonstrate that NAM treatment *in vitro* inhibits proliferation and activation of Teff cells. Therefore NAM treatment could modulate the immunological balance by both increasing tolerance and suppressing immune activation. We envision that NAM treatment as an adjuvant therapy has the potential to benefit JIA patients and potentially other autoimmune diseases. To translate these findings to clinical practice and determine if NAM treatment is useful as a strategy to maintain disease remission after stopping DMARD or biologicals we are in preparation of a phase III clinical trial.

**Disclosure of Interest**

None Declared

## Poster Walk 1: Systemic JIA and MAS

### P001 NEXT GENERATION SEQUENCING ANALYSIS OF FAMILIAL HAEMOPHAGOCYTIC LYMPHOHISTIOCYTOSIS (HLH) RELATED GENES IN MACROPHAGE ACTIVATION SYNDROME (MAS) AND SECONDARY HLH (SHLH)

#### Chiara Passarelli^1^, Manuela Pardeo^2^, Ivan Caiello^2^, Francesca R. Lepri^1^, Antonella Insalaco^2^, Francesca Minoia^3^, Andrea Taddio^4^, Francesco Licciardi^5^, Antonio Novelli^1^, Fabrizio De Benedetti^2^, Claudia Bracaglia^2^

##### ^1^Unit of Laboratory of Medical Genetics; ^2^Division of Rheumatology, IRCCS-Paediatric Hospital Bambino Gesù, Rome; ^3^Reumatologia Pediatrica, IRCCS Istituto Giannina Gaslini, Genoa; ^4^Institute for Maternal and Child Health - IRCCS “Burlo Garofolo”, University of Trieste, Trieste; ^5^SCDU Pediatria II, Immunoreumatologia, Ospedale Pediatrico Regina Margherita, Turin, Italy

###### **Correspondence:** Claudia Bracaglia

**Introduction:** Macrophage activation syndrome (MAS), a severe complication of paediatric rheumatic disease, is currently classified among the secondary forms of HLH (sHLH). Primary HLH (pHLH) are caused by mutation of genes coding for proteins involved in cytotoxic functions. Mice carrying heterozygous mutations in more than 1 pHLH gene carry a higher risk to develop HLH following viral infection, suggesting that accumulation of partial genetic defects may be relevant in HLH.

**Objectives:** To analyse, with next generation sequencing (NGS), genes involved in pHLH in MAS in the context of different rheumatic diseases and in sHLH.

**Methods:** We performed Targeted resequencing on all patients using a panel including the 7 principal HLH-related genes (*PRF1, UNC13d, STX11, STXBP2, Rab27a, XIAP, SH2D1A*) on MiSeq® and NextSeq550® platforms (Illumina, San Diego, CA); all variants identified were confirmed by Sanger. We took into account variants with an allelic frequency in the global population up to 5% in the dbSNP and Ensembl databases.

**Results:** We analysed 125 patients: 47 MAS (40 developed this complication in the context of systemic Juvenile Idiopathic Arthritis (sJIA) and 7 in the context of different rheumatic diseases), 32 sHLH, 22 sJIA (without history of MAS) and 24 with different autoinflammatory diseases (AID). sJIA and AID patients were used as control groups.

We identified at least 1 heterozygous variant in one of the pHLH-related genes in 41 patients with a detection rate of 52%, 45% of MAS and 62% of sHLH patients. More than one variant was identified in 37% patients from both groups, with 19% of both MAS and sHLH patients carrying polygenic variants. In control groups, 54% of sJIA and 33% of AID patients carry at least a variant in the analysed genes, while polygenic variants have been detected in 14% and 8% of control patients, respectively. The most involved genes in both MAS and sHLH groups were *PRF1* and *UNC13d*, while variants in *RAB27a* and *XIAP* have been found only in sHLH patients. The most frequent variants identified in both groups were A91V in *PRF1* gene and R928C in *UNC13d* gene. The A91V variant in *PRF1* gene was identified in 19% of both MAS and sHLH patients, while this variant was present, respectively, in only 5% of sJIA and 4% of AID patients. The R928C variant in *UNC13d* gene was identified in 32% of MAS and 18% of sHLH patients, and in the control group in 9% of sJIA and 17% of AID patients. Considering the patients’ clinical characteristics, relapse, CNS involvement, ICU admission and death, in sHLH we observed that three of the 6 patients (50%) carrying multiple variants had recurrent episodes of HLH and that two of them (33%) presented a severe disease with exitus.

**Conclusion:** Monoallelic variants in pHLH-related genes are more frequent in MAS, sHLH and sJIA and less in AID patients, suggesting different molecular mechanisms involved in the diseases.

Re-occurrence and severity of disease seem to be more frequent and more severe in patients who carry mutations in two genes in sHLH group. These data may support a polygenic model of sHLH.

**Disclosure of Interest**

C. Passarelli: None Declared, M. Pardeo: None Declared, I. Caiello: None Declared, F. Lepri: None Declared, A. Insalaco: None Declared, F. Minoia: None Declared, A. Taddio: None Declared, F. Licciardi: None Declared, A. Novelli: None Declared, F. De Benedetti Grant/Research Support from: Novartis, Novimmune, Hoffmann- La Roche, SOBI, AbbVie, Pfizer, C. Bracaglia: None Declared

### P002 RISK SCORE OF MACROPHAGE ACTIVATION SYNDROME IN PATIENTS WITH SYSTEMIC JUVENILE IDIOPATHIC ARTHRITIS

#### Simone Carbogno^1^, Denise Pires Marafon^2^, Giulia Marucci^3^, Manuela Pardeo^3^, Antonella Insalaco^3^, Virginia Messia^3^, Rebecca Nicolai^3^, Fabrizio De Benedetti^3^, Claudia Bracaglia^3^

##### ^1^Pediatric Area, University of Milan; ^2^Pediatric Unit, Fondazione IRCCS Ca' Grande Ospedale Maggiore Policlinico, Milan; ^3^Division of Rheumatology, IRCCS Ospedale Pediatrico Bambino Gesù, Rome, Italy

###### **Correspondence:** Simone Carbogno

**Introduction:** Macrophage Activation Syndrome (MAS) is a severe, life-threatening, complication of rheumatic diseases in childhood, particularly of systemic Juvenile Idiopathic Arthritis (sJIA), occurring in approximately 25% of the patients with sJIA. The mortality rate of MAS is still significantly high. A score that identify sJIA patients who are at high risk to develop MAS would be useful in clinical practice. There are no parameters available to identify from onset sJIA patients with high risk to develop MAS in their disease course.

**Objectives:** To evaluate whether routine laboratory parameters at disease onset may predict the development of MAS in patients with active sJIA. To define a risk score of MAS for sJIA patients using these parameters.

**Methods:** Laboratory parameters of disease activity and severity (WBC, N, PLT, Hb, ferritin, AST, ALT, gGT, LDH, TGL, fibrinogen, D-dimer and CRP), were retrospectively evaluated in 56 sJIA patients referred to our Division of Rheumatology from 1998 to 2016 with at least one year of follow-up. Laboratory parameters were evaluated during active sJIA, without MAS, at time of hospitalization (T1) and before treatment for sJIA was started (T2). Patients were divided in two groups: group 1 (patients without history of MAS), group 2 (patients with at least one MAS episode during disease course). To calculate a MAS risk score, laboratory parameters, collected at T2, with a statistical significant difference between the two groups of patients were selected.

**Results:** Fourteen patients, that fulfilled the 2016 classification criteria for MAS [1] at time of sampling, were excluded from the analysis. Therefore, we analyzed laboratory parameters of 42 patients with sJIA, 27 of whom without history of MAS (group 1) and 15 who developed at least one episode of MAS during disease course (group 2). Levels of ferritin, AST, LDH, gGT and TGL, collected at T2, were statistically significant higher in patients with a history of MAS compared to those without a history of MAS. For each of these parameters an arbitrary cut-off was defined. In order to define the final score an arbitrary rate was attributed to each parameter. Sensitivity (Se), specificity (Sp), positive predictive value (PPV) and negative predictive value (NPV) were calculated to define the best scoring system. The scoring system with the best sensitivity was chosen (Table 1). A MAS risk score >3 identified 14 out of 15 sJIA patients with a history of MAS and 3 out of 27 sJIA patients without history of MAS.

**Conclusion:** In conclusion we developed a MAS risk score based on routine laboratory parameters that are available worldwide, that can help clinicians to identify these patients early in the disease course. Our results need to be validated in a larger population.

**Reference**

1. Ravelli A et al. 2016 Classification Criteria for Macrophage Activation Syndrome Complicating Systemic Juvenile Idiopathic Arthritis: A European League Against Rheumatism/American College of Rheumatology/Paediatric Rheumatology International Trials Organisation Collaborative Initiative. Ann Rheum Dis. 2016 Mar;75(3):481-9.

**Disclosure of Interest**

S. Carbogno: None Declared, D. Pires Marafon: None Declared, G. Marucci: None Declared, M. Pardeo: None Declared, A. Insalaco: None Declared, V. Messia: None Declared, R. Nicolai: None Declared, F. De Benedetti Grant/Research Support from: Novartis, Novimmune, Hoffmann-La Roche, SOBI, AbbVie, Pfizer, C. Bracaglia: None Declared

**Table 1 (abstract P002).** Laboratory parameters and cut-off used to create the MAS risk score in sJIA patients

Laboratory parameters	Cut-off	Rate
Ferritin (ng/ml)	>900	1
AST (UI/L)	>35	1
LDH (UI/l)	>550	1
gammaGT (UI/L)	>30	2
Triglycerides (mg/dl)	>150	2
Sensitivity (Se)	0.933	CI95% 0.680-0.998
Specificity (Sp)	0.889	CI95% 0.708-0.977
Positive predictive value (PPV)	0.824	CI95% 0.566-0.962
Negative predictive value (NPV)	0.96	CI95% 0.797-0.999

### P003 IDENTIFICATION OF OPTIMAL SUBCUTANEOUS DOSES OF TOCILIZUMAB IN CHILDREN WITH SYSTEMIC JUVENILE IDIOPATHIC ARTHRITIS

#### Fabrizio De Benedetti^1^, Nicola Ruperto^2^, Daniel Lovell^3^, Gerd Horneff^4^, Maria Luz Gámir Gámir^5^, Markus Hufnagel^6^, Joy C. Hsu^7^, Min Bao^7^, Wendy Douglass^8^, Navita L. Mallalieu^7^, Chris Wells^8^, Christopher M. Mela^8^, Hermine Brunner^3^ and PRINTO and PRCSG Investigators

##### ^1^IRCCS Ospedale Pediatrico Bambino Gesù, Rome; ^2^Pediatric Rheumatology International Trial Organization (PRINTO), Istituto Giannina Gaslini Pediatria II–Reumatologia, Genoa, Italy; ^3^Pediatric Rheumatology Collaborative Study Group (PRCSG), Cincinnati Children’s Hospital Medical Center, Cincinnati, USA; ^4^Asklepios Clinic Sankt Augustin and University Hospital of Cologne, Sankt Augustin and Cologne, Germany; ^5^Hospital Ramon y Cajal Unidad de Reumatologia Pediatrica, Madrid, Spain; ^6^Universitätsklinikum Freiburg, Freiburg, Germany; ^7^Roche Innovation Center, New York, USA; ^8^Roche Products Ltd, Welwyn Garden City, UK

###### **Correspondence:** Fabrizio De Benedetti

**Introduction:** The efficacy and safety of intravenous (IV) tocilizumab (TCZ) were demonstrated in patients (pts) with systemic juvenile idiopathic arthritis (sJIA) in the phase 3 TENDER study.^1^

**Objectives:** To investigate dosing regimens of subcutaneous (SC) TCZ in pts with sJIA by bridging from TENDER data for TCZ IV to identify the optimal SC regimen through characterization of the pharmacokinetics (PK), pharmacodynamics (PD), and safety of TCZ SC in pts with sJIA; efficacy was an exploratory objective.

**Methods:** This was a multicenter, open-label, phase 1b study to evaluate the PK, PD, and safety of TCZ SC in pts aged 1-17 years with sJIA and inadequate response to glucocorticoids. Interim analysis was conducted after 24 pts had received TCZ SC for 14 weeks. Pts could be TCZ naive or could switch from TCZ IV to SC. TCZ SC was administered for 52 weeks according to body weight: pts <30 kg received either 162 mg every 10 days before interim analysis or 162 mg every 2 weeks (Q2W) after interim analysis; pts ≥30 kg received 162 mg every week (QW).

**Results:** We enrolled 51 pts, including 25 weighing <30 kg (8 before and 17 after interim analysis) and 26 weighing ≥30 kg. Twenty-six pts (51%) were TCZ naive and 25 (49%) switched from TCZ IV. Median steady state C_min_ was similar for pts <30 kg receiving TCZ 162 mg Q2W and pts ≥30 kg receiving TCZ 162 mg QW; the ranges largely overlapped (Table 1). More than 95% (49/51) of pts treated with TCZ SC had steady state C_min_ higher than the 5th percentile achieved with TCZ IV. The median and range of AUC_2weeks_ were similar for both body weight groups (Table 1). Changes in interleukin-6, C-reactive protein, and erythrocyte sedimentation rate were similar for both body weight groups. Almost all pts had ≥1 adverse event (AE; n = 50; 98.0%). Injection site reactions (ISRs) occurred in 21 pts (41.2%); most were mild, and none led to treatment interruption or withdrawal. The AE rate was 1200.3/100 pt-years (PY) (909.3/100 PY, excluding ISRs). The most common AEs were viral upper respiratory tract infection (13; 25.5%), neutropenia (13; 25.5%), and cough (12; 23.5%). Nine serious AEs occurred in 7 pts (13.7%; 19.3/100 PY); 5 were infections, all in the <30 kg group. Two deaths occurred, both in the <30 kg group. Median JADAS-71 improved from baseline to week 52 for TCZ-naive pts (<30 kg, –13.9; ≥30 kg, –12.4) and was maintained or improved further for pts who switched from TCZ IV (<30 kg, –0.7; ≥30 kg –0.2). At week 52, 29/43 pts (67.4%) had inactive disease (JADAS-71 <1.0).

**Conclusion:** A PK-based strategy was successful in bridging TCZ SC to TCZ IV in pts with sJIA. Dosing regimens of 162 mg Q2W in pts <30 kg and 162 mg QW in pts ≥30 kg provided adequate exposure to support efficacy comparable to that of TCZ IV. Except for ISRs, safety results were consistent with the known safety profile of TCZ in sJIA.

**Reference**

1. De Benedetti F et al. *N Engl J Med*. 2012;367:2385-95.

**Trial registration identifying number:** ClinicalTrials.gov NCT01904292

**Disclosure of Interest**

F. De Benedetti Grant/Research Support from: Novartis, Roche, Pfizer, SOBI, AbbVie, Novimmune, BMS, Sanofi, N. Ruperto Consultant for: AbbVie, Amgen, Biogenidec, Alter, AstraZeneca, Baxalta Biosimilars, Biogenidec, Boehringer, BMS, Celgene, CrescendoBio, EMD, Speaker Bureau of: AbbVie, Amgen, Biogenidec, Alter, AstraZeneca, Baxalta Biosimilars, Biogenidec, Boehringer, BMS, Celgene, CrescendoBio, EMD, D. Lovell Grant/Research Support from: National Institutes of Health, NIAMS, Consultant for: AstraZeneca, Bristol-Myers Squibb, AbbVie, Pfizer, Roche, Novartis, UCB, Forest Research Institute, Horizon, Johnson & Johnson, Biogen, Takeda, Genentech, GlaxoSmithKline, Boehringer Ingelheim, Celgene, Janssen, Speaker Bureau of: Genentech, G. Horneff: None Declared, M. L. Gámir Gámir: None Declared, M. Hufnagel: None Declared, J. C. Hsu Employee of: Roche, M. Bao Employee of: Roche, W. Douglass Employee of: Roche, N. L. Mallalieu Employee of: Roche, C. Wells Shareholder of: Roche, Employee of: Roche, C. M. Mela Employee of: Roche, H. Brunner: None Declared

**Table 1 (abstract P003).** See text for description

	TCZ SC		TCZ IV	
	<30 kg TCZ 162 mg Q2W n = 25	≥30 kg TCZ 162 mg QW n = 26	<30 kg TCZ 12 mg/kg Q2W n = 46	≥30 kg TCZ 8 mg/kg Q2W n = 43
Model-computed steady state PK parameters, median (range)				
C_min,ss_ μg/mL	64.2 (16.6-135.9)	72.4 19.5-157.8)	65.9 (19.0-135.5)	70.7 (5.3-126.6)
C_max,ss_ μg/mL	126.6 (51.7-265.8)	89.8 (26.4-190.2)	274.4 (148.8-444.0)	253.0 (119.6-404.3)
AUC_2weeks,ss_ μg/mL×day	1298 (539-2792)	1154 (334-2370)	1734 (840-2712)	1631 (526-2779)

### P004 INTERSTITIAL LUNG DISEASE IN SYSTEMIC JUVENILE IDIOPATHIC ARTHRITIS PATIENTS IN THE PHARMACHILD REGISTRY

#### Jelle De Groot, Bas Vastert, Gabriella Giancane, Michael Hofer, Ekaterina Alexeeva, Violeta Panaviene, Susan Nielsen, Jordi Anton, Florence Uettwiller, Valda Stanevicha, Maria Trachana, Denise P. Marafon, Constantin Ailioaie, Elena Tsitsami, Troels Herlin, Pavla Dolezalova, Gordana Susic, Berit Flato, Flavio Sztajnbok, Angela Pistorio, Francesca Bagnasco, Alberto Martini, Nico Wulffraat, Nicola Ruperto, Joost Swart

##### PRINTO, Genova, Italy

###### **Correspondence:** Jelle De Groot

**Introduction:** Lung disease might be an underreported complication in patients with systemic juvenile idiopathic arthritis (SJIA). Recently a study describing 25 patients with interstitial lung disease (ILD), pulmonary arterial hypertension (PAH) and alveolar proteinosis (AP) emerged. To what extent these complications occur in the SJIA population is still uncertain.^1^

**Objectives:** This study will investigate how often pulmonary complications occur in children with SJIA in the Pharmachild registry. Possible associated factors and/or complications of lung disease will also be investigated.

**Methods:** Data of the Pharmachild registry, the world's largest pharmacovigilance database, was used. 914 Patients with SJIA were included. Focus lied primarily on pulmonary complications reported in these children. Secondarily, possible correlated events like the macrophage activation syndrome (MAS), death and adverse drug reactions will be analysed.

**Results:** Eighty pulmonary complications were found in 65/914 patients suffering from SJIA (7%). Complications highly suspect for ILD were found in only 9 children (1%). Patients with pulmonary complications had significantly longer follow-up (7.6 years versus 5.1), were younger at diagnosis (3.7 years versus 5.3 years), had significantly more often non-pulmonary complications and were more likely to have ever used Tocilizumab (55.4% to 40.6%) and/or Rituximab (30.8% to 5.8%) (see Table 1).

MAS occurred more often in patients with pulmonary complications (20.0% to 11.5% P=0.050) and they had significantly more adverse drug reactions, especially to Tocilizumab (9.2% to 2.7% p=0.013) and Rituximab (12.3% to 1.2% P<0.001). Fatal disease occurred more often in patients with pulmonary complications compared to those without it (4.6% to 0.6% p= 0.006).

**Conclusion:** Pulmonary complications were found in 7% of the SJIA population. Pulmonary complications are also associated with significant more non-pulmonary comorbidities like MAS, adverse drug reactions and even death. More adequate monitoring of pulmonary function and consistent documentation of lung disease might be helpful especially in patients at highest risk of ILD.

**References**

1) Kimura Y, Weiss JE, Haroldson KL, Lee T, Punaro M, Oliveira S, et al. Pulmonary hypertension and other potentially fatal pulmonary complications in systemic juvenile idiopathic arthritis. Arthritis Care Res (Hoboken). 2013 May;65(5):745-52. doi: 10.1002/acr.21889.

**Trial registration identifying number:** NCT01399281

**Disclosure of Interest**

None Declared

**Table 1 (abstract P004).** Patient characteristics

	Patients with pulmonary complications	Patients without pulmonary complications	P-value
Patient number	65	849	
Observation years total (IQR)	7.6 (4.3-10.2)	5.1 (2.4-9.1)	0.002
Age diagnosis SJIA (IQR)	3.7 (2.3-5.6)	5.3 (2.9-9.3)	<0.001
Patients with non-pulmonary AE/ESI	56 (86.2%)	287 (33.8%)	<0.001
Synthetic DMARDs ever	52 (80.0%)	690 (81.3%)	0.745
Anti-TNF ever	25 (38.5%)	307 (36.2%)	0.691
Anti-IL1 ever	29 (44.6%)	332 (39.1%)	0.430
Tocilizumab ever	36 (55.4%)	345 (40.6%)	0.026
Rituximab ever	20 (30.8%)	49 (5.8%)	<0.001

Values are the number of patients, unless stated otherwise. Continuous variables are analysed by a Mann-Witney-U test, asymp. Sig. (2-tailed). For dichotomous variables Fisher’s exact sig. (2-sided) is used

### P005 THE INTERFERON-GAMMA PATHWAY IS ACTIVATED IN THE LIVER AND IN THE BLOOD OF PATIENTS WITH ACTIVE SECONDARY HEMOPHAGOCYTIC LYMPHOHISTIOCYTOSIS

#### Giusi Prencipe^1^, Claudia Bracaglia^1^, Antonia Pascarella^1^, Ivan Caiello^1^, Paola Francalanci^1^, Manuela Pardeo^1^, Alessandra Meneghel^2^, Giorgia Martini^2^, Antonella Insalaco^1^, Giulia Marucci^1^, Francesco Zulian^2^, Fabrizio De Benedetti^1^

##### ^1^Bambino Gesù Children's Hospital, Rome; ^2^University of Padua, Department of Woman and Child Health, Padua, Italy

###### **Correspondence:** Giusi Prencipe

**Introduction:** An increasing body of evidence obtained both in patients and experimental models of secondary hemophagocytic lymphohistiocytosis (sHLH), including macrophage activation syndrome (MAS), supports the pathogenic role of Interferon-gamma (IFNγ). Nevertheless, to date, few data demonstrating the activation of the IFNγ pathway in target tissues of patients with sHLH during the active phase of the disease are available.

**Objectives:** In this study, in order to further clarify the involvement of IFNγ in the pathogenesis of sHLH, we investigated the activation of the IFNγ pathway in the affected liver of patients with active sHLH. In addition, we evaluated the circulating levels of IFNγ inducible chemokines CXCL9 and CXCL10 and assessed the activation of the IFNγ pathway in peripheral blood mononuclear cells (PBMCs) of patients collected during the course of the disease.

**Methods:** We analysed by real-time PCR the mRNA expression levels of IFNγ and IFNγ-inducible genes in the liver and in peripheral blood mononuclear cells (PBMCs) from two patients with active sHLH and one patient with active MAS, in which the disease was limited to the liver, without systemic involvement. Moreover, both in liver and PBMCs protein lysates, we evaluated by Western Blot analyses the levels of total and Tyrosine (701)-phosphorylated Signal transducer and activator of transcription 1 (STAT1), the transcription factor mainly involved in the activation of the IFNγ signaling pathways. Circulating CXCL9 and CXCL10 have been measured by ELISA.

**Results:** The expression levels of IFNγ and IFNγ-inducible genes were markedly up-regulated in livers from all three patients, compared to control livers. Conversely, slight differences in the expression levels of Type I IFN-inducible genes and other classical pro and anti-inflammatory cytokines were found. Further supporting the activation of the IFNγ pathway, higher protein levels of phosphorylated and total STAT1 were detected in patient livers. Accordingly, we found that mRNA levels of IFNγ-regulated genes and phosphorylated STAT1 protein levels were markedly increased in patient PBMCs collected during the active phase of the disease. Finally, circulating levels of IFNγ-inducible CXCL9 and CXCL10 were markedly increased during the active phase of the disease, paralleling the ferritin’s trend.

**Conclusion:** Our data provide evidence of selective and marked up-regulation of the IFNγ pathway in liver tissue as well as in PBMCs and blood of patients with active sHLH, further supporting the key role of IFNγ in the pathogenesis of the disease, and provide the rationale for the therapeutic use of an anti-IFNγ antibody in sHLH.

**Disclosure of Interest**

G. Prencipe: None Declared, C. Bracaglia: None Declared, A. Pascarella: None Declared, I. Caiello: None Declared, P. Francalanci: None Declared, M. Pardeo: None Declared, A. Meneghel: None Declared, G. Martini: None Declared, A. Insalaco: None Declared, G. Marucci: None Declared, F. Zulian: None Declared, F. De Benedetti Grant/Research Support from: Novartis, Novimmune, Hoffmann- La Roche, SOBI, AbbVie, Pfizer

### P006 LUNG DISEASE IN SYSTEMIC JUVENILE IDIOPATHIC ARTHRITIS (SJIA)

#### Vivian Saper^1^, Gail Deutsch^2^, Paul Guillerman^3^, Ray Balise^4^, Ann N. Leung^5^, Layla Bouzoubaa^4^, Scott Canna^6^, Grant Schulert^7^, Alexei Grom^7^, Tushar Desai^8^, Elizabeth Mellins^1^ and 52 Case Reporters representing 42 contributing institutions

##### ^1^Pediatrics, Stanford University, Stanford, CA; ^2^Pathology, Seattle Children's Hospital, Seattle, WA; ^3^Radiology, Texas Children's Hospital, Houston, TX; ^4^Public Health Sciences, University of Miami, Miami, FL; ^5^Radiology, Stanford University, Stanford, CA; ^6^Pediatrics, Children’s Hospital of Pittsburgh, Pittsburgh, PA; ^7^Pediatrics, Cincinnati Children’s Hospital, Cincinnati, OH; ^8^Pulmonary Medicine, Stanford University, Stanford, CA, USA

###### **Correspondence:** Vivian Saper

**Introduction:** Although not known to be a manifestation of sJIA, high mortality lung disease, including alveolar proteinosis (AP) and pulmonary hypertension (PH), have been increasingly noted.

**Objectives:** To describe clinical, radiologic and biopsy findings in children with sJIA as they develop life threatening parenchymal lung disease.

**Methods:** This retrospective observational case series included children diagnosed until age 18 with sJIA or sJIA-like illness and lung disease. Chest CT and/or lung biopsy was required for inclusion and underwent expert review.

**Results:** 61 included cases were contributed by 52 case reporters (Abulaban,K, Baszis,K, Behrens, E, Birmingham, J, Canna, S, Casey, A, Cidon,M, Cron,R, De, A, DeBenedetti,F, Deterding, R, Ferguson, I, Fishman, M, Goodman, S, Graham, B, Grom,A, Haines, K, Hazen, M, Henderson, L, Ibarra, M, Inman, CJ, Jerath,R, Kingsbury, D, Klein-Gitelman,M, Khawaja, K, Lai, K, Liptzin,DR, Lin, C, Lin, J, Lapidus,S, Milojevic, D, Mombourquette,J, Onel,K, Ozen,S, Perez, M, Phillippi,K, Prahal,S, Radhakrishna,S, Reinhardt, A, Riskalla, M, Rosenwasser,N, Roth, J, Schneider, R, Schonenberg-Meinema,D, Schulert,G, Shenoi,S, Smith,J, Sonmez,E, Stoll, M,Towe, C, Vehe,R, Young, L). 45 sJIA and 16 sJIA-like cases from the United States (54), Europe, the Middle East and Asia were studied. Median age of sJIA onset was 2 years; median time to lung disease was 2 years. Onset characteristics matched reported cohorts with the exception of younger onset age (p<.0001) and a 50x increased odds of Trisomy 21. Prior to lung disease, 46 cases were exposed to one or more inhibitors of IL-1 or IL-6. This pre-exposed group demonstrated unusual clinical and radiologic features including acute erythematous clubbing, a non-evanescent rash, and CT features not typical for the pathology observed. Respiratory symptoms, if present, were subtle. Lung pathology was primarily AP and PH and did not distinguish those pre-exposed from those who were not. Systemic inflammation typically occurred immediately prior to or with lung disease detection. Extent of evaluation for infectious causes varied and did not reveal a consistent pathogen. Of 6 cases with PCR for pneumocystis pneumonia (PCP) on BAL fluid, 4 cases were positive, and these children exhibited PCP compatible disease. Overall fatality rate was 37% and, of those surviving, only 3 report resolution of lung disease.

**Conclusion:** This study is the first to describe a set of unusual clinical and CT features in children with sJIA who developed lung disease during treatment with inhibitors of IL-1 or IL-6. However, this group shared (rare) lung pathologic diagnoses with subjects unexposed to these drugs. Further work is urgently needed as fatality rate is high, incidence appears to be increasing, mechanisms remain unknown and appropriate treatment is yet to be defined.

**Disclosure of Interest**

V. Saper Grant / Research Support from: Novartis, G. Deutsch: None Declared, P. Guillerman: None Declared, R. Balise: None Declared, A. Leung: None Declared, L. Bouzoubaa: None Declared, S. Canna: None Declared, G. Schulert: None Declared, A. Grom: None Declared, T. Desai: None Declared, E. Mellins Grant/Research Support from: Novartis

### P007 PREDICTORS OF EFFECTIVENESS OF INTERLEUKIN-1 INHIBITORS IN SYSTEMIC JUVENILE IDIOPATHIC ARTHRITIS

#### Jessica Tibaldi^1^, Benedetta Saccomanno^1^, Francesca Minoia^2^, Francesca Bagnasco^3^, Angela Pistorio^3^, Andressa Guariento^3^, Roberta Caorsi^3^, Alessandro Consolaro^4^, Marco Gattorno^3^, Angelo Ravelli^4^

##### ^1^Università degli Studi di Genova, Genoa; ^2^Fondazione IRCCS Ca’ Granda, Ospedale Maggiore Policlinico, Milan; ^3^Istituto Giannina Gaslini; ^4^Università degli Studi di Genova/Istituto Giannina Gaslini, Genoa, Italy

###### **Correspondence:** Jessica Tibaldi

**Introduction:** Systemic juvenile idiopathic arthritis (sJIA) is the most severe and a rather distinct subtype of JIA. It is common view that sJIA is the most severe form of childhood arthritis and the most difficult to treat. Recently the use of interleukin(IL)-1 antagonists has led to a significant improvement of the disease’s long-term evolution and has confirmed this cytochin’s key-role in the pathogenesis of sJIA. A number of potential predictors of the therapeutic effectiveness of IL-1 inhibitors have been reported, which include less severe joint disease and increased white blood cell count, shorter disease duration, older age at disease onset and use of IL-1 blockade as first-line therapy. However, because the experience gained so far is still limited, there is a need of further data to better characterize the profile of sJIA patients who are more susceptible to respond to IL-1 blockade.

**Objectives:** To seek for predictors of therapeutic response to interleukin (IL)-1 inhibitors in children with systemic juvenile idiopathic arthritis (sJIA).

**Methods:** The clinical charts of all patients with sJIA who were newly treated with anakinra or canakinumab at our center between 2004 and 2017 were reviewed retrospectively. Predictors included baseline demographic, clinical and laboratory variables as well as previous or concomitant therapies. The effectiveness of IL-1 antagonists was assessed at 1 year after treatment start. Complete clinical response (CCR) was defined as absence of fever, physician’s global assessment ≤ 1, count of active joints ≤ 1, negative C-reactive protein (CRP), and ≥ 75% reduction of corticosteroid dose. According to the intention-to-treat principle, patients who had IL-1 antagonists discontinued before 1 year for any reasons other than disease remission were classified as nonresponders. Statistics included univariate and multivariable analyses.

**Results:**

Of the 65 patients included in the study, 25 (39%) met the criteria for CCR at 1 year, whereas 40 (61%) did not. On multivariable analysis, independent correlations with achievement of CCR were identified for shorter disease duration, lower active joint count, higher ferritin level, and greater activity of systemic manifestations (Table 1). The area under the curve (AUC) of the model was 83%.

**Conclusion:** Our findings help to delineate the clinical profile of sJIA patients who are more likely to benefit from IL-1 blockade. They also underscore the critical need of randomized controlled trials aimed to explore the therapeutic role of IL-1 inhibition early in the disease course.

**Disclosure of Interest**

None Declared

**Table 1 (abstract P007).** See text for description

Explanatory variable	OR (95% CI)	P^£^
Disease duration ≤ 3.9 years	7.15 (1.36-37.52)	0.009
Active joint count ≤ 10	10.25 (1.59-65.93)	0.004
Ferritin > 444 ng/dL	5.69 (1.42-22.88)	0.009
Systemic manifestation score > 3	5.83 (1.38-24.62)	0.011

### P008 DISEASE EVOLUTION IN SYSTEMIC-ONSET JUVENILE IDIOPATHIC ARTHRITIS: PRELIMINARY DATA FROM JIRCOHORTE

#### Michelle Wallimann^1^, Katerina Theodoropoulou^1^, Elvira Cannizzaro^2^, Daniela Kaiser^3^, Traudel Saurenmann^4^, Federica Vanoni^5^, Andreas Woerner^6^, Alexandre Belot^7^, Isabelle Kone-Paut^8^, Etienne Merlin^9^, Olivier Richer^10^, Carine Wouters^11^, François Hofer^12^, Veronique Hentgen^13^, Michaël Hofer^1^

##### ^1^Department Femme Mère Enfant, Pediatric Immunology and Rheumatology Romande, Lausanne University Hospital, Lausanne; ^2^University Children's Hospital, Zurich; ^3^Children's Hospital Lucerne, Lucerne; ^4^Winterthur Cantonal Hospital, Winterthur; ^5^Bellinzona Regional Hospital, Bellinzona; ^6^University Children's Hospital, Basel, Switzerland; ^7^Hôpital Femme Mère Enfant, University Hospital, Lyon; ^8^University Hospital Kremlin-Bicêtre, Paris; ^9^Hôpital d'Estaing, University Hospital, Clermont-Ferrand; ^10^University Hospital Bordeaux, Bordeaux, France; ^11^University Hospital Leuven, Leuven, Belgium; ^12^JIRcohorte, Lausanne, Switzerland; ^13^Hospital Center Versailles, Versailles, France

###### **Correspondence:** Michelle Wallimann

**Introduction:** Systemic onset juvenile idiopathic arthritis (SoJIA) is a potentially severe disease with both systemic and joint inflammation; different evolutive forms were described (monophasic, polyphasic or persistent), but the outcome is hardly predictable at diagnosis.

**Objectives:** This study aims to identify early predictors of disease evolution within the SoJIA population enrolled in the Juvenile Inflammatory Rheumatism cohort (JIRcohorte), an international prospective cohort study.

**Methods:** 152 SoJIA patients with a minimum of two-year follow-up were enrolled. 5 patients were excluded due to missing data on diagnostic visit. Demographics and clinical data were collected (retrospectively if diagnosis < 2015 and prospectively if diagnosis ≥ 2015) and described for 147 patients. At diagnosis, median age and disease duration was 5.3 years and 1.9 months, respectively, male to female ratio was 1:1.2. Median follow-up was 6.7 years. 123 and 98 patients were treated with biologics and DMARDs, respectively. Monophasic evolution is determined by the occurrence of active disease (systemic symptoms and/or arthritis) followed by inactive disease. No recurrence of active disease is observed, and remission is obtained. The polyphasic course is defined by the recurrence of active disease at any time after having achieved remission. A persistent evolution is characterized by the persistence of systemic symptoms and/or arthritis and/or abnormal laboratory results (for at least 24 months).

We present the preliminary results in 30 patients with complete data for disease activity and medication use.

**Results:** Corresponding to their evolution, patients were classified in the monophasic (n=10; MONO), polyphasic (n=14; POLY) or persistent group (n=6; PER). Females were predominant in the MONO and POLY group with 80% and 79%, respectively. No female patient was in the PER group. At diagnosis, all patients in the PER and POLY group had arthritis; only 80% did in the MONO group. Hepatomegaly and splenomegaly was present in 30% and 50% of MONO patients versus 17% and 0% of PER patients, respectively. Joint count during first 6 months was ≥ 5 joints for 83% of the PER population compared to 30% for the MONO. Four patients in the MONO group (40%) did not have any biologics, whereas all patients from the other groups had one or more (POLY: 7 patients had one, 5 patients had two and 2 patients had three different biologics). Disease Modifying Anti-Rheumatic Drugs (DMARDs) were used in 4 patients of the MONO (40%), 13 of the POLY (93%) and 5 of the PER group (83%). Median duration of corticoid-use for the MONO group and the POLY group was 0.3 years and 2.68 years, respectively.

**Conclusion:** Systemic presentation seems to be more prevalent in the MONO group whereas arthritis in the POLY and PER groups. Polyarticular arthritis at 6 months is suggesting persistent disease evolution. Biologics, DMARDs and corticoids are more often and longer used in the POLY and PER groups correlating with a more severe disease course. Early recognition of a monophasic evolution could help the physician to limit the use of treatments, in particular steroids.

Data are under completion for all 152 patients. Precise description of disease evolution and deep phenotypic analysis will be performed.

**Disclosure of Interest**

None Declared

### P009 INSUFFICIENT INTERLEUKIN-10 PRODUCTION AS A MECHANISM UNDERLYING PATHOGENESIS OF SYSTEMIC JUVENILE IDIOPATHIC ARTHRITIS

#### Maya Imbrechts^1^, Anneleen Avau^1^, Jessica Vandenhaute^1^, Bert Malengier-Devlies^1^, Karen Put^1^, Tania Mitera^1^, Nele Berghmans^1^, Oliver Burton^2^, Adrian Liston^2^, Lien de Somer^3^, Carine Wouters^3^, Patrick Matthys^1^

##### ^1^Rega Institute KULeuven; ^2^VIB KULeuven; ^3^University Hospital Leuven, Leuven, Belgium

###### **Correspondence:** Carine Wouters

**Introduction:** Systemic juvenile idiopathic arthritis (sJIA) is a severe childhood immune-inflammatory disorder with unknown etiology.

**Objectives:** One of the concepts is that the disease results from an inappropriate control of immune responses to an initially harmless trigger. In the current study, we investigated whether sJIA may be caused by defects in IL-10, a key cytokine in controlling inflammation.

**Methods:** IL-10 production was analyzed in a sJIA mouse model, which relies on injection of Complete Freund’s Adjuvant (CFA) in IFN-γ deficient mice. Corresponding wild type (WT) mice develop a subtle and transient inflammatory reaction and were used to study the effect of IL‑10 neutralization. Cytokines and CRP were analyzed in plasma of sJIA patients (active: n=10; inactive: n=8) and healthy controls (n=15). Their PBMCs were used to study cell-specific defects in IL-10.

**Results:** Diseased IFN-γ deficient mice showed a defective IL-10 production in T_reg_ cells, B cells and NK cells, with B cells as the major source of IL-10. Neutralization of IL-10 in WT mice resulted in a chronic immune-inflammatory disorder clinically and hematologically reminiscent of sJIA. In sJIA patients, IL-10 plasma levels were strikingly low as compared to pro-inflammatory mediators. In addition, B cells from sJIA patients showed a decreased IL-10 production, both *ex vivo* and after stimulation.

**Conclusion:** Cell-specific IL-10 defects in sJIA mice and patients result in an insufficient IL‑10 production to counterbalance their pro-inflammatory cytokines. IL-10 neutralization in CFA-challenged WT mice converts a transient inflammatory reaction into a chronic disease and represents a model for sJIA in IFN-γ competent mice.

**Disclosure of Interest**

None Declared

## Systemic JIA - MAS I

### P010 EFFECTIVENESS OF MTX BEFORE BIOLOGICS IN SYSTEMIC JIA: SINGLE CENTER EXPERIENCE

#### Hatice Adıguzel Dundar^1^, Ceyhun Acari^2^, Serkan Turkucar^2^, Sevket Erbil Unsal^2^

##### ^1^Department of Pediatrics, Pediatric Rheumatology, Dokuz Eylül Univercity Faculty of Medicine; ^2^Department of Pediatrics, Pediatric Rheumatology, Dokuz Eylul University Faculty of Medicine, Izmir, Turkey

###### **Correspondence:** Hatice Adıguzel Dundar

**Introduction:** Systemic juvenile idiopathic arthritis (SJIA) accounts for 10-20% of all JIA patients. It is characterized by findings of systemic inflammation such as fever, rash, serositis, lymphadenopathy, and hepatosplenomegaly, and arthritis. Arthritis may develop later in life with variable clinical features. Macrophage activation syndrome is the most life threatening complication. Progress in the understanding of pathogenesis has led to the development of cytokine-targeted therapies, biologics.

**Objectives:** This study aims to evaluate the treatment options in SJIA, particularly effect of methotrexate prior to biologics.

**Methods:** A retrospective review of 52 cases of systemic JIA in the last 20 years was performed.

**Results:** Thirty one (59.6%) of the patients were female. The mean age of diagnosis was 89.8 ± 7.88 months and the mean follow-up was 32 ± 18.5 months. Twenty seven (51.9%) had monocyclic, 9 (17.3%) had polycyclic and 16 (30.8%) had polyarticular course. All of the patients had intermittant fever at onset, followed by rash and arthritis, and the least common symptom was serous involvement.

Ten (19.2%) cases had macrophage activation syndrome (MAS). Four of them presented with MAS as the initial diagnosis. The laboratory values of MAS cases were significantly lower in ESR, platelet and fibrinogen levels and higher in ferritin and triglyceride levels when compared with other cases. Eight of the MAS cases (80%) had monocyclic, 1 (10%) had polycyclic and 1 had polyarticular course. In 8 of the MAS cases (80%) the disease was controlled by pulse steroid followed by methotrexate alone.

Fifty (96.2%) cases received pulse steroids. Methotrexate (MTX) treatment was added in 47 (88.5%) cases. Thirty two (61.5%) cases were in remission with methotrexate treatment, whereas 14 (27%) cases required an additional biological agent. Majority of patients with biologics (56.3%) had polyarticular course. Adalimumab (1), anakinra (3), tocilizumab (6), etanercept (8) and canakinumab (9) were the biologics. In 4 cases, cyclosporin was added to control the disease.

Eight patients (15.4%) were transferred to adult rheumatology clinic for persisting arthritis. Two (4.3%) of the patients died due to sepsis and MAS, respectively. Seventeen (32.7%) were in remission under medication, and 25 (48.1%) were in remission without medication.

**Conclusion:** Randomized controlled trials have evidence about effectiveness of biologics, particularly IL-1 and IL-6 inhibitors, in treatment of SJIA. However, this study shows that methotrexate could control the disease in about two thirds of patients, particularly in “monocyclic” SJIA. Biologics such as anakinra, canakinumab, or tocilizumab could be preferred in cases with systemic onset and polyarticular course. Multicentered studies with more patients are needed to support this “real-world data”.

**Disclosure of Interest**

None Declared

### P011 AN UNUSUAL PRESENTATION OF PURINE NUCLEOSIDE PHOSPHORYLASE DEFICIENCY MIMICKING SYSTEMIC JUVENILE IDIOPATHIC ARTHRITIS (SJIA) COMPLICATED BY MACROPHAGE ACTIVATION SYNDROME (MAS)

#### Alessia Arduini^1^, Emiliano Marasco^2^, Gian Marco Moneta^2^, Ivan Caiello^2^, Giulia Marucci^2^, Manuela Pardeo^2^, Antonella Insalaco^2^, Giusi Prencipe^2^, Fabrizio De Benedetti^2^, Claudia Bracaglia^2^

##### ^1^Pediatric Department, La Sapienza University of Rome; ^2^Division of Rheumatology, IRCCS Ospedale Pediatrico Bambino Gesù, Rome, Italy

###### **Correspondence:** Alessia Arduini

**Introduction:** Monogenic primary HLH (pHLH) are caused by mutations of genes coding for proteins involved in cytotoxic activity or occur in the context of immunodeficiency.

**Objectives:** To demonstrate that the evidence of unusual clinical and laboratory findings in a well-known condition needs further investigations to rule out a different diagnosis.

**Methods:** Serum CXCL9, CXCL10 and IL-18 levels measured using ELISA.T cell proliferation was assessed stimulating PBMCs with either anti-CD3 or PMA/ionomycin for five days.

**Results:** A 14 months old Caucasian male was admitted with a history of persistent fever, small palpable cervical lymphnodes, rash, pericardial effusion and arthralgia. He developed overt swelling of wrists and ankles. Laboratory findings showed: elevated CRP and ESR, neutropenia, lymphopenia and anaemia, with normal platelet count. Serum ferritin levels were markedly elevated and lactate dehydrogenase aspartate aminotransferase and triglyceride were also elevated. Fibrinogen was normal. Bone marrow biopsy showed haemophagocytosis. Based on the presence of arthritis in a child with fever and rash a diagnosis of sJIA was made with onset complicated by MAS. Treatment with glucocorticoid was started. Functional tests and genetic analysis of pHLH related genes were negative. After two weeks the patient presented normalization of laboratory parameters except for persistent anaemia and profound lymphopenia. Moreover, a mild muscle hypotone became apparent. These features, unusual in sJIA, triggered additional investigations. These revealed: reduced aptoglobin, high reticulocyte count and positive Coombs test, all suggestive of haemolytic anaemia. Immunological phenotyping revealed a reduced number of B cells, CD4^+^T cells, CD8^+^T cells and a defective proliferative response of T cells to phytohemagglutinin (PHA) and anti-CD3 (OKT3). A metabolic screening showed an increase in urine orotic acid, with normal citrulline serum level and low serum level of uric acid. Based on the presence of haemolytic anaemia, T lymphocyte defect and increase in urinary orotic acid, purine nucleoside phosphorylase (PNP) deficiency was suspected. The diagnosis was confirmed by marked reduction of enzymatic activity of PNP and by genetic analysis. The patient received an haplohydentical bone marrow transplantation (BMT) with a subsequent progressive clinical improvement. Serum levels of CXCL9 and CXCL10, two chemokines directly induced by IFNgamma (IFNγ) and of IL-18, were found markedly elevated at disease onset during active phase of MAS. Cytokines levels decreased progressively during disease course and completely normalized after the BMT.

**Conclusion:** This patient with a clinical presentation highly suggestive for sJIA onset complicated by MAS was later diagnosed with PNP deficiency with HLH. The diagnostic process was driven by some unusual features that became apparent when HLH clinical and laboratory features were controlled with glucocorticoids. Low T cells and profoundly defective response to mitogens suggested an immunodeficiency, low haptoglobin with positive Coombs test suggested an haemolytic anemia and increased urinary orotic acid suggested an alteration of the pyrimidine biosynthetic pathway. These findings were consistent with a diagnosis PNP-deficiency. CXCL9, CXCL10 and IL-18 levels suggested that patients with PNP-deficiency may have a subclinical activation of the IFNγ pathway and indeed they are predisposed to develop sHLH. Informed consent to publish had been obtained from the parents.

**Disclosure of Interest**

A. Arduini: None Declared, E. Marasco: None Declared, G. M. Moneta: None Declared, I. Caiello: None Declared, G. Marucci: None Declared, M. Pardeo: None Declared, A. Insalaco: None Declared, G. Prencipe: None Declared, F. De Benedetti Grant/Research Support from: Novartis, Novimmune, Hoffmann- La Roche, SOBI, AbbVie, Pfizer, C. Bracaglia: None Declared

### P012 SYSTEMIC JUVENILE IDIOPATHIC ARTHRITIS: A SINGLE CENTER EXPERIENCE

#### Kenan Barut, Gurkan Tarcin, Gulberk Tahaoglu, Sezgin Sahin, Amra Adrovic, Ozgur Kasapcopur

##### Pediatric Rheumatology, Istanbul University, Cerrahpasa Medical School, Istanbul, Turkey

###### **Correspondence:** Kenan Barut

**Introduction:** Juvenile idiopathic arthritis (JIA) is the most common chronic rheumatic disease in childhood, divided into several subgroups. The sJIA could be presented by monocyclic, polycyclic or persistent polyarticular clinical course. Macrophage activation syndrome (MAS) represents the most devastating complication that could appear during the disease course. Studies on follow up, treatment response and disease complications of the sJIA patients are spare and rare.

**Objectives:** To evaluate demographic and clinical characteristics and to explore the long-term treatment response and disease complications in a large cohort of sJIA patients from the single center.

**Methods:** Demographic and clinical features of the sJIA patients were reached from the patient’s recrods. The frequency of disease flares, treatment response and side effects were recorded for each patient.

**Results:** A total of 168 sJIA patients were included in the study: 87 (51.8) female, 81 (48.2) male. The clinical features are shown in Table 1. Fifty-three (31.5) patients had monocyclic while 23 (13.7) patients had polycyclic clinical course (mean recurrency of attacks 2.5±2 (IQR:1-4)): in 38 (42). Polyarticular course was present in 92 (54.8) patients. Initially diagnosis of patients were: infection in 86 (51.1), sJIA in 34 (20.4), acute rheumatic fever in 19 (11.3), urticaria in 10 (5.9), Kawasaki disease in 4(2.4) and juvenile systemic lupus erythematosus in 2 patients.

The most common disease complications were: MAS in 20 (11.9), growth retardation in 19 (11.3) and vertebral fracture due to osteoporosis in 3 (1.9) patients. Gastrointestinal symptoms secondary to methotrexate intolerance that led to cessation of treatment were present in 9 (7.1) patients. Among 5 (2.9) patients that developed tuberculosis, 4 (2.3) were under etanercept treatment.

All of the patients were treated with corticosteroids: a doses of 2 mg/kg/day in 118 (70.2) patients  and pulse steroids in 50 (29.8) patients with severe clinical presentation. The methotrexate was used in 126 (75), leflunomide in 5(3), cyclosporine A in 29 (17.3), intravenous immunoglobulin in 19 (11.3), anakinra in 27 (16.1), canakinumab in 27 (16.1), tocilizumab in 18 (10.7), etanercept in 50 (29.8) and adalimumab in 7(4.2) patients. The median time to remission after the initial treatment with corticosteroids was 4 (IQR:2-4) months. The remission off medications was achieved in 82 (48.8) while remission on medications was achieved in 83 (49.4) of patients.

**Conclusion:** Systemic JIA is a subtype of JIA characterized by significant morbidity and mortality rate with macrophage activation syndrome being the most severe disease complication. Corticosteroids represent the main treatment modality. Biological agents should be considered in the steroid-resistant patients. The clinical remission could be achieved and chronic arthritis sequelae could be prevented in a majority of patients with biological agents.

**References**

1. Dewoolkar M, et al. Course, Outcome and Complications in Children with Systemic Onset Juvenile Idiopathic Arthritis. Indian J Pediatr 2017;84:294-8

2. Cimaz R. Systemic-onset juvenile idiopathic arthritis. Autoimmunity reviews. 2016;15(9):931-4.

3. Barut K, Adrovic A, Sahin S, Kasapcopur O. Juvenile Idiopathic Arthritis. Balkan medical journal. 2017;34(2):90-101

**Disclosure of Interest**

None Declared

**Table 1 (abstract P012).** Demographic, clinical features of sJIA

Female/male (n:168)	87(51.8)/81 (48.2)
Mean age at disease onset	76.7±54.5 months (IQR: 28-118)
Mean age at diagnosis	79,7±54.5 months (IQR: 33-121)
Mean delay of diagnosis	3.9 ± 8.7 months (IQR:1-3)
Clinicalfeatures, n (%)	
Typical fever	160(95.2)
Typical rash	99(59)
Lymphadenopathy	45(26.8).
Hepatosplenomegaly	70(41.7)
Arthritis/arthralgia	143(85.1), 25(14.9)
Pericarditis, pleuritis	12(7.1), 3(1.8)

### P013 CHARACTERISTICS OF PATIENTS ENROLLED IN THE FIRST LINE OPTIONS FOR SYSTEMIC JIA TREATMENT (FROST) CONSENSUS TREATMENT PLAN STUDY

#### Timothy Beukelman^1^, Peter A. Nigrovic^2^, George Tomlinson^3^, Vincent Del Gaizo^4^, Marian Jelinek^4^, Laura E. Schanberg^5^, Anne Dennos^5^, Mary Ellen Riordan^6^, Yukiko Kimura^6^, for the CARRA Registry FROST Investigators

##### ^1^University of Alabama at Birmingham, Birmingham; ^2^Brigham and Women's Hospital, Harvard University, Boston, USA; ^3^University of Toronto, Toronto, Canada; ^4^CARRA Parent/Patient Partner; ^5^Duke University, Durham; ^6^Hackensack University Medical Center, Hackensack, USA

###### **Correspondence:** Timothy Beukelman

**Introduction:** The optimal initial treatment for systemic juvenile idiopathic arthritis (sJIA) is unclear. Uncontrolled reports suggest that early treatment with biologic agents likely produces superior short-term clinical outcomes, but many patients may respond well to non-biologic therapies. To further study the initial treatment of sJIA, the Childhood Arthritis and Rheumatology Research Alliance (CARRA) developed Consensus Treatment Plans (CTPs) to formalize and standardize current treatment practices. Subsequently, 4 CTPs were developed: initial systemic glucocorticoid (GC); initial methotrexate (MTX) +/- GC; initial IL-1 inhibition (IL-1i) +/- GC; and initial IL-6 inhibition (IL-6i) +/- GC.

**Objectives:** The FiRst Line Options for Systemic JIA Treatment (FROST) is an observational study designed to assess the effectiveness and safety of each of the 4 CTPs and to the compare the CTPs containing initial biologic therapy (IL-1i and IL-6i) to those that do not contain initial biologic therapy (GC and MTX).

**Methods:** Patients with recent onset sJIA who are initiating therapy are considered for enrollment in FROST. In order to be eligible for FROST, participants must have fever for ≥2 weeks, arthritis for ≥ 10 days, and at least 1 of the following: evanescent rash, generalized lymphadenopathy, hepatomegaly, splenomegaly, or serositis. Treatment assignment is at the discretion of the treating physician and family. All data are collected in the CARRA Registry. To date, 43 CARRA Registry sites have been activated to enroll FROST patients. Biosamples are collected at baseline and at 6 months. Patient reported outcomes (PRO) of presence of fever and rash and pain scores are collected at home using mobile devices every 2 days during the first 2 weeks of the study. The primary study outcome is clinical inactive disease (Wallace ACR provisional definition) and cessation of glucocorticoid therapy at 9 months.

**Results:** Enrollment in the FROST study began in November 2016. As of April 2018, 26 patients have been enrolled at 13 sites, and their baseline characteristics are shown in Table 1. The proportion of patients with specific SJIA disease manifestations was: rash 89%, lymphadenopathy 31%, hepatomegaly 15%, splenomegaly 23%, serositis 8%, polyarthritis 46%. Overall, 15 participants (58%) have completed every requested home PRO during the first 2 weeks of the study, and 19 (73%) have completed ≥50% of the requested home PRO. Study follow-up continues to accrue. Eight patients (31%) have completed the 9-month study visit when the primary study outcome is assessed.

**Conclusion:** Enrollment in the FROST study has begun. Participants enrolled thus far appear to be representative of the general population of patients with sJIA. Home PRO collection is feasible through the CARRA Registry. Additional time is needed to enroll and observe a sufficient number of patients to assess the comparative effectiveness of initial treatments for sJIA.

**Disclosure of Interest**

T. Beukelman Consultant for: UCB, Bristol-Myers Squibb, Sobi, Novartis, P. Nigrovic Grant/Research Support from: AbbVie, Sobi, Novartis, Consultant for: AbbVie, Sobi, Novartis, Bristol-Myers Squibb, G. Tomlinson: None Declared, V. Del Gaizo Consultant for: Sobi, M. Jelinek: None Declared, L. Schanberg: None Declared, A. Dennos: None Declared, M. E. Riordan: None Declared, Y. Kimura: None Declared

**Table 1 (abstract P013).** See text for description.

Characteristic	(N=25)
Age (Mean (SD))	7.2 (5.1)
Female (%)	13 (52%)
Elapsed Days Since Onset of Symptoms (Mean (SD))	45 (55)
Elapsed Days Since Diagnosis (Mean (SD))	7 (8)
Physician Global Assessment (Mean (SD))	6.0 (2.1)
Parent Global Assessment (Mean (SD))	4.7 (3.2)
Number of Active Joints (Median (IQR))	3 (2-7.5)
ESR (Median (IQR))	65 (44-96)
Ferritin (Median (IQR))	891 (284-2350)

### P014 THE FREQUENCY OF MACROPHAGE ACTIVATION SYNDROME IN SYSTEMIC JUVENILE IDIOPATHIC ARTHRITIS CASES AND LONG TERM FOLLOW-UP RESULTS

#### Mustafa Çakan, Şerife Gül Karadağ, Nuray Aktay Ayaz

##### Pediatric Rheumatology, Kanuni Sultan Süleyman Research And Training Hospital, İstanbul, Turkey

###### **Correspondence:** Mustafa Çakan

**Introduction:** Systemic juvenile idiopathic arthritis (sJIA) is a multisystemic disease characterized by fever, rash, arthritis and polyserositis. The most dreadful complication of sJIA is macrophage activation syndrome (MAS) that is seen in 10-25% of the cases. sJIA cases may follow monocyclic, polycyclic and chronic polyarticular course.

**Objectives:** The aim of this study was to demonstrate the frequency of MAS in sJIA cases and to see whether sJIA cases complicated with MAS follow a more severe course in the long term.

**Methods:** Files of sJIA cases that were followed in our clinic between May 2010 and September 2017 were reviewed. To be included in the study, the patient had to be coming regularly to follow-up visits and had to be completed the first six months of the disease.

**Results:** The cohort consisted of 53 sJIA cases. Mean duration of follow-up was 39.0 ± 24.1 months. Mean age at the time of diagnosis was 7.9 ± 4.5 years. There was a female predominance in the cohort (32 females, 21 males). The frequency of MAS was 33.9% (18 cases). MAS was observed much more common in male patients than female patients; 42% vs 28%, respectively.

Bone marrow aspiration was performed to all patients to exclude malignancy. In 27.7% of MAS cases hemophagocytosis was not observed and hemophagocytosis was observed in 8.5% of cases without MAS. Initial laboratory test at the time of diagnosis of sJIA (leukocyte, hemoglobin, platelet, ferritin, triglyceride, ESR, CRP, fibrinogen, AST, ALT, albumin, and LDH) were compared in between patients with MAS and without MAS. Only ferritin and fibrinogen levels showed significant difference in between the groups (p<0.01). Patients that developed MAS had higher ferritin (4482 mg/dl) and lower fibrinogen (371 mg/dl) values than patients without MAS (ferritin 2060 mg/dl, fibrinogen 466 mg/dl) at the time of diagnosis of sJIA. When these laboratory parameters were compared at the time of diagnosis of sJIA and at the time of diagnosis of MAS, all parameters showed significant differences except CRP.

Long term follow-up results showed that monocyclic course was observed in 45%, polycyclic course in 32% and chronic polyarticular course in 23% of the cases. We have observed that patients with MAS segregated equally into three groups.

All MAS patients were initially treated with high-dose methylprednisolone and intravenous immunoglobulin. Additional treatments in MAS were cyclosporine (12 cases), anakinra (7 cases) and plasmapheresis (4 cases).

Biologics were used in 38.8% of all cases. Biologic treatments were needed in 12.5% of monocyclic cases, in 50% of polycyclic cases and in 77% of chronic polyarticular cases. At the time of enrollment, 58% of patients were under remission without medication and 38% were under remission with medication. Only two cases were active and both cases were in the group of chronic polyarticular course.

**Conclusion:** All sJIA patients should be followed for the development of MAS. High ferritin and relatively lower fibrinogen levels at the time of diagnosis of sJIA may be early warning signs of impending MAS. sJIA patients that develop MAS do not seem to portend more guarded prognosis in the long term follow-up. sJIA patients that follow a chronic polyarticular course are possibly one of the hardest group of patients to treat in pediatric rheumatology.

**Disclosure of Interest**

None Declared

### P015 THE IMPORTANT ROLE OF BONE MARROW BIOPSY TO DIAGNOSIS OF MACROPHAGE ACTIVATION SYNDROME

#### Kwangnam Kim^1^, Jong Gyun Ahn^2^, Young Dae Kim^3^

##### ^1^Pediatrics, Hallym Sacred Heart Hosptal, Hallym University Medical Center, Anyang; ^2^Pediatrics, Severance Children's Hospital, Yonsei University, Seoul; ^3^Pediatrics, Ilsan Paik Hospital, Inje University, Goyang, Korea, Republic Of

###### **Correspondence:** Kwangnam Kim

**Introduction:** The diagnosis of MAS is difficult. A fall in platelet count and ESR, in combination with high CRP, are early signs of impending MAS in a febrile patient with an active rheumatologic condition. Other features indicative of MAS are hyperferritinemia, cytopenia involving other cell lines (WBC and RBC), liver dysfunction, coagulopathy, decreasing serum fibrinogen and increasing triglycrides.

2016 criteria for classification of MAS in patients with systemic JIA was conducted of real patient data. These data are based on clinical and laboratory features at the time of disease onset.

**Objectives:** The patient with a condition that could potentially be confused with MAS and active systemic JIA without evidence of MAS. To evaluate the diagnostic convenience and efficiency of the 2016 EULA/ACR/PRINTO classification for MAS in patients with systemic JIA, it will be compared with confirmed MAS by BM biopsy.

Total 13 MAS is confirmed by BM biopsy. All of these patients are applied 2016 EULA/ACR/PRINTO classification for MAS in patients with systemic JIA.

**Methods:** 2016 new criteria was simplified the diagnostic process for the convenience. The MAS diagnosis tools are Ferritin, Platelet count, Aspartate aminotransferase, Triglycerides and Fibrinogen. This study was a retrospective chart review conducted in the pediatric department of a tertiary level teaching hospital from 2002 through 2016. The ILAR criteria were identified in each patient at the time of first presentation to our hospital. Seventy-four patients were diagnosed with sJIA. 12 boys and 15 girls formed this study cohort. A comparison of the baseline laboratory characteristics of patients who fulfilled the ILAR criteria and those with suspected sJIA has been shown in Table 1.

**Results:** Diagnosis of MAS is confirmed by presence of haemophagocytic histiocytes in bone marrow. Bone marrow biopsy confirmed activated histiocytes engulfing a band neutrophil.

**Conclusion:** A cytokine storm is a consistent feature in patients with MAS, and it is a potentially life-threatening complication of rheumatic diseases. The fact that highly effective treatment is needed in the early stage. So do that, early diagnosis is necessary. The conduct of the new 2016 classification critieria for MAS also underscored potential limitations. Additional validation of these criteria will be necessary. The BM biopsy will make up the weakness of limitation and early diagnosis will be more possible.

Referreces

1. Ravelli A. Minola F. Davi S. Horne A. Bovis F. Pistorio A. et al. 2016 Classification Criteria for Macrophage Activation Syndrome Complicating Systemic Juvenile Idiopathic Arthritis: A European League Against Rheumatism/American College of Rheumatology/Paediatric Rheumastology International Trials Organisation Collaboratve Initiative. Atrhritis Rheumatol. 2016;68(3):566-76.

2. Grom A. Horne A Benedetti F. et al. Macrophage activation syndrome in the era of biologic therapy. Nat Rev Rheumatol. 2016;12(5):259-268.

**Disclosure of Interest**

K. Kim Consultant for: pediatric clinical immunology, J. G. Ahn: None Declared, Y. D. Kim: None Declared

**Table 1 (abstract P015).** See text for description

	MAS	sJIA without MAS
Systemic-onset	13	14
Male/Female	7/6	5/9
Age at onset (yrs)	6 (1.1-15.9)	6 (3.1-12.5)
Bone marrow biopsy	13	14
Ferritin >648mg/mL	1,481 (14.9-29970)	587 (73.6-10021)
Platelet count <181k	403 (126-807)	346 (140-923)
AST >48 u/L	43 (17-4613)	99 (15-479)
Triglycerides > 156 mg/dL	150 (57-264)	138 (66-493)
Fibrinogens <360 mg/dL	302 (123-602)	482 (195-820)

CRP (mg/L), 102 (8.4-218.6), 23 (2.0-147.7)

ESR (mm/hr), 73 (4-108), 50 (3-90)

### P016 SYSTEMIC TYPE OF JUVENILE IDIOPATHIC ARTHRITIS IS A RISK FACTOR OF INFLUENZA INFECTION -A MULTI-INSTITUTIONAL QUESTIONNAIRE SURVEY IN JAPAN-

#### Tomohiro Kubota^1^, Yasuo Nakagishi^2^, Nami Okamoto^3^, Hiroaki Umebayashi^4^, Ken-ichi Nishimura^5^, Naomi Iwata^6^, Yuka Okura^7^, Masaki Shimizu^8^, Mao Mizuta^8^, Kosuke Shabana^3^, Masato Yashiro^9^, Takahiro Yasimi^10^, Junko Yasumura^11^, Hiroyuki Wakiguchi^12^, Noriko Kinjo^13^, Syuji Takei^14^, Masaaki Mori^15^

##### ^1^Pediatrics, KAGOSHIMA UNIVERSITY HOSPITAL, Kagoshima City; ^2^pediatrics, Hyogo Prefectural Kobe Children’s Hospital, Kobe; ^3^Pediatrics, Osaka Medical College, Takatsuki; ^4^Pediatrics, Miyagi Children’s Hospital, Sendai; ^5^Pediatrics, Yokohama City University Graduate School of Medicine, Yokohama; ^6^ Immunology and Infectious Diseases, Aichi Children’s Health and Medical Center, Obu; ^7^Pediatrics, KKR Sapporo Medical Center, Sapporo; ^8^Pediatrics, Graduate School of Medical Sciences, Kanazawa University, Kanazawa; ^9^Pediatrics, Okayama University Hospital, Okayama; ^10^pediatrics, Kyoto University Graduate School of Medicine, Kyoto; ^11^Pediatrics, Hiroshima University Graduate School of Biomedical & Health Sciences, Hiroshima; ^12^Pediatrics, Yamaguchi University Graduate School of Medicine, Ube; ^13^Pediatrics, graduate school of medicine, University of the Ryukyus, Nakagami; ^14^Pediatrics, KAGOSHIMA UNIVERSITY HOSPITAL, Kagoshima; ^15^Lifetime Clinical Immunology, Graduate School of Medical and Dental Sciences, Tokyo Medical and Dental University, Tokyo, Japan

###### **Correspondence:** Tomohiro Kubota

**Introduction:** The influenza (Flu) is one of the most prevalent infections in the world and the vaccination is recommended for the patients treated with immunosuppressive therapy and there is some immunological research on the Flu vaccination in the patients with juvenile idiopathic arthritis (JIA). However, the morbidity of Flu in the patient with JIA in the real-world is unknown.

**Objectives:** We aimed to evaluate Flu morbidity and side effect of vaccination in patients with JIA in comparison with the general population in Japan. And clarify the risk factor of Flu infection for the patient with JIA.

**Methods:** From October 2016 to May 2017, a total of 13 participating medical institutions collected questionnaires from patients with JIA. The questionnaires focused on the incidence of Flu infection and Flu vaccination, and the efficacy and side effects of Flu vaccination. The frequency of each item was compared to that in the general population. Multivariable logistic regression analysis was performed to determine the risk factor on the Flu infection.

**Results:** A total of 473 patients with JIA (159 males [34%]; 314 females [66%]) were enrolled (mean age, 13.3 years; range, 2.4-37.3 years). Two hundred sixty-eight (57%) patients got the Flu vaccine; side effects were observed in 58 patients (22%), with no severe cases. Patients were treated with methotrexate (n=235 [50%]; mean dose, 7.35 mg/m^2/week), prednisolone (n=110 [23%]; mean dose, 0.14 mg/kg/day), and biological disease-modifying anti-rheumatic drugs (n=327 [69%]). Seventy-four patients (15.6%) had a Flu infection and the influenza morbidity was significantly smaller in patients with JIA compared to that in the general population in Japan (21%)(p=0.005). JIA disease activity flared in one patient after Flu infection, but no one flared after Flu vaccination. In logistic regression analysis, the patients with systemic JIA were high risk for Flu infection (odds ratio:2.666, 95% confidence interval: 1.321-5.475, p=0.007).

JIA: juvenile idiopathic arthritis, MTX: methotrexate, PSL: prednisolone, bDMARDs: biological disease-modifying anti-rheumatic drugs, OR: odds ratio; CI: confidence interval

**Conclusion:** Influenza vaccination is safe and effective for patients with JIA treated with glucocorticoids and disease-modifying antirheumatic drugs. The risk of Flu infection is significantly high in the patient with systemic JIA.

**Disclosure of Interest**

None Declared

**Table 1 (abstract P016).** Multivariable logistic regression analysis to determine the risk factor on the influenza morbidity

variables	OR	95% CI	p value
Systemic JIA	2.666	1.321-5.475	0.007
MTX	0.750	0.398-1.419	0.373
PSL	0.766	0.386-1.454	0.428
bDMARDs	0.622	0.310-1.213	0.170
vaccine	0.836	0.490-1.429	0.510

### P017 Withdrawn

### P018 LIPID BIOMARKERS IN PATIENTS WITH JUVENILE IDIOPATHIC ARTHRITIS: IS THERE A DIFFERENCE BETWEEN THE POLYARTICULAR AND SYSTEMIC SUBTYPES?

#### Wellington D. Rodrigues, Roseli S. Sarni, Fernando A. Fonseca, Patricia P. Aires, Claudio A. Len, Maria T. Terreri

##### Pediatric Rheumatology, Universidade Federal de São Paulo, São Paulo, Brazil

###### **Correspondence:** Claudio A. Len

**Introduction:** Juvenile idiopathic arthritis (JIA) is the most common chronic rheumatic disease in the pediatric population. These patients coexist with the chronic disease, and present to a greater extent negative outcomes such as osteoporosis and cardiovascular diseases.

**Objectives:** To describe the biomarkers of lipid metabolism related to the cardiovascular risk of children and adolescents with JIA and to relate them to variables of the disease, lipid and glucose profile, nutritional status and food consumption.

**Methods:** Cross-sectional study with 62 JIA patients. The following were evaluated: disease activity and medications used, body mass index, height for age (z score), food intake (24 hour recall), lipid profile (total cholesterol - CT, low density lipoprotein - LDL, high density lipoprotein – HDL, triglycerides - TG and non - HDL), lipid markers such as apolipoproteins A-I and B (Apo A-I and Apo B). The association analyses were stratified as follows: HDL-c and Apo A-I into adequate and borderline/low; LDL-c, CT, TG, NHDL-c and Apo B into adequate and borderline/high; levels of C-reactive protein (CRP) and erythrocyte sedimentation rate (ESR). For statistical analysis: Exact Fischer, Chi-square, Mann-Whitney and Spearman Correlation tests; p <0.05.

**Results:** The mean age and time since diagnosis were 7.7 years (± 4.3) and 5.0 years (± 3.4), respectively. Active disease was observed in 33.9% of the patients and the prevalence of dyslipidemia was 62.9% overall when we considered lipid profile alone (CT, LDL-c, HDL-c, non-HDL-c and TG) and 82.3% when we included altered apolipoproteins (Apo A-I and Apo B). HDL-c and Apo A-I were the most frequently altered lipid markers. When comparing the subtypes, the systemic subtype had a higher value and higher frequency of altered LDL-c and NHDL-c and increased Apo B/Apo A ratio, when compared to the polyarticular subtype (p = 0.017, 0.042 and 0.03, respectively). When we cross-referenced the adequate or borderline/low HDL-c variable against other variables, patients with borderline/low HDL-c presented lower values ​​and higher frequency of altered Apo A-I (p<0.001 and 0.001, respectively). There was no significant correlation between us-CRP and variables related to lipid metabolism. However, ESR showed a negative correlation with Apo A-I levels (r = -0.25, p = 0.047).

**Conclusion:** We conclude that dyslipidemia and alteration of lipid biomarkers are common in patients with JIA. The systemic subtype and elevated ESR were associated with lower concentrations of Apo A-I, indicating the participation of the inflammatory process in this alteration. Furthermore, the systemic subtype presented higher value and frequency of atherogenic particles, suggesting that patients with this subtype are at increased risk for the development of cardiovascular disease.

**Disclosure of Interest**

None Declared

### P019 HEMOPHAGOCYTIC LYMPHOHISTIOCYTOSIS (HLH) MIMICKERS: CXCL9 AS A POTENTIAL BIOMARKER DISTINGUISHING HLH FROM OTHER HYPERFERRITINEMIC SYNDROMES

#### Giulia Marucci, Ivan Caiello, Manuela Pardeo, Virginia Messia, Giusi Prencipe, Antonia Pascarella, Fabrizio De Benedetti, Claudia Bracaglia

##### Division of Rheumatology, IRCCS Ospedale Pediatrico Bambino Gesù, Rome, Italy

###### **Correspondence:** Giulia Marucci

**Introduction:** Increased ferritin is considered biomarker highly suggestive of primary and secondary HLH and it is one of the HLH-2004 diagnostic and MAS guidelines (1, 2), nevertheless it could also be elevated in other inflammatory conditions. Interferon-gamma (IFNγ) and IFNγ induced chemokines, particularly CXCL9, have been demonstrated to be markedly elevated in patients with primary and secondary HLH.

**Objectives:** To describe eight patients with hyperferritinemia who fully or partially met the HLH-2004 diagnostic guideline, but in which the subsequent clinical course and further investigations led to different diagnosis that required different therapies and to investigate the CXCL9 levels in identify diseases that may mimic HLH. To be noted that soluble CD25 was not measured in these patients.

**Methods:** Serum CXCL9 levels were analyzed by DuoSet ELISA KIT DY392 (R&D Systems, Minneapolis, Minn). Normal values of CXCL9 are lower than 700 pg/ml.

**Results:** We identified 8 patients with laboratory features suggestive of HLH, included high ferritin levels (>500 ng/ml). Three of these patients met five of the HLH-2004 criteria, two of them met four criteria and the others two met three criteria. One patient presented only high serum ferritin level and cytopenia. CXCL9 levels were <300 pg/ml in seven of them and approximately 600 pg/ml in one patient. The clinical disease course and the other investigations ruled out the diagnosis of HLH and every patient received a different diagnosis. None of them received treatment for HLH (Table 1).

**Conclusion:** Since HLH is a life-threatening condition, early recognition and promptly therapy are essential to modify the disease course. One of the most typical feature of primary and secondary HLH is high ferritin levels. Due to the severity of the disease, sometimes patients need to be treated before all criteria are fulfilled. The eight patients reported had features highly suggestive of HLH and met the HLH-2004 criteria, or part of them. However they all received a different final diagnosis. Despite all presented with high ferritin levels, CXCL9 was low in all. CXCL9 is a chemokine specifically induced by IFNγ, and has been demonstrated to be markedly elevated in patients with primary and secondary HLH due to the activation of the IFNγ pathway. In these cases CXCL9 was able to differentiate diseases with high ferritin that mimics HLH. High CXCL9 levels appear to be a potential specific biomarker for HLH diagnosis. Early dosage of CXCL9 levels in patients with hyperferritinemia and with clinical suspicion of HLH may be helpful for a timely differential diagnosis.

**Reference**

1. Henter JI et al. HLH-2004: diagnostic and therapeutic guidelines for hemophagocytic lymphohistiocytosis. Pediatr Blood Cancer 2007 Feb; 48(2):124-31.

2. Ravelli A et al. 2016 Classification Criteria for Macrophage Activation Syndrome Complicating Systemic Juvenile Idiopathic Arthritis: A European League Against Rheumatism/American College of Rheumatology/Paediatric Rheumatology International Trials Organisation Collaborative Initiative. Ann Rheum Dis. 2016 Mar;75(3):481-9.

**Disclosure of Interest**

G. Marucci: None Declared, I. Caiello: None Declared, M. Pardeo: None Declared, V. Messia: None Declared, G. Prencipe: None Declared, A. Pascarella: None Declared, F. De Benedetti Grant/Research Support from: Novartis, Novimmune, Hoffmann-La Roche, SOBI, AbbVie, Pfizer, C. Bracaglia: None Declared

**Table 1 (abstract P019).** Patients’ features

Pt	Gender	Months at onset	HLH-2004 Criteria	Ferritin ng/ml	CXCL9 pg/ml	Final Diagnosis
1	M	1	5	9.849	<300	Osteopetrosis
2	M	6	5	800	<300	Vitamin B12 deficiency
3	M	1	5	543	<300	Chronic granulomatous disease
4	M	1	4	15.629	<600	Short bowel syndrome
5	M	3	4	4.378	<300	Cobalamin deficiency
6	F	2	3	37.232	656	Shaken baby syndrome
7	M	165	3	1.400	<300	Ghosal hematodiaphyseal dysplasia
8	M	172	2	1.184	<300	Primitive myelofibrosis

### P020 THROMBOTIC MICROANGIOPATHY: AN UNDER-RECOGNIZED, DREADFUL COMPLICATION OF MACROPHAGE ACTIVATION SYNDROME

#### Francesca Minoia^1,2^, Jessica Tibaldi^2,3^, Valentina Muratore^4^, Romina Gallizzi^5^, Concetta Micalizzi^2^, Angelo Ravelli^2,3^

##### ^1^Fondazione IRCCS Ca' Granda Ospedale Maggiore Policlinico, Milan; ^2^Istituto G. Gaslini; ^3^Università degli Studi di Genova, Genoa; ^4^IRCCS Policlinico San Matteo, Pavia; ^5^Università degli Studi di Messina, Messina, Italy

###### **Correspondence:** Francesca Minoia

**Introduction:** Macrophage activation syndrome (MAS) is part of the spectrum of hemophagocytic disorders and is a serious complication of rheumatologic conditions, mainly systemic juvenile idiopathic arthritis (sJIA). Thrombotic microangiopathy (TMA) is a heterogeneous group of life-threating diseases characterized by microangiopathic hemolytic anemia, thrombocytopenia and organ injury. The association between TMA and hemophagocytic syndromes has been described only in single reports in adult renal transplant recipients and in two pediatric cases of virus-induced hemophagocytic lymphohistiocytosis. TMA complicating MAS has never been reported so far

**Objectives:** To describe clinical and laboratory features, treatment and outcome of a cohort of patients with MAS complicated by TMA

**Methods:** The clinical charts of patients with MAS and TMA seen at 3 Italian tertiary-care pediatric rheumatologic centers were reviewed retrospectively. Demographic, clinical and laboratory data at the time of TMA diagnosis were collected. Therapeutic intervention and outcome were also recorded

**Results:** Five patients who met the criteria for MAS and developed TMA during the course of MAS were identified; one patient was excluded due to insufficient data. The underlying disease was sJIA or a sJIA-like disease in all patients. The median age at MAS onset was 8.6 years (4.2–12.8 yr), and the median interval between onset of MAS and occurrence of TMA was 13.5 days. TMA was characterized in all patients by renal failure with hematuria, and presence of schistocytes in blood smear. Three of the 4 patients experienced severe CNS involvement, with lethargy, seizures, or coma. The main laboratory features at TMA onset are shown in Table 1. For the management of MAS, all patients received high-dose corticosteroids and cyclosporine; in 3 cases anakinra was added. All TMA episodes were treated with plasma-exchange; two patients were also given biologics (rituximab or eculizumab) with good results. During the course of TMA, aggressive and prolonged management in the Intensive Care Unit was required in all cases. All patients survived

**Conclusion:** TMA is a dreadful complication of MAS, which is likely under-recognized. If not diagnosed timely and treated appropriately, TMA may precipitate the course of MAS and lead to a fatal outcome. Clues to suspect TMA in a patient with MAS are the increase in lactic dehydrogenase and decrease in platelet count out of proportion of the other laboratory abnormalities, the drop in haptoglobin level, the finding of schistocytes in blood smear, and the new onset of hematuria. Biologic medications, particularly rituximab and eculizumab may offer an adjunctive therapeutic option for refractory cases

**Disclosure of Interest**

None Declared

**Table 1 (abstract P020).** See text for description

	Patient 1	Patient 2	Patient 3	Patient 4
Hemoglobin, g/dl	6.5	11.1	6.9	9.5
Platelet count, x 10^9^/l	32	63	10	90
Aspartate aminotransferase, U/l	77	1015	119	322
Lactic dehydrogenase, U/l	4,852	5,358	4,877	4,683
Fibrinogen, mg/dl	296	< 70	N/A	259
Ferritin, ng/ml	7,122	138,360	3,3250	> 15,000
BUN, mg/dl	86	161	69	157
Creatinine, mg/dl	0.49	2.32	0.9	1.12
Haptoglobin, mg/dl	< 2	<2	< 5	3

## Poster Walk 2: JDM and vsculitides

### P021 AN INTERNATIONAL CONSENSUS SURVEY OF DEVELOPING CLASSIFICATION CRITERIA FOR JUVENILE DERMATOMYOSITIS-SCLERODERMA OVERLAP

#### Parichat Khaosut^1,2^, Clarissa Pilkington^2^, Lucy R. Wedderburn^2,3,4^, Sandrine C. Lacassagne^2^, on behalf of JDCBS and JDCBS

##### ^1^Paediatrics, Faculty of medicine, Chulalongkorn University, Bangkok, Thailand; ^2^Paediatric Rheumatology, Great Ormond Street Hospital; ^3^ARUK Centre for Adolescent Rheumatology, University College London; ^4^NIHR Biomedical Research Centre, Great Ormond Street Hospital, London, UK

###### **Correspondence:** Parichat Khaosut

**Introduction:** There is no internationally agreed definition of ‘JDM-Scleroderma overlap’ in children and little data on the clinical characteristics, laboratory features and outcome in this specific group of JDM have been published. The goal of this survey is to achieve consensus on a meaningful definition of JDM-Scleroderma overlap in paediatric population and to determine whether overlap features affect outcome and management.

**Objectives:** To develop criteria for the diagnosis of JDM-Scleroderma overlap using an international consensus process.

**Methods:** A survey was circulated to all investigators in the Juvenile Dermatomyositis Cohort and Biomarker Study and Repository (JDCBS) and extended to other members of the Network for JDM in Paediatric Rheumatology European Society (PReS JDM working party) through a Delphi survey. Each individual was asked to identify those clinical manifestations that were felt to be most characteristic to enable them to make the diagnosis of JDM-Scleroderma overlap. The opinions on the importance of identification this subtype of JDM ware collected and the role of myositis autoantibodies has been mentioned in the questionnaire.

**Results:** The survey had a response rate of 26.9% (41 individuals) from both JDCBS and PReS JDM working party. The most common clinical features identified by survey responders as found in JDM patients with scleroderma overlap were sclerodactyly, sclerodermatous skin change proximal to MCP joints, Raynaud phenomenon and ulceration at the tip of fingers, respectively. 95.1% of responders though the scleroderma overlap presentations influence the outcome of JDM, while 86.4% agreed that these features influence the choice of treatment. Interestingly, all of responders (100%) thought that negative myositis autoantibodies could not exclude the diagnosis JDM-Scleroderma overlap, but almost 11% used positive myositis autoantibodies to diagnose this subgroup of JDM. Myositis associated autoantibodies occurring in systemic sclerosis overlap (anti PM-Scl, anti U1-RNP and anti Ku) were thought to be the most commonly associated autoantibodies with JDM-Scleroderma overlap (78%), followed by systemic sclerosis specific autoantibodies (anti Scl70, anti RNA polymerase III) (44%). Although 83% felt that autoantibody profile influences the outcome, only 49% thought that autoantibody profile would influence the choice of medication. 89% of participants in this survey have seen patients with JDM-Scleroderma overlap.

**Conclusion:** This process identified clinical features that clinicians felt to be helpful or important in the diagnosis of JDM-Scleroderma overlap. A further process of secondary survey is necessary to agree an internationally acceptable, clinically usable set of classification criteria.

**Disclosure of Interest**

None Declared

### P022 DEFINING THE RISK OF INTRAVENOUS IMMUNOGLOBULIN RESISTANCE IN NON-ASIAN PATIENTS WITH KAWASAKI DISEASE

#### Maryam Piram^1,2^, Martha Darce^3^, Stéphanie Tellier^4^, Sylvie Di-Filippo^5^, Franck Boralevi^6^, Fouad Madhi^7^, Ulrich Meinzer^8^, Rolando Cimaz^9^, Celine Piedvache^10^, Isabelle Kone-Paut^1,2^, Kawanet study group

##### ^1^Pediatric Rheumatology, CEREMAIA, CHU de Bicêtre; ^2^Univ Paris Sud; ^3^Pediatric Rheumatology, CHU de Bicêtre, Le Kremlin Bicêtre; ^4^Pediatric Rheumatology, Nephrology and Internal medecine, CHU de Toulouse, Toulouse; ^5^Cardiology, Hospices civils de Lyon, Lyon; ^6^Dermatology, CHU de Bordeaux, Bordeaux; ^7^Paediatrics, CHIC, Créteil; ^8^Paediatrics, CHU Robert Debré, Paris, France; ^9^Pediatric Rheumatology, Meyer Hospital, Firenze, Italy; ^10^Clinical Research Unit, CHU de Bicêtre, Le Kremlin Bicêtre, France

###### **Correspondence:** Isabelle Kone-Paut

**Introduction:** About 10-20% of patients with Kawasaki disease (KD) are resistant to intravenous immunoglobulin (IVIg) and are at increased risk of coronary artery abnormalities (CAAs). Early identification is critical to initiate aggressive therapies, but available scoring systems lack sensitivity in non-Japanese populations.

**Objectives:** We investigated the accuracy of 2 Japanese scoring systems and studied factors associated with IVIg resistance in a large multiethnic French population of children with KD to build a new scoring system.

**Methods:** Children admitted for KD between 2011-2014 in 65 centers were enrolled. For patients fulfilling American Heart Association (AHA) criteria, we identified factors predicting resistance to IVIg by multivariate regression analysis. The performance of our score and the Kobayashi and Egami scores were compared in our population and in ethnic subgroups.

**Results:** Overall, 465 children were reported by 84 physicians; 355 (76%) fulfilled AHA criteria (55% European Caucasian, 12% North African/Middle Eastern, 10% African/Afro-Caribbean, 3% Asian and 11% mixed). Eighty patients (23%) needed second-line treatment. Japanese scores had poor performance in our whole population (sensitivity 52-44.5%). Predictors of IVIg resistance were ALT≥30 IU/L, hemoglobin level <10 g/dl, lymphocyte count <1500/mm^3^, modification of extremities, CAAs at initial echocardiography, and time to treatment <5 days. The best sensitivity (80%) and specificity (65%) of this model was with weighted variables and cut-off ≥ 17.5/50 points. The sensitivity remained good in our 3 main ethnic subgroups (78-83%).

**Conclusion:** We identified predictors of IVIg resistance and built a new score with good sensitivity and specificity in a non-Asian population.

**Disclosure of Interest**

None Declared

### P023 THE USE OF INTERLEUKIN 1 RECEPTOR ANTAGONIST (ANAKINRA) IN KAWASAKI DISEASE: A RETROSPECTIVE CASES SERIES

#### Isabelle Koné-Paut^1^, Rolando Cimaz^2^, Jethro Herberg^3^, Oliver Bates^3^, Aurelia Carbasse^4^, Jean Pierre Saulnier^5^, Maria Cristina Maggio^6^, Jordi Anton^7^, Maryam Piram^8^

##### ^1^Pediatric rheumatology, APHP, hôpital de Bicêtre, CEREMAIA, university of Paris Sud, Le Kremlin Bicêtre, France; ^2^Pediatric rheumatology, Meyer hospital, Firenze, Italy; ^3^Pediatrics, Imperial college of London, London, UK; ^4^Pediatric rheumatology, Hôpital arnaud de villeneuve, Montpellier; ^5^Intensive Care Unit, Poitiers university hospital, Poitiers, France; ^6^Pediatrics, University Department Pro.Sa.M.I.G d’Alessandro, Palermo, Italy; ^7^Pediatric rheumatology, Hospital San Joan de Deu, Espluges de Llobregat, Spain; ^8^pediatric rheumatology and dermatology, APHP, hôpital de Bicêtre, CEREMAIA, university of Paris Sud, Le Kremlin Bicêtre, France

###### **Correspondence:** Isabelle Koné-Paut

**Introduction:** Persistent fever and inflammation after infusion of 2g/kg of IVIG, the standard treatment of KD represents a high-risk situation for coronary aneurysms in Kawasaki disease. Identifying patients at risk for IVIG resistance is difficult outside the Asian population, and there remains a critical unmet need to identify an anti-inflammatory treatment that is efficacious in all KD patients. Recent evidence from studies in animals and humans suggest a critical role for interleukin-1 (IL-1) α and β in the pathogenesis of KD.

**Objectives:** To identify the clinical characteristics, reasons for use and response to treatment with anakinra in a retrospective series of patients with Kawasaki Disease (KD).

**Methods:** A retrospective chart review of patients treated with anakinra for KD diagnosed according to the AHA criteria. We compared clinical, biological and echocardiographic characteristics of KD before and after anakinra use. We analysed reasons for use of anakinra, and compared treatment regimens used in 7 European KD referral centres.

**Results:** Eight boys and 3 girls with treatment-refractory KD, aged 4 months to 9 years old, received at least 2 different KD treatments prior to anakinra, which was given on mean at 25 days after disease onset (8 to 87 days). The main reasons for use of anakinra were clinical and biological inflammation, progression of coronary dilatations, and severe myocarditis with cardiac failure. Doses of anakinra ranged from 2 to 8 mg/kg and duration varied from 6 to 81 days. On anakinra treatment, fever disappeared within hours (<24 h) in 3 patients, and within 2 and 6 days in two patients respectively. Six others patients were not febrile at onset of anakinra. In addition, CRP levels fell two to three fold within 48 hours in 7/9 evaluable patients. In terms of its effect on coronary artery dilatation, Z scores decreased in 10/11 patients and increased in one who died suddenly of pericardial hemorrhage.

**Conclusion:** Anakinra used late in the disease course led to a rapid and sustained improvement in clinical and biological inflammation. However, our retrospective analysis did show neither a striking nor a rapid decrease of coronary dilatations and we cannot determine if anakinra itself had an effect on coronary artery dimensions. More robust data will be available soon from the two Phase II ongoing trials, KAWAKINRA using anakinra treatment early after one failure of IVIG treatment (European Clinical Trials no. 2014-002715-41) and ANAKID (ClinicalTrials.gov identifier: NCT02179853), focused on patients with coronary giant aneurysms.

**Disclosure of Interest**

None Declared

### P024 OUTCOME OF 24 PATIENTS WITH CHILDHOOD-ONSET TAKAYASU ARTERITIS: A RETROSPECTIVE STUDY IN TWO TERTIARY CENTRES IN JAPAN

#### Kenichi Nishimura^1^, Seira Hattori^1^, Ayako Murase^1^, Ai Ohnishi^1^, Ryoki Hara^1^, Masao Ogura^2^, Kenji Ishikura^2^, Shuichi Ito^1^

##### ^1^Department of Pediatrics, Yokohama City University Graduate School of Medicine, Yokohama; ^2^Division of Nephrology and Rheumatology, National Center for Child Health and Development, Tokyo, Japan

###### **Correspondence:** Kenichi Nishimura

**Introduction:** Takayasu arteritis (TA) is a chronic large-vessel vasculitis affecting the aorta and its major branches. Glucocorticoids (GCs) can achieve remission in most patients with TA. As most patients suffer relapse after reducing GCs, additional therapy such as methotrexate, azathioprine and intravenous cyclophosphamide (IVCY) has been applied. Recently, the efficacy of TNF-α inhibitors or tocilizumab (TCZ) has been reported. However, there remain insufficient data on clinical characteristics and outcomes of patients with childhood-onset TA, including recent therapeutic advances.

**Objectives:** We aimed to retrospectively analyse the characteristics and outcomes of patients with childhood-onset TA in two tertiary centres in Japan.

**Methods:** Subjects were 24 patients with TA diagnosed when younger than 18 years. Follow-up duration was more than 2 years. Diagnosis of TA was based on the EULAR/PRINTO/PRES criteria. We divided the subjects into two groups: those who did and did not experience relapse. Relapse was defined as NIH criteria for active disease in TA.

We analysed the patients’ background, treatment and complications. We additionally compared two groups according to prednisolone (PSL) reduction rate and whether or not they carried HLA-B52.

Binary data were analysed by Fisher’s exact test, and quantitative data by Mann-Whitney U test. The cumulative relapse rates after the diagnosis were estimated using the Kaplan-Meier method; differences between two groups were assessed by the log-rank test.

**Results:** Median age at onset was 13.0 (range; 0.5-18.0) years old (male:female, 11:13). Thirteen patients (54%) had HLA-B52. Period from onset to diagnosis was 1 (0-66) months. Angiographic classifications were I, IIa, IIb, III, IV and V (n=3, 2, 4, 1, 2 and12), respectively. Four patients and one patient had pulmonary artery involvement and coronary involvement, respectively.

Three patients achieved drug-free remission. Median dose of prednisolone (PSL) was 0.8 (0.4-2.4) mg/kg/day at initiation of treatment and 0 (0-0.3) mg/kg/day at the last visit. Immunosuppressants were used in 13 (54%) from the onset and in 21 (88%) at the last visit. IVCY was used in 10 patients (42%) from the onset, and 4 after the relapse. Some biologics were used in 4 (17%) from the onset, and in 12 (50%) at the last visit.

Twelve patients (50%) experienced relapse, and median number of relapses was 3 (1-6) times. The ratio of relapse was not significantly different between the HLA-B52 positive and negative patients (69% vs 27%, P=0.099). However, median duration from diagnosis to the first relapse was significantly shorter in HLA-B52 positive patients than negative patients (6 vs 51months, P=0.016). The reduction pace of PSL was significantly greater in relapsed patients than non-relapsed patients (0.058 vs 0.025 mg/kg/month, P=0.017). The cumulative relapse rates after the started IVCY or TCZ were not significantly difference between the patient who introduced IVCY (n=14) and TCZ (n=12) (P=0.10).

Nine patients (38%) had cardiovascular complications (aortic valve regurgitation (AR) (n=4), annuloaortic ectasia (AAE) (n=1) and AR+AAE (n=4)). Two patients underwent cardiac surgery. Ten patients had osteoporosis, 2 had surgery for glaucoma and 5 had some mental disorders in their clinical coarse.

**Conclusion:** Approximately half of the patients with childhood-onset TA experienced relapse despite using immunosuppressants and/or biologics. HLA-B52 positivity and rapid reduction rate of PSL significantly increased risk of early relapse. Furthermore, cardiovascular involvement and GCs-related complications were common. Our study suggests the outcome of childhood-onset TA remains unfavourable.

**Disclosure of Interest**

None Declared

### P025 MYOSITIS DAMAGE INDEX IN A COHORT OF INDIAN CHILDREN WITH JUVENILE DERMATOMYOSITIS

#### Santan Godad, Pallavi Pimpale Chavan, Raju P. Khubchandani

##### Pediatric Rheumatology Clinic, Department of Pediatrics, Jaslok Hospital and Research Centre, Mumbai, India

###### **Correspondence:** Pallavi Pimpale Chavan

**Introduction:** International Myositis Assessment and Clinical Studies Group (IMACS) developed the Myositis Damage Index (MDI) structured separately for pediatrics and adults, to provide consistency in myositis outcomes. The MDI documents persistent changes in 11 organ systems thought to be related to damage. There is a dearth of studies on damage caused by Juvenile Dermatomyositis (JDM) especially from less resourced countries and hence our study.

**Objectives:** Primarily to assess the myositis damage index in a cohort of children with JDM from a single centre in Mumbai, India and secondarily to study associations of factors leading to long term damage in these children.

**Methods:** After ethics approval and consents, a total of 23 patients with JDM under regular treatment and *at least* a 2 year follow up, at first study visit were identified. Specifically excluded were children with overlap syndromes eg scleromyositis. The MDI was assessed as *severity* of damage and *extent* of damage at the first study visit and reassessed at a second visit at least *six months* later and only damage present at both visits were scored to give a final severity and extent of damage score. There were no drop outs.

**Results:** 23 children with age range at disease onset 1y - 17.9 y (mean 6.9 y, median 6.8 y, IQR 3.5-8.8) were diagnosed as JDM after a duration of symptoms ranging 1m-30 m (mean 6.8 m, median 5 m, IQR 3-8.5). Their age at the study visit ranged from 5.5 y - 24.8 y (mean 13.4 y, median 12.9 y, IQR 10.4-15.3) after a follow up duration ranging from 2 y - 20.1 y (mean 5.9 y, median 4.2 y, IQR 2.6-8.5, Total 136.5 patient years). The disease course was monocyclic in 14, continuous in 6 and polycyclic in 3. The total MDI *extent of damage* score ranged from 0- 8 / 35 (children) or 37 (adolescents) (mean 2.04, median 2.0, IQR 0.5-2.5) and *severity of damage* score ranged from 0-24.7 /110 (mean 4.7, median 3.5, IQR 1.3-6.4). 17/23 children had damage in one or more organ systems, with 9 showing damage in one organ system, 4 in two organ systems, 3 in three organ systems and 1 child showed damage in six systems. Cutaneous (16/23), endocrine ie growth retardation (6/23) and muscle (5/23) were the most commonly damaged organ systems with 2 children showing skeletal damage while ocular, gastrointestinal and cardiac domains were involved in one each. Importantly, over this duration of follow up there were no children with infection, pulmonary, peripheral vascular disease or malignancy. Correlation between age at onset, gender, time to diagnosis, course of disease and duration of follow up did not yield statistically conclusive results.

**Conclusion:** This is amongst the earliest studies to report damage due to JDM from less resourced countries. Over the median follow up period of 4.2 years about 75% of the children suffer damage in one or more domains, cutaneous damage being the commonest. Uniquely despite high infection rates in the community, this domain was unaffected in our population. Continued enrolment with longer duration of follow up are needed to shed light on factors influencing damage.

**Disclosure of Interest**

None Declared

### P026 PRELIMINARY PROSPECTIVE VALIDATION OF THE JUVENILE DERMATOMYOSITIS ACTIVITY INDEX (JDMAI)

#### Silvia Rosina^1,2^, Giulia Camilla Varnier^1,2,3^, Alessandro Consolaro^1,2^, Pieter van Dijkhuizen^1^, Kiran Nistala^3^, Nicolino Ruperto^1^, Angela Pistorio^1^, Clarissa Pilkington^3^, Angelo Ravelli^1,2^

##### ^1^Istituto Giannina Gaslini; ^2^Università degli Studi di Genova, Genova, Italy; ^3^Great Ormond Street Hospital, London, UK

###### **Correspondence:** Silvia Rosina

**Introduction:** The first composite disease activity score for juvenile dermatomyositis (JDM), named JDMAI, has recently been developed and validated in retrospective patient datasets. It is composed of 4 domains and takes into account the parents’ perception of disease status. The 6 preliminary versions of the tool performed similarly in previous validation analyses.

**Objectives:** To test prospectively the validity of the JDMAI in a sample of JDM patients seen in daily practice.

**Methods:** A total of 53 JDM patients seen consecutively at 2 tertiary-care paediatric rheumatology centers, either at disease onset or at a follow-up visit, a median of 2.3 years after disease onset, were enrolled. Each patient received a retrospective assessment and a cross-sectional evaluation at study entry. Twenty-two patients also underwent a second (prospective) assessment after a median of 3.5 months from study entry. Physician-centered evaluations included assessment of muscle, skin and overall disease activity on a visual analogue scale (VAS) or through the Disease Activity Score (DAS), rating of disease state and course and measurement of disease damage with the Myositis Damage Index (MDI). Laboratory data comprised muscle enzymes and ESR. Parent-reported outcomes were collected through the administration of the Juvenile Dermatomyositis Multidimensional Assessment Report (JDMAR). Validation analyses included assessment of construct validity, internal consistency (with Cronbach’s α), discriminant ability, and responsiveness to change in disease state over time (with standardized response mean, SRM).

**Results:** All JDMAI versions showed strong (r>0.7) correlations with muscle disease activity VAS (0.85-0.87), muscle section of DAS (0.81-0.83), total DAS (0.89-0.92), pain VAS (0.72-0.75), and fatigue VAS (0.92-0.93). The correlations of the different JDMAI versions with the skin index not contained in the specific version (skin disease activity VAS or skin section of DAS) were strong (0.74-0.79). As expected, JDMAI correlations were moderate (r=0.4-0.7) or low (r<0.4) with laboratory parameters (CK, LDH, AST, ALT, and ESR) and with the MDI (0.1-0.11). The SRM was greater in patients judged as improved by the physician or the parent (1.26-1.56) than in those judged as not improved (0.88-1.11). Cronbach’s α was very high (0.89-0.91) for all JDMAI versions. All JDMAI versions discriminated strongly between patients classified in different disease activity states by the physician (p<0.0001) or by the parent (p=0.002-0.005), and between patients whose parents were satisfied or not satisfied with their children’s disease status (Mann-Whitney U test, p=0.0005-0.0008).

**Conclusion:** Validation analyses in this prospective patient sample confirmed that the JDMAI possesses good measurement properties and is a suitable and reliable tool for the assessment of disease activity in children with JDM both in clinical practice and research. Importantly, the new tool revealed a strong capacity to capture the improvement of disease activity over time.

**Disclosure of Interest**

None Declared

### P027 QUALITATIVE NAILFOLD VIDEOCAPILLAROSCOPY ASSESSMENT OF MICROVASCULAR INVOLVEMENT IN JUVENILE DERMATOMYOSITIS

#### Ana V. Villarreal Treviño^1^, Fernando García-Rodríguez^1^, Vicente Baca^2^, Samara Mendieta-Zerón^3^, Manuel de la O-Cavazos^4^, Dionicio Á. Galarza-Delgado^5^, Nadina E. Rubio-Pérez^1^

##### ^1^Pediatric Rheumatology, Hospital Universitario "José Eleuterio González", Monterrey; ^2^Pediatric Rheumatology, Centro Médico Nacional Siglo XXI, Ciudad de México; ^3^Pediatric Rheumatology, ISSEMYM Hospital Materno Infantil, Toluca; ^4^Pediatric; ^5^Rheumatology, Hospital Universitario "José Eleuterio González", Monterrey, Mexico

###### **Correspondence:** Ana V. Villarreal Treviño

**Introduction:** Juvenile dermatomyositis (JDM) is the most common of the pediatric inflammatory myopathies characterized   by inflammatory microvasculopathy.

Nailfold videocapillaroscopy (VCP)  is an in vivo, rapid, and inexpensive imaging technique that allows quantitative assessment of microcirculation. VCP has demonstrated a key role in diagnosis and disease monitoring. The most severe videocapillaroscopy  semi-quantitative changes  previously reported  as loss of capillaries, enlarged loops  and brushy capillaries  are associated with longer  disease duration^1^. There is a lack of  specific quantitative measures of the capillaries  during disease curse.

**Objectives:** To describe  qualitative and  semi-quantitative VCP assessment in Mexican patients with JDM

.

**Methods:** The present study was a cross-sectional observational study that analyzed 192 images from 24 patients with JDM during different  of the disease course, form 3 diferent centers in Mexico. VCP was performed by the same examiner (AVT),  images were obtained  from   all fingers except thumbs of both hands using a videocapillaroscope with a 200x optical probe.  The images were collected, coded, and stored using OptiPix  software (version 1.7.16).Semi-quantitative assessment for architecture were scoredfollowing the international definitions for the capillary abnormalities.2 Quantitative assessment consist in the measurement of the number of capillaries per millimeter, capillary loop length, capillary width,  intercapillary distance  in  micron (μm)^3^.

**Results:** Twenty four patients with JDM were included, 62.5% female. The qualitative assessment  reveal only 4  images  with normal capillaroscopy pattern,   nonspecific alterations  were found in  31.2%.

**Conclusion:** VCP is a noninvasive image method that’s provides a window to microcirculation and allows quantitative assessment of nailfold capillaries with a grave reliability and gave reproducible results. This study found more quantitative alterations than previously reported. A combination of qualitative and quantitative measures can be used for JDM diagnosis, monitoring and response to treatment.

To our knowledge this study is the first in measuring quantitative, semi-quantitative and qualitative parameters of the microvasculature in juvenile dermatomyositis.

**Disclosure of Interest**

None Declared

**Table 1 (abstract P027).** See text for description

	R2	R3	R4	R5 Median (range)	L2	L3	L4	L5
Fingers (n=24)								
Bizarre	2 (0-5)	2 (0-5)	2 (0-5)	2 (0-4)	2 (0-5)	2 (0-7)	2 (0-5)	3 (0-4)
Giant capillary	3 (0-6)	3 (0-7)	3 (0-5)	3 (0-6)	2 (0-6)	3 (0-7)	2 (0-6)	2 (0-6)
Enlarged capillary	3 (0-6)	3.5 (0-7)	4 (0-7)	3.5 (0-8)	3 (0-6)	3.5 (0-7)	4.5 (0-8)	5 (0-8)
Mean in n/mm and in μm (S.D)								
Quantitative								
Density	4.92 (1.79)	5.20 (1.91)	5.08 (2.06)	5.21 (1.28)	5.00 (1.50)	5.71 (2.01)	5.43 (1.67)	6 (2.24)
Total width	75.87	94.02	85.64	71.24	65.55	64.01	72.27	58.9
Total length	299.21	276.05	286.05	284.14	282.41	261.41	291.73	270.99
Intercapillary distance	111.23	104.04	125.62	110.93	109.88	107.66	117.58	98.43

*R2:Right hand second finger, **R3:**Right hand  third finger, **R4:**Right hand fourth finger, **R5:**Right hand fifth finger, **L2:**Left hand second finger**, L3:**Left hand  third finger, **L4:**Left hand fourth finger, **L5:**Left  hand fifth finger

### P028 EMERGING UNIQUE IMMUNE SIGNATURES FOR THE IDIOPATHIC INFLAMMATORY MYOPATHIES: SIMILARITIES AND DIFFERENCES COMPARING ADULT AND JUVENILE DISEASE

#### Meredyth Wilkinson^1,2^, Anna Radziszewska^2^, Elizabeth Jury^3^, Jessica Manson^4^, David Isenberg^3^, Centre for adolescent rheumatology biobank

##### ^1^IIR, Great Ormond Street Institute of Child Health; ^2^Centre for adolescent rheumatology, Division of Medicine; ^3^Rheumatolgy, Division of Medicine, UCL; ^4^Rheumatology, UCLH, London, UK

###### **Correspondence:** Meredyth Wilkinson

**Introduction:** The inflammatory idiopathic myopathies (IIM) are a rare group of myopathic autoimmune diseases diagnosed in both adults and children. Patients’ present with proximal muscle weakness with associated skin changes in dermatomyositis. Immunohistochemical analysis of muscle tissue from these patients has identified immune cell infiltrate and the expression of pro-inflammatory cytokines however, little is known about the peripheral immunological profile in juvenile and adult patient groups.

**Objectives:** To identify specific immune cell signatures and cytokine profiles for disease subtypes and correlate this data with measurements of disease activity.

**Methods:** 44 adult myositis (AM) patients, 15 Adolescent-onset juvenile dermatomyositis (JDM) patients, 25 age-matched adult healthy controls (AHC), and 15 age-matched teenage healthy controls (THC) were recruited with appropriate ethical approval after obtaining written informed consent. Peripheral blood mononuclear cells (PBMC) were isolated from patient and control samples. PBMC from all groups were analysed by flow cytometry to quantify 32 lymphocyte populations including; T-cell, B-cell and monocyte subsets. The AM patients were grouped according to myositis subtype; 19 dermatomyositis (DM), 9 polymyositis (PM), 7 DM + overlap, 4 DM + cancer and 5 PM + overlap). Disease activity was determined by MITAX score and clinicians’ decision on treatment; remission off treatment, remission/mildly active/active on treatment. Cell populations were analysed by group for differences in fold change/adjusted p value (1-way ANOVA with Tukey’s multiple comparison).

**Results:** There was an idenified T cell signature in ADM (increased Th17, Tregs and CD4+ naïve), a B cell signature in APM (CD27+/- B cell ratio) and a T cell signature in JDM (CD4/CD8+ T cell ratio). A decrease in CD8+ central memory T-cells was observed in both AM and JDM patients compared to healthy controls, this reduction was most significant in active disease. AM patients also had reduced CD27+ memory B-cells compared to both JDM patients and healthy controls. AM patients in remission off treatment had increased non-classical monocyte subset frequencies, whereas JDM patients off treatment had elevated CD8+ EMRA T-cells. Th17 cells were significantly increased in ADM compared to JDM samples (mean = 3.68 vs. 0.64, adjusted p value = 0.0032). More specifically increased Th17 cells were found in ADM but not APM (mean = 3.67 vs. 0.68, adjusted p value = 0.0019). However, the Th17 population was highest in AM patients that were in remission on treatment (remission on treatment vs AHC, mean = 2.68 vs. 0.84, adjusted p value = 0.038).

**Conclusion:** This group of diseases are notably heterogeneous both clinically and immunologically as shown by the complex immunophenotyping patterns identified within myositis subgroups. This study identified signatures unique to adult compared to juvenile disease, and between AM patients on treatment and during remission. The Th17 signature concurs with studies pinpointing these cells within the muscle tissue. This data supports the need for further investigation for the use of IL-17A inhibitors in the treatment of the IIM.

**Disclosure of Interest**

None Declared

### P029 VALIDATION OF THE EULAR/ACR CLASSIFICATION CRITERIA FOR JUVENILE IDIOPATHIC INFLAMMATORY MYOSITIS IN JAPAN

#### Kazuko Yamazaki^1^, Akiko Ohta^2^, Shinji Akioka^3^, Tomo Nozawa^4^, Asami Ohara^4^, Haruna Nakaseko^5^, Yuichi Yamasaki^6^, Norimoto Kobayashi^7^, Yutaka Nishida^8^, Satoshi Sato^9^, Shunichiro Takezaki^10^, Naomi Iwata^5^, Ichiro Kobayashi^10^, Masaaki Mori^11^

##### ^1^Pediatrics, Saitama Medical Center, Saitama Medical University, Kawagoe; ^2^Division of Public Health, Department of Social Medicine, Saitama Medical University Faculty of Medicine, Moroyama; ^3^Pediatrics, Kyoto Prefectural University of Medicine, Kyoto; ^4^Pediatrics, Yokohama City University School of Medicine, Yokohama; ^5^Infection and Immunology, Aichi Children’s Health and Medical Center, Ohfu; ^6^Pediatrics, Graduate School of Medical and Dental Sciences, Kagoshima University, Kagoshima; ^7^Pediatrics, Shinshu University School of Medicine, Matsumoto; ^8^Pediatrics, Gunma University Graduate School of medicine, Maebashi; ^9^ Infectious Diseases and Immunology, Saitama Children's Medical Center, Saitama; ^10^Pediatrics, Hokkaido University Graduate School of Medicine, Sapporo; ^11^Lifetime Clinical Immunology, Tokyo Medical and Dental University, Tokyo, Japan

###### **Correspondence:** Kazuko Yamazaki

**Introduction:** There are various diagnostic and classification criteria for myositis, but they are not always suitable for juvenile idiopathic inflammatory myositis (JIIM). To establish standard diagnostic criteria, the International Myositis Classification Criteria Project (IMCCP) published the EULAR/ACR classification criteria for idiopathic inflammatory myositis (IIM) in 2017.

**Objectives:** To evaluate the validity of the EULAR/ACR classification criteria with JIIM cases in Japan.

**Methods:** We used clinical and laboratory data from a group of JIIM patients and a comparator group comprised of non-JIIM patients with mimicking conditions collected at seven major pediatric rheumatology centers in Japan between 2008 and 2015. A diagnostic confirmation made at least 6 months prior to the study by a pediatric rheumatologist was used as the inclusion criterion for this study.

**Results:** The total of 117 patients (68 JIIM patients and 49 non-JIIM patients) were included to the study. For the validation analysis, we applied 55% probability cutoff, which corresponds to “probable IIM”, based on the IMCCP study. The IMCCP’s diagnostic standard gave us an almost equivalent sensitivity (86.7% without muscle biopsies; 92.1% with muscle biopsies) and a higher specificity (100% without/with muscle biopsies) compared to the data reported in the IMCCP study. We also confirmed an improved sensitivity with the EULAR/ACR classification criteria, compared to other traditional diagnostic criteria (e.g. 86.7 % with the EULAR/ACR criteria vs 80.9% with the Bohan and Peter criteria). In terms of the diagnostic accuracy, the agreement proportions between the classification subgroups of the EULAR/ACR criteria and the physician-diagnosed subgroups in our dataset were 100% for Juvenile amyopathic dermatomyositis (DM) and Juvenile hypomyopathic DM. The proportions were 93.2% for JDM and 43.2% for Juvenile polymyositis (JPM), for seven cases (3 JDM patients and 4 JPM patients) were not properly classified as JIIM.

**Conclusion:** Our validation study with Japanese JIIM cases indicates that the EULAR/ACR classification criteria for IIM generally perform better than existing diagnostic criteria for myositis in diagnosing JIIM.

**Disclosure of Interest**

None Declared

## Juvenile dermatomyositis

### P030 A CHILD WITH SEVERE NXP2-POSITIVE JUVENILE DERMATOMYOSITIS SUCCESSFULLY TREATED WITH RUXOLITINIB

#### Florence A. Aeschlimann^1^, Marie-Louise Fremond^1,2^, Darragh Duffy^3^, Gillian I. Rice^4^, Jean-Luc Charuel^5^, Vincent Bondet^3^, Benedicte Neven^1^, Christine Bodemer^6^, Laurent Balu^7^, Anne Hulin^8^, Cyril Gitiaux^9^, Yanick J. Crow^2^, Brigitte Bader-Meunier^1,10^

##### ^1^Pediatric Immunology, Hematology and Rheumatology, Necker - APHP; ^2^Laboratory of Neurogenetics and Neuroinflammation, INSERM UMR 1163; ^3^Immunobiology of Dendritic Cells, Institut Pasteur, Paris, France; ^4^Division of Evolution and Genomic Sciences, Manchester Academic Health Science Centre, Manchester, UK; ^5^Laboratory of Immunology, La Pitié-Salpêtrière; ^6^Dermatology and Pediatric Dermatology, Necker - APHP, Paris; ^7^Pediatrics, CHU de La Réunion, Saint Denis; ^8^Pharmacology and toxicology laboratory, Mondor, Créteil; ^9^Pediatric Neurolophysiology and referral center for neuromuscular diseases, Necker - APHP; ^10^Laboratoty of Immunogenetics of paediatric autoimmune diseases, INSERM UMR 1163, Paris, France

###### **Correspondence:** Florence A. Aeschlimann

**Introduction:** Juvenile dermatomyositis (JDM) is a rare and heterogeneous inflammatory myopathy. Clinical features may be severe and disease course refractory to standard treatment with corticosteroids and methotrexate (MTX). Evidence for a critical role of the type I interferon (IFN) pathway in the pathophysiology of dermatomyositis exists in both childhood and adult forms of the disease.

**Objectives:** To report the first case of severe vasculopathic JDM successfully treated with the JAK 1/2 selective inhibitor ruxolitinib.

**Methods:** Retrospective chart review from initial presentation to last follow-up. We assessed type I IFN status by IFNα digital-ELISA quantification in serum, STAT1 phosphorylation and RNA expression of IFN-stimulated genes in whole blood.

**Results:** A previously healthy 13-year-old girl presented with cutaneous lesions and a severe proximal muscle weakness (Childhood Muscular Assessment Score, CMAS 2/52, and Manual Muscle Testing, MMT 38/80) characteristic of JDM. CK level was elevated and anti-NXP2 antibodies positive. Muscle biopsy revealed severe vasculopathic lesions. Despite treatment with prednisone (1.2mg/kg/day) and MTX, she developed tetraplegia, dysphonia, dysphagia, intestinal involvement, and new skin lesions. Treatment escalation with IVIG, plasma exchange (5/week), rituximab, and increased prednisone resulted in clinical improvement. Thus, plasma exchanges were subsequently spaced out and discontinued 6 months after diagnosis. However, three weeks later, recurrent muscle weakness necessitated the restart of plasma exchanges, increase of prednisone, a 4^th^ dose of rituximab, and initiation of mycophenolate mofetil (MMF). While prednisone and the frequency of plasma exchanges were slowly tapered, she developed two further muscular, cutaneous and intestinal relapses, as well as a diffuse fascia calcinosis, at 12 and 18 months after diagnosis. High levels of IFNα protein in the serum (238, 75, and 46 fg/mL, normal < 10 fg/mL) and an increased expression of ISGs were documented. A constitutive phosphorylation of STAT1 and STAT3 was recorded in T lymphocytes and monocytes from the patient compared with controls. Considering these results, ruxolitinib was started (10mg x 2/day). Rituximab and MMF were discontinued. Within 2 months, CMAS and MMT scores increased from 47 to 52 and from 57 to 79, respectively. Plasma exchanges were discontinued and prednisone decreased. Eight months post initiation of ruxolitinib, JDM was clinically inactive according to the PRINTO criteria and prednisone was tapered (0.15mg/kg/day). Ruxolitinib was well tolerated. Serum levels of IFNα stabilized and STAT1 phosphorylation in monocytes was comparable to a control 4 hours after drug intake. Informed parental consent was obtained for the use of ruxolitinib on a compassionate basis and for conducting the experiments.

**Conclusion:** Efficacy of JAK1/2 inhibitors has previously been reported for severe dermatomyositis in adults and for certain monogenic interferonopathies in children. Our findings suggest that JAK inhibition may also represent an efficacious and well-tolerated therapeutic option in a subset of patients with recalcitrant JDM. Informed consent for publication had been obtained from the parents.

**Disclosure of Interest**

None Declared

### P031 UNDERSTANDING THE LIVED EXPERIENCE OF JUVENILE DERMATOMYOSITIS

#### Polly Livermore^1,2^, Suzy Gray^3^, Kathleen Mulligan^4^, Jennifer Stinson^5^, Lucy R. Wedderburn^6^, Faith Gibson^7,8^

##### ^1^Institute of Child Health, University College London Great Ormond Street Institute of Child Health; ^2^Orchid, Nursing Research, Great Ormond Street Hospital for Children; ^3^Psychology/Rheumatology, Evelina Hospital; ^4^School of Health Sciences, City, University of London, London, UK; ^5^Chronic Pain, Hospital for Sick Children, Toronto, Canada; ^6^Rheumatology/NIHR GOSH Biomedical Research Centre, University College London Great Ormond Street Institute of Child Health, London; ^7^Nursing Research, University of Surrey, Surrey; ^8^Orchid, Nursing Research, Great Ormond Street Hospital for Children, London, London, UK

###### **Correspondence:** Polly Livermore

**Introduction:** Juvenile Dermatomyositis (JDM) is a rare, potentially life threatening condition. Beginning with mild fatigue and weakness, the condition can occur at any age, and currently there is no known cure. Some children and young people can experience fluctuations between remission and relapse throughout their childhood, into adolescence and beyond. There is very little evidence about the psychological impact this condition has on children and young people, and none that has asked them directly, however, through experience from clinical practice and conversations with those affected, we know the effects can be profound.

**Objectives:** The aim of this study was to identify the lived experience of children and young people between the ages of 8 and 19 years of age diagnosed with JDM. This study constitutes the first part of a larger mixed-methods study.

**Methods:** Ethical approval was gained to interview children and young people who were enrolled in a wider cohort study. Young people were interviewed either using creative arts based methods, such as drawing in a comic book and parallel discussion, or with a standard verbal interview. Young people were all asked: ‘what it is like to have JDM?’ Further questions were used to probe or to add clarity. Interviews took place when attending a specialist Rheumatology clinic or the inpatient Rheumatology Ward of a tertiary specialist Rheumatology Centre or during a specially arranged home visit.

**Results:** Fifteen young people offered to share their story, their demographics are shown in Table 1. The interviews lasted between 18 to 130 minutes and all, with permission, were audio recorded. They were transcribed verbatim and analysed using Interpretive Phenomenology.

The overarching metaphor of a ‘Rollercoaster’ was developed from the interview transcripts, to depict the ups and downs of having JDM. From initial difficulties with diagnosis by local health professionals of a rare disease to feeling comfort at getting a diagnosis, to the low of not being able to walk, to the relief when medications start to work and some improvement is felt. The ‘Rollercoaster’ metaphor is visual, easy to explain and understand, and will allow young people to feel some support from seeing the journey ahead.

Within this overarching construct, there are five themes which emerged from the data. These are: being-confused in the beginning; being-different; sick-steroidal-and-scared of the medicines; being-uncertain; and having-acceptance, as the JDM journey continues for many years.

**Conclusion:** This study is the first to ask children and young people what it is like to have JDM, and to recount the ‘ups and downs’ of their lives and describe the ways that JDM affects them. The results from this study will lead to a wider study asking young people all over the United Kingdom to share their story through surveys examining aspects which have been highlighted so far, this will lead into dissemination workshops to share the results found not only with children, young people and their families, but also with health professionals.

**Disclosure of Interest**

P. Livermore Grant / Research Support from: Doctoral Nursing Fellowship funded by NIHR HEE/CDRF, S. Gray: None Declared, K. Mulligan: None Declared, J. Stinson: None Declared, L. Wedderburn Grant/Research Support from: NIHR GOSH BRC & Arthritis Research UK, F. Gibson: None Declared

**Table 1 (abstract P031).** Demographics of the 15 young people interviewed

Gender	6 males, 9 females
Ethnicity	12 White British, 3 other
Age at interview	8 – 18 years (median 12 years)
Age diagnosed	2 – 16 years (median 8 years)
Disease duration	3 weeks – 16 years (median 5 years)
Disease severity	From acute inpatients to remission off medicines

### P032 DISTINCT CLINICAL FEATURES BETWEEN ADULT AND JUVENILE DERMATOMYOSITIS PATIENTS

#### A. T. Melo^1,2^, S. Sousa^3^, A. Cordeiro^3^, I. Cordeiro^1,2^, P. Costa-Reis^4^, F. Oliveira-Ramos^1,2^, J. E. Fonseca^1,2^, R. Campanilho-Marques^1,2^

##### ^1^Unidade de Reumatologia Pediátrica, Serviço de Reumatologia e Doenças Ósseas Metabólicas, Hospital de Santa Maria, CHLN, Centro Académico de Medicina de Lisboa; ^2^Unidade de Investigação em Reumatologia, Instituto de Medicina Molecular, Faculdade de Medicina, Universidade de Lisboa, Centro Académico de Medicina de Lisboa, Lisboa; ^3^Serviço de Reumatologia, Hospital Garcia de Orta, Almada; ^4^Serviço de Pediatria, Hospital de Santa Maria, CHLN, Lisboa, Portugal

###### **Correspondence:** A. T. Melo

**Introduction:** Adult and juvenile patients share several hallmark features of dermatomyositis (DM) but important clinical differences may reflect different disease triggers between children and adults.

**Objectives:** We aimed to compare the disease characteristics between children with juvenile dermatomyositis (JDM) and adults with DM and to explore the presence of nailfold capillaroscopy (NFC) changes abnormalities between the two populations.

**Methods:** Data were analyzed from children and adults who met Bohan-Peter criteria for DM. Childhood Myositis Assessment Scale (CMAS), Manual Muscle Testing (MMT8), muscle enzymes and physicians global assessment (PGA) were recorded at the time of last clinical appointment. Skin disease was assessed using modified skin disease activity score (DAS). By NFC was assessed nailfold capillary density, capillaries shape, presence of hemorrhage and neovascular pattern. Fisher's test was used to test differences between categorical variables and Mann-Whitney U test for continuous not-normally distributed variables.

**Results:** In total 23 patients were analyzed and compared (13 JDM and 10 adult DM). The female:male ratio was 9:4 in JDM and 9:1 in DM. The median age at disease onset was 5.6 years [2.7-10.1] for JDM and 50.6 years [43.3-60.4] for adult DM, the median disease duration was 8.4 years [4.4-15.38] for JDM and 2.3 years [1.2-12.5] for adult DM (p=0.1) and the median time of disease follow-up was 6.7 years [4.3-9.3] for JDM and 2.3 years [1.3-11.3] for adult DM. Table 1 reports the clinical characteristics for both groups. Adult DM had a significantly higher proportion of lung involvement and ANA positivity compared with JDM (p=0.002 and p=0.02, respectively). Dilated capillaries, giant capillaries, hemorrhage and neovascular pattern were found in 86%, 0%, 29% and 14% of JDM patients, respectively, and in 71%, 26%, 43% and 29% of adult patients, respectively, but with no statistically significant difference between children and adults. Clinically inactive disease was found in 38% and 10% of JDM and adult DM patients, respectively. All of those children have stopped medication. There was one case of malignancy in an adult DM patient.

**Conclusion:** Nailfold microvascular abnormalities were found in most of the NFC parameters, in JDM patients assessed for after a median of 8 years of disease, which might reflect the long-term microangiopathy associated with this disease. Regarding the studied clinical features, in our cohort, the adult DM patients have higher lung involvement when compared to JDM.

**Disclosure of Interest**

None Declared

**Table 1 (abstract P032).** Clinical characteristics of children with JDM and adults with DM

Clinical Characteristics	JDM (n=13)	Adult DM (=10)	P value
MMT 80 median [IQR]	78 [73-80]	78 [67-80]	ns
DAS skin median [IQR]	2 [0.5-2]	0.5 [0-2.5]	ns
PGA median [IQR]	0.8 [0-3.5]	2 [0-7]	ns
Presence of Calcinosis (%)	7 (54%)	2 (20%)	ns
Arthritis (%)	6 (48%)	3 (30%)	ns
Interstitial lung disease (ILD) (%)	1 (8%)	7 (70%)	p=0.002
ANA positivity (%)	6 (46%)	10 (100%)	p=0.02
CK U/L median [IQR]	93 [70.5-668]	46 [43-134]	ns
Monotherapy/ Biologic agents with other DMARDs	4 (31%)/3 (23%)	3 (30%)/1 (10%)	ns/ ns

### P033 JUVENILE DERMATOMYOSITIS: DESCRIPTION OF A COLOMBIAN COHORT

#### Angela C. Mosquera^1^, Clara Malagon^1^, Maria del Pilar Gomez^2^, Tatiana Gonzalez^3^, Ricardo Yepez^2^, Camilo Vargas^4^, on behalf of GRIP and Grupo de Reumatología e Inmunología Pediátrica (GRIP) Colombia

##### ^1^Pediatric Rheumatology, UNIVERSIDAD EL BOSQUE, Bogota; ^2^Pediatric Rheumatology, Postgraduate of pediatrics, Libre University, Cali; ^3^Pediatric Rheumatology, Postgraduate of pediatrics, Cartagena University, Cartagena; ^4^Pediatric Rheumatology, Postgraduate of pediatrics, Del Valle University, Cali, Colombia

###### **Correspondence:** Angela C. Mosquera

**Introduction:** Idiopathic myopathies are heterogeneous disorders characterized by muscle weakness and inflammation. Dermatomyositis is the most frecuent subgroup in pediatrics. Juvenile Dermatomyositis (JDM) in addition to muscle involvement is characterized by typical skin rashes (Gottron´s sign and heliotrope rash). Recently, a new proposal of diagnostic criteria was presented but patients at debut may had a great variety of manifestations due to the multisystemic nature of this condition. Close to the diagnosis and in the long term, a great variety of complications can occur and affect the survival and the quality of life of patients

**Objectives:** To descrive debut and course of a group of Colombian patients with Juvenile dermatomyositis

**Methods:** Multi-center, retrospective, descriptive, case series study. The review of medical records with a unique format was carried out in Bogotá, Cali, Cartagena and Barranquilla. The data analysis was performed with SPSS 20

**Results:** N = 94 patients. 67% female with an average age at diagnosis of 7,78 years (range of 1-18 years) and follow-up time of 46 months (SD 39 months). The average time between the onset of symptoms and the definitive diagnosis was 7,6 weeks (1-60). In 37% of patients, skin manifestations preceded muscle involvement and was moderate-severe in 67%. Clinical manifestationts at debut: 88% heliotrope rash, 90% Gottron´s sing, malar erythema 78%, periungueal erythema 67%, skin ulcers 25%, 37% arthritis (31% polyarticular), pulmonary compromise 4%, vasculitis 14% fever 19% and 25% calcinosis (22% mild-moderate). Seventy five percent of patients had CPK < 5000 at debut and 49% had positive antinuclear antibodies. Monocyclic course in 53% of patients. During the follow-up 33% presented calcinosis, 44% muscular atrophy, 52% persistent muscular weakness, 14% osteoporosis and 14% short stature. 99% of patients received steroid at debut (44% intravenous) and 86% during follow-up. 97% received methotrexate and 13% required a biological medication (92% Rituximab). 70% of the patients were classified by the treating rheumatologist as adherent to the treatment and 14% of patients had associated other autoimmune diseases. Patients with mild skin involvement at debut had a higher frequency of monocyclic course (p 0,009)

**Conclusion:** The skin involvement can be the initial manifestation in an important percentage of patients with JDM. The severity of skin involvement can be variable and related to the type of course. JDM requires an intensive, dynamic and monitored management that allows a rapid control of the disease, monitor the appearance of short and long term complications and the association with other autoimmune diseases

**Disclosure of Interest**

None Declared

### P034 POST-STREPTOCOCCAL POLYMYALGIA (3 CASES): PROPOSED COIMBRA CRITERIA

#### João Nascimento^1^, Paula Estanqueiro^1^, Teresa S. Simão^2^, Manuel Salgado^1^

##### ^1^Pediatric Rheumatology Unit, Hospital Pediátrico Coimbra - CHUC, Coimbra; ^2^Pediatrics, Hospital da Senhora da Oliveira, Guimarães, Portugal

###### **Correspondence:** João Nascimento

**Introduction:** Post-streptococcal syndrome consists of a wide spectrum of non-suppurative disorders: erythema nodosum, uveitis and/or scleritis, post-streptococcal glomerulonephritis, periostitis, vasculitis, rheumatic fever and post-streptococcal reactive arthritis. It can also include an entity with myalgia as the solitary presenting symptom called post-streptococcal polymyalgia (PSPM).

**Objectives:** To alert to clinical features of PSPM and present *Coimbra classification criteria*.

**Methods:** Retrospective study. Selection of the patients with post-streptococcal reactive polymyalgia followed in Coimbra’s pediatric rheumatology department in the last 30 years.

Patients were included based on the following criteria (all):

**1)** Diffuse myalgia – acute onset, incapacitating and severe pain;

**2)** Palpation induced muscle pain;

**3)** Normal serum creatine kinase (CK);

**4)** Supporting evidence of a recent preceding streptococcal infection (elevated streptococcal antibodies, or (+) cultures or rapid antigen tests for streptococcus groups;

**5)** No Exclusion criteria: a) > 1. major manifestations of the revised Jones criteria for rheumatic fever (2015); b) periostitis.

**Results:** The unit followed 119 children with poststreptococcal reactive musculoskeletal symptoms: 61 rheumatic fever, 55 poststreptococcal reactive arthritis and 3 had only diffuse myalgia - acute onset, incapacitating and severe pain (one girl and two boys).

The mean age at diagnosis was 5,7 years. Tonsillitis was the previous streptococcal infection in all patients (one scarlet fever). The time span between the infection and myalgia presentation had a variation between 25 and 30 days. The longest myalgia duration was nine weeks and the shortest was one week and four days. Lower limbs were involved in all patients and upper limbs in two. Arthralgia without arthritis was present in two patients. At diagnosis visual analog score (VAS) of muscle pain was ≥ 80 mm and all patients had elevation of inflammatory markers (erythrocyte sedimentation rate between 86 and 102mm/h and C-reative protein between 4,9 and 6 mg/dl) and normal creatine kinase (CK) values. The antistreptolysin O titre (ASLO) performed in all patients were strongly positive (≥ 800 U/l and ≤ 1820 U/I) and echocardiography was normal. Two patients had full recovery with NSAIDs but one refractory patient also needed prednisolone (1mg/kg) during four weeks.

**Conclusion:** Severe diffuse pain and tenderness of skeletal muscles without accompanying arthritis is an under-recognized feature of post-streptococcal disease. High ASLO titre with normal CPK in a child with myalgia should alert PSPM diagnosis. We propose the *Coimbra diagnostic criteria* for PSPM.

**References**

1. Jansen TL, Janssen M, Macfarlane JD, de Jong AJ. Poststreptococcal reactive myalgia: a novel syndrome secondary to infection with group A or G streptococci. Br J Rheumatol. 1998;37:1343–8

2. Subramanian S, Carty JE, Gaywood I. A rare case of recurrent poststreptococcal myalgia. Rheumatology. 2002;41:827–8

3. Arun Babu T, Venkatesh C. Post Streptococcal Myalgia: An Under-recognized Clinical Syndrome. Indian J Pediatr. 2012; 79(3):383–385

**Disclosure of Interest**

None Declared

### P035 EFFECTIVENESS OF ULTRASONOGRAPHY FOR MYOSITIS SCREENING IN CLINICALLY AMYOPATHIC JUVENILE DERMATOMYOSITIS: A CASE SERIES

#### Kazutaka Ouchi^1,2^, Shinji Akioka^1^, Hiroshi Kubo^1^, Norio Nakagawa^1^, Hajime Hosoi^1^

##### ^1^Pediatrics, Kyoto Prefectural University of Medicine, Kyoto; ^2^Pediatrics, Ayabe city hospital, Ayabe, Japan

###### **Correspondence:** Kazutaka Ouchi

**Introduction:** Juvenile dermatomyositis (JDM) is a systemic autoimmune capillary vasculopathy that predominantly affects the muscles and cutaneous tissues. In JDM cases, myosonography can reportedly be used to evaluate disease activity and perform follow-up monitoring. However, the effectiveness of myosonography in clinically amyopathic JDM (CA-JDM) is undetermined.

**Objectives:** To investigate the myosonographic findings in patients with CA-JDM.

**Methods:** Myosonography was performed in three patients with CA-JDM. Case 1 was a 2-year-old male with full range of motion in manual muscle testing (MMT), positive anti-TIF-1γ antibody results, and superficial perivascular dermatitis; case 2 was a 4-year-old male with full range of motion in MMT, negative myositis-specific autoantibody results, and superficial perivascular dermatitis; case 3 was a 14-year-old female with full range of motion in MMT, positive anti-MDA5 antibody results, interstitial pneumonia, and superficial perivascular dermatitis. Case 3 had minimal evidence of myositis on MRI and in a biopsied specimen. Laboratory testing revealed normal creatinine kinase concentration in all three cases. The ultrasonographic assessment was performed in the bilateral biceps brachii and quadriceps femores.

**Results:** In case 1, the right biceps and quadriceps had increased echo intensity compared with the left side, and the power Doppler signal was positive on the perimysium in the left biceps and quadriceps. In case 2, the echo intensity was increased in the bilateral biceps and quadriceps, and there was a positive power Doppler signal on the perimysium in all muscles. In case 3, there was a high echo intensity and positive power Doppler signal on the perimysium in the left quadriceps, and there was a suspected positive power Doppler signal on the epimysium in the left quadriceps.

**Conclusion:** Myosonography detected muscle abnormalities in CA-JDM. Myosonography may be effective for JDM screening in patients with no clinical muscular abnormalities who are suspected of having JDM due to skin characteristics. Informed consent to publish had been obtained from the parents.

**Disclosure of Interest**

None Declared

## Vasculitides I

### P036 EARLY FEATURES OF KAWASAKI DISEASE WITH PYURIA IN FEBRILE INFANTS YOUNGER THAN 6 MONTHS

#### Jong Gyun Ahn^1^, Young Dae Kim^2^, Kwang Nam Kim^3^

##### ^1^Department of Pediatrics, Severance Children's Hospital, Yonsei University College of Medicine, Seoul; ^2^Department of Pediatrics, Ilsan Paik Hospital, Inje University College of Medicine, Goyang; ^3^Department of Pediatrics, Hallym University Sacred Heart Hospital, Hallym University College of Medicine, Anyang, Korea, Republic Of

###### **Correspondence:** Jong Gyun Ahn

**Introduction:** Children with Kawasaki disease (KD) and pyuria have been misdiagnosed with urinary tract infection (UTI).

**Objectives:** We compared clinical and laboratory features at admission between two groups of infants under 6 months of age, who showed initial pyuria, to find useful clinical clues to the initial suspicion of KD.

**Methods:** We reviewed the medical records of febrile infants under 6 months of age with pyuria over a 10-year period (2007-2017). We included infants with sterile pyuria who were finally diagnosed with KD and those with UTI.

**Results:** During the 10-year study period, a total of 121 patients under aged 6 months were diagnosed as KD. 41 (33.9%) of the 121 KD children had pyuria. Of the 41 pyuria patients, 12 had sterile pyuria, 7 had concomitant UTI, and 16 had combined bacterial growth which had to be excluded. The other 6 patients were unidentified because they did not perform the urine culture tests. Two patients satisfied the diagnostic criteria of complete Kawasaki disease, whereas the others were diagnosed with incomplete Kawasaki disease.

During the same period, 378 infants under 6 months of age were diagnosed with UTI. We compared the clinical and laboratory features between the 12 KD infants with sterile pyuria and the 378 UTI infants. The median age of the KD group (5.2 months; range,2.5-5.9 months) was higher than that of the UTI group (3.6 months; range 0.9-6 months) (*P* =.002). The male/female ratios in the KD and UTI infants were 1.4 (7/5), and 2.8 (277/101), respectively.

The days of fever before admission and total duration of fever were longer in the KD group than in the UTI group (*P* < 0.01). Compared with the UTI group, the KD group demonstrated a significantly higher platelet count (*P* = .04), level of C-reactive protein (CRP) (*P* < 0.01) and erythrocyte sedimentation rate (ESR) (*P* < 0.01). UTI group showed higher presence of positive urine nitrite test (*P* < 0.01). No significant differences were evident in blood leukocyte count, absolute neutrophil count, haemoglobin, alanine transaminase, aspartate aminotransferase, albumin, sodium, total bilirubin and urine β2-microglobulin (β2-MG)

**Conclusion:** Our study can provide early clues to suspect KD in febrile infants with pyuria.

**Disclosure of Interest**

None Declared

### P037 ACUTE PANCREATITIS ASSOCIATED WITH TWO COMMON CHILDHOOD VASCULITIS: KAWASAKI DISEASE AND HENOCH-SCHÖNLEIN PURPURA

#### Nuray Aktay Ayaz, Gul Karadag, Ayse Zopcuk

##### Pediatric Rheumatology, Saglik Bilimleri University KSSEAH, Istanbul, Turkey

###### **Correspondence:** Nuray Aktay Ayaz

**Introduction:** Henoch Schönlein purpura (HSP) and Kawasaki disease (KD) are the most common childhood vasculitis. Rare conditions like pancreatitis, aseptic menengitis, pneumonia do not belong to the classical criteria of KD and defined as atypical KD. Similarly gastrointestinal involvement are excepted features of HSP. Few cases of HSP and KD related pancreatitis have been reported. Herein, we report two acute pancreatitis cases associated with KD and HSP.

**Objectives:** A 10-year-old boy consulted with a 6-days history of fever, abdominal pain, vomiting and a scarlatiniform rash. Initial blood tests showed Hb 9.7 g/dl, WBC 14.2 × 10 ^9^/L, platalet count 442 ×10 ^9^/L, C-reactive protein 68.8 mg/L, erythrocyte sedimentation rate 65 mm/hr, albumine: 2.8 g/dL, increased amylase of 639 U/L and lipase 2056 U/L. In echocardiographic examination myocarditis was seen and pro-BNP level was 11418 pg/ml. Intravenous immünoglobulin (IVIG) at a dose of 2 g/kg and aspirin 40 mg/kg/day was given with a preliminary diagnosis of atypical Kawasaki disease. The patient became afebrile 24 hours later. In the second week of the disease thrombocytosis (platelet count, 780 ×10 ^9^/L) and periungual desquamation of his fingers and toes were observed. Coronary arteries were normal and cardiac contractions were good in echocardiography. His pancreatitis had resolved with a normal amylase of 97 U/L and lipase of 29 U/L within two weeeks.

**Methods:** A 6-year-old girl was referred with a 4-days history of vomiting and abdominal pain. Her physicial examination was normal except abdominal tenderness. Initial investigations showed Hb 14.4 g/dl, WBC 41.8 × 10 ^9^/L, platalet count 443 ×10 ^9^/L, C-reactive protein 50.6 mg/L, erythrocyte sedimentation rate 1mm/hr, albumin: 1.9 g/dL. In abdominal ultrasonograpy hydrops of gallbaldder was detected. Few days later purpuric rash on her legs was observed. She was diagnosed as HSP and prednisolone at a dose of 2 mg/kg/day was given to her. Despite steroid treatment, back and chest pain added to abdominal pain. Increased amylase of 344 U/L and lipase 294 U/L levels were detected at the seventh day of disease, but ultrasonographic pancreatitis was not observed. In addition to fat free diet intravenous fluids and pulse steroid treatment (30 mg/kg, for 3 days) was given. In the second week of the disease proteinüria was detected in 24 hour urine testing, and mesengioproliferative glomerulonephritis was diagnosed in renal biopsy. Pulse cyclophosphamide treatment was started her for HSP nephritis. Her pancreatitis resolved within a month

**Results:** HSP and KD are most common types of vasculitis in children. But, HSP and KD associated pancreatitis is uncommon. There are autopsy studies about KD associated pancreatitis. The association between pancreatitis and HSP is rare.

**Conclusion:** In conclusion HSP and KD related pancreatitis is rare. HSP-related pancreatitis should be considered when abdominal pain is prolonged and present with changing character of pain. KD should be considered in the presence of acute pancreatitis and prolonged fever even the patient does not meet all the criteria. Informed consent to publish had been obtained.

**Disclosure of Interest**

None Declared

### P038 CAN WE PREDICT THE COURSE OF KAWASAKI DISEASE: A SINGLE CENTER SERIES FROM THE EASTERN MEDITERRANEAN

#### Elif Arslanoglu Aydin^1^, Erdal Sag^2^, Selcan Demir^2^, Hafize E. Sönmez^2^, Ezgi D. Batu^2^, Yelda Bilginer^2^, Seza Ozen^2^

##### ^1^Department of Pediatrics; ^2^Division of Pediatric Rheumatology, Department of Pediatrics, Hacettepe University, Ankara, Turkey

###### **Correspondence:** Elif Arslanoglu Aydin

**Introduction:** Kawasaki disease (KD) is one of the most common vasculitis of childhood. Coronary artery involvement is the most important complication of the disease and the major cause of the mortality. IVIG resistance is defined as persisting or recurring fever at least 36 hours after the end of intravenous infusion of immunoglobulin (IVIG). Although there are some scoring systems trying to predict, it is not always possible to forsee the IVIG resistance and coronary artery involvement.

**Objectives:** The aim of this study is to assess the success of the Kobayashi scores and other parameters in predicting IVIG resistance and coronary artery involvement in Turkish population.

**Methods:** Patients who were diagnosed in the acute phase of Kawasaki disease at Hacettepe University Faculty of Medicine (Ankara,Turkey) between June 2007- January 2016 were retrospectively and January 2016- February 2018 were prospectively reviewed and included in this study. Clinical findings, laboratory parameters, echocardiographic findings, response to IVIG treatment, Kobayashi, Egami, Formosa, Harada scores were evaluated. The diagnosis of Kawasaki disease was made using the criteria of the American Heart Association.

**Results:** A total of 100 patients with Kawasaki disease were included in this study. Of the patients with KD, 61 (61 %) were male and 39 (39%) were female. The mean age at diagnosis of Kawasaki disease was 48.9 ± 34.9 months, with an age range of 4–164 months. All of the patients had fever while, 77 (77%) of them had conjunctival congestion, 74 (74%) of them had mucosal, 72 (72%) of them had rash, 69 (69%) of them had lymphadenopathy and 68 (68%) of them had extremity changes. Fifty two (52%) patients had complete KD and 48 (48%) patients had incomplete KD.

Patients younger than 1 year (p<0,001) were found to be associated with a significantly higher risk for coronary artery involvement. No significant differences were found between the IVIG-resistant and IVIG-responsive groups in terms of age (p:0.215).

Significant differences were found between the coronary artery lesions (CAL) and non-CAL groups in terms of age, white blood cell and erythrocyte sedimentation rate.

Compared with the IVIG-responsive group, the IVIG-resistant group had higher C-reactive protein (CRP) and gamma glutamyl transferase **(**GGT) levels.

Of the 100 patients who were administered IVIG, 85 (85%) responded to IVIG treatment, whereas 15 (15%) were IVIG resistant. Coronary artery involvement was detected in echocardiography at diagnosis in 38 patients. Twenty patients had Kobayashi high-risk scores, 9 patients had Egami high risk scores. The Formosa score assigned 45 patients as high-risk. The Harada score assigned 54 patients as high-risk. Only the Egami score predicted IVIG resistance in the Turkish population, none predicted coronary artery involvement.

**Conclusion:** Studies from different countries revealed that the risk scores were incapable of defining the IVIG resistance in KD. This may be atributed to ethnic differences**.** The Kobayashi score, Harada score, Formosa score in Turkish population did not predict the IVIG resistance and coronary artery involvement, but high white blood cell count, high erythrocyte sedimentation rate and age ≤1 were associated with coronary artery involvement; high CRP, high GGT, and Egami score are associated with IVIG Resistance in Turkish population.

**Disclosure of Interest**

None Declared

### P039 EOSINOPHILIC GRANULOMATOSIS WITH POLYANGIITIS-A CLINICAL CASE

#### Ana Barbosa Rodrigues^1^, Filipa Oliveira Ramos^2,3^, Patrícia Costa Reis^1,3^

##### ^1^Pediatrics Department, Hospital de Santa Maria, Centro Académico de Medicina de Lisboa; ^2^Pediatric Rheumatology Unit, Rheumatology Department, Centro Académico de Medicina de Lisboa Hospital de Santa Maria; ^3^Unidade de Investigação em Reumatologia, Instituto de Medicina Molecular, Faculdade de Medicina, Universidade de Lisboa, Centro Académico de Medicina de Lisboa, Lisbon, Portugal

###### **Correspondence:** Ana Barbosa Rodrigues

**Introduction:** Eosinophilic granulomatosis with polyangiitis (EGPA) is a rare small and medium-sized-vessel vasculitis characterized by the presence of vasculitis manifestations and eosinophilia in a previously asthmatic patient.

**Objectives:** Increase awareness for the diagnosis of EGPA and discuss possible treatment strategies.

**Methods:** Description of a patient with EGPA.

**Results:** 13-year-old male with allergic rhinitis, asthma and history of dust mite allergy, under therapy with montelukast and inhaled and nasal corticosteroid, with multiple episodes of wheezing and shortness of breath in the last year. The patient presented to the emergency department after seven days of arthralgias, myalgias and asthenia and two days of fever. On physical exam a diffuse petechial rash with hemorrhagic suffusions was detected. Blood tests revealed hemoglobin 15.9g/dL, leukocytes 34990/μL, neutrophils 27.4%, eosinophils 63.2%(23160/μL), platelets 202000/μL, C-reactive protein (CRP) 10.56mg/dL, ALT 129UI/L, AST 92UI/L, LDH 529UI/L and CK 515UI/L. Ceftriaxone and doxycycline were started and the patient was admitted to the hospital. Blood cultures were negative, as well as the *aspergillus galactomannan*antigen and serologies for *Rickettsia conori, erlichia, brucella, leptospira, cytomegalovirus, Ebstein Barr virus, parvovirus, adenovirus, cocsakie and echovírus*.  Within 72 hours a deterioration of the clinical status was observed. The patient had dyspnea, chest pain, signs of respiratory distress, hypoxemia, tachycardia, hipotension and olyguria. It was detected leukocytosis 48390/μL, eosinophilia 30000/μL and elevated CRP 16.5mg/dL, CK 962 mg/dL, troponin 1686ng/L and NT-proNBP 14800pg/mL. Tryptase was normal (3.9mcg/L). Bilateral pulmonary insterstitial infiltrates with bilateral pleural effusion were identifed on chest x-ray and ECG and echocardiogram were suggestive of myopericarditis. The patient was admitted to the pediatric intensive care unit. Antibiotherapy was switched to vancomicin plus meropenem and anti-inflammatory drugs, furosemide, espironolactone and immunoglobulin were started. Due to hemodinamic instability, mechanic ventilation and  aminergic support were needed. Further exams were performed: bronchoalveolar lavage was negative for bacteria and fungi; myelogram showed hypercellular medulla with increased non-clonal eosinophils (41%); skin biopsy revelead leukocytoclastic vasculitis with eosinophils and direct immunofluorescence compatible with vasculitis. Anti-myeloperoxidase (MPO) antineutrophil cytoplasm antibodies (ANCA) were negative. The genetic analysis for hypereosinophilia syndrome mutations was negative. The pediatric rheumatology team was called to observe the patient and, according to the clinical presentation and laboratory results, the diagnosis of EGPA was made. The patient was started on methylprednisolone i.v. 1 g per day for three days. A fast clinical, laboratory and radiological improvement occurred, including complete regression of eosinophilia. Currently, more than six months after this episode, the patient is followed at the pediatric rheumatology outpatient clinic and there are no signs of disease activity under therapy with azathioprine (2mg/kg) and prednisolone in progressively lower doses.

**Conclusion:** We present a patient with EGPA, fulfilling 5 of 6 criteria (asthma, eosinophilia exceeding 10% of the total white blood cell count, history of allergy, pulmonary infiltrates, extravascular eosinophils), associated with musculoskeletal and cardiac abnormalities and negative anti-MPO ANCA. In conclusion, EGPA is a potentially life-threatening condition, clinical suspicion and early treatment of these patients are thereby crucial.

For this case submission written informed consent was obtained from the patient and family.

**Disclosure of Interest**

None Declared

### P040 HENOCH-SCHÖNLEIN PURPURA NEPHRITIS IN CROATIAN CHILDREN: A RETROSPECTIVE STUDY IN FIVE TERTIARY CARE CENTERS

#### Mateja Batnozic Varga^1^, Nastasia Cekada^2^, Mario Sestan^2^, Sasa Srsen^3^, Lucija Ruzman^4^, Maja Zaninovic^4^, Aleksandar Ovuka^4^, Ivana Ozdanovac^1^, Marija Pecnjak^5^, Domagoj Kifer^6^, Marijan Frkovic^2^, Alenka Gagro^7^, Marija Jelusic^2^

##### ^1^Department of Paediatrics, University Hospital Centre Osijek, Osijek; ^2^Department of Paediatrics, University Hospital Centre Zagreb, University of Zagreb, School of Medicine, Zagreb; ^3^Department of Paediatrics, University Hospital Centre Split, Split; ^4^Department of Paediatrics, University Hospital Centre Rijeka, Rijeka; ^5^Department of Paediatrics, University Hospital Centre Zagreb; ^6^Faculty of Pharmacy and Biochemistry, University of Zagreb; ^7^Children's Hospital Zagreb, Zagreb, Croatia

###### **Correspondence:** Mateja Batnozic Varga

**Introduction:** Henoch-Schönlein purpura nephritis (HSPN) is the most severe complication of Henoch-Schönlein purpura (HSP) that can occur at any time of the disease process and includes isolated microscopic or macroscopic hematuria, mild or heavy proteinuria with or without nephrotic syndrome, renal failure and hypertension.

**Objectives:** To determine a possible prognostic factor for earlier HSPN onset, to explore indications for kidney biopsy according to urine analysis and the correlation between 24-h urinary protein levels and biopsy findings, as well as biopsy findings and patient outcome.

**Methods:** The cross-sectional study included all children with HSPN diagnosed by EULAR/PRES/PRINTO criteria from 2009 to 2017 at 5 tertiary care centres in Croatia.

**Results:** Out of 540 patients diagnosed with HSP, 91 children who developed HSPN (16.85%) were included. The patient population with HSPN included 52 boys (57.14%) and 39 girls (42.86%). Median (range) age of HSP diagnosis was 8 years (1.5-17.5) and from the HSP diagnosis to HSPN onset was 1.5 (0-60) months. Median (range) follow-up time was 44 (4-167) months. Nephritis was present in 18.68% of cases at the HSP onset. No statistically significant difference or correlation was found between the time of HSP diagnosis to HSPN onset and gender, age, purpura distribution, joint and gastrointestinal involvement. Kidney biopsy was done in 26 patients (28.57%). Among them, 3 patients had isolated persistent hematuria, 3 had isolated proteinuria and 20 patients had both hematuria and proteinuria. Median (range) 24-h urinary protein levels in patients who underwent biopsy was 1.28 (0-7.46) g/dU. The leading indication for biopsy was simultaneous hematuria and proteinuria (p<0.001), while isolated proteinuria was not a determining factor (p=0.592). Isolated hematuria was a statistically significant factor in detecting patients who were not likely to have a biopsy (p<0.001). Biopsy findings were graded with different classifications: 7 with Oxford classification, 6 with Haas, 1 with ISKDC, while 4 were classified simultaneously with Oxford and ISKDC. Only descriptive findings were available for 8 biopsy specimens. No statistically significant difference was found in 24-h urinary protein levels between different biopsy findings. 68% of HSPN patients were treated with corticosteroids out of which 17.6% patients took immunosuppressive or/and antihypertensive drugs in addition to corticosteroids. Most patients had good outcome, with normal physical exam findings and no signs of renal disease in laboratory tests (78%), or with microhematuria and proteinuria <1g/dU (17.6%), while only 4 patients (4.4%) had a more severe outcome with proteinuria >1g/dU. No significant difference was found in patient outcome for different biopsy findings (p=0.214).

**Conclusion:** Simultaneous hematuria and proteinuria was a statistically significant factor for kidney biopsy. However, while isolated proteinuria was not the sole determining factor, excessive levels of 24-h urinary proteins should be taken in consideration. Due to the small number of patients and no uniform classification generally used in grading biopsy findings, a statistically significant difference in regard to outcome could not be confirmed, indicating the need for both in future research.

**Disclosure of Interest**

None Declared

### P041 POLYARTERITIS NODOSA: OVER 20 YEARS’ EXPERIENCE.

#### Hafize E. Sönmez^1^, Ezgi D. Batu^1^, Gizem Ayan^2^, Kenan Kenan Barut^3^, Berkan Armağan^4^, Abdulsamet Erden^4^, Serdal Uğurlu^5^, Yelda Bilginer^1^, Özgür Kasapçopu^6^, Ömer Karadağ^4^, Sule A. Bilgen^4^, Huri Özdoğan^5^, Seza Özen^4^

##### ^1^Department of Peditarics, Division of Rheumatology, Hacettepe University Faculty of Medicine, Ankara; ^2^Department of Internal Medicine, Cerrahpasa Medical School, Istanbul University, İstanbul; ^3^Department of Peditarics, Division of Rheumatology, Cerrahpasa Medical School, Istanbul University; ^4^Hacettepe University Vasculitis Centre, Ankara; ^5^Division of Rheumatology, Department of Internal Medicine; ^6^Department of Peditarics, Division of Rheumatology, Cerrahpasa Medical School, Istanbul University, İstanbul, Turkey

###### **Correspondence:** Ezgi D. Batu

**Introduction:** Polyarteritis nodosa (PAN) is a necrotizing vasculitis of predominantly medium size vessels.

**Objectives:** The present study aimed to summarize the characteristics of PAN patients, and also analyse the trend of decreasing PAN frequency in the last 25 years, in Turkey.

**Methods:** Paediatric and adult PAN patients followed up in Hacettepe University and Istanbul University Cerrahpasa Faculty of Medicine between 1990 and 2015, were included. The demographics, clinical findings and outcomes were evaluated retrospectively.

**Results:** One hundred thirty-three patients, including 66 children, were enrolled in the study. The median (minimum-maximum) age at symptom onset and diagnosis were 16 (2-75) years and 17 (3-75) years, respectively. The mean follow-up duration was 13 (2-27) years. Among 133 patients, 86 (64.7%) had fever, 108 (81.2%) had skin involvement, 54 (40.6%) had renal involvement, 43 (32.3%) had neurological involvement, 32 (24.1%) had gastrointestinal involvement, 10 (7.5%) had cardiac involvement, 6 (4.5%) had pulmonary involvement. The median (minimum-maximum) leukocyte count, erythrocyte sedimentation rate and C-reactive protein levels at the time of diagnosis were 10400 (6100-32000)/mm^3^, 58 (2-132) mm/h and 5.22 (0-46) mg/dL, respectively. All patients were ANCA negative. Hepatitis serology was analyzed in 121 patients and found positive in 13 of them. *MEFV* mutations were screened among 65 patients, 24 of them had mutations in at least one allele. Biopsy was performed in 109 patients and computerized tomography (CT) angiography was performed in 92 patients. Myalgia and skin involvement were significantly more frequent in children whereas neurologic involvement was much more common among adults (Table 1). The number of PAN patients declined significantly after 2010. 9 patients were re-categorized as DADA2 after 2014 and no patient were diagnosed with FMF+PAN after 2008.

**Conclusion:** Our results suggest a decrease in PAN in our country which may be due to improved healthcare and dissecting mimicking diseases. Further prospective studies with prolonged follow-up could help us to better understand the disease characteristics.

**Trial registration identifying number:**

**Disclosure of Interest**

None Declared

**Table 1 (abstract P041).** Characteristics of paediatric and adult PAN patients

Features, n (%)	Patients (n=133)	Paediatric patients (n=66)	Adult patients (n=67)	P value
Fever	86 (64.7)	50 (75.7)	36 (52,9)	0.06
Myalgia	79 (59.4)	48 (72.7)	31 (46.2)	**0.01**
Abdominal pain	65 (48.9)	40 (60.6)	25 (37.3)	0.05
Skin involvement	108 (81.2)	63 (95.5)	45 (67.2)	**<0.001**
GI^a^ involvement	32 (24.1)	17 (25.7)	15 (22.3)	0.64
Renal involvement	54 (40.6)	22 (33.3)	32 (47.7)	0.09
Cardiac involvement	10 (7.5)	8 (12)	2 (2.9)	0.04
Neurologic involvement	43 (32.3)	14 (21.2)	29 (43.2)	**0.007**
Exitus	6 (4.5)	0 (0)	6 (8.9)	0.007

^a^GI, gastrointestinal

### P042 GENERATION OF ADA2 GENETIC KNOCKOUT IN A MYELOID CELL LINE USING CRISPR/CAS9 GENOME EDITING: AN IN VITRO CELL LINE MODEL TO STUDY GENE THERAPY FOR DADA2.

#### Marina S. Casimir, Ying Hong, Paul Brogan, Despina Eleftheriou

##### Infection, Inflammation and Rheumatology, UCL Great Ormond Street Institute of Child Health, London, UK

###### **Correspondence:** Marina S. Casimir

**Introduction:** Deficiency of adenosine deaminase type 2 (DADA2) is an autosomal recessive genetic disease causing systemic inflammation and vasculitis resembling polyarteritis nodosa, caused by loss of function mutations in the *ADA2* gene. Treatment of DADA2 with biologic agents targeting TNF-alpha is particularly effective, but has side effects, is expensive and is required to be given by injection for life. We are curently exploring an alternative treatment strategy for DADA2 using gene therapy. Since there is no rodent orthologue of *ADA2* an important first objective for this programme of work was to develop a cell line model to explore if it is possible to correct in vitro the defects associated with DADA2 using gene transfer strategies.

**Objectives:** To develop an *ADA2* knockout (KO) monocyte cell line (THP-1) derived using Clustered Regularly-Interspersed Small Palindromic Repeats (CRISPR)/CAS9 and to confirm that this KO has comparable immunophenotype to macrophages from DADA2 patients as indicated by reduced ADA2 expression and M1/M2 skewing and pro-inflammatory cytokine production.

**Methods:** A plasmid delivered CRISPR/CAS9 system was used to generate the *ADA2* KO THP-1 cell line. ADA2 enzyme activity was assessed using a modified commercially available assay and ADA2 protein expression using immunoblotting. THP-1 cells were treated with PMA to induce differentiation into macrophages before polarisation into M1 through LPS/INF-γ stimulation; and M2 polarisation through IL-4, IL-10 and Il-13 stimulation. Immunophenotyping was assessed using qPCR.

**Results:** We first confirmed effective knockout of *ADA2* at both genetic sequencing and protein expression level. We further established reduction of ADA2 enzyme activity by almost 50% in culture supernatants when compared to scramble control THP-1 cells (p<0.01). There was significant upregulated gene expression for several proinflammatory molecules: TNF-α (p<0.001), CXCL-10 (p<0.001), STAT-1 (p<0.01), CCL2 (p=0.05) and IL-1β (p<0.001) in M1 polarised *ADA2* KO THP-1 cells when compared to control THP-1 cells. M2 polarisation of *ADA2* KO THP-1 cells did not affect expression of CD200R and CD206 compared to control cells.

**Conclusion:** We have successfully generated an *ADA2* genetic KO myeloid cell line using the CRISPR/Cas9 system. We confirmed that *ADA2* KO THP-1 cells have a comparable immunophenotype to macrophages from DADA2 patients and exhibit reduced ADA2 enzyme activity. This *in vitro* cell line model can now be used to examine the possibility of reversing the defects associated with DADA2 using gene editing strategies.

**Disclosure of Interest**

None Declared

### P043 KAWASAKI DISEASE IN INTENSIVE CARE UNIT: ARE COMPLETE AND INCOMPLETE FORMS DIFFERENT?

#### Helene Yager^1^, Fleur Le Bourgeois^2^, Blandine Vanel^3^, Isabelle Kone-Paut^1^, Maryam Piram^1^, Bilade Cherqaoui^1^

##### ^1^Pediatric Rheumatology, CHU Bicetre - APHP, CEREMAIA, LE KREMLIN BICETRE; ^2^Pediatric Intensive care unit, CHU Robert Debré - APHP, Paris; ^3^Pediatric Intensive care unit, HFME - HCL, Lyon, France

###### **Correspondence:** Bilade Cherqaoui

**Introduction:** Kawasaki disease (KD) is an acute vasculitis occurring mainly in children under 5 years of age. The central issue is the prevention of cardiac damage that may occur early and be life-threatening, requiring admission in intensive care unit (ICU). At admission in ICU, clinical features of KD may be incomplete or atypical; especially difficult to distinguish from a severe sepsis. In this context, the criteria defined by the American Heart Association (AHA) are not always conclusive to guide the management in acute phase, including initiation of appropriate treatment, such as intravenous immunoglobulin (IVIG).

**Objectives:** The aim was to compare phenotype and evolution of KD hospitalized in ICU (KD-ICU) with complete AHA criteria (cKD-ICU) or incomplete criteria (iKD-ICU).

**Methods:** Complete criteria of KD were defined as: ≥5 days of fever and ≥4 main clinical items. Incomplete criteria were defined as: ≥5 days of fever and ≥2 main clinical items (or ≥7 days of unexplained fever) and inflammatory syndrome with either ≥3 evocative biological abnormalities or coronary dilatation. All patients had to be hospitalized in ICU. We collected retrospectively KD-ICU cases between 2001 and 2018, from a national appeal of French medical societies (*SOFREMIP, GRFUP*). Data regarding clinical, biological, ultrasound and therapeutic features were recorded. Statistics were performed with Fischer test (PRISM 5.0); averages were compared with significance defined as p <0.05 (MedCalc).

**Results:** 25 patients met the criteria of cKD-ICU, 23 patients met the criteria of iKD-ICU. Patients were hospitalized in ICU for: shock (37.5%), unstable hemodynamics (16.7%), acute neurological disorder (10.4%), myocarditis (8.3%), large coronary artery aneurysm (6.2%); ICU hospitalization duration was 7.8 ± 6.2 days, intubation rate of 45.8%, amines / cardiotropes of 45.8%, vascular filling 33.3% (all similar in both groups). 2 patients died of myocardial infarction. Differential diagnoses initially suggested were: unexplained fever / poorly tolerated bacteremia (45.8%), peritonitis / severe colitis (25%), toxin shock (12.5%); the diagnostic delay was 4.4 ± 1 days (similar in both groups). No significant differences were found concerning age at baseline (3.6 ± 0.6 vs. 2.1 ± 0.5 years old), sex ratio (0.9 vs 0.5 M / F, p> 0.05) respectively in cKD-ICU and iKD-ICU patients; total duration of fever, initial biology or extreme values ​​(hemoglobin, platelets, leucocytes, CRP, ASAT, ALAT, natremia) were also similar in both groups. iKD-ICU patients were more frequently hospitalized in ICU before onset of fever (43.4 vs 8%, p = 0.01). The delays between onset of signs or hospitalization in ICU and the first course of IGIV were similar in both groups. Intravenous corticosteroid therapy was used in 36% of cKD-ICU vs 20% of iKD-ICU patients (p = 0.2); a second course of IGIV was used in 52% of cKD-ICU vs 47% of iKD-ICU patients (p = 0.7). All patients received a broad-spectrum antibiotic. Other therapeutics and evolution features were not significantly different between 2 groups.

**Conclusion:** KD-ICU patients had no initial or evolutive differences whether they met complete or incomplete AHA criteria. The strong suspicion of KD is therefore enough to initiate specific therapeutics even in severe complicated forms. An upcoming study will precise the phenotype of patients with KD-ICU to target them earlier, comparing them to KD patients who did not need ICU stay, via the KAWANET French database.

**Disclosure of Interest**

None Declared

### P044 PULMONARY RENAL SYNDROME IN HENOCH SCHONLEIN PURPURA: A RARE BUT SERIOUS COMPLICATION.

#### Katherine Clarke^1^, Elena Kurteva^2^, Neil Sebire^3^, Muthana Al Obaidi^1^

##### ^1^Great Ormond Street Hospital, Department of Paediatric Rheumatology; ^2^Great Ormond Street Hospital, Department of Paediatric Gastroenterology; ^3^Great Ormond Street Hospital, Department of Paediatric Histopathology, London, UK

###### **Correspondence:** Katherine Clarke

**Introduction:** We present the case of a five year old male presenting with fever, abdominal pain and rectal bleeding. He had a history of rash and arthralgia, diagnosed as Henoch Schonlein Purpura (HSP).

**Objectives:** To illustrate the life threatening complications that can infrequently occus with HSP

**Methods:** He had a widespread purpuric rash with necrotic lesions and swollen extremities (figure 1). He was anaemic. Urine dipstick detected proteinuria and erythrocytes. Renal biopsy showed proliferative glomerulonephritis with strong deposition of IgA (figure 2). Anti-neutrophil antibodies, Anti- nuclear antibodies and double stranded DNA were negative with normal complement. IV methyl-prednisolone was commenced. He continued to deteriorate with worsening renal function and respiratory deterioration. Chest X Ray showed bilateral focal abnormalities (figure 3). During intubation, he bled secondary to pulmonary haemorrhage. He required high frequency oscillation ventilation.

**Results:** He received cyclophosphamide and plasmapheresis followed by prednisolone and enalapril. He made slow improvement. He continued to have heavy proteinuria and haematuria but his renal function gradually improved. He made good recovery. Within two weeks he was mobile, off oxygen, with no cutaneous scars and no gastrointestinal symptoms.

**Conclusion:** Henoch-Schönlein purpura (HSP) is the most common type of IgA vasculitis in childhood. It usually runs a benign and self-limiting course [1]. Pulmonary-Renal involvement is a rare but life-threatening complication requiring early recognition and aggressive treatment [2]. In the deteriorating child with HSP, one must be aware of this serious complication.

(1) Petty R, Laxer RM, Lindsley CB, Wedderburn LR (eds): Textbook of Pediatric Rheumatology, ed 7. Philadelphia, Elsevier, 2016.

(2) Aeschlimann FA, Yeung RSM, Laxer RM, et al. A toddler presenting with pulmonary renal syndrome. Case Rep Nephrol 2017; 7:73-80

**Disclosure of Interest**

None Declared

### P045 ANAKINRA AND ABCIXIMAB. SUCCESSFUL MANAGEMENT OF AN INFANT WITH REFRACTARY KAWASAKI DISEASE

#### María Isabel García Ruiz-Santa Quiteria^1^, Marisol Camacho Lovillo^1^, María José Lirola Cruz^2^, Esther Perez Borrego^1^, Israel Valverde Pérez^1^, Ana Mendez Santos^1^, Peter Olbrich^1^, Laura Fernández Silveira^1^

##### ^1^Hospital Universitario Virgen del Rocío; ^2^Instituto Hispalense de Pediatría, Sevilla, Spain

###### **Correspondence:** María Isabel García Ruiz-Santa Quiteria

**Introduction:** Kawasaki Disease (KD) is an acute and self-limited vasculitis, potentially leading to severe complications such as coronary artery aneurysms (CA). Patients with giant aneurysms (z score> 10 or > 8 mm) are at high risk of complications such as thrombosis and stenosis. Young infants and those with refractory KD have a high risk of developing CA. These patients would likely benefit from an early and aggressive treatment, although the most appropriate treatment strategy remains unknown. To date 3 cases of pediatric patients with giant aneurysms have been published showing a good response to anti IL-1 (anakinra). In addition, 3 clinical trials with antiIL-1 are currently ongoing. Abciximab (monoclonal antibody against the GPIIb / IIIa glycoprotein receptor located on the surface of platelets) has been used in cases with giant aneurysms and preliminary data suggests a positive effect on the vascular remodeling.

**Objectives:** We highlight the favorable medium term outcome and suggest the potential benefit of anti-Il-1 therapy and abciximab in this clinical setting.

**Methods:** We report the case of KD with a severe clinical course and the development of multiple, large CA despite an early and aggressive treatment.

**Results:** A 6 months-old male Asian infant was admitted with high fever for 4 days, submandibular adenopathy, conjunctival injection, red lips and macular rash. He was initially treated as a bacterial infection but lack of clinical improvement despite empirical intravenous antibiotic therapy and the development of clinical symptoms suggestive of KD lead to the performance of a trans-thoracic echocardiogram revealing a dilatation of the left coronary artery and an aneurysm of the anterior descending coronary artery. Treatment with intravenous immunoglobulins (IVIG) 2 mg/kg and ASA 50 mg/kg/d was initiated (day 6). Due to the persistence of fever and raised acute phase reactants a second IVIG dose was administered at day 8. Given the severity of symptoms, laboratory tests (49160/mcl leukocytes, PCR 295 mg/l, proBNP 1261pg/ml), the patients’ age and the echocardiographic findings, corticosteroids (30mg/kg/dose) were prescribed (day 8, 9 and 10) without clinical improvement and the patient was therefore transferred to our center.

Because of the refractory clinical picture and the remarkable coronary findings(multiple CA, the highest in right coronary artery:14mm, z-score: 39.89) anakinra(4mg/kg/day) was started on day 11. The dose was increased to 8mg/kg/day, and abciximab(initial dose of 2 mg/kg/day followed by a 12 hour intravenous infusion of 1.6 mg with ICU monitoring) was added to the treatment plan on day 14 with resolution of the clinical symptoms and normalization of the laboratory parameters (except trombocytosis 1295000/mm3). He was discharged 1 month after the onset of symptoms and managed as an outpatient with anakinra (8mg/kg/day), ASA, heparin and tapered corticosteroids. At discharged the ecocardiogram showed a significant dilation of the coronary aneurysms, with 2 giant aneurysms of 9.7 mm, z-score 26.17 and 12 mm, z-score 33.59 in the right coronary artery. Anakinra was stopped one month after discharge. Subsequent echocardiographic controls (seven months later), showed a significant improvement (largest aneurysm 2.5 mm, z-score: 2.93). Current treatment is limited to AAS and heparin.

**Conclusion:** Despite adequate initial KD management, there remains a group of patients which develop serious cardiac complications. The identification of these high risk patients and the establishmentof guidelines forrisk-adjusted therapy are needed in order to optimize the management and subsequently the outcome of these patients. Informed consent had been obtained from the parent.

**Disclosure of Interest**

None Declared

### P046 ARTHRITIS IN THE SUBACUTE PHASE OF KAWASAKI DISEASE

#### Seira Hattori, Ayako Murase, Ai Ohnishi, Kenichi Nishimura, Ryoki Hara, Shuichi Ito

##### Pediatrics, Yokohama City University graduate school of medicine, Yokohama, Japan

###### **Correspondence:** Seira Hattori

**Introduction:** Kawasaki disease (KD) is a systemic vasculitis of medium-sized arteries in young children. KD has various complications, including arthritis, which is a common complication. Arthritis in the febrile acute phase of KD is likely to resolve following improvement of systemic symptoms. However, some patients have persistent or new-onset arthritis in the subacute phase of KD. There have been few studies of arthritis in the subacute phase of KD.

**Objectives:** This study aimed to evaluate the clinical characteristics, laboratory findings, imaging findings, treatment, and prognosis of arthritis in the subacute phase of KD.

**Methods:** We assessed patients with arthritis in the subacute phase of KD in our institute, a tertiary medical facility in Yokohama city, from April 2008 to January 2018. We retrospectively examined the treatment for KD, coronary artery lesions (CALs), affected joints, C-reactive protein (CRP) levels, matrix metalloproteinase-3 (MMP-3) levels, imaging findings, treatment for arthritis, and outcome of arthritis using the patients’ electronic medical records.

**Results:** Fifteen patients were enrolled. The median age of patients was 46 months (range, 10–116 months) and most were boys (60%). Five (33%) patients had arthritis in the acute phase of KD, and the arthritis resolved once with acute treatment of KD, except for one patient. Four (27%) patients were treated with single administration of intravenous immunoglobulin (IVIG) and 11 (73%) required additional IVIG. Infliximab (IFX) was administrated to seven (47%) patients and plasma exchange was performed in one because of IVIG resistance. One patient developed CALs. Arthritis was detected most in the knees (67%), followed by the ankles (47%) and hip joints (40%). Six (40%) patients had a consistent fever. At diagnosis of arthritis, CRP levels increased again in 14 (97%) patients and MMP-3 levels were elevated in 14 (97%) patients. Ultrasound showed joint fluid in all patients, but none showed synovial proliferation accompanied by a positive power Doppler signal. Nonsteroidal anti-inflammatory drugs, steroids, and methotrexate were administered for arthritis in 13 (87%), 11 (73%), and one (6.7%) patient, respectively. Among these treatments, steroids promptly improved arthritis without developing CALs. The median administration period of steroids was 46 days (range, 12–249 days). The arthritis resolved without sequelae in all of the patients, and they successfully discontinued treatment.

**Conclusion:** Arthritis in the subacute phase of KD is relatively common, and the arthritis does not cause destruction of joints and cartilage. However, some patients suffer from persistent arthritis for several weeks. For persistent arthritis, short-term steroid therapy can help to ensure rapid improvement without a new complication of CALs. The pathogenesis of arthritis in the subacute phase of KD remains unclear, but arthritis occurs, despite administration of IFX. Therefore, this condition appears to be unrelated to tumor necrosis factor-α.

**Disclosure of Interest**

None Declared

### P047 THE FIRST REPORT OF POST PENTAVALENT VACCINATION ACUTE HEMORRHAGIC EDEMA OF INFANCY

#### Shabnam Hajiani, Mohsen Jari, Reza Shiari

##### Pediatrics rheumatology, Shahid Beheshti university of medical sciences, Tehran, Iran, Islamic Republic Of

###### **Correspondence:** Mohsen Jari

**Introduction:** Background:Acute hemorrhagic edema of infancy (AHEI) is an immune complex-mediated leukocytoclastic vasculitis and rare disease that age of onset for it usually is 4-24 months. Clinical findings such as low grade fever,edema of face, upper and lower limbs and generalized petechiae,purpura or ecchymosis develop rapidly over 24-48 hours. Upper respiratory tract infection, gastroenteritis, or vaccination have been reported to cause of this disease. AHEI was reported after heptavalent vaccination ( Hemophilus influenza type B, diphtheria, tetanus, acellular pertussis, hepatitis B, polio, and conjugate pneumococcal vaccines).To our knowledge there is^’^nt any report of post pentavalent vaccination ( Hemophilus influenza type B, diphtheria, tetanus, acellular pertussis and hepatitis B)AHEI. asystemic involvement such as arthritis/arthralgia,abdominal pain,intussusception and scrotal pain and testicular torsion are rare.Diagnosis of AHEI is clinical and routine laboratory tests are nondiagnostic, although leukocytosis and thrombocytosis ,elevation of erythrocyte sedimentation rates((ESR), c-reactive protein(CRP) and liver function test may be occured. No effective therapy need for AHEI. The use ofantihistamines and steroids has been controversial . systemic corticosteroids may be used to sever conditions or systemic involvement such as abdominal pain,intussusception ,nephritis ,scrotal pain and testicular torsion. Monitor patients for rare systemic involvement such as renal involvementhas been controversial.

**Objectives:** Case report:Our patient was a 6-month boy injected by pentavalent vaccine 5days ago.since 3 days ago he had low grade fever,edema of face, upper and lower limbs and generalized petechiae,purpura and ecchymosis. His condition was good.Mild scrotal swelling was seen. Other review of system and physical examinations were normal. Labratoary findings showed only high ESR,CRP.Abdominal-pelvic and testicular ultrasonographies were normal. We treated this patient with supportive treatment and after ruled out of infectious diseases single dose 10mg/kg methlprednisolon infusion. There were no complications in follow up. Informed consent for publication had been obtained.

**Methods:** case report

**Results:** case report

**Conclusion:** Conclusion: Pentavalent vaccination can induced AHEI.

**Disclosure of Interest**

None Declared

### P048 TAKAYASU ARTERITIS INITIALLY DIAGNOSED WITH FEVER OF UNKNOWN ORIGIN

#### Özlem Üzüm^1^, Ali Kanık^2^, Kader Vardı^1^, Yeliz Pekçevik^3^, Kayı Eliaçık^1^, Belde Kasap Demir^4^

##### ^1^Departments of Pediatrics, Tepecik Training and Research Hospital; ^2^Departments of Pediatrics, İzmir Katip Çelebi University; ^3^Department of Radiology, Tepecik Training and Research Hospital; ^4^Pediatric Nephrology and Rheumatology, İzmir Katip Çelebi University, İzmir, Turkey

###### **Correspondence:** Belde Kasap Demir

**Introduction:** Fever of unknown origin (FUO) in children is defined as fever >38.3°C (101°F) at least once per day for ≥8 days with no apparent diagnosis after initial outpatient or hospital evaluation that includes a detailed history, thorough physical examination, and initial laboratory assessment. The most common causes are infectious diseases, rheumatologic diseases and malignant diseases. This case is presented to emphasize that Takayasu Arteritis should be considered in patients examined for FUO.

**Objectives:** An 11-year-old girl presented with a fever increasing up to 38.3°C 1-2 times a day for one month, and up to 39°C for the last five days, despite continued antibiotic therapy. It has been learnt that she had a pain in the upper abdominal region for a week and she had lost 10 kilograms in the last two months after using a teeth retainer. On physical examination, body weight was under 3th percentile, while height was at 25-50 percentile. Vitals were normal except a fever reaching up to 38.5°C. There was no finding in systemic examination except general sensitivity in the abdomen. Hemoglobin was 8.1 g/dL. Biochemical values, immunoglobulin levels, and complements were normal. C-reactive protein (14.9 mg/dL) and erythrocyte sedimentation rate (140 mm/h) were high. Rheumatoid factor and viral serology were negative. No microorganism was yielded in the blood, throat or urine cultures. There was no sign of active tuberculosis on chest X-ray and PPD evaluation. Anti-nuclear antibody, anti-dsDNA, and p/c-anti-cytoplasmic antibodies were negative. Ophthalmologic evaluation, echocardiography and abdominal USG were normal. No additional features were found in peripheral smear and bone marrow examination.

**Methods:** Abdominal Doppler USG was performed upon ongoing fever, abdominal pain and high levels of acute phase reactants. The patient was diagnosed with Takayasu Arteritis due to the wall thickening in the first 4-5 cm proximal segment of the anterior mesenteric artery and severe thickening in the left carotid artery wall.

**Results:** She was treated with steroids and azatioprin. Abdominal pain regressed and acute phase reactants decreased to normal limits at the end of the second week.

**Conclusion:** Although Takayasu arteritis is included in the etiology of FUO, diagnosing a patient may take time in the absence of common findings including pulselessness or claudication. Takayasu Arteritis should be kept in mind in patients with FUO and further investigation should be considered. Written consent for publication was obtained from the parents.

**Disclosure of Interest**

None Declared

### P049 GENDER DIFFERENCES IN RISK FACTORS (OF IVIG-RESISTANT) IN PATIENTS WITH KAWASAKI DISEASE

#### Young Dae Kim^1^, Jong Gyun Ahn^2^, Kwang Nam Kim^3^

##### ^1^Department of Pediatrics, Inje University Ilsan Paik Hospital, Goyang; ^2^Department of Pediatrics, Severance Children’s Hospital, Yonsei University College of Medicine, Seoul; ^3^Department of Pediatrics, Sacred Heart Hospital, Hallym University College of Medicine, Anyang, Korea, Republic Of

###### **Correspondence:** Young Dae Kim

**Introduction:** Kawasaki disease (KD) is one of the most common vasculitis that may cause coronary artery complications. KD is more common in boys than in girls with a male-to-female ratio of 1.36:1 to 1.62:1. Thus, it has been suggested that the susceptibility is closely associated with sex differences.

**Objectives:** This study investigated the gender differences in clinical and laboratory findings in patients with KD.

**Methods:** We reviewed the medical records of 148 patients (78 boys and 70 girls) with KD who had been treated at our hospital between September 2012 and March 2017.

**Results:** The incidence of coronary artery aneurysm and intravenous immunoglobulin (IVIG) -resistant KD was significantly higher in the boys as compared with the girls (*P*=0.02). But there were no significant differences in WBC, Hb, CRP, ESR and urine abnormalities, such as proteinuria, pyuria and microscopic hematuria, between the two groups (*P*>0.05). There were significant differences in % neutrophil counts (>80), CRP (>8mg/dL), AST (>100IU/L), ALT (>80IU/L), hyponatremia (<134mmol/L), total bilirubin (<0.9mg/dL) and illness at initial treatment (≤4 days) between IVIG-responsive and IVIG-resistant groups (*P*<0.05).

**Conclusion:** Coronary artery aneurysm and intravenous immunoglobulin (IVIG) -resistant KD was more common in boys and there were several risk factors, such as % neutrophil counts, CRP, illness at initial treatment to progress IVIG resistant KD.

**Disclosure of Interest**

None Declared

### P050 EVALUATION OF THE ROLE OF SIGNAL PEPTIDE-CUB-EGF DOMAIN-CONTAINING PROTEIN (SCUBE)1 AND SCUBE2 IN IMMÜNOGLOBULIN A VASCULITIS

#### Gülbahar Kurt^1^, Ferhat Demir^2^, Suleyman C. Karahan^3^, Mukaddes Kalyoncu^2^

##### ^1^Department of Pediatrics; ^2^Department of Pediatric Rheumatology; ^3^Department of Biochemistry, Karadeniz Technical University, Faculty of Medicine, Trabzon, Turkey

###### **Correspondence:** Gülbahar Kurt

**Introduction:** Immünglobulin A vasculitis (IgAV) is a systemic, inflammatory, leucocytoclastic vasculitis which is caused by IgA deposit to small blood vessels. It is a self-limiting disease and etiopathogenesis of IgAV is still unclear and cytokines such as TNF-α, IL-6 have been implicated in the active phase of the disease. Signal peptide-CUB (complement C1r/C1s, Uegf, and Bmp1)-EGF (epidermal growth factor)-like domain-containing protein (SCUBE)1 is a new described, can be secreted cell surface protein. It has been proven that SCUBE1 and SCUBE 2 has a role in various pathophysiologic processes like vascular endothelial cells, inflammation, cancer metastasis and vascular diseases.

**Objectives:** We aimed to research whether there is a relationship between SCUBE1 and SCUBE2 proteins and IgAV.

**Methods:** Between January 2016 and May 2017, the patient admitting to outpatient clinics in pediatric department of Farabi Hospital in Karadeniz Technical University and diagnosed with IgAV according to Ankara 2008 diagnostic criteria were included. Patients who used steroid or non-steroidal inflammatuary drug in last three months were excluded. Blood samples collected from patients who were in active and recovery phases of their diseases and healhty control group. IL-1, IL-6, TNF-α, SCUBE1 and SCUBE2 levels studied by ELISA method.

**Results:** A total of 26 patients, 13 (50%) male and 13 (50%) female, aged between 3 and 17 years, who were diagnosed with IgAV, were included in the study. The average age in healthy control group was 7.15±3.34 years. There was skin involvement in all of the patients who were diagnosed with IgAV. Twenty three (88.4%) patients had joint involvement, seven (26.9%) patients GI involvement, five (19.2%) patients kidney involment and three (11.5%) patients testicular involvement. IL-1 levels are significantly higher in active phase of disease than in healthy control group and recovery phase of disease (p=0.03). IL1, IL-6 levels were statistically significant higher then in healthy control group (p=0.006 and p=0.01, respectively). SCUBE1 and SCUBE2 levels were not different in active and VIII recovery phases of the disease. SCUBE2 levels have found significantly higher in recovery phases of the disease when compared to healthy control group (p=0,008).

**Conclusion:** Increase of SCUBE2 levels in the recovery phase of disease suggests that SCUBE proteins has a different role from inflammation in tissues and various cell types. We believe that this situation can be elucidated by studies on more patients in this regard.

**Disclosure of Interest**

None Declared

### P051 TOCILIZUMAB AS TREATMENT OPTION FOR ACHIEVING SUSTAINED REMISSION IN A CHILD WITH TAKAYASU ARTERITIS

#### Dragana S. Lazarevic^1^, Jelena Vojinovic^1,2^

##### ^1^Pediatric Rheumatology, Clinic of Pediatrics, Clinical Center Nis; ^2^Pediatrics, Faculty of medicine, University of Nis, Nis, Serbia

###### **Correspondence:** Dragana S. Lazarevic

**Introduction:** Takayasu arteritis represents idiopathic large vessels vasculitis of aorta and its main branches, rarely affecting children.

**Objectives:** Diagnostic and treatment delay can lead to potentially life treating complications if neurological and cardiovascular injuries occur. Reducing inflammation and achieving sustained remission is important therapeutically goal, but not yet standardized.

**Methods:** We report a case of sixteen year old girl with refractory Takayasu arteritis to standard treatment who entered remission and could stopped steroids after inducing tocilizumab

**Results:** Sixteen year old girl was treated with antibiotics as having pneumonia due to fever and cough. After two weeks she was admitted to the pulmology department of our hospital due to prolonged fever, dizziness and weakness, but without reporting other symptoms. After excluding respiratory tract infection and tuberculosis she was transferred to the department of pediatric rheumatology and immunology. Possible infection causes were ruled out as procalcitonin, all cultures, virusology and atypical infections testing were negative. Bone marrow biopsy, detailed immunological and endocrinological laboratory testing were not proved hematological, immunodeficiency or autoimmune disease background. Due to persistently elevated inflammatory markers we suspected on autoinflammatory disease, but feritin was mildly elevated. Slit lamp examination and angiotenzin converting enzym have excluded possibile diagnosis of sarcoidosis. Abdominal ultrasound and echocardiography were normal. After the treatment with non-steroid anti-inflammatory drugs (NSAIDs) fever has gone, but elevated inflammatory markers persisted with profound feeling of fatigue, weakness, and dizziness. Physical examination has revealed less palpabile right radial pulse and higher blood pressure on the same side. F-fluorodeoxyglucose positron emission tomography (PET-CT) have confirmed diagnosis of Takayasu arteritis of thoracic and abdominal aorta. Magnetic resonance angiography (MRA) of the brain was normal. Methyl-prednisolon pulses were induced with gradually steroid tappering and metotrexate treatment, but unfortunately this could not stopped inflammation. Six months later she was steroid dependent with chronic fatigue and persistently elevated inflammatory parameters. MRA of the heart and large blood vessels have revealed evidence of still active inflammation in the thoracic aorta. Decision was made and tocilizumab was added on regular basis treatment. Four months after inducing tocilizumab she was without any symptoms with normalized inflammatory markers and steroids could be tapered and stopped.

**Conclusion:** In the cases of the fever of unknown origin we should consider Takayasu arteritis, even though it is rarely seen in childhood. Early diagnosis and treatment could prevent serious systemic complications. Although we lack treatment algorithm recommendations in children, this is the evidence that early and aggressive inducing of tocilizumab could lead to sustained remission in Takayasu artertitis in children. Informed consent to publish had been obtained.

**Disclosure of Interest**

None Declared

### P052 AUDITORY EVOKED POTENTIALS AND VISUAL EVOKED POTENTIALS: A HELPFUL TEST IN THE DIAGNOSIS AND FOLLOW UP OF KAWASAKI DISEASE

#### Maria Cristina Maggio^1^, Giuseppe Salvo^1^, Rolando Cimaz^2^, Domenico Puma^3^, Crocifissa Maria Ministeri^4^, Giovanni Corsello^1^

##### ^1^University Department Pro.Sa.M.I. “G. D’Alessandro”, University of Palermo, Palermo; ^2^NEUROFARBA Department University of Florence, and AOU Meyer, University of Florence, Florence; ^3^Paediatric Neuropsychiatry Operative Unit, Children Hospital “G. Di Cristina”; ^4^O.U.of Neurophysiopathology, ARNAS, Palermo, Palermo, Italy

###### **Correspondence:** Maria Cristina Maggio

**Introduction:** Kawasaki disease is a systemic vasculitis affecting mainly children; the most serious complications are coronary artery lesions (CAL). Nonetheless, the spectrum of complications involves all the vascular districts, such as the eyes, skin, kidneys, gallbladder, liver, central nervous system. Sensorineural hearing loss is a low diagnosed complication of KD, however, it may be permanent.

**Objectives:** Auditory evoked potentials (ABR) and visual evoked potentials (VEPs) are useful in evaluating children without auditory and/or visual symptoms but with diseases that could sub clinically involve these functions.

**Methods:** We enrolled 52 children (31 M, 21 F; age: 3 months-10 years) with KD and evaluated ABR and VEPs in the acute phase of the disease and during the follow up. We correlated the neurophysiological study with clinical, biochemical parameters, the type of KD (typical, atypical, incomplete) with cardiac involvement and time of IVIG and/or other non-conventional treatment in the acute phase of KD.

**Results:** VEPs were pathological in 6 children (4 M; 2 F) (1 patient with CAL had monoliteral alterations; 2 patients had ABR pathological as well). ABR were pathological in 36/52 patients (69%) (2 patients without CAL had monoliteral alterations). Furthermore, in those patients (31M; 21 F) there were no significant differences in age, time of the diagnosis, time of the first dose of IVIG, biochemical parameters (leukocytes, neutrophils percentage, AST, ALT, gamma-GT, albumin, Na, fibrinogen, D-Dimer) vs KD patients without alterations of ABR and VEPs.

One patient showed a persistent sensorineural auditory loss.

During the follow up, ABR and/or VEPs alterations persisted in the 80% of the patients.

Most of the patients showed alterations of the wave V and the I-V, expression of mesencephalic involvement.

**Conclusion:** We suggest evaluating ABR and VEPs in patients with KD, both in patients with precocious diagnosis and treatment either in children treated later than 10 days with IVIG.

We suggest studying also patients without CAL, in whom neurophysiological study may contribute to the complete follow up of these patients.

**Disclosure of Interest**

None Declared

## Poster Walk 3: JIA basic

### P053 ASSOCIATION OF INCREASED SUN EXPOSURE OVER THE LIFE-COURSE WITH A REDUCED RISK OF JUVENILE IDIOPATHIC ARTHRITIS

#### Justine A. Ellis^1^, Rachel Chiaroni-Clarke^1^, Jane Munro^2,3^, Angela Pezic^4^, Joanna Cobb^1^, Jonathan Akikusa^2,3^, Roger Allen^2,3^, Terence Dwyer^4,5^, Anne-Louise Ponsonby^4^

##### ^1^Genes, Environment & Complex Disease, Murdoch Children's Research Institute; ^2^Paediatric Rheumatology, Royal Children's Hospital; ^3^Arthritis & Rheumatology; ^4^Environmental & Genetic Epidemiology Research, Murdoch Children's Research Institute, Parkville, Australia; ^5^George Institute for Global Health, University of Oxford, Oxford, UK

###### **Correspondence:** Justine A. Ellis

**Introduction:** Cutaneous sun exposure is an important determinant of circulating vitamin D. Both sun exposure and vitamin D have been inversely associated with risk of autoimmune disease, including multiple sclerosis and rheumatoid arthritis. In children with juvenile idiopathic arthritis (JIA), low circulating vitamin D is reportedly common, but disease-related behavioural changes may have influenced sun exposure behaviours (reverse causation).

**Objectives:** We aimed to determine whether sun exposure across the life-course prior to diagnosis is associated with JIA.

**Methods:** Using validated questionnaires, we retrospectively measured sun exposure for 202 Caucasian JIA case-control pairs born and living in Victoria Australia and recruited to the CLARITY JIA Biobank. Cases and controls were matched for year of birth and time of recruitment. All subtypes of JIA were included in the analysis. Measures included maternal sun exposure at 12 weeks of pregnancy, and child sun exposure across the life-course pre-diagnosis. We converted sun exposure to UVR dose using location-specific (Melbourne Australia, latitude 37.5°S) UVR data. We looked for case-control sun/UVR exposure differences at various ages pre-diagnosis, and cumulatively across the pre-diagnosis life-course, using logistic regression, adjusting for potential confounders.

**Results:** Higher cumulative child pre-diagnosis UVR exposure was associated with reduced risk of JIA (e.g. total UVR dose quartile 1 vs 4, adjusted odds ratio (AOR) 0.19, 95% CI 0.04 – 0.85, p = 0.030), with a clear dose response relationship (test for trend p=0.025). UVR exposure at 12 weeks of pregnancy was similarly inversely associated with JIA (e.g. UVR quartile 1 vs 4, AOR 0.32, 95% CI 0.13 – 0.80, p = 0.014) with evidence of a dose response (test for trend p=0.031). Associations were robust to sensitivity analyses for disease duration, and for pre-diagnosis behavioural changes and knowledge of the hypothesis as collected by parent questionnaire.

**Conclusion:** Increased UVR exposure across the pre-diagnosis life-course is associated with reduced risk of JIA in our setting. This suggests lower circulating vitamin D in JIA may be causative, but prospective studies that directly measure pre-disease vitamin D in JIA are required. If confirmed, the associations point to an environmental factor amenable to intervention that may reduce risk of JIA in the population.

**Disclosure of Interest**

None Declared

### P054 AN ACTIVE PRONGF/P75NTR AXIS IN ARTHRITIS PATIENTS INFLUENCES CYTOKINE PRODUCTION IN SYNOVIAL FIBROBLASTS

#### Gaetana Minnone^1^, Luciapia Farina^1^, Marzia Soligo^2^, Luigi Manni^2^, Antonio Manzo^3^, Pierre Miossec^4^, Fabrizio De Benedetti^1^, Luisa Bracci-Laudiero^1^

##### ^1^Division of Rheumatology, Bambino Gesu’ Children Hospital; ^2^Institute of Translational Pharmacology, CNR, Rome; ^3^Division of Rheumatology and Translational Immunology Research Laboratories, IRCCS Policlinico S Matteo Foundation/University of Pavia, Pavia, Italy; ^4^Immunogenomics and Inflammation Research Unit, Edouard Herriot Hospital, Hospices Civils de Lyon and University Claude Bernard Lyon 1, Lyon, France

###### **Correspondence:** Luciapia Farina

**Introduction:** In addition to its well-known neurotrophic activity, the nerve growth factor (NGF) is involved in the immune regulation. Our previous studies showed that the immature NGF, proNGF, is the prevalent form in synovial fluids of patients with juvenile idiopathic arthritis (JIA) and rheumatoid arthritis (RA). The distinct roles of NGF, pro-NGF and their different receptors (TrkA and p75NTR) in the regulation of the inflammatory response are still unclear.

**Objectives:** To investigate if p75NTR and its specific ligand proNGF modulate the activity of synovial fibroblasts and mononuclear cells playing a role in the inflammatory response in the synovia of arthritis patients.

**Methods:** Mononuclear cells (MNC) were purified from blood and synovial fluids of JIA patients. Fibroblasts-like synoviocytes (FLS), obtained from synovial tissue of RA patients (RA FLS), were used to evaluate pro-inflammatory activity of proNGF. Skin fibroblasts (SF) from healthy donors (HD) were used as controls. TrkA, p75NTR, Sortilin, NGF, and cytokines expression were evaluated by quantitative PCR (qPCR). Specific ELISA were used to analyze NGF, proNGF and cytokine concentrations. p75NTR was inhibited using a synthetic inhibitors (LM11A-31) or by small interference RNA in order to evaluate how its inhibition can modify pro-inflammatory pathways.

**Results:** Our data showed that p75NTR expression was significantly up-regulated in MNC purified from blood and synovial fluid of JIA patients and in FLS of RA patients. TrkA expression was highest in HD cells and down-regulated in patient cells. Patient FLS but not MNC expressed high levels of NGF mRNA and high amounts of proNGF. On the contrary, low levels of mature NGF were detected in their conditioned media. Inflammatory stimuli, such as IL-1β, IL-6, LPS, TNFα, further up-regulated both p75NTR expression and proNGF production in synovial FLS. The inhibition of the binding of proNGF to its specific receptor p75NTR using synthetic inhibitor LM11A-31 in synovial FLS resulted in a marked reduction of IL-6 release induced by either IL-1β or other inflammatory stimuli.

**Conclusion:** The abnormal p75NTR expression levels and the modified p75NTR/TrkA ratio observed in JIA and RA patients might have a crucial role in the chronicity of the inflammatory response. In addition to inducing p75NTR up-regulation, inflammatory stimuli increased the release of proNGF in synovial FLS. ProNGF further enhances pro-inflammatory cytokine production, creating a vicious circle that amplify the inflammatory response. Blocking the binding of endogenous proNGF to its receptor p75NTR, using a specific p75NTR inhibitor, strongly reduces the production of inflammatory mediators and suggests the use of p75NTR inhibitors as a new therapeutic approach to chronic arthritis.

**Disclosure of Interest**

G. Minnone: None Declared, L. Farina: None Declared, M. Soligo: None Declared, L. Manni: None Declared, A. Manzo: None Declared, P. Miossec: None Declared, F. De Benedetti Grant/Research Support from: BMS, Pfizer, Abbvie, Novartis, Novimmune, Roche, SOBI, Sanofi, UBC, Consultant for: Roche, Novartis, Novimmune, SOBI, L. Bracci-Laudiero: None Declared

### P055 COMPARISON OF B CELL AND T CELL SUBSETS, CYTOKINE EXPRESSION AND SYNOVIAL PATHOLOGY IN DA AND JIA

#### Charlene Foley^1^, Achilleas Floudas^2^, Mary Canavan^2^, Monika Biniecka^2^, Emma J. MacDermott^1I^, Ronan Mullan^3^, Orla G. Killeen^1^, Ursula Fearon^2^

##### ^1^NCPR, OLCHC; ^2^TBSI; ^3^Rheumatology, Tallaght, Dublin, Ireland

###### **Correspondence:** Charlene Foley

**Introduction:** Down syndrome (DS) is a common chromosomal disorder associated with the development of a range of medical and immune abnormalities such as haematologic malignancies, increased susceptibility to infections, and a high incidence of autoimmune diseases, including Down’s Arthritis (DA). Previous work by our group suggests that the prevalence of DA is 18-21 fold greater than JIA, much higher than the previously reported DA prevalence of 8.7/1000. Children with DA most often follow a polyarticular course of disease, with erosive joint damage observed more frequently in this cohort when compared to a cohort of children with JIA. Small joint involvement is frequently observed, again in a significantly greater proportion (p<0.01) of children with DA than expected in a typical JIA cohort. These characteristics suggest that DA may be distinct from JIA, however little is known about the differences in synovial pathology or immunological regulation. Indeed no studies to date have examined synovial pathology in DA

**Objectives:**

- To examine B-cell and T-cell subsets, and cytokine profiles in children with DA and JIA

- To characterise & compare the synovial membrane immunohistochemistry in DA & JIA.

**Methods:** Multicolour flow cytometry was used to analyse the phenotype of B and T cells in peripheral blood mononuclear cells (PBMCs) from 40 children (n=10 per group; Healthy Control (HC), JIA, DS, DA). Cells were stained with the following panels; Panel 1 B cells (CD38, CD24, CD20, CD80, CD27, IgM, CD138, CD45, CD19, MHCclassII, BCMA, CD40, CD86, IgD); Panel 2 T cell cytokines analysed after 5hours PMA/Ionomycin stimulation (CD3, CD8, CD161, IFN-γ, TNF-α, IL-17a, GM-CSF). Flow cytometry data was assessed by Flowjo software analysis.

Synovial tissue obtained through ultrasound guided biopsy and analysed by immunohistochemistry for CD3, CD20, CD68, Factor VIII (DA n=3; JIA n=6). Synovial Inflammation and lining layer thickness were also scored. Analysis was performed using semiquantification scoring method.

Flow cytometry was performed on PBMC samples from 4 distinct gps; HC (50%F, age 9.2y (2.5-15.6)), JIA (91%F, age 13.2y (8.2-16.1)), DS (45%F, age 6.5y (1-11.9)) & DA (60%F, age 11.4y (3.8-17.8)). All of the children in the DA & JIA cohorts had a polyarticular RF negative pattern of disease. Synovial tissue was obtained from 3 children with DA & 6 JIA cases.

**Results:** Flow cytometry analysis revealed that children with DA have a significantly lower number of circulating CD19^+^CD20^+^ B cells when compared to children with JIA (p<0.05) & HC (p<0.001). However, children with DA have a greater proportion of memory B cells (CD27^+^) when compared to children with DS & no arthritis (p<0.05).

Analysis of cytokine production by CD8^+^/CD8^-^ T cells showed that IFN-γ & TNF-α production was greater in DA compared to both JIA (CD8+IFNγ+ p<0.001; CD8+TNFα+ p<0.01; CD8-IFNγ+ p<0.05; CD8-TNFα p<0.05) and HC (CD8+IFNγ+ p<0.05; CD8+TNFα+ p<0.05; CD8-IFNγ+ p<0.05; CD8-TNFα p<0.01).

Examination of synovial tissue demonstrated higher levels of CD3+ cells (p<0.05), Macrophages (p<0.05), CD20+ cells & FVIII in the joints of children with DA.

**Conclusion:** There are significant differences in B cell populations, cytokine production and immunohistochemical features of synovial tissue in children with DA & JIA. More work is required to verify these results. Preliminary findings may begin to help explain the differences observed in the clinical picture of children with DA & JIA.

**Disclosure of Interest**

None Declared

### P056 MICROENVIRONMENT DRIVEN RE-SHAPING OF PATHOGENIC T EFFECTOR AND REGULATORY SUBSET IN ACTIVE JUVENILE IDIOPATHIC ARTHRITIC PATIENTS

#### Jing Yao Leong^1^, Pavanish Kumar^1^, Phyllis Chen^1^, Joo Guan Yeo^1,2^, Camillus Chua^1^, Sharifah Nur Hazirah^1^, Suzan Saidin^1^, Thaschawee Arkachaisri^1,2^, Alessandro Consolaro^3^, Marco Gattorno^3^, Alberto Martini^3^, Salvatore Albani^1^

##### ^1^Translational Jing Yao Leong Institute/Duke-NUS Academic Medical Centre; ^2^KK Women’s and Children’s Hospital, SINGAPORE HEALTH SERVICES PTE LTD, SINGHEALTH, Singapore, Singapore; ^3^Second Paediatric Division, University of Genoa and G Gaslini Institute, Genova, Italy

###### **Correspondence:** Jing Yao Leong

**Introduction:** We have previously published and identified two dichotomous dysregulated populations within the Teff (CPLs) and Treg (iaTreg) compartments that are inflammatory, antigen experienced, correlating with disease activity and exhibiting similar TCR oligoclonality with synovial T cells. Their commonality in phenotype despite being in two functionally distinct compartments, signal the possibility of a common pathogenic origin during active disease manifestation.

**Objectives:** To determine the common transcriptomic drivers influencing dysregulation in both Teff and Treg compartments during active disease in JIA patients.

**Methods:** Next generation RNA sequencing was performed on sorted CPLs (CD3^+^ CD4^+^ CD14^-^ HLADR^+^ CD25/CD127 Teff gate) and iaTregs (CD3^+^ CD4^+^ CD14^-^ HLADR^+^ CD25^hi^ CD127^lo^ Treg gate) from 16 active disease JIA PBMCs, 8 age-matched healthy PBMCs, and 8 paired SFMCS. The corresponding total pool of Teff (Non-CPLs) or Treg (Non-iaTreg) was also sorted as a comparative control. Sorted cells were lysed and extracted for RNA, and cDNA conversion/amplification were then carried out using SMART-seq v4. Libraries are prepared and multiplexed using Nextera XT DNA library preparation kit, and ran on the Illumina HiSeq High output platform. RNA-Seq raw reads were mapped to human genome using STAR aligner with default options and reads were counted/summarised at gene level by feature Count programme. Differential expression analysis were performed using edgeR package, and pathway enrichments were done under R statistical environment and Reactome.

**Results:** Comparative differential gene expression (DEG) reveal strong transcriptomic convergence between CPLs and iaTregs as compared with the common pool of Teff and Treg. Phylogenetic analysis indicate the convergence has uncoupled the CPLs or the iaTregs away from their respective original compartments (Teff or Treg) into a common branch point. Restriction in TCR sequence oligoclonality in CPLs/iaTregs versus that of the common Teff/Treg pool reinforce the possibility of a common selection pressure. Pathway enrichment analysis reveal similar dysregulated pathways (IFN-g, PD1, CD28 costimulation) within T cell signalling for both CPLs and iaTregs. Furthermore, Weighted gene correlation network analysis (WGCNA) identified strongly coordinated HLA-DR gene network module and suggests its potential role as the driver of pathogenic T cell subsets away from conventional subsets. Gene set enrichment analysis (GESA) suggest that HLA-DR module genes are involved in TNFA signalling, inflammatory response, complement, and apoptosis. Global transcription factors gene regulatory network (TF-GRN) analysis identified several key regulatory molecules (FOXP3, CEBP, SPI and E2F1) driving the convergence of pathogenic CPLs and iaTreg populations. Taken together , we have shown that reveal several layers of mechanism operate to drive the convergence of CPLs and iaTregs, suggesting the possibility of common disease drivers in active JIA patients.

**Conclusion:** Overall the transcriptomic data indicate strong similarity in both pathogenic populations and underscore a potential mechanistic role of the inflammatory microenvironment in shaping two functionally dichotomic populations.

**Disclosure of Interest**

None Declared

### P057 ASSOCIATION BETWEEN THE IL2-IL21 RS6822844 LOCUS POLYMORPHIC VARIANTS AND AGE AT JUVENILE IDIOPATHIC ARTHRITIS ONSET

#### Liliia S. Nazarova^1^, Kseniia V. Danilko^1^, Tatiana V. Viktorova^1,2^, Viktor A. Malievsky^1^

##### ^1^Bashkir State Medical University; ^2^Institute of Biochemistry and Genetics, Ufa, Russian Federation

###### **Correspondence:** Viktor A. Malievsky

**Introduction:** Juvenile idiopathic arthritis (JIA) is the most common chronic rheumatic disease in pediatrics and is characterized by marked clinical heterogeneity, including the age-related features of the disease onset [1, 2].

**Objectives:** The aim of the study was to analyze the relationship between the *IL2-IL21* rs6822844 locus polymorphic variants and age at JIA onset.

**Methods:** The study included 328 patients with JIA from the Republic of Bashkortostan, Russia. Genotyping was performed by real-time PCR method and statistical processing of the results – with the Mann-Whitney U test.

**Results:** The study of the *IL2-IL21* rs6822844 single-nucleotide polymorphism showed, that in the carriers of the GG genotype (n=271), JIA developed statistically significantly earlier than in the carriers of the T allele (4.16 (2.14;8.09) vs 6.00 (3.03;8.78) years respectively, p=0.045). In contrast, patients with the GT genotype (n=54) tended to have a later onset of the disease than the other patients had (6.00 (3.01;8.78) vs 4.19 (2.19;8.09) years respectively, p=0.072). In a sex-stratified analysis, similar patterns for the GG and GT genotypes were found only for girls with JIA, being even more pronounced and in both cases reaching a statistical significance level (GG (n=174) vs GT+TT (n=42): 3.19 (1.90;6.79) vs 4.97 (3.01;9.65) years, p=0.008 and GT (n=41) vs GG+TT: 4.64 (3.01;8.25) vs 3.21 (1.90;6.99) years, p=0.013 respectively). It should be noted, however, that there was only one girl with the TT genotype in the studied JIA patients’ sample, and age at the disease onset of this girl was 9.65 years.

**Conclusion:** In this study, the association between the *IL2-IL21* rs6822844 locus polymorphic variants and age at JIA onset in girls was established.

**References**

1. Ravelli A, Martini A. Juvenile idiopathic arthritis. Lancet. 2007 Mar 3; 369 (9563): 767-78.

2. Donn R, De Leonibus C, Meyer S, Stevens A. Network analysis and juvenile idiopathic arthritis (JIA): a new horizon for the understanding of disease pathogenesis and therapeutic target identification. Pediatr Rheumatol Online J. 2016 Jul 2;14(1):40.

**Disclosure of Interest**

None Declared

### P058 MOLECULAR AND CELLULAR BIOMARKERS THAT DISCRIMINATE JUVENILE IDIOPATHIC ARTHRITIS FROM SEPTIC ARTHRITIS

#### Nadege Nziza^1,2,3^, Mailys Cren^1,3^, Aurelia Carbasse^4^, Perrine Mahe^4^, Marion Delpont^5^, Djamel Louahem^5^, Hugues Chevassus^6^, Mirna Khalil^6^, Thibault Mura^7^, Christian Jorgensen^1,3,8^, Isabelle Duroux-Richard^1,3^, Eric Jeziorski^4^, Florence Apparailly^1,3,8^, Pascale Louis-Plence^1,3^

##### ^1^National Institute of Health and Medical Research, Montpellier; ^2^Arthritis R&D, Neuilly sur Seine; ^3^University of Montpellier; ^4^Paediatric Department; ^5^Pediatric Orthopedic Surgery Unit; ^6^Centre d'Investigation Clinique, University Hospital of Montpellier; ^7^Department of Medical Information, La Colombière Hospital; ^8^Clinical department of Osteoarticular diseases and Biotherapy, University Hospital Lapeyronie, Montpellier, France

###### **Correspondence:** Nadege Nziza

**Introduction:** Juvenile idiopathic arthritis (JIA) and septic arthritis (SA) are the most frequent cause of arthritis among children under the age of 16 years. JIA is an auto-immune disorder involving articular inflammation that persists for more than 6 weeks, while SA is caused by bacterial infection in children joints. Although these diseases have different physiopathological basis and involve different treatments and prognoses, they share clinical similarities.

**Objectives:** To date, there are no markers sufficiently reliable to discriminate between these two forms of arthritis at the onset of the disease. Our goal is to respond to this clinical need by identifying diagnostic biomarkers capable of discriminating between JIA and SA. To that end, we focused on microRNAs (miRNAs) and myeloid cell subsets, both playing a major role in inflammation and autoimmune disorders.

**Methods:** We analyzed serum and synovial fluid (SF) samples from patients with inflammatory or septic arthritis using a miRNAs Whole Transcriptome Assay associated with a next-generation sequencing detection technology to measure the expression level of 2083 miRNAs in a pilot study (oligoarticular JIA (oJIA): n=5; SA: n=3) and validated using RT-qPCR in a replicative study (oJIA: n=9; SA: n=9). In parallel, we performed a phenotypic characterization of peripheral blood (PB) and SF myeloid subpopulations using a flow cytometer in order to identify cellular biomarkers (oJIA: n=9; SA: n=8).

**Results:** Principal component analysis and hierarchical clustering characterizations did not show significant differences between serum miRNAs of oJIA and SA patients. However, we observed a distinct miRNA profile signature in SF between the two diseases with 16 upregulated miRNAs and 5 down-regulated that perfectly discriminates oJIA and SA (p<0.01). In addition, phenotypic characterization revealed the existence of 5 myeloid cell subsets with distinct accumulation profile between the SF of oJIA and SA (p<0.05). A study of their activation profile indicated differences in the expression of cellular markers between the two groups.

**Conclusion:** In this study, we propose for the first time a synovial fluid-based miRNA signature as well as 5 myeloid cell subsets that discriminate between oJIA and SA and might be used as potential diagnosis markers in juvenile arthritis.

**Disclosure of Interest**

None Declared

## Translational science

### P059 EVALUATION OF PLASMA MICRORNA EXPRESSIONS IN PATIENTS WITH JUVENILE IDIOPATHIC ARTHRITIS

#### Ferhat Demir^1^, Alper H. Çebi^2^, Mukaddes Kalyoncu^1^

##### ^1^Department of Pediatric Rheumatology; ^2^Department of Medical Genetics, Karadeniz Technical University Faculty of Medicine, Trabzon, Turkey

###### **Correspondence:** Ferhat Demir

**Introduction:** Juvenile idiopathic arthritis (JIA) is the most common chronic rheumatic disease of childhood and its etiology is unknown. Microribonucleic acids (miRNAs) are small (16–24 nucleotides), non-coding RNA molecules that have roles on the regulation of gene expression at the post-transcriptional stage. It is also known that microribonucleic acid (miRNA)s play a role in immunoregulation.

**Objectives:** We aimed to evaluate plasma expression of some candidate miRNAs that associated with pathogenesis of autoimmunity.

**Methods:** Thirty-one patients diagnosed with JIA and age-sex matched 31 healthy children were enrolled for the study. Plasma levels of four candidate miRNAs (miRNA-16, miRNA-155, miRNA-204 and miRNA-451), which is known to be associated with autoimmunity were examined in all subjects. Plasma levels of miRNAs were measured with Real Time PCR in patients in active and inactive period and in healthy controls. Groups were compared with each other.

**Results:**

Plasma miRNA-155 levels were found to be increased in JIA patients compared to the healthy controls and it was statistically more significant in inactive period. We found that JİA patients had higher levels of miRNA-16 and lower levels of miRNA-204/miRNA-451 expressions compare with the control group, but there was no statistically significant difference. A statistically significant decrease in plasma levels of miRNA-204 was found in patients that were in inactive disease with only methotrexate therapy. Plasma miRNA expressions were compared in JIA subtypes and it was observed that miRNA-204 levels were higher in polyarticular JIA and miRNA-451 levels were higher in entesitis related arthritis without statistical significance.

**Conclusion:** Significant alterations in the plasma expression of miRNA-155 and miRNA-204 suggest to us that these molecules may be related to pathogenesis of JIA. More comprehensive and functional researches about the role of this molecules are needed in this regard.

**Disclosure of Interest**

None Declared

**Table 1 (abstract P059).** The mean plasma miRNA 2^-ΔCT^ level of patients in aJIA, iJIA and HC groups and comparison of it (include JIA patients that treated with only methotrexate)

	Mean 2^-ΔCT^			Fold change/*p value*		
	aJIA	iJIA	HC	aJIA-iJIA	aJIA-HC	iJIA-HC
miRNA-16	2.09	2.27	1.55	-1.08/*0.68*	1.35/*0.55*	1.46/*0.33*
miRNA-155	1.75	3.32	1.78	-1.89/***0.006***	-1.02/*0.51*	1.86/***0.02***
miRNA-204	0.13	0.04	0.15	3.25/*0.07*	-1.15/*0.52*	-3.75/***0.02***
miRNA-451	2.05	2.94	2.29	-1.43/*0.16*	-1.12/*0.65*	1.28/*0.26*

aJIA: active period of juvenile idiopathic arthritis, iJIA: inactive period of juvenile idiopathic arthritis, HC: Healthy control

### P060 RENAL AUTOIMMUNITY IN IPEX SYNDROME

#### Cécile Frachette^1^, Alain Lachaux^2^, Marc Nicolino^3^, Irene Loras-Duclaux^2^, Frederique Dijoud^4^, Nicole Fabien^5^, David Goncalves^5^, Christophe Malcus^6^, Maud Rabeyrin^7^, Olivia Gillion-Boyer^8^, Gwenaëlle Kesler-Roussey^9^, Alexandre Fabre^10^, Bertrand Roquelaure^10^, Michel Tsimaratos^11^, Joelle Terzic^12^, Anne-Laure Mathieu^13^, Sebastien Viel^13^, Justine Bacchetta^1^, Thierry Walzer^13^, Pierre Ray^14^, Alexandre Belot^1^

##### ^1^Pediatric nephrology and rheumatology; ^2^Pediatric Gastroenterology department; ^3^Pediatric endocrinology; ^4^Pathologist departement, Hôpital Femme Mère Enfant, Bron (Lyon); ^5^Immunology, CHU de Lyon, Groupe hospitalier Sud, Pierre-Bénite (Lyon); ^6^Immunology; ^7^Pathologist department, Hôpital Edouard Herriot, Lyon; ^8^Pediatric nephrology, Hôpital Necker-enfants-malades, Paris; ^9^Pediatric nephrology, CHU de Nantes, Nantes; ^10^Pediatric Gastroenterology; ^11^Pediatric nephrology, Hôpital de La Timone Enfants, Marseille; ^12^Pediatric nephrology, Hôpital de Hautepierre, Strasbourg; ^13^Innate Immunity and Autoimmune diseases, Centre international de Recherche en Infectiologie, Lyon; ^14^Genetic department, CHU Grenoble-Alpes, Grenoble, France

###### **Correspondence:** Cécile Frachette

**Introduction:** Immune dysregulation, polyendocrinopathy, enteropathy, X-linked (IPEX) syndrome is a rare monogenic primary immunodeficiency affecting T regulatory lymphocytes caused by *FOXP3* mutation and is associated with multiple autoimmune disorders including enteropathy, diabetes and atopic dermatitis. Kidney involvement is uncommon but has been already reported. Little is known regarding the impact of Tregs deficiency on renal tolerance.

**Objectives:** This study is intended to describe the renal phenotype of IPEX patients and to determine the self reactivity of patient’s sera using a protoarray assay enabling the detection of the autoimmune repertoire across 8000 peptides.

**Methods:** We retrospectively collected clinical, histopathological and immunological data from IPEX patients seen in French pediatric immunology, rheumatology or nephrology centres. We retrieved patients sera and analyse the autoimmune repertoire targeting the kidney with a combined strategy of immunofluorescence crossreactivity on rats cell lines and direct crossreactivity on 8000 peptides (Protoarray, Thermofisher).

**Results:** In the index case, immunofluorescence staining showed linear deposits along the tubular basal membrane suggesting a B-cell mediated immunopathogeny. The patient serum was tested on regular renal cells from rats and indirect immunofluorescence showed crossreaction over species suggesting the presence of autoantibodies targeting the basal membrane in the serum of the patient.

Four other pediatric patients were included in this study in order to better characterize nephropathy in IPEX syndrome. A total of three patients had tubulonephritis, and this feature was the first manifestation of the disease for two of them. One of the patients had membranous nephritis and the other one presented microangiopathy secondary to calcineurin inhibitors toxicity. All patients were treated with immunosuppressive drugs, two underwent hematopoetic stem cell transplantation and one had kidney transplantation. The results of the protoarray in the first patient elicited more than 100 antibodies with high titers. Among them, about 15 were kidney specific. Additional samples may help to detect a common tubulointerstitial autoantibody.

**Conclusion:** This study highlights genetic and clinical heterogeneity of renal lesions in IPEX patients. Autoimmune nephropathy is rare but does exist in IPEX syndrome and may appear as glomerulopathy or tubulopathy. It is noteworthy to define specific autoantibodies that may help clinician to screen IPEX patients for renal lesions.

**Disclosure of Interest**

None Declared

### P061 COMPARISON BETWEEN BIOMARKERS IN SYNOVIAL FLUID AND PLASMA IN JUVENILE IDIOPATHIC ARTHRITIS: PRELIMINARY DATA.

#### Mikhail Kostik^1^, Daria Kozlova^2,3^, Alexandr Popov^2,4^, Dmitry Vasiliev^2,3^, Vera Masalova^1^, Eugenia Isupova^1^

##### ^1^SAINT-PETERSBURG STATE PEDIATRIC MEDICAL UNIVERSITY; ^2^Ltd. "Scientific and Production Firm" ABRIS +; ^3^Sechenov Institute of Evolutionary Physiology and Biochemistry Russian Academy of Sciences; ^4^St. Petersburg State Technological Institute (technical university), Saint-Petersburg, Russian Federation

###### **Correspondence:** Mikhail Kostik

**Introduction:** Juvenile idiopathic arthritis (JIA) – is a chronic immune-mediated inflammatory joint disease in children under the 16 years. The peculiarities of JIA pathogenesis depend on JIA category and related to autoimmune or autoinflammatory mechanisms. Currently there are no unique approaches to evaluate JIA activity. The classical clinical and laboratory test, such as ESR and CRP no always correlate with local inflammation, joint erosions and so on. The finding of new biomarkers is actual, because can help more precise evaluate JIA activity, choose the appropriate treatment and better prognosis of outcomes.

**Objectives:** The aim of our study was to demonstrate possibility using of calprotectin, 14-3-3η and butirylcholinesterase (BUCHE) as a potential JIA biomarkers.

**Methods:** in pilot study were included 13 patients with JIA (4 boys and 9 girls) with enthesitis-related (n=3) and oligoarticular (n=9) JIA categories in whom calprotectin and 14-3-3η levels and activity of BUCHE in blood plasma - BP (n=12) and synovial fluid -SF (n=7) were measured. The onset age of patients was 3.9 (3.1; 8.9) years, disease duration – 3.7 (1.3; 6.6) years. Plasma and synovial fluid supernatant were used to determine the content of interleukin-6, calprotektin, protein 14-3-3η by the ELISA. Statistical analysis of data was carried out with the program Statistica 10.0. We utilized descriptive statistics (Me; IQR), Mann-Whitny and Wilcoxon tests, Pirson’s and Spearmen’s correlation analysis.

**Results:** Obtained data showed that the content of calprotectin in JIA in SF - 57.3 (47.3; 102.8) ng/ml was higher than in blood plasma – 5.8 (3.6; 15.0) ng/ml (р=0.02). It was shown that the content of 14-3-3η protein in JIA SF – 66.7 (56.4; 98.6) ng/ml is also higher than in BP- 46.4 (37.1; 53.1) ng\ml (р=0.02) that is also coincides with literature data obtained for blood plasma 14-3-3η levels in adult persons with rheumatoid arthritis (Walter P. et al., 2014). Activity of typical fraction of BUCHE was higher in BP -47.3 (39.8; 60.6) U/l than in SF - 30.2 (22.8; 39.1) U/l (р=0.027). Activity of atypical fraction of BUCHE was 14.2 (11.6; 16.7) U/l and 6.2 (5.0; 9.1) U/l in BP and SF, relatively (р=0.046), and activitiy of minor fraction of BUCHE was 0.46 (0.1; 1.1) U/l and 14.7 (12.2; 15.5) U/l in BP and SF, relatively (р=0.027). There weren’t found correlation between studied biomarkers and JIA measures, such as onset age, JIA duration, active joints count, ESR, CRP, JIA category, beside positive correlation between minor fraction of BUCHE in BP and active joints count (r=0,998; r<0,05) and negative correlation between CRP and 14-3-3η (r=-0.999; p<0.05) in SF. It was found that levels of calprotectin, 14-3-3η and minor fraction of BUCHE more effective reflect local inflammation than routine markers. We have found positive correlation between BP and SF for calprotectin (r=0.997; r<0.05) and 14-3-3η (r=0.958; r<0.05), but not for minor fraction of BUCHE.

**Conclusion:** We suppose that calprotectin and 14-3-3η in BP can be used as possible non-invasive biomarkers of JIA activity. Further investigations required.

**Disclosure of Interest**

None Declared

### P062 MOLECULAR MECHANISMS OF PATHOGENIC T CELL RESILIENCE IN HUMAN ARTHRITIS

#### Pavanish Kumar^1^, Leong Jing Yao^1^, Bhairav Paleja^1^, Joo Guan Yeo^2^, Jorg van Loosdregt^1^, Suzan Saidin^1^, Camillus Chua^1^, Thaschawee Arkachaisri^3^, Alessandro Consolaro^4^, Marco Gattorno^4^, Alberto Martini^4^, Gary W. Williams^5^, Ken D Pischel^5^, Martin Lotz^6^, Salvatore Albani^1^

##### ^1^Translational Immunology Institute, SingHealth, DukeNUS; ^2^Translational Immunology Institute,SingHealth DukeNUS; ^3^Duke-NUS Graduate Medical School and Rheumatology and Immunology Service, KK Women's and Children's Hospital, Singapore, Singapore; ^4^Second Pediatrics Division, University of Genoa and G Gaslini Institute, Genova, Italy; ^5^Scripps Clinic, La Jolla, California; ^6^Department of Molecular Medicine, The Scripps Research Institute, La Jolla, Callifornia, USA

###### **Correspondence:** Pavanish Kumar

**Introduction:** One of the key elements of immune pathogenesis of human autoimmune arthritis is the resilience of pathogenic T cells. We have previously described that CD4+ T cells in patients with arthritis have an increased level of autophagy than their healthy equivalents. Here, we sought to explore at epigenetic and transcriptional levels the concept of persisting increased autophagy as the consequence of “autophagic memory”, as one of the mechanisms conferring resilience to pathogenic T cells, in particular to a subset of CD4+ T cells (CPL: Circulating Pathogenic-like Lymphocytes), which are significantly more represented in patients with active arthritis and resistant to therapy with biologics

**Objectives:** T-cell resilience is critical to the immune pathogenesis of human autoimmune arthritis Autophagy is essential for memory T cell generation and associated with pathogenesis in rheumatoid arthritis (RA). Our aim was to delineate the role and molecular mechanism of autophagy in resilience and persistence of pathogenic T cells from autoimmune arthritis

**Methods:** Autophagy was assessed in CD4+ T cell subsets from autoimmune arthritis pateints and healthy subjects using flow cytometry. RNA sequencing and methylation array analysis was performed to understand the molecular mechanism of autophagic memory. Transcription-factor gene regulatory network analysis was build to identify key regulators. qPCR was used to confirm the gene expression level of key regulator

**Results:** We demonstrate “Autophagic memory” as elevated autophagy levels in CD4+ memory T cells compared to CD4+ naive T cells and in Jurkat Human T cell line trained with starvation stress. We then showed increased levels of autophagy in pathogenic CD4+ T cells subsets from autoimmune arthritis patients. Using RNA-sequencing, transcription factor gene regulatory network and methylation analyses we identified MYC as a key regulator of autophagic memory. We validated MYC levels using qPCR and further demonstrated that inhibiting MYC increased

**Conclusion:** The present study proposes the novel concept of autophagic memory and suggests that autophagic memory confers metabolic advantage to pathogenic T cells from arthritis and supports its resilience and long term survival. Particularly, suppression of MYC imparted the heightened autophagy levels in pathogenic T cells. These studies have a direct translational valency as they identify autophagy and its metabolic controllers as a novel therapeutic target.

**Disclosure of Interest**

None Declared

### P063 EXTENDED OLIGOARTICULAR AND POLYARTICULAR JUVENILE IDIOPATHIC ARTHRITIS PATIENTS HAVE A SIMILAR B CELL PHENOTYPE WHEN COMPARED TO ESTABLISHED RHEUMATOID ARTHRITIS

#### Rita A. Moura^1^, Alexandre Brito^1^, Susana Oliveira^1^, Rui L. Teixeira^1,2^, Vasco C. Romão^1,2^, Vítor Teixeira^1,2^, Raquel Campanilho-Marques^1,2^, Filipa Oliveira-Ramos^1,2^, João E. Fonseca^1,2^

##### ^1^Instituto de Medicina Molecular João Lobo Antunes, Faculdade de Medicina, Universidade de Lisboa, Lisbon, Portugal; ^2^Rheumatology Department, Centro Hospitalar de Lisboa Norte, EPE, Hospital de Santa Maria, Lisbon Academic Medical Centre, Lisbon, Portugal

###### **Correspondence:** Rita A. Moura

**Introduction:** Our group has recently described that the majority of polyarticular juvenile idiopathic arthritis (pJIA) and a large fraction of extended oligoarticular JIA (oJIA) patients fulfil classification criteria for rheumatoid arthritis (RA) in adulthood. B cells play several important roles in RA pathogenesis, but it is still unclear if the pattern of B cell involvement in pJIA and extended oJIA follows what has been described for adults with RA.

**Objectives:** The main goal of this study was to characterize peripheral blood B cell phenotype and cellular activation in pJIA and extended oJIA patients when compared to established RA.

**Methods:** Blood samples were collected from JIA patients (N=10; mean age 10 ± 4 years), established RA patients treated with synthetic DMARDs (N=10; mean age 72 ± 7 years) and two corresponding groups of age- and sex-matched healthy donors. B cell phenotype was characterized by flow cytometry and B cell apoptosis was assessed after 48H of in vitro cell culture.

**Results:** JIA patients recruited in this study were either classified as extended oJIA (N=6) or pJIA (N=4). Seven JIA patients (4 extended oJIA and 3 pJIA) were treated with methotrexate and three patients (2 extended oJIA and 1 pJIA) were untreated. We found that JIA patients had similar CD19+ B cell levels in circulation when compared to controls, but significantly higher CD19+ B cell frequencies in comparison to established RA. In addition, increased frequencies of transitional (IgD+CD38++) and naïve (IgD+CD27+) B cell subpopulations were observed in JIA patients when compared to RA. However, established RA patients had significantly higher levels of CD21^low^CD38^low^, post-switch (IgD-CD27+) and IgD-CD27- memory B cell subsets when compared not only to controls, but also to JIA patients. No significant differences were detected in pre-switch (IgD+CD27+) memory and plasmablasts (IgD-CD38++) levels in JIA patients when compared to both controls and RA. Furthermore, the frequency of CD5+ B cells, CD5 median fluorescence intensity (MFI), CD40 MFI and CXCR5 MFI B cell expression levels were significantly increased in JIA patients when compared to established RA, but not to controls. No significant differences were observed between JIA and established RA patients in BAFF-R, FcgRIIB, CD21, CD23, CD38, CD86, CD95, HLA-DR, TLR9 and RANKL expression on B cells. After 48H of in vitro cell culture a significantly higher B cell death was found in JIA in comparison to RA patients.

**Conclusion:** The increased frequencies of transitional, naïve and CD5+ B cells in circulation and reduced levels of memory B cell subpopulations in JIA patients when compared to established RA are probably related to an immature immune system present in children when compared to adults. Nevertheless, the similarity in B cell phenotype found between extended oJIA, pJIA and established RA patients suggests an early B cell involvement in the pathogenesis of these two categories of JIA.

**Disclosure of Interest**

None Declared

### P064 THE AUSTRALIAN ARTHRITIS AND AUTOIMMUNE BIOBANK COLLABORATIVE [A3BC] PROTOCOL

#### Jane E. Munro^1^, Lyn March^2^, Craig Willers^3^, Chris Jackson^3^, Meilang Xue^3^, Catherine Hill^4^, Mihir Wechalekar^5^, Susan Lester^6^, Helen Benham^7^, Tony Kenna^7^, Helen Keen^8^, Chandima Perera^9^, Davinder Singh-Grewal^10^, Kevin Murray^11^, Pavla Walsh^12^, Peter Gowdie^13^, Christina Boros^14^, Navid Adib^15^, Joanna Cobb^1^, Rachelle Buchbinder^16^, Marissa Lassere^17^, Jeff Chaitow^18^, Mike Inouye^19^, Claire Barrett^20^, Graeme Carroll^21^, Justine Ellis^1^

##### ^1^Murdoch Children's Research Institute, Melbourne; ^2^Institute for Bone and Joint Research (IBJR): University of Sydney & Royal North Shore Hospital, NSW; ^3^Institute of Bone & Joint Research - Sydney University & Kolling Institute, Sydney; ^4^Royal Adelaide Hospital6; ^5^Flinders Hospital; ^6^Queen Elizabeth Hospital, Adelaide; ^7^University of Queensland Diamantina Institute, Brisbane; ^8^Fiona Stanley Hospital, Perth; ^9^Canberra Hospital, Canberra; ^10^Westmead Children's, Sydney; ^11^Princess Margaret Hospital21; ^12^Princess Margaret Hospital, Perth; ^13^Monash Children's, Melbourne; ^14^Women's and Children's, Adelaide; ^15^Wesley Hospital, Brisbane; ^16^Monash Univeristy, Melbourne; ^17^Uni NSW; ^18^Westmead Childrens, Sydney; ^19^Baker Institute, Melbourne; ^20^Royal Brisbane and Women’s Hospital25, Brisbane; ^21^Private practice, Perth, Australia

###### **Correspondence:** Jane E. Munro

**Introduction:** The australian arthritis and autoimmune biobank collaborative = a3bc a national paediatric and adult rheumatology clinical research network

**Objectives:** The A3BC seeks safer, more effective and evidence-based prevention, diagnosis, treatment and prognosis strategy in arthritis and autoimmune disease. Key A3BC aims are to

Develop a higher level of capability by collaborating across multiple disciplines and sector

Progress precision medicine by growing capacity in open-access, data-linked biobanking

Innovate preventive medicine by forming unique partnerships with population health research

Demonstrate a new era of data linkage and use to inform policy and practice decision-making

-

**Methods:** The A3BC protocol was developed in consultation with leading researchers, clinicians, industry and best practices. A3BC processes will initially run parallel to the Australian Rheumatology Association Database (ARAD), but will merge in the future. The initial A3BC disease focus is Rheumatoid Arthritis, Juvenile Arthritis, Psoriatic Arthritis and Ankylosing Spondylitis.

Recruited through over 40 sites nationally, participants donate blood (20-53mls) and synovial tissue/fluid, stored across 10 biobank nodes as plasma, serum, PBMCs, DNA and RNA. On a project basis, newborn screening cards, urine and/or faeces are accessed.

These samples are accompanied by standardised pre-analytical variables and clinical data, and linked datasets including the ARAD, electronic medical records, Commonwealth health (e.g. Pharmaceutical Benefits Scheme, Medicare)), registries (e.g. cancer, death), longitudinal/lifecourse data (e.g. Juvenile Arthritis national CLARITY project), and consumer entry (My Health Record).

Using cutting-edge capture, real-time analytics and dashboarding processes and systems, all data is integrated and mined for patterns and associations, then effectively communicated to practitioners and policy-makers.

**Results:** The A3BC protocol will result in new dataset integration systems, new multidisciplinary collaborations, and identification of new risk factors, biomarkers and cross-dataset associations. It will improve research by enabling innovative research questions and faster translation. And facilitate health policy/practice decision-making in precision and preventive medicine.

**Conclusion:** The A3BC protocol provides best-practice, quality assured and validated methods to realise its aims. Current experience from recruiting and processing participants demonstrates that the protocol’s methods and technologies are fit-for-purpose.

**Disclosure of Interest**

None Declared

### P065 ROLE OF THE ORAL MICROBIOME IN PEDIATRIC AUTOIMMUNE AND AUTOINFLAMMATORY DISEASE

#### Prasad T. Oommen, Benjamin Reinbeck, Arndt Borkhardt, Ute Fischer

##### ^1^Clinic for Paediatric Oncology, -Hematology and Clinical Immunology, Heinrich-Heine-University, Medical Faculty, Duesseldorf, Germany

###### **Correspondence:** Prasad T. Oommen

**Introduction:** Neither genetic, epigenetic nor environmental factors alone have been able to unravel the pathogenesis of autoimmune and autoinflammatory diseases in children and adolescents. The specific aspects which determine the individual susceptibility to the development of a rheumatic disease are still to be elucidated. As of now, these conditions are seen as multifactorial in respect of pathogenesis. The microbiome has been under discussion as a contributing factor. Microbial communities who play an important role in the physiological development of the immune system may turn into "false friends" leading to the state of dysbiosis.

**Objectives:** We aimed to gain new data in this context via examing the oral microbiome in patients with different rheumatic diseases in children and adolescents.

**Methods:** We examined the oral microbiome by obtaining oral swabs of 11 patients with juvenile idiopathic arthritis and 20 patients with chronic non-bacterial osteomyelitis (CNO).

The hypervariable region V6–V7 of the 16S rRNA gene was amplified and subsequently sequenced. The following bioinformatic analysis was performed with QUIIME (Quantitative Insights Into Microbial Ecology).

**Results:** In accordance to previous studies we were able to identify Actinobacteria and Fusobacteria on the phylum level as microbiota only present in our JIA cohort. In the CNO cohort so far no distinct microbiotic players could be detected. For this entity the only data so far describe an advantage in mice who were fed with a high-fat diet resulting in a shift in the microbial environment leading to a better course of the disease. These mice data could - as of now - not be shown in our patients.

**Conclusion:** The microbiome may open a new door to a better understanding of multifactorial autoimmune and autoinflammatory disease. In accordance to previous studies we were able to identify specific microbiota present only in JIA patients. However, the clinical consequence and the exact immunological effects of dysbiosis are still to be unravelled. As of now, studies of the microbiome may enhance the pathophysiologic understanding of these diseases.

**Disclosure of Interest**

None Declared

### P066 EVALUATION OF THE ROLE OF SIGNAL PEPTIDE-CUB-EGF DOMAIN-CONTAINING PROTEIN (SCUBE)1 AND SCUBE2 IN JUVENILE IDIOPATHIC ARTHRITIS

#### Duygu Selimoğlu^1^, Ferhat Demir^2^, Süleyman C. Karahan^3^, Mukaddes Kalyoncu^2^

##### ^1^Department of Pediatrics; ^2^Department of Pediatric Rheumatology; ^3^Department of Biochemistry, Karadeniz Technical University Faculty of Medicine, Trabzon, Turkey

###### **Correspondence:** Duygu Selimoğlu

**Introduction:** Juvenile idiopathic arthritis (JIA) is a disease that begins in patients under sixteen years of age, continues longer than six weeks, and diagnosed with arthritis in at least one joint. Other causes of arthritis should be excluded for diagnosis of JIA. The etiopathogenesis is still unclear and it is thought that it is the result of the immunological response due to environmental and genetic factors.  *Signal peptide-CUB (complement C1r/C1s, Uegf, and Bmp1)-EGF (epidermal growth factor)-like domain-containing protein* (SCUBE) is a newly defined, secreted cell surface protein. SCUBE1 and SCUBE2 have been shown to play a role in various pathophysiological processes such as vascular endothelial cells, inflammation, cancer metastasis, and vascular diseases.

**Objectives:** We aimed to research whether there is a relationship between SCUBE1 and SCUBE2 proteins, and JIA.

**Methods:** Between January 2016 and May 2017, the patients admitting to outpatient clinics in Pediatric Department of Farabi Hospital in Karadeniz Technical University and diagnosed with JIA according to ILAR criteria were included. Patients who used steroid or non-steroidal anti-inflammatory drugs in last three months were excluded. Blood samples collected from patients who were in active and recovery phases of their diseases and healhty control group. IL (interleukin)-1, IL-6, TNF (tumor necrosis factor)-α, SCUBE1 and SCUBE2 levels were studied by ELISA method. Patients with oligoarticular JIA were classified as Group 1, patients without oligoarticular JIA as Group 2 and these two groups were compared according to the blood results of cytokines and SCUBE’s mentioned above.

**Results:** A total of 24 patients, 11 (45.8%) were male and 13 (54.2%) female, aged between 2 and 17 years old, who were diagnosed with JİA, were included in the study. Fifteen (62.5%) patients were diagnosed as oligoarticular JIA, eight (33.3%) patients polyarticular JIA and one (4.2%) patient systemic JIA. İn the active phase of disease, IL-1 and IL-6 levels found significantly higher in Group 2 than in Group 1 (p=0.01 and p=0.004, respectively). TNF-α and SCUBE1 levels were higher in healing phase of the disease than in active phase (p=0.03 and p=0.01, respectively). TNF-α levels were higher in the active period of the disease than in the control group (p=0.02). SCUBE1 levels in the healthy control group were found to be significantly higher than the active period of the disease (p=0.009). TNF-α levels were higher in the recovery period of the disease than in the control group (p=0.004). SCUBE1 and TNF-α levels were found to be higher in the healing period of disease than in the active period (p=0.02 and p=0.03, respectively). SCUBE2 levels were found to be higher in patients with thrombocytosis in acute period (p= 0.01).

**Conclusion:** These results concluded that SCUBE can not be used as early biomarker in JIA, but SCUBE may has a role of JIA pathogenesis. We believe that this situation can be elucidated by studies on more patients in this regard.

**Disclosure of Interest**

None Declared

### P067 FEATURES OF IMMUNOLOGICAL PARAMETERS IN CHILDREN WITH VARIOUS ARTICULAR PATHOLOGIES.

#### Anna Y. Spivakovskaya^1^, Yuri Spivakovskiy^1^, Yuri Chernenkov^1^

##### ^1^Depertment of Hospitality Pediatrics, Saratov State Medical University, Saratov, Russian Federation

###### **Correspondence:** Anna Y. Spivakovskaya

**Introduction:** Timely diagnosis of various diseases of their nature, manifested by inflammatory changes in the joint apparatus of the child, is one of the priority tasks of modern children's rheumatology. Juvenile idiopathic arthritis (JIA) remains one of the most serious and potentially disabling diseases. At the same time, one of the most complicated tasks is the timely differential diagnosis of various variants of the articular form of juvenile arthritis and reactive arthropathy (ReA).

**Objectives:** To determine the concentration of individual Th1- (IL-1β, IL-6, INF-γ), Th2- (IL-4) and Th17-associated interleukins (IL-17) in serum in children with JIA and ReA.

**Methods:** The study involved 78 children, aged 1.1 years to 16.11 years. A group of children with a verified diagnosis of JIA consisted of 56 patients - 30 patients with an oligoarticular variant of the course of the disease and 26 with a polyatricular variant, an average age of 10.2 ± 3.8 years, a group of ReA - 22 children an average age of 7.49 ± 3.95 years. To compare the revealed relationships, a similar analysis was carried out in the group (n = 20) of relatively healthy children, the average age was 9.2 ± 3.99 years.

The concentration of interleukins in the blood serum was determined by ELISA using ready-made commercial kits for the detection of IL-1β IL-4 IL-6 INF-γ from Vector-Best Europe (Novosibirsk, Russia) and IL-17 firm "eBioscience" (Vienna, Austria).The work was carried on the automated enzyme immunoassay analyzer "Lazurite" (Dynex Technologies Inc., USA).

**Results:** In children with JIA, a higher concentration of Th1-associated cytokines was observed with respect to similar parameters in children with ReA and from the comparison group, so the concentration of IL-1β was 1.5-2 times higher than in patients with ReA and in relatively healthy children , INF-ɣ was 1.9 times higher than in patients with ReA and 3.5 times higher than the values ​​in the comparison group. At the same time, the concentration of Th2-associated cytokine (IL-4) in the blood serum of children with JIA was practically the same as for the comparison group and children with ReA.

In patients with ReA, in the absence of significant differences in the concentration of IL-1β, IL-6, IL-4 in the blood serum from similar parameters in relatively healthy children, 1.8-fold increase in the INF-отмеча level and a 1.5-fold decrease concentration of IL-17.

In children with articular form of JIA and ReA, there was a mixture of immune responses toward the activation of Th1-associated cytokines relative to the parameters of the comparison group, but with JIA this imbalance was greater than in patients with ReA. A key role in mixing immune responses towards the activation of Th1 cells in patients with ReA was played by a change in the ratio INF-ɣ / IL-4. A change in the balance in the Th1 / Th17 cell system was due to a change in the INF-ɣ / IL-17 ratio.

When performing the correlation analysis in patients with different variants of articular pathology, the following significant correlation pairs were obtained: for the oligoarticular version of JIA-INF-ɣ and IL-4; polyarticular JIA-IL-1β and IL-4, IL-1β and IL-17, IL-6 and IL-17; ReA-INF-ɣ and IL-17, 1β and IL-17.

**Conclusion:** The course of JIA and ReA is characterized by a definite unidirectional disorder in the Th1- and Th2-associated cytokines, which fits into the overall picture of the deployment of the cascade of immuno-inflammatory reactions, which is relatively universal, but both by the severity of the established imbalance and by the nature of the revealed relationships immunological parameters has its own characteristics. The most informative at the stage of the differential diagnostic process of the joint form of JIA and ReA can be considered an interrelated analysis of the immunological activity of the inflammatory reaction.

**Disclosure of Interest**

None Declared

### P068 ANTI-CYTOSOLIC 5′-NUCLEOTIDASE 1A AUTOANTIBODIES ARE COMMON IN PATIENTS WITH JUVENILE IDIOPATHIC ARTHRITIS AND ARE NEGATIVELY ASSOCIATED WITH UVEITIS IN PATIENTS WITH OLIGOARTICULAR DISEASE

#### Sarah L. Tansley^1^, Zoe E. Betteridge^2^, Diana Bosneaga^2^, Eleanor Pring^2^, Daniel M. Slade^2^, Angela Midgley^3^, Michael Beresford^3^, Andrew Dick^4^, Wendy Thomson^5^, Lucy R. Wedderburn^6^, Athimalaipet Ramanan^4^, Neil J. McHugh^2^ and Childhood Arthritis Prospective Cohort Study

##### ^1^Rheumatology, Royal National Hospital for Rheumatic Diseases; ^2^Pharmacy and Pharmacology, University of Bath, Bath; ^3^University of Liverpool, Liverpool; ^4^University of Bristol, Bristol; ^5^University of Manchester, Manchester; ^6^Institute of Child Health, UCL, London, UK

###### **Correspondence:** Sarah L. Tansley

**Introduction:** Autoantibodies targeting cytosolic 5′-nucleotidase 1A (cN1a) were first identified in adults with inclusion body myositis but have subsequently been reported in a variety of other autoimmune diseases. They have recently been described in patients with Juvenile Idiopathic Arthritis (JIA) but to date clinical associations are unknown [1].

**Objectives:** We aimed to determine the prevalence and clinical associations of anti-cN1a in a UK cohort of patients with JIA.

**Methods:** We screened sera from 227 patients with JIA enrolled in the Childhood Arthritis Prospective cohort study for anti-cN1a by ELISA (16 systemic, 98  oligoarthritis, 52 rheumatoid factor negative polyarthritis, 12 rheumatoid factor positive polyarthritis, 9 enthesitis related arthritis, 16 psoriatic, 12 undifferentiated). In addition, sera from 45 healthy juvenile controls, 52 patients with Juvenile-onset myositis (JDM) and 20 patients with Juvenile onset Systemic Lupus Erythematosus (JSLE) were also screened. The negative cut-off was defined as more than 5 standard deviations above the mean of 34 normal healthy (adult) serum controls.

**Results:** Anti-cN1a were not identified in any healthy controls nor children with JDM but were present in 3 (15%) of patients with JSLE.

Anti-cN1a were present in 19 (8%) of children with JIA. They were present in all ILAR defined subgroups with the exception of systemic-onset JIA and were most common in those with enthesitis related JIA (22% anti-cN1a positive).

JIA patients with anti-cN1a were typically older at disease onset, although the age range was wide, median 8.8 years (IQR 2.7-12.1) versus 5.5 (IQR (2.0-11.0). The proportion of females was similar in both groups (74%  anti-cN1a positive and 66% anti-cN1a negative). Patients with anti-cN1a were more likely to be ANA positive 84% versus 53% (p=0.02) a dense fine speckle or homogenous immunofluorescence pattern was most common but different immunofluorescence patterns and ANA negative individuals were found in both groups.

Data on uveitis was limited to 139 JIA patients (10 patients with anti-cN1a). Three patients with anti-cN1a had a history of uveitis (one each of rheumatoid factor negative polyarthritis, enthesitis related arthritis and psoriatic arthritis) compared to 56 of 129 patients with available data who were anti-cN1a negative.  Of the 68 patients with oligoarthritis with uveitis data, 10 were anti-cN1a positive  and this group were less likely to develop uveitis than oligoarthritis patients who were anti-cN1a negative (p=0.03).

**Conclusion:** cN1a is a common target of circulating autoantibodies in UK children with JIA and are seen in all subtypes except systemic JIA. In patients with oligoartiuclar JIA, who are at a higher risk of uveitis, the presence of anti-cN1a was negatively associated with a history of uveitis. Further work is needed but patients with oligoarticular JIA and anti-cN1a may be at a lower risk of uveitis and may therefore not require as frequent ophthalmological screening.

**References**

1. Yeker, R.M., et al., *Anti-NT5C1A autoantibodies are associated with more severe disease in patients with juvenile myositis.* Ann Rheum Dis, 2018. **77**(5): p. 714-719.

**Disclosure of Interest**

None Declared

## Poster Walk 4: JIA clinical 1

### P069 ‘A FIRST-DEGREE RELATIVE WITH PSORIASIS’ AS A CRITERION FOR THE ILAR CLASSIFICATION SYSTEM FOR JUVENILE IDIOPATHIC ARTHRITIS

#### Roselie Achten^1^, Joost Swart^1^, Gabriella Giancane^1^, Tom Wolfs^1^, Michael Hofer^1^, Ekaterina Alexeeva^1^, Violeta Panaviene^1^, Susan Nielsen^1^, Jordi Anton^1^, Florence Uettwiller^1^, Valda Stanevica^1^, Maria Trachana^1^, Denise Pires Marafon^1^, Constantin Ailioaie^1^, Elena Tsitsami^1^, Sylvia Kamphuis^1^, Troels Herlin^1^, Pavla Dolezalova^1^, Gordana Susic^1^, Berit Flato^1^, Flavio Sztajnbok^1^, Angela Pistorio^1^, Alberto Martini^1^, Nicolino Ruperto^1^, Nico Wulffraat^1^

##### ^1^PRINTO, Istituto Gaslini, Genova, Italy

###### **Correspondence:** Roselie Achten

**Introduction:** Literature showed the importance of classifying patients into the right Juvenile Idiopathic Arthritis (JIA) category to provide them with optimal treatment^1^. However, a substantial amount of patients were classified as ‘undifferentiated JIA’ (UJIA) due to the strict exclusion criteria, harbouring the risk of losing the indication of the preferred treatment.

**Objectives:** The aim of this study was therefore to investigate the influence of ‘a first-degree relative with psoriasis’, which is an inclusion criterion for psoriatic arthritis and an exclusion criterion for the ‘remaining five JIA categories’. This results in more insight in the accuracy of the classification system for JIA from the International League of Associations for Rheumatology (ILAR).

**Methods:** Analyses were made in a retrospective and prospective cohort of 8,309 patients with JIA. 606 patients were classified as UJIA, 303 patients as ‘psoriatic arthritis’ (PSA) and 7,400 patients as one of the ‘remaining five categories’.

**Results:** 225 (37.1%) patients with UJIA had ‘a first-degree relative with psoriasis’. No significant difference was found between the PSA and UJIA category in the number of patients with ‘a first-degree relative with psoriasis’. None of the abovementioned 225 patients developed psoriasis during the observation period. Only 60 (20.8%) patients were classified as PSA because of the inclusion criterion of having ‘a first-degree relative with psoriasis’ in the PSA category.

**Conclusion:** The exclusion criterion ‘a first-degree relative with psoriasis’ increases the number of patients classified as UJIA. No significant difference was found between the PSA and UJIA category in the number of patients with ‘a first-degree relative with psoriasis’, suggesting this criterion is not of added value for the ILAR classification system for JIA. We conclude that ‘a first-degree relative with psoriasis’ is not a necessary inclusion criterion for psoriatic arthritis. For these reasons, we suggest removing the inclusion and exclusion criterion ‘a first-degree relative with psoriasis’ for all the JIA categories.

**References**

1. Beukelman T, Patkar NM, Saag KG, Et Al. 2011 American College of Rheumatology Recommendations for the Treatment of Juvenile Idiopathic Arthritis: Initiation and Safety Monitoring of Therapeutic Agents for the Treatment of Arthritis and Systemic Features. Arthritis Care Res. 2011;63(4):465–82.

**Disclosure of Interest**

None Declared

### P070 THE FREQUENCY OF JUVENILE SPONDYLOARTHROPATHIES IN CHILDREN WITH FAMILLIAL MEDITERRANEAN FEVER

#### Emre Ozer^1^, Demet Seker^1^, Emir Taner^1^, Amra Adrovic^1^, Sezgin Sahin^1^, Kenan Barut^1^, Oya Koker^1^, Ozgur Kasapcopur^1^

##### ^1^Department of Pediatric Rheumatology, Istanbul University, Cerrahpasa Medical School, Istanbul, Turkey

###### **Correspondence:** Amra Adrovic

**Introduction:** Familial Mediterranean fever (FMF) is the most common monogenic autoinflammatory disease characterized with fever, recurrent episodes of self-limiting polyserositis and arthritis. FMF arthritis is generally acute monoarthritis especially in the larger joints of the lower extremities, healing without a sequelae. However some of the patients develop different type of chronic arthritis, predominantly oligoarticular juvenile idiopathic arthritis (JIA) and juvenile spondyloarthropathies **(**JSpA). Studies on JSpA among childhood FMF patients are scarce.

**Objectives:** To evaluate the frequency of JSpA in a large childhood FMF cohort. Furthermore, we aimed to define main characteristics of JSpA among childhood FMF patients.

**Methods:** A total of 320 juvenile FMF patients were blindly questioned according to recently proposed criteria for JSpA by 3 researchers (EO, DS, ET). A standardized case report form including demographic data, clinical features, MEFV mutation and treatment was prepared and completed for each patient. Patients fulfilled the JSpA criteria were previously classified as probable JSpA. Afterwards, an expert in pediatric rheumatology (OK) reevaluated the classified patients and some of them were confirmed to be a definite while some of them were accepted as potential JSpA patients.

**Results:** 37 patients (11.5%) were initially classified as potential JSpA: 32 (10%) were accepted as definite and 5 (1.5%) as probable JSpA. Demographic, clinical and treatment data of definitive JSPA patients are shown in Table 1.

**Conclusion:** Articular involvement compatible with JSpA could be seen in childhood FMF patients. Spondyloarthropathies were detected in 10% of childhood FMF cases. The M694V mutation is the most common MEFV mutation among JSpA patients with FMF. JSpA should be considered in childhood FMF patients, especially in those chronic arthritis, axial involvement and enthesopathy.

**Disclosure of Interest**

None Declared

**Table 1 (abstract P070).** See text for description

	FMF + Definite JSPA	FMF + Probable JSPA	FMF patients without JIA and JSpA	FMF + JIA (except ERA or JSpA)
Patients, n	32	5	268	15
Female, n (%)	10 (31.25%)	1 (20%)	148 (55.22%)	10 (66.66%)
Age of disease onset, mean ±SD years	7.19 ± 3.68	5.60 ± 4.93	4.91 ± 3.40	4.93 ± 3.32
Age at study, mean ±SD years	14.84 ± 3.70	13.40 ± 1.67	12.51 ± 4.43	10.73 ± 3.57
M694V mutation n(%)	19 / 30 (63.33%)	3 (60%)	148 / 245 (60.40%)	11 (73.33%)
Disease onset >6 yrs	26 (81.25%)	5 (100%)		6 (40%)
Oligorthritis	21 (65.62%)	1 (20%)		14 (93.33%)
Inflammatory back pain	17 / 32 (53.1%)	3 / 5(60%)		0 (0%)
Enthesopathy		3 / 5(60%)		0 (0%)
Sacroiliitis	22 /32(68.7%)	0 / 1 (0%)		0 /5 (0%)
Coxofemoral Arthritis	14 /21(66.7%) 19/32(59.37%)	2 / 5(40%)		0 / 15 (0%)
Tarsometatarsal sensivity		1 / 5(20%)		1 / 7(14.3%)
HLA-B 27 Positivity	12 / 32 (37.5%)	0 / 1(0%)		0 / 3(0%)
Male Gender		4 / 5(80%)		5 /15(33.3%)
Response to NSAID	9 / 22(40.9%)	4 / 5(80%)		2 / 7(28.6%)
Limitation in Schober test (< 4 cm)	22 /32(68.7%)	2 / 5(40%)		0 / 7(0)
	21 / 26(80.7%)			
Family history of SpA group of disease, dactylitis, psoriasis or presence of IBD	7 / 32(21.87%)	1 / 5(20%)		1 /8(12.5%)
	10 / 32(31.3%)			

### P071 DIFFERENCES IN THE PERCEPTION OF DISEASE IMPACT BETWEEN US AND ITALIAN CHILDREN WITH JUVENILE IDIOPATHIC ARTHRITIS: A NETWORK ANALYSIS OF VIRTUAL FOCUS GROUPS

#### Alessandra Alongi^1^, Serena Calandra^1^, Susan Thornhill^2^, Jennifer Stinson^3^, Stephanie Luca^3^, Jennifer Horonjeff^4^, Angelo Ravelli^5^, Jane E. Munro^6^, Esi M. Morgan^7^, Alessandro Consolaro^5^, OMERACT JIA Core Set Working group

##### ^1^UNIVERSITÀ DEGLI STUDI DI GENOVA, Genoa, Italy; ^2^Thornhill Associates, Hermosa Beach, USA; ^3^The Hospital for Sick Children, Toronto, Canada; ^4^Columbia University Medical Center, New York; ^5^Istituto Giannina Gaslini, Genoa, Italy; ^6^Murdoch Children’s Research Institute, Melbourne, Australia; ^7^Cincinnati Children’s Hospital Medical Center, Cincinnati, USA

###### **Correspondence:** Alessandra Alongi

**Introduction:** The OMERACT JIA Core Set Working Group formed in 2015 as an international initiative to revise the existing Core Set with relevant patient/caregiver input. In efforts to develop an updated, patient-centered Core Outcome, virtual focus groups (VFGs) to identify main patient/caregivers-valued themes regarding the physical, mental, and social impact of JIA disease activity states were conducted.

**Objectives:** To identify and prioritize key features defining patient-perceived inactive disease state and to examine possible cross-cultural differences in the main domains

**Methods:** Two sets of paired VFGs were conducted with JIA patients (adolescents and young adults) and parents (split by ages of their children) in two clinics, in the US and in Italy respectively. Eligibility included prior or current experience of JIA disease inactivity (>3 months). A 3-day facilitated online board was held per group, focusing on the impact of JIA on physical, mental and social health, and the perceived differences between active and inactive JIA. Participants were asked to identify and rank their five top priority features defining inactive disease. Content analysis of transcripts was conducted independently in the two centers and compared. Coded transcripts were further analyzed using network analysis, an approach that allows to study the structure of the connections between domains and the impact of specific items, measured by centrality indexes.

**Results:** 86 subjects were included. Caregiver were split by ages of their children (under and over 15 years old in the US sample; under and over 10 years old in the Italian sample). Patients were split in adolescents (15-17 years old in the US sample, 15-18 years old in the Italian sample) and young adults (18-24 years old in the US sample, 19-25 years old in the Italian sample). Qualitative analysis revealed psychosocial impact and limitations in daily activities emerged as main domains in both samples; fear of relapses and burden of medications were indicated as concerns mostly by Italian patients and caregivers, while the impact on children’s activities and family life appeared relevant in US groups. Network models of ranked “top five” remission-defining items identified absence of pain as the single item with the highest degree, closeness and between centrality, indicating high relevance; others central themes in both samples were absence of functional limitations, improved engagement and autonomy. When comparing network models by the two populations, reduction of anxiety and absence of uveitis showed the greatest differences in centrality across the samples, respectively showing higher centrality in the Italian and in the US groups.

**Conclusion:** Data analysis revealed several domains that need to be evaluated for the development of patient/parent-centered outcomes assessment instruments. Network models revealed the central role of pain, functional limitation and restricted participation in patient-perceived remission in both populations. Main differences in the two populations include the role of relapses, treatments and secondary demands in the impact of disease. Further analysis and cross-cultural validation of the results is planned to inform the development of patient/parent-centered outcomes measures.

**Disclosure of Interest**

None Declared

### P072 POLYARTICULAR JUVENILE IDIOPATHIC ARTHRITIS WITH RHEUMATOID FACTOR: A RETROSPECTIVE MONOCENTRIC STUDY OF 46 PATIENTS

#### Charlotte Boussard^1^, Ambre Hittinger^1^, Pierre Quartier-Dit-Maire^1,2^, Wouters Carine^1^, Brigitte Bader-Meunier^1,2^

##### ^1^IMAGINE Institute and Pediatric Immunology, Hematology and Rheumatology Unit, Necker-Enfants Malades Hospital, Assistance Publique Hôpitaux de Paris; ^2^Paris-Descartes University, Paris, France

###### **Correspondence:** Charlotte Boussard

**Introduction:** Rheumatoid factor (RF) positive polyarticular juvenile idiopathic arthritis (JIA) with is a rare subtype of JIA (2-5%) of patients. No series have been published on this topic.

**Objectives:** Our objective was to describe the demographics characteristics and the course of RF positive polyarticular JIA.

**Methods:** We performed a monocentric retrospective study of patients with RF positive polyarticular JIA included between 2008 and February 2018 in the database CEMARA of the French reference center for juvenile arthritis and rare pediatric systemic autoimmune diseases (RAISE). This data base has been approved by the French Commission Nationale Informatique et Libertés. We recorded patients age at onset, gender, familiy history, comorbities, growth-height charts, acute phase reactants, positivity of antinuclear antibodies (ANA) and anti-citrullinated protein antibodies (ACPA), treatments, side effects and course. Clinical remission was defined according to Wallace criteria^1^.

**Results:** Fourty-six patients (40 girls, 6 boys) were included. The median age at onset was 13 years (5-23) and the median follow-up 3 years (1-15). Sixteen patients (35%) had a familial history of auto-immunity or juvenile inflammatory rheumatism. Two patients were siblings from the same non-consanguineous parents. Fourteen patients (30%, 4/6 boys, 10/40 girls) had associated conditions: 4 patients had auto-immune diseases (thyroiditis, vitiligo, Raynaud syndrome, insulin dependent diabetes), 3 (6,5%) had bronchiectasis, 5 (10,8%) a growth retardation. Finally two patients had a Mendelian diseases: Marfan syndrome and hereditary multiple osteochondromas resulting from heterozygote mutation of EXT1. ACPA and ANA were positive in 27/33 (82%) and 19/24 (79%) patients respectively. Twenty-one patients (45,6%) had an elevation of acute phase reactants at disease onset. Eight patients (17%) had erosive arthritis within the first year of evolution. The first line treatment was METHOTREXATE for 34 patients (74%) alone in 11 patients (32%)) or in association with anti-TNF in 23 patients (67%)). Twenty-four/27 patients (88%) were treated by anti-TNF: ETANERCEPT (74%), ADALIMUMAB (11%), and INFLIXIMAB (3.7%). Other biologic agents were RITUXIMAB (n=2), TOCILIZUMAB (n=4). ABATACEPT (n=6). Sixteen patients (35%) received short or sustained treatment with corticosteroids. Primary or secondary resistance for the first biological agent occurred in 3 (6.5%) and 4 patients (8.6%) respectively. Seventeen out of 35 patients (48%) were on remission on treatment or off treatment  (mean follow-up without relapse: 7 and 8 years respectively). Four patients (15%) who were treated by at least one biological agent presented side effects, which required to stop the treatment.

**Conclusion:** Our study confirms the severity of RF positive polyarticular JIA and emphasizes its demographic and immunological heterogeneity. This first report of bronchiectasis in this population emphasizes the need for a careful pulmonary evaluation. The frequency of family auto-immunity history and the occurrence in two siblings suggest that some FR positive polyarticular JIA might be monogenic diseases.

**References**

1. Wallace CA, Giannini EH, Huang B, Itert L, Ruperto N, Null N, et al. American College of Rheumatology provisional criteria for defining clinical inactive disease in select categories of juvenile idiopathic arthritis. Arthritis Care & Research. 1 juill 2011;63(7):929‑36.

**Disclosure of Interest**

None Declared

### P073 FREQUENCY OF THORACOLUMBAR SPINE INVOLVEMENT IN PATIENTS WITH JUVENILE IDIOPATHIC ARTHRITIS

#### Selcan Demir, Fatma B. Ergen^2^, Hafize E. Sönmez^1^, Erdal Sag^1^, Yelda Bilginer^1^, Ustun Aydıngöz^2^, Seza Ozen^1^

##### ^1^Department of Pediatric Rheumatology; ^2^Department of Radiology, HACETTEPE MEDICAL FACULTY, Ankara, Turkey

###### **Correspondence:** Selcan Demir

**Introduction:** Juvenile idiopathic arthritis (JIA), is the most common chronic rheumatic disease among the world and the diagnosis is based on the exclusion of other causes in the presence of arthritis, lasting at least 6 weeks in children under 16 years of age.

**Objectives:** We aim to assess the frequency of thoracolumbar spine (TLS) involvement and Magnetic Resonance Imaging (MRI) findings in the patients with JIA.

**Methods:** Patients who underwent for thoracolumbar MRI, known or suspected JIA between January 2015–2017, were retrospectively re-examined by a musculoskeletal radiologist for the presence of inflammatory (central/corner lesion, costovertebral inflammation, supraspinous or interspinous enthesitis) and erosive (wedging, central erosion) lesions. At the time of spinal MRI we also investigated the presence of sacroiliac joint (SIJ) involvement, peripheral joint (PJ) involvement and symptoms for spine involvement.

**Results:** Totally 108 patients were re-examined. Finally, 52 patients (mean age: 14.5±3.4) with a definitive diagnosis of JIA were enrolled in the study. Among them 41 had enthesitis related arthritis (ERA), 4 had oligoarticular (OA), 5 had polyarticular (PA) JIA, one had psoriatic arthritis (PsA) and one had systemic JIA (sJIA). Among ERA patients there were at least one inflammatory lesion in 23 (56%), one erosive lesion in 3 (%7), and both inflammatory and erosive lesions in 3 (7%) patients. In ERA patients with positive findings at TLS MRI, 19 (82%) had sacroiliitis and 14 (60%) had peripheral arthritis concurrently. 15 (65 %) were clinically asymptomatic in terms of TLS involvement (none of them had inflammatory back pain or morning stiffness, the Schober’s test [>6 cm] and chest expansion [>10 cm] were normal). Among the patients with other subtypes of JIA, there were at least one inflammatory lesion in 5 (45%) and at least 1 erosive lesion in 1 (9%). In non-ERA patients with positive findings at TLS MRI, 6 (83%) were clinically asymptomatic for TLS involvement. Of note, one patient had sacroiliitis and 5 patients had peripheral arthritis in addition to TLS involvement.

**Conclusion:** Although current literature indicates that spinal MRI shows insufficient evidence for detecting early JIA; with this study we demonstrated that, Inflammatory and/or erosive lesions of thoracolumbar spine can be seen in asymptomatic patients of ERA and even in non-ERA JIA patients and the majority of spinal lesions detected in asymptomatic ERA and non-ERA JIA patients were inflammatory.

**Disclosure of Interest**

None Declared

### P074 DAILY PHYSICAL ACTIVITY AND SLEEP IN CHILDREN WITH JUVENILE IDIOPATHIC ARTHRITIS AND HEALTHY CHILDREN IN RELATION TO PAIN

#### Simon Esbersen^1^, Troels Herlin^1^, Birgitte T. Mahler^1^

##### ^1^Pediatrics and Adolescents Medicine, Pediatric Rheumatology, Aarhus University Hospital, Skejby, Denmark

###### **Correspondence:** Simon Esbersen

**Introduction:** Around 30% of children with juvenile idiopathic arthritis (JIA) suffer from daily pain regardless of medication. Pain in JIA causes disturbances in the circadian rhythm compared to healthy children.

**Objectives:** To study if pain is related to disturbed sleep, physical activity, and higher JIA disease activity compared to healthy age-matched controls.

**Methods:** A prospective observational study was conducted between 2016 and 2018. We wanted to include 100 JIA patients and 200 healthy age-matched controls between 6-16 years old. JIA patients were consecutively asked during their routinely visits to our outpatient clinic. Controls were invited by notice on the parents IT-based communication platform in schools. They were asked to complete a sleeping schedule and a pain app daily for one week. One time they answered retrospective surveys about pain, sleep, and activity.

**Results:** 93 with JIA and 188 controls were included. 76% of JIA patients and 43% of controls reported pain in retrospective questionnaires. In one week's observation, 84% with JIA and 70% of controls had pain minimum once. 32% with JIA and 6 % of controls reported daily pain. Pain intensity (VAS score 0-10) reported as median [min;max] was: 1.00 [0;8.25] for JIA patients and 0.29 [0;6.29] for controls. Pain prevalence and pain intensity were significantly higher in JIA patients compared to controls (p<0.05). 55% with JIA and 90% of controls always participated in physical education, thus JIA patients were significantly less active than controls. Pain did not reduce physical activity in JIA, but pain significantly lowered the level of activity in controls (p<0.001). No significantly relation between neither sleep disturbances and study groups nor sleep disturbances and pain was found. Disease activity (JADAS-27) in JIA was significantly positively correlated to pain intensity, and significantly related to reduced physical activity (p<0.05).

**Conclusion:** Pain is common in JIA patients and controls. JIA patients are significantly less active compared to controls. Low physical activity in JIA is not related to pain, but to increased disease activity. Higher pain intensity is related to higher disease activity in JIA. In controls, pain is related to reduced activity.

**Disclosure of Interest**

None Declared

### P075 MUSCULOSKELETAL CONDITIONS AMONG PERINATALLY HIV-INFECTED ADOLESCENTS IN THE CAPE TOWN ADOLESCENT ANTIRETROVIRAL COHORT

#### Sana Mahtab^1,2^, Chris Scott^1^, Takwanisa Machemedze^1,2^, Susan Joubert^1,2^, Nana Akua Asafu-Agyei^1,2^, Landon Myer^3^, Heather J. Zar^1,2^

##### ^1^Department of Pediatrics & Child Health, Red Cross War Memorial Children's Hospital, University of Cape Town; ^2^SA MRC Unit on Child & Adolescent Health; ^3^Epidemiology & Biostatistics, School of Public Health & Family Medicine, Faculty of Health Sciences, University of Cape Town, Cape Town, South Africa

###### **Correspondence:** Sana Mahtab

**Introduction:** Perinatally HIV-infected adolescents (PHIVA) have been shown to have a higher risk of developing musculoskeletal diseases. Increasing access to antiretroviral therapy (ART) has dramatically improved life expectancy but little is known about musculoskeletal disease in PHIVA who are on ART in Africa.

**Objectives:** To study the prevalence and spectrum of musculoskeletal abnormalities in PHIVA on ART using the pediatric Gait, Arms, Legs, Spine (pGALS) screening tool.

**Methods:** HIV-infected and matched HIV-uninfected adolescents (HIV-) enrolled in the Cape Town adolescent antiretroviral cohort (CTAAC) had pGALS examination and screening musculoskeletal questionnaires completed by trained study clinicians. Childhood Health Assessment Questionnaires (CHAQ) were used to assess participants’ musculoskeletal health status; participants with abnormal pGALS examinations were referred to rheumatology.

**Results:** pGALS was performed on 473 PHIVA (median age: 13.1 years; 49% female; median age at ART initiation: 4.3 years) and 101 HIV negative adolescents (median age: 12.8 years; 54% female). Median duration on ART was 8.8 years (IQR 5.8-10.5) with 38% initiating ART at ≤2 years of age. Twenty-three percent of PHIVA were WHO HIV stage 4 at time of HIV diagnosis. At the time of pGALS screening, 60% were on two nucleoside reverse transcriptase inhibitors and a non-nucleoside reverse transcriptase inhibitor and 36% were on two nucleoside reverse transcriptase inhibitors and a protease inhibitor. Eighty percent of the PHIVA had viral load ≤100 copies/ml and the median CD4 count was 711cells/uL.

Of the 23 (4%) participants with abnormal pGALS screening, 21 (4.4%) were PHIVA and 2 (2%) were HIV negative adolescents, p=0.25. 45% of those with abnormal pGALS had abnormal CHAQ, or a positive musculoskeletal screening question. Ten (44%) of participants with abnormal pGALS screening reported physical symptoms (4 had joint pain, 3 had difficulty in getting dressed and 2 had difficulty in both getting dressed and taking stairs, 1 had joint pain along with difficulty in getting dressed and taking stairs).

Fifty percent of the PHIVA with abnormal pGALS were WHO HIV stage 4 while 22% of those with a normal pGALS were WHO stage 4, p=0.02. An abnormal pGALS was associated with longer duration of ART (median duration: 9.9 years vs 8.7 years with normal pGALS, p=0.01).

Amongst the 23 participants with abnormal pGALS, 2 PHIVA were diagnosed with Osgood-Schlatter Disease, 6 (4 PHIVA and 2 controls) with orthopedic conditions including scoliosis or osteochondroma, 9 PHIVA had underlying neurological conditions including cerebral palsy or a prior stroke and 6 PHIVA had mechanical joint conditions.

**Conclusion:** There was a low prevalence of musculoskeletal abnormalities in this group of PHIVA established on ART. Advanced HIV stage and longer exposure to ART were associated with musculoskeletal abnormalities. Screening for musculoskeletal abnormalities may target these subgroups of PHIVA.

**Disclosure of Interest**

None Declared

### P076 LONG-TERM PRESERVATION OF MEASLES AND RUBELLA SPECIFIC-IGG ANTIBODIES IN CHILDREN WITH JUVENILE SPONDYLARTHRITIS ON ANTI-TNF TREATMENT- A PROSPECTIVE CONTROLLED STUDY

#### Despoina Maritsi^1^, Stavroula Papailiou^1^, John Kopsidas^1^, Olga Vougiouka^1^, Maria Tsolia^1^

##### ^1^Second Department of Paediatrics, Medical School, National and Kapodistrian University of Athens, Athens, Greece

###### **Correspondence:** Despoina Maritsi

**Introduction:** Patients with autoimmune diseases are susceptible to infections due to their defective immune system and the receiving immunosuppressive treatment. Juvenile spondylarthritis (jSpA) often requires life-long treatment with biologics, which manage to fully control the disease. However, data regarding response and long-term immunological memory to specific vaccines are lacking.

**Objectives:** We aimed at a comprehensive assessment of how anti-TNfα therapy interferes with vaccine-specific-IgG titers in children with jSpA.

**Methods:** Prospective controlled study including 41 patients with jSpA and 149 matched-healthy controls. All patients had received two doses of MMR vaccine in early childhood. Demographic, clinical and laboratory data were collected. Type and duration of treatment was recorded. Samples were collected at diagnosis and at one and two years’ follow-up. Seroprotection rates as well as measles and rubella-IgG titers were measured and expressed as GMC's. The Hospital’s Research and Ethics’ Committee approved the study; written informed consent was obtained. Statistical significance was set at p<0.05 and analyses were conducted using STATA (version 13.0).

**Results:** The two groups had similar demographic characteristics, vaccination history and immunization status. No significant differences were detected in terms of vaccine type, time interval between the two vaccines as well as mean elapsed time from last vaccination to blood sampling. All patients in the ERA group were HLA-B27 positive. Seroprotection rates were adequate for both groups at all times. Both rubella and measles GMC’s were significantly lower in the jSpA compared to the control group (p<0.01) at one and three years’ follow up but not at diagnosis. During the follow-up period, the jSpA group had greater decrease in antibody levels as indicated from the significant interaction effect of analysis. Subgroup analysis showed that longer disease duration was directly correlated to lower antibody concentrations (p<0.01). Subgroup analysis on other parameters tested such as uveitis, sacroileitis, enthesitis, JADAS scores and type of anti-TNFa treatment did not specifically correlate with antibody loss. The same was true for comcommitant use of synthetic DMARD's.

**Conclusion:** Ant-TNFα treatment seems to reduce measles and rubella GMC’s. Further studies are required to assess long-term immunity conveyed by immunizations given at an early stage in children with rheumatic diseases, especially the ones receiving biologics. However, evaluation of immunization status against all vaccine preventable diseases in such patients may be beneficiary.

**Disclosure of Interest**

None Declared

### P077 DISCORDANCES BETWEEN THE PARENT GLOBAL ASSESSMENT OF DISEASE ACTIVITY AND GLOBAL ASSESSMENT OF GENERAL WELL-BEING IN A MULTINATIONAL COHORT OF 9137 CHILDREN WITH JUVENILE IDIOPATHIC ARTHRITIS

#### Ellen Nordal^1,2^, Alessandro Consolaro^3^, Marite Rygg^3^, Ingrida Rumba-Rozenfelde^3^, Nahid Shafaie^3^, Tadej Avcin^3^, Pierre Quartier^3^, Violeta Panaviene^3^, Nico Wulffraat^3^, Daniel Lovell^3^, Chris Pruunsild^3^, Gordana Susic^3^, Gaëlle Chédeville^3^, Soamarat Vilaiyuk^3^, Yosef Uziel^3^, Maria Trachana^3^, Pavla Doležalová^3^, Pekka Lahdenne^3^, Alberto Martini^3^, Nicolino Ruperto^3^, Angelo Ravelli^3^, For the EPOCA study group

##### ^1^Department of Pediatrics, University Hospital of North Norway; ^2^UiT The Arctic University of Norway, Tromsø, Norway; ^3^Pediatria II Reumatologia, PRINTO, Istituto Giannina Gaslini, Genova, Italy

###### **Correspondence:** Ellen Nordal

**Introduction:** The parent global assessment of disease activity (PDA) has been suggested to replace the parent global assessment of overall well-being (PWB) as the main patient-reported outcome incorporated in the Juvenile Arthritis Disease Activity Score (JADAS). JADAS is an important tool in a treat-to target-strategy in children with juvenile idiopathic arthritis (JIA).

**Objectives:** To investigate any discordance between PDA and PWB among children with JIA.

**Methods:** The EPidemiology, treatment and Outcome of Childhood Arthritis (EPOCA) study collected information on the health status of children with JIA currently followed worldwide. More than 9137 children with JIA from 118 pediatric rheumatology centers in 49 countries were included, and we analyzed the PDA and PWB reported by the parents. The differences between the visual analogue 10-cm scale (VAS) (range 0-10) of PDA and PWB were assessed. Discordance was defined moderate for a VAS difference >1 and ≤3, high for a VAS difference >3, and agreement if VAS difference was ≤1.

**Results:** PDA and PWB were available in 8615/9137 (94.3%) of the EPOCA participants. Mean (SD) PDA was 2.1 (2.7), while mean (SD) PWD was 2.0 (2.6). Among the one-fourth of patients with a VAS difference >1, the number of active joints, percentage of children with morning stiffness, and pain scores were significantly higher in the group assessing PDA higher than PWB. In contrast, there was a higher score indicating impaired quality of life when PWB was higher than PDA (Table 1). Also, in parents assessing PWB higher than PDA, the number of girls and the age at visit were significantly higher.

**Conclusion:** Overall,there is a high rate of agreement between PDA and PWB. In parents rating PDA higher than PWB, patients have higher number of active joints and pain scores, and more morning stiffness, while parents rating PWB highest had lower quality of life scores.

**Disclosure of Interest**

None Declared

**Table 1 (abstract P077).** Clinical characteristics of the cohort according to level of discrepancy between the parent assessment of overall disease activity (PDA) and the parent assessment of overall well-being (PWB).

PDA vs PWB	Total	Agreement	Low discordance		High discordance	
	Median (ICR)	≤+/-1	PDA >1-3 worse	PWB >1-3 worse	PDA >3 worse	PWB >3 worse
Female gender, n (%)	5956 (66.2)	4452 (65.4)	515 (70.3)	477 (69.1)	274 (64.9)	238 (69.8)
Age at visit (years)	11.6 (7.7; 14.8)	11.5 (7.6; 14.8)	11.3 (7.6; 14.8)	12.7 (8.7; 15.6)	10.8 (7.1; 14.9)	12.6 (9.3; 15.4)
Disease duration	4.0 (1.8; 7.0)	4.0 (1.9; 7.0)	3.5 (1.3; 6.7)	4.0 (1.8; 7.4)	3.5 (1.3; 6.6)	3.9 (1.8; 7.2)
Active Joints	0 (0; 2)	0 (0; 1)	1 (0; 3)	0 (0; 2)	1 (0; 2)	0 (0; 2)
Morning stiffness, n (%)	2923 (33.7)	1875 (28.6)	400 (56.2)	301 (45.2)	222 (54.4)	125 (38.1)
Stiffness duration (min)	15-30	15-30	15-30	15-30	15-30	<15
Pain score	1.0 (0; 3.5)	0.5 (0; 2.5)	3.5 (1.5; 5.5)	2.0 (0.5; 5.0)	2.5 (0.0; 5.0)	2.0 (0.0; 5.0)
Quality of Life	3 (1; 7)	2 (0; 6)	5 (3; 9)	7 (4; 11)	4 (2; 8)	8 (4; 11)

Values are the median (1^st^, 3^rd^ quartile), unless indicated n (%). PWB, parent global assessment of overall well-being (range 0-10); PDA, parent global assessment of disease activity (range 0-10)

### P078 BENIGN COURSE UVEITIS ASSOCIATED WITH JUVENILE IDIOPATHIC ARTHRITIS

#### Alessandro Poletto^1^, Maria Elisabetta Zannin^1^, Giorgia Martini^1^, Alessandra Meneghel^1^, Fabio Vittadello^2^, Francesco Zulian^1^

##### ^1^Department of Woman and Child Health, University of Padua; ^2^Centro explora, Padua, Italy

###### **Correspondence:** Alessandro Poletto

**Introduction:** Up to now, few studies have been addressed to the natural history of uveitis associated to Juvenile Idiopathic-Arthritis (JIA-U) in order to properly select the treatment approach since the early disease manifestations.

**Objectives:** Aim of the present study was to identify the clinical characteristics of patients with benign course JIA-U in order to early select the appropriate treatment and predict the final outcome.

**Methods:** Patients with JIA-U with at least 3 year follow, classified according with the SUN criteria for uveitis and followed at our Pediatric Rheumatology/Ophthalmology unit entered the study. Information gathered for each patient included: sex, age at onset of arthritis and uveitis, interval time between arthritis and uveitis onset; uveitis laterality, intraocular inflammation grading, number and yearly distribution of flares, structural complications. Uveitis flare was defined as an increase of cells in the anterior chamber of 2+ or more as compared to the baseline. JIA-U patients in which DMARDs or biological agents have been started at first for arthritis have been excluded. Collected data were analyzed using descriptive statistics

**Results:** A cohort of 75 patients with JIA-U, with a median follow-up of 12.2 years (range 3-23.6), entered the study. In 21 (28%) uveitis has treated with just topical drugs (Group A), the remaining 54 (72%) with systemic treatments (DMARDs or biological agents etc.) (Group B). No relevant differences were found between the two groups as far as sex distribution, JIA subtype, ANA frequency or uveitis laterality. Age at uveitis onset was significantly higher in Group A (5.4 years) than in Group B (4.3 years). The interval time between arthritis and uveitis onset was also greater in Group A (1.56 years) as compared to Group B (0.85 years). No patient in Group A reported ocular complications while in Group B they were 0.61/patient. The mean number of uveitis flares during the observation period was significantly less frequent in Group A than in Group B (1.57 versus 6.96, p < 0.001). The same was observed considering the mean number of relapses/year. Interestingly, patients in Group A had no relapses during the second year of follow-up and achieved a complete uveitis remission, on just topical treatment, after mean 2.6 years from onset, significantly earlier than in severe forms (Group B) (7.37 years, p < 0.001).

**Conclusion:** Benign course JIA-U can be identified as self-limiting condition, with low number of relapses and lack of structural complications. Patients with uveitis onset after five years of age, interval time between arthritis and uveitis onset greater than one year and no relapses during the second year of follow-up are likely to have a mild uveitis course.

**Disclosure of Interest**

None Declared

## JIA (oligo, poly, psoriatic)

### P079 IMPROVEMENT OF PATIENTS WITH JUVENILE IDIOPATIC ARTHRITIS IN TREATMENT WITH BIOLOGICAL THERAPY

#### Cespedes C. Adriana^1^, Villezcas C. Janeth^2^, Torres J. Alfonso^2^

##### ^1^Pediatric rheumatology, High Specialty Medical Unit National Medical Center Hospital general "La Raza", Mexico; ^2^Pediatric rheumatology, High Specialty Medical Unit National Medical Center Hospital generates "La Raza", Coyoacan, Mexico

###### **Correspondence:** Cespedes C. Adriana

**Introduction:** Juvenile idiopathic arthritis (JIA) is the most common rheumatic disease in childhood. In the last two decades, advances in the knowledge of pathogenesis has allowed the development of specific biological therapies for the treatment of JIA with an improvement ACR 50 of 70% on average with anti TNF and Tocilizumab a year of treatment.

**Objectives:** To evaluate the improvement and adverse events in patients with juvenile idiopathic arthritis treated with biological therapy (Etanercept, Adalimumab and Tocilizumab.).

**Methods:** Were included patients with JIA, <16 years who received biological therapy (Adalimumab, Tocilizumab, Etanercept), for a minimum of 6 months, from 2012-2017, all had signed informed consent. Demographic variables were obtained at diagnosis, clinical characteristics, laboratory and improvement variables at the beginning of the biological, 6 months, a year and when entering the study (current).

Descriptive statistics were used, averages, frequencies and percentages for, Wilcoxon and McNemar test to measure differences between two related samples.

Statistical significance was considered p <0.05.

**Results:** Forty-three patients were included, 20 male and 23 female. Average age 12 years, average age at diagnosis 8 years, average evolution time before the biological 24 months. Subtypes: Polyarticular FR+ 16 (37%), Polyarticular FR– 14 (32%), enthesitis related arthritis 6 (14%), systemic 5 (12%), oligoarticular 2 (5%). Extra-articular symptoms: fever 6 (14%) uveitis 5 (12%). Average improvement ACR at 6 months, a year and current with adalimumab 66%, 76%, 74%; Tocilizumab 81%, 80%, 80% and Etarnecept 88%, 67%, 67%, respectively.

The adverse events reported were pain in the puncture site 40%, transaminasemia 9% and seroconversion of PPD 12%.

**Conclusion:** The ACR pedi improvement was greater than 70% with the 3 biological therapies used in this study during the first 6 months of treatment, which is maintained during 28 months of follow-up (ACR average of 66%). There is an adequate safety profile with the biological treatment, without having a tuberculosis case so far.

**Disclosure of Interest**

None Declared

### P080 A DECISION ALGORITHM TO PREDICT SYNOVITIS AND GUIDE CLINICAL MANAGEMENT IN JIA PATIENTS WITH CLINICALLY SUSPECTED TMJ INVOLVEMENT

#### Alessandra Alongi^1^, Gabriella Giancane^1^, Alessandro Consolaro^1^, Giacomo Chiappe^1^, Nicola Laffi^1^, Gian Michele Magnano^1^, Angelo Ravelli^1^

##### ^1^Istituto Giannina Gaslini, Genoa, Italy

###### **Correspondence:** Alessandra Alongi

**Introduction:** Temporomandibular (TMJ) synovitis is a common but rarely symptomatic involvement that can lead to severe functional disabilities in children with Juvenile Idiopathic Arthritis (JIA). Individual clinical signs lack sensitivity and specificity to distinguish acute synovitis from chronic damage or functional alterations, while the current gold standard test to detect acute and chronic changes in TMJ - contrast-enhanced MRI- is demanding and not available everywhere.

**Objectives:** To identify potential patterns of clinical predictors of MRI findings useful to stratify risk of synovitis in patients with suspected TMJ involvement and select which patients mostly require MRI testing and which can be treated directly.

**Methods:** Reports from 166 JIA patients (332 TMJ) followed in our clinic who underwent contrast-enhanced MRI for suspected TMJ involvement between 2007 and 2017 were retrospectively collected and analyzed. Data included TMJ symptoms and their timing, clinical signs of rheumatological and orthodontic examinations preceding MR execution and MRI abnormalities. Using MR findings (synovial fluid, bone marrow edema, synovial enhancement, condylar head erosions, disc displacement) we estimated a latent class model that identified subgroups of patients with different synovitis probability. The ability of clinical findings to predict class membership was then assessed through a Tree Augmented Naive (TAN) Bayes model. Finally, decision analysis network and tree were estimated to identify the most advantage clinical strategy for different combinations of signs and symptoms.

**Results:** Latent class analysis of MR findings classified patients into low (class 1, n = 176) and high (class 2, n = 156) likelihood of TMJ synovitis. Condition probabilities of items in the two groups are showed in the table. The classes showed association with specific profiles of TMJ signs and symptoms. A TAN Bayes model predicted low (class 1), high (class 2), and intermediate (class 3) synovitis probability based on signs and symptoms with an AUC of 0,87 for class 2, 0,89 for class 1 and 0,97 for class 3. 9 variables (limitation since less than 3 months, previous TMJ synovitis, retrognathia, TMJ palpation pain, temporalis palpation pain, laterodeviation at visit, limitation for 3-6 months, reduced condylar translation, laterodeviation since < 3 months) showed direct association with class membership. Based on these variables a Bayesian decision analysis (DAN) was conducted, assigning higher rewards respectively to immediate treatment for patients in class 2, non-treatment for class 1 and testing with MR in subjects with intermediate probability (class 3). The DAN resulted in a decision tree that revealed 4 clinical scenarios where immediate treatment was most favorable, 3 where non-treatment was suggested, and 7 where testing with MR was indicated.

**Conclusion:** Our analysis has the potential to inform clinical practice as it can be used to stratify patients according to TMJ synovitis risk. Particularly in limited-resource settings, the model could provide a diffusible and potentially extendable tool to guide the clinical work-up selecting patients who are most likely to benefit from MR testing.

**Disclosure of Interest**

None Declared

**Table 1 (abstract P080).** See text for description.

Indicators	Class 1	Class 2	Wald	P-value	R^2^
Fluid	-0.96	0.96	8.91	0.002	0.05
Contrast enanchment	-2.52	2.52	8.44	0.003	0.48
Condylar erosion	-1.42	1.42	50.57	<000.1	0.36
Bone marrow edema	-3.27	3.27	2.02	0.150	0.10
Reducted translation	-1.27	1.27	46.28	<0.001	0.28
Disc displacement	-0.91	0.91	4.28	0.038	0.03

### P081 RISK FACTORS OF DEVELOPING AUTOIMMUNE THYROID DISEASE IN PATIENTS WITH JUVENILE IDIOPATHIC ARTHRITIS

#### Laurie Baas^1^, Joost Swart^1^, Gabriella Giancane^2^, Gerd Horneff^3^, Michael Hofer^4^, Ekaterina Alexeeva^5^, Violeta Panaviene^6^, Brigitte Bader-Meunier^7^, Jordi Anton^8^, Susan Nielsen^9^, Fabrizio De Benedetti^10^, Sylvia Kamphuis^11^, Valda Stanevica^12^, Maria Trachana^13^, Laura Marinela Ailioaie^14^, Elena Tsitsami^15^, Ariane Klein^16^, Kirsten Minden^17^, Ivan Foeldvari^18^, Johannes-Peter Haas^19^, Jens Klotsche^17^, Alessandro Consolaro^2^, Francesca Bovis^2^, Francesca Bagnasco^2^, Angela Pistorio^2^, Alberto Martini^2^, Nico Wulffraat^1^, Nicolino Ruperto^2^

##### ^1^Wilhelmina Children’s Hospital, Utrecht, Netherlands; ^2^Istituto Giannina Gaslini, Genova, Italy; ^3^Asklepios Klinic Sankt Augustin, Sankt Augustin, Germany; ^4^Lausanne University Hospital, Lausanne, Switzerland; ^5^Federal State Autonomous Institution, Saint-Petersburg, Russian Federation; ^6^Vilnius University, Vilnius, Lithuania; ^7^Université Paris-Descartes, Paris, France; ^8^Hospital Sant Joan de Deu, Barcelona, Spain; ^9^Juliane Marie Centret, Copenhagen, Denmark; ^10^IRCCS Ospedale Pediatrico Bambino Gesù, Rome, Italy; ^11^Sophia Children’s Hospital, Rotterdam, Netherlands; ^12^Bernu Kliniska Universitates Slimnica, Riga, Latvia; ^13^Hippokration General Hospital, Athens, Greece; ^14^“Alexandru I. Cuza” University, Iasi, Romania; ^15^Aghia Sophia Childrens Hospital, Athens, Greece; ^16^Asklepios Klinik Sankt Augustin, Sankt Augustin; ^17^German Rheumatism Research Centre Berlin, Berlin; ^18^Hamburger Zentrum für Kinder- und, Hamburg; ^19^German Center for Pediatric and Adolescent, Garmisch-Partenkirchen, Germany

###### **Correspondence:** Laurie Baas

**Introduction:** Juvenile idiopathic arthritis (JIA) is a chronic inflammatory disease of unknown origin which can be considered as an autoimmune disease.

**Objectives:** The aim of this study was to evaluate the frequency of symptomatic autoimmune thyroid disease (AITD) in children with JIA and to investigate if there are any factors contributing to a higher risk of developing AITD.

**Methods:** 8,309 patients, with a total observation time of 51,324 patient-years, were studied. PharmaChild, the largest JIA-registry was used for this, containing clinical and laboratory data. Patients who were members of the Paediatric Rheumatology INternational Trials Organisation (PRINTO) were enrolled for the registry. Numerous patient characteristics and the family history were calculated separately for the JIA patients with AITD and the patients without AITD.

**Results:** 96 of the 8,309 patients had AITD, which provided a prevalence of 1,2%. The median age of onset was significantly higher in the children with AITD (9,1 versus 7,1). Furthermore, significantly more females (84,4% versus 67,3%), ANA positivity (51,7% versus 40,8%), rheumatoid factor (RF) positive JIA (9,4% versus 3,8%) and psoriatic JIA (7,3% versus 3,4%) were seen in the AITD group. Additionally,  the AITD group had significantly more first degree relative with any autoimmune disease (26,4% versus 13,4%), or more specific with Hashimoto’s thyroiditis (13,2% versus 2,6%). Systemic JIA was significantly less prevalent in the AITD group (3,1% versus 11,1%).

**Conclusion:** This study showed that there is a higher prevalence of AITD among children with JIA than in the general population. Older at diagnosis of JIA, not having systemic JIA, ANA positivity, RF positive JIA and psoriatic JIA seemed to be risk factors, as well as as having a family history of autoimmune diseases, especially first degree relatives or more specific having a family history of Hashimoto’s thyroiditis.

**Disclosure of Interest**

None Declared

### P082 PREDICTING RESPONSE TO METHOTREXATE IN CHILDREN WITH JUVENILE IDIOPATHIC ARTHRITIS: ROLE OF BIOMARKERS

#### Narendra K. Bagri^1^, Subhradip Karmakar^2^, Partha Haldar^3^, Rakesh Lodha^1^, Sushil K. Kabra^1^

##### ^1^Pediatrics; ^2^Biochemistry; ^3^Centre for Community Medicine, ALL INDIA INSTITUTE OF MEDICAL SCIENCES,NEW DELHI, INDIA, New Delhi, India

###### **Correspondence:** Narendra K. Bagri

**Introduction: Introduction**

Approximately 40% of the subjects fail to respond to 1st line therapy including methotrexate and DMARDs, thereby requiring additional therapy. This additional therapy is offered only after a failed trial of methotrexate owing to non-availability of a reliable predictive biomarker. This approach exposes children to ineffective drugs in addition to accrual of ongoing inflammation and joint deformities. Pretreatment estimation of inflammatory biomarkers like myeloid related protein (MRP) 8/14 may predict the response to methotrexate. We are presenting interim analysis of our ongoing study on role of biomarkers in predicting the response to methotrexate in children with JIA.

**Objectives:** To correlate baseline serum biomarkers; IL2, IL6, IL12, TNF a and  MRP 8/14 levels  in children with JIA before starting methotrexate with response to methotrexate as assessed by American college of Rheumatology (ACR) criteria at 3 months follow-up.

**Methods:** Baseline clinical characteristics of consecutive children with JIA were recorded and blood samples for estimation of biomarkers were collected prior to starting methotrexate. Clinical response at 3 months follow up was assessed as per ACR. Patients achieving ACR ≥50 were classified as responders, whereas those with ACR ≤ 30 were classified as Non-responders to methotrexate. The predictive ability of baseline levels of biomarkers for the response to methotrexate was studied using binary logistic regression models.

**Results:** 44 children (21 girls) with JIA who completed 3 months follow up after starting methotrexate were included for this interim analysis. 20 children were classified as responders while 14 were Non-responders. 10 subjects having an ACR between 30 and 50; have been excluded for this analysis. The median baseline values of MRP 8/14 and IL-6 were significantly higher in responders as compared to non-responders (Table 1). Children with higher baseline values of MRP8/14 and IL-6 were more likely to achieve ACR 50 or more; unadjusted OR (95%CI) 1.02 (1,1.04), p=0.047 and 1.28 (0.99,1.63), p=0.05 for MRP 8/14 and IL-6 respectively.

**References**

1. Moncrieffe H, Ursu S, Holzinger D, Patrick F, Kassoumeri L, Wade A, et al. A subgroup of juvenile idiopathic arthritis patients who respond well to methotrexate are identified by the serum biomarker MRP8/14 protein. Rheumatology. 2013 Aug;52(8):1467–76.

2. Singh G, Athreya BH, Fries JF, Goldsmith DP. Measurement of health status in children with juvenile rheumatoid arthritis. Arthritis Rheum. 1994 Dec;37(12):1761–9.

**Conclusion:**

The interim analyses showed that higher levels of baseline serum MRP8/14 and IL-6 have prognostic value in predicting response to methotrexate. Estimating these biomarkers at baseline may help in predicting the therapeutic response and guiding initial therapy.

**Disclosure of Interest**

None Declared

**Table 1 (abstract P082).** Baseline clinical and biomarker levels

Parameter	Responders (n=20)	Non-responders (n=14)	p value*
Age at onset (months), median (IQR)	72(36,132)	87(30,108)	0.84
Mean delay in diagnosis (months), median (IQR)	21(4,59)	30(18,54)	0.326
JIA Subcategory, n			
SJIA	3	0	0.179
Oligoarticular	3	6	
Polyarticular (RF+)	0	1	
Polyarticular (RF-)	6	2	
Entesitis related arthritis	7	5	
Serum MRP 8/14 (pg/ml), median (IQR)	117.7(87.4,155.8)	78.8(59,118)	0.027
Serum IL-6 (pg/ml), median (IQR)	20.3(16.5,23.7)	14(12.2,18.2)	0.019
Serum IL-2 (pg/ml), median (IQR)	126.5(45.9,134.5)	43(31.9,81.9)	0.21
Serum TNFa (pg/ml), median (IQR)	30.7(27.4,32.9)	32.4(37,28.3)	0.43
Serum IL-12 (pg/ml), median (IQR)	48.4(38.3,57.2)	45.5(34,82)	0.83

* The p values are based on Wilcoxon signed-rank test for comparison of median between responders and non-responders

### P083 LEVELS OF IGG ANTIBODIES AGAINST COW’S MILK PROTEINS ARE RELATED TO DISEASE ACTIVITY IN JUVENILE IDIOPATHIC ARTHRITIS (JIA)

#### Anders Öman^1^, Miika Arvonen^2^, Martin Carlsson^3^, Panagiota Christoforaki^1^, Maryam Poorafshar^4^, Lillemor Berntson^1^

##### ^1^Institution of Women´s and Children´s health, Uppsala University, Uppsala, Sweden; ^2^Department of Pediatrics, Kuopio University Hospital, Department of Pediatrics, Oulu University Hospital and University of Oulu, Oulo, Finland; ^3^Department of Pediatrics, Hudiksvall Hospital, Institution of Women´s and Children´s health, Uppsala University; ^4^Thermofisher Scientific, Uppsala, Sweden, Uppsala, Sweden

###### **Correspondence:** Lillemor Berntson

**Introduction:** The role of the gastrointestinal tract in pathogenesis of JIA has raised attention since some of the risk factors for JIA relate to an altered gastrointestinal immunological situation. Early introduction of cow’s milk is one such risk factor. Cow’s milk includes more than 25 different proteins. In bovine milk β-lactoglobulin is the main whey protein, but antibodies against α-lactalbumin are more common in cow´s milk allergy in children. IgG antibodies against cow’s milk antigens are produced in plasma cells in the intestinal canal. The role of these antibodies is unclear, they have been elevated in cow´s milk allergy but not in cow´s milk intolerance. IgG4 antibodies against a specific food antigen mainly relates to the degree of exposition. Antibodies against nutritional antigens in children with JIA are only scarcely studied.

**Objectives:** We wanted to investigate levels of antibodies against cow’s milk antigens in children with JIA, at high compared with low disease activity, and compare the results with antibody levels in healthy controls. We also wanted to evaluate whether a history of milk intolerance affected the results.

**Methods:** We investigated levels of IgG and IgG4 antibodies against β-lactoglobulin, α-lactalbumin and casein in serum of 65 children with different categories of JIA, at high and low disease activity, and in 30 healthy controls. The analyses were performed with an automated FEIA method, ImmunoCAP ThermoFisher. At low disease activity the majority of children were treated with metotrexat and/or biological agents. Disease activity was assessed by analysis of calprotectin by ImmunoCAP in plasma and E-SR. In the group of children reporting milk intolerance, IgE antibodies against milk antigens were analysed with ImmunoCAP.

**Results:** Analyses revealed a significantly higher concentration of IgG antibodies against all three milk antigens in high compared with low disease activity, Md 4.7 mgA/L compared to 3.4 (p=0.001) for IgG β-lactoglobulin, Md 2.2 mgA/L compared to 0.0 (p=0.032) for IgG α-lactalbumin, and Md 7.3 mgA/L compared to 6.9 (p=0.003) for IgG casein. There was no difference between high and low disease activity in concentration of IgG4 β-lactoglobulin. When comparing children in high disease activity with children in the control group, there was no difference regarding levels of β-lactoglobulin (p=0.29), α-lactalbumin (p=0.14) or casein (p=1.0). The levels of β-lactoglobulin in serum correlated with the levels of calprotectin in plasma in all samples at high and low disease activity, r_s_= 0.26 (p=0,004), as well as with E-SR, r_s_= 0.24 (p=0,007). There was a high correlation between E-SR and calprotectin, r_s_= 0.82 (p<0.001). None of the eight children with current or earlier milk intolerance had positive IgE antibodies against milk antigens and the milk intolerance did not influence levels of IgG antibodies against milk antigens.

**Conclusion:** In this study children with high disease activity in JIA had the same degree of antibody-production against milk antigens in serum as healthy children, but the levels were significantly decreased in lower disease activity. The stable concentration of IgG4 β-lactoglobulin was considered a sign of unchanged exposition of cow´s milk at high compared with low disease activity. Our results support influence on the gastrointestinal immune system by disease activity and treatment in JIA.

**Disclosure of Interest**

None Declared

### P084 APPLICABILITY OF THE CASPAR CRITERIA OF PSORIASIC ARTHRITIS IN A COHORT OF CHILDREN PATIENTS FOLLOWING IN A PEDIATRIC RHEUMATOLOGY UNIT OF A TERTIARY HOSPITAL

#### Alina L. Boteanu, Adela Alia Jimenez, Maria Angeles Blazquez Cañamero

##### Rheumatology, University Hospital RAMÓN Y CAJAL, MADRID, Spain

###### **Correspondence:** Alina L. Boteanu

**Introduction:** The ILAR consensus establishes classification criteria, dividing the JIA into 7 subcategories, with juvenile psoriatic arthritis (APsJ) being one of them. In the adult population, the CASPAR classification criteria are usually used to classify a patient with psoriatic arthritis. However, the two classifications have some differences that sometimes produce confusion

**Objectives:** To assess the applicability of the CASPAR classification criteria in a series of patients previously diagnosed in pediatric age of JPAs or undifferentiated arthritis by exclusion criteria to be male> 6 years old and HLA B27 positive, comparing these with the ILAR classification criteria, through the study of clinical features

**Methods:** Retrospective cross-sectional observational study. Clinical, epidemiological, sociodemographic and analytical variables were collected from 30 patients previously diagnosed with JPAs (6 years in HLA B27-carrying male. It was assessed whether the patients met the ILAR classification criteria as well as the CASPAR classification criteria, which, unlike the previous ones, did not exclude HLA B27 positive patients, considered the family history of the 2nd degree and added a test radiographic

**Results:** The mean age at diagnosis was 11.23 ± 4.6 years; 15 of them being women and 15 men. 15 (15/30) patients presented cutaneous psoriasis at some point during the follow-up, in 5/15 patients psoriasis began before arthritis while 7/15 patients were previously diagnosed with arthritis than cutaneous psoriasis; in 3/15 patients the diagnosis was simultaneous during the medical visit. 9 (9/30) patients presented a family history of 1st degree cutaneous psoriasis and 7/30 of them had a family history of 2nd grade psoriasis. Of the total number of patients, 10 of them would not meet the ILAR classification criteria, 8 because they presented as exclusion criteria being male, HLA-B27 positive and> 6 years of age, among which, 7/8 would fulfill CASPAR criteria, and 2 other patients who were not classified according to ILAR criteria, did meet the CASPAR classification criteria, given the presence in these criteria of negative RF, family history of the 2nd degree and typical radiological alterations, which are not present in the ILAR criteria. 1 (1/30) patient did not meet CASPAR criteria, and belonged to the group of patients excluded from the ILAR criteria for being male> 6 years HLA-B27 +. If we did not take into account the negative FR of the CASPAR criteria, 14 patients would not meet these criteria and if we eliminated the 2nd grade AF, 5 patients would not be classified (among them 2 who meet CASPAR and do not ILAR)

**Conclusion:** In our series of patients despite the fact that the presence of current skin psoriasis contributes 2 points in the CASPAR criteria, only 1 patient would not meet the CASPAR criteria, since the majority of patients present other clinical or analytical manifestations, such as the presence of negative rheumatoid factor or 2nd degree family history. Patients who do not meet criteria for PsA by exclusion criteria, practically all of them would be diagnosed with psoriasic arthritis by CASPAR criteria.

**Disclosure of Interest**

None Declared

### P085 METHOTREXATE RESPONSE IN JUVENIL IDIOPATHIC ARTHRITIS

#### Nilgun Cakar^1^, Ayten Guliyeva^1^, Elif Çelikel^1^, Fatma Aydın^1^, Beyza Doganay^2^, Z. Birsin Ozcakar^1^, Fatos Yalcinkaya^1^

##### ^1^Department of Pediatric Rheumatology; ^2^Biostatistics, Ankara University School of Medicine, Ankara, Turkey

###### **Correspondence:** Nilgun Cakar

**Introduction:** Methotrexate (MTX) is a second line drug in the treatment of juvenile idiopathic arthritis (JIA), either alone or in combination with biologic agents. However, there is variation in the clinical response to MTX.

**Objectives:** The aim of this study was to investigate the role of factors such as age, gender, ANA status, number of active joints, and JADAS-10 in predicting MTX response.

**Methods:** Clinical and laboratory data of oligoarticular and polyarticular JIA patients diagnosed according to the International League of Association for Rheumatology (ILAR) criteria, who had received MTX treatment between January 2012 and March 2017, and continued MTX treatment for at least 12 months were evaluated retrospectively. The age at diagnosis, gender and ANA status were recorded. The number of active and tender joint and levels of acute phase reactants were assessed at baseline, and 3, 6, and 12 months. Disease activity score (JADAS-10) was calculated at onset of MTX and at months 3, 6 and 12. Patients' responses to MTX treatment were assessed according to the American College of Rheumatology Paediatric (PedACR) improvement criteria. At each time the patients were divided into responders and non-responders according to PedACR 30 and 70

**Results:** This study included 55 JIA patients (40 female). Thirt-four were oligoarticular (2 extended oligoarticular) and 21 were polyarticular (5 rheumatoid factor positive) JIA. The mean age at onset of the disease was 7.71± 4.83 years.(mean age was 6.75 ± x4.99 years in patients with oligoarthritis, 9.27 ± 4.21 years in polyarticular patients). The median JADAS 10 at beginning for all 55 children was 19 (10–40), and 16 (10-27)  for oligoarticular, and 28 (19-40) for polyarticular JIA. At month 3, 45% of all patients, %41 of oligoarticular, and 52% of polyarticular JIA patients had a minimum PedACR 30 response. 13 patients (2 at 4^th^ and, 11 at 7^th^ months) received biologic agents along with MTX. These patients were not included in the evaluation of treatment responses at 12^th^ month. The remaining 42 patients had PedACR 30 response at month 12. Moreover 81% of these patients (% oligoarticular, % polyarticular JIA) reached pedACR 70 response at month 12. Patients who had PedACR 30 response at 3 months had a higher rate of reaching the Ped ACR 70 response at 12 months (p=0.009). In patients with oligoarticular JIA, the 12^th^ month Ped ACR response was negatively correlated with the number of involved joints (OR). The initial JADAS 10 did not effect the PedACR response at 3 months (OR), but it was found that the 6^th^ month of JADAS 10, had a significant effect on PedACR 70 response at 12^th^ month (p<0.05). Age, gender and ANA status had no effect on PedACR responses.

**Conclusion:** PedACR 30 response at the 3^rd^ month, JADAS-10 at 6^th^ month and number of active joints in oligoarticular JIA were found to be predictors of the ACR response at 12^th^ month.

**Disclosure of Interest**

None Declared

### P086 CHARACTERIZATION OF PATIENTS WITH POLYARTICULAR IDIOPATHIC JUVENILE ARTHRITIS AND INFILTRATIVE LUNG DISEASE

#### Bilade Cherqaoui^1^, Brigitte Bader-Meunier^2^, Christophe Delacourt^3^, Isabelle Kone-Paut^1^, Alice Hadchouel-Duverge^3^

##### ^1^Pediatric Rheumatology, CHU Bicetre, APHP - CEREMAIA, LE KREMLIN BICETRE; ^2^Pediatric Immunology & Rheumatology, Necker Hospital, APHP - RAISE; ^3^Pediatric Pneumology, Necker Hospital, APHP - RESPIRARE, Paris, France

###### **Correspondence:** Bilade Cherqaoui

**Introduction:** There is an association between juvenile idiopathic arthritis (JIA) and chronic lung disease. These pulmonary disorders are multiple - interstitial (ILD) or non-inflammatory - depending on the type of underlying JIA - mainly polyarticular or systemic. In children, because of absence of accurate and multi-center data, the management of these chronic lung diseases remain difficult.

**Objectives:** This work aimed to constitute a national cohort of JIA patients presenting chronic lung disease.

**Methods:** A retrospective multicenter study was conducted. The cases between 2007 and 2018 were obtained from appeal of French Medical societies (SOFREMIP, *RespiRare*, SFR, CRI, database of pediatric pulmonary arterial hypertension). JIA was defined according to ILAR criteria; all had to have chronic pulmonary disease. Exclusion criteria were: age at onset> 18 years old, systemic JIA, other documented auto-immune or auto-inflammatory disease, asthma, cystic fibrosis, bronchopulmonary dysplasia, chronic lung infection, pulmonary primitive malformation. Data regarding clinical, paraclinical, therapeutic and evolution items were collected.

**Results:** 8 files were selected; 4 met the study criteria. All patients had positive rheumatoid factor polyarticular JIA, with female predominance (3/4), mean age at rheumatism onset of 11.5 years (7.5-16). They all had disabling polyarthritis, involving small/big articulations, mostly symmetrical, involving neck in 3/4 and hips in 1/4 patients, X-ray erosions in 2/4 patients. All had markedly erythrocyte sedimentation rate (32-107 mm, mean=65), hypergammaglobulinemia (14.8-30.8 g/l, mean=22.5), high titers of CCP antibodies, elevated antinuclear antibodies (>1/320) - with specificities in patient 4 (SSA/SSB/RNP without criteria for any connective tissue disease); patient 2 had positive TRAK antibodies without functional consequences. Each patients had different subtypes of ILD (Table 1). Pulmonary symptoms at diagnosis were: cough (2/4), exercise dyspnea (2/4), dyspnea at rest (1/4), hemoptysis (1/4 – patient 4), digital clubbing (1/4 – patient 2), auscultation abnormalities (2/4). In patient 2, ILD/fibrosis was related to *SFTPC* mutation. She had familial history of dominant inheritance of lung fibrosis and 2 ascendants with inflammatory rheumatic disease (rheumatoid arthritis, unclassified). Treatment was based on oral steroids, that could be associated with variable immunomodulation. In contrast to methotrexate - used in all patients, Etanercept was associated in patient 2 with ILD worsening, that improved at treatment discontinuation. Among all patients at last visit, patient 2 was the only who needed nutritive and respiratory support, and died at age of 15 years old, 13 years after lung disease beginning, on a graft waiting list.

**Conclusion:** The chronic pulmonary diseases related to JIA are extremely scarce. In this cohort only ILD form was observed in positive rheumatoid factor polyarticular JIA patients. Treatment strategies of both combined diseases were heterogeneous: oral corticosteroid was used in all, methotrexate did not seem to aggravate ILD, conversely to etanercept. The long-term interest will be to propose consensual recommendations for management of these patients. Informed consent had been obtained from the parents.

**Disclosure of Interest**

None Declared

**Table 1 (abstract P086).** See text for description

Patient / Sex	1 / F	2 / F	3 / M	4 / F
Lung disease	Bronchiolitis Obliterans with Organizing Pneumonia	Surfactant Protein C-associated ILD	Non-specific interstitial pneumonia	Chronic bronchiectasis
Age at ILD onset (y)	16,9	2	14,4	10,9
Chest X-ray and scanner abnormalities	Yes	Yes	Yes	Yes
Diffusion trouble	Yes	Yes	Yes	No
Restrictive syndrome	No	No	No	No
Obctructive syndrome	No	Yes	No	No
Inflammatory alveolar liquid	No	Yes		No
Biologics	Ritux	Eta	Eta, Toci, Aba, Inflix	No
IV steroids	Yes	Yes	No	No

### P087 “ONCE A DAY, ‘CAUSE YOU’RE GETTING LOTS OF INFORMATION”: HOW CHILDREN AND YOUNG PEOPLE WITH JUVENILE IDIOPATHIC ARTHRITIS WANT TO REPORT PAIN

#### Rebecca R. Lee^1^, Amir Rashid^1^, Wendy Thomson^2^, Lis Cordingley^3^

##### ^1^NIHR Manchester Musculoskeletal Biomedical Research Centre, Arthritis Research UK Centre for Epidemiology, Division of Musculoskeletal and Dermatological Sciences; ^2^NIHR Manchester Musculoskeletal Biomedical Research Centre, Arthritis Research UK Centre for Genetics and Genomics, Division of Musculoskeletal and Dermatological Sciences; ^3^Centre for Musculoskeletal Research, Division of Musculoskeletal and Dermatological Sciences, University of Manchester, Manchester, UK

###### **Correspondence:** Lis Cordingley

**Introduction:** Digital, remote, mobile health (mHealth) pain assessments are increasingly used in the research and care of children and young people (CYP) with long-term conditions, such as Juvenile Idiopathic Arthritis (JIA). However, little is currently known about the influence, impact or patient preference for particular ecological momentary assessment (EMA) techniques in pain reporting.

**Objectives:** To explore patient preferences of different pain assessment timing and frequencies in CYP with JIA remotely monitoring pain at home.

**Methods:** The study was a randomised n-of-1 crossover trial. Participants aged between 5 and 16 years of age were recruited from The Childhood Arthritis Prospective Study, UK. Participants were loaned an iPad for the duration of the study. The iPads were uploaded with *My Pain Tracker (MPT),* a valid and acceptable multi-dimensional pain assessment application for CYP with JIA. *MPT* collects information about pain intensity, severity, location, qualities and interference and can be used by CYP to track their pain independently. Participants were randomised into one of four groups. Each group followed different administrative patterns of pain assessment reporting. Four different timing schedules (once-a-week, once-a-day, twice-a-day and as-and-when pain occurred) were randomised within the groups to occur twice within an eight-week data collection period. Semi-structured interviews were conducted over the telephone at the end of the study to explore which timing schedules participants and their parents preferred and why. Questions about subjective measurement reactivity (whether participants or parents noticed a change in pain or emotion in response to particular pain reporting schedules) were also included.

**Results:** Fourteen CYP took part in the study (*M*=11.93, *SD*=2.65). An equal number of CYP reported that they preferred once-a-day and as-and-when reporting schedules (42.9%). As-and-when reporting facilitated flexibility in pain tracking. Some participants considered this an advantage and others, a burden. Flexibility was problematic because participants did not know what level of pain was sufficient to report or how to report when they were in school. Furthermore, CYP were not able to record and review ‘good’ or pain free days with this schedule, which CYP found valuable for evaluating the contrast with ‘bad’ pain days. Creating habits or routines was one of the most important factors for successfully remembering to report pain. A priority for CYP was capturing comprehensive information about recent pain which they could still remember. The once-a-day schedule accommodated these advantages. Higher frequency of pain reporting (more than once-a-day) was perceived to affect mood and fatigue by some participants and their parents. One parent reported a perceived physical change in their child’s pain as a response to more intense pain reporting schedules. The majority of participants and their parents did not report any subjective measurement reactivity, with some suggesting frequent assessment served as a distraction from pain.

**Conclusion:** To our knowledge, this is the first study to investigate the impact of different EMA schedules with CYP with JIA and persistent pain. Findings suggest that daily reporting of pain using multi-dimensional mHealth assessment tools is feasible, sustainable and a reporting preference for CYP with JIA. Findings are important for the development of administrative guidelines for new mHealth pain assessment tools.

**Disclosure of Interest**

None Declared

### P088 ANTIBODIES AGAINST CITRULLINATED PEPTIDE PROTEINS (CCP, MCV AND CYCLIC MCV) IN JUVENILE IDIOPATHIC ARTHRITIS

#### Talia Diaz-Prieto^1^, Ana Villarreal-Treviño^1^, Rocío Maldonado-Velázquez^1^, Enrique Faugier-Fuentes^1^, Andrés Rodríguez-García^1^, Carlos Núñez-Álvarez^2^

##### ^1^Pediatric Rheumatology, Hospital Infantil De México Federico Gómez; ^2^Laboratory of Immunology and Rheumatology, Instituto Nacional De Ciencias Médicas Y Nutrición Salvador Zubirán, Mexico City, Mexico

###### **Correspondence:** Talia Diaz-Prieto

**Introduction:** Juvenile Idiopathic Arthritis (JIA), is the most frequent rheumatic disease in Pediatrics. Within the JIA, there are different subtypes, better prognosis in oligoarticular JIA, but not in JIA polyarticular rheumatoid factor (FR) positive, recent treatments allow patients to be diagnosed and treated as soon as possible to prevent joint damage. It would be useful to have biomarkers for both diagnosis and prognosis. Citrulline vimentin was recognized as a target within the family of citrullinated proteins. The presence of mutated citrullinated vimentin antibodies in JIA has been little studied.

**Objectives:** To determine levels of anti-citrullinated cyclic peptide, citrullinated anti-vimentin and mutated citrullinated vimentin in patients with JIA. Describe clinical and epidemiological characteristics.

**Methods:** Cross-sectional study, patients with JIA from Pediatric Rheumatology of the HIMFG, June 2017. Informed consent, 1ml of blood serum. ELISA technique in INCMNSZ.

**Results:** Total of 62 patients (n = 62), female n = 46. Anti-CCP cut-off value of 9.1U / ml, sensitivity of 71% and specificity of 97.8%. Anti-VMCc value cut 18 U / ml, sensitivity 62.9% and specificity of 88.4%, Anti-VMC value cut 18.7 U / ml, sensitivity 56.5% and specificity of 86.96%, U of Mann-Whitney anti-CMVc patients vs healthy controls p0.5976, anti-CCP patients vs healthy controls p0.5220, anti-VCM patients vs healthy controls p0.003. 12 patients were reported with anti-CCP negative and positive for anti-VMC.

**Conclusion:** A significant difference was observed in anti-MCV levels compared to healthy controls. The anti-VMC could be supportive in the diagnosis of Juvenile Idiopathic Arthritis.

**Disclosure of Interest**

None Declared

### P089 ROLE OF ANTI-CCP, HSCRP AND MUSCULOSKELETAL ULTRASOUND IN DETECTION OF DISEASE ACTIVITY AND SEVERITY OF JUVENILE IDIOPATHIC ARTHRITIS

#### Mervat Eissa^1^, Mona M. Elmetwally^2^, Aldosoki A. Fouda^2^, Mohammed T. M. Abdelhak^3^, Samia A.-H. Abdel-Mageed^2^

##### ^1^Department of rheumatology and rehabilitation, Cairo University; ^2^Department of rheumatology and rehabilitation; ^3^Department of Radiology, Al Azhar University, Cairo, Egypt

###### **Correspondece:** Mervat Eissa

**Introduction:** Juvenile idiopathic arthritis is a common systemic autoimmune inflammatory disease. Anti-CCP is important biomarker to differentiate those patients with aggressive JIA. hs CRP could identify patients in clinical remission and their risk of disease relapse. Ultrasound on the joints is more sensitive in the detection of disease activity than clinical examination alone. The aim: evaluate the role of anti-CCP 2and hs CRP, MSUS in early detection of activity of JIA.

**Objectives:** Evaluate the role of anti-CCP 2 and hs CRP, MSUS, and Doppler study in early detection of activity of JIA children

**Methods:** Assessment of the disease activity of JIA was done using the Juvenile Arthritis Disease Activity Score (JADAS27). Conventional radiography, musculoskeletal US examination in a minimum of two planes (longitudinal and transverse) and Global ultrasound score were done for all 60 patients. Blood tests included (IgM RF was assayed using quantitative immunonephelometry test, hs CRP and Anti-CCP 2 (IgG) was measured by commercially available second-generation ELISA kits DiastatTM Axis, Shield Diagnostics, Dundee, Scotland) were done for all patients and 20 healthy control.

**Results:** 60 JIA patients; (41.66%) males and (58.33%) females. Their mean age was 7.9. Their mean disease duration 3.5. They were divided according to disease activity into 2 groups: group 1: 30 patients with JADAS27 score <3.8, group 2: 30 JIA patients of high disease activity according to JADAS27 score ≥3.8 There was a highly significant positive correlation between ESR, CRP, hs CRP and JADAS27. On the other hand, there was a non-significant positive correlation between anti-CCP levels and JADAS27. Global ultrasound score had a highly significant positive correlation with JADAS27 P < 0.001. Global ultrasound score had a significant positive correlation with hs CRP. Diagnostic performance of different parameters in discrimination of disease activity of group I and group II ROC curve, hs CRP was more sensitive in early detection of disease activity followed by ultrasound score while anti-CCP and routine CRP showed lower global sensitivity

**Conclusion:** hs CRP, MSUS Doppler are important tools in detection of activity of JIA children.

**Disclosure of Interest**

None Declared

### P090 JUVENILE IDIOPATHIC ARTHRITIS AND THE TEMPOROMANDIBULAR JOINT:PREVALENCE, CLINICAL FACTORS AND DIAGNOSTIC TOOLS

#### Lina von Schuckmann^1^, Ivan Foeldvari^1^, Anna Suling^2^, Jens Klotsche^3^, Bärbel Kahl-Nieke^4^

##### ^1^Hamburg Center for Pediatric and Adolescent Rheumatology, Am Schoen Klinik Eilbek; ^2^Department of Medical Biometry and Epidemiology, University Medical Centre Hamburg-Eppendorf, Hamburg; ^3^German Rheumatism Research Centre, Berlin; ^4^Department of Orthodontics, University Medical Centre Hamburg-Eppendorf, Hamburg, Germany

###### **Correspondence:** Ivan Foeldvari

**Introduction:** Temporomandibular joint (TMJ) involvement occurs around 30 to 80 % in children with Juvenile idiopathic Arthritis (JIA). The correlation with certain subtypes with hypothesized. There are several studies looeked at predictors of TMJ involvement. We reviewed in a monocentric study a large patient population to asses the prevalence in the different subtypes and prognostic markers for the TMJ involvement.

**Objectives:** To study the prevalence of TMJ involvement, as well as associated clinical signs, symptoms and factors in children with JIA.

**Methods:** We performed a retrospective chart review on 3,999 consecutive patients who were followed in the Hamburg Centre for Paediatric and Adolescent Rheumatology Eilbek between January 2010 and July 2012. Demographic, clinical, laboratory and radiographic data were collected. TMJ involvement was diagnosed clinically by the attending rheumatologist, or radiologically by MRI.

**Results:** 35 % of 2,413 included patients had TMJ involvement. In a multivariable analysis we found female sex (p = 0.021), increasing number of joints with active arthritis (p < 0.001) and antinuclear antibody (ANA) positivity (p = 0.023) were significantly associated with TMJ involvement. In the oligoarticular group (persistent and extended) was a positive association of TMJ involvement and age (OR = 1.07, p < 0.001), while this association was negative in the enthesitis related arthritis group (OR = 0.94, p = 0.015). A significant correlation revealed between pathological MRI results and laterotrusion of the chin (p = 0.012) and pain on palpation (p = 0.020).

**Conclusion:** Based upon our data we cannot recommend one unique clinical parameter to diagnose TMJ involvement, and MRI is still the gold standard to assure the diagnosis. Female sex, ANA positivity and disease activity were correlated significantly with an increased OR for TMJ involvement, and a significant interaction between age and JIA categories (oligoarticular and enthesistis related arthritis) was found. Further prospective controlled studies are still needed.

**Disclosure of Interest**

None Declared

### P091 VALIDATING AND DEVELOPING A SELECTED QUESTIONNAIRE TO PREDICT EARLY DIAGNOSIS OF JUVENILE IDIOPATHIC ARTHRITIS IN GERMAN POPULATION

#### Tristan Scheer^1^, Jens Klotsche^2^, Claudio Len^3^, Ivan Foeldvari^4^

##### ^1^Asklepius campus Semmelweiss University Budapest, Hamburg; ^2^German Rheumatism Research Center, Berlin, Germany; ^3^Pediatric Rheumatology Unit, São Paulo Federal University, São Paulo, Brazil; ^4^Hamburg Center for Pediatric and Adolescent Rheumatology, Hamburg, Germany

###### **Correspondence:** Ivan Foeldvari

**Introduction:** Juvenile idiopathic arthritis (JIA) is the most common chronic inflammatory rheumatologic disease in children and adolescents with a prevalence of 1 in 1000 children in Germany. An early referral of children suspected to have JIA to a pediatric rheumatologist is essential for an early diagnosis and starting treatment in the therapeutic window to reach a better outcome. An easy-to-use and time efficient questionnaire originally developed by a Brazilian study group led by *Claudio Len* may detect children and adolescent under risk for JIA.

**Objectives:** To determine, whether the German version of the screening tool is reliable for the early diagnose of JIA and secondly, whether a weighting scheme of individual questions improves its diagnostic performance.

**Methods:** The original questionnaire was translated into German by *Dr. med Ivan Foeldvari.* The questionnaires were distributed among patients at the first clinical visit at the Hamburger Centre für Kinder- und Jugendrheumatologie. The questionnaire includes 12 disease-orientated questions each with three possible responses, either: “Yes”, “No” or “I don’t know”. A retrospective evaluation of patients diagnosed with JIA or with a non-inflammatory joint pain (NJP) was performed later on. The sample consisted of patients seen between August 2015 and July 2017. All patients were at least older than 2 years and younger than 17 years at the time of evaluation. Only questionnaires, which were fully answered, were evaluated. Subsequently, a weighting scheme (based on the factor loadings from a confirmatory factor analysis) of individual questions was applied in order to increase the sensitivity of the tool. Standard statistical techniques were used to find associations between the sum scores and clinical characteristics and to compare the weighted and non-weighted sum scores.

**Results:** In total 165 of 800 questionnaires could be evaluated for the study. 133 (81%) were diagnosed with JIA and 32 (19%) with NJP. The group of JIA patients consisted of 79 (59%) girls, whereas the control group consisted of 21 (66%) girls. The analysis of the individual questions was performed by comparing the rate of a positive response to the questions (“Yes”) between the two groups. Four questions showed a highly significant difference by comparing answers of the control group with a subgroup of JIA patients having at least one active inflammatory joint. In particular, questions regarding physical constraints (p= 0.20; AUC= 0.62), joint pain (p= 0.040; AUC = 0.61), swelling in the joints (p= 0.040; AUC =0.60) and stamina (p= 0.047; AUC = 0.60) seem to be relevant in the diagnostic approach. A weight of 3 was assigned to the first and seventh, 2.5 to the fourth and sixth, and 1.5 to the third and fifth item. The diagnostic accuracy of the respective weighted sum score increased from 64% to 68% to discriminate between patients with JIA with at least one active joint and the control group in comparison to the ordinary sum score. An optimal cutoff of 6.0 for referral to a pediatric specialist was calculated.

**Conclusion:** The validation of the questionnaire showed a discriminative difference in patients with clinical diagnosed JIA and a control group diagnosed with NJP. Furthermore, the weighted sum score performed better to differentiate between JIA and NJP patients. The modified questionnaire can be useful to screen for JIA and speed up the referral to a pediatric rheumatologist.

**Disclosure of Interest**

None Declared

**Table 1 (abstract P091).** ROC-Analysis of the questionnaire in different groups

	Sum Score		Weighted Sum Score	
	AUC	95% CI	AUC	95% CI
JIA vs. musculoskeletal pain	0.59	0.49; 0.70	0.63	0.53; 0.74
JIA with at least one active joint vs. musculoskeletal pain	0.64	0.53; 0.75	0.68	0.57; 0.79

### P092 METHYLENETETRAHYDROFOLATE REDUCTASE 677 NUCLEOTIDE MUTATION IS A PREDICTIVE TOOL IN METHOTREXATE NON-RESPONSIVE JIA PATIENTS

#### Vladimir Iacomi, Ninel Revenco

##### Rheumatology, Mother and Child Institute I.M.S.P., Chisinau, Moldova, Republic of

###### **Correspondence:** Vladimir Iacomi

**Introduction:** One of the aim of the international child arthritis associations is to provide proper treatment regimens in children with juvenile idiopathic arthritis (JIA) with proving effectiveness and safety. Methylenetetrahydrofolate reductase (MTHFR) mutations, according to Genome-Wide Association Studies (GWAS) are being involved in most toxicities. The effective response to methotrexate (MTX) treatment in JIA is remaining contradictory.

**Objectives:** To assess the MTHFR gene mutations in children with JIA, treated with MTX for more than 6 months, and correlate them with the Juvenile Arthritis Disease Activity Score (JADAS71) and Pediatric ACR 20,50,70,90 Index.

**Methods:** There has been initiated a PhD observational case-control study, involving at least 24 patients using MTX for JIA treatment. All data is processed through IBM SPSS Statistics 22 programe.

**Results:** There has been examined 18 children yet, including 7 (38,9%) cases of no mutation, 2 (11,1%) cases of T/T homozygotes and 9 (50%) cases of C/T heterozygotes in the 677 nucleotide of the MTHFR gene. The JADAS71 score was higher in the heterozygote cases with the mean value 18,1(p=0,0013), compared to the non-mutation sample - 2,4 (p=0,0022). The Pediatric ACR index in heterozygote sample had a mean value of 22% (p=0,0011) improvement compared to the control group - 37% (p=0,001).

**Conclusion:** There has been determined a significant correlation between the MTHFR gene heterozygote mutation status and the MTX non-responsiveness. The MTHFR mutation screening is an available tool in assessing immunosuppressive therapy failure.

**Disclosure of Interest**

None Declared

### P093 ‘REAL-LIFE’ USE OF MRP8/14 SERUM LEVEL MEASUREMENT IN CLINICAL PRACTICE AS A PREDICTOR OF OUTCOME AFTER STOPPING METHOTREXATE IN PATIENTS WITH JUVENILE IDIOPATHIC ARTHRITIS

#### Beverley Almeida^1,2^, Emma J. Jordan^2^, Jason Palman^2^, Peter Bale^1^, Elizabeth Ralph^1^, Clare Heard^2^, Emily Robinson^2^, Simona Ursu^2^, Kimberly Gilmour^1^, Lucy R. Wedderburn^1,2,3,4^

##### ^1^Great Ormond Street Hospital for Children NHS Trust, London; ^2^University College London Great Ormond Street Institute Of Child Health; ^3^ARUK Centre for Adolescent Rheumatology; ^4^NIHR BRC at Great Ormond Street Hospital, London, UK

###### **Correspondence:** Emma J. Jordan

**Introduction:** Many patients with JIA who reach clinical remission on treatment harbour residual inflammation that is not detectable by standard measures. MTX remains the first line DMARD for JIA, but is frequently poorly tolerated due to gastrointestinal side effects. After stopping MTX there is a significant risk of disease flare. Myeloid related protein (MRP) 8/14 has been shown as a marker of subclinical disease activity and a predictor of patients with risk of disease flare after stopping MTX.

**Objectives:** This study reviewed the impact of measuring serum MRP8/14 concentration in clinical practice, to identify patients who have reduced risk of disease flare if they stop MTX therapy.

**Methods:** MRP8/14 tests performed at a single paediatric centre using a commercially available assay (Bühlmann) were analysed. Patients included all subtypes of JIA, treated by MTX monotherapy at time of test and had not previously been on a biologic or other disease-modifying drug. All were considering stopping MTX. Clinically inactive disease (CID) was defined by Wallace criteria. JIA subtype, gender, age at diagnosis, disease duration, age at sample and disease activity variables were recorded at time of test and over time. Disease flare in those off MTX was defined as a patient that no longer met Wallace criteria and required new intervention. Patients who met the inclusion criteria were divided into those who did or did not meet Wallace criteria, and whether they stopped, weaned or did not stop MTX. Statistical analyses were performed using Prism Graphpad, Version 7.

**Results:** A total of 72 serum MRP8/14 concentrations from 72 patients were analysed. In patients who met Wallace criteria, comparison of serum MRP8/14 concentration values in those who stopped versus those who didn’t stop MTX, demonstrated statistically significant difference (Mann-Whitney test). In the group who stopped MTX (n=18), sensitivity (high MRP8/14 indicating flare) was 86%, specificity (low MRP8/14 indicating no flare) was 18% and the positive predictive value (percentage of patients with a high MRP8/14 result who went on to flare) was 40%. Flares in patients who met Wallace criteria and stopped MTX treatment were plotted over time, split by the MRP8/14 result; low (<4000ng/ml), n=6 and high (>4000ng/ml), n=1. Statistical analysis showed no significant difference between groups, likely due to small numbers in the analysis, however the trend matched previous data.

**Conclusion:** Measurement of serum MRP8/14 can be used in clinical practice to assist therapy decisions and as a predictor of flare post stopping MTX therapy in JIA patients who have achieved CID. However, biomarkers providing greater predictive power are needed to enable more accurate tailoring of treatment.

**Disclosure of Interest**

None Declared

### P094 WHICH ONE IS MORE EFFECTIVE ON FUNCTION AND PSYCHOSOCIAL STATUS IN JIA?: NUMBER OF JOINTS INVOLVED OR PRESENCE OF PAIN

#### Nur Banu Karaca^1^, Hafize Emine Sönmez^2^, Gamze Arın^1^, Erdal Sağ^2^, Aykut Özçadırcı^1^, Selcan Demir^2^, Fatma Birgül Oflaz^1^, Yelda Bilginer^2^, Edibe Ünal^1^, Seza Özen^2^

##### ^1^Department of Physiotherapy and Rehabilitation, Hacettepe University Faculty of Health Sciences; ^2^Department of Pediatric Rheumatology, Hacettepe University Faculty of Medicine, Ankara, Turkey

###### **Correspondence:** Nur Banu Karaca

**Introduction:** Juvenile idiopathic arthritis is a chronic inflammatory childhood disease with symptoms such as joint inflammation, pain, loss of quality of life. The number of joints and presence of pain cause the child to be affected psychosocially as well as affecting functional activity.

**Objectives:** The aim of this study is to examine the results of functional and psychosocial status according to the type of joint involvement and the presence of pain symptoms in children with JIA.

**Methods:** The study included 71 children diagnosed with JIA who applied to Hacettepe University İhsan Doğramacı Children's Hospital Rheumatology Department. After demographic information of children was collected, functional and psychosocial status was assessed with the scale which was developed in Hacettepe University for children with rheumatism and functional status assessed with Child Health Assessment Questionnaire (CHAQ). Children were divided into groups according to joint involvement as oligoarthritis or polyarthritis and presence or absence of pain. Mann-Whitney U test was used to compare the data.

**Results:** Mean age and numbers of children were shown in Table 1. There were no difference between groups, according to disease type (p>0,05). On the other hand, comparing the scores according to pain presence, CHAQ total, CHAQ general VAS assessment, functional and psychosocial status were significantly different (p<0,05).

**Conclusion:** It is concluded that pain is an important determinative on functional, psychosocial and overall disease assessment comparing to number of joints involved. It can be implicated that affected joint number may be compansatible by muscle activation but pain may require a central organisation to be alleviated. Thus, it must be taken into consideration that he child’s ability to cope with pain should be improved.

**Disclosure of Interest**

None Declared

**Table 1 (abstract P094).** See text for description

	Type		p	Pain		p
	Oligoarthritis (n=51)	Polyarthritis (n=20)		Present (n=21)	Absent (n=50)	
Age (years)	10,88±3,81	13,50±3,92	0,016	11,71±3,77	11,58±4,12	0,885
CHAQ Total	0,28±0,29	0,46±0,41	0,127	0,51±0,4	0,26±0,27	**0,012**
CHAQ (General VAS)	2,49±2,43	3,93±3	0,068	4,46±2,86	2,24±2,3	**0,002**
Function	4,72±4,85	5,05±6,32	0,766	7,85±6,6	3,54±4,02	**0,004**
Psychosocial	23±5,67	14,5±5,82	0,363	16±5,74	12,34±5,4	**0,012**

### P095 EXPERIENCES OF PARTICIPATION IN ACTIVITIES AMONG CHILDREN WITH JUVENILE IDIOPATHIC ARTHRITIS

#### Johanna T. Kembe

##### ^1^Function Area Occupational Therapy & Physiotherapy, Karolinska University Hospital, Occupational Therapy, Stockholm, Sweden

**Introduction:** Children with JIA are affected in participation and according to Unicef every child is entitled to daily life participation. Through participating in activities children develop abilities that form upbringing and adult life. Children with JIA have effects on participation that is caused by the disease.

**Objectives:** The aim of the study was to describe experiences of participation in activities among children with JIA.

**Methods:** A qualitative design with an interview method where eight girls in ages 12-15 have been interviewed with semi-structured interviews. Data handling has been executed according to a content analysis.

**Results:** Based on collected data the theme *Participation influences through interaction between the individual and the surroundings* with corresponding categories *symptoms’ immediate effect, physical environment, strategies, personal treatment, relations* and *the individual’s inner life* were formed. Outcome shows that children with JIA experience participation in every day life is influenced by the disease symptoms, physical environment, social environment and the individual herself. Experiences of increased participation were affected by the individual’s choice of strategies, and social environment.

**Conclusion:** Children with JIA experienced that participation in activities was influenced by disease symptoms, which resulted in absence, and excluding participation. The social environment could increase participation through good treatment and understanding, but the social environment could also decrease participation through misunderstanding and misinterpretation about the disease. The individual’s attitude using strategies and adjustments during activities led to increased participation, and those that chose not to, experienced a decreased participation.

**Disclosure of Interest**

None Declared

### P096 PREDICTOR FACTORS OF Q ANGLE IN PATIENTS WITH JUVENILE IDIOPATHIC ARTHRITIS AND HEALTHY CHILDREN

#### Eylul P. Kisa^1^, Serap Inal^2^, Ela Tarakcı^3^, Nilay Arman^4^, Ozgur Kasapcopur^5^

##### ^1^Department of Physiotherapy and Rehabilitation, Faculty of Health Science, Istanbul Aydin University; ^2^Department of Physiotherapy and Rehabilitation, Faculty of Health Science, Istanbul Bahcesehir University; ^3^Department of Neurological Physiotherapy and Rehabilitation; ^4^Department of Physiotherapy and Rehabilitation, Faculty of Health Science, Istanbul University; ^5^Department of Pediatric Rheumatology, Medical Faculty of Cerrahpasa, Istanbul University, Istanbul, Turkey

###### **Correspondence:** Eylul P. Kisa

**Introduction:** Juvenile idiopathic arthritis (JIA) is the childhood disability from a musculoskeletal disorder. It generally affects large joints such as the knee and the ankle. Muscle weakness, poor flexibility, atrophy, pain, and decreased proprioception of the affected joints, abnormal biomechanics, articular cartilage damage are the most common problems in patients with JIA. A greater Q angle has been suggested as a risk factor for musculoskeletal problems in various disease.

**Objectives:** We aimed to investigate the factors affecting Q angle in patients with JIA having pes planovalgus and healthy children.

**Methods:** Thirty-six participants as age range 4-16 years (25 female, 11 male) were enrolled this study. Children divided 2 groups (Group I: patients with JIA have pesplanovalgus, Group II; Healthy children). Hand held dynamometry was used to assess the strength of the lower extremity muscles. Also, Range of Motion (ROM) of lower extremity was evaluated with a universal goniometer. While static balance evaluated with Flamingo Balance Test (FBT), dynamic balance evaluated with Prokin balance system. Prokin balance system assesses perimeter length, Medium equilibrium center (A-P, M-L). Q angle was calculated by universal desktop ruler. Universal Desktop Ruler allows you to measure not only a straight-line distance but any curved distance on the screen. For statistical analysis SPSS Version 19.0 program was used.

**Results:** The means of age were 11.33±4.51 (Group I) and 11.28±1.87 (Group II) years. When the groups were compared with each other, Q angle was significantly higher in Group I (p=0.001). Correlations were found between strength of knee extension (r=-0.560, p=0.016) and ROM of knee flexion (r=0.528, p=0.024 with Q angle for group I. Also, Correlations were found between strength of hip abduction (r=-0.504, p=0.033), strength of hip adduction (r=-0.564, p=0.015), strength of knee extension (r=-0.470, p=0.049) and strength of ankle dorsiflexion (r=-0.713, p=0.001) with Q angle for group I. But, there was no correlation between Q angle and any parameters in Group II (p>0.05). According to linear regression analysis, Q angle was affected by only strength of knee extension for Group I (β=-0.13, p=0.000).  However, it was not found relationships between FBT, A-P and M-L with Q angle for both of the groups (p>0.05).

**Conclusion:** Q angle as excessive dynamic knee valgus is an abnormality of neuromuscular control over the lower limb. This study shows that Q angle was significantly increased and affected by strength of knee extension negatively in patients with JIA having pes planovalgus. So, this clear result suggests that the lower extremity should be evaluated holistically and strengthening of the knee extension should be considered in the treatment of patients with JIA even if the problem is only on the ankle. Besides, in future studies should assess educational status and quality of life.

**Disclosure of Interest**

None Declared

### P097 RECESSIVE MULTIPLE EPIPHYSEAL DYSPLASIA IN DIFFERENTIAL DIAGNOSIS OF JUVENILE ARTHRITIS

#### Aleksey Kozhevnikov^1,2^, Nina Pozdeeva^1^, Evgeniy Melchenko^1^, Michael Konev^1^, Vladimir Kenis^1^, Konstantin Afonichev^1^, Gennadiy Novik^2^

##### ^1^Federal state budget institution “The Turner Scientific Research Institute for Children's Orthopedics” under the Ministry of Health of the Russian Federation; ^2^Federal state budget institution of higher professional education “Saint-Petersburg State Pediatric Medical University” of the Ministry of Health of the Russian Federation, Saint-Petersburg, Russian Federation

###### **Correspondence:** Aleksey Kozhevnikov

**Introduction:** Juvenile arthritis (JA, JIA) is an umbrella-term describing a heterogeneous group of musculoskeletal diseases characterized by chronic synovitis of unknown etiology. Typical classic forms of JIA (oligo, poly, psoria, entheso) are well known. Genetic disorders with musculoskeletal involvement that mimic chronic arthritis should be considered in the differential diagnostics of JIA. The topically of the problem in children is determined by frequent cases of treatment of skeletal dysplasia by the DMARDs.

**Objectives:** The goal of this study was to improve the diagnosis of skeletal dysplasia manifested as JIA.

**Methods:** We carried out a retrospective review of more than fifty children with past-onset chronic undifferentiated arthropathy (low laboratory activity; progressive idiopathic contracture with entheso/synovitis) which were hospitalized at Children Orthopedics Institute, Saint-Petersburg between 2006 and 2016. The data of clinical of joint involvement, laboratory, x-ray, ultrasound, MRI were analyzed.

**Results:** All children were divided into two groups based on their disease. The first group (large) consisted of children with recessive multiple epiphyseal dysplasia (rMED/MED4, gene SLC26A2 Mut, OMIM 226900) characterized by abnormal development of the bone and cartilage of the epiphyses. A second group (small) was children with JIA. Children with congenital or early-onset forms of skeletal dysplasia and other monogenic arthritis-like diseases were excluded from study.

1. Less of morning stiffness, bilateral painless joint contractures and axial deformation of the lower limbs were the main finding of children with pseudo-rheumatoid pathology. Most children with rMED in the early childhood were characterized by a lack of signs of physiological hypermobility, reduced daily motor activity and treated of bilaterally delayed ossification of femoral heads. Morning stiffness and painful joint contractures were actually for children with JIA.

2. Radiology of JIA was characterized by inflammatory dynamic picture: overgrowth of metaepiphysis and joint osteoporosis at onset of disease, development dystrophic and erosive changes of osteochondral tissue at progressive durated and arthros-arthritis deformation like outcome. Symmetric flattening of epiphyses of hands, hips, knees and feet, double-layered patella were x-ray hallmarks of rMED. Avascular necrosis may be superimposed on rMED. Dystrophic spondylolisthesis at the L5-S1 vertebrae to 10-12 years of life was a frequent sign of epiphyseal dysplasia.

3. Instrumental sign of synovitis (ultrasound, MRI) was detected in most children. Synovitis in cases of skeletal dysplasia was secondary nature due to chronic damage and degeneration of the genetic defective articular hyaline cartilage (erosive-like chondrolysis).

4. Sensitivity to anti-inflammatory therapy was not always verified of the genesis of arthropathy and in most cases was dictated by the presence of synovitis.

**Conclusion:** This comparative study was revealed some clinical and instrumental characteristics of the skeletal dysplasia which mimic JIA. Early orthopedic-related "hip problems" at anamnesis, symmetric deformity of epiphyses, double-layered of patella indicate a possible of pseudo-rheumatoid genesis of arthropathy. Sequence of SLC26A2 gene should be recommended for use in cases with the not typical clinical and x-ray findings for JIA.

**Disclosure of Interest**

None Declared

### P098 SERUM CALPROTECTIN LEVELS AS A MARKER OF DISEASE ACTIVITY IN CHILDREN WITH JUVENILE IDIOPATHIC ARTHRITIS

#### Sathish Kumar, Anish S. George

##### Pediatrics, Christian Medical College, Vellore, India

###### **Correspondence:** Sathish Kumar

**Introduction:** Juvenile idiopathic arthritis (JIA) is the most common chronic rheumatic disorder of childhood and encompasses a complex group of disorders comprising several clinical entities with the common feature of arthritis. Calprotectin is a calcium- and zinc-binding protein that belongs to the S100 protein family and is released during the interaction of leucocytes with inflammatory activated endothelium at the sites of inflammation as occurs in JIA. We undertook this study to assess the usefulness of Calprotectin as a marker of disease activity in Indian children with JIA.

**Objectives:** To assess the effetiveness of serum Calprotectin levels as a marker of disease activity in children with JIA.

**Methods:** Children who fulfilled the International League of Associations For Rheumatology (ILAR) criteria for JIA were recruited into the study. Baseline demographic details were collected and Blood counts, ESR, CRP and Calprotectin levels were analyzed in all children after obtaining consent. Children were then divided into 2 groups based on disease activity as per Wallace criteria. Calprotectin levels were also analysed in 10 normal healthy children. Calprotectin levels were measured by using a “Human Calprotectin Kit” which works on the basis of sandwich-enzyme linked immune sorbent assay technology (ELISA).

**Results:** 121 children with JIA were recruited into the study, 63 had active disease and 58 had inactive disease. Systemic onset JIA constituted 42% of the study population and was the predominant disease subtype. Calprotectin levels were elevated in children with active disease compared to those with inactive disease. Mean Calprotectin value in active disease (3954ng/ml) was 2 fold higher than those with inactive disease (1899ng/ml) (p value ˂0.001) and 16 times higher than children who were normal healthy controls (mean of 233ng/ml). Area under curve for Calprotectin was 0.744. For a cut off value of 1760 ng/ml, Calprotectin had a sensitivity of 77% and specificity of 61% for assessment of disease activity in JIA.

**Conclusion:** Serum Calprotectin levels was found to be a good marker of disease activity in children with JIA. However, further studies which involve serial monitoring of Calprotectin levels in a study population will provide additional information about accuracy of these markers.

**Disclosure of Interest**

None Declared

### P099 CROSS-SECTIONAL STUDY OF SERUM CALPROTECTIN LEVELS IN NON-SYSTEMIC JUVENILE IDIOPATHIC ARTHRITIS PATIENTS AND CHILDREN WITH ACUTE INFLAMMATORY DISEASE

#### Lovro Lamot^1,2^, Lea Oletić^1^, Mandica Vidović^1^, Mirta Lamot^1^, Marijana Miler^1^, Nora Nikolac Gabaj^1^, Miroslav Harjaček^1,2^

##### ^1^Sestre Milosrdnice University Hospital Center; ^2^University of Zagreb School of Medicine, Zagreb, Croatia

###### **Correspondence:** Lovro Lamot

**Introduction:** in the past few years, various indicators of disease activity in juvenile idiopathic arthritis (JIA) patients have been investigated, allowing for the interpretation of different aspects of underlying inflammatory process (e.g. clinical, immunological, radiological etc.). Nevertheless, there is still evident lack of satisfactory support in diagnostic and prognostic evaluations in everyday clinical practice, focusing research activities on discovery of adequate biomarker that would support initial diagnosis and allow feasible disease monitoring. While growing number of evidence indicates phagocytic S100 proteins, like MRP8/14 complex (i.e. calprotectin), can be used to detect subclinical inflammatory activity in systemic JIA, their performance in other forms of JIA is still debatable, while in acute infectious diseases it is almost unknown.

**Objectives:** compare serum calprotectin (sCAL), CRP and PCT levels in patients with various forms of non-systemic JIA and previously healthy children with acute febrile illness lasting <3 days, as well as with juvenile arthritis disease activity score (JADAS).

**Methods:** out of 37 patients included in study, 17 were diagnosed with polyarticular form of JIA (pJIA), 8 with oligoarticular form (oJIA), 2 with juvenile spondyloarthritis (jSpA) and 10 with acute febrile respiratory illness of possible viral etiology (AFI). sCAL, PCT and CRP were measured in all of the patients, while JADAS70CRP was measured in all of the JIA patients. Inactive disease was defined as JADAS ≤1. The average duration of JIA was 64,2 (7-132) month, and all of the JIA patients were treated with standard therapy, including biological (n=16) and conventional (n=20) DMARDs. sCAL was measured by a commercial ELISA assay (Buhlmann MRP8/14, Switzerland).

**Results:** among JIA patients, 8 pJIA, 4 oJIA and 1 jSPA had inactive disease, while 9 pJIA, 4 oJIA and 1 jSpA had active disease. Average concentration of sCAL among patients with inactive disease was 1,86 (0,5-4,2) μg/mL, while in patients with active disease it was 2,37 (0,93-5,2) μg/mL (p=0,37). Statistically significant correlation was observed between sCAL and JADAS (Spearman r=0,523; p=0,005) in JIA patients and between sCAL and CRP (Spearman r=0,745; p=0,017) in children with AFI. Average concentration of sCAL in children with AFI was 4,67 (1,67-8,32) μg/mL, significantly higher than in patients with inactive (p=0,0024) or active JIA (p=0,0084) or in all JIA patients (p=0,0012).

**Table:** 

Disease		N	sCAL μg/mL (range)	CRP mg/L (range)	PCT ng/mL (range)	JADAS (range)
Inactive	pJIA	8	1,67 (0,65-2,66)	3,475 (0,4-17,5)	0,03 (0,01-0,10)	0,5 (0-1)
	oJIA	4	2,575 (0,8-4,2)	1,225 (0,5-2,2)	0,02 (0,01-0,03)	0,75 (0-1)
	Total	13	1,86 (0,5-4,2)	2,53 (0,02-17,5)	0,02 (0,01-0,10)	0,5 (0-1)
Active	pJIA	9	2,55 (0,93-4,1)	6,59 (0,2-40,4)	0,01 (0,01-0,02)	9,9 (2-22)
	oJIA	4	2,26 (1,3-4,2)	10,3 (0,7-37,7)	0,01 (0,01-0,02)	7,4 (3-17,7)
	Total	14	2,37 (0,93-5,2)	7,31 (0,2-40,4)	0,01 (0,01-0,02)	8,7 (2-22)
All JIA patients		27	2,11 (0,65-5,2)	4,92 (0,2-40,4)	0,02 (0,01-0,10)	4,6 (0-22)
All AFI patients		10	4,67 (1,67-8,32)	5,93 (0,4-26,7)	0,19 (0,09-0,16)	**/**

**Conclusion:** preliminary results of our cross-sectional study showed sCAL correlates with JADAS in non-systemic JIA patients, although there was no significant difference between those with active and inactive disease. Interestingly, sCAL concentrations in long lasting JIA patients were significantly lower than in children with short lasting viral illness. This is a novel observation that emphasizes the need for vigilance in interpreting sCAL levels in patients with concomitant acute communicable disease and gives a possible explanation for some of the high(er) values noted in patients with clinically inactive non-systemic JIA.

**Disclosure of Interest**

None Declared

### P100 STREPTOCOCCAL SEROLOGY IN A TWO-YEAR COHORT OF JIA PATIENTS - PRACTICAL GUIDANCE

#### Tanya Li^1^, Kathy Gallagher^2^, Peter Bale^2^, Kate Armon^2^

##### ^1^University of Cambridge; ^2^Cambridge University Hospitals NHS Foundation Trust, Cambridge, UK

###### **Correspondence:** Tanya Li

**Introduction:** For a child presenting to the paediatric rheumatology clinic with a swollen, painful joint it can be difficult to differentiate between a post-infective reactive arthritis and JIA. Antistreptolysin O titre (ASOT) and anti-DNase B can be used, but the literature suggests taking two samples 10-14 days apart for interpretation. (1) This can be difficult in the clinical setting.

**Objectives:** · To analyse the use and interpretation of streptococcal serology in new onset JIA patients at a tertiary hospital in the UK

· To compare this to evidence in the literature to recommend practical clinical guidance

**Methods:** The cohort consisted of patients with a new diagnosis of JIA made in 2016-2017. Data was collected from patient records on the following: recent illness, serology results and action taken. P values were calculated using Fisher’s exact test.

**Results:** 36 of the 54 patients identified had streptococcal serology (ASOT and anti-DNase B) at presentation. 16 (84%) of the 19 patients who reported a recent illness received streptococcal serology, compared to 20 (57%) of the 35 patients without recent illness. This difference was not statistically significant (p = 0.06).

There was no difference between the proportion of patients with abnormal titres in those who reported recent illness (7 of 16, 44%) compared to those without (8 of 20, 40%; p = 1). Furthermore, reporting recent illness was not associated with antibiotic use (6 of 16 (38%) compared with 4 of 20 (20%) without; p = 0.29).

In total 10 patients received antibiotics after initial serology results. 10-14 days of penicillin was always given when both ASOT and anti-DNase B titres were raised, but the decision to treat when only one titre was raised was variable. 6 of 11 (54%) patients with one abnormal titre were given antibiotics. Antibiotics were not given to patients with normal serology, or borderline ASOT alone.

No patients had repeat serology within 10-14 days. 5 patients had serology repeated, varying from 5 weeks to 14 months later.

**Conclusion:** This cohort consisted of patients with JIA and did not include those diagnosed with reactive arthritis, which would be valuable. ASOT typically rises 1 week after a streptococcal infection, falling between 2-8 months to baseline. Anti-DNase rises 2 weeks after infection and returns to baseline from 3-12 months. (2) A fourfold rise in titres taken 10-14 days apart provides evidence of recent streptococcal infection. However, taking repeat blood tests in young children can be both traumatic and practically difficult, with limited access to age-appropriate phlebotomy. A history of recent illness is not predictive of a raised titre in the context of JIA. A pragmatic approach, therefore, is to treat a single abnormal titre.

**References**

1. Sen ES, Ramanan AV. How to use antistreptolysin O titre. Arch Dis Child - Educ Pract. 2014 Dec 1;99(6):231–7.

2. Ayoub EM, Wannamaker LW. Evaluation of the Streptococcal Desoxyribo-Nuclease B and Diphosphopyridine Nucleotidase Antibody Tests in Acute Rheumatic Fever and Acute Glomerulonephritis. Pediatrics. 1962 Apr 1;29(4):527–38.

**Disclosure of Interest**

None Declared

**Table 1 (abstract P100).** Number of patients with abnormal serology results at initial presentation (% treated with antibiotics). Reference ranges from local laboratory. Titres given in international units (IU)

	Anti-DNase B (% treated with antibiotics)			
	Normal (< 400)		Abnormal (≥ 400)	
ASOT				
Normal (< 200)	19	0%	0	0%
Borderline (200)	2	0%	4	75%
Abnormal (≥ 400)	7	43%	4	100%

### P101 A UK STUDY: VOCATIONAL EXPERIENCES OF YOUNG ADULTS WITH JUVENILE IDIOPATHIC ARTHRITIS

#### Laura E. Lunt^1,2^, Matthew Bezzant^3^, Ailsa Bosworth^3^, Janet E. McDonagh^1,2^, Kimme Hyrich^1,2^, Wendy Thomson^2,4^, Suzanne Verstappen^1,2,5^

##### ^1^Arthritis Research UK Centre for Epidemiology, Centre for Musculoskeletal Research, Manchester Academic Health Science Centre, The University of Manchester; ^2^NIHR Manchester Biomedical Research Centre, Manchester University NHS Foundation Trust, Manchester Academic Health Science Centre, Manchester; ^3^National Rheumatoid Arthritis Society (NRAS), Maidenhead; ^4^Arthritis Research UK Centre for Genetics and Genomics, Centre for Musculoskeletal Research, The University of Manchester, Manchester; ^5^Arthritis Research UK/MRC Centre for Musculoskeletal Health and Work, University of Southampton, Southampton, UK

###### **Correspondence:** Laura E. Lunt

**Introduction:** Little is known about the experiences of young adults living with Juvenile Idiopathic Arthritis (JIA) preparing for employment and career development.

**Objectives:** The purpose of this study was to understand the impact JIA has on career planning and early employment experiences of young adults (16-30 years).

**Methods:** Using existing literature (including grey literature), an online survey (consisted of 152 questions, 29 items related to young adults two of which were free text questions) was developed and sent to National Rheumatoid Arthritis Society (NRAS) members and distributed to non-members via social media tools including Facebook, Twitter and HealthUnlocked. Data collected included views and experiences in career planning and employment. The data pertaining to young adults are presented here.

**Results:** Of 1241 respondents 19 were young adults with JIA (range 16-30 years), 89% were female and 84% had university or equivalent qualifications. Due to incomplete responses there is missing data on all 19 young adults. 4/13 young adults were studying at university, 9/13 were in paid employment. 9/17 respondents reported their school did not offer additional work-related activities to students with disabilities and/or additional needs. 10/14 young adults felt their school did not provide advice about coping with possible limitations on placements/traineeships due to their arthritis. 11/14 respondents thought about their condition when thinking about future career plans e.g. “I wanted to work as a ranger or similar for the National Trust but it’s a fairly physically demanding job and I knew my joints would suffer so I changed track slightly”. However, 8/14 felt their career advisors did not take their arthritis into account e.g. “I had to cease my physiotherapy master’s degree as my arthritis got too bad to continue and change career choice. I wish there would have been more discussion about it not being a reasonable choice for me at the time as we just didn't have the information then”. 8/14 young adults changed their career plans because of their arthritis. Managing JIA symptoms and a physically demanding role, as well as wanting to stay healthy, were the main reason for changing career. Important aspects of employment included: good relationships with your line manager, work you like doing and a job you can use your initiative.

**Conclusion:** Despite small numbers these results highlight potential current unmet vocational needs of young adults with JIA in the UK and the need for further research with this age group. There appears to be a lack of structured support within schools and universities offered to students with disabilities and/or additional needs, about work-related activities and careers. Young adults with JIA actively consider their condition whilst thinking about career opportunities and value a productive and challenging job with a good working environment, including relationships with colleagues and supervisors.

**Disclosure of Interest**

None Declared

### P102 BODY MASS INDEX AND JUVENILE IDIOPATHIC ARTHRITIS: IS THERE A CORRELATION WITH SEVERITY, PROGNOSIS AND TREATMENT RESPONSE?

#### Ilaria Maccora, Francesca Tirelli, Edoardo Marrani, Teresa Giani, Gabriele Simonini, Rolando Cimaz

##### Rheumatology Unit, University of Florence, A. Meyer Children's Hospital, NEUROFARBA Department, Florence, Italy

###### **Correspondence:** Ilaria Maccora

**Introduction:** There is evidence that obesity could be a risk of factor for the development of RA due both to the mechanical effect of overweight and to the potential pro-inflammatory effects of cytokines produced by adipose tissue.

**Objectives:** To evaluate the role of overweight and obesity in a cohort of JIA patients, in terms of incidence, disease activity, outcome and response to treatments.

**Methods:** This single-center retrospective cohort study evaluated 125 children affected by JIA under treatment with anti-rheumatic agents (NSAIDs/IAS, DMARDs, biologic agents). Change from baseline in ERS, CRP, number of active joints (with distinction between upper and lower limb joints), and BMI was analysed under each treatment until last visit. BMI categories of 5-84th (normal weight), 85-94th (overweight), and ≥ 95th (obese) percentile were used. Patients with systemic JIA or chronic comorbidity under potentially confounding systemic treatments were excluded. Informed consent was obtained by the patients and their family.

**Results:** One hundred twenty-five JIA patients (36% oligoarticular JIA, 42,4% RF-negative polyarticular JIA, 0,8% RF-positive polyarticular JIA, 8% enthesitis related arthritis, l’11,2% psoriatic JIA, and 1,6% undifferentiated unclassified arthritis) were enrolled in the study, 76,8% girls, 23,3 boys. The mean age was 5,9 years (± 3,8). Baseline BMI was ≤ 84th percentile in 73,22% of patients, 85-94th in 19,64%, and ≥ 95th in 7,14%.

We did not observe a significative association between BMI and ERS (p=0,29), CRP (p=0,24), or number of active joints (p=0,45) at baseline, while the involvement of the joints of lower limb was significantly greater (p=0,025) in overweight/obese patients.

We also demonstrated a substantial equality in remission and relapse rates in subjects with different BMI.

**Conclusion:** This study focuses on the complex relationship between overweight/obesity and JIA. A significant correlation between obesity and a greater involvement of the joints of the lower limbs was observed at baseline. Furthermore, we observed that obesity does not influence the course of the disease nor treatment response. These data seem to suggest a prevalent mechanical effect of ponderal excess on JIA, rather than a biochemical influence due to pro-inflammatory cytokines released by adipocytes.

**Disclosure of Interest**

None Declared

### P103 JUVENILE IDIOPATHIC ARTHRITIS AND FITNESS: A TEAMWORK

#### Maria Cristina Maggio^1^, Antonio Palma^2^, Giuseppe Messina^2^, Jessica Brusa^2^, Angelo Iovane^2^, Livia Cimino^1^, Giovanni Corsello^1^

##### ^1^University Department Pro.Sa.M.I. “G. D’Alessandro”; ^2^Department of Psychology and Educational Science, University of Palermo, Palermo, Italy

###### **Correspondence:** Maria Cristina Maggio

**Introduction:** Patients with Juvenile Idiopathic Arthritis (JIA) have limited fitness and reduced aerobic and anaerobic exercise capacity vs. healthy peers. Furthermore, low intensity exercise programs are safe in children with JIA and may improve fitness, joint excursion and quality of life, reduce pain, fatigue and the employ to anti-inflammatory drugs.

**Objectives:** The purpose of the study was to evaluate postural and balance deficits and fitness with specific test battery in children and adolescents affected by JIA.

**Methods:** We enrolled 30 patients with JIA (13 M; 17 F; age: 8-18 years); among those, 7 were evaluated longitudinally in the period 2016-2018, comparing the tests in different periods of the illness. The posturography test was administered with the FreeMed posturography system (the FreeMed baropodometric platform and FreeStep v.1.0.3 software). A specific fitness test battery was used to evaluate the physical fitness level of the patients (Abalakov test, backsaver sit and reach, the toe touch test, sit-up test and hand grip test).

**Results:** 2 M and 5 F was in an acute phase of the disease (1 sJIA; 5 polyarticular JIA; 1 psoriatic JIA).

At the posturography test, the distribution of the weight between left and right was pathological in 15 (4 sJIA; 9 polyarticular JIA; 2 oligoarticular JIA).

The load distribution between forefoot and hindfoot was pathological in all the patients, with a more severe overload in polyarticular JIA patients.

Hand-grip test in 8 patients was <3°Centile; in 11 was < 20°Centile. The patients who performed a regular physical activity program showed fitness test in the normal range, and these parameters were not correlated with the type of JIA and/or the treatment for the arthritis.

Among the patients evaluated in follow up, 2 (1 with sJIA and 1 with polyarticular JIA) maintained an asymmetry in the weight distribution between left and right and a reduced fitness. 5 patients (2 M with sJIA and 3 F with polyarticular JIA) normalized their parameters.

**Conclusion:** The persistent asymmetry of the load distribution between left and right foot and the persistent pathological distribution between forefoot and hindfoot was more frequent in patients with polyarticular JIA.

A regular physical activity program is the best strategy to maintain an adequate fitness and the best control of the disease.

**Disclosure of Interest**

None Declared

### P104 TITLE: “SERUM CALPROTECTIN AND JOINT ULTRASOUND IN PATIENTS WITH JUVENILE IDIOPATHIC ARTHRITIS”

#### Manuela Marsili^1^, Marina Primavera^1^, Giovanni Cannataro^2^, Caterina Di Battista^1^, Giuseppe Lapergola^1^, Roberto Troiani^1^, Debora d'Angelo^1^, Raffaella Faricelli^3^, Francesco Chiarelli^1^, M. Loredana Marcovecchio^4^, Luciana Breda^1^

##### ^1^Department of Pediatrics, University of Chieti; ^2^Department of Neuroscience, Imaging and Clinical Sciences; ^3^Unit of Clinical Pathology, SS Annunziata Hospital, Chieti, Italy; ^4^Department of Pediatrics, University of Cambridge, Cambridge, UK

###### **Correspondence:** Manuela Marsili

**Introduction:** The identification of new biomarkers, the development of more effective outcome measures and the refinement of imaging techniques may foster the implementation of targeted and personalized therapeutic interventions in patients with juvenile idiopathic arthritis (JIA). Among new biomarkers, serum calprotectin (MRP8/MRP14), a complex of calcium- and zinc-binding proteins, seems to be promising. Besides, musculoskeletal ultrasonography (MSUS) is a useful imaging tool for evaluating JIA patient disease activity. Many studies have demonstrated that MSUS can improve the sensitivity and the accuracy in the detection of the exact sites of inflammation in the joint compared to the clinical examination only.

**Objectives:** To evaluate MRP8/MRP14 and MSUS in a group of JIA patients and to verify which of these tools best identifies disease activity.

**Methods:** 70 children with JIA referred to the Rheumatology Unit of the Department of Pediatrics of Chieti, were enrolled. Serum MRP8/MRP14 of each patient was detected by PhiCal Calprotectin ELISA. MRP8/MRP14 was defined normal for values < 3 μg/ml. JADAS-27 was used to define disease activity. At study enrollment, all patients underwent an ultrasound assessment of all joints clinically affected. A total of 452 joints were scanned for the presence of synovial effusion (SE), synovial hyperplasia (SH) and power Doppler (PD) signal. The ultrasound examinations technique as well as definitions and scoring features were based on guidelines provided by the OMERACT study group. In each joint, SE, SH and PD signal were graded on a 0–3 scale. The threshold used for synovial abnormalities was 1.

**Results:** MRP8/MRP14 serum levels were increased in active vs inactive disease patients (p-value <0.001). Significant differences between active and inactive patients were found in CRP, ESR, JADAS score and the prevalence of synovitis (Table 1). According to the score evaluating SE or SH and PD, 20 out of 28 (71.4%) JIA patients with active disease had a score equal to 2 vs 2 out 40 (4.7%) patients achieving remission (p-value 0.001). ESR, CRP, MRP8/MRP14 and synovitis with its specifics components were all positively related to JADAS-27. MRP8/MRP14 was positively correlated with presence of synovitis and its specific characteristics.

**Conclusion:** To our knowledge, this is the first study comparing MRP8/MRP14 and MSUS in a group of JIA patients. We found a significant association between MRP8/MRP14 levels, clinical and laboratory markers of disease activity. Moreover, we demonstrated an association between MRP8/MRP14 serum levels and ultrasonography-determined synovitis. Our study confirmed that both MSUS and MRP8/MRP14 may be considered valid and reliable tools in the assessment of synovial inflammation in JIA. Larger longitudinal studies are needed to establish the role of MRP8/MRP14 and MSUS in the diagnosis and follow-up of childhood arthritis.

**Disclosure of Interest**

None Declared

**Table 1 (abstract P104).** Clinical, laboratory and ultrasound data at baseline between active and inactive patients. Data represent means ± SD or median (interquartile range)

	Active n. 28	Inactive n. 42	p-value
Age at enrollment (years)	7.28±3.79	12.45±4.95	<0.001
JADAS-27 score	10.6±5.5	0.4±0.5	<0.001
Disease duration (years)	1.67±3.25	7.56±4.54	<0.001
MRP8/MRP14 (μg/ml)	3.920 (2.417-5.078)	1.990 (0.85-2.86)	<0.001
ESR (mm/h)	10.0 (6.0-32.0)	7.0 (5.0-9.2)	<0.001
CRP (mg/dl)	0.610 (0.29-1.720)	0.29 (0.29)	<0.001
Synovitis (Yes/no)	20/8	2/40	<0.001

### P105 CREATION OF AN ONLINE LEARNING RESOURCE FOR JIA IN ADULTS

#### Joanne May^1,2^, Nicola Smith^2^, Helen Foster^2^

##### ^1^Paediatric Rheumatology, Oxford University Hospitals, Oxford; ^2^Institute of Cellular Medicine, Newcastle University, Newcastle, UK

###### **Correspondence:** Joanne May

**Introduction:** We present the development of an evidence based online learning resource for rheumatologists who manage adolescents and adults with JIA as part of their routine practice, including translation of a learning needs analysis into learning activity design and the creation of three interactive learning modules.

**Objectives:** Many children and young people with JIA will continue to have active disease as adults and will need treatment delivered by adult rheumatology services. A learning needs analysis of the UK adult rheumatology community identified key areas where further training in the management of JIA is required:

- Knowledge about JIA, including understanding JIA subtypes, how JIA manifests in adults, and how JIA differs from adult onset arthritis.

- Knowledge, skills and guidance for the management of adolescents and young adults with JIA.

- Approaches to management in adults with JIA.

The **JIA in Adults e-learning series** was developed with the British Society for Rheumatology (BSR) to target these educational needs.

**Methods:** The content and format were informed through our research to gather opinion from adult and paediatric rheumatologists in the UK (Smith et al, 2017).  A PACT analysis (People, Activities, Context, Technology) informed the design process. Learning activities were based on constructivist learning theory with cognitive considerations for online learning activity design: activation of prior knowledge, construction of new knowledge and knowledge transfer.

**Results:** The ‘People’ element of the PACT analysis was an essential consideration – experienced clinicians with a varied prior exposure to adolescent and young adult (AYA) rheumatology, both through formal training and clinical practice. Therefore, learning activities designed to activate prior knowledge and therefore optimise the foundation for further leaning were key.

Social considerations and the wider, National and Global view were used to define purpose, motivate and prepare for learning. New knowledge was presented around clinical cases so that key concepts could be explored in depth and related back to prior experience. A series of interactive cases with questioning focusing on *evidence*, *explanation*, *hypothetical reasoning*, *cause and effect* and *synthesis of understanding* were used to develop clinical reasoning skills and to facilitate knowledge transfer to new clinical scenarios. The inclusion of the *‘more knowledgeable other’* in the form of feedback from an expert in the field of AYA rheumatology further enhanced the development of advanced clinical reasoning.

The learning needs analysis demonstrated that many find managing the complex psychosocial needs of AYA to be particularly challenging, therefore the cases explored these particular elements in more depth.

Further cognitive considerations in the module design included enhancing learning through the use of video media, data interpretation, interviews, pictures and quizzes, and through the reduction and segmentation of text.

**Conclusion:** An e-learning series for adult rheumatologists who manage JIA in adults as part of their routine clinical practice was successfully created through the application of an evidence-based learning needs analysis to inform learning activity design, appropriate to the prior experience of the learners through interactive case-based learning.

**Reference**

**Biological treatments for adults with Juvenile Idiopathic Arthritis: a clear need for improved access to targeted education and training.**  Nicola Smith, Tim Rapley Flora McErlane Lianne Kearsley-Fleet, Kimme L Hyrich Helen E Foster. *Rheumatology*, Volume 56, Issue suppl_7, 1 December 2017, kex390.049, https://doi.org/10.1093/rheumatology/kex390.049

**Disclosure of Interest**

None Declared

## JIA-Spondyloarthritis

### P106 THE IMPACT OF ADALIMUMAB ON GROWTH IN PATIENTS WITH PEDIATRIC ENTHESITIS-RELATED ARTHRITIS

#### Ruben Burgos-Vargas^1^, Shirley M. Tse^2^, Kirsten Minden^3^, Pierre Quartier^4^, Jaclyn K. Anderson^5^, Kristina Unnebrink^6^, Ivan Lagunes^5^, Gerd Horneff^7^

##### ^1^Hospital General de Mexico, Universidad Nacional Autonoma de Mexico, Mexico City, Mexico; ^2^2The Hospital for Sick Children, Toronto, Canada; ^3^Charite University Medicine, Berlin, Germany; ^4^Hopital Necker-Enfants Malades, Paris, France; ^5^AbbVie, N Chicago, USA; ^6^AbbVie, Ludwigshafen; ^7^Asklepios Klinik, Sankt Augustin, Germany

###### **Correspondence:** Ruben Burgos-Vargas

**Introduction:** Children with one or more subtypes of juvenile idiopathic arthritis, such as enthesitis-related arthritis (ERA), often exhibit growth impairments.

**Objectives:** To explore the impact of adalimumab (ADA) on growth in pediatric patients (pts) with ERA.

**Methods:** Pts aged 6-<18 with ERA were enrolled in a phase 3, multicenter, randomized, double-blind, study. Following 12 weeks of treatment with ADA (24 mg/m^2^ BSA up to 40 mg every other week [eow]) or placebo, pts were eligible to enroll in an open-label extension and receive ADA eow for up to an additional 192 weeks. For this analysis, all pts who received ≥1 dose of ADA were included, and pts were grouped by baseline height percentiles into 2 categories: ≤25^th^ and >25^th^ percentiles based on the World Health Organization (WHO) growth charts. Mean WHO percentile changes in height, weight, and body mass index (BMI) percentiles were calculated through 204 weeks. Per protocol, bone age and familial height were not collected. Growth and efficacy data were analyzed as observed.

**Results:** Among the 46 pts who received ≥1 dose of ADA in this study, 67% were male with a mean age of 12.9 years; no pts had associated IBD. Eleven pts (24%) were in the ≤25^th^ height percentile, and these pts had a numerically lower baseline height (147.7 cm) and weight (42.5 kg) compared with those pts who were in the >25^th^ height percentile (156.0 cm and 51.5 kg, respectively). Additionally, numerically higher proportions of pts in the ≤25^th^ percentile received concomitant corticosteroids than did the >25^th^ percentile group (54.5% vs 28.6%). Pts in the ≤25^th^ percentile group experienced a larger change in mean height percentile through 204 weeks of ADA treatment (70.3 vs 20.5 for the >25^th^ percentile), a finding that was evident within the first 6 months of treatment. Juvenile males in the ≤25^th^ baseline height percentile demonstrated the numerically highest rates of growth, although similar levels of growth improvement were observed for the lowest quartile of females as well. None of the 11 pts in the ≤25^th^ baseline height percentile remained in this category at their final study visit (Table 1). Similar percentile increases were observed for BMI percentiles between groups. ACR Pedi90 response rates improved over time in both ≤25^th^ and >25^th^ percentile groups, reaching approximately 80% at the end of 3 years treatment with ADA.

**Conclusion:** Long-term ADA treatment was associated with growth improvement and maintenance in children with ERA. These improvements among children in the lowest WHO quartiles at baseline may improve their quality of life and psychosocial environment. ADA treatment improved ERA signs and symptoms, regardless of baseline growth status.

**Reference**

1. Burgos-Vargas R, *et al*. *Arthritis Rheumatol* 2016;68(Suppl 10).

**Disclosure of Interest**

R. Burgos-Vargas Grant / Research Support from: AbbVie, Consultant for: AbbVie, BMS, Janssen, Pfizer, and Roche. , S. Tse Grant / Research Support from: AbbVie, Consultant for: AbbVie and Pfizer, K. Minden Grant / Research Support from: AbbVie and Pfizer, Consultant for: AbbVie, Pfizer, Pharm-Allergan, and Roche/Chugai, P. Quartier Grant / Research Support from: AbbVie, Novartis, Pfizer, and Roche/Chugai, Consultant for: AbbVie, BMS, MedImmune, Novartis, Pfizer, Roche/Chugai, Servier, and Sobi, J. Anderson Employee of: AbbVie, K. Unnebrink Employee of: AbbVie, I. Lagunes Employee of: AbbVie, G. Horneff Grant / Research Support from: AbbVie, Pfizer, and Roche, Consultant for: AbbVie, Novartis, Pfizer, and Roche

**Table 1 (abstract P106).** Distribution of Height and BMI by WHO Percentile at Baseline and Final Visit

n (%)	WHO Percentile	
	≤25^th^	>25^th^
Height, N=46		
Baseline	11 (24)	35 (76)
Final Visit	0	46 (100)
BMI, N=46		
Baseline	10 (22)	36 (78)
Final Visit	3 (7)	43 (93)

### P107 CLINICAL AND THERAPEUTICAL FACTORS LINKED TO THE JOINT INVOLVEMENT IN PEDIATRIC INFLAMMATORY BOWEL DISEASE

#### Emanuela Del Giudice^1^, Ilaria Battagliere^1^, Anna Dilillo^1^, Franca Viola^1^, Giuseppe La Torre^2^, Salvatore Cucchiara^1^, Fabrizio De Benedetti^3^, Marzia Duse^1^

##### ^1^Department of Pediatrics; ^2^Department of Public Health and Infectious Diseases, Sapienza University of Rome; ^3^Division of Rheumatology, IRCCS Ospedale Pediatrico Bambino Gesù, Rome, Italy

###### **Correspondence:** Emanuela Del Giudice

**Introduction:** The association between inflammatory bowel disease (IBD) and joint involvement are well known, but the clinical and therapeutical features linked to them has been poorly investigated mostly in pediatrics.

**Objectives:** To identify factors associated with the joint involvement in pediatric IBD

**Methods:** A retrospective cohort of 83 pediatric IBD with joint complain(56 Crohn’ s disease [CD]/27 Ulcerative colitis[UC] were enrolled in two medical centers in Rome, evaluated at the time of the first rheumatologic manifestation and after 6,12 and 24 months.

The diagnosis of IBD was based on agreed endoscopic and histological criteria, CD and UC disease activity was measured respectively by PCDAI and PUCAI.

Demographic data and disease related IBD variables at first joint complain were recorded for each patient.

Laboratory tests and clinical examination including the type of active, limited joints were assessed. Moreover the gastrointestinal tract affected, the step up or top down therapy approach, the disease joint remission were collected.

Joint complaints were categorized on the basis of clinical documentation as sacroileitis, peripheral arthritis, and arthralgia.

**Results:** Regarding the different types of rheumatic manifestations compared to the IBD diagnosis, arthritis begins earlier than arthralgia(p=0.031). Of the 83 patients,44(53%) had arthralgia and 39(47%) arthritis, and in this last subgroup 23 had peripheral arthritis and 16 sacroileitis. The prevalence of arthritis or arthralgia was not significantly different in both groups: CD(48% 44%) and UC(52%>56%)(p=0.930).16/83 patients enrolled had a sacroileitis,11(69%) had a CD and 5(31%) UC. In IBD group the frequency of small, coxo-femoral and sacroiliac joints complain was higher in the arthritis than arthralgia patients(p=0.006;p=0.001;p=0.001,respectively).

CD patients in both arthritis and arthralgia groups, showed a higher prevalence of the Ileum-colon involvement (L3)(63%vs52%) than ileum (L1)(15%vs27.7%) and colon disease (L2)(22%vs20.7%). Also in the subgroup of sacroileitis in CD the L3 involvement was higher(73%) than L1(18%) and L2(9%).

The joint remission was higher in arthritis patients on top down treatment than those on step up and the difference was already statistically significant at follow up at times 6 and 12 months(p=0.043,p=0.036,respectively).The peripheral arthritis group showed a higher frequency of remission at the follow up at 6,12 and 24 months compared with the sacroileitis.

**Conclusion:** Arthritis is an earlier extraintestinal manifestation in pediatric IBD compared to arthralgia, and sacroileitis is more frequent in CD. Otherwise UC and CD have the same frequency to develop arthritis or arthralgia.

The L3 localization in CD may be related itself to the development of joint involvement.

The top down therapy in case of joint manifestation showed a higher frequency of remission.

**Disclosure of Interest**

None Declared

### P108 SPONDYLOARTHRITIS RESEARCH CONSORTIUM OF CANADA SACROILIAC JOINT INFLAMMATION SCORE IN CHILDREN WITH ENTHESITIS-RELATED ARTHRITIS

#### María Katsicas^1^, Monica Galeano^2^, Martin Pradier^1^, Clara Anoni^2^, Ricardo Russo^1^

##### ^1^Immnulogy & Rheumatology; ^2^Department of Imaging, HOSPITAL DE PEDIATRÍA GARRAHAN, Buenos Aires, Argentina

###### **Correspondence:** María Katsicas

**Introduction:** Enthesitis-related arthritis (ERA) is a category of juvenile idiopathic arthritis considered to be a form of Juvenile Spondyloarthropathy (SpA). Spondyloarthritis Research Consortium of Canada sacroiliac joint score (SPARCC SIS) measures inflammation in sacroiliac joints. It has been used in adults but limited experience exists in children.

**Objectives:** To assess SPARCC SIS in children with ERA. To investigate the association between SPARCC SIS score and clinical–biochemical features

**Methods:** Patients with ERA (according to ILAR criteria) who had sacroiliac (SI) joints MRI were included in a cross sectional study. Demographic features and time of follow up were recorded. ASAS classification criteria were applied to patients. SI joints were examined using T1-weighted images, T2 fast –suppressed and short-tau inversion recovery. The SPARCC was scored independently by 2 radiologists. Readers were blinded to clinical features. SPARCC SIS assessed the presence, depth and intensity of bone marrow edema on consecutive six slices as an increased signal in the iliac and sacrum bones. Scoring is composed by 3 components: bone marrow (BM) edema (0-48), BM intensity (0-12), BM depth (0-12). Maximum:72. Clinical variables were collected in random visits: active joint (AJ), sacroiliac pain (SIP) , lumbar pain (LP),lumbar limitation (LL) by Schöber´s test, wellbeing according to the patient using a visual analogue scale (VASp), disease activity according to the physician (VASphy), VAS pain ,JADAS-10(juvenile arthritis disease activity score), JSpADA(juvenile spondyloarthritis disease activity index), ESR and functional capacity by CHAQ. Treatment with TNF inhibitors (TNFi) were recorded. Descriptive, summary statistics, Intraclass Correlation Coefficient (ICC) for concordance between readers, Kruskal wallis one way AOV -test and post-hoc comparisons were done. Chi^2^ for categoric variables were used.

**Results:**

Thirty patients (83% M) fulfilled inclusion criteria. Median age was 12 (5-17) years and disease duration 5.6 (1-8) years. HLA-B27 positive in 12 (40%). TNFi in 17 (57%). Twenty-five patients (83%) had abnormal SI MRI. SPARCC SIS score was (medians±SD) 24±14.71. ICC for concordance between readers =0.73(p=0.02) IC 95% 0.43-0.88. MRIs were divided into 3 groups according to score. Clinical and demographic variables were compared among groups (Table 1).

There were no significant differences in activity measures (VASp, VASphy, VAS pain, JADAS-10, JSpADA) neither ESR, nor CHAQ among groups. MRI showed inflammation in 7 patients with normal X-rays(p=0.005). All patients fulfilled ASAS criteria for SpA. All patients who fulfilled ASAS-axial (14 patients) showed MRI abnormalities (SPARCC SIS 24 ±13.6). 10/16 patients who were classified as peripheral according to ASAS showed sacroiliitis (24 ±15.09). Eight patients met criteria for Ankylosing Spondylitis (AS) according modified New York Criteria:their SPARCC score was 24±12.0.

**Conclusion:** SPARCC SIS score was associated with age and disease duration. Patients classified as peripheral according to ASAS had similar SPARCC scores to those of patients classified as axial, which probably indicates this classification is not relevant for ERA patients.

**Disclosure of Interest**

None Declared

**Table 1 (abstract P108).** See text for description

	Group 1 n=5 Normal MRI	Group 2 n=16 Score >1 to 24 (median±SD 21±5.7)	Group 3 n=9 Score >24 to 72 (median±SD 36±9.78)	p
Age at study*	10(5-17)	12 (9-16)	14 (7-17)	0.001
Duration of disease*	2 (0-5)	4(1-8)	4(2-6)	0.02
AJ*	1 (0-5)	1(0-16)	2(0-8)	ns
SIP **	3 (60)	8 (50)	5(56)	ns
LP**	1(20)	12(75)	4 (44)	ns
LL **	5 (4.5-5.5)	4.5 (3-7)	5 (3.5-6)	ns

*median (range) **Nº(%)

### P109 DIFFERENCES OF CYTOKINE LEVELS BETWEEN ABORIGINAL AND NON-ABORIGINAL CHILDREN WITH JUVENILE IDIOPATHIC ARTHRITIS IN SAKHA REPUBLIC (YAKUTIA).

#### Fekla Vinokurova^1^, Tatiana Burtseva^1^, Vyacheslav Nikolaev^1^, Vera Argunova^2^, Vyacheslav Chasnyk^3^, Mikhail Kostik^3^

##### ^1^Yakut Scienсе Center of Complex Medical Problems; ^2^National Medical Center, Yakutsk; ^3^SAINT-PETERSBURG STATE PEDIATRIC MEDICAL UNIVERSITY, Saint-Petersburg, Russian Federation

###### **Correspondence:** Mikhail Kostik

**Introduction:** juvenile idiopathic arthritis (JIA) is not uncommon disease among aboriginals in Sakha Republic (Yakutia) - SR(Y), which can be related to high spreading of HLA B27 antigen. Aboriginals in SR(Y) have higher prevalence of enthesitis-related category of JIA and juvenile ankylosing spondylitis, increased family history of rheumatic diseases, especially arthritis and have higher requirement in biologic medicine (anti-cytokine antibodies) for arthritis control compare to Caucasians.

**Objectives:** The aim of our study was to evaluate cytokine profile in aboriginals in SR(Y) and compare to Caucasians have been living in the same area.

**Methods:** In continuous study were included 108 JIA patients before age 18 consisted of 96 SR(Y) aboriginals and 12 Caucasians with JIA whom have been living in SR(Y). All patients have an active disease course. In children HLA B 27 and interleukin-1β, interleukin-6, interleukin-10, interleukin-4, tumor necrosis factor-α, γ-interferon levels were detected.

**Results:** The prevalence of HLA B27 antigen was 50% in RS(Y) aboriginals. We have found differences in cytokines levels between aboriginals and Caucasians: inerleukin-10: 3.75±1.7 and 3.24±0.9 pg/ml (р=0.038), inerleukin-1β: 2.9±8.4 and 2.0±0.9 pg/ml (р=0.0000001), tumor necrosis factor-α: 6.3±9.1 and 4.9±2.2 pg/ml (р=0.00001), γ-interferon: 22.7±11.4 and 20.1±3.5 pg/ml (р=0.007). No differences were observed in inerleukin-4 and inerleukin-6 levels between groups. Inerleukin-6 positively correlated with ESR (r=0.72, p<0.05). There was not found correlation between cytokines level and presence of HLA B27 antigen.

**Conclusion:** the prevalence of pro-inflammatory cytokines were observed in aboriginals in SR(Y) compare to Caucasians. Further investigations required to validate these data in clinical practice.

**Disclosure of Interest**

None Declared

## Uveitis

### P110 THE COURSE OF UVEITIS IN PATIENTS WITH JUVENILE IDIOPATHIC ARTHRITIS TREATED WITH ADALIMUMAB

#### Ekaterina Alexeeva^1,2^, Tatyana Dvoryakovskaya^1,2^, Rina Denisova^1^, Tatyana Sleptsova^1^, Kseniya Isaeva^1^, Alexandra Chomahidze^1^, Anna Fetisova^1^, Anna Mamutova^1^, Victor Gladkikh^3^, Alina Alshevskaya^3^, Andrey Moskalev^3^

##### ^1^Federal State Autonomous Institution “National Medical Research Center of Children's Health” of the Ministry of Health of the Russian Federation; ^2^Federal State Autonomous Educational Institution of Higher Education I.M. Sechenov First Moscow State Medical University of the Ministry of Health of the Russian Federation, Moscow; ^3^Biostatistics and Clinical Trials Center, Novosibirsk, Russian Federation

###### **Correspondence:** Ekaterina Alexeeva

**Introduction:** Although predictors for the development of uveitis in patients with juvenile idiopathic arthritis (JIA) are well known, uveitis regarded as a separate, parallel disease has its own models of disease course, and accordingly, its own trends for remission. Adalimumab (ADA) is a highly effective anti-TNF drug having a strong therapeutic effect on both JIA and uveitis.

**Objectives:** This study aimed to assess the parameters associated with achieving remission of uveitis as a comorbid disease in JIA during ADA therapy.

**Methods:** The study involved 112 patients with uveitis who had initiated ADA therapy at the National Medical Research Center of Children's Health (Moscow). The median duration of JIA was 4 years (IQR 2:8.1); the median duration of uveitis was 1 year (IQR 0:5). Twenty-nine patients (25.9%) had only one eye affected. At ADA treatment initiation, 10 (8.9%) of patients had remission of uveitis. Almost half (49.1%) of the patients had a history of uveitis complication and 17.9% of patients had already undergone eye surgery. Treatment efficacy was evaluated according to the dynamics of clinical and laboratory signs, as well as according to achieving stable remission of uveitis.

**Results:** ADA therapy showed high efficacy in children with uveitis. After the first year of therapy, 89 patients (79.5%) achieved stable remission of uveitis; none of them had uveitis flares during the subsequent follow-up (the maximum follow-up period was 5 years). At the last visit, 92 patients (82.1%) achieved remission of uveitis. Among the remaining 20 patients (Flare group, 17.9%), only one patient demonstrated no positive dynamics of the uveitis course; the other 19 patients developed uveitis flare after 2-17 months of remission. Having compared the Flare group with the Remission group, we found the factors potentially associated with treatment response. The factors associated with recurrent uveitis included the greater number of uveitis flares in the past; complications affecting the vitreous body or the cornea; two eyes affected; and longer JIA duration before uveitis presentation.

**Conclusion:** ADA proved to be highly efficacious when used to treat children with uveitis. However, the severe uveitis affecting eyes with a large number of flares in the past and complications affecting the vitreous body and the cornea reduced the likelihood of achieving stable remission in children with JIA.

**Disclosure of Interest**

None Declared

### P111 THE ETIOLOGIC SPECTRUM OF PEDIATRIC NON-INFECTIOUS UVEITIS

#### Mustafa Çakan^1^, Şerife Gül Karadağ^1^, Dilbade Yıldız Ekinci^2^, Nuray Aktay Ayaz^1^

##### ^1^Pediatric Rheumatology; ^2^Ophthalmology, Kanuni Sultan Süleyman Research And Training Hospital, İstanbul, Turkey

###### **Correspondence:** Mustafa Çakan

**Introduction:** The most common causes of non-infectious uveitis in pediatric age group are rheumatologic diseases [juvenile idiopathic arthritis (JIA), Behçet disease (BD), sarcoidosis, and vasculitides], tubulointerstitial nephritis and uveitis (TINU) and idiopathic uveitis.

**Objectives:** The aim of this study was to demonstrate the etiologic spectrum and long term follow-up results of pediatric non-infectious uveitis cases.

**Methods:** The files of patients with the diagnosis of non-infectious uveitis were reviewed between May 2010 and September 2017.

**Results:** The cohort consisted of 54 juvenile uveitis cases. Mean duration of follow-up for uveitis was 18.6 months (6-60 months). Twenty seven (50%) patients had JIA, 17 (31%) patients had idiopathic uveitis, 6 patients (11%) had BD, and 4 patients (7%) had TINU. The frequency of uveitis in all JIA patients was 6.6% (27/405), and in BD it was 22.2% (6/27).

Juvenile idiopathic arthritis cases consisted of 18 (67%) persistent oligoJIA, 3 extended oligoJIA, 2 enthesitis-related arthritis (ERA), 2 RF-negative polyarticular JIA, 1 psoriatic arthritis, and 1 systemic JIA. Five JIA cases had uveitis at the time of diagnosis of arthritis (4 persistent oligoJIA, 1 RF-negative polyarticular JIA). Only one case had initial diagnosis of uveitis and later diagnosed as ERA. Mean duration between diagnosis of JIA and uveitis was 21.2 months (0-77 months).

Male and female ratio was close to each other in the whole cohort (26 males, 28 females) and in JIA cases (12 males, 15 females). While idiopathic uveitis cases (6 males, 11 females) had female predominance, BD (5 males, 1 female) and TINU (3 males, 1 female) cases had male predominance.

Mean age at the time of diagnosis of uveitis was 9.1 years (1-17.5 years) in the whole cases and this number was 5.0 years in persistent oligoJIA cases, 11.8 years in idiopathic uveitis, and 13.7 years in BD.

Bilateral anterior uveitis was observed in 20 (74%) of JIA cases. Six JIA patients had unilateral anterior uveitis and one case had intermediate uveitis. Anti-nuclear antibody was positive in 90.4% of oligoarticular JIA-uveitis cases and 95.2% of oligoJIA associated uveitis cases were asymptomatic.

Idiopathic uveitis cases had unilateral anterior uveitis (6 cases), bilateral anterior uveitis (5 cases), bilateral intermediate uveitis (5 cases), and bilateral panuveitis (1 case). All of the idiopathic uveitis cases were symptomatic as red eyes being the most common symptom. Three BD patients had bilateral panuveitis, 2 cases had unilateral anterior and 1 case had bilateral anterior uveitis. Only one BD case was asymptomatic. TINU cases had bilateral anterior uveitis (3 cases) and unilateral anterior uveitis (1 case) and all of them had red eyes.

At the time of enrollment 45 uveitis cases (83.3%) were under remission while 9 cases (5 idiopathic uveitis, 2 JIA, 1 BD, and 1 TINU) had active uveitis. Biologics were used in 14 cases of methotrexate-resistant JIA-related uveitis (adalimumab in 11 and tocilizumab in 3 cases). Half of BD uveitis patients were treated with biologics (2 cases with interferon-alpha, 1 case with adalimumab).

Complications of uveitis were observed in 10 cases (18.5%) such as intraocular hypertension (5 cases), adhesions (2 cases), band keratopathy (2 cases) and decreased visual acuity (1 case).

**Conclusion:** Patients with JIA and BD should be regularly checked for uveitis. It is hard to find an etiology in uveitis cases that were sent from ophthalmologist if initial examination and questioning do not reveal an overt rheumatologic disease but simple urine test may help in diagnosis of TINU.

**Disclosure of Interest**

None Declared

### P112 JUVENILE IDIOPATHIC ARTHRITIS, UVEITIS AND ANTI-DFS70 ANTIBODIES – AN UNDEFINED RELATIONSHIP

#### Federica Martinis^1^, Sara Pieropan^1^, Gloria Dallagiacoma^1^, Eugenia Bertoldo^1^, Federico Caldonazzi^2^, Domenico Biasi^1^, Maurizio Rossini^1^, Caterina Mansoldo^3^

##### ^1^Rheumatology Unit, AOUI Verona, Verona; ^2^Paediatric Unit, Ospedale di Rovereto, Rovereto; ^3^Ophthalmology Unit, AUOI Verona, Verona, Italy

###### **Correspondence:** Gloria Dallagiacoma

**Introduction:** Juvenile idiopathic arthritis (JIA) is the most common rheumatic disease of childhood and uveitis is one of the major extra-articular manifestations. Uveitis occurs more frequently in children with antinuclear antibodies (ANA) positive disease and is often asymptomatic and may lead to permanent visual impairment, if not treated properly. ANA is the only biomarker that currently guides uveitis screening in JIA but it remains not conclusive. Among various ANA patterns, anti-DFS70 antibodies has been proposed as a novel biomarker for uveitis screening, being positive in children likely to develop uveitis. By contrast, in adults these antibodies seem to identify healthy individuals among asymptomatic ANA positive subjects.

**Objectives:** Aim of this observational study was to evaluate the correlation between uveitis and anti-DFS70 antibodies in children affected by JIA.

**Methods:** A total of 34 children (22 females, 12 males; median age 13.3 years) admitted to Rheumatology Unit of Verona and affected by JIA were enrolled. Following data were recorded retrospectively: JIA subtype, ANA positivity, anti-DFS70 positivity, presence of uveitis, diagnosed by a well-trained ophthalmologist.

**Results:** In our series oligoarticular ANA+ JIA was the predominant subtype (23 cases, 67.6 % of total), followed by oligoarticular ANA- subtype (6 cases, 17.6 % fo total), polyarticular ANA- JIA (3 cases, 8.8% of total), and lastly polyarticular ANA+ JIA (2 cases, 5.9% of total).

23 (67.8% of total) patients presented a DFS/homogeneous ANA pattern. 6 out of 34 patients (17.6%) developed mono- or bialteral uveitis and all of them were ANA positive, confirming that DFS/homogeneous ANA pattern is the most common pattern associated to uveitis. None of patients presented antibodies anti-DFS70, regardless of clinical history of uveitis.

**Conclusion:** DFS/homogeneous ANA pattern remains the most common pattern seen in JIA patients and seems to be an hallmark of uveitis. In our series, we found that neither JIA nor uveitis risk in JIA patients correlate with anti-DFS70 positivity. The role of these antibodies in children remains thus unclear. Further studies are needed to identify a reliable biomarker to guide ophthalmologic screening in JIA patients and to identify children likely to develop uveitis.

**Disclosure of Interest**

None Declared

### P113 CHOIСE AND SWITCH BETWEEN BIOLOGICAL AGENTS IN JUVENILE IDIOPATHIC ARTHRITIS WITH UVEITIS

#### Anna Ignatova^1^, Nina Seylanova^2^, Elena Zholobova^3^

##### ^1^5th year student; ^2^3'd year student; ^3^Pediatric Rheumatology, Federal State Autonomous Educational Institution of Higher Education I.M. Sechenov First Moscow State Medical University of the Ministry of Health of the Russian Federation, Moscow, Russian Federation

###### **Correspondence:** Anna Ignatova

**Introduction:** Chronic anterior uveitis (CAU) is the most common extra-articular sign of juvenile idiopathic arthritis (JIA) that can result in a serious impairment and/or loss of vision in children. Standard antirheumatic drugs in combination with local therapy are effective in 60%. When these drugs are ineffective, biological agents are used in JIA with CAU. The issue of optimal and personalized biologic therapies prescription and problem of switching between drugs are relevant questions of modern pediatric rheumatology.

**Objectives:** The aim of this retrospective observation was to describe trends in the biologic DMARDs prescription in first and subsequent lines of biologic therapies for the treatment of JIA associated with CAU.

**Methods:** We recruited 53 patients with JIA and eye involvement, who received medical treatment at the Department of Pediatric Rheumatology of Sechenov University since January 2015 to December 2017. Study group included 39 girls and 14 boys (2,8/1). Mean age was 11,19 ± 3,87 years, age of disease onset was 4,80 ± 3,06 years; mean disease duration was 6,39 ± 3,45 years. Among included patients 28 children (53%) had oligoarticular JIA, 25 children (47%) – polyarticular RF- JIA. ANF was positive in 36 patients (68%). Disease onset with initial joint damage was observed in 35 children (66%), with eye involvement – in 7 children (13%), with both joint damage and eye involvement – in 11 children (21%). Thirty-eight (72%) patients had bilateral ocular involvement, 15 patients (28%) – unilateral.

At baseline, 51 patients (96%) received concomitant therapy with non-biological immunosuppressive drugs: 49 children (96%) received Methotrexate, 2 children (4%) Cyclosporine A. Two children (4%) received biologic DMARD as monotherapy. All patients received local therapy due to the ophthalmologist recommendations.

**Results:** As a first biologic abatacept was used in 10 patients, etanercept in 8 patients, infliximab in 1 patient. Adalimumab was used in 34 patients (64,15%±8,23), which was significantly more frequently than abatacept, etanercept and infliximab as a whole – 19 (35,85%±11,01) (t=2,06; P>95,5%).

Later 13 children (24,53%) were switched from a first to a second biologic agent. 11 patients were switched from the other biologics to adalimumab. The main reasons for switching was the appearance of CAU in 4 patients (36%), who were initially treated with etanercept, and aggravation of CAU in 3 patients (27%), 2 of them received etanercept as a first biologic and 1abatacept. The second reason for switching was lack of therapeutic response or inefficacy (in 4 – 36%): 3 patients were initially treated with abatacept and 1 with infliximab. Adalimumab was discontinued due to inefficiency only by 1 patient. One patient discontinued adalimumab due to remission. At the end of our observation alimumab was chosen as treatment for 43 (81,13%) patients with JIA and CAU.

**Conclusion:** Thus, adalimumab was preferred as a first- and second-line biologic agent in treatment of JIA with CAU.

**Disclosure of Interest**

A. Ignatova: None Declared, N. Seylanova: None Declared, E. Zholobova Consultant for: AbbVie, Roche, BMS, Pfizer, Novartis, MSD

### P114 UVEITIS IS RISK FACTOR OF SIGNIFICANT FLARE IN NON-SYSTEMIC JUVENILE IDIOPATHIC ARTHRITIS ON THE FIRST BIOLOGICS.

#### Mikhail Kostik, Ekaterina Gaidar, Lyubov Sorokina, Ilia Avrusin, Elizaveta Orlova, Yuri Korin, Margarita Dubko, Vera Masalova, Tatyana Likhacheva, Ludmila Snegireva, Eugenia Isupova, Olga Kalashnikova, Vyacheslav Chasnyk

##### SAINT-PETERSBURG STATE PEDIATRIC MEDICAL UNIVERSITY, Saint-Petersburg, Russian Federation

###### **Correspondence:** Mikhail Kostik

**Introduction:** Treatment of moderate-severe form of juvenile idiopathic arthritis (JIA) required using biologics in the cases of methotrexate inefficacy or intolerance. The presence of extra-articular features, especially uveitis usually required more intensive treatment approaches. The finding of predictors of biologics efficacy is still actual problem.

**Objectives:** to evaluate the role of uveitis as a risk factor of flare and survival of first biologic medication in non-systemic JIA.

**Methods:** Inclusion criteria: patient whom first biologic was administrated with or without MTX or MTX was discontinued after start of biologics due to different reasons (remission, intolerance, adverse events). Exclusion criteria: treatment with current systemic corticosteroids, infliximab, rituximab. After selection 175 patients were eligible to analysis. We evaluate clinically significant flare with joint involvement (required change of biologic or non-biologic DMARD), time to flare. We compare two groups: i) patients with uveitis (n=32) and ii) patients without uveitis. For statistical analysis we Cox’s regression models, Log-Rank test, x2 test and Mann-Whitny test.

**Results:** the data of comparison between groups depending on the uveitis in the Table 1. There was no difference in the gender distribution, and achievement of remission. The main biologic in non-uveitis group was etanercept (64.3%), in uveitis group – adalimumab (71.9%). Presence of uveitis increases the risk of JIA flare: OR= 3.8 (95%CI: 1.7; 8.7), and cumulative probability of flare: RR=4.5 (95%CI: 1.7; 12.1), р=0.003; Log Rank test, p=0,001; after adjustment on methotrexate RR=3.1 (1.6; 6.0), p=0.0008. In subgroup of patients treated with adalimumab absence of methotrexate increases the cumulative probability of flare RR=6.5 (95%CI: 1.4; 31.1), р=0.02.

**Conclusion:** presence of uveitis was assumed as risk factor of JIA flare, methotrexate can decrease the cumulative flare probability. Further trials are required.

**Disclosure of Interest**

None Declared

**Table 1 (abstract P114).** See text for description

Parameters	Uveitis, yes n=32	Uveitis, no n=143	Р
Onset age, years	3.9 (2.1; 6.2)	5.5 (2.5; 10.5)	0.033
ANA positivity, n (%)	15/26 (57.7)	26/72 (36.1)	0.056
Ongoing methotrexate, n (%)	26 (81.3)	103 (72.0)	0.284
Time before 1st biologics, months	2.9 (1.5; 5.3)	2.2 (1.0; 5.2)	0.343
Remission duration, months	6.5 (2.4; 6.9)	13.1 (6.5; 28.0)	0.078
First significant flare, n (%)	14 (43.8)	24/142 (16.9)	0.0009
Time before first significant flare, months	33.0 (16.7; 61.5)	48.6 (26.4; 65.7)	0.023
Switching of the first biologic, n (%)	7 (21.9)	18/142 (12.7)	0.180
Time before switching of the first biologics, months	49.0 (24.5; 62.1)	52.3 (35.0; 66.2)	0.186

### P115 LONG-TERM OUTCOME AND COMPLICATIONS IN UVEITIS, 18 YEARS AFTER DISEASE ONSET IN A POPULATION-BASED NORDIC JUVENILE IDIOPATHIC ARTHRITIS COHORT

#### Veronika Rypdal^1^, Mia Glerup^2^, Terje Christoffersen^3^, Geir Bertelsen^3^, Ellen Dalen Arnstad^4,5^, Kristiina Aalto^6^, Suvi Peltoniemi^6^, Lillemor Berntson^7^, Maria Ekelund^7,8^, Anders Fasth^9^, Troels Herlin^2^, Susan Nielsen^10^, Peter Toftedal^10^, Rasmus Nielsen^11^, Sanna Leinonen^12^, Regitze Bangsgaard^13^, Susanne Lindqvist^14^, Marite Rygg^5,15^, Ellen Nordal^1^, the Nordic Study Group of Pediatric Rheumatology (NoSPeR)

##### ^1^Department of Pediatrics, University Hospital of North Norway, Tromsø, Norway; ^2^Department of Pediatrics, Aarhus University Hospital, Aarhus, Denmark; ^3^Department of Ophthalmology, University Hospital of North Norway, Tromsø; ^4^Department of Pediatrics, Levanger Hospital, Levanger; ^5^Department of Clinical and Molecular Medicine, NTNU, Trondheim, Norway; ^6^Department of Pediatrics, University of Helsinki, Helsinki, Finland; ^7^Department of Women’s and Children’s Health, Uppsala University, Uppsala; ^8^Department of Pediatrics, Ryhov County Hospital, Jonkoping; ^9^Department of Pediatrics, Institute of Clinical Sciences, Sahlgrenska Academy, University of Gothenburg, Gothenburg, Sweden; ^10^Department of Pediatrics, Rigshospitalet Copenhagen University Hospital, Copenhagen; ^11^Department of Ophthalmology, Aarhus University Hospital, Aarhus, Denmark; ^12^Department of Ophthalmology, University of Helsinki, Helsinki University Hospital, Helsinki, Finland; ^13^Department of Ophthalmology, Rigshospitalet Copenhagen University Hospital, Copenhagen, Denmark; ^14^Department of Ophthalmology; ^15^Department of Pediatrics, St. Olavs Hospital, Trondheim, Norway

###### **Correspondence:** Veronika Rypdal

**Introduction:** Uveitis is the most common extra-articular manifestation in children with juvenile idiopathic arthritis (JIA). Early identification and treatment is important in order to prevent sight-threatening complications and ongoing disease activity in to adulthood. Long-term population-based data on uveitis course, outcome and complications are sought for.

**Objectives:** The aim was to assess long-term course, outcome and complications of uveitis in the Nordic JIA cohort 18 years after disease onset.

**Methods:** A multicenter longitudinal prospective Nordic JIA cohort study in which 510 patients from defined geographical areas of Denmark, Finland, Sweden and Norway with disease onset in 1997-2000 were included. The study aimed to be as population-based as possible. At the 18-year follow-up 434 patients were included. Uveitis information was available for 431 participants, and 272 were examined by an ophthalmologist at the study visit. Otherwise information about uveitis and medication was collected by the pediatric rheumatologist.

**Results:** Uveitis developed in 94/431 (22%) patients during the observation period of 18 years. Non-symptomatic uveitis was found in 73% of the patients, and 84% had an insidious onset of their uveitis. Anterior uveitis was described in 70%. Among the uveitis patients 83% had received treatment with methotrexate and 58 % treatment with biologics at some point during the disease course. At the final study visit 36% had ongoing local treatment with eye drops, while 32% used systemic medication due to, or partly due to, uveitis. 7/94 (8%) had a best corrected visual acuity ≤0.5 in at least one eye. Cataract developed as a complication in 25/94 (27%) and glaucoma in 22/94 (23%). Surgery was performed for cataract in 20/25 (80%) and for glaucoma in 18/22 (82%) of the patients.

**Conclusion:** We found a high incidence of uveitis among nordic young adults with JIA. Cataract and glaucoma were the most common structural complications. For the majority of the young adults with these complications surgery was needed within 18 years after onset of JIA, pointing to severe implications of uveitis in a life-long perspective.

**Disclosure of Interest**

None Declared

### P116 THE SPECTRUM OF NON-INFECTIOUS UVEITIS IN CHILDREN MANAGED AT A TERTIARY PAEDIATRIC RHEUMATOLOGY SERVICE IN CAPE TOWN

#### Waheba Slamang, Christopher Tinley, Christiaan Scott

##### Red Cross War Memorial Children's Hospital and University of Cape Town, Cape Town, South Africa

###### **Correspondence:** Waheba Slamang

**Introduction:** Non-infectious uveitis is a leading cause of blindness in the developed world. Juvenile idiopathic arthritis associated uveitis (JIA-U) is one of the most commonly reported immune mediated cause of uveitis in children. However, as in other developing countries, infectious uveitis is more common in Africa and there is a paucity of data on the epidemiology of non-infectious uveitis in children in this setting. To our knowledge, this is the first description of non-infectious uveitis managed at a tertiary paediatric rheumatology service from sub-Saharan Africa.

**Objectives:** To describe the disease characteristics and treatment of children with non-infectious uveitis at a tertiary paediatric rheumatology service in Cape Town

**Methods:** A retrospective analysis of children managed by the paediatric rheumatology and ophthalmology service for uveitis from 1 January 2010 to 31 December 2017, was conducted. Ethics approval was obtained, and relevant data extracted from patient medical rheumatology and ophthalmology case files. Descriptive statistics were employed and comparisons between JIA-U and idiopathic uveitis groups were made, with p-values < 0.05 considered significant.

**Results:** Thirty-four children (60 eyes) were reviewed - 18 boys and 16 girls with a median age at first visit of 76 months (age range 25 to 156 months).

Chronic anterior uveitis predominated in 23 (68%), acute anterior uveitis occurred in 3 (9%), panuveitis in 4 (14%), followed by 3 (9%) posterior, and 1 (3%) intermediate uveitis.

Fourteen (41.1%) were associated with juvenile idiopathic arthritis (JIA), 12 (35.3%) were considered idiopathic, while 2 (5.8%) were due to sarcoidosis, 1 (3%) to Behcet’s, 2 (5.8%) to human immune deficiency virus (HIV), 2 (5.8%) post streptococcal and 1 (2.9%) to toxocara.

The JIA-U group were characterised by a female preponderance, median age of onset of 56 months and chronic anterior uveitis. Further review according to the International League of Association for Rheumatology (ILAR) classification showed 10 (71,4%) to be oligo-persistent antinuclear antibody (ANA) positive while 3 (21,4%) were poly-articular rheumatoid factor negative and 1 (7,1%) had psoriatic-JIA.

9 (64.2%) of the JIA-U group and 8 (73%) of the idiopathic group presented with complications, predominantly cataracts, followed by band keratopathy and posterior synechiae. There was no significant difference in age and complications at presentation, between the two groups, p-values 0.4 and 0.62 respectively, though a male preponderance was evident in the idiopathic group.

All children were treated with standard initial therapy which included steroids (topical, oral and/ or intravenous) and methotrexate, of which 21 (61.7%) achieved remission. Thirteen children required the addition of a tumour necrosis factor (TNF) inhibitor, due to failure of Mycophenolate Mofetil in 2 and Azathioprine in 7. Eight (61.5%) achieved remission (Table 1).

**Conclusion:** Here, JIA-U appears to have similar disease characteristics as previously described. However, the high rate of complications at presentation, requires further review of overall visual outcomes and the effectiveness of current screening and treatment strategies. Optimum management of non-infectious uveitis therefore necessitates a close relationship between paediatric rheumatologists and ophthalmologists.

**Disclosure of Interest**

W. Slamang Grant / Research Support from: Abbvie, Pfizer, C. Tinley: None Declared, C. Scott Grant / Research Support from: Abvvie, Pfizer. Roche

**Table 1 (abstract P116).** TNF inhibitor treatment

Uveitis Group	Infliximab N=7 *(Remission)*	Adalimumab N= 6 *(Remission)*
Idiopathic	4 *(3)*	5 *(3)*
JIA-U	3 *(1)*	1 *(1)*

### P117 EFFICIENCY AND SAFETY EVALUATION OF BIOSIMILAR INFLIXIMAB FOR TREATMENT OF PEDIATRIC NON-INFECTIOUS UVEITIS IN SINGLE CENTER

#### Betul Sozeri^1^, Esra Kardes^2^, Gizem Leyla Bolac^1^, Betul Ilkay Sezgin Akcay^2^

##### ^1^Pediatric Rheumatology; ^2^Ophtalmalogy, University of Health Sciences, Istanbul, Umraniye Training and Research Hospital, Istanbul, Turkey

###### **Correspondence:** Betul Sozeri

**Introduction:** Biosimilar infliximab (Remsima®) has been introduced in our country, together with other European countries in 2014. Information regarding the use in child age group is limited but it is reported that its therapeutic efficiency and safety is similar to the reference molecule regarding the pediatric Chron disease

**Objectives:** In this study, our aim was to reporte the efficiency and safety of BI used for children with non-infectious uveitis.

**Methods:** In this study, there were 13 subjects (9 boys, and 4 girls) diagnosed with non-infectious uveitis.

BI (Remsima9 treatment had been given in 5 mg/kg in 0.,2.,4 th week and then every 8^th^ week. Ophthalmic assessment of disease activity and ocular complications were measured throughout the trial with the use of slit-lamp biomicroscopy for uveitis activity, according to the SUN criteria. The Drug exposure has been evaluated by calculated of patient year (HY), adverse event (AE) was assessed using the CTCAE criteria. The median values due to the small number of patients are considered.

**Results:** The patients who were include the study, 5 had diagnosed extended oligo JIA, 2 with enthesitis-related arthritis (ERA) ,2 with persistent oligo JIA, 2 with pars planitis and one of them Behcet’s disease. At the time of evaluation, the median age was 10 years (3-13), age at diagnosis of their disease 8 years (1-13), respectively. The median age of uveitis diagnosis was 8 years. The median disease duration before BI was 10 months. All of the patients had methotrexate therapy with BI and BI before.

Only one patient used a different biological agent prior to BI, and revealed they were determined that considered unresponsive.

Other patients (n=12) had biosimilar infliximab as first biologic drug due to the activation of disease after using the drug methotrexate.

After therapy of BI, in all of the patients, joint and eye symptoms were improvement.

The systemic steroid therapy was cut down in the first month in all patients, 2 of them continue prophylactic topical steroids.

The median duration of BI therapy was 10 months. There was one case of anaphylaxis in all the patients, whereas five of them frequent upper respiratory tract infection have been observed as side effect.

**Conclusion:** In this preliminary report, This biosimilar infliximab treatment appears to be safe and effective in pediatric age group on the pediatric patients with non-infectious uveitis. These results must be supported by multicenter studies and registries.

**Disclosure of Interest**

None Declared

### P118 FIRST MULTI-CENTER SURVEY FOR THE TREATMENT IN JUVENILE IDIOPATHIC ARTHRITIS-ASSOCIATED UVEITIS (JIA-U) AT CLINICAL SETTINGS IN JAPAN; THE REPORT FROM PEDIATRIC ASSOCIATION OF JAPAN (PRAJ)

#### Masato Yashiro^1^, Junko Yasumura^2^, Nami Okamoto^3^, Kosuke Shabana^3^, Yuka Okura^4^, Hiroaki Umebayashi^5^, Minako Tomiita^6^, Kenichi Nishimura^7^, Naomi Iwata^8^, Masaki Shimizu^9^, Mao Mizuta^9^, Tomohiro Kubota^10^, Syuji Takei^11^, Masaaki Mori^12^

##### ^1^Pediatrics, OKAYAMA UNIVERSITY HOSPITAL, Okayama; ^2^Pediatrics, Hiroshima University Graduate School of Biomedical & Health Sciences, Hiroshima; ^3^Pediatrics, Graduate School of Medicine, Osaka Medical College, Takatsuki; ^4^Pediatrics, KKR Sapporo Medical Center, Sapporo; ^5^Rheumatics, Miyagi Children’s Hospital, Sendai; ^6^Allergy and Rheumatology, Chiba Children's Hospital, Chiba; ^7^Pediatrics, Yokohama City University Graduate School of Medicine, Yokohama; ^8^Infection and Immunology, Aichi Children’s Health and Medical Center, Obu; ^9^Pediatrics, Graduate School of Medical Sciences, Kanazawa University, Kanazawa; ^10^Pediatrics, Graduate School of Medical and Dental Sciences, Kagoshima University; ^11^Pediatrics, Graduate School of Medical and Dental Sciences, Kagoshima University, Kagoshima; ^12^Lifetime Clinical Immunology, Graduate School of Medical and Dental Sciences, Tokyo Medical and Dental University, Tokyo, Japan

###### **Correspondence:** Masato Yashiro

**Introduction:** There are no established treatment guidelines for patients with juvenile idiopathic arthritis-associated uveitis (JIA-U).

**Objectives:** Therefore, multi-center survey was conducted to examine the present treatment status for JIA-U in Japan to establish treatment guideline in the future.

**Methods:** Questionnaires were sent to medical centers with pediatric rheumatologists at April in 2016 to investigate the patients’ profile and how JIA-U was treated in each center.

**Results:** Of 726 patients involved in this study, 44(6.1%) had JIA-U and 36/44 (81.8%) was oligoarticular onset. Of 40 JIA-U patients recruited by the 2^nd^ detailed survey, 23 (57.5%) had been treated with NSAIDs in 13 (32.5%), MTX in 16 (40.0%), glucocorticoids (GCs) in 1(2.5%), and etanercept (ETN) in 1 (2.5%) by the time when JIA-U was diagnosed. After JIA-U was diagnosed, topical GC was started in all cases in combination with MTX in 3(7.5%) and/or systemic GC in 2(5.0%). Some biologic agents (infliximab (IFX) and adalimumab (ADA)) were used more frequently in JIA-U patients than non-uveitis patients (P <0.0001, P = 0.0029). Of 40 JIA-U patients, 22 patients (55.0%) were treated with biologic agents such as IFX in 9(22.5%), and ADA in 11 (27.5%). Surgical treatments for JIA-U such as intraocular lens implantation underwent in 15 cases (37.5%). Symptoms and findings related to uveitis improved with treatment in all 40 JIA-U patients, and no blindness was observed in this survey.

**Conclusion:** Treatment for JIA-U in real clinical settings in Japan was similar to that of previous reports from other countries. Higher incidence of JIA-U onset in MTX treated patients in Japan may be explained by the differences in administration dose or method. Further investigation with larger scale is essential to establish the evidence-based treatment recommendation for JIA-U.

**Disclosure of Interest**

None Declared

## Poster Walk 5: HPPR - e-health and digital health applications

### P119 SMARTPHONE APPLICATION “MY PATHWAY” GUIDING THE YOUNG PATIENT WITH JIA THROUGH TRANSITION TO ADULT MEDICINE.

#### Anette Pieler, Heidi K. Ipsen, Anne E. Christensen, Peter Toftedal

##### H.C. Andersen Childrens Hospital, Odense University Hospital, Odense, Denmark

###### **Correspondence:** Heidi K. Ipsen

**Introduction:** In 2014 the Region of Southern Denmark created the application (app), “Mit forløb” (“My Pathway”) giving the different departments at the hospitals, the option to create their own specific section of the app. The app is available for everybody but using the communication and registration tools requires access granted by the relevant department.

“My Pathway” is patient empowerment put into practice because the patients can be more involved in their own treatment and gain knowledge about different aspects of their disease and management thereof.

**Objectives:** To provide the young patients with JIA an easy and accessible way to find information on their disease, treatment and rights before and during transition to adult medicine a smart phone app was created.

**Methods:** The information in the app was written in collaboration between health professionals from the pediatric department and the department of Rheumatology, securing a uniform structure and language. It covers JIA, pain management, treatments and their side effects. Also included is general information on the department, its structure and staff.

A chat function was developed which enables communication with the hospital in a flexible way; the patient can write whenever the question occurs and the hospital staff can answer when they have the time and have looked into the patient journal.

“My Pathway” meets the young patient where they are; online anytime and anywhere via the smartphone in their pocket. Regarding transitioning focus was put on patients’ rights and the app was created to it could be accessed both as a patient at the pediatric department and the department of Rheumatology.

**Results:** The app is still a very new initiative and has not yet been widely spread to the young patients. Around 30 young patients have been granted access to the app, and the feedback has been positive.

Feedback from the patients has been a wish for more personalized information, regarding treatment and consultations, and to be able to see future appointments.

As of yet the chat feature has not been in much use probably due to reduced awareness among families and staff.

**Conclusion:** The pediatric team succeeded in creating an app geared towards informing the young patients about JIA, medication, pain-management, transfer to the department of Rheumatology etc.

As the app is a new implementation there must be expected a period of time for adjusting, both for patients and health professionals.

There is need for further development for the app to achieve full potential. Adapting the feedback from the young patients and the health professionals working with the app is an ongoing process.

The app is planned to include all rheumatic diagnoses and also access for younger children and their parents in the future.

**Disclosure of Interest**

None Declared

### P120 REUMA2GO, A PLATFORM TO EMPOWER PATIENTS, PARENTS AND CAREGIVERS BY IMPROVING, DIALOGUE, PARTICIPATION AND MONITORING DISEASE BURDEN

#### Mark Klein^1^, Joost Swart^1^, Astrid Willemsen^1^, Gerrie Joode^2^, Annemarijn van Rheenen^3^, Vicky Seyfert^4^, Nico Wulffraat^1^

##### ^1^Pediatric Rheumatology, Wilhelmina Children’s Hospital, University Medical Center Utrecht; ^2^Pediatrics; ^3^IT, University Medical Center Utrecht, Utrecht, Netherlands; ^4^MyOwnMed, Bethesda, USA

###### **Correspondence:** Mark Klein

**Introduction:** Juvenile idiopathic arthritis (JIA) is the most common chronic rheumatic disease of childhood requiring long-term treatment. JIA has an fluctuating and unpredictable course. The fluctuation of disease activity, asks for continuous monitoring by a pediatric rheumatologist to achieve good health outcomes. Children with JIA live with chronic or recurrent pain and disability, which can severely limit their ability to complete daily physical tasks and participate in school and social activities.

An app was developed with input of patients, parents and caregivers to help close the gap between hospital care and the normal daily life activities of children with JIA in between hospital visits.

**Objectives:** To create an ehealth platform (Reuma2GO) that aims to significantly improve the care for children with JIA and the dialogue between children with JIA, their parents and team of caregivers and hence, to advance true patient participation both in care and research.

**Methods:** We performed a two-day workshop which was attended by patients, parents, app developers and caregivers about the added value of eHealth for the JIA patient and their parents. The participants were asked what they wanted from an app in their therapy that they are not currently finding. The workshop participants then worked together to come up with design and concept features that their ideal app should have and identify the critical success factors giving app developers a clear, clinically-approved model to work to.

The Reuma2GO app tracks joint pain, fatigue, morning stiffness, daily activities and manages appointments and medication. Patients can communicate with health professionals in the app for advice or questions. Weekly feedback is sent on the self-reports by the care team to support patients and parents. The patient-generated data in the app will be integrated into the EMR in the near future.

**Results:** The Reuma2GO app is developed and tested among patients and care professionals according to the agile scrum method. 180 JIA patients and 7 rheumatologists/immunologists are using the Reuma2GO app. The app was evaluated as helpful in self-management and recommended by patients. The app generates a dynamic reflection of the daily life of a JIA patient. This allows patients to efficiently monitor the impact and actual status of their disease.

**Conclusion:** Reuma2GO is an eHealth platform that enables JIA patients to better understand and monitor the burden of the disease and thereby providing patients a way to become more actively involved in their disease management. By sharing their data in between hospital visits both patients and care professionals should benefit by offering more personalized care and using monitored data for research. Planning is to scale up the app to other hospitals in Europe that participate in the PRES network in the coming period.

**Disclosure of Interest**

None Declared

### P121 REUMA2GO-APP CREATES A CONTINUUM OF CARE

#### Mark Klein^1^, Joost Swart^1^, Sytze Roock^1^, Astrid Willemsen^1^, Nathan Buijsse^1^, Gerrie Smink^1^, Annemarijn van Rheenen^2^, Vicky Seyfert^3^, Nico Wulffraat^1^

##### ^1^Pediatric Rheumatology, Wilhelmina Children’s Hospital, University Medical Center Utrecht; ^2^IT, University Medical Center Utrecht, Utrecht, Netherlands; ^3^MyOwnMed, Bethesda, USA

###### **Correspondence:** Mark Klein

**Introduction:** From the patients perspective, the big promise of health apps (mHealth) is to increase self-awareness and self-care. For chronic conditions such as juvenile idiopathic arthritis (JIA), mHealth may provide a way for patients to become more actively involved in their disease management. With input of patients, parents and caregivers the Reuma2GO app was developed in 2017. It was intended to close the gap between hospital care and the normal daily life activities of children with JIA. The Reuma2GO app creates insights for patient, parents and caregivers into all aspects of JIA that influence the burden of disease (e.g. joint pain, fatigue, morning stiffness, daily activities, medication use, side effects). By offering the possibility of instant reporting about health issues one does not have to rely on their memory for conditions that were present weeks before the routine visit. Moreover the app allows for built-in thresholds with automatic alerts to physicians. A closed communication system allows the patient to open discussions with their physician.

We feel that some routine visits of JIA patients, when in a stable inactive episode (either on or off medication), can be skipped as long as the patients agree that they are still doing fine.

**Objectives:** To evaluate if patients/parents are capable of self-monitoring with the Reuma2GO app when they feel the disease is quiet in order to skip future hospital visits.

**Methods:** Data from the Reuma2GO-app, the JAMAR at the hospital visit (question 6: “Considering all the symptoms…. evaluate the level of activity of your child’s illness at the moment”) and the physician’s global assessment of disease activity (PGA) were compared.

The data used from the Reuma2Go app were the following (total score 0-40)

1. How much pain did you have in the past week (0-10)?
2. Do you have pain and/or swelling in your joints today (max 10 joints)?
3. Did you suffer from morning stiffness in the last week (5 step-scale; 2 points per step)?
4. How active is your illness at this moment (0-10)?

We only included those patients that had completed the abovementioned questions in the Reuma2Go-app 0-3 weeks before the routine visit and scored a maximum of 1 point in total and only allowed for question 1 and 4 (=Wallace’s criteria for inactive disease according to parent/patient).

**Results:** 186 (128 Female) patients aged 12.07±4.27 years use the Reuma2GO app.

The distribution of JIA categories was: 19.4% polyarticular RF-negative, 6.9% enthesitis-related arthritis, 3.5% polyarticular RF positive, 11.8% extended oligoarticular, 40.3% persistent oligoarticular, 5.6% systemic JIA, 4.9% artritis psoriatica and 7.6% had undifferentiated arthritis.

Preliminary data show that 17 patients completed the questions and considered the JIA to be inactive. In 16 (94%) of the cases the physician confirmed that the JIA was inactive and the JAMAR question on disease activity reflected the same. Only one patient score a 1/10 at the JAMAR during the visit, while the app scored a 0 before. This patient was suspected to have still a bit of unilateral TMJ arthritis and received a PGA of 1/10 as well, although no change in medication was performed

**Conclusion:** The Reuma2Go-app creates a continuum of care by which the physician does not need to make treatment plans depending on one-point in time conditions at prescheduled visits. Moreover 94% of the patients that felt their disease was quiet were right about it. The only one that changed his/her mind at the visit was backed up by the physician, although no treatment change was needed. Future studies will look into the safety of giving autonomy to patients when they feel a visit can be skipped.

**Disclosure of Interest**

None Declared

### P122 THE TRIALS AND TRIBULATIONS OF DEVELOPING AN ACTIVITY DIARY APP FOR CHILDREN AND YOUNG PEOPLE WITH PERSISTENT PAIN

#### Claire L. Pidgeon

##### Rheumatology Service, Birmingham Women's and Children's Hospital, Birmingham, UK

**Introduction:** Identifcation of a need to replace a paper based activity diary used as part of pain management intervention with a more accessible and user friendly smart technology resource. Researchers within the field of E-technology have demonstrated the use of smart phones for self-monitoring pain and symptoms preferable to paper based forms noting that the information recorded was more accurate and time efficient. Pain diary apps exist however they tend to focus on reporting and recording pain rather than activity levels. At the time of development there were no activity diary apps specifically aimed at children and young people.

**Objectives:** To develop an interactive user-friendly e-diary app that is accessible on android and iOS smart mobile phone.

**Methods:** At concept a business plan was submitted to secure financial funding and obtained authorisation clinical design group within NHS trust. Consultation with multi-disciplinary team and user groups on the style, content, usability. This led to consultations with the nominated app developer to bring the concept to wireframe draft format. Educational content was then researched and drafted. Further consultation with user-groups to sign off the final draft of the app.

**Results:** The development process was initially estimated to be completed within 12 months. In fact the entire process was 3 years in duration, underestimating the volumes of work and time that would be involved. A particularly steep learning process for the therapist who was a novice to app development. Despite this editing drafts and trailing the app was required to ensure that all glitches were identifeid and rectified the app is now a the point of final sign off.  The app is at point of release on app store(s). The next phase is to research the efficacy of the app in the following 12 months.

**Conclusion:** Many challenges emerged during the app development process, with important lessons learnt. Technology is ever-evolving at a fast pace. Ensuring the app was relevant was a key driver in the development process, the additional time taken to complete this project increased the risk that the app would be out of date or other activtiy diary apps may become available before the completion date was reached.

Having to use an external organisation to develop the app had limitations in being able to communicate quickly and easily to ensure alterations to the drafts were completed in a timely manner. Future updates that may be required to the app will be limited as this will have to go through the app developer and will have a cost implication.

Improvements emerged during the development phase that were beyond the scope of the app and were not included in the original costings therefore could not be included. It is hoped once the efficacy of the app is researched that further funding can be secured to include the additional functions to the app to build of the scope.

Engagement with user groups was a vital part of the development process providing opinions on the look, and userability of the app interface. The focus group contributed significant modifications to the final app and included setting reminders and a notification facility as well as assisting with naming the app.

The next phase is to research the efficacy of the app in the following 12 months. An essential part of the process is making links with other researchers in this field to continue to the build on the access to evidence-based e-technology tailored specifically for young people.

**Disclosure of Interest**

None Declared

### P123 GENIA- A PATIENT SUPPORT MOBILE HEALTH APP FOR JIA

#### Sara Rostlund,Johanna Kembe

##### Function Area Occupational therapy & Physiotherapy, Allied Health Professionals Function, KAROLINSKA UNIVERSITY HOSPITAL, ASTRID LINDGREN CHILDREN HOSPITALSTOCKHOLM, Stockholm, Sweden

###### **Correspondence:** Sara Rostlund

**Introduction:** Genia is an app created as a patient support system for improved communication and collaboration with patients, families and the health care team. Its designed for IOS so far and is available in app store in both Swedish and English.

**Objectives:** Through the app, patients can take notes and track self-assessed observations in daily living, which could include Visual Analog Scale trackers on various symptoms, activities and other areas reflecting quality of life. Automatic data collection, such as Steps from Apple eHealth, is also included. In addition users can send Pre-Visit report to health care to help both patients/families and care team to prepare for meetings as well as to help understand patients needs and preferences.

**Methods:** A pilot project using the app has been driven by a physiotherapist, occupational therapist and the head physician of the Childrens rheumatology department in Karolinska University Hospital in Stockholm. Currently 56 patients at the clinic have Genia.

**Results:** The feedback on experience from both patient/families and the clinic include that the app:

1. helps patients/families remember, reflect and prepare

2. support clinic visits

3. support patient activation and communication

**Conclusion:** As health professionals we see great potential in the app to get the patient more active, understanding their disease and aware that their behavior and selfcare can make a different in how they feel.

Genia is co-designed with JIA patients, families and members of the care team and continues to be iterated with feedback and ideas provided by stakeholders. There are further opportunities to co-develop the app to be helpful to patients and clinics, for example the creation of different types of reports, new or improved trackers that could support patients or/and assist care teams to support patients in self-care, etc.

**Disclosure of Interest**

None Declared

### P124 ΤHE IMPACT OF A PHYSIOTHERAPY TELE-REHABILITATION PROGRAM ON CHILDREN WITH JUVENILE IDIOPATHIC ARTHRITIS

#### Maria Stavrakidou^1,2,3^, Maria Trachana^2^, Artemis Koutsonikoli^2^, Kyriaki Spanidou^1^, Alexandra Hristara-Papadopoulou^3^, Ioannis Xinias^4^

##### ^1^Pediatric Physiotherapy, Asklepeio Physiotherapy Clinic; ^2^First Dept of Pediatrics, Pediatric Immunology and Rheumatology Referral Center, Hippokration Hospital; ^3^Postgraduate Program in Pediatric Physiotherapy, Alexandrian Technological Educational Institute; ^4^Third Dept of Pediatrics, Hippokration Hospital, Thessaloniki, Greece

###### **Correspondence:** Maria Stavrakidou

**Introduction:** The Juvenile Idiopathic Arthritis (JIA) chronicity, combined with the psychosocial effects of the entire therapeutic process on children and parents, often leads to non-compliance and a significant financial burden. These parameters, in the era of the economic crisis, are an incentive to seek new approaches to physiotherapy.

**Objectives:** To investigate the applicability of a physiotherapy tele-rehabilitation program on children with JIA and to explore the program’s impact on children and their families.

**Methods:** Thirty JIA patients, with Medical Doctor’s global assessment (MDVAS) <2, who applied a home-exercise program, were selected and randomly divided in the tele-rehabilitation (TRG, n=15) and the control group (CG, n=15). Each child from the TRG participated, additionally at home-exercise program, in a 30-minute tele-session (using personal computers, at home) with a qualified pediatric physiotherapist, twice a week, for 12 weeks, performing their exercises under the supervision and guidance of the specialist. Before and after the tele-rehabilitation program (T1 and T2, respectively), TRG and CG patients and a parent/guardian completed the Juvenile Arthritis Multi-demensional Assessment Report questionnaire (JAMAR) and a relevant one for the implementation and compliance on the home-exercise program. Residual disease was estimated at T1 and T2 from a physiotherapist, different from the one performing the tele-rehabilitation program. At T2, TRG patients and their parents completed a questionnaire, evaluating the tele-rehabilitation program. One month after T2, a reassessment of compliance with the home-exercise program was performed.

**Results:** The children’s median (range) age was 12.8 (8-16) years. Nineteen children (63%) had JIA with poly-articular course and 11 (37%) with oligo-articular. All TRG children completed the tele-rehabilitation program. At T2, the TRG children performed the home-exercise program more frequently (p=0.023), for a longer time (p=0.034) and with less promoting (p=0.004), compared to T1. Moreover, there was increased compliance with the home-exercise program (p=0.001), functionality (p=0.008) and quality of life (p=0.007) and less pain due to JIA (p=0.017). In the CG children, there was no statistical significant change in the abovementioned parameters. The residual disease was improved in both groups (TRG: p=0.002, CG: p=0.018), but the improvement in TRG children was greater (p=0.043). The applicability of the tele-rehabilitation program was rated in a 0-10 VAS scale with a median (range) of 10 (7-10) by children and 10 (9-10) by their parents. The tele-rehabilitation program’s total benefit was rated with a median of 10 (8-10) by TRG children and 10 (9-10) by their parents. Finally, one month after T2, the compliance with the home-exercise program was still greater, compared to T1 (p=0.001).

**Conclusion:** The implementation of an interactive physiotherapy tele-rehabilitation program, is applicable and effective in children with JIA, providing an additional tool in their rehabilitation.

**Disclosure of Interest**

None Declared

## Systemic lupus erythematosus and antiphospholipid syndrome

### P125 ESTIMATED GLOMERULAR FILTRATION RATE IN A SINGLE CENTRE CHILDHOOD LUPUS NEPHRITIS COHORT

#### Manjari Agarwal, Sujata Sawhney

##### Division of Pediatric and Adolescent Rheumatology, Sir Ganga Ram Hospital, New Delhi, India

###### **Correspondence:** Manjari Agarwal

**Introduction:** Ongoing proteinuria causes long term damage to the kidney. This is reflected by a change in estimated glomerular filtration rate(eGFR) over a period of time. A fall in eGFR is a poor marker and heralds damage. There is paucity of data about eGFR in children with lupus nephritis. We undertook this study to add to the existing data

**Objectives:** To estimate the eGFR by using the bedside Schwartz formula at the onset of nephritis,at one year after treatment and at last follow up

**Methods:** Charts were reviewed retrospectively of 82 children with lupus nephritis. Height records at disease onset were available for 69 and these were included for analysis. eGFR was calculated using the bedside Schwartz formula: 0.413*(Height/Creatinine).

**Results:** 69 children with lupus nephritis were included for the analysis of eGFR. Two children did not complete one year of treatment and were thus not included for analysis of eGFR at one year and at last follow up.

Median age at onset of nephritis was 12 years. Median duration of follow up of the cohort was 55 months(9-164months).

Median eGFR at onset of nephritis prior to treatment was 82ml/min./1.73m^2^(IQR 63-115). Median eGFR at one year after treatment was 127ml/min/1.73m^2^(IQR 89-150).eGFR at last follow up was 128ml/min/m^2^(IQR 101-145)

The change in eGFR from baseline to one year was statistically significant (p =<0.001) and the change at last follow up visit from baseline was also better and was statistically significant (p=<0.001). However there was no statistically significant change in eGFR from one year to last follow up value (p=0.72).

39 children had eGFR less than 90ml/min/m2 at onset of nephritis and of these 14 had eGFR <60ml/min/m2.After one year of therapy one child had eGFR< 60ml/min./m2 and 18 had eGFR <90ml/min/m2.

39 children had eGFR less than 90ml/min/m2 at onset of nephritis and of these 14 had eGFR <60ml/min/m2.After one year of therapy one child had eGFR< 60ml/min./m2 and 18 had eGFR <90ml/min/m2.

This could imply that aggressive treatment early on led to a significant improvement and there was not much deterioration subsequently.

**Conclusion:** Stringent follow up is practiced at our unit and a tight treat to a target approach is employed. Early aggressive therapy is the norm. This is probably a reason for better outcomes.

Another possibility is that many children have not spent a long time in the disease course and a longer follow up is needed to actually determine the renal damage.

**Disclosure of Interest**

None Declared

### P126 MORBIDITIES AND COMORBIDITIES IN PATIENTS WITH JUVENILE SYSTEMIC LUPUS ERYTHEMATOSUS AND ITS RELATION WITH THE PED-SDI ORGAN DAMAGE INDEX IN 2 PEDIATRIC RHEUMATOLOGY CLINICS IN BOGOTA, COLOMBIA.

#### Christine Arango^1^, Clara Malagon^2^, Catalina Mosquera^2^

##### ^1^Medicine program, Universidad Tecnologica de Pereira, Pereira; ^2^Pediatric rheumatology program, Universidad El Bosque, Bogotá, Colombia

###### **Correspondence:** Christine Arango

**Introduction:** Juvenile systemic lupus erythematosus (jSLE) is a multisystemic disease that has its onset in childhood. It is accompanied by an important morbidity related to the disease and its therapy. Ped-SDI index is used to measure the organ damage in these patients. Morbidities and comorbidities can impact this index in a positive or negative way.

**Objectives:** To evaluate the relationship between morbidities and comorbidities with the ped-SDI damage index in patients with juvenile systemic lupus erythematosus being followed up in 2 paediatric rheumatology clinics in Bogotá, Colombia.

**Methods:** A descriptive, cross-sectional study of a retrospective cohort of patients evaluated between 1997 and 2016. Morbidities, comorbidities and treatment were identified and the ped-SDI damage index was measured in the last visit. The statistical analysis was done using Stata V12.

**Results:** 112 participants with juvenile systemic lupus erythematosus were included, the female: male ratio was 5,2:1. Sixty-two percent were 12 years or older at the diagnosis. The mean time of follow up was 49,3 months (6-161). Seven patients (6,25%) died during follow up with variable causes of death. The most frequently involved organs were musculoskeletal, mucocutaneous and hematologic. Patients who have taken 3 o more different immunosuppressive drugs (excluding antimalarial and steroids) during their disease, had a statistically higher damage index at last visit. Medication non-adherence was found in 40% of patients, being this related with a higher damage index at last visit. The morbidities associated with a higher organ damage index were acute kidney injury (p=0,0277), chronic kidney disease (p=0,0275), dialysis requirement (p=0,0072), neuropsychiatric (p=0,0020), cardiovascular (p=0,0000), pulmonary (p=0,0000), gastrointestinal (p=0,0082) and ocular involvement (p=0,0001). The presence of polyautoimmunity was also associated with a higher index (p=0,003). In contrast, the presence autoimmune thrombocytopenia was associated with a lower damage index (p=0,0378).

**Conclusion:** These findings showed that morbidities and comorbidities can impact the prognosis of juvenile lupus patients in a positive or negative way. These findings help to understand some different factors that can contribute to organ damage in these pediatric patients. Searching for these conditions and starting an early and directed therapy would impact the prognosis in a positive way.

**Disclosure of Interest**

None Declared

### P127 HEPATITIS AS THE MAIN INITIAL MANIFESTATION OF JUVENILE SYSTEMIC LUPUS ERYTHEMATOSUS (J SLE)– 2 CASE REPORTS

#### Erato Atsali^1^, Ekaterini Siomou^2^, Sapfo A. Alfantaki^3^, Pelagia Katsibri^4^, Sorina Boiu^1^, Lampros Fotis^1^, Ioana Muresan^1^, Vana Papaevangelou^1^, Dimitrios T. Boumpas^4^

##### ^1^3rd Pediatric University Clinic, “ATTIKON” UNIVERSITY HOSPITAL, CHAIDARI; ^2^Faculty of Medicine,School of Health Sciences, UNIVERSITY OF IOANNINA; ^3^UNIVERSITY HOSPITAL OF IOANNINA, Ioannina; ^4^4TH Department of Medicine, “ATTIKON” UNIVERSITY HOSPITAL, CHAIDARI, Greece

###### **Correspondence:** ERATO ATSALI

**Introduction:** Although hepatic disease is not a significant cause of morbidity/mortality in jSLE,it is now considered to have greater clinical significance.

**Objectives:** To report two cases of SLE who presented with abnormal liver function tests several months before the onset of the rest clinical manifestations.

**Methods:** The first case is a 12,5 years old girl who presented with a one month history of malar rash and joint pain (ankles and knees). One and a half year before, elevated transaminases (2fold above the upper normal limit) were noticed during a routine exam. An extensive laboratory work up for viral hepatitis A,B and C,Wilson disease, celiac disease, hepatotropic viruses (EBV),a1 antithrypsin level and autoimmune hepatitis (ASMA,AMA.anti LC-1) had failed to reveal any kind of hepatic disease. Antinuclear antibodies were positive (1/320). Liver elastography was normal.

The second case is a 15,5 years old girl who came to our department with the diagnosis of autoimmune hepatitis (AIH).8 months earlier, laboratory exams done due to a persistent maculopapular rash of hands and feets had revealed a significant increase of transaminase levels (4fold above the upper normal limit). Based on positive serological markers (positive antinuclear, smooth muscles and anti liver cytosol type 1 antibodies) the diagnosis of AIH had been considered as most probable although liver biopsy was normal.

**Results:** On physical examination the first girl had malar rash and arthritis of both knees and ankles. Blood examinations showed leucopenia (3370/μL),low C3 (32,3mg/dl),ANA positive (1/640),anti DNA positive (254units) and mild proteinuria (120mg/24h).The diagnosis of jSLE was set and treatment with hydroxychloroquine and steroids was started. Transaminases level returned to normal quickly after the initiation of treatment.

The second girl had on physical examination reticular peliosis, arthritis of metatarsophalangeal joints and a papular rash of hands and feet. Supplemental laboratory exams revealed low C3 and positive anti Ro antibodies and Coombs test. JSLE was diagnosed while the diagnosis of autoimmune hepatitis was considered as less probable based on normal liver histology. She started hydroxychloquine and prednisone leading to a complete resolution of hepatitis. Nine months later, while receiving a low dose of steroids, she developed a typical malar rash and nephritis with significant proteinuria and hypoalbuminemia. A renal biopsy showed early membranous nephritis. Treatment with pulses of methylprednisolone followed by per os steroids and azathioprine leaded to a complete resolution of symptoms.

**Conclusion:** A wide spectrum of liver disorders is associated with SLE classified as liver parenchymal injury associated with SLE (“lupus hepatitis”),overlap syndromes with autoimmune liver disease and non-autoimmune hepatopathy (drug induced, viral hepatitis, fatty liver, thrombosis). Although abnormalities of liver function are not included in the diagnostic criteria, hepatitis is frequently noticed at disease onset. The diagnosis of SLE should be kept on mind in every child who presents with asymptomatic biochemical liver abnormalities (even if it doesnot fulfill the diagnostic criteria) and proper clinical and laboratory examination should be done towards this direction.

Informed consent to publish these case reports has been obtained from the patients and their parents.

**Disclosure of Interest**

None Declared

### P128 CAROTID INTIMA-MEDIA THICKNESS AS EARLY MARKER OF SUBCLINICAL ATHEROSCLEROSIS: A PRELIMINARY SINGLE-CENTER STUDY IN CHILDHOOD ONSET SYSTEMIC LUPUS ERYTHEMATOUS

#### Francesco Baldo, Federico Annoni, Micaela Carini, Antonio Mastrangelo, Valentina De Cosmi, Carlo Virginio Agostoni, Francesca Minoia, Giovanni Filocamo

##### IRCCS OSPEDALE POLICLINICO CÀ GRANDA, Milan, Italy

###### **Correspondence:** Francesco Baldo

**Introduction:** The association between systemic lupus erythematous (SLE) and early onset atherosclerosis has been widely accepted. In 2006, childhood-onset SLE (cSLE) was included by the American Heart Association and the American Academy of Pediatrics in the list of pediatric diseases with an increased risk of cardiovascular diseases (CVD). Carotid intima-media thickness (IMT) is frequently used as a precocious marker to detect early subclinical atherosclerosis and to identify higher risk patients.

**Objectives:** To assess early subclinical atherosclerosis in patients affected by cSLE, and to investigate its possible correlation with disease related parameters and traditional cardiovascular risk factors

**Methods:** Patients with diagnosis of cSLE by ACR classification criteria, followed at our center were included in the study. Demographic, clinical and laboratory features and therapeutic interventions were retrospectively collected from clinical charts. In all patients IMT of the distal 10mm of the far wall of common carotid artery was determined by ultrasonography with an highly automated method (qIMT). Concurrently with carotid IMT measurement, atherosclerosis-related biomarkers, such as plasma lipid levels and blood pressure were measured, and an accurate evaluation of traditional clinical CVD risk factors (smoke, exercise, familiar history of CVD, renal disease and hypertension) was performed. The SLEDAI was used to measure the disease activity. Correlation between carotid IMT and disease related parameters and traditional CVD risk factors was calculated by means of Spearman's test or Mann-Whitney U test as appropriate.

**Results:** We present the preliminary data on 15 cSLE patients (12 females, 3 males) enrolled so far. The median age at time of IMT evaluation was 15 years. The median SLEDAI was 7 at cSLE onset and 4 at time of IMT evaluation. Median lipid and apolipoprotein plasma levels, uric acid, and renal functional index were in range with age related references. Measured carotid IMT values observed in our 12 patients were in range with age and sex matched published values. In three patients an IMT >75° centile was detected: all of them had a higher SLEDAI at onset and a short disease course (< 3 years). IMT values according to ENA profile and treatment are shown in Table 1.

No traditional blood marker of dyslipidemia nor other disease related features nor therapeutic intervention were associated with IMT in our cohort.

**Conclusion:** Carotid IMT is a non-invasive marker for subclinical atherosclerosis that should be considered in the follow up of cSLE patients. Our data suggest that patients with short disease duration could be at higher risk of having higher IMT values. The correlation with short disease duration may be due to the burden of the induction steroidal therapy or to the effect of new onset uncontrolled disease. Our results are preliminary from a small cohort and needed a further confirmation in a larger prospective sample with a control group.

**Disclosure of Interest**

None Declared

**Table 1 (abstract P128).** Differences of IMT values according to ENA profile or treatment

	negative/untreated		positive/treated		p-value
	median (μ)	IQR	median (μ)	IQR	
Anti-SM	398	18	359	17	0,97
Anti-SSA/Ro	359	57	389	44	0,73
Anti-SSB/La	396	63	359	45,5	0,12
U1-RNP	398	18	342	20	0,0232
azathioprine	392,5	70,5	366	59	0,9
ciclophospamide	392,5	49,25	359	88,5	ns
idroxicloroquine	359	65	400,5	56,25	0,13

### P129 LAMOTRIGINE-INDUCED SYSTEMIC LUPUS ERYTHEMATOSUS

#### Ana Barbosa Rodrigues^1^, Filipa Oliveira Ramos^2,3^, Patrícia Costa-Reis^1,2^

##### ^1^Pediatrics Department, Centro Académico de Medicina de Lisboa, Hospital de Santa Maria; ^2^Pediatric Rheumatology Unit, Rheumatology Department, Centro Académico de Medicina de Lisboa, Hospital de Santa Maria; ^3^Unidade de Investigação em Reumatologia, Instituto de Medicina Molecular, Faculdade de Medicina, Universidade de Lisboa, Centro Académico de Medicina de Lisboa, Lisbon, Portugal

###### **Correspondence:** Ana Barbosa Rodrigues

**Introduction:** Drug induced-lupus erythematosus (DILE) is an autoimmune disorder characterized by typical lupus-like symptoms in the setting of drug exposure. Genetic factors, including differences in drug metabolism and immunologic features, affect the risk of developing the disease. Recently, it was found that DILE might also be associated with epigenetics, since some drugs known to induce a lupus-like phenotype inhibit DNA methylation.

Lamotrigine is used as an anticonvulsant and mood stabilizer drug. Between 5 to 10% of the patients develop a skin reaction to the drug, which, infrequently, can be life threatening, namely Stevens-Johnson syndrome, DRESS syndrome and toxic epidermal necrolysis. DILE associated with lamotrigine has been, very rarely, described.

**Objectives:** Describe a patient with lamotrigine-induced systemic lupus erythematosus in order to improve our knowledge of the drugs and phenotypes associated with DILE.

**Methods:** Clinical description of a patient with more than two years of follow up.

**Results: CASE DESCRIPTION**: 17-year-old female, with panic and generalized anxiety disorders, treated with quetiapine, propranolol and lamotrigine. The latter was started four days before the beginning of the symptoms. The patient presented to the emergency room with a three week history of daily fever, fatigue, odynofagia, arthralgias and myalgias. On physical exam it was identified a  non-pruritic diffuse erythematous maculopapular rash, vasculitic lesions on the fingers and lips and vulvar edema. There were no manifestations of serositis or neurological involvement. Alopecia, oral and nasal ulcers, organomegalies and arthritis were not detected. It was identified leukopenia (3920/μL), lymphopenia (880/μL) and a slightly elevated C-reactive protein (2.9mg/dL), with normal hemoglobine and platelets count; serologies were negative for cytomegalovirus, epstein barr, parvovirus, coxsakie, echovirus, herpes virus, *Mycoplasma pneumoniae*and Chlamydia. The patient was admitted to the hospital and treatment with lamotrigine was immediately stopped. There was a significant clinical improvement including apirexia and regression of the maculopapular rash in less than 24 hours after withdrawal of the drug. Positive antinuclear antibodies (1/80), positive anti-double stranded DNA (anti-dsDNA) antibodies (50.5 UI/mL), positive anti-Ro (SSA) antibodies (163.2 UQ) and low serum C3 (59 mg/dL) and C4 complement level (9 mg/dL) were found; antibodies anti-histones were negative (<1.1 U/mL).

The patient remained asymptomatic for two years under therapy with hydroxychloroquine (6.5mg/kg/day). C3 and C4 remained below normal ranges and anti-dsDNA antibodies persisted elevated. Recently, the patient developed photosensitivity and had a new vasculitic rash on the fingers, which improved with a short course of steroids.

**Conclusion:** We describe a rare case of lamotrigine DILE, who presented with a diffuse erythematous rash, leukopenia, lymphopenia, low serum C3 and C4 and antinuclear and anti-dsDNA antibodies, thereby fulfilling the SLICC criteria. The withdrawal of the drug was essential for clinical improvement. In conclusion, DILE should always be included in the differential diagnosis of a patient with systemic lupus erythematosus and the medical community should be aware of the drugs that might cause it. Drug withdrawal is a fundamental step for a favourable outcome.

For this case submission written informed consent was obtained from the patient and family.

**Disclosure of Interest**

None Declared

### P130 JUVENILE SYSTEMIC LUPUS ERYTHEMATOSUS AND THROMBOTIC THROMBOCYTOPENIA PURPURA, CASE REPORT SIMULTANEOUS DIAGNSOSIS IN A 5 YEARS OLD GIRL

#### Edith A. Benitez Vazquez^1^, Sofia B. Osorio Sagrero^2^, Luis A A. Aparicio Vera^1^, Maria T. Braña Ruiz^2^, Merari Elizabeth G. Cortez^2^, Velka A. Cortez Lopez^1^, Enrique Faugier^1^, Maria D. R. Maldonado Velazquez^1^

##### ^1^Reumatología; ^2^Hospital Infantil de México Federico Gómez, Ciudad de México, Mexico

###### **Correspondence:** Edith A. Benitez Vazquez

**Introduction:** The association of thrombotic thrombocytopenic purpura (TTP) and systemic lupus erythematosus (SLE) is a rare entity and usually occurs in advanced stages of SLE, here we present a case of a female patient 5 years old with simultaneous diagnosis of systemic lupus erythematosus and thrombotic thrombocytopenic purpura.

**Objectives:** Present the clinical case with diagnosis and treatment of systemic lupus erythematosus and thrombotic thrombocytopenic purpura in a pediatric patient.

**Methods:** Description of diagnosis and management of a case of newly diagnosed SLE and PPT in pediátric patient.

**Results:** Women's 5 years previously healthy age with symptoms of fever and arthritis 5 months of evolution, admitted to the institute by the presence of generalized edema and decrese output urine, elevated serum creatinine, proteinuria, metabolic acidosis and hypertension, presenting a rapidly progressive glomerulonephritis requiring renal replacement therapy with hemodialysis, renal biopsy was performed finding diffuse proliferative glomerulonephritis endocapillary, class IV lupus nephritis and thrombotic microangiopathy and hypertensive; The antibody profile denote ANA, antiDNA, anti B2-IgM glycoprotein and anticoagulante lupic positiv. For a history of seizures (7 days prior to admission) craneal MRI showed consistent data are with periventricular ischemia, ultrasound doppler renal absence of arterial and venous flow in both kidneys was performed and required anticoagulation heparin infusion for 21 days.

She was presented persistent thrombocytopenia, elevated DHL, 1% schistocytes peripheral smear and Ac IgG Anti ADAMS 13 positive (activity normal limit) in addition to histological finding of renal biopsy thrombotic microangiopathy integrated diagnostic thrombotic thrombocytopenic purpura, was administered treatment with methylprednisolone, gamma globulin, monthly cyclophosphamide, and plasmapheresis every 48 hours (first sessions entirely with albumin later albumin and plasma 1: 1), requiring 17 sessions, after which presented remission, with platelet count in 150,000, improvement of renal function and neurological status.

**Conclusion:** The incidence of SLE and PTT is unclear, lower incidence of 0.5% reported being more common in adult patients with prolonged evolution of SLE, it is difficult to establish the diagnosis because the two entities have laboratory findings in common, being key finding schistocytes in peripheral blood smears, and antibodies ADAMS 13.

There are no clear for the number of sessions of plasmapheresis patients requiring these guidelines, however The British Committee for Standards in Hematology (BCHS) guidelines recommend continuing the sessions at least two days after having a platelet count above 150.000, normal neurological status, hemoglobin levels and lactic dehydrogenase normal.

The diagnosis of thrombotic thrombocytopenic purpura in patients with SLE is underdiagnosed, it´s essential to consider no matter the time of diagnosis of SLE if there is presence of persistent thrombocytopenia, neurological and kidney disease or histological findings compatible with thrombotic microangiopathy. Informed consent for publication has been obtained from the parent.

**Disclosure of Interest**

None Declared

### P131 INTERRELATION OF BLOOD LIPID PROFILE AND IMMUNO-INFLAMMATORY ACTIVITY IN CHILDREN WITH COMORBID CONDITIONS IN SYSTEMIC LUPUS ERYTHEMATOSUS

#### Ljudmila F. Bogmat^1,2^, Irina N. Bessonova^1,2^, Nataly S. Shevchenko^1,3^

##### ^1^department of cardiorheumatology, Institute for the Health Care of Children and Adolescents of the National Academy of Medical Sciences of Ukraine; ^2^Department of pediatrics; ^3^Department of pediatrics № 2, V.N. KARAZIN NATIONAL UNIVERSITY, Kharkiv, Ukraine

###### **Correspondence:** Ljudmila F. Bogmat

**Introduction:** In the mechanisms of atherosclerotic arterial damage and the formation of comorbid conditions in rheumatic diseases, including systemic lupus erythematosus (SLE), many factors are involved, both traditional and those that are induced by inflammation and damage to the vessels by thrombosis and various kinds of antibodies.

**Objectives:** The aim was to study the relationship between the activity indices of the process (C-reactive protein (CRP), complement titer, circulating immune complexes (CIC)) with lipid profile (total cholesterol, triglycerides (TG), high-density lipoprotein cholesterol), the clotting system (prothrombin index, fibrinogen) and the functional state of the kidneys and liver (serum creatinine, glomerular filtration rate).

**Methods:** 35 children with SLE at the age of 7-18 years were examined, of which 4 (11.4%) were boys and 31 (88.6%) were girls. The majority (in 74.29%) patients was diagnosed with comorbid pathology. In the structure of comorbid conditions, violations of the functional state of the liver (in 40%) and kidneys (in 80%), pulmonary lesions (in 20%) and hip joint prevailed (in 20.0%), as well as cardiovascular disorders (in 11 %). Changes in the lipid spectrum of atherogenic orientation and signs of osteoporosis were recorded in half of patients (in 51.43% and in 50.0%, respectively), and changes in the coagulogram with signs of hypercoagulability occurred in 35.7% of the examined patients.

**Results:** In patients with SLE and comorbid conditions, an inverse correlation was established between the values of CRP and the level of complement (r = -0.477, p <0.05), the complement level was inversely correlated with the level of triglycerides (r = -0.744, p <0.05), and triglyceride levels correlated closely with the level of circulating immune complexes (r = 0.896, p <0.01), which confirms the interdependence of atherogenesis and immunocomplex inflammation.

The level of triglycerides of blood also had an inverse correlation with the parameters of the glomerular filtration rate (r = -0.892, p <0.05) and the straight line with serum creatinine (r = 0.834, p <0.05, and transaminases, in particular with ALT (r = 0.848, p <0.05).

With the help of multiple regression analysis it was found that an increase in the activity of the disease with an increase in the level of circulating immune complexes in children suffering from SLE and comorbid conditions is accompanied by an increase in the level of total cholesterol and triglycerides, a decrease in the complement titer, which is represented by the equation:

$$ {\displaystyle \begin{array}{l}\mathrm{CEC}=1.44278+0.188976\cdotp \mathrm{OKS}\hbox{-} 1.51503\cdotp \mathrm{complement}+0.35388\cdotp \mathrm{TG}\\ {}\left(\mathrm{R}=94.25\%,{\mathrm{R}}^2=90.02\%,\mathrm{p}<0.01\right).\end{array}} $$

**Conclusion:** Thus, changes in the lipid spectrum of atherogenic orientation in patients with SLE of childhood not only take place against the background of the disease, but also contribute to the development of comorbid pathology

**Disclosure of Interest**

None Declared

### P132 DAMAGE INDEX IN CHILDREN WITH SYSTEMIC LUPUS ERYTHEMATOSUS

#### Ljudmila F. Bogmat^1,2^, Irina N. Bessonova^1,2^, Nataly S. Shevchenko^1,3^

##### ^1^department of cardiorheumatology, Institute for the Health Care of Children and Adolescents of the National Academy of Medical Sciences of Ukraine; ^2^Department of pediatrics; ^3^Department of pediatrics № 2, V.N. KARAZIN NATIONAL UNIVERSITY, Kharkiv, Ukraine

###### **Correspondence:** Ljudmila F. Bogmat

**Introduction:** One of the important aspects in estimating the prognosis of patients with systemic lupus erythematosus and creating optimal differentiated therapies for them is to identify potentially irreversible lesions of various organs and systems.

**Objectives:** The purpose of the study was to determine the damage index in children with systemic lupus erythematosus.

**Methods:** A total of 39 children with systemic lupus erythematosus (SCL) aged 7-18 years were examined. The average duration of the disease at the time of the survey was 45.8 ± 2.9 months. The diagnosis of SLE was based on the SLICC classification criteria (2012), an adult rating scale (SLICC / ASR Damage Index) was used to assess the damage index.

**Results:** In 46.2% of patients (18 people), PI was zero. The duration of the disease at the time of examination of patients in this subgroup was 45 ± 3.3 months. The remaining 21 patients (53.8%) had an IE of 1 to 4 points, i.e. there were signs of irreversible damage to certain organs and systems, namely, the nervous, cardiovascular systems, the lungs, the kidneys, the bone and muscle system, and the eyes. The duration of the disease in these patients varied from 3 to 113 months, on average 47 ± 6.9 months.

In 15 cases the IP was equal to 1 point. These patients had an irreversible damage to the nervous (9 persons) and cardiovascular systems (1 person), as well as the kidneys (1 person) and lungs (4 people). In 5 patients, PI was 2 points due to irreversible damage to the organ of vision (4 persons) and musculoskeletal system (1 child). In one patient, the injury index was 4 points due to persistent damage to the organ of vision as a cataract of both eyes and the nervous system in the form of convulsive seizures requiring prolonged use of anticonvulsants.

The most commonly damaged CKD was the damage to the nervous system, which was observed in 25.6% of patients and in all cases was represented by cognitive impairment, and only one patient had convulsive seizures. In the second place, the frequency of occurrence was damage to the organ of vision (in 12.8% of patients). This kind of damage was due to the presence of cataracts of both eyes. Somewhat less frequently, lung damage was detected (10.3% of the subjects) in the form of pleural fibrosis according to the X-ray examination. Irreversible damage to the kidneys, cardiovascular and musculoskeletal systems was noted at the same frequency (2.6%). Damage to the kidneys was represented by the development of massive proteinuria, cardiovascular system - cardiomyopathy, and damage to the musculoskeletal system is due to avascular necrosis of the femoral head.

**Conclusion:** Thus, the obtained results testify to the expediency of the use of PI in systemic lupus erythematosus in childhood in order to clarify the nature and degree of damage to organs and systems, which will allow timely correction of treatment.

**Disclosure of Interest**

None Declared

### P133 ANTIPHOSPHOLIPID SYNDROME IN MEXICAN CHILDREN: EVOLUTION, LABORATORY AND CLINICAL CHARACTERISTICS: A 10-YEAR EXPERIENCE

#### Maria T. Brana^1^, Luis Aparicio^2^, Sofia Osorio^2^, Velka Cortez^2^, Adriana Benitez^2^, Merari Gomez^2^, Andres Rodriguez^2^, Enrique Faugier^2^, Rocio Maldonado^2^

##### ^1^Pediatric Rheumatology, Hospital Infantil de Mexico Federico Gomez; ^2^Hospital Infantil de Mexico, Federico Gomez, Hospital Infantil de México, Mexico, Mexico

###### **Correspondence:** Maria T. Brana

**Introduction:** The antiphospholipid syndrome (APS) is a multisystem and autoimmune disease, which is mainly characterized by the presence of thrombotic events, gestational morbidity, in the presence of high titers of antiphospholipid antibodies. It can present as a primary entity, or secondary to another autoimmune disease. The understanding of this pathology is still evolving and even more in its presentation in the pediatric patients. The presence of antiphospholipid antibodies has been widely reported in pediatric patients with thrombosis. APS is considered the most common acquired cause of a prothrombotic state. At the moment, there are no reliable data of its presentarion in pediatrics. The long-term morbidity and mortality associated with thrombosis events in pediatric population may be minored by determining prognosis and select patients for prophylactic treatment and more rigorous follow-up.

**Objectives:** To describe the clinical presentation and evolution, in addition to laboratory findings, in Mexican pediatric population with diagnosis of APS. Identified patients with arterial or venous thrombosis and its relation with laboratory findings. Describe epidemiology of Mexican pediatric population with APS.

**Methods:** Retrospective cohort study of the Children’s Hospital of Mexico Federico Gomez, last 10 years. We reviewed the data form the clinical archives of the patients with diagnosis of APS according to the Miyakis criteria, from 2007 to 2017. The variables analyzed include age at diagnosis, sex, subtype of APS, clinical finding of thrombosis, laboratory finding of antiphospholipid antibodies and outcome.

**Results:** A total of 29 patients fulfil the diagnosis criteria of APS. The mean age of patients at diagnostic was 9.8 years, with a minor age at diagnosis of 2.2 years and a maximum of 16.4 years of age. Of the total population 52% were females and 48% males. Primary APS was diagnosed in 48%, of which 71% were males. Secondary APS was present in 52% of the population, all diagnosed with Systemic Lupus Erythematosus. Of the patients with secondary APS, 85% were female. Arterial thrombosis was present in 48% of the cases, primarily in the group of secondary APS. Of the total cases 20% presents with a second thrombosis event despite anticoagulant therapy, of this 66% were diagnosed with secondary APS. Lupus anticoagulant (LA) was present in high titers in 93% of the patients, while anti-B2 glycoprotein-1 antibody IgG was present in 27% and IgM in 31%. Anticardiolipin antibody IgG was positive in 17% of the population, while isotype IgM in 24%. All patients receive long term anticoagulant therapy. One male patients, with secondary APS developed catastrophic APS. One patient required of amputation for deep venous thrombosis, and another patient died as a complication of gastrointestinal thrombosis, both males with primary APS.

**Conclusion:** During a 10 year follow up, we diagnosed 29 patients with APS. As reported in literature, a greater percentage of patients are female and present with a secondary APS. Most patients with primary APS are males. In our population, in contrast with what literature report, secondary APS presents with a greater percentage of arterial thrombosis. The mayor site of venous thrombosis was low extremities and of arterial thrombosis was cerebrovascular events. The presence of positive LA implicates a higher risk of thrombosis. Our pediatric population diagnosed with APS requires of a close follow up in order to monitor anticoagulant therapy and to prevent the patients from developing a second thrombotic event that may lead to death.

**Disclosure of Interest**

None Declared

### P134 SEVERE PULMONARY ARTERIAL HYPERTENSION AS THE INITIAL MANIFESTATION OF SYSTEMIC LUPUS ERYTHEMATOSUS IN A 7-YEAR-OLD MALE PATIENT: A CASE REPORT.

#### Maria T. Brana^1^, Luis A. Aparicio^2^, Sofía Osorio^3^, Enrique Faugier^4^, Andres Rodriguez^4^, Rocio Maldonado^4^

##### ^1^Pediatric Rheumatology; ^2^Hospital Infantil de Mexico, Federico Gomez, Hospital Infantil de Mexico Federico Gomez; ^3^Hospital Infantil de Mexico, Federico Gomez, Hospital Infantil de Mexico, Federico Gomez; ^4^Hospital Infantil de Mexico, Federico Gomez, Hospital Infantil de México, Mexico, Mexico

###### **Correspondence:** Maria T. Brana

**Introduction:** Pleural pulmonary manifestations in patients with systemic lupus erythematosus are reported in approximately 5% of cases. Presenting as pleural effusion, alveolar hemorrhage, diffuse interstitial lung disease, pulmonary infections and pulmonary arterial hypertension, among others, they are a manifestation difficult to diagnosis. Pulmonary arterial hypertension is defined as a pressure greater than or equal to 25mmHg. The world health organization has classified pulmonary arterial hypertension into five categories, the first group being associated with connective tissue disease such as systemic lupus erythematosus. Pulmonary arterial hypertension is a rare condition, usually occurring 3 to 5 years after the diagnosis of systemic lupus erythematosus and conditioning a high risk of morbidity and mortality. Initially, the majority of patients with SLE and PAH are asymptomatic and as the disease progresses, they present with progressive dyspnea, fatigue, intolerance, exercise, chest pain and non-productive cough.

**Objectives:** We report the case of a 7-year-old male patient who presented with pulmonary arterial hypertension as the initial manifestation of systemic lupus erythematosus.

**Methods:** Case report.

**Results:** Clinical case

A 7-year-old male patient who was admitted to the Pneumology Department at Children's Hospital of Mexico Federico Gómez due to respiratory distress. In emergency assessment, pulmonary arterial hypertension of 66mmHg was identified, of unknown cause. The patient did not have significant medical background, having enjoyed of good health up to 6 months prior to his admission. He presented with a history of non-quantified fever, as well as episodes of fatigue and dyspnea. Two months before admission, chest pain was added, exacerbated with inspiration. On admission, transthoracic echocardiography revealed severe dilatation of right cavities, moderate tricuspid insufficiency, with left ventricular ejection fraction of 56% and arterial pulmonary pressure of 66mmHg. The diagnostic approach is initiated. Due to a history of pulmonary tuberculosis in the patient grandmother, the patient was studied with BAAR and cervical lymph node biopsy, ruling out the diagnosis. Infectious process causing the manifestations was also ruled out. The patient was discharged with medical treatment, requiring readmission in for 7 days, with facial edema and in lower extremities, generalized pallor, asthenia, adynamia and 4 days before a decrease in urinary volumes and frequency. On admission, right heart failure, secondary to increase of pulmonary hypertension for discontinuation of diuretic administration. A renal biopsy was performed, which was reported as class IV lupus nephropathy, with an index of activity and chronicity of 0. The diagnosis of systemic lupus erythematosus is integrated based on the ACR criteria. Induction of remission of lupus nephropathy based on the CARRA protocol. As treatment was administered the patient showed important clinical improvement.

**Conclusion:** Pulmonary arterial hypertension is a rare condition, usually occurring 3 to 5 years after the diagnosis of systemic lupus erythematosus. In pediatric population, it is reported as a lupus complication in 5 to 14% of patients, and less than 1% as an initial manifestation. It is a clinical complication that gives the patient a high risk of morbidity and mortality. It is important to acknowledge that pulmonary arterial hypertension can be the initial manifestation of lupus in pediatric population. A prompt identification assures a prompt treatment and a better prognosis. Informed consent to publish had been obtained from the parents.

**Disclosure of Interest**

None Declared

### P135 PREDICTING ORGAN DAMAGE IN PATIENTS WITH CHILDHOOD-ONSET SYSTEMIC LUPUS ERYTHEMATOSUS: A RETROSPECTIVE STUDY OVER THE LAST 25 YEARS

#### Nastasia Cekada^1^, Mario Sestan^1^, Emilija Hosticka^1^, Maja Novoselec^1^, Mateja Batnozic Varga^2^, Ivan Padjen^3^, Marijan Frkovic^1^, Domagoj Kifer^4^, Branimir Anic^3^, Drago Batinic^5^, Kristina Potocki^6^, Ivan Malcic^1^, Marija Jelusic^1^

##### ^1^Department of Paediatrics, University Hospital Centre Zagreb, University of Zagreb School of Medicine, Zagreb; ^2^Department of Paediatrics, University Hospital Centre Osijek, Osijek; ^3^Department of Internal Medicine, University Hospital Centre Zagreb, University of Zagreb School of Medicine; ^4^Department of Biophysics, Faculty of Pharmacy and Biochemistry, University of Zagreb; ^5^Clinical Department of Laboratory Diagnosis; ^6^Diagnostic and Interventional Radiology Department, University Hospital Centre Zagreb, University of Zagreb School of Medicine, Zagreb, Croatia

###### **Correspondence:** Nastasia Cekada

**Introduction:** Childhood-onset systemic lupus erythematosus (cSLE) is a multisystemic, chronic autoimmune disease occurring in children and adolescents under 18, presenting heterogeneously, with the course of the disease often more severe than in adults, and the activity of the disease widely being evaluated by Systemic Lupus Erythematosus Disease Activity Index (SLEDAI 2K) and damage by the SLICC/ACR damage index (SDI).

**Objectives:** To compare and explore the correlation between the SLEDAI-2K disease activity index at the time of diagnosis and the SLICC/ACR damage index (SDI) of patients at their last follow up, to examine the quantity of organ damage in regard to patients’ characteristics and disease duration and to predict the risk of organ damage occurrence in time.

**Methods:** The retrospective study included children treated for cSLE in the period from January 1991 to September 2017 at Department of Pediatrics, University Hospital Center Zagreb. The follow up data of patients after the age of majority at 18 and who were under care of the Department of Internal Medicine, University Hospital Center Zagreb was also included. All children were diagnosed according to the ACR 1997 and SLICC 2012 criteria. Data analysis was done using R environment for statistical computing.

**Results:** The disease development of 93 children (74 females and 19 males) with cSLE was examined in this study. The median (range) follow up time was 7 (0.5-24) years and the median (range) age at diagnosis was 13 (5-19) years. Two patients have died during the examined period. Mean (SD) SLEDAI-2K was 18.3 (9.0) at the disease onset. 35 children (38 %) had organ damage at the last follow up with the median (range) SDI 0 (0-7). The first organ systems damaged in affected patients were renal (28%), musculoskeletal (22%), ocular (19%), neuropsychiatric (17%), cardiovascular (11%) and peripheral vascular (2.8%), but with no significant statistical difference regarding the type of organ damage. Kendall rank correlation coefficient was used to evaluate the relationship between SLEDAI-2K at the disease onset and SDI, determining a positive correlation which was statistically significant (τb = 0.252, p = 0.003). However, no significant correlation was determined between the duration of the disease and SDI (τb = 0.042, p = 0.628) or follow up period and SDI (τb = 0.111, p = 0.191) nor was there significant difference of SDI in regard to gender (Asymptotic Wilcoxon-Mann-Whitney Test, p= 0.574). Using Kaplan-Meier method we estimated the decrease in ratio of patients without organ damage since diagnosis or the occurrence of the first symptoms. Since the estimated ratio of patients with organ damage at the endpoint was less than 50%, we could not estimate the time required for damage development in 50% of patients. However, we are 95% sure that the damage is not happening in the first 9 or 10 years after diagnosis or the occurrence of the first symptoms, respectively.

**Conclusion:** The high correlation detected between SLEDAI-2K and SDI indicated that the presentation of the cSLE at onset can be prognostic of the course and long-term prognosis of lupus. Our findings suggest that it is unlikely that organ damage will occur in 50% of patients in the first nine years of the disease course.

**Disclosure of Interest**

None Declared

### P136 RUXOLITINIB TREATMENT IN A PATIENT WITH ANTI-DNASE 2 DEFICIENCY: BENEFITS AND BURDENS

#### Luisa Cortellazzo Wiel^1^, Anna M. C. Galimberti^1^, Giulia Gortani^2^, Serena Pastore^2^, Alessandra Tesser^3^, Andrea Taddio^1,2^, Alberto Tommasini^3^

##### ^1^Paediatrics, University of Trieste; ^2^Paediatrics; ^3^Clinical Immunology, IRCCS Burlo Garofolo, Trieste, Trieste, Italy

###### **Correspondence:** Luisa Cortellazzo Wiel

**Introduction:** A seventeen-year-old boy was admitted to our Hospital after an episode of lipothimia, followed by dyspnea and tachycardia with prolabial cyanosis.

The patient was in a follow-up program for an anti-DNAse2 deficiency syndrome (Rodero et al., Nat Commun, 2017): a newly described, auto-inflammatory genetic disorder with significant overlap with systemic lupus erythematous.

His past medical history was characterized by the presence of neonatal hepatopathy, cytopenia, recurrent fever, polyarticular arthritis, chronic glomerulonephritis, lipodystrophy, lupus pernio and growth retardation. The diagnosis of anti-DNAse2 deficiency was made at the age of 14 by whole exome sequencing and was suggested by the high value of the interferon signature that highlighted the pivotal pathogenetic role of type 1 interferon.

This clinical picture progressively worsened despite several therapeutic trials with glucocorticoids, immunosuppressant and biological agents. Therefore, a trial treatment with two antimalarials (hydrossichloroquine and mepacrine) and ruxolitinib was started, together with abatacept: this treatment had led in the previous months to an overall improvement of his symptoms and a reduction of the glucocorticoids dosage.

**Objectives:** At admission, clinical and instrumental evaluation showed a severe pulmonary hypertension (PAH) with a mean pulmonary pressure of 77 mmHg and a severe dysfunction of his right cardiac chambers. A drug adverse effect was considered and all the treatments were discontinued. Considering the possibility that PAH was an interferonopathy manifestation, similarly to what can occur in SLE, steroid boluses were administered. Moreover, a vasodilatant therapy with iloprost, ambrisentan, sildenafil and diuretics was started. During the hospital course he developed a severe pancytopenia (Hb 10 g/dl, WBC 1020/mmc, PLT 20.000/mmc). Hemophagocytic Limphohistiocytosis (HLH) and malignancy were ruled after performing a bone marrow aspiration, showing an hypocellular bone marrow. A viral infection was also excluded.

**Methods:** Given the poor response to corticosteroids, ruxolitinib was then reintroduced at higher doses than previously administered (10 mg twice daily), based on the previous benefits and on the anecdotal evidence of efficacy in PAH.

**Results:** A dramatic improvement of his pulmonary pressures was noted, more successful than expected with only vasodilatant therapy, with an estimated pulmonary pressure at discharge of 35 mmHg.

His cardiologic follow-up showed a slow but progressive improvement of PAH so far; the symptoms related to his basal condition also improved. Ruxolitinib was maintained at the dose of 7.5mg BID. No other adverse event was noticed a part from a moderate title elevation of BK virus in the urine and a transient herpes zoster infection that rapidly healed with acyclovir therapy.

**Conclusion:** We therefore concluded that specific therapies for genetically determined conditions are double-edged with the need of carefully balancing efficacy with potential adverse events. Great caution should be taken in particular with JAK inhibitors as an abrupt stop of treatment may associate with severe inflammatory rebound. Informed consent to publish had been obtained.

**Trial registration identifying number:** This work was funded by IRCCS Burlo Garofolo grant RC24/17 anf Telethon Foundation grant GGP15241.

**Disclosure of Interest**

None Declared

### P137 EVALUATION OF CHOROIDAL THICKNESS AND PERIPAPILLARY RETINAL NERVE FIBER LAYER IN PATIENTS WITH JUVENILE SYSTEMIC LUPUS ERYTHEMATOSUS

#### Selcan Demir^1^, Abdullah Ağın^2^, Ata Baytaroğlu^2^, Hafize E. Sönmez^1^, Erdal Sağ^1^, Yelda Bilginer^1^, Sibel Kadayıfçılar^2^, Bora Eldem^2^, Seza Özen^1^

##### ^1^Department of Peditarics, Division of Rheumatology; ^2^Opthalmology Departmant, Hacettepe University Faculty of Medicine, Ankara, Turkey

###### **Correspondence:** Selcan Demir

**Introduction:** Systemic lupus erythematosus (SLE) is an autoimmune disease that involves multiple organs, including ocular involvement.

**Objectives:** This study aims to evaluate choroidal thickness and peripapillary retinal nerve fiber layer in patients with juvenile SLE (jSLE).

**Methods:** We have included all jSLE patients (n=21) diagnosed according to Systemic Lupus International Collaborating Clinics classification criteria between January 2017 and April 2017 and age sex-matched control group (n=21). The SLE disease activity index (SLEDAI) was used to assess disease activity. After routine eye examinations, choroidal thickness (ChT) in five points (750 and 1500 microns from the center of the fovea both in the temporal, nasal quadrant and under the fovea), presence of subretinal and/or intraretinal fluid, retinal nerve fiber layer thickness of optic disc (RNFL) were evaluated.

**Results:** In ophthalmologic evaluation, only one patient was active [SLEDAI 2 (1-4)]. One patient had episcleritis, one patient had corticosteroid-induced cataract and glaucoma. Median age was 3,5 years (1-8,5 years), despite the use of hydroxychloroquine retinal pigmentation or visual field defects were absent in patients. There was a significant increase in ChT at 5 points, but RNFL thicknesses were similar compared to healthy controls in jSLE patients. When the relationship between OCT findings and other variables were evaluated, a significant negative correlation was found between the initial creatinine values and the ChT. Intraretinal or subretinal fluid were not present in any of the patients.

**Conclusion:** The results of our study suggest that increased ChT is related to mononuclear cell infiltration, immune complex accumulation, autoantibodies against retinal pigmentation or some vascular mechanism in jSLE patients.

**Disclosure of Interest**

None Declared

### P138 Withdrawn

### P139 SOCIAL AND PROFESSIONAL OUTCOME OF CHILDHOOD ONSET SYSTEMIC LUPUS ERYTHEMATOSUS: A COHORT OF 65 PATIENTS ISSUED FROM PEDOLUP

#### Anita Duncan^1^, Camille Braun^2^, Marine Desjonqueres^1^, Bruno Ranchin^1^, Michael Hofer^3^, Isabelle Durieu^4^, Jean-Christophe Lega^4^, Eric Hachulla^5^, Alexandre Belot^1^

##### ^1^service de rhumatologie et néphrologie pédiatrique, Hôpital Femme Mère Enfant; ^2^Université Claude Bernard, Lyon 1, Lyon, France; ^3^service d’immuno-allergologie et rhumatologie, Centre Hospitalier Universitaire Vaudois, Lausanne, Switzerland; ^4^service de médecine interne, Centre Hospitalier Lyon Sud, Lyon; ^5^service de médecine interne, Hôpital Claude Huriez, Lille, France

###### **Correspondence:** Anita Duncan

**Introduction:** Systemic Lupus Erythematosus (SLE) is a rare, chronic, systemic disease with juvenile onset occurring in 10 to 20% of cases. Its variety of clinical presentations with an unpredictable relapsing-remitting course can make it a challenging illness to diagnose and deal with throughout childhood.

**Objectives:** The aim of this study was to evaluate educational, professional and family outcome along with quality of life appreciation in a cohort of pediatric SLE (SLEp) patients, fields for which data is still scarce.

**Methods:** This multi-center study included all 65 SLEp patients from the PedOLup unit (JIR cohort) of two french centers, Lyon (Hôpital Femme Mère Enfant, Centre Hospitalier Lyon Sud) and Lille (Hôpital Claude Huriez). Data regarding clinical presentation, education, employment, personal family outcome and quality of life were obtained from the PedOLup database and completed with medical records and oral interview.

**Results:** 75,4% of our patients had visceral involvement with a mean disease duration of 13,6 years. Disease activity and damage scores (SLEDAI and SDI) were low at the time of the study (median of 0). The proportion of graduates in those over 25 years of age was similar to national references and 87,3% of patients were presently either employed of following a study course. Half of those over 20 lived as a couple and 34% of them had children with a mean number of person per household comparable to French references. There seemed to be little difference when comparing quality of life in our patients to a healthy French reference population.

**Conclusion:** Despite the severity of juvenile forms of SLE on diagnosis, long term social and professional outcome of patients followed in this cohort seems satisfying. However, this study points out elements of precarity in the field of employment opening the way for thinking about work support and accommodation.

**Disclosure of Interest**

None Declared

### P140 SICKLE CELL DISEASE AND LUPUS NEPHRITIS WITH NEGATIVE SEROLOGIC MARKERS

#### André Garrido^1^, Marta Ezequiel^2^, Andreia Martins^1^, Marta Moniz^2^, Rita Manso^3^, Vanda Anacleto^4^, Teresa Ferreira^5^, Marta Cabral^6^, Helena C. Loureiro^1^, Helena I. Almeida^2^

##### ^1^Paediatric Department; ^2^Paediatric Intensive Care Unit; ^3^Pathology Department; ^4^Paediatric Nephrology; ^5^Paediatric Haematology; ^6^Paediatric Rheumatology, Hospital Prof. Doutor Fernando Fonseca, E.P.E., Amadora, Portugal

###### **Correspondence:** André Garrido

**Introduction:** The presence of immunologic markers (autoantibodies, hypocomplementemia or a positive direct Coombs test) are key features in the Systemic Lupus International Collaborating Clinics (SLICC) criteria for the classification of Juvenile Systemic Lupus Erythematosus (jSLE).

**Objectives:** The authors present a case of a female adolescent with sickle cell disease and lupus nephritis with a severe presentation and without any serologic markers to corroborate the diagnosis.

**Methods:** A 14-year-old female with Sickle Cell Disease (SCD), born and living in Angola until 3 days before admission, was hospitalized in the past 3 months due to a nephrotic syndrome, complicated with pneumonia with empyema, arterial hypertension, seizures and exacerbations of the SCD requiring frequent red blood cell transfusions and long term corticosteroids.

**Results:** The patient arrived to Portugal for further investigation and was admitted in the Paediatric Intensive Care Unit with respiratory deterioration due to a pyopneumothorax, requiring thoracentesis and intravenous antibiotics. At D13 of hospitalization she presented seizures and neurological deterioration. The magnetic resonance imaging performed was suggestive of a posterior reversible encephalopathy syndrome. The renal biopsy revealed a proliferative glomerulonephritis with mesangial and capillary deposition of several types of immune complexes, mainly IgG, also known as “full-house” immunofluorescence pattern, highly suggestive of a class III lupus nephritis. The laboratory results were inconsistent with the diagnosis of jSLE – negative autoantibodies, mild elevation of complement and a negative direct Coombs test. Treatment with mycophenolate mofetil and prednisolone was started in order to induce remission of lupus nephritis, having attained a clinical remission (without proteinuria, normal renal function and normal blood pressure) in one week.

**Conclusion:** In jSLE, renal involvement compromises the prognosis with significant morbidity and mortality. Few cases of lupus nephritis without serologic markers have been described to date. The literature reports two main presentations of seronegative lupus nephritis: one with a lifelong seronegativity, either with renal-limited or extra-renal manifestations; other with a serologic conversion, latter in the course of the disease. The authors hypothesized if the diagnosis of sickle cell disease and previous corticotherapy are related with this atypical presentation. Regardless of the clinical picture, a prompt and accurate treatment should not be delayed. This is an example of how an association of two complex diseases can present an hard treatment challenge. Informed consent to publish had been obtained from the parent.

**Disclosure of Interest**

None Declared

### P141 PAEDIATRIC SYSTEMIC LUPUS ERYTHEMATOSUS PATIENTS IN SOUTH AFRICA HAVE HIGH PREVALENCE AND SEVERITY OF CARDIOVASCULAR MANIFESTATIONS

#### Michael Harrison^1^, Laura Lewandowski^2,3^, Liesl Zuhlke^4,5^, Christiaan Scott^1,3^

##### ^1^University of Cape Town, Cape Town, South Africa; ^2^National Institute of Arthritis, Musculoskeletal, and Skin Diseases, NIH, Boston, USA; ^3^Paediatric Rheumatology; ^4^Division of Paediatric Cardiology, Red Cross War Memorial Children’s Hospital; ^5^Division of Cardiology, Department of Medicine, Groote Schuur Hospital, Cape Town, South Africa

###### **Correspondence:** Michael Harrison

**Introduction:** Paediatric onset of systemic lupus erythematosus (SLE) is associated with a high rate of major organ involvement, and patients of African ancestry tend to develop more aggressive SLE than those of Caucasian descent. A particularly severe disease phenotype has been described in this multi-ethnic South African cohort of children with SLE. Although cardiovascular involvement appears to be common in paediatric SLE, there are few published reports on the subject and none have been conducted in an African paediatric SLE population.

**Objectives:** This study aims to describe the frequency and characteristics of cardiovascular manifestations of paediatric SLE in a multi-ethnic South African cohort.

**Methods:** Demographic, clinical, and echocardiographic data were collected from paediatric SLE patients at 2 centres in Cape Town, South Africa. At the time of investigation, this cohort consisted of 93 participants diagnosed with SLE according to the Systemic Lupus International Collaborating Clinics (SLICC) or 1997 American College of Rheumatology (ACR) SLE criteria, prior to the age of 19 years. Individuals with cardiovascular involvement were identified by retrospective chart review. Cardiovascular involvement was defined as presence of: pericardial effusion, myocarditis, cardiomyopathy, cardiac failure, Libman-Sacks endocarditis, myocardial infarction, deep vein thrombosis, pulmonary embolism, sinus thrombosis, stroke, arrhythmia, central nervous system vasculitis, or other vasculitis. Echocardiographic data were included in the analysis, when available. Statistical analysis was performed with R software package version 3.4.1.

**Results:** Cardiovascular involvement was present in a total of 44 participants, or 47% of the PULSE cohort (Table 1.) This is much higher than previously published reports of 3-32%. Those with cardiovascular involvement did not differ significantly in gender, age at diagnosis, or race/ethnicity from the overall cohort. Echocardiographic data were available for 23 of these participants. The most common cardiovascular manifestation observed in this cohort was pericardial effusion (n=24), followed by cardiac failure (n=8) and stroke (n=7). Cardiovascular manifestations were frequently severe, with one third of cases of pericardial effusion requiring intervention, and 3 patients presenting with cardiac tamponade. Mortality was high in patients with cardiovascular involvement, at a rate of 20.5%.

**Conclusion:** Cardiovascular involvement was found to be common in this multi-ethnic cohort of South African children with SLE, with pericardial effusion being the commonest cardiovascular manifestation. The mortality rate was high, and severe cardiovascular manifestations including stroke and cardiac tamponade were frequent. Prospective research is required to identify risk factors for cardiovascular involvement, in order to inform practice and improve outcomes for this high-risk population.

**Disclosure of Interest**

None Declared

**Table 1 (abstract P141).** Demographic information and cardiovascular manifestations in a paediatric SLE cohort from South Africa

Demographic/Disease Manifestation	% All patients (N=93)	% Patients with cardiovascular involvement (N=44)	% Patients who had echocardiography (N=23)
Female (%, N)	83 (77)	84 (37)	
Median age of diagnosis (SD)	12 (3.4)	11 (3.5)	
African ancestry (%, N)	91 (85)	91 (40)	
Any cardiovascular manifestation (%, N)	47 (44)	100 (44)	
Pericardial effusion (%, N)	26 (24)	55 (24)	
Stroke (%, N)	8 (7)	16 (7)	
Cardiac failure (%, N)	9 (8)	18 (8)	
Left ventricular hypertrophy (%, N)	--	20 (9)	39 (9)
Mortality (%, N)	10 (9)	20 (9)	

### P142 TREATMENT OF NON-RENAL, NON-NEUROPSYCHIATRIC SYSTEMIC LUPUS ERYTHEMATOSUS IN GERMANY – AN ONLINE SURVEY

#### Claas Hinze^1^, Gerd Ganser^2^, Fabian Speth^3^, Christian Hedrich^4^, Kirsten Moenkemoeller^5^, Catharina Schuetz^6^, Klaus Tenbrock^7^, Johannes-Peter Haas^8^ and PRO-KIND working group SLE of the GKJR

##### ^1^Pediatric Rheumatology and Immunology, University Hospital Münster, Münster; ^2^Pediatric Rheumatology, St. Josef Stift, Sendenhorst; ^3^Pediatric Rheumatology, University Medicine Rostock, Rostock, Germany; ^4^Pediatric Rheumatology, University of Liverpool, Liverpool, UK; ^5^Pediatric Rheumatology, Kinderkrankenhaus Amsterdamer Strasse, Cologne; ^6^University Hospital Ulm, Ulm; ^7^University Hospital Aachen, Aachen; ^8^German Center for Pediatric and Adolescent Rheumatology, Garmisch-Partenkirchen, Germany

###### **Correspondence:** Claas Hinze

**Introduction:** Treatment patterns of non-renal and non-neuropsychiatric systemic lupus erythematosus (non-renal, non-NP SLE) in Germany are not well understood and formal treatment recommendations do not exist, unlike for patients with severe lupus nephritis or neuropsychiatric SLE.

**Objectives:** To assess treatment preferences for patients with non-renal, non-NP SLE among German pediatric rheumatologists.

**Methods:** We designed an online survey including a generic case scenario of a patient with moderate juvenile non-renal, non-NP SLE and one main disease manifestation based on the premise that treatment of SLE is typically dictated by the most severe organ involvement. One question targeted general management of patients with non-renal, non-NP SLE. Then, overall, 21 manifestations of SLE were queried, ranging from constitutional symptoms to various organ-related, and laboratory manifestations and from mild to severe. Responses were given via multiple choice, including various pharmacologic treatment options: non-steroidal anti-inflammatory drugs, various disease-modifying antirheumatic drugs, biologics, i.v. immune globulins and anticoagulants. Descriptive statistics are used to report the findings.

**Results:** Forty-seven pediatric rheumatologists experienced in the management of SLE responded. There is consensus that all patients with non-renal, non-NP SLE should receive hydroxychloroquin (97% or respondents), should be counseled about important preventive measures, including sun protection, contraception, avoiding smoking (100%), that vaccinations should be kept up-to-date (91%) and that vitamin D should be considered (25% in all patients, 25% only in case of glucocorticoid therapy and 47% according to blood levels). Regarding pharmacologic management of the different manifestations, there was overall marked variability. For some life-threatening manifestations (myo-/pericarditis with tamponade, pulmonary hemorrhage and mesenteric vasculitis), the majority of respondents opted for high-dose glucocorticoid therapy and intensive immunosuppression with mycophenolate mofetil or cyclophosphamide, similar to what is often used in severe lupus nephritis. The preferences for different glucocorticoid regimens were especially variable, for example, ranging from none to high-dose for constitutional symptoms or fatigue. Methotrexate was preferred for various musculoskeletal manifestations whereas azathioprin was preferred for cutaneous and hematologic manifestations.

**Conclusion:** Treatment preferences for non-renal, non-NP SLE in Germany among pediatric rheumatologists are markedly variable. There is consensus that all patients with non-renal, non-NP SLE should receive hydroxychloroquin, be counseled regarding preventive measures, that vaccinations should be up-to-date and vitamin D substitution be considered. Data obtained with this survey may be helpful in developing consensus-based strategies for the management of non-renal, non-NP SLE.

**Disclosure of Interest**

None Declared

### P143 TREATMENT OUTCOME IN CHILDHOOD LUPUS NEPHRITIS PATIENTS TREATED WITH MYCOPHENOLATE MOFETIL: REAL-WORLD DATA IN COMPARISON WITH ADULT LUPUS NEPHRITIS

#### Seung Min Jung^1^, Hye Won Kim^2^, Hyun-Sook Kim^3^, Yong-Beom Park^1^

##### ^1^Yonsei University College of Medicine, Seoul; ^2^Seoul National University Bundang Hospital, Seongnam; ^3^Soonchunhyang University Seoul Hospital, Seoul, Korea, Republic Of

###### **Correspondence:** Seung Min Jung

**Introduction:** Childhood-onset systemic lupus erythematosus is a rare and severe autoimmune disease with a high prevalence of lupus nephritis (LN). Although there is a growing interest on treatment with mycophenolate mofetil (MMF) in LN, data on treatment outcome in childhood LN is limited.

**Objectives:** This study aimed to evaluate the therapeutic outcome of MMF in childhood LN from a real-world clinical practice, and compare the clinical characteristics with adult LN.

**Methods:** Korean pediatric and adult patients with pathologically proven LN class III, IV, and V were recruited from rheumatology clinic in Severance Hospital, Yonsei University College of Medicine between Nov 2011 and Aug 2017. Patients who treated with MMF for at least 3 months were included in the analysis. The probability of remission after MMF therapy, and the difference between pediatric patients and adult patients were analyzed using the survival analysis and the descriptive statistics.

**Results:** Of 153 patients with pathologically proven LN class III, IV, and V, 16 pediatric patients and 100 adult patients were included in this study. Pediatric patients showed a more preserved renal function, and adult patients had a higher prevalence of LN class IV. Mean protein/creatinine ratio in spot urine was 3.7 and 4.8 in childhood LN and adult LN, respectively. However, the baseline characteristics showed no statistically significant difference between childhood and adult. During median follow-up period of 5 years (childhood 7.5 years vs. adult 4.7 years), 75 % of pediatric patients and 77% adult patients achieved clinical remission of LN after MMF treatment. Median time to remission after initiation of MMF was shorter in pediatric patients (4.3 months vs 7 months), although statistically insignificant. Risk factors for failure of remission in pediatric patients were nephrotic-range proteinuria and seronegativity of anti-dsDNA antibodies, whereas impaired renal function was also important for predicting remission failure in adult LN.

**Conclusion:** This study shows the real-world data on MMF treatment in childhood LN and adult LN. In childhood LN, MMF has a good therapeutic effect, similar to adult LN.

**Disclosure of Interest**

None Declared

## Autoinflammatory diseases I

### P144 FAVORABLE EFFECT OF TOCILIZUMAB IN BLAU SYNDROME

#### Reem Abdwani

##### Child Health, SULTAN QABOOS UNIVERSITY HOSPITAL, Muscat, Oman

**Introduction:** Blau syndrome (BS) is a rare auto inflammatory granulomatous disorder, characterized by a clinical triad of recurrent uveitis, arthritis and dermatitis. It is a dominantly inherited condition due to mutation in the pattern recognition receptor of NOD gene. No therapeutic trials have been conducted in Blau syndrome and only limited case reports regarding treatment are available

**Objectives:** The aim of this study is to report the first Arab patient with BS who was refractory was conventional therapy but had rapid quiescence after using Tocilizumab. We also conducted a systematic literature review about biologic treatments in BS.

**Methods:** A 3.5 year old boy was referred at the age of 8 month for evaluation of intermittent fever, rash and polyarthritis that started at the age of 2 months. He also had a granulomatous panuveitis with posterior synchia and lens cataract. Skin biopsy revealed non-caseating epitheloid granuloma. DNA analysis identified a heterogenous missense mutation in exon 4 of the NOD2 gene. Over the course of years, patient had a difficulty stormy course with severe polyarthritis that was refractory to conventional treatment including pulses of methlyprednislone, oral prednisolone, methotrexate, adalimumab and infliximab. Tocilizumab 12mg/kg every 2 week was introduced 8 months ago with rapid improvement of arthritis with ability to taper oral prednisolone to 2mg/day. Serum Amyloid levels dropped from 246 mg/L to >6.4 within 3 months. Further attempts to decrease the dose or interval of Tocilizumab infusions were unsuccessful.

**Results:** On literature review, we identified 32 cases of Blau syndrome that were treated with biologic agents; Etanercept (13), Infliximab (14), Adalimumab (10), Anakinra (3), Canakiumab (2), Tocilizumab (3), Abatacept (1). The clinical characteristics and treatment outcome are shown in Table 1 (table could not be inserted due to limitations in number of rows)

**Conclusion:** There is wide diversity in the clinical phenotype of Blau syndrome in terms of age of onset, clinical presentation, disease severity and response to treatment which may be related to functional difference in NOD 2 mutation. Informed consent to publish had been obtained.

**Trial registration identifying number:** not applicable

**Disclosure of Interest**

None Declared

### P145 CHRONIC RECURRENT MULTIFOCAL OSTEOMYELITIS: SINGLE CENTER EXPERIENCE

#### Ceyhun Acari^1^, Hatice Adiguzel Dundar^1^, Serkan Türkuçar^1^, Balahan Makay^2^, Erbil Ünsal^1^

##### ^1^Department of Pediatrics, Pediatric Rheumatology Unit, Dokuz Eylul University, Faculty of Medicine; ^2^Pediatric Rheumatology Unit, Dr. Behcet Uz Children's Hospital, Izmir, Turkey

###### **Correspondence:** Ceyhun Acari

**Introduction:** Chronic recurrent multifocal osteomyelitis (CRMO) is an autoinflammatory bone disease that is characterized by sterile bone lesions. 500 CRMO cases are reported as individual cases or case series in the literature.

**Objectives:** In this study, we aim to share our clinical experience of 10 pediatric patients with CRMO.

**Methods:** The files of patients were retrospectively reviewed by pediatric rheumatologist. The cases were analyzed to define symptoms at onset, clinical findings, laboratory parameters and imaging results. Patients were re-evaluated according to CRMO diagnostic criteria.

**Results:** The mean age of cases was 8.7 years (min:3, max: 13) at diagnosis and 7 patients were male and the others were female. The mean lack time for diagnosis was 8.8 months (min:2, max:24). The mean follow-up of cases was 45.1 months. Magnetic resonance imaging (MRI) in all patients and bone scintigraphy in 9 patients were performed. The diagnosis of 9 cases were confirmed by histopathological examination. Two of the cases were brothers. Other diagnoses were Familial Mediterranean fever in two cases, nephrolithiasis in one case and keratite-bound corneal ulcer in one case. At the time of admission, all patients were suffering from pain of bone and/or joint and showed multifocal involvement such as femur (7 cases), pelvic bones (5), tibia (4), clavicle (2), vertebra (2), ankle (2) and mandible (1). In laboratory results, C-reactive protein level was found as elevated (18.7 mg/L [1.8-78]; mean erythrocyte sedimentation rate was found as elevated (51 mm/h [15-119]. Sulfasalazine and nonsteroid antiinflammatory drugs were used in all cases and methotrexate was added in 6 cases. Three cases required steroid treatment. Resistant cases needed anti-TNF (etanercept or adalimumab) in two patients and IV pamidronate in one patient. The mean time of remission was 19.25 months (min: 6, max:48). Currently, three patients are in remission without medication, and five patients are in remission with medication.

**Conclusion:** Chronic recurrent multifocal osteomyelitis (CRMO) is a rare idiopathic inflammatory disease that affects usually children and young adults. Clinical signs and symptoms are nonspecific and usually the diagnosis is delayed. Diagnosis is based on excluding infections especially osteomyelitis and malignant diseases. Mostly, metaphyseal-diaphyseal region of long bones such as the femur and tibia are involved, Laboratory tests show a mild to moderate acute phase elevation. Nonsteroids are used as the first step in the treatment, but usually sulfasalazine or methotrexate is added, particularly with vertebral involvement. Anti-TNF agents may be effective in patients those unresponsive to conventional treatments and frequent relapses. In recent years, bisphosphonate (pamidronate) is used as an effective and safe agent for the treatment of bone lesions, as well as pain control.

**Disclosure of Interest**

None Declared

### P146 COLCHICINE RESISTANCE IN FMF: POSSIBLE RISK FACTORS AND USE OF ANTI-IL-1

#### Hatice Adıguzel Dundar^1^, Ceyhun Acari^1^, Serkan Turkucar^1^, Ozge Altug gucenmez^2^, Balahan Makay^3^, Sevket Erbil Unsal^1^

##### ^1^Department of pediatrics, child rheumatology, Dokuz Eylul University faculty of medicine; ^2^Department of pediatrics, child rheumatology, Dr. Behcet Uz Childrens' Hospital; ^3^Department of pediatrics, child rheumatology, Dr. Behcet Uz Childrens' Hospital, izmir, Turkey

###### **Correspondence:** Hatice Adıguzel Dundar

**Introduction:** Familial Mediterranean fever (FMF) is the most common autoinflammatory disease with autosomal recessive inheritance. There is MEFV gene mutation encoding the pyrine protein in the short arm of chromosome 16 that leads to overexpression of IL-1. The basic treatment in FMF has been colchicine since 1972. Colchicine is effective both in the prevention and treatment of attacks, and in reducing the frequency of amyloidosis. However, there is resistance to colchicine in 5-10% of FMF cases, and anti interleukin-1 (anti IL-1) is effective in these cases.

**Objectives:** In this study, we aimed to find out possible risk factors for colchicine resistance in FMF by comparing the clinical and demographic data of patients with and without colchicine resistance, and to examine anti IL-1 effectiveness in patients with colchicine resistance

**Methods:** Data charts of children with FMF from Dokuz Eylul University childrens’ hospital and Dr.B.Uz childrens’ hospital (n=950) were reviewed. Despite the use of adequate doses of colchicine, the patients with over 3 attacks in last 6 months were considered to be colchicine resistant. Then, colchicine resistant patients were compared with colchicine responsive group.

**Results:** Thirty four (3.6%) of 951 patients had colchicine resistance and all of them used anti IL-1 (canakinumab). 67.7% of these patients were male and 47.1% had positive family history. Median age of symptoms onset was 40 (24-75) months, median age of diagnosis and colchicine onset were 72 (43-115) months. Median diagnosis delay time was 12 (9-47) months and median time of follow-up was 52 (33-99) months. There was not statistically difference between the 2 groups. Most common symptoms were fever, abdominal pain and musculoskeletal symptoms in both groups, while fever (85.3%), arthralgia (53%), arthritis (41.2%) were significantly higher in group with colchicine resistance when compared with other group (p:0.032, p:0.048 and p:0.001 respectively) Other frequent symptoms were myalgia, chest pain and erysipelas like rash in both of groups. The number of attacks in last 6 months after colchicine treatment was statistically higher in group of colchicine resistance than in other group (median numbers of attack: 6(3-10) (p:0,000). Colchicine resistant group response to canakinumab was good; the median number of attacks was 1(0-2) in the last year

When the groups were compared in terms of FMF gene mutation, exon 10 mutation was 88.2% positive in-group of colchicine resistance, while 67.7% in other group. M694V mutation was also significantly higher when compared with other group (85.3% and 50.2% respectively) (p:0.000).

All patients with colchicine resistance responded the canakinumab treatment except one who had enthesitis related arthritis. There was pneumonia in one patient, urinary tract infection in 2 patients, recurrent upper respiratory tract infection in 2 patients after canakinumab treatment. No serious side effects were reported in the patients that use canakinumab.

**Conclusion:** Colchicine is still best treatment in FMF. 96.4% of our cases responded to colchicine. In cases with colchicine resistance, IL-1 inhibition was successful. Resistant cases had specific features. Male dominance, exon 10 with M694V mutation and arthritis constituted the major properties of this group.

**Disclosure of Interest**

None Declared

### P147 FAMILIAL COLD AUTOINFLAMMATORY SYNDROME-2 (FCAS-2), PRESENTING ONLY WITH RECURRENT ARTHRITIS

#### Hatice Adıguzel Dundar^1^, Serkan Turkucar^2^, Ceyhun Acari^1^, Sevket Erbil Unsal^1^

##### ^1^Department of pediatrics, child rheumatology; ^2^Department of pediatrics, child rheumatology, Dokuz Eylul University faculty of medicine, izmir, Turkey

###### **Correspondence:** Hatice Adıguzel Dundar

**Introduction:** Familial cold auto-inflammatory syndrome-2 (FCAS-2) is an autosomal dominant disorder due to the NLRP12 mutation which plays a role in the regulation of proinflammatory cytokines. It is characterized by fever, headache, joint symptoms, urticarial rash that is triggered by cold; usually starting in early infancy. Episodes are generally last for 5-10 days. It can also be accompanied by conjunctivitis, hearing loss and myalgia.

**Objectives:** Presenting a familial cold auto-inflammatory syndrome case with atypical presentation.

**Methods:** Case presentation.

**Results:** A 17-year-old girl presented to outpatient clinic with recurrent arthritis, urticarial rash for 6 years. She had no accompanying fever in any of the episodes. The frequency of attacks increased in last 2 years, often lasting for 10 days, which were unresponsive to NSAIDs. Attacks were triggered irrelevant from cold exposure. She also had clinical diagnosis of mature onset diabetes in young (MODY), and autoimmune thyroiditis. On physical examination, she had arthritis and urticarial rash on left ankle. Acute phase reactants were increased. FMF was the initial diagnosis, however no mutation was found in MEFV gene sequencing. Auto-inflammatory next generation sequencing (NGS) panel demonstrated NLRP-12 gene mutation (p.Arg352Cys) compatible with FCAS-2. Colchicine treatment (1.5 mg/day) was able to provide disease control.

**Conclusion:** Familial cold auto-inflammatory syndrome (FCAS) is characterized by recurrent attacks of cold-triggered fever, arthritis and urticarial rash mostly starting in infancy. This case differs from the classical pattern by old age, and it seems fever is not always one of the presenting symptoms. Informed consent to publish had been obtained from the parent.

**Trial registration identifying number:** Note: Followings case report contains a method which in our thought is not appropriate in a case report abstract. Hence, the method was depicted as “case presentation”. If committee have any suggestion in following days it could be changed accordingly.

**Disclosure of Interest**

None Declared

### P148 MUSCLE ASEPTIC ABSCESSES IN PEDIATRIC PATIENTS DIAGNOSED WITH SYSTEMIC ONSET JUVENILE IDIOPATHIC ARTHRITIS OR POLYARTHRITIS: IS IT A NEW AUTO-INFLAMMATORY DISEASE?

#### Kokou Placide Agbo-Kpati^1^, Pierre Quartier^2^, Sylvain Breton^3^, Brigitte Bader-Meunier^4^

##### ^1^Pediatrie, GHEF-Site de Marne La Vallée Jossigny, JOSSIGNY; ^2^Pediatrie, Paris-Descartes University, Paris, France, 4Imagine Institut and RAISE Reference Center, Pediatric Immunology-Hematology and Rheumatology Unit, Hôpital Necker-Enfants Malades, Assistance Publique-Hôpitaux de Paris, Paris, France; ^3^Radiology, Pediatric Radiology Department, Hôpital Necker-Enfants Malades, Assistance Publique-Hôpitaux de Paris, Paris France; ^4^Pediatrie, Paris-Descartes University, Paris, France, 4Imagine Institut and RAISE Reference Center, Pediatric Immunology-Hematology and Rheumatology Unit, Hôpital Necker-Enfants Malades, Assistance Publique-Hôpitaux de Paris, Paris, France., Paris, France

###### **Correspondence:** Kokou Placide Agbo-Kpati

**Introduction:** Abscesses Aseptic (AA) syndrome is an autoinflammatory disorder mostly reported in adult, of unknown etiology characterized by occurrence of fever, AA and elevated acute phase reactants. It responds to steroid treatment. It is either associated with inflammatory diseases, such as Crohn's disease or relapsing polychondritis, or as « idiopathic ». Pediatric-onset AA has been reported in only 8 patients and never in association with JIA.

**Objectives:** To report the association of JIA and muscle AA.

**Methods:** we retrospectively analyzed the medical notes from patients followed in two pediatric departments from the French FAI2R network for rare autoimmune and autoinflammatory diseases who presented both Juvenile Idiopathic Arthritis and multiple aseptic muscle abscesses. Patients had been included in the CEMARA platform for rare diseases, which benefits from an agreement from the French Commission Nationale Informatique et Liberté. According to the French legislation, no ethics committee agreement was requested for such a retrospective, observational survey.

**Results:** We identified 3 male patients diagnosed with JIA between the age of 4 and 5 years. Patient 1 had systemic-onset with spiking fever, polyarthritis, myalgia, pericarditis; patient 2 had a seronegative polyarticular JIA; patient 3 had Rhumatoid Factor and ANA positive polyarticular JIA. There was no familial anamnesis of interest and the 3 patients were born from unrelated parents. Biological inflammation was present at in all cases with CRP > 70mg/L at diagnosis. Muscle abscesses developed 4, 30 and 48 months respectively after the diagnosis of JIA. At this time, patient 1 was on oral steroids, patient 2 on remission off treatment and patient 3 on oral steroids and subcutaneous etarnercept. Abscesses were revealed by painful tumefactions, associated with fever, flare of polyarthritis and an elevation of CRP. Ultrasound and MRI revealed a collection consistent with abscess in the biceps (patients 1 and 2) or the mesogluteus muscles (Patient 3). Corticosteroids and biologics were stopped and surgical drainage was performed in patient 1 and 2, showing an exudate of polymorphonuclear neutrophils. An extensive microbiological screen for bacteria, fungi and mycobacteria was negative in both cases. We also ruled out in the 3 patients chronic granulomatous disease as well as XIAP and PSTPIP1 mutations. In the 3 patients after 7 to 10 days on large spectrum of antibiotic treatment with no effect on lesions nor on the CRP level, steroid 1 to 2mg/kg/day were re-introduced or initiated, which was associated with quick improvement and a complete resolution of symptoms within 5 to 10 days. After tapering the dose of steroid, patient 1 developed a relapsing-remitting course, with recurrent biceps muscle abscesses that responded each time to increase of the steroid dosage. After 4 to 5 years from the onset of abscesses and the failure of several treatments including, in 2 cases the interleukin (IL)-1 receptor antagonist anakinra, all 3 patients were put on the anti-IL-6 receptor antibody tocilizumab and achieved remission that persisted, off steroid at the latest follow-up, 10 to 12 years later. However, severe bilateral erosive hips arthritis had developed in two patients before the onset of tocilizumab.

**Conclusion:** The diagnosis of muscle AA should be considered in a patient with abscesses associated with polyarthritis, particularly in the absence of documented infection or response to antibiotics. The association of early-onset polyarthritis, systemic inflammation and aseptic muscle abscess is likely a peculiar autoinflammatory disorder that may respond to anti IL-6 blockade.

**Disclosure of Interest**

None Declared

### P149 COMPARISON OF THE DEMOGRAPHIC, CLINICAL AND GENETIC DATA OF CHILDREN WITH FMF WHOSE SYMPTOMS BEGIN BEFORE AGE OF 5 WITH THE CHILDREN HAVING ONSET OF SYMPTOMS AFTER AGE OF 5.

#### Gökçen Erfidan, Gül Karadağ, Ayşe Zopçuk, Mustafa Çakan, Nuray Aktay Ayaz

##### Pediatric Rheumatology, Sağlık Bilimleri University KSSEAH, Istanbul, Turkey

###### **Correspondence:** Nuray Aktay Ayaz

**Introduction:** Familial Mediterranean fever (FMF) is an inherited autoinflammatory disease characterized by recurrent episodes of fever and acute inflammation of the membranes lining the abdomen, joints. FMF usually begins during childhood. Most affected individuals experience their initial episode before the age of 20.

**Objectives:** We aimed to compare the demographic, clinical and genetic data of the patients whose symptoms started before the age five and after the age five to see the effect of age to the severity and outcome of disease, to evaluate the presence of mutations according the the age of symproms onset.

**Methods:** Children who are diagnosed as FMF at Sağlık Bilimleri University KSSEAH Pediatric Rheumatology outpatient-clinic according to Tel-Hashomer Criteria were involved to the study. Patients were grouped based to the age of onset of the symptoms related to FMF. In the first group consisted 100 children with the clinical features that began before age of 5 and the second group consisted 100 children with the clinical features starting after age of 5. All the patients were carrying either heterozygous or homozygous MEFV mutations. Retrospective analysis of the files of the patients were done. Demographic data, genetic analysis, consanguinity, family history, age that first symptom ensue, duration passed until the diagnosis was made, frequency of attacks, duration of attacks and the involved sites during attacks, reports of MEFV analysis, laboratory data including CRP, ESR, SAA, blood counts, urinary protein levels were recorded. Severity of the disease were evaluated according to the Pras score.

S

**Results:** Mean age of the patients was 11.7±4.04. Mean age of the children whose symptoms began before age of 5 (Group 1) was 9.65±3.83 years, mean age of the children whose symptoms began after age of 5 (Group 2) was 13.7±3.09 years. Group 1 consisted 57 girls, group 2 consisted 46 girls. Consanguinity was 35% for group 1and 21% for group 2 (p<0.05). Clinical features started at the mean age of 2.37±1.19 years in group 1 and 8±2.39 years in group 2. The duration that passed till diagnosis was 2.19±1.99 years and was not different between groups. Although group 1 had more frequent attacks than group 2, the duration af attacks were similar in both groups. Colchicine dose was more than the dose needed for age in group 1, 14% of children needed increased doses, while in group 2 only 2% needed higher doses appropriate for their age (p<0.05). Fever and abdominal pain was present in 64% of all patients as the first symptom. Fever only was the presenting symptom in 19 children under age 5, while non of the patienst in group 2 had just fever as the presenting symptom (p<0.05). Artrhitis and chest pain were the presenting symptoms in group 2, but not the first symptom in group 1 (p<0.05). Mean Pras score of patients in group 1 was significantly higher (7.35±1.24) than the mean score of group 2 (6.14±1.55). Homozygous M694V mutations were statistically more frequent in group 1 (p<0.05). Leukocyte counts and CRP levels were higher, hemoglobin concentrations were lower during attacks in group 1 than group 2 (p<0.05).

**Conclusion:** Age of onset of FMF is an important factor while eavluating clinical features and severity of the disease. Increased doses of colchicine may be needed in children with early onset of disease. Severe disease causing mutations are also frequently seen when the disease starts early. Presence of recurrent fevers even without accompanying symptoms must warn the physician about the presence of FMF that will prevent the delay in diagnosis. So, both the complications like amyloidosis and the severe burden of disease will be avoided

**Disclosure of Interest**

None Declared

### P150 TARGETED PERSONALIZED BIOLOGIC THERAPY IN CHILDREN WITH AUTOINFLAMMATORY SYNDROMES

#### Ekaterina Alexeeva^1,2^, Tatyana Dvoryakovskaya^1,2^, Rina Denisova^1^, Tatyana Sleptsova^1^, Kseniya Isaeva^1^, Alexandra Chomahidze^1^, Anna Fetisova^1^, Anna Mamutova^1^, Victor Gladkikh^3^, Alina Alshevskaya^3^, Andrey Moskalev^3^

##### ^1^Federal State Autonomous Institution “National Medical Research Center of Children's Health” of the Ministry of Health of the Russian Federation; ^2^Federal State Autonomous Educational Institution of Higher Education I.M. Sechenov First Moscow State Medical University of the Ministry of Health of the Russian Federation, Moscow; ^3^Biostatistics and Clinical Trials Center, Novosibirsk, Russian Federation

###### **Correspondence:** Ekaterina Alexeeva

**Introduction:** Misdiagnosis in patients with such autoinflammatory syndromes as juvenile idiopathic arthritis (JIA) occurs rather frequently in routine practice. Because of the heterogeneity of JIA and the lack of widespread genetic testing, children with autoinflammatory syndromes can be treated for many years using the standard protocols before they turn out to be ineffective.

**Objectives:** The objective of this study was to estimate the incidence of autoinflammatory syndromes among biologic-naïve patients with JIA and those with a long-term disease, as well as the ineffectiveness of therapy with one or more biologics.

**Methods:** The prospective study conducted at the National Medical Research Center of Children's Health (Moscow) involved 300 patients having a diagnosis of systemic JIA at enrollment. All the patients underwent automated direct sequencing on an ABI 3500 XL automated DNA sequencer (Applied Biosystems) using a BigDye Terminator Cycle Sequencing Ready Reaction kit (Applied Biosystems, Foster City, CA, USA). If no mutations were detected in the hot spots of the *MEFV*, *MVK*, *NLRP3*, and *TNFRSF1A* genes, all the coding and the adjacent intronic regions of the *MVK*, *MEFV*, *TNFRSF1A*, *NLRP3*, *NLRP12*, *CECR1*, *IL1RN*, *TMEM173*, *NLRC4*, *LACC1*, *NOD2*, *PSTPIP*, *LPIN2*, *PSMB8*, and *TRNT1* genes in patients with suspected autoinflammatory syndromes were examined by next-generation sequencing, which allows one to simultaneously analyze extensive areas of the human genome. After genetic verification of the diagnosis, the patients were assigned to the groups receiving either TNFα inhibitors, or anti-IL6 receptor monoclonal antibodies, anti-IL1 monoclonal antibodies. At the final visit (within 1 year), the efficacy and safety of targeted personalized biologic therapy was evaluated.

**Results:** Among the 300 patients enrolled, 180 were biologic-naïve (60%, the naïve group). Among the remaining cohort (40%, the switched group), 27% of patients were previously treated with 2 or more biologics. Prior to molecular genetic examination, only 16% of patients had inactive disease. An ACR70 response (compared with the disease onset) was achieved in 16.3% of patients (27 (15%) naïve and 22 (18.3%) switched patients). Only 8% of patients (13 (7.2%) naïve and 11 (3.6%) switched patients) reached the inactive disease phase/remission.

The conducted molecular genetic study showed that biologic therapy was personalized in 53/180 (29%) naïve patients and 46/120 (38%) switched patients according to the revealed pathogenic variants of the genome. Personalized treatment reduced the JADAS71 index down to < 3 in 71% of patients and allowed 70% of children achieve the inactive disease phase and remission after one-year follow-up

**Conclusion:** The frequency of pathogenic variants of the genome and autoinflammatory syndromes in patients with juvenile idiopathic arthritis is high. The revealed genomic abnormalities make it possible to personalize biologic therapy, which allows achieving the remission rate of > 70%.

**Disclosure of Interest**

None Declared

### P151 THE CHARACTERISTICS OF PEDIATRIC FAMILIAL MEDITERRANEAN FEVER WHO ARE USING CANAKINUMAB

#### Ozge Altug Gucenmez^1^, Balahan Makay^1^, Neslisah Uslu^2^

##### ^1^Izmir Dr. Behcet Uz Children’s Hospital, Izmir; ^2^Koc University, Istanbul, Turkey

###### **Correspondence:** Ozge Altug Gucenmez

**Introduction:** Familial Mediterranean Fever (FMF) is an inflammatory disease related to MEFV gene mutations which is characterized recurrent fever and abdominal pain. Colchicine is used for preventing attacks as the routine treatment. However, the effect of anti-interleukin-1 (IL-1) was shown in colchicine resistant patients.

**Objectives:** It is aimed to present the characteristics of pediatric FMF patients who followed with an anti-IL-1 agent, canakinumab.

**Methods:** The medical files of 29 pediatric FMF patients who are followed in Izmir Dr. Behcet Uz Children’s Hospital were investigated retrospectively.

**Results:** Seventeen male and 12 female cases with colchicine resistant FMF were included in the study. The cases with three or more attacks in the last six months and high AFR while using adequate dose colchicine were defined as the colchicine resistant. The median age was 16 years (min-max: 4.5-19 years), median symptom onset age was 4 years (min-max: 1-15 years), median diagnosis age was 6 years (min-max: 2-15 years), and median follow-up time was 7 years (min-max: 2-14 years). Twenty-two cases were (75.9%) M694V homozygote. Recurrent fever (100%), abdominal pain (86.2%), and arthritis (48.3%) were the most common symptoms. The cases used colchicine for median 7 months (min-max: 2-14 months) prior to canakinumab. Four of the cases (13.8%) have tried anakinra treatment prior to canakinumab. Canakinumab was started with 3-5 mg/kg (max: 150 mg). The cases in the present study were used canakinumab for 11 months (min-max: 2-76 months). Two cases had a urinary tract infection (6.9%), and pneumonia was developed in one case (3.4%) following the canakinumab. Total remission was achieved in 26 cases (89.7%) following canakinumab treatment.

**Conclusion:** Although the colchicine is still the first line therapy in the FMF, anti-IL-1 agents provide effective treatment in colchicine resistant cases. Canakinumab is a promising agent which controls FMF attacks effectively. According to our experience, the colchicine resistant FMF patients might achieve high rates of total remission without severe adverse effects following canakinumab treatment.

**Disclosure of Interest**

None Declared

### P152 CHRONIC RECURRENT MULTIFOCAL OSTEOMYELITIS: FOUR TERTIARY SPANISH HOSPITALS EXPERIENCE. A MULTICENTRIC STUDY.

#### Antía García Fernández^1^, Jaime Arroyo Palomo^1^, Daniel Clemente Garrulo^2^, Belén Serrano Benavente^3^, Javier Nóvoa Medina^4^, Juan Carlos Nieto González^3^, Sergio Machín García^4^, Anahy María Brandy García^5^, Juan Carlos López Robledillo^2^, Indalecio Monteagudo Sáez^3^, Alina Lucica Boteanu^1^

##### ^1^Paediatric Rheumatology, Ramón y Cajal University Hospital; ^2^Paediatric Rheumatology, Niño Jesús University Child Hospital; ^3^Paediatric Rheumatology, Gregorio Marañón General University Hospital, Madrid; ^4^Paediatric Rheumatology, Insular Maternity Child University Hospital of Gran Canaria, Las Palmas de Gran Canaria; ^5^Rheumatology, Central University Clinic Hospital of Asturias, Oviedo, Spain

###### **Correspondence:** Jaime Arroyo Palomo

**Introduction:** Chronic recurrent multifocal osteomyelitis (CRMO) is a rare autoinflammatory polygenic bone disease characterized by aseptic bone inflammation that affects more frequently pediatric population. Its management, clinical, radiological findings and treatment have not yet been standardized.

**Objectives:** The clinical, radiological characteristics were analyzed as well as response to treatment options.

**Methods:** We performed a retrospective, descriptive multicentric study of patients diagnosed of CRMO in four tertiary level hospitals’ pediatric rheumatology section. There were 16 patients included.

**Results:** The median age at diagnosis was 10,5 years, female:male ratio 62,5:37,5%. The delay in the diagnosis had a median of 4.5 months, being less than one year in 11 patients,taking up to 24 months in a patient with unifocal pelvic involvement. Bone pain was the first symptom in 100% of the patients accompanied by fever in 25% of them. A single patient presented perilesional arthritis. A slight-moderate increase on acute fase reactants was observed at the debut of the disease: median ESR 47.5mm/h.

The median number of locations at disease onset was 2.5 (range 1-14), with multifocal involvement in 75%. The most frequent location was tibia (56%), followed by pelvis (44%) and vertebrae (31,25%). Other locations less frequent were: carpus (12.5%), femur (12.5%) mandible (6%) and sternum (6%).

Biopsy was performed in 14/16 patients and bone scintigraphy with Tc99 in 12/16 patients, with pathological uptake observed in 91.6% of cases. MRI was the radiological test of suspected diagnosis in 15/16 patients.

NSAIDs were the initial treatment being Ibuprofen and Naproxen the most frequently used. 5 patients received different antibiotic therapy regimens, without clinical or radiological improvement. 56.25% of patients required other treatments. Systemic corticosteroids were used in 12.5% of patients and bisphosphonates in 43.75%(100% of patients with axial involvement). After 6 months of treatment with biphosphonates, 57.14% had complete remission, 28.57% partial remission and 14.28% worsening. 12.5% of the patients had a torpid evolution, receiving sequential therapies with multiple synthetic or biological DMARDs (Anakinra, Canakinumab, Etanercept), and another 12.5% required surgery.

**Conclusion:** The diagnosis of CRMO represents a big challenge in the absence of pathognomonic features which frequently leads to delay in diagnosis and the initiation of treatment. In our centers the biphosphonates were the treatment strategy used in patients with spinal involvement with 85.67% response at 6 months.

**Disclosure of Interest**

None Declared

### P153 A MALE WITH BLAU SYNDROME AND INFLAMMATORY CNS MANIFESTATION

#### Stefan Berg^1^, Anders Fasth^1^, Gunilla Drake^1^, Yonas Berhane^2^

##### ^1^Dept of Pediatrics, Institute of Clinical Sciences, Gothenburg; ^2^Dept of Pediatrics, Sunderby Hospital, Luleå, Sweden

###### **Correspondence:** Stefan Berg

**Introduction:** Blau syndrome is an autoinflammatory syndrome caused by a mutation in *NOD2*. The syndrome is often a challenge to treat. A variety of anti-inflammatory agents have been used but none has so far being universally effective.

We describe an 18 year-old boy previously treated with several non-biologic DMARDS in combination with TNF-blockade, Il-1 blockade and IL-6 blockade. He has never been free of inflammation and always needed oral corticosteroids (0.15-0.20 mg/kg). His main clinical problems have been arthritis and uveitis. During the last 5 years he had intermittent headache that seems to correlate with flares of the disease. In December 2017 his headache became worse.

**Objectives:** Investigate CNS symptoms in a child with Blau syndrome

**Methods:** Repeated lumbar punctures and serial measurements of CSF biomarkers were performed. Extensive infectious work-up was done.

**Results:** Repeated MRI of the brain between 2011 and 2017 did not show any progress but only a discrete periventricular signaling of unclear significance around both anterior horns. Brain MRI 2018 was unchanged compared to earlier MRIs. The infectious work-up was normal.

**Table:** 

	***CSF findings***			
	181228	180131	180321	180503
opening	27	27	47	33
pressure cm H_2_O				
WBC count (/μl)	188	45	6	7
Lymphocytes (/μl)	180	44	6	6
Neutrophils (/μl )	8	1	0	1
Protein (g/L)	0,71	normal	normal	
NFL(<380ng/L)	4350	4740	50700	pending
GFA(<175ng/L)	390	670	330	pending
Tau (<250 ng/L)	759	526	500	pending
Glucose(mmol/L)	3.0	3.1		
Lactate (mmol/L)	2.3	1.8		
***Serum findings***				
181228; CRP 4 mg/L, WBC 17.1 x 10^9^/L, ANC 13.2 x 10^9^/L, ALC 2.4 x 10^9^/L				

His symptoms and CSF leukocytes have decreased after increasing the corticosteroids and after changing from prednisolone to dexamethasone with better penetrance of the blood-brain barrier.

**Conclusion:** CNS inflammation in Blau syndrome was not described earlier. The results of the investigations clearly points to CNS inflammation in this teenager and that this is possibly a part of Blau syndrome. The late increase of NFL is reflecting the dynamics of this biomarker and not an evidence of worsening of the CNS involvement. Our conclusion is that CNF involvement should be part of the work-up of children with Blau syndrome.

**Disclosure of Interest**

None Declared

### P154 CHARACTERIZATION OF A HOMOZYGOUS KNOCK-IN MOUSE MODEL FOR NLRP3 GENE THAT DEVELOP CINCA DISEASE

#### Arinna Bertoni^1^, Sonia Carta^2^, Chiara Baldovini^3^, Federica Penco^1^, Enrica Balza^2^, Silvia Borghini^4^, Michele Fiore^4^, Paolo Nozza^3^, Emanuela Ognio^5^, Francesca Schena^1^, Marco Di Duca^4^, Roberta Caorsi^1^, Isabella Ceccherini^4^, Alberto Martini^1^, Marco Gattorno^1^, Anna Rubartelli^2^, Sabrina Chiesa^1^

##### ^1^Centro Malattie Autoinfiammatorie ed Immunodeficienze, ISTITUTO GIANNINA GASLINI; ^2^Unità di Biologia Cellulare, San Martino-IST; ^3^UOC Anatomia Patologica; ^4^Genetica Medica, ISTITUTO GIANNINA GASLINI; ^5^UOS Animal Facility, San Martino-IST, Genova, Italy

###### **Correspondence:** Arinna Bertoni

**Introduction:** Cryopirin associated periodic syndromes (CAPS) are autoinflammatory diseases associated to NLRP3 gene mutations, leading to inflammasome hyperactivity and IL-1βhypersecretion. Three phenotypes (FCAS, MWS and CINCA) represent a continuum of the same disease characterized by different severity degrees. Chronic infantile neurologic, cutaneous, articular (CINCA) patients, affected by the most severe form, presented recurrent fever, systemic inflammation and central nervous system disabilities.

**Objectives:**

- increase the knowledge on pathologic consequences of NLRP3 mutations;
- understand central nervous system involvement;
- identify new drugs and molecular targets for the treatment of CAPS diseases.

**Methods:** Cytokines secretion from bone marrow derived dendritic cells (BMDCs) was evaluated by ELISA.

Hystological analysis was evaluated with hematoxylin and eosin staining.

**Results:** We engineered N475K mutation (corresponding to mutation N477K in human) in mouse NLRP3 gene and generated a novel knock-in (KI) model that presents two different phenotypes: heterozygous and homozygous form. Although both KI mimic CAPS human disease, homozygous (homo)-KI mice presented a much more severe phenotype and exhibited typical clinical manifestation of CINCA patients. Homo-KI mice showed severe skin rush and growth delay respect to Wild Type (WT) controls. Survival and body weight were severely decreased. Interestingly, IL-1β, IL-18 and IL-1α secretion was strongly increased respect to WT and heterozygous mice of the same littermate mice, while IL-1RA antagonist secretion was extremely reduced when compared to the same littermate mice.

Hystological analysis showed a generalized inflammatory phenotype characterized by alteration of spleen, lungs, liver and kidneys. More interestingly, homo-KI mice presented serious cerebral complication that prevent walking. Hematoxylin and eosin staining reveal a delay in cerebellum development and an alteration of the lamination of cerebral cortex.

**Conclusion:** Homo-KI mice recapitulate clinical and immunological features of CINCA patients. In the future this mouse model could be used to gain insights into the mechanisms associated to neurological defects and their consequent treatment.

**Disclosure of Interest**

None Declared

### P155 CHRONIC NON INFECTIOUS OSTEITIS- A MULTICENTRE STUDY

#### Chandrika Bhat^1^, Catriona Anderson^2^, Aoibhan Harbinson^3^, Liza McCann^3^, Marion Roderick^1^, Adam Finn^4^, Joyce Davidson^2^, Athimalaipet Ramanan^5^

##### ^1^BRISTOL ROYAL HOSPITAL FOR CHILDREN, Bristol; ^2^Scottish Paediatric and Adolescent Rheumatology Network, Glasgow; ^3^Alder Hey Children’s NHS Foundation Trust, Liverpool; ^4^University of Bristol; ^5^Bristol Medical School, Bristol, UK

###### **Correspondence:** Chandrika Bhat

**Introduction:** Chronic non-infectious osteitis (CNO) or Chronic recurrent multifocal osteomyelitis (CRMO) is a rare auto inflammatory disorder characterised by the presence of sterile bone lesions. The disease affects the metaphyses of long bones, pelvis, vertebra and clavicle.

**Objectives:** To understand the demographics, clinical features and treatment outcomes of Chronic Non-Infectious Osteitis (CNO) from three tertiary paediatric rheumatology services in the United Kingdom.

**Methods:** Children less than 18 years of age diagnosed with CNO between 2001 to 2016 from one tertiary service and between 2001 to 2017 from two tertiary services were included. Clinical notes were reviewed and all pertinent data were collected on a pre-defined proforma. One hundred and thirty one patients were included in the study. The Bristol diagnostic criteria was applied retrospectively.

**Results:** Retrospective analysis of the data showed that the disease was more common in girls than boys (2.5:1), the mean ± SD age at diagnosis was 10.64 ± 2.93 years and the mean ± SD time to diagnosis was 16.48 ± SD 16.18 months. Bone pain was the predominant symptom in 118/129 (91.4%) followed by swelling in 50/102 (49.01%). Associated auto inflammatory conditions included IBD (n=1) and psoriasis (n=5). Raised inflammatory markers were present in 39.68% of the patients. Whole body Magnetic Resonance Imaging (MRI) was a useful diagnostic tool. Metaphyses of long bones were most often involved and the distal tibial metaphyses 65/131 (49.6%) was the most common site. With the use of Bristol Diagnostic Criteria 31.50% of the bone biopsies could have been avoided. Non-steroidal anti-inflammatory drugs were used as first line (81.67%) followed by bisphosphonates (61.79%). The disease was in remission in 80.91% of the patients during the last follow up and 17.55% had recurrent disease.

**Table:** 

CHARACTERISTICS	NUMBER(%)
Age at diagnosis (mean±SD years)	10.64± 2.93
Gender F/M;ratio	94/37;2.54
Fever (n=79)	6(7.59%)
Raised inflammatory markers(n=126)	50(39.68%)
HLA B27 positive(n=15)	1(6.66%)
Number of clinical sites	1.29 (range 0-3)
Number of radiological sites	3.27 (range 1-13)

**Conclusion:** Our multicentre study describes features and outcomes of CNO in a large number of patients in the United Kingdom. It also highlights the ability to avoid biopsies in some children and the need to develop standardised pathways for management of CNO.

**Disclosure of Interest**

None Declared

### P156 VACCINATION IN CHILDREN WITH PFAPA (PERIODIC FEVER WITH APHTHOUS STOMATITIS, PHARYNGITIS, AND ADENITIS) SYNDROME AND OTHER AUTOINFLAMMATORY DISEASES

#### Maša Bizjak^1^, Tadej Avčin^1,2^, Nataša Toplak^1,2^

##### ^1^Department of Allergology, Rheumatology and Clinical Immunology, University Children's Hospital; ^2^Medical faculty, University of Ljubljana, Ljubljana, Slovenia

###### **Correspondence:** Maša Bizjak

**Introduction:** Immunologic processes are altered in patients with autoinflammatory diseases. Little is known about efficacy and safety of vaccinations in these patients, which might affect decision to vaccinate.

**Objectives:** The aim of this study was to determine vaccination coverage in children with PFAPA syndrome and other autoinflammatory diseases, to find out the reasons for potential vaccination dropout and to assess the frequency and nature of potential side effects after vaccination in these patients.

**Methods:** All children with PFAPA syndrome and other autoinflammatory diseases who visited rheumatology outpatient clinic at the University Children's Hospital (UCH) Ljubljana since 2008 were invited to participate in the study. They were asked to provide a written vaccination record and they were sent a questionnaire about the reasons for potential vaccination dropout and potential side effects after vaccination. The study was approved by the national ethics committee. Hereby we report the preliminary results of the study.

**Results:** Since 2008, 191 children with autoinflammatory diseases (including 147 children with PFAPA syndrome) were seen in rheumatology outpatient clinic at UCH Ljubljana. By the end of April 2018 we sent the questionnaires to 78 patients, 26 (33 %) returned the data. Among them, 23 had PFAPA, 2 FMF and 1 CAPS. 17 (65 %) were boys. Median age was 7 (3.5 – 19.2) years, median age at diagnosis, disease onset and end of fever episodes in children with PFAPA was 3.5 (1.6 – 7) years, 1.9 (0.08 – 3.8) years and 6 (3 – 10) years, respectively. Six of the patients with PFAPA still have fever episodes at this point. Five of the patients with PFAPA received methylprednisolone at least once and 9 had a tonsillectomy. Twenty-two (84.6 %) patients had a complete vaccination status according to the Slovenian immunization programme. Reasons for vaccination dropout included recommendation of the primary physician, fear of side effects of vaccination and recurring febrile illness. 6 patients had fever 0-7 days after vaccination that lasted 2-14 days, one of them also had a rash and another one had diarrhoea and vomiting. None experienced serious side effects or disease flares.

**Conclusion:** Vaccination coverage in children with autoinflammatory diseases seems to be lower than in general population in Slovenia. Vaccination didn’t cause serious side effects or worsening of the autoinflammatory disease in our cohort.

**Disclosure of Interest**

None Declared

### P157 CORNELIA DE LANGE SYNDROME WITH A NOVEL MUTATION IN SMC1A GENE IN PATIENT WITH AICARDI-GOUTIÈRES SYNDROME

#### Sorina Boiu^1^, Andrianos Nezos^2^, Isabelle Melki^3^, Argyrios Dinopoulos^4^, Manolis Gialitakis^5^, Erato Atsali^1^, Kleio Mavragani^2^, Periklis Makrythanasis^6^, Vassiliki Papaevangelou^4^, Dimitrios Boumpas^5,7^, Aggelos Banos^5^

##### ^1^Pediatric Rheumatology Unit, Third Department of Pediatrics, “Attikon” University Hospital, National and Kapodistrian University of Athens; ^2^Department of Physiology, School of Medicine, National and Kapodistrian University of Athens, Athens, Greece; ^3^Laboratory of Neurogenetics and Neuroinflammation, INSERM, Paris, France; ^4^Third Department of Pediatrics, “Attikon” University Hospital, National and Kapodistrian University of Athens; ^5^Lab of Autoimmunity and Inflammation; ^6^Systems Biology, Biomedical Research Institute of the Academy of Athens; ^7^Fourth Department of Medicine, “Attikon” University Hospital, National and Kapodistrian University of Athens, Athens, Greece

###### **Correspondence:** Sorina Boiu

**Introduction:** Type I interferonopathies are a clinically heterogeneous group of Mendelian disorders characterized by constitutive upregulation of type I interferon activity. Aicardi-Goutières syndrome (AGS) manifests as an early-onset encephalopathy and mimics a congenital viral infection. Cohesinopathies such as Cornelia De Lange syndrome, are characterized by both physical and mental abnormalities. Mutations in cohesin complex genes (*NIPBL, SMC1, SMC3, RAD21, HDAC8*) result in increased sensitivity to DNA damage and exhibit global gene expression deregulation. However, the true extent of the phenotype associated with pathogenic variants in CdL-related genes is not yet known.

**Objectives:** To report on a patient with combined Cornelia de Lange syndrome and AGS.

**Methods:** A twenty years old male presented since early infancy with violaceous, scaling lesions of the fingers and toes, resorption of distal phalanges, dystrophic nail changes and, violaceous lesions on the nose that worsened during the cold season. He was diagnosed at the age of 7 years with Cornelia de Lange syndrome (CdLS) based on the phenotype associating severe somatic and psychomotor retardation, microcephaly, hypertrichosis, synophrys, arched palate, strabismus, hearing loss, cryptorchidism and unilateral vesicoureteral reflux with kidney scarring. Laboratory investigations showed mild thrombocytopenia and positive anti-thyroid antibodies. A cerebral MRI showed an important intracerebral large vessel involvement with cerebral vascular accidents. The patient was found to be homozygous for a novel NM_015474.3:c.66del:p.(Ser23Glnfs*43) variant in *SAMHD1* gene*.* Whole Exome Sequencing (WES) of the patient was performed. PBMCs were isolated from peripheral blood and cell subsets were monitored for aberrations compared to healthy donors. DNA damage was assayed in cell subsets through phospho-γ-H2AX staining. Finally, RNA-seq (in progress) for CD14^+^ monocytes and total PBMCs of patient will provide a list of differentially expressed genes as well as explain immune abnormalities correlating to the patient phenotype.

**Results:** Using WES, we confirmed the presence of the homozygous mutation in *SAMHD1* gene. Moreover, a novel mutation of *SMC1A* gene associated with Cornelia de Lange syndrome was identified (*SMC1A*, NM_006306.3:c.3306C>A :p.(Asn1102Lys)). The population of classical monocytes (CD14^+^CD16^-^) in the patient was enhanced compared to healthy individuals, while intermediate and non-classical monocytes were not significantly altered. Total T cell abundance was different (reduced at 50%) and much lower for dendritic cells. Levels of DNA damage in PBMCs of patient seem rather similar to healhty ones. RNA-seq analysis is in progress for CD14^+^ monocytes and total PBMCs of patient.

**Conclusion:** We describe a novel mutation in *SMC1A* gene, diagnosing genetically Cornelia de Lange syndrome in a patient diagnosed already with AGS. Immune cell abundance abnormalities are expected to reflect gene expression alterations. Informed consent to publish has been obtained from the parents.

**Disclosure of Interest**

None Declared

### P158 APPLICABILITY OF THE PROVISIONAL CRITERIA FOR CHILDREN BEHCET DISEASE IN A SERIES OF PATIENTS FOLLOWING IN A PEDIATRIC RHEUMATOLOGY UNIT

#### Alina L. Boteanu, Adela Alía Jiménez, Sixto Zegarra Mondragón, Maria Angeles Blazquez Cañamero

##### Rheumatology, University Hospital RAMÓN Y CAJAL, MADRID, Spain

###### **Correspondence:** Alina L. Boteanu

**Introduction:** Behçet's disease (BD) usually appears in young adults between 25 and 40 years of age, although in a smaller percentage of patients the disease can begin in the pediatric age. Classically, the criteria published by the International Study Group of EB (ISGBD-1990) have been used for the diagnosis, but recently new criteria have been proposed of the Study Group on Childhood EB (PEDBD2016) in order to impove the sensitive and specific

**Objectives:** Assess the applicability of the new criteria for the childhood BD in a cohort of patients previously  diagnosed with BD, comparing these with the classification criteria previously used (ISGB1990 and ITR-ICBD 2014) and compare with an adult cohort.

**Methods:** This is a retrospective cross-sectional observational study. Clinical and analytical variables were collected from 9 juvenile patients previously diagnosed with childhood BD, according to criteria of ISGB 1990 and / or of ITR-ICBD 2014, followed in a Pediatric Rheumatoloy Unit in at a tertiary hospital and also from 43 adult patients. It was assessed whether the juvenile patients met the new criteria for provisional classification of childhood EB 2016, analyzing and comparing the spectrum of clinical manifestation of the 2. The mean age at diagnosis was 11.4 ± 5.1 years; 8 of them being women and 1 male. Five patients (5/8) fulfilled the criteria of the ISGB-1990 and eight (8/8) had a score greater than or equal to 4 points that defined the BD according to the criteria of the ITRICBD - 2014. Only 3 of our patients (3/8) fulfilled the new criteria for pediatric BD of the PEDBD-2016. In the group of patients who fullfiell the new criteria: 100% had oral aphthae and genital ulcers as well as joint involvement and skin lesions (3/8). Ocular involvement was detected in two patients and neurological manifestations was seen in two patients; being 2 of them HLA-B51 (+). The 5 patients (5/8) who did not meet these criteria were those who presented mild clinical manifestations as oral ulcers, genitals and/or positive paternity test, with only one of them ocular involvement. One of the patients had incomplete Behcet follow-up

**Conclusion:** In our series, patients classified as pediatric BD by the 2016 criteria presented cutaneous, ocular and neurological manifestations. A greater number of patients met the previous classification criteria, since it was enough to have mucocutaneous manifestations. Additional studies are required to improve the diagnostic performance and characterization of patients with childhood BD

**Disclosure of Interest**

None Declared

### P159 EMAPALUMAB, AN ANTI-INTERFERON GAMMA MONOCLONAL ANTIBODY IN TWO PATIENTS WITH NLRC4-RELATED DISEASE AND SEVERE HEMOPHAGOCYTIC LYMPHOHISTIOCYTOSIS (HLH)

#### Claudia Bracaglia^1^, Giusi Prencipe^1^, Antonella Insalaco^1^, Ivan Caiello^1^, Giulia Marucci^1^, Raffaele Pecoraro^2^, Manuela Pardeo^1^, Pavla Dolezalova^3^, Sarka Fingerhutova^3^, Maria Ballabio^4^, Cristina de Min^4^, Fabrizio De Benedetti^1^

##### ^1^Division of Rheumatology, IRCCS Ospedale Pediatrico Bambino Gesù; ^2^Pediatric Department, La Sapienza University of Rome, Rome, Italy; ^3^Paediatric Rheumatology and Autoinflammatory Diseases Unit, General University Hospital, Prague, Czech Republic; ^4^Novimmune, S.A., Geneva, Switzerland

###### **Correspondence:** Claudia Bracaglia

**Introduction:** Interferon gamma (IFNγ) plays a pathogenic role in primary and secondary HLH. An ongoing phase 2/3 trial with emapalumab in primary HLH provides encouraging preliminary data and a pilot trial in MAS in the context of sJIA has just been initiated. Gain-of-function mutations in *NLRC4* are associated with a distinct autoinflammatory syndrome, with recurrent HLH.

**Objectives:** To report safety and efficacy of emapalumab treatment in two patients carrying *de novo* missense mutations in *NLRC4*, with severe early onset HLH.

**Methods:** Cytokine levels were measured by multiplex assay and by specific ELISAs and expression of IFNγ in freshly isolated PBMCs by cytometry.

**Results:** Pt 1. Caucasian male, presented, at age 20 days, fever and rash and progressively developed clinical and laboratory features of HLH leading to multi-organ failure. A *de novo* missense mutation in *NLRC4* (T337N) was found. High-dose glucocorticoids and cyclosporine-A (CyA) led only to partial improvement. A sepsis triggered HLH reactivation. Emapalumab was started (compassionate use) on background of dexamethasone (13.6 mg/m2) and CyA. After 3 months, the child was discharged in excellent conditions (prednisone 0.3 mg/kg). Infections resolved during treatment with emapalumab. After 7 months of emapalumab treatment, all therapies, including emapalumab, were discontinued, without clinical or laboratory signs of HLH reactivation.

Pt 2. This is 16 months old Caucasian boy with recurrent HLH and vasculitic skin lesions, since 1 month of life, secondary to a *de novo* missense mutation in *NLRC4* (I343N). His disease was not controlled despite treatment with repeated methylprednisolone pulses and chronic daily  glucocorticoid therapy, CyA (5 mg/kg) and anakinra (ranging from 5 to 25 mg/kg/day). When anakinra was withdrawn prior to start emapalumab he immediately developed high-grade fever, skin rash with vasculitic lesions and diarrhoea with laboratory features of HLH. Emapalumab was started (compassionate use) on background of methylprednisolone and CyA with rapid resolution of fever and improvement in biochemical parameters. During emapalumab treatment the patient resolved his initial HLH flare and presented two HLH episodes of mild intensity controlled with moderate intensification of glucocorticoid therapy. These episodes were triggered by systemic infections caused by pathogens translocated from the gut. His diarrhoea persisted with low grade inflammation; emapalumab was eventually withdrawn after 3 months. His subsequent course was characterized by additional mild episodes of MAS. In both patients increased production of IFNγ was demonstrated by high levels of CXCL9 (pt.1: 5670 pg/ml, pt.2: 3310 pg/ml), a chemokine induced specifically by IFNγ, by increased IFNγ expression in NK cells and CD8T cells, and by presence of high levels of total IFNγ bound to circulating emapalumab.

**Conclusion:** In both patients with HLH secondary to *NLRC4*-related disease, treatment with emapalumab was well tolerated, no safety concerned emerged, normalization of all HLH clinical and laboratory abnormalities was achieved. Pt. 1 showed no disease reactivation even in the absence of treatments In pt. 2 IFNγ neutralization has provided control of HLH, while his underlying disease and, in particular, gut inflammation and gut colonization by MDR pathogens remained unchanged. Informed consent to publish had been obtained from the parents.

**Disclosure of Interest**

C. Bracaglia: None Declared, G. Prencipe: None Declared, A. Insalaco: None Declared, I. Caiello: None Declared, G. Marucci: None Declared, R. Pecoraro: None Declared, M. Pardeo: None Declared, P. Dolezalova: None Declared, S. Fingerhutova: None Declared, M. Ballabio: None Declared, C. de Min Employee of: Novimmune, F. De Benedetti Grant / Research Support from: Novartis, Novimmune, Hoffmann- La Roche, SOBI, AbbVie, Pfizer

### P160 A CASE REPORT OF NLRP1-ASSOCIATED AUTOINFLAMMATION WITH ARTHRITIS AND DYSKERATOSIS (NAIAD SYNDROME) IN AN 8-YEAR-OLD RUSSIAN GIRL.

#### Vasily I. Burlakov^1^, Anna Kozlova^1^, Ekaterina Viktorova^1^, Julia Rodina^1^, Elena Deripapa^1^, Dmitry Abramov^2^, Anna Shcherbina^1^

##### ^1^Immunology; ^2^Pathomorphology, FEDERAL RESEARCH AND CLINICAL CENTER OF PEDIATRIC HEMATOLOGY, ONCOLOGY AND IMMUNOLOGY, MOSCOW, RUSSIA, Moscow, Russian Federation

###### **Correspondence:** Vasily I. Burlakov

**Introduction:** NLRP1-associated autoinflammation with arthritis and dyskeratosis (NAIAD syndrome) is a monogenic autoinflammatory syndrome caused by mutations in NLRP1 gene. It was first reported in 2016 and described as a complex syndrome of inflammasome hyperactivation with release of pro-inflammatory cytokines, dyskeratotic and papilloma-like skin lesions, arthritis and features of antibody-mediated autoimmunity with impairment of B-lymphocyte maturation.

**Objectives:** To report a case history of an 8-year-old Russian girl with some clinical features of NAIAD syndrome who was referred to our Center.

**Methods:** genetic defect was confirmed via exome next generation sequencing; lymphocyte immunophenotyping was done according to the standard flow cytometry protocols.

**Results:** We identified a heterozygous mutation in NLRP1 c.160 G>A, p.Ala54Thr in the patient, presenting with severe anemia (hemolytic and chronic inflammation anemia), thrombocytopenia, hyperkeratotic skin rash, dyskeratotic nail changes, additional auricular appendages, alopecia, arthritis and short episodes of alveolitis. The clinical feature that had not been previously described in NAIAD patients was corneal opacity with severe vision impairment. Until 8 years of age the patient suffered mostly from skin and eye sympthoms and cytopenia, that was treated by regular blood transfusions but otherwise had stable clinical condition. While in the process of clinical evaluation in our Center the patient deteriorated rapidly and developed a life-threatening condition with respiratory and acute kidney failure and secondary HLH, requiring artificial lung ventilation. Features of respiratory distress was found on CT-scans. The blood tests showed highly elevated inflammatory serum markers (CRP up to 220 mg/l), hypergammaglobulinemia. Peripheral blood B-cells predominantly consisted of transitory forms with decrease of the post-switch B-cells. Urine tests showed proteinuria and hematuria. After the evaluation of potential clinical significance of the discovered mutation, the patient was started on IL-1R inhibitor (anakinra) and rituximab (N=4) with dramatic improvement in a matter of days. We further enhanced her therapy with ruxolitinib addition, hoping to curb IL-18 hyperproduction. Currently the patient is at home and well, on ruxolitinib and anakinra therapy.

**Conclusion:** Since its first description in 2016 by S. Grandemange et al. there are only 3 patients reported worldwide so far. We conducted a literature search and found that NLRP1 c.160 G>A, p.Ala54Thr mutation was reported in a patient diagnosed with another NLRP1-associated entity – Multiple Self-Healing Palmoplantar Cracinoma (MSPC). Except for skin and corneal lesions the disorder is characterized by the elevated serum IL-1a, IL-1b and IL-18 levels (C.-H. Yu et al, 2017). One more disease associated with mutations in NLRP1 is Corneal Intraepithelial Dyskeratosis (CID) (V.Soler et al, 2013). Informed consent to publish had been obtained from the parent.

**Disclosure of Interest**

None Declared

### P161 ADA2 DEFICIENCY WITHOUT ADA2 MUTATIONS EXPLAINED BY A STRUCTURAL HOMOZYGOUS VARIATION IN 22Q11.1

#### Roberta Caorsi^1^, Alice Grossi^2^, Roberto Cusano^3^, Marta Rusmini^2^, Federica Penco^1^, Francesca Schena^1^, Rosa Anna Podda^4^, Paolo Uva^3^, Marco Gattorno^1^, Isabella Ceccherini^2^

##### ^1^Clinica pediatrica e reumatologia; ^2^UOC Genetica Medica and UOSD Genetica e Genomica delle Malattie Rare, GASLINI INSTITUTE, GENOA, ITALY, Genova; ^3^Centre for Advanced Studies, Research and Development in Sardinia (CRS4), Science and Technology Park Polaris, Pula; ^4^Clinica Pediatrica, Talassemie e Malattie Rare, Ospedale Brotzu e Università degli studi di Cagliari, Cagliari, Italy

###### **Correspondence:** Roberta Caorsi

**Introduction:** ADA2 gene (previously named CECR1 gene), located on chromosome 22q11.1, encodes for adenosine deaminase 2, an enzymatic protein also involved in the homeostasis of endothelial and hematopoietic cells. Loss of function mutations in ADA2 gene are responsible of a rare autosomal recessive condition, named DADA2, characterized by a broad clinical spectrum ranging from a systemic inflammatory disease with vascular and multiorgan involvement, resembling Panarteritis Nodosa, to clinical conditions associated to a variable range of immunodeficiency and immune-deregulation. A certain percentage of patients, despite a consistent phenotype and lack of the ADA2 enzymatic activity, have an incomplete or negative genotype.

**Objectives:** to define the genetics underlying a patient with a clinical phenotype consistent with DADA2 and a deficient enzymatic activity but without any mutation in the coding region of the CECR1 gene.

**Methods:** Whole exome sequencing (WES) was performed in a 9 year old patient with a clinical phenotype consistent with DADA2 (fever, livedo reticularis, hypertension, hypogammaglobulinemia and two episodes of ischemic stroke) and a complete lack of ADA2 activity in circulating monocytes. In particular, we analyzed the proband and the asymptomatic parents by using Illumina’s Nextera Rapid Capture Expanded Exome libraries and Illumina Hiseq2000 Instrument with 75bp paired-end reads. Since no coding mutation was identified as potentially candidate to account for the clinical phenotype, whole Genome Sequencing (WGS) was performed in the proband by TruSeq Nano DNA Library Prep kit and HiSeq 3000 Instrument with 150bp paired-end reads. Structural variants were identified with Manta.

**Results:** a homozygous tandem duplication of the genomic region encompassing exons 3 and 4 (involving 12895 bases), generated by two breakpoints in intron 2 and intron 4 respectively, was assessed by the WGS data. This has led to a gene transcript postulated to contain 1967 instead of 1536 nucleotides, due to an in tandem duplication of the sequences corresponding to exons 3 and 4, thus leading to a frameshift starting from codon V252 followed by a premature stop codon after 11 aminoacid residues (p.V252Gfs11*).

**Conclusion:** The structural variation identified in the patient represents the first evidence of a genetic defect, different from the presence of bi-allelic loss-of-function point mutations, involving the ADA2 gene. A timely identification of ADA2 enzymatic impairment in the presence of a phenotype clearly consistent with DADA2 should lead to a careful and extensive molecular approach in all patients presenting a monoallelic variant or even in the absence of any mutation at standard DNA sequencing.

**Disclosure of Interest**

None Declared

### P162 WHEN A RECURRENT FEVER DOES NOT ONLY DEPEND ON A RHEUMATIC CARDITIS

#### Martina Capponi^1^, Emanuela Del Giudice^1^, Annalisa Di Coste^1^, Metello Iacobini^1^, Flavia Ventriglia^1^, Chiara Passarelli^2^, Fabrizio De Benedetti^3^, Marzia Duse^1^

##### ^1^Department of Pediatrics, Sapienza University of Rome; ^2^Laboratory of Medical Genetics; ^3^Division of Rheumatology, IRCCS Ospedale Pediatrico Bambino Gesù, Rome, Italy

###### **Correspondence:** Martina Capponi

**Introduction:** Autoinflammatory diseases are a group of immunological disorders characterized by ‘seemingly unprovoked’ episodes of fever and inflammation without evidence of autoantibodies or antigen-specific T cells, implying the importance of dysregulation in the innate immune system.

**Objectives:** To report a case of severe rheumatic carditis that, in a significant host predisposition, could trigger an underlying autoinflammatory syndrome (associated with a mutation in MVK gene)

**Methods:** M, a caucasian previously healthy 11-years old boy, diagnosed with a rheumatic fever with carditis at the age of 10 (years old) based on the following criteria: fever, polyarthralgia, erythrocyte sedimentation rate > 60 mm/h, C-reactive protein (RCP) > 3.0 mg/dl, increased anti-streptolysin O titer (1700), prolonged PR interval on ECG and echocardiography/Doppler evidence of mitral and aortic valve regurgitation. He was treated with steroid therapy and secondary prophylaxis with Benzathine penicillin (every 21 days). After two months of the steroid withdrawal, he presented recurrent fever attacks (every 2-3 weeks). Each attack was heralded by chills, followed by a sharp rise in body temperature and lasted 4-5 days with gradual defervescence. Abdominal pain with vomiting or diarrhea and increased acute phase markers almost always accompanied the fever attack. Laboratory exams such as immunologic tests for autoimmunity as well as serology for CMV, parvovirus B19, EBV, adenovirus, coxsackie virus, and antistreptolysin O titer resulted negative or within normal limits. Common causes of infections were ruled out. Intravenous antibiotics were administered at any episode of fever and induced a partial remission of the disease activity and inflammatory markers, but his heart disease dramatically worsened such he needed to undergo to cardiac surgery (mitral valve repair and aortic valve replacement using mechanical prosthesis). Three days after the surgery, because of hyperpyrexia despite antibiotic therapy, he started corticosteroid therapy again achieving a partial remission in the following three months. When off steroid therapy, he developed four fever attacks lasting 4-7 days in 3-5 weekly intervals accompanied by abdominal pain and recurrent serositis (e.g., pleuritis, pericarditis) with acute phase markers increased and infectious disease tests, immunological and autoimmune exams always negative. SAA (serum amyloid A) levels were reported to be high in the inter-episode periods while acute phase markers were negative.

**Results:** Because of the recurrence of pericarditis and steroid dependence, we decided to start the treatment with daily subcutaneous injections of IL-1 receptor antagonist IL-1Ra (Anakinra) at a dosage of 1 mg/kg.

He performed the extended genetic analysis for autoinflammatory syndromes that detected the presence of the genomic variant C.1129G > A in heterozygosity in the MVK gene (mevalonate kinase) due to maternal segregation. After a follow-up of 2 months, we noticed a decreased duration and severity of flare on therapy with Anakinra.

**Conclusion:** Although the identified variant has been described as associated with MVK deficiency (MKD) if present in homozygosity or compound heterozygosity, some patients with classical disease and only a single identified mutation have reported too (Ref. Barron KS Arthritis Rheum. 2013). In our case, because of maternal segregation, we must suppose whether a hypomorfic expression of gene in the mother or the additional influence of other unknown genes as modifiers of the residual mevalonate kinaseactivity. Otherwise the SBEGA infection might have unmasked the underlied MKV. Informed consent to publish had been obtained.

**Disclosure of Interest**

None Declared

### P163 NEONATAL-ONSET MULTISYSTEM INFLAMMATORY DISEASE (NOMID) DUE TO A NEW SEQUENCE VARIANT C.1568T>A OF NLRP3 GENE IN AN INFANT OF ASIAN ANCESTRY

#### Grace Chiang^1^, Catherine T. Chung^2^, Miriam Weinstein^3^, Ronald M. Laxer^1,4^

##### ^1^Division of Rheumatology; ^2^Division of Pathology; ^3^Division of Dermatology, The Hospital for Sick Children; ^4^Department of Paediatrics and Medicine, University of Toronto, Toronto, Canada

###### **Correspondence:** Grace Chiang

**Introduction:** Neonatal onset multisystem inflammatory disease (NOMID) is a rare autoinflammatory disease with onset in infancy. Together with familial cold autoinflammatory syndrome (FCAS) and Muckle-Wells syndrome (MWS), they form the spectrum of cryopyrin-associated periodic syndrome (CAPS) with NOMID at the most severe end of the spectrum. CAPS is caused by single heterozygous germline or somatic gain of function mutations in the *NLRP3* gene encoding the protein cryopyrin. To date, 209 different sequence variants of the *NLRP3* gene and more than 90 heterozygous mutations are identified in patients with CAPS, and the list is expanding [1]. The case report here demonstrates a new sequence variant of *NLRP3* gene in a patient with NOMID phenotype of Asian ancestry.

**Objectives:** To report the finding of a new sequence variant c.1568T>A of *NLRP3* gene in an Asian infant with NOMID

**Methods:** Case report

**Results:** A 10-month old girl developed a generalized skin rash within hours of birth. The rash was described as “migratory” and “waxes and wanes” every day. A skin biopsy at 7 months of age showed periadnexal and perivascular neutrophilic infiltrates. At 8 months of age she developed a prolonged fever of 2 weeks. Physical examination showed frontal bossing, a mild delay of gross motion function and mild central hypontonia. A generalised macular rash was found and some of them were of urticarial morphology.  There was persistent elevation of inflammatory markers with highest C-reactive protein (CRP) of 180mg/L (0.1-1.0mg/L), erythrocyte sedimentation rate (ESR) 77mm/h (2-34mm/h), and serum amyloid A greater than 16,000 ng/ml (1000-5000ng/ml). MRI brain showed macrocephaly with prominent supratentorial ventricular and extra-axial cerebral spinal fluid (CSF) spaces with bilateral papilloedema. Lumbar puncture (LP) showed elevation of white cell count (WCC) 38x10^6/L and protein 0.41g/L (0.15-0.4g/L) in CSF. There was no skeletal deformity found on radiographic bone survey. Next generation sequence genetic testing revealed a rare sequence variant of the *NLRP3* gene (c.1568 T>A). Genetic testing of the parents were negative for this variant. Thus, it appears to have arisen de novo and is likely pathogenic. Anakinra (4mg/kg/day) was started and the fever and rash resolved within a day.  A repeated LP after one year of treatment showed normalization of WCC and protein in CSF. The follow up MRI brain also showed interval improvement of supratentorial extra-axial CSF spaces and resolution of bilateral papilledema. Her development is appropriate to age.

**Conclusion:** NOMID is a severe disease with onset within hours of life. Different systems can be affected and permanent organ damage can occur if treatment is delayed. However, not every feature presents in early infancy and they may appear later in the disease course. The skin biopsy finding of neutrophil infiltrates in our case provides a crucial clue of an autoinflammatory condition. CNS involvement is a unique feature of NOMID in the CAPS spectrum. Treatment with IL-1 blocking agents results in significant clinical and laboratory improvements in NOMID and has become the standard of care [2,3]. The IL-1 receptor antagonist anakinra can penetrate into the CNS and treatment improves CNS inflammation. The list of sequence variants of NOMID or other diseases in the CAPS spectrum is expanding, with emerging new variants among Asian populations. This case report documents a pathogenic sequence variant recently discovered in a newly diagnosed NOMID patient of Asian background. Written onsent for publication was obtained from the parent.

**Disclosure of Interest**

None Declared

### P164 EFFECTIVENESS AND SAFETY OF TREATMENT WITH INTRAVENOUS PAMIDRONATE IN PEDIATRIC PATIENTS WITH BONE DISEASES

#### Mireia L. Corbeto, Consuelo Modesto, Estefanía Moreno

##### Pediatric Rheumatology, Hospital Vall d'Hebron, Barcelona, Spain

###### **Correspondence:** Mireia L. Corbeto

**Introduction:** The use of bisphosphonates has increased in recent decades in patients of pediatric with osteoporosis (OP) and bone pain. The effectiveness and safety data of Pamidronate (PA) in OP secondary schools are limited.

**Objectives:** To describe the effectiveness and safety of PA in pediatric patients with bone diseases.

**Methods:** A retrospective study of patients below 18 years of age who had received PA in a third level hospital between 2012 and 2017 was performed. The demographic, analytical variables and densitometry data have been collected by DXA (L1-L4) prior to PA infusion and 2 years after its onset, the existence of pathological fractures and the adverse events of the treatment. Descriptive and analytical statistics have been used through the IMB SPSS Statistics20 program.

**Results:** Twenty-eight patients (46.4% males) with an average age of 12.6 years received an average total dose of 32 mg (0.8 mg/kg/infusion) of PA. The indication was in 53.6% of the cases OP secondary to corticosteroids, in 39.3% due to bone pain and in 7.1% OP secondary to immobility. In 39.3% of the patients a previous fracture was detected, decreasing to a 32% at 2 years after the treatment has begun. 78.6% of the patients reported an improvement on the pain. Only 2 mild adverse events were reported. A statistically significant increase in BMD was observed in gr/cm² after two years of treatment (0.631 to 0.729, p = 0.001).

**Conclusion:** Treatment with PA increases bone mass 2 years after its onset and provides an improvement on bone pain. Adverse effects were very infrequent.

**Disclosure of Interest**

None Declared

### P165 INTERFERON SIGNATURE IN CHRONIC NON-BACTERIAL OSTEOMYELITIS: PRELIMINARY DATA FROM PROSEPCTIVE SERIES

#### Luisa Cortellazzo Wiel^1^, Anna M. C. Galimberti^1^, Francesco Baldo^1^, Giulia Gortani^2^, Serena Pastore^2^, Alessandra Tesser^3^, Andrea Taddio^1,2^, Alberto Tommasini^3^

##### ^1^Paediatrics, University of Trieste; ^2^Paediatrics; ^3^Clinical Immunology, IRCCS Burlo Garofolo, Trieste, Trieste, Italy

###### **Correspondence:** Luisa Cortellazzo Wiel

**Introduction:** Chronic non-bacterial osteomyelitis (CNO) is an inflammatory disorder of uncertain pathogenesis. On one hand, the occurrence of inflammatory osteomyelitis with similar characteristics in Majeed syndrome and DIRA syndrome supports the classification of CNO among autoinflammatory diseases. On the other hand, the existence of subgroups of CNO with associated autoimmune disorders, such as arthritis and psoriasis, suggest that more complex pathogenic mechanisms may be implied in the disease.

**Objectives:** Since autoinflammatory and autoimmune manifestations are common in interferonopathies, we hypothesized that interferon mediated inflammation could be involved as well in CNO.

**Methods:** To determine the role of interferon in the pathogenesis of the disease we started to quantify the interferon signature (IS) in our CNO patients.

All patients with CNO currently followed at our clinics were enrolled in the study. Blood specimens were withdrawn after obtaining a signed informed consent and collected in RNA preserving tubes. Interferon signature was assessed by analyzing the six genes (IFI27, IFI44L, IFIT1, ISG15, RSAD2, SIGLEC1) and the interferon score was calculated as described by Crow et al (J Clin Immunol, 2017). We considered as positive values above 10 (as almost all SLE display IS>10), and negative score below 5 (as almost all JIA in our experience have IS<5). Values between 5 and 10 were considered border-line.

**Results:** Five patients were enrolled (3 females, 2 males, mean age 16.6 years, range 13.6-20.6). Positive/border-line interferon score was recorded in four out of five patients (Table 1). There was no apparent correlation with disease activity and ESR.

**Conclusion:** Apart from a single patient with concomitant hematologic cytopenia, we detected only mild or border-line elevation of IS scores. It is uncertain whether these low scores can reflect or not an involvement of type 1 interferon in the inflammatory pathogenesis of CNO. However, it is worth noting that these results could be influenced by low disease activity in most of the investigated patients.

Further investigations are needed to evaluate the real role of interferon 1 in the pathogenesis of CNO and the potential utility of this assay in stratification of patients for therapeutic choices.

**Trial registration identifying number:** This work was funded by IRCCS Burlo Garofolo grant RC24/17 and Telethon foundation grant GGP15241

**Disclosure of Interest**

None Declared

**Table 1 (abstract P165).** See text for description.

Pt n.	Age (years)	Sex	IFN score	Disease activity	ESR	Bone localization	Other clinical features
1	20.6	M	18.2	Clinical Remission	15	Clavicula	Low neutrophils, lymphocytes and platelets count. Previous spondyloarthritis.
2	13.6	M	10.9	Clinical remission	24	Multifocal	---
3	17.0	F	9.3	Moderate	13	Multifocal	Ankle arthritis
4	18.2	F	5.7	Moderate	5	Multifocal	Psoriasis
5	13.8	F	0.7	Low	26	Multifocal	---

### P166 AUTOINFLAMMATORY DISEASES: IDENTIFIED TWO VARIANTS OF THE NLRC4 GENE

#### Emanuela Del Giudice^1^, Annalisa Di Coste^1^, Martina Capponi^1^, Eleonora Romeo^1^, Ilaria Battagliere^1^, Chiara Passarelli^2^, Fabrizio De Benedetti^3^, Marzia Duse^1^

##### ^1^Department of Pediatrics, Sapienza University of Rome; ^2^Laboratory of Medical Genetics, IRCCS Ospedale Pediatrico Bambino Gesù; ^3^Division of Rheumatology, IRCCS Ospedale Pediatrico Bambino Gesù Rome, Rome, Italy

###### **Correspondence:** Emanuela Del Giudice

**Introduction:** Autoinflammatory diseases (AIDs) are a group of heterogeneous disorders characterized by recurrent fever and systemic inflammation (aseptic and non-autoimmune) , and the NLRC4- associated autoinflammatory diseases have been only recently described.

**Objectives:** To describe two pediatric cases with two variants of NLRC4 gene with a different spectrum of clinical manifestations

**Methods:** We describe two pediatric patients presented with recurrent fever and nonspecific symptoms.

Patient 1 F., caucasian, 8 years old boy presented with recurrent fever (maximum of 39°C), lasting 3-10 days with intervals of 3 days in apyrexia, not always responsive to oral corticosteroids, associated with rhinitis, cough, not-itchy rash on trunk and face, sometimes periorbital edema and conjunctivitis. In the last two years he developed arthralgia involving the small joints of the hands and bilateral knee pain, in some cases associated with joint swelling. He reported headache, vomiting and recurrent abdominal pain, often diarrhea without blood or mucus. Laboratory tests revealed a persistent increase of inflammatory markers and serum amyloid A (SAA) . The abdominal ultrasound showed a thickening of ileum wall tract of about 30 mm with an intestinal distension and fluid-corpusculated material upstream, without hepatosplenomegaly and the fecal calprotectin was negative. Screening for infectious diseases and malignancies was negative.

Patient 2 A., caucasian, 17 years old girl. At 11 years she was hospitalized due to persistent fever associated with pharyngodynia, rash located on the upper eyelids, edema of the face and disabling joint pain and she was diagnosed a macrophage activation syndrome (MAS). At the age of 15 years she had the second episode of MAS, likely to be triggered by an EBV infection. At 16, the appearance of polyarthritis evolved in MAS in the absence of febrile episodes / previous infectious and a diagnosis of juvenile idiopathic arthritis in a patient with recurrent MAS was made.

**Results:** In the patient 1 the genetic molecular analysis revealed the presence of a heterozygosis mutation of the genomic variant c.2785G> T of NLRC4 gene. This child is currently on therapy with colchicine with clinical and laboratory remission. Otherwise the patient 2 is currently on therapy with methotrexate and anti-TNF Etanercept with good clinical response. The extended analysis for autoinflammatory syndromes has detected the presence of the genomic variant c.2357G> T in heterozygosis of the NLRC4 gene. The related family genetic studies are going in both cases.

**Conclusion:** Mutations in heterozygosis of the NLRC4 gene, although defined as not-causative, appear to be associated with various clinical pictures in the context of auto-inflammatory syndromes, as well as a high susceptibility to developing MAS. In first our cases it could be associated to the autoinflammatory syndrome with enterocolitis (SCAN4), also called as autoinflammation with infantile enterocolitis (AIFEC), while in the second one, according to the reported literature, corresponds to a variant with uncertain meaning. In these cases, genetics can help us in the management of infective episodes, which must be treated promptly and appropriately to prevent the occurrence of complications and focus attention at follow up on onset of gastrointestinal symptoms. Informed consent to publish had been obtained.

**Disclosure of Interest**

None Declared

### P167 EVALUATION OF PLASMA MICRORNA EXPRESSIONS IN PATIENTS WITH FAMILIAL MEDITERRANEAN FEVER

#### Ferhat Demir^1^, Alper H. Çebi^2^, Mukaddes Kalyoncu^1^

##### ^1^Department of Pediatric Rheumatology; ^2^Department of Medical Genetics, Karadeniz Technical University Faculty of Medicine, Trabzon, Turkey

###### **Correspondence:** Ferhat Demir

**Introduction:** Familial Mediterranean fever (FMF) is one of the most common cause of autosomal recessive inherited autoinflammatory disease in childhood. The MEFV mutations cause the impaired function of a protein called pyrine by incorrect coding and that process results with uncontrolled inflammation. Microribonucleic acids (miRNAs) are small (16–24 nucleotides), non-coding RNA molecules that have roles on the regulation of gene expression at the post-transcriptional stage. The plasma expressions of miRNAs are altered in various autoimmune and autoinflammatory diseases. Therefore, miRNAs may have a role in the pathogenesis of autoinflammation and may be used in diagnosis and follow-up of these diseases.

**Objectives:** We aimed to evaluate plasma expression of some candidate miRNAs that associated with pathogenesis of autoimmunity and autoinflammation in patients with FMF.

**Methods:** Thirty patients diagnosed with FMF and age-sex matched 30 healthy children were enrolled for the study. Plasma levels of four candidate miRNA (miRNA-16, miRNA-155, miRNA-204 and miRNA-451), which is known to be related to autoimmunity and inflammation, were examined in all subjects. Plasma levels of miRNAs were measured with Real Time PCR in attack and remission period of patients and healthy controls. Groups were compared with each other.

**Results:** Plasma miRNA-204 levels were decreased 6.5-fold in remission period of FMF patients, compared to the healthy controls (*p <0.001*). This decrease was more prominent in M694V mutation carriers. It was also found that plasma miRNA-155 levels were lower in remission period of FMF patients (*p <0.03*).

**Conclusion:** Our data suggest that, miRNA-155 and miRNA-204 may play a role in the pathogenesis of FMF. Further investigations about the role of miRNAs in FMF may help to elucidate the pathogenesis of the disease.

**Disclosure of Interest**

None Declared

**Table 1 (abstract P167).** The mean plasma miRNA 2-^ΔCT^ level of patients in aFMF, rFMF and HC groups and comparison of it

	Mean 2^-ΔCT^			Fold change/*p value*		
	aFMF	rFMF	HC	aFMF-rFMF	aFMF-HC	rFMF-HC
miRNA-16	0.97	1.05	0.90	-1.09/*0.40*	1.08/*0.96*	1.17/*0.52*
miRNA-155	0.55	0.44	0.69	-1.25/*0.15*	-1.25/*0.18*	-1.56/***0.03***
miRNA-204	0.019	0.002	0.017	9.50/***0.001***	1.10/*0.34*	**-8.50/** ***0.001***
miRNA-451	1.85	2.10	1.59	-1.13/*0.50*	1.16/*0.67*	1.32/*0.85*

aFMF: attack period of Familial Mediterranean fever, rFMF: remission period of Familial Mediterranean fever, HC:Healthy control

### P168 NEW CLASSIFICATION CRITERIA FOR RECURRENT AUTOINFLAMMATORY DISEASES APPLIED TO AN INDEPENDENT COHORT: EXPERIENCE FROM THE JIR COHORT DATABASE.

#### Glory Dingulu^1^, Isabelle Kone-Paut^2^, Sophie Georgin-Lavialle^3^, Pascal Pillet^4^, Anne Pagnier^5^, Etienne Merlin^6^, Daniela Kaiser^7^, Alexandre Belot^8^, Michael Hofer^9^, Veronique Hentgen^10^

##### ^1^Pediatrics, CENTRE HOSPITALIER VERSAILLES, Le Chesnay; ^2^Pediatric Rheumatology, CHU Bicêtre, Kremlin-Bicêtre; ^3^Internal Medicine, CHU Tenon, Paris; ^4^Pediatrics, CHU Bordeaux, Bordeaux; ^5^Pediatrics, CHU Grenoble, Grenoble; ^6^Pediatrics, CHU Clermont Ferrand, Clermont Ferrand, France; ^7^Pediatrics, CHU Luzern, Luzern, Switzerland; ^8^Pediatric Rheumatology, CHU Lyon, Lyon; ^9^Pediatric Rheumatology, CHU Vaudois, Lausanne; ^10^Pediatrics, Centre Hospitalier de Versailles, Le Chesnay, France

###### **Correspondence:** Glory Dingulu

**Introduction:** New classification criteria for the inherited periodic fever syndromes (TRAPS, FMF, MKD and CAPS) have recently been developed during a Consensus Conference held in Genoa in March 2017.

**Objectives:** The aim of our study was to compare these new classification criteria for monogenic recurrent fever syndromes with the diagnoses of clinicians in a real-life setting. For this purpose we used the JIRcohort database, an international platform gathering data of patients with pediatric inflammatory disease.

**Methods:** All the patients included to the JIRcohorte database with a recurrent fever syndrome were enrolled to the study. Criteria were applied to all the patients and then compared to the clinical diagnosis of the treating physician. An analytical study, describing concordance between clinician diagnosis and Genoa criteria classification was performed.

**Results:** Cryopyrin Associated Periodic Syndrom: 14 patients of the JIRcohorte matched Genoa criteria for CAPS. 8 had also been diagnosed CAPS by clinicians. The remaining 6 patients fulfilling the classification criteria for CAPS had various diagnoses made by the clinicians: SURF in 2 cases, PFAPA in 3 cases, FMF in 1 case. Clinicians considered CAPS in 19 further patients but none of them fulfilled the Genoa classification criteria for CAPS. None of these patients displayed a confirmatory genotype for CAPS.

Familial Mediterranean Fever: 153 patients matched Genoa criteria for FMF of whom 77 patients had also been diagnosed as FMF by the treating physician. The remaining 76 patients had various clinical diagnoses made by the treating physician: 14 SURF patients, 12 PFAPA patients, 12 TRAPS patients, 2 MKD patients and 2 CAPS patients. Interestingly 3 out of these 76 patients displayed genetic variants in another gene than *MEFV* while the others matched clinical Genoa criteria for FMF. The new classification criteria for FMF needs to be restricted to patients with genetic variants in *MEFV*. Clinicians diagnosed FMF in 23 further patients not classified as FMF according to the Genoa criteria for FMF: 6 with a heterozygous pathogenic *MEFV* mutation, 1 with a heterozygous VOUS, 3 with homozygous undetermined mutations, 12 without any genetic mutation.

Tumour Necrosis Factor Receptor Associated Periodic Syndrom: 18 patients matched Genoa criteria for TRAPS of whom all had been diagnosed TRAPS by the treating physician. All these patients had pathogenic or likely pathogenic mutations. Clinicians diagnosed TRAPS in 4 further patients, but none of them had a confirmatory genotype.

Mevalonate Kinase Deficiency: 217 patients matched Genoa criteria for MKD, of whom only 5 were diagnosed as having MKD by their treating physician. The remaining 212 patients fulfilling the Genoa criteria for MKD had various diagnoses: 119 PFAPA, 3 CAPS, 31 SURF, 5 TRAPS and 41 FMF with half matching Genoa criteria for FMF. 5 patients had been diagnosed MKD without matching Genoa criteria for MKD: 2 did not have genetical screening, 3 had heterozygous mutation with incomplete data.

**Conclusion:** This study is the first evaluation of the Genoa criteria in a real-life setting. The classification concordance with physician diagnosis is high for patients with confirmatory genotype and helps classifying patients with non confirmatory genotype. The classification concordance with physician diagnosis is low when patients did not display at least one gene mutation.

**Disclosure of Interest**

None Declared

### P169 AUTO INFLAMMATORY DISEASE WITH LIPOID PNEUMONIA, A NEW ENTITY? REPORT OF TWO CASES

#### Anita Duncan^1^, Camille Ohlmann^2^, Philippe Reix^2^, Sophie Collardeau-frachon^3^, Brigitte Bader-meunier^4^, Alexandre Belot^1^

##### ^1^Rheumatology and Nephrology unit; ^2^Pneumology unit, Hopital Femme Mère Enfant; ^3^Histopathology unit, East Hospital Group, Lyon; ^4^Immunology-Hematology and Rheumatology unit, Hôpital Necker Enfants Malades, Paris, France

###### **Correspondence:** Anita Duncan

**Introduction:** Lipoid pneumonia (LP) is a rare entity with two clinical forms. Endogenous LP, putting aside cases associated with an obstructive cause, has been reported to be associated with metabolic, infectious or systemic inflammatory diseases. Diagnosis may be challenging as clinical presentation is insidious and non-specific. Radiological findings are well described with an unusually low density on chest CT scan suggesting the presence of fat. Histopathology, when available, describes lipid laden alveolar macrophages.

**Objectives:** Lipoid pneumonia is scarcely described in pediatric rheumatology and treatment strategy remains unclear.

**Methods:** We report the cases of two patients sharing a similar presentation of auto-inflammatory disease of early onset with the association of arthritis, urticarial eruption and hyper eosinophilia. For both, progression was marked by lung involvement (lipoid pneumonia) with major clubbing in the absence of hypoxia.

**Results:** The auto inflammatory presentation resembles systemic Juvenile Idiopathic Arthritis (JIA) with a form particularly resistant to biotherapies and hypersensitivity reactions to drugs. Biologically, we did not detect any marker of auto immunity and genetic analysis with whole exome sequencing did not enable us to identify mutations in the genes associated with auto inflammatory diseases nor new variants in a common gene to the two patients. However, it is interesting to point out that under high doses of anti interleukine 1(IL1) therapy (10mg/kg), these patients maintain positive circulating levels of IL1 suggesting a central role of the inflammasome in the presentation of these patients. The early onset of clubbing associated with lipoid pneumonia and in the absence of hypoxia seems characteristic and syndromic of this entity.

**Conclusion:** The combination of systemic autoinflammation with lipoid pneumonia (AILP) illustrates a new clinical entity with IL1 hypersecretion.

**Disclosure of Interest**

None Declared

### P170 LESSON FROM THE EUROFEVER REGISTRY AFTER 10 YEARS OF ENROLLMENT

#### Martina Finetti, Silvia Federici, Joost Frenkel, Seza Ozen, Helen Lachmann, Luca Cantarini, Pavla Dolezalova, Fabrizio De Benedetti, Annette Jansson, Isabelle Koné-Paut, Paul Brogan, Gayane Amaryan, Jordi Anton Lopez, Joost Swart, Michael Hofer, Troels Herlin, Efimia Papadopoulou-Alataki, Romina Gallizzi, Marco Cattalini, Consuelo Modesto, Yonatan Butbul Aviel, Yosef Uziel, Alberto Martini, Nicola Ruperto, Marco Gattorno

##### Clinica Pediatrica e Reumatologia, Istituto Giannina Gaslini on behalf of Centers affiliated to Eurofever project and for the Paediatric Rheumatology International Trials Organisation (PRINTO), GENOA, Italy

###### **Correspondence:** Martina Finetti

**Introduction:** In 2008 the Paediatric Rheumatology European Society (PReS) promoted an International Project for the study of Autoinflammatory Diseases (AIDs) named Eurofever, whose main purpose is to create a web-based registry for the collection of information in AIDs patients.

**Objectives:** To implement the Registry with the new recently described AIDs and to increase the collection of longitudinal data.

**Methods:** The data analyzed in the study were extracted from the Eurofever registry, which is hosted in the PRINTO website (http://www. printo.it). From February 2015 we started the longitudinal collection of follow-up data with particular focus on treatment, modification of the clinical picture, onset of complication/adverse events.

**Results:** Up to date 4046 patients have been enrolled in the Registry from 62 different countries (3773 of them with complete baseline demographic information, represented in Table 1). Most of patients (2618; 69%) reside in western Europe, 489 (13%) in central-eastern Europe, 452 (12%) in southern-eastern Mediterranean countries (Turkey, Israel, North Africa), 127 in Asia, 76 in South America and 11 in Australia. Compared to the first Eurofever report (Toplak et al, 2012) we have observed an increase of enrollment in central-eastern Europe (from 6 to 13% of the total). 3444 (91%) patients have a pediatric onset of disease (< 16 years); 400 (12%) of them received diagnosis in adult age. 329 (9%) patients had an adult onset (81 FMF patients, 52 TRAPS patients, 40 CNO patients, 12 Schnitzler syndrome). The median onset age is 4 years (range 1 month – 75 years), the median diagnosis age is 8 years (range 1 month - 78 years). The median diagnosis delay is 2 years (range 0-76 years); the diseases with the longer diagnostic delay were NLRP-12 mediated FCAS2 (mean 25 years, range 5-71), CAPS (mean 14 years, range 0-65), PAPA (mean 13 years, range 0-50) and TRAPS (mean 12 years, range 0-76). In 3292 pts (81%) complete clinical information from disease onset to diagnosis are also available. At the moment of enrollment 3062 patients were in the pediatric age, 230 were adults. Longitudinal data on treatment are available for 448 pts (11%).

**Conclusion:** The enrolment in Eurofever Registry is still ongoing. The analysis of data will improve our knowledge both on the natural history of the single disease and on the efficacy/safety of treatment commonly used in the clinical practice.

**Disclosure of Interest**

M. Finetti: None Declared, S. Federici: None Declared, J. Frenkel: None Declared, S. Ozen: None Declared, H. Lachmann: None Declared, L. Cantarini: None Declared, P. Dolezalova: None Declared, F. De Benedetti: None Declared, A. Jansson: None Declared, I. Koné-Paut: None Declared, P. Brogan: None Declared, G. Amaryan: None Declared, J. Anton Lopez: None Declared, J. Swart: None Declared, M. Hofer: None Declared, T. Herlin: None Declared, E. Papadopoulou-Alataki: None Declared, R. Gallizzi: None Declared, M. Cattalini: None Declared, C. Modesto: None Declared, Y. Butbul Aviel: None Declared, Y. Uziel: None Declared, A. Martini: None Declared, N. Ruperto Grant / Research Support from: The G. Gaslini Hospital, which is the public Hospital where NR works as full time public employee, has received contributions from the following industries for the coordination activity of the PRINTO network: BMS, GlaxoSmithKline (GSK), Hoffman-La Roche, Novartis, Pfizer, Sanofi Aventis, Schwarz Biosciences, Abbott, Francesco Angelini S.P.A., Sobi, Merck Serono. This money has been reinvested for the research activities of the hospital in fully independent manners without any commitment with third parties., Speaker Bureau of: NR has received speaker’s bureaus and consulting fees from the following pharmaceutical companies: AbbVie, Amgen, Biogenidec, Alter, AstraZeneca, Baxalta Biosimilars, Biogenidec, Boehringer, BMS, Celgene, CrescendoBio, EMD Serono, Hoffman-La Roche, Italfarmaco, Janssen, MedImmune, Medac, Novartis, Novo Nordisk, Pfizer, Sanofi Aventis, Servier, Takeda, UCB Biosciences GmbH., M. Gattorno Grant / Research Support from: MG has received unrestricted grants from Sobi and Novartis., Speaker Bureau of: MG has received speaker bureaus from Sobi and Novartis.

**Table 1 (abstract P170).** Demographic features of enrolled patients

Disease	Pts number	M/F	Mean onset age (years, range)	Mean diagnosis age (years, range)	Western Europe	Eastern Europe	Eastern- Southern Mediterranean	Others Countries
Monogenic Disease								
FMF	1078	561/517	6 (0-66)	10 (0-72)	540	120	290	128
CAPS	296	145/151	5 (0-57)	19 (0-77)	239	27	13	17
TRAPS	271	138/133	9 (0-63)	21 (0-77)	238	25	4	4
MKD	204	92/112	2 (0-45)	12 (0-51)	175	17	5	7
Blau disease	49	25/24	3 (0-29)	8 (0-45)	24	1	3	21
PAPA	40	20/20	6 (0-18)	19 (2-57)	31	5	2	2
DADA2	13	10/3	4 (0-10)	12 (6-18)	10	-	3	-
NLRP-12	12	6/6	12(0-29)	37 (4-76)	11	-	-	1
SAVI	3	1/2	1 (0-4)	10 (1-17)	2	-	-	1
DIRA	3	0/3	1mth	2 (0-7)	2	-	1	-
Majeed	3	3/0	18 (0-55)	23 (1-68)	3	-	-	-
CANDLE	1	0/1	10	12	1	-	-	-
Multifactorial autoinflammatory disease								
PFAPA	664	376/288	2 (0-29)	5 (0-37)	333	224	94	13
CNO	564	210/354	11 (0-62)	12 (1-68)	513	16	24	11
Undefined fever	346	170/176	12 (0-73)	17 (0-75)	295	40	4	7
Behcet	214	108/106	9 (0-56)	12 (2-57)	189	14	9	2
Schnitzler	12	5/7	53 (36-75)	57 (41-78)	12	-	-	-
Total	3773	1870/1903	-	-	2618	489	452	214

### P171 MUTATIONS IN TRNT1 GENE ASSOCIATED WITH ENHANCED TYPE I IFN SIGNALLING

#### Marie-Louise Frémond^1,2^, Isabelle Melki^1,2,3^, Sven Kracker^4^, Vincent Bondet^5^, Darragh Duffy^5^, Gillian I. Rice^6^, Yanick J. Crow^1,7^, Brigitte Bader-Meunier^2,8^

##### ^1^Laboratory of Neurogenetics and Neuroinflammation, INSERM UMR 1163; ^2^Paediatric Haematology-Immunology and Rheumatology, Necker AP-HP; ^3^General Paediatrics, Robert Debré AP-HP; ^4^Laboratory of Human Lymphohematopoiesis, INSERM UMR 1163; ^5^Immunobiology of Dendritic Cells, Institut Pasteur, Paris, France; ^6^Division of Evolution and Genomic Sciences, Manchester Academic Health Science Centre, Manchester; ^7^Centre for Genomic and Experimental Medicine, Institute of Genetics and Molecular Medicine, , Edinburgh, UK; ^8^Laboratory of Immunogenetics of Paediatric Autoimmunity, INSERM UMR 1163, Paris, France

###### **Correspondence:** Marie-Louise Frémond

**Introduction:** Efficacy of TNF inhibitors in sideroblastic anaemia with immunodeficiency, fevers, and developmental delay (SIFD) has been recently reported^1^. The authors also demonstrated high levels of IL-6, IL-12p40, IL-18, and interferon (IFN)-γ in two patients.

**Objectives:** To describe activation of the type I IFN pathway in two patients with SIFD.

**Methods:** We assessed type I IFN status by IFN-α digital-ELISA quantification in serum, STAT1 phosphorylation and RNA expression of IFN-stimulated genes (ISGs) in whole blood.

**Results:** Patient 1 (P1) was a 12-month-old girl referred because of recurrent attacks of fever with high levels of CRP from the age of two months, with or without documented infections, chronic microcytic anaemia and failure to thrive. Aseptic febrile manifestations included vulvitis, parotiditis, adenitis, and neutrophilic panniculitis. She developed progressive lymphopenia, with undetectable levels of B cells by age 15 months, so that IVIG was initiated at this time. At last follow-up, aged six years, her height and development were normal and she was no longer subject to recurrent infections. However, she continued to experience three to four febrile attacks each month, associated with elevated CRP levels. Whole exome sequencing identified compound heterozygous mutations in the *TRNT1* gene (c.1213G>A, p.G405R / c.1057-7C>G)*.* Patient 2 (P2), a girl born at term to first-cousin parents presented with intrauterine growth retardation (IUGR) and severe neonatal anaemia necessitating blood transfusion on the first day of life. During infancy she required regular blood transfusions because of sideroblastic anaemia, which resolved spontaneously at the age of four years. She also demonstrated developmental delay and severe auto-inflammatory flares associated with fever and high levels of CRP, diarrhoea and dehydration. Mild T and NK lymphopenia together with a profound B cell defect were evident at four years of age. Sanger sequencing of *TRNT1* identified a homozygous mutation in exon 7 (c.977T>C, p.I326T). Using an ultra-sensitive digital ELISA combined with a high specificity pan-IFN-α antibody pair, we observed an increased concentration of IFN-α protein in the serum from P1 (246.44 fg/mL, healthy controls <10 fg/mL) and P2 (108.18 and 38.05 fg/mL, healthy controls < 10 fg/mL), comparable to the levels measured in the context of certain monogenic type I interferonopathies. We also recorded an increased expression of ISGs in the whole blood of P1 and P2 on 3 occasions. In an *ex vivo* flow cytometry assay, STAT1 and STAT3 were constitutively phosphorylated in T lymphocytes and monocytes from P2. Both patients also displayed a negative IFN score on one occasion, suggesting a fluctuating biological process.

**Conclusion:** These data indicate a constitutive activation of the type I IFN pathway in patients with biallelic mutations in *TRNT1*, and thus suggest a possible role for type I IFN in the pathogenesis of SIFD. An increase in serum IFN-α protein and an enhanced expression of ISGs have been previously reported in one patient^2^. How aberrant tRNA processing secondary to TRNT1 dysfunction might mediate enhanced type I IFN production and signalling remains to be determined. Informed consent to publish had been obtained from the parents of the two patients.

**References**

1. Giannelou A *et al*. *Ann Rheum Dis* 2018;77(4):612-619

2. Frans G *et al*. *J Allergy Clin Immunol* 2017 ;139(1):360-363

**Disclosure of Interest**

None Declared

### P172 NEW MUTATION OF TREX1 - A STEP TOWARD THE DEFINITIVE UNDERSTANDING OF AICARDI-GOUTIÈRES SYNDROME

#### Marijan Frkovic^1^, Sanda Huljev Frkovic^1^, Daniel Franjic^2^, Marija Jelusic^1^

##### ^1^Department of Pediatrics, University Hospital Centre Zagreb, Zagreb, Croatia; ^2^Department of Neurobiology, Kavli Institute for Neuroscience, New Haven, USA

###### **Correspondence:** Marijan Frkovic

**Introduction:** So far several mutations of three prime repair exonuclease 1 (*TREX1*) were identified as cause of Aicardi-Goutières syndrome (AGS). This rare autoimflammatory disease has been recently in focus of research due to its pathogenetic and phenotypic similarities with systemic lupus erythematosus (SLE).

**Objectives:** Autors present case of a child with AGS caused by newly discovered mutation *of TREX1* gene.

**Methods:** Retrospective analysis of AGS patient medical data including results of whole exome sequencing.

**Results:** A seven days old female newborn was referred to our department of Pediatrics due to diffuse petechiae, hypotonia with intermittent tremor and high-pitched crying. The child was born from the third, normal pregnancy, at term, BW 3520 g, BL 50 cm, Apgar scores 10/10. Family history is unremarkable.

Diagnostic evaluation revealed mild trombocytopenia combined with cerebrospinal fluid lymphocytosis. Results of serological tests and PCR for intrauterine and perintal infections were negative. MR brain scans showed simplified gray matter gyration, cerebelar hypoplasia and multiple periventricular and basal ganglia calcifications.

Due to suspicion of AGS, whole exome sequencing was performed and two heterozygous mutations in *TREX 1* gene have been identified. The mother and dauther are carryers of c.506G>A mutation previously linked to AGS. The father and dauther have novel, to our best knowledge, undescribed mutation c.197T>G.

Beside experimental attempts, there is no specific therapy for this condition so far. Two-year follow-up of our patient was marked by classical signs of AGS: severe developmental delay, occasional occurrence of chilblain like nail folds dyscolorations and mild progression of periventricular calcifications verified by repeated MR brain scans.

**Conclusion:** Our discovery of new TREX1 mutation represent a small step toward the definitive understanding of AGS. Reveling the mysteries of this rare interferonopathy will eventually improve our comprehension of SLE.

Written consent for participation in this presentation was obtained from both parents of the child.

**Disclosure of Interest**

None Declared

### P173 RESPONSE TO TOFACITINIB IN A CASE WITH FAMILIAL CANDLE-LIKE DISEASE.

#### Anna M. C. Galimberti^1^, Luisa Cortellazzo Wiel^1^, Giulia Gortani^2^, Serena Pastore^2^, Flavio Faletra^3^, Alessandra Tesser^4^, Andrea Taddio^1,2^, Alberto Tommasini^4^

##### ^1^Pediatrics, University of Trieste; ^2^Pediatrics; ^3^Human Genetics; ^4^Clinical Immunology, IRCCS Burlo Garofolo, Trieste, Italy

###### **Correspondence:** Anna M. C. Galimberti

**Introduction:** We report the case of an 18-year-old girl with clinical symptoms supportive of CANDLE syndrome. Her medical history included: lupus pernio, ulcerative lesions distributed to extremities and face, alopecia areata, periorbital edema, diffuse arthralgias, poliarticular arthritis and growth hormone deficiency. Another remarkable clinical feature was multiple painful pannicular nodules, which resulted in a severe diffuse lipodystrophy, particularly on the face, with subsequent need to undergo a course of lipofilling. She displayed a highly positive interferon signature, but her genetic evaluation, including whole exome sequencing, performed taking into account the family history of connective tissue disease, was inconclusive.

**Objectives:** Her disease had always showed a moderate response to corticosteroids, while several therapeutic attempts with immunosuppressant drugs (cyclosporine, azathioprine, ciclophosfamide, hydroxychloroquine), intravenous immunoglobulins and biological agents (anakinra, infliximab and abatacept), together with hyperbaric therapy did not lead to significant improvement of her symptoms.

**Methods:** Given the severity of the cutaneous involvement with its related emotional burden and acknowledging the role of interferon in the pathogenesis of the disease, a therapy with 10 mg BID tofacitinib (JAk1-JAK3 inhibitor) was started.

**Results:** She displayed a prompt improvement of her lipodystrophy and chilblains along with a slow but progressive hair re-growth. An almost complete healing of the panniculitic lesions was observed, along with the halving of her interferon signature score.

**Conclusion:** However, during therapy she developed raised levels of CK and LDL cholesterol. While the latter is an adverse event frequently reported in subjects treated with tofacitinib and easily manageable, increased CK are rarely observed and more difficult to be interpreted. For this reason, the dosage of the drug was reduced to 7,5mg BID. No other adverse event was noticed. Informed consent to publish had been obtained from the patient.

**Trial registration identifying number:** This work was funded by IRCCS Burlo Garofolo grant RC24/17 and Telethon Foundation grant GGP15241.

**Disclosure of Interest**

None Declared

### P174 TYPE I INTERFERON AND CXCL9 GENE EXPRESSION SIGNATURES IN HEALTHY CONTROLS, MONOGENETIC INTERFERONOPATHIES AND VARIOUS PEDIATRIC RHEUMATIC DISEASES

#### Claas Hinze, Melanie Saers, Georg Varga, Toni Weinhage, Helmut Wittkowski, Dirk Foell

##### Pediatric Rheumatology and Immunology, University Hospital Münster, Münster, Germany

###### **Correspondence:** Claas Hinze

**Introduction:** Type I interferon (IFN) gene expression signatures have been found to be strongly up-regulated in patients with various monogenetic diseases, i.e. in type I interferonopathies. These extremely rare diseases share manifestations with autoimmune connective tissue diseases. Others have used so-called IFN scores to assess the degree of type I IFN expression in peripheral blood.

**Objectives:** To report patterns of IFN gene expression in patients with monogenetic interferonopathies, pediatric rheumatic diseases and healthy controls.

**Methods:** PAXgene blood samples were obtained from children with rheumatic diseases and healthy controls (children with non-inflammatory conditions). We measured the expression of several IFN-stimulated genes (ISGs), namely ISG15, RSAD2, IFIT1, IFI44L, IFI27, SIGLEC1 (preferentially activated by type I interferons) and CXCL9 (preferentially activated by IFN-gamma) compared to the housekeeping genes GAPDH and RPL by SYBR Green PCR. Expression values were normalized to the same batch of healthy control RNA. The median normalized expression value of ISG15, RSAD2, IFIT1, IFI44L, IFI27 and SIGLEC1 (the IFN score) was assessed. Descriptive statistics were performed to analyze the findings.

**Results:** Among 24 children with non-inflammatory disease in PAXgene-derived RNA, there was strong correlation among the individual probe sets within the IFN score (Pearson’s r ranging from 0.57-1,00) but no relevant correlation of those probe sets with CXCL9; the correlation was even stronger among patients with varying degrees of type IFN activation. The 5^th^ and 95^th^ centile for the IFN score among healthy children were 1 and 4.8 and for CXCL9 2.4 and 13.7, respectively. We could demonstrate a robust and persistent up-regulation of the IFN-score in patients with known monogenetic type I interferonopathies, including C1q deficiency, SAVI or Aicardi-Goutières syndrome. The highest IFN-score was demonstrated in a patient with new-onset anti-NXP2 antibody-positive juvenile dermatomyositis, and this was responsive to change, normalizing with therapy and clinical remission. In contrast, some patients with Still’s disease demonstrated a marked up-regulation of CXCL9 with normal IFN-score.

**Conclusion:** We have established normal values for a type I IFN gene and CXCL9 gene expression signature. Assessment of the expression of ISGs may be useful in clinical practice, e.g. in order to screen for potential monogenetic type I interferonopathies, autoimmune connective tissue diseases and to assess changes in disease activity upon treatment. The use of an extended gene expression panel to also include CXCL9 which is up-regulated by IFN-gamma may assist in the understanding of the disease process in individual patients.

**Disclosure of Interest**

None Declared

### P175 DIVERSITY IN PRESENTATION AND EVOLUTION OF AUTOINFLAMMATORY DISEASES IN A FRENCH PEDIATRIC COHORT OF REFERRAL CENTER OF RHEUMATOLOGY.

#### Ambre Hittinger, Sorina Boiu, Brigitte Bader-Meunier, Wouters Carine, Pierre Quartier-Dit-Maire

##### IMAGINE Institute and Pediatric Immunology, Hematology, Rheumatology Unit, Necker-Enfants Malades Hospital, Assistance Publique Hôpitaux de Paris, Paris, France

###### **Correspondence:** Ambre Hittinger

**Introduction:** The autoinflammatory diseases (AID) include monogenic and polygenic disorders characterized by primary dysfunction of the innate immune system^1^.

**Objectives:** Our objective was to analyze the clinical spectrum, genetic background, therapeutic strategy and outcome in a cohort of AID patients followed in a reference Pediatric Rheumatology center.

**Methods:** We performed a retrospective analysis of the medical records of monogenic AID patients followed in the French reference center for juvenile arthritis and rare pediatric systemic autoimmune diseases (RAISE) between May 2007 and February 2018. Patients had been included in the French data base system for rare diseases (CEMARA) with an agreement of the Commission Nationale Informatique et Liberte. According to the French legislation, no ethics committee approval was requested for such a retrospective survey.

**Results:** One hundred sixty-six patients were included: 21 Cryopyrin-Associated Periodic Syndromes (CAPS), 5 TNF-Receptor-Associated Periodic Fever Syndrome (TRAPS), 16 Mevalonate Kinase Deficiency (MKD), 38 Familial Mediterranean Fever (FMF), 20 Behçet’s Disease (BD), 61 Chronic Recurrent Multifocal Osteomyelitis (CRMO) and 5 Synovitis, Acne, Pustulosis, Hyperostosis, Osteitis Syndrome (SAPHO). Demographics features (Table 1), show a median follow-up of 8 years (0-26), a sex-ratio of 62/104 and a median age of 8 years at disease onset. Eighty-two patients (49%) have relevant familial history. Clinical manifestations included musculoskeletal (83%), fever (50%), gastrointestinal (47%), mucocutaneous (35%), neurological (21%), ocular (16%), cardiorespiratory (10%) and genitourinary (0,6%) findings, lymphadenopathy with/or hepatosplenomegaly (12%) and growth impairment (12%). Complications/sequelae appeared in 40% of cases. One mutant allele was found in 18/21 CAPS, 4/5 TRAPS and 11/38 FMF. Two mutant alleles were present in 14/16 MKD and 20/38 FMF. The most used treatment were NSAID (62%), biological DMARDs (37%), colchicine (35%) and corticosteroids (31%). Sixty-six patients received one or more biological DMARDs: Anakinra (56%), Canakinumab (50%), Etanercept (30%), Adalimumab (4.5%), Infliximab (4.5%) and Tocilizumab (3%). Anti-interleukin-1 therapy and colchicine proved efficacy in CAPS and FMF, respectively. In addition, favorable responses have been demonstrated anti-interleukin-1 therapy in MKD, TRAPS, and colchicine-resistant FMF patients, as well as Etanercept in TRAPS, MKD and CRMO patients non-responsive to NSAIDs. Fifty-seven% and 41% of patients were in complete and partial remission, respectively, at last visit.

**Conclusion:** AID in children are associated with a broad spectrum of manifestations. Early diagnosis and referral are essential, as efficient therapy can be proposed in most cases and early complications avoided.

**Reference**

1. Russo RAG, Brogan PA. Monogenic autoinflammatory diseases. Rheumatology (Oxford). 2014 Nov 1;53(11):1927–39.

**Disclosure of Interest**

None Declared

**Table 1 (abstract 175).** Demographical data and evolution of different disease groups

	CAPS	TRAPS	MKD	FMF	BEHCET	CRMO	SAPHO	All patients
Number of patients (families)	21 (19)	5 (5)	16 (15)	38 (34)	20 (20)	61 (61)	5 (5)	166 (159)
Gender (M;F)	8;13	1;4	5;11	16;22	12;8	18;43	2;3	62;104
Age at disease onset* (years)	0† (0†-11)	5,5 (0-11)	1 (0†-11)	3 (0-9)	6 (0-12)	9.5 (3-14)	8 (4-15)	8 (0-15)
Duration of follow-up* (years)	7 (0,5-26)	3,75 (0-9)	4,25 (0-18)	3,5 (1-18)	2,75 (0-16)	12.5 (0-17)	2 (0-2,5)	8 (0-26)
Complications or side effects (%)	15 (71)	3 (60)	9 (56)	9 (23)	15 (75)	15 (24)	1 (20)	67 (40)
Number of biologic DMRADs*	2 (1-3)	1 (1-1)	2 (0-4)	0 (0-2)	0 (0-2)	0 (0-1)	0 (0-0)	0 (0-4)
Clinical remission on treatment (%)	18 (85)	0 (0)	9 (56)	24 (63)	7 (35)	15 (24)	0 (0)	73 (44)
Clinical remission off treatment (%)	0 (0)	1 (20)	0 (0)	2 (5)	0 (0)	13 (21)	3 (60)	19 (11.5)
Relapse after remissions (%)	2 (9)	1 (20)	3 (18)	1 (2)	0 (0)	7 (11)	0 (0)	14 (8)

*median (range); † day 1 of life

### P176 Withdrawn

### P177 CLINICAL CHARACTERISTICS AND PREDICTIVE FACTORS OF RECURRENT HISTIOCYTIC NECROTIZING LYMPHADENITIS

#### Hyun Joo Jung

##### Pediatrics, Ajou University Hospital, Suwon, Korea, Republic Of

**Introduction:** Although histiocytic necrotizing lymphadenitis (HNL) or Kikuchi-Fujimoto disease, which is characterized by self-limiting regional lymphadenopathy with prolonged fever, has a low recurrence rate, this rate appears to be higher and the disease progresses to severe forms associated with autoimmune diseases such as systemic lupus erythematosus (SLE) in some previous reports.

**Objectives:** We analyzed the clinical characteristics of patients with HNL focusing on cases with recurrence to define the predictive factors of recurrent HNL.

**Methods:** This was a retrospective study of patients diagnosed with Kikuchi-Fujimoto disease from April 1995 to May 2017 in South Korea. Electronic medical records were searched for clinical and laboratory manifestations. Patients with pathologically confirmed HNL through biopsy of an enlarged lymph node were enrolled and recurrence was defined as having additional episodes of febrile lymphadenopathy before or after the pathological diagnosis.

**Results:** A total of 151 pediatric patients under 18 years of age (31.5%) and 480 patients of all ages was analyzed. In the case of children under 18 years of age, the proportion of boys (43%; n = 65) and girls (57%; n = 86) was similar, but in adults, the ratio of women increased with 95 men (28.9%) and 234 women (71.1%). At the pathologic diagnosis, 455 (94.8%) had lymphadenopathy and were mainly involved in the cervical lymph nodes. Fever was present in 381 of the patients (79.4%). Cutaneous lesions (skin rash or maculopapular erythema) (5%) were the most common extranodal symptoms, and complained of myalgia, fatigue, cough, or rhinorrhea. Laboratory wise included leucopenia (22.5%; n = 108), neutropenia (9.8%; n = 47), and lymphopenia (33.3%; n = 160). Antinuclear antibodies (ANAs) were present in 14.4% of the patients (n = 69), of whom 32 (46.4%) were children under 18 years of age. Fifty-four patients (11.3 %) experienced recurrent HNL, and 1 patient recurred up to 4 times. In comparison between patients with recurrent HNL and non-recurrent HNL groups, more extranodal symptoms including cutaneous lesions, myalgia, or fatigue (p=0.013) and a prolonged duration of lymphopenia were significantly associated with recurrent HNL. The number of patients with lymphopenia was higher in the recurrent HNL groups.

**Conclusion:** Our results suggest that recurrent HNL is more frequent than reported, and recurrent HNL should be considered in patients with extranodal symptoms or prolonged lymphopenia.

**Disclosure of Interest**

None Declared

### P178 EVALUATION OF CASES DIAGNOSED WITH CRMO; SINGLE CENTER EXPERIENCE

#### Sara Sebnem Kilic, Sukru Cekic, Yasin Karali

##### Pediatric Rheumatology, ULUDAG UNIVERSITY MEDICAL FACULTY, Bursa, Turkey

###### **Correspondence:** Sara Sebnem Kilic

**Introduction:** Chronic recurrent multifocal osteomyelitis (CRMO); is a rare autoinflammatory bone disease characterized by recurrent, sterile inflammatory lesions occurring primarily in children and adolescents. Symptoms of presentation may range from mild unspecific bone pain, local swelling and warmth to severe pain, malaise, fevers and even fractures.

**Objectives:** In this study, we aimed to evaluate our patients who had a diagnosis of CRMO , retrospectively.

**Methods:** Six patients who were diagnosed with CRMO between 2010-2017 years were included in the study. The CRMO diagnosis was based on characteristic clinical features and magnetic resonance imaging findings. The clinical data were obtained from the records of electronic files.

**Results:** The female to male ratio of the cases was 4/2 and the median age was 11.15 years (6-12). The age of diagnosis was 10.35 years (4-12.5), the median period for diagnosis delay was 3 years (0.75-8). The most common complaint was localized pain (n = 6, 100%). Accompanying diseases were detected in 3 patients; 1 case had inflammatory myositis, 1 case had PFAPA syndrome and 1 case had selective IgA deficiency. Multifocal bone involvement was present in 4 (66%) cases and unifocal bone involvement in 2

(33%) cases. The most common site of disease was femur. Acute phase reactants were high most of the cases; elevated erythrocyte sedimentation rate (ESR) in 5 cases (83.3, n=6), elevated c-reactive protein level in 4 cases (66.6%, n = 6), elevated serum amyloid a level in 3 cases (60%, n=5), and elevated fibrinogen in 2 cases (50%, n=4) were present. ANA was found positive at low titer in only 1 case, whereas rheumatoid factor was negative in all cases. Non-steroidal anti-inflammatory drugs were prescribed in all cases and anti TNF drugs in 3 (Etanercept in 2 cases and adalimumab in 1 case).

The diagnosis of CRMO is difficult and no consensus exist on diagnosis and treatment. Multifocal bone lesions with characteristic radiological findings are very suggestive of CNO. The first line treatment is usually NSAIDs, however, anti TNF treatment are needed in some patients to achieve for remission. Our case is the second one who had inflammatory myositis and CRMO according the literature.

**Conclusion:** The diagnosis of CRMO is difficult and no consensus exist on diagnosis and treatment. Multifocal bone lesions with characteristic radiological findings are very suggestive of CNO. The first line treatment is usually NSAIDs, however, anti TNF treatment are needed in some patients to achieve for remission. Our case is the second one who had inflammatory myositis and CRMO according the literature.

**Disclosure of Interest**

None Declared

### P179 A CASE OF BEHÇET’S DISEASE IN A CHILD : A YOUNG GIRL WITH MULTIPLE ULCERATION AND STENOSIS OF THE HYPOPHARYNX AND LARYNGOPHARYNGEAL INVOLVEMENTS

#### Noriko Kinjo, Kazuya HAMADA, Koichi NAKANISHI

##### Pediatrics, University of the Ryukyus, Nishihara-cho, Japan

###### **Correspondence:** Noriko Kinjo

**Introduction:** Behςet’s disease (BD) is a chronic, relapsing, multisystem inflammatory disorder classified as vasculitis and characterized by recurrent oral and genital ulcerations, uveitis, and protean clinical signs of skin, central nervous system, musculoskeletal, and gastrointestinal involvements. Oral ulceration is more common in younger patients. However, there are few reports about chronic hypopharyngeal ulceration, are rare involvements in child in early phase. We report the case of a 4-year-old Japanese girl with refractory laryngeal edema who was successfully treated with multidrug therapy including infliximab (IFN).

**Objectives:** This report aimed to scrutinize of BD-related laryngeal and hypopharyngeal manifestations in a Japanese young girl. We show that rare clinical phenotype in early phase of pediatric-onset BD.

**Methods:** A young girl with multiple ulceration and stenosis of the hypopharynx and laryngopharyngeal involvement at onset.Clinical features, especially laryngeal findings by laryngoscopy in our patient were described in detail about the findings symptoms at onset and during the course. By using the paediatric Behςet’s disease (PEDBD) classification criteria, the case was diagnosed.

**Results:** In a 4-year-old Japanese girl with a 2-year history of recurrent fever, recurrent ulcerative gingivostomatitis, snoring or respiratory disorder during sleeping, was referred to our hospital with the suspicion of rheumatic diseases after tracheotomy was enforced due to the breathing disorder and dysphagia. Before tracheotomy, physical examination revealed that her voice was hoarse and dysphasia. Soft tissue lateral neck film revealed straightening of the cervical spine with edema of the epiglottis and upper airway. Laryngeal endoscopy findings showed that laryngeal and hypopharyngeal involvements were edematous severely. After tracheotomy, the patient received oxygen supplementation, methylprednisolone. Involvement in ulcerative colitis was estimated by using colon fiverscopy. By using the PEDBD classification criteria, the case was diagnosed.Her symptoms except for intestinal involvement did not change with these maneuvers. On admission, the patient showed body temperature of 37 °C, heart rate of 107/min, respiratory rate of 28/min, and blood pressure of 108/66 mmHg. Genital ulcers were absent, and repeat ophthalmologic examinations showed no symptoms. Laboratory examinations revealed the presence of nonspecific inflammation: white blood count, 11,100/μL; C-reactive protein (CRP) level, 2.0 mg/dL; electron spin resonance (ESR) level, 20 mm/h. Hypocomplementemia was absent. The results of immunological examinations were negative for specific autoantibodies, including antinuclear antibody, anti-dsDNA antibodies, and anti- neutrophil cytoplasmic antibodies. The test for HLA- B51 was positive. Bone marrow examination showed no evidence of malignancy. Then, colchicine and 1mg/kg/day of prednisolone (PSL) therapy was started. When the dose of PSL was decreased, her snoring and respiratory disorder relapsed. Therefore, infliximab (IFX) therapy was started and the dose of PSL was gradually decreased. For approximately 1 years, she showed a good general condition with laryngeal swelling improved.

**Conclusion:** There is a case of BD in a child, who has multiple ulceration and stenosis of the laryngeal and hypopharyngeal involvementsThe Upper airway manifestations could be critical to identify potentially life-threatening laryngeal involvement at onset by direct laryngoscopy. In our case might deal with the successful treatment of a laryngopharyngeal involvements with IFX as anti-TNF-alpha agents. Informed consent to publish had been obtained from the parent.

**Disclosure of Interest**

None Declared

### P180 ADENOSINE DEAMINASE TYPE 2 (ADA2) ENZYME ACTIVITY IN PAEDIATRIC RHEUMATIC DISEASES

#### Kateřina Kobrová^1,2^, Šárka Fingerhutová^2^, Pavla Doležalová^2^

##### ^1^Department of Paediatrics, Masaryk Hospital, Ústí nad Labem; ^2^DEPARTMENT OF PAEDIATRICS AND ADOLESCENT MEDICINE, GENERAL UNIVERSITY HOSPITAL IN PRAGUE, Praque, Czech Republic

###### **Correspondence:** Kateřina Kobrová

**Introduction:** Adenosin deaminase type 2 (ADA2), encoded by CECR1 gene, is an important enzyme in purine metobolism and a growth factor for endothelial and myeloid cells. Loss-of-function mutation in CECR1 gene causes deficiency of ADA2 (DADA2) leading to an autoinflammatory disease with vasculitic features. While DADA2 patients have decreased ADA2 enzyme activity, its increase has been shown in other inflammatory diseases like systemic lupus erythematosus (SLE). Less is known about ADA2 in other conditions.

**Objectives:** To assess ADA2 activity in a spectrum of inflammatory diseases and in controls in order to optimize its use as a screening test for DADA2 in the Czech Republic.

**Methods:** Serum ADA2 enzyme activity was measured using Adenosin Deaminase Assay Kit (Diazyme Laboratories) with using specific ADA1 inhibitor in patients recruited from our clinics and from controls after appropriate consent. Enzyme activity was expressed in U/L and values were compared among disease groups and controls using Mann-Whitney test.

**Results:** Total of 109 individuals were examined: 88 children (mean age 10.2, SD 5.4yrs) and 21 healthy adult volunteers. Disease groups were as follows: children with stroke of unknown aetiology (Stroke), hyper-IgD syndrome (HIDS), systemic vasculitis (VAS), systemic lupus erythematosus (SLE), juvenile dermatomyositis (JDM), PFAPA syndrome (Periodic Fever, Aphtae, Pharyngitis, Adenitis), children with non-inflammatory conditions. ADA2 activity in Stroke, HIDS and JDM patients was comparable to controls while patients with vasculitis had lower and patients with SLE and PFAPA higher ADA2 activity than controls (p=0.012;0.001;0.025 respectively. (Table 1). The lowest absolute ADA2 activity was detected in a 18 years old patient with HIDS (3 U/L) while 17 years old SLE patient had the highest activity (23 U/L).

**Conclusion:** We have demonstrated wide range of ADA2 enzyme activity in a spectrum of rheumatic diseases in children. As age influences ADA2 activity inversely highest levels in the youngest PFAPA subgroup is not surprising. Mechanisms that lead to increased ADA2 in SLE noticed also by others are not fully understood. Nevertheless, in respect to DADA2 low enzyme activity is of importance, though its exact cut-off value has not yet been established. All our patients had ADA2 activity above levels reported in DADA2. It appears that ADA2 activity assessment is a feasible method that may serve as a useful screening test in patients with clinical picture suggestive of DADA2.

**Disclosure of Interest**

None Declared

**Table 1 (abstract P180).** See text for description.

Group	N	Age mean (SD)	ADA2 U/L median (IQR)
Stroke	14	9.6 (5.0)	10.0 (9;11)
HIDS	8	15.7 (3.0)	8.0 (7; 12.5)
VAS	5	16.6 (1.1)	6.0 (6;9)
SLE	6	16.1 (0.9)	16.0 (15;17)
PFAPA	21	4.7 (2.8)	12.0 (11;13)
JDM	14	12.5 (4.4)	8.5 (8;10)
NONINFLAMMATORY	20	9.2 (4.0)	10.0 (9;12)
ADULTS	21	40 (12.8)	10.0 (9;11)

### P181 CHARACTERIZATION OF A GROUP OF 15 PATIENTS WITH MEVALONATE KINASE DEFICIENCY: SYMPTOMS AND TREATMENT WITH IL-1 INHIBITORS

#### Anna Kozlova^1^, Vasiliy Burlakov^1^, Tatiana Varlamova ^1^, Elena Raikina^1^, Olga Barabanova^2^, Sergey Zimin^2^, Anna Shcherbina^1^

##### ^1^Immunology, Federal State Budgetary Institution "National Medical Research Center of Pediatric Hematology, Oncology and Immunology named after Dmitry Rogachev" of the Ministry of Healthcare of the Russian Federation; ^2^Immunology, Children's Clinical Hospital N9, Moscow, Russian Federation

###### **Correspondence:** Anna Kozlova

**Introduction:** Mevalonate kinase deficiency (MKD) is a rare autosomal recessive autoinflammatory disease caused by mutations in MVK gene.

**Objectives:** MKD patients typically have an early onset of symptoms including recurrent episodes of high fever, abdominal pain, diarrhea and vomiting, arthralgia and lymphadenopathy (AIDAI criteria for HIDS). However not all patients have typical symptoms at the time of onset. MKD treatment remains an unsolved problem, since none of the modalities previously used for MKD treatment are fully effective in the disease control.

**Methods:** We conducted a retrospective analysis of clinical features of fifteen patients (7 females, 8 males) with genetically confirmed MKD. Ten patients received therapy with inhibitors of IL-1 (Anakinra and\or Canakinumab). One of the  patients died from amyloidosis and macrophage activation syndrome (MAS) prior to treatment initiation, her diagnosis was verified *post mortem*.

**Results:** Thirteen patients had manifested within  the first 6 months of life, one – at the age of 1.5 years, one – at three years of age. During the course of the disease all patients had periodic fever and peripheral lymphadenopathy ( mainly cervical group), as well as abdominal pain, nausea\vomiting. Six patients had diarrhea, sometimes with blood, one patient suffered from  severe constipation. Rash was seen in eight patients, myalgia, artralgia were observed only in nine. Oral ulcers were noted in eight children. Three patients had neurological involvement, one patient had it as the main symptom. One patient had periorbital edema and hyperemia during attacks, which to our knowledge, have not been reported previously in MKD. Thrombocytopenia, neutropenia were only one. One patient developed amyloidosis and MAS before IL1 inhibitor treatment initiation, which led to her death. In patients receiving anti-IL-1 therapy AIDAI index decreased from 58.3±11,2 before to 1,5±1,4 after 6 month of therapy (p =0.003).

**Conclusion:** MKD symptoms can be variable and sometimes atypical, which requires physician’s awareness. In our cohort of MKD patients anti IL-1 therapy was highly effective.

**Disclosure of Interest**

None Declared

## Treatment I

### P182 COMPARATIVE EFFICACY OF ETANERCEPT AND ADALIMUMAB IN CHILDREN YOUNGER THAN 4 YEARS

#### Ekaterina Alexeeva^1,2^, Tatyana Dvoryakovskaya^1,2^, Rina Denisova^1^, Tatyana Sleptsova^1^, Kseniya Isaeva^1^, Alexandra Chomahidze^1^, Anna Fetisova^1^, Anna Mamutova^1^, Victor Gladkikh^3^, Alina Alshevskaya^3^, Andrey Moskalev^3^

##### ^1^Federal State Autonomous Institution “National Medical Research Center of Children's Health” of the Ministry of Health of the Russian Federation; ^2^Federal State Autonomous Educational Institution of Higher Education I.M. Sechenov First Moscow State Medical University of the Ministry of Health of the Russian Federation, Moscow; ^3^Biostatistics and Clinical Trials Center, Novosibirsk, Russian Federation

###### **Correspondence:** Ekaterina Alexeeva

**Introduction:** In 2011, Etanercept (ETA) was approved for clinical application in patients with juvenile idiopathic arthritis (JIA) older than 2 years of age; adalimumab (ADA) was approved in 2013. However, the available data for these patients are not sufficient even in large-scale registers. Uveitis in older children is a factor taken into consideration when choosing anti-TNF therapy, so we believe that its onset at early age may affect efficacy of treatment with different anti-TNF drugs.

**Objectives:** This study aimed at evaluating comparative efficacy of ADA and ETA in children of young age depending on their uveitis status.

**Methods:** Comparative analysis involved patients who had initiated ETA (n=49) or ADA (n=25) therapy at the National Medical Research Center of Children's Health (Moscow) at an age of ≤4 years. None of ETA patients had previous history of uveitis. ADA patients were allocated into 2 subgroups depending on their uveitis status. Patients in group 1 had active uveitis (n=13), while group 2 patients had no active uveitis (n=12). Treatment efficacy was evaluated according to the dynamics of clinical and laboratory signs using the ACRPedi criteria. The Wallace criteria were used to evaluate whether or not remission had been achieved.

**Results:** Therapy with ETA and ADA in children under 4 years of age was found very efficacious as soon as after the first month. The CHAQ and JADAS disease activity scores, CRP and ESR laboratory values, morning stiffness duration, and the VAS score (assessed by both patient and physician) significantly decreased after 4-week therapy in all three groups (p<0.01). After 3 months of therapy, the number of affected joints (swollen or painful joints, joints with the limited range of motion and with active arthritis) reduced substantially in all three groups (p<0.01).

The percentage of ETA patients who achieved ACR50/70/90 by the end of follow-up period, with allowance for patients who discontinued treatment because of AEs or poor efficacy, was 85.7/83.7/77.6%, respectively. The percentage of ADA patients who achieved ACR50/70/90 by the end of follow-up period was 76.9/76.9/69.2% (ADA patients with uveitis) and 75/58.3/41.7 (ADA patients without uveitis). The groups were characterized by different dynamics of achieving ACR (Figs. 1-2). Treatment efficacy was comparable in ADA patients with uveitis and ETA patients without uveitis, while significantly fewer ADA patients without uveitis reached ACR70/90.

Comparable percentages of ETA patients and ADA patients with uveitis achieved remission (53.1% and 53.8%, respectively), while only 3 (25%) of ADA patients without uveitis achieved long-term clinical remission (p-values are insignificant)

**Conclusion:** In patients younger than 4 years of age, efficacy of ADA and ETA treatment differs in different patient groups according to baseline clinical parameters. ADA shows higher efficacy in achieving ACR70/90 and inactive stage of the disease according to the Wallace criteria in patients with uveitis as compared to patients with the negative uveitis status. Children without uveitis show better response to ETA, although there is a 6-10% risk of de novo uveitis. Therefore, ADA is the drug of choice for children with uveitis under 4 years of age, while ETA is preferable in children without uveitis.

**Disclosure of Interest**

None Declared

### P183 ETANERCEPT CONCENTRATION AND IMMUNOGENICITY DO NOT INFLUENCE THE RESPONSE TO ETANERCEPT IN PATIENTS WITH JUVENILE IDIOPATHIC ARTHRITIS

#### Brigitte Bader-Meunier^1^, Roman Krysiek^2^, Irene Lemelle^3^, Christine Pajot^4^, Aurelia Carbasse^5^, Isabelle Melki^6^, Pierre Quartier^1^, Jean-Marc Treluyer^1^, Alexandre Belot^7^, Salima Hacein-Bey-Abina^2^, Saik Urien^1^

##### ^1^Hôpital Necker, PAris; ^2^Hôpital Kremlin Bicêtre, Assistance Publique-Hôpitaux de Paris , Le Kremlin Bicêtre; ^3^CHRU Nancy, Vandoeuvre les Nancy; ^4^Hôpital Purpan, Toulouse; ^5^Hôpital A de Villeneuve, Montpellier; ^6^Hôpital R.Debré, Paris; ^7^Hospices Civils de Lyon, Bron, France

###### **Correspondence:** Brigitte Bader-Meunier

**Introduction:** Approximately 10–22% of biologic naive juvenile idiopathic arthritis (JIA) patients discontinue etanercept (ETN) within 12 months because of primary inefficacy or loss of response. In adult patients with rheumatoid arthritis (RA) and ankylosing spondylitis (AS), the results of studies that investigated the relationship between serum ETN levels and clinical response are conflicting. In children, no study has investigated the possible relationship between ETN circulating level or immunogenicity and disease activity in a population of JIA patients

**Objectives:** To investigate the relationship between etanercept (ETN) levels, the presence of anti-drug antibodies to ETN (ADAb) and clinical response to ETN in patients with Juvenile Idiopathic Arthritis (JIA).

**Methods:** Prospective study of JIA patients under 18. Clinical and pharmacological data were collected at two visits. Clinical remission and JIA activity on ETN were assessed according to the Wallace criteria and the Juvenile Arthritis Disease Activity Score (JADAS) respectively. ETN and ADAb serum levels assessments were determined using ELISA-based assays. The relationship between ETN concentration and the remission status or disease activity was assessed by an univariate logistic analysis and a multiple regression analyses adjusted on age, size descriptors, co-treatments and JIA classification.

**Results:** One hundred and twenty-six patients were enrolled. The median duration of ETN treatment at inclusion was 569 days (range 63-2,330). ADAb were undetectable (< 10 ng/ml) in 171/224 (76%) samples, and were > 25 ng/mL in 2. No significant relationship between ETN concentration and the remission status or disease activity was found using either univariate or multiple logistic regression analyses. No correlation was found between the remission status and the detection of ADAb.

**Conclusion:** This study did not demonstrate any correlation between circulating ETN levels and JIA activity in a large population of patients with JIA previously treated with ETN for at least 2 months. As described for adults, it confirms that ETN is marginally immunogenic in pediatric patients. These results do not support monitoring ETN concentrations or ADAb for the management of such JIA patients.

**Trial registration identifying number:** The study is registered at Clinical Trials.gov under number NCT02030613. All patients (or their parents) gave written informed consent

**Disclosure of Interest**

None Declared

### P184 EFFICACY OF JOINT INJECTIONS WITH APPLICATION OF CORTICOSTEROID IN PATIENTS WITH JUVENILE IDIOPATHIC ARTHRITIS

#### Marek Bohm, Valentina Leone, Tania Amin, Mark Wood

##### Paediatric Rheumatology, Leeds General Infirmary, Leeds, UK

###### **Correspondence:** Marek Bohm

**Introduction:** Although nowadays intraarticular corticosteroid injections may be considered a somewhat obsolete method of treatment of juvenile idiopathic arthritis, they still might serve as an alternative to stronger but more expensive DMARDs/biologic drugs.

**Objectives:** To assess the outcome of joint injections (JI) with application of corticosteroid in children with juvenile idiopathic arthritis (JIA) and factors affecting the time to flare of arthritis.

**Methods:** Clinical documentation of patients with JIA under the care of the Paediatric Rheumatology Department in Leeds General Infirmary, UK, who received joint injections with triamcinolone hexacetonide (or in some cases methylprednisolone acetate if small joints were injected) between January 2015 and December 2016 was analysed. The time to flare of arthritis was recorded based on follow-up visits. We evaluated predictors such as gender, age of disease onset, oligo- or polyarticular course of disease, disease duration, general anaesthesia, concurrent medication, CRP and ESR at the time of JI application.

**Results:** 117 patients (41 boys and 76 girls in age 1-17 years) with JIA received in total 460 intraarticular injections (75 patients in general anaesthesia). 75 children had history of arthritis in 5 or more joints. Flare of arthritis was observed in 64.1% of children after a median of 115 days. The cumulative probability of survival without flare of synovitis was 63.2% after 6 months and 49.6% after 12 months. In our cohort of patients, the outcome of joint injections was not influenced by any of named predictors.

**Conclusion:** Even in the age of DMARDs and biologic drugs, joint injections with application of corticosteroid can be considered an effective therapy in the treatment of JIA.

**Disclosure of Interest**

None Declared

### P185 BIOLOGICS IN PEDIATRIC RHEUMATOLOGY

#### Mustafa Çakan, Şerife Gül Karadağ, Nuray Aktay Ayaz

##### Pediatric Rheumatology, Kanuni Sultan Süleyman Research And Training Hospital, İstanbul, Turkey

###### **Correspondence:** Mustafa Çakan

**Introduction:** Biologics are medications that target specific molecules expressed on cells or secreted into the extracellular space. Use of biologics has increased in pediatric rheumatology since 2000s and patients with sequels have decreased sequentially.

**Objectives:** The aim of this study was to demonstrate indications of biologic usage in pediatric rheumatology.

**Methods:** Between May 2010 and September 2017, the files of all patients that biologics were used were reviewed. The patient had to be using a biologic agent for at least three months and had to be coming regularly to follow-up visits.

**Results:** In this study, 236 biologic agents were used in 172 patients. Mean duration of biologic use was 18.7 ± 14.0 months. In 69% of patients only one biologic was used while 31% of the patients needed 2 or more biologics. Six patients needed 3^rd^ biologic, one patient 4^th^ and one patient needed 5^th^ biologic agent.

Indications for biologic use were; JIA (123 cases), FMF (29 cases), CAPS (5 cases), HIDS (4 cases), Behçet disease (3 cases), CRMO (2 cases) and one case for each of the following diseases; Takayasu disease, SLE, DADA2, idiopathic recurrent pericarditis, idiopathic uveitis and pyoderma gangrenosum.

Juvenile idiopathic arthritis was the most common indication for biologics. Biologics were needed in 30.3% of all JIA cases (123/405). The most common subtypes that biologics were used in JIA were extended oligoarticular JIA (64.7%) and rheumatoid factor positive polyarticular JIA (57.1%). With biologic treatment, remission was achieved in 79% of cases while 21% of cases were active despite use of biological treatment. In 14 JIA cases biologics were used for refractory uveitis.

Familial Mediterranean fever was the second most common indication for biologic use with 29 cases. Total number of FMF patients that were followed during the same period was 1097, so the frequency of colchicine-resistant FMF was 2.6%. M694V homozygous mutation was present in 76% of colchicine-resistant FMF patients. With biologics complete remission was achieved in all FMF patients.

In 236 biologic agents, etanercept (34%) was the most commonly used agent followed by anakinra (19%), adalimumab (17%), and canakinumab (14%).

In 29% of patients isoniazid prophylaxis were used due to tuberculin skin test positivity. None of the patients develop tuberculosis infection during follow-up.

**Conclusion:** Biologics are being used more frequently in pediatric rheumatology. With the report of good outcomes biologics are used at the earlier stages of the diseases to prevent sequel formation. Our cohort also supports this opinion that biologics seem to be effective and safe in children with rheumatic diseases.

**Disclosure of Interest**

None Declared

### P186 EFFICACY AND SAFETY OF INTRA-ARTICULAR STEROID THERAPY IN CHILDREN WITH JUVENILE IDIOPATHIC ARTHRITIS

#### Nilgun Cakar^1^, Elif Çelikel^1^, Ayten Guliyeva^1^, Fatma Aydın^1^, Beyza Doganay^2^, Zeynep B. Özçakar^1^, Fatoş Yalçınkaya^1^

##### ^1^Department of Pediatric Rheumatology; ^2^Biostatistics, Ankara University School of Medicine, Ankara, Turkey

###### **Correspondence:** Nilgun Cakar

**Introduction:** The usefulness of intra-articular steroids (IAC) are well described, including rapid resolution of synovitis, relief of pain, support for normal functioning, and reduction of arthritis complications. IACs may have side effects, but they may be relatively mild compared to those associated with disease modifying antirheumatic drugs (DMARDs) or biologics.

**Objectives:** To investigate the efficacy and safety of IAC treatment in single and multiple joints in children with juvenile idiopathic arthritis (JIA) and to investigate the factors staying in remission of synovitis.

**Methods:** The clinical records of 41 patients who received IAC injection between January 2012 and December 2017 were reviewed. A total of 65 procedures were evaluated for efficacy, length of time between repeat injections, safety, and post-procedural complications. The corticosteroid used was triamcinolon hexacetonide for large joints and methylprednisolone for small or difficult to access joints. The effect of JIA subgroups, disease duration until performing IAC, ANA status, acute phase reactants and concurrent methotrexate (MTX) and biological drug treatment on the duration of remission in joints treated with IAC was investigated.

**Results:** A total of 65 IACs were performed in 41(27 female) patients. Among 41 patients, 32 had persistent oligoarthritis, 4 extended oligoarthritis, 3 polyarthritis and 2 enthesitis related arthritis. The median age at diagnosis was 7 (1.5-16) years, median follow-up time was 38 (5-69) months and the median time of the first IAC injection was 2 (0-49) months after diagnosis. Anti-nuclear antibodies were positive in 28(68%) patients. The median ESR was 33( 1-120) mm/h, and CRP was 9( 4-110) mg/L at the time of injection.

First IAC injection was done in 47 knees, 4 ankles, and 1 each wrist, elbow and proximal interphalangeal joint. In 13 patients two joints were injected at the same time. Repeat injection was done in 11 joints, all were knee.

Twenty-one of 41 patients received IAC, 17 received concurrent or ongoing MTX with IAC, 4 received MTX and anti-TNF therapy. The duration of remission without exacerbation of synovitis in joints treated with injection was found to be median 11,5 (range 1-66 ) months. The median interval between repeat injections was 27.5 (6-57) months. Three joints did not respond to IAC injection.

Subgroups of the disease and disease duration had no effect on the duration of remission. The duration of remission was longer in boys (11.5 vs 8 months), in ANA positives (13,5 vs 10 months), and in patients with concurrent MTX treatments (12 vs 9 months) . However, the differences were not statistically significant. The level of acute phase reactants had not been shown to correlate with the duration of remission.

No early or late adverse events were observed after the IAC procedure.

**Conclusion:** In single or multiple joints, IAC treatment induced remission in a significant proportion of patients without major side effects.

**Disclosure of Interest**

None Declared

### P187 TOSILIZUMAB TREATMENT IN JUVENILE IDIOPATHIC ARTHRITIS: SINGLE CENTER EXPERIENCE

#### Selcan Demir, Hafize E. Sönmez, Elif A. Aydın, Yelda Bilginer, Seza Özen

##### Deparment of Pediatric Rheumatology, HACETTEPE MEDICAL FACULTY, Ankara, Turkey

###### **Correspondence:** Selcan Demir

**Introduction:** Tocilizumab is a monoclonal antibody against interleukin-6 that has recently emerged as an alternative treatment modality for juvenile idiopathic arthritis (JIA).

**Objectives:** We aimed to discuss the clinical and laboratory findings and treatment response of JIA cases to tocilizumab therapy.

**Methods:** The study group consists of JIA patients who were treated with tocilizumab and followed up between the years of 2014-2016 in the Department of Pediatric Rheumatology of Hacettepe University Faculty of Medicine. Clinical characteristics, laboratory findings, treatment responses and disease activity were reviewed from medical charts and electronic files of the patients, retrospectively.

**Results:** There were 20 JIA patients aged between 0-18 years, were followed up from 2014 to 2016 and received tocilizumab treatment in our clinic. Treatment response could be not evaluated in two patients since they developed anaphylactic reactions due to tocilizumab. Of the remaining 18 patients, seven of them (38.9%) had polyarticular JIA, and eleven (61.1%) had systemic JIA. Platelet counts, erythrocyte sedimentation rate(ESR) and C-Reactive Protein(CRP) levels, active joint counts, and JADAS71 were significantly decreased at the third month in both polyarticular and systemic JIA, while there were not any significant differences between the third and sixth months. All of the patients with polyarticular JIA had low disease activity at six months. Eight patients with systemic JIA had an inactive disease at six months, whereas the remaining three patients had high levels of CRP without presence of any clinical symptoms. Steroid treatment was terminated at the sixth month in all patients except for three patients who continued to receive 0.05-0.25 mg/kg steroid treatment. Two patients developed thrombocytopenia, one patient developed macrophage activation syndrome, and one patient had elevated transaminases due to tocilizumab treatment.

**Conclusion:** Previous studies have shown that tocilizumab treatment is well-tolerated, effective, and safe for use in JIA patients. In the present study, we also demonstrated the efficacy of tocilizumab treatment in JIA patients from our clinic.

**Disclosure of Interest**

None Declared

### P188 AN ALTERNATIVE TREATMENT IN BIOLOGICAL DRUG ALLERGY: DESENSITIZATION

#### Selcan Demir, Hafize E. Sönmez, Erdal Sag, Yelda Bilginer, Özge Soyer, Seza Ozen

##### Department of Pediatric Rheumatology, HACETTEPE MEDICAL FACULTY, Ankara, Turkey

###### **Correspondence:** Selcan Demir

**Introduction:** The introduction of biologic drugs (BD) (such as monoclonal antibodies, cytokines, and fusion proteins) has represented a major breakthrough for the treatment of rheumatic diseases during the last two decades. However due to their high molecular weight, long half-life and parenteral use, hypersensitivity reactions may be seen frequently and may limit their use.

**Objectives:** In cases where the use of the drug is absolutely necessary, the drug may be given by desensitization if there is no contraindication.

**Methods:** In this report, we presented four cases with rheumatic diseases who were successfully treated by desensitization following anaphylaxis due to that biological drug**.**

**Results:** Four patients (1 systemic lupus erythematosus, 1 granulomatous polyangiitis, 1 polyarteritis nodosa, and 1 systemic juvenile idiopathic arthritis) participated [duration of follow-up: 5.5 (0.5-9) years]. Two patients had anaphylaxis during rituximab infusion and two patients had anaphylaxis during tocilizumab infusion [number of infusion dosage: 3 (2-5)]. All patients had active disease at time of reaction. Two patients had received another biologic drug previously. All patients were administered premedication before desensitization [methylprednisolone 2 mg / kg (maximum 60 mg) before 4 hours and 1 mg / kg hydroxyzine (25 mg maximum), ranitidine 2 mg / kg, paracetamol before 1 hour]. In the presence of respiratory distress, montelukast was also added. Desensitization protocol consisted of 12 step, drug dosage was increased by every 15 minutes. After the desensitization protocol, all patients received a median of 12 (8-15) drug infusions without any reaction.

**Conclusion:** In some cases, biologic drugs may be the only treatment alternative, however allergic reactions restrict the usage of them**.** In referral centers, desensitization protocols may help to control disease activity in these resistant patients.

**Disclosure of Interest**

None Declared

### P189 LONG-TERM SAFETY AND EFFICACY OF ETANERCEPT IN GREEK JIA ADULTS AT THE TRANSITION PERIOD

#### Despoina Dimopoulou^1^, Maria Trachana^2^, Dimitrios Deligeorgakis^1^, Artemis Koutsonikoli^2^, Polyxeni Pratsidou-Gertsi^2^, Alexandros Garyfallos^1^

##### ^1^Rheumatology Unit, 4th Department of Internal Medicine; ^2^Pediatric Immunology and Rheumatology Referral Center, 1st Department of Pediatrics, Aristotle University, Hippokration Hospital, Thessaloniki, Greece

###### **Correspondence:** Despoina Dimopoulou

**Introduction:** Our published follow-up assessment on adults with Juvenile Idiopathic Arthritis (JIA) until 2011, revealed that half of the patients had flares in adulthood, despite previous administration of several anti-TNFs.

**Objectives:** a) To describe the long-term outcome of JIA adults after transition, who had received Etanercept (ETN) (from 8/2001 to 12/2017), as the only or the last anti-TNF. B) To evaluate our model of transition in a subgroup of refractory to conventional DMARDs JIA patients, who had attended the 1^st^ Greek Rheumatology Transition Clinic for Pediatric Rheumatic Diseases, following its establishment in 2013.

**Methods:** Retrospective cohort analysis of Greek adults with established diagnosis of JIA. The disease activity state was assessed by 2 contemporary quantitative tools: the Juvenile Arthritis Disease Activity Score-10 (JADAS 10) (Clinical Remission, CR, Low, Moderate and High Disease Activity, LDA, MDA, HDA, respectively) and Wallace criteria (CR on/off ETN). Baseline was defined as the 1^st^ ETN dose.

**Results:** 35 patients (female 25), aged (mean±SD) 23.5±4.6 years, were followed for a median (IQR) of 9 (3.6, 12.7) years. The median (IQR) patients’ age at the 1^st^ ETN dose was 16.4 (12.1, 17.8) years and the lag time from JIA onset to baseline was 6.5 (2.8, 12.5) years. The majority of the patients had a polyarticular course (27/35, 77.1%), regardless of the JIA subtype. ANA positivity was recorded in 16/35 (45.7%) and previous uveitis in 4/35 (11.4%). Nine patients (25.7%) had previously received other anti-TNFs (Adalimumab n=6, Infliximab n=3). The baseline median (IQR) patients’ JADAS-10 was 14/40 (9.5, 20.5) and the ETN yr-administration was 3.5 (2, 5.5). 33/35 (94.3%) patients were compliant to ETN and the study drug was well-tolerated. Regarding safety, only 3 patients (8.6%) experienced 6 adverse events (0.04/patient-years), namely injection site reaction (n=1), uterine hemorrhage (n=1), transient ANA positivity (n=1) and uveitis (n=3, 2/3 as flares). Uveitis under ETN led to the drug discontinuation in 2 patients (1 with new, 1 with pre-existing). Four patients discontinued ETN due to efficacy loss and one due to latent TB infection. 7/35 (20%) achieved CR and discontinued ETN after 3.3 (2, 4.5) years (median, IQR). The remaining 21 patients were still on ETN at the last evaluation. The median (IQR) percent duration of CR remission on ETN, according to the Wallace criteria, was 86%. CR off ETN lasted 1.17 (0.8, 4.3) years (median, IQR). In respect to the disease activity state at the last assessment: A) among the ETN ongoing receivers, 66.7% were in a CR state, 19% in LDA, 14.3% in MDA (median JADAS: 1). B) CR achievers still remaining off ETN, sustained this state in 85.7% (median JADAS: 0).

**Conclusion:** ETN in the critical transition period proved to be safe as adverse events were rare. ETN had a long-term and sustained efficacy, allowing CR off anti-TNF in 20% of the patients. Our transition policy supported the patients’ meticulous follow-up and compliance to ETN in adulthood.

**Disclosure of Interest**

None Declared

### P190 INFECTIOUS ADVERSE EVENTS IN CHILDREN WITH JUVENILE IDIOPATHIC ARTHRITIS: DATA FROM THE JIRCOHORTE

#### Cécile Dumaine^1^, Véronique Hentgen^2^

##### ^1^Pediatrics, Hôpital Robert Debré, Paris; ^2^Pediatrics, Hôpital Mignot, Le Chesnay, France

###### **Correspondence:** Cécile Dumaine

**Introduction:** Over the last fifteen years, the development of biological agents improved dramatically the outcome of children suffering from Juvenile Idiopathic Arthritis (JIA). Adult studies indicate that treatment with biological agents lead to an increased risk of serious infections, but very little is known about this risk in children.

**Objectives:** The main objective of our study is to assess the infectious adverse events (IAEs) occurring in JIA children treated with biological agents.

**Methods:** Patients were selected from the retrospective module of the JIRcohorte, a multicenter registry for children with inflammatory and rheumatological diseases. Data concerned the period between January, 10th 2001 and August 2015. Besides demography, diagnosis and treatment options of the patients, infectious adverse event (IAE) were retrieved. For every infectious side effect, the date, the severity, the need for an hospitalization, the accountability of the treatment on the occurrence, and the consequence of the event on the biologic course were noted. The type of microorgansim and the affected organ were also analyzed. Incidence rates were expressed in number of events per 100 person-years (100p-y), and OR were calculated; for their comparison, we used the Fischer test with a significance threshold at 0.05.

**Results:** Six hundred eighty-six patients with JIA from thirteen French-speaking rheumatologic reference centers were included in the study. A total of 1095 treatments with biological agent, for 3075.4 person-years of exposure were analyzed. One hundred eighty-four IAE occurring under a treatment with biologics were described, that is to say an incidence rate of 6.0 events/100 p-y, 15.5/100 p-y with Tocilizumab, 9.6/100 p-y with Canakinumab, 7.4/100 p-y with Abatacept, 6.9/100 p-y with Golimumab, 6.7/100 p-y with Aankinra, 6.3/100 p-y with Infliximab, 4.8/100 p-y with Etanercept, and 3.7/100 p-y with Adalimumab. Risk of developing an infection was significantly higher with IL6 antagonists than with TNF-inhibitor (TNFi) (OR 3.67 IC95% [2.41 ; 5.49] p<0.001), or with IL1 antagonists (OR 2.34 IC95% [1.7 ; 4.4] p=0.004). Forty point eight percent of the IAE affected the upper respiratory tract or the ENT sytstem. A total of 12 IAE were described as severe or very severe, which represented an incidence rate of 0.4/100p-y, 43% occurring with TNFi. The risk of developing a severe or very severe infection was higher with IL6 antagonist or IL1 antagonist than with TNFi. Twenty-two herpes zoster infections, including twenty under biologics were reported (0.7/100 p-y), and there were more virus due to immunosuppression with IL6 antagonist than with TNFi. No case of tuberculosis, opportunistic infection, or death was reported.

**Conclusion:** Infectious complications with biologics occurring in children treated for JIA are rare, and in most of the cases have a mild or moderate severity, affecting mainly the upper respiratory tract or the ENT.

**Disclosure of Interest**

None Declared

### P191 ASSESSING THE CLINICAL RELEVANCE AND RISK MINIMIZATION OF ANTIBODIES TO BIOLOGICS IN JUVENILE IDIOPATHIC ARTHRITIS (JIA) (ABIRISK) - PRELIMINARY RESULTS

#### Martina Finetti, Gabriella Giancane, Francesca Bagnasco, Pavla Dolezalova, Elena Tsitsami, Maria Trachana, Isabelle Koné-Paut, Despoina Maritsi, Erkan Demirkaya, Giovanni Filocamo, Pierre Quartier, Olga Vougiouka, Rolando Cimaz, Rebecca Nicolai, Helga Sanner, Alina L. Boteanu, Alma N. Olivieri, Elzbieta Smolewska, Elisabeth Solau Gervais, Maria C. Maggio, Valda Stanevicha, Seza Ozen, Michael Hofer, Mihaela Spirchez, Maria G. Magnolia, Veronika Vargova, Denis Mulleman, Florian Deisenhammer, Marc Pallardy, Xavier Mariette, Laura Carenini, Mariangela Rinaldi, Alberto Martini, Nicolino Ruperto and for the Paediatric Rheumatology International Trials Organisation (PRINTO)

##### Clinica Pediatrica e Reumatologia - PRINTO, Istituto Giannina Gaslini, GENOA, Italy

###### **Correspondence:** Martina Finetti

**Introduction:** ABIRISK is a project funded by Innovative Medicine Initiative, with the aim to investigate anti-drug antibody (ADA) formation in the treatment of JIA with biologics (BPs). A major limitation to the use of biologics is the development of ADA that may decrease the efficacy of BPs.

**Objectives:** The aim of this project is to improve the capability to predict biologic immunogenicity in JIA patients.

**Methods:** JIA Patients (by ILAR criteria) followed by 24 PRINTO centres in 12 countries were prospectively enrolled and treated with Etanercept, Adalimumab or Tocilizumab. Patient’s data were obtained from Pharmachild, a pharmacovigilance data registry of JIA patients. For each patient detailed demographic and clinical information were reported; biologic samples were collected for PK and ADA detection before therapy start as well as at periodic visits up to month 18 of follow-up. Disease activity was assessed with Juvenile Arthritis Disease Activity Score-10 (JADAS10) and JIA American College of Rheumatology (JIA ACR) criteria.

**Results:** 148 patients were included in the analysis. Five patients were considered twice because treated with 2 different sequential biologics. Demographic and clinical data by therapy are represented in the table. 54% of the patients were treated with Adalimumab. Disease duration was higher in the group receiving Tocilizumab. Disease activity showed a pattern of improvement over time both globally and for each treatment group (Table 1). Anti-adalimumab and anti-tocilizumab antibodies were detected respectively in 14 and 3 patients, while no patient developed antibodies anti-etanercept.

**Conclusion:** Preliminary data show a global improvement of disease activity during follow-up period. Analysis of the correlation between drug concentration/ADA development and clinical information will help to determine which patients will respond best to which biologic.

**Disclosure of Interest**

M. Finetti: None Declared, G. Giancane: None Declared, F. Bagnasco: None Declared, P. Dolezalova: None Declared, E. Tsitsami: None Declared, M. Trachana: None Declared, I. Koné-Paut: None Declared, D. Maritsi: None Declared, E. Demirkaya: None Declared, G. Filocamo: None Declared, P. Quartier: None Declared, O. Vougiouka: None Declared, R. Cimaz: None Declared, R. Nicolai: None Declared, H. Sanner: None Declared, A. Boteanu: None Declared, A. Olivieri: None Declared, E. Smolewska: None Declared, E. Solau Gervais: None Declared, M. Maggio: None Declared, V. Stanevicha: None Declared, S. Ozen: None Declared, M. Hofer: None Declared, M. Spirchez: None Declared, M. Magnolia: None Declared, V. Vargova: None Declared, D. Mulleman: None Declared, F. Deisenhammer: None Declared, M. Pallardy: None Declared, X. Mariette: None Declared, L. Carenini: None Declared, M. Rinaldi: None Declared, A. Martini: None Declared, N. Ruperto Grant / Research Support from: The G. Gaslini Hospital, which is the public Hospital where I work as full time public employee, has received contributions from the following industries : BMS, GlaxoSmithKline (GSK), Hoffman-La Roche, Novartis, Pfizer, Sanofi Aventis, Schwarz Biosciences, Abbott, Francesco Angelini S.P.A., Sobi, Merck Serono for the coordination activity of the PRINTO network. This money has been reinvested for the research activities of the hospital in fully independent manners besides any commitment with third parties., Consultant for: Consultancies and speaker's bureaus from AbbVie, Ablynx, Amgen, AstraZeneca, Baxalta Biosimilars, Biogen Idec, Boehringer Ingelheim, Bristol-Myers Squibb, Celgene, Eli-Lilly, EMD Serono, Gilead Sciences, Janssen, MedImmune, Novartis, Pfizer, R-Pharm, Roche, Sanofi, Servier, Takeda

**Table 1 (abstract P191).** Demographic and clinical characteristics of the JIA patients by therapy. Data are medians (1st –3rd quartiles) or frequencies (%).

	Adalimumab n=80 (54%)	Etanercept n=36 (24%)	Tocilizumab n=32 (22%)	Total n=148
Age at onset, years	6.1 (3.1-10.4)	4.9 (1.9-9.7)	6.1 (3.8-9.1)	5.8 (2.7-10.1)
Age at JIA diagnosis, years	6.8 (3.3-11.9)	5.5 (2.1-10.6)	6.4 (4.1-9.2)	6.4 (3.2-11.0)
Disease duration, years	2.4 (0.9-4.8)	`2.2 (1.1-5.5)	4.1 (1.4-6.5)	2.6 (1.1-5.8)
Female	60 (75.0)	26 (72.2)	23 (71.9)	109 (73.6)
ILAR JIA category				
Systemic	2 (2.5)	0	7 (21.9)	9 (6.1)
Oligoarthritis	30 (37.5)	7 (19.4)	9 (28.1)	46 (31.1)
Polyarthritis	27 (33.8)	23 (63.9)	13 (40.6)	63 (42.6)
Psoriatic arthritis	1 (1.2)	0	0	1 (0.7)
Enthesitis related arthritis	16 (20.0)	3 (8.3)	0	19 (12.8)
Undifferentiated arthritis	4 (5.0)	3 (8.3)	3 (9.4)	10 (6.7)
Anti-Drug Antibodies*	14 (18%)	-	3 (9%)	17 (11%)
Anti-Drug Antibodies**	5 (6%)	-	2 (6%)	7 (5%)
JADAS 10				
M0	12.6 (7–17)	15.6 (10–18.5)	15.6 (11.6–23.2)	13.5 (8.5–18.3)
M1	4 (1.5–6.5)	5.7 (3.4–12.7)	5.5 (3–11.4)	4.5 (2–8)
M3	2.7 (1–5.9)	2.2 (0–7.7)	2.5 (0.7–6)	2.5 (0.5–5.9)
M6	1.5 (0–5)	1 (0–8)	2 (0.5–7.5)	1.7 (0–5.8)
M12	0.5 (0–5)	0.5 (0–4.2)	2 (0.5–4.5)	0.5 (0–4.7)
ACR 70				
M1	30 (46.2)	8 (33.3)	5 (31.3)	43 (41.0)
M3	40 (62.5)	17 (63.0)	10 (62.5)	67 (62.6)
M6	41 (67.2)	18 (62.1)	13 (76.5)	72 (67.3)
M12	42 (71.2)	19 (76.0)	13 (76.5)	74 (73.3)

*in at least 1 determination ** in at least 2 determination

### P192 METHOTREXAT TREATMENT IN 2 PATIENTS WITH ARTHRITIS AFTER LIVER TRANSPLANTATION ON PERSISTENT TACROLIMUS TREATMENT.

#### Agnieszka Gazda, Beata Kołodziejczyk, Piotr Gietka

##### Clinic of Paediatric Pheumatology, Nationale Institute of Geriatrics, Rheumatology and Rehabilitation, Warsaw, Poland

###### **Correspondence:** Agnieszka Gazda

**Introduction:** We are presenting 2 patients after liver transplantation who developed juvenile idiopathic arthtiris. Patients received combined therapy of tacrolimus and methotrexat.

**Objectives:** Case 1: Boy, born 18.08.2013, underwent liver transplantation in 2014, from mother, due to cirrhosis in the course of atresia of the bile ducts, on the chronic tacrolimus, heviran treatment.

In 08.2015 patient developed symptoms: pains and swelling of knees, wrists, elbows. In laboratory tests:ESR-95mm/h, Rheumatoid factor (RF) negative, antinuclear antibodies (ANA) negative;  the number of EBV virus copies 3870, after correction of dose of tacrolimus 2050, other infections were excluded: CMV ( DNA CMV negative), Borelia brg, Yersinia sp, Tuberculosis, Campylobacter, Mycoplasma pn , Clostridium dif.. In USG active inflammation was found with effusion , swelling of synowial membrane. The Juvenile idiopathic arthritis polyarticular onset was diagnosed. Patient received encorton , metotrexat , tacrolimus. The remission was achieved.

**Methods:** Case 2 : Boy with autism, born 2003, in the neonatal period surgery due to obstruction of the duodenum secondary to the annular pancreas and imperfect turn of the intestine ( bowel restitution ); in the first year of life operational correction of ASD, VSD.

In 2012 patient underwent liver transplantation due to congenital extrahepatic portosystemic shunts  with hyperamonaemia and hepato-pulmonary syndrome and focal changes in the liver, tacrolimus treatment was conducted. Since 11.2015 there have been: pain, big edema and limitation of joint mobility in right wrist , rigth foot ( MTP joins). CRP-24 mg/l, ESR-54mm/h, WBC- 13 G/L, present HLA B27 antigen , negative RF, ANA, infection were excluded: Borelia brg, Yersinia sp, Tuberculosis HCV . In Xray of hand (04.2016)- generalized osteoporosis and periarticular osteoporosis bones of wrist and bones of IV ,V MCP joints of right hand. In feet Xray-hypetrophy of further physis of metatarsal bones of right foot. In USG thickened synovium with intensive increased vascularity in the joints of right wrist and MCP; also in right MTP 3-4, PIP 3-4 joints, thickened synovium in the MTP 1-2 joints and the toe IP. USG of liver – no abnormalities. Enthesitis related arthritis was diagnosed. Methotrexat was used to treat 15mg/ week , then 20mg/week ( 15mg.m2 bs), a gradual resolution of inflammatory changes in the joints was achieved. In 2017 in USG of right foot was in norm, in right wrist was thickening, moderately increased vascularity of synovium , intrarticular glikokortykosteroid was injected to the wrist. Within 2 years of treatment liver enzymes were correct. In January 2018, was noticed temporarily increase of GGTP-154 U/L , ALAT-34 n 26 U / L, AST-23 U / L, CMV DNA - not detected, EBV DNA - not detected. Ultrasound showed transplanted liver unimpaired, non-biliary oblong vein, portal vein width 6mm, PV vmax 48cm / sec, HAV max 69cm / sec, hepatic flow three-phase normal. In Liver biopsy 01.2018: The organ structure preserved, without features of acute cell rejection. The stipend of fibrous connective tissue with the creation of port-port bridges within the port space (S3 according to Ishak). Discrete inflammatory infiltrates of a chronic nature in the area of the gantry-biliary and focaly intralobulary, no cholestasis, no steatosis, no ductopenia.

The hepatologist decided to add to treatment encorton, continue tacrolimus and methotrexat.

**Results:** The use of methotrexate due to JIA in patients after liver transplantation on tacrolimus treatment allowed for rapid improvement in joints.

**Conclusion:** During 2 years of obserwation frequent laboratory control and liver assessment in USG and liver biopsy showed a stable function of the transplanted organ. Informed consent to publish had been obtained.

**Disclosure of Interest**

None Declared

### P193 THE IMPACT OF A LONG PHARMACOLOGICAL TREATMENT ON RENAL FUNCTION IN PATIENTS WITH JIA

#### Maria Francesca Gicchino, Pierluigi Marzuillo, Stefano Guarino, Anna Di Sessa, Alma N. Olivieri

##### Department of Women, Child and General and Specialistic Surgery, University if the Study of Campania Luigi Vanvitelli, Naples, Italy

###### **Correspondence:** Maria Francesca Gicchino

**Introduction:** Juvenile Idiopathic Arthritis (JIA) represents the most common chronic rheumatic disease in childhood and an important cause of short and long term disability. Non steroidal antinflammatory drugs (NSAIDs) and intra-articular steroids represents the first line treatment for JIA. Systemic steroids, Disease modifying anti-rheumatic drugs (DMARDs) and biologic drugs are used in children with severe disease. It is not possible at onset of disease to predict when a child can suspend pharmacological treatment, so children affected from JIA have to continue pharmacological treatment for several months, or in some case for several years.

**Objectives:** We aimed to investigate if a long exposure to pharmacological treatment can be responsible of renal injury, evaluating patients affected from JIA without primary kidney disease.

**Methods:** We retrospectively evaluated 110 patients suffering from JIA. JIA diagnosis was made according to ILAR criteria and treatment was assigned with recommendations of the American College of Rheumatology. For each patient we recorded the type and the duration of pharmacological treatment and the presence of renal injury. Renal injury was defined by the presence of hypertension, proteinuria or reduced eGFR. Hypertension was defined by the persistence of systolic and/or diastolic blood pressure greater than the 95th percentile corrected for age, gender and height. Proteinuria was defined on a first morning void as persistent (confirmation within 3 months) UPr/Cr greater than 0.5 mg/mg for children lessthan 2 years old and greater than 0.2 mg/mg for those more than 2 years old. Reduced eGFR was defined as eGFR less than 90 ml/minute/1.73 m2 for children older than 2 years and according to specific reference values for age for children less than 2 years old.

**Results:** The mean age at the last follow-up visit was 13.3±5.61 years. Nine out of 110 patients (8.1%) showed kidney injury (8 with hypertension and 1 with proteinuria). Considering the whole population, the cumulative proportion of patients free from renal injury at 216 months from the JIA onset was 56.8%. The patients with renal injury were significantly older (18.4±3.1 vs 12.9±5.6; p=0.004), with a longer JIA duration (152.1±58.3 vs 75.2±56.7; p= 0.0003) and with higher number of months of treatment with NSAIDs (70.0±50.1 vs 39.9±38.4; p=0.02). There was a linear relation between duration of JIA and duration of NSAIDs administration (R-squared 46.2%; p<0.0001)

**Conclusion:** The longer duration of the JIA and longer duration of treatment with NSAIDs are associated to the development of renal injury.

**Disclosure of Interest**

None Declared

### P194 COMPLEMENTARY AND ALTERNATIVE MEDICINE IN PEDIATRIC RHEUMATOLOGY PATIENTS - A SINGLE CENTER STUDY

#### Barbara Grubisic-Cabo^1^, Mandica Vidovic^1^, Lovro Lamot^1,2^, Zarko Bajic^3^, Miroslav Harjacek^1,2^

##### ^1^Pediatric Clinic, Department of Clinical Immunology and Rheumatology, Sestre Milosrdnice University Hospital Center, Zagreb, Croatia; ^2^University of Zagreb School of Medicine; ^3^Biometrika Healthcare Research, Zagreb, Croatia

###### **Correspondence:** Barbara Grubisic-Cabo

**Introduction:** Rheumatic diseases are one of the most common chronic diseases in childhood. Although novel therapies are emerging, there is still a significant number of patients not reaching remission easily. That is why many parents/legal guardians turn to complementary and alternative medicine (CAM), usually not revealing it to their physicians. Even though valid tools for assessing CAM use exist, such as questionnaires, they are not routinely used probably due to their relative complexity.

**Objectives:** To assess the prevalence and patterns of CAM use and variables associated with it in children with chronic rheumatic diseases in a single center pediatric rheumatology clinic.

**Methods:** We conducted the cross-sectional study from April to May 2017 at the Department of Clinical Immunology and Rheumatology, Sestre Milosrdnice University Hospital Center, Zagreb, Croatia, on the consecutive sample of parents/legal guardians of the children diagnosed with chronic rheumatic diseases. The outcome was the self-reported frequency of CAM use. For the purposes of our study, we developed an anonymous questionnaire to answer our study questions and written informed consent was obtained from all the parents/legal guardians prior to enrollment in the study.

**Results:** Out of the 100 children whose parents/legal guardians were interviewed, 67 (67%) had juvenile idiopathic arthritis (JIA) while other diagnosis included systemic lupus erythematosus, juvenile scleroderma, juvenile dermatomyositis, etc. 63 (63%; CI 95% 54%−73%) of parents/legal guardians have used CAM in the treatment of their child. Out of those, 43/63 parents/legal guardians (68,25%) did not inform any medical staff about it. The majority of them, 55/63 (87%; CI 95% 77%−94%) used it concomitantly to their standard therapy and 40/63 (64%; CI 95% 50%−75%) declared their intent to use it in the future as well. Only 6/63 (10%; CI 95% 4%−20%) were sure they would no longer use it. Out of the listed CAM groups, the most commonly used were herbal remedies (30.49%) along with orthomolecular therapy (25%) such as vitamins, minerals, immune boosting products and probiotics. Statistical analyses of collected data showed that a significantly higher frequency of CAM use was associated with parents/legal guardians with lower monthly income (OR 9.66; p<0.05), acquaintances who use CAM (OR 4.58; p<0.05) and certain types of disease (JIA-polyarticular type; OR 5.40; p<0.05; JIA-oligoarticular type; OR 4.30; p<0.05). Parents/legal guardians from medical professions have used CAM less often than those from non-medical professions (OR 0.27; p<0.05).

**Conclusion:** Our study showed that the frequency of CAM use is associated with the nature of the parent/legal guardian’s profession, their monthly income, acquaintances who use CAM and their child’s diagnosis. CAM was concomitantly used to the standard therapy and medical staff was not informed about it (87%; 68.25%, respectively). Due to rarely documented CAM side-effects and the possible interferences between CAM and standard therapy, it is essential for pediatric rheumatologists and other medical personnel to improve the communication with families in order to achieve optimum disease management outcomes. Therefore, more data on population characteristics and commonly used types of CAM, would allow medical staff to raise awareness of the benefits and consequences that such therapies offer.

**Disclosure of Interest**

None Declared

### P195 BIOLOGIC MEDICATION ACCORDING TO PATIENT AND DISEASE CHARACTERISTICS IN JUVENILE IDIOPATHIC ARTHRITIS – A HOSPITAL BASED REGISTRY

#### Trude M. Ingebrigtsen, Helga Sanner, Torhild Garen, Berit Flatø

##### Department of Rheumatology, Oslo University Hospital, Rikshospitalet, Oslo, Norway

###### **Correspondence:** Trude M. Ingebrigtsen

**Introduction:** Patients with juvenile idiopathic arthritis (JIA) and their use of biologic medication were registered in our hospital-based registry at Oslo University Hospital, Rikshospitalet in the period 2008-2015.

**Objectives:** To evaluate patient and disease characteristics, according to use of biologic medication, from the registry.

**Methods:** JIA patients were classified using the ILAR criteria and data was registered several times for each patient. The registrations are divided into “start up”, “controls” and “discontinuation”. Gender, date of registration, self-reported Childhood Health Assessment Questionnaire (CHAQ) and physicians’ assessment of disease activity (VAS 0-10cm) were recorded, in addition to number of active joints, number of joints with limited range of motion, ESR and CRP. For patients under the age of 16 years CHAQ were reported by one of the parents. CHAQ disability index (range 0-3) was calculated.

**Results:** In the period 2008-2015 a total of 1047 registrations concerning biologic treatment were made on 264 patients with JIA. 178 (66%) were females. Mean age time at start up registration was 10.7 years (SD 4.7), at registrations from control visits 12.1 years (SD 4.0) and at registrations from discontinuation 12.4 years (SD 4.2).

The numbers of registrations were distributed as follows; start up 92 (9%), controls 901 (86%) and discontinuation of biologics 54 (5%) (18 due to remission, 25 due to insufficient response and 13 due to adverse events). Physicians’ assessment of disease activity, number of active joints, number of joints with limited range of motion, CRP, ESR and CHAQ were significantly higher in the “start up medication group” than in the registrations from control visits; all p`s <0.01 (data not shown). When comparing registrations from control visits with those in the discontinuation group, disease variables were significantly better in the 18 registrations from patients discontinuing biologics due to remission”; all p`s<0.05, and significantly worse in registrations from patients discontinuing due to insufficient response; all p`s <0.05 (data not shown).

Severe disability (CHAQ score >= 1.5), was found in 146 (14%) of the total registrations; 24 (26%) in the “start up group”, 113 (13%) in the "control group", and 9 (17%) of the registrations in the "discontinuation group"; however, no significant differences between the groups were found

**Conclusion:** Registrations on biologic medications consist mainly of patients with polyarticular and extended oligoarticular JIA (55% of registrations), but also a relatively large proportion of systemic JIA (21% of registrations). The most frequently used biologics were etanercept, followed by adalimumab, tocilizumab and infliximab.

Disease activity measures, inflammatory parameters and physical status improved from start up registrations to control visits.

Severe disability was found in a relatively large proportion of registrations.

**Disclosure of Interest**

None Declared

**Table 1 (abstract P195).** Biologic medication according to JIA cathegory.

Type of medication	Registrations*	Systemic JIA*	Poly (RF neg) JIA*	Poly (RF pos) JIA*	Oligo (persistent) JIA*	Oligo (extending) JIA*	Enthesitis related JIA*	Psoriatic JIA*	Undifferentiated JIA*
Registrations, total	1047 (100)	231 (21)	363 (35)	98 (9)	113 (11)	138 (13)	66 (6)	31 (3)	7 (1)
Etanercept	490 (47)	34 (7)	177 (36)	63 (13)	45 (9)	94 (19)	54 (11)	21 (4)	2 (0.4)
Infliximab	91 (9)	10 (11)	34 (37)	12 (13)	24 (26)	4 (4)	-	4 (4)	3 (3)
Adalimumab	219 (21)	15 (7)	104 (48)	15 (7)	41 (19)	24 (11)	12 (6)	6 (3)	2 (1)
Anakinra	45 (4)	42 (93)	3 (7)	-	-	-	-	-	-
Tocilizumab	155 (15)	109 (70)	34 (22)	6 (4)	1 (0.6)	5 (3)	-	-	-
Abatacept	24 (2)	6 (25)	7 (29)	2 (8)	2 (8)	7 (29)	-	-	-
Other	26 (2)	18 (69)	4 (15)	-	-	4 (15)	-	-	-

*Numbers of registrations (%)

### P196 DOES METHOTREXATE INTOLERANCE DIFFER BETWEEN CHILDREN WITH JUVENILE IDIOPATHIC ARTHRITIS AND CHILDREN WITH ACUTE LYMPHOBLASTIC LEUKAEMIA?

#### Nini Kyvsgaard^1^, Torben Mikkelsen^1^, Mikael Thastum^2^, Anne E. Christensen^3^, Peder S. Wehner^4^, Karsten Nysom^5^, Troels Herlin^1^

##### ^1^Pediatric and Adolescent Medicine, Aarhus University Hospital, Denmark, Aarhus N; ^2^Department of Psychology and Behavioral Sciences, Aarhus University, Aarhus; ^3^Department of Paediatric Rheumatology; ^4^Department of Paediatric Haematology and Oncology, Odense University Hospital, Odense; ^5^Pediatric and Adolescent Medicine, Rigshospitalet, Copenhagen, Denmark

###### **Correspondence:** Nini Kyvsgaard

**Introduction:** Low-dose methotrexate (MTX) is a cornerstone in the treatment of juvenile idiopathic arthritis (JIA) with a polyarticular course and an essential component of the maintenance treatment of acute lymphoblastic leukaemia (ALL). MTX intolerance associated to low-dose MTX treatment is a known phenomenon within JIA and a tool quantifying MTX intolerance has been developed(1).

**Objectives:** To compare MTX intolerance to low-dose MTX treatment in children with JIA and children with ALL.

**Methods:** Enrolled were children diagnosed with JIA[1]or ALL[2], treated with low-dose MTX and aged ≥ 9years. Children were excluded if not fluent in Danish or if cognitively impaired. Children with JIA were enrolled from December 2013 - July 2016 and children with ALL from April 2015 - August 2017. MTX intolerance was determined using the MTX intolerance severity score (MISS)(1) completed by the patient’s parents. The MISS total score ranges from 0 to 36. A child is categorized MTX intolerant if MISS total score ≥6 and min. 1 point for an anticipatory and/or associative and/or behavioural symptom. A blood sample was drawn for an erythrocyte-MTX-polyglutamate concentration measurement (E-MTX-PG).The local research ethics committee approved this cross-sectional study.

1. According to ILAR criteria
2. Treated on the Nordic Society of Pediatric Hematology and Oncology (NOPHO) ALL-2008 protocol

**Results:** The MISS and the Ery-MTX-PG analysis were completed for 119 children with JIA and 22 children with ALL. The JIA group was treated with a lower MTX dose than the ALL group and had a lower E-MTX-PG level than the ALL group (Table 1). Within the JIA group there was no difference in the median MTX dose between the subgroup treated with low-dose MTX orally (MTX_O_) and the subgroup treated subcutaneously (MTX_sc_) (Median MTX dose: MTX_O_=9.6 mg/m2/week (IQR: 9.0-10.7) and for MTXsc =9.8 (IQR: 8.7-11.0), p=0.87). But the mean E-MTX-PG level was lower in the MTX_O_subgroup compared to the MTX_sc_subgroup (mean E-MTX-PG: MTX_O_=4.6 (95% CI: 4.1 – 5.1) and for MTX_SC_=5.6 (95% CI: 5.2-6.1), p=0.0033).

The JIA group had a higher MISS than the ALL group (for JIA median MISS =8 (IQR: 3-14) and for ALL =1 (IQR: 0-3), p<0.0001). In addition, the JIA group had a larger proportion of MTX intolerant children compared to the ALL group (JIA: 73/119 (61%) versus ALL: 4/22 (18%); p<0.001). We found no differences between boys or girls being MTX intolerant neither in the JIA group, nor in the ALL group.

**Conclusion:** The level of MTX intolerance was higher and more attributed to anticipatory, associative and behavioural symptoms in the JIA group compared to the ALL group. This finding contrasted the fact that the JIA group was treated with a lower MTX dose and had a lower E-MTX-PG level than the ALL group. However, the MTX treatment duration was longer in the JIA group compared to the ALL group. Furthermore, within the ALL group the MTX intolerant children had longer duration of low-dose MTX treatment compared to the MTX tolerant children.

**Trial registration identifying number:**

**Reference**

(1) Bulatovic M, Heijstek MW, Verkaaik M, van Dijkhuizen EH, Armbrust W, Hoppenreijs EP, et al. High prevalence of methotrexate intolerance in juvenile idiopathic arthritis: development and validation of a methotrexate intolerance severity score. Arthritis Rheum 2011 Jul;63(7):2007-2013.

**Disclosure of Interest**

None Declared

**Table 1 (abstract P196).** See text for description

Patient Group	JIA (N=119)	ALL (N=22)	p
Age, median (IQR) years	13.3 (11.3-15.1)	11.9 (10.2-14.6)	0.18
Gender, girls: boys	81 : 38	9 : 13	-
MTX_O_: MTX_SC_, n	45 : 74	22 : 0	-
MTX dose, median (IQR) mg/m^2^/week	9.7 (9.0-10.9)	14.7 (10.4-20.0)	0.0002
MTX_intol_ : MTX_tol_	9.6 (8.6-11.0) : 10.1 (9.4-10.9)^a^	16.3 (14.7-18.4): 13.3 (9.0-21.0)^b^	^a^0.22 ; ^b^0.55
E-MTX-PG, mean (95% CI) nmol/ mmol Hb	5.3 (4.9-5.6)	6.5 (5.5-7.6)	0.028
MTX_intol_: MTX_tol_	5.4 (5.0-5.9) : 5.0 (4.5-5.5)^a^	7.6 (4.5-10.8) : 6.3 (5.0-7.5)^b^	^a^0.21; ^b^0.31
MTX treatment duration, median (IQR) days	336 (141-766)	114 (71-254)	0.0003
MTX_intol_: MTX_tol_	370 (143-766) : 262 (141-629)^a^	300 (191-354) : 97 (64-197)^b^	^a^0.38 ; ^b^0.033

### P197 IS METHOTREXATE-INDUCED NAUSEA OR METHOTREXATE INTOLERANCE IN JUVENILE IDIOPATHIC ARTHRITIS ASSOCIATED WITH ANXIETY OR COPING STRATEGIES?

#### Nini Kyvsgaard^1^, Torben Mikkelsen^1^, Mikael Thastum^2^, Anne E. Christensen^3^, Troels Herlin^1^

##### ^1^Pediatric and Adolescent Medicine, AARHUS UNIVERSITY HOSPITAL, DENMARK, Aarhus N; ^2^Department of Psychology and Behavioral Sciences, Aarhus University, Aarhus; ^3^Department of Paediatric Rheumatology, Odense University Hospital, Odense, Denmark

###### **Correspondence:** Nini Kyvsgaard

**Introduction:** Gastrointestinal adverse effects to low-dose MTX treatment are a clinical challenge in juvenile idiopathic arthritis (JIA) and include MTX-induced nausea and MTX intolerance. The latter is a compound concept of stomachache, nausea, vomiting and behavioural symptoms associated with low-dose MTX. Inter-individual variation exists in the level of MTX-induced nausea and MTX intolerance. For high-dose chemotherapy anxiety and coping strategies have been associated to chemotherapy-induced nausea and vomiting(1).

**Objectives:** To investigate if MTX-induced nausea or MTX intolerance is associated with anxiety or coping strategies in children with JIA treated with low-dose MTX.

**Methods:** Children were eligible if diagnosed with JIA (ILAR criteria), aged ≥9 years and treated with low-dose MTX. If children were cognitively impaired or not fluent in Danish they were excluded. Enrolment was from December 2013 - July 2016**.** The anxiety level was determined using Beck’s Youth Inventory – Anxiety (BAI-Y)(2). Coping strategies were evaluated by a nausea coping questionnaire (NCQ)[1]. MTX-induced nausea was registered by the children’s completion of a nausea diary. MTX intolerance was assessed by the MTX intolerance severity score(MISS)(3) completed by the parents. The local research ethics committee approved this cross-sectional study.

1. Subscales: Information seeking/problem solving, seeking social support, positive self-statements, behavioural distraction, cognitive distraction, externalizing and internalizing/catastrophizing. Frequency of use: 1=never, 2=hardly ever, 3= sometimes, 4 = often, 5 = very often.

**Results:** Enrolled were 121 JIA patients (82 girls: 39 boys), the median age (IQR) was 13.3 (11.3-15.1) years.The MISS was completed for 120 children, 77 children completed the nausea diary (min. 7 days) and 119 completed the BAI-Y and the NCQ. MTX was given orally to 45 patients (MTX_O_) and subcutaneously to 75 patients (MTX_SC_). Seventy-three children (61%) were MTX intolerant (MISS≥6)(3) (MTX_O_: 23/45 (51%); MTX_SC_: 50/75 (67%); p=0.07) and the median MISS score (IQR) was 8 (3-13.5) (MTX_O_: 6 (1-12); MTX_SC_: 9 (4-14); p=0.044). Fifty-six children (73%) had MTX-induced nausea deduced from the diaries (MTX_O_: 16/27; MTX_SC_: 40/50; p=0.051). The BAI-Y raw score was higher in the MTX-induced nausea (diary) subgroup compared to all others, but there was no difference in BAI-Y raw scores between the MTX intolerant and MTX tolerant subgroups (Table 1). Unfavourable coping strategies were used more often in the MTX intolerant subgroup and the MTX-induced nausea (diary) subgroup compared to all others.

1. The scores of the subscales externalising and internalising/catastrophizing are summed up, ranging from 10 to 50.

**Conclusion:** MTX-induced nausea was associated with unfavourable coping strategies (externalizing and catastrophizing) and anxiety in children with JIA, MTX intolerance was associated with unfavourable coping strategies. These psychological factors need attention when children with JIA commence low-dose MTX treatment, in order to intervene when appropriate.

**Trial registration identifying number:** (1) Tyc VL, Mulhern RK, Jayawardene D, Fairclough D. Chemotherapy-induced nausea and emesis in pediatric cancer patients: an analysis of coping strategies. J Pain Symptom Manage 1995 Jul;10(5):338-347.

(2) Thastum M, Ravn K, Sommer S, Trillingsgaard A. Reliability, validity and normative data for the Danish Beck Youth Inventories. Scand J Psychol 2009 Feb;50(1):47-54.

(3) Bulatovic M, Heijstek MW, Verkaaik M, van Dijkhuizen EH, Armbrust W, Hoppenreijs EP, et al. High prevalence of methotrexate intolerance in juvenile idiopathic arthritis: development and validation of a methotrexate intolerance severity score. Arthritis Rheum 2011 Jul;63(7):2007-2013.

**Disclosure of Interest**

None Declared

**Table 1 (abstract P197).** See text for description

	JIA (N=121)	p
MTX_O_: MTX_SC_, n	45 : 76	
MTX dose, median (IQR) mg/m^2^/week	9.7 (9.0 – 10.9)	-
MTX_o_ : MTX_SC_	9.6 (9.0-10.7) : 9.8 (8.8-11.1)	0.86
MTX treatment duration, median (IQR) days	340 (142-766)	-
MTX_o_ : MTX_SC_	261 (143-543) : 417 (134-853)	0.39
BAI-Y raw score (total 0 – 60), mean (95%CI)	27.4 (26.2 – 28.6)	-
MTX_intol_ : MTX_tol_(MISS)	27.8 (26.1-29.4) :26.9 (25.3-28.6)	0.48
MTX-induced nausea: All others (Diary)	28.0 (26.3-29.6) : 24.4 (22.8-26.0)	0.0024
Unfavourable Coping Strategies[1], mean (95% CI)	16.5 (15.5-17.5)	-
MTX_intol_ : MTX_tol_ (MISS)	17.3 (15.9-18.7) :15.2 (13.8-16.5)	0.0287
MTX-induced nausea: All others (Diary)	17.7 (16.2-19.3) :13.0 (11.6-14.4)	<0.0001

### P198 ADVERSE DRUG EVENTS IN THE TREATMENT OF CHILDHOOD RHEUMATIC AUTOIMMUNE DISEASES

#### Claudio A. Len, Manar A. Said, Gustavo G. B. Alves, Daniela G. Piotto, Maria Teresa Terreri

##### Uinit of Pediatric Rheumatology, Universidade Federal de São Paulo, São Paulo, Brazil

###### **Correspondence:** Claudio A. Len

**Introduction:** Autoimmune rheumatic diseases (AIRDs) need treatment that includes disease-modifying antirheumatic drugs, immunosuppressants and biological agents. However these medications can result in several adverse events (AEs) that vary in severity, requiring the use of other medications, dose reduction or even the suspension of the medication that caused the AE. AEs contribute to disease morbidity or even mortality in some cases. The severity of AEs depends on the medication doses, route of administration, use duration, drug association, or presence of other risk factors.

**Objectives:** To describe the AEs of all drugs used to treat AIRDs and to assess their severity, associated factors, medical procedures given and outcome of these events in pediatric patients with juvenile idiopathic arthritis (JIA), juvenile systemic lupus erythematosus (JSLE) and juvenile dermatomyositis (JDM).

**Methods:** retrospective descriptive and analytical study of a cohort of children and adolescents treated at a specialized center in pediatric rheumatology. The study evaluated all the AEs of the drugs reported in the patient's charts. The data were compiled through a specific questionnaire for each disease and then stored in excel spreadsheets. The associations between the aggravating factors for the AEs, such as patient age, dose, route of administration, and duration of medication use, were evaluated in patients treated with methotrexate (MTX) and glucocorticoids.

**Results:** Of 662 patients (391 JIA, 162 JSLE e 69 JDM) from our center, 547 patients were included and 951 AEs were recorded. MTX was the most common cause of AEs occurring in 63.3% of patients, followed by glucocorticoids. Other medications such as cyclophosphamide, cyclosporine, azathioprine, mycophenolate, leflunomide, chloroquine, etanercept, infliximab, adalimumab, tocilizumab, rituximab and human immunoglobulin also caused AEs. Abatacept did not cause any EA. Most patients with MTX-associated AEs had gastrointestinal intolerance and increased liver enzyme levels. When the AEs were stratified by disease, they were associated to MTX doses ≥ 0.6 mg / kg/week. Doses ≥ 0.5 mg/kg/day of glucocorticoids were associated with increased AEs. On the other hand, treatment with pulse therapy with corticosteroid did not induce AEs. Two patients (0.2%) had life-threatening AEs and 35 (3.7%) had severe AEs. No death related to medication was recorded. The most frequent medical procedures for the AEs were withdrawal or reduction of medication or change in route of administration, or use of other medications, apart from advice to the patients.

**Conclusion:** This study is unique because it is the largest retrospective study in the literature that focused on the AEs of the medications used during the treatment of AIRDs in real-life of children and adolescents at a specialized center. We concIuded that the AEs associated with the treatment of AIRDs are frequent and need constant monitoring and the associated factors to the occurrence of AEs such as age, dose, time of treatment, route of administration should be evaluated. This study showed that the occurrence of AEs may be related to the disease itself or other associated treatments.

**Disclosure of Interest**

None Declared

## Miscellaneous rheumatic diseases

### P199 PROTECTION AGAINST HEPATITIS B IN IMMUNOCOMPROMISED PEDIATRIC RHEUMATOLOGY AND GASTROENTEROLOGY PATIENTS

#### Najla Aljaberi^1^, Emily Smitherman^1^, Allen Watts^1^, Dana Dykes^2^, Jennifer Huggins^1^, Enas Ghulam^3^, Emily Smitherman^1^

##### ^1^Pediatric Rheumatology; ^2^Division of Gastroenterology, Hepatology and Nutrition, Cincinnati Children's Hospital Medical Center; ^3^2Department of Environmental Health, University of Cincinnati, Cincinnati, USA

###### **Correspondence:** Najla Aljaberi

**Introduction:** Hepatitis B infection remains a significant public health challenge, particularly for patients on chronic immunosuppressive therapy, due to a considerable mortality risk associated with hepatitis B reactivation.

**Objectives:** Our primary aim was to determine the burden of non-immunity against hepatitis B, provide insight into factors leading to lack of immunity against hepatitis B and establish the basis for the need for universal screening of these patients. Secondarily, we determined serologic response to a single hepatitis B vaccination booster.

**Methods:** Subjects are patients seen at the rheumatology and inflammatory bowel disease (IBD) clinics who are immunocompromised. We use a clinical algorithm as part of standard practice to check hepatitis B serology in immunocompromised patients, offer a booster vaccination if needed, and then repeat serology to determine the response. The results of anti-HBsAb are reported as positive, negative or indeterminate. Immunity is defined as a positive result for anti-HBsAb. An indeterminate or negative result for anti-HBsAb is non-immune. A retrospective chart review was performed to collect demographic and clinical factors as well as serology results. Descriptive statistics were calculated for all variables. R software was used to perform all analyses. For continuous variables, mean and standard deviation are reported, and comparisons were calculated using two-sample t-tests. For categorical variables, frequency and percentage are reported, and comparisons were calculated using chi-square tests.

**Results:** A total of 592 patient charts between the ages of 7 and 30 years old were reviewed. Non-immunity was defined as a negative or indeterminate hepatitis B surface antibody level. Out of the 592 patients, 70% were non-immune. The highest portion of non-immune patients were those between the ages of 16-20 years (p=0.005) (Table 1). There was no statistically significant difference between immune and non-immune patients with regards to diagnosis (p=0.32), age at the start of treatment (p=0.62), duration of treatment (p=0.054) or type of medications (except for anti B-cell). Seventy one percent of non-immune patients received a booster dose of hepatitis B vaccine and 68% of those re-screened responded appropriately to the vaccine. Of note, one patient was identified with a previously unknown hepatitis B infection.

**Conclusion:** A majority of patients were non-immune to hepatitis B. Lack of immunity is highest at 16-20 years. No association was found between age at the start of treatment or diagnosis and non-immunity. Being on anti B-cell was associated with non-immunity. A majority of patients responded appropriately to the booster vaccination. Results support screening of immunocompromised patients and further analysis may better delineate other factors and guide the screening protocol.

**Disclosure of Interest**

None Declared

**Table 1 (abstract P199).** Demographics and clinical characteristics by immune status

	Non-immune (n=414)	Immune (n=178)	P-value
Age (y)			
7-10 years	30 (7%)	20 (11%)	0.00
11-15 years	129 (31%)	39 (22%)	
16-20 years	188 (46%)	73 (41%)	
> 20 years	67 (16%)	46 (26%)	
Biologic*			
Anti TNF	310 (75%)	139 (78%)	0.95
Anti IL-6	42 (10%)	12 (7%)	0.44
Anti IL-1	16 (4%)	3 (2%)	0.29
Anti T-cell	35 (8%)	11 (6%)	0.60
Anti B-cell	14 (3%)	14 (8%)	0.00
Others	9 (2%)	1 (1%)	0.20

Continuous variables reported as mean (standard deviation). Categorical variables reported as frequency (percentage). P-values in bold are statistically significant

* This reflects the number of patients with history of medication use (% is reported out of total patients)

### P200 SYNOVIAL BIOPSY IN CHILDREN: A SINGLE CENTER EXPERIENCE

#### Sofia Carvalho Barreira^1,2^, Joana Silva-Dinis^1,2^, Patrícia Martins^1,2^, Emília Vitorino^3^, Raquel Campanilho-Marques^1,2^, João Eurico Fonseca^1,2^, Filipa Oliveira-Ramos^1,2^

##### ^1^Unidade de Reumatologia Pediátrica, Serviço de Reumatologia e Doenças Osseas Metabólicas, Hospital de Santa Maria, CHLN, Centro Académico de Medicina de Lisboa; ^2^Unidade de Investigação em Reumatologia, Instituto de Medicina Molecular, Faculdade de Medicina, Universidade de Lisboa, Centro Académico de Medicina de Lisboa; ^3^Serviço de Anatomia Patológica, Hospital de Santa Maria, CHLN, Centro Académico de Medicina de Lisboa, Lisboa, Portugal

###### **Correspondence:** Sofia Carvalho Barreira

**Introduction:** Synovial tissue biopsy is a safe and well tolerated way of studying multiple articular diseases. Many forms of juvenile idiopathic arthritis (JIA) present as a monoarthritis and synovial biopsy results might be useful to exclude other causes of arthritis namely infection or conditions with typical histological features such as synovial benign tumors or crystal-induced arthritis.

**Objectives:** To report our experience with synovial biopsies in pediatric age.

**Methods:** Descriptive study with a retrospective analysis of patients under 18 years-old performing a synovial biopsy in Hospital de Santa Maria since 2006. Demographic, clinical and anatomopathological data were collected.

**Results:** Since 2006, 35 children had 36 synovial biopsies (25 knees, five wrists, three hips, and three ankles). Twenty-one were male (60%), with a mean age of 11.1± 6.0 years. Indication for biopsy was monoarthritis in 27 patients (23 knee, two hip and two ankle arthritis), synovial cyst in six, knee oligoarthritis in one and a trochanteric lesion in one patient. Symptoms were present for a mean time of 8.2 ± 14.1 months. Procedures were performed by our Pediatric Rheumatology Unit in 19 cases (16 ultrasound-guided needle biopsies and 3 mini-arthroscopies), Orthopedic department in 10 cases (7 open biopsies and 3 arthroscopies), and Pediatric Surgery in seven cases. From the 27 patients with monoarthritis, 15 were classified as JIA (Table 1). In the remaining cases, histological aspects and culture confirmed septic arthritis in 5 patients, one patient had a pigmented villonodular synovitis and another one a moderate inflammatory infiltrate with important vessel congestion and hemosiderin deposits suggestive of hemarthrosis (consistent with the history of hemophilia). After magnetic resonance imaging, four patients with normal biopsies or with a mild to moderate inflammatory infiltrate had the diagnosis of a mechanical lesion. The patient with knee oligoarthritis had a chronic synovitis with extensive fat tissue that might correspond to a *lipoma arborescens*, which was also the diagnosis of the trochanteric lesion. Synovial cyst was confirmed in 5/6 cases, the other corresponding to a giant cell tumor of the tendon sheath. No major complications were reported for the procedures.

**Conclusion:** Synovial biopsy has been performed in children in our center over the last 12 years with good results. There is an increasing number of biopsies in the last few years, namely by less invasive US-guided and arthroscopic procedures, which are reliable for synovial tissue sampling and are replacing open biopsies. In children, synovial biopsy can give an important clue in the differential diagnosis of an initial monoarthritis.

**Trial registration identifying number:**

**Disclosure of Interest**

None Declared

**Table 1 (abstract P200).** Classification, anatomopathology and treatment of JIA patients

JIA category and histological findings	Number of patients	Synoviorthesis (n)	PDN (n)	MTX (n)	ETN (n)
Oligoarticular	10	10	1	2	0
ANA -	6	6	0	0	0
Chronic synovitis	3	3	0	0	0
Mild inflammatory infiltrate	3	3	0	0	0
ANA+	4	4	1	2	0
Chronic synovitis	4	4	1	2	0
ERA	4	4	3	3	3
Chronic synovitis	3	3	3	3	3
Mild inflammatory infiltrate	1	1	0	0	0
Psoriatic arthritis	1	1	0	1	0
Inflammatory infiltrate suggestive of septic arthritis	1	1	0	1	0

ANA: antinuclear antibodies, ERA: enthesitis-related arthritis, PDN: prednisolone, MTX: methotrexate, ETN: etanercept

### P201 DEMOGRAPHY AND TREATMENT OF COMPLEX REGIONAL PAIN SYNDROME AMONG CHILDREN

#### Martin L. Boesen^1^, Anne E. Christensen^2^

##### ^1^Pediatric Rheumatology, State Hospital Denmark, Copenhagen; ^2^Pediatric Rheumatology, Hans Christian Andersen Children's Hospital , Odense, Denmark

###### **Correspondence:** Martin L. Boesen

**Introduction:** Complex regional pain syndrome (CRPS) is a rare, but chronic and invalidating pain syndrome that affects both children and adults. Hans Christian Andersen Children’s Hospital (HCA) in the Region of Southern Denmark has a multidisciplinary inpatient programme for children affected by this disease.

**Objectives:** To describe the incidence of the disease, the demography, symptoms and psychosocial characteristics of the patients and to evaluate our treatment results.

**Methods:** Medical records of children referred to HCA for CRPS in an eleven-year period from 2004 to 2014 were investigated.

**Results:** Twenty-nine children were identified, equalling a mean incidence of 2,4 per 100.000 children annually. The patients were mainly ethnic Danish (97%) girls (90%) with a median age of 11 years [8 -14]. Their body mass index (BMI) was normal and most had been active athletes prior to disease development with normal social contacts but at admittance they were experiencing social isolation and had ceased all physical activities. Ad admittance the children were in severe pain, most frequently the condition involved a lower extremity (86 %) and 59% were depending on a wheelchair or crouches. The time from symptom appearance to diagnosis was 174 days [1-1280].

At discharge, after a median hospitalization of 14 days, all had remarkably pain reduction or were completely free of pain, and everyone was reintroduced to their social life. Using the affected extremity was achieved by 97% at discharge and by the end of the outpatient training program (1 month later) almost half could run and the rest improving. Setbacks were seen in 6 patients (21%), but all, except one, were managed solely at our outpatient clinic.

**Conclusion:** CRPS in children was a rare but invalidating disease. In our material it was most often seen in girls around the age of 11 years affecting a lower extremity. The children were prior to the disease physical active with a normal BMI. Despite the long diagnostic and referral delay great improvement was achieved with our in-patient treatment approach.

**Disclosure of Interest**

None Declared

### P202 TREATMENT AND OUTCOME OF CASTLEMAN DISEASE IN CHILDREN: A SINGLE-CENTRE EXPERIENCE

#### Grace Chiang^1^, Ali Amid^2^, Bo Ngan^3^, Brian M. Feldman^1,4^, Ronald M. Laxer^1,4^

##### ^1^Division of Rheumatology, The Hospital for Sick Children, Toronto; ^2^Division of Pediatric Hematology and Oncology, Children Hospital of Eastern Ontario, Ottawa; ^3^Division of Anatomical Pathology, The Hospital for Sick Children; ^4^Department of Paediatrics and Medicine, University of Toronto, Toronto, Canada

###### **Correspondence:** Grace Chiang

**Introduction:** Castleman disease (CD) is a rare and heterogeneous disease. The pathophysiology and factors contributing to disease outcome are poorly understood. CD is considered a lymphoproliferative disorder and classified into two anatomically-defined categories: multicentric (MCD) and unicentric (UCD). Patients with MCD usually present with florid systemic symptoms. The most promising and currently approved standard treatments for MCD patients are interleukin 6 (IL-6) receptor antibodies (tocilizumab and siltuximab) [1-3]. In contrast, patients with UCD seldom have systemic symptoms and can usually be treated by surgical resection of the affected lymph nodes (LNs). Current understanding of CD in paediatric patients is very limited [4-9] and our case series is the largest case series reported to date.

**Objectives:**

1. To describe the clinical presentation, diagnosis, treatment, and outcomes of paediatric patients with CD
2. To identify possible prognostic factors associated with outcome

**Methods:** This is a retrospective chart review study, including paediatric patients with a histologically-confirmed diagnosis of CD diagnosed over the last 20 years at The Hospital for Sick Children (SickKids), a tertiary care referral centre in Ontario, Canada.

**Results:** There were a total of 16 patients diagnosed with CD at SickKids in the past 20 years. Five had MCD and eleven had UCD. Of the MCD patients, 3 (60%) were male. The age at diagnosis ranged from 2 to 17 years old with the mean/median of 13.2/17 years old.  Two showed plasma cell subtype histologically and two belonged to the mixed type; one had an undefined subtype. All of the MCD patients tested negative for HIV and four patients tested for HHV8 were also negative. Two MCD patients diagnosed in 1999 died (one committed suicide and the other died of irreversible pulmonary fibrosis complicated by pulmonary haemorrhage and multiorgan failure). The three surviving MCD patients, including one with TAFRO (Thrombocytopenia, Ascities (anasarca), microcytic Anaemia, myeloFibrosis, Renal dysfunction and Organomegaly) syndrome, were diagnosed in recent years. All of them were diagnosed at the age of 17 years old with a duration of follow up of 0.6 to 10 months before their transition to adult care. They were treated with rituximab, tocilizumab and siltuximab respectively. The patient treated with Rituximab (4 weekly doses) responded very well clinically. However, she still has persistently high serum inflammatory markers. In contrast, both patients treated with anti-IL6 receptor antibodies achieved clinical and biochemical remission. There were 11 patients with UCD (54% female). The mean/median age at diagnosis was 11.27/11 years old. Only one had systemic symptoms; a 17 year-old girl who was also diagnosed with systemic lupus erythematosus. Among all patients with UCD, the cervical lymph nodes were the most commonly involved site (63%). The other lymph nodes affected included the axillary, mediastinal/thoracic, inguinal and intra-abdominal lymph nodes. The SickKids record showed one case of recurrence of UCD at the inguinal lymph node with successful clearance after repeated surgeries.

**Conclusion:** Patients with UCD generally had good outcome and seldom recurred after surgical removal of involved lymph nodes. They are usually systemically well apart from those associated with other autoimmune conditions [10]. The outcome of patients with MCD appeared to be favorable with the treatment of biologics like monoclonal anti-CD20 antibody and IL-6 receptor antibodies. Extension of data collection after patients being transferred to adult care is underway for studying protracted outcome.

**Disclosure of Interest**

None Declared

### P203 GAIT IN CHILDREN AND ADOLESCENTS WITH IDIOPATHIC MUSCULOSKELETAL PAIN

#### Maria da Conceição R. G. da Costa^1^, Hilda A. Oliveira^2^, Maria Teresa R. A. Terreri^1^, Jamil Natour^2^, Claudio A. Len^1^

##### ^1^Pediatic Rheumatology Unit, Department of Pediatrics; ^2^Rheumatology Division, Department of Medicine, Universidade Federal de São Paulo, São Paulo, Brazil

###### **Correspondence:** Maria da Conceição R. G. da Costa

**Introduction:** Idiopathic musculoskeletal pain syndrome (IMPS) is defined as the presence of intermittent pain in three or more body regions for at least three months, excluding organic diseases that could explain the symptoms. Its etiopathogenesis is multifactorial and includes physical factors such as lower central pain threshold, increased nociceptive receptor sensitivity and autonomic nervous system disorders, and emotional factors and social. In some patients, there may be other clinical findings related to pain, such as joint hypermobility and obesity, among others. Some points of the etiopathogenesis of IMPS are still unknown, among them, gait disorders.

**Objectives:** To study the gait of children and adolescents with IMPS by means of dynamic baropodometry.

**Methods:** 32 patients with IMPS and 32 healthy controls without pain, matched by age, sex, social class, and Body Mass Index (BMI) were enrolled. All were evaluated for pain intensity through the Visual Analogue Scale (VAS) and gait evaluation using dynamic baropodometry (FootWork Pro, AM cube, Gargas, France).

**Results:** The mean age of the IMSP group was 13.6 years (SD = 2.1, range 9.8 - 16.9) and the mean age of the control group was 13.5 years (SD = 2.0, range 9.6 - 16.5). The mean pain scale was 5.4 cm in the IMPS group. In gait, the mean right foot velocity of the IMPS group was significantly lower than that of the control group (*p* = 0.034), the time of the IMPS group step was significantly higher than that of the control group (*p* = 0.003) and the pace of the IMPS group was significantly lower than that of the control group (*p* = 0.001).

**Conclusion:** Children and adolescents with IMPS present gait alterations when evaluated by dynamic baropodometry.

**Disclosure of Interest**

None Declared

### P204 Withdrawn

### P205 IMPACT OF LONG TERM BENZYLPENICILLIN PROPHYLAXIS ON THE QUALITY OF LIFE OF PATIENTS WITH RHEUMATIC FEVER

#### Giancarla Di Landro, Francesca Minoia, Marta Torcoletti, Fabrizia Corona, Denise Pires Marafon, Carlo Agostoni, Giovanni Filocamo

##### Fondazione IRCCS Ca' Granda Ospedale Maggiore Policlinico, Milan, Italy

###### **Correspondence:** Giancarla Di Landro

**Introduction:** Rheumatic fever (RF) is an acute immune-mediated consequence of group A beta-hemolytic streptococcus (GABHS) infection. Rheumatic heart disease is the most threatening feature and the main cause of morbidity and mortality. Due to the high risk of recurrence in case of another streptococcal infection, RF patients need a long term secondary antibiotic prophylaxis in order to prevent a relapse. The use of intramuscular benzylpenicillin is still recommended worldwide as the most effective drug for secondary prophylaxis against GABHS. However its effect on health-related quality of life (HRQL) has never been investigated so far.

**Objectives:** To evaluate the impact of long term prophylaxis with intramuscular benzylpenicillin on the quality of life of patients with rheumatic fever

**Methods:** RF patients in secondary antibiotic prophylaxis for more than 1 year, referring to our pediatric rheumatologic center, and their parents were asked to fulfil a HRQL questionnaire: due to its high face validity the Pediatric Quality of Life (PedsQL 4.0) was chosen.

An age-matched group of healthy children and their parents were used as control samples considering the absence of a control group of patients with previous RF off secondary prophylaxis, due to ethical reasons.

The Global Score (GB) was calculated as the sum of all the items in the four domains evaluated (physical, emotional, social and school functioning); the Psychosocial Health Score (PHS) was the sum of all items related to emotional, social and school functioning domains. Comparisons between groups were analysed by means of Mann-Whitney U test.

**Results:** PedsQL questionnaire was sent to 28 families of RF patients and 18 controls respectively. The median age of RF patients was 9,8 years with a median duration of secondary prophylaxis of 20 months, and 9,4 years for controls . All the RF patients had a complete adherence to prophylaxis. No relapses were observed. The main results are shown in Table 1.

Patients with RF and their parents scored lower than controls in school scale domain. Patient with RF had a trend to lower levels of GB and also PHS compared to aged matches controls.

No differences were detected between parents and children in control groups

**Conclusion:** Although RF families have a good compliance to long term secondary prophylaxis with intramuscular benzylpenicillin, this prolonged treatment affect the quality of life of both children and parents.

The results of this study are preliminary, but if confirmed on a bigger sample, might stimulate a collaborative effort in reviewing the evidence on secondary prophylaxis in RF patients.

**Disclosure of Interest**

None Declared

**Table 1 (abstract P205).** See text for description

Age at visit (years)	PATIENTS GROUP (n=28)		CONTROL GROUP (n=18)		PATIENTS VS CONTROL GROUP		CHILDREN VS PARENTS	
	*Median (1st - 3rd quartile)*		*Median (1st - 3rd quartile)*		*(Mann Whitney U test)*		*(Mann Whitney U test)*	
	9.8 (9.0-14.0)		9.4 (7.6-10.0)		0,1			
QUESTIONARY	CHILDREN	PARENTS	CHILDREN	PARENTS	CHILDREN	PARENTS	PATIENTS GROUP	CONTROL GROUP
Physical scale	93.8 (78.2-93.8)	87.5 (65.7-96.9)	93.8 (90.7-96.9)	93.8 (87.5-98.5)	0.20	0.21	0,84	0.96
Emotional scale	77.5 (60.0-91.3)	67.5 (50.0-92.5)	85.0 (80.0-95.0)	80.0 (70.0-95.0)	0.30	0.24	0,55	0.56
Social scale	97.5 (86.3-100.0)	97.5 (80.0-100.0)	100.0 (92.5-100.0)	95.0 (72.5-100.0)	0.40	0.76	0,84	0.22
School scale	75.0 (57.5-85.0)	70.0 (60.0-86.3)	95.0 (80.0-97.5)	90.0 (80.0-100.0)	**0.0085**	**0.021**	0,98	0.93
Psychosocial heath summary score	83.4 (67.1-92.1)	76.7 (68.4-89.6)	93.3 (85.0-97.5)	86.7 (75.0-96.7)	**0.050**	0.17	0,84	0.51
TOTAL SCORE	87.0 (66.0-92.7)	78.8 (66.9-90.5)	94.6 (88.1-96.8)	90.2 (77.2-96.2)	**0.047**	0.11	0,7	0.57

### P206 OCULAR ROSACEA: AN UNDERDIAGNOSED DISEASE WITH HIGH MORBIDITY

#### Marco A. Fernandes^1^, Cláudia Arriaga^1^, Paula Estanqueiro^1^, Catarina Paiva^2^, Guilherme Castela^2^, Manuel Salgado^1^

##### ^1^Paediatric Rheumatology Unit; ^2^Paediatric Ophthalmology Unit, Centro Hospitalar Universitário de Coimbra (CHUC), E.P.E, Coimbra, Portugal

###### **Correspondence:** Marco A. Fernandes

**Introduction:** Chronic red eye is a common cause of paediatric rheumatology consultation to exclude a rheumatic disease. Paediatric ocular rosacea (POR) is a underdiagnosed disease, even by ophthalmologists. Since 1999 twelve cases of POR were diagnosed in our Paediatric Rheumatology Unit and Pediatric Ophthalmology Unit, the majority of which with significant diagnostic delay.

**Objectives:** To alert to clinical features, diagnosis and therapeutic approaches of POR.

**Methods:** Retrospective study. Patients were include based in POR Coimbra criteria: (> 3 criteria): a) Chronic or recurrent keratoconjunctivitis and/or red eye and/or photophobia; b) Chronic or recurrent blepharitis and/or hordela and/or chalazia; c) Eyelid telangiectasia; d) Features of cutaneous rosacea and/or periorifical dermatitis; e) Positive family history of cutaneous and/or ocular rosacea.

**Results:** Twelve children: seven boys and five girls. Mean age at diagnosis was 10,5 years (±4,0); with a median diagnostic delay of 2,5 years (IQR 3,9). Bilateral hyperaemia, ocular irritation and eyelid inflammation were found in all patients. Significant improvement occurred with a minimum of three months treatment with oral macrolides (erythromycin or clarithromycin) or topical azithromycin. Leukoma occurred in 6 children and spontaneous corneal perforation in 1.

**Conclusion:** POR is a disease with high morbidity. POR Coimbra criteria can improve its prompt recognition avoiding treatment delay. A high level of suspicion is needed in patients with red eye when chronic blepharoconjunctivitis and ocular irritation are reported, even in the absence of skin signs. Oral or topical treatment with macrolides or topical tetracyclin for at least 3 months has shown good results, avoiding significant sequelae such as blindness.

**References**

1. Arriaga C, Domingues M, Castela G, Salgado M. Pediatric ocular rosacea, a misdiagnosed disease with high morbidity: Proposed diagnostic criteria. World J Dermatol 2016;5(2):109.

2. Hammersmith KM. Blepharokeratoconjunctivitis in children. Cur Opin Ophthal 2015;26(4):301–5.

3. Gupta N, Dhawan A, Beri S, Dsouza P. Clinical spectrum of pediatric blepharokeratoconjunctivitis. J AAPOS. 2010 Dec;14(6):527-9. doi: 10.1016/j.jaapos.2010.09.013. Epub 2010 Nov 19.

4. Vieira AC, Mannis MJ. Ocular rosacea: Common and commonly missed. J Am Acad Dermatol. 2013;69(6).

5. Miguel AI, Salgado MB, Lisboa MS, Henriques F, Paiva MC, Castela GP. Pediatric Ocular Rosacea: 2 Cases. Eur J Ophthal. 2012;22(4):664–6.

**Disclosure of Interest**

None Declared

### P207 A CASE OF RECURRENT RHABDOMYOLYSIS TRIGGERED BY FEVER

#### Maria Francesca Gicchino, Giovanni Lodato, Alma N. Olivieri

##### Department of Women, Child and General and Specialistic Surgery, University of the Study of Campania Luigi Vanvitelli, Naples, Italy

###### **Correspondence:** Maria Francesca Gicchino

**Introduction:** Carnitine palmitoyltransferase II (CPTII) deficiency is a long-chain fatty-acid oxidation disorder. Carnitine palmitoyl transferase II is localized in the inner mitochondrial membrane and catalyzes the formation of acyl-coenzyme A. Three phenotypes of CPT II deficiency are known: a lethal neonatal form, a severe infantile hepatocardiomuscular form and a mild myopathic form. The disease follows an autosomal recessive mode of inheritance. Clinical features are attacks of muscle weakness, myalgia, pain and rhabdomyolysis with or without renal failure. Trigger factors are fever, prolonged exercise and exposure to cold.The severity of attacks is highly variable and some of these attacks may be complicated by acute renal failure . The CPTII deficiency is the most common cause of hereditary myoglobinuria and the most frequent disorder of lipid metabolism affecting skeletal muscle.

**Objectives:** To emphasize considering CPT II deficiency in a patient with recurrent episodes of rhabdomyolysis triggered by fever.

**Methods:** A six years old child came to our department because of fever, myalgia and generalized muscular weakness. She had a personal history of diffuse muscular pain and generalized weakness triggered by fever . Before coming to our observation both Duchenne Muscular Dystrophy and Becker Muscular Dystrophy were excluded. When she was admitted to our department she was unable to walk and presented generalized weakness, muscular pain, and fever. Laboratory text showed an increase of serum creatine kinase levels (15,400 U/L, normal value < 190U/L), aspartate aminotransferase (586 U/L, normal value <30U/L), alanine aminotransferase (382 U/L, normal value< 30U/L). Complete blood count and renal function tests were in normal range.  Her urine was brown-colored, while urinalysis was negative for red blood cells. She was managed with intravenous hydration with immediate clinical and laboratory improvements. The recurrence of severe episodes of rhabdomyolysis and normal creatine kinase value between attacks suggested Carnitine palmioyltransferase II (CPTII) deficiency .

**Results:** The genetic analysis of the CPT II gene revealed homozygosity for the p.Ser1133Leu mutation, so CPTII deficiency was diagnosed. A low- fat and rich in complex carbohydrates diet was prescribed. She was advised to avoid physical exertion and exposure to cold and to receive additional carbohydrates before exercise and during illness.

**Conclusion:** CPT II deficiency should be suspected in children with recurrent rhabdomyolysis triggered by fever, exercise and exposure to cold . Molecular genetic analysis of the related gene should be performed to confirm the diagnosis. A prompt diagnosis and a careful management are essential to prevent further episodes of rhabdomyolysis. Consent to publish had been obtained.

**Disclosure of Interest**

None Declared

### P208 DETERMINATION OF DIAGNOSTIC VALUE (VALIDITY) LEUKOCYTE ESTERASE (URINE DIPSTICK STRIP) IN THE DIAGNOSIS OF SEPTIC ARTHRITIS

#### Mehrnoush Hassas Yeganeh, Reza Shiari, Khosro Rahmani, Vadood Javadi Parvaneh

##### Paediatric Rheumatology, CHILDRAN HOSPITAL, Tehran, Iran, Islamic Republic Of

###### **Correspondence:** Mehrnoush Hassas Yeganeh

**Introduction:** Septic arthritis is one of the most deadly diseases that the absence of rapid diagnosis and initiation of treatment can cause irreparable effects. There are several diagnostic features of the disease, which in some cases takes time and also don’t have a high degree of confidence. So finding new rapid and affordable methods to diagnose septic arthritis is very important.

**Objectives:** This study aimed to determine the diagnostic value of leukocyte esterase (urine dipstick bar) is designed for the diagnosis of septic arthritis.

**Methods:** Diagnostic study was carried out on 47 children with suspected septic arthritis admitted to Mofid children’s hospital on 2014-15. After obtaining the consent of the parents and child, joints involved aspirated. Aspirated liquid analyzed for bacterial infection culture, smear, white blood cell count and biochemical. The joint fluid aspiration was evaluated for leukocyte esterase by using the urinary Dipstick bar immediately. Demographic data, clinical and laboratory findings were recorded in questionnaire form. The data were entered in SPSS software version 21.

**Results:** In the present study joint fluid cultures were positive in 45% that female to male ratio was 1:4.3. Using positive cultures, the urine leukocyte esterase strips had a sensitivity of 95.2%, specificity of 100%, PPV of 100%, and NPV of 96.3%. When using a synovial fluid white blood cell greater than 50000 WBC/mL, these values were 92.3, 76.5%, 60.0%, and 96.3%, respectively. ROC curve analysis showed the area under the curve for urine leukocyte esterase strip was more than white blood cells.

**Conclusion:** This study showed that the use of leukocyte esterase strip is rapid, reliable and effective method for diagnosis of septic arthritis in children.

**Disclosure of Interest**

None Declared

### P209 VENOUS MALFORMATIONS MASQUERADING AS AN EPISODIC KNEE MONO-ARTHRITIS

#### Ruby Haviv^1^, Veronica Moshe^2^, Eugene Kotz^3^, Uri Rimon^4^, Yosef Uziel^5^

##### ^1^Pediatric Rheumatology Unit, Department of Pediatrics, Meir Medical Center, Sackler School of Medicine; ^2^Pediatric Rheumatology Unit, Department of Pediatrics; ^3^Department of Clinical Imaging, Meir Medical Center, Kfar-Saba; ^4^Department of Clinical Imaging, Sheba Medical Center, Ramat Gan; ^5^Pediatric Rheumatology Unit, Department of Pediatrics, Meir Medical Center, Sackler School of Medicine, Tel Aviv University, Kfar-Saba, Israel

###### **Correspondence:** Ruby Haviv

**Introduction:** Episodic monoarthritis is not a common presentation in rheumatology clinics. It may be a manifestation of mechanical, hematological, or metabolic disease, or numerous inflammatory disorders. Eight case reports and case series have placed different vascular malformations as an optional diagnosis for episodic or chronic knee swelling. Intra-articular venous malformations are very rare. Some of these lesions may expand abruptly following trauma or hormonal changes, presenting as an episodic joint swelling, and be misdiagnosed as an infection or juvenile idiopathic arthritis (JIA).

**Objectives:** To characterize patients with venous malformations of the knee and offer key clinical features leading to diagnosis.

**Methods:** Patients' medical and imaging files were reviewed and analyzed.

**Results:** Within a period of 1 year, four patients were diagnosed with venous malformations of the left knee in the Pediatric Rheumatology Unit of Meir Medical Center in Israel. Patients' ages at diagnosis were 3 to 8.5-years-old, the youngest was the only female. Time to diagnosis since first visit in the rheumatology clinic was 7 months to 6.5 years. Symptoms began during the first or second year of life, and consisted primarily of episodic swelling of the knee, along with tenderness and limping, but without fever or any extra-articular manifestation (including uveitis). Some episodes began after minor local trauma and lasted days to months. Physical examination revealed swollen and mildly tender left knee, and mild limitation of the range of motion. Two patients had vascular skin lesions on their left lower limb. Laboratory workup was unrevealing, in general, with the exception of highly elevated C-reactive protein in the female patient, during a single episode (she was later found as heterozygote for the V726A mutation in the MEFV gene). Anti-inflammatory drugs (including colchicine in the girl) and intra-articular corticosteroid injections in 3 patients had no measurable effect on the course of the disease. Bloody fluid was aspirated from the knees of two patients. Differential diagnosis included JIA, familial Mediterranean fever, anatomic disorders of the knee and pigmented villo-nodular synovitis. As radiographs, bone scintigraphy and ultrasonography revealed inconsistent findings, MRI scans were performed, and diagnosis was finally made. At the time of writing this report, two patients were already successfully treated with sclerotherapy.

**Conclusion:** Intra-articular vascular malformation should be suspected in patients with dermal vascular malformations, episodic monoarthritis, without extra-articular involvement, with normal inflammatory markers, history of aspiration of bloody synovial fluid, or when anti-inflammatory treatment fails. MRI is the diagnostic modality of choice, revealing high T2 and low T1 signal intensity and patchy enhancement after intravenous contrast medium injection. Treatment of the vascular lesion depends on the type of malformation and the flow within it and can include excisional surgery, embolization, sclerotherapy and irradiation.

**Disclosure of Interest**

None Declared

### P210 WISKOTT-ALDRICH SYNDROME WITH A NOVEL MUTATION PRESENTING WITH FEBRILE NEUTROPHILIC DERMATOSES: AN AUTOINFLAMMATION INSTEAD OF AUTOIMMUNITY

#### Assunta Chi Hang Ho^1^, Chung Mo Chow^1^, Po Ki Ho^2^, Ting Fan Leung^1^

##### ^1^Paediatrics , PRINCE OF WALES HOSPITAL, CHINESE UNIVERSITY OF HONG KONG; ^2^Paediatrics, Queen Elizabeth Hospital, Hong Kong, Hong Kong

###### **Correspondence:** Assunta Chi Hang Ho

**Introduction:** Wiskott-Aldrich Syndrome (WAS, OMIM#301000) is a X-linked primary immunodeficiency syndrome. Classical triad includes eczema, micro-thrombocytopenia and recurrent infections. Making the diagnosis may be challenging since patients may not always present with the classical symptoms. Presented here is a young boy with known thrombocytopenia since neonatal period. The diagnosis of WAS was only established after the development of recurrent symptoms suggestive of autoinflammation. The presentation of autoinflammation instead of the more commonly reported autoimmunity^1^ may be related to his novel mutation of the WAS protein gene.

**Objectives:** not applicable

**Methods:** not applicable

**Results:** CF first presented at 3 weeks old with PR bleeding. He was referred to the paediatric haematologist due to refractory thrombocytopenia despite IVIg and platelet transfusion. The first Mean Platelet Volume (MPV) was 7.4 fl (7-10) before any treatment. Despite extensive investigation, the cause for thrombocytopenia remained uncertain. At 5 months he had mild ear discharge with good response to topical antibiotics. The bleeding episodes continued.

At 7 months old CF started to have pyrexia of unknown origin, recurrent rashes and polyarthritis. The rashes were in various forms, including erythematous maculopapular, urticarial and extensive livedo reticularis(fig). He also had marked inflammatory response (elevated ESR and CRP, leukocytosis, hypoalbuminaemia). All sepsis screen was negative. Bone marrow examination were repeated for total 3 times and were negative for malignancy. Skin biopsy showed neutrophilic dermatoses without vasculitis (perivascular and interstitial neutrophil infiltration, focal extravasated red cells but not much nuclear dust, no vasculitis). He had positive ANA only. His clinical picture was suggestive of an autoinflammation though he did not fit into a single entity. Systemic corticosteroid was started. However he only responded to very high dose (2mg/kg/day). Fever recurred whenever tapering was attempted.

Taking together the known thrombocytopenia, WAS was suspected. Genetic test confirmed a novel insertion mutation causing a frameshift mutation (T insertion at nucleotide position 431-2 in exon 4, causing a frameshift start at Lysine 144 and a stop codon (TGA) created at position 168(K144fsX168)). 2 months later he underwent haematopoeitic stem cell transplant.

**Conclusion:** In this case the diagnosis of WAS was only suspected after the onset of autoinflammation as the platelet size was normal. Neutrophilic dermatoses (ND) may be considered a continuum with other autoinflammatory conditions^2^, especially in patients with mark systemic symptoms like in our case. Having said that ND is also reported in autoimmune diseases like SLE. Our patient’s presentation may relate to his particular WAS protein mutation.

Early recognition of this possible association could help making the diagnosis earlier and hence definitive treatment. Informed consent to publish had been obtained from the parents.

**References**

1 Schurman SH, Candotti F. Autoimmunity in Wiskott-Aldrich Syndrome. Curr Opin Rheumatol(2003)15:446-453

2 Navarini AV, Satoh TK, French LE. Neutrophilic dermatoses and autoinflammatory diseases with skin involvement-innate immune disorders. Semin Immunopathol(2016)38:45-56

**Disclosure of Interest**

None Declared

## Systemic JIA - MAS II

### P211 WHOLE BLOOD PHOSPHORYLATED STAT1 LEVELS IN PATIENTS WITH ACTIVE MACROPHAGE ACTIVATION SYNDROME AND SECONDARY HEMOPHAGOCYTIC LYMPHOHISTIOCYTOSIS

#### Antonia Pascarella, Claudia Bracaglia, Emiliano Marasco, Ivan Caiello, GianMarco Moneta, Giulia Marucci, Manuela Pardeo, Fabrizio De Bedenetti, Giusi Prencipe

##### Division of Rheumatology, Ospedale Pediatrico Bambino Gesù, Rome, Italy

###### **Correspondence:** Antonia Pascarella

**Introduction:** A large body of evidence demonstrates the pivotal role of interferon gamma (IFNγ) in the pathogenesis of secondary hemophagocytic lymphohistiocytosis (sHLH) and macrophage activation syndrome (MAS). IFNγ is a key endogenous activator of macrophages and exerts its biological activities by phosphorylation of the transcription factor Signal transducer and activator of transcription 1 (STAT1).

**Objectives:** In this study, we aimed to investigate whether the phosphorylation status of STAT1 in whole blood cells represents a good biomarker for the identification of patients at early stages of MAS/ sHLH.

**Methods:** Whole blood samples from patients with suspected untreated MAS/sHLH (n=7) and suspected treated (glucocorticoids) MAS/sHLH (n=9) were collected prospectively. As controls, whole blood samples from patients with active systemic Juvenile Idiopathic Arthritis (sJIA) without MAS at sampling (n=6) and healthy subjects (HS, n=7) were used. Fresh whole blood cells were left unstimulated or stimulated with different concentrations of IFNγ (0.01, 0.1, 1, 10 ng/ml) for 10 minutes. The intracellular phosphorylation levels of Tyrosine (701) STAT1 (pSTAT1) were evaluated by flow cytometry. Results have been expressed as Delta mean fluorescence intensity (MFI), calculated by subtracting the MFI of cells stained with isotype control antibody (Ab) from that stained with anti-pSTAT1 Ab. Anti CD3, CD14 and CD16 staining was performed to discriminate the monocyte, neutrophil, natural killer- and T- cell subpopulations. In addition, western blot (WB) analyses were carried out on unstimulated whole blood cell lysates, in order to further evaluate the basal levels of pSTAT1 and total STAT1.

**Results:** In both treated and untreated MAS/sHLH patients, flow cytometric analyses showed no significant differences in pSTAT1 levels in unstimulated monocyte, neutrophil, natural killer and T cell subpopulations, compared to sJIA and healthy subjects. Interestingly, we found that, compared to sJIA and healthy subjects, in patients with untreated MAS/sHLH, pSTAT1 levels were significantly higher in monocytes (p<0,01 Vs HS, p<0,05 Vs sJIA and p<0,01 Vs HS, p<0,05 Vs sJIA, for stimulation with 1 and 10 ng/ml of IFNg respectively) and neutrophils (p<0,05 Vs HS, p<0,05 Vs sJIA and p<0,01 Vs HS, p<0,01 Vs sJIA) stimulated with the higher concentrations of IFNγ (1 and 10 ng/ml). In contrast, we did not find differences in the levels of pSTAT1 observed in stimulated monocytes and neutrophils from treated MAS/sHLH patients or those observed in cells from active sJIA and healthy subjects. In order to further evaluate the phosphorylation status of STAT1, we also performed WB analyses on patient whole blood cell lysates. We found that pSTAT1 levels were markedly higher in untreated MAS/sHLH patients and also in treated MAS/sHLH patients, compared to active sJIA patients without MAS.

**Conclusion:** Our results demonstrate that the combined evaluation of pSTAT1 levels by flow cytometry in monocytes and neutrophils stimulated with high doses of IFNγ and by WB in fresh whole blood cell lysates show high levels of pSTAT1 and might contribute to the identification of patients at early stages of MAS/sHLH. WB might be more sensitive and technically simpler approach, although it takes at least 2 days. In addition, our results further support the involvement of IFNγ in the development of the diseases, as suggested by the increased phosphorylated STAT1 levels exclusively in patients with active MAS/sHLH and not in patients with active sJIA.

**Disclosure of Interest**

A. Pascarella: None Declared, C. Bracaglia: None Declared, E. Marasco: None Declared, I. Caiello: None Declared, G. Moneta: None Declared, G. Marucci: None Declared, M. Pardeo: None Declared, F. De Bedenetti Grant / Research Support from: BMS, Pfizer, Abbvie, Novartis, Novimmune, Roche, SOBI, Sanofi, UBC, Consultant for: Roche, Novartis, Novimmune, SOBI, G. Prencipe: None Declared

### P212 SYSTEMIC ONSET JUVENILE IDIOPATHIC ARTHRITIS: A SINGLE CENTER EXPERIENCE

#### Erdal Sag, Berna Uzunoglu, Fatma Bal, H. Emine Sonmez, Selcan Demir, Yelda Bilginer, Seza Ozen

##### Division of Pediatric Rheumatology, Hacettepe University Faculty of Medicine, Department of Pediatrics, Ankara, Turkey

###### **Correspondence:** Erdal Sag

**Introduction:** Systemic onset juvenile idiopathic arthritis (sJIA) is an autoinflammatory disease, characterized by typical fever and systemic features such as arthritis, rash, lymphadenopathy, hepatosplenomegaly and serositis.

**Objectives:** In this study, we aimed to figure out the clinical and laboratory features, treatment responses and prognosis of sJIA patients from a tertiary rheumatology center.

**Methods:** Patients whom were classified as sJIA due to ILAR criteria and being followed at Hacettepe University Department of Pediatric Rheumatology between 2010-2017 were included in this study. Patient files were analyzed retrospectively for clinical and laboratory features.

**Results:** 75 sJIA (%56 male) patients were included. The mean age at diagnosis was 6,45±4,80 yrs. At the time of diagnosis all of the patients had typical fever while 78,7% of them had arthritis, 66,2% of them had rash, 37,3% of them had arthralgia, 28,4% of them had hepatosplenomegaly (HSM), 20,3% of them had lymphadenopathy, and 17,6% of them had serositis. 24% of the patients had MAS (macrophage activation syndrome) features at the time of diagnosis while in total 36% of the patients had at least one MAS attack anytime on disease course. Patients who had MAS at the time of diagnosis were older than other sJIA patients (8,9 vs 5,3 yrs; p=0,004), they had less WBC (7166 vs 17864/mm^3^ p<0,001) and platelet (179000 vs 689000/mm^3^ p<0,001) count, and had a lower Erythrocyte Sedimentation Rate (ESR: 12 vs 63/hr, p<0,001) as expected.

These group of patients had more serositis (%44,4 vs %7,5; p<0,001) and hepatosplenomegaly (%55,6 vs %18,9; p= 0.004) however there was no significant difference in terms of arthritis, arthralgia, rash and lymphadenopathy.

All of the patients were treated with NSAID or corticosteroids at the beginning of the disease. 20% of the patients reached remission with NSAID, corticosteroid or DMARDs however rest of the patients needed at least one biologic drug.

Anakinra was the most common first-line biologic treatment choice (n=45). Among the patients whom remission was achieved, 44% of them were treated with anakinra, 12% with canakinumab, 10,7% with tocilizumab. 46% of the patients had recurrent attacks however 54% had one attack (26% monophasic, 28% persistant). 18,7% of the patients had polyarticular joint involvement during the disease course. There was no significant difference between the patients with polyphasic and monophasic course in terms of laboratory and clinical features other than HSM (%42,4 vs %17,1; p=0,016 respectively).

40% of the patients are being followed without any treatment after remission was achieved. Five out of 8 patients who had remission with tocilizumab had polyarticular joint involvement, thus it can be speculated that anti IL-6 treatment is very beneficial in this specific group of patients.

**Conclusion:** sJIA is an important disease with high risk of morbidity and mortality. As our center is one of the most important tertiary referral rheumatology center, we expect to see higher MAS incidence than the previous reported sJIA series. Although 1/5 of the patients achieved remission without any biological agent, they are still the most beneficial treatment in most of the sJIA patients. Anti-IL1 drugs are the mostly preferred treatment choice and they have the highest success rate. Anti-IL-6 agents are very efficient in patients with systemic onset polyarticular course.

**Disclosure of Interest**

None Declared

### P213 SOLUBLE CD163, A UNIQUE BIOMARKER TO EVALUATE THE DISEASE ACTIVITY, EXHIBITS MACROPHAGE ACTIVATION IN SYSTEMIC JUVENILE IDIOPATHIC ARTHRITIS

#### Naoto Sakumura^1^, Masaki Shimizu^1^, Mao Mizuta^1^, Natsumi Inoue^1^, Yasuo Nakagishi^2^, Akihiro Yachie^1^

##### ^1^Department of Pediatrics, School of Medicine, Institute of Medical, Pharmaceutical and Health Sciences, Kanazawa University, Kanazawa; ^2^Department of Pediatric Rheumatology, Hyogo Prefectural Kobe Children's Hospital, Kobe, Japan

###### **Correspondence:** Naoto Sakumura

**Introduction:** Serum soluble CD163 (sCD163) level is a valuable diagnostic marker in hemophagocytic lymphohistiocytosis (HLH) and systemic juvenile idiopathic arthritis (s-JIA) associated macrophage activation syndrome (MAS). However, it is still unclear whether serum sCD163 level is useful for the assessment of disease activity of s-JIA. Furthermore, it is unknown whether sCD163 expression is modified by interleukin (IL)-6 blocking.

**Objectives:** This study aims to investigate the clinical significance of serum sCD163 levels as a predictor of the disease activity of s-JIA.

**Methods:** In this study, we examined 63 patients with s-JIA, four with Epstein–Barr virus-induced HLH (EBV-HLH), and seven with Kawasaki disease (KD), along with 14 healthy controls. We quantified serum cytokine levels (sCD163, neopterin, IL-18, IL-6) by enzyme-linked immunosorbent assay and compared the results with the clinical features of s-JIA.

**Results:** Serum sCD163 levels were significantly elevated in patients with s-JIA associated MAS and EBV-HLH compared to those in patients with acute-phase s-JIA and KD. In addition, serum sCD163 levels profoundly increased with the progress of MAS and correlated positively with the disease activity of s-JIA, even in patients receiving tocilizumab. Furthermore, serum sCD163 levels significantly decreased in the inactive phase compared to those in the active phase and normalized in remission.

**Conclusion:** The correlation between macrophage activation and serum sCD163 levels might be a unique indicator of the disease activity and a potential diagnostic laboratory criterion for clinical remission in patients with s-JIA, including those receiving tocilizumab.

**Disclosure of Interest**

None Declared

### P214 SEVERE LUNG DISEASE ASSOCIATED WITH SYSTEMIC JUVENILE IDIOPATHIC ARTHRITIS – THE CINCINNATI EXPERIENCE

#### Grant S. Schulert^1,2^, Chris Towe^1,2^, Ndate Fall^1,2^, Shima Yasin^1,2^, Brenna Carey^1,2^, Bruce Trapnell^1,2^, Kathryn Wikenheiser-Brokamp^1,2^, Jason Woods^1,2^, Jennifer Huggins^1,2^, Esi Morgan^1,2^, Tracy Ting^1,2^, William Bernal^3^, Elizabeth Mellins^4^, Mona Riskalla^5^, Matthew L. Stoll^6^, Alexei A. Grom^1,2^

##### ^1^University of Cincinnati College of Medicine; ^2^Cincinnati Children's Hospital Medical Center, Cincinnati; ^3^University of California San Francisco, San Francisco; ^4^Stanford University, Stanford; ^5^University of Minnesota, Minneapolis; ^6^University of Alabama Birmingham, Birmingham, USA

###### **Correspondence:** Grant S. Schulert

**Introduction:** Severe lung disease including pulmonary alveolar proteinosis (PAP) and interstitial lung disease is an increasingly recognized complication of Systemic Juvenile Idiopathic Arthritis (SJIA). The inflammatory features and patterns of this lung disease are poorly described.

**Objectives:** To describe the experience of SJIA-associated lung disease in a large US referral center as well as novel immunological and pathophysiological findings.

**Methods:** Informed consent was obtained from all patients and/or their legal guardians through protocols approved by the CCHMC IRB. Clinical data was abstracted from medical records, and cellular, biomarker, and genomic analysis performed as indicated.

**Results:** Since 2010, we have evaluated 12 patients with SJIA-associated lung disease. The number of new patients evaluated has markedly increased during this timeframe, with 5 newly diagnosed patients evaluated in 2017 alone. Considering only local patients, the incidence of severe lung disease in SJIA patients may be as high as 5-10%.

Most patients with SJIA-associated lung disease had refractory disease despite treatment with multiple biologics, predominantly systemic features, and history of overt or subclinical macrophage activation syndrome (MAS). Patients with active SJIA and lung disease also had markedly higher serum IL-18 levels than patients without lung disease (median 45,854ng/ml vs 7,194, p<0.01), levels comparable to that seen in MAS.

Chest imaging (CT and MRI) findings included diffuse ground-glass opacities, subpleural reticulation, interlobular septal thickening, and lymphadenopathy. F-18-FDG positron tomography was also performed in one patient, showing multifocal uptake consistent with an inflammatory process.

Five patients had open lung biopsy. While there was substantial variability in findings, typical findings included patchy but often extensive lesions histologically comprised of lymphoplasmacytic (CD4+ and CD8+) infiltrates and mixed features of PAP and endogenous lipoid pneumonia. Air spaces were filled with granular lipoproteineous material typical for PAP, foamy macrophages and cholesterol clefts. Pleural and interlobular septal collagenous fibrosisas well as vasculopathy was prominent is some cases.

PAP results from disordered surfactant homeostasis, often secondary to defective alveolar macrophage GM-CSF signaling. However, SJIA-associated lung disease patients had several features atypical for GM-CSF signaling related PAP. Bronchoalveolar lavage (BAL) fluid often did not reflect biopsy findings and typically did not contain the abundant proteinaceous material seen in PAP with only a minority of macrophages being lipid-laden (3-25%). BAL fluid did contain high levels of IFNg-induced chemokines CXCL9-11, observed in serum and tissue during MAS. Whole exome sequencing was performed on seven patients, which failed to reveal any variants associated with familial PAP. Finally, isolated alveolar macrophages showed normal STAT5 phosphorylation in response to GM-CSF, suggesting that this signaling axis is functional.

**Conclusion:** Severe lung disease is increasingly detected in children with SJIA, particularly in association with MAS. Pathologic hallmarks of disease are often absent from BAL fluid, supporting utility of lung biopsy. This entity has distinct clinical and immunologic features from other disorders including PAP, and represents an uncharacterized inflammatory lung disease.

**Disclosure of Interest**

G. Schulert: None Declared, C. Towe: None Declared, N. Fall: None Declared, S. Yasin: None Declared, B. Carey: None Declared, B. Trapnell: None Declared, K. Wikenheiser-Brokamp: None Declared, J. Woods: None Declared, J. Huggins: None Declared, E. Morgan: None Declared, T. Ting: None Declared, W. Bernal: None Declared, E. Mellins: None Declared, M. Riskalla: None Declared, M. Stoll Consultant for: Novartis, A. Grom Grant / Research Support from: Novartis, NovImmune, AB2Bio

### P215 CLINICAL SIGNIFICANCE OF SERUM INTERLEUKIN-18 LEVEL FOR THE DIAGNOSIS AND THE ASSESSMENT OF DISEASE ACTIVITY AND REMISSION IN SYSTEMIC JUVENILE IDIOPATHIC ARTHRITIS

#### Masaki Shimizu^1^, Mao Mizuta^1^, Natsumi Inoue^1^, Yasuo Nakagishi^2^, Naomi Iwata^3^, Ryuhei Yasuoka^3^, Akihiro Yachie^1^

##### ^1^Department of Pediarics, SCHOOL OF MEDICINE, INSTITUTE OF MEDICAL PHARMACEUTICAL AND HEALTH SCIENCES, KANAZAWA UNIVERSITY, Kanazawa; ^2^Department of Pediaric Rheumatology, Hyogo Prefectural Kobe Children’s Hospital, Kobe; ^3^Department of Infection and Immunology, Aichi children’s Health and Medical Center, Obu, Japan

###### **Correspondence:** Masaki Shimizu

**Introduction:** Previous reports showed serum IL-18 level is significantly elevated in active phase of systemic juvenile idiopathic arthritis (s-JIA). However, it is still unknown whether serum IL-18 level is a useful biomarker for the diagnosis of s-JIA and the differentiation from other inflammatory diseases. Furthermore, there are few long-term follow-up studies to examine whether serum IL-18 level is useful for the assessment of the disease activity and remission in s-JIA.

**Objectives:** To investigate clinical significance of serum interleukin (IL)-18 level for the diagnosis of systemic juvenile idiopathic arthritis (s-JIA) and clinical usefulness of serum IL-18 level for the assessment of the disease activity and remission in s-JIA.

**Methods:** One hundred sixteen patients with s-JIA, 128 other inflammatory diseases and 20 healthy controls were analyzed. Longitudinally measurement of serum IL-18 levels from active phase through remission or relapse were performed in forty one patients with s-JIA. Serum IL-18 levels were quantified by enzyme-linked immunosorbent assay. Results were compared with clinical features and disease course of s-JIA.

**Results:** Serum IL-18 levels in s-JIA were significantly elevated compared to other inflammatory diseases. The cutoff value of serum IL-18 levels for the differentiation s-JIA from other diseases was 4560 pg/ml, with sensitivity of 99.14 %, and specificity of 96.15 %. Serum IL-18 levels were correlated positively with the following measures of disease activity: ferritin, asparartate transaminase and lactate dehydrogenase. The longitudinal kinetics of serum IL-18 levels from active phase to remission or relapse were closely correlated with disease activity of s-JIA.

**Conclusion:** Serum IL-18 levels >4560 pg/ml might be useful for the diagnosis of s-JIA. Furthermore, serum interleukin-18 level reflects the disease activity of s-JIA. Thus, serum IL-18 level might be a promising biomarker for the diagnosis and the assessment of disease activity of s-JIA.

**Disclosure of Interest**

None Declared

### P216 CLASSIFICATION CRITERIA IN SYSTEMIC-ONSET JUVENILE IDIOPATHIC ARTHRITIS (SJIA): EVALUATION OF THE YAMAGUCHI AND ILAR ‘MODIFIED’ CRITERIA IN A SJIA POPULATION.

#### Katerina Theodoropoulou^1^, K. Bouayed^2^, V. Hentgen^3^, I. Kone-Paut^4^, C Wouters^5^, F Hofer^6^, E Cannizzaro^7^, E Merlin^8^, C Ballot^9^, D Kaiser^10^, P Pillet^11^, A Woerner^12^, V Devauchelle^13^, U Meinzer^14^, K Retornaz^15^, M Hofer^1^

##### ^1^Pediatric Rheumatology, CHUV, Lausanne, Switzerland; ^2^Hôpital d’Enfants Ibnou Rochd Casablanca, Casablanca, Morocco; ^3^CH de Versailles – Hôpital André Mignot; ^4^Centre Hospitalier Universitaire Kremlin-Bicêtre, Paris, France; ^5^UZ Leuven, Leuven, Netherlands; ^6^CHUV, Lausanne; ^7^Kinderspital Zürich, Zürich, Switzerland; ^8^Centre Hospitalier Universitaire de Clermont-Ferrand, Clermont-Ferrand; ^9^CHRU Besançon, Besançon, France; ^10^Luzerner Kantonspital, Lucerne, Switzerland; ^11^CHU de Bordeaux, Bordeaux, France; ^12^Universitäts-Kinderspital beider Basel, Basel, Switzerland; ^13^CHRU Best, Brest; ^14^Hôpital Robert Debré, Paris; ^15^CHU Marseille-Hôpital Nord, Marseille, France

###### **Correspondence:** Katerina Theodoropoulou

**Introduction:** Early diagnosis and treatment of systemic-onset juvenile idiopathic arthritis (SJIA) are crucial in order to avoid long-term complications. However, the existing International League of Associations for Rheumatology (ILAR) classification criteria for SJIA have been criticized. A substantial percentage of SJIA patients fail to fulfill them, mostly because of the absence of arthritis, or due to the exclusion criteria requiring regular testing of HLAB27, which is often not performed in real-life clinical practice.

**Objectives:** To evaluate and compare the existing Yamaguchi and ILAR classification criteria as well as a new set of ILAR ‘modified’ criteria without HLAB27 testing, in an international cohort of SJIA patients.

**Methods:** This is a multicentre, retrospective and inception cohort study, through the international platform JIRcohorte. The existing ILAR classification criteria, a new set of ‘modified’ ILAR classification not including HLAB27 testing and the Yamaguchi criteria were applied in SJIA patients followed in one of the participating centres in Switzerland, France, Belgium and Morocco. 50 SJIA patients with no more than one missing criteria in the database were enrolled (sex ratio 1:1) and compared to a control group of 46 patients with other autoinflammatory and autoimmune diseases presenting with systemic and articular manifestations.

**Results:** Of 50 SJIA patients enrolled, only 56% (28/50) fulfilled the ILAR classification criteria for SJIA, 14% (7/50) were classified in another JIA category and the remaining 30% (15/50) were unclassified. Among the 22 patients who failed to fulfill the ILAR criteria, 4 (18%) had no arthritis, 9 (41%) had no other systemic manifestations than fever at diagnosis, 1 (5%) had family history of psoriasis, and in 8 patients (36%) HLAB27 testing has not been performed. ILAR ‘modified’ criteria identified 74% (37/50) of SJIA patients (kappa value 0.73). Their sensitivity and specificity in the discrimination of SJIA patients from the control group were of 74% and 100% respectively, their positive predictive value (PPV) of 100% and their negative predictive value (NPV) of 78%. Yamaguchi criteria were accomplished in 46% (23/50) of SJIA patients (kappa value 0.41), with a sensitivity of 44%, a specificity of 98%, a PPV of 96% and a NPV of 62% respectively.

**Conclusion:** A substantial percentage of SJIA patients (44%) followed through the international, real-life JIRcohorte platform, failed to fulfill the existing ILAR classification criteria. The application of Yamaguchi criteria in our SJIA population was no more advantageous. However, ILAR ‘modified’ criteria had a better diagnostic value raising questions about the usefulness of HLAB27 testing in SJIA. The frequent absence at diagnosis of arthritis or systemic manifestations other than fever, may suggest the need of development of international consensus based strategies for diagnosis and treatment of probable and definitive SJIA.

**Disclosure of Interest**

None Declared

### P217 SUSTAINED REMISSION OFF MEDICATION IN PATIENTS WITH CHRONIC ACTIVE SYSTEMIC JUVENILE IDIOPATHIC ARTHRITIS (SJIA) TREATED WITH CANAKINUMAB

#### Maria Tsinti^1^, Vasiliki Dermentzoglou^2^, Elena Tsitsami^2^

##### ^1^Pediatric Rheumatology Unit, First Department of Pediatrics, University of Athens, Medical School, Children's Hospital Aghia Sophia; ^2^Pediatric Rheumatology Unit, First Department of Pediatrics, University of Athens, Medical School, Children's Hospital Aghia Sophia, Athens, Greece

###### **Correspondence:** Maria Tsinti

**Introduction:** Anti-IL-1 treatment has improved outcomes in sJIA. Remission and treatment discontinuation in severe longstanding sJIA is a desirable goal.

**Objectives:** To indicate that IL1β inhibition leads into off medication remission longstanding active SJIA with predominately systemic features

**Methods:** Case-series

**Results:** Patient details are presented in Table 1. **Patient 1** initially treated with steroids (CS) developed 2 MAS events on the 1^st^month necessitating IVCS pulses (IVMP). MTX was added; a 3^rd^MAS event developed during CS tapering. Anakinra (ANA) was added 4 months after onset and changed to CAN for adverse event-AE. A4^th^MAS event developed on CAN-1 year-and MTX-5 months after CS withdrawal. It subsided by IVMP, and cyclosporine. MTX was discontinued after 18 months and CAN after 2,7years remission. **Patient 2** initially manifested MAS treated by CS. Disease relapsed 4 months after CS withdrawal;CS were reinitiated along with MTX. 11 months after onset a relapse remitted by CS; ANA permitted CS taper. MTX was discontinued after 3 years for AE; ANA sustained remission for 7 months. Due to poor compliance ANA was changed to CAN. After 1 year CAN was withdrawn. After 21month remission a flare with fever and monoarthritis remitted by CAN reinitiation. **Patient 3** suffered 2 relapses during CS tapering in the 1^st^ month; MTX was added and discontinued in 3 months for relapse during CS tapering; 6 months after onset ANA allowed CS discontinuation. After 3 months ANA was changed to CAN due to AE. ANA washout led to reactivation. Remission was achieved in 8 months. CAN was discontinued after 2 years. **Patient 4** suffered 5 years of active disease presenting by CNS insulting MAS. MTX was added and discontinued in 6 months due to flare after CS withdrawal. ANA&CS were added 10 months after onset maintaining minimal activity for 10 months until a 2nd MAS. HLH treatment protocol led to remission for 2 months; for 1 year minimal activity under NSAID persisted. 4 years after onset fever, rash and arthritis remitted by CAN administration 2 mg/kg/month and Intraarticular CS. A flare after 4 months remitted by dose increase. For 1 year transient spontaneously remitting rash and arthritis occurred. The disease remained in remission on CAN for 19 months; it was discontinued due to thrombocytopenia after initiation of Growth Hormone initially considered AE of CAN/ GH, still persisting after 2,8years. **Patient 5** manifested fever, rash, polyarthritis and carditis attributed to C.*burnettii* unresponsive to antibiotics abating by CS. Disease flared 2 months after CS withdrawal necessitating reinitiation. MTX was added 6 months after onset. A flare 10 months after onset off CS remitted by ANA-withdrawn after 2 months for AE; disease flared and was treated by CS& CAN; MTX was discontinued after 6 months. CAN was discontinued by interval-increase after sustaining remission on medication for2,6years and 1year off medication. A flare with systemic manifestations remitted by CAN reinitiation

**Conclusion:** Canakinumab leads into off medication remission highly active SJIA. Informed consent to publish had been obtained.

**Disclosure of Interest**

None Declared

**Table 1 (abstract P217).** See text for description.

Gender/current age/age at SJIAonset-years{*y*}	1	2	3	4	5
	M/12/4,0	F/15/7,6	M/12/6,6	M/16/5,5	M/17/12,3
MAS before/on CAN	3/1	1/0	0/0	2/0	0/0
Drugs before /CS on CAN initiation	MTX, ANA, CYC,CS/ yes	MTX, ANA, CS/ -	MTX, ANA, CS/ -	MTX, ANA, CS,HLH-protocol/ -	MTX, ANA, CS/ yes
IVMP before/on CAN/ relapses during CS withdrwal	2/1/2	-/-/2	-/-/3	5/0/5	/--/2
activity on CAN initiation/flares under CAN treatment	-/1	-/-	yes/-	yes/2	-/-
Time(t) from onset to CAN initiation *y*	0,5	4,1	0,7	4,1	1,2
*t* of CAN administration *y*	3,6	1,0	2,7	3,0	2,6
remission *t* on/off CAN y	2,6/2,5	1,0/1,8	1,6/2,5	1,6/3,4	2,6/1
*t* from CAN initiation to remission *y*	-	-	0,8	1,2	-
AE	URIs, pneumonia	-	-	Herpes zoster, URIs	URIs

### P218 SAFETY OF TOCILIZUMAB IN PATIENTS AGED <2 YEARS WITH ACTIVE SYSTEMIC JUVENILE IDIOPATHIC ARTHRITIS TREATED FOR ONE YEAR

#### Sunethra Wimalasundera^1^, Inmaculada Calvo Penades^2^, Ruben Cuttica^3^, Hans-Iko Huppertz^4^, Rik Joos^5^, Diana Milojevic^6^, Margalit Rosenkranz^7^, Kenneth Schikler^8^, Tamas Constantin^9^, Wendy Douglass^1^, Chris Wells^1^, Yukiko Kimura^10^, Carine Wouters^11^

##### ^1^Roche Products Ltd, Welwyn Garden City, UK; ^2^Hospital Universitario y Politécnico La Fe, Valencia, Spain; ^3^Hospital General de Niños Pedro de Elizalde, Buenos Aires, Argentina; ^4^Professor Hess Children's Hospital, Bremen, Germany; ^5^ZNA, Antwerp, and UZ, Gent, Belgium; ^6^Tufts Medical Center, Boston; ^7^Children’s Hospital of Pittsburgh of UPMC, Pittsburgh; ^8^University of Louisville Medical School, Louisville, USA; ^9^Semmelweis University, Budapest, Hungary; ^10^Hackensack University Medical Center, Hackensack, USA; ^11^University Hospital Gasthuisberg, Leuven, Belgium

###### **Correspondence:** Carine Wouters

**Introduction:** The US Food and Drug Administration approved intravenous (IV) administration of tocilizumab (TCZ) for the treatment of patients (pts) ≥2 years (y) of age with systemic juvenile idiopathic arthritis (sJIA) in 2011 based on results of the phase 3 TENDER study.^1^ This approval was associated with a postmarketing requirement to investigate the use of TCZ in pts with sJIA <2 y (study NP25737). Results from the 12-week (wk) main evaluation period (MEP) have been reported.^2^

**Objectives:** Safety results following completion of the optional extension period (OEP) of NP25737 (until 52 wk from baseline or 2 y of age, whichever was longer, was reached) are now reported.

**Methods:** NP25737 was a multicenter, open-label, single-arm study to evaluate the pharmacokinetics and safety of IV TCZ 12 mg/kg every 2 wk for 12 wk in pts <2 y with active sJIA for ≥1 month whose treatment with corticosteroids and nonsteroidal anti-inflammatory drugs failed and who were receiving stable background therapy. After the 12-wk MEP, pts could participate in the OEP and continue TCZ treatment (without requirement for stable background therapy) to evaluate long-term safety. Cumulative adverse events (AEs) over the entire study period are reported.

**Results:** Seven of 11 pts enrolled in the MEP continued to the OEP and received ≥1 dose of TCZ. Across the entire study period (n = 11), the median number of TCZ doses was 11.0 (range, 2-26) and the median duration of exposure to TCZ was 22.1 wk (range, 4.1-58.1). Most pts (10/11; 90.9%) had ≥1 AE; most AEs were mild or moderate and unrelated to study drug. The most common AEs were upper respiratory tract infection (6/11 pts; 54.5%), hypersensitivity, neutropenia, rash, viral upper respiratory tract infection, and vomiting (3/11 pts each; 27.3%). Seven serious AEs occurred in 5 of 11 pts (45.5%); 2 occurred during the OEP (transaminases increased, histiocytosis hematophagic), 3 occurred during the MEP (hypersensitivity), and 2 occurred during the safety follow-up of the MEP (sJIA flare, hand-foot-and-mouth disease). AEs leading to dose modification occurred in 5 of 11 pts (1 in MEP, 4 in OEP) mostly because of infections, neutropenia, and elevated liver enzymes; all were mild or moderate. AEs leading to withdrawal occurred in 5 of 11 pts (45.5%): during the OEP, 1 pt was withdrawn because of a serious AE of increased transaminases; during the MEP, 3 pts were withdrawn because of serious hypersensitivity reactions to TCZ, and 1 pt was withdrawn because of thrombocytopenia. No deaths were reported during the study. AE rates per 100 pt-y of exposure are ?A3B2 show $132#?>reported (Table 1).

**Conclusion:** During the OEP, long-term treatment with TCZ was well tolerated in sJIA pts <2 y, and no additional safety signals were reported in the OEP beyond those reported in the MEP or observed previously for pts with sJIA ≥2 y.

**References**

1. De Benedetti F et al. *N Engl J Med* 2012;367:2385-95. 2. Mallalieu NL et al. *Arthritis Rheumatol* 2017;69(S10):A2856.

**Trial registration identifying number:** ClinicalTrials.gov NCT01455701

**Disclosure of Interest**

S. Wimalasundera Employee of: Roche, I. Calvo Penades: None Declared, R. Cuttica Consultant for: Roche, Novartis, Lilly, GSK, BMS, Janssen, Speaker Bureau of: Roche, Novartis, Lilly, GSK, BMS, Janssen, H.-I. Huppertz: None Declared, R. Joos: None Declared, D. Milojevic: None Declared, M. Rosenkranz: None Declared, K. Schikler: None Declared, T. Constantin: None Declared, W. Douglass Employee of: Roche, C. Wells Shareholder of: Roche, Employee of: Roche, Y. Kimura Speaker Bureau of: Novartis, SOBI, C. Wouters: None Declared

**Table 1 (abstract P218).** AE Rates

	TCZ 12 mg/kg IV Every 2 Wk		
	MEP n = 11	OEP n = 7	Entire Study Period n = 11
Total pt-y at risk	2.3	5.1	7.4
No. of AEs (rate per 100 pt-y at risk)			
Any AE	32 (1396.4)	47 (926.5)	79 (1072.7)
Serious AE	5 (218.2)	2 (39.4)	7 (95.1)
AE with fatal outcome	0	0	0
AE leading to withdrawal	4 (174.6)	1 (19.7)	5 (67.9)
AE leading to dose interruption	1 (43.6)	12 (236.5)	13 (176.5)

### P219 SERUM INTERLEUKIN 18 AS A BIOMARKER FOR SYSTEMIC JUVENILE IDIOPATHIC ARTHRITIS AND MACROPHAGE ACTIVATION SYNDROME AND USE OF RECOMBINANT HUMAN IL-18 BP IN A PATIENT WITH REFRACTORY DISEASE

#### Shima Yasin^1^, Rachel Brown^1^, Maggie Henderlight^1^, Ndate Fall^1^, Krista Solomon^1^, Scott Canna^2^, Charlotte Girard^3^, Cem Gabay^3^, Edwardo Schiffrin^4^, Andrew Slight^4^, Alexei Grom^1^, Grant Schulert ^1^

##### ^1^Rheumatology, Cincinnati Children’s Hospital Medical Center and Department of Pediatrics, University of Cincinnati College of Medicine, Cincinnati; ^2^Pediatric Rheumatology & Immunology, Children’s Hospital of Pittsburgh/UPMC, Pittsburgh, USA; ^3^Rheumatology, Geneva University Hospitals and University of Geneva, Geneva; ^4^AB2Bio, Lausanne, Switzerland

###### **Correspondence:** Shima Yasin

**Introduction:** Systemic juvenile idiopathic arthritis is an autoinflammatory disorder with prominent innate immune activity. Macrophage activation syndrome is a severe, potentially fatal complication of sJIA.

IL-18 is felt to play a key role in pathogenesis of sJIA and in particular MAS; possibly through IFN-g activation.IL-18 binding protein (IL-18BP) is an endogenous inhibitor that binds IL-18. However, clinical effects of blocking IL-18 in sJIA are still unknown.

**Objectives:** To examine total serum IL-18 levels in sJIA patients with regards to disease activity, MAS features, and other SJIA and MAS biomarkers. Additionally, we report the use of recombinant IL-18 BP (rIL-18BP) in a patient with severe, refractory sJIA.

**Methods:** Serum samples obtained from 40 established sJIA patients. Total IL-18, CXCL9 and S100 protein were measured. In addition, in one patient serial total serum and free IL-18 levels were determined. We compared IL-18 levels in patients with active vs inactive disease and with history of MAS vs no MAS.

**Results:** Total serum IL-18 levels (in pg/ml) were higher in patients with active sJIA (Median:16499, Interquartile Range(IQR):4816-240986, N=21), but were still persistently elevated (Median: 1163, IQR (587-38539), N=19, upper limit of normal 540) in majority of patients with inactive disease (p=0.0004). Patients with history of MAS had significantly higher IL-18 levels (Median: 13880, IQR (4212-62628), N=20) as compared to those without MAS history (Median: 956, IQR (276-5425), p=0.0001). Patients with active fever and arthritis at the time of sampling had significantly higher IL-18 levels compared to those without (P=0.0173 and 0.0170 respectively). Also, those with systemic features and elevated ESR/CRP showed significant difference from their counterparts (p=0.0225 and 0.002 respectively). We observed moderate significant correlation between total IL-18 and CXCL9 (correlation coefficient of 0.558 (p=0.0002), as well as S100A8/A9 and A12 (r=0.474 and 0.456).

Given these findings by us and others, we utilized Tadekinig alfa (rhIL-18BP) in a 5 year old male with sJIA and persistently elevated free IL-18 levels. His course was complicated by recurrent MAS, failure of all prior biologic treatments, chronic high dose steroids with flare of sJIA and MAS upon attempts to wean steroids. He was started on rhIL-18BP 2mg/kg/dose subcutaneous every 48h in addition to continuous IL-1 inhibition. His initial total and free serum IL-18 levels were high at 117356 pg/ml and 46.8 pg/ml respectively. Although his total IL-18 remained elevated after start of rhIL-18BP, free IL-18 was undetectable.

In addition, since initiation of rIL-18BP, steroid dose was decreased by more than 50% with increase in linear growth. While on rIL-18BP, he developed two MAS episodes (triggered by parainfluenza and acute gastroenteritis), both were mild and easily controlled.

Recently, he had worsening lung disease and rIL-18BP was tapered. Immediately after, he developed MAS flare. This time MAS flare was very severe and difficult to control.

**Conclusion:** Total serum IL-18 levels were elevated in the majority of sJIA patients regardless of disease activity, with higher levels in patients with active disease and those with history of MAS. This indicates that IL-18 might be an important driver of sJIA and MAS. Use of rIL-18 BP improved patient disease course with less frequent and easily controlled MAS episodes. This response raises optimism about use of rIL-18 BP in patients with IL-18 driven disease preventing life-threatening MAS.

**Disclosure of Interest**

S. Yasin: None Declared, R. Brown: None Declared, M. Henderlight: None Declared, N. Fall: None Declared, K. Solomon : None Declared, S. Canna Consultant for: AB2Bio, C. Girard: None Declared, C. Gabay Shareholder of: Ab2 Bio, Grant / Research Support from: Pfizer, Roche, AB2 Bio, Consultant for: Roche, Pfizer, BMS, Merck, Sanofi, Regeneron, Eli Lilly, Novartis, Ab2 Bio, Speaker Bureau of: Roche, Pfizer, BMS, Merck, Sanofi, Regeneron, Eli Lilly, Novartis, Ab2 Bio, E. Schiffrin: None Declared, A. Slight: None Declared, A. Grom Grant / Research Support from: NovImmune, AB2Bio, Consultant for: Novartis, Jun, G. Schulert : None Declared

### P220 MACROPHAGE ACTİVATİON SYNDROME: A SINGLE CENTRE EXPERIENCE FROM TURKEY

#### Ayşe Zopcuk, Şerife G. Karadağ, Mustafa Çakan, Nuray A. Ayaz

##### Pediatric Rheumatology, Sağlık Bilimleri University Kanuni Sultan Süleyman Training and Research Hospital, Istanbul, Turkey

###### **Correspondence:** Ayşe Zopcuk

**Introduction:** Macrophage activation syndrome is a rare and potentially fatal disorder, caused by excessive activation and proliferation of T lymphocytes and macrophages. It can be triggered by drugs, malignancies, infections, and rheumatic diseases.

**Objectives:** This study was established as a retrospective review of the patients diagnosed and followed-up in Sağlık Bilimleri University KSSEAH Pediatric Rheumatology clinic as MAS between the years 2010 and 2018. Our purpose was to report our single-center experience with MAS.

**Methods:** The medical charts of patients diagnosed and followed-up as MAS between the years 2010–2018 retrospectively. Totally 25 patients were enrolled to the study. Data recorded included age, sex, age at-diagnosis, delay to the diagnosis, laboratory data, treatment modalities and outcomes.

**Results:** In our study, a total of 25 patients with macrophage activation syndrome were reviewed. 20 patients were diagnosed with macrophage activation syndrome secondary to systemic juvenile idiopathic arthritis (soJIA). Two patients were diagnosed with MAS secondary to Kawasaki disease (n=1) and periodic fever syndrome (n=1). Virus-related MAS (1 parvovirus B19, 1 leishmania and CMV, 1 streptococcus) was progressed in three patients. Thirteen (52%) of these patients were female and twelve (48%) were male. The mean age at the time of diagnosis was found to be 6.9 ± 0.4 years. The mean time between soJIA diagnosis and MAS diagnosis was 4.7days. The most common clinical finding at presentation (100%) was increased body temperature. Bone marrow aspiration was performed at all patients except one. While hemophagocytosis was not observed in 7 (29.1%) of MAS patients, hemophagocytosis was observed in 3 (12.5%) patients without MAS finding. Systemic steroid treatment was administered to all patients except one patient. Cyclosporine A was given to fourteen patients (56%), biological agents were given to ten patients (40%). Plasmapheresis was performed in three patients. Six patients (24%) received intensive care support. Mortality was not observed. In patients with systemic JIA who were diagnosed with MAS, monocyclic course was observed in 9 (45%) patients, polycyclic in 7 (35%) patients and chronic polyarticular in 4 (20%) patients. In the last control, 13 (52%)patients were in remission without medication, while 12 (48%) patients were in remission with medication.

**Conclusion:** Macrophage activation syndrome (MAS) is the term used to describe a potentially life-threatening complication of systemic inflammatory disorders, which occurs most commonly in systemic juvenile idiopathic arthritis (JIA), although its occurrence in patients with other autoimmune or autoinflammatory conditions, i.e., adult- and childhood onset systemic lupus erythematosus, Kawasaki disease, and periodic fever syndromes, is being reported with increased frequency. Virus-associated MAS is a well-recognized entity. Most cases are related to Epstein–Barr virus (EBV), cytomegalovirus (CMV), and herpesvirus, while streptococcus and parvovirus B19-induced MAS is very rare. Complete recovery can be provided with early and efficient treatment in macrophage activation syndrome.

**Disclosure of Interest**

None Declared

## Vasculitides II

### P221 PARVOVIRUS INFECTION AND KAWASAKI DISEASE: ONE DISEASE FOR TWO SIBLINGS

#### Maria Cristina Maggio^1^, Rolando Cimaz^2^, Annalisa Alaimo^3^, Calogero Comparato^3^, Daniela Di Lisi^3^, Clotilde Alizzi^4^, Sabrina Spoto^3^, Maria Assunta Garofalo^3^, Giovanni Corsello^1^

##### ^1^University Department Pro.Sa.M.I. “G. D’Alessandro”, University of Palermo, Palermo; ^2^NEUROFARBA Department, University of Florence, and AOU Meyer, Florence; ^3^Paediatric Cardiology Operative Unit, Children Hospital “G. Di Cristina”; ^4^Children Hospital “G. Di Cristina”, ARNAS, Palermo, Palermo, Italy

###### **Correspondence:** Maria Cristina Maggio

**Introduction:** Kawasaki disease (KD) is rarely described in siblings in the same time. In these cases, an infectious trigger must be excluded.

**Objectives:** We describe the clinical course of two brothers who showed severe KD all at once, secondary to Parvovirus infection.

**Methods:** A 9-month-old female showed fever, pallor, vomiting, bilateral non-secreting conjunctivitis, rash. Anamnesis revealed that 12 days before, she had fever, spontaneously resolved. At admission, 9 days after fever onset, she showed fever, conjunctivitis, pharyngitis, rash, and cervical adenopathy. Haematological parameters showed: leukocytosis, neutrophilia; anaemia; CRP: 2.31; ESR: 120. ECG and echocardiography were normal, including coronary Z-scores. She showed positive Parvovirus IgM. Spontaneous defervescence occurred. Further cardiological evaluation was performed to exclude a pericarditis secondary to Parvovirus, and at day 26 after fever onset, coronary artery lesions (CAL) were documented: proximal right coronary artery Z-score of 6.02; left main coronary Z-score: 5.72; left anterior descending Z-score: 5.78. The child was promptly treated with IVIG plus ASA.

A further echocardiographic evaluation showed worsening of CAL, with a sacciform aneurysm in the left anterior descending artery (Z-score:5.08). Laboratory test did not show inflammation; however, the girl was treated with 3 bolus doses of intravenous methylprednisolone (30 mg/kg/dose). The Z-score of CAL did not change and the patient was treated with anakinra (4 mg/kg/day), with a progressive improvement of CAL, and after 2 months, Z-scores normalized.

The 7-year-old brother presented fever, vomiting at the same time of the sister, with spontaneous resolution after 4 days. Four days later, he presented again fever with abdominal pain, tachypnoea and tachycardia, secondary anuria. He had: leukocytosis, neutrophilia, anemia; CRP: 0.24; CPK: 773; creatinine: 0.77; BUN: 111; elevated myocardial necrotic enzymes (c-Troponin T: 91.4; Pro-BNP: > 70.000).

Echocardiogram showed generalized hypokinesia, a severe reduction of the ejection fraction (EF) (20-25%); increased left atrium (Z-score: 3.3) and mitral valve with moderate insufficiency. He received dopamine, dobutamine, furosemide plus steroids. He showed a constant improvement of echocardiographic parameters, plasmatic enzymes and clinical signs. In 16^th^ day he was discharged with an EF of 45% and persistent septal hypokinesia. However, specific serology anti-Parvovirus was tested and showed increased IgM, with negative IgG. The cardiological outcome revealed a progressive improvement of EF, which reached the 50%.

**Results:** CAL significantly improved after anakinra, at the contrary, the clinical evolution in the brother was different.

**Conclusion:** We describe familial KD in two siblings which had the same infectious trigger (Parvovirus). The brother was diagnosed as a post-viral myocarditis. However, considering the two parallel and different evolution, the girl showed late CAL with aneurisms, and the brother a Kawasaki shock syndrome picture with myocardial dysfunction. Viral illnesses are recognised trigger of KD, and in these cases the rareness is the coincident KD in two siblings, with different and severe clinical course. Noteworthy, the girl had aneurisms which resolved with anakinra, a therapy which has been recently shown to be promising for this disease. Informed consent to publish had been obtained from the parents.

**Disclosure of Interest**

None Declared

### P222 PANCREATITIS IN HENOCH-SCHONLEIN PURPURA. A SINGLE-CENTRE OBSERVATIONAL STUDY

#### Maria Cristina Maggio, Saveria Sabrina Ragusa, Giuseppe Salvo, Clotilde Genesia Alizzi, Giovanni Corsello

##### University Department Pro.Sa.M.I. “G. D’Alessandro”, University of Palermo, Palermo, Italy

###### **Correspondence:** Maria Cristina Maggio

**Introduction:** Henoch-Schönlein purpura (HSP) is the most frequent vasculitis in children. Typically, it is characterized by palpable purpura, joints swelling, arthralgia, abdominal pain with possible intestinal bleeding. In more severe cases, the patients show acute abdomen.

Acute pancreatitis is a rare dramatically evolutive, life-treating manifestation of SHS and it can be associated with a fulminant course. Persistent abdominal pain, need to be investigated by the dosage of serum pancreatic amylase, lipase and by abdominal MRI. In these patients, corticosteroid treatment is recommended and must be associated with parenteral feeding.

**Objectives:** We analysed the full series of children with HSP admitted to our paediatric unit in the period 2011-2018.

**Methods:** We retrospectively collected data of 50 children (age: 4-14 years), with HSP who needed hospitalization.

4/45 patients (9%) developed an acute pancreatitis.

All the patients were males, age:3-10 years. All the patients did not show other risk factors of pancreatitis.

**Results:** The treatment was parenteral feeding in 100% of the patients. One patient with pancreatitis and nephrotic syndrome received 3 bolus doses of methylprednisolone (30 mg/kg/dose) followed by prednisolone (2 mg/kg/day) and mycophenolate; one patient with pancreatitis and acute renal failure, received 3 bolus doses of methylprednisolone (30 mg/kg/dose) followed by a single-dose of cyclophosphamide (750 mg/m2), followed by azathioprine (50 mg/day). One patient showed a mild pancreatitis and healed with prednisolone (2 mg/kg/day) and parenteral feeding. One patient showed acute pancreatitis, associated with acute intestinal bleeding, orchitis, was treated with 3 bolus doses of methylprednisolone (30 mg/kg/dose) followed by prednisolone (2 mg/kg/day). Steroids treatment was progressively tapered until the complete resolution of the pancreatic involvement.

**Conclusion:** Acute pancreatitis is a rare and life-treating manifestation of HSP. High-doses steroids are a recognised and useful treatment, associated with parenteral feeding. In non-responders to steroids, immune suppression treatment is the second-line treatment to induce remission.

The choice depends on associated manifestations of HSP and on associated failure of other organs.

**Disclosure of Interest**

None Declared

### P223 A CASE OF KAWASAKI DISEASE SUCCESSFULLY TREATED WITH ANAKINRA

#### Angela Mauro^1^, Roberto Rega^2^, Luigi Martemucci^3^, Rita Sottile^3^

##### ^1^Pediatrics, Rheumatology Unit, "Santa Maria della Pietà" Hospital, Nola; ^2^ Department of Respiratory Diseases, University of Naples Federico II; ^3^Rheumatology Unit, Santobono-Pausilipon Children’s Hospital, Naples, Italy

###### **Correspondence:** Angela Mauro

**Introduction:** Kawasaki disease (KD) is a systemic vasculitis that affects the medium-and small-size arteries.Some children do not respond to the first line of therapy.The treatment for IVIG non-responsive patients is controversial,but adding steroids to the second IVIG dose can be effective at reducing the incidence of CAA. For those non-responders to this second step of therapy,other approaches have been described.In recent years,some reports have suggested the role that Anakinra can play in the treatment of severe or resistant cases of KD.

**Objectives:** We present a case of an IVIG and steroids-resistant KD treated with Anakinra.

**Methods:** We present the case of a one-year-old with persistent fever for six days,a generalized rash,lymphadenopathy,conjunctivitis,cheilitis,swollen hands and feet,diarrhoea and irritability.Blood tests revealed increased C-reactive protein (100.80 mg/L),D-Dimer (532.00 ng/mL) and Fibrinogen (810.00 mg/dL).There was increased platelet count (532,000/mm3) and hyperneutrophilia (neutrophils 79.80 %);He showed low levels of serum albumin (2.9 g/dl),serum sodium (130 mEq/L) and serum chloride (90 mEq/L).Blood cultures and serological tests for infection were negative.He underwent an echocardiography that showed an enlarged coronary diameter,(right 3.9 mm, left 3.6 mm; circumflex 3.1 mm, proximal anterior inter-ventricular 3.1 mm; worse Z score +5).

**Results:** From the presence of fever that had lasted for more than five days,the lymphadenopathy,conjunctivitis,the cutaneous rash and the oedema of the hands and feet with cardiac involvement,we diagnosed complete Kawasaki disease and started IVIG (2 g/Kg) and Aspirin (80 mg/Kg).Despite the initial IVIG treatment, the fever,rash,conjunctivitis,cheilitis and mild oedema of hands and feet remained,and additional IVIG doses and three metilprednisolone (30 mg/kg) pulses were administered in the following days.The patient underwent another echocardiography that did not show any changes.Maintenance treatment with oral prednisone (0.5 mg/kg/day) was initiated.The patient became afebrile, and the rash, cheilitis and conjunctivitis gradually disappeared.Serological markers of inflammation returned to the normal range.After ten days, the patient’s fever recurred with irritability, exanthema,conjunctivitis, and hand and feet desquamation.Blood tests showed a significant increase of CRP (65.30 mg/L),ESR (50 mm/h);anaemia (Hb 10.4 g/dL) and more platelets (566,000) were also registered.Echocardiography did not show changes.Further Metilprednisolone pulses were administered over three days.After one day the patient repeated the Echocardiography, which showed a worsening of the enlargement of the coronary diameter,(right 6 mm (Z score 13),left 6 mm (Z score 9.9),proximal anterior inter-ventricular 5.7 mm (Z score 15).There was an aneurism in the diagonal branch of left anterior descending coronary (7.2 mm; Z Score >10) with a thrombus inside.After three days, the patient’s fever continued.Due to the persistence of the fever,the worsening of the echocardiography features and blood tests,with the agreement of his parents,other intravenous treatments with IL-1RA were initiated (Anakinra 2 mg/kg subcutaneously once a day for two weeks).One day later, the fever disappeared and CRP, ESR, platelet and haemoglobin levels returned to normal in the subsequent blood tests.The Aspirin dose was reduced to the antiplatelet dose and Clopidogrel (5 mg) was added.

**Conclusion:** Treatment with Anakinra was maintained for two weeks, and Prednisone (0.5 mg/kg/dy), Aspirin and Clopidogrel.There were no flares of the disease after discontinuing the Anakinra and blood tests were within the normal range. Echocardiography tests showed an improvement in his coronary enlargement. Written informed consent was obtained from the patients for publication of Case Report.

**Disclosure of Interest**

None Declared

### P224 SUCCESSFUL TREATMENT OF HAEMORRHAGIC BULLOUS HENOCH-SCHONLEIN PURPURA WITH INTRAVENOUS IMMUNOGLOBULINS

#### Angela Mauro^1^, Rega Roberto^2^, Luigi Martemucci^3^, Rita Sottile^3^

##### ^1^Rheumatology Unit, Rheumatology Unit, Department of Pediatrics, "Santa Maria della Pietà" Hospital, Nola, Naples, Italy, Nola; ^2^Department of Respiratory Diseases, Division of Pneumology, University of Naples Federico II; ^3^Pediatrics, Rheumatology Unit, Santobono-Pausilipon Children’s Hospital, Naples, Italy

###### **Correspondence:** Angela Mauro

**Introduction:** Henoch-Schonlein purpura(HSP) is the most common childhood systemic vasculitis.The disease affects the skin,the gastrointestinal tract,the joints and kidneys.In rare cases the skin can present as haemorrhagic,bullous or necrotic lesions.

**Objectives:** We describe a case of HSP presenting with severe skin lesions that did not respond to standard therapy with corticosteroids,and was therefore treated with intravenous immunoglobulins(IVIG).

**Methods:** An 11-year-old girl was admitted to our department with fever,abdominal pain,arthralgia,severe purpuric palpable rash and haemorrhagic bullae distributed on the buttocks and lowerextremities.Physical examination showed a purpuric palpable rash,multiple haemorrhagic bullae and vesicles ranging in size from five to 30mm on the buttocks and lower limbs.The abdomen was painful but soft.Blood tests revealed a white blood cell (WBC) count of 20.730/ml with normal differential, platelets (PLT) 336.000/ml,haemoglobin (Hb) 13.3 g/dl,erythrocyte sedimentation rate (ESR) 30 mm/1h (nv 20 mm/1h), C-reactive protein (CRP) 13 mg/L.She tested negative for streptococcal tests showed Antistreptolisine (ASLO) 778 IU/mL (nv 0-200) and anti-DNAse 1170 IU/mL (nv 0-200).Microscopic analysis of the patient’s urine showed mild proteinuria (30mg/dl) and an occult blood test on a stool sample was negative.Abdominal ultrasound was unremarkable.A skin biopsy revealed peri-nuclear infiltration of polymorphs,nuclear leukocyte and mononuclear cells with IgA deposition in immunofluorescence studies,typical of leukocytoclastic vasculitis.

**Results:** Clinical findings were consistent with HSP and she was started on oral prednisone (1mg/kg/daily),as well as antibiotics with teicoplanin for the super-infection of skin lesions (Staphylococcous Aureus).After ten days she was discharged,with an improvement in skin appearance.She was given a schedule to progressively taper and stop steroid therapy within a month.At the 15-day follow-up,she presented new onset of purpuric rash on her lower extremities and scars of previous bullae.We decided to increase the dosage of corticosteroid therapy from 0.5mg/Kg to 1mg/kg/die.At the one-month follow-up she presented a new purpuric rash and haemorrhagic bullae on her lower extremities,associated with abdominal pain.She was therefore readmitted to our department.Blood tests revealed WBC 10.730/ml with normal differential count,PLT 300.000/ml,Hb13.2 g/dl,ESR 40 mm/1h,CRP 15 mg/L;creatinine,were within range.The results of urinalysis,an occult blood test of a stool sample and an abdomen ultrasound were all negative.She started an IVIG infusion (2g/kg),while still on oral prednisone (1mg/kg/daily),progressively tapered.After treatment she showed good improvement in respect of her skin lesions and was discharged.At the 15-30-60 day follow-up visits the patient was in good general condition;she did not have any new lesions but only scars on her lower limbs.

**Conclusion:** There is no consensus about the management for cutaneous manifestations in HPS.In the case of rare skin complications,such as haemorrhagic bullae lesions,immunosuppression with corticosteroid therapy is often necessary in order to control the inflammation and limit the expanse of necrosis.Unfortunately,our patient gained only partial benefit from corticosteroid therapy.As a consequence,we decided to treat her with an IVIG (2g/kg as a single infusion) associated with oral prednisone (1mg/kg/daily),progressively tapered,which induced rapid and persistent resolution of symptomatology.Written informed consent was obtained from the patients for publication of Case Report.

**Disclosure of Interest**

None Declared

### P225 SUPERFICIAL VENOUS THROMBOSIS AND RECURRENT ORAL APHTHOUS ULCERS IN A CHILD- A CASE REPORT

#### Ana Raquel Mendes^1^, Sandrina Braga^2^, Catarina Vilarinho^3^, Maria Antónia Costa^4^, Cristina Ferreira^5^, Teresa São Simão^5^

##### ^1^Pediatrics, Centro Materno-Infantil do Norte, Centro Hospitalar do Porto, Porto; ^2^Vascular Surgery; ^3^Dermatology; ^4^Ophthalmology; ^5^Pediatrics, Hospital Senhora da Oliveira, Guimarães, Portugal

###### **Correspondence:** Ana Raquel Mendes

**Introduction:** Behçet ´s disease is a systemic inflammatory disease characterized by recurrent oral aphthae affecting the oral and genital mucosa, and several systemic manifestations such as ocular, neurologic or vascular disease, skin lesions, gastrointestinal involvement or arthritis. Even though its peak age of onset is in young adults 20 to 40 years of age, it ocasionally occurs in children.

**Objectives:** To report a pediatric-onset HLA-B51 positive case of Behçet ´s disease.

**Methods:** Review of the medical records.

**Results:** An 11-year-old boy with recurrent oral aphthae was referred from the pediatric emergency department to the pediatric rheumatology consultation after two episodes of great saphenous vein superficial thrombophlebitis. He had been experiencing daily oral aphthae with spontaneous resolution after 10 days for the past three years (>3 episodes in a year). Besides, his mother described various episodes of folliculitis with formation of pustules over the previous months. He denied fever, rash, weight changes, sleep disturbance, headaches, abdominal pain, change in bowel habits or diuresis, respiratory symptoms, visual disturbances or fatigue. His physical exam and pediatric gait arms legs spine exam was normal except for two oral aphthae and some acneiform lesions. Laboratory data including complete blood count with renal function, ionogram, reactive c protein, coagulation studies, viral markers for hepatitis B and C and HIV, herpes simplex and varicella-zoster virus serology and calprotectin were negative. Immunglobulin levels were normal and autoimmunity studies with beta 2 glycoprotein antibodies, lupus anticoagulant, anti-HLA-B27, anti-nuclear, anti-SSA and SSB, anti-RNP, anti-SM, anti-Scl-70, anti-JO-1, anti-cardiolipin and anti-ds-DNA antibodies and markers for celiac disease were negative. Urine testing showed discrete proteinuria. A pathergy test was performed, which was negative. His echocardiogram was normal and his ophtalmologic evaluation did not show any signs of uveitis. Thus, a diagnosis of Behçet ´s disease was established according to the International Classification Criteria for Behçet ´s disease. HLA- B51 testing was later requested, which was positive. According to the EULAR 2008 guidelines, he initiated colchicine without further episodes of oral aphthae. He currently maintains a regular follow-up in the pediatric rheumatology and vascular surgery consultation, without any further occurences except for an episode of self-limited epididymitis.

**Conclusion:** Although the diagnostic criteria for Behçet ´s disease are well-established in adults, diagnosis and treatment is still a challenge in the pediatric population, as there are no pathognomonic laboratory tests and diagnostic criteria are not adapted to children. The consensus classification criteria have been recently developed for pediatric Behçet ´s disease in an attempt to increase the sensitivity of the prior criteria. We described a case illustrating that a detailed clinical history and a thorough physical exam associated with a high level of suspicion remains the most suitable approach. These patients require a periodic ophtalmologic evaluation and a close vigilance of prothrombotic states.

Written informed consent has been obtained from the legal representative of the subject involved.

**Disclosure of Interest**

None Declared

### P226 Withdrawn

### P227 EFFECTS OF SUBCUTANEOUS INJECTION OF TOCILIZUMAB FOR CHILDHOOD TAKAYASU ARTERITIS AND ITS EVALUATION MEANS

#### Nami Okamoto, Kosuke Shabana, Yuko Sugita, Keisuke Shindo, Hiroshi Tamai

##### Department of Pediatrics, OSAKA MEDICAL COLLEGE, Takatsuki-city, Japan

###### **Correspondence:** Nami Okamoto

**Introduction:** Last year, a subcutaneous injection of tocilizumab (scTCZ) was approved for Takayasu arteritis (TA) over 12 years of age in Japan. Based on our experience in pediatric patients, we describe the effects and the issues.

**Objectives:** To consider the positioning of scTCZ for chilhood TA, the appropriate dosage form and the evaluation means.

**Methods:** We examined medical information that was extracted from patients’ charts, such as vital signs, the Indian Takayasu Arteritis Clinical Activity Score (ITAS2010), results of blood examination, echocardiography, vascular ultrasonography and other imaging inspection, and evaluated the change before and after the initiation of scTCZ by the dosage of 125mg once a week.

**Results:** There are two children with TA treated with scTCZ. Case 1 is a 12-year-old girl who developed reversible cerebral vasoconstriction syndrome combined with cerebral infarction. Because of the upper extremity blood pressure differential and cervical murmurs, contrast CT and vascular ultrasonography were performed that revealed the wall thickening and stenosis of cervical and subclavian arteries. HLA-B52 was positive, but other blood examination shows no abnormality, including erythrocyte sedimentation rate (ESR), C-reactive protein (CRP) and interleukin (IL)-6. Oral prednisolone (PSL) was started that resulted in no improvement of artery wall thickening, then intravenous TCZ (8mg/kg, every other week) was initiated 4 months after the onset. The thickening of arteries, blood flow acceleration and ITAS2010 improved, and prednisolone was decreased to 5mg/day. 11 months after the onset, the dosage of TCZ was switched to 125mg subcutaneously once a week and the effect continues. Case 2 is an 11-year-old girl whom complicated with ulcerative colitis and had been treated with azathioprine, prednisolone and infliximab. She complained fever, fatigue, leg pain after exercise, and was accompanied with high CRP level. Positron emission tomography revealed integration at cervical, subclavian, brachial and femoral arteries. The upper extremity blood pressure differential and cervical murmurs were confirmed. The symptoms, the inflammatory response on blood examination and the findings of vascular ultrasonography improved by the administration of systemic PSL, however, serum ferritin level remained high and the thickening of arteries exacerbated as the PSL was tapered. Infliximab was switched to scTCZ 24weeks after the diagnosis and there has been no deterioration of disease activity with the lowest level of serum ferritin. In both cases, vascular ultrasonography and ITAS2010 enabled us to detect disease activity, even if CRP was negative and was not useful as an evaluation means.

**Conclusion:** The scTCZ was effective for childhood TA, however, there are many issues that should be discussed; timing of indication, presence or absence of immunosuppressants, dosage form for patients under 12 years of age, safety for complications such as UC, evaluation index instead of CRP, the minimum ?A3B2 show $132#?>maintenance dose of PSL, and so on. Consent to publish had been obtained.

**Reference**

Nakaoka Y, Isobe M, Takei S, Tanaka Y, Ishii T, Yokota S, et al. Efficacy and safety of tocilizumab in patients with refractory Takayasu arteritis: results from a randomized, double-blind, placebo-controlled, phase 3 trial in Japan (the TAKT study). Ann Rheum Dis 2018;77:348–354.

**Disclosure of Interest**

None Declared

**Table 1 (abstract P227).** The Evaluation index before and after scTCZ administration

	CRP at diagnose (mg/dl)	CRP after scTCZ (mg/dl)	ESR at diagnose (mm/1h)	ESR after scTCZ (mm/h)	Serum IL-6 at diagnose (pg/ml)	Serum IL-6 after scTCZ (pg/ml)	ITAS2010 at diagnose	ITAS2010 after scTCZ
Case1	0.14	<0.01	4	2	<1.5	8.2	15	3
Case2	10.25	<0.01	68	4	Not studied (0.07 with 0.5mg/kg of PSL)	85	5	3

### P228 WILLIAMS-BEUREN SYNDROME DIAGNOSE IN 15-YEAR-OLD PATIENT WITH SUSPECTED SYSTEMIC VASCULITIS

#### Maria B. Tomaszek^1^, Ilona Jaszczuk^2^, Violetta Opoka-Winiarska^1^, Monika Lejman^3^

##### ^1^Department of Pediatric Pulmonology and Rheumatology; ^2^Department of Pediatric Hematology, Oncology and Transplantology; ^3^Department of Pediatric Oncology and Hematology, Medical University of Lublin, Poland, Lublin, Poland

###### **Correspondence:** Violetta Opoka-Winiarska

**Introduction:** Middle aortic syndrome (MAS) is a clinical condition generated by segmental narrowing of the abdominal or distal descending thoracic aorta. The syndrome may have a different etiology: acquired, caused for example by Takayasu disease or congenital, attributed to developmental aortic anomalies. The Williams-Beuren syndrome is a congenital, genetically conditioned disorder, caused by deletion on the long arm of chromosome 7, in the region 7q11.23. It is characterized by a group of congenital malformations with dysmorphic features, abnormal psychomotorical development, intellectual disability, cardiovascular defects, most often in the form of supravalvular aortic stenosis or peripheral pulmonary artery stenosis, muscular tension disorders and a changed behavioral profile.

**Objectives:** The aim of our study is to present the diagnosis the Williams-Beuren syndrome in patient with suspected systemic vasculitis.

**Methods:** Retrospective analysis of patient's history.

**Results:** The first symptoms: palpitations and chest pains after physical exertion were first noticed at the age of 15. Subsequently, the patient was evaluated by a cardiologist and a nephrologist – they diagnosed hypertension, aortic valve regurgitation and mitral valve insufficiency. Directed to the Department of Rheumatology with suspected systemic vasculitis. During observation, the patient was seen to be capable of tolerating moderate physical exertion, and variable blood pressure was observed with the difference between L and P of the upper limb. Subsequent stages of diagnosis revealed thoracic, abdominal and aortic stenosis, as well as stenosis in the renal and internal carotid arteries. The patient met the criteria of Takayasu arteritis: angiographic abnormality, pulse deficit blood pressure discrepancy and hypertension but PET / CT scans showed no irregularities. The results of laboratory tests (including morphology, hemostasis, inflammation, pANCA, cANCA) were also normal. The patient was referred to the genetic counselling. Numerous dysmorphic features were noticed in the physical examination. An MLPA study was performed using a set of probes for region 7q11.23, confirming the presence of deletions. The results of the molecular tests let to the diagnosis of Williams-Beuren syndrome. Correct MLPA results from both parents confirmed that the deletion in the patient's 7q11.23 region is de novo.

**Conclusion:** In the diagnosis of patients with vasculitis symptoms, congenital diseases, including rare genetic syndromes, should be considered. Multidisciplinary cooperation is a prerequisite for making the correct diagnosis. Informed consent to publish had been obtained from the parent.

**Disclosure of Interest**

None Declared

### P229 TAKAYASU ARTERITIS IN CHILDREN: A RETROSPECTIVE REVIEW FROM A TERTIARY REFERRAL HOSPITAL

#### Inês Pizarro Madureira Salgado Da Costa^1^, José Diogo Martins^2^, Margarida P. Ramos^1^

##### ^1^Área de Pediatria Médica, Hospital D. Estefânia; ^2^Serviço de Cardiologia Pediátrica, Hospital de Santa Marta, Centro Hospitalar Lisboa Central, EPE, Lisboa, Portugal

###### **Correspondence:** Inês Pizarro Madureira Salgado Da Costa

**Introduction:** Takayasu arteritis is an idiopathic large-vessel vasculitis that primarily affects the aorta and its major branches. Chronic granulomatous inflammation leads to stenosis and occasionally aneurysms of the involved portions of the arteries, producing a wide variety of symptoms. High-dose corticosteroids are the mainstay of therapy but adjunct immunosuppression is frequently required. TNF-a blocking agents have shown to be effective in refractory cases.

**Objectives:** Characterization of patient demographics, clinical features, disease extent, treatment and outcomes in children with Takayasu arteritis

**Methods:** Retrospective data analysis of a case series of children fulfilling the EULAR/PRINTO/PRES criteria for childhood Takayasu arteritis in a tertiary hospital setting during a 10-year period.

**Results:** A total of 4 children (3 female), with a median age at diagnosis of 7 years (5 -10 years) were identified during this period. The most common clinical features at presentation were systemic symptoms and cardiovascular involvement (4/4). Weak peripheral pulses, discrepant systolic blood pressure between limbs and arterial bruits were present in all cases. Arterial hypertension was detected in half of the patients. Neurological manifestations were also very common (3/4), with seizures at the initial presentation in 2 patients and ischemic stroke in 1 case. A minority of patients complained of gastrointestinal (1/4) and respiratory (1/4) symptoms. Acute phase reactants (ESR and CRP) were elevated in all patients. Thrombocytosis (3/4) and chronic disease anaemia (2/4) were also commonly found.

The distribution of the involved arteries according to the angiographic classification of Takayasu arteritis showed a pattern type V in 3 cases, one of which was VP+. The remaining case displayed a pattern IIa.

First-line treatment in all cases included high-dose corticosteroids and methotrexate. Cyclophosphamide was added in 2 cases. Due to a severe and refractory disease course, TNF-a blocking agents were administered in all patients (Infliximab, 2 cases; Adalimumab, 2 cases).

Endovascular procedures were required in 2 patients with irreversible stenosis: renal artery percutaneous transluminal angioplasty (2 cases) and pulmonary artery stenting (1 case).

There were no relevant infections or mortality during the follow-up period. Repeated magnetic resonance angiography to assess disease activity showed improvement in vascular lesions in 3 patients, with remission in 1 case. None of the patients presented disease progression with involvement of new vessels.

**Conclusion:** The diagnosis of childhood Takayasu arteritis remains challenging, mainly due to the insidious clinical course and the lack of specificity of the presenting symptoms. The abnormal peripheral pulses in the setting of elevated inflammatory markers should raise suspicion for this condition.

Previous studies found that constitutional features like low-grade fever, weight loss and arthralgia are more frequently found in childhood Takayasu arteritis compared *with* adult patients. In our series these symptoms were present in all patients.

We report a higher frequency of neurological involvement compared with other paediatric reports, perhaps reflecting the severity of the angiographic pattern and the delay in diagnosis.

None of our patients achieved stable remission with high-dose corticosteroids and methotrexate only. Anti-TNFa showed to be beneficial in refractory patients with clinical improvement in the majority of the cases, as previously reported in other studies.

**Disclosure of Interest**

None Declared

### P230 CHILDHOOD TAKAYASU ARTERITIS AFTER ALLOGENEIC HEMATOPOIETIC STEM CELL TRANSPLANTATION FOR CORRECTION OF HYPER-IGE SYNDROME - CASE PRESENTATION AND REVIEW OF LITERATURE

#### Christiane Reiser^1^, Andreas Kurringer^1^, Antonius Schuster^2^, Anita Lawitschka^3^, Jasmin B Kuemmerle-Deschner^4,5^, Christian Huemer^1^

##### ^1^Department of Pediatrics; ^2^Department of Radiology, Landeskrankenhaus Bregenz, Bregenz; ^3^Department of Pediatrics, University hospital Vienna, Vienna, Austria; ^4^Department of Pediatrics, University hospital Tuebingen; ^5^Autoinflammation Reference Center Tuebingen, Tuebingen, Germany

###### **Correspondence:** Christiane Reiser

**Introduction:** Childhood Takayasu (c-TA) as a rare large vessel vasculitis remains challenging from the initial diagnostics to the managment of the disease, accordingly morbidity and mortality remains high. In literature, there exist some promising reports of treating otherwise treatment-resistant systemic vasculitis with autologous peripheral blood stem cell transplantation.

**Objectives:** To demonstrate the difficulty of managing c-TA after allogeneic stem cell transplantation due to Hyper-IgE Syndrome

**Methods:** Case report

**Results:** Here, we report on the case of a 10 year old boy who developed c-TA several months after allogeneic hematopoietic stem cell transplantation having received for the correction of a severe Hyper-IgE-Sydrome. At the time of diagnosis of c-TA the patient was off systemic immunosuppressive treatment, with stable donor chimerism and without any clinical evidence of graft versus host disease.

The management of the immunosuppressive therapy was challenging, but the introduction of infliximab resulted in stabilisation of the disease course.

**Conclusion:** The difficulty of monitoring disease activity due to lack of specific biomarkers, the importance of radiological follow-up, the common complications and the non-steroidal treatment options will be discussed here. Informed consent to published had been obtained from the parents.

**Disclosure of Interest**

None Declared

### P231 WHAT THE VARIABILITY AND CYCLES IN KAWASAKI DISEASE (KD) ARE TELLING US ON THE NATURE OF THIS PEDIATRIC VASCULITIS

#### Xavier Rodó^1,2^, Silvia Borras^1^, Tomoko Kojima^3^, Roger Curcoll^4^, Joan Ballester^1^, Teresa Moreno^5^, Atsushi Matsuki^6^, Josep-Anton Morguí^4^ and International Kawasaki Disease Consortium

##### ^1^Climate and Health Program, ISGlobal (Barcelona Institute of Global Health); ^2^ICREA, Barcelona, Spain; ^3^Kumamoto University, Kumamoto, Japan; ^4^Land, Atmosphere and Oceans Lab, ICTA, UAB, Bellaterra; ^5^Institute for Environmental Assessment and Water Studies (IDÆA), Spanish National Research Council (CSIC), Barcelona, Spain; ^6^Institute of Nature and Environmental Technology, Kanazawa University, Kanazawa, Japan

###### **Correspondence:** Xavier Rodó

**Introduction:** Kawasaki disease (KD) epidemiology and its etiology are the subject of vivid debates after more than 50 years of its initial characterization by T. Kawasaki. Among the many causes invoked, environmental as well as genetic studies pointed to incomplete views on what is still today, a medical mystery.

**Objectives:** The identification of regular variability cycles modulating population epidemiology of KD in Japan at temporal scales ranging from seasonality to interanual cycles and their close association to variability in wind dynamics, raised new questions and altered our former understanding of KD. At seasonal timescales,wind intensity and wind direction closely mimick variability in KD. At longer scales, regional climate features, like interhemispheric winds in the north and midlatitude Pacific Ocean and the El Niño-Southern Oscillation (ENSO) phenomena seem to play a dominating role. However, despite daily dynamics were also proved to be related between local winds and hospital case admissions, variability at these fast time scales has never been studied in relation to local air physics and air quality

**Methods:** Machine learning data clustering and a suite of classification techniques were applied to both KD incidence and environmental datasets in Kumamoto (Japan), during two intervals covered with intensive daily surveillance. KD incidence, physical air determinants and air chemistry factors are pooled together in these analyses.

**Results:** Interestingly, new associations emerge that strongly link the nature of air masses to very high frequency cycles in KD. Results are supported by analyses reproduced during different synoptic weather conditions and among typologies of KD epidemiological events, suggesting a clear role of air determinants in the population epidemiology of this vasculitis.

**Conclusion:** Air quality and its dynamics should be considered as a determinant factor of enhanced KD incidence in Japan. These results may be the basis for widening our long-standing views on this paradigmatic disease, and be useful in the case of other similar diseases. Specific surveys aimed at precisely characterizing these air determinants are urgently needed to unravel what constellation of environmental factors seem to be modulating KD incidence.

**Disclosure of Interest**

None Declared

### P232 DYSREGULATION OF B AND TFH CELLS FUNCTIONS IN DADA2 PATIENTS.

#### Francesca Schena^1^, Claudia Pastorino^1^, Federica Penco^1^, Stefano Volpi^1^, Roberta Caorsi^1^, Francesca Kalli^2^, Daniela Fenoglio^2^, Annalisa Salis^3^, Ignazia Prigione^1^, Paola Bocca^1^, Francesca Antonini^4^, Alice Grossi^5^, Gianluca Damonte^3^, Isabella Ceccherini^5^, Gilberto Filaci^2^, Alberto Martini^1^, Elisabetta Traggiai^6^, Marco Gattorno^1^

##### ^1^Second Pediatric Division and Centro malattie autoinfiammatorie e immunodeficienze, ISTITUTO GIANNINA GASLINI, Genova; ^2^Department of Internal Medicine; ^3^3 Department of Experimental Medicine, Center of Excellence for Biomedical Research; ^4^Core Facilities Flow-Cytometry and Cell imaging Lab; ^5^Medical Genetics, ISTITUTO GIANNINA GASLINI, Genoa, Italy; ^6^Novartis Institutes for Biomedical Research, Basel, Switzerland

###### **Correspondence:** Francesca Schena

**Introduction:** ADA2 Deficiency is a new autoinflammatory disease characterized by systemic vasculopathy and episodes of strokes, but some patients can present mild immunodeficiency. The defect is due to a loss of function mutation of *CECR1* gene, coding for Adenosine Deaminase 2 protein. This protein regulates the catabolism of extracellular adenosine, which we have shown is an important regulator of Class Switch Recombination in B lymphocytes.

**Objectives:** ADA2 Deficiency is a new autoinflammatory disease characterized by systemic vasculopathy and episodes of strokes, but some patients can present mild immunodeficiency. The defect is due to a loss of function mutation of *CECR1* gene, coding for Adenosine Deaminase 2 protein. This protein regulates the catabolism of extracellular adenosine, which we have shown is an important regulator of Class Switch Recombination in B lymphocytes.

**Methods:** 14 patients carrying mutations in *CECR1* were examined. They showed clinical history with livedo reticularis, fever, vasculitis and neurological symptoms. 6 patients presented hypogammaglobulinemia or recurrent infections. We analyzed peripheral B and T cell phenotype by flow cytometry. B cells isolated from healthy donors and DADA2 patients have been cultured alone or in co-culture with CD4+ T cells and *in vitro* B cell proliferation has been evaluated by CFSE dilution. *In vitro* B cell differentiation to Immunoglobulin secreting cells in response to TLR9 agonist and T cell help has been evaluated by ELISA/ELISPOT assay. Moreover cytokines production and CD40L expression from patients T cells have been evaluated.

**Results:** Flow cytometric analysis showed a significant reduction of memory B cell compartment (CD19+CD27+) in DADA2 patients, in particular in Swich memory B cell subset.

We also observed a reduced absolute number of CD4+ and CD8+ T cells, whereas the subpopulations of T cells resulted normal, with the exception of TEMRA CD8+ T cells, significantly expanded in DADA2 patients. Moreover we identified an expansion of circulating Tfh cells in DADA2. Then we investigated a role of ADA2 in B cells: we found that ADA2 is expressed, secreted by B cells, but the enzymatic activity is impaired in patients’ B cells that show functional impairment in proliferation and differentiation.

We addressed *in vitro* the interaction of B and T cells, essential for generation of memory.

Analysis of proliferation and differentiation showed that proliferation of patients’ B cells is not sustained from patient’s T cells and IgG secretion is significantly reduced in the presence of CD4+ T cells obtained from DADA2 patients with respect to normal CD4+ T cells. Moreover T cells from DADA2 show an impairment in the production of IL21, key cytokine for B cell help and a significantly downregulation of CD40L.

**Conclusion:** Our findings suggest that CECR1 mutation could affects directly B cell function and leads also to an impairment of T cell help functions.

**Disclosure of Interest**

None Declared

### P233 A FAMILY CASE OF ADA2 DEFICIENCY WITH CECR1 MUTATION

#### Caroline Schnider^1^, Katerina Theodoropoulou^1^, Fabio Candotti^2^, Federica Angelini^1^, Matthieu Perreau^2^, Orbicia Riccio^2^, Michael Hershfield^3^, Michael Hofer^1^

##### ^1^Pediatric Immuno-Rheumatology; ^2^immuno-allergology, CHUV, Lausanne, Switzerland; ^3^Rheumatology, Medecine, Duke, Durham, USA

###### **Correspondence:** Caroline Schnider

**Introduction:** ADA2 deficiency (DADA2) is an immunological disease caused by autosomal recessive mutations in the *CERC1* gene coding for ADA2 enzyme. DADA2 is causing various and heterogeneous manifestations, including systemic inflammation, vascularitis, thrombotic events, cytopenias, immune deficiency, and autoimmunity. Timely diagnosis and treatment are crucial to prevent cerebral strokes as well as other multisystemic serious complications of the disease.

**Objectives:** We describe here the case of a 4-year old Caucasian child diagnosed with DADA2 in the context of positive family history.

**Methods:** Case report.

**Results:** This 4-year-old boy, originating from Portugal, presented with persistent fever in the last 3 months, lower limbs pain, systemic inflammation, anaemia, livedo reticularis, and hypogammaglobulinemia M and A. The personal history showed failure to thrive since the age of 2 years. Family history revealed consanguinity and a fourth degree relative with the diagnosis of DADA2 due to a homozygous R169Q mutation. After exclusion of infectious and oncologic causes, targeted genetic analysis was performed and showed the presence of the same R169Q mutation with enzymatic tests confirming the absence of ADA2 activity. After diagnosis, oral steroids were introduced with remission of symptoms and normalization of inflammatory markers within 3 weeks and was followed by anti-TNF alpha therapy. Genetic testing of the parents is ongoing.

**Conclusion:** DADA2 diagnosis may be challenging with no specific symptoms at presentation; the presence of livedo, hypogammaglobulinemia and thrombotic events should raise the awareness of physicians. We describe here a case of DADA2 promptly diagnosed due to the positive family history. Early diagnosis of DADA2 is essential to prevent serious complications of the disease, highlighting the importance of genetic testing in all family members. However, the management of presymptomatic patients remains a matter of controversy. In the absence of biomarkers predicting the onset of the disease, a close and regular follow-up is recommended in case of the mutation. Written informed consent was obtained from the patients for publication.

**Disclosure of Interest**

None Declared

### P234 CLINICAL AND HISTOPATHOLOGICAL PROGNOSTIC FACTORS AFFECTING THE RENAL OUTCOMES IN CHILDHOOD ANCA-ASSOCIATED VASCULITIS

#### Gül Özçelik^1^, Hafize E. Sönmez^2^, Sezgin Şahin^3^, Ayşim Özağarı^4^, Meral T. Bayram^5^, Rümeysa Y. Çiçek^6^, Evrim K. Çakıcı^7^, Elif Çomak ^8^, Kenan Barut^6^, Nihal Şahin^9^, Sevcan Bakkaloğlu^10^, İbrahim Gökçe^11^, Ali Düzova^12^, Yelda Bilginer^2^, Ceyhun Açarı^13^, Engin Melek^14^, Beltinge D. Kılıç^15^, Semanur Özdel^16^, Amra Adroviç^3^, Özgür Kasapçopur^3^, Erbil Ünsal^13^, Harika Alpay^11^, Diclehan Orhan^17^, Rezan Topaloğlu^12^, Ruhan Düşünsel^18^, Seza Özen^2^

##### ^1^Department of Pediatric Nephology, SBÜ. Sisli Hamidiye Etfal Training and Research Hospital, İstanbul; ^2^Department of Peditarics, Division of Rheumatology, Hacettepe University Faculty of Medicine, Ankara; ^3^Department of Peditarics, Division of Rheumatology, Cerrahpasa Medical School, Istanbul University; ^4^Department of Pathology, Sisli Etfal Training and Research Hospital, İstanbul; ^5^Department of Pediatric Nephology, Dokuz Eylul University Faculty of Medicine, İzmir; ^6^Department of Pediatric Nephology, Cerrahpasa Medical School, Istanbul University, İstanbul; ^7^Department of Pediatric Nephology, Sami Ulus Training and Research Hospital, Ankara; ^8^Department of Pediatric Nephology, Akdeniz University Faculty of Medicine, İzmir; ^9^Department of Peditarics, Division of Rheumatology, Erciyes University Faculty of Medicine, Kayseri; ^10^Department of Pediatric Nephology, , Gazi University Faculty of Medicine, Ankara; ^11^Department of Pediatric Nephology, Marmara University Faculty of Medicine, İstanbul; ^12^Department of Pediatric Nephology, Hacettepe University Faculty of Medicine, Ankara; ^13^Department of Peditarics, Division of Rheumatology, Dokuz Eylul University Faculty of Medicine, İzmir; ^14^Department of Pediatric Nephology, Çukurova University Faculty of Medicine, Adana; ^15^Department of Pediatric Nephology, Gaziantep University Faculty of Medicine, Gaziantep; ^16^Department of Peditarics, Division of Rheumatology, Sami Ulus Training and Research Hospital; ^17^Department of Pathology, Hacettepe University Faculty of Medicine, Ankara; ^18^Department of Pediatric Nephology, Erciyes University Faculty of Medicine, Kayseri, Turkey

###### **Correspondence:** Hafize E. Sönmez

**Introduction:** Antineutrophil cytoplasmic antibody-associated vasculitides (AAV) are very rare in childhood vasculitides with an increased risk of morbidity and mortality. Five factor score (FFS) is a tool designed to assess prognosis at diagnosis of the patient with systemic necrotizing vasculitides and is found to be a good predictor for survival rate in adult studies.

**Objectives:** This study was aimed to evaluate renal prognostic factors in childhood AAV with the perspective of ANCA serotype, histopathological classification and FFS.

**Methods:** Pediatric AAV patients from 11 referral centers in Turkey, who were followed up between January 2006 and March 2018, were included to study. The demographics, clinical findings, AAV subtypes, outcomes, and FFS were evaluated retrospectively. Kidney biopsies were classified histopathologically according to the Berden classification.

**Results:** Totally 39 patients, including 68.4% GPA, 21.1% MPA and 10.5% EGPA were enrolled in the study. Among all patients, 74.4% had renal involvement, 56.4% ear-nose- throat (ENT) involvement, 51.3% had musculoskeletal involvement.  PR3 ANCA was positive in 48.7% and MPO ANCA was positive in 30.8%. During the follow-up, impaired renal function and end-stage-renal-disease (ESRD) were assessed in 69.2% and 28.2%, respectively. At the diagnosis, FFS was ≥ 2 in 53.8%. The most common histopathologic classifications were as follows; crescentic type in 40.7%, sclerotic type in 25.9%. ETN and renal involvement statistical significance among the AAV subgroups (p <0.001, p = 0.001, respectively). Presence of PR3-ANCA antibody was higher in the GPA group. Gastrointestinal and renal involvement, MPO-ANCA positivity, serum creatinine levels and impaired renal function during the follow-up were significantly higher in patients with FFS ≥ 2, compared to patients with FFS <2. Patients with FFS ≥ 2 had more common crescentic, mixed and sclerotic histopathologic findings in biopsies. By logistic regression analysis forward method, the strongest single-risk factor among all the parameters was the initial level of creatinine in patients with ESRD, compared to the other patients (p = 0,007)

**Conclusion:** We have presented the association between poor histopathologic findings and MPO-ANCA positivity, impaired renal function and FFS ≥ 2 at the diagnosis. The most important predictor factor for renal prognosis was initial serum creatinine level.  In conclusion, evaluation of the FFS, ANCA serology and the creatinine levels may help to predict renal prognosis.

**Disclosure of Interest**

None Declared

### P235 TWO CASES OF REFRACTORY KAWASAKI DISEASE SUCCESSFULLY TREATED WITH ANAKINRA

#### Francesca Tirelli, Ilaria Maccora, Teresa Giani, Gabriele Simonini, Rolando Cimaz

##### Rheumatology Unit , University of Florence, Meyer Children's Hospital, Neurofarba Department, Florence , Italy

###### **Correspondence:** Francesca Tirelli

**Introduction:** Kawasaki Disease (KD) is a self-limited childhood vasculitis of unknown origin and coronary artery aneurisms are its most significant complication. Timely treatment with Intravenous Immunoglobulin (IVIG) represents the standard care, but more than 10% of children are unresponsive. Many treatment options have been reported, but the most effective therapy for refractory cases is still not defined. Although mice models have suggested a significant role of Il-1 in the pathogenesis of KD and coronary artery aneurisms, treatment with Interleukin - 1 Receptor Antagonist (Il - 1 RA) has been seldom used.

**Objectives:** We report two patients with refractory KD who achieved remission with Anakinra following IVIG and systemic corticosteroid failure.

**Methods:** Describing case series.

**Results:** Case #1 is a previously healthy four-month-old male, presenting for high fever not responsive to empiric antimicrobial therapy, marked irritability and poor feeding, bilateral conjunctivitis, micropapular rash involving face and extremities, keilitis, perianal hyperemia and mild hand and feet edema. KD was diagnosed and the patient was started on aspirin and IVIG on day 5^th^ from the onset of fever; a second dose of IVIG was administered two days later because of persistent fever. Despite this treatment, his general conditions deteriorated and cutaneous rash worsened significantly, evolving into necrotic-hemorragic vasculitic lesions. Pulse methylprednisolone was started on day 8^th^, followed by high dose oral prednisone. Ten days later the patient relapsed and a third IVIG infusion was administered. Seriated echocardiographies showed from the 23^rd^day of illness a marked dilation of both right and left coronary arteries.

Case #2 is a previously healthy 4-year-old male who was admitted to General Pediatric Unit because of 4 days high-grade fever, feeding difficulties, conjunctival injection, cervical adenopathy, mucositis, abdominal rash and palm and sole erythema. KD was diagnosed, aspirin was started and 2 IVIG infusions were administered without improvement. Seriated echocardiographies showed coronary involvement with mild dilation of left coronary artery. From the 8^th^day of fever, 3 pulses of methylprednisolone were administered, followed by oral prednisone, with initial improvement. Four days later he was febrile again and in poor general conditions with severe irritability.

Given the resistance to IVIG and corticosteroids, IL1 - RA (anakinra) was started in both patients at the dose of 2 mg/kg once a day, subcutaneously. They both showed a rapid improvement; particularly, patient #1 showed a gradual resolution of the severe cutaneous vasculitic lesions. Two weeks after starting biological therapy, both patients could be discharged from hospital on anakinra, tapering oral corticosteroid. Treatment with anakinra was maintained for 1 month in both children and no relapses occurred after its discontinuation; patients did not experience any side effect during therapy.  Echocardiographic controls showed progressive reduction of coronary dilation with complete resolution after 5 months in patient #1 and after 6 weeks from the onset of KD symptoms in patient #2.

**Conclusion:** Our case series suggests that treatment with IL-RA may be considered as an effective and safe therapeutic option for refractory KD. Informed consent to publish was obtained from the parents.

**Disclosure of Interest**

None Declared

### P236 SECRETORY IMMUNOGLOBULIN A IN PEDIATRIC HENOCH-SCHÖNLEIN PURPURA: A PROTECTING ROLE AGAINST BACTERIAL TRANSLOCATION?

#### Tu Anh Tran^1,2^, Renaud Cezar^2,3^, Pierre Corbeau^3^, Marc Fila^4^, Eric Jeziorski^5^, Nastassja Protsenko^1^, Jean Philippe Lavigne^6,7^, Catherine Remy^6,7^, Anne Filleron^1,2^

##### ^1^Pediatrics, Nîmes University hospital , Nîmes; ^2^INSERM U1183, Montpellier University, Montpellier; ^3^Laboratory of Immunology, Nîmes University hospital, Nîmes; ^4^Pediatric Nephrology; ^5^Pediatric Infectious Disease, Montpellier university hospital, Montpellier; ^6^Laboratory of Bacteriology, Nîmes University hospital; ^7^INSERM U1047, Nîmes, France

###### **Correspondence:** Tu Anh Tran

**Introduction:** Henoch-Schönlein purpura (HSP) is the first cause of systemic vasculitis in childhood, characterized by immunoglobulin A (IgA) deposits in small vessels. Two-thirds of children have abdominal involvement with at least abdominal pain due to sub-mucosal hemorrhage and edema of the intestinal walls.

**Objectives:** We report data on microbial translocation, as well as serum and in stool IgA levels in pediatric Henoch-Schönlein purpura.

**Methods:** Three groups of children, 3 to 15.6 years old, were included: group A, acute HSP (n=30); group B, remitting HSP (n=30); group C, healthy controls matched for age (n=38). Bacterial translocation was evaluated by measuring plasma bacterial 16S rDNA levels using quantitative PCR. Serum IgA levels were quantified by immunonephelometry. Secretory IgA (sIgA) concentrations in stools were measured by ELISA.

**Results:** 63% and 73% of children in group A and B, respectively, presented with gastrointestinal manifestations. Mean 16S rDNA levels tended to be lower in A (7.90 [SD, 3.72] cp/μl) and B (9.84 [SD, 5.11] cp/μl) than in C (12.45 [SD, 8.1630] cp/μl). Mean serum IgA levels were significantly higher in A (1.95 [SD, 0.84] mg/mL) than in B (1.20 [SD, 0.53] mg/mL, p=0.02) and C (0.94 [SD, 0.77] mg/mL, p=0.0001). By contrast, sIgA levels were significantly lower in A (3.81 [Sd 8.40] mg/mL) than in C (6.25 [Sd 10.84] mg/mL, p=0.045).

**Conclusion:** Bacterial translocation was lower in HSP children than in healthy subjects. Serum IgA were higher during acute HSP, while secretory IgA levels were paradoxically reduced in stools. These data provoke the hypothesis that IgA overproduction in HSP might prevent microbial translocation. The low stool sIgA levels observed in acute HSP might be the consequence of IgA consumption during this process. Further studies are necessary to test this hypothesis. Informed consent to publish had been obtained.

**Disclosure of Interest**

None Declared

### P237 RENAL AUTO-TRANSPLANTATION IN A PATIENT WITH TAKAYASU ARTERITIS

#### Ece Mekik^1^, Fatma Aydın^1^, Acar Tüzüner^2^, Suat Fitöz^3^, Seray Akbağ^1^, Eda D. Kurt-Şükür^1^, Umman Şanlıdilek^4^, Elif Çelikel^1^, Zeynep B. Özçakar^1^, Nilgün Çakar^1^, Fatoş Yalçınkaya^1^

##### ^1^Pediatric Nephrology&Rheumatology Department; ^2^General Surgery Department; ^3^Pediatric Radiology Department; ^4^Radiology Department, Ankara University School of Medicine, Ankara, Turkey

###### **Correspondence:** Fatoş Yalçınkaya

**Introduction:** Takayasu Arteritis (TA) is a large vessel vasculitis which primarily affects the aorta and its primary branches. Renal arterial involvement is common in TA. In severe cases, it can cause total occlusion of renal arteries.

**Objectives:** We hereby present a case of Takayasu arteritis causing bilateral renal artery stenosis, leading renal failure and treated with renal auto-transplantation (RAT).

**Methods:** A case with Takayasu Arteritis and bilateral renal artery stenosis were presented.

**Results:** A 17-year old male patient applied to our hospital with swelling in the periorbital area and legs. On physical examination, he had periorbital and pretibial edema and periumblical bruit. His blood pressure was 185/115 mmHg on both upper and 240/160 mmHg on lower extremities. Initial creatinine level was 1.19 mg/dL; ESR and CRP levels were 63 mm/h and 52.4 mg/L, respectively. Renal doppler ultrasonography demonstrated total occlusion of the left renal artery, severe stenosis of right renal artery (nearly occluded) and vascular wall thickening of the abdominal aorta and superior mesenteric artery (SMA). Magnetic resonance (MR) angiography showed wall thickening and perivascular inflammation of aorta and its major branches which is consistent with Takayasu arteritis. Significant narrowing in SMA and right renal artery, total occlusion of left renal artery and T2 signal decrease in left kidney suggested diffuse ischemia and infarct. Accordingly, he was diagnosed as Takayasu arteritis, and given pulse methylprednisolone (PMP) treatment immediately at his first day of admission. However within 12 hours, he became anuric, his edema increased, and creatinine level elevated to 4 mg/dL. Urgent hemodialysis (HD) was performed and due to the rapid progression of the disease intravenous pulse cyclophosphamide (CYC) was added. Despite daily HD and intense immunosuppressive therapy with CYC and 5 day course PMP, creatinine levels continued rising (7 mg/dL). On the sixth day of admission, thoracoabdominal aortography was performed but the attempt for revascularization of renal arteries by balloon angioplasty was unsuccessful. Considering his severe renal failure and inadequate response to the medical and endovascular interventions, RAT was performed. His relatively preserved right kidney was dissected and directly anastomosed to right external iliac artery. Postoperatively, diuresis started immediately, creatinine levels decreased slightly right after surgery, and returned to normal levels (0.99 mg/dL) on post-operative day 10. Immunosuppressive therapy was continued with 60 mg/day oral corticosteroid (CS) and second dose of CYC was given 3 weeks after RAT.

**Conclusion:** This case shows that early diagnosis and immediate appropriate interventions are life-saving in TA. In experienced transplant centers, RAT performed in selected cases is a kidney-saving method and provides dialysis-free-survival. Informed consent to publish had been obtained from the parents.

**Disclosure of Interest**

None Declared

## JIA (oligo, poly, psoriatic)

### P238 THE CLINICAL SIGNIFICANCE OF SERUM SCD30/SCD26 RATIO IN PATIENTS WITH JUVENILE IDIOPATHIC ARTHRITIS

#### Mao Mizuta^1^, Masaki Shimizu^1^, Naoto Sakumura^1^, Hitoshi Irabu^1^, Maiko Takakura^1^, Natsumi Inoue^1^, Yasuo Nakagishi^2^, Akihiro Yachie^1^

##### ^1^Department of Pediatrics, Graduate School of Medical Sciences, Kanazawa University, Kanazawa; ^2^Department of Pediatric Rheumatology, Hyogo Prefectural Kobe Children’s Hospital, Kobe, Japan

###### **Correspondence:** Mao Mizuta

**Introduction:** Juvenile idiopathic arthritis (JIA) is known as an autoimmune disease and the severity of inflammation in arthritic joints is thought to be due to the imbalance of pro-inflammatory and anti-inflammatory cytokines. Pro-inflammatory type 1 helper (Th1) and anti-inflammatory type 2 helper (Th2) T cell activity are shown as serum levels of soluble CD26 (sCD26) and sCD30.

**Objectives:** The aim of this study is to assess serum levels and clinical significance of sCD26, sCD30 and sCD30/sCD26 ratio in patients with JIA.

**Methods:** Forty eight JIA patients (19 patients with RF+ poly-articular JIA (p-JIA), 8 with RF- p-JIA, and 21 with oligo-articular JIA (o-JIA)) and 9 healthy controls (HCs) were enrolled. Serum levels of sCD26 and sCD30 were measured using enzyme linked immunosorbent assay. Furthermore, we investigated the correlation between these levels and clinical manifestations.

**Results:** In JIA patients, serum CD26 levels were lower and serum CD30 levels were higher compared to HCs, although not statistically significant. In RF+ and RF- p-JIA patients, serum sCD30/ sCD26 ratio was significantly elevated compared to HCs in active phase. Serum sCD30/ sCD26 ratio was significantly decreased in inactive phase compared to active phase. Furthermore, in RF+ p-JIA patients, serum sCD30/sCD26 ratio was correlated with arthritic disease activity including tender joint counts, swollen joint counts and serum levels of matrix metalloprotease-3. On the other hand, In RF- p-JIA and o-JIA patients, those correlation were not observed.

**Conclusion:** The serum sCD30/sCD26 ratio might be a useful biomarker for disease activity and joint inflammation in patients with RF+ p-JIA. Increasing Th2 cell activity might have an important role in the pathogenesis of RF+ p-JIA.

**Disclosure of Interest**

None Declared

### P239 PHYSICAL ACTIVITY IN PATIENTS WITH JUVENILE IDIOPATHIC ARTHRITIS AND JUVENILE SISTEMIC LUPUS ERYTHEMATOSUS: IMPACT ON THE HEALTH AND ACTIVITY OF THE DISEASE. COLOMBIA.2017

#### Pilar Perez, Catalina Mosquera, Clara Malagon

##### RHEUMATOLOGY PEDIATRIC, EL BOSQUE UNIVERSITY, BOGOTA, Colombia

###### **Correspondence:** Catalina Mosquera

**Introduction:** The benefits of physical activity are multiple and are enhanced when it is done routinely^1^. Pediatric patients with rheumatological diseases develop chronic proinflammatory conditions, which causes an increase in the burden of disease and the appearance of comorbidities such as overweight, obesity and cardiovascular disease that, associated with sedentary lifestyle, increases morbidity and mortality in the medium and long term^2^. Studies in the population with juvenile idiopathic arthritis (JIA) and juvenile systemic lupus erythematosus (SLEj) show that these children are less active than their healthy counterparts, causing an increase in the rates of disease and damage^3^

**Objectives:** Quantify the physical activity performed by patients with JIA and SLEj treated in the pediatric rheumatology consult of a pediatric service in the city of Bogotá and in a population without rheumatological diseases in the first half of 2017

**Methods:** Analytical cross-sectional study of patients with JIA, SLEj and without rheumatological pathologies. Physical activity was evaluated with the validated physical activity questionnaire for children (PAQ - C, for children under 13 years old) or adolescents (PAQ - A, age 14-16 years), scoring tool from 0 (without physical activity) to 5 (intense physical activity). The patients completed the child health questionnaire (CHAQ) and assessed activity and damage associated with the disease.

**Results:** The study included 95 patients with rheumatologic disease (JIA: 52 patients and SLEj: 43 patients) and 100 healthy individuals. Average age of presentation of the disease 13.5 years (SD 3.1 years range 4 - 18 years). Regular physical activity was performed by 28.8% of patients with JIA, 19% without pathology and 13.9% of patients with SLEj. The median physical activity (PAQ) in individuals with JIA was 1.8 (SD: 1.0, range: 1 to 5), 1.6 (SD: 0.79, range: 1 to 4) for patients with SLEj and 1.59 (SD: 0.911, range: 1 to 4) for the population without rheumatological pathology, the 3 groups of patients had a very low intensity in physical activity. Patients with JIA in this series had activity indexes measured by JADAS10 with a median of 7 (SD: 7.7, range: 0 -33) and patients with SLEj, with an activity index measured by SLEDAI with a median of 1.8 (SD 1.9). , range: 0-6). The damage index in patients with SLEj was reported with a median of 0.37 (SD: 1.15, range: 0 - 7) and in patients with JIA the index of joint damage was reported with a median of 0.94 (SD: 2.3, range: 0-11) and the average extra-articular damage index was 0.32 (SD: 1.09, range: 0-5). There was a low inverse correlation between the PAQ activity score and the activity and damage indexes for JIA and SLEj, respectively. The average of sedentary hours was 3.3 both for patients with pathology and for individuals without any disease. The most frequent cause of physical inactivity in all patients was lack of time. Overweight was documented in 25% of patients with SLEj, 19% in JIA and 11% of the population without rheumatic disease. 6% of patients without rheumatic disease were classified in the obesity range in contrast to 2% of patients with JIA and SLEj.

**Conclusion:** Both the population with rheumatological diseases and individuals without documented disease have low rates of regular physical activity. In this group of patients with JIA and SLEj the high rates of physical activity are related to lower rates of both disease activity and damage.

**Disclosure of Interest**

None Declared

### P240 REFERRAL PATTERNS FOR TMJ SCREENING IN JIA PATIENTS FROM EASTERN DENMARK: A POPULATION BASED STUDY DESCRIBING A 21 YEAR PERIOD

#### Marek S. Zak^1^, Sarah Kathem^2^, Ib J. Christensen^2^, Susan Nielsen^1^, Sven Kreiborg^2^

##### ^1^Paediatric Rheumatology Unit, Copenhagen University Hospital, Rigshospitalet; ^2^Special Clinic for Congenital and Acquired Craniofacial Anomalies at Department of Odontology, Faculty of Health and Medical Sciences, University of Copenhagen, Copenhagen, Denmark

###### **Correspondence:** Susan Nielsen

**Introduction:** Juvenile Idiopathic Arthritis (JIA) patients from Eastern Denmark, a well-defined geographic area comprising of the Copenhagen municipality and adjacent rural districts including smaller cities, villages and islands located in the eastern part of the country where about 45% (2.5 mill.) of the Danish population live, are at the time of diagnosis routinely referred to regular temporomandibular joint (TMJ) evaluation at the Special Clinic for Congenital and Acquired Craniofacial Anomalies at Department of Odontology, Faculty of Health and Medical Sciences, University of Copenhagen.

**Objectives:** The objectives for the current analysis were to characterise the referral patterns during a 21 year observation period from 1996 to 2016.

**Methods:** Data collection was based on dental and medical records including demographics and JIA related variables. In order to look for trends, the 21 year period was divided into three consecutive 5 year periods and a 6 year period (2011-2016).

**Results:** In total 1472 JIA patients (female/male = 959/513 (65%/35%)) with a median age at diagnosis of 7.7 years; age range: 0-18 years (1^st^ quartile 3.5, 3^rd^ quartile 11.3) were referred to regular TMJ screening. A steady increase in the number of referred new JIA cases was observed during the observation period: 1996-2000: N=203; 2001 – 2005: N=221; 2006 – 2010: N=330; and 2011 – 2016: N=715. Throughout the observation period the gender ratio, age at onset and ILAR subtype distribution were significantly altered. The proportion of males increased (p=0.006), age at onset decreased (p=0.003) and ILAR subtype was altered (p<0.001). In addition, the fraction of patients with TMJ involvement fell dramatically from 28 to 9% (p<0.001). A similar decline was observed in the fraction of patients with uveitis (p=0.002).

The analysis suggests more than doubling incidence of JIA in Eastern Denmark over a 21 year period. This could be attributed to increased awareness and centralisation of JIA treatment during the period, but possibly also other factors as gradual introduction of musculoskeletal ultrasound (MSK US) over the observation period possibly resulting in JIA diagnoses in “borderline” mild cases that previously would not have fulfilled the criteria for a JIA diagnosis. An increase in the fraction of very mild cases is consistent with unchanged absolute numbers of patients with TMJ involvement and patients with uveitis. Availability of improved JIA treatment options could potentially also have contributed to an increased fraction of milder cases.

**Conclusion:** Although the current study suggests dramatically increasing incidence of JIA in Eastern Denmark, the results must be interpreted with great caution due to potential confounding factors including increased JIA awareness and new technological developments in diagnostic tools e.g. MSK US. The data suggests that due to increasing use of MSK US we may be diagnosing many more mild JIA cases that previously would not have been diagnosed with JIA.

**Disclosure of Interest**

None Declared

### P241 THE PHYSICIAN GLOBAL ASSESSMENT OF DISEASE ACTIVITY IN JUVENILE IDIOPATHIC ARTHRITIS IS NOT CONSISTENT ACROSS PHYSICIANS OF PEDIATRIC RHEUMATOLOGY IN SWEDEN

#### Soley Omarsdottir, Bo Magnusson and The Board of the Paediatric Rheumatology Registry

##### Astrid Lindgren Children´s Hospital, Karolinska University Hospital , The Swedish Paediatric Rheumatology Registry, Stockholm, Sweden

###### **Correspondence:** Soley Omarsdottir

**Introduction:** Physician global assessment (PGA) is considered an important responsive measure of disease activity in juvenile idiopathic arthritis (JIA) and is included in the composite juvenile arthritis disease activity score. However, studies of inter-observer agreement of PGA are scarce and guidelines for rating PGA in JIA are lacking.

**Objectives:** To evaluate the consistency of PGA scoring across physicians treating children with JIA in Sweden.

**Methods:** Twenty fictive JIA cases were introduced orally and in written to 26 physicians attending an annual national Swedish meeting of pediatric rheumatology. A mentimeter was used to collect information about the physicians and the PGA rating score for the cases presented. In order to mimic ratings on a 10-cm visual analoug scale, participants were allowed to chose a score between 0 and 100, where 0=no activity and 100=maximum activity.

**Results:** Most physicians were specialists, women, aged > 55 years and worked at county hospitals. Eighteen (69%) had more than 8 years experience of pediatric rheumatology. The PGA scores for some cases presented varied considerably, the median of differences in PGA scores across physicians was 70 (range 5-86). The distribution of the medians of the physicians' individual PGA scores also differed, the mean was 42.5 (range 20-86). Only in a few cases, PGA score was significantly affected by physicians' age (1 case), place of work (4 cases), profession (1 case) or pediatric rheumatology experience (1 case). Physicians' characteristics did not affect distribution of the individual scores significantly.

**Conclusion:** PGA scores of cases with JIA were not consistent across physicians treating JIA patients in Sweden. Establishment of guidelines for PGA rating in JIA would be valuable in order to create more congruent disease activity evaluation across physicians.

**Disclosure of Interest**

None Declared

### P242 THE VACCINATION STATUS OF PATIENTS WITH JUVENILE IDIOPATHIC ARTHRITIS

#### Paulina Frańczak-Chmura, Maria B. Tomaszek, Aleksandra Sobiesiak, Violetta Opoka-Winiarska

##### Department of Pediatric Pulmonology and Rheumatology, Medical University of Lublin, Poland, Lublin, Poland

###### **Correspondence:** Violetta Opoka-Winiarska

**Introduction:** Disorders of immune system as well as immunosuppressive treatment of patients with rheumatic diseases increase risk and severe course of infections. Safe and effective vaccinations are key to preventing many infections. Vaccinations are often postponed or overlooked in patients with rheumatic diseases, both children and adults.

**Objectives:** The aim of this study was to check vaccination status of children with JIA from one rheumatology centre in Poland.

**Methods:** Status of vaccination in children with JIA treated at the Rheumatology Clinic in Lublin was tested based on child vaccination books. It was recorded using a questionnaire together with date of the disease.

**Results:** Study included 53 patients with JIA diagnosed. Their mean age was 9,94 years (range 2 – 16) and the mean age of diagnosis 5,72 years. All children in the time of the survey were on DMARDs, 28 % (15/53) on biologic DMARDs (9 – ETA, 4-TCZ, 2-ADA). Until the disease was diagnosed, all patients were vaccinated according to the Polish immunization program (mandatory vaccination), while in the time of the survey only 49% (26/53) of children have completed all mandatory vaccinations on schedule. The most often postponed vaccination was MMR (9/27). Other vaccinations recommended by EULAR and national program (paid by parents) were rarely used. Only 26,4% of patients received pneumococcal vaccination, 9,4% meningococcal and 1,7% - influenza vaccine. Disease exacerbation and disseminated infection were physician and parents' main concern.

**Conclusion:** Vaccination status of children with JIA is low despite EULAR [1] and Polish recommendation [2]. The educational programs are still needed for parents and physicians on the safety and efficacy of vaccinations of children with rheumatic diseases.

**References**

1. Heijstek MW, Ott de Bruin LM, Bijl M et al. EULAR recommendations for vaccination in paediatric patients with rheumatic diseases. Ann Rheum Dis. 2011; 70:1704-12. 2. Opoka-Winiarska V. Vaccination in paediatric and adult patients with rheumatic diseases. Stand Med, Pediatr 2017;14:77-84.

**Disclosure of Interest**

None Declared

### P243 THE NEXT STEP TOWARDS TREAT-TO-TARGET MANAGEMENT OF JUVENILE IDIOPATHIC ARTHRITIS: THE CLINICAL SIGNIFICANCE OF ENDOTHELIAL PERMEABILITY IN THE ASSESSMENT OF DISEASE ACTIVITY

#### Krzysztof Orczyk, Elzbieta Smolewska

##### Department of Pediatric Rheumatology, Medical University of Lodz, Lodz, Poland

###### **Correspondence:** Krzysztof Orczyk

**Introduction:** Achieving the optimal balance between the benefits of early aggressive therapy and the possible risk of overtreatment is the crucial objective of the future management of children freshly diagnosed with juvenile idiopathic arthritis (JIA). Serological biomarkers have become a promising perspective for appropriate assignment of patients into separate therapeutic approaches in accordance with the treat-to-target concept. Studies involving rheumatoid arthritis (RA) patients suggest the potential predictive value of the marker of endothelial permeability called vascular endothelial cadherin (VE‑cadherin).

**Objectives:** The aim of the study was to evaluate the potential usefulness of measuring serum levels of VE‑cadherin in diagnosing and monitoring disease activity in JIA patients.

**Methods:** Serum concentrations of VE-cadherin were measured in 80 patients (mean age 10.4±4.4 years) diagnosed with JIA (excluding systemic subtype) and 29 age- and sex-matched healthy controls. Blood samples were obtained twice in 53 JIA patients (with average interval of 102.4±26.0 days) in order to assess dynamics of VE‑cadherin levels. Database included patients’ history and laboratory tests results.

**Results:** Serum levels of VE-cadherin were higher in the study group than in healthy controls (6.69±2.13 ng/ml vs 5.13±2.86 ng/ml, p<0.001). However, the difference was not statistically significant (p=0.11) for the results obtained at second time point (5.57±2.10 ng/ml). As a diagnostic biomarker of JIA, VE‑cadherin had 87.5% sensitivity and 69.0% specificity for the cutoff level 4.36 ng/ml (Youden index 0.56). VE-cadherin concentrations were independent of JIA subtype. In 40 patients (75.5%) VE‑cadherin level was decreased at second time point (mean difference 1.27±1.06 ng/ml). In 29 of them (72.5%) the reduction of JADAS27 was observed as well.

VE-cadherin concentrations at baseline were significantly decreased in patients using glucocorticosteroids (p=0.00296). Lower VE-cadherin levels at second time point were observed in patients with positive history of both systemic and intraarticular administration of glucocorticosteroids during the first hospitalization (p=0.00485 and p=0.0131, respectively).

**Conclusion:** VE-cadherin can be utilized as a screening test in diagnostic process of JIA. Moreover, it may become a reliable marker of response to treatment. Nevertheless, usage of glucocorticosteroids may reduce its predictive value. Further investigation in more consistent group of patients is necessary to validate the potential clinical use of this biomarker.

**Disclosure of Interest**

None Declared

**Table 1 (abstract P243).** General characteristics of the study group

No. of patients	1^st^ time point (n=80)	2^nd^ time point (n=53)
Female (%)	54 (67.5%)	36 (67.9%)
Oligoarticular JIA (%)	54 (67.5%)	35 (66.0%)
Enthesitis-related JIA (%)	14 (17.5%)	8 (15.1%)
Polyarticular RF-negative JIA (%)	11 (13.8%)	9 (17.0%)
Polyarticular RF-positive JIA (%)	1 (1.2%)	1 (1.9%)
High disease activity (%)	49 (61.3%)	19 (35.8%)
Parent acceptable symptom state (%)	5 (6.3%)	6 (11.3%)
Minimal disease activity (%)	7 (8.7%)	12 (22.7%)
Remission (%)	19 (23.7%)	16 (30.2%)

### P244 ARTICULAR INVOLVEMENT IN PEDIATRIC INFLAMMATORY BOWEL DISEASE (IBD)

#### Carolina Porfito, Francesca Orlando, Carmen Laurenza, Teresa Lastella, Roberta Naddei, Marina Amico, Maria Alessio

##### Mother and Child Department, Federico II University, Naples, Napoli, Italy

###### **Correspondence:** Carolina Porfito

**Introduction:** Articular involvement is reported as the most common extraintestinal manifestation of IBD in childhood (5-20%).

**Objectives:** To evaluate epidemiologic data, clinical features and laboratory data of patient affected by IBD presenting articular manifestations.

**Methods:** This is a retrospective cohort study involving 104 patients (age 4-18) affected by IBD (according to the revised Porto Criteria 2014) followed by our Gastroenterology Unit. We evaluated sex, age at diagnoses, growth chart, inflammatory index (ESR, CRP, calprotectin), Disease Activity Index (PCDAI o PUCAI), endoscopic localization and histology of lesions (Paris Criteria), abdomen ultrasound with study of the last ileal loop, presence of extraintestinal manifestations (EIMs) and therapy. Patients with articular involvement were divided in 2 groups: peripheral arthropathy type 1 (pauciarticular, duration <10 weeks) and peripheral arthropathy type 2 (polyarticular, duration> 10 weeks).

**Results:** We evaluated 104 patients, 51 females (49%) and 53 males (51%), with average age 14.3 aa, 40 with MC diagnoses, 61 with RCU diagnoses and 3 indeterminate colitis. The median age at diagnosis was 11.2 y for MC and 9.5 y for RCU. Among patients with MC, 52.5% had ileocolonic involvement, 20% cecal, 12.5% colonic, 15% of the upper digestive tract. Among patients with RCU, 32.7% had extensive colitis, 37.7% pancolitis, 14.7% ulcerative proctitis, 13% left colitis, 1.6% ileal lymphoid hyperplasia. Presence of one of the EIMs was reported in 15.3% of patients: erythema nodosum 0.96%, sclerosing cholangitis 2.84% (exclusively in patients with RCU) and articular symptoms/signs 11.5%. In the group of patient with articular involvement, 67% were female and 33% male; 50% had diagnosis of MC and 50% of RCU. The average age of onset of the underlying disease is 12 y for the MC and 11.8 y for the RCU. In 17% of patients, articular symptoms were observed at the onset of the disease while in 42% of the patients they were associated to a clinical exacerbation. The mean values of ESR, CRP and calprotectin were increased. Arthralgia has been identified in 75% of patients with articular involvement. The joints involved were knee in 25%, coxo-femoral in 42%, sacroiliac in 16.5% and tibio-tarsia in 16.5%. Involvement was monolateral in 84% of cases, bilateral in 16%. Only 1 patient presented monolateral knee and bilateral sacroiliac arthralgia. Disease activity index at the onset of articular symptoms was pathological in 8 patients (67%): 4 patients with MC (PCDAI> 10) and 4 patients with RCU (PUCAI> 10). Two patients (16.6%) reported a traumatic event preceding the onset of articular manifestations. In 11 cases (91.6%) articular symptoms were transient, in 1 case (8.4%) the manifestations led to a polyarticular arthritis diagnosis. The articular manifestations were treated with paracetamol (33.3%), ibuprofen (25%), naproxen (8.3%), rectal steroid therapy (16.6%), physiokinesitherapy (8.3%). The patient affected by polyarticular arthritis is currently in therapy with Methotrexate and Adalimumab.

**Conclusion:** In our cohort, the articular manifestations are more frequently associated to MC (14% of patients) than to RCU (10% of patients). The majority of patients (91.6%) have peripheral arthropathy type 1 or pauciarticular (92% of patients, 100% of whom affected by MC and 83.3% with RCU). Despite of the current literature, our patients affected by MC with articular manifestations, had prevalently ileocecal localization of bowel disease. All patients responded to a short-term treatment with anti-inflammatory drugs, in 1 case physiokinesitherapy was necessary. The patient with polyarticular arthritis diagnosis required Methotrexate and Adalimumab to control articular disease.

**Disclosure of Interest**

None Declared

### P245 IMPACT OF JUVENILE IDIOPATHIC ARTHRITIS ON THE PARENTS’ WORK UNDER THE SPECTRUM OF THE NATIONAL STATUS OF UNEMPLOYMENT

#### Polyxeni Pratsidou-Gertsi, Stylianos Mamalis, Sindy Tzimou, Vasiliki Bolou, Maria Trachana

##### First Dept of Pediatrics, Aristotle University, Pediatric Immunology and Rheumatology Referral Center, Ippokration Hospital, Thessaloniki, Greece

###### **Correspondence:** Polyxeni Pratsidou-Gertsi

**Introduction:** According to the recent National Statistics, the rate of unemployment in Greek citizens >25yrs is about 25% (26% Females) and the raising pool of pensioners <50yrs, 39%.

**Objectives:** To investigate the impact of Juvenile Idiopathic Arthritis (JIA) on the work life of parents having off springs with the disease, due to lack of relevant data.

**Methods:** This prospective case-control study analyzed the parental responses through a developed for the study questionnaire. Between 6/2017 and 12/2017, 107 consecutive parents (107 JIA pts) escorting their child in the Outpatient Pediatric Rheumatology Clinic consented to participate in the survey. The majority were mothers (F: 85, mean aged 42 yrs) and consisted the JIA-parental (JIA p) Group. 111 parents (F:49, mean aged 38.7yrs, p>0.7) of healthy children during the same period served as controls, after their given consent (Hp).

**Results:** The JIA subgroups did not differ from the expected distribution (p=). Among patients 86 (80.37%) were on medication. The 2 groups (JIAp and Hp) did not significantly differ in terms of: educational level (p=0.82), percent of the remaining family members requiring assistance (p=0.97), self- or spouse unemployment (p=0.43, p=0.16), reduced work performance manifested by fatigue, anxiety or depression (p=0.77), or resign or work dismissal (p=0.8, p=0.8, respectively). In contrast, less JIAp than Hp were working in the Private Sector (p=0.01) or had a steady-fullterm work schedule (p=0.04), while more JIAp had been retired (p=0.02). An increased but non-significant trend for a prolonged work absence >60 hours /month) attributed to JIA (17% vs 0%, p=0.07) in the JIAp was observed. The parental observation or performance of drug administration or home physio-exercise program was not associated with the parental time spent with the affected child (p=0.48). Additionally, the presence of JIA flares or worsening was not associated with an increased duration of home care in respect to the Hp (p=0.68).

**Conclusion:** The contemporary and holistic JIA management did not adversely affect the working state of these Greek parents or their spouses in respect to work performance, the duration of home care of the affected child, regardless of the drug administration or home physio-exercise program. Noteworthy, an increased but non-significant chance of retirement was recorded among the parents having a child with JIA.

**Disclosure of Interest**

None Declared

### P246 DISTRIBUTION OF SERUM HOMOCYSTEINE LEVELS IN JUVENILE IDIOPATHIC ARTHRITIS PATIENTS COMPARED WITH CONTROLS: A STUDY FROM BANGLADESH.

#### Mohammad Imnul Islam, Shahana Rahman, Fakhrul Alam, Mohammed Mahbubul Islam

##### Department of Paediatrics, Dhaka, Bangladesh, Bangabandhu Sheikh Mujib Medical University (BSMMU), Dhaka, Bangladesh

###### **Correspondence:** Shahana Rahman

**Introduction:** Abnormal levels of serum homocysteine may be found in JIA patients. There are some studies about the level of homocysteine (Hey) in children with JIA and the results are conflicting, but no study is available in Bangladesh concerning JIA and homocysteine level.

**Objectives:** This study was done with the objectives to assess homocysteine levels in JIA patients and to compare the levels with control group, to see whether there was any change in the level of homocysteine among JIA patients.

**Methods:** This was a cross sectional study conducted in the Department of Paediatrics, Bangabandhu Sheikh Mujib Medical University (BSMMU), Dhaka from January 2016 to March 2017. A total of 50 children with JIA were included. Demographic characteristics, duration of illness and type of JIA, were recorded in a predesigned questionnaire. Controls were selected from the patients attending Paediatrics OPD.

**Results:** The majority of the patients belonged to the age group of 11-15 years. Males were more than females. Almost two third (64%) patients had normal and 36% had high homocysteine levels among the cases. On the other hand, 100% had normal homocysteine levels in control group. The difference of mean homocysteine level was significant (p<0.05) between the two groups. Hyperhomocysteinemia was found in 69% of JIA patients having duration of illness more than one year. Mean homocysteine levels of polyarticular RF positive JIA and SJIA were found significantly higher in comparison to the controls.

**Conclusion:** Hyperhomocysteinemia were found in 36% of JIA cases and it increased with the longer disease duration. Homocysteine levels were higher among Polyarticular and systemic JIA cases.

**Disclosure of Interest**

None Declared

### P247 CHARACTERIZATION OF A COHORT OF COLOMBIAN PATIENTS WITH RENAL INVOLVEMENT IN JUVENILE IDIOPATHIC ARTHRITIS

#### Maria F. Reina^1^, Olga L. Prado^2^, Pilar Guarnizo-Zuccardi^1^

##### ^1^Pediatric Rheumatology post graduate program; ^2^Pediatric Nephrology post graduate program, Universidad El Bosque, Bogota, Colombia

###### **Correspondence:** Maria F. Reina

**Introduction:** Juvenile idiopathic arthritis (JIA), is the most prevalent cause of chronic arthritis at pediatric age. It is associated with various extra-articular manifestations. The renal involvement is not studied frequently. Previously, the most common overt renal disease related to JIA was renal amyloidosis. Actually, some studies have revealed subtle tubular and glomerular abnormalities in children with JIA. Renal lesions in patients with JIA are usually complications of the underlying rheumatic disease or side-effects of antirheumatic drugs.

**Objectives:** Describe the renal involvement according to the subtype of JIA, in a cohort of patients with JIA identified in 2 pediatric rheumatology clinics in Colombia in 2018.

**Methods:** Multi-center, retrospective, descriptive study. The medical records of the last 100 patients assessed were reviewed and the information was recollected using a data collection format.

**Results:** N=100. 56% of patients were female. Sex ratio F 1.3 - M 1. The median age of admission to the study was 14 years (interquartile range of 5 years). Mean time between the diagnosis and the onset of renal involvement was 16.28 months (SD 25 months). The proportion of patients according to subtype of JIA was: oligoarticular 27%, polyarticular RF negative 23%, arthritis related enthesitis (ERA) 19%, systemic 17%, polyarticular RF positive 10%, psoriatic 3% and undifferentiated 1%.

Prevalence of renal involvement during follow-up was 36% and it was evidenced by hematuria, proteinuria or an altered glomerular filtration rate (GFR). 37% of the patients with oligoarticular JIA developed renal involvement, polyarticular RF negative 34.8%, polyarticular RF positive 30%, ERA 42%, systemic 35% and in the only one patient with undifferentiated arthritis. 10% of patients had some abnormality in the uranalysis: 16% leukocyturia and 3% hematuria. 69 patients who collected a 24-hour urine sample, 54% presented proteinuria (54% mild proteinuria, 43% moderate and 3% in the nephrotic range), 1 patient had resolution of proteinuria and the other persisted with proteinuria mild. 18 patients a sample was recorded for calciuria in 24 hours, 11% was positive.

Mean serum creatinine was 0.64 mg/dl (SD 0,27, range 0.3-1.0 mg/dl). The average TFG was 113 ml/min/1.73 (SD 26,17, range 63.9-184 ml/min/1.73). 68% had normal GFR, 19.5% decreased GFR and 12.3% elevated GFR. 3 patients presented abnormal renal ultrasonography (nephrolithiasis, grade III vesicoureteral reflux and mild pyelectasis) with normal GFR. Nobody required renal biopsy. There were no statistical differences in subtype of JIA, clinical manifestation and renal involvement.

Treatment at renal involvement: 80.5% received steroids, 61% NSAIDs, 94.5% methotrexate, 2.7% sulfasalazine, 2.7% leflunomide and 29.7% biologics. A statistically significant difference was found between treatment and renal involvement (p<0,001).

**Conclusion:** In this cohort, renal involvement was 36%. Proteinuria and alteration in GFR were the most frequent. A greater proportion of renal involvement was observed in patients who received steroids, methotrexate and NSAIDs. In patients with JIA, uranalysis and renal function should be routinely monitored for early diagnosis of renal disease, which causes morbidity.

**Disclosure of Interest**

None Declared

### P248 COMPLEMENTARY AND ALTERNATIVE THERAPY USE IN CHILDREN WITH JUVENILE IDIOPATHIC ARTHRITIS: A QUESTIONNAIRE BASED STUDY

#### William D. Renton^1,2^, Taylor Mills^1,2^, Jane E. Munro^1,2^

##### ^1^Royal Children's Hospital; ^2^Murdoch Children's Research Institute, Melbourne, Australia

###### **Correspondence:** William D. Renton

**Introduction:** Complementary and alternative therapy (CAT) use is common among children and young people with juvenile idiopathic arthritis (JIA)(1)(2)(3)(4). There has been no previous research on this topic in Australia. In addition, little is known about patterns of CAT use and the impact on existing clinical care.

**Objectives:** To determine the prevalence of CAT use, define the types of therapies being used, assess factors associated with CAT use, evaluate perceived efficacy and to explore whether patients had discussed CATs with their treating specialist.

**Methods:** A prospective, cross-sectional, questionnaire-based study of patients attending the Royal Children’s Hospital, Melbourne, Australia was conducted via convenience sampling between June and December 2017. Patients with JIA under the age of 20 were included. χ^2^ test and two tailed unequal variances t-test were used for statistical analysis.

**Results:** 198 patients were included for analysis. 74.7% had previously had intra-articular steroid injections, 69.2% had used a disease modifying anti-rheumatic drug (DMARD) and 31.3% had used a biologic DMARD. 67 (33.8%) patients had ever used one or more CATs. The most frequently used CATs were vitamins and minerals (17.6%), probiotics (13.1%), dietary changes (11.6%) and non-vitamin/non-mineral supplements (10.1%). Patients who had used CATs had previously tried more conventional medications, experienced more conventional medication related side-effects and had better conventional medication adherence compared to those who had not. 173/198 (87.4%) respondents agreed or strongly agreed that conventional medications were helpful in managing their arthritis. Comparatively, 31/67 (46.3%) of CAT users agreed or strongly agreed that CATs were helpful. Only 20.0% of patients had been asked about previous CAT use by their treating doctor.

**Table:** 

	CAT	Non-CAT	Total	p-value
Number of medications tried (SD)	4.55 (± 0.243)	3.77 (± 0.170)	4.04 (± 0.142)	0.0094
Number of medication side-effects (SD)	2.49 (± 0.346)	1.15 (± 0.160)	1.60 (± 0.164)	0.0006
Medication adherence (SD)	96.6 (± 1.12)	91.6 (± 1.84)	93.3 (± 1.29)	0.0215
SD = standard deviation				

**Conclusion:** Use of CATs is common among children with JIA and a broad range of therapies are used. Despite recent therapeutic advances, medication related side-effects continue to be a problem for many patients and may lead to patients trying unconventional therapies. Treating specialists are often unaware that these therapies are being used. Given the potential for harm (5) and possible interactions with conventional medications, we would urge all clinicians to routinely enquire about CAT use.

**References**

1. Feldman DE, Duffy C, De Civita M, Malleson P, Philibert L, Gibbon M, et al. Factors associated with the use of complementary and alternative medicine in juvenile idiopathic arthritis. Arthritis Rheum. 2004;51(4):527-32.

2. Seburg EM, Horvath KJ, Garwick AW, McMorris BJ, Vehe RK, Scal P. Complementary and alternative medicine use among youth with juvenile arthritis: are youth using CAM, but not talking about it? J Adolesc Health. 2012;51(2):200-2.

3. April KT, Feldman DE, Zunzunegui MV, Descarreaux M, Malleson P, Duffy CM. Longitudinal analysis of complementary and alternative health care use in children with juvenile idiopathic arthritis. Complement Ther Med. 2009;17(4):208-15.

4. Southwood TR, Malleson PN, Roberts-Thomson PJ, Mahy M. Unconventional remedies used for patients with juvenile arthritis. Pediatrics. 1990;85(2):150-4.

5. Lim A, Cranswick N, South M. Adverse events associated with the use of complementary and alternative medicine in children. Arch Dis Child. 2011;96(3):297-300.

**Disclosure of Interest**

None Declared

### P249 SUBOPTIMAL MUSCLE STRENGTH AND BONE HEALTH IN PATIENTS WITH OLIGO- AND POLYARTICULAR JUVENILE IDIOPATHIC ARTHRITIS DIAGNOSED IN THE ERA OF BIOLOGICS

#### Kristine Risum^1^, Kristin Godang^1^, Anne Marit Selvaag^1^, Bjørge Herman Hansen^2^, Øyvind Molberg^1^, Jens Bollerslev^1^, Inger Holm^1^, Hanne Dagfinrud^3^, Helga Sanner^1^

##### ^1^Oslo University Hospital; ^2^Norwegian School of Sport Sciences; ^3^Diakonhjemmet Hospital, Oslo, Norway

###### **Correspondence:** Kristine Risum

**Introduction:** Previous studies indicate that juvenile idiopathic arthritis (JIA) patients compared to healthy controls have lower muscle strength, poorer bone health and impaired body composition with higher fat mass and low lean mass. However, knowledge about these components of physical fitness is limited in JIA patients diagnosed in the era of biologics.

**Objectives:** To compare muscle strength, total bone mineral density (BMD) and body composition in JIA patients diagnosed in the era of biologics with healthy controls; to compare muscle strength, total BMD and body composition between patient subsets; to examine associations between disease variables and muscle strength and total BMD in patients.

**Methods:** Sixty patients (50 girls) with persistent oligo JIA and polyarticular disease (extended oligoarthritis and polyarticular RF +/-), ages 10-16 years, and 60 age- and sex-matched controls were included. Isokinetic quadriceps and hamstrings muscle strength (Cybex) and isometric hand grip strength (Baseline dynamometer) were measured bilaterally. Total BMD and body composition were measured by Dual-energy X-ray Absorptiometry. Current pain, pain and fatigue during previous week, pubertal status, blood values and physical activity (PA) levels (accelerometry) were assessed in all participants. In patients, disease activity and functional disability were evaluated by validated tools.

**Results:** Biologic drugs were used by 42 % of the patients. Patients had lower muscle strength bilaterally compared with controls in almost all variables tested. Results for the dominant side are shown in Table 1. Total body BMD and total body BMD z-scores were lower in patients compared to controls (Table 1). Total fat mass and fat mass percentage were comparable between patients and controls, while there was a tendency for patients to have lower lean mass compared to controls, p=0.06. Muscle strength, total BMD and body composition were comparable between patient subsets. In patients, higher muscle strength was associated with higher age, male sex, higher BMI and higher vigorous PA levels. Higher BMD was associated with higher total lean mass, higher age and female sex. No disease related variables were identified as determinants for muscle strength or BMD.

**Conclusion:** Muscle strength and BMD are lower in JIA patients diagnosed in the biological era compared to controls. Body composition is more favorable in JIA patients than previously reported, with comparable fat mass and fat mass percentage between patients and controls.

**Disclosure of Interest**

None Declared

**Table 1 (abstract P249).** Muscle strength and bone health in patients and controls

	JIA total (n=60)	Controls (n=60)	*p*	JIA oligo (n=30)	JIA poly (n=30)	*p*
Quadriceps strength dominant side (PT) ^a^	124 (38)	146 (34)	0.001	131 (33)	118 (42)	0.186
Hamstring strength dominant side (PT) ^a^	65 (26)	83 (22)	<0.001	64 (25)	66 (28)	0.600
Quadriceps endurance dominant side (TW) ^b^	2470 (633)	2660 (466)	0.063	2516 (650)	2424 (624)	0.578
Hamstring endurance dominant side (TW) ^b^	1065 (461)	1376 (409)	<0.001	1109 (470)	1018 (455)	0.332
Hand grip strength dominant side (kg)	23.2 (7.4)	27.9 (8.4)	0.002	22.9 (7.0)	23.5 (7.8)	0.776
BMD_TB_ (g/cm^2^)	0.971 (1.127)	1.033 (0.128)	0.009	0.970 (0.128)	0.972 (0.129)	0.939
BMD_TB_ z-score (g/cm^2^)	0.152 (0.810)	0.712 (0.910)	0.001	0.200 (0.909)	0.103 (0.709)	0.648

Numbers are mean (SD). ^a^ PT: Peak torque given in newton meter divided by body weight x 100, ^b^ TW: Total work given in Joule divided by body weight x 100. BMD_TB_: total body bone mineral density

### P250 CHILDREN’S AND PARENTS’ PERSPECTIVES ON OUTCOMES FOR JIA TRIALS COMPARING DIFFERENT CORTICOSTEROID DELIVERY ROUTES: A QUALITATIVE STUDY

#### Louise Roper^1^, Frances Sherratt^1^, Eileen M. Baildam^2^, Flora McErlane^3^, Michael W. Beresford^4^, Helen Foster^3^, Bridget Young^5^

##### ^1^Department of Psychological Sciences, Institute of Psychology, Health and Society, University of Liverpool; ^2^Department of Paediatric Rheumatology, Alder Hey Children's NHS Foundation Trust, Liverpool; ^3^Department of Paediatric Rheumatology, Great North Children's Hospital;, Institute of Cellular Medicine, Newcastle University, Newcastle; ^4^Department of Paediatric Rheumatology, Alder Hey Children's NHS Foundation Trust;, Department of Women's and Children's Health, Institute of Translational Medicine; ^5^Department of Psychological Sciences, Institute of Psychology, Health & Society, University of Liverpool , Liverpool , UK

###### **Correspondence:** Louise Roper

**Introduction:** Randomised controlled trials (RCTs) are central to improving the clinical care and outcomes of children and young people (CYP) with juvenile idiopathic arthritis (JIA). RCTs have traditionally been designed by research teams with outcomes selected by researchers and healthcare professionals. However, it is children and their families who live with the effects of the illness and its treatments. It is increasingly clear that they can and must be involved in prioritising different outcomes to research teams. Accessing children’s and parents’ unique perspectives and experiences of JIA can help to inform the selection of outcomes. As part of a wider process, qualitative interviews with children with JIA and their parents may help to identify which outcomes are most important to children treated with corticosteroids for JIA and their families.

**Objectives:** In the context of a feasibility study for a planned trial comparing corticosteroid delivery routes, we explored children’s and parents’ experiences of corticosteroid treatments and the outcomes they considered important in the assessment of the impact of the disease and its treatments on their everyday life.

**Methods:** Semi-structured qualitative interviews were conducted with children who had recently received corticosteroids to treat JIA, as well as with their parents. Participants were recruited from four UK paediatric rheumatology centres. Audio recorded interviews were transcribed and analysed thematically, drawing on the Framework Method.

**Results:** Twenty eight children and parents (9 children [aged 8-16 years], and 19 parents) from 15 families were interviewed. Children’s and parents’ experiences of steroids often varied depending on individual characteristics like needle phobia, or fear of anaesthetics (required for intra-articular injections). They evaluated corticosteroid treatments in terms of whether these relieved pain, fatigue, stiffness and swelling, improved mobility, lead to changes in weight and appetite and the impact on children’s social life and education. Children prioritised outcomes related to quality of life and social and school life. Typically, parents’ priorities concerned “controlling” the JIA and short term treatment effects.

**Conclusion:** Previous research has focused on children’s and parent’s experiences of JIA but not their perspectives on steroid treatments in JIA. There was considerable overlap in the outcomes that children and parents regarded as important in evaluating such treatments. However, children prioritised quality of life, while parents emphasised managing and controlling disease flares. These insights may be useful in everyday clinical practice and could enrich the development of a core outcome set as part of a wider consensus process.

Children’s and family perspectives on outcomes may inform the development of research studies that are more meaningful and important to patients. Using outcomes that are more relevant to stakeholders could ultimately improve recruitment to JIA clinical trials and achievement of outcomes that are important to patients and their families.

**Disclosure of Interest**

None Declared

### P251 THE ASSOCIATION BETWEEN SERUM LEVEL OF VITAMIN D AND DISEASE ACTIVITY IN CHILDREN WITH RHEUMATIC DISORDERS

#### Marta Laizane^1^, Ieva Saulite^2^, Ingrida Rumba-Rozenfelde^1,2^

##### ^1^Faculty of Medicine, University of Latvia; ^2^Children’s Clinical University Hospital, Riga, Latvia

###### **Correspondence:** Ingrida Rumba-Rozenfelde

**Introduction:** Vitamin D deficiency is widespread and re-emerging major health problem globally. 13.0% of European individuals have serum 25(OH)D concentrations <30 ng/mL on average in the year [Cashman et al., 2016]. Although vitamin D is known to have an immunomodulatory effect [Myszka et al., 2014], it is still unclear whether deficiency is associated with higher disease activity. Some studies show no associations of serum 25(OH)D levels with musculoskeletal disease activity or functional status [Pakchotanon et al., 2013], while others suggest that vitamin D deficiency may be linked to disease severity in rheumatoid arthritis [Kostoglou-Athanassiou et al., 2012].

**Objectives:** The study was carried out in order to determinate the prevalence of hypovitaminosis D in pediatric population with rheumatic disorders and to find out whether there is significant association between serum concentration of vitamin D and disease activity.

**Methods:** A retrospective analysis of 98 subjects with rheumatic disorders, aged 1-18 years, was performed. Patients were divided into two groups: patients with juvenile idiopathic arthritis (JIA) (n=58) and patients with postviral reactive arthritis (n=40).

Serum concentration of 25-hydroxyvitamin D vitamin was determined by electrochemiluminescence method.

During the study data of patients’ age, sex, height, weight, serum 25-hydroxyvitamin D, rheumatoid factor, titers of antinuclear antibodies, anti-dsDNA antibodies and anti-TNF alpha were gathered.

The relationships between serum concentration of vitamin D and disease activity markers were found using Pearson’s correlation coefficient; differences of values were analyzed using Mann-Whitney-Wilcoxon test.

**Results:** Vitamin D level of analysed patients ranged from 8,17 to 52,71 ng/mL. 69,39% (n=68) were diagnosed with vitamin D deficiency (serum 25-hydroxyvitamin D (25[OH]D) below 30 ng/mL). Serum level of vitamin D in group of patients with JIA is significantly lower than in group of patients with reactive arthritis (p = 0.036). Weak negative correlations between vitamin D level and patients age (r = - 0.443; p = 0.000) and body mass index (r = - 0.372; p = 0.002) were found. Positive correlation between vitamin D and ESR was found in JIA patients (r =0.446; p = 0.002). There were found no statistically significant relationships between vitamin D level and any other paramether.

**Conclusion:** Vitamin D deficiency is commonly seen in children with rheumatic disorders. The results of the study indicate that patients with JIA have significantly lower serum level of vitamin D than patients with reactive arthritis. Adolescents have lower vitamin D concentration, comparing to younger children, which is also consistent with previous studies [Mithal et al., 2009; Rockell et al., 2005; Looker et al., 2002].

Further investigation that include such information as eating habits, the use vitamin D supplements and sun exposure are required in order to expand this study.

**Disclosure of Interest**

None Declared

### P252 ANTIBODY RESPONSES TO DIPHTHERIA/TETANUS VACCINE IN PAEDIATRIC PATIENTS WITH POLYARTICULAR-COURSE JUVENILE IDIOPATHIC ARTHRITIS RECEIVING SUBCUTANEOUS ABATACEPT

#### Nicolino Ruperto^1^, Hermine I. Brunner^2^, Nikolay Tzaribachev^3^, Gabriel Vega-Cornejo^4^, Ingrid Louw^5^, Ivan Foeldvari^6^, Vladimir Keltsev^7^, Maria Eliana Paz Gastañaga^8^, Carine Wouters^9^, Rik Joos^10^, Bernard Lauwerys^11^, Kirsten Minden^12,13^, Johannes C. Breedt^14^, Xiaohui Li^15^, Marleen Nys^16^, Robert Wong^15^, Daniel J. Lovell^2^, Alberto Martini^17^

##### ^1^Clinica Pediatrica e Reumatologia – PRINTO, Istituto Giannina Gaslini, Genoa, Italy; ^2^PRCSG, Cincinnati Children’s Hospital Medical Center, Cincinnati, USA; ^3^Pediatric Rheumatology Research Institute, Bad Bramstedt, Germany; ^4^Hospital México Americano, Guadalajara, Mexico; ^5^Panorama Medical Centre, Cape Town, South Africa; ^6^Hamburg Centre for Pediatric and Adolescent Rheumatology, Hamburg, Germany; ^7^GBUZ Samara region Togliatti City Clinical Hospital No.5, Togliatti, Russian Federation; ^8^Clínica Anglo Americana, Lima, Peru; ^9^University Hospital Gasthuisberg, Leuven; ^10^Universitair Ziekenhuis Gent, Ghent; ^11^Université Catholique de Louvain, Brussels, Belgium; ^12^German Rheumatism Research Center; ^13^Charité University Medicine, Berlin, Germany; ^14^University of Pretoria, Pretoria, South Africa; ^15^Bristol-Myers Squibb, Princeton, USA; ^16^Bristol-Myers Squibb, Braine L’Alleud, Belgium; ^17^Direzione Scientifica, Istituto Giannina Gaslini, Genoa, Italy

###### **Correspondence:** Nicolino Ruperto

**Introduction:** Due to a higher susceptibility to infections than adults, paediatric patients with polyarticular‐course juvenile idiopathic arthritis (pJIA) receiving abatacept or other biologic DMARDs with immunosuppressive effects may be at increased risk of vaccine-preventable infections such as diphtheria and tetanus.

**Objectives:** To evaluate antibody responses to diphtheria/tetanus vaccine in paediatric patients with pJIA receiving SC abatacept.

**Methods:** This sub-study enrolled 2 to 5-year-old patients with pJIA from the Phase III, open-label, single-arm trial (NCT01844518)^1^ of weekly weight-tiered SC abatacept: 10–<25 kg (50 mg), 25–<50 kg (87.5 mg) and ≥50 kg (125 mg). Patients who participated in this sub-study received diphtheria/tetanus vaccine prior to enrolment; the vaccine was not administered during the study period. Blood samples to assess protective antibodies to diphtheria and tetanus were measured in patients with at least 2 months of abatacept treatment. Assessment for protective antibody levels was performed by a central laboratory^2^ using quantitative multiplex bead assays with protective levels defined as >0.1 IU/mL based on central laboratory and World Health Organization criteria.^2–4^

**Results:** A total of 29 patients were enrolled in this sub-study. All patients were white, from either Europe or South America, aged 2–5 years and weighed 12–26 kg. Abatacept exposure durations were: 19/29 (66%) >12 months, 1/29 (3%) 6–12 months and 9/29 (31%) 2–<6 months. At the end of the sub-study, all 29/29 (100%) patients had protective antibody levels to tetanus (range: 0.2–23.2 IU/mL). Protective antibody levels to diphtheria were observed in 26/29 (90%) patients (range: 0.2–8.0 IU/mL). The remaining three patients each had a protective antibody level of 0.1 IU/mL, which bordered the lower threshold of protection. No cases of diphtheria or tetanus were observed. There was no evidence of recognizable association between infection risk and abatacept treatment.

**Conclusion:** In this sub-study, paediatric patients with pJIA aged 2–5 years who received ≥2 months of SC abatacept were able to maintain effective diphtheria/tetanus vaccination protection.

**References**

1. Brunner HI, et al. *Arthritis Rheumatol* 2018; doi: 10.1002/art.40466

2. ARUP Laboratories, Diphtheria & Tetanus Antibodies, IgG, http://ltd.aruplab.com/Tests/Pub/0050595, accessed Apr 2018

3. World Health Organization, Tetanus position paper (Feb 2017), http://www.who.int/immunization/policy/position_papers/tetanus/en/, accessed Apr 2018

4. World Health Organization, Diphtheria position paper (Aug 2017), http://www.who.int/immunization/policy/position_papers/diphtheria/en/, accessed Apr 2018

**Trial registration identifying number:** clinicaltrials.gov, NCT01844518

**Disclosure of Interest**

N. Ruperto Grant / Research Support from: Bristol-Myers Squibb, Roche, Janssen, Novartis, Pfizer, Sobi, Consultant for: AbbVie, Ablynx, Amgen, AstraZeneca, Baxalta Biosimilars, Biogen Idec, Boehringer Ingelheim, Bristol-Myers Squibb, Celgene, Eli-Lilly, EMD Serono, Gilead Sciences, Janssen, MedImmune, Novartis, Pfizer, R-Pharm, Roche, Sanofi, Servier, Takeda, Speaker Bureau of: AbbVie, Ablynx, Amgen, AstraZeneca, Baxalta Biosimilars, Biogen Idec, Boehringer Ingelheim, Bristol-Myers Squibb, Celgene, Eli-Lilly, EMD Serono, Gilead Sciences, Janssen, MedImmune, Novartis, Pfizer, R-Pharm, Roche, Sanofi, Servier, Takeda, H. Brunner Consultant for: AstraZeneca, Bristol-Myers Squibb, Genentech, Janssen, Novartis, Pfizer, Sanofi, Takeda, Speaker Bureau of: Genentech, Novartis, N. Tzaribachev: None Declared, G. Vega-Cornejo: None Declared, I. Louw: None Declared, I. Foeldvari: None Declared, V. Keltsev: None Declared, M. E. Paz Gastañaga Grant / Research Support from: Pfizer, Novartis, Sanofi, GlaxoSmithKline, C. Wouters: None Declared, R. Joos: None Declared, B. Lauwerys Grant / Research Support from: UCB, Janssen, Pfizer, Consultant for: Bristol-Myers Squibb, Servier, Pfizer, K. Minden Grant / Research Support from: AbbVie, Pfizer, Roche, Speaker Bureau of: AbbVie, Pfizer, Roche, Pharm-Allergan (honoraria for lectures), J. Breedt: None Declared, X. Li Shareholder of: Bristol-Myers Squibb, Employee of: Bristol-Myers Squibb, M. Nys Shareholder of: Bristol-Myers Squibb, Employee of: Bristol-Myers Squibb, R. Wong Shareholder of: Bristol-Myers Squibb, Employee of: Bristol-Myers Squibb, D. Lovell Grant / Research Support from: AbbVie, Bristol-Myers Squibb, NIH, Pfizer, Roche, Consultant for: AbbVie, Boehringer Ingelheim, Bristol-Myers Squibb, Celgene, Genentech, GlaxoSmithKline, Janssen, Johnson & Johnson, Novartis, Takeda, UCB, Speaker Bureau of: Genentech, A. Martini Grant / Research Support from: Istituto Giannina Gaslini has received contributions from: Bristol-Myers Squibb, Roche, Janssen, Novartis, Pfizer, Sobi for the coordination activity of the PRINTO network, Consultant for: On behalf of the Istituto Giannina Gaslini for AbbVie, Boehringer Ingelheim, Novartis, R Pharm

### P253 JUVENILE ARTHRITIS MANAGEMENT IN LESS RESOURCED COUNTRIES (JAMLESS): CONSENSUS RECOMMENDATIONS FROM THE CRADLE OF HUMANKIND

#### Christiaan Scott^1^, Mercedes Chan^2^, Waheba Slamang^3^, Lawrence O. Okong'o^4^, Ronald M. Laxer^5^, Ross Petty^2^, Maria M. Katsicas^6^, Francis Frederick^7^, James Chipeta^8^, Gail Faller^9^, Gecilmara Pileggi^10^, Claudia Saad Magalhaes^11^, Carine Wouters^12^, Helen E. Foster^13^, Raju Khubchandani^14^, Nicolino Ruperto^15^, Ricardo Russo^16^

##### ^1^Paediatrics and Child Health, Red Cross War Memorial Children's Hospital and University of Cape Town, Cape Town, South Africa; ^2^BC Children's Hospital, Vancouver, Canada; ^3^Red Cross War Memorial Children's Hospital and University of Cape Town, Cape Town, South Africa; ^4^University of Nairobi, Nairobi, Kenya; ^5^University of Toronto and The Hospital for Sick Children, Toronto, Canada; ^6^Hospital de Pediatria Garrahan, Buenos Aires, Argentina; ^7^School of Medicine, Muhimbili University of Health and Allied Sciences, Dar es Salaam, Tanzania, United Republic of; ^8^Departments of Paediatrics and Child Health , University of Zambia School of Medicine, Lusaka, Zambia; ^9^Wits Donald Gordon Medical Centre and the University of the Witwatersrand, , Johannesburg, South Africa; ^10^Clinical Research Center of Ribeirão Preto Medical School University of Sao Paulo; ^11^Sao Paulo State University, Sao Paulo, Brazil; ^12^KU Leuven - University of Leuven, Leuven, Belgium; ^13^Newcastle University, UK and Great North Children's Hospital, Newcastle upon Tyne, UK; ^14^Jaslok Hospital and Research Center, Mumbai, India; ^15^Istituto di Ricovero e Cura a Carattere Scientifico Istituto Giannina Gaslini, Genoa, Italy; ^16^Hospital de Pediatría Garrahan, Buenos Aires, Argentina

###### **Correspondence:** Christiaan Scott

**Introduction:** Juvenile idiopathic arthritis (JIA) is the most prevalent chronic rheumatic disease in childhood and a major cause of pain and disability. The majority of children and their families live in less resourced countries (LRC) and face significant socioeconomic and healthcare challenges impacting their ability to access high-quality pediatric rheumatology care. These include, but are not limited to: poverty, political unrest, high burdens of endemic disease, low healthcare spending and limited access to medications. Current recommendations for standards of care and treatment for JIA do not consider children living in LRC.

**Objectives:** To develop recommendations for the care of children with JIA in less resourced countries.

**Methods:** A group of experienced paediatric rheumatologists from less resourced countries was convened with additional inputs from a steering group of international paediatric rheumatologists with experience developing recommendations and standards of care for JIA. Following a needs assessment survey of 502 healthcare workers caring for children with JIA in LRC’s, a literature review was carried out and recommendations formulated using Delphi technique and a final consensus conference. The degree of consensus, level of agreement and level of evidence for these recommendations are reported.

**Results:** The general amount of available evidence for critical appraisal was low, reflecting a shortage of research specifically focused on less resourced countries. Recommendations cover 5 themes: 1) Diagnosis 2) Referral and Monitoring 3) Education and Training 4) Advocacy and Networks and 5) Research. Thirty-five final statements highlight endemic diseases relevant to the diagnosis and treatment of JIA in less resourced nations. They endorse existing guidelines for the treatment of JIA,vaccinations in the context of rheumatic disease and, uveitis screening. They also advocate for standards of care to pediatric rheumatology services and strategies to raise awareness of JIA through education of stakeholders as well as make arguments for the support and development of research and researchers in JIA in developing nations. All but one statement achieved 100% consensus and levels of agreement were high.

**Conclusion:** Our recommendations offer novel insight and present consensus-based strategies for the care of children with JIA in less resourced countries. The emphasis on communicable and endemic diseases influencing the diagnosis and treatment of JIA serves as a valuable addition to existing recommendations on the management of JIA. Statements presenting standards of care aim to guide best practices in the development of pediatric rheumatology in LRCs. With increasing globalization, these recommendations – as a whole – provide educational and clinical utility for clinicians worldwide. The low evidence base for our recommendations is an impetus for further inquiry towards optimizing care for children with JIA in LRC’s and around the world.

**Disclosure of Interest**

C. Scott Grant / Research Support from: International League of Associations for Rheumatology (ILAR) Grant, M. Chan: None Declared, W. Slamang: None Declared, L. Okong'o: None Declared, R. Laxer: None Declared, R. Petty: None Declared, M. Katsicas: None Declared, F. Frederick: None Declared, J. Chipeta: None Declared, G. Faller: None Declared, G. Pileggi: None Declared, C. Saad Magalhaes: None Declared, C. Wouters: None Declared, H. Foster: None Declared, R. Khubchandani: None Declared, N. Ruperto: None Declared, R. Russo: None Declared

### P254 LUNG FUNCTION IN CHILDREN WITH JUVENILE IDIOPATHIC ARTHRITIS (JIA)

#### Marija Šenjug Perica^1^, Davor Plavec^2^, Ivana Maloča Vuljanko^3^, Lana Tambić Bukovac^1^

##### ^1^Department of Pediatric and Adolescent Rheumatology; ^2^Research Department; ^3^Lung function laboratory , Srebrnjak Children’s Hospital, Zagreb, Croatia

###### **Correspondence:** Marija Šenjug Perica

**Introduction:** Although symptomatic lung involvement is uncommon in children with JIA there have been reports of significant restrictive pattern manifest at lung function test reports.

**Objectives:** To evaluate lung function in children with JIA.

**Methods:** Lung function testing including diffusion capacity for carbon monoxide (DLCO) was performed in 58 children diagnosed with different subtypes of juvenile idiopathic arthritis according to ILAR criteria. Forced vital capacity (FVC), forced expiratory volume in 1 second (FEV1), FEV1/FVC ratio, forced expiratory flow between 25-75% of vital capacity (FEF25-75%), peak expiratory flow rate (PEFR) and DLCO were measured. Data regarding current and previous treatment modalities (NSAR, DMARD, biologic therapy) was extracted using medical charts and CRP level, sedimentation rate and disease activity were determined at the time of lung function testing. None of the included patients had any symptoms of pulmonary disease.

**Results:** Out of 58 children with JIA, 72.4% were female and 27.6% male, with median age of 14.1 (8.3-20.7) years. Patients were diagnosed with oligo JIA (31%), poli JIA (31%), ERA (19%), systemic JIA (10.3%), psoriatic JIA (5.2%) and reactive arthritis (3.5%). In 9 patients results of DLCO were inadequate for interpretation, as well as data of lung function tests in 2 patients. At the time of lung function testing 74.1 % (43 patients) were being treated with NSAR, 24.1% (14 patients) with low dose oral corticosteroids, 36.2% (21 patient) with DMARD and 17.2% (10 patients) with biologic therapy. During the previous treatment course 44.8% were treated with methotrexate and 19.0% with biologic therapy.

Lung function tests did not reveal any obstructive or restrictive alteration of ventilation in patients. Mean values were FVC 95.7%, FEV1 97.5%, FEV1/FVC 89.2%, FEF 25-75 99.3%, MEF25 110.9%. Although mean value of DLCO among patients was 80.2%, there were 10 patients with DLCO lower than 70%, and 7 of them were previously treated with methotrexate. Analysis revealed that DLCO<70% is associated only with previous MTX treatment (Chi square=3.78, p=0.051) and not with other treatment modalities or current disease activity.

**Conclusion:** In our group of patients with different JIA subtypes testing revealed normal lung function. However, a marginal decrease of DLCO in small number of patients was associated with previous MTX treatment. Although MTX may cause severe adverse pulmonary side-effects in adult population, those are rare in children, and marginally decreased DLCO in our and previous studies support relative safety of MTX treatment in JIA.

**Disclosure of Interest**

None Declared

### P255 THE RELATIONSHIP BETWEEN SERUM LEVEL OF VITAMIN D3 AND CLINICAL FEATURES OF JUVENILE IDIOPATHIC ARTHRITIS IN CHILDREN

#### Nataly S. Shevchenko^1,2^, Iuliia V. Khadzhynova^1,2^

##### ^1^Department of pediatrics № 2, V.N. KARAZIN NATIONAL UNIVERSITY; ^2^department of cardiorheumatology, INSTITUTE OF CHILDREN AND ADOLESCENTS HEALTH CARE, Kharkiv, Ukraine

###### **Correspondence:** Nataly S. Shevchenko

**Introduction:** Juvenile idiopathic arthritis (JIA) is a systemic inflammatory disease which affects mainly joints. The search for factors that regulate the development and the course of the JIA remains relevant for the future. One of them is the provision of vitamin D in children with JIA, which role is not fully understood.

**Objectives:** The aim of this study was to study of the relationship of individual characteristics of the disease course (duration of the disease, disease activity, number of injured and active joints) with the level of the vitamin D metabolite in children with JIA.

**Methods:** Thirty three patients with JIA were examined. The median age of patients was 10,5 ±1,7 years, from 1,8 to 17,6 years (23 female, 10 male) .The total duration of the disease was 3 years 4 months ± 1.3 years, from 2 months to 13 years 2 months.The serum level of vitamin D was measured through blood test by chemiluminescence method. The relationship between the level of vitamin D and disease activity was analyzed based on juvenile arthritis disease activity score (JADAS 27).

**Results:** The average level of vitamin D in serum was 23,05±6,4 ng/ml. It was found that the level of vitamin D was correlated with age of patients (r = -

- 0,43; p < 0,05). Using the regression method, a relationship was established between the level of vitamin D in serum and the age of the child, the number of affected joints and the number of active joints (33,48 – 0,065*age of the patient - 0,55* number of injured joints + 0,37 * number of active joints ).The results of the present study indicated that there was no significant relationship between the level of vitamin D in serum and disease duration and activity.

**Conclusion:** JIA patients are presented with low vitamin D levels. It was found the relationship between the vitamin D deficiency, age of the patient and the number of affected and active joints.

**Disclosure of Interest**

None Declared

### P256 USING ETHNOGRAPHY TO UNDERSTAND TRANSITION IN YOUNG ADULTS WITH JIA

#### Teresa A. Simon^1^, Matt Bradley^2^, Daniel J. Lovell^3^, Brian Shakley^2^, Chen Zhang^2^, Lisa Clark^3^, Nicolino Ruperto^4^, David C. McDonald^2^, Hermine I. Brunner^3^

##### ^1^Bristol-Myers Squibb, Princeton; ^2^LIFT 1428, Chattanooga; ^3^Cincinnati Children’s Hospital Medical Center – PRCSG, Cincinnati, USA; ^4^Istituto G. Gaslini Pediatria II Reumatologia, Clinica Pediatrica e Reumatologia – PRINTO, Genoa, Italy

###### **Correspondence:** Teresa A. Simon

**Introduction:** The abatacept (ABA) Registry^1^ is a medication registry aimed at studying the long-term safety of ABA in juvenile idiopathic arthritis (JIA). Transfer of care is a vulnerable period when young adults with JIA (YAs) are often lost to follow-up for unclear reasons. Participants entering the ABA Registry are on average 13 years old^1,2^; hence 10 years of follow-up includes transferring care from paediatric to adult rheumatology.

**Objectives:** To understand the motivations and beliefs surrounding transition from paediatric to adult care in YAs in the ABA JIA Registry.

**Methods:** An ethnographic qualitative study to understand YA/caregiver beliefs/behavioural drivers related to the realities of transitioning from a paediatric to an adult care environment was conducted. A healthcare ethnographer performed interviews and used a formal interview guide with YAs/caregivers to better understand their lived and clinical environment experiences. Corresponding clinicians were also interviewed. Interviews were audio and video recorded. Transcripts were analysed and coded to identify common themes related to the objective.

**Results:** Completed interviews consisted of five YA/caregiver in-person at-home interviews and four corresponding paediatric rheumatologist interviews. All participants were identified and consented by study coordinators at Cincinnati Children's Hospital Medical Center through the ABA JIA Registry. See Table 1 for YA descriptive data. Preliminary overall emerging themes for a successful transition are as follows. Process begins when patients (pts) are children and includes active and ongoing efforts to teach, encourage and practice behaviours necessary to instil a sense of independence, confidence and responsibility as pts enter adulthood. For parents, knowledge of symptoms and treatment are prioritized. For YAs, measures are taken so YAs feel supported, comfortable and confident in their ability to live with JIA and manage care. For treating physicians, the goal is to facilitate the learnings, behaviours and practices in children and parents to have positive outcomes in transition. In-depth coding is ongoing and will be available at the time of presentation.

**Conclusion:** YAs, caregivers and physicians agree that an early start may be key for a successful transition; however, each has a unique perspective that needs to be considered.

**References**

1. Lovell DJ, et al. *Arthritis Rheumatol* 2017;69(Suppl 10): abstract 2272.

2. Ruperto N, et al. PReS Congress 2016: poster P215.

**Disclosure of Interest**

T. Simon Shareholder of: Bristol-Myers Squibb, Employee of: Bristol-Myers Squibb, M. Bradley: None Declared, D. Lovell Grant / Research Support from: AbbVie, Bristol-Myers Squibb, NIH, Pfizer, Roche, Consultant for: AbbVie, Boehringer Ingelheim, Bristol-Myers Squibb, Celgene, Genentech, GlaxoSmithKline, Janssen, Johnson & Johnson, Novartis, Takeda, UCB, Speaker Bureau of: Genentech, B. Shakley: None Declared, C. Zhang: None Declared, L. Clark: None Declared, N. Ruperto Grant / Research Support from: Bristol-Myers Squibb, Roche, Janssen, Novartis, Pfizer, Sobi, Consultant for: AbbVie, Ablynx, Amgen, AstraZeneca, Baxalta Biosimilars, Biogen Idec, Boehringer Ingelheim, Bristol-Myers Squibb, Celgene, Eli-Lilly, EMD Serono, Gilead Sciences, Janssen, MedImmune, Novartis, Pfizer, R-Pharm, Roche, Sanofi, Servier, Takeda, Speaker Bureau of: AbbVie, Ablynx, Amgen, AstraZeneca, Baxalta Biosimilars, Biogen Idec, Boehringer Ingelheim, Bristol-Myers Squibb, Celgene, Eli-Lilly, EMD Serono, Gilead Sciences, Janssen, MedImmune, Novartis, Pfizer, R-Pharm, Roche, Sanofi, Servier, Takeda, D. McDonald: None Declared, H. Brunner Consultant for: AstraZeneca, Bristol-Myers Squibb, Genentech, Janssen, Novartis, Pfizer, Sanofi, Takeda, Speaker Bureau of: Genentech, Novartis

**Table 1 (abstract P256).** Characteristics of Participating Pts with JIA Approaching Transition

Pt number	Pt 1	Pt 2	Pt 3	Pt 4	Pt 5
Age at registry entry, years	15	13	13	14	13
Current age, years	19	18	18	16	17
Distance from paediatric rheumatology centre, km (miles)	90 (56)	39 (24)	126 (78)	248 (154)	48 (30)
Duration in registry, years	4.1	5.0	4.9	2.4	4.8
Duration of abatacept treatment, years	4.1	2.1	5.2	3.0	3.2
Current pt pain score*	4	3	5	0	8.5
Recent well-being score^†^	4	1.5	4.5	0	8.5
Physician-perceived ability to successfully transfer JIA care^‡^	10	7	8	10	7
Physician Global Assessment of current disease activity^§^	0	0.5	1.5	1	2

*Self-rated: 0=none, 10=extreme; ^†^Self-rated: 0=well, 10=poor; ^‡^Physician-rated: 0=unqualified; 10=fully qualified; ^§^Physician-rated: 0=no activity, 10=maximum activity (all 0.5 increments)

### P257 PREVALENCE OF CO-EXISTING AUTOIMMUNE DISEASE IN JUVENILE IDIOPATHIC ARTHRITIS

#### Teresa A. Simon^1^, Nitesh Ray^2^, Sanket Singhal^2^, Hugh Kawabata^3^, Dan Lovell^4^

##### ^1^Bristol-Myers Squibb, Princeton, USA; ^2^Mu Sigma, Bangalore, India; ^3^Consultant at Bristol-Myers Squibb, Princeton; ^4^Cincinnati Children’s Hospital Medical Center – PRCSG, Cincinnati, USA

###### **Correspondence:** Teresa A. Simon

**Introduction:** Juvenile idiopathic arthritis (JIA) is one of a number of autoimmune (AI) diseases. After cancer and heart disease, AI diseases are the most common type of disease in the US, with an overall estimated prevalence of 3.2 and 5.9% in the US^1^ and Europe,^2^ respectively, depending on the source, with a corresponding increase in mortality.^3^ Many AI diseases share a common pathogenic mechanism, but there are few to no published studies quantifying the occurrence of other AI diseases in patients with JIA.^4^

**Objectives:** To estimate the prevalence of coexisting AI diseases in patients diagnosed with JIA, stratified into two cohorts (<18 and ≥18 years of age).

**Methods:** Using a US medical claims database, two cohorts of patients with a diagnosis of JIA (ICD-9 code 714.3x) between 1 January 2006 and 30 September 2015 were identified in the MarketScan Commercial and Supplemental Medicare database. Patients were required to have ≥180 days of continuous health plan enrolment before the qualifying JIA diagnosis. For each of the JIA cohorts, member age at diagnosis was determined. Thirty-seven different AI diseases were assessed by ICD-9 code. Coexisting AI diagnosis codes were identified 12 months prior to and post JIA diagnosis. A minimum of two of the same diagnosis codes for an AI disease was considered co-existing and contributed to the overall prevalence estimate. A similar study of co-existing AI diseases in an adult RA cohort is also presented to highlight differences in the frequency of AI diseases between patients diagnosed with JIA and those diagnosed with RA.

**Results:** The most frequently reported co-exiting AI diseases, defined as being present in ≥1% of patients in the <18 and ≥18 year cohorts with a JIA diagnosis, and in an RA population, are presented (Table 1). Mean age was 10 years for the <18 cohort, 36 years for the ≥18 cohort and 53 years for the adult RA cohort. Most patients were female. Uveitis was the most frequently occurring AI disease in patients with JIA <18 years of age and SLE was the most common AI disease in patients with JIA ≥18 years of age. Sjögren’s syndrome was most common in adult patients with RA.

**Conclusion:** These results show variability in AI prevalence by age group among patients with JIA and separately for patients with RA. Whether this variability is a consequence of the limitations of healthcare claims data requires further investigation. Understanding the co-existence of AI diseases among patients with JIA may play an important role in patient management, treatment decisions and outcomes.

**References**

1. Jacobson DL, et al. *Clin Immunol Immunopathol* 1997;84:223–43.

2. Eaton WW, et al. *J Autoimmun* 2007;29:1–9.

3. Walsh SJ, Rau LM. *Am J Public Health* 2000;90:1463–6.

4. Simon TA, et al. *Adv Ther* 2017;34:2481–90.

**Trial registration identifying number:** Not applicable

**Disclosure of Interest**

T. Simon Shareholder of: Bristol-Myers Squibb, Employee of: Bristol-Myers Squibb, N. Ray Consultant for: Bristol-Myers Squibb, S. Singhal Consultant for: Bristol-Myers Squibb, H. Kawabata Shareholder of: Bristol-Myers Squibb, Employee of: Bristol-Myers Squibb (retired), D. Lovell Grant / Research Support from: AbbVie, Bristol-Myers Squibb, NIH, Pfizer, Roche, Consultant for: AbbVie, Boehringer Ingelheim, Bristol-Myers Squibb, Celgene, Genentech, GlaxoSmithKline, Janssen, Johnson & Johnson, Novartis, Takeda, UCB, Speaker Bureau of: Genentech

**Table 1 (abstract P257).** Prevalence of co-existing AI diseases

Outcome	JIA <18 years n=7883	JIA ≥18 years n=4867	RA ≥18 years n=286,601
Uveitis	4.44 (3.99, 4.89)	1.50 (1.16, 1.84)	0.52 (0.49, 0.55)
SLE	1.13 (0.90, 1.36)	3.10 (2.62, 3.59)	3.78 (3.71, 3.85)
Type 1 diabetes mellitus	0.79 (0.59, 0.98)	1.87 (1.49, 2.25)	1.83 (1.78, 1.88)
Chronic urticaria	1.74 (1.45, 2.03)	1.71 (1.34, 2.07)	0.71 (0.67, 0.74)
Ankylosing spondylitis	1.67 (1.39, 1.96)	1.48 (1.14, 1.82)	1.16 (1.12, 1.20)
Crohn’s disease	1.18 (0.94, 1.42)	1.62 (1.27, 1.98)	1.12 (1.08, 1.16)
Ulcerative colitis	0.44 (0.30, 0.59)	1.36 (1.03, 1.68)	0.87 (0.83, 0.90)
Sjögren’s syndrome	0.15 (0.07, 0.24)	1.29 (0.98, 1.61)	2.42 (2.36, 2.48)
Raynaud’s syndrome	0.63 (0.46, 0.81)	1.23 (0.92, 1.54)	1.12 (1.09, 1.16)

Data are presented as prevalence rates, % (95% CI). SLE=systemic lupus erythematosus

### P258 RESULTS OF IMMUNIZATION WITH A PNEUMOCOCCAL POLYSACCHARIDE VACCINE IN CHILDREN WITH JUVENILE IDIOPATHIC ARTHRITIS WITHOUT SYSTEMIC MANIFESTATIONS.

#### Ekaterina Alexeeva^1,2^, Margarita Soloshenko^1^, Tatyana Dvoryakovskaya^1,2^, Rina Denisova^1^, Ksenia Isaeva^1^, Anna Mamutova^1^, Nikolay Mayansky^1^, Natalia Tkachenko^1^, Irina Zubkova^1^, Tatyana Kaluzhnaya^1^, Anna Gayvoronskaya^1^, Marika Broeva^1^, Marina Fedoseenko^1^

##### ^1^Federal State Autonomous Institution “National Medical Research Center of Children's Health” of the Ministry of Health of the Russian Federation; ^2^Sechenov First Moscow State Medical University (Sechenov University), Moscow, Russian Federation

###### **Correspondence:** Margarita Soloshenko

**Introduction:** Patients with juvenile idiopathic arthritis (JIA) have an increased risk of being infected. Approximately half of all serious infections in children with JIA are associated with airway involvement.

**Objectives:** Our aim was to study the efficacy and safety of the pneumococcal 13-valent conjugate vaccine (PCV) in children with JIA.

**Methods:** In a prospective cohort study, 5 groups were formed: children with JIA in the remission phase on methotrexate therapy (group Ia) or etanercept (group Ib), with JIA in the active phase prior to the appointment of methotrexate (group IIa) or etanercept (group IIb), control group (conditionally healthy children). 0.5 ml of the 13-valent PCV was administered once subcutaneously during therapy in patients in the remission phase or 3 weeks before the appointment of methotrexate or etanercept in patients in the active phase. The main study outcome was the proportion of patients with a protective (40 mg/L) level of specific anti-pneumococcal antibodies (anti-SPP) IgG to Streptococcus pneumoniae 4 weeks after vaccination. In addition, we assessed the incidence of infectious events before and after vaccination as well as changes in the content of a high-sensitivity C-reactive protein, S100 protein, and post-vaccination period.

**Results:** The study included 125 children. Four weeks after vaccination, the protective level of anti-SPP IgG was established in 21 (84%) patients in the 1st, 23 (92%) in the 2nd, 22 (88%) in the 3rd, 24 (96%) in the 4th and 5th groups (p =1.0). Increase in the concentration of S100 protein and high-sensitivity C-reactive protein after vaccination was not noted. JIA exacerbation episodes were not recorded in any patient.

**Conclusion:** Vaccination with the 13-valent PCV in children with JIA is highly effective and is not accompanied by the flare of JIA.

**Disclosure of Interest**

E. Alexeeva Grant / Research Support from: Roche, Pfizer, Centocor, Novartis, M. Soloshenko: None Declared, T. Dvoryakovskaya Grant / Research Support from: Roche, Pfizer, R. Denisova: None Declared, K. Isaeva: None Declared, A. Mamutova: None Declared, N. Mayansky1: None Declared, N. Tkachenko: None Declared, I. Zubkova: None Declared, T. Kaluzhnaya: None Declared, A. Gayvoronskaya: None Declared, M. Broeva: None Declared, M. Fedoseenko: None Declared

### P259 ULTRASOUND CHANGES IN JOINTS INDUCED BY INTRA-ARTICULAR CORTICOSTEROID INJECTION IN JUVENILE IDIOPATHIC ARTHRITIS.

#### Betul Sozeri^1^, Duygu Kurtulus^2^

##### ^1^Pediatric Rheumatology; ^2^Phisical Therapy and Rehabilitation, University of Health Sciences , Istanbul, Umraniye Training and Research Hospital, Istanbul, Turkey

###### **Correspondence:** Betul Sozeri

**Introduction:** Ultrasonography (US) studies carried out on joints of juvenile idiopathic arthritis (JIA) patients in clinical remission demonstrate the presence of subclinical synovitis. The significance of subclinical synovitis and the positive power Doppler (PD) signal on US in JIA.

**Objectives:** The objectives of this study were to assess whether the changes detected by US induced by intra-articular corticosteroid injection in JIA patients.

**Methods:** We evaluated 49 joints (47 knees, 1 tibiotalar and 1 elbow) of 32 patients who diagnosed JIA. We used grey-scale US by high frequency transducer (7.5-10) MHz at study entry and after a therapeutic intervention. Each joint was scored for grey-scale (GS) and power Doppler (PD) abnormalities according to a 4-point semiquantitative scale. Pre- and post-treatment US scores were compared and the sensitivity to change of GSUS and PDUS was estimated. US assessment was performed separately, immediately after the clinical evaluation, by an experienced pediatric rheumatologist (BS) with certificated by EULAR. Medical records were reviewed for JIA subtype and state of disease. Clinical examination, including routine joint examination was carried. Clinical response was assessed using the ACR paediatric (pedi ACR) response criteria

**Results:** Five patients had polyarthritis, 5 had enthesitis-related arthritis, 22 had oligoarthritis. Nine patients (28%) underwent intra-articular corticosteroid injection (IACI) only, 23 (71,9 %) were given IACI and systemic medications. The medication used were methotrexate (22 patients), Sulfasalazine (2 patients), and methotrexate and biologic (5 patient).

Synovial hyperplasia, joint effusion, PD signal and tenosynovitis in at least one joint were detected in 77.4%, 100%, 33.3% and 15% of patients, respectively. Both GSUS and PDUS scores improved significantly (p<0.0001) from baseline to follow-up. At the follow-up visit, 18/49 (36.7%) joints complete resolution among these patients 2 had minimal synovial hyperplasia. Although, 31/49 (63.3%) joints residual US abnormalities were judged in remission on clinical grounds.

**Conclusion:** US is a sensitive tool to assess therapeutic response in patients with JIA. Subclinical disease on US is common in joints with clinically-defined remission. Further studies are needed to establish the impact of US on therapeutic decision-making in JIA.

**Disclosure of Interest**

None Declared

### P260 ANTI-CITRULLIN PROTEIN ANTIBODIES IN JUVENILE IDIOPATHIC ARTHRITIS

#### Erik Sundberg^1^, Raya Saleh^2^, Karin Lundblad^2^, Helena Erlandsson Harris^2^

##### ^1^Women´s and Childrens´s Health; ^2^Medicine, KAROLINSKA, Stockholm, Sweden

###### **Correspondence:** Erik Sundberg

**Introduction:** Anti-Citrullin Protein Antibodies (ACPAs) are known to precced onset of Reumatoid Arthritis in adults with years to decades and differentiate between different clinical outcomes. Less is known about clinical correlation with ACPA subtypes in Juvenlie Idiopathic Arthritis (JIA) patients.

**Objectives:** We set out to study Cyclic Ctrullin Peptide (CCP) antibodies and ACPA subtypes in patients with JIA in order to correlate precense of antibodies with clinical characeristics.

**Methods:** 280 patients from the Juvenile Arthritis Bio Bank Astrid Lindgrens Children Hospital (JABBA) was analysed with regard to CCP antibodies with av CCP-2 ELISA. APCA sub specificities were analysed in a subset of 90 patients in a Immunocap- ISAC chip system and results were compared to clinical parameters obtained from the Swedich Pediatric Rheumatology registry and medical charts.

**Results:** Results show a precense of CCP antibodies of 6 % compared to 3% in a healthy pediatric control group. All CCP positive patients were female and most commonly poly arthritis patients.  CCP titers were significantly higher in CCP positive JIA patients as compared to CCP positive healthy controls and JIA patients with CCP antibodies display a greater number of ACPA subtypes. ACPA subtypes in the JIA patients included antibodies to Enolase, Vimentin, Fibrogen and antibodies , CPP3, directed to Porphyrominas Gingivalis a known periodontal pathogen. Correlation to clinical outcomes are ongoing.

**Conclusion:** CCP precense in our JIA cohort correspond to findings from other investigators. An increased numer of ACPA subtypes in CCP positive JIA patients indicate epitope spreading in the pathogenic process. Correlation of ACPA subtypes compared to clincal outcomes are ongoing. Interestingly antibodies to CCP-3 were detected strengthening the relation of pediatric periodontal helath and JIA.

**Disclosure of Interest**

None Declared

### P261 REFERENTIAL SKILLS PARTICIPATORY IN JIA FOR CHILDREN, ADOLESCENTS AND PARENTS

#### Sonia Trope^1^, Alexandre Belot^2^, William Fahy^3^, Pierre Quartier Dit Maire^4^, Christelle Sordet^5^, Jean-David Cohen^6^, REAJI working group

##### ^1^ANDAR, PARIS; ^2^CHU, Lyon; ^3^KOURIR; ^4^Necker Hospital, PARIS; ^5^CHU, Strasbourg; ^6^CHU, Montpellier, France

###### **Correspondence:** Sonia Trope

**Introduction:** In a context of strong development of Therapeutic Patient Education (TPE) in Juvenile Idiopathic Arthritis, there is no reference system to support the process of program construction or educational actions.

**Objectives:** The objective was to conduct a participatory plan to build a referential of skills that could help young patients and parents and be useful to healthcare professionnals. (HCP)

**Methods:** On the initiative of an association of patients and a rheumatologist experienced in TPE, a steering committee (COPIL) was constituted bringing together rheumatologists, pediatricians and associative representatives. After a qualitative phase (review of literature and focus groups of children, adolescents and parents), the COPIL identified skills of knowledge, know-how and know-how-to-be in different areas of interest for people affected by JIA. The work by topic allowed the balance between the biomedical and psychosocial.

For the quantitative phase, all the French competence centers as well as the KOURIR association and the RESRIP Network were invited to participate in a validation using different online questionnaires. Each center had to involve HCP, children and parents. For each area, the participants had to indicate the three skills that seemed to them to be priorities and could, if they wanted, to propose a competence not listed. The analysis consisted in classifying the answers by type of responder (Children, parents, HCP) to identify the priorities of each group, then to weight the skills of each theme (3 points for skills ranked first, 2 points for second place skills, 1 point for third place skills). Finally an average was made for the overall ranking.

**Results:** 19 competence centers, Kourir and RESRIP contributed to this approach. Thirteen themes were explored (disease, treatment, pain, care, myself, information, relationship to others, relationship to the health care team, schooling, home, living daily, fatigue and sleep, physical activity-sport-rehabilitation). The "children" skills questionnaire brought together 78 respondents (19 children, 22 parents, 37 HCP), the "teenagers" questionnaire 50 respondents (10 teenagers, 17 parents, 23 HCP), the "parents of children" questionnaire 59 answering machines (8 children, 28 parents, 23 HCP) and the questionnaire "parents of adolescents" 30 respondents (6 teens, 14 parents, 10 HCP). Priorities differ among groups. On the subject of treatment in particular, we note a tendency of parents to privilege theoretical knowledge whereas children focus their choices on coping and living with JIA and the HCP put forward intermediate skills.

**Conclusion:** This collaborative and participatory work makes it possible to identify the children's priorities, without neglecting the expected skills by the parents and the HCP. The classification by theme will allow teams wishing to develop educational approaches to rely on real notions and not only on HCP projections. This repository wants to be closer to problems and expectations of children and their families. It is not, however, a list of ideal skills that all child, adolescent or parent must master but a source of reflection to implement custom actions.

**Disclosure of Interest**

None Declared

### P262 BIOPSYCHOSOCIAL STATUS OF JIA PATIENTS: PERSPECTIVES OF DAILY LIVING ACTIVITIES, DISEASE ACTIVITY AND FAMILY IMPACT

#### Edibe Ünal^1^, Ezgi Deniz Batu Akal^2^, Emine Hafize Sönmez^3^, Zehra Serap Arıcı^4^, Pınar Kısacık^1^, Gamze Arın^1^, Nur Banu Karaca^1^, Duygu Aydın Haklı^5^, Reha Alpar^5^, Yelda Bilginer^3^, Seza Özen^3^

##### ^1^Department of Physiotherapy and Rehabilitation, Hacettepe University Faculty of Health Sciences; ^2^Ankara Training and Research Hospital; ^3^Department of Pediatric Rheumatology, Hacettepe University Faculty of Medicine, Ankara; ^4^Şanlıurfa Training and Research Hospital, Şanlıurfa; ^5^Department of Biostatistics, Hacettepe University Faculty of Medicine, Ankara, Turkey

###### **Correspondence:** Edibe Ünal

**Introduction:** Juvenile Idiopathic Arthritis (JIA) is the most frequent chronic rheumatic disease during childhood. It can result in disabilities, loss of quality of life and mood changes. Furthermore, literature reviewing the effects of arthritis on children and family is inconsistent, with studies showing significant difference or not, compared to healthy children.

**Objectives:** The purpose of this study is to present results regarding the functional status, psychosocial status and disease activity of children with JIA, and their effects on the child's family. The second aim is to present the correlations between these parameters.

**Methods:** The study included children diagnosed with JIA who applied to Hacettepe University İhsan Doğramacı Children's Hospital Rheumatology Department. After demographic data was collected, all children were assessed with Child Health Assessment Questionnaire (CHAQ) for daily living activities, with the Juvenile Arthritis Disease Activity Score (JADAS) for disease activity and with a newly developed scale from Hacettepe University Faculty of Health Sciences Department of Physiotherapy and Rehabilitation for children with rheumatism by Edibe Ünal for functional and psychosocial status. Cut-off point was accepted as ≤2.7 for disease activity. The Family Impact Scale (FIS) was used to assess parents’ perspective.

**Results:** A hundred and ninety-six children were included in the study. The Mean age of children was 12,44±3,97 and female/male ratio was 55,6/44,4. Although the mean JADAS score was 3,33±4,21, it only detected active disease in 81 children. There was a moderate correlation between CHAQ (Pain) and functional scores of Ünal’s scale and JADAS score. CHAQ total score was well correlated with function and psychosocial scores. The correlation between FIS and other scales was very low (Table1).

**Conclusion:** This study concluded that pain and function were affected by disease activity. It was also shown that the psychosocial states that children expressed with their own knowledge are related to their participation in daily living activities. It has been determined that these changes do not reflect in the perspective of the family. It was concluded that the functional and psychosocial aspects of JIA children should also be taken into consideration.

**Disclosure of Interest**

None Declared

**Table 1 (abstract P262).** Correlations

	CHAQ Total	CHAQ Pain	CHAQ Overall	Function	Psychosocial	FIS
JADAS						
r	0,362	0,499	0,336	0,423	0,216	0,245
p	0,000	0,000	0,000	0,000	0,003	0,002
CHAQ (Total)						
r		0,384	0,355	0,757	0,507	0,292
p		0,000	0,000	0,000	0,000	0,000
CHAQ (Pain VAS)						
r			0,357	0,438	0,308	0,210
p			0,000	0,000	0,000	0,006
CHAQ (General VAS)						
r				0,360	0,358	0,296
p				0,000	0,000	0,000
Function						
r					0,474	0,273
p					0,000	0,000
Psychosocial						
r						0,291
p						0,000

### P263 TENDONS GLUCOCORTICOID INJECTIONS IN JUVENILE IDIOPATHIC ARTHRITIS (JIA): FAKE OR RELIABLE TREATMENT OPTION?

#### Andrea Uva^1^, Rebecca Nicolai^2^, Fabrizio De Benedetti^2^, Silvia Magni-Manzoni^2^

##### ^1^Department of Pediatrics, Sapienza-University of Rome; ^2^Rheumatology Division, IRCCS Bambino Gesù Children’s Hospital, Rome, Italy

###### **Correspondence:** Andrea Uva

**Introduction:** Due to the spread use of ultrasound (US)-guidance, JIA patients undergoing glucocorticoid joint injections are more frequently injected in periarticular sites, such as tendons. However, the frequency of tendon injections in JIA patients is unknown and little is available about the frequency of remission at the injected tendons.

**Objectives:** To identify the clinical features of JIA patients with tendon involvement treated with glucocorticoid injection, the tendon sites most commonly injected, the frequency of and the time for achieving remission in the injected tendons.

**Methods:** Records of patients with JIA from the database of glucocorticoid injection procedures performed at the study centre in a 5 years period which included tendon injections were reviewed. Demographic and clinical features, sites of injection, procedure setting, glucocorticoid used, time to achieve local remission, disease activity in the injected sites at the last follow-up, and eventual local side-effects were recorded.

**Results:** 262 procedures with an amount of 610 tendon injections in 182 JIA patients (79% females) were identified, out of a total of 715 injection procedures (37%). Persistent oligoarthritis was represented in the 54.4% of the patients, extended oligoarthritis in 19.8% (36), polyarthritis in 24.7%, systemic JIA in 1.1%. The median age of the patients at the time of the injection was of 8 years (IQ 4.6-11.3). All procedures were US-guided; 97% were performed under general anaesthesia. Acetate methylprednisolone was the glucocorticoid used in all the injections except 16 (2.6%), performed with triamcinolone hexacetonide. Type and frequencies of the most injected sites are shown in Table 1. In 226/262 procedures (86%), patients experienced remission in the injected tendons after a median of 2.3 months (IQ, 1.9-3.2). Forty patients underwent repeated procedure in the same tendon due to flare or persistent activity after at least 2 months from the first injection. A total of 61 tendons (10%) were re-injected. At the last follow up, after a median of 21.7 months (IQ 8.4-36), 165 children out of 182 (90.6 %) were in remission in the injected site. Thirty-five out of the 40 patients who underwent to repeated injection in the same site (87.5%) also showed remission at the last follow-up. Twenty out of 182 patients (11%) showed local side effects (subcutaneous atrophy, cutaneous hypopigmentation); no other side effects were reported.

**Conclusion:** Tendons have been frequently injected at a tertiary care Paediatric Rheumatology centre for the management of inflammatory involvement outside the joint space in children with JIA, mostly represented by the oligoarthritis subtype. The posterior tibialis tendon and the flexor digiti proprii tendons were the most frequently injected. Local remission was observed in 86% of the procedures after a median of 2.3 months and was found in the majority of the patients at the last follow up, after a median of almost 2 years. Further studies are planned to explore the impact of concomitant medication in achieving and maintaining local remission and the role of imaging in supporting the definition of remission in the injected sites.

**Disclosure of Interest**

None Declared

**Table 1 (abstract P263).** Frequency of injections in specific tendons in the 182 JIA patients of the study

Type of tendons injected	Nr. of injections (%)
Posterior tibialis tendon	172 (28.2%)
Flexors digiti proprii tendons (from I to V)	135 (22.1%)
Peroneals	113 (18.5%)
Wrist extensors (from I to VI compartment)	67 (11%)
Flexor digitorum of the foot tendon	56 (9.2%)
Flexor hallucis longus tendon	22 (3,6%)
Others	45 (7.4%)
Total	610 (100%)

### P264 EVALUATION OF CLINICAL ACTIVITY AND TREATMENT RESPONSE IN UKRAINIAN CHILDREN WITH JIA

#### Yulia Vyzhga

##### Vinnytsya national medical university N.I. Pirogov, Vinnytsya, Ukraine

**Introduction:** According to official statistic data in Ukraine there are more than 3 thousand kids suffer from JIA and disease has strong tendency to spreading. The most dangerous, that up to 50% of the patients lose functional abilities within 3 – 5 years of life, up to 30% of them has high activity of the inflammatory response on basic treatment. Well known, that pathogenesis of the JIA is mediated by molecular interactions between immune cells, inflammatory cytokines, macrophages, that lead to hyper activation of the inflammation response, somehow connected with NF-kB. Previous in vitro studies presented important role of NF-kB for generation of the active response on the treatment of the process, caused by active inflammation reaction. Goals of the JIA treatment are suppression of inflammation activity, dangerous clinical signs, limit iatrogenic effect of concomitant treatment and support condition of the patient. Ukrainian kids with JIA has limited access to biology treatment, that is why very often we get complicated variants if the disease with significant influence on patients quality of life.

**Objectives:** The aim of our study was to evaluate efficacy of the standard basic treatment, and in a combination with available biological drugs, its influence on inflammation cytokines, NF-kB in kids with JIA.

**Methods:** For the achievement of the goals we checked up 78 kids of our center with estimated diagnose of JIA, mid age of them – 12,7 years, mid duration of the disease – 19 months. Most of the kids had polyJIA (52%) in seronegative subtype, seropostivite variant of polyJIA was met rarely – in 7,6%, all other cases – 40,4% were presented by oligoJIA; we didn’t take into account sJIA. For characteristic of the clinical features, we took into account amount of the active joints, results of CHAQ, VAS, ESR value. According to JADAS27-ESR score, most of the patients had moderate disease activity (5,2±0,7) – 62,8%. For estimation of the laboratory activity we checked absolute amount of CRP, IL-1β, IL-6 and NF-kB in serum of the patients with JIA, by using of the ELISA method.

**Results:** Laboratory activity of the inflammatory response was presented by enlarged concentration of CRP (7,2±3,1g/l), ІL-1β (5,8±4,2 pg/ml; N ranges <5 pg/ml), IL-6 (10,2±2,4pg/ml; N ranges <9 pg/ml), and as well high concentration of NF-kB (70,5±3,1pg/ml; N ranges 20 – 48 pg/ml). Concentration of CRP, IL-1β was slightly increased through all groups of clinical variants JIA, IL-6 and NF-kB correlated with activity of the clinical signs, were significantly higher in patients with polyJIA. To evaluate efficacy and response on treatment, we checked="checked" the same laboratory parameters in 6 months after the treatment: with methotrexate in 51 (65,3±3,9%) patients, with methotrexate+adalimumab in 17 (21,8±1,9%) and methotrexate+tocilizumab in 10 (12,8±1,4%) patients. In a 6 months after the treatment 66,7% patients with MTX achieved ACR30 response, 76,4% of patients MTX+ADA and 90,0% of patients MTX+TOC achieved ACR50. In a group of MTX patients we didn’t observe significant decrease of IL-6 or NF-kB concentration, it was at the equivalent level with primary results. Group of the patients with MTX+ADA showed significant lowering of CRP, IL-1β, and IL-6 on 28,7% to compare with baseline level. In group of MTX-ADA decreasing of NF-kB wasn’t clinically significant. Group of the patients that received MTX+TOC presented the best results – with significant decreasing of CRP, IL-1β and IL-6. As well was observed significant decreasing of NF-kB on 41,7 %, that can be estimated as pathogenically linked therapeutic effect.

**Conclusion:** So, patients with JIA that meet high concentration of IL-6 and NF-kB as laboratory predictors of high clinical activity, are more lucky to get good treatment response in case of start therapy of MTX-TOC to compare with MTX-ADA or isolated MTX treatment.

**Disclosure of Interest**

None Declared

### P265 MONOARTICULAR JUVENILE IDIOPATHIC ARTHRITIS: AN ENTITY IN ITS OWN RIGHT. A PROOF-OF-CONCEPT STUDY

#### Francesco Zulian^1^, Caterina Politi^2^, Vanessa Cecchin^2^, Alessandra Meneghel^2^, Giorgia Martini^2^

##### ^1^Department of Woman and Child Health; ^2^University of Padua, Padova, Italy

###### **Correspondence:** Francesco Zulian

**Introduction:** To date, monoarticular Juvenile Idiopathic Arthritis (monoJIA) is included in the oligoarticular subgroup (oligoJIA) although a few studies, in the past, have highlighted some distinctive features.

**Objectives:** We investigated whether patients with persistent isolated monoJIA have peculiar clinical features and outcome as compared to those with oligoarticular disease.

**Methods:** From a cohort of patients with oligJIA followed for at least 5 years since the disease onset, monoJIA were selected on the basis of the exclusive involvement of one single joint during the overall disease course. Each patient was followed with check visits, including ophthalmologic evaluation, every 3-4 months. The following parameters were considered: sex, age at onset, presence and family history of joint hypermobility (JH), ANA, presence and site of active arthritis and uveitis, treatment during the overall course of the disease and final outcome. Treatment was grouped in four categories: intra-articular corticosteroid injection (IACs), non-steroidal anti-inflammatory drugs and/or oral corticosteroids (AIDs), MTX and other disease-modifying anti-rheumatic drugs (DMARDs), and biological agents (BAs). Outcome was defined according to Wallace criteria^3^ as: clinical remission (CR), clinical remission on medication (CRM) and active disease (A).

**Results:** 236 patients with oligoJIA, followed for mean 9.6 years (5-22.6) entered the study. 172 (72.9%) presented oligoJIA and 64 (27.1%) monoJIA. Within the monoarticular forms, the knee was the most affected site (79.9%) followed by ankle (10.9%). MonoJIA resulted significantly different from oligoJIA for the lesser frequency of ANA+ (75.0 vs 85.5%, p = 0.05) and uveitis (18.8 vs 37.2%, p = 0.007), the higher frequency of JH (85,9 vs 70.3%, p = 0.015) and family history for JH (60.0% vs. 31.3%; p = 0.010).

As for treatment, a greater use of systemic drugs were recorded in oligoJIA than in monoJIA (P = 0.000) while the frequency of subjects undergoing IACS injection was comparable between the two groups (93.0% vs 89.1%). At 5 year follow up, 79.9% monoJIA were in CR and only 10.9% were still active. Conversely, only 24.4% oligoJIA were in CR and 52.3% were still active (p 0.0001).

**Conclusion:** MonoJIA seems to be an entity in its own right with less frequency of ANA and uveitis, less severity course and better long-term outcome than oligoJIA. These preliminary findings may open new insights on JIA classification and, maybe, on a different treatment approach.

**Disclosure of Interest**

None Declared

## Poster walk 6: LES and scleroderma

### P266 HER2 AS A URINARY BIOMARKER OF LUPUS NEPHRITIS ACTIVITY: RESULTS OF A MULTICENTER PEDIATRIC PROSPECTIVE STUDY AND AN ADULT STUDY

#### Patricia Costa-Reis^1^, Kelly Maurer^2^, Laura E. Schanberg^3^, Jon M. Burnham^2^, Emily von Scheven^4^, Kathleen M. O'Neil^5^, Marisa K. Gitelman^6^, Michelle Petri^7^, Kathleen E. Sullivan^2^

##### ^1^Hospital de Santa Maria, Lisbon, Portugal; ^2^The Children's Hospital of Philadelphia, Philadelphia; ^3^Duke University, Durham; ^4^University of California San Francisco, San Francisco; ^5^Indiana University, Indianapolis; ^6^Children’s Hospital of Chicago, Chicago; ^7^Rheumatology, Johns Hopkins University School of Medicine, Baltimore, USA

###### **Correspondence:** Patricia Costa-Reis

**Introduction:** It was recently shown that the Human Epidermal Growth Factor Receptor 2 (HER2) participates in the pathogenesis of Lupus Nephritis (LN). HER2 expression is dramatically increased in the kidneys of patients with LN and not in other proliferative glomerulonephritides. Furthermore, HER2 is increased in the kidneys of NZM2410 mice, a lupus nephritis mouse model, and correlates with disease activity. Interferon alpha was identified as a driving force for HER2 overexpression. Interestingly, a connection was found between HER2 and epigenetics. HER2 overexpression is associated with a decrease on miR-26a and miR-30b, two microRNAs that control the expression of cell cycle related genes, including Cyclin E2, in human mesangial cells. It is, therefore, hypothesized that HER2, miR-26a and miR-30b control mesangial cell proliferation in LN.

Preliminary data showed that urinary (u) HER2 is increased in LN, but further studies are necessary to address the real clinical value of uHER2 for the diagnosis and monitoring of this disease and as a prognostic tool.

**Objectives:** To determine the clinical value of uHER2 as a biomarker of LN activity.

**Methods:** A multicentre prospective study of children with LN was performed. Clinical data and urine samples were collected in each visit. Simultaneously, clinical data and urine samples from adult patients with systemic lupus erythematosus (SLE) were obtained from the Johns Hopkins lupus cohort. Controls were age- and gender-matched individuals without renal disease. Active lupus nephritis was defined as renal SLEDAI ≥4. Urine supernatants were analyzed by commercial enzyme-immunoassays for HER2, TWEAK and VCAM1.

T tests and Mann-Whitney U tests were used for comparisons between samples. The relationship between HER2 and SLEDAI and HER2 and other urinary biomarkers was defined by Spearman’s correlation.

**Results:** We analyzed 100 children with LN followed at five Pediatric Rheumatology Academic Centers, median age 15 YO, female to male ratio 3.4:1, 33% African-American, 31% Hispanic, 18% Asian and 18% Caucasian. Mycophenolate mofetil was used in 89% and IV cyclophosphamide in 34% of the patients.

Children with LN had significantly higher levels of uHER2 compared with controls (p=0.008), particularly those with active LN at time of urine collection (p=0.003). uHER2/Creatinine correlated with uTWEAK/Creatinine (p=0.0001) and uVCAM1/Creatinine (p=0.018) levels.

Regarding the adult study, 142 SLE patients and 23 controls were analyzed. uHER2/Creatinine levels were increased in SLE patients when compared to controls (p=0.04), once again, this difference was higher in patients with active LN at the time of urine collection (p=0.004). Furthermore, uHER2/Creatinine levels correlated with SLEDAI at the time of urine collection (p=0.0039).

**Conclusion:** A new non-invasive biomarker is much needed to guide clinical practice in LN. In two large prospective studies, one in children and another in adults, it was shown that uHER2 is significantly increased in LN patients, particularly when the disease is active, and that it correlates with SLEDAI and other LN biomarkers. These are promising results for the role of HER2 in clinical practice. Moreover, these results establish a rationale for the development of other studies focusing on the use of the already available HER2 drugs for the treatment of LN.

**Disclosure of Interest**

None Declared

### P267 METHOTREXATE IN LINEAR SCLERODERMA: LONG-TERM OUTCOME IN 50 CHILDREN FROM A SINGLE PEDIATRIC RHEUMATOLOGY CENTRE

#### Gloria Fadanelli, Anna Agazzi, Fabio Vittadello, Alessandra Meneghel, Francesco Zulian, Giorgia Martini

##### Pediatric Rheumatology Unit, Department of Woman and Child Health, University Hospital of Padua, Padua, Italy

###### **Correspondence:** Gloria Fadanelli

**Introduction:** Linear Scleroderma (LS) is the most common subtype of Juvenile Localized Scleroderma (JLS) and the one most frequently associated with tissue damage and functional disability. During the last decade, methotrexate (MTX) has been used as first choice agent for LS but, to date, available data on long-term outcome are partial and incomplete.

**Objectives:** To assess the disease course and the long-term outcome of children with LS treated with MTX since diagnosis.

**Methods:** We conducted a retrospective and cross-sectional study including children with LS followed at our Paediatric Rheumatology Centre between 2000 and 2016. Patients treated with MTX for at least one year and currently followed at our Centre with at least one visit in the last 2 years were included. Disease course was evaluated by retrospective analysis of clinical features, type of treatment and recurrence. Outcome at the last visit was assessed by evaluation of disease activity and severity of tissue damage and/or functional impairment by Localized Scleroderma Cutaneous Assessment Tool (LoSCAT) and thermography.

**Results:** 50 patients, 24 with LS of the trunk/limbs and 26 with LS of the face, entered the study. The mean follow-up was 7.8 years (median 6.8, range 2.1-16.3) and MTX treatment mean duration was 3.1 years (median 2.9, range 1.8-8.5 years). Only 16% patients did not respond to first therapy with MTX, 16% had at least one flare of the disease, on average 30 months after starting MTX, but 10% presented recurrent relapses. Within 5 years follow-up, 81.8% of patients achieved partial remission (inactive disease on treatment) and 18.2% complete remission (inactive disease, off treatment for >2 years), but by 10 years follow-up 80.0% obtained complete remission. In the group of 16 patients with >10 years of follow-up, complete remission was reported in 87.5%. At last evaluation tissue damage was mild in 42% of cases, moderate in 32% and severe in 26%. Various degree of functional limitation was reported in 41.7% of patients with LS of the limbs. Severity of tissue damage was not related to LS subtype and disease duration, while functional disability was more frequent in LS of the limbs (p=0.039). Subtype of LS and severity of tissue damage were not associated with disease recurrence and achievement of remission, while length of therapy was related to disease relapses (p=0.040) and severity of tissue damage (p=0.003).

**Conclusion:** Most patients with LS treated with MTX achieve complete remission without flares and only a minority present repeated relapses and active disease after more than 10 years. The long-term monitoring of the patients even after therapy withdrawal is crucial to promptly identify possible flares and treat them adequately, as they tend to recur. Overall aesthetic and functional sequelae are moderate, probably because tissue damage establishes early in disease course as its severity does not seem to be related to disease duration.

**Disclosure of Interest**

None Declared

### P268 ASSESSING THE PREVALENCE OF LOCALIZED SCLERODERMA IN CHILDHOOD USING ADMINISTRATIVE CLAIMS DATA FROM THE UNITED STATES

#### Tim Beukelman^1^, Fenglong Xie^2^, Ivan Foeldvari^3^

##### ^1^Department of Pediatrics; ^2^Department of Medicine, University of Alabama at Birmingham, Alabama, USA; ^3^Hamburg Center for Pediatric and Adolescent Rheumatology, Am Schoen Klinik Eilbek, Hamburg, Germany

###### **Correspondence:** Ivan Foeldvari

**Introduction:** Juvenile Localised Scleroderma (jlSc) is believed a rare autoimmune disease, which occurs 10 times more often then systemic sclerosis in childhood and is believed to have a prevalence of one per 100 000 children(1). There is no prevalence data published.

**Objectives:** We aimed to calculate the prevalence of jlSc in childhood using administrative claims data.

**Methods:** We used Truven MarketScan® commercial insurance claims data from the United States for the years 2010 through 2014, inclusive. MarketScan contains billing records associated with physician office visits, outpatient infusions, and pharmacy claims, among other data, and is intended to be representative of all persons covered by employer-sponsored health insurance in the United States. In each individual calendar year, we identified all persons in the claims data who were less than 16 years old.

**Results:** The results for each calendar year are shown in Table 1. There were approximately 1600-2200 children per year with diagnoses of localized scleroderma and <3% of them had concurrent diagnoses of systemic sclerosis or mixed connective tissue disease. The prevalence estimates in each year ranged from 3.2 to 3.6 per 10,000 children. The proportion of children with localized scleroderma who received methotrexate in each year ranged from 3.2% to 4.4%.

**Conclusion:** This prevalence data showes a higher than expected prevalence compared to the published incidence data with with 2.7 per 100 000 to 2.5 children per million per year. We need more prevalence data from other resources to reassure our findings.

**Disclosure of Interest**

None Declared

**Table 1 (abstract P268).** The estimated prevalence of juvenile localised Scleroderma in the United States

Year	Total Children (N)	Diagnosis Code for Localized Scleroderma (N)	No Diagnosis Code for Systemic Sclerosis or Mixed Connective Tissue Disease (N)	Use of Methotrexate	Estimated Prevalence per 10,000 Children [95% CI]
2010	5,894,628	2064	2006 / 2064	75 / 2006	3.4 [3.3-3.6]
2011	6,231,475	2269	2222 / 2269	86 / 2222	3.6 [3.4-3.7]
2012	6,278,118	2198	2154 / 2198	68 / 2154	3.4 [3.3-3.6]
2013	4,950,018	1732	1692 / 1732	61 / 1692	3.4 [3.3-3.6]
2014	4,933,523	1620	1588 / 1620	71 / 1588	3.2 [3.1-3.4]

### P269 AFTER 12 MONTHS OBSERVATION PERIOD THE PATIENTS RELATED OUTCOMES IMPROVE SIGNIFICANTLY IN THE JUVENILE SCLERODERMA INCEPTIONS COHORTE. WWW.JUVENILE-SCLERODERMA.COM

#### Ivan Foeldvari^1^, Jens Klotsche^2^, Ozgur Kasapcopur^3^, Maria Teresa Terreri^3^, Maria Katsikas^3^, Valda Stanevicha^3^, Tadey Avcin^3^, Rolando Cimaz^3^, Mikhail Kostik^3^, W.-Alberto Sifuentes-Giraldo^3^, Flavio Sztajnbok^3^, Jordi Anton^3^, Simone Appenzeller^3^, Dana Nemcova^3^, Maria Jose Santos^3^, Christina Battagliotti^3^, Lillemor Berntson^3^, Jürgen Brunner^3^, Despina Eleftheriou^3^, Liora Harel^3^, Mahesh Janarthanan^3^, Tilmann Kallinich^3^, Monika Moll^3^, Susan Nielsen^3^, Vanessa Smith^3^, Kathryn Torok^3^, Nicola Helmus^1^

##### ^1^Hamburg Center for Pediatric and Adolescent Rheumatology, Am Schoen Klinik Eilbek, Hamburg; ^2^German Rheumatism Research Center, Berlin; ^3^jSSc Collaborative Group, Hamburg, Germany

###### **Correspondence:** Ivan Foeldvari

**Introduction:** Juvenile systemic scleroderma (jSSc) is an orphan disease with a prevalence 3 in a 1 000 000 children(1).  There is not much published data about the course of jSSc with standardized assessment of the patients. We report our date form juvenile scleroderma inception cohorte with a follow up of 12 months in the cohort.

**Objectives:** to evaluate the development of the disease during the first 12 months in the cohorte

**Methods:** The juvenile scleroderma inception cohorte is a prospective multicenter registry of patients with jSSc, who fullfill the adult classification criteria, and have age of disease onset of less then 16 years of age and less then 18 years at the time of inclusion in the cohort. We evaluted the patients characteristics of the patients, who were followed 12 months in the registry.

**Results:** 60 patients were followed 12 months in the registry. 77% were female and 75% had diffuse subtype. 18% had overlap feutures. Mean disease duration at time of inclusion was 3.2 years. Mean age of onset of Raynauds was 8.8 years and the first non-Raynauds 9.5 years. 78% received DMARDs at the time of inclusion and 90% after 12 months. 87% of the patients were ANA positive, around 35% anti-Scl70 positive and around 2% anticentrome positive.  The mean modified skin score decreased from 18 to 15.1. The frequency of Raynaud´s stayed around 75%. The frequency of the nailfold capillary changes increased from 67% to 72%, but the frequency of active ulcerations decreased from 23% to 19%. The number of patients with FVC <80 % decreased from 41% to 17% (p=0.027). The number of patients with DLCO< 80% increased from 46 to 54% (p=0.651). The number of patients wiht pulmonary hypertension assessed by ultrasound decreased from 12% to 8% ( p=0.543). No patient developed hypertension or renal crisis. Gastrointestinal involvement stayed stable around 37%. Number of swollen joints decreased from 22% to 10% (p=0.08). Total muscle weakness decreased from 24 % to 7% (p=0.031) amd elevated CK from 28% to 11% (p=0.045) too. Several patient related outcomes improved significantly. Patient global disease activity (VAS 0-100) from 51 to 36(p=0.021) and patient global disease damage (VAS 0-100) from 49 to 37 (p=0.049) as physitian global disease activity (VAS 0-100) from 50 to 31 (p=0.001) and physitian ulceration activity (VAS 0-100) from 17.5 to 8.5(p=0.038).

**Conclusion:** Over the 12 months observation period patient related outcomes , number of patients with decreased FVC, number of swollen joints and number of patients with muscle weakness improved significantly and fortunately the other clinical parameters stayed stable. It seems, that the current treatment can reach improvement on some areas and at least stabilisation of the patients.

1. Beukelman T, Xie F, Foeldvari I. Assessing the Prevalence of Juvenile Systemic Sclerosis in Childhood Using Administrative Claims Data from the United States. Journal fo Scleroderma and Related Disorders. 2018;3.

**Disclosure of Interest**

None Declared

### P270 UPDATE FROM THE JUVENILE SCLERODERMA INCEPTION COHORT. WWW.JUVENILE-SCLERODERMA.COM

#### Ivan Foeldvari^1^, Jens Klotsche^2^, Ozgur Kasapcopur^3^, Amra Adrovic^3^, Kathryn Torok^3^, Valda Stanevicha^3^, Flavio Sztajnbok^3^, Maria Teresa Terreri^3^, Ekaterina Alexeeva^3^, Jordi Anton^3^, Maria Katsikas^3^, Vanessa Smith^3^, Tadey Avcin^3^, Rolando Cimaz^3^, Mikhail Kostik^3^, Thomas Lehman^3^, W.-Alberto Sifuentes-Giraldo^3^, Simone Appenzeller^3^, Mahesh Janarthanan^3^, Monika Moll^3^, Dana Nemcova^3^, Maria Jose Santos^3^, Christina Battagliotti^3^, Lillemor Berntson^3^, Blanca Bica^3^, Jürgen Brunner^3^, Patricia Costa Reis^3^, Despina Eleftheriou^3^, Liora Harel^3^, Gerd Horneff^3^, Tilmann Kallinich^3^, Dragana Lazarevic^3^, Kirsten Minden^2^, Susan Nielsen^3^, Yosef Uziel^3^, Nicola Helmus^1^

##### ^1^Hamburg Center for Pediatric and Adolescent Rheumatology, Am Schoen Klinik Eilbek, Hamburg; ^2^German Rheumatism Research Center, Berlin; ^3^jSSc Collaborative Group, Hamburg, Germany

###### **Correspondence:** Ivan Foeldvari

**Introduction:** Juvenile systemic scleroderma(jSSc) is an orphan disease with a prevalence of 3 in 1 000 000 children(1). There is no large date published regarding the clinical presentation of jSSc. The juvenile systemic scleroderma inception cohort is a multinational cohort with a prospective standardized assessment of the patients. We present the clinical characteristics of the patients at time of the inclusion in the cohort.

**Objectives:** Evalution of the jSSc patients at the time of inclusion in the juvenile scleroderma inception cohorte

**Methods:** The juvenile scleroderma inception cohorte is a prospective multicenter registry of patients with jSSc, who fullfill the adult classification criteria, and have an age of disease onset of less then 16 years and less then 18 years of age at the time of inclusion in the cohort. We evaluated the patients characteristics at time of inclusion in the cohort.

**Results:** Till 15th of April 2018 hunderdnine patients were included. 80% of them had diffuse subtype. 90% of them were caucasian and 79% female. 15% of them had overlap feutures. Mean age of onset of Raynauds was 9.7 years in the diffuse subtype(djSSc) and 10.5 years in the limited subtype(ljSSc) ( p=0.62). The mean age of non-Raynauds was 10.2 in the djSSc and 11.2 in the ljSSc (p=0.52). Mean disease duration at time of inclusion was 3.4 in the djSSc and 2.5 in the ljSSc group. Around 80% received DMARDs. ANA positivity was around 86% in both groups. Anti-Scl70 was 32% in djSSc and 39% in the ljSSc group. Anticentromere positivity was 4% in the djSSc and 11% in the ljSSc group(p=0.28). Mean Modified Rodnan skin score was 18.2 in the djSSc and 7.7 in the ljSSc (p=0.001). Gottron papulae were significantly more common the djSSc woth 27% compared to 7% in the ljSSc group. History of ulceration was significantly more common in the djSSc with 57% comparedot 29% in the ljSSc group ( p=0.010). FVC<80% occured in 34% in hte djSSc and 21% in the ljSSc group(p=0.295). DLCO <80% occured 44% in the djSSc and 43% in the ljSSc group. Pulmonary hypertension assessed by ultrasound occured 7% in both groups. No hypertension or renal crisis was reported or observed. Gastointestinal involvement occured 35% in the djSSc and 24% in the ljSSc (p=0.28). Number of swollen joints were observed around 20% in both groups. Muscle weakness wtih joint contractures were present in 12% in the djSSc and 28% in the ljSSc group. Tendon friction rub was present in 4 % in djSSc and 7% in the ljSSc group. djSSc patients had significantly worse scores for physitian global disease activity (VAS 0-100) 41.6 compared to 30.7 ( p=0.041) and for physitain global disease damage(VAS 0-100) 38.2 compared to 18.9 ( p=0.023).

**Conclusion:** In this large cohort of jSSc patients are surprisingly not many significant differences between djSSc and ljSSc.  According the physitian global the djSSc paitents have a significantly more severe disease.

1. Beukelman T, Xie F, Foeldvari I. Assessing the Prevalence of Juvenile Systemic Sclerosis in Childhood Using Administrative Claims Data from the United States. Journal fo Scleroderma and Related Disorders. 2018;3.

**Disclosure of Interest**

None Declared

### P271 THE PHAGOCYTIC SIGNATURE IN JUVENILE-ONSET SYSTEMIC LUPUS ERYTHEMATOSUS (JSLE) CAN BE CAUSED BY STIMULANTS IN THE BLOOD

#### Anna Elisa A. Glaser^1^, Angela Midgley^1^, Helen L. Wright^2^, Matthew Peak^3^, Michael W. Beresford^4^

##### ^1^Department of Women's and Children's Health; ^2^Department of Musculoskeletal Biology, University of Liverpool; ^3^Clinical Research Division; ^4^Department of Paediatric Rheumatology, Alder Hey Children’s NHS Foundation Trust, Liverpool, UK

###### **Correspondence:** Anna Elisa A. Glaser

**Introduction:** JSLE is a multisystem autoimmune disease with autoantibodies potentially caused by dysregulated phagocytosis and increased apoptotic burden. Our metabolomics data from JSLE serum and transcriptomic analysis of neutrophils (PMN) suggest dysregulation of phagocytosis related genes (PRGs), many of which are dependent on the interferon (IFN)-induced gene signature (IGS) of the patient.

**Objectives:** Investigate the contribution of IFNa, TNFa and apoptotic environment to the expression of PRGs.

**Methods:** PMNs from healthy controls and JSLE patients were tested for expression of PRGs (CR3, FCGR3B(CD16b), TLR2, S100A9) and IGS (IFI6, LY6E, OAS2, IFI44L) genes. CD16b, TLR2 and S100A9 protein was measured with flow cytometry and ELISA. Phagocytic ability was assessed by phagocytosis assays using different pHrodo coated bioparticles. Apoptotic supernatant was collected from 1.5x10^6^ PMNs incubated for 24 h at 37°C. Healthy paediatric control PMNs were stimulated with either IFNa (10ng/ml) or TNFa (1ng/ml). Healthy adult control PMNs were stimulated with apoptotic supernatant (+/- 5%PBMC) or media. PRG expression was measured with qPCR. Protein expression was measured with flow cytometry and ELISA.

**Results:** Significant increase in expression (see Table 1) was found for CD16b, TLR2 and S100A9 mRNA and protein in JSLE patients compared to controls; CD16b was only significant after patients were stratified by their IGS. This profile suggests a strong phagocytic ability which we confirmed with phagocytosis assays. We found IFNa to regulate CD16b expression, while apoptotic supernatant caused CD16b shedding. TNFa increased TLR2. We did not find significant differences in S100A9 protein expression for any stimulation.

**Conclusion:** Expression of PRGs and proteins is strongly up-regulated in JSLE patients and can partly be explained by factors in the lupus environment. A “ready to phagocytose” phenotype could make neutrophils oversensitive to phagocytic triggers or danger molecules. This is further suggested by our metabolomics data indicating that neutrophils are active in patients even in non-flaring patients. Over-reactive cells can cause harm to the body once flares occur. The molecules investigated are therefore potential targets for preventive treatment.

**Disclosure of Interest**

None Declared

**Table 1 (abstract P271).** See text for description.

Condition	Time	Assay	Number of replicates	p-value for detected significant changes
Control JSLE	0 h	mRNA	n=13	p=0.003 TLR2; p=0.01 S100A9 for Ctrl vs. JSLE
				Stratified JSLE: CD16b p=0.01 IFN high vs Ctrl;
				p=0.02 IFN high vs IFN low
		protein	n=7 control	p=0.02 TLR; p=0.03 S100A9
			n=6 JSLE	
		phagocytosis	n=7 control	p=0.04 E.coli
			n=6 JSLE	
IFNa	2 h	mRNA	n=6	p=0.03 CD16b
	7 h	protein	n=5	p=0.02 CD16b
TNFa	30min	mRNA	n=5	n=6 required for analysis
	2 h	protein	n=6	p=0.03 TLR2
Apoptotic supernatant	2h, 6h	mRNA	n=5	p=0.01 CD16b
	6h	protein	n=5	p=0.02 CD16b

### P272 INTERFERON-Γ CORRELATES WITH DISEASE ACTIVITY IN PEDIATRIC SYSTEMIC LUPUS ERYTHEMATOSUS AND POTENTIATES THE PRODUCTION AND THE ACTIVITY OF TYPE I INTERFERONS

#### Gian Marco Moneta^1^, Claudia Bracaglia^1^, Ivan Caiello^1^, Chiara Farroni^2^, Luisa Bracci-Laudiero^3^, Rita Carsetti^2^, Fabrizio De Benedetti^1^, Emiliano Marasco^1^

##### ^1^Division of Rheumatology; ^2^B Cell Physiopathology Unit, Immunology Research Area, OSPEDALE PEDIATRICO BAMBIN GESÙ; ^3^Institute of Translational Pharmacology, CNR, Roma, Italy

###### **Correspondence:** Emiliano Marasco

**Introduction:** Pediatric systemic lupus erythematosus (pSLE) is an autoimmune disease characterized by the production of autoantibodies against self-antigens and immune dysregulation, resulting in tissue damage. Standard treatments of pSLE are high-dose glucocorticoids and immunosuppressive agents; however, a sustained response is still not achieved in a significant proportion of patients. In the last decade, several studies showed an up-regulation of genes induced by type I interferons in peripheral blood and tissues of pSLE patients. The expression of this group of genes, known as the interferon signature, correlates with disease activity. More recently the type II interferon has been implicated in pSLE; however, its precise role has not been extensively investigated yet.

**Objectives:** To investigate the role of type II IFN, IFNγ, in the pathogenesis of pSLE evaluating: 1) the expression levels of IFN-related genes and serum levels of IFN-related chemokines in the peripheral blood of pSLE patients; 2) the cross-talk between type II and type I IFNs.

**Methods:** Expression levels of type I IFN-induced genes (IFI27, IFI44L, IFIT1, RSAD2, ISG15, SIGLEC1) and type II IFN-induced genes (CXCL9, CXCL10, IDO1) in peripheral blood of pSLE patients was evaluated by quantitative PCR (qPCR). Serum levels of IFNγ-related chemokines were measured by ELISA. For each patient, SLEDAI score was calculated. Human peripheral blood mononuclear cells (PBMCs) from 6 HD were stimulated *in vitro* with recombinant human IFNγ and IFNα2b and gene expression was evaluated by qPCR.

**Results:** Expression levels of both IFNα-induced genes and IFNγ-induced genes were increased in the peripheral blood of pSLE patients with active disease (n=12) compared to healthy donors (HD) (n=10) and pSLE patients with inactive disease (n=13). We developed a type II IFN score similarly to the type I IFN score described by Crow et al. Type I and type II IFN scores were calculated for each pSLE patient. The type II IFN score significantly correlated with the SLEDAI (r=0.64, p<0.01); as previously reported, also the type I IFN score significantly correlated with SLEDAI (r=0.67, p<0.01). To confirm the gene expression data, we evaluated the serum levels of two cytokines induced by IFNγ, CXCL9 and CXCL10, and we found that both were increased in pSLE patients compared to HD.

We, then, investigated a possible crass-talk between type I and type II IFNs: we found that IFNγ induced the expression of type I IFN-related genes in human PBMCs in a dose-dependent manner. Moreover, IFNγ upregulated the expression of both TLR7 and TLR9, two potent inducer of IFNα by pDC. Finally, human PBMCs stimulated with recombinant IFNα2b strongly up-regulated the expression of IFNγ, thus, once activated, IFNα can potentiate the production of IFNγ.

**Conclusion:** Our data suggest a potential role of IFNγ in the pathogenesis of pSLE. IFNγ-induced genes in whole blood and serum levels of IFNγ-induced chemokines are increased in patients with pSLE patients; moreover, a type II IFN score correlates with disease activity. We show that type I interferon and IFNγ establish a positive crosstalk that can potentiate their reciprocal biological activity in SLE.

**Disclosure of Interest**

None Declared

### P273 RITUXIMAB (RTX) IN PEDIATRIC DISEASES: DESCRIBING ITS PHARMACODYNAMICS WITH A FOCUS ON B-CELL DEPLETION AND REPOPULATION, INFECTIONS AND ANTI-DRUG ANTIBODIES

#### Amara Nassar-Sheikh Rashid^1^, Rosanne E. Kampinga^1^, Marjolein Peters^1^, Michiel J. S. Oosterveld^2^, Taco W Kuijpers^1^, J. Merlijn van den Berg^1^, Dieneke Schonenberg-Meinema^1^

##### ^1^Department of Pediatric Hematology, Immunology, Rheumatology and Infectious Diseases; ^2^Department of Pediatric Nephrology, Emma Children’s Hospital, Academic Medical Center, Amsterdam, Netherlands

###### **Correspondence:** Amara Nassar-Sheikh Rashid

**Introduction:** Rituximab (RTX) is increasingly used in rheumatologic [1, 2], hematologic [3] and renal diseases [4]. The induced B cell depletion can lead to hypogammaglobulinemia and thus an increased risk of infection [5]. B cell depletion is not always achieved, and this has a negative effect on therapeutic response [6]. Anaphylaxis is a frequent side effect of RTX and has been associated with the occurrence of anti-drug antibodies (ADA) against RTX [7].

**Objectives:** To describe in different pediatric patient groups the pharmacodynamics of RTX in children by outcome variables, i.e. success of B-cell depletion and time of B cell repopulation, as well as the risk factors for severe infections and anaphylaxis.

**Methods:** Patient data of children who received RTX between 2008 and 2017 at our center were retrospectively collected. Three patient subgroups were defined: autoimmune diseases (AID), immune dysregulation (ID) and renal diseases (RD). B cell repopulation was defined as a number above the cut-off value of B cell depletion (=0.050*10^6/l or <2% of the total amount of lymphocytes).

**Results:** B cell measurements were performed in 53/55 patients. B cell depletion was not achieved in 9 patients. In the 35 patients with B cell repopulation, median time until repopulation was 155 days (IQR 105-222): in the AID group (n=12) 129 days (IQR 77.5-243, p=0.363), in the ID group (n=5) 172 days (IQR 154-181, p=0.574) and in the RD group (n=18) 163 days (IQR 121-229, p=0.847). After RTX treatment, in 36 patients IgG levels were measured of which 14 (39%) had low IgG levels on at least one occasion (median 7 g/L [range 0.6 - 38.1g/L]). Severe infections leading to hospitalization occurred in 15 (27%) cases. An allergic reaction during or directly after RTX infusion was observed in 27 patients (49%). Anaphylaxis, defined as a systemic allergic reaction, characterized by impairment of airway, breathing, circulation or consciousness, occurred in 10 of these patients (18% of total cohort). Seven patients were tested for anti-RTX antibodies of whom 6 tested positive: 5 patients in the AID-group and one patient with renal disease. Allergic reactions occurred in all 6 while RTX failed to induce B cell depletion in 4 of these.

**Conclusion:** Time-to-B-cell-repopulation after RTX did not significantly differ between different pediatric patients groups. Severe infections were common (27%) in the cohorts studied. It is unclear from our data whether this is merely related to RTX treatment. Presence of ADA against RTX seems to predict failure of B-cell depletion and/or anaphylaxis after RTX treatment.

**Disclosure of Interest**

None Declared

### P274 PREDICTORS OF SECONDARY RAYNAUD’S PHENOMENON IN CHILDREN

#### Muthana Al Obaidi^1^, Charalampia Papadopoulou^2^, Sami Hussain^3^, Linda Lei^3^, Despina Eleftheriou^2,4^

##### ^1^Paediatric Rheumatology, Great Ormond Street Hospital NHS Foundation Trust; ^2^Infection, Inflammation and Rheumatology, UCL Great Ormond Street Institute of Child Health; ^3^UCL Medical School, UCL; ^4^Centre for Adolescent Rheumatology, Arthritis Research UK, London, UK

###### **Correspondence:** Charalampia Papadopoulou

**Introduction:** Raynaud’s phenomenon (RP) is characterised by abnormal vascular responses manifesting clinically with bi/triphasic colour changes of the digits. The vasospasm can be evoked by cold temperature, but other precipitating events may have a role in its pathogenesis, mostly when occurring in young patients. It can be a primary condition (PRP) or the very first presenting symptom of various systemic inflammatory conditions in which case it is referred to as secondary RP (SRP). Although extensive special testing is not always necessary, every patient with a diagnosis of Raynaud phenomenon (RP) should be carefully evaluated to distinguish the primary from the secondary disorder. Risk factors at diagnosis that predict evolution of presumed PRP into SRP remain unclear.

**Objectives:** To describe the presenting clinical features and laboratory investigations , risk factors and treatment in children with primary and secondary RP seen in a large tertiary paediatric and adolescent rheumatology centre Great Ormond Street Hospital, London and to identify baseline predictors of likely SRP.

**Methods:** Retrospective single-centre study. Data collection of demographic, clinical, laboratory and treatment characteristics of children and adolescents with RP was performed. Patients were classified as having PRP if no associated systemic inflammatory symptoms were recorded up to the last follow-up visit. Predictors examined included ethnicity, sex, age at disease onset, anti-nuclear antibody, double stranded DNA, complement factor 3 and 4, age and disease duration, full blood count and acute-phase reactants, and concomitant systemic medications. Continuous data were summarised as median and range. Comparison between groups was performed using Chi-square test for categorical data and Mann-Whitney U Test for continuous data.

**Results:** A total of 128 patients (103 females) with RP, median age 11 (range 7 – 14) years old, with a median age at disease onset 10 (range 7-13) years were included. Sixty were diagnosed with PRP and 68 with SRP. Children with SRP were more commonly of Caucasian ancestry (p=0.008) and had constant symptoms (p=0.022), arthritis (p=0.001), ulceration (p=0.029), dilated nailfold capillaries (p=0.002) and positive ANA (p=0.0004) when compared to children with PRP. Nifedipine was the commonest first line treatment (35%) followed by glyceryl trinitate patch (26%). On logistic regression analysis, Caucasian origin (p=0.002), arthritis (p=0.006), positive ANA (p=0.002), and dilated nailfold capillaries (p=0.007) were the strongest predictors associated with secondary RP.

**Conclusion:** Differentiation between primary and secondary RP in children remains challenging due to the lack of specific biomarkers. In our cohort, 53% of referred children developed a systemic inflammatory disease over time highlighting the significance of close monitoring and re-visiting the possible diagnosis of PRP. Nailfold capillaroscopy may be a useful non-invasive tool to allow in addition to other laboratory tests and clinical signs the differentiation between the two forms of RP.

**Disclosure of Interest**

None Declared

### P275 THE INTERFERON BIOMARKER SIGLEC1 REFLECTS DISEASE ACTIVITY IN PEDIATRIC SYSTEMIC LUPUS ERYTHEMATOSUS

#### Sae Lim Von Stuckrad^1^, Jens Klotsche^2^, Julia Thumfart^3^, Robert Biesen^4^, Christian Meisel^5^, Nadine Unterwalder^5^, Tilmann Kallinich^1^

##### ^1^Pediatric Rheumatology, University Hospital Charité; ^2^German Rheumatism Research Center, a Leibniz Institute, Epidemiology unit, German Rheumatism research Center; ^3^Pediatric Nephrology, University Hospital Charité, Berlin, Germany; ^4^German Rheumatism Research Center, German Rheumatism Research Center; ^5^Labor-Berlin, Immunology, University Hospital Charité, Berlin, Germany

###### **Correspondence:** Sae Lim Von Stuckrad

**Introduction:** SIGLEC1 (sialic acid-binding Ig-like lectin 1, CD169) is a monocytic adhesion molecule induced by interferon-α. In adult systemic lupus erythematosus (SLE), SIGLEC1 correlates cross-sectionally and longitudinally with disease activity_1,2_.

**Objectives:** The aim of this work was to examine whether SIGLEC1 also reflects the disease activity in pediatric SLE.

**Methods:** Over a period of 29 months, from October 2014 to March 2017, the disease activity was clinically evaluated using SLEDAI (SLE-Disease Activity Index-2000). In 28 consecutive pediatric SLE patients (mean age 16 years, range 3 - 38 years, 86% female, 14% male), the number of SIGLEC1 molecules was measured quantified and standardized by the amount of SIGLEC1 molecules per CD14 + monocyte in peripheral blood monocytes at 158 ​​times using flow cytometry in a routine laboratory. At the same time, the level of anti-ds DNA antibody titer (ELISA) and the concentration of complement factors C3 and C4 (nephelometry) were determined. The association between SIGLEC1, C3, C4 and ds DNA-antibody with SLEDAI was estimated using a mixed linear model to model the repeated measurement of parameters within a patient. The cut-off for the change in SIGLEC1 between two consecutive visits to predict clinical improvement or worsening in SLEDAI was chosen on the maximum Youden Index_3_.

**Results:**

The density of SIGLEC1 molecules on the surface of the monocytes based on two visits, correlated with the SLEDAI (129 determinations, beta_ST_ 0.24, p <0.006), but not with the C3 (110 determinations, betaST -0.11, p = 0,31), the C4 (108 determinations, beta_ST_ -0.04, p <0.67) and the anti-dsDNA-antibodies (107 determinations, beta_ST_ 0.13, p = 0.19). SIGLEC1 is a more change-sensitive biomarker than the conventional laboratory parameters C3, C4 and ds-DNA-AB (Tbl. 1). Patients with an increase in SIGLEC1 of > 1063 molecules / monocyte between two visits show a higher probability (OR = 9.4, p <0.001) of clinical worsening (SLEDAI-2k ≥ 2 points_4_). Patients who show a decrease > 2902 SIGLEC1 molecules / monocyte have a higher chance (OR = 6.8, p<0.001) to have a clinical improvement (SLEDAI ≤ 2 points).

**Conclusion:**

1. SIGLEC1 is significantly increased in active pSLE

2. There is a significant relationship between the measured interferon biomarker SIGLEC1 and the disease activity in pSLE patients

3. A cut off in SIGLEC1 can be defined which correlates with clinical worsening

**Thus, SIGLEC1 represents a potential marker for activity monitoring in pSLE disease.**

**Disclosure of Interest**

None Declared

**Table 1 (abstract P275).** Correlation of change of biomarker from previous to current visit (d visitt-1 and visitt) with the clinical course (SLEDAI-2k)

	SIGLEC1	C3	C4	ds-DNA-ab
SLEDAI-2k	n = 129	n = 110	n = 108	n = 107
	beta_ST_ = 0,24	beta_ST_ = -0,11	beta_ST_ = -0,04	beta_ST_ = 0,13
	**p = 0,006**	p = 0,31	p = 0,67	p = 0,20

betaST: standardized beta-coefficient; n: number of analyzed visits; ds-DNA ab: double-stranded DNA antibodies

## Scleroderma

### P276 LIPID DISORDERS AND THYROID FUNCTION IN CHILDREN WITH JUVENILE SCLERODERMA

#### Iryna Chyzheuskaya, Lyudmila Byelyaeva, Tamara Yuraga

##### Pediatrics, BELARUSIAN MEDICAL ACADEMY OF POSTGRADUATE EDUCATION, Minsk, Belarus

###### **Correspondence:** Iryna Chyzheuskaya

**Introduction:** Subclinically passing endocrine pathology plays an important role in the development and progression of the atherosclerotic process and is often underestimated not only by rheumatologists, but also by endocrinologists. The mechanism of development of autoimmune thyroid pathology, as well as systemic diseases of connective tissue, is closely related to the imbalance of the immune system, mainly with the Th1 immune response. Under the influence of pro-inflammatory cytokines, the expression of receptors activating the apoptosis of follicular cells of the thyroid gland occurs, which ultimately leads to the development and progression of hypothyroidism. Deficiency of thyroid hormones can promote the appearance of hypercholesterolemia, cause disturbances in the state of the autonomic nervous system and energy metabolism.

**Objectives:** The aim of the study was to evaluate the state of lipid metabolism and thyroid function in children with juvenile scleroderma

**Methods:** 40 children with juvenile scleroderma at the age from 5 to 16 years were examined. The parameters of the immune system, lipid spectrum of blood and thyroid hormone levels were studied.

**Results:** According to the results of immunological examination, a significant increase in the relative amount of B-lymphocytes, a redistribution of immunoregulatory subpopulations of T-lymphocytes by decreasing the number of CD8 + cells against a background of increased number of CD4 + cells, hyperproduction of total IgG and IgM, a significant increase in TNF-α and a decrease in serum IFN-γ was established in children with juvenile scleroderma when compared with the control group (P <0.01).

As a result of the thyroid function study, a significant (p <0.05) decrease in the levels of total and free T3 and T4 in the blood serum of children with JS was compared with that in the children of the control group: the mean levels of total T3 in children with JS were 1,4 (0,8, 1,9) nmol / l, in children of the control group - 2.1 (1.5, 2.4) nmol / l; the average values of free T3 in children with JS 3.4 (3.1, 4.1) nmol / L, in children of the control group - 5.1 (2.7, 5.8) nmol / l; the mean levels of total T4 in children with with JS were 84.4 (73.8, 99.2) nmol / l, in children of the control group - 102.1 (93.1, 108.6) nmol / l; mean free T4 in children with JS were 11.8 (11.2, 12.8) nmol / l, in children of the control group - 14.6 (12.6, 18.9) nmol / l. The level of TSH in children with JS was significantly higher than in children of the control group - 3.1 (2.6, 4.6) mIU / l in children with JS, 1.9 (1.5, 5.4) mIU / L in children of the control group.

The children with JS have violations in the lipid spectrum of atherogenic orientation, characterized by hyperlipidemia> 7.0 g / l (62.5%), hypertriglyceridemia> 1.1 mmol / l (22.5% of children), hypercholesterolemia (32.5% of children ) and dyslipoproteinemia in the form of a decrease in HDL cholesterol and an increase in LDL, an increase in the ApoV / ApoA ratio> 1 (17.5%) and a decrease in ApoE (p <0.001), which together with hemostasis changes can be regarded as a risk factor for atherothrombosis.

**Conclusion:** The atherogenic orientation of lipid metabolism caused by the autoimmune process and hypothyroidism of the thyroid gland is characteristic for children with juvenile scleroderma.

**Disclosure of Interest**

None Declared

### P277 EFFECTS OF SENSE AND FUNCTIONALITY CHANGES IN THE HANDS ON ACTIVITY AND PARTICIPATION IN PATIENTS WITH JUVENILE SCLERODERMA

#### Arzu Dag^1^, Ela Tarakci^2^, Amra Adrovic^3^, Ozgur Kasapcopur^3^

##### ^1^Department of Physiotherapy and Rehabilitation, Faculty of Health Science, Istanbul Yeni Yuzyil University; ^2^Department of Physiotherapy and Rehabilitation, Faculty of Health Science, Istanbul University; ^3^Department of Pediatric Rheumatology, Istanbul University, Cerrahpasa Medical School, Istanbul, Turkey

###### **Correspondence:** Arzu Dag

**Introduction:** Juvenile scleroderma is a rarely seen chronic connective tissue disorder.The hands are commonly affected in patients with JS, and Raynaud’s phenomenon, finger swelling and joint pain are the primary manifestations of the disease. Skin thickening is a manifest consequence of JS. The hands account for only a small fraction of the total skin area of the body, but are necessary for many important functions in daily life.

**Objectives:** The purpose of our study is to examine the effects of sense and functionality changes in the hands on activity and participation in patients with JS.

**Methods:** 30 patients (26 girls, 4 boys) with JS between the ages of 6-18 and 30 healthy controls (20 girls, 10 boys) with similar age and gender were included in our study. 14 of 30 JS patients were JSS and the rest of them were JLS.The inclusion criterias were to have been diagnosed with Juvenile Scleroderma 6 months prior to the ILAR classification and to be at the mental level that could respond to the assessments to be used in the study. The follow-up form was prepared considering the relevant parameters such as gender, socio-demographic status, clinical family story of JS disease. Children rated their pain severity on a six-item Wong-Baker FACES® Pain Rating Scale (WBS) from none to worst. Hot-cold, pain, and touch sense were evaluated in both groups. Vibration sence is assessed with 256Hz diapozon, light touch-deep pressure sensation is with Semmes Weinstein monofilament (SWMT) test, static-dynamic 2 point 2-point discrimination test is with a discriminator, localization sensation test is with a pencil. In addition moberg pickup test, stereognosis evaluation, wrist joint position sense, sympathetic activation were evaluated. The hand joint range of motion was measured by goniometer , hand grip strength by "Jamar Plus + Hand Dynomometer (JPHD)", the pinch gripping force by "Jamar® Pinch Gauge - Hydraulic" pinchmeter, the hand mobility by modified Hand Mobility in Scleroderma (mHAMIS). Children completed their activtiy and participant performance status with “Childhood Health Assessment Questionnaire (CHAQ)”, “Duruoz Hand Index (DHI)”, “Jebson Taylor Hand Function Test(JTHFT)”, “Community Integration Questionnaire (CIQ)” tests. The quality of life were evaluated with “Scleroderma Health Assessment Questionnaire(SHAQ)”. Statistics were analyzed with the SPSS for Windows 21.0 program.

**Results:** The mean age of the JS group was 14.06±3.24, while the mean age of the control group was 12.43±3.24 years. There were significantly differences between the two groups in pain (WBS), light touch-deep pressure sensation, sense of touch localization, range of motion, functional assessment (JTHFT), activity and participation scores (CHAQ, DHI) and quality of life scores (SHAQ)(p<0,05). Almost half of the JS group describes the character of their pain as inflammable or whining.There was a significant correlation between SWMF and CHAQ (r=0,52), SWMF and DEI (r=0,62), SWMF and SHAQ-Total (r=0,57) scores. There was a significant correlation between resting pain and CHAQ-Total (r=0,576) and Resting Pain and DEI (r=0,674) scores.

**Conclusion:** Consequently, sense and functional changes after hand involvement in JS patients cause limitations in daily living activity and negatively affect the effective use of the hands.Assessments of sensation symptoms that affect the functionality, activity level and participation should be taken into consideration in patients with JS.In addition, we think that the use of sensory therapies in JS treatment will be an important factor in increasing the effectiveness of rehabilitation.

**Disclosure of Interest**

None Declared

### P278 AFTER 24 MONTHS OBSERVATION PERIOD THE PATIENTS RELATED OUTCOMES IMPROVE SIGNIFICANTLY IN THE JUVENILE SCLERODERMA INCEPTIONS COHORTE. WWW.JUVENILE-SCLERODERMA.COM

#### Ivan Foeldvari^1^, Jens Klotsche^2^, Ozgur Kasapcopur^3^, Maria Teresa Terreri^3^, Tadey Avcin^3^, Rolando Cimaz^3^, Mikhail Kostik^3^, Maria Katsikas^3^, Dana Nemcova^3^, Christina Battagliotti^3^, Lillemor Berntson^3^, Jürgen Brunner^3^, Liora Harel^3^, Tilmann Kallinich^3^, Kirsten Minden^3^, Monika Moll^3^, Maria Jose Santos^3^, Kathryn Torok^3^, Nicola Helmus^1^

##### ^1^Hamburg Center for Pediatric and Adolescent Rheumatology, Am Schoen Klinik Eilbek, Hamburg; ^2^German Rheumatism Research Center, Berlin; ^3^jSSc Collaborative Group, Hamburg, Germany

###### **Correspondence:** Ivan Foeldvari

**Introduction:** Juvenile systemic scleroderma (jSSc) is an orphan disease with a prevalence 3 in a 1 000 000 children(1).  There is not much published data about the course of jSSc with standardized assessment of the patients. We report our date form juvenile scleroderma inception cohorte with a follow up of 24 months in the cohort.

**Objectives:** to evaluate the development of the disease during the first 24 months in the cohorte

**Methods:** The juvenile scleroderma inception cohorte is a prospective multicenter registry of patients with jSSc, who fullfill the adult classification criteria, and have age of disease onset of less then 16 years of age and less then 18 years at the time of inclusion in the cohort. We evaluted the patients characteristics of the patients, who were followed 12 months in the registry.

**Results:** 40 patients were followed 24 months in the registry. 80% were female and 77.5% had diffuse subtype. 20% had overlap feutures. Mean disease duration at time of inclusion was 3.2 years. Mean age of onset of Raynauds was 8.2 years and the first non-Raynauds 8.7 years. 73% received DMARDs at the time of inclusion and 95% after 24 months. 85% of the patients were ANA positive, around 25% anti-Scl70 positive and around 3% anticentrome positive.  The mean modified skin score decreased from 14.4 to 12.9. The frequency of Raynaud´s stayed around 87.5%. The frequency of the nailfold capillary changes increased from 55% to 62%, but the frequency of active ulcerations decreased from 23% to 20%. The number of patients with FVC <80 % decreased from 42% to 39% (p=0.8). The number of patients with DLCO< 80% decreased from 54 to 44% (p=0.58). The number of patients with pulmonary hypertension assessed by ultrasound increased from 5% to 10% ( p=0.396). No patient developed hypertension or renal crisis. Gastrointestinal involvement decreased from 32.5% to 22.5% (p=0.317). Number of swollen joints decreased from 23% to 17.5% (p=0.53). Total muscle weakness decreased from 5 % to 0% (p=0.224) amd elevated CK from 17% to 11% (p=0.54) too. Several patient related outcomes improved significantly. Patient global disease activity (VAS 0-100) from 49.2 to 29.4(p=0.001) ,patient global disease damage (VAS 0-100) from 43.9 to 29.8 (p=0.013) and patient Raynaud activity VAS 0-100) from 26.7 to 14.2 (p=0.045)as physitian global disease activity (VAS 0-100) from 48.3 to 33.2 (p=0.021) .

**Conclusion:** Over the 24 months observation period patient related outcomes improved significantly and fortunately the other clinical parameteres did not revealed any significant deterioration. It seems, that the current treatment can reach improvement judged by the patients and stablisation of the disease.

1. Beukelman T, Xie F, Foeldvari I. Assessing the Prevalence of Juvenile Systemic Sclerosis in Childhood Using Administrative Claims Data from the United States. Journal fo Scleroderma and Related Disorders. 2018;3.

**Disclosure of Interest**

None Declared

### P279 DO RAYNAUD PHENOMENON NEGATIVE JUVENILE SYSTEMIC SCLERODERMA PATIENTS HAVE A DIFFERENT PATTERN OF ORGAN INVOLVEMENT AS RAYNAUD PHENOMENON POSITIVE PATIENTS?

#### Ivan Foeldvari^1^, Jens Klotsche^2^, Ozgur Kasapcopur^3^, Amra Adrovic^3^, Kathryn Torok^3^, Valda Stanevicha^3^, Maria Teresa Terreri^3^, Ekaterina Alexeeva^3^, Maria Katsikas^3^, Vanessa Smith^3^, Flavio Sztajnbok^3^, Jordi Anton^3^, Tadey Avcin^3^, Rolando Cimaz^3^, Mikhail Kostik^3^, Thomas Lehman^3^, W.-Alberto Sifuentes-Giraldo^3^, Simone Appenzeller^3^, Mahesh Janarthanan^3^, Monika Moll^3^, Dana Nemcova^3^, Maria Jose Santos^3^, Christina Battagliotti^3^, Lillemor Berntson^3^, Jürgen Brunner^3^, Patricia Costa Reis^3^, Despina Eleftheriou^3^, Liora Harel^3^, Tilmann Kallinich^3^, Kirsten Minden^2^, Susan Nielsen^3^, Yosef Uziel^3^, Nicola Helmus^1^

##### ^1^Hamburg Center for Pediatric and Adolescent Rheumatology, Am Schoen Klinik Eilbek, Hamburg; ^2^German Rheumatism Research Center, Berlin; ^3^jSSc Collaborative Group, Hamburg, Germany

###### **Correspondence:** Ivan Foeldvari

**Introduction:** Juvenile systemic scleroderma (jSSc) is an orphan disease, with an estimated prevalence of 3 per 1000 000 children. Most jSSc patients primarily present with Raynaud phenomenon (RP).

**Objectives:** We investigated in our patient of the juvenile scleroderma inception cohort, how fare patients with (RP+) and without (RP-) RP differed in their clinical presentation at enrolment.

**Methods:** The jSSc is a prospective cohort of jSSc patients. Patients were enrolled who were diagnosed with jSSc, had a jSSc onset age under 16 years and were younger as age of 18 years at the time of inclusion. The patients are prospectively assessed every 6 months according to a standardized protocol. We reviewed the organ involvement pattern of our patients currently followed in the cohort.

**Results:** 100 patients are currently followed in the cohort and 89 (89%) of them had RP. The female/male ratio was lower in the RP+ group, 3.7:1 compared to 4.5:1(p=0.808). Diffuse subtype was more common in the RP+ group, 72% compared to 63%. Mean age of onset of first non- Raynaud symptomatic was 10.4 years in both groups. Mean disease duration was slightly higher in the RP+ group, 3.4 compared to 2.2 years. ANA positivity was higher in the RP+ group, 88% compared to 70% (p=0.48). Anti-Scl70 was 34% in the RP+ and 20% in the RP-group (p=0.34). Interestingly 7% of RP + but none of the RP+ were anti-centromere positive. The mean modified skin score was lower in RP + group (mean of 14.8 compared to 17.0). There were significantly more nailfold capillary changes (70% compared to 18%, p=0.001) and a higher rate of history of ulceration in the RP+ group (49% compared to 20%, p=0.083). Decreased DLCO and FVC<80% was higher in the RP-negative group with 45%/50% compared to 37.5%/31% respectively. Pulmonary hypertension occurred in 7% in the RP+ group and there was no case in the RP- group (p=0.335). RP- group had a higher rate of urinary sediment changes 18% compared to 4.5% in the RP+ group (p=0.07). No renal crisis or hypertension was reported in neither groups. Gastrointestinal involvement was similar between the two groups with around 35%. Occurrence of swollen joints was similar in both groups as the frequency of muscle weakness with around 20%. The tendon friction rub occurred around 10% in both groups. In the patient related outcomes, there was only a difference in rating of Raynauds activity.

**Conclusion:** The RP– group differed from RP + group in the clinical presentation at enrolment. The absence of Raynaud phenomenon was associated with a decreased rate of history of ulceration, no occurrence of pulmonary hypertension. Interestingly higher rate of urinary sedimentary changes and no anticentromere positivity was observed in RP- patients.

**Disclosure of Interest**

None Declared

### P280 EVALUATING THE VALIDITY OF SIX-MINUTE WALK TEST IN JUVENILE SYSTEMIC SCLEROSIS

#### Oya Koker^1^, Amra Adrovic^2^, Sezgin Sahin^2^, Kenan Barut^2^, Rukiye Eker^1^, Ozgur Kasapcopur^2^

##### ^1^Department of Pediatric Rheumatology, Istanbul University, Istanbul Medical Faculty; ^2^Department of Pediatric Rheumatology, Istanbul University, Cerrahpasa Medical School, Istanbul, Turkey

###### **Correspondence:** Oya Koker

**Introduction:** Pulmonary vascular disease and interstitial lung fibrosis are the leading causes of morbidity and mortality in Juvenile Systemic Sclerosis (JSSc). Six-minute walk test (6MWT) is a self-paced submaximal exercise test used for evaluating functional exercise capacity, prognosis and response to therapy in patients with cardiopulmonary diseases. While the results of studies on the availability of the test in adults are contradictory, there are limited data concerning the usefulness of 6MWT in children with JSSc.

**Objectives:** To evaluate the walking distance and oxygen desaturation during the 6MWT in JSSc, and to establish correlations between the 6MWT results and other clinical findings in children with JSSc.

**Methods:** 25 JSSc, 27 Juvenile Systemic Lupus Erythematosus (JSLE) and 30 healthy controls were included. The test is conducted according to the guidelines recommended by the American Thoracic Society (ATS), standardized in 2002. The Borg Scale which is a well-validated scoring system on a 0-10 point scale was used to determine the patient self reported fatigue and dyspnea levels.

**Results:** A total of 25 (22 female/3 male) JSSc , 27 (21 female/6 male) Juvenile Systemic Lupus Erythematosus (JSLE) and 30 (16 female/14 male) healthy controls were included. The mean age of patients was 16,44±3,19 years for JSSc, 16,74±3,59 years for JSLE and 15,57±1,50 years for healthy controls. There was no significant difference between patients and healthy controls according to the age at the study.

Mean walking distance was 480,18 ± 47,22 m in JSSc; 513,66 ± 51,70 m in JSLE and 553,21 ± 41,65 m in healthy controls. JSSc patients walked significantly less distance comparing to controls (p <0.001). The mean oxygen saturation in JSSc was 98.0 ± 0.9% before the test and 97.5 ± 1.6% after the test (p = 0,01). JSSc patients with lung involvement walked less than those without lung involvement (476.08 ± 47.13 m vs .483.96 ± 48.89 m). However, there was no statistically significant difference (p = 0.68). JSSc patients with carbon monoxide diffusion capacity (DLco) ≤ 60% walked less than those with DLco ≥ 60% (466.92 ± 45.74 m vs. 485,33 ± 48,05 m). However, no statistically significant correlation was detected. (p = 0.39). No significant difference was found when patients walking distances were compared to activity scores (Juvenile Systemic Sclerosis Severity Score(J4S)) (p = 0.26). Lower extremity pain during and after the test was more statistically significant in JSSc patients (p = 0.001). Patients with myalgia were found to walk less than those without myalgia (498.61 ± 46.94 m vs. 460.20 ± 40.29 m) (p = 0.038).

**Conclusion:** The findings of our study showed that patients with JSSc have limited walking distances.The  pulmonary involvement and associatively high disease activity score are unable to determine the results of the 6MWT in JSSc patients. However, the musculoskeletal involvement may influence the walk distance and complicate interpretation of the 6MWT in JSSc patients. Since there is a limited number of studies regarding the role of 6MWT in the evaluation of JSSc, we believe that the results of our study will enlighten the future studies.

**Disclosure of Interest**

None Declared

### P281 LONG-TERM PERSISTENCE OF RUBELLA AND MEASLES ANTIBODIES IN CHILDREN WITH JUVENILE SCLERODERMA ON IMMUNOSUPPRESSIVE TREATMENT- A CASE CONTROL STUDY.

#### Despoina Maritsi, Ekaterini Markante, Alexandros Panos, Olga Vougiouka, Maria Tsolia

##### Second Department of Paediatrics, Medical School, National and Kapodistrian University of Athens, Athens, Greece

###### **Correspondence:** Despoina Maritsi

**Introduction:** Juvenile localized scleroderma (jLScle), a limited autoimmune disease, theoretically renders patients susceptible to infections due to their defective immune system and the immunosuppressive treatment. However we lack data regarding response and long-term immunity conveyed by specific vaccines.At present, we are experiencing major measles’ outbreaks throughout Europe.Measles infection harbors potentially serious consequences. Rubella infection, on the other hand, may harbor significant long-term sequences for the mother and the fetus. Thus it is critical that we understand the impact of jLScle on young patients’ immunity to measles and rubella.

**Objectives:** In this study we determined the immune status against measles and rubella in previously vaccinated jScle patients, prior to commencement of treatment and at one and three years, and compared this to healthy controls.

**Methods:** This was a prospective controlled study including 28 newly diagnosed jLScle patients and 56 healthy controls. All participants had two doses of the live attenuated MMR vaccine in early childhood. Demographic, clinical and laboratory data were collected. Type and duration of treatment was also recorded. Seroprotection rates and rubella-measles-IgG titers were measured at enrolment (prior to commencement of treatment) and at 1 and 3 years. Total IgG levels were measured simultaneously. Measles and rubella specific IgG antibodies were assessed by ELISA and were expressed as GMC’s. The cut-off value for seroprotection was deemed at 10IU/ml and 120IU/ml for measles and rubella respectively. The Hospital’s Research and Ethics’ Committee approved the study; written informed consent was obtained. Statistical significance was set at p<0.05 and analyses were conducted using STATA 13.0.

**Results:** The two groups had similar demographic characteristics, vaccination history and immunization status. No significant differences were detected in terms of vaccine type, time interval between the two vaccines as well as mean elapsed time from last vaccination to blood sampling. Seroprotection rates were adequate for both groups. Nonetheless, the jLScle group had marginally lower seroprotection rates at one and three years (both measles and rubella). Measles-specific-GMC’s were similar between the patient and control group at all time points. Rubella-specific-GMC’s were significantly lower in the jLScle compared to the control group (p<0.01) at one and three years’ follow up but not at diagnosis. None of the participants had hypogammaglobulinaemia at the time of blood sampling The use of methotrexate did not seem to exceed any significant effect on seroprotection rates but had a negative impact on rubella-GMC’s. The use and duration of systemic steroids as well as topical treatments did not have any effect on rubella or measles-specific-GMC’s.

**Conclusion:** In conclusion, although seroprotection rates were similar between the two groups, rubella GMC’s were significantly lower in the jSLcle group. Further studies are required to address the question of long-term immunity conveyed by immunizations given at an early stage in children with rheumatic diseases.

**Disclosure of Interest**

None Declared

### P282 DIFFERENT TYPE 1 INTERFERON RESPONSE IN TWO PATIENTS WITH PAEDIATRIC ONSET SYSTEMIC SCLEROSIS

#### Sonia Melo Gomes^1^, Giulia Varnier^2^, Ebun Omoyinmi^1^, Sandrine Lacassagne^2^, Nigel Klein^1^, Paul Brogan^1^

##### ^1^Rheumatology, Institute of Child Health; ^2^Rheumatology, Great Ormond Street Hospital, London, UK

###### **Correspondence:** Sonia Melo Gomes

**Introduction:** Systemic sclerosis is an auto-immune disease characterized by small-vessel vasculopathy, dysregulation of innate and adaptive immunity, and extensive fibrosis of the skin and visceral organs which can lead to loss of organ function. However its pathogenesis is not completely understood. In the past few years research in adult patients has focused on the role of type I interferon in the pathogenesis of the disease, with variants in several interferon related genes being identified as a genetic risk factor.

**Objectives:** The aim of this study was to characterize the type I interferon response in paediatric patients with systemic sclerosis as well as to assess the potential contribution of genetic variants in *IRF7* to this response.

**Methods:** DNA was extracted from saliva using Oragene saliva collection pods and DNA extraction protocol. Exons 1 to 9 of the *IRF7* gene were sequenced by Sanger using an AB1 sequencer. Peripheral blood was collected to PAXgene tubes for RNA stabilization and extraction. RNA was transformed into c-DNA using High Capacity c-DNA Reverse Transcriptase kit. Changes in gene expression were assessed by real-time polymerase chain reaction. The interferon score was calculated by assessing the relative expression of six interferon related genes (*SIGLEC1, RSAD2, ISG15, IFI44L, IFI27, IFIT1*). Patients’ demographics were collected from medical records.

**Results:** Two patients with paediatric-onset systemic sclerosis were recruited into this study. Both patients were female and black British. Patient 1 is 16 years old and has long standing disease with multiple organ involvement diagnosed at the age of 9 years. Patient 2 is 5 years old and has early stage disease (less than 6 months since diagnosis).

A high interferon score was detected in Patient 1, with long standing disease, but not in Patient 2. Patient 1 also had a modest increase in expression of *IRF7* (2.3 fold), but not in the other interferon related genes tested.

Sanger sequencing of the *IRF7* gene revealed the presence of the same variant, p.K192E, in both patients. Although frequent in the general population, this variant has been linked in GWAS studies to increased risk of Systemic Lupus Erythematosus (SLE) and Rheumatoid Arthritis.

**Conclusion:** Our results show an increased type I interferon response in a patient with long standing disease. A high interferon score has been shown both in long standing disease and early stage disease in adult patients. However, our patient with early stage disease did not have increased expression of any of the interferon related genes tested. Interestingly, both patients presented the same variant in *IRF7*, which has been linked with disease risk for SLE. Variants in other interferon related genes could eventually have additional relevance to the disease phenotype. Larger studies in paediatric patients with systemic sclerosis are needed to determine the relative importance of type I interferon response in the pathogenesis and progression of this disease. Informed consent to publish had been obtianed.

**Disclosure of Interest**

None Declared

### P283 PROSPECTIVE VALIDATION OF CONE BEAM COMPUTED TOMOGRAPHY FOR THE ASSESSMENT OF DISEASE PROGRESSION IN LINEAR SCLERODERMA OF THE FACE IN COMPARISON WITH THE CURRENTLY USED TECHNIQUES

#### Alessandra Meneghel^1^, Stefano Puggina^2^, Eleni Kamburi^1^, Giorgia Martini^1^, Fabio Vittadello^1^, Francesco Zulian^1^

##### ^1^Department of Woman and Child Healt, University Hospital of Padua, PEDIATRIC RHEUMATOLOGY UNIT, Padova; ^2^Iniziativa Medica, Monselice (PD), Italy

###### **Correspondence:** Alessandra Meneghel

**Introduction:** Currently, the techniques used for the monitoring of Localized Scleroderma of the face (LSF) have significant limitations.

**Objectives:** We prospectively evaluated the reliability of the Cone Beam Computed Tomography (CBCT), a non-invasive, reproducible technique with a radiation dose lower than a conventional CT, to quantify changes of facial asymmetry over time as compared with the clinical and instrumental methods currently in use.

**Methods:** Consecutive patients with LSF, followed from January 2009 to December 2017 at our Pediatric Rheumatology Center, entered the study. CBCT was carried out following the same method previously described^1^. Measurements of total thickness, soft tissue and bone thickness were taken in both affected and unaffected sides of the case. These data ​​allowed us to calculate the Absolute Rate of Change (ARC) of the lesion over time and the comparison with the ARC obtained from the healthy side, allowed us to calculate the Relative Rate of Change (RRC). The judgment of stability or worsening of the lesion was compared with the one derived from the physician global assessment (PGA), infrared teletermography (IT) and sequential clinical photographs (SCP). The Sensitivity-to-change of CBCT was assessed by Standardized Response Mean (SRM) and Effect Size (ES)

**Results:** 26 subjects with LSF, 15 females and 11 males, mean age 7.6 years (range 1.2-17.8) entered the study. 18 patients presented Parry Romberg syndrome (PRS), 5 En coup de sabre (ECDS) and 3 facial hemiatrophy (FH). The disease duration at the first CBCT was 3.7 years (range 0-28). During the study period, 69 CBCTs have been performed. On average, each patient underwent 2.7 CBCTs, 15 patients underwent two CBCTs, 8 patients three, and one patient 4, 5 or 6 CBCTs each, respectively. In all, the total thickness of the affected and unaffected side were evaluated. A worsening of CBCT with RRC > 5%, was found in 10 out of 40 evaluations (25%). The agreement between the CBCT and SCP was found in 20 out of 37 evaluations (54%). The comparison of the four clinical-instrumental methods performed in 40 evaluations, revealed an overall agreement of 66.7%. CBCT results were consistent with the PGA in 67.6% evaluations, with IT in 62.2%. The sensitivity to change of CBCT over time was very good at the mandibular condyle level (SRM values ranging from 0.53 to 0.77, ES 0.40-0.50) on the healthy side. The affected side reported SMR and ES values <0.5.

**Conclusion:** CBCT is an innovative technique that allows to assess the changes of the affected side, usually the *non-growth*, of LSF in comparison with the healthy side, during the developmental phase of pediatric patients. CBCT represents a reliable and objective tool for monitoring LSF over time in association with the other clinical-instrumental methods currently in use. Its practical benefit lies in its potential of correctly addressing therapeutic changes including the timing of start the reconstructive surgical process.

**Disclosure of Interest**

None Declared

### P284 NAILFOLD CAPILLAROSCOPY, COMPUTERIZED COLOR TELETHERMOGRAPHY AND IMMUNOLOGICAL FINDINGS IN 224 CHILDREN WITH RAYNAUD'S PHENOMENON

#### Mario Sestan^1^, Nastasia Cekada^1^, Daniel Turudic^1^, Mateja Batnozic Varga^2^, Marijan Frkovic^1^, Jagoda Stipic^3^, Marko Baresic^4^, Domagoj Kifer^5^, Marija Jelusic^1^

##### ^1^Department of Paediatrics, University Hospital Centre Zagreb, University of Zagreb School of Medicine, Zagreb; ^2^Department of Paediatrics, University Hospital Centre Osijek, Osijek; ^3^Department of Neurology; ^4^Department of Internal Medicine, Division of Clinical Immunology and Rheumatology, University Hospital Centre Zagreb, University of Zagreb School of Medicine; ^5^Department of Biophysics, Faculty of Pharmacy and Biochemistry, University of Zagreb, Zagreb, Croatia

###### **Correspondence:** Mario Sestan

**Introduction:** Raynaud's phenomenon (RP) is a condition characterized by periodical vasospasm in response to cold temperatures or emotional stress exposure, that in the vast majority of patients exists without other disorders, but sometimes is associated with several autoimmune rheumatic diseases and, to distinguish between these two types, clinical examination, laboratory findings, nailfold capillaroscopy and computerized color telethermography (CCTT) are necessary.

**Objectives:** The aim of this research was to analyze RP features in children in correlation with the most frequently associated laboratory tests, CCTT and nailfold capillaroscopy.

**Methods:** This retrospective study included children who were clinically recognized as RP in the period from 2011 to 2017 at University Hospital Centre Zagreb. Laboratory data included serum level of IgG, C3, C4, CH50, RF, presence of ANA and ANCAs. Nailfold capillaroscopy and CCTT were utilized to distinguish between primary and secondary RP. McNemar's test was used to evaluate whether capillaroscopy or CCTT was more effective in the diagnosis of secondary RP and to estimate was there a difference between capillaroscopy and CCTT in matching with the results of immunological laboratory findings.

**Results:** Out of 224 children with RP, 169 were females (75.4%) and 55 were males (24.6%) with female/male ratio 3:1. CCTT was performed in 188 patients (83.9%), nailfold capillaroscopy in 89 patients (39.7%) and both investigations in 80 patients (35.7%). CCTT classificated 15 patients as primary RP, 57 patients as secondary RP (among them, 33 had at least one pathological laboratory finding), while in 47 patients no classification could be made. In 69 patients clinically recognized as RP, CCTT findings were normal. Among patients classificated as secondary RP on CCTT, the most of them, 14 (24.6%), were diagnosed with juvenile idiopathic arthritis (JIA). There were 5 patients (8.8%) with systemic sclerosis (SSc), 2 (3.5%) with mixed connective tissue disease (MCTD), 1 (1.7%) with systemic lupus erythematosus, 11 (19.3%) with undifferentiated connective tissue disease (UCTD), whilst 24 patients (42.1%) had no evident other disease. The appearance of abnormal capillaroscopic pattern was found in 17 patients (among them, 11 had at least one pathological laboratory finding) and nonspecific capillaroscopic alterations were noticed in 27 patients (among them, 16 had at least one pathological laboratory finding), while 45 patients had normal capillaroscopic findings. Among patients with the appearance of abnormal capillaroscopic pattern, 5 (29.4%) were diagnosed with SSc, 3 (17.6%) with JIA, 2 (11.8%) with MCTD, 1 (5.9%) with dermatomyositis, 2 (11.8%) with UCTD, whilst 4 patients (23.5%) had no evident rheumatic disease. All patients with RP diagnosed with SSc and MCTD had both the appearance of abnormal capillaroscopic pattern and CCTT findings consistent with secondary RP. No statistically significant difference between capillaroscopy and CCTT in predicting the diagnosis of secondary RP was determined (McNemar's test, χ^2^ = 0.042, p = 0.838) nor was there significant difference between capillaroscopy and CCTT in regard to the results of laboratory findings (χ^2^ = 1.042, p = 0.307).

**Conclusion:** Both nailfold capillaroscopy and CCTT have important role in the diagnosis of RP in children. We found that both methods were equally effective in the diagnosis of secondary RP and that there was no difference between them in regard to the results of immunological laboratory findings distinctive with secondary RP.

**Disclosure of Interest**

None Declared

### P285 CLINICAL MANIFESTATIONS AND TREATMENT OF JUVENILE LOCALIZED SCLERADERMA

#### Fatemeh Tahghighi, vahid ziaee, seedreza raeeskarami

##### Department of Pediatric Rheumatology, Children's Medical Center .Tehran University of Medical Sciences, tehran, Iran, Islamic Republic Of

###### **Correspondence:** Fatemeh Tahghighi

**Introduction:** Juvenile localized scleroderma (JLS) is a rare condition in childhood. we studied the clinical features of JLS in children who refered to Children's Medical Center of Tehran University of Medical Sciences between 2005 and 2017.

**Objectives:** Juvenile localized scleroderma (JLS) is a rare condition in childhood. we studied the clinical features of JLS in children who refered to Children's Medical Center of Tehran University of Medical Sciences between 2005 and 2017.

**Methods:** The study was conducted by collecting information on the demographics, family history, clinical and laboratory features, and treatment of patients with Juvenile localized scleroderma.

**Results:** A total of 41 patients , 28 (68.29%) were female and13 (31.70%) male. The female to male ratio was 2.15:1. The mean age of onset was 9.5 years(range 2-16). The mean duration of disease from diagnosis was 1.5 years . The most common of constitutional sign were fatigue 36(87.80%)and weight loss 21(51.21%) .And other signs were : articular 22(53.65%), raynaud ҆s phenomen 11(26.82%) , vascular 12(29.26%), gastrointestinal 3 (7.31%), Uveitis 2(4.87%).None of patients have symptoms of involved cardiac,pulmonary and renal . Six patients (14.63%) had a positive family history for rheumatic or autoimmune diseases. ANA was positive in 56.09% of the patients. Scl-70 was detected in 7.31% of the patients, anticentromere antibody in 2.43% ,anti-double-stranded DNA in 7.31%, anti-cardiolipin antibody in 14.63% and rheumatoid factor in 19.51%.Oral prednisolone and Methotrexate were the drugs most frequently used.

**Conclusion:** Treatment with oral corticosteroid and methotrexate led to disease improvement in the most patients and the prognosis of these patients was good in our study.

**Disclosure of Interest**

None Declared

## Systemic lupus erythematosus and antiphospholipid syndrome

### P286 CARDIO-FACIO-CUTANEOUS SYNDROME WITH GERMLINE B-RAF MUTATION COMPLICATED WITH REFRACTORY SYSTEMIC LUPUS ERYTHEMATOSUS

#### Utako Kaneko

##### Pediatrics, Niigata University, Niigata, Japan

**Introduction:** RASopathies are neurodevelopmental syndrome caused by heterozygosity for germline mutations in genes that encode protein components of RAS/MAPK pathway. There are several reports of RASopathies complicated with autoimmune disease, such as Noonan syndrome with germline mutation of *PTPN11* or *SHOC2* associated with systemic lupus erythematosus (SLE) ^[1]^. Recently, it is revealed that somatic mutations of *KRAS* and *NRAS* cause autoimmune lymphoproliferative syndrome (ALPS)-like disease, which called RAS-associated-ALPS-like disease (RALD) ^[2]^. Some patients with RALD showed clinical and laboratory features of SLE.

**Objectives:** We aim to investigate the relationship between *BRAF-*associated RASopathy and development of SLE.

**Methods:** We present a Japanese case of cardio-facio-cutaneous syndrome with germline *BRAF* mutation complicated with SLE refractory to conventional treatments.

**Results:** A 5-year-old girl with cardio-facio-cutaneous syndrome with germline mutation of *BRAF* was referred to hospital due to recurrent fever and erythema. Laboratory data showed pancytopenia, hypocomplementemia, high erythrocyte sedimentation rate, positive antinuclear antibody, elevation of serum anti-dsDNA antibody, and proteinuria. She was diagnosed as having SLE. The histological findings of renal biopsy showed lupus nephritis ISN/RPS class III. She received three times of methylprednisolone pulse therapy and combination of mycophenolate mofetil and tacrolimus that showed poor response. Interestingly, peripheral blood immunophenotyping by flow cytometry showed increased TCRαβ+CD4-CD8-double negative T cell, which indicate dysfunction of T cell apoptosis such as ALPS.

**Conclusion:** This is the first report of SLE with germline mutation of *BRAF*. The prognosis and clinical features of SLE with RASopathies are various depending on the related component of RAS/MAPK pathway. This case would be more severe compared with previous reports. To investigate the difference of each gene mutation of RASopaties associated with SLE might lead to new insights of monogenic SLE.

**References**

[1] Bader-Meunier B, et al. Are RASopathies new monogenic predisposing conditions to the development of systemic lupus erythematosus? Case report and systematic review of the literature. Semin Arthritis Rheum. 2013;43:217-219.

[2] Calvo KR, et al. JMML and RALD (Ras-associated autoimmune leukoproliferative disorder): common genetic etiology yet clinically distinct entities. Blood. 2015;125:2753-2758.

Informed consent for publication of this case report was obtained from the parents of the patient.

**Trial registration identifying number:**

**Disclosure of Interest**

None Declared

### P287 PREVALENCE AND CLINICAL ASSOCIATIONS OF ANTI C1Q ANTIBODIES IN PEDIATRIC SLE

#### Sathish Kumar, George I. Vettiyil

##### Pediatrics, Christian Medical College, Vellore, India

###### **Correspondence:** Sathish Kumar

**Introduction:** Anti-C1q has been associated with systemic lupus erythematosus (SLE) as well as in other connective tissue diseases. They have been considered as a marker for disease activity and presence of nephritis in previous studies

**Objectives:** To determine the prevalence of anti- C1q antibodies in the pediatric SLE population

To determine clinical associations of elevated anti- C1q antibody levels especially with lupus nephritis.

**Methods:** Sera of pediatric SLE patients who fulfilled ACR criteria for SLE were recruited. After obtaining informed consent, blood samples were tested for anti- C1q antibody by commercially available ELISA kit. Prevalence of anti-C1q and its association with lupus nephritis were determined.

**Results:** Out of total 150 children with SLE, anti- C1q positivity was present in 95 children (64%), at a cut off value of 20U/ml. Children with proteinuria, low C3, low C4 and anti dsDNA positivity had were significantly more likely to have anti- C1q antibody positivity. Children with lupus nephritis were significantly more likely to have anti C1q antibodies positive than children without renal involvement (74% vs. 51% , p= 0.02). Among the children with lupus nephritis, children with active renal disease were more likely to have anti- C1q positivity than in children with quiescent disease (88% vs. 53% , p= 0.002). Anti-C1q antibodies had a sensitivity of 74% and specificity of 54% at a cut off value of 22U/L , for renal disease in pSLE

**Conclusion:** Out study confirms previous findings of the association of anti-C1q antibodies with nephritis and disease activity in pSLE. Anti-C1q antibody titers were found to have positive correlation with renal disease in children with pediatric SLE, and could be used as an adjunctive biomarker in monitoring disease activity in children with lupus nephritis.

**Disclosure of Interest**

None Declared

### P288 REDUCED TRAIL AND BCL-2 AND INCREASED CASPASE 3 EXPRESSION IN NK CELLS FROM JUVENILE SYSTEMIC LUPUS ERYTHEMATOSUS (JSLE) PATIENTS RELATED WITH DISEASE ACTIVITY, NEUROPSYCHIATRIC INVOLVEMENT AND NEPHRITIS

#### Bernadete L. Liphaus^1^, Simone C. Silva^1^, Patricia Palmeira^1^, Clovis A. Silva^2,3^, Claudia Goldenstein-Schainberg^3^, Magda Carneiro-Sampaio^1^

##### ^1^Laboratory of Medical Investigation, Children’s Institute, Universidade de São Paulo; ^2^Rheumatology Unit, Children's Institute, Universidade de São Paulo; ^3^Disciplina de Reumatologia, Universidade de São Paulo, São Paulo, Brazil

###### **Correspondence:** Bernadete L. Liphaus

**Introduction:** Natural killer (NK) cells are critical for modulating immune response^1^. JSLE patients studies demonstrated depleted number of NK cells and defective cytolysis that related with disease activity^1,2^.

**Objectives:** To evaluate whether apoptosis-related proteins expressions in NK cells contribute to its defects in JSLE patients and relate with disease activity parameters, neuropsychiatric involvement and nephritis.

**Methods:** Thirty-five JSLE patients (30 girls, mean age 15.1±2.7 yrs, ACR criteria), 7 sex- and age-matched healthy individuals (6 girls, mean age 13.0±2.9), 4 JIA disease controls (3 girls, mean age 10.7±4.8 yrs, ILAR criteria) and 13 JDM controls (8 girls, mean age 13.1±3.5 yrs, Bohan and Peter criteria) had Fas, FasL, TRAIL, TNFR1, Bcl-2, Bax, Bim and Caspase 3 expressions in NK (CD3-CD16+CD56+) cells simultaneously determined by flow cytometry. Disease activity parameters included SLEDAI score, ERS, CRP, anti-dsDNA, C3 and C4 levels. Eleven patients had active disease (SLEDAI ≥4), 21 had positive anti-dsDNA, 9 had neuropsychiatric involvement and 28 had nephritis. Mann-Whitney test was used for two group comparisons and Spearman’s rank for correlations. P values <0.05 were considered significant.

**Results:** Reduced proportion of TRAIL-expressing NK cells was observed in JSLE patients (median 11.4 vs. 51.9%, p=0.03), in those with active disease (median 5.4%, p=0.02), in patients with positive anti-dsDNA (median 6.6%, p=0.009), in those without neuropsychiatric involvement (median 13.8%, p=0.03) and in patients with nephritis (11.4%, p=0.02) compared with healthy controls. Reduced proportion of Bcl-2-expressing NK cells was seen in JSLE patients (median 81.8 vs. 94.3%, p=0.04), in those with inactive disease (83.7%, p=0.04) and in patients without neuropsychiatric involvement (median 80.0%, p=0.03) compared with healthy controls. JSLE patients with nephritis also had increased proportion of caspase 3-expressing NK cells (median 2.5 vs. 1.0%, p=0.03) compared with healthy controls. Surprisingly, proportion of FasL-expressing NK cells positively correlated with SLEDAI score (r=0.6, p=0.002) and with C3 levels (r=0.5, p=0.005). No differences were observed comparing JSLE patients, JIA and JDM disease controls.

**Conclusion:** JSLE patients present altered expressions of TRAIL, Bcl-2 and caspase 3 apoptosis-related proteins in NK cells, particularly in those with active disease, with positive anti-dsDNA, with nephritis and those without neuropsychiatric involvement, which may contribute to NK cells defects and consequently to disease pathogenesis.

**References**

1. Fogel LA, et al. Arthritis Res Ther. 2013; 15:216. 2-Yabuhara A, et al. J Rheumatol. 1996; 23(1):171-7.

Authors thank FAPESP (grant 2012/22997-4) for supporting this study.

**Disclosure of Interest**

None Declared

### P289 MUTATION OF LRBA GENE IN A JUVENILE SYSTEMIC LUPUS ERYTHEMATOSUS (JSLE) PATIENT

#### Bernadete L. Liphaus^1^, Iris Caramalho^2^, Jocelyne Demengeot^2^, Clovis A Silva^3^, Magda Carneiro-Sampaio^1^

##### ^1^Laboratory of Medical Investigation, Children’s Institute , São Paulo, Brazil; ^2^Instituto Gulbenkian de Ciência, Oeiras, Portugal; ^3^Rheumatology Unit, Children’s Institute , São Paulo, Brazil

###### **Correspondence:** Bernadete L. Liphaus

**Introduction:** SLE has been mainly associated with primary immunodeficiencies of early complement components and selective IgA deficiency^1,2^. LRBA deficiency leads to immuno-deficiency of heterogenous presentation, as respiratory infections, hypogammaglobulinemia, reduced number of specific B cell subsets, functional T cell defects and aberrant autophagy^3^. LRBA gene mutations have also been associated with autoimmune diseases, including inflammatory bowel disease, endocrinopathy, autoimmune hemolytic anemia and thrombocytopenia, and occasionally arthritis but not SLE.

**Objectives:** To report clinical and laboratory features of a JSLE patient carrying a novel mutation in LRBA gene.

**Methods:** Whole exome sequencing was performed from -80ºC stored genomic DNA (PIDBioRep)^4^.

**Results:** A 10-year-old girl born from healthy consanguineous parents presented with wheezing and cough since 1-year-old, and respiratory infections, arthralgia and chronic bloodless liquid diarrhea since 7 years of age. A sister with bronchitis, few diarrhea episodes and hypertiroidism had been diagnosed with Graves’ disease. Our patient initial tests showed normal sodium and chloride sweat dosage, negative stool cultures, non-specific colitis in colon biopsy, increased IgG, decreased IgM that afterwards normalized (39 mg/dL and 130 mg/dL, respectively), normal IgE and decreased IgA (1.0 mg/dL), normal C3, C4 and CH50 levels and immunophenotyping. After 6-month follow-up she had polydipsia and polyuria, serum glucose was 724 mg/dL, anti-insulin antibody was positive while anti-GAD and anti-IA2 were negative. Diagnosis of diabetes mellitus was established and NPH insulin initiated. After 1-year follow-up she presented acute polyarthritis (cervical spine, left elbow, wrists and knees), autoimmune hemolytic anemia (hemoglobin of 7.3 g/dL and positive Coombs test), alopecia, malar rash and pericarditis. Autoantibodies analyzes revealed homogeneous ANA 1/1,280, positive anti-dsDNA (119, normal up to 50 IU/mL), anti-cardiolipin IgG (13 GPL, normal up to 10 U/mL), anti-TG and anti-TPO with normal free T4 level, and negative anti-Sm, antiSSA/Ro and antiSSB/La. As patient fulfilled criteria for JSLE, prednisone, hydroxychloroquine and cyclosporine were initiated. During follow-up she presented respiratory infections, venous thrombosis, septic shock and died at 20 years of age from pneumonia. The whole exome sequencing identified a novel homozygous 1-bp deletion in exon 44 of the LBRA gene, resulting in a frameshift with introduction of a premature stop codon (p.Trp2248Glyfs*26). This mutation is located in a highly conserved region within the BEACH domain that interacts with CTLA4 and prevents its degradation, and is predicted to result in nonsense-mediated mRNA decay of the transcript, leading to absence of protein.

**Conclusion:** LRBA-deficient patients have been shown to present decreased intracellular and cell-surface CTLA4 and reduced Treg function, which is likely to explain their multiple autoimmune syndromes, now shown to include SLE. Therefore, abatacept might be a therapeutic option for JSLE cases in which LRBA deficiency may be suspected, such as in patients presenting other autoimmune diseases. Informed consent for publication had been obtained from the parents.

**References**

1. Jesus AA, et al. Lupus 2011;20:1275-84; 2-Carneiro-Sampaio M, et al. J Clin Immunol. 2008;28:S34-S41; 3-Alkhairy OK, et al. J Clin Immunol. 2016;36;33-45; 4-Liphaus BL & Carneiro-Sampaio M. Biopreservation and Biobanking 2013; 11:324-6.

Authors thank São Paulo Research Foundation (FAPESP grants 2008/58238-4 and 2012/22997-4) and Fundação para a Ciência e a Tecnologia, Portugal (Postdoctoral fellowship SFRH / BPD / 111454 / 2015) for their financial support.

**Disclosure of Interest**

None Declared

### P290 CHARACTERIZATION OF A COHORT OF COLOMBIAN MALE PATIENTS WITH JUVENILE SYSTEMIC LUPUS ERYTHEMATOSUS

#### Angela C. Mosquera^1^, Clara Malagon^1^, María del Pilar Gómez^2^, on behalf of GRIP, Camilo Vargas^3^, on behalf of GRIP, Tatiana Gonzalez^4^, on behalf of GRIP, Christine Arango^5^, on behalf of GRIP, Pilar Perez^1^, on behalf of GRIP, Karen Jimenez^1^, on behalf of GRIP and Grupo de Reumatología e Inmunología Pediátrica ( GRIP ) Colombia

##### ^1^Pediatric Rheumatology, Universidad El Bosque, Bogota; ^2^Pediatric Rheumatology, Postgraduate of pediatrics.Libre University; ^3^Pediatric Rheumatology, Postgraduate of pediatrics, Del Valle University, Cali; ^4^Pediatric Rheumatology, Postgraduate of pediatrics, Cartagena University, Cartagena; ^5^Pediatric Rheumatology, Universidad Tecnológica de Pereira, Pereira, Colombia

###### **Correspondence:** Angela C. Mosquera

**Introduction:** Systemic Lupus Erythematosus (SLE) is a complex autoimmune disease. Genetic an environmental factors intervene in its physiopathology. It is dercribed the involvement of estrogens in the pathogenesis and course of the disease^1^. In pediatric patients the differences in frequency of SLE are smaller between male and female individuals. Variations in the clinical presentation and severity of both adult and pediatric male patients are described in the literature^2^.

**Objectives:** To describe the clinical and paraclinical characteristics of a cohort of male patients with Juvenile Systemic Lupus Erythematosus.

**Methods:** Multicenter, descriptive, retrospective study. A unique data collection format was used. Analysis of the information was made with SPSS20

**Results:** N = 57. Mean age at diagnosis 12 years (SD 2.8 and range 4-17 years), 60%> 12 years. Average follow-up time of 26 months (SD 23m). The commitment by organs /systems at debut was: 1. Skin in 52.6% 2. Oral / nasal ulcers 16% 3. Arthritis 56% (poly 49%) 4. Serositis 32% 5. Renal 68%, kidney biopsy at onset 18/39, 61% class IV nephropathy, 15% required dialysis 6. Hematologic: 21% hemolytic anemia, 19% thrombocytopenia , 33% leukopenia / lymphopenia 7. Neurological 5.3%. Autoantibody profile: 95% positive antinuclear antibodies, titers> 1: 640 63% and 54% mottled pattern. 77% had positive anti-DNA antibodies, 51% positive anti-SM antibodies, C3 hypocomplementemia 95% and C4 hypocomplementemia 97%.Other clinical manifestations at onset: Antiphospholipid syndrome 14%, 7% Hashimoto's thyroiditis, 14% infection (88% pneumonia). All patients received steroid at debut 60% in IV pulses and 53% cyclophosphamide (CFM). During follow-up, 11 patients had renal involvement, 43% had Class IV nephropathy. Immunosuppressants frequency during the course was: 42% CFM, 53% Azathioprine, 30% Mycophenolate mofetil, 10% Methotrexate. 28% used more than one medication. 10% of patients required Rituximab. Complications during follow-up: 23% short stature, 18% pubertal delay, 7% depression, cataract and chronic dialysis, 5% osteoporosis 3.5% fibromyalgia and 2% kidney transplant. Mortality 10%. A statistically significant difference was found between prepubertal disease onset (under 12 years of age) and frequency of short stature (p 0.019). There were no statistical differences in clinical manifestations at onset, autoantibody profile, treatment and other complications between pre- and postpubertal patients.

**Conclusion:** In this cohort of male patients with juvenile SLE the mean age of diagnosis was in early adolescence with a higher frequency of cases in postpubertal patients. The skin, hematological and renal manifestations were the most frequent at debut. The patients required an intensive treatment for the control of the disease and had a high prevalence of complications especially in relation to growth and a mortality of 10%. Lupus in this cohort of males behaves in a similar manner to that reported in the literature of patients with juvenile SLE.

**Disclosure of Interest**

None Declared

### P291 A HOMOZYGOUS TREX1 MUTATION IN A PATIENT WITH FAMILIAR CHILBLAIN LUPUS PHENOTYPE

#### Rosa M. Alcobendas^1^, María Bravo^2^, Sara Murias^1^, Agustín Remesal^1^, Clara Udaondo^1^, Marta Feito^1^

##### ^1^Pediatric Rheumatology; ^2^Inmunology and genetics, university hospital La Paz, Madrid, Spain

###### **Correspondence:** Sara Murias

**Introduction:** In recent years, several mutations located in the TREX1 gene have been associated with different disease phenotypes. Aicardi-Goutières syndrome, familiar chilblain lupus (FCL), systemic lupus erythematosus (SLE) and retinal vasculopathy with cerebral leukodystrophy are included among these conditions.

**Objectives:** To describe the clinical and analytical characteristics of a patient with a homozygous mutation in the TREX1 gene.

**Methods:** Clinical chart review

**Results:** We present a boy, of gypsy ethnicity and son of non-consanguineous parents, who consulted in our unit at 6 years of age due to swelling, limitation and pain affecting knees, ankles and elbows. The child also reported recurrent severe episodes of pallor and erythema involving fingers and toes during several years, which had even led to amputation of the distal phalanx of the second finger of the left hand due to necrosis. Initially he was diagnosed with Juvenile Idiopathic Arthritis (JIA), antinuclear antibodies (ANA) negative, and secondary Raynaud syndrome. Etanercept and methotrexate were started with modest response. However, two years later, several intense chilblain appeared, affecting both auricular pavilions, and all fingers and toes. Given the suspicion of associated vasculopathy, immunological screening was performed, with no relevant findings; and a genetic study of autoinflammatory/autoimmune diseases was carried out by NGS panel, detecting a homozygous mutation in the TREX1 (R97H). This mutation has been previously reported as associated with Aicardi-Goutières syndrome, and also described in one patient with early onset SLE who presented cerebral vasculitis one year after disease onset^1,2^. Nevertheless, the clinical features and the laboratory results of our patient (Raynaud syndrome, chilblain and arthritis with no neurologic symptoms and absence of autoantibody) permit to include him among familiar chilblain lupus phenotype^2^. When the family tree data were collected, a history of chilblain, deafness, abortion due to malformations, neurological delay from the neonatal period, arthritis and amputations were found through both family branches. A genetic study of the patient relatives will be performed in the near future, in order to confirm wether the various familiar antecedents are related to the mentioned mutation as this option seems probable.

**Conclusion:** To our knowledge, this is the first time that a R97H mutation in homozygosis is described in a patient with FCL phenotype. However, the family history suggests that he same mutation may be associated to different phenotypes too, as has been previously described in the literature^2,3.^

Written informed consent has been obtained

**Disclosure of Interest**

None Declared

### P292 PROTEIN LOSING ENTEROPATHY ASSOCIATED TO SYSTEMIC LUPUS ERYTHEMATOSUS IN CHILDHOOD: CASE REPORT AND REVIEW OF LITERATURE

#### Roberta Naddei, Francesca Orlando, Marina Amico, Teresa Lastella, Carolina Porfito, Maria Alessio

##### Mother and Child Department, University of Naples Federico II, Naples, Italy

###### **Correspondence:** Roberta Naddei

**Introduction:** Protein Losing Enteropathy (PLE) is a rare expression of intestinal diseases characterized by loss of serum proteins into the gut lumen. PLE has been seldom associated to Systemic Lupus Erythematosus (SLE) and has been rarely described in infancy as clinical presentation of SLE.

**Objectives:** To describe a case of pediatric Lupus Protein Losing Enteropathy (LUPLE) and provide an update on the characteristics of LUPLE in children.

**Methods:** Report of a patient with recurrent unexplained episodes of hypoalbuminemia and review of literature concerning LUPLE, carried out searching MEDLINE database via PubMed, with no limits on the date of publications.

**Results:** A 10-year-old Vietnamese boy was referred to our Pediatric Rheumatology Unit for the third episode of sudden anasarca. He previously presented two steroid-responsive episodes of hypoalbuminemia with oedema, ascribed to nephropathy, even if no nephrotic proteinuria was detected. At the admission, the serum albumin was undetectable and raised levels of acute phase reactants were detected (Erythrocyte sedimentation rate: 75 mm/h; C-reactive protein: 3,85 mg/dl). Liver and kidney function tests were normal and 24-h urinary protein quantification was negative for proteinuria. An increase of faecal alpha-1 antitrypsin (αAT) was disclosed (7.21 mg/g with normal range 0.38-0.5) and ^99m^Tc-labelled human serum albumin scintigraphy (^99m^Tc-HAS) confirmed severe PLE, mostly deriving from the duodenojejunal area. Gastrointestinal biopsies revealed gastric and duodenal lymphangiectasias and a colonic mucosal chronic inflammation. Positivity of antinuclear antibody (ANA) with speckled appearance and SSA/SSB antibodies (146 U and 70 U with normal range <20) was detected. Thereby, the diagnosis of LUPLE was suspected. Treatment with intravenous methylprednisolone was started, followed by daily oral prednisone combined to daily oral azathioprine, with a progressive improvement in values of serum albumin and clinical response. Almost two years later, the patient developed other clinical findings that fulfilled the Systemic Lupus International Collaborating Clinics (SLICC) diagnostic criteria for SLE.

The systematic literature searching revealed 7 cases of LUPLE in children younger than 16 years. Considering reported cases and the present case, this condition is prevalent in females (6/8) and in Asiatic patients (3/8). PLE could represent either the first presentation of the collagen disease (4/8) or a successive complication. All the 8 patients showed peripheral oedema but only one patient presented gastrointestinal symptoms, such as diarrhoea and abdominal pain. To identify gastrointestinal protein loss and protein leakage site, αAT faecal clearance and ^99m^Tc-HAS are respectively the most specific diagnostic methods. As concerned gastrointestinal histology, non-specific inflammation involving at least one of duodenum and colon was observed in 5 patients and lymphangiectasias were detected in our and in another patient. Concerning autoimmune profile, ANA antibody is usually present; positivity of anti-DNA and anti-SSA/SSB is described in the half of the reported children. All but one reported pediatric cases were treated with both steroids and immunosuppressive treatment in order to control gastrointestinal disease or the other manifestations of the autoimmune disease.

**Conclusion:** The diagnosis of LUPLE has to be suspected in every child with hypoalbuminemia without evidence of renal and hepatic disease, even without gastrointestinal symptoms. It could represent a manifestation of the disease either already diagnosticated or not detected yet.

Written consent for publication was obtained from the parents of patient.

**Disclosure of Interest**

None Declared

### P293 SAFETY AND HUMORAL IMMUNE RESPONSE AFTER A BOOSTER DOSE OF TDAP VACCINE IN JUVENILE SYSTEMIC LUPUS ERYTHEMATOSUS PATIENTS AND CONTROLS

#### Octavio A. B. Peracchi, Claudio A. Len, Yamada Juliana, Aline Nicacio, Maria Isabel D. Moraes-Pinto, Maria Teresa Terreri

##### Universidade Federal de São Paulo, São Paulo, Brazil

###### **Correspondence:** Octavio A. B. Peracchi

**Introduction:** Pertussis infections have increased worldwide and knowledge on the immune response after adult Tdap vaccine is scarce in patients with autoimmune diseases.

**Objectives:** This study evaluated the immune response through antibody profile after Tdap vaccine in juvenile systemic lupus erythematosus (jSLE) and controls. The jSLE group was also monitored for possible disease flares.

**Methods:** We included 21 jSLE and 19 healthy adolescents who had already received three whole-cell DTP vaccine (DTwP) plus two booster doses and had lymphocytes count > 500. Blood samples were collected immediately before the Tdap vaccine and 14 and 28 days thereafter.Tetanus, diphtheria and pertussis antibodies were tested by ELISA.

**Results:** jSLE group median age was 15y (9-18y) and control group, 15y (9-15.6y). The immune response was evaluated according to antibody levels as shown in Table 1. Both groups had a similar response for antibody levels for tetanus and diphtheria. The opposite was seen for pertussis, with similar antibody levels. In the control group 96.7% on D14 and 100% on D28 seroconverted to pertussis (p=0.012). On the other hand, in the jSLE group, many adolescents did not seroconvert on D14 and D28 (54.5% vs 63.6%, p = 0.581). Protective levels were noted for tetanus (p=0.031) and diphtheria (p<0.001) in jSLE group. No difference was found between groups on the frequency of adverse events (p=0.081), except for local tenderness, which was more frequent in the control group (p=0.042). Disease flares were not observed after the vaccine.

**Conclusion:** In this study, children and adolescents with jSLE and healthy controls showed adequate humoral immune response to tetanus and diphtheria. The response to pertussis after Tdap vaccine seemed to be less evident in jSLE patients.

**Disclosure of Interest**

None Declared

**Table 1 (abstract P293).** Tetanus, diphtheria and pertussis antibodies for jSLE patients and control group on days 0, 14 and 28 (mean and SD)

	D0	D14	D28	p^1^
Tetanus				
Control	0.33 ± 0.45 ^B^	41.46 ± 41.73^A^	47.50 ± 44.07 ^A^	<0.001^b^
Lupus	0.20 ± 0.18 ^B’^	39.08 ± 54.76 ^A’^	46.57 ± 59.77 ^A’^	<0.001^b^
Diphtheria				
Control	1.12 ± 1.40 ^B^	40.43 ± 33.23 ^A^	29.98 ± 21.17 ^A^	<0.001^b^
Lupus	0.21 ± 0.28 ^B’^	14.67 ± 21.99 ^A’^	11.65 ± 14.20 ^A’^	<0.001^b^
Pertussis				
Control	8.16 ± 8.26 ^B^	90.68 ± 105,69 ^A^	76.81 ± 90,59 ^A^	<0.001^b^
Lupus	15.55 ± 13.50 ^B’^	53.35 ± 50.69 ^A’^	52.70 ± 50.38 ^A’^	<0.001^b^

p^1^ – Friedman test (^b^)

(A) and (B) mean differences in Dunn-Bonferroni multiple comparison test in the control group

(A’) and (B’) mean differences in Dunn-Bonferroni multiple comparison test in the jSLE group

Kolmogorov-Smirnov test for tetanus (p=0.007), diphtheria (p<0.001) and pertussis (p<0.001)

### P294 BULLOUS SYSTEMIC LUPUS ERYTHEMATOSUS

#### Atique Ahemad^2^, Pallavi Pimpale Chavan^1^, Shaila R. Khubchandani^2^, Raju P. Khubchandani^1^

##### ^1^Pediatric Rheumatology Clinic,Department of Pediatrics; ^2^Department of Histopathology, Jaslok Hospital and Research Centre, Mumbai, India

###### **Correspondence:** Pallavi Pimpale Chavan

**Introduction:** Bullous systemic lupus erythematosus (BSLE) is an autoantibody mediated vesicobullous disease characterized by presence of circulating anti- type VII collagen antibodies. BSLE manifests commonly in second or third decade of life and is rarely seen in children.

**Objectives:** We describe this rare variant in a girl who was difficult to treat in the initial weeks but is now in remission.

**Methods:** A 11 year old female initially presented to another centre in June 2017 with arthralgia, hair fall and oral ulcers. She was diagnosed as systemic lupus with no other organ involvement and started on hydroxychloroquine.

She presented to us in November 2017 - 5 months later, with extensive skin eruptions on face, neck, trunk, extremities and oral mucosa since 3 weeks. On examination, her weight was 54 kg with height of 155 cm, she had tense vesico-bullous lesions filled with clear fluid on face, neck, trunk, extremities and in the oral cavity. Systemic examination was normal. Her SLEDAI score was 4. There was no recent drug ingestion.

Her hemogram, erythrocyte sedimentation rate and urinalysis were normal, Direct Coombs Test was 1+ with C3 - 53.3 mg/dl, C4- 11.5 mg/dl and negative dsDNA, APLA IgG/IgM and lupus anticoagulant. Skin biopsy was performed and immunofluorescence examination showed IgG and C3 positive linear deposits at the dermo-epidermal junction with positive IgM, IgA, C1q and fibrinogen.

**Results:** She was pulsed with methylprednisolone (30mg/kg) for 3 consecutive days, followed by oral prednisolone (1mg/kg/day), dapsone (1mg/kg), hydroxychloroquine and supplements. As the new lesions continued to erupt, dose of dapsone was increased at the end of Week 2 (1.5mg/kg) and methotrexate (10mg /sq m po once weekly) was added. With no change in clinical status, Mycophenolate Mofetil (MMF) (330mg/sq m/dose, twice a day po) was added at the end of Week 4 and titrated upwards (500mg/sq m,twice a day po) in Week 12. By Week 15 there were no new crops and she finally achieved remission with no fresh lesions and normal hemogram and complement levels with residual hyperpigmentation. Steroid tapering has commenced successfully and currently she is on oral prednisolone (0.15mg/kg/day), dapsone (1.5mg/kg), MMF (500 mg/sq m/dose, twice a day po), methotrexate (10mg/sq m po once a week), hydroxychloroquine and supplements.

**Conclusion:** Vesicobullous lesions in lupus may morphologically resemble Stevens Johnson syndrome but are easily differentiated on histology and immunofluorescence. Such cutaneous lesions are rare in childhood and our case was rather severe in the initial weeks, refractory to Dapsone which usually is effective in this form. In our case the SLEDAI does not accurately reflect the true intensity or activity of the skin domain. Informed consent had been obtained.

**Disclosure of Interest**

None Declared

### P295 SPONDYLOENCHONDRODYSPLASIA – A CASE FROM INDIA

#### Pallavi Pimpale Chavan^1^, M Moreews^2^, Shashi Ranade^3^, A Belot^4^, Raju P. Khubchandani^1^

##### ^1^Pediatric Rheumatology Clinic,Department of Pediatrics, Jaslok Hospital and Research Centre, Mumbai, India; ^2^University of Lyon 1 & INSERM U1111, Lyon, France; ^3^Consultant Pediatrician, Ranade Hospital, Karad, India; ^4^Pediatric Rheumatology Unit, HFME, HCL, Lyon, University of Lyon 1 & INSERM U1111, Lyon, France

###### **Correspondence:** Pallavi Pimpale Chavan

**Introduction:** Spondyloenchondrodysplasia (SPENCD) is a very rare genetic immuno-osseous dysplasia characterized by skeletal anomalies (short stature, platyspondyly) and enchondromas in the long bones or pelvis. SPENCD may have a heterogeneous clinical spectrum with neurological involvement or autoimmune manifestations, such as systemic lupus erythematosus (SLE).

**Objectives:** We describe an Indian child with SPENCD, who presented with features of SLE , skeletal dysplasia and enchondromas.

**Methods:** A 9 year old male born of second degree consanguineous marriage presented with fever, oral ulcers, leg pain with morning stiffness since 1 month. He was worked up by his primary paediatrician for pyrexia of unknown origin and a summary of laboratory tests performed included, hemogram with hemoglobin 8.7 gm/dl, white cell count 5900 /cu mm, platelet 1.85 x 10^3^ cu mm, erythrocyte sedimentation rate 100 mm/hr, antinuclear antibody 4+. Suspecting connective tissue disease child was referred to us for further evaluation.

**Results:** On further enquiry, there was past history of multiple medical visits for poor growth since infancy and a bony swelling of the right forearm leading to incomplete supination for which he was operated at age 5 years. No details were available. His milestones were appropriate for age with normal scholastic performance. His weight (16.9 kg) and height (113cm) were below the 3rd centile. On examination, he had pallor, facial rash, mucositis of the palate, generalised lymphadenopathy, triangular facies, prominent anteverted ears, prominent conjunctival blood vessels, supernumerary teeth, right elbow swelling, paucity of body hair. On systemic examination abdomen was soft with hepatosplenomegaly and other systems were normal.

On further investigation his dsDNA was 4+, Direct Coombs test was positive, C3 - 19.1 mg/dl, C4 - 3.36 mg/dl, urinalysis was normal, Anti Ro and lupus anticoagulant were negative and APLA IgG was positive and APLA IgM negative. The diagnosis of SLE was confirmed.

Considering, consanguineous marriage, short stature and right elbow swelling with other subtle dysmorphisms described above, a syndromic form of lupus was suspected and a skeletal X ray analysis showed extensive areas of metaphyseal abnormalities with epiphyseal remodelling changes and platyspondyly. Hence a clinical diagnosis of Spondyloenchrondrodysplasia (SPENCD) was considered. Sanger sequencing of *ACP5* encoding the protein TRAP revealed homozygous non sense mutation (c.849T>A, p.(Tyr283stop) confirming the diagnosis of SPENCD. He was further investigated for diseases associated with SPENCD, which revealed normal thyroid function, IGF – 1, immunoglobulin levels and 2DECHO.

He was started on hydroxychloroquine (5mg/kg/day), oral prednisolone (0.6mg/kg/day), low dose aspirin, supplements and was advised sun protection. On follow up after 2 months, he had gained 1 kg of weight, was afebrile and there was no arthritis, mucositis or rash

**Conclusion:** SPENCD is an immunosseous dysplasia and a very rare cause of monogenic lupus. This, is the second case of SPENCD reported from India. The entity should be suspected in children with SLE with a consanguineous background who have short stature and bony abnormalities. We expand the phenotype here with additional dysmorphic features comprising supernumerary teeth, triangular facies, prominent anteverted ears and prominent conjunctival blood vessels not described before to the best of our knowledge. Informed consent for publication had been obtained.

**Disclosure of Interest**

None Declared

### P296 RESPONSE TO THE TREATMENT WITH DOUBLE IMMUNOSUPPRESSOR (CYCLOPHOSPHAMIDE AND MYCOPHENOLATE MOFETIL) IN PEDIATRIC PATIENTS WITH CLASS IV LUPUS NEPHRITIS

#### Yuridiana Ramirez, Talia Dìaz, Enrique Faugier, Rocio Maldonado

##### Hospital Infantil de México Federico Gómez, Mexico City, Mexico

###### **Correspondence:** Yuridiana Ramirez

**Introduction:** Systemic Lupus Erythematosus (SLE) is an autoimmune disease with multisystem involvement. In pediatric patients, renal disease is higher in comparison with that observed in adults, and it is reported that up to 99% of patients with SLE develop a certain degree of affection throughout the disease. Lupus Nephropathy in Hispanic patients is more aggressive, with reports of failure to first-line treatment in up to 43% of cases, which is why multitarget therapies are currently being studied, the use of two or more immunosuppressants, to achieve a complete remission in patients with lupus nephropathy, specifically class IV.

**Objectives:** The objective of the study was to evaluate the response to treatment using schemes with double immunosuppressant: intravenous cyclophosphamide associated with mycophenolate mofetil or azathioprine.

**Methods:** In material and methods, 60 case files of patients treated at this Institution were reviewed, of which only 40 complied with the treatment schedule for at least 6 months and had a written report of renal biopsy for Class IV Lupus Nephropathy performed at the Hospital Infantil de México.

**Results:** From the results it was found that 82% corresponded to female patients and 18% to male patients, the average age during follow-up was 12.4 years. The main pattern reported of ANA antibodies was a homogeneous pattern and 92.5% presented anti-double-stranded DNA by Elisa positive. The response to the treatment was evaluated with 2 parameters, the first one based on renal function (creatinine) and urinary sediment finding that 67.5% had complete remission, while based on the response to 12-hour proteinuria, a complete remission was reported. 46% Improvement was observed in other indices such as hemoglobin, creatinine and complement levels. The adverse effects of infectious type reported were 28% pneumonia and 22% upper respiratory infection, with a mean hospital stay of 5.1 days (3.5). Only 20% of the patients presented vomiting associated with the application of cyclophosphamide.

**Conclusion:** We conclude that our population presented a response greater than 50% with the use of double immunosuppressive scheme, a value that is comparable with that reported in other series and also without presenting a higher rate of associated adverse effects. Although the study presented limitations due to the fact that not all the indices were requested to assess remission according to the current definitions, it is an initial descriptive study that shows that the therapy used in our institution presents good results.

**Disclosure of Interest**

None Declared

### P297 CLINICAL PROFILE OF SEVEN CHILDREN WITH HEREDITARY COMPLEMENT DEFICIENCY FROM A CENTER IN MUMBAI, INDIA

#### Prajakta Ranade^1^, Yanick J. Crow^2^, Pallavi Pimpale Chavan^3^, Luis Seabra^4^, Gillian Rice^5^, Raju Khubchandani^6^

##### ^1^Paediatrics, St John's Medical College Hospital, Bangalore, India; ^2^Centre for Genomic And Experimental Medicine, Institute of Genetics and Molecular Medicine, Edinburg, UK; ^3^Pediatrics, Jaslok Hospital & Research Centre, Mumbai, India; ^4^Neurogenetics and Neuroinflammation, Laboratory of Neurogenetics and Neuroinflammation, Paris, France; ^5^Division of Evolution and Genomic Science, Manchester Academic Health Science Centre, Manchester, UK; ^6^Pediatrics/Paediatric Rheumatology, Jaslok Hospital & Research Centre, Mumbai, India

###### **Correspondence:** Prajakta Ranade

**Introduction:** While systemic lupus erythematosus (SLE) is classically considered as a multifactorial polygenic disease, hereditary monogenic forms are well recognized. Deficiencies in the early components of the classical pathway strongly predispose to the development of SLE, of which C1q deficiency is the commonest. Children with C1 q deficiency are estimated to develop SLE in 93% of cases. We report our experience with this entity.

**Objectives:** To describe the clinical and laboratory profile and follow up of a cohort of seven children with hereditary complement deficiency.

**Methods:** We reviewed our data on seven children with hereditary complement deficiency and obtained a genetic diagnosis in 5 of these 7 patients using next-generation sequencing. Informed consent was taken from the parents and the data was tabulated in the study proforma.

**Table:** 

Serial / Sex	CSM	Onset (m)/time to diagnosis (m)	Clinical	Positive Antibody profile	C3/C4/CH50	Genetics	Treatment	Followup (m)/Outcome
1/F	N	10 / 7	Rash, mucositis; neuroregression	ANA Ds DNA Anti Ro	175/23/42.1	Homozygous for a c.606delA / p.Gly204Alafs*78 variant in C1QA	HCQ Sx AZT	21/Improved
2/ F	Y	24 / 24	Rash; mucositis;, arthritis; herpes zoster	ANA Anti Ro	203/73/54	Homozygous for a c.622C>T / p.Q208* STOP variant in C1QA	HCQ, Sx, AZT MMF	96 /Improved
3/ M	Y	21 / 12	Rash; mucositis; delayed development; hyperreflexia	ANA Ds DNA Anti Ro ACLA IgG	158/47.7/ 43	Homozygous for a c.622C>T / p.Q208* STOP variant in C1QA	HCQ, Sx AZT	9 / Improved
4/ M	Y	12/ 24	Rash; mucositis; hyperreflexia; seizures	ANA Anti Ro	117/39/18.6	Homozygous for a c.171delT / p.Gly58Alafs*224 variant in C1QA	HCQ Sx AZT MMF	34 /Improved
5 / F	Y	27/ 4	Rash, mucositis; neuroregression; hemiparesis; cerebral atrophy; hyperreflexia; hypothyroidism	ANA Anti Ro ACLA IgM Anti TPO	179/42.8	Homozygous for a c.79C>T / p.Arg27* variant in C1QA	HCQ Sx Cyc AZT	24/ Improved
6/ M	Y	42/0.5	Rash; mucositis	ANA ACLA IgM	114/NA/ NA	Afflicted sibling of Sr no 2 Not tested	HCQ Sx	15/ Improved
7/ F	Y	48/36	Rash; mucositis; arthritis; proteinuria; infections (varicella,scabies, impetigo); hypothyroidism	ANA	30/ NA/ 35.7	Demise before testing	HCQ, Sx, Cyc MMF	Death

Abbreviations: F: Female, M: Male, CSM: Consanguinity, Y: Yes, N: No, m: months, Sx: Steroids, AZT: Azathioprine, Cyc: Cyclophosphamide, MMF: Mycophenolate

**Results:**

**Conclusion:** Based on our study and others reported, hereditary complement deficiency (notably C1Q deficiency) is the commonest form of monogenic lupus in our part of the world. Afflicted children present most commonly with rash and mucositis below 5 years of age and there is a significant delay in diagnosis due to lack of physician awareness. Neurologic features are frequently seen while renal manifestations are not. Neuroregression responds to immunosuppressive therapy with children improving on their mental, motor and speech milestones. Except in one patient major life-threatening infections have not been a challenge despite high infection rates in the community coupled with immunosuppression.

**Disclosure of Interest**

None Declared

### P298 LUPUS NEPHRITIS. CLINICAL AND HISTOPATHOLOGICAL FEATURES AT ONSET

#### Maria F. Reina, Clara Malagon, Angela C. Mosquera

##### Pediatric Rheumatology post graduate program, Universidad El Bosque, Bogota, Colombia

###### **Correspondence:** Maria F. Reina

**Introduction:** Juvenile systemic lupus erythematosus (jSLE), develops kidney involvement among up to 75% of patients and 10-50% of them progress on to end-stage renal disease. Lupus nephritis (LN) is more frequent and severe in young patients and causes significant morbidity and mortality.

**Objectives:** Describe the clinical, laboratory, histopathological features and treatment in a cohort of LN patients.

**Methods:** Multi-center, retrospective and descriptive study. Medical records were reviewed and the information was recollected using a data collection format. The data analysis was performed with SPSS 20.

**Results:** N = 163. Sex ratio F4: M1, mean age was 12 years (4-16). 37,4% developed jSLE before their 11th birthday. The most common clinical manifestations were: arterial hypertension 48.5%, mild to moderate proteinuria 34.4%, nephritic-nephrotic syndrome 33.1%, hematuria 24%, nephrotic syndrome 20.9% and nephritic syndrome 13,5%. 19% developed acute kidney injury (AKI) and 8.6% progresses on to early chronic kidney failure. The mortality rate was 13.5%. 18% of patients with class IV nephropathy died during follow-up.

137(84%) biopsies were analyzed. The distribution by nephropathy subtype was: 46.6% class IV, followed by 12,9%class II, 9.2% class V, 6.7% class III and 5.8% presented mixed changes (class IV-V in 6 and III-V in 2 patients) and 3.7% histology was modified by treatment. Patients with class IV nephropathy developed: nephrotic syndrome 46,7% (p=0.002), nephritic-nephrotic syndrome 62% (p=0.032) and AKI 76,9% (p=0.057). 86,6% (p=0.002) of class V patients developed nephrotic syndrome. There were no differences between types of LN and the profile of autoantibodies or levels of hypocomplementemia.

Induction treatment: 81,6% received pulses of methylprednisolone plus 76% intravenous cyclophosphamide and 5,5% mycophenolate mofetil (MMF). Maintenance treatment: 32% received MMF and 54% azathioprine. 11.7% required Rituximab, 5.5% intravenous immunoglobulin and 2.5% plasmapheresis. Early dialysis was used for patients with class IV nephropathy and severe deterioration of renal function.

**Conclusion:** In this cohort, LN was severe and associated with significant morbidity which is similar to previously reported studies in medical literature. The most frequent subtype was class IV nephropathy; 64% of them developed nephritic-nephrotic syndrome or nephrotic syndrome with a significant percentage to complications at onset and course of the disease. Second most frequent was class II nephropathy and this one subtype did not have an adequate pathological clinical correlation. The biopsy is essential to classify LN and guide the immunosuppressive treatment.

**Disclosure of Interest**

None Declared

### P299 TYPE I IFN SIGNALING: USEFUL TOOLS IN DIAGNOSIS AND FOLLOW-UP ON AUTOINFLAMMATORY/AUTOIMMUNE DISORDERS

#### Martina Soliani^1^, Francesca Ricci^2^, Donatella Vairo^3^, Silvia Giliani^4^, Marco Cattalini^2^

##### ^1^Pediatric Department, ASST of Cremona, Cremona; ^2^Rheumatology unit, Children's hospital, University of Brescia, ASST Spedali civili; ^3^Institute for Molecular Medicine “A. Nocivelli”; ^4^ Institute for Molecular Medicine “A. Nocivelli”, Department of Molecular and Translational Medicine, University of Brescia, Unit of Cytogenetics and Medical Genetics, Spedali Civili, Brescia, Italy

###### **Correspondence:** Francesca Ricci

**Introduction:** Compelling evidence from studies in systemic lupus erythematosus (SLE) demonstrates that Interferonα (IFNα) and type I IFN signaling are implicated in disease pathogenesis; moreover, upregulation occurring in IFNα production/signaling has been linked to a group of autoinflammatory monogenic diseases named type I interferonopathies.

**Objectives:** The aim of our study was to evaluate type I IFN signaling by assessing two read-outs of IFN activation: STAT1 (both total STAT1 and phosphorylated pSTAT1), and Interferon Score (IS)[1], mirroring the expression of 6 IFN stimulated genes, in two different populations. In a group of SLE patients we wanted to evaluate if the assessment of type I IFN signaling correlates with clinical phenotype/laboratory inflammatory markers (anti dsDNA antibodies)/disease activity and then could be a useful tool for monitoring treatment response. In a group of undiagnosed patients, strongly suggestive for a systemic autoimmune/autoinflammatory disorder (SAD), we aimed to evaluate if the assessment of type I IFN signaling could help in clustering the kind of disorder.

**Methods:** In the last 3 years we have enrolled 21 patients. 9/21 were diagnosed with SLE (ACR criteria). 12/21 patients were highly suggestive for SAD. During periodical evaluations, disease activity index was performed, blood samples were collected and type I IFN signaling was evaluated as compared to healthy controls.

**Results:** In the 9 SLE patients we detected a strong upregulation of type I IFN signaling. These data are in agreement with recent publications, in which type I IFN is described as a main contributor to SLE pathogenesis. Comparison among controls, SLE and SAD groups at time of first examination (T1), showed a significant increase in IS, pSTAT1 and STAT1 in the SLE group as compared to controls (IS: p<0,0001, pSTAT1 and STAT1: p<0,001). Moreover, in the SLE group, a significant reduction of IS, pSTAT1 and STAT1 was evident by comparing samples collected at T1-T2 (ΔT: 6-8 months) and this mirrored both clinical improvement measured by SLEDAI and lowering of anti-ds DNA antibody titres. Results from the SAD group are apparently heterogeneous: only some patients showed an activation of type I IFN axis, but this was quite expected given the criteria by which the patients were recruited.

**Conclusion:** We noticed a good concordance between SLEDAI, anti dsDNA antibodies and pSTAT1/IS, thus stating that the evaluation of type I IFN signaling can be a useful tool for evaluating therapeutic effectiveness in SLE patients. In the SAD group, the evaluation of type I IFN signaling helped us to discriminate among disorders: when the IFN pathway was inactivated, we could more easily rule out previously hypothesized diagnosis of an autoimmune disorder. In those patients where a strong type I IFN activation was detected without a final diagnosis, a strict follow up has been provided, as the upregulation of the IFN pathway may be an early clue of SAD.

**Disclosure of Interest**

None Declared

### P300 Withdrawn

### P301 THE PERFORMANCE OF THE NEWLY PROPOSED EULAR/ACR CLASSIFICATION CRITERIA IN JUVENILE-ONSET SYSTEMIC LUPUS ERYTHEMATOSUS

#### Sezgin Sahin^1^, Sule Bektas^1^, Amra Adrovic^1^, Oya Koker^2^, Kenan Barut^1^, Ozgur Kasapcopur^1^

##### ^1^Pediatric Rheumatology, Istanbul University, Cerrahpasa Medical School; ^2^Pediatric Rheumatology, Istanbul University, Istanbul Medical School, Istanbul, Turkey

###### **Correspondence:** Sezgin Sahin

**Introduction:** The sensitivity and specificity of the ACR-1997 and SLICC-2012 classification criteria in juvenile-onset systemic lupus erythematosus (SLE) are already studied. In previous reports, the main limitations of the ACR-1997 and SLICC-2012 were low sensitivity and low specificity, respectively. To avoid misclassifications, a new set of classification criteria have been developed by the collaboration of the European League Against Rheumatism (EULAR) and the American College of Rheumatology (ACR) and the draft was presented at the 2017 ACR/ARHP Annual Meeting in San Diego, California. After application on 500 SLE patients and 500 controls, the sensitivity and specificity were found as 98% and 97%, respectively.

**Objectives:** To compare the sensitivity and specificity of the new EULAR/ACR criteria with those of the 1997 American College of Rheumatology (ACR) criteria and 2012 Systemic Lupus International Collaborating Clinics criteria in juvenile-onset SLE patients.

**Methods:** Juvenile SLE patients initially were evaluated by ACR-1997, SLICC-2012 and EULAR/ACR classification criteria at baseline, when the diagnosis for the first time had been established by an expert pediatric rheumatologist (OK). All data were obtained from patient records. The diagnostic sensitivity of the three sets of classification criteria were further tested within 1 year of diagnosis and at last patient visit, longitudinally. Subjects with a clinical diagnosis other than SLE for at least 1 year-period, consecutively enrolled as controls. Since patients with autoinflammatory diseases, particularly with FMF constitute the vast majority of our outpatient appointments, these subjects were excluded.

**Results:** A total of 104 juvenile-onset SLE patients were enrolled for the sensitivity performance of classification criteria at diagnosis and 104 controls (69 juvenile idiopathic arthritis, 9 juvenile systemic sclerosis, 5 juvenile dermatomyositis, 1 mixed connective tissue disease, 15 vasculitis and 5 other diseases) for specificity at their last visit. Since the follow-up period was less than 1 year, 12 SLE subjects excluded after baseline evaluation. Finally, 92 SLE subjects were eligible for sensitivity evaluation within 1 year of diagnosis and at last visit. The median age of the SLE patients at diagnosis of clinician was 13.0 years (range 3.1–17.9 years) with a median disease duration of 5.0 years (IQR 3.0-8.0 years). The female-to-male ratio was 4.7:1. The newly developed EULAR/ACR classification criteria were more sensitive than SLICC-2012 and ACR-1997 at diagnosis (93.3% versus 91.3% and 85.6%, respectively), and at first year (95.7% versus 94.6% and 90.2%, respectively (p>0.05). At last visit the sensitivity of the new set of criteria and SLICC-2012 were same (97.8 %), but higher compared to ACR-1997 criteria (95.7%).

Specificity of the EULAR/ACR criteria (86.5%, n=90) were found to be higher than SLICC-2012 (81.7%, n=85). Compared to SLICC-2012, an additional 5 subjects among 104 controls without SLE succeeded to get rid of misclassification as SLE by the newly developed criteria. However, the performance of the new EULAR/ACR criteria in diagnostic specificity (86.5%), could not reach the level of ACR-1997 criteria (89.4%).

**Conclusion:** Juvenile-onset systemic lupus erythematosus was classified by the newly proposed EULAR/ACR criteria with higher sensitivity compared with SLICC-2012 and ACR-1997 at disease onset and within one year of diagnosis. Although the difference was not significant, the new set of criteria seem to be capable of recruiting more children with juvenile SLE to clinical trials. The performance of the newly developed criteria seems more successful in specificity compared to the SLICC-2012.

**Disclosure of Interest**

None Declared

### P302 THE RELATIONSHIP BETWEEN JUVENILE SYSTEMIC LUPUS ERYTHEMATOSUS AND THE TRANSCRIPTION FACTORS NF-ΚB AND PPAR-GAMMA

#### Sezgin Sahin^1^, Sinem Durmus^2^, Amra Adrovic^1^, Kenan Barut^1^, Remise Gelisgen^2^, Hafize Uzun^2^, Ozgur Kasapcopur^1^

##### ^1^Pediatric Rheumatology; ^2^Biochemistry, Istanbul University, Cerrahpasa Medical School, Istanbul, Turkey

###### **Correspondence:** Sezgin Sahin

**Introduction:** Systemic lupus erythematosus (SLE) is an autoimmune disease characterized by high-levels of autoantibodies mainly targeting nuclear antigens and loss of self-tolerance. Peroxisome-proliferator activated receptor gamma (PPARγ) and nuclear factor-kappa beta (NF-κB) are transcription factors, which, within normal levels, have shown to be crucial in immunomodulation namely, activation and development of normal lymphocytes, negative and positive selection of T and B cells. High-levels of NF-κB has inflammatory properties such as release of autoreactive T cells. On the contrary, PPARγ has anti-inflammatory effects, which has been demonstrated to be effective when used early in prevention of disease in murine models of systemic lupus erythematosus.

**Objectives:** Herein, we investigated whether NF-κB and PPARγ could exert opposite effects in the immune response and the possible implications in immunomodulation of juvenile systemic lupus erythematosus.

**Methods:** Serum NF-κB and PPARγ levels were measured in 42 juvenile systemic lupus erythematosus. In addition,19 juvenile systemic sclerosis and 25 age-matched healthy children were selected for patient control and healthy control, respectively. We have also assessed the relation of these transcription factors with organ involvement patterns, disease activity, damage scores, autoantibody and acute phase reactant levels.

**Results:** The control group did not differ from the juvenile SLE patients for age (p>0.05). According to our study, serum NF-κB levels (ng/mL) of juvenile SLE and juvenile systemic sclerosis patients were significantly higher (1.87 ±1.0 and 2.17 ±1.0 versus 1.25 ±0.7), while serum PPARγ levels (ng/mL)were significantly lower than that of healthy controls (1.58 ±0.6 and 1.52 ±0.5 versus 2.03 ±0.9) (p<0.05). The difference in serum levels of both transcription factors was not significant between juvenile systemic lupus erythematosus and juvenile systemic sclerosis. In patients with juvenile systemic sclerosis serum NF-κB levels negatively correlated with serum PPARγ levels (R= -0.49; p=0.032); however, this relationship was not observed in juvenile SLE patients and healthy controls. There was a significant correlation between rodnan skin score and NF-κB levels in juvenile systemic sclerosis patients (R^2^=0.45, p<0.05).

**Conclusion:** Increased serum NF-κB levels represent upregulated signaling cascades, so it is associated with increased levels of pro-inflammatory cytokines. Since juvenile systemic sclerosis and juvenile systemic lupus erythematosus are autoimmune diseases, patients had high levels of NF-κB and low levels of PPAR than controls, as expected. Previous studies revealed that PPARγ activation inhibits NF-κB transcriptional activity. Correlation results in juvenile systemic sclerosis cohort were compatible with this finding, however not in juvenile systemic lupus erythematosus patients. This could be due to the limited number of patients. Further studies with large number of patients are needed to better elucidate the implication of these transcription factors in therapeutic pathways.

**Disclosure of Interest**

None Declared

### P303 LUPUS NEPHRITIS IN A SINGLE-CENTER COHORT OF JUVENILE SYSTEMIC LUPUS ERYTEMATOSUS: DELVING INTO THE TIME OF PROTEINURIC REMISSION

#### Sofia Torreggiani^1,2^, Antonio Mastrangelo^1^, Antonella Petaccia^1^, Denise Pires Marafon^1^, Edoardo Bison^1,2^, Giovanni Montini^1^, Giovanni Filocamo^1^, Francesca Minoia^1^

##### ^1^Fondazione IRCCS Ca' Granda Ospedale Maggiore Policlinico; ^2^Università degli Studi di Milano, Milano, Italy

###### **Correspondence:** Sofia Torreggiani

**Introduction:** The majority of patients with juvenile systemic lupus erythematosus (jSLE) develop lupus nephritis (LN), a major cause of morbidity and mortality. Recent evidence in adult-onset SLE suggests that prompt achievement of proteinuric remission is associated with improved long term renal outcomes. Data on time to proteinuric remission in jSLE are still limited.

**Objectives:** To describe the clinical course of LN in our cohort of jSLE patients and to analyze potential clinical and laboratory features associated with proteinuric remission.

**Methods:** The clinical records of jSLE patients followed at our centre in the last thirty years were retrospectively reviewed. To be included in the study patients needed to satisfy ACR criteria for SLE, to have an age at SLE diagnosis <18 years, a biopsy-proven LN, and at least 5 years of follow-up. Demographic, clinical, laboratory and histological data at LN onset were collected. Significant proteinuria was defined as proteinuria >0,5 g/day or urine protein creatinine ratio (UPCR) >0,5. To be classified as in proteinuric remission patients needed to achieve a proteinuria under the cut-off in two consecutive visits. Data of patients with and without persistent significant proteinuria at 1 year were compared using Fisher's exact test or Mann-Whitney U test as appropriate. Correlation between time to proteinuric remission and variables at LN onset was calculated by means of Spearman's Rho test or Mann-Whitney U test, as appropriate.

**Results:** Twenty-six patients were included in the study, 22 were females. Median age at jSLE onset was 12,9 years (range 5,1-16,2), and at LN onset 13,6 (range 6,2-23,7). In 19/26 patients (73%) kidney involvement was present at jSLE diagnosis. The majority of patients were histologically classified with LN class III and IV (69,2%). All patients received high dose corticosteroids as induction treatment; in 7 patients cyclophosphamide i.v. pulses were added. Twelve patients (46%) received ACE-inhibitors or angiotensin II receptor blockers (ARB). Proteinuric remission was achieved in 73,9% and in 82,6% of patients at 1 and 5 years from LN onset, respectively. Median time to proteinuric remission was 5,4 months (range 0,2-68,8 months). In two patients (7,6%) a severe renal damage occurred. The main features of patients with and without significant proteinuria at 1 year from LN onset are presented in Table 1. Although statistical significance was not achieved, persistence of proteinuria seemed associated with a younger age and nephrotic proteinuria at LN onset. No patient with Class II LN developed persistent significant proteinuria. Correlation analyses highlighted that patients treated with ACE-inhibitors/ARB presented a faster remission of proteinuria (p=0,03).

**Conclusion:** Our cohort suggests that younger patients with nephrotic proteinuria at LN onset present a higher risk of prolonged proteinuria and should be carefully monitored. Treatment with ACE-inhibitors/ARB could be useful to achieve a faster proteinuric remission. Due to the small size of our population, these results need confirmation in larger studies. Furthermore, a longer follow-up will allow to evaluate the role of time to proteinuric remission on long term renal outcome in jSLE.

**Disclosure of Interest**

None Declared

**Table 1 (abstract P303).** See text for description

Significant proteinuria at 1 year	Yes (n=9)	No (n=14)	p-value
Median age at LN onset, yr	12.8	14.3	0.19
Class II LN, n (%)	0/9 (0)	4/14 (28.6)	0.13
Nephrotic proteinuria at LN onset, n (%)	4/9 (44.4)	2/14 (14.3)	0.16
Leukopenia at LN onset, n (%)	0/9 (0)	4/13 (30.8)	0.12
Hypertension at LN onset, n (%)	2/4 (50)	9/12 (75)	0.55
Median SLEDAI at LN onset	12	17	0.30
Anti-dsDNA, n (%)	8/9 (88.9)	12/13 (92.3)	1.0
Anti-Sm, n (%)	2/9 (22.2)	5/14 (35.7)	0.66

### P304 UNEXPECTED PRESENTATION OF JUVENILE SYSTEMIC LUPUS ERYTHEMATOUS; ACQUIRED ANGIOEDEMA

#### Zahide Ekici Tekin^1^, Gulcin Otar Yener^2^, Selcuk Yuksel^2^

##### ^1^Department of Pediatric of Rheumatology, Pamukkale University School of Medicine, Denizli, Turkey; ^2^Department of Pediatric of Rheumatology, Pamukkale University School of Medicine, Denizli, Turkey

###### **Correspondence:** Selcuk Yuksel

**Introduction:** An acquired form of angioedema (AAE) with symptoms of swelling in subcutaneous and mucosal tissues is clinically similar to hereditary angioedema but it is rarer than the hereditary type.

**Objectives:** The AAE occurs rarely the existence of a systemic lupus erythematosus (SLE). The antibodies in SLE may change the structure of C1-INH or diminish its regulatory capacity.

In this study, we aim to present AAE as the initial presentation of SLE and literature review.

**Methods:** We presented a case report and provided information about AAE related to SLE. Hence, literature was researched using the keywords “acquired angioedema,” “systemic lupus erythematous,” and “C1 esterase inhibitor protein.”

**Results:** A 16 year old girl was admitted to our clinic with persistent angioedema in the eyelids and lips, alongside polyarthralgia with fatigue.

Her family and she had no remarkable history for recurrent angioedema or rheumatological disease.

On examination, she had bilateral periorbital and lip swelling without dyspnoea. There was a pain and limitation in the joints of both hands and fingers. The other organ systems were normal on examination.

Laboratory investigations showed leukopenia with a white cell count of 2.19 x 109/L, serum C-reactive protein 0.09 mg/dL (<0.5), erythrocyte sedimentation rate 90 mm/h (0-20), IgG 1811 mg/dL (600-1400). Immunological tests showed a major classical pathway-mediated complement depletion with low C3 of 28.1 mg/dl (90-180) and a low C4 of 2 mg/dl (10-40). C1-INH level was normal of 21.70 mg/dL (18-40). There was no hematuria or proteinuria. . In addition, ANA, anti-dsDNA, anti-Smith antibody in high titer and the direct Coombs tests with complements were positive. According to the SLICC criteria, she was diagnosed as SLE and AAE with the presence of polyarthralgia, leukopenia and positivity of immunologic criteria. After pulse methylprednisolone therapy (1 gr/day), the patient received oral prednisolone (2 mg/kg/day), hydroxychloroquine (4 mg/kg/day) as maintenance treatment. After seven days, the angioedema resolved. The patient has been under treatment for 2 years with prednisolone (4 mg/day) and hydroxychloroquine (4 mg/kg/day) and has experienced neither SLE flares nor angioedema.

Angioedema can occur as either hereditary or acquired types due to vasodilation and increased vascular permeability resulting from inflammatory mediators, especially bradykinin. Bradykinin production is normally regulated by C1-INH. C1-INH has also inhibits the plasma kallikrein-kinin system, which releases bradykinin.

The relationship between SLE and acquired C1-INH deficiency is not fully understood yet. It may be associated with the existence of autoantibodies against C1-INH. In few reports it is suggested that chronic consumption of C2 and C4, production of C2 kinin and other active products resulting from SLE appeared to induce the development of AAE. Why severe complement consumption develops in SLE and sets off AAE is unknown. The complicated reactions during complement consumption notably increase vascular permeability, resulting in a subcutaneous and mucosal edema.

To date, 18 AAE cases were associated with SLE, two patients were male, and the others were female. All patients had positive ANA results. Complement levels of 17 patients were low. Five patients were presented with glomerulonephritis. Three patients developed neuropsychiatric manifestations. The angioedema in lids, face, extremities or intestine had benign prognoses and responded to basic SLE treatment. Patients’ treatments were variable according to their clinical symptoms.

**Conclusion:** Although it is a rare condition, AAE might occur during SLE with a functional or quantitative deficiency of C1-INH. New onset AAE in adolescent girls should be investigated to look for autoimmunity conditions and SLE. Informed consent had been obtained.

**Disclosure of Interest**

None Declared

## Poster walk 7: Autoinflammatory diseases

### P305 DISTINCT CEREBROVASCULAR FEATURES IN PATIENTS WITH ADA2 DEFICIENCY

#### Roberta Caorsi^1^, Maria Savina Severino^2^, Carlo Gandolfo^2^, Angelo Ravelli^3^, Andrea Rossi^2^, Marco Gattorno^1^

##### ^1^Clinica pediatrica e reumatologia; ^2^Department of Neuroradiology, Gaslini Institute, Genoa, Italy; ^3^Clinica pediatrica e reumatologia, Department of pediatrics, Gaslini Institute and University of Genoa, Genoa, Italy, Genova, Italy

###### **Correspondence:** Roberta Caorsi

**Introduction:** Mutations of CECR1 have been recently reported as causative of an inflammatory condition characterized by an early onset vasculopathy resembling polyarteritis nodosa. The clinical manifestations of the disease are heterogeneous with a wide range of severity. Patients with a more serve phenotype present early onset cerebral stroke, which can be either ischemic or hemorrhagic.

**Objectives:** to describe the neuroradiologic features of patients affected by ADA2 deficiency with central nervous system (CNS) involvement.

**Methods:** We reviewed the contrast-enhanced brain MR, MR angiography (MRA), and digital subtraction angiography (DSA) examinations of 5 patients with a confirmed molecular diagnosis of ADA2 deficiency and CNS involvement. The patients were two brothers (R312X and E328D mutations in compound heterozygosis) and three other unrelated patients: a male homozygous for the T360A mutation, a girl homozygous for the G47R mutation and a girl with a structural variation in 22q11.1 leading to not functional enzyme.

**Results:** Four patients presented multiple acute and/or chronic small ischemic infarcts involving the basal ganglia and the midbrain, in keeping with small-vessel occlusions (lacunar strokes). One patient additionally presented a large hemorrhagic infarct in the temporal lobe. Areas of focal accumulation of hemosiderin due to intraparenchymal bleeding were also noted. Interestingly, three patients presented an abnormal contrast-enhancing soft tissue in the interpeduncular cistern, encasing the midbrain perforating arteries. These neuroradiological findings regressed after the treatment with anti-TNF agents. The fifth patients presented a large haemorrhagic infarct in the frontal lobe and after few years a subarachnoid haemorrhage due to the rupture of aneurysm of anterior communicating artery; an aneurysm of the left superior cerebellar artery was also detected. Inflammatory alterations of the vessels were not demonstrated. The brain MRA and DSA performed in all patients showed no relevant vascular stenosis of the major arteries of the circle of Willis.

**Conclusion:** Typical lacunar strokes in patients with ADA2 deficiency are due to occlusion of small perforating arteries, which can be missed on conventional arterial imaging focusing on the vessel lumen of relatively large arteries. We hypothesize that the abnormal soft tissue detected in the interpeduncular cistern in the present patients may represent an abnormal inflammatory perivascular response, leading to small vessel stenosis. The presence of aneurysms in one patient, without signs of inflammation, enlighten a different pattern of vascular involvement of DADA2 mainly represented by structural rather than inflammatory alterations. Wider use of contrast-enhanced high-resolution MR examinations may better demonstrate vascular manifestations in patients with ADA2 deficiency.

**Disclosure of Interest**

None Declared

### P306 RECURRENT PERICARDITIS: THE USE OF IL-1 INHIBITOR

#### Camilla Celani^1^, Brigitte Bader Meunier^2^, Virginia Messia^1^, Manuela Pardeo^1^, Claudia Bracaglia^1^, Giulia Marucci^1^, Pierre Quartier Dit Maire^2^, Fabrizio De Benedetti^1^, Antonella Insalaco^1^

##### ^1^Rheumatology, Bambino Gesù Children Hospital, Rome, Italy; ^2^Unité d'Immunologie-Hématologie et Rhumatologie pédiatrique, Paris, France

###### **Correspondence:** Camilla Celani

**Introduction:** Recurrent pericarditis is a complication of acute pericarditis and affects 15-30% of patients after an initial attack. The etiology is poorly understood and about 80% of recurrent pericarditis are "idiopathic". The therapy seems to be very important to avoid the recurrences. Conventional treatment includes NSAIDs, glucocorticoids, colchicine, and in the last years, treatment with Anakina (anti IL-1) became common in the clinical practice

**Objectives:** To analyze clinical findings and treatment in a cohort of pediatric patients presented with recurrent pericarditis.

**Methods:** patients with at least two episodes of idiopathic pericarditis, followed at Necker Hospital (Paris) and at Bambino Gesù Childern’s Hospital (Rome) between 2006 and 2016 were included in the study. Patients with a known history of autoimmune or autoinflammatory disease were excluded.

**Results:** Thirty-two patients (19 F and 13 M) with recurrent pericarditis were included. The median age at disease onset (first episode of pericarditis) was 11.8 years (range 8-17). The first episode was treated differently: 15 (46%) patients received NSAIDs, 6 (18.8%) and colchicine, and  11 (34.4%)  glucocorticoids. Patients who had received glucocorticoids at the first episode showed relapsed before than the others ,respectively 1.9 vs 6 months with a statistical difference (p<0.02). The first treatment did not influence the number of relapses in our study. Moreover we can divided our study population in two groups: Group 1 (20 patients): recurrence of pericarditis was treated only with NSAIDs, colchicine or glucocorticoid (alone or associated); Group 2 (12 patents) in which the treatment with anakinra (IL 1 inhibitor) was necessary. The group 2 showed a higher number of relapses than the group 1 (median 2.93 vs 1.73 p<0.01). In the group 2, there were more steroid-dependent patients in comparison to the group 1 (respectively 72.7% vs 21.1 ; p<0.005). In the group 2, all patients treated with anakinra showed a complete response within few days (in median after 2-3 days) and were able to discontinue the glucocorticoid therapy. During daily treatment with Anakinra no relapse was reported. In 7 out of 12 patients, the dose of anakinra was tapered and of these patients relapsed. The tapering strategy was to progressively reduce the days of administration during the week. The mean time from the start of anakinra to tapering was 11 ±3,4 months (range 6-15 months) in the 4 patients who experienced relapse versus 17.6 ±4.6 months (range 15-23 months ; p<0.04) in those who did not flare. All the patients who relapsed responded quickly to the reintroduction of anakinra. In 5[IA2] out of 12 patients, anakinra was discontinued after a mean duration on treatment of 32.2 months (range 16-58) ; two patients of these 5 patients relapsed within 2 months, and anakinra was reintroduced with a good response.

**Conclusion:** Our study shows the high variability of treatments of recurrent pericarditis and underlines the importance of a standardized therapy to avoid relapses. Glucocorticoid therapy is associated with a shorter period to flares than the other treatment. Anakinra is an effective treatment; however, discontinuation of anakinra lead to relapses in many cases, principally when it was tapered or stopped within a few months. This study suggests that IL- 1 inibithion should be used rather than steroids in patients with inadequate response to NSAIDs and colchicine . Moreover our data suggests also that a longer term therapy with anakinra reduces the risk of relapses. Further experience on larger population is needed to define treatment duration.

**Disclosure of Interest**

None Declared

### P307 COMPARISON BETWEEN THE CLASSIFICATION OF PATIENTS WITH HEREDITARY RECURRENT FEVER BASED ON GENETIC OR CLINICAL AND GENETIC INFORMATION

#### Silvia Federici^1,2^, Federica Vanoni^3,4^, Francesca Bovis^5^, Nicola Ruperto^6^, Michael Hofer^7^, Marco Gattorno^6^

##### ^1^Division of Rheumatology, Ospedale Gaslini, Genova; ^2^Division of Rheumatology, IRCCS Ospedale Pediatrico Bambino Gesù, Rome, Italy; ^3^Department of Pediatrics, Ospedale San Giovanni, Bellinzona; ^4^Unité Romande d'Immuno-rhumatologie Pédiatrique, CHUV,University of Lausanne, Lausanne, Switzerland; ^5^Biostatistics Unit, Department of Health Sciences, University of Genoa; ^6^Division of Rheumatology, Istituto Giannina Gaslini, Genoa, Italy; ^7^Unité Romande d'Immuno-rhumatologie Pédiatrique, CHUV, University of Lausanne, Lausanne, Switzerland

###### **Correspondence:** Silvia Federici

**Introduction:** Recently a group of geneticists classified the variants reported in Infevers at March 2017 for the *MEFV*, *MVK*, *TNFRSF1A* and *NLRP3* gene and propose a genotype interpretation according to the combination of the single variants and mode of inheritance of the diseases (*J Med Genet 2018)*

**Objectives:** To evaluate the concordance between the pathogenicity classification proposed by geneticists and the classification in 360 real patients blinded evaluated by 33 experienced experts

**Methods:** 360 patients with recurrent fever were analyzed during the process of patients’ validation performed for the identification of the new Eurofever classification criteria (Gattorno, PRES 2017, in preparation).We compared the pathogenicity classification of the genotype with the final classification reached by the experts. According the geneticists classification each variant was classified in pathogenic (P), likely pathogenic (LP), variant of unknown significance (VOUS), benign (B), likely benign (LB) in a validated or provisional way. Variant for which a consensus was not reached were classified as unsolved.The genotype was considered i)confirmatory, if the patient carries 1 P or LP variant in autosomal dominant (AD) diseases or 2 P/LP variants in autosomal recessive (AR) disease; ii)consistent, in presence of 2 P or LP variants (not phased) or 1 P/LP variant and 1 VOUS or unsolved variant in AR diseases; iii)of uncertain significance, if composed by 1 VOUS variant in AD diseases or 2 VOUS variants in AR diseases.We defined as not confirmatory the genotype of patients carrying 1 or more variants and not included in the previous groups (i.e heterozygous patients for AR diseases) or negative patients for genetic analysis

**Results:** A total of 281 patients reached a consensus during the evaluation by the experts. The majority of patients with consensus obtained the classification as confirmatory or consistent genotype. The most relevant discrepancy observed was the lack of classifications of some heterozygous patients (7 FMF and 2 MKD) for AR diseases in the consensus group. The 2 MKD patients were positive for mevalonic acid in the urine. Only 1FMF, 1 CAPS and 2 TRAPS with uncertain pathogenic variants reached a consensus among the experts (Table 1)

**Conclusion:** Generally a good correlation between the two methods of classification was observed. Nonetheless a limitation of this method might be the lack of classification of a subgroup of patients (heterozygous patients in AR disease) that otherwise reached a consensus on the diagnosis when considering both clinical and genetic data.

**Disclosure of Interest**

None Declared

**Table 1 (abstract P307).** See text for description

	PTS with consensus		PTS with no consensus	
	Pts	Genetic classific.	Pts	Genetic classific.
FMF	36	Conf/cons 27 (75%)	15	Conf/cons 3 (20%)
		Unc. signif 1 (3%)		Not class 2 (13%)
		Not class 1 (3%)		Not confirm 10 (67%) (9 heteroz for P variants)
		Not confirm 7 (19%) (7 heteroz for P variants)		
MKD	56	Conf/cons 50 (96%)	3	Conf/cons 1 (33%)
		Not confirm 2 (4%) (2 heteroz for P variants)		Not confirm 2 (67%) (2/3 heteroz for P variants)
CAPS	32	Conf/cons 27 (84%)	19	Unc. signif 13 (68%) (Q703K,V198M)
		Unc. signif 1 (3%) (V198M)		Not confirm 6 (32%) (genetic NEGATIVE)
		Not class 4 (13%)		
TRAPS	39	Conf/cons 36 (93%)	1 1	Conf/cons 2 (18%)
		Unc. signif 2 (5%) (R92Q,D12E)		Unc. signif 7 (64%) (R92Q,P46L)
		Not class 1 (2%)		Not confirm 1 (9%) (genetic NEGATIVE)
				Not class 1
UNDEF.	81	Conf/cons: 1 for *MVK*	14	Unc. signif: 2 for *TNFRSF1A, 3* for *NLRP3*
		Unc. signif 2 for *MEFV*		Not confirm 1 for *MEFV*
		Not confirm: 9 for *MEFV*, 7 for *NLRP3*, 8 for *TNFRSF1A*		
PFAPA	37	Unc. signif (1 for *NLRP3*, 3 for *TNFRSF1A)*	17	Unc. signif: 1 for *NLRP3*, 1 for *TNFRSF1A*
		Not confirm: 3 for *MEFV*		Not confirm: 2 for *MEFV,* 1 for *MVK*

### P308 LONG–TERM SAFETY OF DIFFERENT DOSES OF CANAKINUMAB (<2, 2–<4 AND 4–<8MG/KG) IN PATIENTS AGED <4 TO 65 YEARS FROM Β-CONFIDENT-REGISTRY

#### Jasmin B. Kuemmerle–Deschner^1^, Ulrich A. Walker^2^, Hugh Tilson^3^, Philip Hawkins^4^, T Van der Poll^5^, Kristina Franke^6^, Antonio Speziale^7^, Eleni Vritzali^7^, Hal H. Hoffman^8^

##### ^1^Pediatrics, University Hospital Tuebingen, Tuebingen, Germany; ^2^Rheumatology, University Hospital Basel, Basel, Switzerland; ^3^University of North Carolina, Chapel Hill, Chapel Hill, USA; ^4^University College London Medical School, London, UK; ^5^Academic Medical Center, University of Amsterdam, Amsterdam, Netherlands; ^6^IQVIA, Durham, USA; ^7^Novartis Pharma AG, Basel, Switzerland; ^8^Rheumatology, Allergy, and Immunology, University of California at San Diego, La Jolla, USA

###### **Correspondence:** Jasmin B. Kuemmerle–Deschner

**Introduction:** Canakinumab, an IgG1 human anti-IL-1 monoclonal antibody, has been shown efficacious and safe in the treatment of patients with all cryopyrin associated periodic syndrome (CAPS) phenotypes^1^. However, no data are available for real-life safety of different doses of canakinumab in CAPS patients of different age groups. Here we analyse the safety of different doses of canakinumab in CAPS and non-CAPS patients from a real-life study (Beta-confident registry).

**Objectives:** To monitor the long-term safety of different CAN doses (<2, 2−<4 and 4−<8mg/kg) across different age groups (<4 to 65 years) in pts with CAPS and other autoinflammatory syndromes.

**Methods:** The β-CONFIDENT Registry was a multicenter, long-term, prospective, observational study conducted at 39 sites across 13 countries. Patients with different CAPS phenotypes and those with other autoinflammatory diseases receiving canakinumab at physician’s discretion were enrolled in the registry. Cumulative safety data were reported as exposure-adjusted incidence rate per 100 pt–years (IR/pyr) from enrollment of the first pt (November 2009) until the end of study (December 2015). Patients were followed up for at least 1 yr. The protocol did not mandate any visits or procedures. All observed and reported adverse events (AEs) and serious AEs (SAEs) were recorded for the following age groups: <4, 4−<12, 12−<18, 18−<65 and ≥65 yrs.

**Results:** Of the 285 pts enrolled, 21% (n=60) discontinued the study mainly due to loss to follow-up (35%, n=21) followed by AEs (10%, n=6), poor efficacy (8%, n=5) and pt preference (3%, n=2). In total, 1114 AEs and 155 SAEs were reported in 223 pts (110.7 IR/100 pyr) and 83 pts (15.4 IR/100 pyr), respectively. Exposure-adjusted incidence rate of AEs (IR/100 pyr) among pts in the <4 and 4 –<12 yr age group, were lowest in pts who received <2 mg/kg (130.3 and 59.7, respectively) compared with pts who received 2–<4 mg/kg (450.8 and 169.6, respectively) and 4 –<8 mg/kg (121.5 and 90.0, respectively) CAN. In pts aged 12 –<18 yrs, IR/100 pyr were lowest in pts who received 2–<4 mg/kg doses (118.2) compared with pts who received <2 mg/kg (169.6) and 4 –<8 mg/kg (139.4) CAN. Similarly, in the 18 –<65 yr age group, IR/100 pyr were lowest in pts who received <2 mg/kg (93.1) compared to pts who received 2–<4 mg/kg (100.7) and 4 –<8 mg/kg (154.4) CAN. In the ≥65 yr age group, IR/100 pyr decreased with increasing dose (<2 mg/kg: 26, 2−<4 mg/kg: 17). Overall, 5, 13, 19, 84 and 7 SAEs were reported in the <4, 4−<12, 12−<8, 18−<65 and ≥65 yr age groups, respectively. One death (metastatic rectal adenocarcinoma in a 76-yr-old Muckle-Wells syndrome patient) was reported.

**Conclusion:** The incidence of adverse events in each dose group increased with age (<4−<65 yrs). No meaningful pattern of AEs was observed, however, with increasing doses. Canakinumab demonstrated a safety profile consistent with previous reports^2,3^, and is well tolerated in CAPS patients aged <4−65 years.

**References**

1. Kuemmerle-Deschner JB, et al. *Ann Rheum Dis.* 2015; 74 (S2): 850. 2. Hoffman HM, et al. *Arthritis Rheumatol*. 2016; 68 (suppl 10). 3. Kuemmerle-Deschner JB*, et al Ann Rheum Dis* 2016;75:617.

**Trial registration identifying number:** NCT01213641

**Disclosure of Interest**

J. Kuemmerle–Deschner Consultant for: Novartis Pharmaceutical Corporation, SOBI, and Baxalta, U. Walker Consultant for: Novartis Pharmaceutical Corporation, H. Tilson Consultant for: Novartis Pharmaceutical Corporation, P. Hawkins: None Declared, T. Van der Poll: None Declared, K. Franke Consultant for: Novartis Pharmaceutical Corporation, A. Speziale Employee of: Novartis Pharma AG, E. Vritzali Employee of: Novartis Pharma AG, H. Hoffman Grant / Research Support from: Burroughs–Wellcome, Consultant for: Novartis Pharmaceutical Corporation, Sobi, Speaker Bureau of: Novartis Pharmaceutical Corporation

### P309 LONG TERM OUTCOMES AND TREATMENT RESPONSE IN PATIENTS WITH TNF RECEPTOR-ASSOCIATED AUTOINFLAMMATORY SYNDROME (TRAPS): RETROSPECTIVE EXPERIENCE FROM A NATIONAL REFERRAL CENTRE.

#### Riccardo Papa^1^, Dorota M. Rowczenio^2^, Charalampia Papadopoulou^3^, Tamer Rezk^2^, Paul Brogan^3^, Taryn Youngstein^2^, Philip N. Hawkins^2^, Helen J. Lachmann^4^

##### ^1^Autoinflammatory Diseases and Immunodeficiencies Centre, Pediatric and Rheumatology Clinic, Giannina Gaslini Institute, University of Genoa, Genoa, Italy; ^2^National Amyloidosis Centre, Division of Medicine, Royal Free Campus, University College London; ^3^Department of Infection, Inflammation and Rheumatology, UCL Great Ormond Street Institute of Child Health; ^4^National Amyloidosis Centre, Division of Medicine, , Royal Free Campus, University College London, London, UK

###### **Correspondence:** Riccardo Papa

**Introduction:** Secondary, AA amyloidosis and infertility are most common and severe complications of Tumour necrosis factor Receptor-Associated Periodic Syndrome (TRAPS) in adults.

**Objectives:** To define the best treatment approach in patients with TRAPS and the effect on long-term outcomes.

**Methods:** We reviewed all data of 100 patients carrying a total of 40 *TNFRSF1A* gene variants who were referred to fever clinic at the National Amyloidosis Centre in London up to January 2018. The Auto Inflammatory Diseases Activity Index (AIDAI) was used to estimate the long-life disease severity.

**Results:** 29 patients had intronic variants of the *TNFRSF1A* gene and displayed milder disease than the 71 patients with mutations affecting coding regions with an AIDAI score <5 (P<0.005), less abdominal pain and skin rashes but more frequent headache or mouth ulcers during fever attacks (P<0.001 and P<0.05, respectively), none developed AA amyloidosis. Amyloidosis affected 13 patients and the strongest association was duration of untreated disease, independent of the AIDAI score. Almost 70% of patients required maintenance therapy. Anti-interleukin (IL) 1β drugs were the most frequently used, in 53 patients, with the highest efficacy rate (86% complete response), while Etanercept was less effective and discontinued in 72% of 25 patients. No patients on anti-IL1β treatment developed amyloidosis and 10 patients with amyloidosis have been successfully treated with anti IL-1 agents with preservation of native renal function in 7 and excellent long term transplant function in 2. Nine women had a history of failure to conceive and seven had successful pregnancies without fertility treatment following complete disease control with anti-IL1β drugs. Long term safety profiles for anti IL-1 agents were excellent even in the presence of comorbidity.

**Conclusion:** Anti-IL1β drugs are the best maintenance treatment in TRAPS with potential to reverse the most serious disease complications of AA amyloidosis and infertility. The diagnosis of TRAPS should be considered very carefully in patients carrying intronic variants of the *TNFRSF1A* gene.

**Disclosure of Interest**

None Declared

### P310 CLINICAL AND HISTOLOGICAL PRESENTATION OF THE SKIN INVOLVEMENT IN BLAU SYNDROME

#### Julie Poline^1^, Olivier Fogel^2^, Christine Pajot^3^, Corinne Miceli^2^, Eric Hachulla^4^, Alain Cantagrel^5^, Carine Wouters^6^, Philippe Brissaud^7^, Arnaud Petit^8^, Guillaume Sarrabay^9^, Caroline Galeotti^1^, Juliette Mazereeuw-Hautier^10^, Antoine Petit^11^, Marie Dominique Vignon-Pennamen^12^, Ulrich Meinzer^13,14^, Emmanuelle Bourrat^11,13^

##### ^1^Department of Paediatric Rheumatology, National Reference Centre for Auto-inflammatory Diseases, Kremlin Bicetre Universitary Hospital, Kremlin Bicetre; ^2^Department of Rheumatology, Cochin Universitary Hospital , Paris; ^3^Department of Paediatric Nephrology and Internal Medicine, Purpan Universitary Hospital, Toulouse; ^4^Department of Internal Medicine, Reference Centre for Rares Systemic and Auto-immunes Diseases, C. Huriez Universitary Hospital, Lille; ^5^Department of Rheumatology, Purpan Universitary Hospital, Toulouse, France; ^6^Department of Paediatric Rheumatology, Leuven Universitary Hospital, Leuven, Belgium; ^7^Department of Rheumatology, X.Bichat Universitary Hospital; ^8^Department of Paediatric Haematology, A. Trousseau Universitary Hospital, Paris; ^9^Department of rares diseases Genetic, A. de Villeneuve Universitary Hospital, Montpellier; ^10^Department of Dermatology, Purpan Universitary Hospital, Toulouse; ^11^Department of Dermatology; ^12^Department of Anatomopathology, Saint Louis Universitary Hospital; ^13^Department of Paediatric Internal Medicine, Rheumatology and Infectious Diseases, R. Debré Universitary Hospital; ^14^INSERM, UMR 1149, Paris, France

###### **Correspondence:** Julie Poline

**Introduction:** Blau syndrome (BS) is a rare monogenic form of sarcoidosis with paediatric onset resulting from mutations in the pattern recognition receptor NOD2. It is phenotypically characterized by the triad of granulomatous polyarthritis, uveitis and skin involvement. Although skin involvement occurs in about 2/3 of cases, only very little is known about its initial clinical presentation and evolution during disease course.

**Objectives:** The aim of this study is to describe skin manifestations of BS.

**Methods:** We retrospectively studied a French national cohort of 20 patients with a genetically confirmed BS. Patients have been identified through a call to SOFREMIP and Dermatology French Society. Epidemiological clinical data have been collected and data about skin involvement (medical record, photographies, biopsies) were analyzed by two independent expert dermatologists. 5 skin biopsies were analyzed by one anatomopathologist.

**Results:** The mean age at diagnosis was 14.3 (0.25–54) years. Skin involvement, present in 15/20 (75%) patients, was early at a median age of 1.5 (0.25-20) years and always inaugural of BS except in one case. Skin lesions have been initially considered as nonspecific dermatitis in 2/15 patients. Skin lesions were mainly (13/15 patients) diffuse erythematous or flesh-colored micropapules, sometimes with livedoid disposition, or nodules (2/15 patients) which coexisted in one patient. All skin biopsies revealed dermic granuloma without caseous necrosis. 9/15 (60%) of patients got a dermatological consultation and 7/15 (47%) a skin biopsy. Average time before diagnosis was 6.3 (-0.8-41) years and 1 (0-3) in case of skin biopsy. Mean age at diagnosis in patients with skin involvement was earlier (10.6 y vs 14.3y) and significantly lower 2.1 (0.25-4.2) years when a skin biopsy has been realized.

**Conclusion:** Skin involvement in BS is often unrecognized or considered as a nonspecific dermatitis. The skin lesions occur in early childhood, are inaugural with stereotypical clinical manifestations and a dermal granulomatous histology. These observations suggest that a better recognition of evocative lesions and a skin biopsy could allow earlier diagnosis and management of patients.

**Disclosure of Interest**

None Declared

### P311 STING-ASSOCIATED VASCULOPATHY WITH ONSET IN INFANCY (SAVI) WITHOUT CUTANEOUS OR ARTICULAR FEATURES

#### William D. Renton^1^, Sarah L. Clarke^1,2^, Athimalaipet V. Ramanan^1^

##### ^1^Department of Paediatric Rheumatology, Bristol Royal Hospital for Children; ^2^MRC Integrative Epidemiology Unit, University of Bristol, Bristol, UK

###### **Correspondence:** William D. Renton

**Introduction:** STING-associated vasculopathy with onset in infancy (SAVI) is a recently described autoinflammatory disease caused by gain-of-function mutations in the TMEM173 gene (1). Most cases are due to de novo mutations; however, familial cases have been reported (2). Manifestations include progressive interstitial lung disease (ILD), polyarthritis and cutaneous changes including acral violaceous plaques and digital ulceration.

**Objectives:** To describe a patient with ILD and a pathogenic TMEM173 mutation without the typical features of cutaneous vasculopathy and arthritis.

**Methods:** The index case presented at 4 months of age with failure to thrive, increasing oxygen requirement, tachypnoea and intermittent fever. Past history was significant for prematurity (28+5 weeks gestation) and chronic lung disease having been discharged home at 3.5 months of age on low flow oxygen.

Progressive deterioration necessitated mechanical ventilation. No infectious agent was identified and antimicrobial therapy failed to alter the disease course. Lung biopsy was non-diagnostic, showing interstitial fibrosis and pneumocyte hyperplasia.

The patient’s mother and maternal grandmother had been affected by severe arthritis and ILD suggesting a familial syndrome. The patient’s mother was diagnosed with rheumatoid factor (RF) positive juvenile idiopathic arthritis (JIA) aged 2. She developed respiratory issues in young childhood and lung biopsy had shown non-specific changes of lymphoid interstitial pneumonia. Her symptoms had been stabilised with regular rituximab having previously failed treatment with methotrexate, mycophenolate and adalimumab. The patient’s maternal grandmother died at the age of 38 having undergone a single lung transplant aged 30. She too had been diagnosed with RF positive JIA and subsequently with rheumatoid arthritis associated pulmonary fibrosis.

The family history and clinical features of ILD, fever, acute phase response, positive antinuclear antibodies and non-response to conventional immunosuppressive therapy raised the possibility of SAVI.

**Results:** Bidirectional Sanger sequencing revealed a heterozygous c.463G>A; p.(Val155Met) mutation in the *TMEM173* gene in the child and mother. Functional assay confirmed significant upregulation of interferon stimulated genes in both patients. Posthumous genetic evaluation of the patient’s grandmother is underway with results awaited.

Treatment with baricitinib led to clinical and laboratory improvement. The patient is thriving six months after starting treatment but remains dependent on home oxygen treatment.

**Conclusion:** SAVI has a varied phenotype and we present a case without the typical cutaneous and articular manifestations. Prompt recognition and treatment is important to prevent irreversible lung disease. This diagnosis should be considered in children with ILD where other more common causes are not apparent; even in the absence of typical cutaneous or joint findings. Accurate identification of rare, inheritable diseases in children can allow for retrospective diagnoses and more precise treatment for family members.

Written informed consent for publication of their clinical details was obtained from the parent of the patient.

**References**

1. Liu Y, Jesus AA, Marrero B, Yang D, Ramsey SE, Montealegre Sanchez GA, Tenbrock K, Wittkowski H, Jones OY, Kuehn HS, Lee CC. Activated STING in a vascular and pulmonary syndrome. New England Journal of Medicine. 2014 Aug 7;371(6):507-18

2. Jeremiah N, Neven B, Gentili M, Callebaut I, Maschalidi S, Stolzenberg MC, Goudin N, Frémond ML, Nitschke P, Molina TJ, Blanche S. Inherited STING-activating mutation underlies a familial inflammatory syndrome with lupus-like manifestations. The Journal of clinical investigation. 2014 Dec 1;124(12):5516-20

**Disclosure of Interest**

None Declared

### P312 ASSOCIATION OF CHRONIC RECURRENT MULTIFOCAL OSTEOMYELITIS (CRMO) AND INFLAMMATORY BOWEL DISEASE (IBD) IN A TERTIARY REFERRAL CENTRE: THE GREAT ORMOND STREET HOSPITAL EXPERIENCE

#### Kulsoom Riaz^1^, Paul Humphries^2^, Daniela Knafelz^1^, Sandrine Compeyrot-Lacassagne^3^

##### ^1^Gsstroenterology; ^2^Radiology; ^3^Rheumatology, GOSH, London, UK

###### **Correspondence:** Kulsoom Riaz

**Introduction:** Recurrent Multifocal Osteomyelitis (CRMO) is an aseptic inflammatory bone disease that typically affects the metaphases of the long bones. It affects children, adolescents and young adults. The main presenting feature is local bone pain and/or swelling. It has a protracted course for years with exacerbations and improvement with treatment. The diagnosis of CRMO can be made on clinical presentation and confirmed by magnetic resonance imaging (MRI) and bone biopsy. On MRI examination there are mostly multifocal, and symmetrical lesions (especially in the metaphysics of tubular long bones, flat bones and spine). CRMO can be associated with skin, gut involvement and other rheumatological conditions.

**Objectives:** We report a single centre experience (GOSH) of the association of CRMO and IBD.

**Methods:** We reviewed reports of whole body MRI scans of 300 children, done in last 5 years between January 2012 and December 2016. Patients with MRI scan results consistent with CRMO were included in the study. Retrospective analysis of electronic clinical records of these patients was done and we recorded their clinical symptoms at presentation, any associated illnesses and Family history. Histopathological/ microbiological findings of bone biopsies were reviewed to rule out haematological, infectious or malignant causes.

**Results:** Twenty three patients were included in the study.Five were male and eighteen female. These children were 8-18 years old with median age of 15 years. The clinical feature of CRMO at first presentation are as under.All patients presented with musculoskeletal symptoms like backache, clavicular involvement, or joint pain.Five patients (21%) presented with abdominal pain and blood in stool. These patients were diagnosed as Inflammatory bowel disease(IBD) on the endoscopic and histopathological findings. Three patients presented with gut symptoms first and later on they developed joint pain and swelling. However one patient presented with joint pain to start with and diagnosed as CRMO. This patient later on developed gut symptoms. One patient presented with simultaneous onset of diarrhoea, blood in stool, abdominal pain and joint pain with swelling.There is significantly more raised inflammatory markers in the IBD/CRMO group than in the CRMO alone groupHistory of trauma was present in 13% of patients who presented with musculoskeletal symptoms.Hyper mobility was present in four patients. Juvenile idiopathic arthritis was an associated diagnosis in three patients. One patient was diagnosed as Enthesitis related arthritis (ERA) and CRMO overlap. Psoriasis, palmoplantar pustulosis , acne, atopic dermatitis, dermatitis artefacta were the skin conditions associated with CRMO but not in IBD/CRMO overlap group.There was family history of connective tissue disorders in six (26%) out of 23 patients, including systemic lupus erythematosus, ankylosing spondylitis, Crohn’s disease, rheumatoid arthritis, antiphospholipid syndrome and psoriasis. Only one patient (4%)out of 23 was HLAB27 positive.

**Conclusion:** CRMO has a varied presentation. We identified that CRMO is associated with IBD in 21% of the patients. Further studies are needed to identify whether the CRMO/IBD overlap group has a separate phenotype.

**Disclosure of Interest**

None Declared

### P313 ASSESSMENT OF COMPLIANCE IN PATIENTS WITH FAMILIAL MEDITERRANEAN FEVER IN RESPECT TO THE DISEASE OUTCOME

#### Vasiliki Sgouropoulou, Maria Trachana, Florence Kanakoudi-Tsakalidou, Evaggelia Farmaki, Polyxeni Pratsidou-Gertsi

##### Pediatric Immunology and Rheumatology Referral Centre, 1st Department of Paediatrics, Hippokration Hospital, Aristotle University, Thessaloniki, Greece

###### **Correspondence:** Vasiliki Sgouropoulou

**Introduction:** Familial Mediterranean Fever (FMF) patients with a poor compliance to follow-up (FU) appointments and treatment may not only be mischaracterized as resistant to colchicine, but potentially may have an unfavorable long-term disease outcome.

**Objectives:** Primary objective: To assess the level of compliance in patients with FMF, in respect to the disease outcome and the development of amyloidosis. Secondary objective: To detect the tolerant patients, who need additional treatment for the disease control.

**Methods:** A retrospective study of all patients with an established diagnosis according to Tel-Hashomer criteria and a minimum FU of 6 months was performed. After 2015, the auto-inflammatory diseases activity index (AIDAI) was given to all patients attending the FU appointments, aiming to the disease self-monitoring. For the purpose of the study, good compliance to the scheduled FU was defined as consistent attendance to all visits (twice yearly). Compliance to treatment was assessed according to the family/patient information given in the FUs. In those with continuous colchicine administration, the presence of clinical or subclinical inflammation was recorded to estimate the disease gravity.

**Results:** Over a 31 year period (1986 – 2017), 71 patients (female: 42) with a median age of 5.66 years at diagnosis, a median lag time to diagnosis 1.83 years, a median disease duration of 15.63 years and a median age of 19.5 years at the final visit were recorded. Their cumulative FU duration was 917.87 years and 12.92 FU years per patient. All patients were receiving colchicine. Totally, the overall compliance to the scheduled FU was good 54.92 % (39/71). Regarding colchicine, 50/71 (70.42%) patients had a good compliance. Among these compliant patients, good response to colchicine was recorded in 47/71 patients (66.19%), partial response in 20/71 (28.16%) and no response in 4/71 (5.63%). Clinical remission was achieved in these 4 recalcitrant patients, with step-up therapy, namely canakinumab (2), methotrexate (1, for sacroiliitis) and infliximab (1, for co-existence of Crohn disease). Regarding the non-compliant patients (21/71, 29.58%), 7 were lost to FUs and 4/21 experienced period crises. The remaining 10/21 patients, finally adhered to treatment and achieved remission. No cases of amyloidosis were observed.

**Conclusion:** Findings of the study revealed an overall non-compliance in less than half of the patients, nevertheless with a declining rate, attributed to an improved patient/family experiential disease education. Of note, clinical remission was accomplished in a high percentage of patients. The detection of the low percentage of the actually non-responders, led to the intensification of the treatment with additional DMARDS, which finally resulted to a favorable disease outcome.

**Disclosure of Interest**

None Declared

### P314 INTERFERON SIGNATURE IN CHILDHOOD RHEUMATIC DISEASES

#### Hafize E. Sönmez^1^, Çağatay Karaaslan^2^, Adriana Almeida de Jesus^3^, Ezgi D. Batu^1^, Banu Anlar^4^, Betül Sözeri^5^, Raphaela Goldbach-Mansky^3^, Seza Özen^1^

##### ^1^Department of Pediatric Rheumatology; ^2^Department of Molecular Biology, Hacettepe University Faculty of Medicine, Ankara, Turkey; ^3^Translational Autoinflammatory Diseases Studies, National Institute of Allergy and Infectious Diseases, Washington , USA; ^4^Department of Pediatric Neurology, Hacettepe University Faculty of Medicine, Ankara; ^5^Department of Pediatric Rheumatology, Umraniye Training and Research Hospital, İstanbul, Turkey

###### **Correspondence:** Hafize E. Sönmez

**Introduction:** Several rheumatic diseases are characterized by overexpression of type I interferon(IFN)-inducible or viral response genes, termed the IFN signature**.** Recently, this signature has been reported in a novel group of monogenic autoinflammatory interferonopathies.

**Objectives:** We aimed to study a previously suggested set of IFN score in patients with suggestive clinical features and develop a clinical score based on the features of patients with a high IFN score and compare to patients with other autoimmune diseases such as systemic lupus erythematous(SLE) and juvenile dermatomyositis(JDM).

**Methods:** We identified 12 patients with a probable interferonopathy based on their clinical features. We compared the IFN signature with 22 controls(8 healthy children, 6 oJIA, 4 SLE, 4 with JDM). The expression of 28 IFN-related genes was quantified from RNA from whole blood using NanoString technology. Summary scores for IFN6/IFN28/ and a z-score for CXCL10(IP10) were calculated(as described elsewhere) Four of the probable interferonopathy patients had mutations related to the IFN pathway genetic analysis on the other are ongoing. Depending on the features of patients with a significantly high IFN signature, we created a preliminary clinical score to identify autoinflammatory interferonopathies based on literature review and expert opinion (Table 1).

**Results:**

The median IFN6, and IFN28, scores were significantly higher in the probable interferonopathy cases as compared to healthy controls and oJIAs patients, but did not differ from SLE and JDM patients. Interestingly, *CXCL10*(IP10) scores were higher in the probable interferonopathy group than the SLE and JDM groups. The mean clinical score was 3(3-5) among these patients. Thus patients with high IFN signature, lacking features of SLE and JDM may be classified to have a autoinflammatory interferonopathy and the suggested clinical score may be helpful to the clinician. Further genetic analysis will enable defining the underlying mutation in the pathway and treatment results on IFN blocking treatments are needed to confirm the pathogenic role of type I IFN in these diseases.

**Conclusion:** We suggest to use this new clinical score for screening patients with undifferentiated inflammatory diseases, and the IFN signature quantified in the 6-gene or 28-gene(preferably) IFN score to identify patients with potential autoinflammatory interferonopathies. The mutation screening for specific mutations is ongoing in these patients.

**Disclosure of Interest**

None Declared

**Table 1 (abstract P314).** See text for description

Clinical preliminary criteria	Presumed IFN mediated AIDs n=12	Heathy controls n=8	Autoimmune diseases (SLE or JDM) n=8
1. Skin manifestations (nodular erythema, violaceous plaques in cold-sensitive acral areas)	12/12	0/8	0/8
2. Vasculopathy (chill-blain like rash, microangiopathic vasculopathy, gangrene/ulcers/infarcts in acral areas)	6/12	0/8	1/8
3. Lipodystrophy	2/12	0/8	0/8
4. Joint manifestations (contractures, non-erosive arthritis)	5/12	0/8	2/8
5. Myositis (patchy)	2/12	0/8	0/8
6. CNS manifestations (basal ganglia calcifications, leukoencephalopathy, white matter disease, L/P lymphocytic findings)	8/12	0/8	0/8
7. Pulmonary involvement (interstitial lung disease, pulmonary fibrosis, pulmonary hypertension	2/12	0/8	1/8
8. Leukopenia/lymphopenia with flares	4/12	0/8	4/8
Median 6-gene IFN score (minimum-maximum)	137.87 (0.251-380.09)	-2.54 (-3.57-2.48	106.26 (-1.47-381.69)
Median) 28-gene IFN score (minimum-maximum)	324.01 (24.66-949.98)	0.83 (-7.91-16.79)	299.9 (2.75-475.96)
CXCL10 z-score (STD)	186.07	46.91	57.59

### P315 HIGH PREVALENCE OF RARE FBLIM1 GENE VARIANTS BUT NO CORRELATION WITH SPECIFIC CLINICAL FEATURES IN AN ITALIAN COHORT OF CNO PATIENTS.

#### Andrea Taddio^1,2^, Adamo P. D'Adamo^1,2^, Giovanna Ferrara^1^, Manuela Pardeo^3^, Marco Cattalini^4^, Martina Finetti^5^, Antonella Meini^4^, Clotilde Alizzi^6^, Gabriele Simonini^7^, Virginia Messia^3^, Serena Pastore^2^, Alberto Tommasini^2^, Rolando Cimaz^7^, Antonella Insalaco^3^, Marco Gattorno^5^

##### ^1^University of Trieste; ^2^Institute for Maternal and Child Health - IRCCS "BURLO GAROFOLO" - , Trieste; ^3^Department of Pediatric Medicine, Division of Rheumatology, Bambino Gesù Children's Hospital, Rome; ^4^Pediatric Clinic, University of Brescia and Spedali Civili of Brescia, Brescia; ^5^UO Pediatria II, Institute "G. Gaslini", Genoa; ^6^Ospedale dei Bambini "G. Di Cristina”, Palermo; ^7^Pediatric Rheumatology Unit, AOU Meyer, University of Florence, Florence, Italy

###### **Correspondence:** Andrea Taddio

**Introduction:** Chronic Non-bacterial Osteomyelitis (CNO) is a rare inflammatory disorder that is characterized by onset of pain, local bone expansion and radiological findings suggestive of osteomyelitis, usually at multiple sites. Although CNO pathogenesis remains still unknown, the hypothesis that CNO might be a genetic disease in the spectrum of autoinflammatory disorders has acquired importance. The strongest evidence comes from the syndromic forms of CNO (Majeed syndrome and Cherubism) and from the presence of chronic inflammatory osteomyelitis in two monogenic diseases caused by mutations of genes involved in the activation of the NLRP3 (pyogenic arthritis, pyoderma gangrenosum and acne: PAPA) and IL-1 beta receptor (deficiency of IL-1 receptor antagonist: DIRA). Although it has been suggested the existence of genes contributing to sporadic CNO, the identification of a gene related to its aetiology has not been identified so far; however it has been recently demonstrated that FBLIM1, a protein involved in the regulation of bone remodeling, could be involved in the pathogenesis of sterile bone inflammation [1]

**Objectives:** To sequence FBLIM1 in a cohort of 83 Italian patients with CNO and correlate the possible results with clinical manifestations.

**Methods:** The coding regions of FBLIM1 were sequenced in a cohort of 83 CNO patients using DNA purified from blood. PCR products were sent for Sanger sequencing. Only rare (global MAF < 2%), coding variants detected were taken into account. Clinical evaluation between patients with rare variants and those without was performed. Fisher’ s exact test was used to compare categorical and ordinal data, and Student’ s t test was used to analyze continuous data.

**Results:** Ten out of 83 patients presented at least one rare coding variant in FBLIM1 gene. One patient was a compound heterozygote (rs114077715/rs146575757), while a patient carried an homozygous mutation (rs540511146). Eight patients presented a heterozygous variants never described before (Table 1). All patients presented classical features of CNO and statistical differences between patients with presence of FBLMI1 and those without genetic mutations were not found in terms of gender prevalence, positive family history, age at onset, number of sites involved, presence of fever, arthritis and skin involvement as well as remission prevalence at the end of follow-up.

**Conclusion:** Our data seem to confirm a possible role of FBLIM1 in the pathogenesis of CNO suggesting that CNO is a disorder of chronic inflammation and imbalanced bone remodeling.

**References**

1. Recessive coding and regulatory mutations in FBLIM1 underlie the pathogenesis of chronic recurrent multifocal osteomyelitis (CRMO).

**Trial registration identifying number:** Our data seem to confirm a possible role of FBLIM1 in the pathogenesis of CNO suggesting that CNO is a disorder of chronic inflammation and imbalanced bone remodeling.

**References**

1. Recessive coding and regulatory mutations in FBLIM1 underlie the pathogenesis of chronic recurrent multifocal osteomyelitis (CRMO).

**Disclosure of Interest**

None Declared

**Table 1 (abstract P315).** CNO patients with rare variants of FBLIM1

Patient	Variant	MAF	Amino Acid change	
2	rs41310367	0.01	c.250+32 C>A	Heterozygote
3	rs41310367	0.01	c.250+32 C>A	Heterozygote
4	rs114077715	0.011	c.931G>A	Heterozygote
6	rs 187479896	0.0008	c.717+14 A>G	Heterozygote
7	rs144567113	0.01	c.718-29 C>T	Heterozygote
8	rs144567113	0.01	c.718-29 C>T	Heterozygote
9	rs144567113	0.01	c.718-29 C>T	Heterozygote
10	rs766409425	0.00002	c.541+13G>A	Heterozygote

## Autoinflammatory diseases II

### P316 COLCHICINE: AN EFFECTIVE TREATMENT OPTION FOR UNCLASSIFIED AUTOINFLAMMATORY DISEASES IN CHILDREN

#### Jasmin B. Kuemmerle-Deschner^1^, Anna L. Schock^1^, Sandra Hansmann^1^, Susanne M. Benseler^2^

##### ^1^Department of Pediatrics, Division of Pediatric Rheumatology, UNIVERSITY HOSPITAL TUEBINGEN, Tuebingen, Germany; ^2^Rheumatology, Department of Pediatrics, Alberta Children's Hospital, University of Calgary, Calgary, Alberta, Canada

###### **Correspondence:** Jasmin B. Kuemmerle-Deschner

**Introduction:** Children and adults with clinically and genetically defined autoinflammatory diseases (AID) including CAPS, TRAPS and HIDS can receive expensive Interleukin-1 (IL-1) inhibitors in many countries around the world. However, patients suffering from unclassified autoinflammatory conditions characterized by recurrent fevers and organ dysfunction and the absence of a known pathogenic mutation commonly have no access to these treatment options.

**Objectives:** The aim of this study was to explore the efficacy and safety of colchicine treatment in children and adults with autoinflammatory diseases without pathogenic mutations.

**Methods:** Consecutive children and adults with autoinflammatory diseases without pathogenic mutations treated with colchicine were included in this single centre study and observed for a median of 12,94 months (range 1,25 – 66,73). Clinical features, autoinflammatory disease activity indices (AIDAI), inflammatory markers ESR, CRP, SAA and S100, frequency and duration of flares and physician global assessment of disease activity (VAS) were recorded serially and compared at baseline and while receiving Colchicine therapy.

**Results:** A total of 39 patients were included in the study. These were 16 girls and 23 boys, median age at start of colchicine therapy was 4 years (range 1 – 54). The diagnoses included PFAPA in 15, mutation-negative FMF in 11, autoinflammation with low-penetrance variants in nine (all *NLRP3)* and other unclassified AID in four patients. Recurrent fever was the leading symptom, mostly associated with arthralgia and myalgia. The mean disease activity decreased from 4.4 at baseline to 2.2 on colchicine. Mean SAA-levels decreased from 159 to 63.3mg/l, CRP levels from 6.4 to 2.3mg/dl. Flare frequency was reduced in 72% and remained unchanged in 28% of patients. Flare duration was reduced in 82%, unchanged in 14% and increased in only 4% of patients. Most common adverse events were abdominal pain and nausea in 50% of patients and appeared to be dose dependent.

**Conclusion:** Children and adults with unclassified autoinflammatory diseases may benefit significantly for colchicine therapy. Control of clinical disease activity and improved inflammatory markers were documented in 59% of patients. Colchicine should be considered in patients with active inflammatory disease with no access to IL-1 inhibitors. Controlled trials are needed to further explore this approach.

**Disclosure of Interest**

None Declared

### P317 NEW VARIANT IN THE IL1RN-GENE ASSOCIATED WITH LATE ONSET AND ATYPICAL PRESENTATION OF DIRA

#### Jasmin B. Kuemmerle-Deschner^1^, Konstanze Hoertnagel^2^, Susanne Schlipf^3^, Sandra Hansmann^1^, Anton Hospach^4^, Susanne M. Benseler^5^, Xiao Liu^6^, Alexander Weber^6^

##### ^1^Department of Pediatrics, Division of Pediatric Rheumatology, UNIVERSITY HOSPITAL TUEBINGEN; ^2^Praxis für Humangenetik, Tuebingen; ^3^Kinderarztpraxis Dr. Lakner, Schwäbisch-Gmünd; ^4^Zentrum für pädiatrische Rheumatologie, Klinikum Stuttgart, Olgahospital, Stuttgart, Germany; ^5^Rheumatology, Department of Paediatrics, Alberta Children's Hospital, University of Calgary, Calgary, Alberta, Canada; ^6^Department of Immunology, University of Tübingen, Tübingen, Germany

###### **Correspondence:** Jasmin B. Kuemmerle-Deschner

**Introduction:** Deficiency of the interleukin-1 receptor antagonist (DIRA) is an autoinflammatory disease characterized by severe systemic inflammation with bone and skin involvement present in the first days of life.

**Objectives:** We report a novel variant in the *IL1RN*-gene associated an atypical phenotype of DIRA.

**Methods:** A 3 year-old Caucasian boy presented with recurrent monthly episodes of fever and fatigue, associated with lymphadenopathy, pericarditis, pleuritis, pancreatitis, and arthritis involving sacroiliac, hip, knee and ankle joints in the absence of any skin involvement. Symptoms had started at age one and had progressed over time to life-threatening episodes requiring intensive care therapy. Throughout, inflammatory parameters including ESR, CRP, SAA, S100A8/9, leukocytes, and platelets were highly elevated. Treatment with colchicine and steroids improved symptoms, however did not prevent flares. Immune deficiencies were ruled out; genetic testing for FMF, CAPS, TRAPS, HIDS and DITRA did not reveal variants in the associated genes.

**Results:** Whole exome sequencing detected a novel homozygous stop variant c.62C>G; p.Ser21* in the *ILRN* gene (NM_173842.2). Mother, father and brother are heterozygous for the same variant. In addition, three variants of unknown significance were identified in the patient's *PCGF5, CPA1* and *SPTA1* genes. Functional studies revealed only marginal secretion of IL-1RA in the patient’s unstimulated leucocytes and after stimulation with IL-1β and LPS, confirming the disease-causing nature of the variant. Subsequently, IL-1 inhibition with anakinra at 2mg/kg/d was started resulting in complete resolution of clinical symptoms, normal inflammatory markers and dramatically improved energy levels. Intolerance to daily subcutaneous injections prompted a switch to canakinumab at 4mg/kg/4 weeks. However patient’s and mother’s assessment of disease activity was inferior on canakinumab compared to anakinra. After four months a flare appeared and lead to return to anakinra.

**Conclusion:** This is the first report of the novel c.62C>G; p.Ser21* variant in the *IL1RN*-gene primarily causing severe serositis in a homozygous carrier, while heterozygous family members were completely symptom free. Skin disease, one of the most prominent features in other patients with DIRA was not observed in this patient, while Il-1 inhibition was likewise effective.

Pathogenic variants in all reported DIRA patients so far affect all 4 isoforms of IL-1RA [1]. The different phenotype in the patient reported here, may be due to the selective loss of secreted IL1RN. Informed consent to publish had been obtained.

1 Aksentijevich et al. N Engl J Med 2009; 360:2426-2437

**Disclosure of Interest**

None Declared

### P318 SAPHO SYNDROME IN ADOLESCENCE: A CASE SERIES

#### Ilaria Maccora, Francesca Tirelli, Edoardo Marrani, Teresa Giani, Grabriele Simonini, Rolando Cimaz

##### Rheumatology Unit, University of Florence, A. Meyer Children Hospital, NEUROFARBA Department, Florence, Italy

###### **Correspondence:** Ilaria Maccora

**Introduction:** SAPHO (Synovitis, Acne, Pustulosis, Hyperostosis and Osteitis) syndrome is a rare disease and sufficient data on its prevalence are unavailable. Usually it occurs in patients with average ages of 30 and 50 years, while is uncommon in pediatric age, where is prevalent among adolescents. Due to its insidious presentation, often it remains unrecognized or misdiagnosed.

**Objectives:** To assess clinical features, laboratory and radiological findings in our patients affected by SAPHO syndrome and to evaluate the response to different therapies.

**Methods:** Clinical, laboratory, radiological data, and treatments were collected from all patients presenting to Meyer Children's Hospital of Florence from 2015 to March 2018 diagnosed with SAPHO syndrome. Informed consent was obtained by the patients and their family.

**Results:** Four patients (2 female and 2 male) with a mean age of 14 years and 9 months (range 140-179 months) were collected. The median time between disease onset and diagnosis was 9,5 months, with a range of 3-22 months. All four patients had osteoarticular symptoms and skin involvement. Two patients had already been diagnosed with Crohn disease (CD) one, and Ulcerative colitis (UC) the other and were both under infliximab treatment at the time of SAPHO diagnosis. Skin involvement in these two cases was characterized by psoriasis vulgaris and palmoplantar pustulosis psoriasis. The other two patients were under isotretinoin therapy for a severe acne. All patients suffered from back pain, 3 of them complained with pain at sternoclavicular joints, 2 at coxofemoral joints, 1 at shoulders and 1 in the costal, tibial and mandibular bones. Two patients displayed a persistent, low fever. Three patients showed increased ESR and the two CRP. Whole body MRI was performed in three patients and it showed signs of osteitis and synovitis, while bone scintigraphy was performed only in one patient and exhibited an increased uptake in affected bone with the typical “bull’s head” appearance in the sternoclavicular region. All patients received NSAID and successively corticosteroid without clinical and radiological response; one was treated with cyclosporine with partial response. All patients finally received an anti-TNFα treatment (3 Adalimumab and 1 Etanercept) with a complete clinical and laboratory recovery in three of them, while one only had a partial improvement because of the brief follow-up. Moreover, two of them received intravenous bisphosphonate (Pamidronate 1mg/kg/day for 3 days) in consideration of a serious involvement of vertebral bodies, but in one of them it was discontinued for side effects.

**Conclusion:** SAPHO syndrome, as we observed in our patients, has a high impact on general health and quality of life. In two cases we evaluated the association with IBDs, and we observed the disease onset under treatment with anti-TNFα. While in the others two cases the disease onset was under isotretinoin. There is a lively debate if these drugs may be considered triggers or cure. Whole body MRI and bone scintigraphy have been useful tools to define the diagnosis, while no patients needed bone biopsy. Anti-TNFα agents have proven to be effective in our patients, and they could be considered as a standard treatment to control this disease.

**Trial registration identifying number:**

**Disclosure of Interest**

None Declared

### P319 ISOLATED PYODERMA GANGRENOSUM AND ADALIMUMAB:CASE REPORT IN PAEDIATRIC AGE

#### Maria Cristina Maggio^1^, Fabio Cardinale^2^, Saveria Sabrina Ragusa^1^, Giuseppe Salvo^1^, Mirella Milioto^3^, Giovanni Corsello^1^

##### ^1^University Department Pro.Sa.M.I. “G. D’Alessandro”, University of Palermo, Palermo; ^2^O.U. of Paediatrics, Azienda Ospedaliero-Universitaria Policlinico di Bari, Children Hospital “Giovanni XXIII", Bari; ^3^O.U. of Paediatrics, Children Hospital “G. Di Cristina”, ARNAS, Palermo, Palermo, Italy

###### **Correspondence:** Maria Cristina Maggio

**Introduction:** Pyoderma Gangrenosum (PG) is a sterile neutrophilic disorder, rarely described in children and adolescents, and frequently it is known as secondary to other chronic inflammatory diseases. However, epidemiological, clinical and therapeutic data on paediatric PG are numerically limited and no randomized controlled trials have been published. Associated diseases in paediatric cases are inflammatory bowel diseases, vasculitis, immune deficiencies, PAPA Syndrome.

The treatment with systemic steroids and cyclosporine is well documented in the literature as the first-line treatment. In non-responders, other treatment lines are indicated, as: corticosteroids and mycophenolate mofetil, mycophenolate mofetil and cyclosporine, tacrolimus, dapsone, infliximab, plasmapheresis. In case reports adalimumab has been used as the treatment for patients who did not respond to other drugs.

**Objectives:** We evaluated the efficacy of adalimumab in PG non-responder to steroids, steroids plus cyclosporine, dapsone plus cyclosporine.

**Methods:** We describe the clinical course of a 16-years-old female affected by PG at the left leg since age 13. Autoimmune tests were negative, as abdominal US with evaluation of the thickness of bowel wall, tTG, AGA, IgA, IgG, IgM, IgE, C3, C4, transaminases, ocular study with slit lamp were negative. She showed HLA DQ8, as the mother affected by Celiac disease. Two biopsies studies confirmed the diagnosis of PG, and the treatment with steroids and antibiotics was started.

However, the disease gone on and cyclosporine was associated to steroids. In a further line of treatment, she was treated with dapsone plus cyclosporine. PG extended to the right leg and worsened with acute lesions. She was treated with topic steroids and antibiotics, with a further extension of the lesions. We proposed to start adalimumab (40 mg s.c. /14 days) and the tapering of topic steroids.

**Results:** Skin injuries progressively improved, and after 3 doses of adalimumab PG is limited to the atrophic lesions in the zones of previous ulcerative lesions.

**Conclusion:** Adalimumab is a safe and promising treatment in isolated paediatric PG and can reduce the use of steroids or other immune suppressive drugs. We highlight the role of accurate diagnostic procedures to primary exclude associated diseases. Informed consent to publish had been obtained from the parents.

**Disclosure of Interest**

None Declared

### P320 CHRONIC NON-INFECTIVE OSTEITIS (CNO) PRESENTING AS A LYTIC SKULL LESION

#### Catriona Anderson^1^, Greg Irwin^2^, Neil Martin^1^

##### ^1^Department of Paediatric Rheumatology, Royal Hospital for Children, Glasgow; ^2^Department of Radiology, Royal Hospital for Children, Glasgow, UK

###### **Correspondence:** Neil Martin

**Introduction:** Chronic Non-Infective Osteitis (CNO), is a rare autoinflammatory condition which results in one or more sterile inflammatory lesions of the bony skeleton. The most common presenting complaints are bony pain and swelling. Clinical course varies and diagnostic delay is common, often resulting in significant pain for those affected. It is characterised by a relapsing and remitting course, and long-term outcome is unclear. It is a diagnosis of exclusion with a wide differential including infection and malignancy. It can affect any part of the skeleton but most often affects the metaphyses of long bones, clavicle, pelvis and vertebrae^1^. It has previously been reported that the skull is almost never involved^2^. We report a rare case of multifocal CNO with a large lytic skull lesion which healed following a course of ibuprofen.

**Objectives:** To describe a rare case of multifocal CNO with a large lytic skull lesion which healed following a course of ibuprofen.

**Methods:** Retrospective case review.

**Results:** A previously well 9 year old girl presented with a one month history of pain and swelling of the forehead, with associated headaches and lethargy. There was no evidence of rash or fever.

Past medical history included two discrete episodes of right foot pain and swelling 10 months previously. X-rays showed expansion and sclerosis of the 2^nd^ metatarsal, with corresponding cortical thickening with oedema on MRI. Her symptoms resolved spontaneously without treatment and she was kept under clinical surveillance.

There was a strong family history of psoriasis affecting her father, maternal aunt and maternal grandmother. There were autoimmune problems including lichen sclerosis, Sjogren’s and thyroid problems on her maternal side.

On examination she had an obvious soft tissue swelling over the bridge of her nose, which was tender on palpation. Vision, eye movements and cranial nerves were intact.

Initial investigations showed mildly raised inflammatory markers (CRP 11 and ESR 45). Further investigations included normal lymphocyte subsets, complement and neutrophil burst suppression test. RF, ANA and ANCA were negative. MRI showed evidence of frontal osteitis with perforation of the external table of the skull vault. CT head with contrast showed the appearance of osteitis of the frontal/nasal bones. She received 7 days of empirical IV antibiotics to cover for infection without improvement. Blood cultures were negative.

High dose Ibuprofen was commenced (10mg/kg QDS) pending biopsy of the lesion. Whole body MRI was performed, which showed high signal lesions in both 2nd metatarsals, left greater trochanter and the sternum.

Endoscopic biopsy showed a non-specific, broadly reactive process, with areas of monocytic inflammation, vascular granulation tissue and neutrophilic inflammation, with giant cells in all areas. There was no evidence of sarcoma or Langerhan’s cell histiocytosis. Bone biopsy culture was negative.

Following extensive MDT discussion, a diagnosis of CNO was made and ibuprofen was commenced as first line treatment. 1 month post-discharge, the forehead lesion was less swollen and headaches less frequent.

3 months after starting ibuprofen CT of her facial bones and sinuses showed significant improvement with healing of the previous frontal and nasal bony destruction. Although she has subsequently reported further pain at the site of her original right second metatarsal lesion.

**Conclusion:** We report an atypical case of CNO with skull involvement in a 9 year old girl. As this is an unusual location for CNO this led to a wide differential diagnosis including infection, LCH and osteosarcoma. Pathological exclusion of these and the presence of multiple other lesions on whole body MRI allowed the diagnosis of CNO to be made. Informed consent to publish had been obtained from the parents.

**Disclosure of Interest**

None Declared

### P321 MODERN CLASSIFICATIONS OF NEUTROPHILIC DISEASE CAN BE APPLIED TO PEDIATRIC PATIENTS

#### Marie Bucchia^1^, Ulrich Meinzer^2,3^, Emmanuelle Bourrat^2,4^, The French Pediatric Neutrophilic Dermatosis Study Group

##### ^1^Centre Hospitalier Le Mans, Le Mans; ^2^Hôpital Robert Debré; ^3^Centre de référence des maladies rhumatologiques et auto-immunes rares de l'enfant (RAISE), Hôpital Robert Debré, APHP; ^4^Centre de référence des maladies rhumatologiques et auto-immunes rares de l'enfant (RAISE), Paris, France

###### **Correspondence:** Ulrich Meinzer

**Introduction:** Pyoderma gangrenosum (PG) and Sweet's syndrome (SS) were initially described independently according to their cutaneous elemental lesions. Based on observations in adult patients it was subsequently shown that PG and SS share many diseases characteristics. This led to the development of the current "modern" classification. In this classification PG is considered as the prototypical deep ND, SS as the prototypical dermal ND and sub-corneal pustular dermatosis as the prototypical superficial ND. Furthermore, association of clinical and histological features of two different types of prototypic ND simultaneously or successively in the same patient were called "atypical" forms ND. The validity of these "modern" classifications systems of ND has not been studied in pediatric patients.

**Objectives:** We conducted a retrospective French cohort study to describe ND and neutrophilic disease occurring in children and to determine age-dependent particularities.

**Methods:** Patients were recruited via the members of the French Society of Pediatric Inflammatory Diseases (SOFREMIP) and the French Society of Pediatric Dermatology (SFDP).

**Results:** Twenty-seven patients diagnosed between 1998 and 2017 with PG or SS were included in this study. Children with SS were younger than children with PG (2.7 months [0.9-7.8] versus 12.4 [3.4-15]; p = 0.03. The number of multiple skin lesions was higher in patients diagnosed with PG than in patients with SS. No differences were found with respect to all other studied From these results we concluded that PG and SS in children share many clinical features.

Typical PG and SS were observed in 11/27 (41%) patients whereas atypical forms were observed in 16/27 (59%) patients. We observed overlapping forms of ND in 3/27 (11%) patients and association of two different ND in 7/27 (26%) patients. No differences with regard to the distribution of typical/atypical forms was found when comparing patients diagnosed with PG versus SS.

Extracutaneous clinical manifestations were observed in 22/27 (81%) children**.**Neutrophilic disease was observed in 17/27 (63%) patients; it was confirmed in 4/27 (15%) patients and probable in 15/27 (55%) patients. The presence of neutrophilic disease was similar in patients diagnosed with PG when compared to patients diagnosed with SS, though there was a trend (p=0.01) to more neutrophilic disease in patients diagnosed with SS. The frequency of neutrophilic disease was comparable in infants (8/10;80%), young children (2/5;40%), and adolescents (7/12;58%). The location of extra-cutaneous of neutrophilic disease was similar in infants, young children and adolescents. However, neutrophilic disease of the upper respiratory tract, as well as cardiac neutrophilic disease were specific to infants.

An association to another disease was observed in 19/27 (70,4%) patients. The proportion of patients with associated diseases was similar in patients diagnosed with PG and patients diagnosed with SS. In infants and young children, a large spectrum of diseases was associated to ND. In adolescents only IBD and Behçet's disease were associated to ND, with IBD being by far the most frequent associated disease (9/10 patients).

**Conclusion:** Pediatric patients diagnosed with PG and SS share many clinical features. Atypical forms of ND, as well as overlap and simultaneous occurrence of different types of ND can be observed in children, especially in infants. Finally, ND can be associated to a broad spectrum of diseases in young children. Our study suggests that modern classification systems of ND from adults can be applied to children and that ND occurring in infants requires careful diagnostic follow up and clinical management.

**Disclosure of Interest**

None Declared

### P322 AN ATYPICAL CASE OF STING-ASSOCIATED VASCULOPATHY WITH ONSET IN INFANCY (SAVI)

#### Virginia Messia, Manuela Pardeo, Camilla Celani, Gian Marco Moneta, Ivan Caiello, Giulia Marucci, Claudia Bracaglia, Fabrizio De Benedetti, Antonella Insalaco

##### ^1^Rheumatology, Bambino Gesu' Children Hospital, Rome, Italy

###### **Correspondence:** Virginia Messia

**Introduction:** STING-associated vasculopathy with onset in infancy (SAVI) is a newly described type I interferonopathy, caused by autosomal dominant gain-of-function mutations in Transmembrane Protein 173 *(TMEM173)* gene. It is characterized by early-onset systemic inflammation with increased levels of acute phase reactants, a severe cutaneous vasculopathy, leading to extensive tissue loss, and a severe interstitial lung disease (ILD).

**Objectives:** to describe an atypical clinical phenotype.

**Methods:** A 4 year-old young girl presented since the first months of life with recurrent fevers, arthralgia, persistent cough and failure to thrive (below the 5th percentiles). She never showed skin rash. At the age of 3, she was admitted to our hospital for persistent fever and arthritis of left knee and right ankle; she started ibuprofen without any benefit. Laboratory tests showed persistently elevated CRP and serum amyloid A levels, persistent microcytic anemia and increased IgG levels, highly positive antinuclear antibody (title 1:640, homogeneous pattern) with positive p-ANCA. Immunological and cytogenetic studies performed on bone marrow were normal; both sweat test and genetic test for cystic fibrosis were negative. She started low-dose glucocorticoids with incomplete response, persistent cough and polyarthritis in small joints. Chest radiograph and CT showed features of severe interstitial lung disease (ILD) with bronchiectasis and small nodules. Extensive investigations for bacteria, virus and fungi and cytological tests on blood and bronchoalveolar lavage were negative. A lung biopsy showed desquamative interstitial pneumonia. So, she started high-dose methylprednisolone, subcutaneously methotrexate and rituximab. Whole blood of the patient and healthy donors (HD) (n = 10) were collected into PAXgene tubes. The type I IFN signature was defined by the expression levels of six IFN-regulated genes (IFI27, IFI44L, IFIT1, ISG15, RSAD2, SIGLEC1), measured by quantitative polymerase chain reaction (qPCR). As previously described by Rice GI, the median fold change of each IFN-induced gene, when compared with the median of the combined HD, was used to calculate the type I IFN score. The mean type IFN score of the HD plus two SD was calculated; we considered as positive a type IFN score higher of 1,42.

**Results:** Because of a persistent inflammatory state, ILD and polyarthritis, an autoinflammatory syndrome was considered and, in particular, Copa syndrome was suspected; however, sequencing of *coatomer associated protein subunit alpha (COPα)* gene showed no mutations. Molecular analysis of the *TMEM173* gene by NGS showed the presence of c.463G>A (p.Val155Met) variant in heterozygotic status. Whole-blood gene expression studies demonstrated increased IFN signature (182,2), well in the range for those reported in the literature in patients with SAVI and other interferonopathies. Elevated levels of CXCL-10 (>1000 pg/ml) were also found.

**Conclusion:** The clinical picture of SAVI syndrome is characterized by early-onset (before 8 weeks of age) cutaneous vasculitis, recurrent fevers, ILD, and systemic inflammation. Our patient never developed skin rash unlike the cases described in literature. Therefore, lack of typical skin lesions should not exclude this clinical suspicion. The respiratory and systemic inflammatory component of the disease may predominate. In the context of early age of onset, ILD, failure to thrive and recurrent fevers, SAVI must be considered as a differential diagnosis. A better understanding of the genotype–phenotype correlation is needed to improve SAVI management and prognosis.

**Disclosure of Interest**

None Declared

### P323 OVER TEN YEAR OUTCOME OF A COHORT OF 9 PEDIATRIC PATIENTS WITH CHRONIC NON-INFECTIOUS OSTEITIS (CNO) TREATED WITH INTRAVENOUS PAMIDRONATE

#### Paivi M. Miettunen^1^, Chloe M. Stephenson^1^, Alexandra Rice^1^, Alberto Nettel-Aguirre^1^, Xingchang Wei^2^

##### ^1^Paediatrics; ^2^Diagnostic Imaging, UNIVERSITY OF CALGARY, Calgary, Canada

###### **Correspondence:** Paivi M. Miettunen

**Introduction:** Chronic non-infectious osteitis (CNO) predominantly affects patients < 18 years of age. We have previously described short-term outcome of consecutive pediatric CNO patients treated with intravenous pamidronate (IV-PAM).

**Objectives:** We now present the long-term clinical, magnetic resonance imaging (MRI), and bone resorption data in this same cohort.

**Methods:** Patients: All consecutive pediatric CNO patients initially treated with IV-PAM between 2003-2008 at a single tertiary pediatric centre were prospectively followed until 2017.

Intravenous Pamidronate (IV-PAM) dosing: 0.5 mg/kg (maximum 30 mg) for the first dose and 1 mg/kg (maximum 60 mg) for all subsequent doses (maximum annual dose 11.5 mg/kg). IV-PAM was administered either as 1 day/month x 9, or as 3-day cycles every 1-3 months if the patient lived far away. Whole body MRI (WBMRI) was performed at baseline, at pain resolution and at 6 and 12 months during the first year, and also at time of suspected flare. In case of WBMRI-confirmed flare unresponsive to non-steroidal anti-inflammatory agents (NSAIDs), the patient received a repeat treatment with IV-PAM (discontinued when pain resolved with follow-up MRI at 6 months).

Visual analog scale for pain (VAS, 0=no pain; 10=maximum pain) and bone resorption marker urine N-telopeptide/urine creatinine (uNTX/uCr) were measured at baseline, preceding each IV-PAM, at 12 months, and also at time of WBMRI-confirmed CNO flare. Each patient’s uNTX/uCr level was compared to age and sex specific norms.

**Results: Patient cohort:** Nine patients (5 F: 4 M) were treated initially between 2003-2008, with a median (range) age at treatment of 12.9 (4.5-16.3) years, and median (range) duration of symptoms of 18 (6-36) months. All patients had multifocal disease: 2/9 with spinal disease, one of which presented with several vertebral compression fractures at baseline.

**Initial response:** VAS decreased from 10/10 to 0-3/10 by the end of first 3-day treatment for all patients. The mean (range) time to complete MRI resolution of bone inflammation was 6.0 (2-12) months. The mean [confidence interval (CI)] baseline uNTX/uCr was 738.83 (CI 464.25, 1013.42) nmol/mmol/creatinine with all uNTX/uCr levels decreasing below age-and sex specific normal levels after the first IV-PAM.

**Long-term follow-up:** Median (range) of follow-up was 13 (11-14) years: 5/9 patients had MRI confirmed CNO recurrence, 4 patients flared within 12-18 months after 1^st^ IV-PAM dose and responded to one-day IV-PAM, 1 patient flared after 72 months and responded to 3 once-monthly 1-day IV-PAM, and 2 patients had a second flare at 18 months following the second IV-PAM treatment, which responded to NSAIDS. The VAS ranged from 3-6/10 with each flare. No patients flared while their uNTX/uCr was suppressed below age and sex appropriate levels. No spinal CNO patient developed new vertebral fractures during follow-up: 1 patient developed chronic back pain despite no active disease on MRI and no compression fractures. Two patients developed arthritis 10 and 12 years after CNO diagnosis. At final follow-up, 7/9 patients were on no medications and 2/9 required NSAIDS and/or sulfasalazine for arthritis only with inactive CNO. No patient developed osteonecrosis of the jaw.

**Conclusion:** Pamidronate resulted in initial resolution of pain and MRI documented inflammation in all patients; with equal efficacy with disease flares. No new spinal compression fractures nor new spinal lesions developed after IV-PAM was initiated. No patient flared while his/her uNTX/uCr remained suppressed. We propose that IV-PAM is an effective and safe second-line therapy in difficult to treat CNO. We recommend a MRI documentation of suspected flare, as non-inflammatory pain can occur.

**Disclosure of Interest**

None Declared

### P324 HAPLOINSUFFICIENCY OF THE A20: A DIAGNOSTIC CHALLENGE

#### Lucía Lacruz Pérez^1^, Concepcion Mir Perello^1^, Ana Perrela Artero^2^, Laura Ventura Espejo^2^, Rociio Casado Picón^3^, Natalia Martínez Pomar^4^

##### ^1^Pediatric Rheumatology Unit. Pediatric Department; ^2^Pediatric Department; ^3^Son Espases Universitary Hospital., Mallorca, Spain; ^4^Immunology Department, Son Espases Universitary Hospital., Mallorca, Spain

###### **Correspondence:** Concepcion Mir Perello

**Introduction:** Autoinflammatory diseases have revolutionized the concept of classical rheumatic disease. Most of them are produced by a “novo” mutation (only 10% have family history of disease). Haploinsufficiency of A20 was described in 2016, it has dominant inheritance and it is caused by mutations in the TNFAIP3 gene. A20 protein is an inhibitor of transcription factor NF-kB signaling pathway, which is key to inflammatory responses. This new autoinflammatory disease presents high penetration and patients present clinical symptoms at early age with similar phenotypes to Behçet's disease.

**Objectives:** We present a clinical case diagnosed of Haploinsufficiency of A20 with a mutation not described in the literature.

**Methods:** 7-year-old boy with histamine intolerance and non-IgE-mediated food allergies. At the age of 4, pain begins in lower limbs, reason why he attended a Paediatric Rheumatologist. He associated perineal irritation and frequent conjunctivitis, as well as episodes of oral aphthae and recurrent headaches. In addition, he presented abdominal pain, diarrhea, genital aphthae, 3 day self-limiting febrile episodes, asthenia, arthralgia, increased irritability and aggressiveness. Prior to diagnosis, it was considered as an autoinflammatory process and treated with colchicine with partial response. He precised dose increase and association of subcutaneous methotrexate. Among others, Behçet's disease and familial Mediterranean fever were considered as differential diagnosis. He presented normal serum levels of immunoglobulins and complement, negative antinuclear and anticardiolipin antibodies and negative HLA-B51. No mutations in MEFV gene on genetic studies. Analysis of TNFAIP3 gene detected heterozygosis the mutation p.W356R located in exon 7, that confirmed diagnosis of haploinsufficiency of A20.

Throughout family history, we found years of evolution of similar symptoms in a sister, his mother and grandmother. Genetic study showed that the mutation p.W356R is segregated with disease because it was only detected in the three affected relatives.

**Results:** We report an haploinsufficiency of A20 case with a new mutation p.W365R, which not described in literature or registered in the database of genome diversity Exome Aggregation Consortium (EXAC). In silic studies of functional damage of the mutation with SIFT applications show a deleterious effect and with Polyphen applications a possible damage. We emphasize that family study was carried out in 3 generations and shows that the mutation is segregated with the autoinflammatory disease.

**Conclusion:** It is important to consider autoinflammatory diseases in differential diagnosis when recurrent symptoms of this type appear, since only its suspicion will enable adequate diagnosis and treatment. Informed consent to publish had been obtained.

**Disclosure of Interest**

None Declared

### P325 DIAGNOSTIC RATE OF AUTOINFLAMMATORY DISEASES EVALUATED BY FEVER PATTERN IN CHILDHOOD AND ADULT ONSET PATIENTS

#### Takako Miyamae^1^, Manabu Kawamoto^1^, Yumi Tani^1^, Aki Hanaya^2^, Takayuki Kishi^2^, Yasushi Kawaguchi^1^, Hisashi Yamanaka^1^

##### ^1^Institute of Rheumatology, Tokyo Women's Medical University; ^2^Department of Pediatrics, Tokyo Women's Medical University, Tokyo, Japan

###### **Correspondence:** Takako Miyamae

**Introduction:** The diagnosis of autoinflammatory diseases (AID) relies on careful interpretation of the clinical phenotype and results from molecular genetic analysis.

**Objectives:** To investigate diagnostic rate of AID by fever pattern in two onset-age groups, childhood and adult onset patients.

**Methods:** Final diagnosis of patients suspected to have AID in the two groups with polymorphisms of AID responsible genes analyzed from 2005 to 2016 in our institute was evaluated by fever pattern with information of clinical manifestations and course. Genomic DNA was isolated from the patients’ peripheral blood and a polymerase chain reaction (PCR) was used to amplify the indicated exons of 10 genes [MEFV (exons 1-10), TNFRSF1A (exons 2-4), MVK (exons 9-11), NLRP3 (exon 3-6), NOD2 (exon 4), LI1RN (exons 2-4), IL36RN (exons 2-5), PSMB8 (exons 2, 3, and 5), NALP12 (exons 3 and 9), PSTPIP1 (exons 10 and 11), TNFAIP3, and NLRC4.] After cleaning the PCR products, cycle sequencing was carried out using the Big Dye® Terminator v3.1 kit and analyzed with an ABI 3130xl Prism Genetic Analyzer. Genetic polymorphisms within above genes were examined.

**Results:** All 227 individuals(140 childhood-onset: 0.3~15 (median 5.0) years and 87 adult onset: 16~64 (median 30.5) years of onset age)were classified into following four subgroups: 1. Periodic fever (n=72 and 27 in childhood and adult onset, respectively), 2. Recurrent fever lacking of regular period (n=47 and 41), 3. Persistent fever (n=12 and 11), 4. No fever, but with probable manifestations of AID (n=9 and 8). Diagnostic rate of AID was highest in Subgroups 1 (69.4% and 40.1% in childhood and adult onset, respectively), followed by Subgroup2(29.8% and 17.1%)including PFAPA (n=34 and 1) , FMF (n=20 and 15), CAPS (n=6 and 1), TRAPS (n=3 and 1) in the two subgroups. No one was diagnosed as AID in Subgroup 3 and 4 except 4 adult onset cases with hydroarthopathy diagnosed as FMF.

**Table:** 

	Childhood-onset		Adult onset	
	n=140 (M:F=79:61)		n=87 (M:F=27:60)	
	n	Diagnosis of AID	n	Diagnosis of AID
1.Periodic fever	72 (51.4%)	50 (69.4%)	27 (31.4%)	11 (40.1%)
2.Recurrent fever lacking of regular period	47 (33.6%)	14 (29.8%)	41 (47.1%)	7 (17.1%)
3.Persistent fever	12 (8.6%)	0	11 (12.6%)	0
4.No fever, but with probable manifestations of AID	9 (6.3%)	0	8 (9.2%)	4 (50%)
Total	140	64 (45.7%)	87	22 (25.3%)

**Conclusion: Conclusion:** AID was identified higher in childhood-onset patients compared with adult-onset. In both onset age groups, AID was mostly identified in patients with periodic fever and never diagnosed in patients with persistent fever. Fever pattern is one of useful factors to estimate probability of AID.

**Disclosure of Interest**

None Declared

### P326 Withdrawn

### P327 CLINICAL ASSESSMENT OF PFAPA SYNDROME PATIENTS IN A TERTIARY PEDIATRIC HOSPITAL

#### Juan Manuel Mosquera Angarita^1^, Emma Fortes Marin^2^, Andrea Zacarías Crovato^1^, Estíbaliz Iglesias Jiménez^1^, Violeta Bittermann^1^, Rosa Bou-Torrent^1^, Joan Calzada-Hernández^1^, Judith Sánchez-Manubens^1^, Jordi Antón López^1^

##### ^1^Pediatrics, Hospital Sant Joan de Déu, Esplugues de Llobregat, Barcelona; ^2^Universitat de Barcelona, Barcelona, Spain

###### **Correspondence:** Juan Manuel Mosquera Angarita

**Introduction:** PFAPA syndrome is characterized by periodic fever, aphthous stomatitis, pharyngitis and adenitis and is

the most prevalent autoinflamatory disease.

PFAPA syndrome is characterized by periodic fever, aphthous stomatitis, pharyngitis and adenitis and is the most prevalent autoinflamatory disease.

**Objectives:** To describe the initial presentation, clinical course and therapeutic response of PFAPA and PFAPA-like children visited in the HSJD, and to assess if there are differences between both groups and to compare it with the clinical manifestations of PFAPA described in the published studies.

**Methods:** Children with clinical manifestations of PFAPA or PFAPA-like visited from January 2004 to December 2015 were enrolled. Data were gathered from medical records.

**Results:** 85 patients with PFAPA syndrome evaluated. Onset of symptoms was 27.6 months and the symptomatology stopped at 7.2 years. In 24.1% of the patients a positive familiar history of periodic fever of unknown origin was reported. At disease onset the episodes of fever have a regular recurrence every 3- 6 weeks. Pharyngitis (85.9%), cervical adenitis (70.6%) and aphthous stomatitis (60%) were frequently described with the fever. Other associated symptoms were abdominal pain, headache, arthralgia, myalgia, rash and diarrhea. Clinical symptoms of PFAPA-like group were similar unless age of onset and end, 6 years and 9,5 years, respectively. The fever decreased in severity, duration and frequency with corticosteroids in both groups. A single dose of prednisone (1 mg/kg/day) was enough for clinical abortion in most patients. Tonsillectomy was a successful option in the prevention of recurrences in selected patients.

**Conclusion:** There are no important clinical differences between our PFAPA patients and the published PFAPA data. PFAPA-like group have similar clinical manifestations compared to PFAPA, except the onset and the end ages.

**Disclosure of Interest**

None Declared

### P328 SPANISH REGISTRY OF AUTOINFLAMMATORY DISEASES

#### Rosa M. Alcobendas^1^, Jordi Anton^2^, Daniel Clemente^3^, Alina Boteanu^4^, Marisol Camacho^5^, Jaime de Inocencio^6^, Rocio Galindo^7^, Enriqueta Peiró^8^, Maria Jose Lorente^9^, Cristina Zarallo^10^, Sara Guillen^11^, Marta Medrano^12^, Rafael Diaz-Delgado^13^, Inmaculada Calvo^14^, Sara Murias^1^, Juantxo Arostegui^15^

##### ^1^Pediatric Rheumatology, university hospital La Paz, Madrid; ^2^Pediatric Rheumatology, Hospital Sant Joan de Deu, Barcelona; ^3^Pediatric Rheumatology, Hospital Niño Jesus; ^4^Pediatric Rheumatology, Hospital Ramon y Cajal, Madrid; ^5^Pediatric Rheumatology, Hospital Virgen del Rocío, Sevilla; ^6^Pediatric Rheumatology, Hospital Doce de Octubre, Madrid; ^7^Pediatric Rheumatology, Hospital Regional de Málaga, Málaga; ^8^Pediatric Rheumatology, Hospital Marques de Valdecilla, Santander; ^9^Pediatric Rheumatology, Hospital Virgen de Arrixaca, Murcia; ^10^Pediatric Rheumatology, Hospital Infanta Cristina, Badajoz; ^11^Pediatric Rheumatology, Hospital de Getafe, Madrid; ^12^Pediatric Rheumatology, Hospital Miguel Servet, Zaragoza; ^13^Pediatric Rheumatology, Hospital Severo Ochoa, Madrid; ^14^Pediatric Rheumatology, Hospital La Fe, Valencia; ^15^Genetics, Hospital Clinic, Barcelona, Spain

###### **Correspondence:** Sara Murias

**Introduction:** Autoinflammatory diseases are a group of diseases characterized by inflammatory attacks in the absence of infectious, neoplastic or autoimmune etiology. In many of them mutations in single genes have been identified being called monogenic or hereditary periodic fever syndromes Since 1999, when this term was proposed by Don Kastner for the first time, new entities previously unknown are progressively being described.

**Objectives:** Principal: To review the demographic data, clinical characteristics and response to treatment of all patients included in the Spanish registry of autoinflammatory diseases (SpRAD). Secondary: To identify patients not currently included in the Eurofever registry and enroll them.

**Methods:** Retrospective cross-sectional study was performed. The study was approved by the ethics committees of all hospitals and, accordingly, the participants had to sign an informed consent prior to inclusion in the registry.

**Results:** 287 patients from 14 centres in 9 different Spanish regions were identified, 144 were males (50%). Genetic study had been performed in 144 individuals (50%), and 18 inconclusive mutations belonging to 14 patients were found. 106 had a clinical diagnosis of monogenic disease, being the most frequent: familiar Mediterranean fever (FMF) (40%), hyper Ig D syndrome (HIDS) (25%), cryopyrinopathies (11%) and tumor necrosis factor receptor-associated periodic syndrome (TRAPS) (8.5%). As expected, the most common identified mutation among TRAPS patients was p.R92Q (67%). Three patients diagnosed with deficiency of adenosine deaminase 2 (DADA2) were included, all of them with symptoms resembling polyarteritis nodosa. The rest of cohort, 180 patients, were classified as suspected polygenic diseases, including systemic-onset juvenile idiophatic arthritis (34%), periodic fever, aphtous stomatitis, pharyngitis and adenopathy syndrome (PFAPA) (23%), chronic multifocal recurrent osteomyelitis (CMRO) (25.5%), Behçet syndrome (12%) and nonspecific recurrent fever (5.5%). Globally, the most frequent clinical manifestations at onset were fever, cutaneous symptoms, arthritis or arthralgia, lymphadenopathies and abdominalgia. One patient, who was finally diagnosed with chronic infantile neurological, cutaneous, and articular (CINCA) syndrome, showed deafness. Two HIDS patients presented advanced kidney failure due to amyloidosis. 118 patients were still on treatment at the time of the study cut: corticosteroids (46.5%), colchicine (40%), anti IL1 (43%), anti IL6 (23%), anti TNF (23%) and JAK inhibitor in one patient.

**Conclusion:** Autoinflammatory diseases are infrequent diseases. For this reason, registries are necessary in order to gather as many patients as possible, with the aim of improving these diseases description, avoiding misdiagnosis and developing therapy strategies.

**Disclosure of Interest**

None Declared

### P329 ISG15 DEFICIENCY PRESENTING WITH AUTOINFLAMMATORY SYMPTOMS INVOLVING BRAIN AND SKIN

#### Sara Murias^1^, Maria Bravo^2^, Marta Feito^3^, Luz Y. Bravo^2^, Montserrat Bret^4^, Clara Udaondo^1^, Rosa M. Alcobendas^1^, Agustin Remesal^1^, Eduardo Lopez-Granados^2^, Raul De Lucas^3^

##### ^1^Pediatric Rheumatology; ^2^Immunology; ^3^Pediatric Dermatology; ^4^Pediatric Radiology, University Hospital La Paz, Madrid, Spain

###### **Correspondence:** Sara Murias

**Introduction:** ISG15 is a type I interferon (IFN)-inducible gene, encoding an ubiquitin-like protein which is an effector of IFN-α/β- dependent antiviral immunity in mice. Previous reports, based on only 6 reported patients, have suggested that this deficiency does not lead to increased viral infections in humans, but do promote autoinflammation.

**Objectives:** We present a 6 year old girl, recently diagnosed with this rare disease. We describe the striking presentation of this case, its diagnostic/therapeutic approaches, and clinical outcome.

**Methods:** Medical chart was reviewed.

**Results:** At the age of six months, the patient showed spontaneous and severe deep and necrotizing ulcerations involving groins, genital area and proximal thighs; with associated fever, increased acute phase reactants, and strabismus corresponding to sequelae of choriorretinitis. After exhaustive study, no infectious/traumatic etiology was found. The biopsy showed necrosis of the epidermis, dermis and upper subcutis and vascular thrombosis in one margin. Despite parenteral antibiotics, surgical debridement and vacuum-assisted wound closure therapy, healing was achieved only after oral steroids treatment. Firstly considered an early onset Behçet disease, etanercept was started with incomplete response, since systemic flares still appeared albeit milder. IL-1 blockade was initiated (anakinra) with persistence of systemic symptoms, and was thus withdrawn. Adalimumab was subsequently started and had to be retired, due to a rapid development of anti-adalimumab antibodies at high levels. The patient had recurrent systemic inflammation flares, becoming steroid-dependent. At the age of 4, a deep and severe necrosis followed by ulceration appeared on her neck, which led to hospital admission for metilprednisolone intravenous pulses. Finally, IL-6 blockade was started with quick and complete response of systemic symptoms. Nevertheless, mild skin flares have appeared twice, being controlled with short courses of oral steroids. In September 2017, a broader genetic study by next generation sequencing (NGS) panel was performed, and two heterozygous mutations affecting ISG15 gene were discovered. After literature review involving 6 patients, skin lesions were interpreted as affecting skin areas specifically above lymphadenopathies. Moreover, the basal ganglia brain calcifications that some of the previously reported patients exhibited led to perform brain CT scan, which demonstrated the presence of striking calcifications. At present, the patient is still on tocilizumab, her growth is normal, and has not presented serious infections.

**Conclusion:** Data reported so far show that ISG15 deficiency behaves as an autoinflammatory disease but also as an immunodeficiency, due to its involvement in type-I and type-II IFN regulation pathways. Thus, affected individuals exhibit higher IFNα and lower IFNγ responses in comparison with healthy donors. This condition upholds the growing evidence supporting the existence of a “continuum” between autoimmune/autoinflammatory diseases and immunodeficiency conditions. IL-6 blockade has been effective in this single patient, being to our knowledge the only ISG15 deficiency case who has been reported as respondent to tocilizumab.

This patient’s parents have given their consent for presenting the case to this congress and for further publication.

**Disclosure of Interest**

None Declared

### P330 SUCCESSFUL TREATMENT WITH CANAKINUMAB IN AN ADOLESCENT PATIENT WITH A VERY EARLY ONSET OF TOPHACEOUS GOUT

#### Maria I. Kaleda^1^, Irina P. Nikishina^1^, Alina V. Kharlamova^2^, Nurali Z. Zokirov^3^

##### ^1^Pediatrics, V. A. Nasonova Research Institute of Rheumatology, Moscow; ^2^Pediatrics, CDC “Zdorovie”, Rostov-on-Don; ^3^Pediatrics, Central Children's Hospital of Federal Medical Biological Agency of Russia, Moscow, Russian Federation

###### **Correspondence:** Irina P. Nikishina

**Introduction:** Childhood onset gouty tophus is a very rare phenomenon. The conventional pharmacotherapy using for adequate hyperuricemia control and acute gouty arthritis treatment are limited in cases of severe tubulointersitial nephritis. Our experience of single injection of Canakinumab in adolescent patient seems to be very successful. Such experience has not been described earlier in available literature.

**Objectives:** Presentation of our experience with Canakinumab therapy in an adolescent patient with an early onset severe tophaceous gout and severe chronic kidney disease.

**Methods:** Case report.

**Results:** A 13 yo male patient with normal physical, psychosocial and cognitive development presented with acute onset arthritis in the right elbow (intensive pain, swelling, hyperemia and local hyperthermia) without potential triggering factors in November 2015. Because of the provisional diagnosis of acute hematogenous osteomyelitis the patient was hospitalized for surgical procedure (osteoperforation of right humerus) and antibacterial therapy. For the next several months he suffered from multiple episodes of acute arthritis in metatarsophalangeal joint of the left toe, left ankle joint, left elbow joint, interphalangeal joints of 3 and 5 fingers on the right hand. Diagnosis JRA, polyarthritis was established in April 2016. The patient was treated by NSAIDs (i/m and per os) and sulfasalazine without any effect. Joints’ deformities and numerous tophies developed very rapidly. The diagnosis of tophaceous gout was suspected in June 2017. The highest documented serum level of uric acid was 990.0 μmol/L. Allopurinol therapy 200 mg/day started. By October 2017 lab findings were as follows: serum creatinine 152 μmol/L, urea 8.1 mMol/L, uric acid 397.1 μmol/L. *Schwartz* estimate glomerular filtration rate (GFR) was 63.2 ml/min, diurnal proteinuria -0.1 g/day. Ultrasound and scintigraphic findings confirmed chronic urate tubulointerstitial nephritis. Imaging modalities (US, radiography, MRI) identified severe bones destructions typical for chronic tophaceous gout. Acute arthritis in the right ankle with significant intra-articular effusion and inflammatory changes in the synovial fluid and formation of crystals of monurate sodium broke out in November 2017. Molecular-genetic testing confirmed the syndrome of Kelley-Seegmiller (the hemizygotic mutation in the gene *HPRT1*). A single 150 mg dose of Canakinumab was injected in order to gain some control over disease activity with excellent result. Its sustainable effect provided the possibility of urate-lowering therapy. Febuxostat at 80 mg/day was administered instead of allopurinol. The patient didn’t have attacks of acute arthritis during 4 next months. Lab findings in April 2018 were as follows: serum level of uric acid – 297 μmol/L, creatinine - 161.0 μmol/L.

**Conclusion:** Canakinumab injection provided rapid reverse of gouty arthritis acute attack, and subsequent achievement of target serum uric acid levels after administration of febuxostat in an adolescent patient with severe tophaceous gout and chronic urate tubulointerstitial nephritis, which limited the allopurinol, NSAIDs and colchicine using. Informed consent to publish had been obtained from the parent.

**Disclosure of Interest**

None Declared

### P331 BEYOND NSAIDS : SECOND-LINE THERAPEUTIC AGENTS FOR CHRONIC RECURRENT MULTIFOCAL OSTEOMYELITIS

#### Daire O'Leary^1,2,3^, Orla G. Killeen^1,3^, Emma Jane MacDermott^1^, Anthony G. Wilson^3^

##### ^1^National Centre for Paediatric Rheumatology, Our Lady's Children's Hospital; ^2^National Children's Research Centre; ^3^School of Medicine, University College Dublin, Dublin, Ireland

###### **Correspondence:** Daire O'Leary

**Introduction:** Chronic recurrent multifocal osteomyelitis (CRMO) is a rare autoinflammatory disease affecting bone. Untreated CRMO can result in complications such as vertebral compression fractures and leg length discrepancy. First line treatment is with non-steroidal anti-inflammatory drugs (NSAIDs) with a reported response rate of 50-80%. Limited data is available on the efficacy of second-line treatments which include methotrexate, bisphosphonates and biologic agents.

**Objectives:** To describe the experience of the National Centre for Paediatric Rheumatology (NCPR) treating currently attending CRMO patients with second-line agents.

**Methods:** Retrospective chart review of current patients requiring second-line agents attending the National Centre for Paediatric Rheumatology. Persistent active disease on NSAIDs was defined as persistent pain with tenderness/warmth or persistent bone oedema on MRI in at least one lesion site after >4 weeks treatment (CARRA consensus treatment guidelines).  Response to treatment was defined as clinical and/or radiological improvement without complete resolution. Remission was defined as normal ESR, absence of inflammatory pain or clinically active lesions, resolution of marrow oedema on MRI and absence of new lesions on whole-body MRI (modified CARRA criteria for treatment failure).

**Results:** Clinical charts of 30 patients with CRMO were reviewed. The median age at onset was 8.5 years, and median follow-up was 3 years. The median number of sites 3 (1-21), 87% had multifocal disease. Second-line treatment was required in 60%. The indications for second-line treatment were persistent active disease on NSAIDS or the presence of spinal lesions or cosmetically significant mandibular lesion.

A total of 19 patients received methotrexate, either alone (n=8 )or in combination with a biologic agent (n=11). Three received pamidronate and 11 received a biologic agent with methotrexate. None of the patients who received pamidronate achieved remission and all subsequently received methotrexate +/- a biologic. Of those who received methotrexate monotherapy, 1 is in remission on treatment, 1 remains in remission off treatment, 4 have responded but not achieved remission and 2 have received <2 months treatment.

Of those on biologic combination therapy, 2 patients are in remission on anti-TNF combination treatment, 1 discontinued biologic treatment due to a hypersensitivity reaction. The remaining patients have responded but have yet to achieve remission.

**Conclusion:** Treatment with second-line agents has led to a symptomatic improvement in all patients but remission in only 4 patients.

Combination therapy of methotrexate and a biologic agent may be the most favourable option but randomised controlled trials with clearly defined response and remission criteria are required.

**Disclosure of Interest**

None Declared

### P332 SUCCESSFUL TREATMENT OF TRNT1 MUTATIONS RELATED DISEASE WITH ETANERCEPT: TWO PEDIATRIC CASE REPORTS

#### Francesca Orlando, Carlo Maria Gallinoro, Daniela Melis, Maria Alessio

##### Mother and Child Department, University of Naples Federico II, Naples, Italy

###### **Correspondence:** Francesca Orlando

**Introduction:** *TRNT1* is a nuclear gene encoding a ubiquitous enzyme (CCA-adding tRNA nucleotidyl transferase enzyme) necessary for aminoacylation of both mitochondrial and cytosolic tRNA. TRNT1 mutations are associated to heterogeneous phenotypes and multisystem involvement of variable severity and progression.

**Objectives:** to characterize clinical features, laboratory assessment and treatment strategies in 2 patients affected by TRNT1 mutations referred to our pediatric rheumatology unit.

**Methods:** The patient 1 (P1) is a twenty years old female. From the first month of life she experienced recurrent fever associated to painful cutaneous lesions, edema of the hands, raised inflammatory markers and anemia. The symptoms were moderately controlled by corticosteroid therapy. One of these episodes was characterized by severe anemia (Hb 6.6 g/dl), requiring blood transfusion. At the age of 8 months she was found to have mild hypogammaglobulinemia, but no other alterations were found on immunological investigations. She showed normalization of the immunoglobulin levels at the age of 22 months. The physical examination found facial dysmorphisms, brittle hair, developmental delay and microcephaly. Splenomegaly was reported by abdomen ultrasound. Isolated Growth Hormone Deficiency (GHD) was diagnosticated at the age of 3 years and it was treated with recombinant human GH (rhGH) for the next 4 years, without improvement. At the age of 8 years she was diagnosed with bilateral sensorineural hearing loss. At the age of 10 she was diagnosticated with posterior subcapsular cataract, probably related to chronic steroid treatment. The patient 2 (P2) is a ten years old female. From four months of age she suffered from recurrent fever, associated from the age of 11 months to painful cutaneous lesions. Laboratory assessment showed elevated serum inflammatory markers, mild anaemia, hypogammaglobulinemia and reduced levels of B-cells. Growing up, she showed failure to thrive, developmental delay, facial dysmorphisms, brittle, microcephaly, hepato-splenomegaly and swelling of the right ankle. At the age of two she was diagnosticated with sensorineural hearing loss and bilateral cataract. At the age of 5 she started therapy with rhGH for GHD.

**Results:** Suspecting Chronic Infantile Neurologic Cutaneous and articular (CINCA) syndrome,even though negativity of molecular analysis of CIAS1, they started treatment with anti-IL1 (Anakinra) at the age of 9 (P1) and 2 (P2). Due to the partial response, P1 shifted to anti-TNFa therapy (Etanercept) at the age of 11 with significant improvement. P2 showed nonresponse to Anakinra so she shifted to Etanercept after four months. Whole exome sequencing was performed resulting in two mutation of TNRT1: P1 c.608G>A (p.Arg203Lys), c.1246A>G (p.Lys416Glu); P2 c.938delT (p.Leu313fs), c.1246A>G (p.Lys416Glu).  During the 9 years follow-up the patients underwent six-month clinical and laboratory assessment, showing normalization of inflammatory index and resolution of recurrent fever and associated symptoms.

**Conclusion:** Recently, treatment with Etanercept was reported in three patients, showing steady improvement of clinical and laboratory parameters. In this study we extend the cohort of patients described with TRNT1 mutations, we report a new mutation (c.938delT) and the longest follow up in patients treated with Etanercept, confirming efficacy and safety of this treatment.

Informed consent for the publication was obteined from the parents.

**Disclosure of Interest**

None Declared

### P333 FREQUENCY OF INFLAMMATORY DISEASE IN PATIENTS WITH FAMILIAL MEDITERRANEAN FEVER

#### Gulcin Otar Yener^1^, Selcuk Yuksel^1^, Zahide Ekici Tekin^1^, Ilknur Girisgen^2^

##### ^1^Pediatric Rheumatology; ^2^Pediatric Nephrology, Pamukkale University School of Medicine, Denizli, Turkey

###### **Correspondence:** Gulcin Otar Yener

**Introduction:** The MEFV gene, which causes autosomal recessive Familial Mediterranean fever (FMF) disease, encodes pyrin protein to control inflammation, and mutations in this gene can cause fail to function and control the inflammation. Therefore, it has been reported that various inflammatory diseases are associated with FMF disease.

**Objectives:** The aim of this study is to investigate the frequency of FMF associated inflammatory diseases in children.

**Methods:** FMF patients who were followed at department of pediatric rheumatology in Pamukkale University in the last five years had been retrospectively reviewed. 26 of 322 FMF patients (7.8%) were diagnosed with FMF and inflamatory diseases.

**Results:** İnflamatory diseases were as followings: 10 sacroiliitis (38.5%), 5 vasculitis (19.2%), 2 oligoarticular juvenile idiopathic arthritis (7.7%), 1 polyarticular juvenile idiopathic arthritis (3.8%), 1 juvenile psoriatic arthritis (3.8%), 3 P-FAPA (11.5%), 1 inflammatory bowel disease (3.8%), and 1 celiac disease (3.8%). Five patients (19.2%) who were diagnosed FMF and vasculitis were diagnosed with 4 henoch schonlein purpura (HSP) and 1 hypocomplementemic urticarial vasculitis syndrome. Erythema nodosum developed during follow-up in 2 patients. M694V mutation in the MEFV gene was detected in 15 (57.6%) of 26 FMF patients with concomitant disease. 11.5% of M694V mutation was homozygous (1 patient polyarticular JIA, 1 patient sacroileitis, 1 patient HSP). All of the patients were receiving additional medication due to comorbidities while they were receiving colchicine treatment. These treatments were 4 methotrexate, 4 sulfasalazine, 2 etanercept, 1 tosilizumab, 1 intraarticular steroid.

**Conclusion:** Some inflammatory diseases including juvenile idiopathic arthritis, P-FAPA, vasculitis, celiac and inflammatory bowel disease were more common with FMF. M694V mutation in childhood FMF patients may be a susceptibility factor for related diseases. It should be routinely monitored in terms of other inflammatory diseases in our country where FMF is common.

**Disclosure of Interest**

None Declared

### P334 FAMILIAL CHRONIC RECURRENT MULTIFOCAL OSTEOMYELITIS

#### Gulcin Otar Yener^1^, Selcuk Yuksel^1^, Zahide Ekici Tekin^1^, Ilknur Girisgen^2^

##### ^1^Pediatric Rheumatology; ^2^Pediatric Nephrology, Pamukkale University School of Medicine, Denizli, Turkey

###### **Correspondence:** Gulcin Otar Yener

**Introduction:** Chronic recurrent multifocal osteomyelitis (CRMO) is an idiopathic, auto inflammatory disease characterized by exacerbations and healing periods.

**Objectives:** We aimed to present two siblings diagnosed as CRMO who were resistant to nonsteroidal anti-inflammatory drugs and steroid treatments. Their parents were first-degree cousins.

**Methods:** We reviewed the records of clinical symptoms, treatment modality and clinical course in those siblings and analyzed to data.

**Results:** Case 1:

A 13-year-old boy was admitted to our clinic with swelling on jaw and gait disturbance. Past medical history revealed that he was diagnosed with juvenile idiopathic arthritis at two years old in another rheumatology clinic and treated with some anti- inflammatory drugs such as steroid and naproxen. However he could not visit regularly. On physical examination at first presentation he suffered from limitation of mandibular movement and pain of right leg. There was no arthritis. Acute phase markers were higher (C reactive protein: 6.6 mg/dl and erythrocyte sedimentation rate: 74 mm/hour). MR imaging revealed contrast enhancement in the right mandibular and ramus bones, right ramus erosion. It was diagnosed as chronic osteomyelitis in his pathology and tissue culture was negative. Firstly the symptoms were controlled with short-term steroid therapy, then the steroid was gradually reduced and ceased. Meanwhile, methotrexate 15 mg / m2 /week was started. Since there was no response, etanercept 50 mg / week was commenced at third month. Although etanercept was administered for 6 months and no response was obtained, biologic treatment was switched to adalimumab (40 mg every two weeks). Significant clinical improvement was seen after a months of the adalimumab treatment and high acute phase markers decreased within six months (C reactive protein:0.22 mg/dl and erythrocyte sedimentation rate:38 mm/hour). He has no complaints for 10 months.

Case 2:

A 18-year-old boy presented with a swollen and painful right posterior leg following a minor trauma. Ten years ago he was admitted to another center with this the same complaint and biopsy was performed since it was presumed osteosarcoma. The pathology was found to be compatible with chronic osteomyelitis and also tissue culture was negative. He did not visit regularly the polyclinic like his brother, and he had complaints as irregular consume of steroid and sulfasalazine drug treatment for a long time. On physical examination, right femur and tibia sensitivities were observed. Acute phase reactants were high (C reactive protein: 4.1 mg/dl and erythrocyte sedimentation rate: 80 mm/hour). The whole body MR detected chronic multifocal osteomyelitis in the region extending from the right femur and proximal tibia shafts to the distal metaphyseal region. Methotrexate 15 mg / m2 / week and adalimumab 40 mg / 2 weeks was commenced. The clinical response was complete at 3 months of treatment and acute phase reactants decreased within 9 months of treatment (C reactive protein:0.02 mg/dl and erythrocyte sedimentation rate:27 mm/hour). The genetic screening of patients was planned. Informed consent to publish had been obtained from the patient.

**Conclusion:** CRMO treatment should be individualized. Therapy choice can be variable such as only nonsteroidal anti-inflammatory drugs or steroid or anti-TNF therapy. These CRMO brothers thought to us that there is a strongly probable genetic basis in the etiology of the disease.

**Disclosure of Interest**

None Declared

### P335 EVALUATION OF ISSF SCORES IN TURKISH CHILDREN WITH FAMILIAL MEDITERRANEAN FEVER: SINGLE CENTER EXPERIENCE

#### Fatih M. A. Özdemir^1^, Nesrin Gülez^2^, Balahan Makay^2^

##### ^1^Pediatrics; ^2^Pediatric Rheumatology, Dr. Behçet Uz Children's Hospital, İzmir, Turkey

###### **Correspondence:** FATİH M. A. ÖZDEMİR

**Introduction:** Although familial Mediterranean fever (FMF) is the most common autoinflammatory disease in the world, many problems of FMF management are not standardised for optimal disease control and follow-up. A standardised assessment of disease severity is required for therapeutic decisions in managing patients with FMF. There are several severity scoring systems for FMF. Because there is no consensus on any of them and they were not consistent with each other, an international group of FMF experts developed and initially validated a new severity scoring system for FMF, which was called “international severity scoring system for FMF” (ISSF). It is an easily applicable and widely acceptable scoring system for research and clinical practice.

**Objectives:** To evaluate the disease severity scores of our patients by using ISSF and compare them with literature data.

**Methods:** The patients who were diagnosed as FMF according to the Tel-Hashomer criteria and being followed at Department of Rheumatology in Behçet Uz Children’s Hospital for at least 6 months were consecutively enrolled in this cross-sectional study. Demographic data, FMF symptoms, MEFV mutation, disease duration, time to delay for diagnosis, duration of follow-up, duration of attacks, and disease severity score were recorded for each patient. Patients were classified as mild, moderate and severe according to ISSF. The study protocol was approved by the local ethical committee.

**Results:** A total of 160 patients (88 females and 72 males) were enrolled. The mean age of the patients was 12 ± 4.3 years. Thirty four (%21.3) patients have mild, 113 (%70.5) patients moderate, 13(% 8.2) patients have severe score according to ISSF. The most frequently identified mutations in our study population were M694V (%54.3) and E148Q (%18). Homozygous patients (M694V, M694I, M680I, and V726A) had more severe disease. The disease severity score was positively correlated with the duration and dose of colchicine. There was not a significant correlation between age at disease onset and ISSF scores. When the patients were evaluated according to the age groups, the duration of the attack was shorter in older patients (p< 0.05). Besides, different organ involvement during attacks was higher in patients older than 7 years.

**Conclusion:** The compatibility of the data obtained from our study with the literature in general indicates that ISSF is a suitable tool for assessing disease severity. Furthermore, the results of this study suggests that addition of “existence of some pathological MEFV mutations” as a criterion of ISSF may be useful.

**Trial registration identifying number:** FMF, ISSF

**Disclosure of Interest**

None Declared

### P336 EXACERBATION OF PREVIOUS PSORIASIS DURING TREATMENT WITH ETANERCEPT IN CHILDREN WITH CHRONIC NON-BACTERIAL OSTEOMYELITIS

#### Manuela Pardeo, Virginia Messia, Camilla Celani, Claudia Bracaglia, Rebecca Nicolai, Giulia Marucci, Fabrizio De Benedetti, Antonella Insalaco

##### Division of Rheumatology, IRCCS Ospedale Pediatrico Bambino Gesù, Rome, Italy

###### **Correspondence:** Manuela Pardeo

**Introduction:** In a recent series of 486 cases of Chronic Non-bacterial Osteomyelitis (CNO) from the Eurofever international registry, association between psoriasis and CNO was observed in 4% of the patients. The tumor necrosis factor-alpha (TNF-α) inhibitors drugs are increasingly used in treating psoriasis.  In the Consensus Treatment Plans for Chronic Non-bacterial Osteomyelitis anti-TNF-αare also a therapeutic option for patients with refractory CNO. Based on these considerations we decided to treat 3 patients with CNO and psoriasis with etanercept but we observed a worsening of skin involvement.

**Objectives:** To report the worsening of psoriasis in patients with CNO in relation to use of etanercept.

**Methods:** Children with diagnosis of CNO associated with psoriasis and treated with etanercept were included. Subsequent response to this therapy was recorded.

**Results:** Pt1: caucasian female with diagnosis, at age of 10 years, of CNO (long bones and vertebral lesions) and psoriasis (scalp and left plantar). Due to clinical and radiological worsening of bone involvement after non-steroidal anti-inflammatory drugs (NSAIDs) and 2 cycles of pamidronate (1 mg/kg/day for 3 consecutive days every 3 months), we decided to start etanercept.  After 3 months of therapy psoriasis got significantly worse. We discontinued etanercept with initial improvement of skin lesions. Pt2: 17 year-old caucasian female with CNO (long bones, vertebral and pelvic lesions) and psoriasis (scalp and plaque psoriasis on the legs) treated at the onset of the disease with IV cycles of pamidronate and sulfasalazine. Despite an initial and persistent good response, after 2 years of treatment with sulfasalazine the patient showed clear evidence of lack response. We started etanercept and we observed, after 3 months of therapy, an improvement of clinical and radiological bones involvement but a worsening of psoriasis. The patient refused to discontinue etanercept. After 6 months of therapy she had persistent remission of CNO and stable skin lesions. Pt3: caucasian female with diagnosis, at age of 8 years, of CNO (vertebral, pelvic and long bones lesions) and psoriasis (scalp and periauricular lesions). Due to D6 and D9 vertebral wedge deformities patient was treated with glucocorticoids and pamidronate. After 3 months she had a persistent polyarthralgia associated with radiological increase in the number of bone lesions. We started etanercept and we complete pamidronate cycles (a total of 4 cycles). She had good clinical response without pain or functional impairment and with decrease of bone lesions. After starting etanercept we observed a severe exacerbation of psoriasis with increase in the number of skin lesions (scalp, plaque on the legs and nails). After 6 months of therapy we decided to discontinue etanercept. At the last follow up visit, 2 months later, skin and bone lesions were stable.

**Conclusion:** We report three cases of CNO and psoriasis treated with etanercept with worsening of skin lesions. It is unclear the mechanism that leads to exacerbation of previous psoriasis during the course of treatment with etanercept. Further studies with a larger number of patients are needed to clarify this mechanism and the management of the patients. Should be discontinued etanercept, switched to another anti-TNF-α or combined with another systemic treatment such as methotrexate or cyclosporine? Informed consent to publish had been obtained from the parents.

**Disclosure of Interest**

M. Pardeo: None Declared, V. Messia: None Declared, C. Celani: None Declared, C. Bracaglia: None Declared, R. Nicolai: None Declared, G. Marucci: None Declared, F. De Benedetti Grant / Research Support from: Novartis, Novimmune, Hoffmann- La Roche, SOBI, AbbVie, Pfizer, A. Insalaco: None Declared

### P337 A CHILD WITH MAJEED SYNDROME FROM INDIA

#### Pallavi Pimpale Chavan, Raju P. Khubchandani

##### Pediatric Rheumatology Clinic,Department of Pediatrics, Jaslok Hospital and Research Centre, Mumbai, India

###### **Correspondence:** Pallavi Pimpale Chavan

**Introduction:** Majeed syndrome is a very rare autosomal recessive, autoinflammatory disorder characterized by a triad of chronic recurrent multifocal osteomyelitis, congenital dyserythropoietic anemia and neutrophilic dermatosis caused by LPIN2 gene mutation. 12/13 mutation positive cases identified till 2017 have been reported from the Middle East.

**Objectives:** We describe our brief experience with the second family with a child afflicted with Majeed syndrome from India.

**Methods:** A 3 year old male child born of a consanguineous marriage, presented with recurrent irregular episodes of fever with irritability and failure to thrive since 5 months of age . At 9 months of age parents noticed, small skin ‘boils’ over the trunk and limbs, which recurred every 15-20 days along with the fever and stayed for 4-5 days. At 18 months, parents noticed that he refused to bear weight and walk and would cry on handling.

Gradually the episodes of painful small skin boils, limb pains which the parents could not localize and fever increased in frequency and after multiple pediatric, orthopaedic consults he was referred to us by a pediatric neurologist who had been referred the case to rule out Fabry disease. The pediatric neurologist had asked for whole exome sequencing and referred the case to us for clinical evaluation sensing a musculoskeletal rather than neurological disease.

**Results:** On examination the weight (10 kg) and height (83 cm) were below 3^rd^ centile. He had pallor, painful swelling over the left tibia, motor delay with hamstring tightness and mild bilateral knee contractures. Language and social milestones were normal. There were no skin lesions at the time of evaluation and systemic examination was normal.

Review of his past investigations revealed a persistent, microcytic hypochromic anemia (Hemoglobin 8.3-10.6 gm/dl) with raised erythrocyte sedimentation rate (54-105 mm/hr), C-reactive protein (37-98 mg/l) and platelets (4.3-8.9 x10^3^ cu mm).

His genetic mutation for LPIN 2 returned positive with homozygous missense variation in exon 17 of the LPIN2 gene resulting in amino acid substitution of cysteine to arginine at codon 736 (p.Arg736Cys;ENST00000261596). Meanwhile a bone scintigraphy done, showed increased vascularity and osseous activity in proximal shaft of left tibia with mild osseous activity at D9 and D10 vertebrae. A bone marrow examination was not performed as a conclusive mutation diagnosis was obtained.

He showed a dramatic response to Ibuprofen with abatement of fever, no skin boils and improvement of general mood and playfulness. With a view to attempt to omit NSAIDs intravenous Pamidronate was commenced. After the first course at 10 weeks follow up, he has gained weight (11kg), is afebrile, walks with support with no new skin lesions.

**Conclusion:** Paucity of awareness amongst the medical community about these rare illnesses are responsible for diagnostic delays and complex referral pathways. With the high rates of consanguinity in several parts of our country we believe that we are seeing just the tip of the iceberg of monogenic autoinflammatory diseases. Due to non availability of Interleukin-1 inhibitors in India, which are highly effective at controlling osseous and systemic inflammation in Majeed syndrome, we have limited treatment options to offer to our patient. We have offered educational resource and genetic counselling to the family. Written informed consent was obtained from the patients for publication.

**Disclosure of Interest**

None Declared

### P338 FAMILY IMPACT AND HEALTH-RELATED QUALITY OF LIFE IN PERIODIC FEVER, APHTOUS STOMATITIS, PHARYNGITIS AND CERVIKAL ADENITIS (PFAPA) SYNDROME BEFORE AND AFTER TONSILLECTOMY

#### Karin Rydenman^1,2^, Carina Sparud-Lundin^3^, Anna Karlsson^4^, Stefan Berg^1,5^, Anders Fasth^1,5^, Per Wekell^1,2^

##### ^1^Department of Pediatrics, Institute of Clinical Sciences, Sahlgrenska Academy, University of Gothenburg, Gothenburg; ^2^Department of Pediatrics, NU-Hospital Group, Uddevalla; ^3^Institute of Health and Care Sciences, Sahlgrenska Academy, University of Gothenburg; ^4^Department of Rheumatology and Inflammation Research, Institute of Medicine, Sahlgrenska Academy, University of Gothenburg; ^5^The Queen Silvia Children’s Hospital, Gothenburg, Sweden

###### **Correspondence:** Karin Rydenman

**Introduction:** PFAPA is an autoinflammatory disease characterized by recurrent febrile episodes with associated symptoms from the mouth and throat, with a typical onset before 5 years of age. Tonsillectomy has been shown to be curative in a majority of patients, but remains debated as PFAPA is self-limiting and usually resolves spontaneously before adulthood. However, the consequences of the recurring fevers in terms of health-related quality of life and family impact have not been previously studied.

**Objectives:** The aim of this pilot study was to investigate health-related quality of life in children with PFAPA and the impact of the disease on their families before and after tonsillectomy.

- Is the health-related quality of life affected in children with PFAPA, and does it improve after tonsillectomy, as measured by Pediatric Quality of Life Inventory™ 4.0 Generic Core Scales (PedsQL™ 4.0 GCS) ?

- What impact does PFAPA have on the family of patients and does it change after tonsillectomy, as measured by PedsQL™ Family Impact Module (PedsQL™ FIM)?

**Methods:** Children with typical PFAPA referred for tonsillectomy in our clinics were enrolled consecutively. Parents to these patients answered PedsQL™ 4.0 GCS to measure health-related quality of life during fever episodes, between episodes and six months after tonsillectomy. Family impact of PFAPA was measured by PedsQL™ FIM filled in by the parents before and six months after tonsillectomy. Parents were also asked to keep a diary of fever episodes during the study period. The PedsQL™ scales were scored according to The PedsQL™ Scoring Algorithm, were items are reversely scored and linearly transformed to a 0-100 scale, so that higher scores indicate better outcome. The total scale score for each patient was obtained by computing the mean of all items on the scale.

**Results:** In this pilot, six patients and their parents have completed the study. The patients had a median of 7,5 episodes (range 5-9) during the six months prior to tonsillectomy. After tonsillectomy, all patients were free of symptoms during the six months of follow-up. Total scale scores on PedsQL™ 4.0 GCS were low during fever episodes with a median of 51,1 (range 43,5-55,4) and improved markedly between episodes to a median of 88,1 (range 67,4-96,6) and after tonsillectomy to a median of 93,9 (range 82,6-99). This also showed a tendency to lower scores between fever episodes before tonsillectomy compared to after tonsillectomy, although no further statistical analysis was made due to the low number of patients. Total scale scores on PedsQL™ FIM had a median of 61,5 (range 42,4-74,3) before tonsillectomy and improved to a median of 93,8 (range 52,1-100) after tonsillectomy. One family stood out with a low and unchanged score on the family impact module after tonsillectomy. In this family, a younger sibling of the patient was diagnosed with PFAPA during the study period.

**Conclusion:** Even though PFAPA syndrome is a benign disease with a good long-term prognosis, this small pilot study indicates that it has considerable negative impact on the child affected by the disease and the entire family. Tonsillectomy may be beneficial as ceasing of the fever episodes improves the wellbeing of the child and the family. There is a plan to continue this study with inclusion of a greater number of patients.

**Disclosure of Interest**

None Declared

### P339 CHRONIC RECURRENT MULTIFOCAL OSTEOMYELITIS IN CHILDREN: A SINGLE CENTER EXPERIENCES OVER FIVE YEARS

#### Erdal Sag^1^, H. Emine Sonmez^1^, Selcan Demir^1^, Yelda Bilginer^1^, Bilge Ergen^2^, Ustun Aydingoz^2^, Seza Ozen^1^

##### ^1^Division of Pediatric Rheumatology, Hacettepe University Faculty of Medicine, Department of Pediatrics; ^2^Department of Radiology, Hacettepe University Faculty of Medicine, Ankara, Turkey

###### **Correspondence:** Erdal Sag

**Introduction:** Chronic recurrent multifocal osteomyelitis (CRMO) is a rare disease characterized by sterile bone inflammation. It is an orphan disease with many unclear aspects in diagnosis, treatment and follow-up.

**Objectives:** The aim of this study is to report our experiences of pediatric CRMO patients, as a tertiary pediatric rheumatology center, regarding clinical and laboratory findings, treatments, disease activity and treatment responses.

**Methods:** Children who were diagnosed with chronic recurrent multifocal osteomyelitis (CRMO), and were followed-up in the Pediatric Rheumatology Unit of Hacettepe University between January 2008 and January 2017, were included in this study. Clinical characteristics and laboratory findings (including radiological and pathological evaluation) were reviewed from medical charts and electronic files of the patients. In addition, treatments, treatment responses and disease activity were documented retrospectively.

**Results:** There were 15 CRMO patients (8M/7F) with a median age at diagnosis of 10.5 yrs. Bone pain was the most common presenting symptom. All of the patients had multifocal bone lesions. Vertebra (66%) and femur (66%) were the most commonly affected bones. 8 of the patients also had sacroiliitis however only one of them was HLA-B27 positive. Whole-body MRI used as a diagnostic tool in 13 patients revealing bone marrow edema (84.6%), osteitis (69.2%) and periosteal reaction (61.5%). Bone biopsy was performed in 6 patients showing sterile osteomyelitis with mixed inflammatory infiltration and sclerosis. All patients were initially treated with NSAIDs, however DMARDs, anti-TNF agents or pamidronate were added to therapy due to inadequate treatment response. Clinical remission was achieved in 12 patients (1 with NSAID, 3 with methotrexate, 1 with pamidronate and 7 with an anti-TNF agent). During the follow-up period, relapses were observed in four patients presented with pain and/or a newly formed bone lesion on MRI. However, finally all of these patients also reached remission. Three patients continued to have clinical and radiologic active disease despite anti-TNF treatment in two patients and pamidronate in one patient.

**Conclusion:** CRMO is a chronic disease which may have a progressive or relapsing-remitting course. NSAIDs and anti-TNF drugs are the most efficacious treatment, however pamidronate may also be used for refractory cases. Although there is no consensus on follow-up parameters, WB-MRI is a very useful tool to show active disease even the patient is in clinical remission. Improvement of the knowledge about this rare disease may help to enlighten the unknowns of the disease.

**Disclosure of Interest**

None Declared

### P340 CRYOPYRIN-ASSOCIATED PERIODIC SYNDROME (CAPS) IN REAL CLINICAL PRACTICE OF PEDIATRIC RHEUMATOLOGIST IN ONE SINGLE CENTER OF RUSSIA

#### Evgeny S. Fedorov^1^, Svetlana O. Salugina^1^, Ekaterina Y. Zaharova^2^, Elena Kamentz^2^

##### ^1^Pediatric, Scientific Research V.A.Nasonova Rheumatology Institute; ^2^heredity metobolic disords, FSBI “Research Centre for Medical Genetics”, Moscow, Russian Federation

###### **Correspondence:** Svetlana O. Salugina

**Introduction:** Cryopyrin-associated periodic syndrome (CAPS) is a rare group of monogenic autoinflammatory diseases (AIDs) associated with a mutation in the gene NLRP3.

**Objectives:** To characterize clinical and laboratory manifestations, genetic features, therapy options, the course of the disease in patients with CAPS in the practice of pediatric rheumatologist.

**Methods:** It was an observational study of pediatric and adult patients with clinical diagnosis CAPS according to the Russian Federal Rheumatology Center from 2007 to 2017. All patients passed the examination generally accepted in rheumatology including molecular genetic tests of NLRP3 gene.

**Results:** The study included 25 patients with CAPS (17 children, 8 adults), there were 11 males and 14 females. A total of 22 pts were diagnosed with MWS and 3 pts had CINCA/NOMID. The aged of pts were from 6 months up to 57 years, the duration of the disease was from 1 month up to 44 years. Fever was noted in all patients, skin rashes in 92%, eye symptoms in 52%, sensorineural hearing loss in one third of patients, arthralgia/arthritis in 60%, neurological disorders in 40%. All patients except one (with CINCA/NOMID) showed mutations in the gene NLRP3, all in heterozygous state. The mutations were p.T348M in 7 pts, Q703K in 13, V198M in 4, other mutations in 6. 11 out of 25 pts received canakinumab (Il-1 inhibitor) for 1 - 5 years. The complete response to therapy was received in 9, partial response-in 2 pts previously treated by anakinra.  There were no major adverse events (AE) or serious AE (SAE).

**Conclusion:** Despite the rarity, the patients with CAPS are found in the practice of pediatric rheumatologist. Canakinumab is very effective and save treatment for CAPS pts. But the final diagnosis and appointment of therapy should be carried out with the participation of experts in the field of AID.

**Disclosure of Interest**

None Declared

### P341 CANAKINUMAB TREATMENT IN A CASE OF PAEDIATRIC SWEET SYNDROME

#### Lucrezia Sarti, Ilaria Maccora, Francesca Tirelli, Edoardo Marrani, Teresa Giani, Gabriele Simonini, Rolando Cimaz

##### Rheumatology, Meyer Children's Hospital, Florence, Italy

###### **Correspondence:** Lucrezia Sarti

**Introduction:** Sweet Syndrome (Acute Febrile Neutrophilic Dermatosis) is an uncommon disease, extremely rare in children, where it accounts 5% - 8% of all cases. The clinical suspicion is usually confirmed by the presence of a diffuse infiltrate of mature neutrophils in the upper dermis at biopsy. On the bases of recent researches that suggest an aberrant hyperproduction of interleukin‐1 in the pathogenesis of this dermatosis, Anakinra, human interleukin 1 receptor antagonist protein, has been used with success in some adults.

**Objectives:** We report our experience with Canakinumab (human anti-IL-1β monoclonal antibody) in a paediatric case of Sweet Syndrome.

**Methods:** A five-years old male was admitted to our hospital for an abrupt onset of fever, multiple, indurated painful red papules, and plaques, associated with increased values of CRP, and ESR and neutrophilia. On histopathological examination, neutrophilic and lymphohistiocytic infiltrate and edema were observed.

**Results:** On the basis of anamnestic, clinical, hystophtologic data and laboratory tests the diagnosis of Sweet’s Syndrome was made and prednisone was started. Despite a clear cutanous improvement, lesions apperared to flear at each attempted steroid withdrawal. Anakinra was than introduced with a gradual good response. Because of the scarce compliance due to the inconvenience of daily injections, three months later, Anakinra was switched to Canakinumab (1 injection every 28 days). At a 4-months follow-up visit the patient’s cutaneous lesions were nearly resolved with minimal intervention.

**Conclusion:** To our knowledge, this is the first report of anti-IL-1β monoclonal antibody treatment in Sweet syndrome. In our experience Canakinumab proved to be safe and effective. Informed consent had been obtained from the parent.

**Disclosure of Interest**

None Declared

### P342 DIVERGENT RESPONSE OF REFRACTORY RECURRENT PERICARDITIS (RP) TO TWO DIFFERENT IL1 BLOCKING AGENTS: DOES IL-1Α HAVE A CRUCIAL ROLE IN THE PATHOGENESIS OF RP?

#### Sara Signa^1,2^, Matteo D'Alessandro^1,2^, Marta Bustaffa^1,2^, Rita Consolini^3^, Angela Miniaci^4^, Martina Bizzi^3^, Andrea Pession^4^, Leonardo Oliveira Mendonça^1^, Roberta Caorsi^1^, Angelo Ravelli^1,2^, Marco Gattorno^1^

##### ^1^Clinica Pediatrica e Reumatologia, IRCCS Istituto G.Gaslini; ^2^DINOGMI, Università di Genova, Genova; ^3^Clinica pediatrica, Università di Pisa, Pisa; ^4^Pediatric Unit, Department of Medical and Surgical Sciences, S. Orsola - Malpighi Hospital, University of Bologna, Bologna, Italy

###### **Correspondence:** Sara Signa

**Introduction:** Recurrent pericarditis (RP) is a common complication of acute pericarditis (15–30%). Treatment regimen consists of a combination of non-steroidal anti-inflammatory drugs (NSAIDs) with colchicine with the addition of corticosteroids in resistant or intolerant cases. Anakinra is a therapeutic option as steroid-sparing agent. Anakinra neutralises the biologic activity of interleukin-1α (IL-1α) and interleukin-1β (IL-1β) by inhibiting their binding to IL-1 type I receptor, while Canakinumab is a monoclonal antibody against IL-1β only.

**Objectives:** To describe two cases of refractory recurrent pericarditis with a divergent response to two different IL-1 blockers.

**Methods:** The first patient is a 8-years old girl with recurrent pericarditis started in April 2015 (6 years old), after surgical correction of an atrial septal defect. NSAIDs and oral steroids were started and then gradually tapered, with prompt relapse after the steroid suspension requiring a pericardiocentesis. The child showed a steroid-dependent recurrent pericarditis, with several relapses if tapering was attempted and no benefit from colchicine and NSAIDs. After five relapses Anakinra was started with a fast and complete clinical response, but discontinued after 2 weeks for severe local injection reactions. She was firstly evaluated in our Centre in April 2016 and in July 2016 therapy with canakinumab 150 mg (approximately 4 mg/kg) every 4 weeks was started, together with steroids (then gradually tapered) and NSAIDs.

The second patient is a 10-years-old girl with a history of idiopathic recurrent pericarditis, started in April 2017 with typical clinical picture, need of an urgent pericardiocentesis at onset and initial benefit from NSAIDs and colchicine. However, ten days after the first episode a relapse occurred and therapy with anakinra was established with dramatic and complete clinical response. Two months later, while being in complete remission, anakinra was replaced with canakinumab (2.5 mg/kg/dose) due to patient’s poor compliance to daily injections.

**Results: Patient 1:** She experienced four relapses during Canakinumab therapy (July 2016 -December 2017), despite the modification of the schedule (4 mg/kg every three weeks) associated with the reintroduction of colchicine since April 2017 and continuous steroid treatment with repeated flares at every attempt of tapering. In January 2018 a procedure of desensitization from anakinra was performed, with success. Anakinra therapy is currently ongoing (12 weeks follow up), in association with low-dose colchicine. An almost complete withdrawn of steroid is currently undergoing with no signs of flare.

**Patients 2**:Ten days after the first Canakinumab injection she experienced a relapse requiring oral steroids. Anakinra (2 mg/kg/day) was subsequently re-started allowing steroid tapering in few days. The patient showed complete remission in anakinra as monotherapy with a good tolerance. After further 6 months follow-up under anakinra, the patient did not show any clinical, laboratory or echocardiographic signs of relapse.

**Conclusion:** We describe two cases of substantial failure of the treatment with anti-IL-1b monoclonal antibodies treatment in steroid-dependent idiopathic RP. In both case a good response to the treatment with recombinant IL-1 receptor antagonist was achieved. These anecdotal and preliminary observations suggest a different efficacy of the two IL-1 blockers in the management of recurrent pericarditis and support a possible pivotal role of IL-1α in the pathogenesis of this condition. Informed consent to publish had been obtained from the parents.

**Disclosure of Interest**

S. Signa: None Declared, M. D'Alessandro: None Declared, M. Bustaffa: None Declared, R. Consolini: None Declared, A. Miniaci: None Declared, M. Bizzi: None Declared, A. Pession: None Declared, L. Oliveira Mendonça Grant / Research Support from: ESID 2017 fellowship, R. Caorsi: None Declared, A. Ravelli: None Declared, M. Gattorno: None Declared

### P343 PAPA SYNDROME - FIVE FAMILIAL CASES FROM BULGARIA

#### Krastina H. Stefanova-Kelly^1,2^, Anastasia Shishmanova^1^, Lyubov Chochkova^1^, Vasilka Todorova^1^, Juan Aróstegui^3^

##### ^1^Pediatrics and Genetic Diseases, St.George University Hospital, Plovdiv, Bulgaria; ^2^Pediatrics and Medical Genetics, Medical University - Plovdiv, Plovdiv, Bulgaria; ^3^Department of Immunology-CDB, Hospital Clinic, Barcelona, Spain

###### **Correspondence:** Krastina H. Stefanova-Kelly

**Introduction:** PAPA syndrome (Pyogenic arthritis, Acne and Pyoderma gangrenosum) is a rare autosomal dominant autoinflammatory disorder. To our knowledge it has not been described in Bulgarian patients before.

**Objectives:** To describe the phenotype of five family members with PAPA syndrome from Bulgaria; to report one novel mutation of exon 11 of the PSTPIP1 gene, and a second one not previously associated with PAPA syndrome, and to share our experience with different treatments.

**Methods:** We present five family members from three generations. The children have recurrent pyogenic arthritis, one with Pyoderma gangrenosum as well. Their father and paternal grandmother have suffered similar, although less frequent, episodes as children, developed severe acne as adolescents and started suffering from Pyoderma gangrenosum as adults. Genetic tests confirmed mutations in exon 11 of the PSTPIP1 gene. One (c.766C>A) had been previously described in patients with hyperzincemia and hypercalprotectinaemia, but not in PAPA syndrome, the second was a novel mutation, c.766C>A.

**Results:** We established earlier onset of arthritis in subsequent generations (the youngest of our patients had his first episode at 7 months of age). Pyoderma gangrenosum which usually appears in adulthood started at the age of 4 years in the same patient.

Intraarticular steroids provided mixed results. Treatment with Salazopirine in one patient with active arthritis, and with Resochin in another was unsuccessful. Oral steroid therapy for Pyoderma gangrenosum had variable, but mostly unsatisfactory results.

**Conclusion:** We report 2 mutations in the PSTPIP1 gene – one novel and one not previously associated with PAPA syndrome. Treatment with some of the standard DMARDs, used in JIA, does not seem to be effective. Informed consent to publish had been obtained from the parents and patients.

**Disclosure of Interest**

None Declared

### P344 TEN-YEAR OUTCOME OF DIFFUSE SCLEROSING OSTEOMYELITIS OF THE MANDIBLE TREATED WITH INTRAVENOUS PAMIDRONATE: SUSTAINED RESOLUTION OF PAIN AND BONE INFLAMMATION WITH IMPROVED COSMETIC APPEARANCE - A CASE STUDY

#### Chloe Stephenson^1^, Alexandra Rice^1^, Xingchang Wei^1^, Weiming Yu^2^, Rebeka Stevenson^1^, Paivi M. Miettunen^1^

##### ^1^Paediatrics; ^2^Pathology, University of Calgary, Calgary, Canada

###### **Correspondence:** Chloe Stephenson

**Introduction:** Diffuse sclerosing osteomyelitis of the manidble (DSMO) is a unifocal form of chronic non-bacterial osteitis (CNO) and predominantly affects patients < 18 years. Intravenous pamidronate (IV-PAM) has been reported to be effective in multifocal CNO in short term.

**Objectives:** To describe a long-term clinical and radiologic outcome of a DSMO patient following treatment with IV-PAM.

**Methods:** A 4-year old Caucasian female was diagnosed with DSMO and prospectively followed from 2007-2017. She presented with a 20-month history of facial asymmetry and painful bony expansion of the left hemimandible. Imaging (radiographs, CT) showed an expansile lesion with radiolucency, some ground-glass appearance, periosteal reaction and soft-tissue swelling. Infectious osteomyelitis was ruled out, due to negative blood cultures and inefficacy of IV and oral antibiotics. Fibrous dysplasia was then considered. She remained symptomatic despite ibuprofen (10 mg/kg/dose 4 times daily) and a jaw replacement surgery was planned due to progressive symptoms. Bone biopsy was performed and histopathology demonstrated paucity of the spindle cells characteristic to fibrous dysplasia, and revealed multiple osteoblasts and inflammatory plasma cells. Whole-body MRI confirmed an isolated lesion in the left hemimandible with soft tissue edema and the diagnosis of DSOM was made at age 4 years. She was referred to rheumatology after failing NSAIDs and analgesics. Examination revealed marked swelling to the left mandibular area. Laboratory investigations showed elevated erythrocyte sedimentation rate [28mm/hr (normal <10 mm/hr)] and platelet count [606 10^9^/L (normal 150-400 10^9^/L)]. C-reactive protein was normal. She was started on monthly 1-day IV-PAM infusions (1st dose 0.5mg/kg; each subsequent dose: 1mg/kg). The response to treatment was assessed according to visual analogue score for pain (VAS, 0=no pain; 10=maximum pain), sequential MRIs and clinical photos.

**Results:** She received 8 monthly IV-PAM infusions. The base-line VAS was “10”, which decreased to “0” after the first infusion and VAS remained “0” throughout the remainder of treatment. MRI documented resolution of abnormal signal at 5 months with gradual mandibular remodeling and sustained improvement at 12 months. Clinical photographs documented the gradual improvement of facial symmetry and improved affect, which was sustained during follow-up. She had no side effects to IV-PAM apart from nausea following the first infusion.

She had three MRI confirmed flares at 22, 67 and at 120 months at the identical site with pain and decreased mouth opening. Her pain VAS ranged from 4-6/10 during flares. Each flare responded to Naproxen monotherapy both clinically and by MRI and she required no further IV-PAM. She required dental braces at age 10-years. She remains asymptomatic at 120 month follow-up with excellent functional and cosmetic outcome.

**Conclusion:** DSMO is challenging to diagnose and treat due to its rareness and lack of uniformly effective treatment. IV-PAM was effective in this young refractory patient in resolution of pain, mandibular remodeling and cosmetic improvement that was sustained during long-termfollow-up. We recommend a bone biopsy of mandibular CNO/DSMO to help confirm the diagnosis to differentiate it from fibrous dysplasia of the jaw. Informed consent to publish had been obtained.

**Disclosure of Interest**

None Declared

### P345 A CASE OF MULTISYSTEMIC INFLAMMATORY DISEASE WITH TYPE I INTERFERON DYSREGULATION IN A GIRL WITH AN HETEROZYGOUS DNASE II MUTATION

#### Giada Zanella^1^, Giulia Gortani^2^, Serena Pastore^2^, Alberto Tommasini^2^, Flavio Faletra^2^, Alessandra Tesser^2^, Erica Valencic^2^, Elisa Piscianz^2^, Andrea Taddio^1,2^

##### ^1^University of Trieste; ^2^Institute for Maternal and Child Health Burlo Garofolo, Trieste, Italy

###### **Correspondence:** Andrea Taddio

**Introduction:** A type I interferonopathy due to DNase II deficiency has ben recently described in three cases with multi-organ diseases.

**Objectives:** To describe the case of an italian 14-year-old girl with a complex clinical phenotype in which an autoinflammatory disease was suspected and a new deleterious heterozygous DNase II mutation was found.

**Methods:** The most meaningful clinical aspects of our patient are:

- severe hemolytic anemia detected at six weeks of age, requiring red blood cell transfusions and finally resolved at three years of age
- cholestatic hepatitis beginning at age 8 months; markers of cholestasis normalized at age 2 years but she developed an inactive liver cirrhosis with stable hepatosplenomegaly and mild thrombocytopenia
- failure to thrive and recurrent hypoglycemia with need of enteral feeding till age of 6 years
- antibody-negative insulin dependent diabetes mellitus with onset at 10 years of age
- normal early psychomotor development with subsequent learning difficulties at school. Brain MRI at age 11 showed multiple alterations of signal intensity in the subcortical and periventricular white matter
- recurrent episodes of enteritis and keratitis
- normal erythrocyte sedimentation rate and C-reactive protein with fluctuating hypergammaglobulinemia and antinuclear antibodies

**Results:** The major classes of inborn errors of metabolism, cirrhosis and autoimmune diseases were investigated and ruled out.

The peculiar clinical picture put together signs that taken overall can be reminiscent of a rare DNAse II deficiency.

In particular, early onset and self-healing anemia and cholestatic hepatopathy, subcortical white matter dystrophy, non-autoimmune insulin dependent diabetes and hypergammaglobulinemia are all described in the recent report about DNAse II deficiency. Moreover, we describe an increased interferon signature and thus decided to sequence DNAse II gene. The analysis revealed a heterozygous mutation (c.621delC deletion on the exon five) predicted to be deleterious. However, the same deletion was found in patient’s father, prompting us to perform whole-exome sequencing in the search for other genetic anomalies. So far, the analysis didn’t held any definite explanation for the disease.

**Conclusion:** Several evidence support a role for the mutation in DNAse II in the disease of the patient but other yet unknown genetic and environmental factors have to be investigated. Informed consent to publish had been obtained.

**Disclosure of Interest**

None Declared

### P346 PREVALENCE OF CRANIAL INVOLVEMENT IN A COHORT OF ITALIAN PATIENTS WITH CHRONIC NON-BACTERIAL OSTEOMYELITIS

#### Andrea Taddio^1,2^, Giovanna Ferrara^2^, Manuela Pardeo^3^, Marco Cattalini^4^, Francesco La Torre^5^, Martina Finetti^6^, Antonella Meini^4^, Clotilde Alizzi^7^, Barbara Teruzzi^8^, Gabriele Simonini^9^, Virginia Messia^3^, Serena Pastore^1^, Alberto Tommasini^1^, Rolando Cimaz^9^, Antonella Insalaco^3^, Marco Gattorno^6^

##### ^1^Institute for Maternal and Child Health - IRCCS "Burlo Garofolo" -; ^2^University of Trieste, Trieste; ^3^Department of Pediatric Medicine, Division of Rheumatology, Bambino Gesù Children's Hospital, Rome; ^4^Pediatric Clinic , University of Brescia and Spedali Civili of Brescia, Brescia; ^5^Pediatric Rheumatology Regional Center, Department of Pediatrics, Antonio Perrino Hospital, Brindisi; ^6^UO Pediatria II, Institute "G. Gaslini", Genoa; ^7^Ospedale dei Bambini "G. Di Cristina”, Palermo; ^8^A. O. L. Sacco, Milan; ^9^Pediatric Rheumatology Unit, AOU Meyer, University of Florence, Florence, Italy

###### **Correspondence:** Andrea Taddio

**Introduction:** Chronic Non-Bacterial Osteomyelitis (CNO) is a non-infectious inflammatory disease characterized by uni- or multifocal bone lytic lesions. Symptoms include pain, local swelling and warmth. CNO mainly affects the metaphyses of the long bones, the pelvis, shoulder girdle and, less commonly, the spine and the mandible. Only four cases of CNO involving the cranium have been reported so far [1]

**Objectives:** To describe the prevalence and the clinical manifestations of CNO patients with neurocranium involvement among an Italian cohort of CNO patients.

**Methods:** This is a retrospective study. Charts of patients with CNO admitted to eight Pediatric Rheumatology centres were reviewed. Data collection included patient and family history, age at diagnosis, sex, number and location of bone lesions at onset, diagnostic imaging, laboratory data at presentation and at last visit, histological findings, comorbidity and response to treatments.

**Results:** Eighty-six patients were enrolled in the study. 31 were males and 55 were females. The mean age at diagnosis was 9.4 years (SD ± 3.3). Only 11 patients presented a monofocal involvement while 75 patients presented a multifocal disease at onset. 3 out of 86 patients (3.5%) presented a cranial involvement at onset. These three patients were females; the age of presentation was 2 years old for one patient and 12 for the last two. All of them presented multifocal lesions at onset (12, 20 and 13 lesions respectively) but none of them complained clinical symptoms attributable to skull involvement (headache, vomiting, visual impairment, focal neurological involvement, etc). Cranial involvement were detected only with bone scintigraphy and then confirmed by magnetic resonance imaging and/or computed tomography. 2 patients presented fever and two skin manifestations. Laboratory inflammatory indeces were increased in two patients. All underwent bone biopsy in another bone site confirming the diagnosis. All patients received NSAIDs, without response. Two patients received corticosteroid and then methotrexate reaching clinical remission. One patient received pamidronate without benefit and she still presents an active disease at the end of follow-up.

**Conclusion:** This is the first report of cranial involvement in a cohort of patients affected by CNO. Cranial involvement confirms to be a rare localisation and it seems to be associated with multifocal involvement. In our cohort, in contrast with the 4 patients reported in literature, all patients were asymptomatic. In case of cranial involvement radiological control may be needed during follow-up.

**Reference**

1. Foley MR, Moshfeghi DM, Wilson MW et al. Orbital inflammatory syndromes with systemic involvement may mimic metastatic disease. Ophthal Plast Reconstr Surg. 2003;19:324-7

**Disclosure of Interest**

None Declared

### P347 EXPANDING THE GENOTYPING OF PATIENTS WITH AUTOINFLAMMATORY SYNDROMES

#### Maria Tsinti^1^, Maria Zamanakou^2^, Gedeon Loules^2^, Vasiliki Dermentzoglou^1^, Elena Tsitsami^1^

##### ^1^Pediatric Rheumatology Unit, First Department of Pediatrics, University of Athens, Medical School, Children's Hospital Aghia Sophia, Athens; ^2^CeMIA SA, Larissa, Greece

###### **Correspondence:** Maria Tsinti

**Introduction:** Presentation of monogenic autoinflammatory (AIF) diseases is markedly heterogenous. Next Generation Sequencing (NGS) increases the possibility for the detection of variants associated with AIF manifestations. However, interpretation of NGS results in everyday clinical practice is difficult.

**Objectives:** To detect variants of inflammatory genes possibly associated with AIF diseases.

**Methods:** 41 children with symptoms compatible with AIF disease were genotyped by a custom NGS protocol analyzing 25 genes associated with monogenic AIF diseases (mean coverage >92%). Infections, immunodeficiencies and autoimmune diseases were excluded. Variants are reported according to the HGVS and the guidelines for the genetic diagnosis of hereditary recurrent fevers.

**Results:** Variants of uncertain or unknown significance (VOUS - VUS) detected in AIF genes are reported in Table 1. In 13/41 patients either common or benign, or no variants were detected. All 13 had been referred for Periodic Fever (PF) unresolved posttonsillectomy and unresponsive to steroids. In 9/13 fever eventually resolved, 1 is treated with colchicine and in 3/13 symptoms recur. In 16/41 patients with unspecific PF, variants of uncertain or unknown significance (VOUS - VUS) were detected (Table 1). In 10 out of the latter 16 patients fever resolved after >2 years follow-up; the other 6 are diagnosed with PFAPA. In 2/41 patients (5%) pathogenic variants were detected: (**1)** the p.Val726Ala *MEFV* variant was detected (along with a *TNFRSF1A* VUS) in a 4 year old male with recurrence of fever post tonsillectomy, who responded to colchicine. (**2)** the p.Val377Ile *MVK* variant was detected in a 4 year old male with recurrent fever, rash, failure to thrive, dutch ancestry, elevated urinary mevalonic acid and good response to steroids, (along with *PLCG2, NLRP7 & IL10RB* VUS). In the rest 10/41 patients VOUS and/or VUS were detected, alone or in combination, in genes correlated with their clinical phenotypes (FMF, HIDS, TRAPS, Blau, Inflammatory Bowel Disease, Table 1).

**Conclusion:** Expanding the genotyping of patients with AIF clinical phenotypes uncovers not yet reported or extremely rare variants of genes associated with AIF diseases. Our results provide evidence in favor of the pathogenicity of some of these variants. Combined expression of AIF variants observed in two patients with typical clinical picture carrying pathogenic *MEFV* and *MVK* variants along with VUS in other inflammatory genes indicate the complex genetic associations of these initially regarded as mendelian disorders.

**Disclosure of Interest**

None Declared

**Table 1 (abstract P347).** VOUS and VUS associated with AIF diseases that have been detected in our patients

*MEFV*	*MVK*	*NOD2*	*TNFRSF1A*	*NLRP12*	*LPIN2*	*PLCG2*	*IL10RA*	*IL10RB*
p.Glu148Gln ‡‡ p.Pro369Ser p.Ser339Phe	p.Thr356Met*	p.Met950Leu** p.Met513Thr† p.Arg791Gln †	p.Asn145Ser p.Arg568His p.Arg121Gln‡	p.Thr861Ser***	p.Cys874Phe p.Pro149Leu p.Pro626Ser p.Lys387Glu p.Glu601Lys p.Glu601Lys	p.Asn798Ser p.Ala313Gly p.Pro522Arg p.Ala420Thr	p.Glu58Lys P.Arg412Trp††	p.Val148Met
*AP1S3*	*CARD14*	*CECR1*	*NLRP7*	*RBCK1*	*SH3BP2*	*SLC29A3*	*TMEM173*	
p.Phe4Cys	p.Met931Lys p.Ser200Asn p.Arg826Tr	p.Met267Ile	p.Arg156Gln Cys399Tyr	p.Gly242Val p.Pro347Arg	p.Ala212Val p.Arg534Trp	p.Arg83His p.Glu331Lys	p.Ala313Thr p.Gly192Val	

*No variants were detected in the following tested genes: IL1RN, NLRP3, PSMB8, PSTPIP1, IL10, IL36RN, NLRC4*

‡‡ 2 patients with FMF, 2 patients with resolved PF; *HIDS; **IBD; †Blau, same patient; ‡TRAPS; ***infantile Raynaud’s Phenomenon; ††IBD

### P348 BLAU SYNDROME IN A FEMALE PATIENT ASSOCIATED WITH TWO NOD2 VARIANTS

#### Maria Tsinti^1^, Vasiliki Dermentzoglou^1^, Maria Zamanakou^2^, Elena Tsitsami^1^

##### ^1^Pediatric Rheumatology Unit, First Department of Pediatrics, University of Athens, Medical School, Children's Hospital Aghia Sophia, Athens; ^2^CeMIA SA, Larissa, Greece

###### **Correspondence:** Maria Tsinti

**Introduction:** Blau syndrome is a rare granulomatous autoinflammatory disorder. Optimal response to anti-TNFa treatment has been reported, however therapeutic outcomes are diverse probably due to functional differences in *NOD2* mutations.

**Objectives:** To report the clinical picture of Blau syndrome in a patient carrying 2 *NOD2* variants and the response to anti-TNFa treatment with adalimumab.

**Methods:** Case Report.

**Results:** A 19 month old girl was referred to the pediatric rheumatology department from the dermatology department for an 8-month history of generalized ichthyasiform skin eruption. The rash appeared on the age of 11 months on the shoulders and then spread to the knees trunk and face. Multiple coalescing generalized slightly scaly red to violaceous flat-topped papules were present on examination. Her sclerae were mildly injected. Skin biopsy had already been performed revealing granulomatous dermal inflammation; granulomas contained epithelioid cells, langerhans-type multinucleated giant cells, a few lymphoid cells, without evidence of necrosis and perivascular lymphomononuclear infiltration around dermal vasculature. Other causes of granulomatous inflammation i.e fungal and mycobacterial infections, CVID, CGD and Jobb’s syndrome were excluded. Clinical examination revealed significant boggy edema of her wrists and ankles, knees and fingers and contractures of both elbows. Meticulous history revealed the presence of morning stiffness since 6 months. Musculoskeletal ultrasonography corroborated the presence of arthritis in these sites and revealed the presence of tenosynovitis in the wrists and ankles. Onycholysis on the 4^th^ and 5^th^ right and the 4^th^ left finger was noticed. The presence of granulomatous skin inflammation and polyarthritis raised the suspicion of BLAU syndrome; ophthalmologic examination revealed nummular keratiris with subepithelial opacities but absence of anterior or posterior chamber inflammation. Her growth and development were normal. Family history was negative. Laboratory findings were: mild leukocytosis (WBC=17040/uL), mild anemia (Hb=9,6 mg/dl), mild increase of inflammatory markers (ESR=40 mm/hr, CRP= 19mg/dl), elevated IgG (1470 mg/dl) and IgE (271 IU/ml), normal SACE (37,4 IU/L) and absence of autoandibodies and RF. Serum calcium and urine Ca/Cr ratio were normal. Direct and Indirect Agglutination Tests were positive (for IgG). Chest XRay and abdominal US were normal. Next generation sequencing for genes of inherited autoinflammatory diseases revealed the presence of 2 Variants of *NOD2* (both of Uncertain Significance): c.1538T>C (p.Met513Thr) and c.2372G>A (p.Arg791Gln). Treatment with intravenous methylprednisolone pulse (30 mg/kg/3days) followed by oral prednisolone gradually tapered over 4 months and topical eye steroids were given. Subcutaneous Methotrexate-MTX was initiated. Rash waned in the 1^st^ month of treatment. Due to persistence of polyarthritis after 3 months of MTX treatment adalimumab 24mg/m^2^/2weeks was added. After 8 months of MTX and 5 months of adalimumab treatment and 4 months after discontinuation of steroids arthritis, keratitis and rash have remitted; mild elevation of ESR=45mm/hr persists.

**Conclusion:** This female toddler with Blau syndrome with onset in infancy had optimal response to methotrexate and adalimumab treatment and no severe eye involvement. The concomitant presence of 2 *NOD2* variants is not frequently reported in Blau patients. The 1538T>C (p.Met513Thr) has been previously detected in heterozygocity in a patient with Blau syndrome and found to cause constitutive NFκB activation whereas the c.2372G>A (p.Arg791Gln) that has been associated with spondyloarthropathy possibly explains the presence of onycholysis on hand nails, a manifestation not characteristic of Blau syndrome. Informed consent to publis had been obtained.

**Disclosure of Interest**

None Declared

### P349 FMF CASES PRESENTING ONLY WITH ARTHRITIS: IS THERE ANY DIFFERENCE?

#### Serkan Turkucar^1^, Hatice Adiguzel Dundar^1^, Ceyhun Acari^1^, Ozge Altug Gucenmez^2^, Balahan Bora Makay^3^, Sevket E. Unsal^4^

##### ^1^Department of Pediatrics, Pediatric Rheumatology Unit, Dokuz Eylul University Faculty of Medicine; ^2^Depatment of Pediatrics, Pediatric Rheumatology; ^3^Department of Pediatrics, Pediatric Rheumatology, Izmir Dr Behcet Uz Children's Hospital; ^4^Department of Pediatrics, Pediatric Rheumatology, Dokuz Eylul University, Faculty of Medicine, izmir, Turkey

###### **Correspondence:** Serkan Turkucar

**Introduction:** Familial Mediterranean Fever (FMF) is the most frequent autoinflammatory disease in eastern Mediterranean. It is mostly presented with recurrent fever, peritonitis, pleuritis and arthritis. Some patients might present with only arthritis during episodes.

**Objectives:** This study aimed to examine the differences between typical FMF attacks and cases with only arthritis.

**Methods:** The charts of 890 pediatric FMF cases were reviewed. There were 15 cases presented with only arthritis. Demographic and laboratory data were compared between the 2 groups.

**Results:** The mean age of 15 cases presenting with only arthritis was 13.83 (5.41-17.91) years, 5 were male (33.3%) and 10 were female (66.6%). At onset of symptoms, the mean age of cases with arthritis was 8.16 (1.16-15) years and was statistically higher than the control group (p<0.05). There were no statistically significant differences in age at diagnosis, delay at diagnosis and family history between two groups. The mean platelet volume (MPV) and neutrophil / lymphocyte ratio (NLR) values reflecting subclinical inflammation in attack-free period also did not differ statistically between the two groups. The groups did not differ in having either M694V or exon 10 mutations. Patients with only arthritis did not have proteinuria, colchicine unresponsiveness and indication for biologics.

**Table:** 

	FMF cases with only arthritis n=15	Typical FMF cases n=890	p value
Sex	%33.3 m (n=5) %66.6 f (n=10)	%51.3 (456) m %49.7 (434) f	p>0.05
**Onset age of symptoms (year)**	**8.16 (1.16-15)**	**4.5 (0.16-16.6)**	**p<0.05**
Age of Diagnosis (year)	10.8 (2.66-15)	5.05 (1-17.5)	p>0.05
Delay Times (year)	1.08 (0-9.33)	1.25 (0-17)	p>0.05
Exon-10 Mutation	%66.7	%66.6	p>0.05
M694V Mutation	homozygous %13.3 (n=2) heterozygous %33.3 (n=5)	homozygous %12.9 (n=113) heterozygous %38.1 (n=333)	p>0.05
Colchicine Responsiveness and indication for Biological Agents	0	%2.5	p>0.05
NLR	1,33 (0,1-6,8)	1,23 (0,1-23,46)	p>0.05
MPV	8,30 (7,6-10,40)	8,20 (5,80-12,60)	p>0.05

**Conclusion:** Recurrent fever, abdominal pain, chest pain, and arthritis are major clinical symptoms of FMF. However; there is a certain group presenting only with arthritis, and continue to have only articular symptoms throughout the disease. They have a milder disease course without proteinuria, colchicine unresponsiveness and indication for biologics. They have different origins from all over the Anatolia, but they live in the Aegean region. Previous studies done with monozygotic twins born in Anatolia who have same MEFV mutations showed that the ones raised in Europe had milder disease course. The difference between patients only with arthritis and the other group could be attributed to living in western Anatolia, and having Mediterranean type diet. Further studies with larger group of patients all over the country are needed to support this hypothesis.

**Disclosure of Interest**

None Declared

### P350 MALIGNANT PERITONEAL MESOTHELIOMA IN YOUNG ADULT WITH CAPS MUTATION AND AUTOINFLAMMATORY SYMPTOMS: CASE STUDY

#### Clara Udaondo, Agustin Remesal, Sara M. Murias, Rosa M. Alcobendas

##### Pediatric Rheumatology, University Hospital La Paz, Madrid, Spain

###### **Correspondence:** Clara Udaondo

**Introduction:** Differential diagnosis of autoinflammatory syndrommes may include solid tumours. Malignant peritoneal mesotheliomas represent less tan 10% of mesotheliomas, and they are exceptional in younger tan 40 years.

**Objectives:** We present a specially challenging case of a malignant peritoneal mesothelioma diagnosed in a 17 year old boy with clinical presentation and genetic compatible with autoinflammatory syndrome, its management and outcome.

**Methods:** Review of his medical chart and literature search

**Results:** At 12 years of age the patient presented with a two month history of evening fever, loss of weight, elevation of acute phase reactants and captation of multiple supra and infra diafragmatic adenopathies in PET-TC. Infectious and malignant causes were discarded initially. Genetic testing of hereditary autoinflammatory syndrommes was performed, showing heterozygose V-198-M mutation in NLRP3 gene – compatible with CAPS syndromme, and he initially responded well to corticosteroids. IL-1 blockade (anakinra) was administered without any success. He was then empirically treated with intravenous IL-6 blockade (tocilizumab) with complete response, and remained asymptomatic for 5 years. Treatment was then switched to subcutaneous IL-6 blockade with reappearance of his symptoms. PET-TC revealed multiple adenopathies and addition of retroperitoneal nodules. Exploratory laparotomy and biopsies were performed, showing methastatic peritoneal mesotelioma. BAP-1 gene was tested showing no mutation. The patient is still under chemotherapy following our hospital protocoles.

**Conclusion:** The differential diagnosis of CAPS syndrome is wide, including rare solid tumors among other conditions. There are some peculiarities that made this specially challenging, such as the age of the patient, fever and adenopathies at onset, indolent clinical course and genetic testing supporting the diagnostic of presumption of CAPS syndrome. This is the first published case of malignant peritoneal mesothelioma with the presence of V-198-M mutation in NRLP3 gene. The absence of response to IL-1 blockade (anakinra) was against the diagnosis of CAPS. IL-6 blockade has been effective in this single patient, being to our knowledge the only malignant peritoneal mesothelioma case who has been reported as respondent to tocilizumab. Informed consent to publish had been obtained from the parent.

**Disclosure of Interest**

None Declared

### P351 EVALUATION OF THE CURRENT DISEASE SEVERITY SCORES IN PEDIATRIC FAMILIAL MEDITERRANEAN FEVER

#### Gizem Ozcan^1^, Şemsa Caycı^2^, Nermin Uncu^3^, Ozge Basaran^1^, Fatma Aydın^4^, Atilla Elhan^5^, Nilgun Cakar^4^

##### ^1^Department of Pediatric Rheumatology; ^2^Department of Pediatric Nephrology; ^3^University of Health Sciences, Ankara Child Health and Diseases Hematology Oncology Training and Research Hospital; ^4^Department of Pediatric Rheumatology; ^5^Biostatistics, Ankara University School of Medicine, Ankara, Turkey

###### **Correspondence:** Nermin Uncu

**Introduction:** Familial Mediterranean fever (FMF) is an autosomal recessive disease, characterised by recurrent, self-limited attacks of fever and serositis. In addition to the Pras and Mor disease severity scoring systems defined for adult FMF patients, the International Severity Scoring System (ISSF), recently described for children, is also used to assess disease severity.

**Objectives:** To determine the disease severity of our patients according to Pras, Mor and (ISSF) scoring system , and to evaluate the consistency of these three systems.

**Methods:** The clinical and demographic data of 205 patients who met the FMF diagnostic criteria were evaluated retrospectively. All patients screened for MEFV mutations. Patients grouped as homozygous for M694V mutation, homozygous for other than M694V, or compound heterozygous, heterozygous for any mutant alleles, and with no mutation. The disease severity scores of all patients and each groups were assessed by Pras, Mor and ISSF scoring systems. The compatibility of the disease severity scoring systems was investigated.

**Results:** According to Pras, Mor and ISSF scores 29.8%, 44.4% and 6.3% of the patients had severe disease respectively. While the majority of patients (66% and 64%) had moderate disease severity according to Pras and ISSF scores, this rate was low in the Mor (17%). Pras, Mor and ISSF scores were inconsistent with each other, and it was a poor fit between them (generalized Kappa: 0,140 ± 0,029; p <0,001).

When the disease severity were evaluated according to mutations, M694V homozygous patients had higher disease severity score than the other groups in all scoring systems (p<0.001). According to Pras, Mor and ISSF scoring systems 58%, 62% and 12% of the patients with homozygous M694V mutations had severe disease, respectively. Findings, such as leg pain, elevated acute phase reactants at attack free period, pyuria and proteinuria which was used in ISSF-score, were found 39%, 18%, 13% and 5% of patients respectively.

**Conclusion:** Pras, Mor and ISSF scores determine the disease severity by evaluating different aspects of the disease. Unlike the others, ISSF Scoring system evaluate chronic sequelaes like as growth retardation, anemia and splenomegalia besides amyloidosis, organ dysfunction, duration of attacks, prolonged elevated acute fase reactants after attacks, leg pain and etc. The benefit of the assessment of disease severity according to ISSF scoring system for the treatment planning of FMF patients should be supported by further clinical trials.

**Disclosure of Interest**

None Declared

### P352 CLINICAL SIGNIFICANCE OF SOLUBLE TNF RECEPTOR I/II RATIO FOR THE DIFFERENTIAL DIAGNOSIS OF TUMOR NECROSIS FACTOR RECEPTOR-ASSOCIATED PERIODIC SYNDROME FROM OTHER INFLAMMATORY DISEASES

#### Junko Yasumura^1^, Masaki Shimizu^2^, Masao Kobayashi^1^, Akihiro Yachie^2^

##### ^1^Pediatrics, Hiroshima University Graduate School of Biomedical & Health Sciences, Hiroshima; ^2^Pediatrics, Graduate School of Medical Sciences, Kanazawa University, Kanazawa, Japan

###### **Correspondence:** Junko Yasumura

**Introduction:** Tumor necrosis factor receptor-associated periodic syndrome (TRAPS) is an autosomal dominantly inherited autoinflammatory syndrome caused by mutations in *TNFRSF1A*. Symptoms of TRAPS are recurrent fever, abdominal pain, myalgia, exanthema, arthralgia/arthritis, and ocular involvement. The clinical features and laboratory parameters of patients with TRAPS and other autoinflammatory syndromes including systemic juvenile idiopathic arthritis (s-JIA) tend to overlap, particularly in patients with atypical genetic mutations and symptoms. Although the pathogenesis of TRAPS remains unknown, one of possible mechanisms of TRAPS is impaired TNFR1 proteolytic cleavage and receptor shedding.

**Objectives:** To investigate clinical significance of serum soluble TNF receptor I level and soluble TNF receptor I/II ratio for the differential diagnosis of TRAPS and other autoinflammatory syndromes.

**Methods:** Thirteen active episodes from 4 patients with TRAPS, 156 patients with active s-JIA, and 63 with active Kawasaki disease (KD), 23 active episodes from 15 patients with Familial Mediterranean fever (FMF), along with 18 healthy controls (HC) were analyzed. Serum soluble tumor necrosis factor receptor (sTNFR) I and II levels were measured using enzyme-linked immunosorbent assay.

**Results:** Serum sTNFRI levels in patients with s-JIA (median 2760 pg/ml, range 900-8250 pg/ml) and KD (2520, 1240-6200) were significantly elevated compared to TRAPS (920, 540-1900), FMF (1230, 580-11000) and HC (835, 600-1280). Those levels in patients with TRAPS and FMF were not elevated compared to HC. sTNFRI/II ratio in patients with s-JIA (0.392, 0.127-1.169), KD (0.333, 0.161-0.681) and FMF (0.382, 0.252-0.618) were significantly elevated compared to TRAPS (0.276, 0.126-0.391) and HC (0.275, 0.210-0.356). The receiver operating characteristic curve analysis indicated that the cutoff for sTNFRI/II ratio to differentiate TRAPS from other autoinflammatory diseases was 0.305 with a sensitivity of 73.6% and a specificity of 76.9%.

**Conclusion:** These findings indicate that sTNFRI/II ratio <0.305 might be useful for the differential diagnosis of TRAPS and other autoinflammatory diseases. Low level of serum sTNFRI and sTNFRI/II ratio in TRAPS might be associated with impaired TNFR1 proteolytic cleavage.

**Disclosure of Interest**

None Declared

### P353 CLINICAL-ANALYTICAL CHARACTERIZATION OF 52 DIAGNOSED PATIENTS OF BEHÇET DISEASE WITH INCLUSION OF PEDIATRIC CASES IN A SPANISH TERTIARY HOSPITAL

#### Sixto Zegarra Mondragon, Alina Boteanu, Adela Alia, Monica Vazquez

##### Rheumatology, Hospital Ramon y Cajal (Spain), Madrid, Spain

###### **Correspondence:** Sixto Zegarra Mondragon

**Introduction:** Behçet's disease (BD) is a chronic and recurrent inflammatory disease of unknown etiology, classified into polygenic autoinflammatory diseases or variable vessel vasculitis. It has a wide spectrum of symptoms with a very variable range of severity, from mucocutaneous involvement to neurological manifestations, systemic vascular or severe ocular manifestations. About 5.4 – 7.6% of Behçet's cases have a pediatric debut.

**Objectives:** To evaluate and compare the clinical and laboratory manifestations of a series of 52 patients, adults and children, diagnosed with BD according to the classification criteria of the International Study Group of BD (ISGBD-1990).

**Methods:** Retrospective cross-sectional observational study, which included 43 adult patients and 9 pediatric patients diagnosed with EB in the Rheumatology Department of a Madrid tertiary hospital. The clinical-analytical characteristics of both groups were evaluated, as well as the correlation of HLA-B51 with the described symptomatology.

**Results:** The mean age at diagnosis of BD was 36.9 ± 11.8 years in adults and 11.4 ± 5.1 years in children. 27.3% of adults and 11.1% of children with BD were male, with oral ulcers close to 90% in both groups. Contrary to what was reported in other series, genital ulcers were more frequent in children (77.8% versus 65.9% of adults), as was the presence of uveitis (44.4% in children compared to 22.7% in adults) and neurological manifestations (22.2% in children versus 6.8% in adults). Joint involvement was also more frequent in children (88.9% versus 52.3% in adults), as well as fever (44% in children versus 14% in adults); being these two manifestations the only parameters that were associated in a statistically significant way with their presentation in the pediatric age in BD. In contrast, skin involvement and vascular manifestations were more frequent in adults. The positivity of HLA-B51 did not correlate statistically with any clinical manifestation, but those who had it had a mean age at diagnosis of 26.5 years compared to a mean of 39 years in those who did not present this genetic marker.

**Conclusion:** Behçet's disease presents with a wide spectrum of clinical manifestations, potentially serious, ranging from skin lesions to neurological or vascular manifestations. In our series, patients diagnosed at pediatric age most frequently had systemic manifestations (fever), arthritis or severe clinical manifestations such as neurological involvement or uveitis. Limitations: a small number of pediatric cases included in our study.

**Disclosure of Interest**

None Declared

## Poster walk 8: JIA treatment

### P354 DO CHILDREN WITH JUVENILE IDIOPATHIC ARTHRITIS PLAY AN ACTIVE ROLE IN THEIR TREATMENT ADHERENCE? FIRST RESULTS OF THE RUMAJI STUDY.

#### Guillaume Montagu^1^, Ellie Mevel^1^, William Fahy^2^, Linda Rossi-Semerano^3^, Elisabeth Solau-Gervais^4^, Sonia Tropé^5^, Jean-David Cohen^6^

##### ^1^Research, _unknowns; ^2^Administrateur, Kourir, Paris; ^3^Pediatric Rheumatology, CHU de Bicêtre, Le Kremlin-Bicêtre; ^4^Rheumatology, CHU de Poitiers, Poitiers; ^5^Director, ANDAR, Paris; ^6^Rheumatology, CHU Lapeyronie, Montpellier, France

###### **Correspondence:** Jean-David Cohen

**Introduction:** Adherence to DMARDs such as methotrexate and biologics is critical for patients with Juvenile Idiopathic Arthritis (JIA). Notwithstanding, few studies exists on that topic and we lack information to understand the grounds for adherence.

**Objectives:** The RUMAJI study aims, among others, to understand and decipher the parents and children adherence mechanisms and practices.

**Methods:** Qualitative methods were chosen in order to investigate parents’ and children’s everyday life with JIA and its treatment. An ethnographic study was designed by a multidisciplinary team including rheumatologists, pediatricians, patient associations members and anthropologists. Parents, children and doctors were interviewed. The study involved 6 doctors (3 paediatricians and 3 rheumatologists) and 15 families (enough to reach saturation), recruited from 5 centers, by diversity of clinical and sociological profiles. The panel included 17 children with JIA, 11 girls and 6 boys, median age 10 [3 ; 17], median disease duration 2.5 [1 ; 15]. 4 children were treated with conventional DMARDs in monotherapy, 4 with biologic DMARDs in monotherapy, 5 with cDMARD-bDMARD association and 4 with NSAIDs only.

Doctors interviews were conducted first. Parents and children interviews were conducted by anthropologists at family’s home using in-depth semi directive and biographic methods. 3 fields were explored: organization of everyday life with JIA, treatment practices, impact on school and social activities. Interviews were recorded and transcribed for analysis.

**Results:** Adherence results from an appropriation process of the JIA and treatment that require both an active role from parents and children, even before the transition. The setting of a partnership-based doctor-children-and-parents relationship has also a positive effect in the family active role. The active role played by children could be either stimulated or inhibited at home according to the family’s structure, social background, parents’ attitudes toward their child (participation to the decision, explanation of the disease).

Children’s active role includes in particular: 1) negotiations with parents and physicians, 2) experiments with the treatment (forgetting or involuntary switch from the parents, changing the dosage on their own initiative) and 3) participation to the treatment administration and ritualization.

The manner children consider and manage their DMARDs is the result of an arbitration depending on the positive (a) and side effects (b) they felt in their body and the effects noted by the doctors (c) during the examinations and test results. Dealing with these 3 dimensions requires to link together both a theoretical and practical knowledge of JIA. Thus, children build their own and singular knowledge of their disease and treatment, which is a source of control of their body and their life.

**Conclusion:** Qualitative methods, through an ethnographic study starting from children, parents and doctors point of view, underline the active role they played by children in their care. Adherence to DMARDs could be improved by supporting children’s implication as soon as the beginning of JIA.

Acknowledgements

This work was supported by an institutionnal grant from NORDIC Pharma to ANDAR. All medical experts and patients volunteered.

**Disclosure of Interest**

G. Montagu Consultant for: Roche, E. Mevel: None Declared, W. Fahy: None Declared, L. Rossi-Semerano: None Declared, E. Solau-Gervais: None Declared, S. Tropé: None Declared, J.-D. Cohen: None Declared

### P355 BIOSIMILAR USE IN CHILDREN AND YOUNG PEOPLE WITH JUVENILE IDIOPATHIC ARTHRITIS IN A REAL-WORLD SETTING IN THE UNITED KINGDOM

#### Diederik De Cock^1^, Lianne Kearsley-Fleet^1^, Eileen Baildam^2^, Michael W. Beresford^3,4^, Helen Foster^5^, Taunton Southwood^6^, Wendy Thomson^7^, Kimme Hyrich^1,8^, BCRD investigator group

##### ^1^Arthritis Research UK Centre for Epidemiology, University of Manchester, Manchester; ^2^Clinical Academic Department of Paediatric Rheumatology, Alder Hey Children's NHS Foundation Trust; ^3^Alder Hey Children's NHS Foundation Trust Hospital; ^4^Institute of Translational Medicine (Child Health), University of Liverpool, Liverpool; ^5^ Great North Children's Hospital, Newcastle University, Newcastle; ^6^School of Immunity and Infection, Institute of Clinical Sciences, University of Birmingham, Birmingham; ^7^Arthritis Research UK Centre for Genetics and Genomics, University of Manchester, Manchester Academic Health Science Centre; ^8^National Institute of Health Research Manchester Musculoskeletal Biomedical Research Centre, Central Manchester NHS Foundation Trust, Manchester Academic Health Science Centre, Manchester, UK

###### **Correspondence:** Diederik De Cock

**Introduction:** Most research concerning biosimilar use in musculoskeletal diseases has been done in adults. Little to no data regarding the use of biosimilars in Children and Young People (CYP) with juvenile idiopathic arthritis (JIA) exist.

**Objectives:** To describe the characteristics of CYP with JIA starting biosimilars in the United Kingdom (UK) following their approval in the UK with musculoskeletal diseases.

**Methods:** The Biologics for Children with Rheumatic Diseases (BCRD) is an ongoing prospective UK study recruiting CYP with JIA starting biologic therapies other than Enbrel (followed in a separate parallel study) from 2010 onwards. Baseline information is collected via questionnaires completed by the treating physician or affiliated clinical research nurse. Follow-up data including disease activity measures and changes in drug therapy are collected at 6 months, 1 year and annually thereafter. From 30-Sept-2015, data has been captured on infliximab and etanercept biosimilars available in the UK.

**Results:** To 15 April 2018, 71 patients in total were identified in the BCRD study starting a biosimilarof which 53 (74%) starting an infliximab biosimilar and 18 (25%) an etanercept biosimilar (Table 1). Of these, 45 (63%) started a biosimilar as their first biologic therapy. Seven (10%) patients switched from their originator. The median (IQR) time on originator before switch was 1.6 (0.7-4.1) years. Nineteen (27%) switched to an infliximab biosimilar from a non-originator biologic with 12 switching for efficacy reasons and 7 for safety reasons. The majority (n=14) switched from adalimumab. Active uveitis was present in 10 patients at the point of starting a biosimilar. Follow-up data out to 2 years were available in 26 patients. Seven patients switched to another biologic in this period: four infliximab biosimilar patients switched to tocilizumab and 2 infliximab biosimilar patients switched to adalimumab. One patient switched back from etanercept biosimilar to originator due to increased pain. Infliximab biosimilars were interchanged in 6 patients due to changes in hospital procurement. No serious adverse events were reported. Two cases of recurrent uveitis in patients on an infliximab biosimilar switching from a non-originator biologic were noted.

**Conclusion:** Biosimilars in children and young people with JIA are being used as both first-line and subsequent-line biologic therapy. Currently few UK children have been switched directly from an originator product. Whether a move towards switching all children receiving originator products to biosimilar products, as has been seen in rheumatoid arthritis, will occur is currently unknown but it is imperative that the safety of these treatment decisions are captured in patient registers.

**Disclosure of Interest**

None Declared

**Table 1 (abstract P355).** See text for description

	Biological naïve (n=45)	Switched from a non-originator (n=19)	Switched from originator (n=7)
Biosimilar			
infliximab	30 (67%)	19 (100%)	4 (57%)
etanercept	15 (33%)	0 (0%)	3 (43%)
Age (years) at biosimilar start	13 (10-15)	14 (11-15)	15 (12-16)
Disease duration (years)	2 (1-5)	9 (3-12)	7 (2-13)
Active Joints (71 joints)	4 (1-7)	1 (0-2)	0 (0-0)
Limited Joints (71 joints)	4 (1-7)	2 (0-4)	0 (0-1)
Patient Global (0-10cm)	7 (4-8)	2 (0-4)	2 ( 1-5)
CHAQ (0-3)	2 (1-3)	0 (0-1)	0 (0-1)
Active uveitis at start of therapy	0 (0%)	10 (56%)	1 (17%)

### P356 ADVERSE EVENTS OF SPECIAL INTEREST UPON BIOLOGICS FOR TREATMENT OF JUVENILE IDIOPATHIC ARTHRITIS

#### Fabian Ebach^1^, Ariane Klein^1^, Gerd Ganser^2^, Ivan Foeldvari^3^, Kirsten Minden^4^, Anton Hospach^5^, Gerd Horneff^1^

##### ^1^ASKLEPIOS, Sankt Augustin; ^2^Sankt Joset Stift, Sendenhorst; ^3^H amburg Centre Ped Rheumatol, Hamburg; ^4^DRFZ, Berlin; ^5^Olga Hospital, Stuttgart, Germany

###### **Correspondence:** Fabian Ebach

**Introduction:** The Biker registry aims to provide long term surveillance of biologic drugs which is particularly important in pediatric patients (pts) who may require prolonged treatment

**Objectives:** To evaluate long-term rates of serious adverse events (AE) and AEs of special interest (AESI) in clinical practice in pediatric pts with juvenile idiopathic arthritis (JIA).

**Methods:** Safety data from pts treated with biologics and registered in the German BIKER registry were analyzed. In addition to seriousness of AE, 25 AESI were defined including malignancy, serious and medically important infection, uveitis, chronic inflammatory bowel disease, cytopenia, hepatic event, anaphylaxis, depression, macrophage activation syndrome, pregnancy and death. Events per 100 patient-years (PY) of exposure were calculated using AEs reported after first dose through 70 days after last dose. Rates were compared by Wald test.

**Results:** A total of 3975 courses of biologics with a total exposure time of 7592 PY were identified with Etanercept (ETA, 5376 PY), Adalimumab (ADA, 1334 PY), Tocilizumab (TOC,435 PY), Abatacept (ABA, 109 PY), Infliximab (INF, 99 PY ), Anakinra (ANA, 96 PY), Canakinumab (CAN, 71 PY) and Golimumab (GOL, 67 PY). Differences in JIA category distribution and concomitant treatment between these cohorts were noted. A total of 3586 (47.2/100 years) AE, 461 (6.1) SAE and 629 (8.3) AESI were reported. The most common AESI were uveitis (194(2.6)) followed by medically important infections (155(2.0)), cytopenias (62(0.8)), hepatic events 39(0.5), anaphylaxis (28 (0.4))), other autoimmunopathies (25 (0.3)), chronic inflammatory bowel disease (CED, 23 (0.3)), depression (17 (0.2)), macrophage activation syndrome )12 (0.2)), malignancies (8(0.1)) and pregnancies (8(0.1)). There were marked differences in the rate of AESI in the cohorts with different biologics. Uveitis reports were most common in TNF-antibody treated cohorts, infections upon GOL, TOC and ANA, cytopenias upon TOC and CAN, hepatic events upon TOC, anaphylaxis upon INF and TOC, CED upon ETA and INF (Table 1). One case of latent TB but no further opportunistic infections were reported. There was a single death upon treatment due to sepsis in a ETA patient.

**Conclusion:** Surveillance of pharmacotherapy as provided by BIKER is an import approach especially in the case of long term treatment of children. These data provide support for the long-term and comparative safety of biologics in JIA pts. Overall, tolerance is acceptable. Differences between several biologics were noted and should be considered in daily patient care.

**Disclosure of Interest**

None Declared

**Table 1 (abstract P356).** See text for description.

AESI	ETA 5376 PY	ADA 1334 PY	GOL /2PY	INF 99PY	ABA 109 PY	TOC 435 PY	ANA 97 PY	CAN 71 PY	All 7592 PY
Uveitis, n; RR; p	106;2,01;p<0,0001	70;2.64;p<0.0001	5;2,77;p=0.025	9;3,69;p=0,0001	2;0,71; n.s.	2;0,17; p=0,013	0	0	194
Infection, n;RR; p	90;0.57;p=0,0005	32;1,22; n.s.	6;4.21; p=0.0005	3;1,5; n.s.	1,0,44; n.s.	18;2.17; p=0.002	4;2,06; n.s.	1;0,68; n.s.	155
Cytopenia, n ; RR; p	30;0.39;p=0,0002	1;0,07; 0=0,011	0	1,1,24; n.s.	0	22,9.07;p<0.0001	2,2,59;n.s.	6;11.35;p<0.0001	62
Hepatic Event, n ; RR; p	17;0.32;p=0.0004	6,0,85;n.s.	0	1,2,0;n.s.	1,1,8;n.s.	13;8.25;p<0.0001	0	1,2,78;n.s.	39
Anaphylaxis, n ; RR; p	3;.05[;p<0.0001	3,0,52;n.s.	1;3,62;n.s.	5,15.1;p<0.0001	2,4.90;p=0.030	15;16.49;p<0.0001	0	1;3,65;n.s.	30
Autoimmunopathy, n ; RR; p	8,0.19;p=0,0001	10;3.13;p=0,005	1;4,37;n.s.	1;3,17;n.s.	2,5.97;p=0.015	3,2,24;n.s.	0	0	25
CED, n; RR; p	19;1.96;n.s.	2;0,45;n.s.	0	2;7.21;p=0.008	0	0	0	0	23
MAS, n ;RR; p	2,0.08;p=0.001	0	0	0	0	616.49;p<0.0001	17,05;n.s.	335.3;p<0.0001	12
Malignancy, n; RR ;p	6,1.23;n.s.	0	0	1,10,9; p=0,026	0	1,2,35;n.s.	0	0	8

### P357 JOINT INJECTION PRACTICES IN PEDIATRIC RHEUMATOLOGY - RESULTS FROM A GLOBAL SURVEY

#### Anita Dhanrajani^1^, Raju Khubchandani^2^, Acknowledgements: PRES and CARRA/PRCG

##### ^1^Pediatric Rheumatology, Hospital for Sick Children, Toronto, Toronto, Canada; ^2^Pediatric Rheumatology, Jaslok Hospital and Research Centre, mumbai, India

###### **Correspondence:** Raju Khubchandani

**Introduction:** Corticosteroid joint injections are a routine procedure in pediatric rheumatology. There is a dearth of existing literature on joint injection practices. The authors conducted an online survey targeting pediatric rheumatologists across the globe, to highlight variations and areas of consensus in joint injection practices.

**Objectives:** 1. To describe global variations in intra-articular injection practices.

2. To explore the relation of these variations with physician demographic features

**Methods:** An electronic survey consisting of 22 questions, divided in two sections based on patient age (0-5 years, > 5 years) was designed on the web app – Survey Monkey. The questions addressed practices related to pre-procedural sedation, choice of anesthetic agent, dose and type of steroid molecule, number of joints injected, use of ultrasound, and post procedure complications. The survey was distributed via email to physicians registered in the PRINTO and CARRA/PRCSG networks. Analysis was performed using GraphPad Prism version 6.

**Results:** 310/358 respondents (87%) routinely performed intraarticular injections. Majority performed the procedure in an outpatient procedure room, day care or minor operation theatre (85%). Almost 80% injected more than one joint per sitting (median:4, mode:2). 52% used ultrasound to guide joint injections either routinely or selectively. Ultrasound was reported to be most useful for the hip joint. Ignoring availability, the molecule of choice for joint injections was Triamcinolone hexacetonide (78%). More than 50% respondents reported that Triamcinolone hexacetonide was either not available or sporadically available in their country. The dose of steroid used for large and small joint injections was 1 mg/kg and 0.5 mg/kg respectively (64% and 55% respectively). 68% of respondents used pre-procedure local anesthesia (LA): either EMLA cream, subcutaneous lidocaine, or a combination. Additionally, short anesthesia (49%) and oral sedation (19%) were offered. 80% stated that the most commonly observed complication was local subcutaneous atrophy. Post-procedure, most respondents monitored the patient in the hospital until the effect of anesthesia subsided (77%). Less than half the respondents (47%) reported following significantly different practices for the <5year age group. The commonest age dependent practice was choice of anesthesia in 72%. With regards to the physician characteristics, 60% had received a formal training in joint injections, and 49% had more than 15 years of pediatric rheumatology clinical practice.

The variations in practices based on geographical location of the respondents and years of training was analysed with chi-square and Fishers exact tests. In the USA 47% of respondents used pre-procedure LA, compared to 75% in the UK, and this difference was significant (Chi-square=4.540, df=1, p=0.033). None of the other practices varied significantly by geographical location. The practices were not significantly different in physicians with differing number of years of clinical experience. 67% of physicians who had received formal training followed different practices for 0-5-year age group whereas none of those without formal training did. This difference was statistically significant with Fishers exact test (p<0.0001).

**Conclusion:** There was consensus (about 80%) in some joint injection practices like location of joint injection, multiple joints being injected in one sitting, and choice of steroid molecule. However, the dose of steroid, choice of sedation, post procedure activity limitation, and use of Ultrasound was highly variable. Some of these variations may be attributed to geographical location of training and presence or absence of formal training. There is a definite need for developing uniform global consensus plans for intraarticular joint injection practices.

**Disclosure of Interest**

None Declared

### P358 AN INTERIM REPORT OF THE CHILDHOOD ARTHRITIS AND RHEUMATOLOGY RESEARCH ALLIANCE (CARRA) START TIME OPTIMIZATION OF BIOLOGIC THERAPY IN POLYARTICULAR JIA (STOP-JIA) STUDY

#### Sarah Ringold^1^, George Tomlinson^2^, Pamela F. Weiss^3^, Laura E. Schanberg^4^, Brian Feldman^5^, Mary Ellen Riordan^6^, Vincent Del Gaizo^7^, Anne Dennos^8^, Katherine Murphy^7^, Yukiko Kimura^9^ and The CARRA Registry Investigators

##### ^1^Pediatrics, Seattle Children's Hospital, Seattle, USA; ^2^University of Toronto, Toronto, Canada; ^3^Pediatrics, Children's Hospital of Philadelphia, Philadelphia; ^4^Pediatrics, Duke University, Durham, USA; ^5^Pediatric Rheumatology, Hospital for Sick Children, Toronto, Canada; ^6^Pediatrics, Joseph M. Sanzari Children's Hospital, Hackensack UMC, Hackensack; ^7^CARRA, Milwaukee; ^8^Duke Clinical Research Institute, Durham; ^9^Pediatric Rheumatology, Joseph M Sanzari Children's Hospital, Hackensack UMC, Hackensack, USA

###### **Correspondence:** Yukiko Kimura

**Introduction:** The Childhood Arthritis and Rheumatology Research Alliance (CARRA) developed consensus treatment plans (CTPs) for new-onset polyarticular JIA (pJIA). These CTPs represent commonly used strategies for starting biologic medications to treat pJIA. The CARRA Start Time Optimization of Biologic Therapy in Polyarticular JIA (STOP-JIA) study is an observational and prospective study that will assess the effectiveness of the various CARRA CTPs.

**Objectives:** The proportion of patients achieving Clinical Inactive Disease (CID) off of glucocorticoids and patient-reported outcomes at 12 months will be compared.

**Methods:** Untreated pJIA patients are enrolled in the CARRA Registry, and prescribed treatment as outlined in one of the CTPs. The CTPs follow the 3 most common treatment strategies for pJIA: 1) Step-Up (start DMARD only, and a biologic added later only if needed); 2) Early Combination (both a DMARD and a biologic are started initially); and 3) Biologic First (a biologic is started alone without a DMARD). Follow-up visits are performed at 3, 6, 9 and 12 months following enrollment and data are recorded in the Registry. The CTP used is chosen by the provider and patients/families at baseline. All treatment and follow-up visits are standard of care. There is no randomization or blinding.

**Results:** Three hundred and fifty-three patients were enrolled in STOP-JIA between 1 Nov 2015 and 27 April 2018, at 55 US and Canadian sites. Table 1 shows baseline characteristics for the 320 patients with completed data entry. 65% started the Step-Up CTP, 33% the Early Combination CTP and 10% the Biologic First CTP. The JIA categories enrolled, by descending frequency were: RF negative polyarticular (63%), RF positive polyarticular (17%), enthesitis related (8%), psoriatic (6%), extended oligoarticular (3%), and undifferentiated (2%). 1,215 total visits have been entered, with 152 patients completing the 12-month visit. Twenty-one Serious Adverse Events (SAE) or Events of Special Interest (ESI) were reported. These included 9 infections (including one case of septic shock), 4 uveitis (new-onset) and 3 hepatitis.

**Conclusion:** Completion of enrollment (400 patients) in STOP-JIA is expected by July 2018. All CTP choices are being used, but the Step Up strategy is the most common. Baseline characteristics are similar between groups, except for some differences in gender, the number of active joints, JADAS and the percentage of patients prescribed oral steroids. Comparative effectiveness of these 3 strategies will be assessed following completion of the 12-month visit data collection for the full cohort.

**Trial registration identifying number:** NCT02593006

**Disclosure of Interest**

S. Ringold Grant / Research Support from: CARRA, Inc, G. Tomlinson: None Declared, P. Weiss Grant / Research Support from: CARRA, Inc, Consultant for: Lilly, L. Schanberg Grant / Research Support from: CARRA, Inc, Consultant for: SOBI, UCB, Sanofi, B. Feldman Consultant for: Pfizer, BMS, Abbvie, OPTUM, AGILITY, M. E. Riordan: None Declared, V. Del Gaizo Grant / Research Support from: CARRA, Inc, Consultant for: SOBI, A. Dennos Grant / Research Support from: CARRA, Inc, K. Murphy Grant / Research Support from: CARRA, Inc, Y. Kimura Grant / Research Support from: CARRA, Inc

**Table 1 (abstract P358).** Baseline Patient Characteristics

	Total (n= 320)	Step up (n= 211)	Early Combination (n=77)	Biologic First (n=32)
Female N (%)	232 (72)	156 (74)	55 (71)	21 (66)
White N (%)	218 (68)	152 (72)	48 (65)	18 (56)
Age in yrs – mean (range)	10 (1-18)	10 (1-18)	11 (1-18)	11 (1-18)
Number of Active joints - mean (range)	13 (5-50)	12 (5-49)	16 (5-50)	11 (5-41)
Physician Global Assessment of Disease Activity - mean (range)	5 (0-10)	5 (0-10)	6 (1-10)	6 (1-10)
Juvenile Arthritis Disease Activity Score - mean (range)	18 (5-29)	17 (5-29)	20 (6-29)	20 (14-28)
CHAQ Score - mean (range)	1 (0-3)	1 (0-3)	1 (0-3)	1 (0-3)
Oral steroids prescribed at baseline - N (%)	84 (26)	57 (27)	22 (29)	5 (16)

### P359 DOES METHOTREXATE DISCONTINUATION INCREASE THE FLARES RATE AND AFFECT SURVIVAL OF FIRST ADMINISTERED BIOLOGIC IN NON-SYSTEMIC JUVENILE IDIOPATHIC ARTHRITIS?

#### Mikhail Kostik, Lyubov Sorokina, Ilia Avrusin, Elizaveta Orlova, Yuri Korin, Ekaterina Gaidar, Margarita Dubko, Vera Masalova, Tatyana Likhacheva, Ludmila Snegireva, Eugenia Isupova, Olga Kalashnikova, Vyacheslav Chasnyk

##### Saint-Petersburg State Pediatric Medical University, Saint-Petersburg, Russian Federation

###### **Correspondence:** Mikhail Kostik

**Introduction:** Biologics are the most effective drugs for treatment of juvenile idiopathic arthritis (JIA) in the cases of methotrexate (MTX) inefficacy or intolerance. The question about superiority of combination therapy (biologics+MTX) above monotherapy is still open. In some studies were shown that combination therapy is more effective, but others did not show any differences. The problem of MTX intolerance has been still actually, especially in adolescent patients. Several studies confirm that MTX can prevent development anti-drug antibodies avoiding to loss their efficacy. In patients with remission the MTX discontinuation leads to flare of JIA in 10-15%.

**Objectives:** to evaluate the role of MTX discontinuation in flare risk and survival of first biologic medication in non-systemic JIA.

**Methods:** in the study were included 692 non-systemic JIA patients, whom biologic therapy was prescribed. Inclusion criteria: patient whom first biologic was administrated with or without MTX or MTX was discontinued after start of biologics due to different reasons (remission, intolerance, adverse events). Exclusion criteria: treatment with current systemic corticosteroids, infliximab, rituximab. After selection 175 patients were eligible to analysis. We evaluate achievement of the remission, according C. Wallace criteria, flare, time to flare, change to biologics due to inefficacy and time to change. We compare two groups: i) patients with biologics with ongoing methotrexate and ii) patients with biologics alone, whom MTX was discontinued or not prescribed. For statistical analysis we Cox’s regression models, Log-Rank test, x2 test and Mann-Whitny test.

**Results:** there were no differences in initial data of studied population between groups, despite the MTX therapy. The flares were detected in 21.8% in whole group, 21.1% in MTX+biologics and 23.9 in biologics alone group (p=0.69), and time to flare was 20.8 (8,8; 34.5) and 19.3 (8.5; 21.0) months, respectively (p=0.37). There were no differences in cumulative probability of flare in both groups (LogRank test, p=0.7; RR=0.73 (95%CI: 0.28; 1,9), p=0.53). In 14.4% patients biologic was change due to inefficacy: 14.8% in MTX+biologic and 13.0% in biologic group (p=0.77), time to switch first biologics was 17.1 (6.8; 37.7) and 16.8 (4.0; 40.0) months, respectively (p=0.68). There were no differences in cumulative probability of biologic switch in both groups (LogRank test, p=0.63; RR=1.38 (95%CI: 0.36; 5.2), p=0.63). There were no differences of flare rate and biologic survive in etanercept, adalimumab, abatacept and tocilizumab subgroups despite on ongoing MTX. There were no differences in flare probability (p=0.4) and probability of switching biologics (p=0.46) between subgroup of TNFa inhibitors: etanercept mono; etanercept+MTX, adalimumab mono, adalimumab+MTX.

**Conclusion:** we suppose that discontinuation of MTX due to remission or intolerance does not increase the flare rate and probability of flare, switching the first biologics and probability to switch in non-systemic JIA patients with first administered biologics. Further trials are required.

**Disclosure of Interest**

None Declared

### P360 THERAPEUTIC DRUG MONITORING OF BIOLOGICALS IN CHILDREN WITH JUVENILE IDIOPATHIC ARTHRITIS (JIA): AN OVERVIEW OF CURRENT PRACTICE IN ANTI-TNF THERAPY

#### Amara Nassar-Sheikh Rashid^1^, Dieneke Schonenberg-Meinema ^1^, Annick de Vries ^2^, Theo Rispens^2^, Taco W. Kuijpers^1^, Gertjan Wolbink^2^, J. Merlijn van den Berg^1^

##### ^1^Department of Pediatric Hematology, Immunology, Rheumatology and Infectious Diseases, Emma Children’s Hospital, Academic Medical Center; ^2^Department of Immunopathology, Sanquin, Amsterdam, Netherlands

###### **Correspondence:** Amara Nassar-Sheikh Rashid

**Introduction:** Juvenile idiopathic arthritis (JIA) is the most prevalent pediatric rheumatic disease [1]. Long term complications include physical disability and a decreased quality of life [2]. Since the introduction of anti-TNF drugs for JIA, its prognosis has improved significantly [1, 3]. Personalized medicine is the next step to improve treatment in JIA. Anti-TNF trough levels and demonstration of the presence of anti-drug antibodies (ADA) could help individualize treatment decisions in JIA patients, but evidence supporting this is missing.

**Objectives:** To describe cross-sectional data of anti-TNF trough levels and ADA, combined with decision effects, in children with JIA.

**Methods:** Patients' records in children with JIA using etanercept, adalimumab or infliximab were retrospectively checked for measurements of anti-TNF trough levels and ADA. Anti-TNF trough concentrations and ADA were measured using an enzyme-linked immunosorbent assay (ELISA) and antigen-binding test. Data on age, sex, JIA subtype, reason for testing and the decision effect of trough level or presence of ADA on the current therapy were collected.

**Results:** Eighty-one anti-TNF trough levels were measured in 45 children with JIA. A wide variety of anti-TNF trough levels was found. Therapeutic drug concentrations, according to adult ranges in RA and IBD [4,5,6], were found in 11 (58%) patients on etanercept (n=19), 2 (14%) on adalimumab (n=14) and 8 (17%) on infliximab (n=48). Four patients on adalimumab and one patient on infliximab showed ADA. All of these five patients had non-detectable drug trough levels. Reasons for testing trough level and/or presence of ADA were loss of response (20%), partial or no response (40%), measurement after dosage increase (2%), remission (17%), uveitis flare (9%) and allergic reaction (11%). Treatment decisions were influenced by trough levels in 70/81 (86.4%) of measurements and in 5/5 (100%) of patients with ADA.

**Conclusion:** Measuring anti-TNF trough levels and ADA was a valuable tool in making personalized treatment decisions in JIA. Treatment changes included dose/frequency increase, or stopping and switching treatment in the presence of ADA combined with undetectable drug levels. Variation in anti-TNF trough levels seems even greater in children than in adults. More data are needed to access optimal therapeutic drug levels in anti-TNF treatment in JIA and to implement this strategy more widely.

**Disclosure of Interest**

None Declared

### P361 ESTIMATED PEAK OXYGEN UPTAKE AND SUBMAXIMAL PARAMETERS FROM A SUBMAXIMAL TREADMILL TEST ARE COMPARABLE BETWEEN PATIENTS WITH JUVENILE IDIOPATHIC ARTHRITIS DIAGNOSED IN THE ERA OF BIOLOGICS AND CONTROLS- A CONTROLLED CROSS-SECTIONAL STUDY

#### Kristine Risum^1^, Anne Marit Selvaag^1^, Øyvind Molberg^1^, Hanne Dagfinrud^2^, Helga Sanner^1^

##### ^1^Oslo University Hospital; ^2^Diakonhjemmet Hospital, Oslo, Norway

###### **Correspondence:** Kristine Risum

**Introduction:** Poor submaximal capacity is previously reported in juvenile idiopathic arthritis (JIA) patients, and is often assessed by the 6 minute walking test. However, due to improved management of JIA, there is a need for a more physically challenging submaximal test to measure submaximal capacity in JIA patients, and also to estimate peak oxygen uptake (VO_2 peak_).

**Objectives:** To estimate VO_2peak_ and measure submaximal parameters using an eight-minute submaximal treadmill test^1^ in oligo- and polyarticular JIA patients and controls. Further, to study associations between disease variables and estimated VO_2peak_ and walking distance in patients. The submaximal treadmill test has shown good validity at group level in JIA patients.

**Methods:** Patients with persistent arthritis or polyarticular disease were recruited consecutively at Oslo University Hospital. Age- and sex-matched controls were selected randomly from the National Registry. In all participants, VO_2peak_ was estimated using the eight-minute submaximal treadmill test. Submaximal parameters included heart rate and rating of perceived exertion (Borg _6-20_) at 3 and 8 minutes, speed and walking distance. Current pain, and pain and fatigue during the previous week were assessed by a Numeric Rating Scale. Disease activity, functional ability, disease duration and current medication were registered in patients. Differences between groups were tested with t-tests and correlations with Spearman’s rho correlation coefficients.

**Results:** Sixty JIA patients (50 girls), 30 with persistent oligoarthritis and 30 with polyarticular disease (extended oligoarthritis and polyarticular RF +/-) aged 10-16 years and 60 controls were included. Biologic drugs were used by 25 (42 %) patients. No significant differences were found in estimated VO_2peak_ (ml/kg/min) in patients vs controls or in patients with persistent oligoarthritis vs polyarthritis (Table 1). Submaximal parameters did not differ significantly between patients and controls or between JIA subsets. In patients, there were no correlations between any disease variables and estimated VO_2peak_ or walking distance (all r’s<-0.3, p=NS).

**Conclusion:** Estimated VO_2peak_ and submaximal responses from a submaximal treadmill test are comparable between JIA patients and controls, and also comparable between patients with persistent oligo arthritis and polyarticular disease. The encouraging results may possibly be explained by advances in the multidisciplinary management of JIA, including biologic therapy and individualized tailored patient education about physical activity.

**Reference**

1. Ebbeling CB, Ward A, Puleo EM, Widrick J, Rippe JM. Development of a single-stage submaximal treadmill walking test. Med Sci Sports Exerc. 1991;23(8):966-73.

**Disclosure of Interest**

None Declared

**Table 1 (abstract P361).** Submaximal treadmill test in patients and controls

		JIA total (n=60)	Controls (n=60)	p-value	JIA oligo (n=30)	JIA poly (n=30)	p-value
Estimated VO_2peak_ (ml/kg/min)		43.2 (10.3)	44.8 (8.0)	0.35	44.0 (7.8)	42.4 (12.3)	0.53
Walking distance, m		747 (97)	740 (70)	0.68	756 (73)	737 (117)	0.45
Speed km/h		6.1 (0.8)	6.1 (0.7)	0.67	6.2 (0.6)	6.0 (1.0)	0.33
3 min	HR	134 (9)	132 (9)	0.67	132 (9)	136 (9)	0.08
	Borg _6-20_	9.6 (2.0)	9.5 (2.2)	0.76	9.9 (2.0)	9.3 (2.0)	0.23
8 min	HR	163 (14)	159 (14)	0.17	163 (14)	163 (13)	0.98
	Borg _6-20_	13.0 (2.2)	12.5 (2.3)	0.34	13.2 (1.9)	12.8 (2.5)	0.45

Numbers are mean (SD). HR=heart rate

### P362 THE SAFETY PROFILE OF ADALIMUMAB ACROSS GEOGRAPHIC REGIONS AND DOSING ADMINISTRATIONS AMONG PATIENTS WITH JUVENILE IDIOPATHIC ARTHRITIS ENROLLED IN THE STRIVE REGISTRY

#### Nicola Ruperto^1^, Hermine Brunner^2^, Kabita Nanda^2^, Mary Toth^2^, Ivan Foeldvari^1^, John Bohnsack^2^, Diana Milojevic^2^, Daniel Kingsbury^2^, Katherine Marzan^2^, Elizabeth Chalom^2^, Gerd Horneff^1^, Rolf-Michael Kuester^1^, Jason Dare^2^, Maria Trachana-Pilavaki^1^, Mareike Bereswill^3^, Hartmut Kupper^3^, Alberto Martini^1^, Daniel Lovell^2^

##### ^1^PRINTO-IRCCS Gaslini, Genova, Italy; ^2^PRCSG, Cincinnati Children's Hospital Medical Center, Cincinnati, USA; ^3^AbbVie Deutschland GmbH & Co. KG, Ludwigshafen, Germany

###### **Correspondence:** Nicola Ruperto

**Introduction:** Adalimumab (ADA) has been approved for the treatment of polyarticular juvenile idiopathic arthritis (pJIA) with long-term use often required to maintain disease control.

**Objectives:** To describe the safety of ADA among children with pJIA treated in current clinical practice with fixed-dosing (F-D, by weight category, in the United States [US] and Australia) or body surface area-dosing (BSA-D, in European countries).

**Methods:** This is a year (yr) 8 interim analysis of an ongoing, multicenter, observational registry of patients (pts) with pJIA with up to 10 yr safety follow-up. Pts included in the registry were treated with ADA, alone or in combination with methotrexate (ADA±MTX), or MTX alone as a comparison arm, according to routine clinical care in PRINTO/PRCSG centres in the US, Australia, and Europe. This analysis included only ADA±MTX-treated pts who were sub-grouped according to enrolling site into two groups: F-D (10 - <15 kg, 10 mg every other week [eow]; 15 - <30 kg, 20 mg eow; ≥30 kg, 40 mg eow) or BSA-D (24 mg/m^2^ [maximum of 40 mg] eow). MedDRA observational adverse events (AEs) were recorded from registry entry through last contact, irrespective of duration of registry treatment with ADA±MTX.

**Results:** Of the 537 pts enrolled in the ADA±MTX arm, 272 and 263 received F-D and BSA-D, respectively. At registry entry, F-D and BSA-D groups were similar for mean JIA duration (3.5 yrs vs. 3.9 yrs), JADAS27(CRP) (10.8 vs. 12.2), and CHAQ-DI (0.6 for both). Registry follow-up in the F-D and BSA-D groups were comparable [mean (range) in yrs: 3.96 (0.00–8.92) vs. 3.58 (0.00–7.01)]. Overall, 166 pts (61%) in the F-D and 128 (49%) in the BSA-D group discontinued registry drug. Frequencies and rates of observational AEs per 100 pt years of observation time (from registry entry up to last contact, irrespective of drug treatment duration) were comparable between groups (Table 1), including rates of serious infection. Two pts from the BSA-D group reported latent TB, although there were no cases of active TB across enrolling sites. One pt (0.2%) from the BSA-D group reported an event of opportunistic infection (fungal oesophagitis). There were no reports of death, malignancy, oral candidiasis, demyelination, or congestive heart failure.

**Conclusion:** Overall, ADA±MTX was well-tolerated in pts with pJIA where administration has been with a fixed dose, per weight category, or based on BSA, respectively.

**Disclosure of Interest**

N. Ruperto Grant / Research Support from: AbbVie Inc., AstraZeneca, Bristol-Myers Squibb, Janssen Biologics B.V., Eli Lilly and Co., “Francesco Angelini”, GlaxoSmithKline, Italfarmaco, Novartis, Pfizer, Roche, Sanofi Aventis, Schwarz Biosciences GmbH, Xoma, and Wyeth Pharmaceuticals, Employee of: GASLINI Hospital, Speaker Bureau of: Astellas, AstraZeneca, Bristol-Myers Squibb, Italfarmaco, Janssen Biologics B.V., MedImmune, Roche, and Wyeth/Pfizer, H. Brunner Grant / Research Support from: AbbVie, AstraZeneca, Centocor, Bristol-Myers Squibb, Boehringer-Ingelheim, Pfizer, Regeneron, Hoffman La-Roche, Novartis, Takeda, UCB, Genentech, Lilly, Janssen, Ablynx and R-Pharm, Speaker Bureau of: Genentech Pharmaceuticals and Novartis, K. Nanda Consultant for: Novartis, M. Toth: None Declared, I. Foeldvari Consultant for: AbbVie and Novartis, J. Bohnsack Consultant for: Novartis, D. Milojevic: None Declared, D. Kingsbury Grant / Research Support from: AbbVie, K. Marzan Grant / Research Support from: AbbVie, E. Chalom Speaker Bureau of: AbbVie, G. Horneff Grant / Research Support from: AbbVie, BMS, MSD, Novartis, Pfizer, and Roche, R.-M. Kuester Grant / Research Support from: AbbVie and Wyeth/Pfizer, J. Dare Grant / Research Support from: AbbVie, AstraZeneca, BMS, Horizon Pharma, Medac, Pfizer, Roche, and UCB, M. Trachana-Pilavaki Grant / Research Support from: AbbVie, Novartis Hellas, and Pfizer, Speaker Bureau of: BMS, Novartis Hellas and Pfizer, M. Bereswill Employee of: AbbVie, H. Kupper Employee of: AbbVie, A. Martini Grant / Research Support from: AbbVie Inc., AstraZeneca, Bristol-Myers Squibb, Janssen Biologics B.V., Eli Lilly and Co., “Francesco Angelini”, GlaxoSmithKline, Italfarmaco, Novartis, Pfizer, Roche, Sanofi Aventis, Schwarz Biosciences GmbH, Xoma, and Wyeth Pharmaceuticals, Employee of: GASLINI Hospital, Speaker Bureau of: Astellas, AstraZeneca, Bristol-Myers Squibb, Italfarmaco, and MedImmune, D. Lovell Grant / Research Support from: AbbVie, Amgen, AstraZeneca, Centocor, Bristol-Myers Squibb, Forest Research, Pfizer, Regeneron, Hoffman La-Roche, Novartis, UBC, Xoma, and Genentech, Speaker Bureau of: Wyeth Pharmaceuticals

**Table 1 (abstract P362).** Overview of the observational adverse events per 100 PYs

	Fixed-dosing ADA±MTX (N=272)	BSA-dosing ADA±MTX (N=263)
	PYs=1076	PYs=940.8
	E (E/100 PYs)	E (E/100 PYs)
Any AE	453 (42.1)	358 (38.1)
AE at least “possibly drug related” per the investigator	155 (14.4)	101 (10.7)
Severe AE	47 (4.4)	26 (2.8)
Serious AE	56 (5.2)	92 (9.8)
AE leading to discontinuation of study drug or study	27 (2.5)	34 (3.6)
Infectious AE	160 (14.9)	119 (12.6)
Serious infectious AE	24 (2.2)	20 (2.1)
Injection site-related AE	18 (1.7)	20 (2.1)

E, events; PYs, patient years (Observation time, irrespective of study drug treatment duration)

## Psycho-social aspects and rehabilitation

### P363 EFFECTS OF INDIVIDUALIZED EXERGAME TRAINING ON UPPER EXTREMITY PROBLEMS IN ADOLESCENTS WITH JUVENILE IDIOPATHIC ARTHRITIS

#### Nilay Arman^1^, Ela Tarakci^2^, Kenan Barut^3^, Sezgin Sahin^3^, Amra Adrovic^3^, Ozgur Kasapcopur^3^

##### ^1^Faculty of Health Science, Division of Physiotherapy and Rehabilitation, Department of Physiotherapy and Rehabilitation; ^2^Faculty of Health Science, Division of Physiotherapy and Rehabilitation, Department of Neurological Physiotherapy and Rehabilitation; ^3^Cerrahpasa Medical Faculty, Department of Pediatric Rheumatology , ISTANBUL UNIVERSITY, İSTANBUL, Turkey

###### **Correspondence:** Nilay Arman

**Introduction:** Juvenile idiopathic arthritis (JIA) is most common chronic rheumatic disease in childhood. The upper extremity involvement in JIA causes muscle imbalance, joint destruction, pain, stiffness and limitations on activities of daily living in varying degrees. It has been reported that improvements of upper extremity functions were achieved by exergame training in various disease groups.

**Objectives:** The aim of this study was to investigate effects of individualized exergame training on upper extremity problems in adolescents with JIA.

**Methods:** 18 patients (15 girls, 3 boys) with JIA (8 oligoarticular, 18 polyarticular) who have at least one involvement upper extremity joint, participated in this study. Muscular strength of upper extremity was measured by using a portable digital handheld dynamometer. Also, Range of Motion (ROM) of upper extremity was evaluated with a universal goniometer. Severity of pain (during exercise, activity and rest), fatigue and stiffness were measured by Numeric Rating Scale (NRS). Besides, CHAQ was used for assessing functional ability. Training was structured in two 4-weeks phases with Xbox Kinect^TM^ Games, allowing to adapt the training according to individual training progress. In the first four weeks, games included movement patterns with low frequency were preferred (Volleyball, Darts and Bowling). In the second phase, games with fast movements for endurance were preferred (Fruit Ninja, Table Tennis, Boxing). The games were set as Individualized for 45-60 minutes by the physiotherapist. All the participants completed an 8 weeks (3 times in a week) individualized exergame training program.

**Results:** The mean age and duration of disease was 13.89±2.13 (age range 12-18), 7.50±4.27 years, respectively. 16 of patients had bilaterally involvement of wrist joint and 15 of them involvement elbow joint (7 bilaterally, 8 unilaterally) and 11 of them also had finger involvement. Statistically significant differences of pre and post-treatment were found for almost all the scores of ROM, CHAQ and NRS, except some scores of finger ROMs (p<0.05). And also, all muscles strength of upper extremities were statistically significant increased (p<0.001).

**Conclusion:** Our exergaming protocol that included Xbox Kinect^TM^ Games has showed improvements on pain, fatigue, stiffness and upper extremity functions in adolescents with JIA. The results of this study that the integration of exergame with Xbox Kinect^TM^ Games seems to have positive effects on upper extremity and is thus potentially beneficial for the long-term effectiveness of rehabilitation programs in adolescents with JIA.

**Disclosure of Interest**

None Declared

### P364 TUTTI ALLA PARI - SENSITIZATION PATH FOR SOCIAL INCLUSION OF CHILDREN WITH CHRONIC AND RARE RHEUMATIC DISEASES

#### Antonella Celano, Francesco La Torre, Adele Civino, Raffaella Arnesano, Annalisa Sticchi

##### Italian national Association people with rheumatic and rare diseases, lecce, Italy

###### **Correspondence:** Antonella Celano

**Introduction:** “Tutti alla pari” is a project made by Apmar Onlus. From the analysis of family and kids needs, it comes out that it’s difficult for a kid to feel himself involved in the society system. It is not rare that the kid with a rheumatic disease is socially excluded and sometimes bullied. Involve kids with disabilities is a challenge that can be won focusing on competence and collaboration. Differences must be accepted at school first by the activation of inclusive paths.

**Objectives:** Developing an empowerment path, starting from young people, to build up a most respectful and inclusive society; defining a common strategy to fight social exclusion of people with chronic diseases, especially children.

**Methods:** Create a participative process and an active involvement of social and institutional actors that take care of border risking minors. It involved pediatricians, parents, formers and journalists, through punctual, sensitizing and informative actions. 15 communication experts, 20 pediatricians, 18 formers and 25 parents took part in 3 participative labs. After those workshops a handbook that collects the “best practices for social inclusion” was realized and It was spread in 20 school and in 25 regional associations of patients with chronic and rare pathologies. The identified method aims to create a participative process and an active involvement of all social and institutional actors, that take care of border risking minors. It’s been involved paediatricians, parents, formers and journalists, through punctual, sensitizing and informative actions. 15 communication experts, 20 paediatricians, 18 formers and 25 parents have been involved in 3 participative labs. Their participation gave birth to the realization of a handbook that collects the “best practices for social inclusion”, that was spreaded in 20 school and in 25 regional associations of patients with chronic an

**Results: Involving the youngest**: experience labs organized in 16 schools through which kids could put themselves in a peer with rheumatoid arthritis’ shoes. By wearing gloves and special suits they experienced all the functional limitations of the disease. The action had important feedbacks, collected in written witnesses and videos elaborated by kids.

**Formers involvement**: 3 didactic seminars were realized about the social inclusion and the involvement of kids with chronic and rare pathologies. Bullism and cyber bullism and other topics were treated during those meetings dedicated to family and teachers. The seminars, attended by 200 people, have been developed by qualified professionals (psychologists, communication experts, journalists).

**Activities monitoring**: It aimed to measure the efficiency of the involved resources and the effectiveness of interventions related to the targets. It all started from the logistics of the intervention and the analysis of activities to elaborate monitoring schedules, addressed to the internal staff of the project, and questionnaires addressed to both direct and indirect targets.

**Conclusion:** To modify cultural attitude towards disability we must know what it means, through empathy, positive inclusion and respect. This kind of engagement is a priority in schools, in extra familiar, work and extra scholastic contexts. It must involve specialized staff to stay in touch with families, doctors and associations. “Tutti alla pari” showed how this target should be reached, through heterogeneous competences and multi-skilled professional resources.

**Disclosure of Interest**

None Declared

### P365 BUILDING A MULTIDISCIPLINARY TRANSITION CLINIC FOR ADOLESCENTS WITH RHEUMATIC DISEASES.

#### Nadina E. Rubio-Perez^1^, Ana C. Arana-Guajardo^2^, Fernando Garcia-Rodriguez^1^, Ana V. Villarreal-Treviño^1^, Antonio Lopez-Rangel^3^, Oscar Salas-Fraire^4^, Patricia Ancer-Rodriguez^5^, Maria E. Corral-Trujillo^6^, Juan G. de la Cruz-Gonzalez^7^, Dionicio A. Galarza-Delgado^6^, Manuel E. De la O-Cavazos^1^

##### ^1^Pediatric Rheumatology, DEPARTAMENTO DE PEDIATRIA, HOSPITAL UNIVERSITARIO "DR. JOSE E. GONZÁLEZ", UANL; ^2^Rheumatology; ^3^Psychiatric Department; ^4^Rehab and Sports Medicine, HOSPITAL UNIVERSITARIO "DR. JOSE E. GONZÁLEZ", UANL; ^5^NUTRITIONAL SERVICE; ^6^Rheumatology; ^7^Rehab and Sports Medicine, HOSPITAL UNIVERSITARIO "DR.JOSE ELEUTERIO GONZALEZ", Monterrey, Mexico

###### **Correspondence:** Fernando Garcia-Rodriguez

**Introduction:** Adolescence is a critical period in lifetime when patients with a rheumatic condition (RC) has to deal with disease acceptance; therefore, an uninterrupted and adequate attention must be provided to this population. Different transition programs (TP) are currently working around the world however, a minority of those include a multidisciplinary team to conduct transition.

**Objectives:** To describe a program that provide an uninterrupted, multidisciplinary and coordinated attention to adolescents with RC during transition from pediatric to adult services in Mexico.

**Methods:** During January 2017, a multidisciplinary group of specialist from our hospital were invited to participate in TP. Disciplines included in the team were decided by consensus of pediatric and adult rheumatologist with the aim of cover the most important issues that affects outcome in adolescents with RC.

From February to June 2017, we conducted systematic meetings to discuss logistic/organizational aspects, evaluation tools, and established goals and time that patients should stay in the program. A transition strategy was created based on literature, general TP and reports from other transition clinics.

**Results:** Adult and pediatric rheumatologists, sports medicine, psychiatrist, nutritionist, clinical psychologist, nurse, and social services integrated the team. Got Transition questionnaire evaluated transition skills of adolescents during TP.

TP beginning at Pediatric Rheumatology Clinic (PedRh, pre-transition), following by a Transition Clinic (TC) where skills (Sk), counseling (Co), multidisciplinary attention (MA), workshops (Wo), and psychosocial support (Psy) are offered to adolescents and families. Finally, post-transition follow-up at Adult Rheumatology Clinic once the patient were ready to transfer (Table 1).

During TC, visits are planned to be every 3 months unless closer follow-up where necessary. All specialists evaluate the patient every visit and meetings are conducted once a month to discuss cases and established strategies/goals for each patient.

Despite planned period to stay in TC are two years, it depends on skills that each patient demonstrate during TP, therefore, some could maintain at the same phase more than one visit to assure success of program.

**Conclusion:** Here we shown the structure of an organized, specialized, multidisciplinary, integrated, and reproducible TP for adolescents with RC.

**Disclosure of Interest**

None Declared

**Table 1 (abstract P365).** See text for description.

Phase	Facility	Physician	Attendee	Centered	Age	Goal
1	PedRh	Ped Rheum	Patient and Caregiver	Caregiver	12 to 15	Co, Explain the program
2	TC	Ped and Adult Rheum	Patient and Caregiver	Patient	16 to 18	Sk, Co, MA, Wo, Psy
3	TC	Ped and Adult Rheum	Patient	Patient	16 to 18	Sk, Co, MA, Wo, Psy
4	TC	Ped and Adult Rheum	Patient	Patient	16 to 18	Sk, Co, MA, Wo, Psy
5	TC	Adult Rheum	Patient	Patient	16 to 18	Sk, Co, MA, Wo, Psy
6	Adult Clinic	Adult Rheum	Patient	Patient	18 to 22	Sk, Co, MA, Wo, Psy
7	Adult Clinic	Adult Rheum	Patient	Patient	18 to 22	Post-transition f/u
8	Adult Clinic	Adult Rheum	Patient	Patient	18 to 22	Post-transition f/u

### P366 PERCEPTION OF TRANSITION AND SELF CARE SKILLS IN ADOLESCENTS WITH RHEUMATIC DISEASES AND THEIR PARENTS.

#### Fernando Garcia-Rodriguez^1^, Elias E. Uresti-Arriaga^1^, Ana V. Villarreal-Treviño^1^, Jesus D. Muñoz-Zepeda^1^, Ana C. Arana-Guajardo^2^, Dionicio A. Galarza-Delgado^2^, Manuel E. de la O-Cavazos^1^, Nadina E. Rubio-Perez^1^

##### ^1^Pediatric Rheumatology, DEPARTAMENTO DE PEDIATRIA, HOSPITAL UNIVERSITARIO "DR. JOSE E. GONZÁLEZ", UANL; ^2^Rheumatology, HOSPITAL UNIVERSITARIO "DR. JOSE E. GONZALEZ", Monterrey, Mexico

###### **Correspondence:** Fernando Garcia-Rodriguez

**Introduction:** Through their lifetime, young people with chronic conditions will experience a transition from pediatric care to adult care facilities; this is an important period for the patients since now they will have to take care of themselves.

The main goal of transition is to maximize lifelong functioning through the provision of high-quality, developmentally appropriate healthcare services that continue uninterrupted as the individual moves from adolescence to adulthood.

In pediatric clinics, care is provided to patients accompanied by their tutors; therefore, the sense of preparation for independence varies between one part and the other. To our knowledge, there are no reports that identified differences in self-care skills between patients and caregivers, and no information on variables that could influence on that perception has been To compare the perception of self-care skills for transition between adolescents and their caregivers and analyze clinical, familiar and socioeconomic factors that could influence on that perception.

**Methods:** Between January and April 2018 we included patients with rheumatic diseases that were diagnosed during childhood (under 16 years old) and at the time of their participation were between 12 and 22 years old. We excluded patients with more than one condition, neurological disabilities, or refuse to participate (patient or tutor). Patients were recruited from Outpatient Clinics from University Hospital “Dr. Jose E. González” at Monterrey, Mexico.

We collected clinical (age, gender, disease onset, activity status, medications, complications, and adverse events), familial (family composition), and socioeconomic (household characteristics, available services) variables. Self-care abilities and perceptions were evaluated with the Transition Readiness Assessment for Youth Questionnaire Spanish Version 2.0 (2014, 25729) and Transition Readiness Assessment for Parents/Caregivers Questionnaire Spanish Version 2.0 (2014, 25729). Variables were described with frequencies, means, medians, and were analyzed with Chi Square, Mann-Whitney, t test, and correlation tests as convenience.

**Results:** Perception in 31 patients and their parents were evaluated. Most of them were female (70%), median age 17 (12 - 21) years, and juvenile idiopathic arthritis (JIA) as the most prevalent disease (48%). A high part of our patients was classified as low and median low income families and a few proportion of the parents have superior education.

Among transitions questionaires, “Need to change to adult centered care” was answered with a median of 9 (5 - 10) by caregivers, that was similar to answer by patient (9, 1 – 10, P = 0.42). On “Perception of capability of adult-centered health care”, the answers were also similar (9 vs 8). Despite this, other specific areas of “disease knowledge” and “use of health-care services knowledge” were overestimated by caregivers when compared with patients.

No differences were seen when results by disease were analyzed.

**Conclusion:** Both patients and tutors are aware of the importance of a adequate transition preparation, also both have the same perception of preparedness of the patient. Both sides think they aren’t prepared enough for self care in emergency situations and the use of medications by themselves. Based on the results of this questionnaire we should focus our efforts in educating patients on how to react on an emergency situations as well as the self administration of the medications they use on their daily life.

**Disclosure of Interest**

None Declared

### P367 UNDERSTANDING BARRIERS AND DRIVERS THROUGH HEALTHCARE FOCUS GROUPS TO GUIDE THE DEVELOPMENT OF MUSCULOSKELETAL MODELS OF CARE FOR CHILDREN/ADOLESCENT IN A MIDDLE-INCOME COUNTRY

#### Heide Kunzmann^1^, Tony Woolf^2^, Josephine Erwin^3^, Helen Foster^4^, Christiaan Scott^1^

##### ^1^Paediatric Rheumatology, University of Cape Town, Cape Town, South Africa; ^2^Bone and Joint Research Group; ^3^Bone & Joint Research Office, Royal Cornwall Hospital, Truro; ^4^Paediatric Rheumatology, Newcastle University, Newcastle, UK

###### **Correspondence:** Heide Kunzmann

**Introduction:** Diseases of poverty remain the dominating health priority in South Africa and other Low- and Middle Income Countries (LMIC). Recent efforts towards strategic planning on the prevention and control of non-communicable diseases (NCDs) reflect the growing relevance of NCD’s in LMIC’s where infectious diseases burden is on the decline.

**Objectives:** We set out to identify barriers and drivers that influence access for children and adolescents presenting with musculoskeletal (MSK) symptoms with the aim to use this information to improve MSK care outcomes trough the development of models of care and deliver appropriate and applicable service improvement strategies. In doing so we hope to develop a model that can be replicated in other NCD’s and in other LMIC healthcare systems.

**Methods:** Five focus group interviews were done over a period of two weeks. The focus group participants consisted of Community Service Medical Officers (COSMO’s), Medical Officers (MO’s), Family Physician Consultants and Registered Nurses in the Eden and Central Karoo districts within the Western Cape Province of South Africa. There were forty-one participants. 25 of the 41 participants completed the questionnaire exploring their training with regard to the MSK system and their own perceived confidence in identifying and treating children with a MSK presentation.

**Results:** Twenty of the 25 participants were general practitioners, two where specialist family physicians (consultants) and 3 participants did not specify. The average level of experience was 8.8 years. Eight-eight percent (n=22) had undergraduate training in the examination of the paediatric MSK. 8% (n=2) had training in the paediatric gait, arms, legs and spine (pGALS), but did not use it in day-to-day practice. Teaching of the paediatric MSK system in children was predominantly delivered by paediatricians and paediatric orthopedic surgeons. 84% of the participants felt confident in some, but not all aspects of the MSK examination. Using short case studies the participants had to rate their confidence on a scale of 0-10. The confidence score for the MSK cases had a mean of 4.16-5.52 (SD 1.93-2.63), compared to communicable disease case that had a mean of 7.2 (SD 1.68).

Good relationship fostered by the secondary hospital paediatricians was identified as the biggest driver. Other drivers were, electronic platform for note keeping, communication via certain social media applications and gained knowledge of system and disease by an intern first working in a secondary hospital prior working in a peripheral hospital. Barriers were the healthcare providers own perceived limitation and exposure/knowledge to MSK diseases, poor history given by the person accompanying the patient, limitations in after hour investigations, unpredictable availability of ambulance services and socioeconomic factors of the patient population.

**Conclusion:** The focus groups identified barriers and drivers that play a role in whether children and adolescents presenting with MSK symptoms receive the “*right care*, delivered at the *right time*, by the *right team*, in the *right place*, with the *right resources*” these insights will direct further investigation into the drivers and barriers in the other districts of South Africa and other LMICs. The results could give direction in developing policies and Models of Care to improve MSK care outcomes for children and adolescents.

**Disclosure of Interest**

None Declared

**Table 1 (abstract P367).** How comfortable are you managing this case?

	Mean	SD
Case 1 (Limp)	5,52	1,93
Case 2 (Delayed Walking)	4,72	2,26
Case 3 (Shortness of breath and difficulty breathing)	7,2	1,68
Case 4 (Fever and arthritis)	4,52	2,63
Case 5 (Swollen joint)	4,16	2,26

### P368 A MULTIDISCIPLINARY APPROACH OF CHRONIC OSTEOARTICULAR PAIN IN CHILDREN AND ADOLESCENTS: THE LAUSANNE EXPERIENCE

#### Mejbri Manel^1^, Caroline Schnider^1^, Nicolas Lutz^2^, Anne-Emmanuelle Ambresin^3^, Alain Deppen^4^, Sandrine Vaucher^5^, Hofer Michael^1^

##### ^1^Unité Romande d’Immuno-Rhumatologie pédiatrique DFME; ^2^Unité pédiatrique de Chirurgie Orthopédique et Traumatique DFME; ^3^Division Interdisciplinaire Santé des Adolescents DFME; ^4^Service Universitaire de Psychiatrie de; ^5^Service de physiothérapie, CENTRE HOSPITALIER UNIVERSITAIRE VAUDOIS , Lausanne, Switzerland

###### **Correspondence:** Mejbri Manel

**Introduction:** Chronic pain disorder is a common and under-recognized problem who is increasing in the pediatric population.Osteoarticular pain is one of the most common symptoms.This is a significant problem leading to a decrease in quality of life,school absenteeism and social withdrawal.A multidisciplinary approach is essential to evaluate and manage those patients whom have an unsatisfactory evolution despite primary medical care.Starting In 2014,a group of 4 medical specialists(pediatric rheumatologist, pediatric orthopedist, child psychiatrist and pediatrician specialist in adolescent)and one physiotherapist started a joint outpatient clinic assessing such children.

**Objectives:** The aim of this study was to describe and outline some characteristics of all patients seen at this platform up to now.

**Methods:** A retrospective descriptive study was performed based on medical records of patients seen at our center in Lausanne between November 2014 and January 2018.Epidemiological,clinical and therapeutic data was collected and analyzed accordingly.

**Results:** A total of 35 patients were reviewed.The patients were in most cases(82%)referred by the pediatric rheumatologic or orthopedic surgeon.The sex ratio F/H was 3.3 (27 girls for 8 boys).The average age at the time of consultation was 12.8 years(9 to 18 years).A triggering event was found in 48% of our patients with the notion of trauma in 35% of these cases.The average duration of symptoms was 3.6 years prior to referral to the platform (1-11 years).Chronic pain affected more than 5 joints in 75% of the cases.School absenteeism was noted in 22% of the children with 2 cases of withdrawal.The platform revealed in 52% a primary paindisorder,in 28% an associated orthopedic problem,in 25% a difficult psychosocial situation and in 5% a rheumatologic problem.Therapeutic proposals were mainly personalized physiotherapy or mind body approaches such as hypnoses and depending on the findings,follow-up in orthopedics,rheumatology,child psychiatry or adolescent specialist consultation.

**Conclusion:** A long delay before children with chronic pain reach the platform was noticed.This could explain the severity of the presentation and the significant impact on school attendance and social life.The integrative clinical approach highlighted the multifactorial aspects of chronic pain and led to the development of an adapted multidimensional treatment to improve the prognosis and reduce the negative impact of chronic pain.We encourage pediatricians to detect and refer such patients early.

**Disclosure of Interest**

None Declared

### P369 ESTABLISHING A SELF MANAGEMENT AQUATIC PROGRAMME IN ADOLESCENTS WITH MUSCULOSKELETAL PAIN AND DISABILITY

#### Joanne May, Joanna Sheehan, Julia Smith

##### Paediatric Rheumatology, Oxford University Hospitals, Oxford, UK

###### **Correspondence:** Joanne May

**Introduction:** The paediatric rheumatology and paediatric chronic pain teams in Oxford provide highly specialised rehabilitation services with interventions ranging from one-to-one therapy to intensive inpatient rehabilitation programmes. It was identified that further ongoing community based support would be beneficial for the maintenance of physical fitness and wellbeing once the input of the specialist teams was no longer indicated.

**Objectives:** An opportunity to develop an innovative approach to community based self-management group aquatic therapy was identified by bringing together the expertese of Fluid Motion, a social enterprise group with establised success in delivering community group aquatic therapy for adults using artificial intelligence technology, the clinical teams from the paediatric rheumatology and peadiatric chronic pain services in Oxford, and the research experience of the musculoskeletal team at Oxford Brookes University.

We aimed to develop a self-management aquatic therapy programme for adolescents with musculoskeletal pain and disability using individualised digital progragrammes, enhanced with AI technology.

**Methods:** The specialist physiotherapists identified patients that were actively under their care between the ages of 12-18 year.

The group had a range of diagnoses but all had the common finding of ongoing musculoskeletal pain and disability.

It was recognised that a higher proportion would have a chronic pain condition.

The level of function required to take part was agreed as, independently mobile, able to get in and out of pool without assistance, independent dressing (parents will not be permitted to stay at pool side).

It was acknowledged that some patients may be inappropriate for group work including those with specific behavioural difficulties.

**Results:** Outline of the programme:

Patients were invited to attend a 7 week aquatic self management group under the supervision of our physiotherapists.

The patients had all been assessed by the specialist physiotherapists and identified as being appropriate for the programme.

The physiotherapists selected exercises from the digital rehabilitation package in order to create an individualised programme for each participant.

The paediatric rheumatology hydrotherapy pool was used. This was identified as an appropriate place to assess the initial intervention with a view to hosting future programmes in community pools.

5-6 patients were in each group.

The physiotherapist was in the pool for each session to provide support with the physiotherapy assistant on poolside.

Patients were introduced to their programme on the tablet before entering the pool.

The the patients used the tablets during each session and provided real time feedback on their experiences to inform development of their programme using AI technology.

Reassessment was carried out in the final session.

All patients had to commit to 6 out of 7 sessions including the final session.

Assessing the intervention:

Rheumatology patients completed CHAQ assessments before and after the programme.

Chronic pain patients completed the Oxford pain questionnaire including a pain map before and after the programme.

Physical tests, were completed in clinic before the intervention and in the 7th session of the programme

**Conclusion:** We identified a need to provide ongoing support for young people with musculoskeletal pain and disability once the input of highly specialised services was no longer indicated and recognised the potential for supervised self management group aquatic therapy. We present our experience of designing a programme using digital technology that can be easily utilised by young people during their therapy sessions and provide real time feedback to progress their therapy using AI technology.

**Disclosure of Interest**

None Declared

### P370 INTRODUCTION OF SPECIALIST PEDIATRIC RHEUMATOLOGY PHYSIOTHERAPY AND OCCUPATIONAL THERAPY CLINICS AT SIDRA MEDICINE - QATAR

#### Julie Melville, Aimie O'Hara

##### Allied Health , Sidra Medicine, Doha, Qatar

###### **Correspondence:** Julie Melville

**Introduction:** Physiotherapy (PT) and occupational therapy (OT) are widely accepted as mainstay treatments for Paediatric rheumatological conditions. Historically in Qatar children with rheumatological illness were referred to a paediatric rheumatologist, then to general outpatient therapy services hosted in separate sites. There were no formal feedback mechanisms between therapy services and the pediatric rheumatologist.

**Objectives:** This study reviews the patterns of referral to PT and OT services since opening the Pediatric Rheumatology clinic at Sidra Medicine. The study also presents specific outcomes; range of movement and goal attainment.

**Methods:** Patients referred for OT or PT were identified through Sidra Medicine’s electronic medical records system. All patients referred for OT or PT were identified between June 2017 and March 2018. A retrospective review of the EMR was conducted by the therapists. Passive range of movement measurements and goal ratings were compared at baseline and at the final assessment within the designated timeframe of the review.

**Results:** A total of 88 patients were referred to the rheumatology service.  Of these 15(18%) were referred for therapy. Seven (39%) of these were seen jointly by OT and PT while 7(39%) were seen by PT alone. One patient was seen for OT assessment and intervention only.

There were 4 Qatari nationals and 11 non-nationals. Juvenile Idiopathic Arthritis (JIA) was the most frequent diagnosis with 9 patients. There were 3 patients with scleroderma and 2 patients with Juvenile Dermatomyositis (JDM).

Five patients with restricted range of movement and functional limitations were seen for therapy. The remaining 10 patients were seen for strengthening only. Range of movement measurements were taken at commencement of therapy and at the end of the period included in the data. All patients set goals for therapy in conjunction with their treating therapist and these were reviewed at every session to assess progress.

The results of the initial and final range of motion measurements and goal attainment are summarized below in Table 1.2.

All patients demonstrated an improvement in passive range of movement in the joints assessed. Two of the patients continued to experience an uncontrolled disease flare (patient 3 and patient 5) however despite this demonstrated improvements in passive range of movement following therapy intervention.

At Sidra Medicine goals are set with the patient and family and reflect the functional goals that the patient and/or their family are hoping to achieve by attending therapy. Four of the 8 patients met all of their established goals.  Two patients met 66% of their goals and are continuing with therapy to meet these remaining goals. A final two patients did not meet their goals however both continued to have active disease. These are the same two patients who experienced the smallest improvements in passive range of motion of their joints.

**Conclusion:** Although only a small number of patients have been seen for specialist OT and PT treatment at Sidra the review suggests that outcomes for these patients in terms of improved range of motion and attainment of treatment goals is positive. The pediatric rheumatologists report that the increase in communication with treating therapists has positively affected their ability to manage disease flares ensuring improved care.

**Disclosure of Interest**

None Declared

**Table 1.2 (abstract P370).** See text for description

Patient	Diagnosis	Mean PROM Improvement (in degrees)	Median PROM Improvement (in degrees)	Goals set	Goals Met in time frame	Goals Continuing	% Met
One	JIA-ERA	16.75	13	2	2	0	100%
Two	Scleroderma	11.66	10	6	4	2	66%
Three	Scleroderma	7.77	5	5	0	5	0%
Four	Scleroderma	10.71	7.5	3	2	1	66%
Five	JIA-Poly-Articular	13.75	15	2	0	2	0%
Six	JIA-ERA	NA	NA	1	1	0	100%
Seven	JIA-Oligo	NA	NA	1	1	0	100%
Eight	JDM	NA	NA	2	2	0	100%

### P371 ADOLESCENTS KNOWLEDGE ABOUT JIA AND THEIR TREATMENT

#### Karina Mördrup^1^, Anna Vermé^1^, Karin Palmblad^1^, Cecilia Bartholdson^2^

##### ^1^Pediatric Rheumatology Unit, Karolinska University Hospital; ^2^Department of Pediatric Neurology and Muscular Skeletal Disorders and Homecare, Karolinska Institutet, Stockholm, Sweden

###### **Correspondence:** Karina Mördrup

**Introduction:** It is important for adolescents with a chronic disease as Juvenile Idiopathic Arthritis (JIA) to get acquainted with their condition. This allows the young people to take responsibility for their own disease and medication. Health care professional’s knowledge about the adolescents knowledge regarding their illness and treatment is very limited.

**Objectives:** To examine the level of knowledge adolescents has about their illness and treatment and to clarify in which area they need education from health care professionals. As to examine difficulties with procedures as join injections and blood samples.

**Methods:** To map the knowledge of patients who are 18 years old and are to be transferred to adult care. The patients have received and responded to the Medical, Exercise, Pain and Social Support Questionnaire (MEPS). The questionnaire consists of four dimensions, medical domain, exercise domain, pain domain and social domain with varying number of subheadings. The questions are answered using a VAS scale, between zero and one hundred.

**Results:** : All Thirty patients that was invited to participate, answered the questionnaire. The results will be illustrated with examples from each domain. In the medical domain the result shows that patients have poor knowledge of juvenile idiopathic arthritis (JIA) and treatment. The median value of the question: How much knowledge do you have about the medical background to childhood rheumatism, was 54,5. Only a few patients have a great deal of fear of sampling and cortisone injections. Results from the exercise domain showed that the median value for joining the school gymnastics was 89. Furthermore, the question; Do you know how to reduce your pain significantly, from the pain domain resulted in a median value of 46. The questions about the social domain are about how valuable the patients think it is to meet others with the same diagnosis and if it is considered worthwhile with a patient association. The answers showed that the patient did not think this had any difference.

**Conclusion:** This study of patients' knowledge illustrates that their knowledge of their disease and treatment varies. The range is from lowest to highest score on almost every question. In general, the study reveal that a large portion of the young patients have surprisingly poor knowledge of their illness and treatment. However, we thought that several of these patients would find it difficult to have joint injections and take blood samples, which they didn´t. We see this as a very pleasing result.

**Disclosure of Interest**

None Declared

### P372 EDUCATING THE YOUNG PATIENT WITH JUVENILE IDIOPATHIC ARTHRITIS IN PREPARATION FOR THE TRANSITION TO ADULT MEDICINE.

#### Anette Pieler, Heidi K. Ipsen, Anne E. Christensen, Peter Toftedal

##### H.C. Andersen Childrens Hospital, Odense University Hospital, Odense, Denmark

###### **Correspondence:** Anette Pieler

**Introduction:** In 2014 Department of Rheumatology at Odense University Hospital experienced that their young patients had a lack of knowledge about their own disease, treatment and insight into life as an adult patient. Health professionals from both the adult and pediatric department participated in a brainstorm process, also including the young patients and their families. They found, as expected, that the young patients had very little knowledge about their own disease and what to expect as an adult patient. The pediatric team decided to take action in improving the standard for transition.

**Objectives:** To help identify and strengthen the young patients level of self-care and improving their understanding of their own disease. To inform and prepare them for the transition from the children’s hospital to adult medicine. Here they will be expected to take a more independent and active part in their treatment.

**Methods:** 1) More focus was given in the pediatric consultations about living with JIA and going to consultations as an adult. This was begun well in advance before the actual transition.

2)  The patients were guided to the location of the adult department of Rheumatology and welcomed by a nurse there at the end of their last pediatric consultation. This was only an option if the patient chose to receive further treatment at the same hospital. Others chose hospitals closer to their home.

3)   Development of an application for smartphones that contains information regarding the disease; medication and the organization of the outpatient clinic at both departments with an option to write directly to the nurses.

**Results:** These different methods were well received by both patients and parents, although there was still in most instances a parent-guided tendency in the consultations.

It was evident that there were still hurdles for the patients to climb in regards to making direct contact and starting to take ownership of their own disease and treatment. Several patients got the application on their phone; none has used it to write directly in to the nurses, instead their parents called the nurses as usual.

**Conclusion:** The feedback from the department of Rheumatology is positive as they are encountering more prepared patients that have a better understanding of the way the consultations and treatments will be handled.

Further efforts are needed in supporting the young patients to take a more active role in their treatment and in the understanding of their disease.

Many, especially among the ones that have been diagnosed at an early age, have very little insight into their own disease and treatment, while their parents are often fully understanding of the different aspects.

The team has started to plan a group session of 4-6 young patients to get them together, without parents, for a couple of hours one afternoon where the focus would be JIA, and the treatment thereof. A board game about JIA has been developed in an effort to make talking to others about their disease easier, without having to share personal details about themselves.

**Disclosure of Interest**

None Declared

### P373 THE INFLUENCE OF PSYCHOSOCIAL FACTORS IN CHILDREN WITH JUVENILE IDIOPATHIC ARTHRITIS AND THE IMPACT OF A COGNITIVE-BEHAVIORAL PSYCHOLOGICAL TREATMENT

#### Verena M. Balbo^1^, Francesco Rogari^2^, Teresa Giani^3^, Gabriele Sinonimi^4^, Susanna Esposito^5^, Rolando Cimaz^6^

##### ^1^Rheumathology Unit, University of Florence, Meyer children's hospital Neurofarba Department, firenze; ^2^S.C. Clinica Pediatrica, University of Perugia, Perugia; ^3^Rheumatology unit; ^4^Rheumathology unit, University of Florence, Meyer children's Hospital, Neurofarba department, firenze; ^5^S.C. child care center, University of Perugia, perugia; ^6^rheumathology Unit, University of Florence, Meyer children's Hospital, Neurofarba departme, Firenze, Italy

###### **Correspondence:** Francesco Rogari

**Introduction:** Juvenile Idiopathic Arthritis (JIA) is the most common childhood rheumatic disease. The chronic disease, and specifically the rheumatic one, is characterized by a varied symptomatology, with an alternation between acute, remission and reactivation phases of the disease that may induce disability, functional and social impairments of variable importance on the basis of medical (clinical subtype, late diagnosis, adequacy of the treatment) and non-medical factors (attachment styles, Life events, pain coping strategies, emotional state of child and parent).

There are scientific evidences that Cognitive Behavioral Therapy (CBT) is a treatment that improves coping skills and reduces pain, joint inflammation, physical disability and secondary depressive symptoms in patients affected by cronic pain.

**Objectives:** The study investigates the psychological factors of children with Juvenile Idiopathic Arthritis (JIA) at the onset of the disease and its association with physician’s functional and pain assessment in the follow-up.

The secondary aim was to evaluate the efficacy of an individual cognitive behavioral treatment for children with JIA on the global patient’s outcome and their parents both in terms of functional and pain assessment.

**Methods:** We performed an open longitudinal study on consecutive onset JIA patients (any type of JIA as per the International League of Associations for Rheumatology). Children underwent a psychological interview, they were submitted questionnaires evaluating the attachment with the caregiver (Separation Anxiety Test, SAT), psychopathology (Child Behavior CheckList, CBCL), strategies of pain facing (Coping) for children; anxiety and emotional state (STAI-Y 1 STAI-Y 2) for caregivers and life-events in children and caregivers.

Physician’s assessment was extracted from physical examination (disease activity, active joint count), laboratory variables (antinuclear antibodies, hemoglobin, C-reactive protein and erythrocyte sedimentation rate) and Child Health Assessment Questionnaire (CHAQ), given at the same time.

Children and parents in the treatment condition completed questionnaires prior to treatment and directly after treatment.

Some children were also assigned to an individual psychological cognitive-behavioral treatment of 8 sessions, based on psycho-educating children and parents on pain and anxiety mechanisms, teaching children to restructure pain related negative automatic thinking, assertiveness training and relaxtion training. Results were measured from self-reported scales. Clinical data was collected by a rheumatologist.

**Results:** Data from 40 patients with JIA (24 females; 16 man). At baseline, median age was 8.5 years (3-16 years); 12 patients do not report life events; 22 report life events one at baseline (64.7%); caregivers state-anxiety are not significantly associated with a type of juvenile arthritis.

Results show a trend in the reduction of anxiety and emotional state from children and parents who have carried out the CBT intervention but this decrease does not reach a statistical significance (p= .44).

**Conclusion:** Preliminary analysis showed that stressful life events that may promote the slatentization of AIG are: new education classes, illness of a family member, parents’ separation, grief in family. This findings from suggest that Cognitive Behavioral intervention for JIA may help patients and their parents in reduction the burden of their illness.

**Disclosure of Interest**

None Declared

### P374 EVALUATION OF THE IMPACT OF A SUMMER CAMP IN CHILDREN WITH JUVENILE IDIOPATHIC ARTHRITIS

#### Joana Silva-Dinis^1,2^, A. T. Melo^1^, Raquel Campanilho-Marques^1,2^, Patrícia Costa-Reis^1,3^, A. F. Mourão^1,4^, Pedro Dias-Ferreira^3^, J. E. Fonseca^1,2^, Filipa Oliveira-Ramos^1,2^

##### ^1^Unidade de Reumatologia Pediátrica, Serviço de Reumatologia e Doenças Osseas Metabólicas, Hospital de Santa Maria, CHLN, Centro Académico de Medicina de Lisboa; ^2^Unidade de Investigação em Reumatologia, Instituto de Medicina Molecular, Faculdade de Medicina, Universidade de Lisboa, Centro Académico de Medicina de Lisboa; ^3^Departamento de Pediatria, Hospital de Santa Maria, CHLN, Centro Académico de Medicina de Lisboa; ^4^Serviço de Reumatologia, Hospital Egas Moniz, CHLO, Lisboa, Portugal

###### **Correspondence:** Joana Silva-Dinis

**Introduction:** Summer camps for children with chronic conditions like Juvenile Idiopathic Arthritis (JIA) seem to have a positive impact on physical, psychological, emotional and social aspects. The first Portuguese Summer Camp for Children with JIA was an initiative of the Pediatric Rheumatology Unit of Hospital de Santa Maria, in Lisbon. There have been 2 editions (Ed): the 1^st^ in 2016 and the 2^nd^ in 2017, with one-week duration and 19 participants each.

**Objectives:** The goal of this prospective pre-post study was to evaluate the impact of the Summer Camp for children with JIA.

**Methods:** Inclusion criteria were all children who participated in the 1^st^ or 2^nd^ Ed of the JIA Summer Camp and completed the 4 questionnaires: the Childhood Health Assessment Questionnaire (CHAQ), the Functional Assessment of Chronic Illness Therapy (FACIT), the KIDSCREEN-52 and the “Emotional and Social Competence Evaluation Scale” (EACSE) questionnaires. These were done at 2 separate time periods: "pre-camp", in the 3 months that preceded the camp; and "post-camp", within 3 months after camp. Informed consent was obtained from parents. Questionnaires with missing answers were excluded, as were children that only responded pre or post camp.

**Results:** A total of 33 questionnaires were completed by 20 children who participated in one or both editions. Table 1 shows the demographic characteristics.

The median CHAQ result was 0.125 (IQ 0—0.312) pre-camp and 0 (IQ 0—0.25) post-camp. There was a negative variation in pre and post camp CHAQ with statistical significance difference (SSD) (p-value 0.035).  The median FACIT result pre-camp was 46 (IQ 43-50) and post-camp was 45 (IQ 42.5-49), with no SSD. In the KIDSCREEN questionnaire, the domain of Parent Relations and Home Life and the domain of Social Support and Peers had a positive SSD of 0.33 and 0.45 (p-value 0.47 and 0.45, respectively). Other domains did not have SSD. The median global score was 3.60 (IQ 3.47 – 3.78) pre-camp and of 3.54 (IQ 3.50 – 3.83) post-camp, with no SSD observed. In the EACSE Score, the Emotional Regulation domain had a positive SSD of 0.8 (p-value 0.34). The median global score pre-camp was 4.0 (IQ 3.0-5.0) with no variation in post-camp score. There was also no SSD observed in other domains.

**Conclusion:** The Summer Camp for children with JIA was a pioneer activity in Portugal, as was the analysis of Camp impact in these children. The domain of Parent Relations and Home Life and the domain of Social Support and Peers in KIDSCREEN and the Emotional Regulation domain of the EACSE questionnaires showed a positive SSD, which represents an improvement in these domains. Similarly, the negative SSD in CHAQ also substantiates the positive functional impact of this Summer Camp for children with JIA. These results corroborate the idea that Summer Camps have a positive impact in children with JIA.

**Disclosure of Interest**

None Declared

**Table 1 (abstract P374).** See text for description

Male (%)	10 (50%)
Median age in years (IQ)	12 (10-14)
JIA category	
Persistent oligoarticular	9 (45%)
Extended oligoarticular	3 (15%)
Polyarticular rheumatoid factor negative	3 (15%)
Enthesitis-related arthritis	3 (15%)
Psoriathic arthritis	2 (10%)
Prednisolone / Methotrexate / Biologic therapy (%)	1 (5%) / 14 (70%) / 2 (10%)

### P375 ERGOTHERAPY INTERVENTION FOR CHILDREN AND ADOLESCENTS WITH JUVENILE IDIOPATHIC ARTHRITIS

#### Ela Tarakci^1^, Nilay Arman^2^, Kenan Barut^3^, Sezgin Sahin^3^, Amra Adrovic^3^, Ozgur Kasapcopur^3^

##### ^1^Faculty of Health Science, Division of Physiotherapy and Rehabilitation, Department of Neurological Physiotherapy and Rehabilitation; ^2^Faculty of Health Science, Division of Physiotherapy and Rehabilitation, Department of Physiotherapy and Rehabilitation; ^3^Cerrahpasa Medical Faculty, Department of Pediatric Rheumatology, ISTANBUL UNIVERSITY, İSTANBUL, Turkey

###### **Correspondence:** Ela Tarakci

**Introduction:** Juvenile idiopathic arthritis (JIA) is the most common chronic pediatric rheumatic disease. Depending on the underlying pathologies, the common symptoms of this disorder are the limitations of the upper extremity joint movement angles, muscle imbalance and the functional limitations caused by the contracture due to these patients. Wrist joint is the most affected joint in the upper extremity and daily life activities such as feeding, personal care, and self mobility activities that use upper extremity are commonly limited with these children. Ergotherapy can help to reduce a child's pain. It can help to maximize their strength, endurance, and physical function. Ergotherapy can make them more independent in their activities of daily living.

**Objectives:** The aim of this study was to investigation of effectiveness of individual ergotherapy intervention for rehabilitation in patients with JIA who have involvement wrist joint.

**Methods:** 21 patients with JIA (17 girls and 4 boys), age range 6-18 participated in this study. The patients were recruited from the pediatric rheumatology outpatient clinic of the Department of Pediatric Rheumatology of the Istanbul University Faculty of Cerrahpasa Medicine.

Range of motions (ROMs) of hand, grip strength, functional ability, fatigue and quality of life were assessed with a goniometer, hand dynamometer, Childhood Health Assessment Questionnaire (CHAQ), Numeric Rating Scale (NRS), and the Pediatric Quality of Life Inventory (PedsQL), respectively. The ergotherapy sessions were designed according to the needs of the individual. The sessions consisted of activities that improved fine motor skills and increased joint mobility, hand-eye coordination, and daily life skills training. The patients completed an 8 week individually planned ergotherapy program 3 times a week at the department of physical therapy and rehabilitation. Duration of the each therapy session was 30-45 minutes.

**Results:** The mean age and duration of disease was 13.48±3.50 (age range 8-18), 4.95±3.59 years, respectively. The means of the pre/post treatment scores of NRS fatigue were 5.33±1.88/2.00±1.54, PedsQL-patients; 63.39±18.48/83.40±13.81, PedsQL-parents; 58.23±16.76, CHAQ-total; 1.11±0.60/0.25±0.36, CHAQ-pain; 46.67±29.51/10.48±18.83 and CHAQ-well being; 61.67±25.11/30.95±19.27, respectively. Also, statistically significant differences were found between pre and post-treatment of ROMs of wrist flexion and extension, hand grip and pinch grips scores (p<0.05).

**Conclusion:** The study demonstrated that participating in an 8-week individually planned ergotherapy program improves ROMs of hand, grip strength, fatigue, physical function and the quality of life in patients with JIA. We think that patients with JIA who have affected wrist joint may live independently through individualized ergotherapy program.

**Disclosure of Interest**

None Declared

## Treatment II

### P376 SWITCHING OF BIOLOGIC AGENTS AMONG CHILDREN WITH JIA IN THE CHILDHOOD ARTHRITIS AND RHEUMATOLOGY RESEARCH ALLIANCE REGISTRY

#### Melissa L. Mannion^1^, Fenglong Xie^2^, Daniel B. Horton^3^, Sarah Ringold^4^, Colleen K. Correll^5^, Anne Dennos^6^, Timothy Beukelman^1^, CARRA Registry Investigators

##### ^1^Pediatric Rheumatology; ^2^Rheumatology, University of Alabama at Birmingham, Birmingham; ^3^Pediatric Rheumatology, Rutgers University, New Brunswick; ^4^Pediatric Rheumatology, Seattle Children's Hospital, Seattle; ^5^Pediatric Rheumatology, University of Minnesota, Minneapolis; ^6^Duke Clinical Research Institute, Durham, USA

###### **Correspondence:** Melissa L. Mannion

**Introduction:** Published treatment recommendations state to change biologic medications when the goal of inactive disease status is not attained, but the switching of biologic agents has not been well-studied.

**Objectives:** To examine the frequency of switching of biologic agents in the treatment of JIA in clinical practice in North America.

**Methods:** Using retrospective and prospective data from the Childhood Arthritis & Rheumatology Research Alliance(CARRA) Registry, we identified patients with JIA categories other than systemic arthritis who newly started a biologic agent after 1 January 2008 and had at least 12 months of observable time following medication start. “Switchers” were defined by stopping the first biologic agent and starting a different biologic agent within 6 months of the discontinuation. “Delayed switchers” were defined by stopping the first biologic agent and starting a different biologic agent more than 6 months after the discontinuation. “Non-switchers” were defined by no use of any other biologic agent. Chi-square and Wilcoxon rank sum were used to compare patient characteristics among the different switching patterns.

**Results:** 708 children with JIA newly started a biologic agent during the study period and had sufficient data available for analysis. 146 (21%) were switchers, 45 (6%) were delayed switchers, and 517 (73%) were non-switchers. Among switchers, the median time between starting the first biologic agent and switching biologic agents was 309 days (interquartile range (IQR) 151 - 630) and the median time between the first and second biologic medications was 0 days (IQR 0 – 31). Among delayed switchers, the median time between starting the first biologic agent was 1184 days (IQR 669-1442) and the median time between the first and second biologic medications was 587 days (IQR 335-858). Etanercept was the most commonly initiated first biologic agent (N=476,67%). The proportion of patients who used etanercept as the first biologic agent was greater among all switchers (73%) compared to non-switchers (65%; p=0.04). The proportion of patients who took concurrent methotrexate was greater among all switchers (84%) compared to non-switchers (76%; p=0.03). The prevalence of chronic uveitis and inflammatory bowel disease was similar between switchers and non-switchers. Medication ineffectiveness (N=77, 52%), flare of disease (N=19, 13%), and medication intolerance (N=16, 11%) were the most commonly reported reasons for discontinuing the first biologic agent among all switchers.

**Table:** 

	All biologic initiators N= 708	Non switchers n=517	Switchers n=146	Delayed switchers n=45
Age (years) median, range, IQR	10 (IQR 6, 13)	10 (6, 13)	10.5 (8, 14)	8 (4, 11)
RF+ poly	90 (13%)	69 (13%)	16 (11%)	5 (11%)
RF- poly	376 (53%)	278 (54%)	71 (49%)	27 (60%)
Persistent Oligo	66 (10%)	51 (10%)	11 (8%)	4 (9%)
Extended oligo	44 (6%)	32 (6%)	10 (7%)	2 (4%)
ERA	72 (10%)	49 (10%)	21 (14%)	2 (4%)
psoriatic	44 (6%)	27 (5%)	14 (10%)	3 (7%)
etanercept	476 (67%)	336 (65%)	103 (71%)	37(82%)
adalimumab	152 (21%)	118 (23%)	29 (20%)	5 (11%)

**Conclusion:** Following first initiation of biologic agent use for JIA, nearly 30% of children switched to a second biologic medication after a median of 14 months. All switchers were more likely to have initiated etanercept and more likely to have taken concurrent methotrexate compared to non-switchers. Future studies are needed to examine the short- and medium-term clinical outcomes following biologic switching to help identify the optimal timing of switching and the most effective second-line biologic agent.

**Disclosure of Interest**

M. Mannion Grant / Research Support from: CARRA Publication Grant, F. Xie: None Declared, D. Horton: None Declared, S. Ringold: None Declared, C. Correll: None Declared, A. Dennos: None Declared, T. Beukelman: None Declared

### P377 HIP INTRA-ARTICULAR CORTICOSTEROID INJECTION IN PATIENTS WITH JIA: WHAT IS THE EFFICACY OF THIS TREATMENT?

#### Marta Mazzoni^1,2^, Carlo Gandolfo^3^, Stefania Viola^1^, Alessandro Consolaro^1,2^, Angelo Ravelli^1,2^, Clara Malattia^1,2^

##### ^1^Clinica Pediatrica e Reumatologia, G. Gaslini; ^2^Università degli Studi di Genova; ^3^Neuroradiologia, G. Gaslini, Genoa, Italy

###### **Correspondence:** Marta Mazzoni

**Introduction:** The efficacy of intra-articular (IA) steroid injections in patients with JIA has already been demonstrated. However, the value of IA steroid injection into hip joint remains uncertain, since in very few studies systematic imaging follow-up was conducted with conventional radiography (CR) or magnetic resonance imaging (MRI).

**Objectives:** To evaluate the effect of hip steroid injection in terms of reducing synovitis and preventing joint damage progression in patients with JIA.

**Methods:** All JIA patients who received ultrasound guided intra-articular triamcinolone hexacetonide (IATH) injection for the treatment of coxitis at the Study Unit between 2013 and 2018 and underwent imaging assessment before and after local treatment were included in the present study. Pre- and post-therapeutic assessment included: clinical work-up, bilateral hip radiographs (median duration of the interval between the two radiographs 1.5 years) and bilateral hip MRIs (median duration of the interval between the two MRIs 1 year). Joint damage progression was assessed according to the Childhood Arthritis Radiographic Score of the Hip (CARSH). MRIs were assessed by using the Outcome Measure in Arthritis Clinical Trials (OMERACT) Rheumatoid Arthritis Scoring System (RAMRIS) synovitis score. Descriptive statistics were reported in terms of absolute frequencies or percentages for categorical data, while in terms of medians, first and third quartiles (1st - 3rd q) for continuous quantitative data. Comparison of categorical data was performed by means of the Fisher’s Exact test.

**Results:** A total of 13 patients (10 F, 3 M; 6 systemic, 3 polyarticular and 4 oligoarticular -onset JIA patients; median age at the time of IATH 16.2 years; median disease duration 8.8 years) received IATH in 17 arthritic hips. Nine patients had unilateral coxitis, while 4 had bilateral hip involvement. Immediate pain relief and improved hip mobility occurred in 13 out of 17 hip joints (76.5%). Nine out of 17 hips (53%) were in clinical remission at 3 months follow-up visit. Follow-up MRI showed an improvement of synovitis in 4 out of 17 hips (23.5%) and a complete resolution of synovitis in 3 out of 17 hips (17.6%). In the remaining 10 hip joints (58,8%) synovitis didn't improve. Joint damage progression was assessed in 14/17 hips. Structural deterioration was observed in 5 out of 14 hips (35.7%). Four out of 9 hips (44.4 %) with persistent synovitis experienced joint damage progression *versus* 1 out of 5 hips (20%) that had an improvement or complete resolution of synovitis (p=0,58). None of the hips that achieved complete resolution of the synovitis experienced damage progression. Notably, all cases of structural deterioration occurred in patients with diagnosis of systemic JIA. Any avascular necrosis of the femoral heads (FHN) or other complications were not observed.

**Conclusion:** IATH treatment of coxitis in JIA is a safe therapy yielding a high remission rate from a clinical point of view. Clinical improvement however does not equate to imaging resolution of synovitis. The group of non-responders, with long-term persistent synovitis as detected by imaging, experienced a higher frequency of joint damage progression compared to patients with complete remission or improvement of coxitis after IATH. None of the patients that achieved complete resolution of the synovitis experienced structural deterioration. It is important to highlight that most of our patients had polyarticular or systemic JIA with long disease duration, thus justifying a more severe disease course.

**Disclosure of Interest**

None Declared

### P378 PROPOSAL FOR THE DEVELOPMENT OF BIOLOGICS IN PEDIATRIC RHEUMATOLOGY FIELD IN JAPAN

#### Masaaki Mori

##### Lifetime Clinical Immunology, Tokyo Medical and Dental University, Tokyo, Japan

**Introduction:** Favorable outcomes in the inflammatory state are expected without carrying over organ failure to adulthood if the principle of early diagnosis and early therapeutic interventions are maintained, and it is not too much to say that the advent of biologics brought us this improvement. In pediatric rheumatology field, four biologics (tocilizumab, etanercept, adalimumab, palivizumab and abatacept) have been approved in Japan by April 2018. Especially, new approval of pediatric indication for tocilizumab, etanercept and adalimumab that are anti-rheumatic agents have greatly changed the medical treatment in pediatric rheumatology field, by which many clinicians have realized the change of the time from “CARE” to “CURE”

**Objectives:** For the development, approval and early introduction into clinical practice of biologics in pediatric field, we need to grasp the pesent situation of the development to approval of biologics as anti-rheumatic agents for children in Japan, and visualize the pesent problems and give a proposal for the future.

**Methods:** We reviewed the time lag from the development to approval between inside and outside of Japan and between adult use and pediatric use using each Application document.

**Results:** As results of analysis in various directions, it is apparent that the duration of a review period required for the preparation of clinical trials and the approval in PMDA clearly reduced compared with the past. Thus, it was speculated that a rate limiting step in the process from development to approval was the duration of clinical trials between its start to end. Therefore, it is necessary to consider about the following 3 keywords to promote the development of biologics and their early practical use: they were “Registry”, “Centralization” and “Global cooperation”, all of which are related to the reduction of duration of a clinical trial.

**Conclusion:** To reduce the duration of clinical trial, it is essential to complete “a world-scale registry system” by developing the registry system established by Pediatric Rheumatology Association of Japan. Then, it is important to carefully plan to participate into the international network using “the world-scale registry system”, and aim at global cooperative trials in which we can ensure the sufficient number of entries from Japan.

**Disclosure of Interest**

M. Mori Grant / Research Support from: AbbVie GK, Ayumi Pharmaceutical,

### P379 SERIOUS OPPORTUNISTIC LOWER RESPIRATORY INFECTIONS IN PEDIATRIC RHEUMATIC DISEASES DURING TREATMENT WITH BIOLOGICS

#### Anne Katrine Ogstrup, Anne H. Spannow, Mette Holm, Sune Rubak, Jens E. Veirum, Mia Glerup, Troels Herlin

##### Pediatrics and Adolescent Medicine, Aarhus University Hospital, Aarhus , Denmark

###### **Correspondence:** Anne Katrine Ogstrup

**Introduction:** With the advent of targeted therapy as biologics for the treatment of juvenile idiopathic arthritis (JIA) and other pediatric rheumatic diseases outcome has improved considerably. However, a major concern in patients treated with these immune-modulators has been the increased risk of opportunistic infections.

**Objectives:** To explore the clinical and paraclinical findings following serious opportunistic lower respiratory tract infection (LRTI) observed in rheumatic children treated with immune-modulating therapy.

**Methods:** Single center study of consecutive cases with severe LRTI observed during a two-year period from January 1st 2016 to December 31^st^ 2017 in the Pediatric Rheumatology Clinic, Aarhus. Clinical data, as well as imaging and laboratory results were extracted from the medical records.

**Results:** We collected 4 patients (3 girls, 1 boy) diagnosed with severe opportunistic LRTI: Two with JIA (1 with systemic JIA and recurrent macrophage activating syndrome (MAS), 1 with rheumatoid factor negative polyarticular JIA, 1 with hypereosinophilic syndrome, 1 with mixed connective tissue disease (MCTD). Mean age at diagnosis was 115 months (range 50-186), disease duration at time of diagnosis of LRTI was 69 months (range 7-150). All had been treated with biologics (2 with 1 and 2 with 3 different drugs) for 23 months (range 2-63), all being pre-tested negative for latent tuberculosis infection. In addition, three patients received corticosteroids at time of LRTI diagnosis, duration of treatment was 30 months (range 5-66). Clinical symptoms were rather sparse. Mild cough was observed in two patients, but none had fever, tachypnea? or dyspnea. Auscultation of the lungs were normal in all patients. Elevation of the acute phase reactants (mean ESR 58 (range 8-103), mean CRP 71 mg/L (range 0.6 – 141)) was seen in 3 patients, which was not related to the activity of the rheumatic disease. Pulmonary abscess was detected in one patient by FDG-PET-CT. Aspirate from the abscess gave positive cultures with *Staphylococcus aureus*. In two of three patients high-resolution-CT scan of the lungs revealed multiple bilateral infiltrates, the third being normal. Bronchial alveolar lavage was performed in three patients resulting in the detection of *Pneumocystis jiroveci* in all three and in addition in one of these patients cultures with *Staphylococcus aureus* and *Aspergillus* galactomannan antigen (DNA) was found, but in neither of the three aspirates cultures with *Mycobacterium tuberculosis* were found. All patients recovered after receiving relevant antimicrobial therapy.

**Conclusion:** Children with rheumatic diseases treated with biologics with or without the combination of corticosteroids may have a rather asymptomatic course when infected with opportunistic lower respiratory tract infections. Comprehensive investigations of a possible infectious cause must be undertaken even if symptoms are mild or incongruent with abnormalities in ESR or other inflammatory parameters.

**Disclosure of Interest**

None Declared

### P380 RELATIONSHIP BETWEEN GENE POLYMORPHISM OF THE FOLATE EXCHANGE AND THE EFFICACY AND TOXICITY OF MTX IN THE TREATMENT OF CHILDREN WITH JIA

#### Elena Ponochevna^1^, Elena Okhotnikova^1^, Zoya Rossokha^2^, Svetlana Kyriachenko^2^, Larysa Sheiko^3^, Oksana Grischenko^4^, Maryana Mariyan^5^

##### ^1^Pediatric, National Medical Academy for Postgraduate Education Named After P.L.Shupyk; ^2^State Institution "Reference-centre for molecular diagnostic of Public Health Ministry of Ukraine"; ^3^Department of medical and laboratory genetics, National Medical Academy for Postgraduate Education Named After P.L.Shupyk; ^4^Pediatric, National children's specialized hospital ”OKHMATDYT”, ^5^Pediatric, National Academy of postgraduate education named after P. L. Shupyk, Kiev, Ukraine

###### **Correspondence:** Elena Ponochevna

**Introduction:** Methotrexate (MTX) is the “gold standard” treatment of juvenile idiopathic arthritis (JIA); however, this therapy is not always effective and predictable, most often in connection with toxicity.The aim of our study was to identify the relationship between inefficient assignment of MTX and the presence of folate exchange genes polymorphism.

**Objectives:** In the period between 2016 and 2017 in the Department of Pediatrics of the hospital “OKHMATDYT” 79 children between the ages of 5 and 18 (mediane 12.8 years old) were diagnosed with JIA. All children were initially treated with MTX. 15 children were also given biological therapy during the first year of treatment.

**Methods:** Due to the ineffectiveness of basic therapy and toxic effects of MTX(nausea, vomiting, toxic hepatitis), 11 children were further studied for the molecular genetic study of folate exchange genes polymorphism (MTHFR C677T, rs1801133 and A1298C, rs1801131; MTR1 A2756G; rs1805087; MTRR A66G; rs1801394; RFC1 G80A; rs1051266), homocysteine levels, folic acid and vitamin B12 in the serum. Of the 11 children examined, 4 had a systemic variant of JIA with the active arthritis, and 7 of them had a polyarticular variant, which was complicated by uveitis in 4 patients.

**Results:** Among the examined children, the frequency of polymorphous variants of the folate exchange genes were: for the MTHFR gene: 677CC - 55.56%, 677CT - 44.44% and 1298AA - 55.56% 1298AC - 33.33%, 1298CC - 11.11%. The genes of MTR1: 2756AA - 55.56%, 2756AG - 33.33%, 2756GG - 11.11%, and MTRR: 66AA - 44.44%, 66AG - 33.33%, 66GG - 22.22%. Distribution of polymorphous variants of the folate transporter gene RFC: 80GG -22,22 %%, 80GA-66,67%, 80AA-11,11%. 4 children had showed an increase in homocysteine levels: 5.9-8.6 μmol/l(norm-5 μmol / L), 2-increasing the level of vitamin В 12: 980-1530 pg/ml (norm-174-878 pg/ml), the level of folic acid in serum was within regulatory limits (normal 3-17 ng/ml). Children with higher level of homocysteine and vitamin B12 had systemic JIA.

**Conclusion:** Our research shows that when genes polymorphism of folate exchange is present, there is a high likelihood that MTX will be toxic and it can use as prognostic factor. The prognosis of efficacy is discretionary, since the side effects of MTX are dose-dependent and the working dose is probably not achieved due to early toxic effects. Further studies are needed to assess the importance of these markers to predict the effectiveness of MTX treatment.

**Trial registration identifying number:**

**Disclosure of Interest**

None Declared

### P381 SEROLOGIC RESPONSES TO VACCINES IN THIRTY PORTUGUESE CHILDREN WITH RHEUMATC DISEASES UNDER IMMUNOSSUPPRESSION

#### Helena Sousa^1^, Margarida P. Ramos^2^

##### ^1^Serviço de Pediatria, Hospital de Vila Franca de Xira, Vila Franca de Xira; ^2^Area da Pediatria Médica, Hospital de Dona Estefânia, Centro Hospitalar Lisboa Central, EPE, Lisboa, Portugal

###### **Correspondence:** Margarida P. Ramos

**Introduction:** It is being recognized that serologic response to vaccines may be impaired in pediatric patients with rheumatic diseases (pedRD), related to the disease and immunosuppression. In 2017, Portuguese National Vaccination Programme (PNV) postponed tetanus and diphtheria immunization from 20 to 25 years and may pose these patients at even higher risk. At the moment, there are no published data in Portuguese pedRd

**Objectives:** Evaluate serologic response to vaccines in Portuguese pedRD receiving immunosuppressive treatment (IT).

**Methods:** Thirty children with RD attended in a Pediatric Rheumatology Unit, under immunosuppression, were recruited between January to March of 2018. Blood analyses were processed in the same laboratory. Serum samples for Diphtheria-Tetanus (DT) antitoxin antibodies were obtained on the date of recruitment. Laboratory reference values for tetanus and diphtheria antitoxin antibodies (TAA and DAA) recommends: vaccination for values under 0.1 UI/mL (TAA and DAA), and booster for values under 0.5 (TAA) and 1 UI/mL (DAA). Anti-hepatitis A and anti-HBs antibodies were evaluated in different times before and along the treatment. Pos-vaccination anti-varicella-zoster virus (anti-VZV) IgG antibodies were measured during the first three months of IT. All had the PNV for the vaccines (V) analyzed, updated, previously and during IT. HBV and Tetanus-Diphtheria (TD) V were administrated, according the PNV (HBV:3 doses in the first year; TD: 3 doses in the first year, 18 months, 5 and 10 years). HAV and VZV are not included in the PNV. VZV was administrated in susceptible patients before starting IT. All had normal previous immunoglobulins and complement evaluation and no history of frequent or severe infections.

**Results:** 30 children (13±5 years); 19 female (63%) with diagnosis including juvenile idiopathic arthritis (JIA, n=18), SLE/others connective tissue disease (n=6), vasculitis (n=2), auto-inflammatory diseases (n=1), chronic uveitis (1); with mean disease duration of 42±37 months and total IT of 27±28 months. Almost all (n=27) had history of high IT for longer than 3 months. IT included biologics (anti-TNF) (n=8); Glucocorticoids (GC) (n=7), methotrexate (MTX) (n=19); Cyclosporine (n=2) and Ciclophosphamide (n=1). In the last three months 17/30 have been under high IT (of which 8 with anti-TNF). TAA was evaluated in 28/30, of those 7 (25%) revealed unprotected values (<0.5), in patients with ages ranging from 3 to 16 years (median 11±5 years). Six were on high IT (anti-TNF in 3; MTX+GC in 2; MTX alone in one), and one in “low” MTX dose (a 16-year-old). DAA was obtained in 27/30; with unprotected values (<1UI/mL) in 67%(n=18); of those, three had results under 0.1 UI/mL (16 year old under MTX “high”; 17 year-old with GC and MTX “low”; 8 year-old with MTX “high”). Pre-treatment AcHBs evaluation was negative in 15/30 (mean ages 16+/-3 years). Booster doses were administrated during IT in 6 of these; of those, 3 persisted negative (all under anti-TNF). HAV was administrated in 21/30 (nine one dose only; twelve two doses). Anti-HA persisted negative in 5/18 evaluated; 2 in the two doses group. VZV was administered in 9 patients, all 5 with subsequent serologic evaluation had protective values.

**Conclusion:** Even being a small sample, this is for the best of our knowledge, the first evaluation in Portuguese pedRD. These patients demonstrated impaired responses to the V evaluated and alerts to the real necessity of routine investigation of the humoral response and administration of booster V, even in younger ages. Also it does not seem very cautious to postpone Td vaccination from 20 to 25 years in these patients. More and better designed studies are needed.

**Disclosure of Interest**

None Declared

### P382 VACCINATION COVERAGE IN PORTUGUESE CHILDREN WITH RHEUMATIC DISEASES UNDERGOING IMMUNOSUPPRESSIVE THERAPY

#### Helena Sousa^1^, Sónia Carvalho^2^, Mariana Rodrigues^3^, Iva Brito^3^, Patrícia Costa Reis^4^, Teresa Rocha^3^, Margarida P. Ramos^5^, Maria José Santos^6^, Filipa O. Ramos^4^, Marta Cabral^7^, Margarida Guedes^8^

##### ^1^H. V. F. Xira, V. F. Xira; ^2^CHMA, Famalicão; ^3^H. S. João, Porto; ^4^H. Santa Maria; ^5^H. D. Estefânia, CHLC; ^6^H. Garcia Orta; ^7^H. Fernando Fonseca, Lisboa; ^8^CMIN, Porto, Portugal

###### **Correspondence:** Mariana Rodrigues

**Introduction:** Children with rheumatic diseases (RD) are at increased risk of infections, due to the immunosuppressive effect of the disease or its treatment. Effective and safe vaccination is key in reducing the burden of infections in this population. However, data on vaccination status in Portuguese children with RD remains scarce*.*

**Objectives:** Evaluate the vaccination status of Portuguese children with RD receiving immunosuppressive treatment (IT).

**Methods:** Multicentre study of consecutive patients with RD undergoing IT observed at a Pediatric Rheumatology Clinic, from January to March of 2018. Patients were evaluated regarding vaccination status and reasons for potential dropout. Demographic data and information about the disease, drug treatments and vaccinations were collected. Chi-squared test was used to study categorical variables.

The Portuguese National Vaccination Program (PNV) includes Hep B, IPV, DTaP, Hib, MenC, MMR, HPV for girls and PCV13 for children born after 2015. HPV for boys, varicella, other meningococcal and flu vaccines are not included. In 2015, children receiving IT became eligible for free pneumoccocal vaccines (PCV13 and PPV23).

**Results:** We studied 120 patients (66% female), mean age 8±5 years, from seven Portuguese Pediatric Rheumatology Units, with a diagnosis of JIA (n=82), uveitis (n=10), jSLE (n=10), other connective tissue diseases (n=7), vasculitis (n=5) and autoinflammatory diseases (n=2). Most patients had been on high dose IT (82%), including biologics (34%).

Before initiating IT 98% had an updated PNV and 73% had received one dose of PCV13. At least one dose of Flu and MenB vaccines was given in 12 and 5% of the patients, respectively.

Time elapsed between diagnosis and IT (> or < 3 months) was not associated with the prescription of extra-PNV vaccines before treatment, namely varicella.

During IT, immunizations were in accordance to the PNV in 91% of the patients. Also, 28 patients were eligible for MMR vaccination (eg, low dose methotrexate), but 11 did not receive it. MMR was administered in six patients under high dose IT (including three with biologics), but no complications were reported. Adequate pneumococcal vaccination was performed in 30%; 74% of patients received flu vaccine before or during IT. No boys received HPV.

Patients on high-dose IT did not receive more extra-PNV vaccines than patients with low-dose IT.

Six cases of varicella in unvaccinated patients were reported during treatment, and three required hospitalization.

**Conclusion:** Vaccination rates in Portuguese children with RD are sub-optimal.

The low prevalence of extra-PNV safe recommended vaccines (such as other meningococcal) can be partially explained by their high cost.

Despite the small sample, MMR vaccination during high-dose IT patients was not associated with complications. Large controlled studies are required to evaluate the safety of live attenuated vaccines, especially with high IT and/or during outbreaks.

Since primary health care services in Portugal are overall responsible for vaccinations, optimal communication with tertiary services is indispensable to ensure that the best care is provided.

It is essential to promote compliance with recommendations about vaccination of children with RD and provide free recommended vaccines to this group of patients.

**Disclosure of Interest**

None Declared

### P383 EFFICACY OF THE REINTRODUCTION OF TREATMENT WITH ANTI-TNF IN PATIENTS WITH JIA

#### Lucía Rodríguez, Juan Manuel Mosquera, Andrea Zacarías, Joan Calzada, Estibaliz Iglesias, Rosa Bou, Violeta Bittermann, Judith Sánchez, Clara Jimenez, Jordi Antón

##### Hospital Sant Joan de Deu, Esplugues de Llobregat, Barcelona, Barcelona, Spain

###### **Correspondence:** Lucía Rodríguez

**Introduction:** In our clinical practice, once the remission with a biological agent is reached, we try to withdraw the medication, but we have 82% of relapses.

**Objectives:** To analyze the response to the reintroduction of anti-TNF treatment in patients with Juvenile Idiopathic Arthritis (JIA).

**Methods:** Unicentric, retrospective study, through review of clinical history, of the years 2015 and 2016.

The Wallace criteria were used to measure inactivity and the disease was classified as inactive, remission with treatment and remission without treatment. Through JADAS-71 we defined remission, minimal disease activity and acceptable symptoms.

**Results:** 633 patients with JIA were visited, of which 31.3% were treated with a biological agent. Of the patients with non-systemic JIA treated with anti-TNF, 24 patients restarted administration after suspension because of remission. The JADAS-71 at relapse was 7.8 ± 6.6 (median 6.1, range: 1.8-32.2). Time to reach inactivity after restart was 5.26 ± 3.55 months (median 4.83 months, range: 0.93-12.17). An 87.5% reached inactivity upon reintroduction. Patients who did not reach inactivity, had a reduction in JADAS-71 of 72.27% compared to the time of reintroduction of anti-TNF.

**Conclusion:** Patients who restart treatment with anti-TNF after remission have a good response. More studies are needed to identify which factors are associated with a worse response to reintroduction.

**Disclosure of Interest**: None Declared

### P384 LONGITUDINAL EFFECTIVENESS OF ABATACEPT IN JUVENILE IDIOPATHIC ARTHRITIS: RESULTS FROM AN ONGOING JIA REGISTRY

#### Nicolino Ruperto^1^, Daniel J. Lovell^2^, Nikolay Tzaribachev^1^, Andrew Zeft^2^, Rolando Cimaz^1^, Valda Stanevica^1^, Gerd Horneff^1^, John Bohnsack^2^, Thomas A. Griffin^2^, Ruy Carrasco^2^, Maria Trachana^1^, Jason A. Dare^2^, Ivan Foeldvari^1^, Richard K. Vehe^2^, Teresa A. Simon^3^, Alberto Martini^1^, Hermine I. Brunner^2^

##### ^1^PRINTO, Istituto Gaslini, Genoa, Italy; ^2^PRCSG, CHMC Cincinnati, Cincinnati; ^3^Bristol-Myers Squibb, Princeton, USA

###### **Correspondence:** Nicolino Ruperto

**Introduction:** Abatacept (ABA) is a selective T-cell co-stimulation modulator approved for use in juvenile idiopathic arthritis (JIA). Efficacy and safety of ABA in patients (pts) with JIA has been demonstrated in two Phase III studies.^1,2^ This ongoing study provides additional monitoring in a real-world setting.

**Objectives:** Describe the longitudinal effectiveness of ABA in pts with JIA using registry data.

**Methods:** Using a standardized protocol, clinical sites in the Pediatric Rheumatology Collaborative Study Group and Paediatric Rheumatology International Trial Organization enrolled pts with JIA currently taking/starting ABA in this longitudinal registry. Planned duration of follow-up is 10 years (yrs); data shown were collected up to March 31, 2017 (3.5 yrs of follow-up). Follow-up effectiveness of ABA was assessed based on disease activity at baseline (BL), 3 and 6 months (mths), and 1, 2, 3 and 3.5 yrs. Safety data were collected at each visit.

**Results:** 367 pts with JIA were enrolled; 354 were included in this analysis. Total ABA exposure was 364.5 pt-yrs (median); ABA treatment duration was 6.5 mths. At BL, 281 (79%) pts were female, age was 13.6 yrs (median), 15 (4%) pts were aged 2–5 yrs, JIA duration was 4.3 yrs and active joint count was 0 (mean 2.9). JIA categories were systemic (2%), oligoarticular (22%), polyarticular RF– (57%), polyarticular RF+ (9%), psoriatic (3%), enthesitis-related (3%) and undifferentiated (4%). At 1-yr follow-up, pts had low Physician Global Assessment of Disease Activity, low Juvenile Arthritis Multidimensional *Assessment* Report (JAMAR) scores and improved joint assessment (Table 1). A higher percentage of pts achieved clinical inactive disease after 1 yr of follow-up vs BL (Table 1). This trend continued despite low numbers of pts with 3 and 3.5 yrs of follow-up. During the last 6 mths of follow-up (3–3.5 yrs), seven new serious AEs were reported, but no new cases of treatment-related serious infection, malignancy or tuberculosis were observed.^3^

**Conclusion:** In this JIA cohort, abatacept was safe and well tolerated with no new safety risks identified.^1-3^ This longitudinal analysis further supports the persistent effectiveness of abatacept in pts with JIA.^1-3^

**References**

1. Brunner HI, et al. *Arthritis Rheumatol*. 2018; doi: 10.1002/art.40466

2. Ruperto N, et al. *Lancet* 2008:**372**:383–91.

3. Lovell DJ, et al. *Arthritis Rheumatol* 2017;**69**(Suppl 10): abstract 2272.

4. Wallace C, et al. *Arthritis Care Res (Hoboken)* 2011;**63**:929–36.

**Disclosure of Interest**

N. Ruperto Grant / Research Support from: Bristol-Myers Squibb, Roche, Janssen, Novartis, Pfizer, Sobi, Consultant for: AbbVie, Ablynx, Amgen, AstraZeneca, Baxalta Biosimilars, Biogen Idec, Boehringer Ingelheim, Bristol-Myers Squibb, Celgene, Eli-Lilly, EMD Serono, Gilead Sciences, Janssen, MedImmune, Novartis, Pfizer, R-Pharm, Roche, Sanofi, Servier, Takeda, Speaker Bureau of: AbbVie, Ablynx, Amgen, AstraZeneca, Baxalta Biosimilars, Biogen Idec, Boehringer Ingelheim, Bristol-Myers Squibb, Celgene, Eli-Lilly, EMD Serono, Gilead Sciences, Janssen, MedImmune, Novartis, Pfizer, R-Pharm, Roche, Sanofi, Servier, Takeda, D. Lovell Grant / Research Support from: AbbVie, Bristol-Myers Squibb, NIH, Pfizer, Roche, Consultant for: AbbVie, Boehringer Ingelheim, Bristol-Myers Squibb, Celgene, Genentech, GlaxoSmithKline, Janssen, Johnson & Johnson, Novartis, Takeda, UCB, Speaker Bureau of: Genentech, N. Tzaribachev: None Declared, A. Zeft: None Declared, R. Cimaz: None Declared, V. Stanevica: None Declared, G. Horneff Speaker Bureau of: AbbVie, Pfizer, Chugai, Roche, Novartis, J. Bohnsack: None Declared, T. Griffin: None Declared, R. Carrasco Grant / Research Support from: Bristol-Myers Squibb, Pfizer, Consultant for: Non-remunerative positions of influence at Forest Research, Speaker Bureau of: Genetech, M. Trachana: None Declared, J. Dare Grant / Research Support from: AbbVie, AstraZeneca, Bristol-Myers Squibb, Horizon Pharma, Medac, Pfizer, Roche, UCB, I. Foeldvari Consultant for: Bayer, Genentech, Medac, Novartis, R. Vehe: None Declared, T. Simon Shareholder of: Bristol-Myers Squibb, Employee of: Bristol-Myers Squibb, A. Martini Grant / Research Support from: Istituto Giannina Gaslini has received contributions from: Bristol-Myers Squibb, Roche, Janssen, Novartis, Pfizer, Sobi for the coordination activity of the PRINTO network, Consultant for: on behalf of the Istituto Giannina Gaslini for AbbVie, Boehringer Ingelheim, Novartis, R-Pharm, H. Brunner Consultant for: AstraZeneca, Bristol-Myers Squibb, Genentech, Janssen, Novartis, Pfizer, Sanofi, Takeda, Speaker Bureau of: Genentech, Novartis

**Table 1 (abstract P384).** Assessment of Disease Activity

Endpoint	BL (n=354)	3 mths (n=286)	6 mths (n=252)	1 yr (n=231)	2 yrs (n=81)	3 yrs (n=27)	3.5 yrs (n=14)
Physician Global Assessment of Disease Activity*^3^	2.0 (0.1)	1.5 (0.1)	1.5 (0.1)	1.2 (0.1)	0.8 (0.1)	1.2 (0.3)	1.1 (0.4)
Clinical inactive disease (Wallace criteria), %^3,4^	33	32	38	48	52	37	29
Joint assessment	3.1 (0.4)	2.7 (0.4)	2.8 (0.4)	2.1 (0.4)	1.1 (0.3)	1.9 (1.0)	2.1 (0.9)
Tenderness/pain							
On motion	2.9 (0.3)						
Active		2.0 (0.3)	2.2 (0.3)	1.8 (0.3)	1.2 (0.4)	3.0 (1.2)	2.6 (1.2)
JAMAR functional^†^							
Child^3^	5.4 (0.4)	4.7 (0.4)	4.1 (0.4)	4.1 (0.4)	3.0 (0.5)	3.5 (0.9)	4.6 (1.5)
Parent	7.2 (0.7)	5.7 (0.5)	4.4 (0.4)	4.9 (0.9)	2.9 (0.5)	4.1 (1.2)	5.3 (1.8)
JAMAR HRQoL^‡^							
Child^3^	7.0 (0.3)	6.0 (0.4)	5.5 (0.3)	5.1 (0.4)	4.5 (0.6)	6.5 (1.1)	7.0 (1.5)
Parent	8.3 (0.7)	7.0 (0.6)	6.8 (0.7)	6.3 (0.9)	6.4 (1.7)	4.4 (1.1)	7.1 (2.9)

Data are mean (SE), unless otherwise indicated

*Visual analogue scale 0–10; 0=inactive; ^†^range 0–15, 0=no functional limitation; ^‡^range 0–15, 0=best possible HRQoL

### P385 MALIGNANCY AND JUVENILE IDIOPATHIC ARTHRITIS IN BIOLOGIC NAIVE PATIENTS : JIA SINGLE TERTIARY CENTER REGISTRY DATA

#### Velma Selmanovic^1^, Aida Omercahic-Dizdarevic^2^, Adisa Cengic^1^, Edo Hasanbegovic^3^, Meliha Sakic^3^, Senada Mehadzic^3^, Ismail Lutolli^3^, Mirna Sarajlic^1^

##### ^1^Allergology, Rheumatology and Clinical Immunology, Children's Hospital University Clinical Center; ^2^Allergology, rheumatology and Clinical Immunology, Children's Hospital, University Clinical Center; ^3^Hematooncology, Children's Hospital University Clinical Center , Sarajevo, Bosnia and Herzegovina

###### **Correspondence:** Velma Selmanovic

**Introduction:** Children with juvenile idiopathic arthritis (JIA) appear to have an increased rate of incident malignancy compared to children without JIA, although data are inconsistent.^1,4^ The relationship between JIA and malignancy is uncertain. High incidence risk of new autoimmune disease in patients affected with JIA was established.^2^ Childhood cancer survivors are at increased risk for certain types of autoimmune diseases (AID)^3^ and there is lack of data of JIA in those patients.

**Objectives:** To evaluate the frequency of malignant disease in a cohort of patients included in JIA Registry in single tertiary center, to assess the time of manifestation onset with respect to the time of diagnosis of JIA and to assess presence of another autoimmune disease in the same patient.

**Methods:** medical records of registered patients were reviewed. Data on JIA diagnosis, malignant and autoimmune disease were analyzed. The time of each manifestation onset was determined and compared with the time of JIA diagnosis.

**Results:** as of the May 2018, 176 patients were registered in er JIA Registry in single tertiary cent (females 118 (67%), males 58 (33%)). Three patients (1,7%) were identified with malignancy in biological naive patients. Patient 1, boy, with unremarkable personal and family history regarding autoimmune and malignant disease, was diagnosed as systemic onset JIA and synovial sarcoma at the same time. Treated accordingly for SoJIA and malignancy (surgery, followed with hemotherapy (CWS 2012 Protocol: iphosphamide, vincristin, actinomicin) and radiotherapy. On month 10th of therapy (while receiving radiotherapy) developed macrophage activation sy as well as diagnosed as autoimmune thyroiditis (AIT). Patient 2, girl 15,5y, developed hard to treat enthesitis-related arthritis while on maintenance therapy (MTX 50 mg/weekly) for T-cell Non – Hodgkin lymphoma (ALL BFM 2009 Protocol) previously diagnosed also as AIT. Patient 3, boy 15y, diagnosed as juvenile spondiloarthropaty, with history of allogeneic bone marrow transplantation (BMT) at age 6y for acute myeloid leukemia, AIT and hyperinsulinemia. Patients were not receiving biologics.

**Conclusion:** In JIA, cancer is a relatively rare event, which is reassuring for patients and physicians. Registry data revealed 3 patients with JIA, malignancy and hypothyreosis at the same time. Synovial sarcoma and systemic onset JIA is quite rare comorbidity. Two other patients developed JIA during of after treatment for hematological malignancy – we don't know if it could be atribute to genetic instability or something else. Persistent immune abnormalities after treatment with chemotherapy predispose to the development of autoantibodies, which are central to the pathogenesis of many autoimmune diseases, probably including JIA . Long-term follow up and large sample in JIA registry are warranted.

**Disclosure of Interest**

None Declared

### P386 RITUXIMAB IN REFRACTORY JUVENILE ONSET RHEUMATIC DISEASES: REPORT OF 10 CASES

#### Sandra Sousa^1^, Ana C. Duarte^1^, Filipa Oliveira-Ramos^2,3^, Maria J. Santos^1,3^

##### ^1^Serviço de Reumatologia, Hospital Garcia de Orta, Almada; ^2^Unidade de Reumatologia Pediátrica, Serviço de Reumatologia e Doenças Ósseas Metabólicas, Hospital de Santa Maria, CHLN, Centro Académico de Medicina de Lisboa; ^3^Unidade de Investigação em Reumatologia, Instituto de Medicina Molecular, Faculdade de Medicina, Universidade de Lisboa, Centro Académico de Medicina de Lisboa, Lisboa, Lisboa, Portugal

###### **Correspondence:** Sandra Sousa

**Introduction:** Rituximab (RTX) is a chimeric monoclonal antibody directed against the CD20 antigen on the surface of B lymphocytes. It binds to CD20 and causes B cells death by different mechanisms. In what concerns rheumatic diseases, RTX is only approved for the treatment of rheumatoid arthritis and ANCA-associated vasculitis in adults. However, there is growing evidence of its utility in other diseases, including in pediatric patients.

**Objectives:** We aim to report the efficacy and safety of rituximab in pediatric patients diagnosed with juvenile onset rheumatic diseases refractory to conventional treatment.

**Methods:** A retrospective review was made of all medical records of patients under 18 years old treated with RTX in two pediatric rheumatology units in Lisbon.

**Results:** Ten patients, 8 girls, 8 Caucasian/2 African, mean age of diagnosis 13.8 (min 8-max 18) years, were treated with RTX: 6 with systemic lupus erythematosus (JSLE), 2 with juvenile dermatomyositis (JDM), 1 with polyarteritis nodosa (PN) and 1 with ANCA-associated vasculitis (AAV). All of them were refractory to previous treatments that included oral prednisolone (n=10), methylprednisolone pulses (n=3, 2 JSLE, 1 AAV), cyclophosphamide pulses (n=5, 2 JSLE; 1 PN, 2 JDM), methotrexate (n=2, 1 JSLE, 1 JDM), hydroxychloroquine (n=5, 4 JSLE, 1 JDM), mycophenolate mofetil (n=3, 3 JSLE), azathioprine (n=9, all except 1 JDM), IvIg (n=3, 2 JSLE, 1 JDM) and infliximab (n=1, 1 JDM).

The mean disease duration until receiving RTX was 26 (min 5-max 68) months. Renal involvement (3 class IV lupus nephritis and 1 pauci immune glomerulonephritis) was the main indication for RTX.

In total, they received 21 cycles of RTX (min 1-max 4). The scheme of RTX frequently used was 1000 mg two weeks apart, except for the PN and 2 JDM that used 375 mg/m^2^ four weeks and 575 mg/m^2^ in two weeks apart, respectively. The average time between cycles was 12 (min 6-max 26) months.

In all but one patient, disease activity improved when comparing the period before and after RTX. The SLEDAI score for JSLE, decreased from a mean of 10.8 (min 8–max 17) to 3.8 (min 2–max 5); the BVAS score for AAV decreased from 17 to 0, and the CMAS score for JDM also improved from a mean of 20 to 51, as well as the MMT8 score from a mean of 54 to 75. The patient with PN patient did not respond to RTX, the BVAS score got worse from 5 to 30 and the patient was switched to infliximab with good response until nowadays.

Adverse events occurred in 1 patient who had hypogammaglobulinemia without the need of immunoglobulin replacement.

**Conclusion:** Rituximab might have a role in the treatment of refractory pediatric rheumatic diseases. However controlled studies and long term follow-up are needed to evaluate its safety and efficacy.

**Disclosure of Interest**

None Declared

### P387 TRANSDERMAL DELIVERY OF METHOTREXATE USING POLYVINYL ALCOHOL- BASED HYDROGEL FORMING MICRONEEDLES: BIOAVAILABILITY AND PHARMACOKINETICS AFTER MULTIPLE DOSING IN RAT MODEL

#### Ismaiel A. Tekko^1^, Gaoyun Chen^1^, Ryan F. Donnelly^1^, James McElnay^1^, Helen McCarthy^1^, Madeleine Rooney^2^

##### ^1^School of Pharmacy; ^2^Centre for Experimental Medicine, Queen`s University Belfast, Belfast, UK

###### **Correspondence:** Ismaiel A. Tekko

**Introduction:** Methotrexate (MTX) is the only disease modifying drug used in the treatment of Juvenile idiopathic Arthritis (JIA), other than biologics. Its oral administration is mostly associated with nausea and vomiting. The alternative subcutaneous injections are painful and very stressful for children causing considerable anxiety [1]. In previous studies, we have reported a novel polyvinyl alcohol (PVA)- based hydrogel-forming microneedle patch as a minimally invasive, pain-free drug delivery system which was able to deliver MTX transdermally in a controlled and a sustained manner in both *in vitro* and *in vivo* settings [2,3].

**Objectives:** To evaluate the robustness, relative bioavailability and pharmacokinetics of MTX transdermally delivered from its PVA-based hydrogel-forming microneedle patch (HFM-patch) after single and multiple dose in comparison with oral and subcutaneous administration in hairless Sprague Dawley rats.

**Methods:** A total of 24 hairless Sprague Dawley rats were randomly divided into 4 cohorts (6 per cohort). A dose of 2.56 ± 0.1 mg/kg MTX was orally administrated to cohort 1. A dose of 2.56 ± 0.1 mg/kg MTX was subcutaneously administered into the back of each rat in the second cohort. Two HFM- patches containing MTX dose of 5.2 ± 0.5 mg/kg was applied onto the back of each animal in the third cohort. These were applied once weekly for three weeks. The forth cohort was used as a negative control. Blood samples were collected at predetermined time intervals over 21 days. MTX and its polyglutamates (MTX-PGs _2-5_) were quantified by a developed and validated HPLC-MS method.

**Results:** Following the first dose, the maximum blood concentration for MTX after SC, oral and transdermal administration, 373.4 ± 37.5, 62.10 ± 3.4, and 33.8 ± 5.9 nM/mL, respectively and this was achieved at 0.5, 1 and 24hrs, respectively after dosing. The AUC 0→ 48 was, 1136, 588 and 1103 nM/mL/hr, respectively. The relative bioavailability of MTX after oral administration in comparison with SC was 51.8%, while it was 44.2% after transdermal dosing, correcting for the dose difference.

HFM-patch delivered MTX in a controlled and sustained manner over the patch application period i.e. 24 hrs with no high bolus blood concentrations. This is essentially important in reducing the drug related side effects such as nausea and vomiting.

Nearly similar pharmacokinetic profile trend were also produced after the 2^nd^ and 3^rd^ dose. This suggests that the HFM-patch can deliver MTX at fairly consistent rates.

The other MTX polyglutamates (MTX-PGs_2-5_) appeared after the first dose at various rates and increased gradually over the study period.

**Conclusion:** PVA-based HFM-patch is an efficient, minimally invasive and robust transdermal drug delivery system can reliably deliver a reasonably consistent doses of MTX in a controlled and sustained manner. This delivery system for MTX could to be used in treatment of JIA and other diseases. Further studies is needed to evaluate its safety and efficiency in a long term studies.

**Disclosure of Interest**

None Declared

### P388 A LARGE PROPORTION OF PATIENTS WITH NON-SEPTIC ACUTE ARTHRITIS RECEIVE UNNECESSARY INVASIVE TREATMENTS

#### Marion Thomas, Ulrich Meinzer

##### Pédiatrie générale, maladies infectieuses et médecine interne, Hôpital Robert Debré, PARIS, France

###### **Correspondence:** Marion Thomas

**Introduction:** Acute arthritis, a common cause of consultation in paediatric emergency wards, can be categorized as septic (SA), juvenile idiopathic arthritis (JIA), and undetermined arthritis (UA). An early accurate diagnosis is essential to provide appropriate treatment and follow-up.

**Objectives:** The aim of this study was to determine whether clinical and biological data may allow early differentiation between septic and non-septic arthritis.

**Methods:** We retrospectively analysed clinical and biological data corresponding to a joint aspiration collection from patients treated at the Robert-Debré University Hospital in Paris between 9/2015 and 9/2017. The initial and final diagnoses were recorded. Patients were classified as having SA, JIA defined according to ILAR criteria, UA or other diagnoses. If no follow up was available, final diagnosis of SA was considered if bacteria were identified on culture or PCR, otherwise diagnosis of UA remained.

**Results:** 129 joint aspiration samples from 118 patients were analysed. The final diagnosis of acute arthritis was SA for 45 patients (38.1%), UA for 39 patients (33%), JIA for 23 patients (19.5%) and 10 patients (8.5%) had other diagnoses. Clinical and biological findings did not show major differences between the three groups. Antibiotics were administered in 97.8% of patients with SA, as well as in 39.1% of patients with a final diagnosis of JIA and 84.2% with UA. Arthrotomy was performed in 62.2% patients having SA, 30.4% of patients with JIA, 46.1% of patients with UA and 50% patients with other diagnosis.

**Conclusion:** We did not identify any clinical or biological parameters allowing us to distinguish SA versus arthritis of other causes. However, this study shows that a large proportion of patients with non-septic arthritis receive unnecessary invasive treatments, highlighting the urgent need for identification of new diagnosis markers.

**Disclosure of Interest**

None Declared

### P389 ASSESSMENT OF VACCINATION COVERAGE AMONG PEDIATRIC PATIENTS WITH RHEUMATIC DISEASES

#### Maria Trachana^1^, Maria Stoila^1^, Anna Kirchegina^2^, Despoina-Christina Pavlidou^1^, Polyxeni Pratsidou-Gertsi^1^

##### ^1^First Department of Pediatrics, Aristotle University, Pediatric Immunology and Rheumatology Referral Center, Ippokration Hospital, Thessaloniki; ^2^Deparment of Ophthalmology, Laikon Hospital, Athens, Greece

###### **Correspondence:** Maria Trachana

**Introduction:** Patients with Pediatric Rheumatic Diseases (PRD) due to their immunomodulatory treatment are at risk for infections.

**Objectives:** Primary: To study the vaccination coverage of children and adolescents with PRD due to the lack of relative Greek studies. Secondary: To investigate the reasons of incomplete vaccinations.

**Methods:** In this case-control prospective study, all patients of the Referral Center with PRD were enrolled, following a patient/parent consent over a 6-mo period (1/2017 -7/2017). Demographics and disease characteristics were recorded in a pre-formatted registry along with their vaccination status according to their personal Child Health Booklet. Additionally, the vaccinations of age-matched healthy students (HS) were collected. The vaccination coverage was estimated according to the National Immunization Program for the relevant age group.

**Results:** The vaccination status of 101 children/adolescents (Male: Female 32:69) with PRD and a mean age of 11 years was recorded. The control group was a population of 66 HS (Male: Female 25:41), with a mean age of 9.76 years. 45.5% of patients with PRD and 42.40% of HS were  found with missing immunizations, mainly due to their physician’s recommendation (p=0.81). In respect to the type of vaccination administered, complete vaccination for DTP-Hib was recorded in 60% of the PRD patients and 68.2% of the HS (p=0.32); for pneumococcus the relevant percentages of the studied groups were 90% and 92. 4%, respectively (p=0.65). One dose for measles’s protection with MMR had 97% of the PRD and 100% of HS (p=0.26) and for HPV 18.18% and 0%, respectively (p=0.21). As for the influenza vaccination, at least one dose was recorded in 40% of the PRD and 15.15% of the HS (p=0.001).

**Conclusion:** Vaccination coverage of Greek pediatric patients with PRD is not inferior to the relevant of HS, indicating the acceptance and compliance to the recommended National Immunization Program, especially for pathogens leading to invasive infections. About half of the PRD patients comply with the EULAR influenza vaccination recommendation. In HS, the absence of regarding HPV and lower level of influenza immunization is in alliance to the National Immunization Program regarding age and disease indications.

**Disclosure of Interest**

None Declared

### P390 THE POSSIBILITY OF INCREASING THE INTERVALS BETWEEN INJECTIONS OF TNF-ALFA INHIBITORS IN CHILDREN WITH JUVENILE IDIOPATHIC ARTHRITIS: A SINGLE-CENTER OBSERVATIONAL STUDY

#### Nadezhda Tsurikova, Elena Ligostaeva, Irina Tsiganok, Vadim Avdeenko, Natalia Kobzeva

##### Rostov Regional Children Hospital, Rostov-on-don, Russian Federation

###### **Correspondence:** Nadezhda Tsurikova

**Introduction:** Nowadays a lot of patients, who are suffering from juvenile idiopathic arthritis (JIA), are treated with anti-TNF therapy. The issues of duration of anti-TNF therapy in children with JIA, receiving anti-TNF therapy and reached remission on it, are relevant and debatable.

**Objectives:** To evaluate the efficiency of anti-TNF therapy after 3 years of treatment with TNF-alpha inhibitors in children suffering from JIA and having a remission of at least one year, which was achieved on anti-TNF therapy, in 2 compared groups on different treatment regimens.

**Methods:** This prospective study included 40 children (mean age 12.7±2.4) suffering from JIA (mean disease duration 5.2±1.4), receiving anti-TNF-therapy for 3 consecutive years (20(50%) children were treated with adalimumab(ADA), 20(50%) – with etanercept(ETA)). All children also received methotrexate(MTX) (mean duration of MTX therapy was 4.6±0.5) and had a remission (mean duration of remission was 1.4±0.3). The first group included patients who after 3 years of anti-TNF therapy continued anti-TNF therapy for another 6 months in standard dose and in standard scheme (this group included 10(25%) children who were treated with ADA and 10(25%) children who were treated with ETA). The second group included children who after 3 years of anti-TNF therapy continued anti-TNF therapy for the next 6 months in standard dose, but the intervals between injections of TNF-alpha inhibitors were doubled (this group also included 10(25%) children who were treated with ADA and 10(25%) children who were treated with ETA). Both groups were comparable in age, disease duration, and duration of treatment with both MTX and TNF-alfa inhibitors. Efficacy was determined using the American College of Rheumatology (ACR) Pediatric (Pedi) response criteria at the time of inclusion in this study and after 12 and 24 weeks.

**Results:** At the time of inclusion in our study 90% improvement according to ACR pedi criteria were registered in both groups. After 12 weeks of treatment all children in the first group maintained 90% improvement according to ACR pedi criteria, in the comparison group in 100% of children were registered 70% improvement according to ACR pedi criteria, and 18(90%) children had 90% improvement according to ACR pedi criteria. After 24 weeks of treatment in all children (100%) in the first group remained 90% improvement according to ACR pedi criteria, in the comparison group 4(20%) children didn’t achieve 30% improvement according to ACR pedi criteria (2 children were treated with ADA, 2 – with ETA), 16(80%) patients had 70% improvement according to ACR pedi criteria, and 15 (75%) children – 90% improvement according to ACR pedi criteria.

**Conclusion:** According to the obtained data, we can conclude that after 3 years of therapy with TNF-alpha inhibitors (ADA or ETA) in the standard dose, standard scheme and with the presence of remission for at least one year, it is possible to correct anti-TNF therapy by increasing the intervals between the injections of ADA or ETA.

**Disclosure of Interest**

None Declared

### P391 RATES OF TUBERCULOSIS INFECTION IN PATIENTS WITH JUVENILE IDIOPATHIC ARTHRITIS PRECEDING ANTI-TNF THERAPY FOR MORE THAN 2 YEARS

#### Nadezhda Tsurikova, Elena Ligostaeva, Irina Tsiganok, Vadim Avdeenko, Natalia Kobzeva

##### Rostov Regional Children Hospital, Rostov-on-don, Russian Federation

###### **Correspondence:** Nadezhda Tsurikova

**Introduction:** Nowadays awareness about rates of latent tuberculosis infection (LTBI) in patients with juvenile idiopathic arthritis preceding anti-TNF therapy for a long period of time is very relevant.

**Objectives:** To evaluate rates of LTBI in patients with juvenile idiopathic arthritis preceding anti-TNF therapy for more than 2 years.

**Methods:** This was a retrospective study that included JIA patients treated with anti-TNF therapy for more than 2 years. 43 JIA patients with current age of 10.1±3.4 years, mean disease duration of 3.8±1.9 years were included. All patients received a single anti-TNF: 24(55.8%) patients were treated with etanercept, another 19(44.2%) patients were treated with adalimumab. All patients were screened for LTBI prior to anti-TNF using tuberculin skin test (TST), chest computer tomography (CT) and history of exposure to tuberculosis. Patients were regularly followed at 6-month intervals. Before patients started receive anti-TNF therapy, LTBI screening (solely TST-positive) was positive in 7(16.2%) patients: 4(9.3%) patients in group of etanercept and 3(6.9%) - in group of adalimumab, no one had active tuberculosis (TB) or history of TB exposure. All this patients were treated with standard course of anti-TB therapy before the beginning of anti-TNF therapy.

**Results:** Through more than 2 years duration anti-TNF therapy in patients with JIA, LTBI screening was positive in 11(25.5%) patients: 2(4.6%) had TST-positive (1(2.3%) patient was treated with adalimumab and 1(2.3%) with etanercept) and history of TB exposure, 7(16.2%) had solely TST-positive (3(6.9%) patients were treated with etanercept and 4(9.3%) with adalimumab), 1(2.3%) patient had active TB which was diagnosed with positive TST and changes on chest CT (patient was treated with adalimumab).

**Conclusion:** According to our research LTBI appears to be prevalent among patients treated with adalimumab. Screening for LTBI in patients on anti-TNF therapy may reduce risk of active tuberculosis, however, current methods may not be fully effective.

**Disclosure of Interest**

None Declared

### P392 EXPERIENCE OF BIOLOGICAL AGENTS: EVALUATION OF 80 CASES

#### Serkan Turkucar, Ceyhun Acari, Hatice Adiguzel Dundar, Sevket E. Unsal

##### Departments of Pediatrics, Pediatric Rheumatology, Dokuz Eylul University Faculty of Medicine, Izmir, Turkey

###### **Correspondence:** Serkan Turkucar

**Introduction:** Controlling of systemic inflammation is possible by blockage of some specific proteins and cells in inflammatory steps that play a role in the etiopathogenesis of rheumatic diseases.

**Objectives:** Our clinical experiments with biological agents such as etanercept, canakinumab, adalimumab and tocilizumab, which are used to inhibit TNF-α, IL-1 and IL-6, are presented in this study.

**Methods:** Demographic, clinical and laboratory data of 80 children with rheumatic diseases were reviewed. Tuberculosis incidence and other side effects were noted.

**Results:** Study population consisted of 80 cases, include 39 (48.8%) male and 41 (51.2%) female. Twelve cases had (15%) systemic juvenile idiopathic arthritis (S.JIA), 24 (30%) polyarticular JIA (P.JIA), 11 (17.5%) oligoarticular JIA (EIA), 22 (27.7%) familial Mediterranean fever (FMF), 3 (3.75%) hyper immunoglobulin D syndrome (HIDS), 1 (1.25%) Cryoprin-associated periodic syndrome (CAPS) and one case of psoriatic arthritis (JPsA). There was a special case with Farber disease receiving tocilizumab. Fourteen cases (17.5%) were on canakinumab, 17 (21.25%) on etanercept, 17 (21.25%) on tocilizumab , and 32 (40%) were on adalimumab. In addition, 23 cases needed to switch from one biologic to another in order to control the disease. . Demographic, clinical and laboratory evaluation of cases with receiving biological treatments are presented in table.

**Conclusion:** The use of biological agents in paediatric rheumatology practice is increasing in order to better control of the disease with much less damage and increased quality of life. This study results support this fact, and cases resistant to conventional DMARDs went into remission following biologic treatment. In this evaluation, we noticed clinical and laboratory remission in our resistant cases in a short time after biological treatment. Although upper and lower respiratory tract infections, tuberculosis and malignancy have been observed in some cases, the relationship between treatment and infections has not been fully documented. Since the use of biological agents increases to suppress inflammation in uncontrolled rheumatic diseases day by day, there is a need for studies and evaluations in wider case series across the national spectrum in case of efficacy and side effect profiles.

**Disclosure of Interest**

None Declared

**Table 1 (abstract P392).** Cases with receiving biological treatments (n=80)

Sex	%48.8 male (n=39) %51.2 female (n=41)
Onset of Symptoms (year)	4.41 (0.16-17)
Diagnosis time (year)	7 (0.66-16)
Family History of Rheumatic Disorders	%26.25 (n=21)
Receiving Steroid Treatment	%37.5 (n=30)
Types of MEFV mutations	27.5% (n=22) MEFV mutation (+) 63.6% (n= 14) exon-10 mutations 54.5% (n=12) have M694V mutations 31.8% (n=7) have M694V homozygous
ANA positivity	27.5 (n=22)
Infection and malignancy after treatment	Tuberculosis infection (n=1) Varicella zoster (n=1) Recurrent upper respiratory tract infections (n=2) Acute Lymphoblastic Leukaemia (n=1)
Need of Tuberculosis Prophylaxis	%26.25 (n=21)
Mean Remission Time (months)	2.00 (1-18)

### P393 HYPOGAMMAGLOBULINEMIA FOLLOWING RITUXIMAB THERAPY IN PAEDIATRIC RHEUMATOLOGY PATIENTS- EXPERIENCE FROM A TERTIARY CENTRE

#### Samundeeswari Deepak^1^, Kishore Warrier^1^, Rachel O'Brien^2^, Elizabeth McDermott^2^, Satyapal Rangaraj^1^

##### ^1^Paediatric Rheumatology, Nottingham Children's Hospital; ^2^Immunology, Nottingham University Hospitals NHS Trust, NOTTINGHAM, UK

###### **Correspondence:** Kishore Warrier

**Introduction:** Rituximab is a genetically engineered chimeric monoclonal antibody that depletes the B-cell population by targeting cells bearing the CD20 surface marker. Common adverse effects of rituximab therapy include infusion reactions and infections. Although hypogammaglobulinemia post Rituximab therapy has been reported, there is no clear evidence on the incidence of hypogammaglobulinemia and the trend of the serum immunoglobulin(Ig) levels over time in paediatric patients.

**Objectives:** To analyse the incidence of hypogammaglobulinemia post rituximab therapy in children treated with Rituximab in a tertiary paediatric rheumatology centre.

**Methods:** An initial audit was carried out reviewing the case notes of children (<18 years of age) who received Rituximab for a rheumatological indication from 2001 to 2016.This has been reported previously, highlighting 26% of patients with hypogammaglobulinaemia secondary to Rituximab therapy. A further review was carried out to analyse the course and trends of immunoglobulin levels till January 2018.

**Results:** With the extended review 9 out 19 patients (47%) were noted to develop secondary hypogammaglobulinemia. One patient's immunoglobulin had reverted back to normal following cessation of Rituximab therapy. Overall 14/19 patients have suffered recurrent infections, of which 8 had hypogammaglobulinaemia. During the extension phase of the study,2 patients died. Patient 1 collapsed with respiratory arrest following acute epiglottitis (enterovirus on throat swab) and subsequent cardiac arrest; patient 2 had died from ARDS following Pneumocystis jirovecii pneumonia and CMV viraemia.4/9 (44%) of the patients with hypogammaglobulinemia required immunoglobulin replacement therapy and a further 3 (33%) are under the consideration of the immunology team.

**Conclusion:** In our cohort of patients, the incidence of hypogammaglobulinemia was 47%( 9/19). This appears to have increased over time with the number of cycles patients received. Reversal of hypogammaglobulinaemia was observed only in 5% on cessation of Rituximab therapy.

The results highlight the need for close monitoring of patients while on and after Rituximab therapy for hypogammaglobulinemia and appropriate management with multidisciplinary input involving the immunologist.

The limitations for this study are the small number of patients, the effect of previous and concomitant therapy with other immunosuppressive medications including cyclophosphamide and loss of few patients to follow up.

**Disclosure of Interest**

None Declared

## Poster walk 9: JIA clinical 2

### P394 OUTCOME FOLLOWING MSUS ASSESSMENT AND GUIDED STEROID KNEE INJECTION IN JUVENILE IDIOPATHIC ARTHRITIS

#### Madeleine Rooney^1^, Abhra Chowdhury^2^, Catherine McAllister ^3^, Jenny Curry^3^, Jenny Wyatt^3^, Paul jackson^3^

##### ^1^Center Experimental Medicine, Queens University of Belfast, Belfast, UK; ^2^Rheumatology , Mission Hospital Durgapur, Durgapur, India; ^3^Paediatric Rheumatology, Belfast Hospital Trust, Belfast, UK

###### **Correspondence:** Abhra Chowdhury

**Introduction:** Intra-articular knee injection is a common procedure in paediatric rheumatology. However unlike adults, in children it is mostly carried out under general anaesthesia which has its own risks. Thus the effect of the intra-articular injection has to be optimal and long lasting. We undertook this study to find out whether ultrasound (US)assessment and guided intraarticular knee injections leads to a better outcome.

**Objectives:** 1 To evaluate the outcome following ultrasound assessed and guided intra-articular knee injection in patients with juvenile idiopathic arthritis (JIA)

2 To determine whether the base demographic, clinical, laboratory and ultrasonographic findings can predict the outcome.

**Methods:** We followed consecutive patients who fulfilled the criterion for diagnosis of JIA and required an intraarticular steroid injection to knees. We recorded demographics; disease subtype. Clinical assessments included; pain, swelling and limitation of movement. Classified as mild, moderate and marked. Patients underwent US assessment using a MyLab70. Joint effusion and synovial hypertrophy (SH) were classified as mild, moderate and marked. Standard blood tests were performed.

Under GA, US-guided IACI of the affected knee/s was performed using Triamcinolone Hexacetonide 20-40mg. Patients were followed up every 3 months for 2 years. Statistical analysis included Kaplan Meier survival analysis and ANOVA.

Study was approved by ORECNI 10/NIR01/11

**Results:** 112 patients underwent 158 ultrasound guided knee injections. Sixty four had oligo; 29 had poly; 14 had extended oligo; 4 systemic JIA and one juvenile Sp. Median disease duration 6 months (Range 1-145 months). Median age at injection 90 months (range 15-261 months). Median ESR was 13 mm Hg (range 2-117), median CRP 2.7 mg/dl (range 0.3-183 mg/dl). 50 patients had positive antinuclear antibody. 6patients received steroids; 33 patients received methotrexate; 14 patients received biologics.

Kaplan Meier survival analysis, demonstrated that overall, 64% of joints remained clinically quiet at 2 years.

There were 58 flares in 158 knees by 2 years. The number of flares according to type of JIA and at end of 3, 6, 12, 18 and 24 months is given in Table 1.

Those with minimal SH pre injection had significantly better outcome at 2 yrs. ( 72% remission) compared to those with marked SH, where just 51% were in remission.

There was no significant difference in flare rates between oligoarticular and polyarticular subtypes. Concomitant steroid or methotrexate did not alter the rates of flares. Longer duration of current episode, higher ESR and CRP and lower total steroid dose were found to predict flares.

**Conclusion:** This is the first study in paediatric rheumatology to use ultrasound guidance in all intra-articular injections. Our outcomes are significantly better than those previously published^1^ where 69% were in remission at 6 months compared to our 88%. Whilst another retrospective study^2^ observed 49% were still in remission after approximately 2 years compared to our 64%.

IACI performed under US control appears to improve the outcome in JIA and MSUS informs the clinician regarding the long term outcome

**References**

1. A Ravelli, S M Manzoni, S Viola, A Pistorio, N Ruperto and A Martini. J Rheumatol 2001;28; 9

2. Peter Marti & Luciano Molinari & Isabel B. Bolt &Reinhard Seger & Rotraud K. Saurenmann. Eur J Pediatr (2008) 167:425–430

**Disclosure of Interest**

None Declared

**Table 1 (abstract P394).** See text for description

Types	No of knees injected	Number of knees having flare				
		3 month	6 month	12 month	18 month	24 month
Oligoarticular	84	2	9	12	2	4
Extended oligoarticular	24	1	1	5	3	
Polyarticular	43	1	5	5	2	2
Systemic JIA	6				3	
Juvenile spondyloarthropathy	1			1		
Total	158	4	15	23	10	6
Percentage		2.53%	9.49%	14.55%	6.32%	3.79

### P395 DETERMINANTS OF PATIENT ‘PERCEPTION OF DISEASE ACTIVITY IN A MULTINATIONAL COHORT OF OLIGOARTYCULAR AND POLYARTICULAR JUVENILE IDIOPATHIC ARTHRITIS

#### Alessandra Alongi^1^, Chiara Trincianti^1^, Yasser El Miedany^2^, Neil Martin^3^, Maka Ioseliani^3^, Miroslav Harjacek^3^, Miroslav Harjacek^3^, Amita Aggarwal^3^, Soad Hashad^3^, Sulaiman M. Al-Mayouf^3^, Yaryna Boyko^3^, Dimitrina Mihaylova^3^, Ruben Burgos-Vargas^3^, Ekaterina Alexeeva^3^, Veronika Vargova^3^, Erkan Demirkaya^3^, Ilonka Orban^3^, Christiaan Scott^3^, Nicolino Ruperto^3^, Angelo Ravelli^3^, Alessandro Consolaro^3^, for the Paediatric Rheumatology International Trials Organisation (PRINTO)

##### ^1^Istituto Giannina Gaslini, Clinica Pediatrica e Reumatologia - PRINTO, Genova; ^2^Istituto Giannina Gaslini, Clinica Pediatrica e Reumatologia - PRINTO, Genoa; ^3^Istituto Giannina Gaslini, Clinica Pediatrica e Reumatologia - PRINTO, Genoa, Italy

###### **Correspondence:** Alessandra Alongi

**Introduction:** Patient’s perspective of disease status is a critical, yet difficult to capture component of outcome in Juvenile Idiopathic Arthritis (JIA). Some patients exhibit high perceived disease activity, pain levels and impaired health-related quality of life despite current inactive disease.

**Objectives:** 1) To examine the relation between pain, physical functioning, quality of life, damage measures and perception of activity in different disease states.

2) To identify determinants of disease activity perception in patients classified in remission.

**Methods:** Patients enrolled in The EPidemiology, treatment and Outcome of Childhood Arthritis (EPOCA) study were included in this analysis. Data from an unselected of 2102 patients with oligoarticular (n=352) and polyarticular (n=1750) were considered. Evaluated variables included the quantitative items of the Juvenile Arthritis Multidimensional Assessment Report - VAS Pain, VAS Disease Activity, VAS Well Being, Juvenile Arthritis Functional Score (JAFS), physical health (JQLPsH) and psychosocial health (JQLPhH) Quality of Life Scale - and the Juvenile Arthritis Damage Index. Interactions among patient-reported physical disability, physical and psychosocial life quality, damage, global well-being and activity perception were explored using network analysis; clustering and centrality indexes were estimated as measures of the strength of connections and relevance of specific items, then compared across patients in activity and remission states. In addition, Pearson’s correlation and recursive partitioning were used to identify determinants of patient ‘activity perception and global well-being VAS scores in the remission group (n=546).

**Results:** Network analysis revealed a highly clustered structure, with specific physical function and participation-related items connected to psychological components through pain score. Clusters appeared stable across activity and remission groups; walking and items related to cervical limitations revealed higher centrality in active patients, while impaired hand function was more central in patients in remission. In patients with active disease, pain, difficulties in energy-consuming activities, walking and impaired hand function showed the highest closeness centrality, indicating a high ability to predict other items. After adjusting for disease activity (JADAS10), pain, sadness, anxiety, difficulties in energy-consuming and social activities remained tightly interconnected, exhibiting the highest degree centrality; no substantial differences in the structure were found between polyarticular and oligoarticular patients. In the remission group, patient-perceived disease activity correlated well with well-being (r= 0.81, p<0,001) and pain (r= 0.73, p<0,001) scales. Recursive partitioning identified pain VAS > 4 (p<0.001), JQLPhH score > 6 (p=0.001), pain VAS < 1 (p<0.001) and difficulty in energy-consuming activities (p<0.001) as predictors defining 5 subgroups with different levels of VAS disease activity in patients in remission.

**Conclusion:** Network analysis revealed the connection between pain and specific psycho-functional domains and their impact on patient-perceived disease activity. These findings may inform the development of patient-centered disease assessment tools and the therapeutic targeting of symptoms that are likely to have the greatest impact on patient’s perception of treatment effectiveness and global health status.

**Disclosure of Interest**

A. Alongi: None Declared, C. Trincianti: None Declared, Y. El Miedany: None Declared, N. Martin: None Declared, M. Ioseliani: None Declared, M. Harjacek: None Declared, M. Harjacek: None Declared, A. Aggarwal: None Declared, S. Hashad: None Declared, S. M. Al-Mayouf: None Declared, Y. Boyko: None Declared, D. Mihaylova: None Declared, R. Burgos-Vargas: None Declared, E. Alexeeva: None Declared, V. Vargova: None Declared, E. Demirkaya: None Declared, I. Orban: None Declared, C. Scott: None Declared, N. Ruperto Grant / Research Support from: The G. Gaslini Hospital, which is the public Hospital where NR works as full time public employee, has received contributions from the following industries for the coordination activity of the PRINTO network: BMS, GlaxoSmithKline (GSK), Hoffman-La Roche, Novartis, Pfizer, Sanofi Aventis, Schwarz Biosciences, Abbott, Francesco Angelini S.P.A., Sobi, Merck Serono. This money has been reinvested for the research activities of the hospital in fully independent manners without any commitment with third parties, Speaker Bureau of: NR has received speaker’s bureaus and consulting fees from the following pharmaceutical companies: AbbVie, Amgen, Biogenidec, Alter, AstraZeneca, Baxalta Biosimilars, Biogenidec, Boehringer, BMS, Celgene, CrescendoBio, EMD Serono, Hoffman-La Roche, Italfarmaco, Janssen, MedImmune, Medac, Novartis, Novo Nordisk, Pfizer, Sanofi Aventis, Servier, Takeda, UCB Biosciences GmbH., A. Ravelli: None Declared, A. Consolaro: None Declared

### P396 “CHILDREN WITH ARTHRITIS AT SCHOOL” A PUBLICATION BY APMAR TO HELP CHILDREN, FAMILIES AND TEACHERS

#### Antonella Celano, Francesco La Torre and Adele Civino, Raffaella Arnesano, Annalisa Sticchi

##### Italian national Association people with rheumatic and rare diseases, lecce, Italy

###### **Correspondence:** Antonella Celano

**Introduction:** “Children with arthritis at school” is a brief text accompanied by illustration spread by APMAR Onlus to give psychological support to children that suffer from juvenile idiopathic arthritis and to their families and teachers.

**Objectives:** The main goal of the book is spotlighting necessities of children who are forced to live with juvenile idiopathic arthritis and raise awareness of these diseases in teachers and school staff in general. The aim is helping teachers and families to give a proper psychological support to children with arthritis. Furthermore, the publication tries to show how to take care of kids according to their needs.

**Methods:** The book - realized with the contribution of Adele Civino, a pediatrician at the “Cardinal Panico” Hospital of Tricase (LE), Gilda Panzera primary school teacher, the psychologist Silvia Taccone and the illustrator Salvatore Santuccio – was diffused by APMAR in schools and pediatrician’s offices.

**Results:** With the book “Children with arthritis at school” APMAR Onlus reached the purpose of focusing people's attention on rheumatic diseases. In particular, the association spread information about juvenile idiopathic arthritis (Juvenile" in this context refers to an onset before age 16 and "idiopathic" refers to a condition with no defined cause) and gave guidelines that allow teachers and families to make easier for the youngsters to attend school and make the best of it.

“Children with arthritis at school” provide information about drugs and teach how to recognize symptoms. It also promotes the importance of communication: primarily between teachers and the family, but also between the school staff and the pediatrician that attends the child. Create a dialogue is fundamental to help kids enjoying their school career, improve their quality of life and avoid them to feel different or sink into depression.

Children well-being pass through the acceptance of the pathology. To manage this achievement, they need the psychological support of their families, their classmates and their teachers. The book gives some practical advice addressed to adults.

In the book teachers will find very practical recommendations. For example, tables useful for learning how to intervene in a proper way depending on the situation and on the needs or pains showed by the kid. “Children with arthritis at school” also provide a chart that helps physical education teachers to know which activities can be easily done by the children and which not.

Together with “Children with arthritis at school” APMAR created a comic that illustrates how to deal with these diseases and how to recognize the most common symptoms. A comic allows to communicate with children in a language that they know and that is adequate for their age. The project also leads to a Youtube version of the comic called “A new challenge to live together. Rheumatic diseases explained to children”. The video is in Italian subtitled in English and is visible at this link: https://youtu.be/o12g3Zz3N-g.

Furthermore, as part of the same communication campaign, APMAR realized a video with the popular Italian singer Alessandra Amoroso (https://www.youtube.com/watch?v=8mZSirhXo8U) focused on the disease awareness.

**Conclusion:** The diffusion of the short text “Children with arthritis at school” was welcomed with approval by families and teachers. School fulfils the essential role of educate but also it allows kids to grow both at a psychological and social level. This is the reason why running an informative campaign among teachers is very important. The book raise awareness and allow many children who suffer from juvenile idiopathic arthritis to feel more comfortable at school. It also turns on the debate about these diseases and spread the knowledge of their symptoms matching the APMAR mission of sensitise public opinion towards rheumatic pathologies.

**Disclosure of Interest**

None Declared

### P397 LONG TERM OUTCOMES AND DISEASE COURSE OF CHILDREN WITH JUVENILE IDIOPATHIC ARTHRITIS ENROLLED IN THE RESEARCH IN ARTHRITIS IN CANADIAN CHILDREN EMPHASIZING OUTCOMES COHORT - A SINGLE CENTRE EXPERIENCE

#### Amieleena Chhabra, Kristin Houghton, David Cabral, Kimberly Morishita, Lori B. Tucker, Ross E. Petty, Jaime Guzman

##### Division of Rheumatology, BC Children Hospital, VANCOUVER, Canada

###### **Correspondence:** Amieleena Chhabra

**Introduction:** Long term outcomes and clinical course of patients with JIA may have improved since the pre-methotrexate era when by early adulthood many patients had persistent active disease and deformities.

**Objectives:** To assess long-term outcomes of patients with JIA recruited at one centre in the Research in Arthritis in Canadian Children Emphasizing Outcomes (ReACCh-Out) prospective inception cohort.

**Methods:** Clinical charts of patients with JIA recruited at one of the 16 centres in the ReACCh–Out cohort (diagnosed 2005-2010) were assessed at the last available follow-up visit for disease activity, clinical remission on or off medications (Wallace criteria), and articular and extra articular damage

**Results:** From the identified eligible cohort 236 subjects (97.5%) had follow-up information until discharged or transferred to adult care; 135(57.2%) were females and the median age was 17.2y (IQR 13, 18). Median follow-up period since diagnosis was 5.6 y (IQR 3.4, 8.2) with maximum follow up of 13.1 years; 64 patients (27%) had active disease at last follow up visit and 56 (24 %) were in remission on medications. Nearly half of the patients were off medications (119, 50.4%). Twenty patients (8.4%) had uveitis during their disease course while 7 (2.9%) had active uveitis at last visit. Growth related complications included micrognathia (11, 5%), and leg length discrepancy (11, 5%). Radiographic joint damage was uncommon: 40 patients (17%) had at least one erosion or joint space narrowing, 2 (1%) had avascular necrosis and 5 (2%) required joint surgery. Table 1 depicts outcomes at last visit for each JIA category. Patients with systemic JIA had the highest frequency of remission off medications (68%). Most patients with RF-positive polyarticular JIA were still on treatment (78%). Half of the patients were on at least one anti-rheumatic medication with approximately 20% on each of NSAID, DMARD or Biologic. Raised inflammatory markers were rare (4%). Changes in ILAR category were observed in 29(12.2%) patients, including 9 who changed from unclassified to a specific category by the last clinic visit and 12 oligo- persistent who became oligo-extended.

Numbers are median and IQR, unless otherwise indicated

**Conclusion:** This long-term follow-up of an inception cohort diagnosed early in the biologic era suggests a reduction in permanent damage, good prognosis for systemic JIA and a much lower progression to an oligoarthritis extended category than previously reported. Considerable disease activity persists in a quarter of the patients. Extension of this study to the entire cohort will help confirm findings and identify predictors of long-term outcome.

**Disclosure of Interest**

None Declared

**Table 1 (abstract P397).** Selected outcomes at last clinic visit for patients recruited at one ReACCh-Out center

Group	Age in years	Years since diagnosis	Active disease	Remission on meds	Remission off meds	Still on treatment	X-ray damage	Uveitis complications.
Oligoarthritis (n=93)	14.1 (11.8, 17.1)	7.8 (2.8, 10.1)	19 (20%)	26 (28%)	46 (49%)	45 (48%)	8 (9%)	2 (2%)
ERA (n=51)	17.9 (16.9, 18.1)	4.6 (3.0, 6.2)	17 (33%)	7 (14%)	27 (53%)	24 (47%)	9 (18%)	0
Poly RF-Neg (n=39)	17.7 (12.8, 18.1)	5.4 (3.8, 8.1)	16 (41%)	10 (26%)	12 (31%)	24 (62%)	6 (15%)	0
Systemic (n=22)	17.1 (15.5, 17.6)	5.8 (3.7, 8.1)	2 (9%)	5 (23%)	15 (68%)	7 (32%)	4 (18%)	0
Psoriatic (n=9)	18.1 (17.1, 18.2)	5.4 (4.3, 5.7)	2 (22%)	2 (22%)	5 (56%)	4 (44%)	5 (56%)	0
Poly-RF-Pos (n=9)	17.9 (16.2, 18.6)	6.0 (5.6, 8.1)	4 (44%)	4 (44%)	1 (11%)	7 (78%)	4 (44%)	0
Undifferentiated (n=13)	17.1 (9.7, 17.7)	6.3 (4.7, 7.7)	4 (31%)	2 (15%)	7 (54%)	6 (46%)	4 (31%)	0
All (n=236)	17.0 (13.0, 18.0)	5.6 (3.4, 8.2)	64 (27%)	56 (24%)	113 (48%)	117 (50%)	40 (17%)	2 (0.9%)

### P398 RISK OF MALIGNANCY IN CHILDREN WITH JUVENILE IDIOPATHIC ARTHRITIS: A REGISTER-BASED COHORT STUDY

#### Annacarin Horne^1^, Bénédicte Delcoigne ^2^, Bo Magnusson^1^, Karin Palmblad^1^, Johan Askling^2^

##### ^1^Pediatric rheumathology, Karolinska Hospital; ^2^Karolinska Institute, Stockholm, Sweden

###### **Correspondence:** Annacarin Horne

**Introduction:** The risk of malignancies, in particular malignant lymphomas, in Juvenile Idiopathic Arthritis (JIA) patients is low but has been reported to be elevated compared to the general population.

**Objectives:** To assess the risk of cancer in patients with JIA in comparison to non-JIA individuals both in the pediatric age range and in adulthood.

**Methods:** Register-based cohort study of patients with a first JIA diagnosis recorded from 1987 to 2016, based on data retrieved from the Swedish Patient Register (with information on hospitalizations (1987 and onwards) and outpatients visits (2001 and onwards)), and the Swedish Cancer Register. JIA patients were matched (on sex and age) to five non-JIA individuals sampled from the Swedish child population. All participants were followed up from six months after date of 2nd visit with a JIA diagnosis (for JIA patients) or the corresponding date (for non-JIA individuals) until the first date of the following events: cancer diagnosis, migration, death, 18 years of age, or 31 Dec. 2016. The occurrence of malignancies was compared through standardized incidence rates (SIR). The same analysis was rerun allowing participants to be followed up through adult ages (age at 31 Dec. 2016).

**Results:** 7988 JIA patients and 39714 non-JIA individuals were identified and followed up, with a median follow-up time until 18 years old of 4,7 years and a median follow-up time into adulthood of 9.5 years. Fourteen malignancies (among which 6 lymphomas) until the age of 18, were recorded among the JIA patients and 37 (4 lymphomas) among the non-JIA individuals. Allowing the follow-up to cover ages above 18, identified 37 malignancies (11 lymphomas) among the JIA patients and 132 (15 lymphomas) among the non-JIA individuals.

**Conclusion:** JIA patients are at increased risk to develop cancer and especially malignant lymphoma compared to the general population. There is no signal that the risk of cancer in JIA patients has been increasing over the last years. The low numbers of cancer cases in JIA patients underscores that the absolute risk is low.

**Disclosure of Interest**

A. Horne: None Declared, B. Delcoigne : None Declared, B. Magnusson: None Declared, K. Palmblad: None Declared, J. Askling Grant / Research Support from: Johan Askling has or has had research agreements with Abbvie, BMS, MSD, Pfizer, Roche, Astra-Zeneca, Eli Lilly, Samsung Bioepis, and UCB, mainly in the context of safety monitoring of biologics via ARTIS., Consultant for: Karolinska Institutet has received remuneration for JA participating in advisory boards arranged by Pfizer and Eli Lilly.

**Table 1 (abstract P398).** Risk of cancer in a cohort of patients with JIA as compared with a general population comparator group by calendar period of first identification of JIA versus all JIA, stratified by age at time of cancer diagnosis

	JIA by year of identification		
	1987–1998	1999-2016	All
Age 0–17 years			
All cancers			
Rate/100000 person-years			
JIA	86,7	26,6	32,0
Non-JIA	0	18,7	17,2
RR (95% CI)	--	1.43 (0.71-2.88)	1.86 (1.01 -3.44)
Lymphomas			
Rate/100000 person-years			
JIA	57,8	8,0	13,7
Non-JIA	0	2,1	1,9
RR (95% CI)	--	3.75 (0.84-16.76)	7.39 (2.08 -26.19)
All ages-into adulthood			
All cancers			
Rate/100000 person-years			
JIA	62,8	34,2	46,8
Non-JIA	4,8	30,2	34,2
RR (95% CI)	13.03 (1.36 -125.3)	1.13 (0.70 -1.82)	1.34 (0.93 -1.93)
Lymphomas			
Rate/100000 person- years			
JIA	41,9	9.8	13,9
Non-JIA	0	3,2	3,9
RR (95% CI)	--	3.01 (1.09- 8.28)	3.53 (1.62-7.69)

### P399 ANXIETY SYMPTOMS PREDICT POOR SLEEP QUALITY IN CHILDREN WITH JUVENILE IDIOPATHIC ARTHRITIS

#### Birgitte Mahler^1^, Johanne Jeppesen^2^, Troels Herlin^1^

##### ^1^Pediatric Rheumatology, Arhus University Hospital; ^2^Psychology and Behavioural Sciences, Aarhus University, Arhus, Denmark

###### **Correspondence:** Birgitte Mahler

**Introduction:** Poor sleep quality is common in children with juvenile idiopathic arthritis (JIA). Sleep quality is affected by the child’s activity in disease and experience of pain. Psychological variables seem to have an impact on the sleep quality as well.

**Objectives:** The aim of this study was to examine how pain, disease activity, and psychological wellbeing differs in children with JIA with poor or normal sleep quality, and the likelihood of these variables on having poor sleep quality.

**Methods:** In the outpatient clinic patients diagnosed with JIA age 6-16 were included. Child and a parent completed questionnaires regarding the child’s sleep quality in The Children’s Sleep Habits Questionnaire (CSHQ), anxiety symptoms in Spence Children’s Anxiety Scale (SCAS), wellbeing in WHO-5 Wellbeing Index (WHO-5)), positive and negative mood in The Positive and Negative Affect Schedule (PANAS). Pain intensity was measured using the VAS 1-10 scale from the Juvenile Arthritis Multidimensional Assessment Report (JAMAR). Disease activity was calculated as JADAS-27. Exclusion criteria were JIA in remission off medication, co-morbidity, non- Danish speaking patients.

**Results:** In total 62 patients were included. Results indicated that 55% of the children reported poor sleep quality (CSHQ>41). Children with poor sleep quality reported significant lower level of wellbeing and higher levels of anxiety, negative mood, and pain intensity, and showed higher level of disease activity (*p* =.02-.001). A logistic regression was performed to ascertain the effect of pain, disease activity, wellbeing, anxiety, and negative mood on the likelihood, that the children had poor sleep quality. The model was statistically significant (chi^2^ (4) = 16.50, *p=*.006) and explained 36.4% of the variance in poor sleep quality and correctly classified 75.5% of cases. Increasing level of anxiety symptoms was significantly associated with an increased likelihood of poor sleep quality (*p*=.01). None of the other predictor variables made a unique, significant contribution.

**Conclusion:** Disease activity, pain, and psychological wellbeing were negatively affected in children with poor sleep quality. Especially the level of anxiety symptoms were a significant predictor of having poor sleep quality, which highlights the importance of focusing on the impact of anxiety and worry on children’s sleep quality.

**Disclosure of Interest**

None Declared

**Table 1 (abstract P399).** Data is reported as median (minimum; maximum)

Number of patients	57
Girls/Boys	42/15
Age (years)	12.7(6.1; 16.8)
Patients on DMARDs and/or biological medication	43
sJIA, poly RF+, poly RF-, oligo, enthesitis, PsA, Undifferentiated	8%, 0, 15%, 38%, 7%, 7%, 15%
Pain intensity VAS 0-10	1.75 (0;7.7)
JADAS 27 (Range 0-57)	1.8 (0;16.3)
CSHQ (Range 33-99)	43 (36-61)

### P400 TRANSITION READINESS ASSESSMENT QUESTIONNAIRE (TRAQ): EXPERIENCE OF A PAEDIATRIC RHEUMATOLOGY UNIT

#### Patricia Martins^1,2^, Sofia C. Barreira^1,2^, Joana Silva-Dinis^1,2^, Juliana Meira^3^, Raquel Campanilho-Marques^1,2^, Filipa Oliveira-Ramos^1,2^, João E. Fonseca^1,2^

##### ^1^Unidade de Reumatologia Pediátrica, Serviço de Reumatologia e Doenças Ósseas Metabólicas, Hospital de Santa Maria, CHLN, Centro Académico de Medicina de Lisboa; ^2^Unidade de Investigação em Reumatologia, Instituto de Medicina Molecular, Faculdade de Medicina, Universidade de Lisboa, Centro Académico de Medicina de Lisboa; ^3^Escola de Psicologia e Ciências da Vida, Universidade Lusófona de Humanidades e Tecnologias, Lisboa, Portugal

###### **Correspondence:** Patricia Martins

**Introduction:** The Transition Readiness Assessment Questionnaire (TRAQ) was developed to evaluate the transition process of young people, with chronic pathology, into adult medicine. It consists of 20 items grouped in 5 subscales (Medication Management, Management of the consultations, Monitoring of Health Problems, Communication with Health Technicians and Management of Daily Activities), and the answers fit into a Likert scale of 5 points (increasing scale). The scores result from the sum of the items of each subscale, with larger values indicating a greater degree of preparation for the transition.

**Objectives:** To identify points of greater fragility in the transition to adult care in order to develop strategies for a proper planning.

**Methods:** Cross-sectional study including adolescents and young adults with chronic rheumatic conditions followed in the transition clinic of our Paediatric Rheumatology Unit, with ages between 16 and 22 years in whom the recently validated Portuguese version of TRAQ was applied. Demographic, clinical data and the responses to the TRAQ questionnaire were analysed.

**Results:** 27 participants were interviewed. Of these, 19 (70.4%) were female, with a mean age of 17.9 ± 1.2 years, 23 were in school (85.2%), with an average schooling of 11.1 years. In the population studied, 41% were able to perform their therapy without help and, in this group, about half were using non-biological or biological disease-modifying antirheumatic drugs. In the overall evaluation of each topic of the questionnaire, the one with the highest score refers to Communication with Health Technicians with an average score of 4.81, with the lowest score corresponding to Consultation Management (3,30). In this topic, the points related to Money Management and Health Insurance are the areas with a greater lack of knowledge. For the remaining topics in ascending order we found: Monitoring of health problems (3.44), Medication Management (4.12) and Daily Activities Management (4.31).

**Conclusion:** The transition to adult care is a crucial process of emerging adolescents / adults with chronic diseases. The application of this questionnaire proves to be fundamental in the preparation for the transition, namely in the orientation of the educational interventions provided by the health professionals. Therefore, the scores of each topic are useful in identifying the areas that require a higher level of intervention and thus contribute to better prepare the adolescents with chronic diseases to enter the adult system. In our Paediatric unit we conclude that the Consultation Management is the area where the results are lower and thus a greater degree of investment in this area is needed.

**Disclosure of Interest**

None Declared

### P401 “YOU CAN'T DO THIS, YOU'VE ARTHRITIS”: EXPLORING THE EXPERIENCES OF EDUCATION AND EMPLOYMENT OF YOUNG PEOPLE WITH ARTHRITIS

#### Albert Farre^1^, Sara Ryan^2^, Abigail McNiven^2^, Sue Ziebland^2^, Janet E. Mcdonagh^3^

##### ^1^Institute of Applied Health Research, University of Birmingham, Birmingham; ^2^Primary Care Health Sciences, Unviersity of Oxford, Oxford; ^3^Centre for MSK Research, University of Manchester, Manchester, UK

###### **Correspondence:** Janet E. Mcdonagh

**Introduction:** Vocational development is an integral component of adolescence with several key educational transitions occurring during this stage of life. In the context of long term health conditions and/or disability including rheumatic disease, vocational morbidities are increasingly recognised. However, only a few studies to date have used qualitative methods to report on the perspective of young people with arthritis [1,2].

**Objectives:** To understand the experiences of education and employment of educational and vocational outcomes for young people with arthritis, from the perspective of young people themselves.

**Methods:** We used secondary analysis of narrative and semi-structured interviews (n=49) which had been video or audio recorded from a primary qualitative study on the experiences and information and support needs of young people with arthritis [3]. A purposive, maximum variation sampling strategy had been employed. The dataset consisted of 40 young people. The dataset consisted of 39 young people (median age at interview 20, range 10 to 28 years; median age at diagnosis 11, range 2 months to 22 years) and 10 carers of young people who had been diagnosed with arthritis in childhood, adolescence or young adulthood. Data analysis for the primary study combined a thematic approach with the grounded theory technique of constant comparison. NVivo software was used to assist data management and coding. The secondary analysis process aligned with Heaton’s categories of supra and supplementary analysis [4]. All 49 original transcripts were re-coded with the overarching aim to identify all material relevant to vocational experiences. We also undertook a series of workshop meetings in which the re-coded data were discussed and reviewed jointly by the authorship group to add a further layer of scrutiny, and debate and refine the emerging findings.

**Results:** Three key themes were identified: (i) The impact of the unpredictability of arthritis symptoms on education and vocation; (ii) the negotiation of disclosure, understanding, support and flexibility in the workplace or educational setting; and (iii) the appraisal and reappraisal of life’s goals in the context of an uncertain prognosis.

Findings illustrated how young people with arthritis are faced with a range of challenges and disruptions in their everyday life at a time when key developmental tasks occur, including the educational and vocational aspects of their development. Appropriate support and flexibility in the workplace or educational setting were identified as enablers to successful educational and vocational outcomes. However, negotiating disclosure was not a straightforward process for such young people, with a range of concerns and expectations acting as barriers to disclosure. Furthermore, participants’ accounts revealed how disclosure is a necessary but not always sufficient step towards achieving an understanding and supportive environment in school or the workplace.

**Conclusion:** There is a need to strengthen the health-school/work interface to improve the educational and vocational outcomes for young people with arthritis. Addressing disclosure with the young person and employing effective interventions to improve communication, understanding and awareness beyond the clinical domain and across workplace/educational settings are key challenges for health professionals and important areas for further research.

[1] Hanson H, Hart RI, Thompson B et al. Disabil Rehabil. 2017 ; 13:1-8

[2]. Shaw KL, Hackett J, Southwood TR, McDonagh JE . Br J Occupational Therapy 2006;69(3): 98-105.

[3] McDonagh JE, Simmons B, Raisanen U, Zeibland S. Rheumatology (2014) 53 (suppl 3): iii18

[4] Heaton, J., 2004. Reworking qualitative data. Sage, London.

**Disclosure of Interest**

None Declared

### P402 PUBERTAL DELAY DESPITE INTENSIVE TREATMENT OF JUVENILE IDIOPATHIC ARTHRITIS: RESULTS OF A LONGITUDINAL STUDY

#### Philomine Van Pelt^1,2^, Anita Hokken-Koelega^3^, Radboud Dolhain^4^, Aike Kruize^5^, Hans Bijlsma^5^, Nico Wulffraat^2^

##### ^1^Rheumatology and Paediatric Rheumatology, Erasmus University Medical Centre , Rotterdam; ^2^Paediatric Rheumatology and Immunology, University Medical Centre , Utrecht; ^3^Paediatric Endocrinology, Erasmus Medical Centre Rotterdam; ^4^Rheumatology, Erasmus University Medical Centre, Rotterdam; ^5^Rheumatology, University Medical Centre, Utrecht, Netherlands

###### **Correspondence:** Philomine Van Pelt

**Introduction:** Delayed puberty and reduced adult height have been reported in JIA before the era of biologicals. Long-term consequences of delayed puberty are among others growth disturbances, low bone mineral mass and decreased fertility. Treatment with anti-TNF restores growth, but data on puberty are unknown

**Objectives:** We evaluated onset and course of puberty and growth, in JIA-patients who are treated intensively, including the possibility of biologicals, and identified variables related with puberty and growth

**Methods:** In a longitudinal JIA-cohort, all consecutive patients (10 -21yrs) were followed for three years. Annual examinations were performed regarding demographic and disease-related items as well as Tanner pubertal stages and anthropometric measurements. Median ages at reaching each stage were estimated by Kaplan-Meier curves. Parametric tests were used to determine differences between patients and healthy controls, non-parametric tests between patient-groups. Univariate analyses and mixed models were used to identify associated variables

**Results:** 138 patients were included (66% girls). Median disease-duration was 7,8 years (IQR 3.7-10.5), median JADAS-27 3,7 (IQR 1,3-8,0), DAS-28 2,16 (1,5-2,8). Puberty onset was 1,2yrs delayed in girls (p<0,01), in boys 0,6 yrs (ns). The progression was also delayed: end-stage (Tanner-5) in girls was 3,3yrs delayed (p<0,01), in boys 1,7 yrs (p<0,01). A positive association was found for longer disease-duration and a lower BMI. Biological-use was not associated. Both for girls and boys, standardized height was not different at the onset and at the end of puberty

**Table:** 

Tanner stage	Breast stage (A)		Genital stage (B)		Pubic hair girls (C)		Pubic hair boys (D)	
	JIA	Controls	JIA	Controls	JIA	Controls	JIA	Controls
2	11.9 (10.6-13.3)*	10.7 (9.0-12.2)	12.1 (10.5-13.7)	11.5 (..-13.0)	11.9 (10.6-14.1)*	11.0 ( 9.4-12.5)	12.5 (10.6-13.8)*	11.7 (9,2 -13.4)
3	13.4 (11.9-16.1)*	11.9 (10.5-13.1)	13.3 (11.6-15.3)	12.9 (11.3-14.5)	12.8 (11.7-15.5)*	11.9 (10.6-13.2)	13.1 (11.4-15.1)	12.9 (11.6-14.5)
4	15.0 (13.4-18.5)*	12.8 (11.5-14.5)	15.1 (13.1-16.8)*	13.9 (12.5-15.7)	14.8 (12.9-17.9)*	12.7 (11.4-14.3)	15.2 (13.4-17.1)*	13.8 (12.5-15.2)
5	17.7 (14.8-20.2)*	14.3 (12.5-19.5)	17.0 (13.9-20.7)*	15.3 (13.6-19.5)	17.2 (14.2-20.2)*	13.8 (12.1-17.7)	16.6 (13.9-20.6)*	15.0 (13.3-17.4)

**Conclusion:** In contrast to normalized longitudinal growth, we found in JIA-patients in disease remission or with low disease activity a delayed onset and progression of puberty despite intensive treatment including biologicals. Eventually, puberty was completed and normal adult height was reached. The effects on bone mass and fertility will have to be evaluated in cohort studies

**Disclosure of Interest**

None Declared

### P403 PROGRESS OF EULAR/PRES TASK FORCE TO PRODUCE STANDARDIZED PROCEDURES FOR ULTRASOUND IMAGING IN PAEDIATRIC RHEUMATOLOGY

#### Jelena Vojinovic^1^, Paz Collado^2^, Iustina Janta^3^, Loreto Carmona^4^, Clara Malattia^5^, Silvia Magni-Manzoni^6^, Gordana Susic^7^, Nikolay Tzaribacev^8^, Viviana Ravagnani^9^, Linda Rossi Semerano^10^, Damjana Kljucevsek^11^, Stefano Lanni^12^, Daniel Windschall^13^, Annamaria Iagnocco^14^, Esperanza Naredo^15^

##### ^1^University of Nis, Faculty of Medicine, Serbia, Nis, Serbia; ^2^Hospital Universitario Severo Ochoa, Madrid; ^3^Barcelona University, Barcelona; ^4^Institute for Musculoskeletal Health, Madrid, Spain; ^5^Istituto Giannina Gaslini , Genoa; ^6^IRCCS Ospedale Pediatrico Bambino Gesù, Rome, Italy; ^7^Institute for Rheumatology, Belgrade, Serbia; ^8^Pediatric Rheumatology Research Institute, Bad Bramstedt, Germany; ^9^ASST Mantova, C. Poma Hospital, Mantova, Italy; ^10^CHU de Bicêtre, Le Kremlin Bicetre, France; ^11^University Children Hospital, Ljubljana, Slovenia; ^12^IRCCS Istituto G.Gaslini, Genoa, Italy; ^13^Asklepios Hospital Weissenfels, Weissenfels, Germany; ^14^University of Turin, Turin, Italy; ^15^Fundación Jiménez Díaz, Autónoma University, Madrid, Spain

###### **Correspondence:** Jelena Vojinovic

**Introduction:** Musculoskeletal ultrasound (MSUS) is non-invasive, highly patient acceptable, easy to perform and repeatable imaging method in pediatric rheumatology. Yet, it is dependent on sensitivity of the machine used and the skills of the operator. Additional specificities are unique features of the growing skeleton, which include age-related variation of the thickness of the articular cartilage (due to incomplete ossification) and the presence of physiologically detected vascularization even in healthy children.

**Objectives:** EULAR/PReS task force objective was to develop EULAR/PReS Standardized Procedures for Ultrasound Imaging in Pediatric Rheumatology through a consensus process among rheumatologists, paediatric rheumatologists, and radiologists highly experienced in the performance, teaching and research in paediatric MSUS in rheumatologic diseases.

**Methods:** In the first phase we performed a systematic literature review (SLR) on guidelines for MSUS for children published by international societies and articles on how to perform MSUS scanning in children. Based on the SLR results, project conveners (JV and PC) formulated a Delphi survey by selecting the items to be included (i.e. musculoskeletal anatomic structures evaluable by ultrasound, scanning technique, and their lesions/abnormalities detectable by ultrasound at the principal joint areas). The Delphi survey was distributed among a broad panel of experts in MSUS (i.e. rheumatologists, paediatric rheumatologists, radiologists), selected by their high experience in the performance, teaching and research in MSUS in children. Based on the Delphi results main anatomical structures (for definitions, photo and video recordings) were selected to be used in the final phase organized as an exercise (lasting 4 days) on live healthy children models. The meeting involved: 16 project participants (13 pediatric US experts, fellow, as well as, AHP and PARE representative), 16 healthy children models (representing four different age groups) accompanied by their parents (who has signed informed consent to participate), 4 photo/imaging technicians, 2 expert US machine technicians.

**Results:** Structures from nine musculoskeletal areas (i.e. shoulder, elbow, wrist and hand, hip, knee, ankle and foot) in four age groups of children were selected. Detailed scanning procedures (i.e. patient position, probe placement, scanning method and bony/other landmarks) were used to produce the photo and video records of the scanning procedures. As a result, we obtained photo and video image library with a detailed description of the standardized procedures, which can be used as EULAR/PReS web-based educational application.

**Conclusion:** This task force has produced a consensus-based comprehensive and practical framework on standardized procedures for MSUS imaging in pediatric rheumatology.

**Disclosure of Interest**

None Declared

## Disease outcome

### P404 TRANSITION CARE OF PATIENTS WITH CHRONIC RHEUMATIC DISEASE IN A TERTIARY MEDICAL CENTER IN TURKEY

#### Amra Adrovic^1^, Esra Pehlivan^1^, Ovgu Kul^1^, Sezgin Sahin^1^, Kenan Barut^1^, Oya Koker^1^, Serdal Ugurlu^2^, Huri Ozdogan^2^, Ozgur Kasapcopur^1^

##### ^1^Department of Pediatric Rheumatology; ^2^Department of Internal Medicine, Division of Rheumatology, Istanbul University, Cerrahpasa Medical School, Istanbul, Turkey

###### **Correspondence:** Amra Adrovic

**Introduction:** Transitional care is a purposeful, planed movement of adolescence and young adults with chronic condition from childhood- to adult-oriented health care systems. Well-organized, systematic transitional health care is of high importance for providing the continuous medical treatment and for reaching optimal outcomes. Up to date, there is no unique, consensus-based model for patients’ transition from childhood- to adult oriented health care centers in Turkey.

**Objectives:** We aimed to assess the transitional care among patients with juvenile onset rheumatologic disease in referral tertiary center in Turkey.

**Methods:** The transitional policlinic is held once per month at the department for pediatric rheumatology, consisting of 2 adult rheumatologist, 4 pediatric rheumatologist and 1 adult rheumatology nurse. Patients who have undergone the transitional care in the time period from May 2014 to December 2017 were called by telephone, by two different investigators (EP,OK). Thirty-three (22%) patients were not reached and 17 (11%) of theme were excluded from the study due to short post-transitional period (<6 months). Consequently, 97 (66%) patients have been reached and included in the study. Data on demographic, clinical and socio-economic features and experience with transitional practice were collected by using a structured questionnaire, which was fulfilled during the phone conversation between investigator and patient.

**Results:** A total of 147 patients (79 (54%) females) underwent transition process and 97 of them were included in the study (Table 1). There was no statistically significant difference between different patients groups regarding the age of transition. The education levels of patients were as following: university 60 (61.9%), high-school 21 (21.6%), middle-school 13 (13.4%), primary school 3 (3.1%). Majority of patients was single at the time of study (79 (81.4%) patients) while only 18 (18.6%) patients were married; half of them being child owner. At the time of study, 44 (30%) patients were employed and mean age at employment was 19.06±3.1 years. Most of patients had health insurance at the time of study (94 (96.9%)). Seventy-one (73.2%) patients continued their regular follow up at adult department while 26 (25.8%) patients discontinued medical treatment. The most common reasons for cessation of follow up were the work/school absence (20 (76.9%)), followed by patients’ personal reasons (2(7.6%)) and dissatisfaction with adult clinic services (4(15.5%)). Most of patients reported satisfaction with transition process: 96 (99%).

**Conclusion:** Well-organized transitional health care is of crucial importance for the continuous and optimal health care of adolescents and young adults with chronic rheumatic disease. Further studies with higher number of patients would reveal the relevance of described transitional care model.

**Disclosure of Interest**

None Declared

**Table 1 (abstract P404).** See text for description

	Patients that were included in the study N=97
Female, n (%)	58 (59.7%)
Age at transition, mean±SD	21.4±1.4 years
Age at study mean±SD	22.8±1.8 years
JIA, n(%)	26 (26.8%)
Oligoarticular JIA	2 (2%)
Polyarticular JIA	10 (10%)
Systemic JIA	2 (2%)
JSPA	11 (11%)
Psoriatic arthritis	1 (1%)
FMF	60 (61.8%)
Connective Tissue Disease, n(%)	7 (7%)
jSLE	5 (5%)
JDM	1 (1%)
Mixed cryoglobulinemia	1 (1%)
Vasculitis, n(%)	4 (4%)
Takayasu arteritis	1 (1%)
Behçet’s disease	3 (3%)

### P405 A SYSTEMATIC REVIEW OF QUALITY OF PROGNOSIS STUDIES IN CHILDHOOD-ONSET SYSTEMIC AUTOIMMUNE RHEUMATIC DISEASES

#### Kaien Gu^1^, Annaliese Tisseverasinghe^2^, Carol Cooke^1^, Lily S. H. Lim^3^

##### ^1^University of Manitoba, Winnipeg, Canada; ^2^Department of Medicine, University of Manitoba; ^3^Children's Hospital Research Institute of Manitoba, Winnipeg, Canada

###### **Correspondence:** Kaien Gu

**Introduction:** Many individuals, including adults, live with childhood-onset (onset <18 years) systemic autoimmune rheumatic diseases (ChildSARDs), which comprise of childhood-onset systemic lupus erythematosus (SLE), Sjogren’s syndrome, idiopathic inflammatory myositis (IIM), systemic sclerosis, and vasculitides. With increasing survival of these patients in the past few decades, many now live into adulthood with their disease. However, little is known about ChildSARD outcomes in adulthood. Adult rheumatologists therefore have inadequate evidence to guide their management of these patients. Patients and their families also have little information about their possible outcomes in adulthood.

**Objectives:** To systematically review prognosis studies performed on adults with childhood-onset systemic autoimmune rheumatic diseases (ChildSARD), examine for quality of studies (for risk-of-bias) and the scope of outcomes studied, so as to identify gaps of knowledge and methodological issues. To recommend future directions of research in adulthood outcomes of ChildSARD and advice on common pitfalls in study designs to avoid.

**Methods:** We developed a search strategy with an academic librarian to identify all full-length English articles in MEDLINE and Embase (January 1990 – May 2017) reporting on any adulthood outcomes of ChildSARDs. Our strategy was iteratively fine-tuned and finalized after peer-review by librarians. Hand-searching supplemented the literature search. We excluded case reports, case series, letters, editorials, and short reports. If a study included both pediatric and adult subjects, we assessed the study only if the median (or mean, assuming no skewed distribution) age at outcome ascertainment was ≥ 18 years. Information about outcomes, prognostic factors, and study designs was recorded. All studies were graded independently by two reviewers (after prior training for adequate agreement) using the Quality in Prognosis Studies risk-of-bias tool.

**Results:** Of 14,100 articles, 26 publications met our inclusion criteria. Two studies (7%) were longitudinal (repeat measures) while the remainder assessed outcomes on a single occasion. Only 10 (36%) studies focused exclusively on adults. Studies by publication times were: 4% (1990-1999), 18% (2000-2009), and 79% (2010-2017). Most studies (82%) were conducted in either North America or Europe. The most commonly studied diseases were SLE (in 61% of studies), IIM (18%), and systemic sclerosis (11%). The most commonly reported primary outcomes were organ damage (29%), cardiovascular outcomes (14%), and mortality (14%). The mean ages at outcome assessment were 19.5 – 46.8 years for adult-only studies and 19.3 – 35 years for mixed studies. Moderate to high risk-of-bias was found in 100% of studies for study participation, 90% for study attrition, 61% for prognostic factor measurement, 36% for outcome measurement, 89% for confounding, and 54% for statistical analysis.

**Conclusion:** There is clear need for more information about adulthood outcomes of ChildSARDs. Longitudinal data collection and analyses were especially lacking. We recommend that future studies on ChildSARD outcomes be undertaken in the framework of a longitudinal cohort, have adulthood outcomes separately reported from outcomes in childhood, and have study populations clearly defined to allow for accurate MESH coding so as to facilitate easy searching for such information and for knowledge dissemination. Careful attention should be paid especially during study design to reduce bias in choice of study populations, accounting for attrition and confounding.

**Disclosure of Interest**

None Declared

### P406 A SYSTEMATIC REVIEW OF EMPLOYMENT OUTCOMES OF ADULTS WITH CHILDHOOD-ONSET SYSTEMIC AUTOIMMUNE RHEUMATIC DISEASES

#### Kaien Gu^1^, Tania Gottschalk^1^, Lily S. H. Lim^2^

##### ^1^University of Manitoba; ^2^Children's Hospital Research Institute of Manitoba, Winnipeg, Canada

###### **Correspondence:** Kaien Gu

**Introduction:** Many individuals, including adults, live with childhood-onset systemic autoimmune rheumatic diseases (ChildSARDs), with improved survival in recent decades. Employment is a major milestone of independence in adulthood and has obvious impacts on socioeconomic attainment and sense of achievement. Employment is often associated with access to health insurance and healthcare. Therefore, employment is an important outcome for adults with ChildSARDs as they enter into adulthood.

**Objectives:** To systematically review current literature on employment outcomes of adults diagnosed with ChildSARDs.

**Methods:** Systemic autoimmune rheumatic diseases (SARDs) in our study include: systemic lupus erythematosus (SLE), inflammatory myositis, Sjogren’s syndrome, systemic sclerosis, and systemic vasculitides. We developed a search strategy with an academic librarian and iteratively refined it by peer-review before finalization. MEDLINE, Embase, and Scopus were searched for full-length English articles between January 1990 and November 2017. References of review articles were hand searched. Articles identified were screened by 1 reviewer to identify eligible studies; eligibility was confirmed by review of the full-length articles. If a study contained adults with ChildSARDs and adult-onset SARD patients, we contacted the corresponding authors for clarification on the number of ChildSARD patients and requested the employment outcomes of those individuals. We extracted information on study design, employment outcome measures, prognostic factors, and confounders using a standard data collection form. Eligible studies were graded independently by 2 reviewers, after training to ensure acceptable agreement, using the Quality in Prognosis Studies (QUIPS) risk-of-bias tool.

**Results:** Five publications arising from 4 studies from 2109 abstracts. Four publications studied SLE and 1 studied juvenile dermatomyositis (JDM). In total, there were 296 patients (257 SLE, 39 JDM). Study participants had a mean age of 23.3-29 years with a mean disease duration of 7.6-15 years. Of the 5 studies, only 1 was longitudinal (at least 3 repeated outcome measurements). 80% (4/5) of studies were from North America. No study made comparisons between the study population and corresponding age/sex-matched general population. Moderate to high risks-of-bias were commonly found especially in study population (100%), prognostic factor measurement (80%), and confounding (80%). 47% of the 296 total patients were working (full/ part-time), 21% were studying, the remaining 32% were not working (for various reasons, variably reported in different studies). Reasons for patients being in different work categories were not reported, and only 1 study made comparisons with the national average employment rate. Only 2 studies assessed work productivity, and one measured work characteristics. Income was reported in two studies. Multiple prognostic factors for employment were identified in 3 studies; generally, these were either disease characteristics or workplace support and satisfaction.

**Conclusion:** There is scarce information on employment outcomes of adults with ChildSARDs. Other than SLE and JDM, no other SARDs were represented. There was little information on work characteristics; studies tended to focus on either sociodemographic or disease aspects but not both. Important study domains, such as population and confounding, are at moderate-high risk-of-bias. Future studies should aim to study other ChildSARD diseases, with careful attention to study design, and investigators should collect more comprehensive information on employment beyond work status, compare patient outcomes to the general population, and test both sociodemographic and disease related factors.

**Disclosure of Interest**

None Declared

### P407 ESTIMATION OF THE CARDIOVASCULAR SYSTEM STATUS IN PATIENTS WITH RHEUMATIC DISEASES OF CHILDREN

#### Olena A. Oshlyanska^1^, Illya A. Chaikovsky^2^, Agar G. Artsymovych^3^

##### ^1^1.Department of Pediatrics № 1, 2. Department of connective tissue disorders in children, 1.Shupyk National Medical Academy of postgraduate education, 2.State Institute of Pediatrics, Obstetrics and Gynecology, Academy of Medical Sciences of Ukraine; ^2^V.M. Glushkov Institute of Cybernetics of NAS of Ukraine; ^3^Department of connective tissue disorders in children, State Institute of Pediatrics, Obstetrics and Gynecology, Academy of Medical Sciences of Ukraine, Kyiv, Ukraine

###### **Correspondence:** Olena A. Oshlyanska

**Introduction:** Among the causes of death in patients with rheumatic diseases (RD) 38.1% are disorders of the cardiovascular system, which can be caused by immune mechanisms and are often not detected.

**Objectives:** To investigate the possibility of revealing electrophysiological changes in myocardium of children with RD using a new software-hardware complex (SHC) with a 4-level estimate "Cardio plus P".

**Methods:** With the help of the SHC 27 patients with RD during remission period, with no clinical, instrumental and laboratory signs of heart disease were examined. 9 boys, 18 girls were examined, 10.1±3.6 y old, duration of the disease 1.45±0.51 y. 54% of them received low-dose GC. Their results were compared with healthy ones.

SHC registers a 12-channel ECG with a multi-level analysis of the signal-averaged complex. It studies alternate parameters (standard deviation, level of the overall adaptive potential, rapid rate of heart rate, sympathetic and parasympathetic regulation, generalized signs of heart failure, symmetry of the T wave), it takes into account the variability of complexes, allows control of regulation and functional state of the myocardium (by the form of ST, amplitude and waves square indexes, angles of deviations), detects a violation of the rhythm, indirectly assesses the psycho-emotional state. The monitor demonstrates the graphical form of the 4-level estimate of ECG analysis and the integral index of the functional state of the cardiovascular system.

**Results:** Analysis of the data showed that only in 14.2% of cases for HSC there were no violations of the heart rate. The comprehensive assessment of regulation showed a significant decrease in patients with RD (65.82±1.97 at a rate of 76-100), mostly due to vegetative imbalance. The triangular index grew to 25525.2±27.6 (norm 9-100), the voltage index up to 164.59±33.46 (0-120). Operational control of the myocardium was also decreased to 55.5±2.41 (75-100). The decrease in the amplitude of the wave T in the leads I and AVL and its increase in the leads of the III AVF with its asymmetry indicated the presence of cardiometabolic changes. The integral indices of the ST-T form were changed in all leads, the highest of I 60.36±3.74 (75-100), the ratio of the amplitudes of the waves T and R increased 0.83±0.21 (0.14-0.33) The ratio of ECG phases in patients with RD was 27.82±5.43 (75-100). The absence of a bias of the ST segment after 0.08 s after the point J reflected the absence of hypoxia. The index of myocardial reserve in RD decreased to 63.77±2.59 (75-100). The patients noted a general insignificant psycho-emotional stress. The composite index of myocardium state was 61.13±2.18%, and only 11.1% of patients had normal indexes. Dependence on anemia (r=0.35), thyroid gland damage (r=0.72), ESR (r=0.45), LDH activity (r=0.86), phosphorus content (r=0,99), titers of antiphosphatidyl ethanolamine (r=0.71) and dsDNA antibodies (r=0.33) were detected. It did not depend on the previous carditis and the presence of foci of infection, dysproteinemia, CRP.

**Conclusion:** The use of HSC allows to detect changes in the cardiovascular system in children with RD and latent heart rhythm disturbances three times more often. Electrocardiographic changes are observed in 81% of patients with RD. They are mainly due to vegetative and metabolic disorders.

**Disclosure of Interest**

None Declared

### P408 PULMONARY FUNCTION ANALYSIS IN CHILDREN WITH RHEUMATIC DISEASES

#### Nataly S. Shevchenko^1,2^, Mariana A. Bugaevska^1^

##### ^1^Department of pediatrics № 2, V.N. Karazin National University; ^2^Department of Cardiorheumatology, Institute for the Health Care of Children and Adolescents of the National Academy of Medical Sciences of Ukraine, Kharkiv, Ukraine

###### **Correspondence:** Nataly S. Shevchenko

**Introduction:** The lesions of the respiratory system, namely the pulmonary tissue, are not specific for the development of poly- and oligoarticular juvenile arthritis. However, sclerotic damage to the mesenchymal tissue is one of the complications of long-term therapy with methotrexate and one of the factors determining the long-term prognosis of these patients of adult age. Rheumatic diseases are characterized by frequent involvement of respiratory organs. In children, pulmonary function disorders often occur latently, with minimal clinical manifestations, which is due to the high compensatory abilities of the child's organism.

**Objectives:** For the timely diagnosis and prevention of pulmonary complications the ventilatory lung function in children with rheumatic diseases was assessed.

**Methods:** Spirometry was performed in 129 children from 5 to 18 years with rheumatic diseases, among them 35 boys and 94 girls. In the structure of diagnoses were patients with JIA - 89 people (69%), with SLE - 18 people (13.95%), with scleroderma 12.40% (16 people), dermatomyositis - 4 people (3.1%), nodular polyarteritis - 2 people (1.55%).

**Results:** 29 children showed a violation of the ventilation function of the lungs (which amounted to 22.8% of the total number of patients). In all cases, the violations were as a restrictive type. Among them lung lesions were noted more often in patients with SLE (up to 44%), in patients with JIA to 21%, with systemic scleroderma - 12%. Among children with dermatomyositis and nodular polyarteritis, there were no abnormalities, which is probably due to the small number in the structure of the subjects. An analysis based on gender were carried out , it showed that girls made up 84% with JIA, and with SLE - 75%, with systemic scleroderma, violations were detected only in girls. In most patients, external respiratory function impairments were mild or moderate severity, and severe respiratory ventilation dysfunction was detected only in patients with JIA. A correlation analysis was made of the relationship between the spirogram score and individual patient parameters, such as gender, age, physical development by BMI. There were not reveal a reliable relationship between these parameters. An additional analysis of the correlation was made with the duration of the disease in patients with JIA. As a result, a weak positive relationship was found with the duration of the disease (r = 0.36, p <0.05).

**Conclusion:** In children with juvenile idiopathic arthritis before the age of 18 we found signs of a violation of the respiratory function of a restrictive type, which is indicate of a lesion of the pulmonary parenchyma. The revealed changes depend on the duration of the disease and do not depend on the clinical parameters of the patients.

**Trial registration identifying number:**

**Disclosure of Interest**

None Declared

## Miscellaneous rheumatic diseases

### P409 CHARACTERIZATION OF A GROUP OF 5 PATIENTS WITH IGG4-RELATED DISEASE: SYMPTOMS AND TREATMENT

#### Anna Kozlova, Vasiliy Burlakov , Dmitriy Abramov, Aleksandra Laberko, Garik Sagoyan, Anna Shcherbina

##### Immunology, Federal State Budgetary Institution "National Medical Research Center of Pediatric Hematology, Oncology and Immunology named after Dmitry Rogachev" of the Ministry of Healthcare of the Russian Federation, Moscow, Russian Federation

###### **Correspondence:** Anna Kozlova

**Introduction:** Immunoglobulin G4-related disease (IgG4-RD) is a chronic systemic inflammatory condition with an unclear pathophysiology and IgG4-positive plasma cells infiltration of various organs and parts of the body.

**Objectives:** If untreated, the disease can lead to fibrosis and irreversible organ damage. IgG4-RD mostly has been described in adults, hence it is generally unknown among pediatricians.

**Methods:** We conducted a retrospective analysis of clinical features and response to therapy of five patients (one female, four males, median age 13,6 years) with IgG4-related disease, treated in our Center. The diagnosis was confirmed by detection of lymphoplasmacytoid infilatration with >30% of cells expressing IgG4 in all, and elevated IgG4 serum concentration in 4 cases.

**Results:** Three patients had localized lesions (orbit, hip muscle, perirancreatic tissue, respectively), two – multiorgan disease with polylymphadenopathy, pulmonary, renal and hepatic foci, dacryoadenitis with edema of the eyelids. Autoimmune thrombocytopenia (70 х10^9^/l), neutropenia (0,79 х10^9^/l) were present in one patient. Rituximab therapy was successful in 2 cases (one patient received monotherapy with rituximab, another one – Rituximab and Sirolimus). Two other patients received JAK inhibitor therapy (ruxolitinib) with good effect. No side effects were noted. One patient underwent surgery - the infiltration in the abdominal cavity was removed with positive effect without specific therapy.

**Conclusion:** IgG4-RD symptoms can be diverse and sometimes atypical, so dealing with this pathology requires physician’s awareness. Rituximab was effective in patients with multi-organ manifestations, and JAK inhibitor (Ruxolitinib) was effective in patients with mono-focal disease. Steroids are routinely used in IgG4-RD as a first line of treatment with significant side effects. We propose that alternative drugs could be used in IgG4-RD,  especially in pediatric patients to achieve fast remission with significan morbidity

**Disclosure of Interest**

None Declared

### P410 ONCOHEMATOLOGICAL DISEASES MIMICKING RHEUMATOLOGICAL DISORDERS: REPORT OF TWO CASES

#### Teresa Lastella, Francesca Orlando, Roberta Naddei, Marina Amico, Carolina Porfito, Maria Alessio

##### Mother and Child Department, University of Naples Federico II, Napoli, Italy

###### **Correspondence:** Teresa Lastella

**Introduction:** The approach to the rheumatological patient often takes to the individuation of pathognomonic clinical and laboratory signs. Nevertheless, their presence should not exempt the physician from the search of differential diagnostic elements, mostly in oncohematological purview.

**Objectives:** To describe the workup of two cases misdiagnosed as rheumatic disease.

**Methods:** Report of two clinical cases referred to the pediatric rheumatology unit of the University of Naples Federico II.

**Results:** The first patient is a 15-year old girl suffering from pale skin and fatigue from 3 months. At the admission to the local hospital, the laboratory investigations revealed microcytic anemia (Hb 7.5 g/dl), raised ESR (120 mm) and CRP (143.6 mg/L); no thrombocytopenia and no leukopenia were found. Infectious diseases were excluded as well as inflammatory bowel disease. Autoimmune panel was performed and a slight increase of ANA and Lupus Anticoagulant was found. A Systemic Lupus Erythematosus (SLE) onset was suspected. The girl was referred to our center and laboratory examinations were performed: slight increase of Anti Histone Antibodies, positive Direct Coombs Test, weakly increase of ANA, non-hemolytic anemia and hypergammaglobulinemia were found. During the clinical examination, no skin involvement was noticed, but a supraclavicular lymphadenopathy and hepatosplenomegaly were evidenced. Clinical and immunologic criteria were not sufficient for SLE diagnosis. An ultrasound of lymph node was performed and showed an inhomogeneous and hypoechoic structure, richly vascularized. Total body PET was performed, showing hyperaccumulation of lymph node sites above the diaphragm. Lymph node biopsy led to the diagnosis of Hodgkin’s Lymphoma (nodular sclerosis subtype). Chemotherapy and radiotherapy were performed and 3 months later, the total body PET was completely negative.

The second case report is about a previously well 8-year-old boy, with 1-month history of multiple subcutaneous plaques and nodules, involving the extremities, trunk and face, onset after a trauma. Panniculitis was diagnosed based on skin biopsy and the child was treated with low doses of steroids. After 6 months, he was admitted to our Unit with high fever, malaise, myalgia and extended cutaneous lesions. Clinical examination revealed liver and spleen enlargement; laboratory data showed anemia, leukopenia and elevated levels of ERS, CRP, AST, ALT, LDH, ferritin and low level of albumin. He was negative to screening of EBV and tuberculosis infection. By the review of the first skin biopsy, Subcutaneous Panniculitis T-cell lymphoma (SPTCL), an unusual type of peripheral T-cell lymphoma, was suspected. Another surgical skin biopsy was performed. The histopathological picture showed a pleomorphic cellular infiltrate and cell surface markers consistent with the diagnosis of SPTLC. Liver biopsy revealed systemic hemophagocitic syndrome, while bone marrow examination and bone biopsy were normal; after a week the boy experienced disseminated intravascular coagulation. He was admitted to the Pediatric Hematology and Oncology Unit and initially treated with chemotherapy and local radiotherapy, then autologous bone marrow transplantation was performed and now he is completely recovered.

**Conclusion:** The isolated finding of positive autoimmune laboratory data, without suggestive clinical features, should not immediately lead to the diagnosis of rheumatologic disorders, but requires more investigations to exclude oncohematological disease. Likewise, suggestive clinical signs should in any case, lead to differential diagnosis, if the clinical course is atypical or if the response to therapy is inadequate.

Informed consents for the publication were obteined from the parents.

**Disclosure of Interest**

None Declared

### P411 LEARNED LESSONS FROM GRIP REGISTRY OF POLYAUTOIMMUNITY IN COLOMBIAN JUVENILE PATIENTS

#### Clara Malagon^1^, Maria D. P. Gomez^2^, on behalf of GRIP Investigation Group, Angela C. Mosquera^3^, Camilo Vargas^4^, on behalf of GRIP Investigation Group, Tatiana Gonzalez^5^, on behalf of GRIP Investigation Group, Christine Arango^6^, on behalf of GRIP Investigation Group, Pilar Perez^1^, on behalf of GRIP Investigation Group, Lorena Martin^7^, on behalf of GRIP investigation group

##### ^1^Pediatric Rheumatology, Universidad El Bosque, Bogota; ^2^Pediatric Rheumatology, Posgraduate of pediatrics, Universidad Libre, Cali; ^3^Pediatric Rheumatology., Universidad El Bosque, Bogota; ^4^Pediatric Rheumatology. Posgraduate of pediatrics, Universidad Del Valle, Cali; ^5^Pediatric Rheumatology. Posgraduate of pediatrics, Universidad de Cartagena, Cartagena; ^6^Pediatric Rheumatology. Posgraduate of Pediatrics, Universidad Tecnológica de Pereira, Pereira; ^7^Pediatric Rheumatology, Hospital Universitario San Ignacio, Bogotá, Colombia

###### **Correspondence:** Clara Malagon

**Introduction:** The association of two or more autoimmune diseases (AIDs) in a single patient may be explained due the fact that those diseases share pathogenic mechanisms, many clinical manifestations are common and the profile of autoantibodies is unspecific for several AIDs and those associations may cause significant morbidity . There are limited information about polyautoimmunity on juvenile patients. Three years ago was started a registry of patients with polyautoimmunity documented during the follow up at a pediatric rheumatology clinics ( 17 centers on 5 Colombian cities). A preliminary report was presented a PRES meeting 2016. We continue this registry, including new patients and updating the information.

**Objectives:** Identify the clusters of polyautoimmunity observed on those patients and describe the clinical, laboratory, histopathological features and treatment in juvenile patients with polyautoimmunity (2 AIDs) and multiple autoimmune syndrome (≥3 AIDS) and analyze the clusters of AIDs observed.

**Methods:** Multi-center, retrospective and prospective study. Medical records were reviewed and the information was recollected using a data collection format. Statistical analysis performed using SPSS 20.

**Results:** N = 313 Sex ratio F22: M1, mean age was 11,3 years (1-16). The first AIDs documented was named as “chaperone” disease followed by the additional AIDs at a variable of time needed to confirm the diagnosis and classified as simultaneous when the interval was less than 6 months or sequential if that interval was longer. Those intervals varied widely between associations. 28 AIDs were identified. The shorter interval was for AIDs associated to SLE and the longer was for Sjogren Syndrome ( confirmed by minor salivary gland biopsy). Some were re classified because developed additional AIDs since their entrance to the registry to the last cut point. 40 patient developed multiple autoimmune syndrome

Thyroid was the most common organ-specific AID and SLE was the most frequent systemic AID with polyautoimmunity. SLE preceded, diagnosed simultaneously or followed by other 7 AIDs correspond to the biggest cluster followed by Hashimoto, JIA and Sjogren in association to another AIDs. Other 16 types of associations were documented.

**Conclusion:** What we have learn??

First: Polyautoimmunity is not an uncommon event on juvenile patients and patients with a AID are at risk of additional AIDs as it has been confirmed on adult patients but the type of associations may vary between populations (ethnicity).

Second: Some associations must be searched at the time of diagnosis and during the follow up because may determine severe complications and immunomodulation, hormone supplementation and additional prophylatic and therapeutic measures may benefit the prognosis of the patients.

Third: If an atypical clinical course or changes of the symptoms and clinical signs on a patient with AIDs are observed, to check the functional tests of the potential target organs and autoantibody profile is indicated in order to rule out polyautoimmunity.

**Disclosure of Interest**

None Declared

### P412 FOUR CASE REPORTS ABOUT BENIGN AND MALIGNANT TUMORS WITH OSTEOARTICULAR SYMPTOMATOLOGY AT ONSET: ACUTE LYMPHOBLASTIC LEUKEMIA, OSTEOID OSTEOMAS, EWING SARCOMA, PIGMENTED VILLONODULAR SYNOVITIS

#### Angela Mauro^1^, Rita Sottile^2^, Antonio Mellos^3^

##### ^1^Rheumatology Unit, Department of Pediatrics, "Santa Maria della Pietà" Hospital, Nola, Naples, Italy, Nola; ^2^Rheumatology Unit, Department of Paediatrics, Santobono-Pausilipon Children’s Hospital, Naples; ^3^Rheumatology Unit, Department of Paediatrics, Santa Maria della Pietà Hospital, Nola, Italy

###### **Correspondence:** Angela Mauro

**Introduction:** Osteoarticular pain can not only be the sign of arthritis and musculoskeletal syndromes, but also of benign or malignant tumors.

**Objectives:** We describe four paradigmatic cases of acute lymphoblastic leukemia (ALL), osteoid osteomas (OO), Ewing Sarcoma (ES), pigmented villonodular synovitis (PVS).

**Methods:** Diagnostic hypotheses were based on the medical history, physical examination, blood and imaging tests.

**Results:** Case report 1**.** Since about a month, a six-year-old male child complained of nocturnal pain in the lower limbs with awakening, intermittent fever, asthenia, loss of appetite, headache, weight loss and reported upper airway infection in the last two months. Physical examination showed cutaneous pallor, apathy but absence of hepatosplenomegaly, lymphadenopathy, petechiae, ecchymoses, scrotal swelling and signs of arthritis. Ocular fundus examination was normal. Blood tests showed mild anemia, neutrophilic leukocytosis, thrombocytopenia, increased erythrocyte sedimentation rate *(*ESR) and C-reactive protein (CRP), increased lactic dehydrogenase and urate. Search for homovanillic and vanillylmandelic acid in the urine was negative. Microscopic observation of peripheral blood highlighted the presence of blasts. Bone marrow needle aspiration and biopsy confirmed the suspicion of ALL.

Case report 2. Since about two months, a 14-year-old teenage boy reported pain in the right lower limb, more intense at night, not associated with fever and increased in intensity despite oral administration of ibuprofen. Clinical examination showed lameness and functional limitation of the right coxo-femoral joint. Blood tests were all normal, while radiograph at the proximal femur showed an oval radiolucent lesion of about one centimeter in diameter, surrounded by a sclerotic ring. Computerized tomography (CT) was performed and scintigraphy showed an increased uptake with technetium 99m. Diagnostic suspicion of OO was confirmed following the removal of the lesion.

Case report 3. A 15-year-old teenage boy since about a month complained of pain referred to the anterior surface of the left thigh, associated with fever and which resulted in nighttime awakening. At physical examination the patient was limping; laboratory tests showed increased ESR and CRP. Radiological examinations revealed an elongated lytic bone lesion, disrupting the cortex and surrounded by onion-skin periosteal reaction. Bone biopsy confirmed the diagnosis of ES.

Case report 4. A 16-year-old teenage girl came to our observation for joint pain in her right knee since more than six months. Physical examination showed swelling, pain and functional limitation of the knee joint. Blood tests turned out to be normal, ultrasonography showed signs of synovial hypertrophy and intra-articular effusion; joint aspiration highlighted dark-brown synovial fluid. Magnetic resonance imaging (MRI) revealed a hypertrophic synovial membrane with a villonodular appearance. Diagnosis of PVS was confirmed following surgical excision.

**Conclusion:** In the clinical suspicion of an osteoarticular tumor, blood tests do not provide specific diagnostic indications and radiography can show if the lesion is osteolytic or osteogenic. CT and MRI evaluate the extent of the lesion, while scintigraphy may be useful in the location of the tumor. Definitive diagnosis of a benign or malignant tumor rests on the histological evaluation of an open or fine-needle biopsy. Informed consent to publish had been obtained.

**Reference**

Jackson TM, Bittman M, Granowetter L. Pediatric Malignant Bone Tumors: A Review and Update on Current Challenges, and Emerging Drug Targets. Curr Probl Pediatr Adolesc Health Care. 2016; 46(7):213-228.

**Disclosure of Interest**

None Declared

### P413 OSTEOARTICULAR MANIFESTATIONS OF HAEMATOLOGICAL DISEASES: THREE CASE REPORTS ON SICKLE CELL ANEMIA, HEMOPHILIA A AND BETA THALASSEMIA MINOR

#### Angela Mauro^1^, Rita Sottile^2^, Antonio Mellos^3^

##### ^1^Rheumatology Unit, Department of Pediatrics, "Santa Maria della Pietà" Hospital, Nola, Naples, Italy, Nola; ^2^Rheumatology Unit, Department of Paediatrics, Santobono-Pausilipon Children’s Hospital, Naples; ^3^Rheumatology Unit, Department of Paediatrics, Santa Maria della Pietà Hospital, Nola, Italy

###### **Correspondence:** Angela Mauro

**Introduction:** Osteoarticular manifestations may be the first sign of non malignant hematological diseases, such as hemophilia and hemoglobinopathies.

**Objectives:** We describe in sequence three paradigmatic case reports on sickle cell anemia, beta thalassemia minor and hemophilia A manifested at onset with osteoarticular symptomatology.

**Methods:** Diagnosis were formulated in consideration of reported clinical history, signs highlighted at the physical examination and results of laboratory tests.

**Results:** Case report 1. Two year old black male child, whose parents were both african, presented  inconsolable afebrile crying, macroscopic haematuria and acute swelling of the fingers of both hands and feets. Physical examination showed dactylitis of hands and feet, scleral jaundice, splenomegaly and absence of focal neurological signs. Blood tests showed moderate anemia, hyperbilirubinemia, reticulocytosis and increased lactic dehydrogenase, while chest radiography and abdomen ultrasound were normal. Microscopic observation of the peripheral blood smear showed sickle-shaped red cells; the suspected diagnosis of sickle cell anemia was confirmed by high-performance liquid chromatography, sickle cell test and search for genetic mutation.

Case report 2. Seven year old girl came for widespread joint pains without fever since several months. Physical examination revealed mild scleral jaundice, hepatosplenomegaly and pain in the mobilization of hips, ankles, wrists, knees and elbows without signs of joint limitation. Blood tests showed mild hyperbilirubinemia and moderate microcytic and hypochromic anemia with sideremia, transferrin, ferritin and inflammatory indices in the norm. Radiological examinations were normal, while the hemoglobin electrophoresis showed a percentage increase in hemoglobin A2, confirming in association with DNA analysis the suspicion of beta thalassemia minor.

Case report 3. Sixteen months old male child, came to our observation for the swelling of both ankles and knees in the absence of fever. Physical examination revealed several hematomas on the skin surface and considerable swelling of ankles and knees with reduced joint mobility. Blood tests showed a lengthening of the activated partial thromboplastin time (APTT). In the suspicion of hemophilia A, the factor VIII dosage was performed, which was reduced thus confirming the diagnostic hypothesis.

**Conclusion:** Sickle cell anemia occurs almost exclusively in black children and the joint symptomatology of hands and feet is usually symmetrical. Thalassemias are a heterogeneous group of syndromes in which bone pain is often reported. Non-steroidal anti-inflammatory drugs (NSAIDs) should be avoided in patients with haemophilia; joint aspiration has a limited therapeutic role, but should be preceded by the administration of factor VIII. Finally, research of the etiology of osteoarticular clinical signs must include these haematological diseases if there are suggestive clues that justify the request for targeted diagnostic tests. Informed consent to publish had been obtained.

**Reference**

Jean-Baptiste G, De Ceulaer K.Osteoarticular disorders of haematological origin. Baillieres Best Pract Res Clin Rheumatol. 2000 ;14(2):307-23. Review

**Disclosure of Interest**

None Declared

### P414 MUSCULOSKELETAL DISEASE MIMICKER: CONSIDER VITAMIN C DEFICIENCY

#### Kirsty McLellan^1^, Neil Martin^1^, Paula Beattie^2^, Iain Horrocks^3^, John Hamilton^4^, Stuart Henderson^5^, Mary Ray^6^, Jo Walsh^1^

##### ^1^Paediatric Rheumatology; ^2^Paediatric Dermatology; ^3^Paediatric Neurology; ^4^Paediatric Biochemistry, Royal Hospital for Children, Glasgow; ^5^Paediatrics, Raigmore Hospital, Inverness; ^6^Paediatrics, Royal Alexandra Hospital, Paisley, UK

###### **Correspondence:** Kirsty McLellan

**Introduction:** Often forgotten in the developed world, vitamin C deficiency occasionally presents to rheumatology. With non-specific multisystem presentation, children can pose a diagnostic challenge and often have extensive investigations to rule out malignancies, neuromuscular disorders or arthritis.

**Objectives:** To describe cases referred to rheumatology with musculoskeletal symptoms explained by vitamin C deficiency over previous 6 years, with serum vitamin C level <15umol/L.

**Methods:** Retrospective case review

**Results:** We present a series of 4 autistic children whose wide variety of phenotypes were explained by vitamin C deficiency and responded to supplementation.

Case 1: 12 year old boy, background Fragile X and autism, with 5 weeks nocturnal hand pain and reduced function, unresponsive to simple analgesia. Joint examination revealed hypermobile fingers only. Subsequent review identified limited diet and bleeding gums. Vitamin C level was 12umol/L and symptoms completely resolved with vitamin C treatment.

Case 2: 3 year old autistic girl, with 4 months intermittent limp, finger swelling with morning stiffness; diagnosis of juvenile idiopathic arthritis(JIA) had been considered. Diet was very restricted. No evidence of synovitis on rheumatology review. Ankle X-ray showed osteopenic bones. Vitamin C level was 4umol/L and she was treated with vitamin C.

Case 3: 13 year old autistic boy, previously diagnosed with Henoch-Schonlein Purpura, with 1 month limp, painful legs, right knee and ankle swelling and vasculitic rash. Diet was poor with no fruit or vegetables. Rheumatology review identified he was non-weight bearing, thickened left ankle, with pain on hip movement. Dermatology review elicited follicular hyperkeratosis, with coiled follicular hairs. Vitamin C level was <1umol/L and he was successfully treated with vitamin C, with rapid resolution of symptoms, with only some mild residual joint contractures.

Case 4: 6 year old autistic boy, thought to have JIA, with 4 months painful legs, lower limb rash and then progressive stiffness and pain. He had a limited diet but reported to take daily multivitamins. On examination, non-blanching follicular rash on lower limbs, painful hips on internal rotation and warm, full ankles. He had brisk reflexes, truncal and proximal weakness. He was investigated thoroughly by Neurology, including lumbar puncture and MRI brain and spine which was essentially normal. After 10 day admission on hospital diet, symptoms improved. Vitamin C level subsequently returned at <1umol/L and he responded well to treatment.

**Conclusion:** We present a series of 4 autistic children with restricted diets, 3 of whom had been given other diagnoses, and several had extensive investigations. Increased awareness following the herald case facilitated earlier diagnosis in the subsequent patients and recognition of other patient groups at potential risk, including those with inflammatory bowel disease. As well as classical perifollicular petechiae, hyperkeratosis, gingival bleeding, bony manifestations may precede in the paediatric population. Pathophysiology includes defective bone structure; degradation of bone trabeculae, haemorrhages within the subperiosteal layer and osteoporosis. Musculoskeletal phenotypic and radiographic variability amongst vitamin C deficient paediatric patients should not dissuade clinicians from the diagnosis.

Awareness of this seldom recognised condition, in any child, but especially autistic children, along with the phenotypic variability is necessary to prevent consequent morbidity and unnecessary investigations and treatment. Informed consent to publish had been obtained.

**Disclosure of Interest**

None Declared

### P415 ARTHRITIS- A PREVIOUSLY UNRECOGNISED FEATURE OF KBG SYNDROME

#### Kirsty McLellan^1^, Gulshan Malik^2^, Joyce Davidson^1,2^

##### ^1^Paediatric Rheumatology, Royal Hospital for Children, Glasgow; ^2^Paediatrics, Royal Aberdeen Children's Hospital, Aberdeen, UK

###### **Correspondence:** Kirsty McLellan

**Introduction:** With just over 100 cases reported in the literature, KBG syndrome is a rare genetic condition with characteristic developmental delay, dysmorphic features, macrodontia and skeletal anomalies. Arthritis has not previously been described in individuals with KBG syndrome.

**Objectives:** To describe the clinical course of this case of KBG syndrome.

**Methods:** Retrospective case note review.

**Results:** Case description:

This 17 year old girl, presented aged 20 months with bilaterally swollen knees and elbow stiffness. She had a background of global developmental delay (speech and motor), with significant behavioural issues and learning difficulties. Examination elicited bilateral knee flexion contractures and left wrist pain. Initial management included non-steroidal anti-inflammatories, physiotherapy and splints. Over the subsequent few years she developed wrist and 3^rd^ proximal interphalangeal (PIP) joint restriction. She was noted to have relative short stature (9-25^th^ centile) and some dysmorphic features, with a prominent forehead, hypertelorism, brachydactyly and 5^th^ finger clinodactyly and single palmar crease on right hand. Paediatric psychiatry assessment was requested aged 4, due to her unusual behaviour and social interaction, and she was diagnosed with an atypical autistic disorder.

By age 6, when she was first seen in paediatric rheumatology, her joint symptoms had progressed. Clinical examination confirmed inflammatory polyarthritis especially of her wrists, knees, ankles and PIP joints. She had short-lived benefit from multiple intra-articular joint injections. She had a partial response to methotrexate but stopped this due to poor tolerance. She had various biological therapies all with limited response; etanercept for 6 months aged 10, infliximab aged 12 stopped after 8 months due to an infusion reaction, tocilizumab for 18 months from age 13 which was stopped as it was ineffective. She has now been on abatacept for 4 years with reasonable control of her arthritis.

She developed increasing back pain and stiffness with unusual radiographic appearances: elongated vertebral bodies on x-ray and MRI with endplate changes and loss of anterior vertebral body height. She subsequently developed dystrophic toe nails. Aged 14 years she developed seizures.

She had an extensive genetic and metabolic diagnostic work-up with normal karyotype, normal organic and amino acids. Possible diagnoses including Rett’s syndrome, Smith Magenic syndrome and mucopolysaccharidoses were excluded. Chronic infantile neurological cutaneous and articular syndrome (CINCA), was considered but clinical picture and investigations were not thought to be consistent and no mutation in NLRP3 was identified.

At the age of 14, a genetic mutation in ANKRD11 gene, diagnostic of KBG syndrome was identified. KBG is an autosomal dominant condition cause by mutations in ANKRD11, which has an essential role in controlling acetylation and expression of genes during development of the nervous system*.* Mutations of this gene occur at 16q24.3. Phenotype typically includes learning difficulties and developmental delay, macrodontia, with short stature, seizures, feeding difficulties and autism all prominent features. Recognised skeletal features include short stature, costovertebral abnormalities, clinodactyly, but to our knowledge there are no previous descriptions of inflammatory arthritis as a feature.

**Conclusion:** We describe a case of KBG syndrome with arthritis being a key feature in her clinical picture. We suggest consideration of this diagnosis in children presenting with arthritis in the context of developmental delay and other characteristic clinical features.

**Disclosure of Interest**

None Declared

### P416 AUTOIMMUNE LIVER DISEASE AND NECROTISING MYOPATHY IN A 15-YEARS-OLD BOY

#### Marta Mesquita^1^, Cristina Gonçalves^1^, Paula Estanqueiro^2^, Manuel Salgado^2^, Isabel Gonçalves^1^

##### ^1^Liver transplantation Unit, Coimbra Hospital and University Centre; ^2^Rheumatology Unit, Pediatric Hospital - Coimbra Hospital and University Centre, Coimbra, Portugal

###### **Correspondence:** Marta Mesquita

**Introduction:** Immune-mediated necrotising myopathy (IMNM) is a rare and severe subtype of Idiopathic inflammatory myopathy (IIM). It results from an immune reaction that promotes muscle destruction with consequent necrosis and regeneration, causing proximal muscle weakness. IMNM can be idiopathic, secondary to viral infections, malignancy and connective tissue diseases or be associated with statin administration.

**Objectives:** To present a clinical case of rare association of IMNM and Autoimmune liver disease.

**Methods:** Description of a case report.

**Results:** A 15-years-old healthy boy was admitted to the Liver Unit in April 2017 due to epistaxis and increased liver enzymes levels. A type 1 Autoimmune Hepatitis (AIH) was diagnosed (hypergammaglobulinemia, positive antinuclear and anti-smooth muscle antibodies and histological interface hepatitis) and he started immunosuppressive protocol with steroids and azathioprine. In June 2017 he begun generalized lower limbs myalgia. During the following months the patient had a fluctuating pattern of muscle pain without weakness or cutaneous abnormalities. In parallel, he maintained an oscillating increased liver enzymes and creatine kinase (CK) levels (range: 100-4 610 U/L). The immunosuppressive schedule was modified (steroid, mycophenolate and cyclosporine) but was unsuccessful in inducing remission. In January 2018, after clinical worsening with a limitative mechanical back pain, he was admitted for further investigation and a clinical evaluation by paediatric rheumatologist was performed. The patient presented an extensive 12 cm right lumbar pain that got worsen with muscle masses palpation, without trigger points to myofascial syndrome, and with a normal osteoarticular examination including sacroiliac manoeuvres. A muscle limb ultrasound was performed disclosing muscle oedema. The anti-actine and anti-centromere antibodies were positive, the myositis specific antibodies panel was negative. Muscle histology was compatible with IMNM as well as the electromyography. At present, the patient is under steroids and high doses of mycophenolate and there were notice some improvement in muscle complaints as well as decreased of liver enzymes' and CK levels.

**Conclusion:** There are only few reported adult cases about the association of autoimmune liver diseases with IIM. In contrast to the present case report, IMNM typically presents with a pronounced acute/subacute symmetrical muscle weakness associated to an increased CK levels, and myalgia may or may not be present. The finding of predominant necrosis with scarce inflammation in the muscle histology is a critical tool in the diagnosis. These patients often require intensive immunomodulation and are prone to relapse.

**References**

1. Oldroyd A, Lilleker J, Chinoy H. Idiopathic inflammatory myopathies – a guide to subtypes, diagnostic approach and treatment. Clin Med (Northfield Il). 2017; 17(4):322–8.

2. Basharat P, Christopher-Stine L. Immune-Mediated Necrotizing Myopathy: Update on Diagnosis and Management. Curr Rheumatol Rep. 2015; 17(12).

3. Sedej K, Toplak N, Praprotnik M, Luzar B, Brecelj J, Avčin T. Autoimmune hepatitis as a presenting manifestation of mixed connective tissue disease in a child Case report and review of the literature. Pediatr Rheumatol. 2015; 13(1):13–6.

4. Hounoki H, Shinoda K, Ogawa R, Taki H, Tsuneyama K, Tobe K. Simultaneously developed polymyositis and autoimmune hepatitis. BMJ Case Rep. 2011; 9–11.

Written informed consent for publication of the clinical details was obtained from the patient's parent.

**Disclosure of Interest**

None Declared

### P417 OSTEOARTICULAR INFECTIONS AFFECTING THE AXIAL AXIS

#### Rosa M. Alcobendas^1^, Agustin Remesal^1^, Sara Murias^1^, Clara Udaondo^1^, Cristina Calvo^2^

##### ^1^Pediatric Rheumatology; ^2^Pediatric and infectious diseases, university hospital La Paz, Madrid, Spain

###### **Correspondence:** Sara Murias

**Introduction:** Sacroiliitis and spondylodiscitis of infectious origin are uncommon. Together, they account for approximately 2-4% of total osteoarticular infections (OAI). They usually occurs in children with fewer than 5 years of age. The clinical presentation is insidious, which can cause a long diagnostic delay.

**Objectives:** To describe the clinical characteristic and outcome of these OAI.

**Methods:** Descriptive, retrospective study of the last 12 years (January / 2005 - December / 2017) of children <18 years diagnosed of infectious sacroiliitis or spondylodiscitis in a tertiary hospital. Epidemiological, clinical and analytical variables were analyzed.

**Results:** During the study period 382 children were diagnosed of osteoarticular infection, affecting axial axis in 25 patients ( 6.5%, 13 spondylodiscitis and 12 sacroiliitis), 17 were females (68%). The mean age at onset was 25.5 ± 10 months, all of them younger than 4 years. The mean delay time until diagnosis was 15.6 ± 8 days (3-32). Eleven patients (44%) presented fever at some point in the evolution, being other reasons for consultation: rejection of sitting (44%), limp (36%), pain (28%), immobility (24%), irritability (20%) and abdominalgia or hyperlordosis (both 8%). In addition, 9 patients (36%) associated previous or concomitant upper respiratory infection. Blood test was performed in all patients, obtaining erythrocyte sedimentation rate (ESR) and C reactive protein (CRP) in 22 and 23 cases, respectively. In 20 patients (80%), ESR was over than 20 mm/h (mean 61,4 ± 28.7). On the other hand, only 8 patients (32%) had CRP higher than 20 mg/L (median 20, IQR= 28-4). The leukocyte count was normal, and 13 patients (52%) had thrombocytosis > 400,000. Blood cultures were negative in all patients.

Bone scan was performed in 23 patients, confirming the diagnosis in 22. The remaining 2 cases were diagnosed by magnetic resonance. Spondylodiscitis were all located at the lumbosacral level (4 L5-S1, 3 L4-L5, 2 L3- L4, 1 L2, 1 L2-L3, 1 S1). All were treated with antibiotics (cefuroxime axetil in 20 cases, amoxicillin clavulanic acid in 4, and 1 clindamycin). Eleven patients received intravenous treatment (mean duration 2.5 days, IQR 3-5). The total duration of antibiotic treatment was median 28 days (IQR 21-28). All the children had a good response to the treatment and did not present follow-up sequelae.

**Conclusion:** Osteoarticular infections affecting the axial axis were infrequent in our hospital. Moreover, the symptoms at onset was non-specific and without accompanying fever in more than half cases. For those reasons, high index of suspicion is needed. The rejection of sitting or irritability were complains that should put us on alert. Correct diagnosis is essential to start treatment early, avoiding possible complications. In our series the outcome was good in all cases, without complications or sequelae.

**Disclosure of Interest**

None Declared

### P418 PEDIATRIC MIXED CONNECTIVE TISSUE DISEASE: CLINICAL AND IMMUNOLOGICAL PATTERNS OF DISEASE EXPRESSION IN A COHORT OF MEXICAN CHILDREN.

#### Sofia Osorio, ANDRES NOE R. GARCIA, ROCIO MALDONADO, ENRIQUE FAUGIER, MARIA BRAÑA, LUIS APARICIO

##### REUMATOLOGY, HOSPITAL INFANTIL DE MEXICO FEDERICO GOMEZ, Ciudad de mexico, Mexico

###### **Correspondence**: Sofia Osorio

**Introduction:** Mixed Connective Tissue Disease (MCTD) is a clinical entity characterized by a combination of signs and symptoms of Systemic Lupus Erythematosus (SLE), Systemic Sclerosis (SSC) and polymyositis/dermatomyositis (PM/DM) with high titers of antibodies targeting the U1 small nuclear ribonucleoprotein particle (U1 snRNP) in peripheral blood.

Pediatric Mixed Connective Tissue Disease (pMCTD), in terms of rheumatic disease, is rare. Multiple studies have reported a frequency of 0.1 -0.5 %, with no ethnic predisposition. Median age of onset is 11 years, with female predominance of 6:1. Only 23% of MCTD cases present in childhood.

**Objectives:** This study aims to describe the epidemiological, clinical and immunological features of pMCTD in a Mexican pediatric hospital in the last 5 years.

**Methods:** We conducted a retrospective study by reviewing the medical records of pediatric patients treated for mixed connective tissue disease (Kasukawa´s criteria) between January 2013 and March 2018. For each case of pMCTD the parameters studied were epidemiological, clinical and immunological features.

**Results:** We found a cohort of 7 patients with a mean age at diagnosis of 13 ± 1.2 years old, six (85.7%) patients were female. The range in the diagnosis delay was 2 to 60 months. The most common clinical manifestations were arthritis (100%), Raynaud (85.7%), hand edema (71%), sclerodactyly (71%), Pulmonary Arterial hypertension (42.9%), lung restriction (28.6%), gastroesophageal reflux (28%), delay in voiding (14%), neurological alteration (28.6%), muscle weakness (28%) and vasculitis (85.7%). Respect the immunological features we found that hiper IgG (100%), antinuclear antibodies (100%) and anti RNP/Sm (100%) were present in all the patients while double-stranded anti-DNA antibodies were positive in 57% patients. During follow-up, the management consisted of systemic steroids in 100%, methotrexate in 66%, nifedipine in 57%, and ciclophosphamide in 57%.

During the evolution of the patients we identified that a patient died secondary to an infectious process related to immunosuppressive therapy, a patient is on induction therapy with monthly cyclophosphamide for pulmonary arterial hypertension and the rest of the patients are without clinical activity with therapy maintenance.

**Conclusion:** The EMTC is a very uncommon disease in pediatrics, with a very variable clinical presentation, uncertain and potentially fatal prognosis. This study suggests that, in mexican children, the expression pattern of the observed disease is similar to what has been described in patients with pMCTD from other series.

**Disclosure of Interest**

None Declared

### P419 KIMURA DISEASE TREATED WITH CETIRIZINE AND MONTELUKAST – A FIVE MONTH FOLLOW UP

#### Pallavi Pimpale Chavan^1^, Shaila R. Khubchandani^2^, Raju P. Khubchandani^1^

##### ^1^Pediatric Rheumatology Clinic,Department of Pediatrics; ^2^Department of Histopathology, Jaslok Hospital and Research Centre, Mumbai, India

###### **Correspondence:** Pallavi Pimpale Chavan

**Introduction:** Kimura’s disease, syn : eosinophilic lymphogranuloma is a chronic inflammatory disorder of unknown aetiology with angiolymphatic proliferation. It is a disease of middle aged Asian males and is seen less frequently in children.

**Objectives:** We describe a seven year old with steroid-dependent Kimura disease, who responded dramatically to cetirizine (histamine H1 receptor-blocker) and montelukast (leukotriene receptor antagonist).

**Methods:** A seven year old boy , weight 17 kg and height 111 cm, presented with complaints of multiple axillary and inguinal lymph node swellings and fever since 2 years of age, for which he was evaluated elsewhere and diagnosed as a case of Kimura disease. He was initially started on oral steroids to which he responded well but lymph node swelling and fever would recur as soon as steroids were tapered to 10mg (0.6 mg/kg/day). He was thus constantly on steroids for about five years and showed significant growth retardation. Azathioprine was attempted as a steroid-sparer, but was discontinued since he developed transaminitis. He was then referred to us for further management.

**Results:** The clinical details were reviewed and diagnosis reconfirmed by a review of the earlier biopsy. The child was started on cetirizine and montelukast (available as a combination) after a literature search showed scattered reports favouring these agents as steroid-sparers in this condition. Steroid taper was initiated. On first follow up after 6 weeks, he was afebrile and there was no adenopathy on examination. Parent VAS was 10/10. He was continued on cetirizine and montelukast, steroids progressively tapered and stopped in three months. He started gaining weight (18 kg) and height (112.5 cm). Two months have elapsed since cessation of steroids and he continues to remain asymptomatic as per a recent telephonic interview with parents. We propose to discontinue montelukast and observe him in the coming weeks.

**Conclusion:** Cetirizine and Montelukast should be considered as initial steroid-sparing options in Kimura disease, especially in the pediatric age group where they have a much safer adverse effect profile compared to other immunosuppressives. Response to these drugs whose mechanism of action is known should help to provide a clue to the pathogenesis of this mysterious illness. Informed consent for publication had been obtained

**Disclosure of Interest**

None Declared

### P420 FARBER DISEASE: INITIAL RESULTS FROM AN ONGOING GLOBAL NATURAL HISTORY STUDY COLLECTING RETROSPECTIVE AND PROSPECTIVE CLINICAL DATA IN ACID CERAMIDASE DEFICIENCY, WHICH IS OFTEN MISDIAGNOSED AS JIA

#### Alexander Solyom^1^, Paul Harmatz^2^, Carlos Ferreira^3^, Pranoot Tanpaiboon^3^, Norberto Guelbert^4^, Nur Arslan^5^, Balahan Makay^5^, Neslihan Mungan^6^, Fatma Bulut^6^, Ratna Puri^7^, Sunita Bijarnia^7^, Maja DiRocco^8^, Seza Ozen^9^, Ezgi D. Batu^9^, Marta Torcoletti^10^, Erik Sundberg^11^, John Mitchell^12^, Dustin Tetzl^13^, Bo Magnusson^11^

##### ^1^Enzyvant, Basel, Switzerland; ^2^UCSF Children's Hospital Oakland, Oakland; ^3^Children's National Medical Center, Washington, USA; ^4^Children's Hospital of Cordoba, Cordoba, Argentina; ^5^Dokuz Eylul University Hospital, Izmir; ^6^Cukurova University Hospital, Adana, Turkey; ^7^Sir Ganga Ram Hospital, Delhi, India; ^8^Istituto Giannina Gaslini, Genoa, Italy; ^9^Hacettepe University Hospital, Ankara, Turkey; ^10^University of Milan, Milan, Italy; ^11^Karolinska University Hospital, Stockholm, Sweden; ^12^Montreal Children's Hospital, Montreal, Canada; ^13^Enzyvant, New York, USA

###### **Correspondence:** Dustin Tetzl

**Introduction:** Mutations in the *ASAH1* gene cause acid ceramidase deficiency (Farber disease), accumulation of the pro-inflammatory and pro-apoptotic lipid ceramide, and a distinct set of clinical features, which can vary greatly in severity. Typically, Farber disease presents in childhood with polyarticular arthritis or joint contractures, subcutaneous nodules and a hoarse or weak voice due laryngeal nodules. The prevalence of Farber disease is currently unknown, but it is likely underdiagnosed due to lack of awareness of the clinical presentation and of the availability of diagnostic testing. Acid ceramidase enzyme replacement therapy is currently under development.

**Objectives:** The primary purpose of the study is to establish the natural history of Farber disease, through analysis of retrospective and prospective data on patients, including living patients who have and have not undergone hematopoietic stem cell transplantation (HSCT) and deceased patients. Additionally, the goal is to establish a set of clinical, laboratory (biomarkers), and functional data characterizing distinct groups within this population.

**Methods:** The “Observational and Cross-Sectional Cohort Study of the Natural History and Phenotypic Spectrum of Farber Disease” was initiated in November 2017. 19 patients have been enrolled in the study to date (9 living, 10 deceased), from 11 sites in 7 countries. Demographic and epidemiologic data is available in these patients, as is information about the onset of symptoms and time to diagnosis in most cases.

**Results:** The ratio of females to males is 8:11. 68% (13/19) of the patients enrolled were born in 2008 or later. The ages of the living patients, who may be able to participate in the prospective portion of the study, range from 2-28 years (mean: 12.2 years), with 5/9 (55.5%) living patients currently under the age of 12 years. All patients enrolled to date developed joint disease (arthritis and/or contractures) and subcutaneous nodules at some point over the course of their disease. In the patients for whom this data is available, subcutaneous nodule size ranges from 1-50mm in diameter, and the current number of nodules per patient ranges from 1-68.

**Conclusion:** This is the first study to systematically collect both retrospective and prospective data in both transplanted and non-transplanted patients with Farber disease. Further patients are expected to be enrolled from additional countries and centers in 2018. The data currently available supports clinical evidence indicating that Farber disease has a broad clinical spectrum, with symptoms and age at onset overlapping with polyarticular JIA. It may take years for typical symptoms to appear together, indicating that additional awareness of Farber disease symptoms and phenotypes among pediatric rheumatologists may allow earlier and more efficient diagnosis.

**Trial registration identifying number:** NCT03233841

**Disclosure of Interest**

A. Solyom Shareholder of: Enzyvant, Employee of: Enzyvant, P. Harmatz: None Declared, C. Ferreira: None Declared, P. Tanpaiboon: None Declared, N. Guelbert: None Declared, N. Arslan: None Declared, B. Makay: None Declared, N. Mungan: None Declared, F. Bulut: None Declared, R. Puri: None Declared, S. Bijarnia: None Declared, M. DiRocco: None Declared, S. Ozen: None Declared, E. Batu: None Declared, M. Torcoletti: None Declared, E. Sundberg: None Declared, J. Mitchell: None Declared, D. Tetzl Employee of: Enzyvant, B. Magnusson: None Declared

### P421 CHALLENGES IN ACHIEVING CONSENSUS FOR VACCINATION WITH LIVE ATTENUATED VACCINES IN CHILDREN WITH RHEUMATOLOGICAL DISEASE — THE VARIABILITY OF VACCINATION PRACTICES ACROSS THE GLOBE

#### Natasa Toplak^1^, Yosef Uziel^2^, Raju Khubchandani^3^, Mario Abinun^4^, Erato Atsali^5^, Isabel Bolt^6^, Christina Boros^7^, Yaryana Boyko^8^, Joan Calzada-Hernandez ^9^, Tomas Dallos^10^, Sarka Fingerhutova^11^, Marco Gattorno^12^, Veronique Hentgen^13^, Lovro Lamot^14^, Balahan Makay^15^, Kirsten Minden^16^, Violetta Opoka-Winiarska^17^, Ilona Orban^18^, Gecilmara Pileggi^19^, Chris Pruunsild^20^, Skirmante Rusoniene^21^, Marite Rygg^22^, Andrejs Scegolevs^23^, Jelena Vojinović^24^, Nico Wulffraat^25^, PRES Vaccination working party

##### ^1^Univ. Children's Hosp., Ljubljana, Slovenia; ^2^Meir Med. Center, Tel Aviv, Israel; ^3^Jaslok Hosp. and Research Center, Mumbai , India; ^4^Newcastle Univ., Newcastle upon Tyne, UK; ^5^National and Kapodestrian Univ., Athens, Greece; ^6^Inselspital, Univ. Hosp., Bern, Switzerland; ^7^Univ., Adelaide, Australia; ^8^West. Ukr. Spec. Ped. Med. Centre , Lviv, Ukraine; ^9^Hosp. Sant Joan de Déu, Barcelona, Spain; ^10^Comenius Univ. Med. School, Bratislava, Slovakia; ^11^General Univ. Hosp.,1st Faculty of Med., Prague, Czech Republic; ^12^G. Gaslini institute, Genova, Italy; ^13^French Ref. Center for AI diseases, Versailles, France; ^14^Sestre milosrdnice Univ. Hosp. Centre, Zagreb, Croatia; ^15^Behçet Uz Children Hosp., Izmir, Turkey; ^16^Charité Univ. Med. and German Rheum. Res. Center, Berlin, Germany; ^17^Med. Univ., Lublin, Poland; ^18^National Inst. of Rheum. and Physioth., Budapest, Hungary; ^19^Ribeirão Preto Med. School, Univ., Sao Paulo, Brazil; ^20^Children’s Clinic, Univ. Hosp., Tartu, Estonia; ^21^Clinic of Children Dis., Vilnius Univ., Vilnius, Lithuania; ^22^Fac. of Med. and Health Sci., NTNU, and Dept. of Pediatrics, St. Olavs Hosp., Trondheim, Norway; ^23^Univ. Children’s Hosp., Riga, Latvia; ^24^Univ., Niš, Serbia; ^25^Univ. Med. Center, Utrecht, Netherlands

###### **Correspondence:** Natasa Toplak

**Introduction:** Due to the paucity of randomised controlled studies concerning vaccination in children with rheumatic diseases, the level of evidence for recommendations for vaccinations in these children is low. Booster doses of live attenuated vaccines might be considered in children with rheumatic diseases treated with immunosuppressive therapy, but data from multicentre studies are lacking. Moreover, national vaccination programs, parental obligation to vaccinate their children and vaccine coverage rates vary greatly among countries.

**Objectives:** To highlight differences in the current national vaccination policies, and to develop a platform for future multicentre initiatives for uniform vaccination practices for children with rheumatic diseases treated with immunosuppressive drugs.

**Methods:** The PReS Vaccination working group was formed during the 2017 PReS meeting in Athens. Paediatric rheumatologists from 34 countries were invited to participate.

**Results:** Data were collected from 25 countries who responded. Vaccinations are mandatory in 12/21 European countries (Croatia, Czech Republic, France, Greece, Hungary, Italy, Latvia, Poland, Serbia, Slovakia, Slovenia, Ukraine). The vaccination schedules and coverage differ among countries. The first MMR vaccine is recommended at 11-15 months-of-age in all countries and most recommend the second dose before 2 years-of-age or at 6 years; however in Spain it is at 2-4 years, in the UK at 3-5 years, and in Hungary, The Netherlands, Estonia, Norway, Poland and Slovakia at the age of 9 years or later. Mandatory programs, as compared to optional vaccination, do not always ensure higher coverage. For example, in Australia, Israel, The Netherlands and Norway where vaccinations are optional, the vaccination rate is high, at around 95%. However, coverage for MMR fell below 95% in Croatia, Czech Republic, Serbia and Slovenia, where vaccination is mandatory. Vaccinations were optional in France and Italy; however, due to low coverage, they are now mandatory.

**Conclusion:** There are considerable differences amongst countries in vaccination programmes, coverage, and in parental obligation to vaccinate their child. A powerful anti-vaccine campaign has gained momentum in many countries and has resulted in a significant drop in vaccination coverage to a level that is no longer sufficient for herd immunity. This is especially dangerous for children with rheumatic diseases on immunosuppressive therapy. Our future goals are to prospectively examine the outcomes of live vaccination in children with rheumatic diseases who are treated with immunosuppressive drugs and hopefully to demonstrate that booster doses of live attenuated vaccines are safe and protective.

**Disclosure of Interest**

None Declared

## Autoinflammatory diseases

### B01 TNF-RECEPTOR RELATED PERIODIC SYNDROME (TRAPS) PRESENTED BY RECURRENT PERICARDITIS

#### Ceyhun Acari^1^, Gültaç Evren^2^, Ferhat Demir^3^, Mukaddes Kalyoncu^3^, Erbil Ünsal^1^

##### ^1^Department of Pediatrics, Pediatric Rheumatology; ^2^Department of Pediatrics, Pediatric Intensive Care Unit, Dokuz Eylul University Faculty of Medicine, Izmir; ^3^Department of Pediatrics, Pediatric Rheumatology, Karadeniz Technical University, Medicine of Faculty, Trabzon, Turkey

###### **Correspondence:** Ceyhun Acari

**Introduction:** Pericarditis is the inflammation of pericard and may occur following infections, malignancy, autoimmune and autoinflammatory diseases. It might be acute, chronic and recurrent. It might be life threatening by altering the hemodynamics of the heart and causing constructive pericarditis.

**Objectives:** The patient with TRAPS was reported.

**Methods:** The patient's file rewieved retrospectively. Phsical examination, laboratory test, imaging and genetic analyses results evaluated.

**Results:** CASE: A fifteen-year-old girl was consulted to the emergency department with high fever and chest pain. She was then transferred to intensive care unit due to severe pleural effusion with tachypnea and tachycardia. The pleural fluid was exudative, she had fibrinous pericarditis. The bacterial cultures including tuberculosis were negative. She continued to have severe respiratory distress despite pericardiotomy, and pericardiectomy was performed. Fibrinous pericarditis was confirmed by histochemical study of pericardiectomy material and neither malignancy nor tuberculosis were detected. Histopathological examination of pleura consisted of mainly neutrophils. In laboratory, hemoglobin was 9.4 g/dl, leukocytes were 12300/mm^3^, (76% neutrophil, 14% lymphocyte), thrombocytes were 427000/mm^3^, C-reactive protein was 126 mg/L, erythrocyte sedimentation rate was 89 mm/h, procalcitonin was 0.28 ng/ml, troponin was <0,01 ng/ml, and liver and renal function tests, immunoglobulin levels were normal. Anti-nuclear antibodies and anti-neutrophilic cytoplasmic antibodies were found as negative and C3, C4 levels were normal. No malignancy was detected in the bone marrow. High dose IV prednisolone successfully controlled the disease. Colchicine was added due to pericarditis protocol. Detailed history revealed recurrent chest pain and fever attacks. TRAPS was suspected, and p.arg121Gln mutation in TNFRSF1A gene was detected which was recorded as pathological. She was discharged with colchicine, but had another similar attack, which was controlled by anti IL-1 treatment.

**Conclusion:** TRAPS, also known as Hibernian fever, is an autosomal dominant inherited autoinflammatory disease, which is localized in 12p13 chromosome. TRAPS presents with recurrent attacks of fever lasting up to 1-4 weeks, myalgia, periorbital edema, and serosal inflammation (peritonitis, pleuritis). Rarely, pericarditis is reported during the attacks^1^. A study indicates that TRAPS constituted 6% of the idiopathic recurrent pericarditis^2^. TRAPS and other rare autoinflammatory diseases should be kept in mind in the differential diagnosis of recurrent pericarditis. Informed consent for publication had been obtained from the parents.

**Disclosure of Interest**

None Declared

### B02 Withdrawn

### B03 HYPERIMMUNOGLOBULIN D SYNDROME: 4 SIBLINGS CASE

#### Serife G. Karadag, Ayşe Zopcuk, Mustafa Çakan, Nuray Aktay Ayaz

##### Pediatric Rheumatology, KANUNI SULTAN SULEYMAN TRAINING AND RESEARCHING HOSPITAL, Istanbul, Turkey

###### **Correspondence:** Serife G. Karadag

**Introduction:** Hyperimmunoglobulin D syndrome (HIDS) is an autosomal recessive periodic fever syndrome presenting with fever, vomiting, diarrhea, abdominal pain, rash, arthralgia, and arthritis.

**Objectives:** Herein, we describe 4 HIDS cases in the same family.

**Methods: Case 1:** A 6 year old boy who applied with recurrent fever, abdominal pain, vomiting, joint swelling and rash every month for 4 years. There was no mutation in the MEFV gene, and colchicine treatment did not prevent his attacks. G18R heterozygote mutation was detected on the MVK genome. The patient is followed without attack by treatment with anti-IL-1 (canakinumab).

**Case 2:** A 3 year old girl, with complaints of fever, vomiting, leg pain for 2 to 3 days every 2-3 months was using colchicine with FMF diagnosis but no improvement in her attacks.

Genetic analysis revealed a G18R heterozygote mutation in the MVK gene. After the anti-IL-1 therapy attacks resolved.

**Case 3:** A 2 year old girl, was admitted with episodes of fever, diarrhea and vomiting that lasted 3-4 days, which was observed 4 times in the last 2 months. Genetic analysis revealed a G18R heterozygote mutation in the MVK gene. The patient is followed without attack by treatment with anti-IL-1.

**Case 4:** A 8 year old girl was admitted with complaints of recurrent fever, abdominal pain and headache**.** Genetic analysis revealed a G18R heterozygote mutation in the MVK gene. Following the initiation of anti-IL-1 therapy, the complaints were resolved.

**Results:** With anti-IL1 therapy, 4 siblings are followed without attacks.

**Conclusion:** Symptoms may be different in patients with HIDS even if they are the members of the same family or carrying the same mutation. HIDS should be considered when recurrent fever episodes are accompanied by rash, arthritis, vomiting and diarrhea. Anti-IL1 treatment is very effective in reducing symptoms and prevention of amyloidosis. Informed consent to publish had been obtained.

**Disclosure of Interest**

None Declared

### B04 Withdrawn

### B05 CRYOPYRIN-ASSOCIATED PERIODIC SYNDROME (CAPS): THE DESCRIPTION OF THE CLINICAL CASE CONFIRMED BY A MUTATION IN THE NLRP3 GENE

#### Ivan Kriulin^1^, Ekaterina Alexeeva^1,2^, Tatiana Dvoryakovskaya^1,2^, Bella Bursagova^2^, Kirill Savostyanov^2^, Natalya Ghurkova^2^, Alexandr Pushkov^2^

##### ^1^Sechenov University; ^2^National Medical Research Center of Children’s Health, Moscow, Russian Federation

###### **Correspondence:** Ivan Kriulin

**Introduction:** Cryopyrin-associated periodic syndromes (CAPS) are a group of rare congenital auto-inflammatory diseases with onset in the first year of life with the advent of fever, urticarial rash and various options for joint removal - from arthralgia to recurrent and persistent arthritis in severe cases, as well as damage to the nervous system.

**Objectives:** Consider the clinical case of the child with cryopirin-associated periodic syndrome.

**Methods:** The laboratory - instrumental examination was conducted of 1 year and 8 months old boy, who entered the rheumatological department of the National Medical Research Center of Children’s Health with the systemic juvenile idiopathic arthritis.

**Results:** The patient A. entered the rheumatological department with diagnosis systemic JIA. Complaints: fever up to 39.0°C, a rash, pain and limitation of movements in the joints. On examination: urticarial rash all over the body, hepatosplenomegaly, lymphadenopathy, polyarthritis with involvement of interphalangeal joints of hands and feet, wrists, elbows, hips, knees, ankles, temporomandibular joints and the cervical spine. Blood tests: hypochromic anemia (Hb 82 g/l), thrombocytosis (platelets 650 x 10^9^/l), ESR - 45 mm/h, CRP level - 96.88 mg/l, ferritin - 2334.86 ng/ml (norm of 15 - 80 ng/ml). The child was consulted by the audiologist and ophthalmologist - the pathology of hearing and vision was not presented. The child is suspected of an auto-inflammatory syndrome, because of the severe condition, debuting in first year and the resistance to the standard anti-rheumatic therapy (prednisolone 13 mg/day, tocilizumab 12 mg/kg once every 14 days within one month). Exon 04 of the NLRP3 gene with adjacent intron regions were examined by the method of direct automatic sequencing. A mutation c.943A> G in the heterozygous state, resulting in the amino acid substitution p.I315V, was detected. A previously prescribed topic was described in patients with cryopyrin-associated periodic syndrome. The "Cryopyrin - associated periodic syndrome” diagnosis was confirmed by the previous research study results, pathogenetic therapy was prescribed by giving the genetically engineered biological agent - canakinumab (interleukin 1 inhibitor) for subcutaneous injection at a dose of 4 mg/kg (52 mg) once every 4 weeks. After the first injection of canakinumab, the rash disappeared, but fever, hepatosplenomegaly, lymphadenopathy persisted, macrophage activation syndrome developed (ferritin - 3318.01 ng/ml, white blood cells 6.71 x 10^9^/l). The child was given glucocorticoids for oral administration (prednisolone at a dose of 2 mg/kg, 25 mg per day) and for injection (dexamethasone 20 mg /m^2^ / day in 2 divided doses). The therapy was effective, patient`s condition improved significantly - fever, rash, hepatosplenomegaly, lymphadenopathy, arthritis resolved (Hb 111 g/l, ESR - 3 mm/h, CRP level – 1.48 mg/l, ferritin – 25.55 ng/ml). The second and third injection of canakinumab gave no reaction. Intravenous glucocorticoids were withdrawn the dose of oral GK was reduce to 18.75 mg/day. At the moment, the patient is on therapy (canakinumab 52 mg once every 4 weeks, prednisolone 5 mg per day) for more than six months without signs of systemic inflammation.

**Conclusion:** Cryopyrin - associated periodic syndrome, with onset at an early age, characterised by an aggressive course and inefficiency of glucocorticoid therapy. To verify the diagnosis and to provide targeted therapy to children with a clinical picture of systemic juvenile arthritis, a molecular genetic study should be performed to exclude or confirm a monogenic autoimmune syndrome. Informed consent to publish had been obtained from the parent.

**Disclosure of Interest**

None Declared

### B06 EARLY ONSET SARCOIDOSIS ASSOCIATED WITH NOD2 MUTATION: A CASE REPORT

#### Francesca Orlando, Roberta Naddei, Marina Amico, Carolina Porfito, Teresa Lastella, Maria Alessio

##### Mother and Child Department, University of Naples Federico II, Naples, Italy

###### **Correspondence:** Francesca Orlando

**Introduction:** Early onset sarcoidosis (EOS) is characterized by a triad of polyarthritis, uveitis and rash. The familial cases, manifesting the classic clinical triad and an autosomal transmission pattern, have been termed Blau syndrome (BS). Mutations involve the NOD2 gene.

**Objectives:** To describe clinical presentation and treatment strategies in a patient affected by EOS.

**Methods:** Case report of a patient affected by pediatric sarcoidosis referred to Pediatric Rheumatology Unit of Federico II University of Naples.

**Results:** The patient (female, six years old) was referred to our Unit at the age of 2 years. The family history revealed a mother with rheumatoid arthritis and visual loss due to panuveitis. The clinical history disclosed that at the age of 12 and 15 months the patient had two episodes of diffused rash spontaneously resolved in two weeks. From the age of 18 months recurrent hip pain, defined as transient synovitis of the hip, was reported. It was treated successfully with non-steroideal antinflammatory drugs (NSAID) but pain would reappear once stopped the treatment. Clinical evaluation showed polyarticular symmetrical arthritis of wrists, elbows, knees, proximal interphalangeal (PIP) joints. Polyarticular Juvenile Idiopathic Arthritis (JIA) diagnosis was made and treatment with NSAID and Methotrexate was started without significant improvement, therefore Etanercept was added and NSAID stopped. Screening of uveitis was performed every six months. Persisting arthritis of right wrist after 7 months of therapy, ultrasound was performed showing tenosynovitis. Therefore, she underwent intra-articular injection of glucocorticoids with partial response. At the age of 4 years, she presented papuloerythematous rash, mainly on the trunk, infiltrated and confluent in plaques, so skin biopsy was performed resulting consistent with sarcoid-like granulomatous disease . Suspecting BS molecular analysis was done. At the age of 5 years, she presented scotoma and visual loss; the ocular assessment found bilateral anterior uveitis and posterior bilateral synechiae. She started systemic glucocorticoid therapy and shifted from Etanercept to Adalimumab with improvement of both ocular and articular disease. The molecular analysis of NOD2 showed missense mutation R334W consistent with BS. The ocular disease evolved into iridolenticular synechiae and band keratopathy. She started topical treatment with improvement. Currently, the articular and cutaneous manifestations are controlled by the combined therapy with Adalimumab and Methotrexate. The ocular disease is steady.

**Conclusion:** The patient presents typical clinical manifestations of pediatric sarcoidosis, confirmed by skin biopsy. R334W is one of the most common mutations reported in the international Blau registry. The presence of typical clinical manifestations, NOD2 mutation and family history led to the diagnosis of Blau Syndrome. The molecular analysis of NOD2 gene in the mother is ongoing.

Informed consent for the publication was obteined from the parents.

**Disclosure of Interest**

None Declared

### B07 EARLY ONSET SACROILIITIS IN FAMILIAL MEDITERRANEAN FEVER

#### Elif Çelikel, Zeynep Birsin Özçakar, Nilgün Çakar, Fatma Aydın, Fatoş Yalçınkaya

##### Pediatric Rheumatology, ANKARA UNIVERSITY, Ankara, Turkey

###### **Correspondence:** Zeynep Birsin Özçakar

**Introduction:** Sacroiliitis has rarely been reported in patients with familial Mediterranean fever (FMF), especially in the pediatric population.

**Objectives:** In this report we present patients with FMF related sacroiliitis who had very early disease onset.

**Methods:** Two cases with documented sacroiliitis at 3 and 4.5 years of age were presented.

**Results:** A 5-year-old male patient had the complaints of intermittent hip pain and limping attacks with duration of 15 days, started at 2 years of age. He had also recurrent fever attacks and his father has FMF. At 3 years of age sacroiliac joint magnetic resonance imaging (MRI) showed left sided sacroiliitis and MEFV gene analysis was compatible with homozygous p.M694V mutation. HLA-B27 was negative. Colchicine was commenced and his complaints improved. Seven months later hip pain and limping attacks started again and described partial improvement with ibuprofen. At the age of 5 years, control MRI revealed bilateral sacroiliitis. Salazopyrin was added to his treatment.

The second patient is a 7 year old boy who had the diagnosis of ulcerative colitis at the age of 14 months. He was referred to rheumatology clinic due to hip pain, low back pain and gait disturbance at 3 years of age. He had family history of FMF and ongoing elevated acute phase reactants but no typical FMF attacks. HLA-B27 was negative. Mutation analysis revealed p.M694V/p.M680I, sacroiliac joint MRI showed pelvic enthesitis and colchicine was started. His complaints improved. At the age age of 4.5 years, due to intermittent complaints and elevated acute phase reactants, control MRI was performed and showed bilateral sacroiliitis. Adalimumab was commenced. He had no complaints thereafter.

**Conclusion:** Familial Mediterranean fever related sacroiliitis can be seen in very small children. Furthermore, in patients with early onset sacroiliitis FMF should be investigated in the differential diagnosis. Written consent for publication was obtained from the parents

**Disclosure of Interest**

None Declared

### B08 EFFICACY OF INTERLEUKIN-1 TREATMENT AT TISSUE LEVEL IN A PATIENT WITH RENAL AMYLOIDOSIS SECONDARY TO FAMILIAL MEDITERRANEAN FEVER

#### Selçuk Yüksel^1^, Nagihan Yalçın^2^, İlknur Girişgen^3^, Gülçin Otar Yener^1^, Zahide Ekici Tekin^1^

##### ^1^Pediatric Rheumatology; ^2^Pathology; ^3^Pediatric Nephrology, Pamukkale University School of Medicine, DENIZLI, Turkey

###### **Correspondence:** Selçuk Yüksel

**Introduction:** Although there are previous studies demonstrating regression of proteinuria due to amyloidosis secondary to familial Mediterranean fever (FMF) by colchicine and/or anti-interleukin 1 treatment, there is no study about response to therapy at tissue level of amyloidosis secondary to FMF (1,2).

**Objectives:** We aimed to evaluate the influence of colchicine and anti-interleukin 1 treatment at tissue level in a case of FMF with amyloidosis.

**Methods:** A 10-year-old boy presented with nephrotic range proteinuria (83.5 mg/m2/hour, 2 g/day) and high level acute phase reactants in May 2011. First kidney biopsy revealed renal amyloidosis. Mediterranean fever mutation test showed homozygous for M694V, R202Q and E148Q mutations. Colchicine (1.5 mg/day) and enalapril (7.5 mg/day)  was commenced. Although the proteinuria was reduced to 10 mg/m2/hour within 2 years by this treatment, acute phase reactants did not return to normal values. Therefore, second kidney biopsy was performed in July 2013, since ongoing amyloidosis and subclinical inflammation, anti-interleukin 1 (anakinra 2mg/kg/day) was commenced as an additional treatment to colchicine and enalapril. Two months later, proteinuria (<4mg/m2/hour) and acute phase reactants were normal. In Semptember 2015 anakinra was switched to canakinumab (2mg/kg/month). Remission was successfully maintained, he didn’t experience attacks, after switching to canakinumab. After obtaining from parental and the patient consents, third kidney biopsy was performed in April 2017. Creatinine clearance never deteriorated during follow-up period. Three renal biopsies were re-evaluated and compared according to the hystopathological classification, scoring and grading system established by Sen et al. (4).

**Results:**

**Table:** 

The renal amyloidosis scoring and grading system items	First Biopsy (May 2011)	Second Biopsy (July 2013)	Third Biopsy (April 2017)
Class of glomerular amyloid deposition (GAP)	Class II Mesangial minimal amyloid deposition + 2	Class III Focal mesangiocapillary amyloid deposition (including nodular amyloidosis) +3	Class III Focal mesangiocapillary amyloid deposition (including nodular amyloidosis) +3
Percentage of glomerular amyloid deposition (GA%)	%10-25 +2	%25-50 +3	%25-50 +3
Vascular amyloid deposition (VA)	Moderate +3	Focal +2	Minimal +1
Interstitial amyloid deposition (IA)	Minimal +1	Absent 0	Minimal +1
Interstitial fibrosis and tubular atrophy (Ifib)	Absent 0	%10-25 +2	%10-25 +2
Interstitial inflammatory infiltration (Iinf)	%1-10 +1	%1-10 +1	%1-10 +1
Glomerular sclerosis (GS)	Absent 0	Absent 0	Absent 0
**The renal amyloid prognostic score (RAPS)**	**9**	**11**	**12**
**Amyloid grade**	**II**	**II**	**II**

Although the renal amyloid grade remained stable, RAPS progressed slowly.

**Conclusion:** We concluded that anti-interleukin 1 treatment in patient with amyloidosis secondary to FMF could not stop progression at tissue level, but it could lead to clinical improvement. We need to new treatment strategies that not only to remove the inflammation, but also to dissolve amyloid deposits. Informed consent for publication had been obtained.

**References**

1. Varan Ö, Kucuk H, Babaoglu H, Guven SC, Ozturk MA, Haznedaroglu S, Goker B, Tufan A. Efficacy and safety of interleukin-1 inhibitors in familial Mediterranean fever patients complicated with amyloidosis. Mod Rheumatol 2018; 26:1-9

2. Özçakar ZB, Özdel S, Yılmaz S, Kurt-Şükür ED, Ekim M, Yalçınkaya F. Anti-IL-1 treatment in familial Mediterranean fever and related amyloidosis. Clin Rheumatol 2016; 35:441-6

3. Sen S, Sarsik B. A proposed histopathologic classification, scoring, and grading system for renal amyloidosis: standardization of renal amyloid biopsy report. Arch Pathol Lab Med 2010; 134:532–44

**Disclosure of Interest**

None Declared

## Bone in rheumatic disease

### B09 POSSIBILITIES OF CORRECTION OF OSTEOPENIC STATES IN CHILDREN

#### Anna Y. Spivakovskaja, Yury M. Spivakovskiy, Yury V. Chernenkov

##### Department of Hospitality Pediatrics, SARATOV STATE MEDICAL UNIVERSITY, Saratov, Russian Federation

###### **Correspondence:** Yury M. Spivakovskiy

**Introduction:** The modern period of studying different pathogenetic links of the course of JIA is characterized by a special attention to the formation of changes in bone tissue. Violations of the bone tissue due to a decline in mineral bone density can be registered as the first signs of arthritis and in later stages of the disease.

**Objectives:** To evaluate the possibility of non-drug correction of vitamin d deficiency for the prevention of bone metabolism disorders in children with articular form of JIA.

**Methods:** The study included 32 patients with articular JIA and 28 children with other arthritis, which were divided into three equal and comparable groups. In each of the groups applied different methods of correction of vitamin D deficiency, namely: subgroup A – children receive a balanced diet and a specialized product for enteral of food containing 52 IU of vitamin D_3_ 100 ml (non-medicament correction of deficiency/insufficiency of vitamin D); subgroup B – children only received nutrition, subgroup С - children were on medical correction of deficiency/insufficiency of vitamin D officinal drugs.

**Results:** In subgroup "А" all children before correction were in deficit (75 %) or deficiency (25%) of vitamin D. After correction, the proportion of children with a level of 25(OH)D below 30 ng/ml did not exceed 30%. Before the beginning of the correction in all children with oligoarticular variants (OAV) of JIA from subgroup “A” vitamin D deficiency was registered (below 20 ng/ml), after correction more than 62% of the examined children reached the level of 25(OH)D more than 30ng/ml in serum. With polyarticular variant (PAV) JIA succeeded in all children with vitamin D insufficiency increase its level in blood serum above 30 ng/ml, while 50% of children with a deficiency to increase the level of vitamin D in the blood serum before 21-29 ng/ml. The use of non-pharmacological correction of vitamin D deficiency in children with other arthritis in history has allowed more than 80 % of patients to achieve normal values of 25(Oh)D in serum (more than 30 ng/ml). In subgroup "B" 95% of children at the beginning of the study had a level of 25(OH)D in serum below 30 ng/ml. After 4 weeks of observation, there was a slight improvement in the indicators 25(OH)D in serum, and the proportion of children who have overcome the threshold of 30 ng/ml, increased to 10%. Among children with other arthritis in the anamnesis it was possible to achieve a twofold increase in the proportion of patients with a level of 25(OH)D in serum more than 30 ng/ml. In subgroup "C" 90% of children at the time of the study had vitamin D deficiency (serum 25(OH)D level below 20 ng/ml), only 2 patients had vitamin D level in the zone of insufficiency (21-29 ng/ml). After 4 weeks of medical correction in patients of this subgroup, the proportion of children who overcame the threshold of 30 ng/ml was 85%. In the subgroup "C" after 4 weeks of medical correction in all patients with polyarthritis, in 90% of children with oligoarthritis and in 87,5% of cases in children with other arthritis in the history noted a positive result-the concentration of 25 (OH)D in serum levels above 30 ng/ml.

**Conclusion:** The increase in the subgroups "A" and "C" after the 4-week course as a non-pharmacological and pharmacological correction of deficit of 25(Oh)D in serum in the proportion of children with a normal supply of vitamin D is from 0 to 75%, and 90% of cases, respectively, demonstrates the effectiveness of the applied methods, and the lack of significant differences in the achieved level of 25(Oh)D in the serum of patients in these subgroups confirms the possibility of using non-pharmacological correction of vitamin D deficiency for the prevention of osteopenia, based on the use of specialized products for enteral nutrition.

**Disclosure of Interest**

None Declared

## Disease outcome

### B10 SURVEY OF DISEASE CHARACTERISTICS, FUNCTIONAL STATUS AND DAMAGE OF JIA PATIENTS FOLLOWED IN A TERTIARY PEDIATRIC RHEUMATOLOGY CENTER

#### Ioana A. Muresan^1^, Sorina Boiu^1^, Erato Atsali^1^, Lampros Fotis^1^, Vana Papaevangelou^1^, Dimitrios Boumpas^2^

##### ^1^Pediatric Rheumatology Unit,3RD Department of Pediatrics; ^2^Rheumatology Department, Attikon University Hospital, National and Kapodestrian University of Athens,Medical School,Athens,Greece, Athens, Greece

###### **Correspondence:** Ioana A. Muresan

**Introduction:** Important advances in management of JIA have been made over the last few decades. However, there are still differences with regard to outcomes across European countries.

**Objectives:** To assess disease activity, functional status and damage in JIA patients of all subtypes followed in a southeastern Pediatric Rheumatology tertiary center (Greece).

**Methods:** JIA patients consecutively seen in a Pediatric Rheumatology reference center between January and April 2018 were enrolled in this observational cross-sectional study. Outcome measures included numbers of active and limited joints, physician’s VAS of overall disease activity, parent’s VAS of global wellbeing and pain, CHAQ, articular and extra-articular damage. Criteria for inactive, low, moderate and high disease activity were applied [1; 2]; for patients with enthesitis-related arthritis, (ERA) the absence of enthesitis was an additional criterion. CHAQ score, overall wellbeing and pain scores were divided into 4 categories: 0 (no disability), >0 and ≤0.5 (mild disability), >0.5 and ≤1.5 (moderate disability), and >1.5 (severe disability), as previously described [3]. For statistical analysis, non-parametrical tests were used.

**Results:** A total of 24 JIA patients were included. Our cohort comprised 67% oligoarticular JIA, 12% polyarticular RF-negative JIA and 21% ERA patients with median disease duration of 1 year (range 1 month - 15 years). Treatment comprised NSAIDs (54%), MTX (71%), intra-articular corticosteroids (50%), systemic corticosteroids (25%) and biotherapy (38%). Criteria for inactive disease and low, moderate and high disease activity were met by 13% and 8%, 50%, 29% of patients, respectively. Uveitis developed in 13% of patients, all with oligoarticular JIA. The median value of CHAQ score was 0 (range 0-1.875). The majority of patients had no or mild functional disability (88%) and pain (63%); however moderate or severe overall well being impairment was present in more then half of the patients (58%). Within JIA subgroups, ERA patients had significantly worse scores for CHAQ, VAS wellbeing and pain. CHAQ significantly correlated with disease activity (p<0.0001), physician’s global VAS (p<0.0001) and with VAS scores for overall wellbeing (p=0.0009) in altogether JIA patients. Articular and extra-articular damage were found in 8% and 13% of patients, respectively.

**Conclusion: Conclusion.** In the present cohort, the majority of patients had moderate disease activity but good functional scores, probably related to the short disease duration. ERA patients still represent a management challenge. A good control of disease is essential in order to preserve the functional abilities and the overall wellbeing of JIA patients.

**References**

1. Wallace, C. A., N. Ruperto and E. Giannini (2004). "Preliminary criteria for clinical remission for select categories of juvenile idiopathic arthritis." J Rheumatol 31(11): 2290-2294

2. Consolaro, A., N. Ruperto, A. Bazso, A. Pistorio, S. Magni-Manzoni, G. Filocamo, C. Malattia, S. Viola, A. Martini and A. Ravelli (2009). "Development and validation of a composite disease activity score for juvenile idiopathic arthritis." Arthritis Rheum 61(5): 658-666

**Disclosure of Interest**

I. Muresan Shareholder of: none, Grant / Research Support from: none, Consultant for: none, Employee of: none, Paid Instructor for: none, Speaker Bureau of: none, S. Boiu: None Declared, E. Atsali: None Declared, L. Fotis: None Declared, V. Papaevangelou: None Declared, D. Boumpas: None Declared

## Imaging

### B11 A RARE PRESENTATION OF CYSTIC GANGLIONOSIS IN AN 18 MONTH OLD CHILD

#### Jayne M. MacMahon^1^, Michael Carter^2^, Michael Moore^3^, Niamh McSweeney^1^

##### ^1^Department of Paediatrics and Child Health, Cork University Hospital; ^2^Department of Paediatrics and Child Health, Cork Uuniversity Hospital, Cork; ^3^Department of Radiology, Cork University Hospital, Cork, Ireland

###### **Correspondence:** Jayne M. MacMahon

**Introduction:** Cystic ganglionosis is a benign condition whereby multifocal soft tissue masses arise adjacent to joints and tendons. Management is conservative unless there is damage to underlying bone in which case surgical resection is rarely indicated.

**Objectives:** We present the case of an 18-month old girl, who presented for assessment of multiple soft tissue masses on her hands and feet that had developed over the preceding 4months.

**Methods:** She was otherwise well and there was no family history of note. Blood tests including full blood count, inflammatory markers, thyroid function, renal and bone profile were normal. On examination, she was noted to have painless multiple soft tissue swellings, varying in size from 0.5cm to 4cm across her phalanxes and the dorsum of her feet. These were raised with no overlying skin changes. An xray of her foot showed no evidence of bone or joint space abnormality. She went on to have an ultrasound of her right foot, where multiple well defined simple fluid structures consistent with ganglion cysts were evident. This was confirmed with a MRI of her right foot, which showed multiple simple cysts adjacent to and communicating with the joints of the foot, predominantly the tarus.

**Results:** A diagnosis of cystic ganglionosis was made and she has been referred to the National Centre of Rheumatology for further review.

**Conclusion:** Cystic ganglionosis is rare in children and to our knowledge this case is only the third such case reported in a child less than 12yrs of age.[i] [ii] Informed consent to publish had been obtained from the parent.

[i] Meyer, N.P., Meyers, A.B., Szabo, S. et al. A rare case of cystic ganglionosis in a child with associated imaging findings. Skeletal Radiol. 2016 Mar45(30: 419-26.

[ii] Shinawi M, Hicks J, Guillerman RP, et al. Multiple ganglion cyst (‘cystic ganglionosis’): an unusual presentation in a child. Scand J Rheumatol. 2007 Mar-Apr;36(2):145-8.

**Disclosure of Interest**

None Declared

## Immunodeficiency and infection related arthritis

### B12 EFFECT OF ORTHOSIS AND CLINICAL PILATES EXERCISES ON HYPERIMMUNGLOBULIN E SYNDROME: A CASE REPORT

#### Gamze Arın^1^, Nur Banu Karaca^1^, Erdal Sağ^2^, Yelda Bilginer^2^, Edibe Ünal^1^, Seza Özen^2^

##### ^1^Department of Physiotherapy and Rehabilitation, Hacettepe University Faculty of Health Sciences; ^2^Department of Pediatric Rheumatology, Hacettepe University Faculty of Medicine, Ankara, Turkey

###### **Correspondence:** Gamze Arın

**Introduction:** Hyperimmunoglobulin E syndrome (HIES) is a rare primary immunodeficiency disease characterized by elevated serum immunoglobulin E levels, recurrent skin and lung infections, chronic dermatitis and various connective tissue and skeletal abnormalities.

**Objectives:** To investigate the effectiveness of orthosis and cilinical pilates exercises for eliminating knee joint deficits in the presence of HIES.

**Methods:** 12,5 years old boy with HIES, applied to the rheumatology department with complaints of the weakness and stiffness of the gastrocnemius muscles and effusion on the knees for the last 2 months. It was reported that the complaints were relieved by removal of fluid from the patient's knees, but there was an abnormality in the direction of the genu valgus in the knee and the development of walking difficulty due to it. The patient was directed to physiotherapy at this stage. A goniometric measurement was made for knee joint positional sensation while lying on the back of the patient and antropometric measurement was made for the edema. For functional evaluation, observational walking analysis, 10 m walking test, time up and go test (TUG) were performed. The Childhood Health Assessment Questionnaire (CHAQ) was administered for activities of daily living and pain. Exercise training was given once a week for 8 weeks. The home exercise program was given for other days. Clinical Pilates exercises were given for the proprioception and muscle strength. It was decided to support the knee with a double articulated orthosis covering the knee and ankle to increase medial-lateral control. The skin was followed up frequently and edema-related orthosis adaptations were made.

**Results:** After 8 weeks of training, improvement in knee position sensation, TUG, CHAQ scores was observed (Table 1). However, the improvement observed until the sixth week was delayed due to the effusion on the knees in the seventh week. This decline was reflected in the results of anthropometric measurements and changes in pain scores at week 8.

**Conclusion:** In the case of HIES, selected clinical pilates exercises for ankle and knee joints and orthosis practice was found to be beneficial for the patient's walking. Considering the attacks of the disease, it was concluded that the patient should be evaluated at frequent intervals. Informed consent to publish had been obtained.

**Disclosure of Interest**

None Declared

**Table1 (abstract B12).** The patient's first and 8th week evaluation scores

	First Assessment		Second Assessment	
Edema: Antropometric measurements	Right	Left	Right	Left
Middle of the knee (cm)	38,5	41,5	43	43
Middle of the calf (cm)	24,8	26,4	27	36
Ankle (cm)	28	20,5	29,5	23
Perception of knee position: Femur-tibia angle (degree)	30^o^	25^o^	11^o^	13^o^
TUG (sec)	13,3		9,3	
10 m walk (sec)	11,93		18,73	
CHAQ	1,125		0,875	
Pain (cm)	5,1		5	

## Immunoregulation and basic science

### B13 INFLAMMATORY SEROSITIS: WHEN IT IS NOT RHEUMATOLOGIC NOR INFECTIOUS

#### Sofia Costa^1^, Leonor Rocha^1^, Patrícia Silva^2^, Paula Estanqueiro^1^, Alexandra Dinis^3^, António Pires^2^, Leonor Carvalho^3^, Manuel Salgado^1^

##### ^1^Rheumatology Department; ^2^Cardiology Department; ^3^Intensive Care Unit, Hospital Pediátrico, Centro Hospitalar e Universitário de Coimbra, Coimbra, Portugal

###### **Correspondence:** Sofia Costa

**Introduction:** Pericarditis, pleuritis and/or peritonitis can be present in infectious, malignant or inflammatory processes. Inflammatory serositis are common associated with rheumatologic and auto-inflammatory diseases. There are other conditions that may be complicated by a transitory autoimmune reaction responsible by reactive inflammatory serositis: Inflammatory Serositis not Rheumatologic nor Infectious (ISnRnI).

**Objectives:** To describe the different etiologies, treatment and evolution of ISnRnI.

**Methods:** Retrospective study of inflammatory serositis observed in the Pediatric Intensive Care Unit and/or the Pediatric Rheumatology Department of our hospital in the last 30 years.

Exclusion criteria: Chronic rheumatologic, auto-inflammatory and infectious diseases.

**Results:** Twenty-two cases of ISnRnI were selected: 62,5% male, with a median age of 9 years. Thirty-six per cent had pericardial effusion only, 46% had both pericardial and pleural effusions and 18% had poliserositis (pericardial, pleural and peritoneal). Seven were idiopathic, and 15 were considered postpericardiotomy syndrome: 7 post cardiac surgery, 2 post cardiac catheterization, 3 related to chest trauma, 1 following surgery for pectus excavatum and 2 were diagnosed with an anterior mediastinal mass.

Fever, chest pain and respiratory signs were the most frequent symptoms and 3 patients had cardiac tamponade at presentation. The median of days at the beginning of the symptoms, in “postpericardiotomy syndrome” cases, was 33 days (min. 0 days; max. 180 days).

The median diagnostic delay was 2 days (min. 0 days; max. 120 days). Eighty-six per cent of the patients was admitted in the Intensive Care Unit. Antibiotics were initiated in 86% of the patients. Nonsteroidal anti-inflammatory drugs (NSAIDs) were used in 14 patients, 5 were managed with NSAIDs and colchicine and 5 patients received corticosteroids. Pericardiocentesis was performed in 46% of the patients, thoracentesis in 9% and both techniques in 27% of the patients. Recurrence was observed in 8 (36%) patients, median days after the first episode being 35 days. One patient died with cardiorespiratory arrest during pericardial drain replacement.

**Conclusion:** Different conditions from rheumatologic and auto-inflammatory diseases can cause serositis. Any anterior mediastinum procedure or trauma can induce an immunologic reaction with inflammatory serositis, such as postpericadiotomy syndrome. Both diagnostic delay and unnecessary antibiotic use are frequent in these cases. NSAIDs and colchicine are essential in the management of inflammatory serositis, with oral corticosteroids reserved for complicated cases. Colchicine is useful in preventing the frequent relapses.

**References**

1. Castellani C, Saxena AK. Pleural and Pericardial Associations After Minimal Pectus Repair. In: Saxena AK. Chest Wall Deformities. Springer-Verlag Heidelberg, 2017:383-387.

2. Gouriet F, Levy PY, Casalta JP. Etiology of Pericarditis Cohort of 1162 Cases. Am J Med. 2015;128(7):784.e1-8.

3. Yukumi S, Suzuki H, Kashu Y. Postpericardiotomy syndrome after thymothymectomy: report of two cases. Gen Thorac Cardiovasc Surg. 2012;60(7):462-4.

4. Imazio M, Hoit BD. Post-cardiac injury syndromes. An emerging cause of pericardial diseases. Int J Cardiol. 2013;168(2):648-52.

5. Raval J, Nagaraja V, Eslick GD. The Role of Colchicine in Pericarditis – A Systematic Review and Meta-analysis of Randomised Trials. Heart Lung Circ. 2015;24(7):660-6.

**Disclosure of Interest**

None Declared

### B14 MOLECULAR GENETIC STUDIES OF HUMAN LEUKOCYTE ANTIGEN (HLA) CLASS II ALLELES AND CLINICAL PROFILE OF ACUTE RHEUMATIC FEVER IN LATVIA

#### Marina Visnevska^1^, Valda Stanevica^1^, Jelena Eglite^2^, Vita Rovite^3^, Zane Davidsone^1^, Andrejs Scegolevs^4^, Ruta Santere^4^

##### ^1^Rīga Stradiņš University; ^2^Rīga Stradiņš University, Joint Laboratory of Clinical Immunology and Immunogenetics; ^3^Latvian Biomedical Research and Study Centre; ^4^Children’s Clinical University Hospital, Riga, Latvia

###### **Correspondence:** Marina Visnevska

**Introduction:** Acute rheumatic fever (ARF) is an autoimmune disease following group A streptococcal (GAS) infection. Despite being rare, in Latvia new cases still occur. RF remains a clinical syndrome which has variable manifestations according to genetic predisposition, prevalence of GAS strains, and socio economical conditions. There are data that genetic susceptibility to RF is linked to HLA class II alleles. However, studies in different populations have given conflicting results of susceptibility and/or protective alleles [1]. In Latvian patients diagnosed with RF between 1995 and 2001, DRB1*07 allele was associated with the disease [2]. Since 2001, 27 newly diagnosed RF cases have been reported in Latvia. Therefore, a thorough understanding of immunological mechanisms involved in the development of RF is required.

**Objectives:** To describe the clinical characteristics of ARF in children in Latvia between 1995 and 2016 and to investigate immunogenetic markers of HLA-DRB1; -DQB1; -DQA1 in patients diagnosed with RF since 2001.

**Methods:** Part A: A retrospective study included 96 children diagnosed with RF between 1995 and 2016 at Children’s Clinical University Hospital (CCUH). We analysed medical case reports, evaluating clinical and laboratory data and compliance with the Jones criteria. Statistical analysis was performed using SPSS Statistics.

Part B: A prospective study included 21 out of 27 patients diagnosed with RF between 2001 and 2016, and 100 healthy controls. Patients were genotyped for HLA-DRB1; -DQB1; -DQA1 using RT-PCR with sequence-specific primers. Statistical analysis was performed using DOS StatCalc program, Cochran-Mantel-Haenszel test and Fisher’s exact correction.

**Results:** Part A: Among 96 RF patients 64.6% (n=62) were boys. The age of patents at time of diagnosis varied between 4 and 17 years, the median age was 10 years (IQR 5-15). Carditis (85.4%) and polyarthritis (42.7%) were the most frequent Jones criteria. Most commonly (in 41.5% of cases) patients had either mitral valve or multivalvular (MV+AV) lesions. Sydenham's chorea occurred in significantly fewer patients (16.7%). Erythema marginatum and subcutaneous nodules were rarely observed (10.4%, 1% respectively). Of the minor Jones criteria the most common were arthralgia (81.3%), elevated inflammatory markers (79.2%) and fever (61.5%).

Part B: Significant associations were found between the RF and control groups. Alleles DRB1*04:01 (OR=1.28, p=0.027), *07:01 (OR=9.8, p=0.001) and *11:01 (OR=1.38, p=0.029) were more often observed in the group with RF and described as risk alleles. The risk variants were also DQB1*03:01 (OR=1.13, p=0.01), *03:02 (OR=2.12, p=0.017), *04:01-2 (OR=1.6, p=0.01) and DQA1*02:01 (OR=1.90, p=0.015), *03:01 (OR=1.75, p=0.018) alleles*.* The frequencies of DQB1*06:02-8 (OR=0.46, p=0.019) and DQA1*05:01 (OR=0.99, p=0.001), *06:01 (OR=0.67, p=0.01) alleles were decreased and described as protective for RF.

**Conclusion:** ARF most commonly occured in school-aged children, and boys were more affected by the disease*.* Clinical presentation was typical with cardiac (85.4%), polyarticular (42.7%) and neurological involvement (16.7%). Our study confirms that certain HLA class II alleles are associated with risk or protection from RF.

**References**

1. Bryant PA, Robins-Browne R et al. Some of the people, some of the time: susceptibility to acute rheumatic fever. *Circulation*. 2009;119:742-75.

2. Stanevicha V, Eglite J et al. HLA class II DR and DQ genotypes and haplotypes associated with rheumatic fever among a clinically homogeneous patient population of Latvian children. *Arthritis Res Ther.* 2007,9(3),58-67.

**Disclosure of Interest**

None Declared

## JIA (oligo, poly, psoriatic)

### B15 COMPARISON OF TURKISH AND BRITISH CHILDREN WITH JIA: A PILOT STUDY

#### Gamze Arın^1^, Lauren Trotman^2^, Susan Maillard^2^, Yelda Bilginer^3^, Edibe Ünal^1^

##### ^1^Department of Physiotherapy and Rehabilitation, Hacettepe University Faculty of Health Sciences, Ankara, Turkey; ^2^Great Ormond Street Hospital for Children, London, UK; ^3^Department of Pediatric Rheumatology, Hacettepe University Faculty of Medicine, Ankara, Turkey

###### **Correspondence:** Gamze Arın

**Introduction:** Juvenile Idiopatic Arthritis (JIA) is one of the most common chronic, rheumatic diseases in children. The prevalence of disease in a systematic study is reported to be 1,6-23/100,000 in Europe. In Turkey, this ratio has been reported as 6.4/100.00.

**Objectives:** The aim of this study is to compare the characteristics of children with JIA in the Turkish and British communities.

**Methods:** The study included children of the same age and sex, diagnosed with JIA who were followed in the Pediatric Rheumatology Department of the Hacettepe University İhsan Doğramacı Children's Hospital and the Great Ormond Street Hospital for Children. The Child Health Assessment Questionnaire (CHAQ) was used to assess the general health status of children, the visual analogue scale of the clinician and the family was used to assess the general condition of the child. Demographic data and active and limited joint numbers were recorded.

**Results:** A total of 26 children participated in the study (n=13 for British, n?13 for Turkish). There was no difference in age, diagnosis, sex, CHAQ, and number of active joints in the two groups (p>0,05). However, there was a difference between limited joints and general health assessments from family and clinician (p<0,05).

**Table:** 

	TURKISH (n=13)	BRITISH (n=13)	**p**
Age	12,9±1,38	12,9±1,38	1,000
BMI	21,07±3,05	20,63±6,31	0,309
Sex	9F/4M	9F/4M	1,000
CHAQ	0,33±0,34	0,87±0,87	0,202
Active Joints	0,54±0,87	1,17±1,52	0,324
Limited Joints	0,33±0,65	1,83±2,55	**0,045**
Parental VAS	1,38±2,06	5,46±2,87	**0,001**
Physician VAS	1±2,08	4,09±2,58	**0,003**

**Conclusion:** As a result of this pilot study, there was no difference in the CHAQ scores of children with JIA when age and gender matched, but there were differences in the general situation assessment between the family and the clinician. This study will be continued by increasing the number.

**Disclosure of Interest**

None Declared

### B16 EFFICACY OF INTRA ARTICULAR CORTICOSTEROID INJECTIONS AND METHOTREXATE IN POLYARTHRITIS ASSOCIATED WITH HARLEQUIN ICHTHYOSIS

#### Simone Carbogno^1^, Francesco Baldo^1^, Sofia Torreggiani^1^, Sophie Guez^2^, Francesca Minoia^2^, Giovanni Filocamo^2^

##### ^1^University of Milan; ^2^Fondazione IRCCS Cà Granda Ospedale Maggiore Policlinico Milano, Milan, Italy

###### **Correspondence:** Simone Carbogno

**Introduction:** Harlequin ichthyosis (HI) is the most severe variant of autosomal recessive congenital icthyosis and it is due to mutations in ABCA12 gene. HI is clinically characterized at birth by hyperkeratotic plates covering the entire body, ectropion, eclabium, poorly developed ears, and joint contractures. Inflammatory joint involvement has been rarely described. [1] The management of polyarthritis in patients with HI is challenging, due to the difficult balance with an increased infectious risk and with disease’s progression. Moreover little is known about effects of conventional DMARDs and biologics on dermatological manifestations.

**Objectives:** We report the case of young boy with HI who developed a polyarthritis successfully treated with intra-articular steroid injections and methotrexate.

**Methods:** We present cinical details of young boy with HI who developed a polyarthritis. Written informed consent for publication of his clinical details and clinical images was obtained from the patient.

**Results:** A 6 year-old male with HI was referred to our rheumatology centre because of chronic polyarthritis. The child was born at term with classical features of HI. During his first years of life he was hospitalised several times due to severe infections. At 4 years of age he developed chronic arthritis with symmetrical involvement of knees; suspecting a septic arthritis associated with HI, he was unsuccessfully treated with antibiotics. Two years later he presented to our centre with erythrodermic skin, joint contractures, severe ectropion. Swelling, pain and severe limitation of motion of hips, knees, ankles, mid-feet, wrists, elbows and left shoulder was detected. The patient was unable to walk since 2 years. Laboratory examinations showed microcytic anemia, mild leukocytosis, thrombocytosis and mild elevation of C-reactive protein (0.79 mg/dl). Antibodies to nuclear antigens, extractable nuclear antigens, cyclic citrullinated peptide and rheumatoid factor were negative. Uveitis screening was negative. He was treated with multiple intra-articular corticosteroid injections (IACI) in both knees, wrists, elbows, and ankles and he was started on methotrexate; due to his severe dermatological involvement oral route was preferred. One month later a dramatic response to treatment was observed and the child started walking again. A limitation of motion at the mid-feet and ankles persisted. Considering the brilliant response to injective treatment, a multiple ultrasound-guided IACI in the mid-feet (previously not injected) is going to be performed at this time. We did not observe any adverse events or infections related to the treatment so far (almost two months follow up).

**Conclusion:** To the best of our knowledge this is the youngest HI patient with polyarthritis described so far, [2,3] and the first one treated with IACI. The increased susceptibility to infection and the growth retardation reported in children with HI make systemic glucocorticoid therapy and global immunosuppression undesirable. IACI allows to avoid most of the systemic side effects of glucocorticoid treatment and results in a more effective and rapid improvement of articular function. In our patient, combined treatment with IACI and methotrexate, successfully controlled articular symptoms and improved his quality of life. Informed consent to publish had been obtianed.

**Reference**

1) Raghuvanshi S, et al. Harlequin Ichthyosis and Inflammatory Arthritis: Case Reports of a very Rare Combination. Rheumatology 2015; 54, suppl_1, i55

2) Clement S A et al. Harlequin ichthyosis and juvenile idiopathic arthritis: a rare combination. Clin Rheumatol 2007; 26: 460–462.

3) Chan YC, et al. Harlequin Ichthyosis in Association with Hypothyroidism and Juvenile Rheumatoid Arthritis. Pediatr Dermatol 2003; 20: 421–426.

**Disclosure of Interest**

None Declared

### B17 CLINICAL SIGNIFICANCE OF HLA B 27 IN JUVENILE IDIOPATHIC ARTHRITIS

#### Adisa Čengić^1^, Aida Dizdarevic^1^, Velma Selmanović^1^, Elma Fejzić^2^

##### ^1^Allergology, Rheumatology and Clinical Immunology, Pediatric Clinic, Clinical Centre University of Sarajevo; ^2^Institute for transfusion medicine, Sarajevo, Bosnia and Herzegovina

###### **Correspondence:** Adisa Čengić

**Introduction:** HLA-B27 plays an important role in the classification of Juvenile idiopathic arthritis (JIA) and it is associated with enthesitis related arthritis^1^, .spondyloarthropathies and reactive arthritis.^2^

**Objectives:** Our objective is to investigate associations of HLA-B27 and the joint involvement in JIA patients and to determine which of the JIA subtypes most frequently has positive HLA-B27.

**Methods:** The retrospective analysis included 21 HLA-B27 positive JIA patient hospitalized in a referral rheumatology centre in the period of two years  Patients were followed for sixth months. The diagnosis of JIA was based on criteria by the International League of Association for Rheumatology (ILAR).^3^ Detection of HLA –B27 was done by using a DNA-based PCR-SSP test.

**Results:** In our group of 21 HLA B27 positive JIA patients, there were 14 boys (66%) versus 7 girls (33%)

The age of JIA onset was 8.4 years (range: 3.2-15 y). Most frequently involved joint was ankle in 12 patients (57.1 %), followed by knee joint (9 patients -42.8%). Small joints of hands were inflamed in 7 patients (33%) and feet and as well as hip joint in 6 children (28.5%). Sacroileitis was detected in 5 patients (23.8%). Uveitis was diagnosed in 2 children (9.5%) and both of them were boys.

Most frequently diagnosed JIA subtype was enthesitis related arthritis (ERA) in 8 patients (38.1%), followed by an oligoarticular JIA in 7 patients (33%) and polyarticular JIA in 5 children (23.8%). One patient could not fit any JIA category so he was diagnosed as having undifferentiated JIA.

**Conclusion:** HLA-B27 antigen can predict development of ERA in children. ERA is usually treatment resistant so knowing HLA B27 status could help in more aggressive therapy approach.

**Disclosure of Interest**

None Declared

### B18 VITAMIN D PROFILE IN JUVENILE IDIOPATHIC ARTHRITIS PATIENTS IN A TERTIARY CARE HOSPITAL IN BANGLADESH

#### Mohammad Imnul Islam, Sufia Khatun, Shahana Rahman, Mohammed Mahbubul Islam, Manik Kumar Talukder

##### Department of Paediatrics, Dhaka, Bangladesh, Bangabandhu Sheikh Mujib Medical University (BSMMU), Dhaka, Bangladesh

###### **Correspondence:** Shahana Rahman

**Introduction:** There are multifactorial causes of decrease in bone mass in JIA patients which correlate with the duration of active disease.By measuring the vitamin D level we can assess the deficiency or insufficiency earlier and can predict the risk of osteoporotic bone fracture and give appropriate supplementation of vitamin D and calcium.

**Objectives:** This study was done to determine the status of serum 25(OH)D in patients with JIA and to see the relationship among various subtypes and disease duration.

**Methods:**

It was a cross sectional study. Thirty newly diagnosed cases ofJIA attending the pediatric rheumatologyclinicof Bangabandhu Sheikh Mujib Medical University (BSMMU), Dhaka, BangladeshfromJuly 2014 to December 2015 were included. Thirty age and sex matched control were selected. Serum 25(OH)D was measured in cases and controls.

**Results:** Among JIA patients, 60 % and among controls 33% had hypovitaminosis D. In JIA group the mean level of serum 25 (OH) D was lower than control group and the result is statistically significant in cases of polyarticular JIA and systemic JIA(SJIA).There is significant difference of the mean values of vitamin D levels in JIA and control groups for the cases of hypovitaminosis D. Level of 25(OH)D was decreased as disease duration increased.

**Conclusion:** More than half of JIA patients had hypovitaminosis D.It is more significant in cases of polyarticular JIA (RF negative) and systemic JIA(SJIA).There was negative relationship between 25(OH)D level and disease duration.

**Disclosure of Interest**

None Declared

**Table 1 (abstract B18).** Comparison of level of serum 25(OH)D in JIA and control group (n=30+30)

*p-value	JIA(n=30) Mean±SD Control(n=30) Mean±SD
Oligoarticular (n=4)	34.83±8.27 0.37
ERA (n =7)	29.38±11.57 0.58
Polyarticular RF + (n =2)	26.66±16.26 31.47±6.11 0.39
Polyarticular RF – (n=9)	24.5±8.31 0.02
Systemic JIA(SJIA) (n = 8)	23.22±13.13 0.05
S.25(OH)D(ng/ml) (n= 30)	26.86±10.97 31.47±6.11 0.135

*unpaired student t-test

### B19 CHARACTERIZATION OF PATIENTS WITH JUVENILE IDIOPATHIC ARTHRITIS FOLLOWED AT THE MULTIDISCIPLINARY CLINIC OF PEDIATRIC RHEUMATOLOGY OF THE UNIVERSITY HOSPITAL CENTER OF THE ALGARVE

#### Ana L. Gomes^1^, Graça Sequeira^2^, Filipa Mestre^3^

##### ^1^Physical Medicine and Rehabilitation; ^2^Rheumatology; ^3^Pediatrics, University Hospital Center of the Algarve , Faro, Portugal

###### **Correspondence:** Graça Sequeira

**Introduction:** Juvenile Idiopathic Arthritis (JIA) is a group of diseases of unknown origin, lasting more than 6 weeks, beginning before the age of 16 years. It is the most frequent rheumatic disease in childhood, affecting predominantly girls, especially in the oligoarticular subtype (the most frequent, corresponding to 50% of cases). Treatment is based on a pharmacological combination, rehabilitation and psychosocial support. The manifestation of the disease is demonstrated by the inflammatory activity. The implication of the inflammatory activity in the daily life activities is a focal point of the evaluation.

**Objectives:** The aim of this study is to characterize patients with JIA followed at the Multidisciplinary Clinic of Pediatric Rheumatology of the University Hospital Center of the Algarve (CHUA).

**Methods:** Descriptive observational study based on the clinical record and the national registry of rheumatic patients (Reuma.pt) of all patients with JIA, followed during 2017, at the Multidisciplinary Clinic of Pediatric Rheumatic Diseases of CHUA. Sociodemographic, clinical, analytical and therapeutic data were analysed. We have included the reference to Physical Medicine and Rehabilitation (MFR). Statistical analysis was performed using Microsoft Excel 2010.

**Results:** Sample of 10 patients (total of 32), 6 boys and 4 girls. The average age of onset was 6.6 years (1-15); age at diagnosis 6.8 years and duration of illness 4.2 years (1-10). The distribution of the ILAR classification was: persistent oligoarticular 60%, arthritis / entesitis 30% and systemic 10%. Analytically we noted ANA and HLA B27 positive in 30% of the cases; the RF and anti-CCP were always negative. 60% of the patients had active disease and 40% were in remission; in relation to CHAQ / HAQ, we obtained a mean score of 0.28 points, with only 2 patients presenting values ​​different from zero; on the JADI A and E scale we obtained an average score of 0.1 and 0.2 points, respectively. 50% of patients had already been exposed to corticosteroids (CE), 60% to synthetic DMARDs and 10% to biological DMARDs; currently only 10% are under CE, remaining 60% with synthetic DMARDs (Methotrexate) and 10% with biological DMARDs (Etanercept). The cumulative exposure to therapy was 0.76 years for CE, 3.11 years for synthetic DMARDs and 0.1 years for biological DMARDs. 20% of patients already had (at some point during the course of the disease) been referred to the MFR consultation, both for elbow flexion deformity.

**Conclusion:** The high frequency of JIA justifies the 31% of patients found; there was no predominance of expected gender, despite the dominance of the oligoarticular category. This category occurs preferentially in girls and before the age of 6; in our sample we found 4 (in this category), with onset of the disease before 6 years old. Two of the 3 patients with HLA B27 positive belonged to the arthritis / enthesitis category, as expected. This category occurs predominantly in boys, after 7-8 years; the 3 cases obtained fit these characteristics. Positive evolution is expected, given negativity for RF and anti-CCP, as well as absence of polyarticular and systemic variants. Although 60% of the patients still have active disease, in 50% of them the duration of the disease and the follow-up is less than 2 years, which may explain this percentage. The main purposes of the treatment are pain control, preservation of joint amplitudes, management and prevention of complications and facilitation of adequate psycho-motor development. Thereby, MFR will play a predominant role in the multidisciplinary team responsible for the support provided to these patients, as seen by the 20% of patients referred to this clinic.

**Disclosure of Interest**

None Declared

### B20 STRUCTURE OF JUVENILE IDIOPATHIC ARTHRITIS ACCORDING TO THE REGISTER OF CHILDREN WITH RHEUMATIC DISEASES IN THE CITY OF MOSCOW

#### Vladislav Sevostyanov^1^, Elena Zholobova^2^

##### ^1^The Research Institute for health organization and medical management, Moscow; ^2^I.M. Sechenov First Moscow State Medical University, Moscow, Russian Federation

###### **Correspondence:** Vladislav Sevostyanov

**Introduction:** In recent decades, there has been a tendency to increase the number of diagnosed systemic diseases, including juvenile idiopathic arthritis (JIA). The working group on pediatric rheumatology established the register of children with rheumatic diseases in Moscow. The tasks of the register include the study of clinical and epidemiological features of the course of JIA in Moscow, the calculation of the need for basic and genetically engineered biological drugs .

**Objectives:** To analyze the structure of juvenile idiopathic arthritis, as well as the structure of Antirheumatic therapy.

**Methods:** Observational descriptive study. The study included 752 patients living in Moscow between the ages of 1 and 17 years, including 480 females (63.8%) and 272 males (36.2%) suffering from JIA.

**Results:** 752 patients at the age of 1-17 years with various options of juvenile idiopathic arthritis were enrolled in the study (male - 272 (36.2%), female - 480 (63.8%)). Among them 283 (37,6%) suffer from polyarticular seronegative JIA, 269 (35,7%) oligoartiсular JIA, 82 (11%) the JIA systemic form, 15,7% are the share of other JIA forms. Average age of patients was 10,8 years, average age of a debut of a disease of 68,5 months (5 years 7 months). The interval between a debut of a disease and establishment of the diagnosis averages 9,2 months, an interval from a disease debut prior to specific therapy of 12,5 months. An age of a debut of a disease for male patients was significantly higher. Female patients had uveitis more often (р =0.0011). DMARD therapy was received by 73,7% of patients (84,1% of cases it was Methotrexate. Biological therapy was received by 43,1% of patients. In structure of biological therapy inhibitors the TNFα prevail (etanercept and adalimumab – 71%). Etanercept was received - 41,7% of all children who are on biological therapy, adalimumab– 29,3%.

**Conclusion:** The problem of studying of JIA is one of the actual areas of Pediatrics. The register of children suffering from rheumatic diseases was established first in Moscow. The work to be done to identify patients with the debut of JIA, as well as patients consisting on the account in the pediatric rheumatologist with the purpose of setting the dispensary record and inclusion in the register.

**Disclosure of Interest**

None Declared

### B21 EXERCISE-THERAPY IN REAL CLINICAL PRACTICE PEDIATRIC RHEUMATOLOGY DEPARTMENT

#### Tatiana A. Shelepina

##### Pediatric, FEDERAL STATE BUDGETARY SCIENTIFIC INSTITUTION “SCIENTIFIC RESEARCH V.A.NASONOVA INSTITUTE OF RHEUMATOLOGY, Moscow, Russian Federation

**Introduction:** JIA severe disabling disease, which leads to motor disorders and disrupts the daily activity of a sick child. and preserving the functionality of the child Exercise therapy is easy method physical activation patients with JIA.

**Objectives:** Analysis of the functional status of patients with JIA in specializedpediatric rheumatology department and the results of the rehabilitation of inpatient and outpatient treatment.

**Methods:** 306 patients aged 2 to 17 years with various JIA-types(a systemic - 7 %,poly-57%,oligo-36%,); 118pts not have problems with function of joints only weakness of musculature, 154 have functional problem in joint without disorder walking and self-service, 34 have problems with walking. There were used: 306 Card of patients with register variant exercise-therapy-in group, individually with instructor, self-dependent. In the functional treatment of patients, general restorative, mobilizing and corrective complexes of physiotherapy exercises were used.

**Results:**

**Table:** 

Days of exercise therapy	% patients
>10days(full course)	36%
6-9days(not full course)	25%
<6 days (Teaching exercises)	39%
Middle 6days	

**Conclusion:** Term exercise**-**therapy in pediatric department is not long, but all patients must have recommendations on exercise-therapy for home. Only regular exercise by the patient with the support of parents and with regular supervision by a physiotherapist can achieve satisfactory results in terms of functional status.

**Disclosure of Interest**

None Declared

### B22 STATUS OF HEMOSTASIS SYSTEM IN CHILDREN WITH PATIENTS WITH YUIA

#### Ljudmila F. Bogmat^1,2^, Viktoria V. Nikonova^1^, Nataly S. Shevchenko^1,3^, Irina N. Bessonova^1,2^

##### ^1^Department of cardiorheumatology, Institute for the Health Care of Children and Adolescents of the National Academy of Medical Sciences of Ukraine; ^2^Department of pediatrics; ^3^Department of pediatrics № 2, V.N. Karazin National University, Kharkiv, Ukraine

###### **Correspondence:** Nataly S. Shevchenko

**Introduction:** It is known that chronic non-specific inflammation in rheumatoid arthritis (RA) acts as an inducer of activation of the coagulation link of hemostasis, contributes to the deficiency of physiological anticoagulants, as well as to the decrease of fibrinolytic activity, which creates pathogenic preconditions for the occurrence of hemocoagulation disorders.

**Objectives:** It was considered necessary to determine the features of the hemostasis system in children with juvenile idiopathic arthritis (UIA) with comorbid pathology.

**Methods:** 97 children (5-18 years) of patients with UIA with oligo and polyarticular variants of the disease were examined. Patients were divided into two groups. The first group consisted of 38 children (39.2%) without symptoms of comorbidity, and the second group, with signs of comorbidity, included 59 children (60.8%). In the group with the presence of comorbidity, the signs of high activity of the rheumatoid process, such as C-reactive protein and circulating immune complexes, were significantly more often recorded. The components of the blood coagulation system were determined from the data of the coagulogram, which included determination of the level of fibrinogen (FG, g / l), prothrombin index (PTI,%), thrombin time (PM, c), activated partial thromboplastic time (AFTV, c), international normalized the ratio (MNF, U.S.), D-dimer, y. at. Statistical processing was performed using the Statgrafics 16.0 application package.

**Results:** In the group of children with signs of comorbidity, an increase in the prothrombin index (p <0,05) was found, as well as a probable prolongation of thrombin time (p <0,03) indicating the probability of thrombotic formation. The parameters of the study of the coagulation system were also studied in a group of patients with signs of kidney damage. There is a probable increase in the prothrombin index (p <0.05), which may indicate a possible development of thrombosis. As for atherosclerosis and thrombosis, inflammation is the main binding factor; we analyzed parameters of hemostasis in a group of children with UIA with signs of atherogenic orientation changes in the lipid spectrum. Signs of the development of thrombocytopenia development were revealed by parameters of PTI (p <0,05). Only in this group of patients a probable increase in the level of D-dimer, the main marker of thrombotic readiness (p <0,05), was established.

**Conclusion:** Thus, in children with UIA, with signs of comorbid pathology, signs of thrombophilic formation have been identified, as evidenced by an increase in both the prothrombin index and the main marker of thrombotic readiness - the D-dimer. Thus, precisely inflammation is the most important factor that supports the threat of development of atherosclerosis, atherothrombosis and venous thrombosis in children with SLE, and it is dangerous not only the process of inflammation in the vascular wall itself, but also the pathological influence on the endothelium of the cells of the immune system and products of the systemic inflammatory responses - acute phase proteins and circulating immune complexes.

**Disclosure of Interest**

None Declared

### B23 SYNOVIAL CHONDROMATOSIS IN DIFFERENTIAL DIAGNOSTICS OF JUVENILE IDIOPATHIC ARTHRITIS (JIA), AND THE ROLE OF IMAGING METHODS

#### Eva Vrtikova^1^, Elena Košková^1^, Jana Sedláková^1^, Zuzana Pechočiaková^2^

##### ^1^National Institute of Rheumatic Diseases, Piešťany; ^2^Department of Pediatrics, University Hospital, Nitra, Slovakia

###### **Correspondence:** Eva Vrtikova

**Introduction:** In the differential diagnostics of juvenile idiopathic arthritis (JIA), different rare diseases that mimic chronic arthritis, should be considered. Our article describes the case, where the rare disease - Synovial chondromatosis had to be excluded in spite of findings by imaging methods.

**Objectives:** A 4 year old female patient presents with complaints of walking difficulties, morning stiffness, swelling of right knee, and limitation of movement. Blood results did not show increased inflammatory activity. The patient was RF and ANA negative. Slit-lamp examination excluded uveitis. Based on signs and symptoms, the diagnosis of Juvenile idiopathic arthritis was considered. Plain radiograph of the knee showed soft tissue swelling. Findings of MRI examination revealed the diagnosis of primary synovial chondromatosis (Reichel´s syndrome). However, the musculoskeletal sonography showed a typical image of proliferative synovitis with high power doppler signal and small amount of anechoic fluid. Inflammatory etiology was confirmed by analysis of the synovial fluid. The diagnosis of JIA was confirmed and the patient was treated by intra-articular application of triamcinolone hexacetonide.

**Synovial chondromatosis** (Reichel´s syndrome or Reichel-Jones- Henderson syndrome) is a benign cartilaginous metaplasia of the synovium, which may affect any synovial joint. Typically, free fragments of cartilage originating from the synovium of the joint are seen in the joint cavities. The disease is usually monoarticular, it affects most commonly the knee, hip, or elbow and less commonly the wrist, ankle, and shoulder. It involves rarely the TMJ. The joint most commonly involved is the knee joint. The condition is connected with pain, swelling and limitation of movement. It typically affects adults between 30 to 50 years old, and occurs extremely rarely in children. Synovial chondromatosis exists as primary and secondary. In primary form the cause is unknown, frequently without known prior trauma or inflammation. Diagnosis is made by radiographs, computed tomography (CT), magnetic resonance imaging (MRI) , by surgery and histology. The prognosis of this disease is good. Asymptomatic patients do not require therapy. Symptomatic patients should undergo arthroscopic or surgical removal of intra-articular bodies. There have been documented very rare cases of malignant transformation. Written consent of the legal guardian has been obtanied to publish this case study.

**Methods:** Case report.

**Results:** Six months after administration of triamcinolone hexacetonide to the right knee, the patient is asymptomatic with complete clinical and ultrasound remission.

**Conclusion:** Although MRI confirmed the diagnosis of Reicheľs syndrome, due to the age of the patient and clinical and ultrasound findings we decided to treat the patient according to the guidelines for JIA. Treatment of the patient was successful without surgical intervention. Imaging methods can be helpful in differential diagnosis of JIA, however, findings might sometimes be confusing or misleading.

**Disclosure of Interest**

None Declared

### B24 STERNOCLAVICULAR JOINT INVOLVEMENT IN A CHILD WITH OLIGOARTICULAR JUVENILE IDIOPATHIC ARTHRITIS

#### Zahide Ekici Tekin^1^, Gulcin Otar Yener^1^, Nuran Sabir^2^, Selcuk Yuksel^1^

##### ^1^Department of Pediatric of Rheumatology, Pamukkale University School of Medicine; ^2^Department of Radiology, Pamukkale University School of Medicine, Denizli, Turkey

###### **Correspondence:** Selcuk Yuksel

**Introduction:** Although the sternoclavicular joint (SCJ) is a real synovial articulation that can be affected actively during systemic inflammatory disease such as juvenile or adult rheumatoid arthritis (RA), there were no reports of SCJ arthritis in juvenile idiopathic arthritis (JIA).

**Objectives:** In this article, we report a case of a boy who is newly diagnosed with oligoarticular JIA with unexpected joint involvement.

**Methods:** Case Report

**Results:** A formerly healthy 11-year-old boy with a month history of left ankle swelling and pain with morning stiffness was admitted to our clinic with SCJ pain and swelling for one week.

He reported no hospitalization and regular use of medication in his previous medical history.

Family history was not remarkable for rheumatological disease.

At presentation, the child had pain and limitation of movement of the left ankle. The SCJ was warm, effused and painful when pressure was applied. There was no enthesitis or complaints in other peripheral joints. The other organ systems were healthy upon physical examination.

Laboratory evaluation revealed significantly elevated erythrocyte sedimentation rate and C-reactive protein levels, and an ANA test was positive. According to the physical, laboratory and imaging evaluations, and bone marrow aspiration, there was no evidence for malignancy and infectious disorders.

After the evaluation of clinical and radiological findings, the child was diagnosed with oligoarticular JIA without uveitis and was started on 10 mg of weekly subcutaneous methotrexate in addition to oral prednisolone 2 mg/kg daily. Prednisolone was tapered and ceased within 4 weeks.

Two weeks after stopping steroids, despide efficient methotrexate treatment the patient presented at our department with re-exacerbation of pain and swelling in the SCJ. Acute phase reactants were risen again and the MRI of the left SCJ was consistent with marked bone marrow edema, joint effusion, synovial thickening and bone erosion. Meanwhile etanercept (0.8 mg/kg/week subcutaneous) was added. The responses to treatment in the third and sixth month were optimal according to the American College of Rheumatology (ACR) Paediatric 30/70. It was observed that the patient had significant healing after a year without any adverse reaction to treatment.

We report a case study of a boy with oligoarticular JIA who presented with arthritis of unexpected joint involvement. Oligoarticular JIA is the most prevalent chronic inflammatory arthritis of childhood. SCJ involvement in JIA has not been reported yet. However, symptomatic SCJ involvements in adult RA were reported at varying rates. Recently, Rodríguez-Henríquez et al. reported that 15% of RA patients had synovitis in the SCJ according to ultrasound. If the patient has no complaint, SCJ involvement might be missed upon routine rheumatological examination.

**Conclusion:** We concluded that SCJ involvement in JIA is very rare, but it may actively participate in the inflammatory process, as do other peripheral synovial joints. Careful clinical examination of the musculoskeletal system is essential for early diagnosis, and advanced radiological tools simplify the evaluation of joints in early phases effectively. In addition, biologic agents particularly seem to be more useful in early phase. Informed consent for publication had been obtained from the parent.

**Disclosure of Interest**

None Declared

## Macrophage activation syndrome

### B25 PARVOVIRUS B19 INDUCED MACROPHAGE ACTIVATION SYNDROME IN A CHILD WITH JUVENILE PSORIATIC ARTHRITIS UNDER SULPHASALAZINE TREATMENT

#### Nuray Aktay Ayaz, Ayşe Zopçuk, Gül Karadağ

##### Pediatric Rheumatology, Sağlık Bilimleri University KSSEAH, Istanbul, Turkey

###### **Correspondence:** Nuray Aktay Ayaz

**Introduction:** Juvenile idiopathic arthritis (JIA) is the most common chronic rheumatic disease in childhood, macrophage activation syndrome (MAS) is a rare and potentially fatal complication of JIA. It can be triggered by drugs, malignancies, infections, and rheumatic diseases.

**Objectives:** Herein, we describe Parvovirus B19 Induced MAS in a child with Juvenile Psoriatic Arthritis under sulphasalazine (SAZ) treatment

**Methods:** A 14 year old boy with a 3 years diagnosis of JPsA (arthritis together with a psoriatic rash, dactylitis, nail pitting and psoriasis in a first-degree relative) was SAZ, because of unresponsive to NSAID, steroids, intraarticular steroids, oral and subcutaneous methotrexate and leflunomide treatments. During the third week of SAZ, the patient complained of fever, fatigue, nausea, vomiting, rash, diffuculty in breathing. In his physical examination, the general condition was poor, tachypneic, dyspneic, body temperature: 39.4 ° C, pulse rate: 120 min, respiration rate: 25 / min, blood pressure: 160/80 mm/Hg. An indurated, confluent erythema over the cheeks(‘’slapped-cheek” appearence) and a symmetric maculopapuler rash on his arms, legs and trunk, sparing the palms and soles were detected. There was petechia on hard palate. Respiration and heart sounds were normal. The liver and spleen was not palpable. In the laboratory review**;** hemoglobin: 12.1 g/dL, hematocrit: 36.3%, leukocyte count: 21 000/mm3, eosinophil: 1590/mm3, platelet count: 108 000/mm3, C-reactive protein (CRP): 8.06 mg/dL (n=<0.79), erythrocyte sedimentation rate (ESR): 1 mm/1st hour, procalcitonin: 0.59 ng/mL, BUN: 8 mg/dL, creatinin: 0.52 mg/dL, AST: 694 U/L, ALT: 749 U/L, gamma glutamyl transferase (GGT): 143 IU/L, lactate dehydrogenase (LDH):851 IU/L, total bilirubin: 3.9 mg/dl, direct bilirubin: 3.1 mg/dl, albumin: 2.5 g/dl, cholesterol: 89 mg/dL, triglyceride: 178 mg/dL, HDL cholesterol: 13 mg/dL, LDL cholesterol: 57 mg/dL, fibrinogen: 159 mg/dL, D-dimer: 2.89 mg/L and ferritin: 840 ng/mL. Eosinophilia, atypical lymphocytes and monocytes were observed in peripheral blood smear. Abdominal ultrasonography revealed splenomegaly and liver size was at the upper border. The serum was positive for anti-parvovirus B19 IgM and negative for anti-parvovirus B19 IgG. Echocardiography was normal. Bone marrow aspiration showed an increased number of macrophages with prominent hemophagocytosis. From these findings, we considered that he had sulfasalazine-induced MAS associated with possible active Parvovirus B19 infection.

**Results:** Treatment with sulfasalazine was discontinued. He was treated by plasmapheresis, methylprednisolone pulse therapy and intravenous immunoglobulin (IVIG) therapy. Thereafter, oral prednisolone was tapered with dramatic improvement of the clinical symptoms and laboratory findings.

**Conclusion:** The present case demonstrated that combining steroid, IVIG and plasmapheresis is an effective therapeutic strategy for MAS, complicated by hypercytokinemia, in patients with juvenile idipathic arthritis

**Disclosure of Interest**

None Declared

### B26 CASE SERIES OF MACROPHAGE ACTIVATION SYNDROMES OCCURRING IN A DEPARTMENT OF PEDIATRIC RHEUMATOLOGY

#### Laura Buchtala, Frank Weller-Heinemann, Hans-Iko Huppertz

##### Pediatric Rheumatology, Klinikum Bremen-Mitte, Bremen, Germany

###### **Correspondence:** Laura Buchtala

**Introduction:** Macrophage activation syndrome (MAS) is a potentially life-threatening condition featuring fever and organ failure. Its pathogenesis is poorly understood. It occurs as a complication of severe inflammatory diseases and/ or is triggered by infections, particularly due to Epstein-Barr virus. Diagnosis in patients with systemic juvenile arthritis (sJIA) is based on the classification criteria published by Ravelli et al. in 2005, and edited in 2016.

**Objectives:** We performed an analysis of cases of MAS, using the Ravelli criteria.

**Methods:** We evaluated 13 episodes of MAS treated in our Department of Pediatric Rheumatology over a period of 12 years.

**Results:** There were no fatal outcomes. Five girls and five boys aged 6,3 – 16,3 years were affected, three patients showed two episodes of MAS. Of the 10 episodes in patients with sJIA, five began at the time of diagnosis, or immediately afterwards. One case occurred in a patient with suspected sJIA (without arthritis), and two were associated with infections. The Ravelli criteria were met in eight cases. In another three cases the criteria valid at the time (prior to 2016) were fulfilled. Two cases were interpreted as MAS without fulfilling the criteria.

There was considerable variation in laboratory findings and clinical courses among the patients. The cases occurring under therapy with tocilizumab showed milder clinical symptoms and lower ferritin concentrations. The episodes occurring under high-dose steroid therapy tended to have fewer abnormal laboratory findings, other than elevated ferritin.

**Conclusion:** The current diagnostic criteria for MAS in patients with sJIA do not encompass well patients treated with biological medications. Therefore they should be reevaluated.

**Disclosure of Interest**

None Declared

### B27 HIGH DOSE ANAKINRA FOR MACROPHAGE ACTIVATION SYNDROME (MAS) IN INFANT KAWASAKI DISEASE. A CASE REPORT

#### Anne Estmann Christensen

##### Hans Christian Andersen Children Hospital, Odense University Hospital, Odense , Denmark

**Introduction:** A 12-week-old boy presenting with atypical Kawasaki disease (KD) complicated with macrophage activation syndrome (MAS) is reported.

**Objectives:** -

**Methods:** The infant presented with cerebral irritability, pain, takypnea and vomiting during 10 days. He had no history of fever or rash. Ad admittance mild pleiocytosis (37 x 10E6/L) in the cerebrospinal fluid (CSF) was found. Antibiotic treatment was initiated without any clinical response. No bacteria or viral DNA was detected in CSF, blood, urine, or airways. Pericardial effusion was seen on echocardiogram in addition to severe dilatation of coronary arteries (LAD: 4.2 mm Z-score 12,68; RCA: 4,2 mm Z-score 9,3). Treatment with intravenous Immunoglobulin (IVIG) and aspirin was initiated for KD without any response. Addition of infliximab reduced the pericardial effusion but otherwise the condition was unchanged. High dose methylprednisolone was initiated followed by prednisolone but 5 days later criteria for MAS were fulfilled. High dose Anakinra was started resulting in dramatically improvement in his clinical condition.

**Results:**

**Table:** 

Time point/treatment given Variable (Normal Value)	At diagnosis IVIG 2 g/kg	After 3 days Remsina 5 mg/kg	After 9 days IVIG 2g/kg	After 11 days Methylpred. 2 doses + prednisolone	After 16 days Anakinra 8 mg/kg	After 3 weeks	After 7 weeks
Haemoglobin, mmol/l (6-8,4)	5,9	5,5	5,9	5,8	6,4	6,5	7
White cell count x 10^9^/l (3-14)	36	28,1	38,7	33,1	45,2	35,5	20,1
Platelets x 10^9^/l (175-565)	1360	699	1466	1618	1988	1409	924
Ferritin, μg/l (22-400)	837	823	731	724	3349	481	88
ALT, U/l (5-45)	101	75	17	23	457	136	40
CRP mg/l (<6)	116	116	117	100		55	1,1
Fibrinogen mmol/l (5,5-11,5)					12	24,6	8,4
Triglyceride mmol/l (<2)	1,0				5,6	2,6	2,5
Pericardial effusion (yes/no)	yes	yes	(no)			no	no

**Conclusion:** This case shows benefit of interleukin-1 receptor blockade for refractory kawasaki disease complicated by MAS in an infant.

**Trial registration identifying number:** Written informed consent for publication is obtain from the parents.

**Disclosure of Interest**

None Declared

### B28 MACROPHAGE ACTIVATION SYNDROME COMPLICATING SYSTEMIC JUVENILE IDIOPATHIC ARTHRITIS ASSOCIATED WITH ACUTE HEPATITITS A INFECTION

#### Ljubinka Damjanovska-Krstikj

##### Rheumatology Clinic Skopje, Skopje, Macedonia, The Former Yugoslav Republic Of

**Introduction:** Macrophage activation syndrome (MAS) is potentially life-threatening complication of systemic inflammatory disorders, which occurs most commonly in systemic juvenile idiopathic arthritis (sJIA), its adult equivalent, as well as in with other autoimmune or autoinflammatory conditions. It is being reported with increased frequency. It is potentially fatal condition which can results with death of 8% of patients. MAS is characterized by an over-whelming inflammatory reaction due to an uncontrolled and dysfunctional immune response involving the continual activation and expansion of T lymphocytes and macrophages, which results in massive hypersecretion of pro-inflammatory cytokine.

**Objectives:** We are presenting the case of 22 year old patient who is suffering from sJIA since he was 4 years old, who developed MAS because of acute hepatitis A infection.

**Methods:** The patient was prone to different infections like cutaneous, upper respiratory tract, urinary infections because of the long term immunosuppressive treatment, glucocorticoid use and insufficient water supply in his village. He was also treated for septic sacroiliitis, have chronic Q fever and many other infective complications to his sJIA. He presented with 2 weeks history of high fever 39,5 degrees Celsius, salmon colored rash on his back coinciding with the fever, arthralgia and arthritis of 12 joints, dark colored urine, low appetite and exhaustion. Few days later he had yellow colored skin and sclera. The diagnostic tests for hepatitis A IgM antibodies were positive. He also had positive IgM and IgG antibodies against Ebstein Barr Virus.

The laboratory results are presented in Table 1. He fulfilled EULAR classification criteria for

sJIA associated MAS from 2016.

The other laboratory results were as follow: ALT 1990 U/L, LDH >2234 U/L , GGT 1000 U/L, Albumins 25 , total bilirubin 108 (ref 20 Mmol/L), direct bilirubin 90 Mmol/L, APTT shortened 22 sec. D-dimers >5000 ng/ml CRP 60 mg/L.

The patient was immediately put on high-dose 1mg/kg glucocorticosteroids and immunosuppressants- we used 3 mg/kg of cyclosporine. MAS was successfully treated and his sJIA is now in 6 months remission with only 50 mg of cyclosporine daily and low dose prednisone.

**Results:**

**Conclusion:** Fulminant viral hepatitis may cause MAS, with hepatitis A virus being more frequently associated with MAS than the other hepatotrope viruses. Fifteen cases (including children) have been described, mainly in Asia: three of these patients also had a concurrent rheumatological disease including sJIA. Hepatitis A can mimic MAS as well, but high awareness and immediate immunosuppressive treatment could prevent the potentially fatal consequences of MAS in patients with sJIA. Informed consetn to publish had been obtained from the patient.

**Disclosure of Interest**

None Declared

**Table 1 (abstract B28).** See text for description.

ESR mmHg	Hemoglobin g/L	Platelets No	AST U/L	Ferritin ng/ml	Triglycerides U/L
68	94 g/L	60 x10/9 L	288	3500	2,8
36	71 g/L	20 x10/9 L	296	3946	4,8
After the treatment with 3mg/kg of cyclosporine					
20	130 g/L	241	23	<500	2,8

### B29 LIFE-THREATENING MAS WITH NEUROLOGICAL INVOLVEMENT RESPONDING TO VERY HIGH DOSE IV ANAKINRA

#### Akhila Kavirayani, Seilesh Kadambari, Robin Basu-Roy, Shelley Segal, Amrana Qureshi, Kathryn Bailey

##### Oxford University Hospitals NHS Trust, UK, Oxford, UK

###### **Correspondence:** Akhila Kavirayani

**Introduction:** Severe MAS(Macrophage Activation Syndrome) with neurological,renal & bleeding complications is challenging to manage

**Objectives:** To describe life-threatening MAS responding to high dose iv Anakinra

**Methods:** A 9 year old Caucasian girl presented with high fevers for 3 weeks,severe abdominal/leg pains,no rashes & no infectious focus.Past/family history non-contributory.She underwent an appendicectomy(normal appendix & abdominal MRI).Fevers continued with high nflammatory markers(ferritin 5000)& rising Creatinine.Upon transfer to our centre, she was febrile/delirious & collapsed with rapid deterioration & multi organ dysfunction. She was ventilated,needing inotropes for profound hypotension/poor cardiac output(with resultant ischaemic necrotic patches on limbs).Severe renal failure needed haemofiltration.Concurrent ferritin was 192549,CRP 180,ESR<1,Creatinine 257,falling Haemoglobin/platelets(necessitating transfusions),fibrinogen<0.3,Triglycerides 3.9,ALT 242,LDH 3218,prolonged PT/aPTT,bone marrow showed haemophagocytosis,all these confirming MAS/HLH.^1^

**Results:** She was given pulsed IV methyl Prednisolone & IV Anakinra 0.5mg/kg/hour, IV Immunoglobulin,antibiotics,antivirals. Dexamethasone was added with Candida/PCP prophylaxis.MAS parameters slightly improved with ferritin plateauing at 35000.Inotropes were gradually discontinued but she remained on maximal ventilation & haemofiltration. Bleeding dyscrasias necessitated multiple platelet/cryoprecipitate transfusions. She developed unreactive pupils & clonus.A CT brain showed no focal findings. She was too unstable for a lumbar puncture. Cerebral Function Monitoring tracing was equivalent to a flat EEG.As for neuroHLH,immunosuppression was intensified and Dexamethasone was doubled^.2^ Simultaneously Anakinra was steeply escalated to 2mg/kg/hour(48mg/kg/day) for 3 days, the rationale for this being the role of IL1 blockade in reducing mortality in sepsis associated MAS.^3^ In addition, IL1 inhibitors are highly neuroprotective experimentally, inhibiting systemic acute phase response to stroke and have been shown to be safe in acute Sub Arachnoid haemorrhage, reducing peripheral inflammation with proposed neuroprotection.^4^

1 dose of Etoposide as per creatinine clearance was also administered. Within 40 hours of this regimen,clear signs of life were obvious. A 2nd pulse of Methyl prednisolone was administered whilst weaning Anakinra. Haemofiltration was discontinued within 3 days & ventilation within a week. Dexamethasone was changed to oral Prednisolone & Ciclosporine added. Her hospital stay was complicated by respiratory aspergillosis & bacterial line infection,both appropriately treated. All initial infectious serology was negative as was autoimmune serology, malignancy screen & HLH genetics. Due to knee synovitis on followup,she is being treated as presumed systemic JIA with MAS.Recently gene sequencing has shown an intronic variation for TRAPS,of unknown significance. Remarkably she is neurologically intact,apart from psychology support for longstanding anxiety & is on daily Anakinra, Ciclosporine & weaning Prednisolone.

**Conclusion:** Conclusion:

Severe MAS/HLH requires close collaborative working between multiple specialties including paediatric intensivists, rheumatologists, haematologists and infectious disease specialists

High dose IV Anakinra contributed to reversing severe neurological obtundation in our patient & is a therapeutic option in neuroHLH, given its potential neuroprotective role

**Trial registration identifying number:**

**References**

1. Ravelli A, Minoia F, Davì S, Horne A, Bovis F, Pistorio A et al. 2016 Classification Criteria for Macrophage Activation Syndrome Complicating Systemic Juvenile Idiopathic Arthritis: A European League Against Rheumatism/American College of Rheumatology/Paediatric Rheumatology International Trials Organisation Collaborat. Arthritis & Rheumatology. 2016;68(3):566-576

2. Horne A, Wickström R, Jordan M, Yeh E, Naqvi A, Henter J et al. How to Treat Involvement of the Central Nervous System in Hemophagocytic Lymphohistiocytosis?. Current Treatment Options in Neurology. 2017;19(1)

3. Shakoory B, Carcillo J, Chatham W, Amdur R, Zhao H, Dinarello C et al. Interleukin-1 Receptor Blockade Is Associated With Reduced Mortality in Sepsis Patients With Features of Macrophage Activation Syndrome. Critical Care Medicine. 2016;44(2):275-281

4. Galea J, Ogungbenro K, Hulme S, Patel H, Scarth S, Hoadley M et al. Reduction of inflammation after administration of interleukin-1 receptor antagonist following aneurysmal subarachnoid hemorrhage: results of the Subcutaneous Interleukin-1Ra in SAH (SCIL-SAH) study. Journal of Neurosurgery. 2018;128(2):515-523.

Consent

Mother has given consent recorded in medical notes

**Disclosure of Interest**

None Declared

### B30 MACROPHAGE ACTIVATION SYNDROME IN A CHILD WITH JUVENILE DERMATOMYOSITIS- CLINICAL CASE

#### Katya Temelkova, Stefan Stefanov, Margarita Ganeva, Dimitrina Mihaylova, Albena Telcharova-Mihaylovska, Vanya Kaleva

##### Children University Hospital Sofia, Sofia, Bulgaria

###### **Correspondence:** Katya Temelkova

**Introduction:** We report a clinical case of a 9-year old girl diagnosed with juvenile dermatomyositis, complicated with macrophage activation syndrome , probably triggered by Herpes simplex infection.

**Objectives:** Our objective is to describe the presentation of macrophage activation syndrome in a child with newly diagnosed juvenile dermatomyositis

**Methods:** Our patient is 9-year old girl who presented to a Pediatric Department with a 6-month history of abnormal gait and lower limbs pain, which appeared mainly in the evening and after physical activities. On admission she had polyarthritis, maculopapular rash over the cheeks and erythema over the nasal tip. The lab studies revealed leucopenia with lymphopenia, mild anemia, slightly elevated ESR, positive ANA titer and negative anti-dsDNA. Treatment with NSAIDs and Methotrexate was prescribed, but the parents decided to try alternative therapy - homeopathy, and refused the treatment.

In the following months the child deteriorated with progressive muscle weakness, difficulty walking, skin rash, alopecia, persistent fever, progressive weight loss and abdominal pain. On examination there was marked symmetrical proximal muscle weakness and the girl was unable to perform the usual daily activities on her own. Characteristic cutaneous changes were observed - heliotrope discoloration of the eyelids with periorbital edema and a malar rash.She had also hyperpigmented lesions around the elbows, knees and ankles, Gottron's papules and aphtous stomatitis. Due to the clinical signs a diagnosis of JDM was suspected,but was inconclusive due to the normal lab studies with normal creatine phosphokinase levels, normal inflammatory and immunology markers and the technical inability to perform electromyography.Finally the diagnosis was supported by magnetic resonance imaging of the thigh muscles revealing the characteristic pattern of muscle involvement in JDM. What made the case more perplexing were the additional overlap clinical signs of subtle arthritis with flexion contractures at the elbows and knees, alopecia, butterfly-like rash and aphtous stomatitis. For the latter, a positive result for HSV infection was received . In addition we observed jaundice with enlarged liver and spleen, lab studies revealed hepatitis and cholestasis, so an exclusion of underlying reasons for the development of hepatitis (viruses, autoimmune hepatitis, drug induced, metabolic diseases) was required.

Overall, a diagnosis of JDM complicated with MAS (the lab studies revealed pancytopenia, normal ESR, elevated liver enzymes, increased bilirubin levels, elevated LDH levels, decreased levels of albumin and total protein, hypertryglyceridemia, hyperferritinemia) was established.

**Results:** Pulse therapy with high‐dose intravenous methylprednisolone and intravenous immunoglobulin was initiated. After almost two months of hospital stay, the girl was discharged with great improvement in the range of motions and normal laboratory markers

**Conclusion:** MAS is a rare and life-threatening complication of JDM, most commonly triggered by infections.In our case the triggering factor might be the HSV infection .JDM can present with inconclusive lab studies and our challenge was also associated with the differential diagnosis of liver involvement in MAS. Informed consent to publish had been obtained from the parent.

**Disclosure of Interest**

None Declared

### B31 MACROPHAGE ACTIVATION SYNDROME INITIAL DIAGNOSIS: THE OUTCOME

#### Olga Vougiouka, Eleni-Maria Papatesta, Maria Zairi, Maria Tsolia

##### Second department of pediatrics, P&A.Kyriakou Childrens Hospital, Athens, Greece

###### **Correspondence:** Olga Vougiouka

**Introduction:** Macrophage Activation Syndrome (MAS) is a critical, overinflammatory condition complicating Systemic onset Juvenile Idiopathic Arthritis (SoJIA), Systemic Lupus Erythematosus (SLE) or it can be triggered by other factors such as infections, malignancies or drugs.

**Objectives:** The final diagnosis and outcome of children diagnosed with MAS at a tertiary referral center

**Methods:** Patients who were hospitalized from 1990-2017 at a teaching Athens Pediatric Hospital Department and met the HLH 2004 or the EULAR/ACR/PRINTO 2016 criteria for MAS (ferritin >684 ng/ml and any 2 of the following platelet count ≤181 x 10^9^/litre, Aspartate aminotransferase >48 units/litre, triglycerides > 156 mg/dl, fibrinogen ≤360mg/dl) or exhibited macrophage hemophagocytosis in bone marrow biopsy specimens.

**Results:** 11 children were included in our study with a female predominance (6 girls and 5 boys). The mean age at diagnosis was 8.5 years (range:3.5–16years). Only one patient had a diagnosis of SoJIA, which was recognized several days before the diagnosis of MAS. The mean duration of their symptoms prior to diagnosis was 20 days (range: 9-44 days), while the mean duration of hospitalization prior to diagnosis was 8 days (range: 1-24 days). All patients presented with fever, 7 presented with rash and joint paint and 2 with arthritis. Regarding the laboratory investigation which correlates with the MAS criteria, mean ferritin was 16.714 ng/ml (range 150-44.000), mean platelet count was 170 x 10^9^/ liter (range 48-459 x 10^9^/liter), aspartate aminotransferase was 172 U/l (range 24-621), mean triglycerides 260 mg/dl (range 79-700) and mean fibrinogen 309 mg/dl (range 70-577). 7 patients revealed macrophage hemophagocytosis in bone marrow biopsy. 4 patients were admitted to the Intensive care Unit. Furthermore, 6 patients developed neurologic complications. All patients received systemic corticosteroids (prednisolone, pulses of methylprednisolone or dexamethasone), 4 patients were treated with biologic agents, 4 patients with etoposide, whereas 5 patiens initially were treated with intravenous immunoglobulin. All patients survived and clinically recovered. One patient presented with relapse of MAS, 1 year after the first episode. 9 patients were diagnosed with SoJIA and 2 with possible SoJIA, with mean time of follow up: 62.4 months (range 4-146 months).

**Conclusion:** High suspicion with subtle clinical or laboratory changes in a protracted febrile patient is necessary for prompt diagnosis and management of MAS. Validated classification criteria have been published in 2016, but treatment guidelines have only developed for primary HLH. Our patients were treated with different therapeutic protocols, according to the severity of their clinical status and their response. Although biologics have rarely implicated as triggering factors in single case reports, in our case, as in others, they worked perfectly well. Prompt diagnosis, judicious use of corticosteroids, immunosuppressants, biologics and supportive therapy, ends to a favorable outcome even in this life threatening syndrome.

**Disclosure of Interest**

None Declared

## Miscellaneous rheumatic diseases

### B32 PEDIATRIC MESENTERIC PANNICULITIS: THREE CASES

#### Ceyhun Acari^1^, Hatice Adiguzel Dundar^1^, Alper Soylu^2^, Erdener Ozer^3^, Erbil Unsal^1^

##### ^1^Department of Pediatrics, Pediatric Rheumatology; ^2^Department of Pediatrics, Pediatric Nephrology; ^3^Department of Pathology, Dokuz Eylul University Faculty of Medicine, Izmir, Turkey

###### **Correspondence:** Ceyhun Acari

**Introduction:** Mesenteric panniculitis is a rare inflammatory process leading to fibrosis. It involves the mesenteric adipose tissue with an unknown etiology. Malignancy, tuberculosis, trauma, drugs, and surgery are considered as the predisposing factors. Three cases with different etiology are presented.

**Objectives:** In this article three pediatric cases of mesenteric panniculitis with different etiologies were presented.

**Methods:** The files of the three MP cases were reviewed and clinical, laboratory, imaging results, treatment and outcomes were assessed.

**Results:** Case 1:

A five-month-old girl was hospitalized following her consultation to the emergency department with discomfort and vomiting. She had dehydration with abdominal distention with no bowel sounds. Computerized tomography revealed generalize fluid in the abdominal cavity. Exploratory laparotomy demonstrated intense lipoid deposits in the mesenteric of the small and large intestines and the appendix. Histopathological examination showed necrotic foci, suppurative inflammation and fibrinous exudate of the omentum, compatible with mesenteric panniculitis.

Case 2:

A ten-year-old boy was consulted to emergency department with abdominal pain and nausea for two weeks. There was rebound tenderness in the right lower quadrant. The abdominal ultrasonography revealed appendicitis and the patient was operated. The omentum tissue was compatible with mesenteric panniculitis, and lymphoid hyperplasia with fibrolipomatous appearance was detected in the histopathologic investigation. The patient recovered following the operation. Consent had been obtained from the parents.

Case 3:

A fifteen-year-old girl with RF negative polyarticular JIA was in remission for three years. She had a progressive worsening of general condition with vomiting. Bilateral hydronephrosis was detected in abdominal ultrasonography, and a further MRI revealed retroperitoneal fibrosis surrounding perirenal tissue and ureters. Histopathologic examination, adipose tissue necrosis and septal fibrosis, but there was no amyloid deposition. IgG4 related disease was excluded. She was diagnosed as mesenteric panniculitis. Initial therapy with high dose steroids was successful. The maintenance therapy was provided with methotrexate and azathioprine. Tocilizumab therapy was started due to recurrence. She is still under remission for the last year.

**Conclusion:** Mesenteric panniculitis is characterized by chronic nonspecific inflammation of the mesenteric adipose tissue of the small intestine and colon. It is reported as a very rare condition in children. Also, mesenteric panniculitis was characterized with different findings in these three cases and came up in an early age as 5-month old. It should be in the differential diagnosis of children with acute abdomen. Consent had been obtained from the parents.

**Disclosure of Interest**

None Declared

### B33 PEDIATRIC AUTOIMMUNE BULLOUS DISORDERS DIFFERENTIAL DIAGNOSIS IN RHEUMATOLOGY

#### Marinabiondina Amico^1^, Francesca Orlando^2^, Roberta Naddei^2^, Carolina Porfito^2^, Teresa Lastella^2^, Maria Alessio^2^

##### ^1^Mother and Child Department; ^2^Mother and Child Departement, Federico II University of Naples, Naples, Italy

###### **Correspondence:** Marinabiondina Amico

**Introduction:** Autoimmune blistering diseases (AIBDs) are a group of disorders mostly affecting the skin and mucous membranes. Occasionally, other organ systems may be involved, such as eye.

**Objectives:** We present two cases of severe childhood AIBDs referred to our Department with diagnosis of Rheumatic diseases.

**Methods:** Case report

**Results:** A 9-year-old girl with three years history of conjunctivitis with conjunctival scarring evolved in ocular sicca syndrome, was referred to our Department with diagnosis of Sjögren's syndrome, treated with high doses of steroids. Clinical history revealed also mucocutaneous involvement, with oral and anovulvar ulceration. Laboratory data showed anemia, elevated serum levels of ERS and CPR, normal serum levels of autoantibodies (ANA, anti-dsDNA, ENA). Biopsies of buccal mucosa, vulval skin, parotid and colon were performed. The histopathological study showed an infiltrate of lymphocytes, plasma cells and eosinophils; the direct immunofluorescence (DIF) showed linear deposition of IgG and C3 at the mucosal/submucosal junction. These findings were considered consistent with Sjögren's syndrome, and inflammatory bowel disease. Six months later the girl was admitted to our Department: the clinical examination revealed bilateral fornix foreshortening, symblepharon, entropion, corneal scarring, blisters around the nails, oral ulcerations; the vulval mucosa was completely denuded and adhesions were developing between the anterior aspects of the labia majora. The airway examination showed bilateral choanal atresia and laryngeal stenosis due to scarring. She started corticosteroid and she underwent surgery to remove cicatricial lesions of eyes, vulval area and choanae. Immunofluorescence findings of the surgical biopsies reveals the presence of linear homogeneous deposits of IgG and C3 along the epithelial basement membrane zone, therefore diagnosis of cicatricial pemphigoid (CP) was made. Ciclosporine was introduced without improvement, therefore after one month on the therapy was shifted to tacrolimus with initial response but worsening of ocular disease after two months. Many therapeutic trials were performed (thalidomide, cyclophosphamide and etanercept) without improvement. The patient was admitted to Intensive Care Unit, she underwent tracheostomy and died after 4 months. The second case is a 15-year-old girl with recent onset of oral e genital ulcers, referred to our Unit with suspect of Behçet’s disease. She was on prednisone and cyclosporine. Laboratory data showed raised inflammatory markers and ANA positivity. At the admission the clinical examination revealed facies cushingoide, strie rubrae, multiple eruptions and blisters was found in her face, trunk, genital area and proximal limbs. Skin biopsy showed intraepidermal suprabasal acantholysis with infiltrate of lymphocytes and eosinophilis. Direct immunofluerescence showed IgG deposits at the surface of epidermal keratinocytes and confirmed the diagnosis of pemphigus vulgaris (PV). Oral prednisone combined with mycophenolate mofetil (MMF) resulted in complete clinical remission off therapy.

**Conclusion:** Cicatricial pemphigoid and pemphigus vulgaris are autoimmune disorders, rare in pediatric age. Both could involve skin and eyes. Immunohistopathologic evaluation with direct immunofluorescence are essential for diagnosis and repeat biopsies may be useful in patients with atypical disease courses. Corticosteroids and immunosuppressants were the mainstay of treatment. The inclusion of these disorders in differential diagnosis of rheumathologic diseases with dermatologic and ocular manifestations is important to start an appropriate and early treatment.  Informed consent to publish had been obtained.

**Disclosure of Interest**

None Declared

### B34 CONCOMITANT JUVENILE MYASTHENIA GRAVIS AND POLYMYOSITIS IN A CHILD – A CASE REPORT

#### Ana C. Castro^1^, Marta Veríssimo^1^, Andreia Martins^1^, Marta Cabral^2^, Catarina Luís^3^, Helena Loureiro^1^

##### ^1^Paediatrics; ^2^Paediatric Rheumatology; ^3^Paediatric Neurology, Hospital Prof Doutor Fernando Fonseca, EPE, Amadora, Portugal

###### **Correspondence:** Ana C. Castro

**Introduction:** Causes of weakness in children include disorders that involve any component of the lower motor neuron: anterior horn cell, peripheral nerve, neuromuscular junction or muscle; sometimes differential diagnosis can be challenging.

**Objectives:** Report of a patient with a rare combination of two autoimmune diseases both contributing to muscle weakness.

**Methods:** A 7-year-old boy presented to the emergency department with a three-week evolution of left knee pain and upper and lower limbs weakness. Parents denied previous episodes of muscle weakness, frequent falls or choking, fever or previous infections. Personal and family background were irrelevant.

Physical examination showed myopathic gait with axial and appendicular hypotonia, predominant proximal muscle weakness with normal deep tendon reflexes. No skin rash, dyspnea or dysphonia were noted.

During hospital stay clinical worsening was noted with reduced muscle strength and fluctuation of symptoms such as ptosis and episodes of dysphonia. Childhood myositis assessment scale (CMAS) total score was 8/52.

**Results:** Blood tests revealed slightly elevated creatine kinase (534,15UI/L) and myoglobin (209 UI/L); aldolase, calcium and phosphorus were normal. ANAs were 1/320 with positive anti-dsDNA antibodies. Anti-PL 12 and anti-acetylcholine receptors (AchR) antibodies were both positive.

Reactive C-Protein, erythrocyte sedimentation rate, RNA synthetase antibodies and other immunologic markers were normal/negative. Investigation for infectious and metabolic diseases was negative. Cardiology, Ophtalmology and Ear, Nose and Throat evaluations were normal.

Electromyography was suggestive of primary muscle disease. Repetitive nerve stimulation was not performed due to non-compliance. Chest CT scan was normal and lower limbs MRI showed no evidence of myositis.

Biopsy from lateral vastus muscle revealed T-cell infiltrates with muscle fiber necrosis and healthy muscle fibers expressing MHC class I antigen, all typical pathological features of polymyositis.

The patient started Pyridostigmine (1mg/Kg/dose qid) and intravenous Immunoglobulin (1g/Kg in two doses). He additionally started a physiotherapy program. Partial clinical improvement was noted 48 hours after initial dose with a CMAS score of 21/52.

After biopsy results came to knowledge, he was started on Methylprednisolone (5 pulses of 30mg/Kg) followed by Prednisolone (2mg/Kg/d) and Methotrexate (17mg/m^2^/week).

At 3-weeks follow-up he recovered to almost normal strength with a CMAS total score of 44/52.

**Conclusion:** Polymyositis and Myasthenia Gravis are rare autoimmune disorders of childhood affecting muscle and neuromuscular junction, and constitute a great diagnostic challenge.

The diagnostic approach to our patient was difficult owing to clinical overlap of both autoimmune disorders and an absence of typical features: sudden onset of weakness with rapid progression in the absence of cutaneous features, ptosis, diplopia, bulbar symptoms at presentation. Serologic testing, biopsy and clinical improvement with a trial of Pyridostigmine and immunosuppressants allowed confirmation of both diagnoses and appropriate management of both conditions.

Written informed consent for publication of his clinical details and/or clinical images was obtained from the patient’s parent.

**Disclosure of Interest**

None Declared

### B35 LEUKOCYTOCLASTIC VASCULITIS AS THE INITIAL CLINICAL MANIFESTATION OF PRIMARY SJÖGREN'S SYNDROME IN A 13-YEAR-OLD FEMALE PATIENT: CASE REPORT

#### Velka A. Cortez López, Maria T. Braña, Edith A. Benitez Vazquez, Merari E. Gomez Cortes, Luis Aparicio Vera, Sofia B. Osorio Sagrero, Enrique Faugier Fuentes, Maria R. Maldonado

##### Reumatologia pediatrica, Hospital Infantil de México, México Distrito Federal, Mexico

###### **Correspondence:** Velka A. Cortez López

**Introduction:** Sjögren's Syndrome (SS) is a chronic autoimmune disease characterized by inflammation of the lacrimal and salivary glands, secondary to infiltration of lymphocytes and in the presence of auto antibodies (Anti-Ro and Anti-La). Primary SS occurs without association of other systemic autoimmune diseases, and is more often in women of medium to advanced age, being rare in childhood. It presents as dry mucosa surfaces of the mouth and eyes. In addition, 40% of the patients have extraglandular involvement, such as skin manifestations, musculoskeletal and neurological disorders. Systemic vasculitis manifestations of SS occur in approximately 5 to 10% of adult patients, however, the incidence decreases with age. Half of the individuals that develop vasculitis presents with a single episode, and histologically findings of leukocytoclastic vasculitis or necrotizing arteritis. These patients develop a more serious illness and have a worse prognosis.

**Objectives:** We describe a pediatric patient with leukocytoclastic vasculitis as the initial clinical manifestation of Sjögren's Syndrome.

**Methods:** Descriptive. Clinical case presentation.

**Results:** A 13 year old female, referred to the Pediatric Rheumatology Department, with a year of palpable purpuric lesions, in both lower extremities, limited to the knees, and associated with painful edema of the ankles. The manifestations became recurrent, being treated with low dose oral steroid for two months without remission. Subsequently the patient developed dry eye, with recurrent episodes of conjunctivitis and arthralgia in both ankles and knees. Laboratory finding include elevated ESR 43mm/hr, and FR, 37.1 Ul/ml, anti-SS-A (Ro) 198.74 RU/ml and anti-SS-B (La) 200 RU/ml, IgA 411 mg/dl and IgG 2410 mg/dl, IgG1 11600 mg/dl, ANA fine speckled 1:640. The rest of immunological and serology profile was negative.  Minor salivary gland biopsy reported chronic sialodenitis with inflammatory lymphoplasmocytic infiltrate and intralobular infiltrate around the ducts and the rest of the lobules, compatible with SS. Skin biopsy reported leukocytoclastic vasculitis. With this finding we integrated primary SS and we started high dose steroid for ten days and immunomodulation with hydroxychloroquine, in addition to sunscreen and eye lubricant. The patient responded to this treatment, with remission of cutaneous manifestations and improvement of ocular symptomatology.

**Conclusion:** We describe the case of a 13-year-old patient with diagnosis of primary SS, fulfilling the clinical, laboratory and histology ACR-SICCA 2012 criteria, having as primary manifestation leukocytoclastic vasculitis. Leukocytoclastic vasculitis is a very rare systemic manifestation of SS, reported in 5 to 10% of adults. There are no data of the prevalence of this vasculitis in pediatric population with SS, since the wide spectrum of cutaneous disease is yet to be described. Pediatric presentation of SS is different from that of the adults, making it a diagnosis challenge. It is important to acknowledge that leukocytoclastic vasculitis can be the initial manifestation of pediatric SS. A prompt identification assures an early treatment allowing an improvement in quality of life and preventing the development of secondary complications. Informed consent to publish had been obtained from the parents.

**Disclosure of Interest**

None Declared

### B36 FABRY’S DISEASE: A DIAGNOSTIC CHALLENGE. CASE REPORT

#### Adriana S. Diaz-Maldonado^1^, Sergio Rueda^2^, Luisa F. Imbachí- Yunda^3^

##### ^1^Pediatric Rheumatologist, Fundacion Homi - Care for Kids; ^2^Internal medicine - Rheumatologist, Universidad Militar Nueva Granada; ^3^pediatrIcian, Fundacion Homi, Bogota, Colombia

###### **Correspondence:** Adriana S. Diaz-Maldonado

**Introduction:** The Fabry’s disease (FD) is a lysosomal storage disease, X linked, that may affect both men and women. Mutations in alpha Gal A gene carry a partial or total deficiency of the Galactosidase A alpha enzyme with secondary globotriaosylceramide (Gb3) accumulation in the skin, eyes, kidney, heart, brain and peripheral nervous system. It generates clinical heterogeneous profiles with some specific features according to the age groups.

**Objectives:** To describe a Fabry’s disease pediatric clinical case confirmed whose clinical manifestation was the neuropathic pain.

**Methods:** A report about a Fabry’s disease case in a pediatric patient is submitted

**Results:** 12-year old male patient, with no pathological personal antecedents. Two-year evolution clinical profile showing fever episodes each 4 months without associated infectious symptoms with a duration of 3 to 5 days and spontaneous improvement. He has been admitted in hospital, infectious processes discarded. In addition, he presents burning pain episodes, described as “burning sensation” in soles and palms with an approximate 10-minute duration, which spontaneously or when dipping his hands in cold water get better, followed by a hand redness and then paleness, without cyanosis. These episodes occur each 2 to 3 months and are not related to fever. During the last 6 months, said episodes have been more frequent. No other symptoms. No alterations found when submitted to the physical exam. Routinely labs tests such as creatinine, ureic nitrogen, glomerular filtration rate and transaminases, normal. Urinalysis without microalbuminuria or proteinuria. Serology for cytomegalovirus, Epstein barr, toxoplasma and HIV, negative; 0 mm tuberculin. Medical imaging studies on abdomen and thorax with no alterations. Electromyography and nerve conduction studies on the four extremities, normal. Because of FD suspicion, alpha galatosidase A studies were performed in a drop of blood in filter paper, reporting in 0,48 nmol/ml/hour (reference for males with FD 0,0 – 03 nmol/ml/hour). Serum complement (C3, C4, Ch50), ferritin, albumin, immunoglobulin A, G, M, Normal. Immunoglobulin E elevated for his age. He was assessed by pediatric ophthalmology confirming the presence of *cornea verticillata.* Molecular study performed evidencing a GLA sequence analysis abnormal: exon 1, c.128delG, pGly43Alafs*78 previously reported in the database as a pathological mutation. LysoGB3 levels: 112 ng/ml (<0,8 ng/ml) by high-resolution liquid chromatography. Sent to genetics where family tree and counselling are carried out and starting a therapy of enzymatic replace with alpha-galactosidase beta each 2 weeks. Levels of enzymatic antibodies taken, negative.

**Conclusion:** FD is the second more frequent lysosomal storage disease because among its pathophysiological aspects, the abnormal Gb3 storage in multiple tissues endothelium, triggers structural alterations which alter the normal function thereof resulting in a large set of clinical manifestations. The neurological compromise, typical of an early damage of both the little somatic peripheral nervous fibers and the autonomic ones is one of the earliest observed symptoms predominantly in males. The neuropathic pain of the case presented was the only clinical manifestation, and it teaches us to highly suspect of the large variety of symptoms, which must be timely recognized by the clinician in order to make an early diagnosis and thus to initiate the enzymatic supply therapy to improve both the prognosis and the patient’ life quality. Informed consent to publish had been obtained from the parent.

**Disclosure of Interest**

None Declared

### B37 Withdrawn

### B38 ORBITAL MASS: THE PRESENTATION OF IGG4-RELATED DISEASE IN 12- YEAR- OLD CHILDREN

#### Vadood Javadi Parvaneh^1^, Reza Shiari^2^, Khosro Rahmani^2^

##### ^1^Pediatric Rheumatology; ^2^Shahid Beheshti University of Medical Sciences, Tehran, Iran, Islamic Republic Of

###### **Correspondence:** Vadood Javadi Parvaneh

**Introduction:** IgG4-related disease (IgG4-RD) is a wide spectrum of fibro-inflammatory diseases that affect many organ systems and characterized by storiform fibrosis, mild to moderate tumor-like eosinophile and significant IgG4- producing plasma cell infiltration in histopathology, and the high level of serum IgG4 concentration. Its pathophysiological mechanism is unclear; however, when untreated may lead to fibrosis and irreversible organ damage. This rare disease mostly has been described in middle-aged adults and more rarely in children. Enlargment of lacrimal and salivary glands, cervical lymphadenopathy, retro-orbital and other organ pseudotumors, pancreatitis, sclerosing cholangitis and mesenteritis,retroperitoneal fibrosis, pulmonary and aortic inflammatory disease, thyroiditis, and joint disease are some aspects of the disease.

**Objectives:** To report a boy with orbital mass with final diagnosis of IgG4-related disease

**Methods:** -

**Results: Case report**: Herein, a 12-year-old boy is reported which presented with orbital mass. He had high IgG4 serum level (175 mg/dl) and histopathologic manifestation of pseudotumor compatible with IgG4-RD. Investigation for other organ involvements as part of the IgG4-RD spectrum was normal. After resection of tumor and treatment with an oral corticosteroid, 9 months follow-up has not been shown flare.

**Conclusion:** Recognition of IgG4-RD spectrum condition leads to early diagnosis and treatment of the disease and so is important because it is usually responsive and prevent fibrosis and organ damages. Informed consent to publish had been obtained from the parent.

**Disclosure of Interest**

None Declared

### B39 Withdrawn

### B40 NOT JUST KAWASAKI DISEASE:APPROACH TO PERSISTENT FEVER IN PEDIATRIC AGE THROUGH THE DESCRIPTION OF 5 CASE REPORTS ABOUT PFAPA SYNDROME, FMF, SYSTEMIC LUPUS ERYTHEMATOSUS, JUVENILE DERMATOMYOSITIS AND SYSTEMIC JUVENILE IDIOPATHIC ARTHRITIS

#### Angela Mauro^1^, Rita Sottile^2^, Antonio Mellos^3^

##### ^1^Rheumatology Unit, Department of Pediatrics, "Santa Maria della Pietà" Hospital, Nola, Naples, Italy, Nola; ^2^Rheumatology Unit, Department of Paediatrics, Santobono-Pausilipon Children’s Hospital, Naples; ^3^Rheumatology Unit, Department of Paediatrics, Santa Maria della Pietà Hospital, Nola, Italy

###### **Correspondence:** Angela Mauro

**Introduction:** Fever is the most common reason for pediatric counseling and when it becomes persistent, even the most rare causes need to be considered.

**Objectives:** We present the diagnostic approach to persistent fevers in pediatric patients by describing 5 exemplary case reports.

**Methods:** Diagnostic hypotheses were formulated according to the medical history, detection of pathological signs at the physical examination and the results of laboratory and imaging tests.

**Results:** Case report 1. 3-year-old girl with high fever recurrent every month since a year, lasting about 5 days and associated with pharyngitis, aphthous stomatitis, cervical lymphadenitis with neutrophilic leukocytosis and increased erythrocyte sedimentation rate (ESR) and C-reactive protein (CRP). Between episodes, the patient was asymptomatic and laboratory tests were normal. With the passing of the years, these episodes were reduced in intensity and frequency until they disappeared completely 6 years later. The patient, nowaday adult, has grown up normally thus confirming PFAPA syndrome diagnosis.

Case report 2. In the last 6 months, 8-year-old male child reported 5 episodes of periodic fever, lasting about 3 days and associated with abdominal and thoracic pain, arthralgia and myalgia particularly in the lower limbs. Laboratory tests showed neutrophilic leukocytosis and increased ESR and CRP, while diagnostic imaging tests were negative. Suspected diagnosis of FMF was confirmed by genetic analysis and satisfactory response to colchicine therapy.

Case report 3. 14-year-old girl with intermittent fever (37,5°C- 37,8°C) since 2 month, associated with asthenia and pains in the lower limbs. Physical examination showed arthritis of the knees. Diagnostic tests showed anemia, increased ESR and CRP, presence of antinuclear antibodies (ANA), anti-native DNA and anti-Sm antibodies confirming the diagnosis of SLE.

Case report 4. 10-year-old girl reported muscle weakness associated with low-grade fever since about a month. Physical examination showed muscular hypostenia in the upper and lower limbs and in the neck muscles, Gottron papules in the metacarpophalangeal joints and eyelid rash. Blood tests showed an increase in ESR, CRP, muscle enzymes (LDH, aldolase, CK and transaminases) and presence of ANA. Electromyography revealed reduction in amplitude and duration of action potentials; magnetic resonance imaging revealed signs of muscular inflammation confirmed at biopsy, thus supporting JDM diagnosis.

Case report 5. 13-year-old girl reported several episodes of persistent fever lasting 3 - 4 consecutive days since at least 1 year. Physical examination revealed maculo-erythematosus rash, splenomegaly and asymmetric polyarthritis of the knee, hip, ankle, wrist and hip, while blood tests showed anemia, increased ESR and CRP, leukocytosis and thrombocytosis. Blood culture, bone marrow needle aspiration, the research of vanilmandelic and homovanillic acid in the urine all turned out to be normal. Radiological examinations of the joints affected by signs of arthritis highlighted just joint space narrowing and erosions, thus finally confirming s-JIA diagnosis.

**Conclusion:** Persistent fevers in pediatric patients may have not only an infectious, vasculitic or oncologic etiology, but also autoimmune or autoinflammatory causes. Informed consent to publish had been obtained.

**Reference**

Kawasaki T. Kawasaki disease. Int J Rheum Dis. 2014; 17(5):597-600.

**Disclosure of Interest**

None Declared

### B41 LONGITUDINAL ANALYSIS OF A PEDIATRIC RHEUMATOLOGY PRACTICE IN A PORTUGUESE CENTER

#### Joao M. Nascimento, Paula Estanqueiro, Manuel Salgado

##### Pediatric Rheumatology Unit, Hospital Pediátrico Coimbra CHUC, Coimbra, Portugal

###### **Correspondence:** Joao M. Nascimento

**Introduction:** Longitudinal evaluation of the pediatric rheumatology practice help to establish the spectrum of childhood rheumatic diseases and the role of pediatric rheumatology programs in evaluating children rheumatic diseases and conditions that mimic rheumatic diseases, exposing the difficulties to achieve some diagnosis.

**Objectives:** To analyze a longitudinal maintained pediatric rheumatology clinic disease prospective registry.

**Methods:** A total of 3.451consecutive referrals to Coimbra’s Pediatric Rheumatology Department during the period 1987–2017 (31 years) were analyzed. The main diagnosis, 1 to 4 per patient, were prospectively analyzed. The diagnostic criteria of each disease or syndrome were those used in recent textbooks of Pediatric Rheumatology^1,2^. Patients were classified in 25 large groups. The non-classifiable conditions were grouped into symptomatic groups.

The main diagnosis (no more than four per patient) were recorded.The diagnostic criteria of each disease were those used in recent textbooks of Pediatric Rheumatology^1,2^. Patients were classified in 25 large groups. The non-classifiable conditions were grouped into symptomatic groups.

**Results:** Among the 3.451 patients we established 4.147 different diagnosis: average 1,2 diagnosis/child, justifying 120% of diagnosis comparatively to the number of patients.

The distribution of the 25 groups was: 1. Pediatric Juvenile arthritis (JIA) 15,9%; 2. Nonspecific musculoskeletal pain 15,5%; 3. Amplified pain syndromes 8,9%; 4. Connectivitis (systemic lupus erythematous, neontal lupus erythematous, juvenile dermatomyositis, sclerodermas, mixed connective disease an undifferentiated connective tissue, Sjogren syndrome) more Arthropaties (Chronic inflammatory bowel disease, Immunodeficiencies, Erythema pernium, Celiac disease, Hypertrophic osteoarthropathy, Paraneoplasic arthritis) 8,5%; 5. Orthopedic conditions 6.0%; 6. Laboratory diagnostic investigation unduly valued for excess 5,5%; 7. Systemic vasculitis 5,2%; 8. Uveitis (5,0%); 9; Reactive arthritis, Recurrent transient synovitis of the hip and Palindromic rheumatism 4,9%; 10. Vascular acrosyndomes 4,8%; 11. Infections 3,8%; 12. Nonspecific arthritis 3,7%; 13. Dermatologic conditions including Erithema nodosum 3,5%; 14. Rheumatic fever and Poststreptococcal arthritis 3,4%; 15. Recurrent oral aphthous 3,3%; 16. Allergic conditions, mainly serum-like disease and Idiopathic Chronic Urticaria 2,7%; 17. Autoinflammatory diseases 2,5% (CRMO and PFAPA 2,1%) ; 18. Degenerative conditions, including skeletal dysplasias 1,7%; 19. Prolonged or recurrent fever 1,4%; 20. Autoimmune thyroiditis, autoimmune hepatitis and celiac disease 1,3%; 21. Malignant diseases 1,2%; 22. Others ophthalmologic conditions 1,1%, 23. Normal variants 1,0%; 24. Vascular malformations 1,0% (primary and secondary lymphedema 0,6%); 25. Miscellaneous conditions 9,9%.

Globally, “Rheumatic Diseases” represent 45,4% (Groups 1, 4, 7, 8, 9, 14, 17) and “Non Rheumatic Diseases” 54,6%.

**Conclusion:** The many differential diagnosis of the rheumatic diseases justifies the reason for the many different pathologies observed in pediatric rheumatology units. It also justify that less than half of the patienets observed in our unit had the classic “Reumatic Diseases”.

The sum of Nonspecific musculoskeletal pain, Amplified pain syndromes and excess Laboratory diagnostic investigation represents 30% of patients observed, also justifying the need of training in basic issues of pediatric rheumatology.

**References**

1. Petty RE, Laxer RM, Lindsley CB, Wedderburn LR. Textbook of Pediatric Rheumatology. 7^th^ ed, Elsevier, Philadelphia, 2016.

2. Sawhney S, Aggarwal A. Pediatric Rheumatology. Springer, Singapore, 2017.

3. Rosenberg AM. Longitudinal Analysis of a Pediatric Clinic Population. J Rheumatol 2005;32:1992-2001

**Disclosure of Interest**

None Declared

### B42 ASSOCIATION OF RAGHIB SYNDROME WITH AUTOIMMUNE PANCREATITIS (EXTREMELY RARE ASSOCIATION

#### Shima Salehi^1^, Mohammad R. Goudarzi^2^, Azizollah Yosefi^3^

##### ^1^Pediatric Rheumatology, Ali-asghar Children's Hospital,Iran University of Medical Siences; ^2^Pediatric Cardiology; ^3^Pediatric gastroenterology, Hazrate Rasool General Hospital,Iran University of Medical Siences, Tehran, Iran, Islamic Republic Of

###### **Correspondence:** Shima Salehi

**Introduction:** Herein we describe an unusual finding of raghib syndrome(an unroofed left superior vena cava) in a case of autoimmune pancreatitis with IgG4 related disease(AIP/IgG4RD) .

**Objectives:** Autoimmune pancreatitis (AIP) is a rare form of chronic pancreatitis with characteristic clinical, histologic, and morphologic findings rarely reported in children which clinically present with abdominal pain, weight loss, and jaundice also introduced by Kamisawa as a syndrome characterized by elevated serum IgG4 levels, prominent lymphoplasmacytic infiltrates with increased IgG4^+^ plasma cells, and dense sclerosis.time is still needed till its cardiac complications and assosiation be declared.

**Methods:** after thorough history taking and physical examination a standard transthoracic echocardiography performed by expert pediatric cardiologist.

**Results:** after visualization of a dilated coronary sinus,contrast echocardiography by injecting agitated salin via left antecubital vein performed at first, which revealed entrance of bubbles into left atrium that is charactristic of unroofed LSVC.then it was confirmed with a more accurate procedure of transesophageal echocardiography.

**Conclusion:** Raghib syndrome is an extremely rare congenital heart defect characterized by persistent left superior vena cava (LSVC) draining into the left atrium and absence of the coronary sinus.An unroofed left superior vena cava (LSVC) is usually asymptomatic and diagnosed incidentally although it gives rise to increased risk of paradoxical embolism. The standard technique for the diagnosis is echocardiography, either transthoracic or transoesophageal.Up to now some cardiac complications like lymphoplasmacytic aortitis is described in association with AIP but best of our knowledge occurrence of raghib syndrome or unroofed LSVC in a case of AIP hasnt been reported yet

**Disclosure of Interest**

None Declared

## New diseases

### B43 AUTOIMMUNE LYMPHOPROLIFERATIVE SYNDROME, ALPS: AN EMERGENT MOLECULAR PATHOLOGY

#### Adriana S. Diaz-Maldonado^1^, Yefry Aragon^2^, Veronica Pardo-Manrique^3^

##### ^1^Care for Kids - Homi; ^2^Fundación HOMI; ^3^Pontificia Universidad Javeriana, Bogota, Colombia

###### **Correspondence:** Adriana S. Diaz-Maldonado

**Introduction:** The Autoimmune Lymphoproliferative Syndrome (ALPS) is a disease associated to alterations in apoptosis due to a genetic defect (mutations of the *FAS*, *FASLG* and *CASP10* genes), characterized by a non-neoplasic lymphoproliferative state and fever for more than 6 months.

**Objectives:** Describe clinical presentation, treatment and outcomes of ALPS patients followed at a Pediatric Hospital Center in Colombia.

**Methods:** A retrospective observational study was conducted, 2300 clinical records of children attended at Fundación HOMI between 2010 and 2016 . Based CIE-10 codes 31 cases met clinical criteria of NIH for ALPSDemographic characteristics, clinical presentation and laboratories were recollected in a Microsoft Excel® sheet. Stata 8® software was used to statistical analysis

**Results:** Of the 31 patients , 67.7% were male, Average age at diagnosis was 6 years old (6 months to 15 years old). Among the reasons for consultation we found that fever and lymphadenopathy was the most frequent symptom (96.7%), followed by hepatomegaly (58%), splenomegaly (54%), exanthema (25.8%) and arthritis 2(2.6%). Doubly negative lymphocytes high count was founded in in all cases (values between 1.5% and 16.2%). B12 vitamin average was 987.5 ng/L (range 237-2000ng/L) and total immunoglobulin G levels was found 240 – 5104 mg/dl. Molecular studies for mutations in the *FAS, FASLG y CASP10* genes were negatives in 9 cases, in 7 patients was pending at moment of this revision, and was not possible to do in 15 due to administrative limitations from insurances.Table 1 summarizes the characteristics of the patients. About treatment in acute phase, 20 patients received oral corticosteroids, 7 patients with thrombocytopenia received intravenous immunoglobulin (IVIg) , and 8 children with hemolytic anemia received methylprednisolone. Maintenance medications include: Cyclosporine A in 6 patients, methotrexate in 5 cases with joint manifestations, mycophenolate mofetil in 3 children, sirolimus in 2 cases with refractory thrombocytopenia and rituximab in 1 patient.

**Conclusion:** All patients this study configure probable diagnosis of ALPS since none has positive genetic test for apoptosis mutations, although 40% of the cases do not have any mutation according to the literature. The ALPS is confused with other hematology, oncology and autoimmune diseases for its presentation. The ALPS is a non-prevalent genetic disease that should be considered in patients with signs of chronic lymphoproliferation without neoplasic pathology.

**Disclosure of Interest**

None Declared

**Table 1 (abstract B43).** See text for description.

Variable	Description	No. (%)
Sex	Women	10 (32.3)
	Men	21 (67.7)
Vital status at the time of review	Alive	30 (96.7)
	Dead	1 (3.3)
Patients who received some treatment	Yes	22 (71)
	No	9 (29)
Number of medications received	One	10 (32.3)
	More than one	12 (38.7)

## Psycho-social aspects and rehabilitation

### B44 PHYSIOTHERAPY FOLLOW UP WITHIN FOUR WEEKS AFTER INTRA-ARTICULAR STEROID INJECTION

#### Anna Haavisto Olow, Linn Hoel

##### Physiotherapy, Queen Silvia Children's Hospital, Gothenburg, Sweden

###### **Correspondence:** Anna Haavisto Olow

**Introduction:** Background:

When treating active arthritis in juvenile idiopathic arthritis (JIA) intra-articular corticosteroid injections (IAS) are an action of importance (1-4). IAS has been proven a safe and efficacious treatment for active arthritis either as single treatment or in combination with medicine therapy. IAS have been shown to drastically relieve pain, swelling and to increase mobility in the injected joint (1-4). At our clinic the patients who receive IAS are to be offered a follow up at the physiotherapy department or occupational therapy department (depending on the joint injected) within two to four weeks following the injection, according to the care program. As to this date there has been no survey at our clinic to show how many patients are followed up as agreed.

**Objectives:** 1) How many of the patients are offered follow up at the physiotherapy clinic within two to four weeks after IAS? 2) What is the time between the injection and the actual follow up? 3) What was the effect of the IAS?

**Methods:** A retrospective review of the patient registry and charts at the rheumatology clinic at Queen Silvia Children's Hospital, during January 1st - December 31st 2017.

**Results:** The total number of individual patients who received IAS during 2017 at the rheumatology clinic was 63. The number of appointments for IAS was 93 and the number of injections given was 174. The department of physiotherapy followed up a total of 92% the patients relevant for physiotherapy. Out of these, 53% were followed up within 28 days. Of the patients followed up 46% experienced good effect of the injection, though the effect was not always well documented in the charts. See Table 1 for details.

**Conclusion:** All patients were offered follow up, however not always in time. Of patients 92% were followed up and 46% received good effect from the injection. This retrospective study has lead to a further development of the post-injection assessment of the patients with a more structured guide how to evaluate the effect of injections. A further question to evaluate is the actual care programme, if it functions as intended, with an efficient communication within the team, and as to what happens in case of no effect of the injection, are the patients offered a doctor’s appointment earlier or even another injection? Another important matter is if the care programme is relevant for the patient.

**Disclosure of Interest**

None Declared

**Table 1 (abstract B44).** Demografics of patients undergoing IAS and results of the survey

Individual patients	63, 19 male*
Disease subtype	
Systemic	-
Oligoarticular	45
Polyarticular	10
Psoriatic	1
ERA	2
Undifferentiated	1
Juvenile dermatomyositis	1
Seropositive RA	1
Purulent arthritis	1
Joint pain	1
Appointments for IAS	93*
Injections	174*
Relevant for physio: appointments for IAC/patients	75/ 49 (14 male)
Follow ups within time	40 (53%)
Total number of follow ups	69 (92%)**
Median time to follow up	27 (range 8-119)
Effect at follow up when documented ***	
+ (good)	32 (46%)
+/- (major effect but not complete)	14 (20%)
- (no or minor effect)	18 (26%)

*4 patients (2 male); 7 appointments; 10 injections: only have their doctor at DSBUS rest of team at local hospital. **100% were offered follow up, 6 appointments cancelled by patient/family. ***Effect was not well documented in charts at all follow ups

### B45 RHEUMATIC DISEASE IN CHILDREN AND HORSE ASSISTED THERAPY

#### Barbara L. Teruzzi^1^, Costantino De Giacomo^2^

##### ^1^Maternal and Child Health -Division of Pediatrics-Pediatric Rheumatology; ^2^Maternal and Child Health -Division of Pediatrics, ASST GOM Niguarda, milano, Italy

###### **Correspondence:** Barbara L. Teruzzi

**Introduction:** Rheumatic diseases in children are a multisystemic, heterogeneous group of diseases involving mainly the muscoloskeletal system resulting in disability. The multidisciplinary approach is essential in the management of rheumatic diseases in children.

**Objectives:** Aim of this study is to evaluate the rehabilitation aspect of the child with chronic rheumatic disease in a more holistic view of his care and his recovery. Rehabilitation must be based not only on diagnostic and prognostic evaluation of the damage but also the awareness of the repercussions of the motor impairment on the psychomotor, intellectual, and emotional development. Equestrian rehabilitation (ER) is not a method but a rehabilitative practice using the horse as a mean of rehabilitation, the horse facilitates postural, motorial, perceptive and relational-motivational reeducation; ER is therefore a therapeutic intervention methodology that is an individual rehabilitation project.

**Methods:** Besides clinical assessment of different types of arthritis, the specific rehabilitative aspects (joint evaluation, muscular strength, CHAQ, VAS dolor) must be evalueted, around which must organizing the intervention and the objectives. ER consists of 3 levels of intervention:1.horse therapy (hippotherapy), uses bodily features of the horse to the gait where the horse transmits to the child over three levels (longitudinal, transverse and sagittal plane) the correct human gait, playing a physiological pattern movement. At this level you are working on fine motor movements, balance, laterality, body image and cognitive aspects of attention-perception; 2.re-education trought horse riding, aims to achieve autonomy in the conduct of the horse; at this level you are working on the body pattern, lateralization movements with the use of the reins in the conduct of the horse, the balance on the saddle with stirrups, on fine motor coordination, on taking the reins, and space-time continuity, both sensory and motor; at this level we can introduce the trotting gait stimulating the adaptive, coordinative capacities in the relationship with the horse. The motivational aspect are so stimulated that leads to the pattern of movement; 3.equestrian rehabilitation therapeutic group (disability group).

**Results:** In horse-assisted therapy observations are made on the effects on the neuromuscular system of the patient caused by the mechanical influence of the horse walk. Specific to the horse therapy is that the patient continuously receives impulses from the horse walk, which lead to a relaxed perception of the body, equilibrium, and coordination of movement.

**Conclusion:** In horse-assisted therapy observations are made on the effects on the neuromuscular system of the patient caused by the mechanical influence of the horse walk. Specific to the horse therapy is that the patient continuously receives impulses from the horse walk, which lead to a relaxed perception of the body, equilibrium, and coordination of movement.

**Trial registration identifying number:** Rehabilitative therapy during childhood and adolescence is aimed primarily to prevent primary damage effects produced by sensory-motor development. The child with a disability due to chronic rheumatologic disease must follow a rehabilitation program aimed to the functional motor recovery and also psycho-affective recovery in which the horse is the rehabilitation mean.

**Disclosure of Interest**

None Declared

### B46 NEUROLOGICAL EVALUATION OF THE MATURITY OF CHILDREN BORN TO WOMEN WITH RHEUMATIC DISEASES

#### Ana V. Villarreal Treviño^1^, Fernando García-Rodríguez ^1^, Dionicio Galarza-Delgado^2^, Manuel de la O-Cavazos^3^, Itzel Pérez-Onofre^2^, Lorena Pérez-Barboza^2^, Cassandra Skinner^2^, Mayra G. Sánchez-Muñoz^3^, Rubí Martinez-Mendoza^3^, Lorena I. González-Palaú^3^, Yesenia Medina-delaCruz^3^, Adriana Nieto^3^, Nadina Rubio-Pérez^3^

##### ^1^Pediatric Rheumatology; ^2^Rheumatology; ^3^Pediatric Department , Hospital Universitario "José Eleuterio González", Monterrey, Mexico

###### **Correspondence:** Ana V. Villarreal Treviño

**Introduction:** Previous studies have linked maternal Rheumatoid Arthritis, Systemic Lupus Erytematosus, and other rheumatic diseases with preterm birth and low birthweight. Now a growing evidence suggest increased rates of learning delays among offspring of mothers with rheumatic diseases and associations between autism spectrum disorders, dyslexia and other neurocognitive dysfunction. There is a lack of evidence about neurological maturational changes and behavioral functioning the first year of life of children born to women with rheumatic diseases, and the relation between maturational abnormalities and their mothers disease diagnosis, evolution and treatment.

**Objectives:** To describe the neurological assessment and birth outcomes of children born to women with rheumatic diseases.

**Methods:** Children born between July 2017 and April 2018 of mothers attended at Hospital Universitario “José Eleuterio González” in Monterrey, Nuevo León, México with rheumatic diseases were included in this cross-sectional study. Descriptive data, demographic, clinical and laboratory results were collected form mothers and offspring. Amiel-Tison Neurological Assessment at Term (ATNAT), a 35 item test was used to evaluate the maturity of the children.

**Results:** Nine children were evaluated, the median of gestational birth age was 38.6 (33.4-43.4) weeks, the birth of female gender was more frequent (n=7), the mean of birth weight was 2,990 grams (S.D ± 105.2 grams) , the mean of length was 48.8 ± 2.47cm , cephalic perimeter median 33.07 (34-36) cm.

The median of mothers age was 32 years (19 – 42), Rheumatoid Arthritis was the most frequent rheumatic disease (55.7%), the disease was considered active in 71.4%, with only 2 patients were without medication and the most frequently received treatment was sulfasalazine. The neurological assessment showed alteration in 88.8%, with predominance in muscle tone as spontaneous motor activity (n=7), the 57% showed hypertonia in neck flexors. When directed, there were alterations in the way of loading the child, which was modified predominantly by pain in the upper extremities, position which would explain the alterations in the tone found in the patients evaluated.

**Conclusion:** This study showed an important relation between the mother diagnoses, its activity degree, and neurological maturity abnormalities in their children. The musculoskeletal affection in rheumatic diseases especially the upper extremities apparently affects the maturity of the children due to the erroneous racing way. We propose the realization of prospective studies that evaluate the response to interventions and the modification in neuronal maturation in these patients. In addition to reporting evidence of alterations in the neurodevelopment of children of mothers with musculoskeletal involvement, secondary probably to changes in the way of parenting.

**Disclosure of Interest**

None Declared

## Scleroderma and related syndromes

### B47 JUVENILE SCLERODERMA: A SINGLE CENTRE EXPERIENCE FROM TURKEY

#### Gül Karadağ, Mustafa Çakan, Ayşe Zopçuk, Nuray Aktay Ayaz

##### Pediatric Rheumatology, Sağlık Bilimleri University KSSEAH, Istanbul, Turkey

###### **Correspondence:** Nuray Aktay Ayaz

**Introduction:** Juvenile scleroderma is a rare multisystemic autoimmune disease. The two main forms of the disease are localized scleroderma (LS) and systemic sclerosis (SSc). In juvenile LS fibrosis involves limited areas of the skin.

**Objectives:** This study was established as a retrospective review of the patients diagnosed and followed-up in Sağlık Bilimleri University KSSEAH Pediatric Rheumatology outpatient-clinic as JS between the years 2012 and 2018. Our purpose was to report our single-center experince with JS.

**Methods:** The medical charts of patients diagnosed and followed-up as JS between the years 2012–2018 retrospectively. Totally 24 patients were enrolled to the study. Data recorded included age, sex, age at disease-onset, age at-diagnosis, delay to the diagnosis, presence of consanguinity, laboratory data, family history of both scleroderma and any other autoimmune disease, treatment modalities and outcomes.

**Results:** In our study, a total of 24 patients with juvenile scleroderma were reviewed. One patient (a boy, 4.17 %) had SSc and 23 patients (%95.83) had LS. The ratio of female (n: 19) to male (n: 5) was 3.8:1. The mean age 12.38±3.97, mean age at the onset of symptoms was 9.13 ± 4.13 years. All patients were treated with oral prednisolone and oral or subcutaneous MTX. The treatment of 4 patients (16.67 %) were changed to mycophenolate mofetil and 1 (4.17 %) was changed to cyclosporine because of unresponsiveness or intolerance. Eight patients (33.33 %) were treated with local steroids and 4 were treated with topical tacrolimus (16.67 %) in addition to systemic treatment. Two generalized scleroderma patients treated with IVIG (8.33%).

**Table:** 

Capillaroscopic findings [n (%)]	4 (16.67%)
Raynaud’s phenomenon [n (%)]	3 (12.50%)
Arthralgia [n (%)]	6 (25.00%)
Arthritis [n (%)]	4 (16.67%)
Contracture [n (%)]	7 (29.17%)
Uveitis	0 (0%)

**Conclusion:** SSc is very rare in children. The incidence of juvenile SSc is around 0.05 per 100,000 children. In our study the ratio of SSc to LS was 1/23. Juvenile LS (JLS) is more common in girls (female to male ratio 2.4: 1) with mean age-at onset of 7.3 years. According to our results, female-to-male ratio of LS was 3.8/1 and the mean age-at-onset of LS was similar to literature as 9.13±4.13 years. Laboratory results are suggestive, but not specific enough for diagnosis of scleroderma. There is no single therapy for JS. MTX had been successfully used both in adults and in children with localized scleroderma. MTX and/or corticosteroids (oral or intravenous) are accepted as first line treatment.

In conclusion, juvenile scleroderma is very rare in children, and characterized by the thickening and hardening of the skin. Most presented as a localized form of the disease. Close monitoring of the disease status and treatment response is important for clinical evaluation and early clarification of disease complication. The prognosis of LS in children is

better than in adults. If disease showed progressing clinically, it should be treated aggressively even when the skin lesion are localized initially. There is a need for new studies for better understanding of the prognosis, evaluation of the disease and new treatment modalities.

**Disclosure of Interest**

None Declared

## Spondyloarthritis (SpA) and enthesitis related arthritis (ERA)

### B48 HOW SAPHO SUPRISED US- CASE REPORT

#### Dusana Moravcikova^1^, Katarina Okalova^1^, Katarina Novotna^2^, Karol Kralinsky^1^ and Children's University Hospital Banská Bystrica

##### ^1^Paediatrics; ^2^Paediatric Radiology, Children's University Hospital Banská Bystrica, Banska Bystrica, Slovakia

###### **Correspondence:** Dusana Moravcikova

**Introduction:** SAPHO syndrome (synovitis, acne, pustulosis, hyperostosis, osteitis) belongs to autoinflammatory diseases without genetic correlation. The incidence of the disease is very low, and the disease is classified as an orphan disease. In addition to skin manifestation, clinical manifestation includes musculoskeletal symptoms, with a dominant affection of the axial skeleton, therefore the SAPHO syndrome is currently classified as juvenile spondylartropathy. A frequent location of aseptic inflammation, in addition to axial involvement, is the area of the anterior wall of the chest – sternoclavicular and sternocostal joints. The syndrome may not be fully expressed – skin manifestations often occur independently, and only after several months, synovitis and osteitis develop, or vice versa. In the active stage of the disease, increased inflammatory parameters (erythrocyte sedimentation, CRP) can be observed. Imaging examinations (magnetic resonance imaging and scintigraphy) have an irreplaceable role in diagnosis. Differential diagnosis is necessary to exclude particularly an infectious process and neoplasia, therefore biopsy examination is a “gold standard”. Current treatment options include the use of disease-modifying anti-inflammatory drugs (NSAIDs, antibiotics, corticosteroids, sulfasalazine, methotrexate). For relapsing and high-activity forms of the disease, there is sufficient data on the use of biological treatment (adalimumab, infliximab). The response to the treatment determines the prognosis of the patients; with the good response to DMARDs, the prognosis is optimal.

**Objectives:** We present the case of a 17-year-old patient initially admitted with back pain in the lumbosacral region. In the course of two weeks, the condition progressed into a spastic triparesis with the impossibility of independent walking. The patient had a history of a total and local acne conglobata treatment lasting for several months. Laboratory parameters showed high inflammatory activity with insufficient response to broad-spectrum antibiotic therapy. Cerebrospinal fluid analysis and imaging examination of brain, C, Th, LS spinal cord without pathology. Due to oedema and tenderness of the sternocostal joint to the left, the MR examination was carried out with the finding of an inflammatory process – osteitis; the SAPHO syndrome is considered for the first time. Scintigraphy with a picture of high rearrangement and increased perfusion in the clavicle, in the sternoclavicular joints, sacroiliac articulations, in both ischial bones. Biopsy of the sternoclavicular joint confirmed chronic non-specific synovitis. Based on both clinical and laboratory findings, the patient met the SAPHO syndrome criteria.

**Methods:** Case report.

**Results:** The purpose of the case report was to point to differential diagnostic pitfalls, which led to a definitive diagnosis of autoinflammatory disease. At the same time, we provide an overview of the current therapeutic options for this rare disease , especially the effectiveness of biologic drugs in our case.

**Conclusion:** The purpose of the case report was to point to differential diagnostic pitfalls, which led to a definitive diagnosis of autoinflammatory disease. At the same time, we provide an overview of the current therapeutic options for this rare disease , especially the effectiveness of biologic drugs in our case. Informed consent to publish has been obtained from the patient.

**Disclosure of Interest**

None Declared

## Systemic JIA

### B49 THE CHARACTERISTICS OF PATIENTS WITH SYSTEMIC JUVENILE IDIOPATHIC ARTHRITIS IN IZMIR DR. BEHCET UZ CHILDREN’S HOSPITAL

#### Ozge Altug Gucenmez^1^, Balahan Makay^1^, Neslisah Uslu^2^

##### ^1^Izmir Dr. Behcet Uz Children’s Hospital, Izmir; ^2^Koc University, Istanbul, Turkey

###### **Correspondence:** Ozge Altug Gucenmez

**Introduction:** Systemic juvenile idiopathic arthritis (sJIA) has a special place among childhood arthritis with a broad spectrum of mild to severe joint problems and significant extraarticular features.

**Objectives:** It is aimed to present the characteristics of sJIA patients who are followed in Izmir Dr. Behcet Uz Children’s Hospital.

**Methods:** The medical files of 12 sJIA patients who are followed in Izmir Dr. Behcet Uz Children’s Hospital were investigated retrospectively.

**Results:** Six girls and six boys with sJIA were included in the study. The median age was 115.5 months (min-max: 15-195 months), median diagnosis age was 74,5 months (min-max: 10-122 months), and median follow-up time was 20 months (min-max: 1-84 months). The median delay for the diagnosis was 12 days (min-max: 7-30 days). All the cases had intermittent fever. The half of the patients showed rashes and/or joint involvement. Serositis was present in only one patient (8.3%). The complications were as follows: macrophage activation syndrome (MAS) in 4 patients, and severe growth retardation due to long-term corticosteroid use in 1 patient. Lymphadenopathy, HSM, and uveitis were not present in any cases. The cases were using the following medications: NSAID (8.3%), steroid (41.7%), methotrexate (66.7%), anakinra (8.3%), canakinumab (25%), tocilizumab (33.3%). While no remission was achieved in 1 case, 9 cases achieved remission under medical treatment. Two patients (16.7%) have achieved remission without need of medications. The laboratory findings regressed to the normal levels following the treatment (p<0.05).

**Conclusion:** sJIA is a differential diagnosis and it might take a while to diagnose a patient with sJIA. This delay might lead unwanted situations as MAS. sJIA might be controlled with proper treatment plans.

**Disclosure of Interest**

None Declared

### B50 Withdrawn

## Systemic lupus erythematosus and antiphospholipid syndrome

### B51 CORRELATION BETWEEN SPOT PROTEIN /CREATININE RATIO AND 24 HOUR URINARY PROTEIN EXCRETION IN CHILDHOOD LUPUS NEPHRITIS

#### Manjari Agarwal, Sujata Sawhney

##### Division of Pediatric and Adolescent rheumatology, SIR GANGA RAM HOSPITAL, NEW DELHI, India

###### **Correspondence:** Manjari Agarwal

**Introduction:** In children with lupus nephritis, 24 hour estimation of urinary protein measurement is considered as gold standard. But it is fraught with difficulties of collection.This study was undertaken to find out if a random urine spot protein/creatinine is reliable and can be interchanged with a 24 hour urine protein estimation

**Objectives:**

1. To find out the correlation of spot protein/creatinine estimation and 24 hour urine protein estimation
2. To find the value of spot protein/creatinine ratio that best correlates with the 24 hour protein value

**Methods:** 209 paired samples of spot protein/creatinine ratio and 24 hour urine protein were retrospectively recorded from charts of 64 children with lupus nephritis.

Statistical analysis was done using SPSS version 16.1.

**Results:** Spot protein creatinine ratio showed a good correlation with 24 hour urine protein values with p value <0.01.The correlation was poor for spot protein/creatinine ratio <0.5(p value=0.5).

Using ROC, the cut off value of spot protein creatinine ratio to predict nephrotic range proteinuria (>1gram) was 1.81 with area under curve 95%(0.875). 95% confidence interval(0.817-0.932). Sensitivity 73.1%, specificity 90.8%. Positive predictive value is 0.79, negative predictive value 0.87.

**Conclusion:** The spot protein/creatinine ratio in ranges>0.5 correlate well with 24 hour protein values and can be reliably used in routine clinical practice.The urine spot protein/creatinine ratio can thus be utilised fairly accurately as a guideline to determine whether child has nephrotic range proteinuria or not.It however cannot be used interchangeably in higher values of spot protein/creatinine ratio.

**References**

Christopher-Stine L, Petri M, Astor BC, Fine D. Urine protein-to-creatinine ratio is a reliable measure of proteinuria in lupus.J Rheumatol 2004;31;1557-1559

Lane C,Brown M,Dunsmuir W,Kelly J,Mangos G. Can spot urine protein/creatinine ratio replace 24 h urine protein in usual clinical nephrology? Nephrology 2006; 11, 245–249

**Disclosure of Interest**

None Declared

**Table 1 (abstract B51).** Correlation between spot protein/creatinine ratio and 24 hour urine protein

Spot protein/creatinine ratio	n	24 hour urine protein	Significance (p value)
		r value	
overall	209	0.669	0.001
<0.5	65	-0.083	0.51
0.5-1	45	0.347	0.019
>1	99	0.579	0.01

### B52 DIFFICULTIES IN DIAGNOSING LUPUS-LIKE SYNDROMES

#### Olena A. Oshlyanska^1^, Agar G. Artsymovych^2^

##### ^1^Department of Pediatrics № 1, Shupyk National Medical Academy of postgraduate education; ^2^Department of connective tissue disorders in children, State Institute of Pediatrics, Obstetrics and Gynecology, Academy of Medical Sciences of Ukraine, Kyiv, Ukraine

###### **Correspondence:** Agar G. Artsymovych

**Introduction:** The symptom complex of some rheumatic diseases develops gradually. Various combinations and overlop-syndromes are described. In recent years, more scholars have focused on the development of genetically determined forms of autoimmune diseases, including lupus, an analysis of the peculiarities of their clinical course and treatment.

**Objectives:** To analyze the features of the pediatric case of systemic lupus erythematosus (SLE).

**Methods:** analysis of data of medical documentation

**Results:** Boy 14 y old. From birth, there was a rash on the skin. At 1,5 y he had thrombocytopenia. Frequent infectious diseases. At 3 y. episodes of bronchoconstriction. At 12 y. edema of the ankle joint, hyperthermia, hepatolienal syndrome, carditis, positive antinuclear antibodies. Diagnosed sJIA: GC, methotrexate. The effectiveness of therapy was partial: pain syndrome and temporary synovitis persisted, pericarditis was detected. GC was increased periodically. At 13 y. rescribed tocilizumab. At 14 y. cramps of limbs were added, polyneuropathy was registered on EMG. Antibodies to MPO, PT3, glomerular membrane, APL were negative. At MRI of the brain, pathological changes were not detected at that time. At 14.5 y., bullous changes of the skin. At 15 y. daily loss of consciousness, convulsions in the limbs, pain in the joint, gait disturbance. Child had exogenous hypercorticism, malar rash, erythema on the elbow and popliteal, livedo on the lower limbs, keloid scars on the lumbar cluster. Leg and foot were paste. Weakening of breathing in the lower parts of the lungs, tachycardia 115, liver +2 cm. No synovitis found. Left tongue deviation. Weakness in the left leg. Increase of tendon reflexes and positive plantar reflex on the left, knees and ankles clonus. EEG the predominance of slow-wave paroxysms in the mid-back segments of the cerebral hemispheres. Blood leukopenia, thrombocytopenia, ESR 2 mm/h. Significant changes in the biochemical study were not detected. Calcium, kalium, Ck were normal, LDH 335U/l. Results of immunological studies: IgG 5.0, IgA 0.43, IgM 0.31 g/l, ANA, APLAb, lupus anticoagulant, anti-dsDNA negative. Complement level is normal. Blood lymphocyte subpopulation: lymphopenia, deficiency of B-lymphocytes 2.58%. US-examination: hepatomegaly. Spirography: disturbance of ventilation capacity of the lungs by obstructive type. CT of chest: exudative pericarditis, inflammatory fibrosis S1-3 on the left, S3 on the right. Head MRI: cerebrovascular accident ischemic type in the basin of the right posterior cerebral artery. In order to clarify the nature of the inflammation, a skin biopsy was performed: lymphocytic infiltration around the vascular bundles, vascular walls with microvessel thickening, fibrinoid swelling, endothelium with manifestations of dystrophy. A part of vessels are with obliteration of lumens, vacuolic dystrophy of the epidermis, focal fibrinoid swelling. These changes allowed to completely eliminate sarcoidosis and treat the disease as SLE. Due to the impossibility of conducting genetic studies, clinical differential diagnosis was performed between the forms of the congenital lupus. The presence with an early onset, infectious syndrome, lung injury, CNS, hematologic and lymphoproliferative syndrome, cold changes, absence of antibodies to DNA, ANCA, normal level of complement and IgM, indicated the possibility of interferonopathy. Correction of therapy has been performed: the dose of GС was increased to 1.5 mg/kg/d, DMARD has been changed to cyclophosphamide, anti-convulsant and antihypertensive drugs have been added.

**Conclusion:** Possibility of development of identic symptoms in rheumatic diseases can lead to the appointment of therapy before establishing the final diagnosis. Improving the possibilities of in-depth examination of the child will promote the appointment of effective therapy. Informed consent to publish has been obtained.

**Disclosure of Interest**

None Declared

### B53 VENOUS THROMBOSIS AS A FIRST SIGN OF ANTI PHOSPHOLIPID SYNDROME SECONDARY TO SYSTEMIC LUPUS ERYTHEMATOSUS

#### Nihal Sahin^1^, Sümeyra Özdemir Çiçek^1^, Ali Baykan^2^, Ruhan Düşünsel^1^

##### ^1^Pediatric Rheumatology; ^2^Pediatric Cardiology, Erciyes University Faculty of Medicine, Kayseri, Turkey

###### **Correspondence:** Nihal Sahin

**Introduction:** Antiphospholipid syndrome (APS) is an autoimmune disease that presents with recurrent arteriovenous thrombosis, repeated pregnancy loss and elevated titers of antiphospholipid antibodies. APS may occur as an isolated clinical entity (primary APS) or in association with other diseases, mainly systemic lupus erythematosus (SLE). The incidence of thrombosis in children is significantly lower than in adults but the rate of APS-associated thrombosis in children appears to be higher than in adults with other common prothrombotic risk factors. We presented 16-year-old, female with SLE secondary to APS with deep vein thrombosis as initial sign.

**Objectives:** .

**Methods:** Case reports

**Results:** A 16-year-old girl who was admitted to the emergency room with the cause of pain and swelling of the left leg, hospitalized with left-sided popliteal vascular thrombosis in Doppler USG findings. Her medical and family history is unremarkable. In physical examination, she was obese with a BMI 34 (>95p) and her left leg was swollen, hyperemic and painful. Vital signs were normal. She had no rash in her body except the discoid rash on her cheek. Blood cell counts, liver and kidney function and urinary tests were normal, CRP: 61.08 mg / L, ESR: 6 mm / hr. In the investigations performed with the cause of obesity, insulin resistance was shown and abdominal USG was normal. Coagulation parameters was found as aPTT: 48,2 secs, PT: 12,7 secs, INR: 1,16 INR, D-dimer 550 [mu] g / L. First, the anti-factor Xa was given and then replaced by warfarin. Metformin treatment was added for insulin resistance. Investigations were carried out by the Department of pediatric hematology for thrombosis etiology. Lupus anticoagulant was highly presence. Factor V Leiden, MTHFR, prothrombin, PAI genetic analyzes were performed (Tests are pending). She was consulted to department of pediatric rheumatology with the reason of discoid rash. Complement C4 (6,9 mg/dl) was decreased. Immunological tests show presence of antinuclear antibodies, anti-double stranded DNA antibodies, a positive direct Coombs test and presence of anti-phospholipid antibodies (Lupus Anti-Coagulant high positive, anticardiolipine antibodies IgM: 69 and anti-B2-glycoprotein I IgM: >200). In the light of clinical and laboratory findings, diagnosis of secondary antiphospholipid syndrome to systemic lupus erythematosus was made.

**Conclusion:** Venous thrombosis in children is uncommon. In most cases several clinical risk factors for thrombosis are present. In adolescents it may be the first manifestation of SLE. Therefore, SLE and APS should be kept in mind in and investigated in children with thrombosis.

**Disclosure of Interest**

None Declared

### B54 ASSESSMENT OF ANTI-NUCLEAR ANTIBODY TEST RESULTS IN 0-18 AGE GROUP ADMITTED TO THE HOSPITAL: A SINGLE CENTER STUDY

#### Nihal Şahin^1^, Sümeyra Özdemir Çiçek^1^, Neslihan Günay^2^, Ayse S. Pınarbaşı^2^, Sibel Yel^2^, İsmail Dursun^2^, Muammer H. Poyrazoğlu^1^, Huseyin Kılıç^3^, Ruhan Düşünsel^1^

##### ^1^Pediatric Rheumatology; ^2^Pediatric Nephrology; ^3^Medical microbiology, Erciyes University Faculty of Medicine, Kayseri, Turkey

###### **Correspondence:** Nihal Şahin

**Introduction:** Antinuclear antibodies (ANA) are important for diagnosis of rheumatic diseases. However, a positive ANA test is obtained different conditions and healthy people. So routine antibody screening is not recommended if there aren’t appropriate clinical signs.

**Objectives:** This study aimed to evaluate the ANA positivity rate detected in Erciyes University laboratories.

**Methods:** In Erciyes University Hospitals ANA tests requested by different specialties in 2013-2017 years assessed retrospectively. ANA have been determined by indirect immunofluorescence (IIF). ANA test results obtained from medical records of serology laboratory.

ANA have been determined by indirect immunofluorescence (IIF)

**Results:** 5999 tests requested by different specialties obtained. There were 4940 patients when recurrent tests removed. 1323 (26,8%) of tests were positive and 3617 (73,2%) of tests were negative. 684 (51,7%) of positive tests had ≥1/1000 antibody titers, 628 (47,5%) had ≤1/100, 6 (0,4%) had 1/320 titers. 917 patients (69,3%) were female, 1695 patients (46,9%) were male in positive results, and 1921 patients (53,1%) were female, 1695 patients (46,9%) were male in negative results. 3812 patients were requested from different pediatric clinics. 1670 (43,8%) patients were requested from pediatric rheumatology clinic and 1229 (32,2%) patients were requested from other pediatric clinics. When ANA assessed according to date 637 (12,9%) patients were requested in 2013, 953 (19,3%) in 2014, 1154 (23,3%) in 2015, 1038 (21%) in 2016, 1158 (23,4%) in 2017.

**Table:** 

Department of pediatrics	≤1/100	≥1/1000	1/320	Undetermined	Positive n (%)	Negative n (%)	Total n (%)
Rheumatology	251 (6,5%)	313 (8,2%)	6 (0,2%)	3 (0,1%)	573 (15%)	1097 (28,8%)	1670 (43,8%)
General pediatric	112 (2,9%)	94 (2,5%)	0	2 (0,05%)	208 (5,4%)	705 (18,5%)	913 (23,9%)
Neurology	37 (0,95%)	37 (0,95%)	0	0	74 (1,9%)	343 (9%)	417 (10,9%)
Gastroenterology	26 (0,7%)	39 (1%)	0	0	65 (1,7%)	307 (8%)	372 (9,7%)
Hematology and oncology	14 (0,4%)	21 (0,5%)	0	0	35 (0,9%)	140 (3,7%)	175 (4,6%)
Infectious disease	3 (0,1%)	4 (0,1%)	0	0	7 (0,2%)	46 (1,2%)	53 (1,4%)
Nephrology	6 (0,1%)	10 (0,3%)	0	0	16 (0,4%)	22 (0,6%)	38 (1%)
Other department	17 (0,4%)	15 (0,4%)	0	0	51 (1,3%)	142 (3.7%)	174 (4.5%)
TOTAL	466 (12,2%)	533 (14%)	6 (0,1%)	5 (0,1%)	1010 (26,4%)	2802 (73,6%)	3812 (100%)

**Conclusion:** In the recent years, the frequency of test requests has increased, since the determination of ANA is more available. It is considered that also requested ANA by other pediatric departments besides the pediatric rheumatology department. Compared to pediatric rheumatology department, other departments have a higher negative test result rates. Clinical findings of patients need to be evaluated in detail while ANA requesting.

**Disclosure of Interest**

None Declared

## Treatment

### B55 OUR MODEST EXPERIENCE WITH INTRAARTICULAR TRIAMCINOLONE HEXACETONIDE IN JUVENILE IDIOPATHIC ARTHRITIS.

#### Almira Ćosićkić^1^, Sanimir -.- Suljendić^2^, Nedim -.- Smajić^1^

##### ^1^Clinic for orthopedics and traumatology; ^2^Department of Paediatric Surgery, University Clinical Centre Tuzla, Bosnia and Herzegovina, Tuzla, Bosnia and Herzegovina

###### **Correspondence:** Almira Ćosićkić

**Introduction:** Although effective in all subtypes of JIA, intraarticular steroid injection is most frequently used in children with arthritis where non-steroidal anti-inflammatory drugs (NSAIDs) have been ineffective in controlling the disease and early in the disease to rapidly resolve synovitis, provide pain relief, facilitate or obviate physiotherapy and rehabilitation, and allow the withdrawal or avoidance of regular systemic treatment. However, intraarticular steroids may be used to treat an arthritis flare in children already maintained on second line agents

**Objectives:** to evaluate the therapeutic response to intraarticular triamcinolone hexacetonide (TH) injections in patients with juvenile idiopathic arthritis

**Methods:** Among 17 patients with JIA (15 oligoarticular, 2 polyarticular) included in the study, 14 girls and 3 boys. Mean age was 7.3 years, age of diseases onset was 5.4±1.4 years. Triamcinolon hexacetonide was administered (1mg/kg body weight ) in the knee joints. Prior to triamcinolon hexacetonide administration 4/17 children recived NSAID, 11/17 NSAID and two children recived anti TNF inhibitor. For most children activity of arthritis was a moderate, mean ESR was 13.4±9.3 mm/h; CRP 22±8.1mg/dl (ref <3.0mg/dl).

**Results:** After administration of TH, the remission of the joint inflammation lasting three months had four children (2 oligoarticular, 2 polyarticular forme of JIA), while complete remission at least 6 months was obtained in 11/17 (64.7%) children. For two children, with oligoarticular arthritis, remission was achieved lasting 13 months after intraarticular TH, those children also recived anti TNF-α inhibitor.

**Conclusion:** Our modest experience has confirmed that the triamcinolone hexacetonide is an effective and safe therapy for inflammatory joint disease in JIA, particularly in the oligoarticular form

**Disclosure of Interest**

None Declared

### B56 BIOLOGICAL TREATMENT IN JUVENILE IDIOPATHIC ARTHRITIS: SWITCH REASONS AND ADVERSE EVENTS

#### Nihal Şahin^1^, Sümeyra Özdemir Çiçek^1^, İsmail Dursun^2^, Muammer H. Poyrazoğlu^1^, Ruhan Düşünsel^1^

##### ^1^Pediatric Rheumatology; ^2^Pediatric Nephrology, Erciyes University Faculty of Medicine, Kayseri, Turkey

###### **Correspondence:** Nihal Şahin

**Introduction:** Biological agents (BA) against proinflammatory cytokines (TNF alpha, IL-6, IL-1), which play a role in juvenile idiopathic arthritis (JIA) pathogenesis have become more frequently used treatment option in recent years. Many adverse events related to BA have been reported, but long term results are not sufficient for pediatric patients. In addition, the switch of BA is required due to many reasons during follow-up.

**Objectives:** This study aimed evaluate the adverse events and switch reasons of biological agents based on patient follow-up data in JIA.

**Methods:** This was a retrospective study in Erciyes University Hospital, Department of pediatric rheumatology, Kayseri, Turkey. In 2008-2017, 115 patients who received biological treatment with JIA were included in the study. Adverse events and switch reasons during the treatment were obtained from the medical records.

**Results:** There were 115 patients treated with BA. 43 patients (37.4%) had enthesitis related arthritis (ERA), 30 patients (26.1%) oligoarthritis, 21 paitents (18.3%) RF negative polyarthritis, 4 patients (3.5%) RF positive polyarthritis, 12 patients (10.4%) systemic JIA, 2 patients (1.7%) juvenile psoriatic arthritis and 3 patients (2.6%) undifferentiated arthritis. Mean age of diagnosis was 9,05±4,56 years. Cumulative follow-up duration was 569,87 years (mean: 4,95±2,82 years, min-max: 0,54-14,21 years) and cumulative follow up on BA was 298,04 years (mean: 2,59±1,77 years, min-max: 0,09-7,46 years). Sixty patients (52.17%) were treated with only one BA. In this patients, etanercept in 26 patients, adalimumab in 28 patients, infliximab in 3 patients, tocilizumab in 3 patients were given as the first BA. Thirty-one patients with inactive disease, 19 patients with active disease were still treated with first BA. Biological agents were stopped in 9 patients treated with one BA (clinical remission: 6, adverse event: 2, lack of effectiveness: 1). Twenty-nine patients had once, 7 patients had twice, 2 patients had three times, 1 patients had five times, 1 patient seven times switches. For all BA switches (total n=61), the reasons were lack of effectiveness in 36 (59%), disease activation in 9 (14.8%), adverse event (AE) in 8 (13.1%), noncompliance to treatment in 6 (9.8%) and macrophage activation syndrome in 2 (3.3%). In follow-up on BA, 0.67 AE/patient-year and 0,18 SAE/patient-year were recorded. Death and malignancy were not seen. Sixty-one percent of the adverse events (n = 122) were infections. Most common infections were respiratory tract system infections (44.7%). Ten local reactions, 4 allergic reactions, 3 anaphylaxes were documented.

**Conclusion:** In JIA, biological agents are switched for many reasons such as lack of effectiveness, disease activation, adverse event and noncompliance to treatment. In our study, most common reason of switch was the lack of effectiveness. Also, many adverse events such as infections are frequently seen during treatment with these agents.

**Disclosure of Interest**

None Declared

## Uveitis

### B57 Withdrawn

## Vasculitides

### B58 PEDIATRIC NEUROBEHÇET DISEASE; SINGLE CENTER EXPERIENCE

#### Ceyhun Acari^1^, Hatice Adiguzel Dundar^1^, Serkan Türkuçar^1^, Ozge Gucenmez^2^, Neslisah Uslu^1^, Erbil Ünsal^1^

##### ^1^Department of Pediatrics Pediatric Rheumatology, Dokuz Eylul University Faculty of Medicine; ^2^Department of Pediatrics Pediatric Rheumatology, Dr.Behçet Uz Children's Hospital, Izmir, Turkey

###### **Correspondence:** Ceyhun Acari

**Introduction:** Behçet Disease (BD) is described as a vasculitis that can affect arteries or veins of any size, and is characterized by recurrent oral and/or genital aphtous ulcers accompanied by cutaneous, ocular, articular, gastrointestinal, and/or central nervous system inflammatory lesions.

BD is extremely rare in pediatric population and children only consist of 5.3-7.6% of all the Behçet patients. Neurologic involvement is 5-15% in pediatric Behçet.

**Objectives:** The aim of study, pediatric Behçet patients with neurologic involvement evaluate that demografic datas and clinical features, nerological finding and treatments.

**Methods:** Four pediatric Behçet patients with neurologic involvement according to International Study Group Criteria who are followed in Dokuz Eylul University, Faculty of Medicine, Pediatric Rheumatology Unit was included in the study.

**Results:** There were 2 female and 2 male patients. The ages were between 11.5 and 14.5 years (average age: 13.4 years) in the time of the diagnosis. The delay in the diagnosis was 10.5 months (1-27 months). Average follow-up time was detected as 41.2 months. Headache was present in all patients, while a patient reported blurring of vision, a patient reported orbital pain and other two patients reported nausea and vomiting. The prediagnosis was pseudotumor cerebri in two patients, thrombophilia in one patient and multiple sclerosis in one patient. Behçet family history and recurrent aphthous lesions in the family were present in one patient. All patients had oral aphthous lesions and one patient had genital aphthous lesions. Three patients presented with papilla edema. Pathergy test was negative in all patients. Lumbar puncture was performed in three patients for diagnostic and therapeutic purposes. HLA B51 was screened in two patients and both were positive. MRI revealed venous sinus thrombosis in three patients, optic nerve thickening was observed in two patients and demyelination plaques were found in one patient without thrombosis. ANA and ANCA tests were found as negative, and thrombosis tests were normal in all patients. All patients were receiving azathioprine, there patients were using steroids and low molecular weight heparin additionally. A patient with severe apthous lesion was using colchicine, another patient was using methotrexate and sulfasalazine due to sacoiliitis Adalimumab was started to patient with unresponsive optic nerve compression and sinus venous thrombosis. While three patients who had thrombosis were followed in the remission clinically and radiologically, but the patient with the parenchymal lesion did not reach to remission. Remission was achieved following 8.6 months in average.

**Conclusion:** Neurological involvement of Behçet disease is rare in the childhood. Neurological involvement is classified in two groups as parenchymal involvement due to inflammatory meningo-encephalitis process and non-parenchymal involvement due to vascular process. While meningo-encephalitis is more common in adult patients, children have cerebral venous thrombosis more commonly. In a study which covers both adult and pediatric cases, thrombosis was reported 88.5% of the pediatric cases, only three cases had parenchymal involvement. Three of the four cases in our study also presented vascular involvement. The same study reported the average diagnosis age as 13.0 for Behçet disease, and 13.5 for NeuroBehçet disease. Our results are similar (average Neuro Behçet diagnosis age: 13.4 years) to this study.

**Disclosure of Interest**

None Declared

### B59 IGA VASCULITIS WITH HEMATEMESIS AND BULLOUS RASH TRIGGERED BY CHLAMYDIA BRONCHITIS

#### Katerina Bouchalova^1^, Tereza Veberova^1^, Lenka Sigutova^1^, Alena Matejova^1^, Lucie Sulovska^1^, Frantisek Kopriva^1^, Kamila Michalkova^2^, Lenka Zbrozkova Bakaj^2^, Dagmar Pospisilova^1^

##### ^1^Department of Pediatrics; ^2^Radiology Department, Faculty of Medicine and Dentistry, Palacky University and University Hospital, Olomouc, Czech Republic

###### **Correspondence:** Katerina Bouchalova

**Introduction:** IgA vasculitis could present rarely with hematemesis. In a proportion of patients IgA vasculitis is preceeded by respiratory infection.

**Objectives:** A report of a 6 year old girl with IgA vasculitis (rash, arthritis, gastrointestinal symptoms including hematemesis) is presented.

**Methods:** case report

**Results:** The girl presented with arthritis, and rash on extremities with history of mild bronchitis. No fever, normal urine and stool were found. Clinical examination revealed palpable purpura on extremities, arthritis of wrists and insteps. Inflammatory markers were elevated (CRP 49 mg/l, leucocytosis 15.6 x10to9/l, neutrophilia 74%, thrombocytosis 493 x10to9/l), and borderline erythrocyturia was present. Further urine tests were normal. Analgetics, rutin plus vitamin C, and rest were recommended. Since the rash worsened, and bullous morphs appeared on instep on day 6, the girl was admitted to the clinic, and topical treatment using betamethasone and gentamycine cream was recommended by dermatologist. Serology revealed active *Chlamydia* sp. infection, thus clarithromycin was added. Later, flebothrombois of right cephalic vein led to initiation of anticoagulation therapy. Antiphospholipid antibodies were negative. Furthermore, gastrointestinal symptoms appeared on day 10, including abdominal pain and mild hematemesis (once). Ultrasonography excluded intussusception, and prednison 1mg/kg/day was prescribed. Her clinical status improved, and corticosteroid detraction is performed at the time of abstract submission (day 34).

**Conclusion:** To our best knowledge, there are only few IgA vasculitis cases with hematemesis described in literature. *Chlamydia species* are frequent causes of respiratory infection. Here, this infection represented a trigger of IgA vasculitis. The treatment using antibiotics and prednisone led to early improvement of clinical status.

Parents agreed with presentation of patient´case, including photodocumentation.

Grants: KB was supported by National Sustainability Programme (LO1304).

**Disclosure of Interest**

None Declared

### B60 TAKAYASU ARTERITIS IN A 13-YEAR-OLD GIRL: WHEN A RARE DISEASE HAS AN ATYPICAL PRESENTATION

#### Raffaella Carlomagno^1,2^, Anne-Lise Hachulla Lemaire^3^, Jean-Paul Vallée^4^, Mehrak Anooshiravani-Dumont^5^, Maurice Beghetti^6^, Igor Leuchter^7^, Alexandra Goischke^8^, Aurélie Wanders^9^, Regula Corbelli^10^, Silvana el Zoghbi^10^, Michael Hofer^1,2^

##### ^1^Pediatric Rheumatology, Centre Hospitalier Universitaire Vaudois (CHUV), Lausanne; ^2^Pediatric Rheumatology, Hôpitaux Universitaires de Genève (HUG); ^3^Radiology, Hopitaux Universitaires de Genève; ^4^Radiology; ^5^Pediatric Radiology; ^6^Pediatric Cardiology; ^7^Pediatric Otorhinolaryngology; ^8^Pediatric Nephrology; ^9^Pediatrics; ^10^Pediatric Pneumology, Hôpitaux Universitaires de Genève (HUG), Geneva, Switzerland

###### **Correspondence:** Raffaella Carlomagno

**Introduction:** Takayasu arteritis(TA) is a large vessels vasculitis characterized by intramural granulomatous inflammation of the pulmonary arteries, aorta and its major branches, leading to wall thickening, stenosis, occlusion and thrombus formation(1). Relapsing polychondritis(RP) is a rare disease characterized by recurrent flares of destructive inflammation of cartilage. To date, only one case of a pediatric patient with recurrent polychondritis and TA has been reported in literature(2).

**Objectives:** To describe an atypical clinical presentation of TA.

**Methods:** Case report.

**Results:** We report the case of a 13-year-old caucasian girl who presented with stridor and respiratory distress at the age of 11. The naso-pharyngo-laryngoscopy showed an isolated subglottic swelling. Infectious researches were negative; no evidence for granulomatosis with polyangiitis nor sarcoidosis was found. She was treated with a one-month course of oral prednisone leading to complete resolution and then a quick tapering of the steroid therapy, with no relapse ever since.

After one uneventful year, she complained of low-grade intermittent fever and transient erythematous rash, with no other symptom. Clinical examination was normal. Blood tests showed a moderate inflammation with elevated CRP, ESR and IgG as well as a slight inflammatory anemia; otherwise complete blood count, blood smear, liver and renal functions and urinalysis were normal; auto-antibodies were negative. Thoracic x-ray, pulmonary fonction, cardiac and abdominal US and large infectious researches were negative. After 10 months of unchanged symptoms, she presented with arterial hypertension and a pathologic Doppler on renal arteries. A thoraco-abdominal CT-scan was performed showing wall thickening, stenosis and enlargement of the descending thoracic and abdominal aorta, renal and mesenteric arteries, the coeliac trunk and lobar and segmental pulmonary arteries. The total body angio-MRI showed similar findings, without involvement of cerebral and cervical arteries. The diagnosis of TA was confirmed and a therapy with steroids, infliximab and methotrexate was started on March 2018.

**Conclusion:** The diagnosis of TA can be very challenging in the absence of specific symptoms, as in our patient. As an early diagnosis can possibly reduce the risk of permanent vascular complications, TA should be considered in the presence of persistent and unexplained biologic inflammation, particularly in adolescent girls.

Regarding the subglottic swelling, its origin is not completely understood, but a non-infectious chondritis seems to be the most reasonable explanation. Even if our patient did not meet the criteria for the RP, a possible association between the chondritis and TA should be considered, as they could both be the consequence of a unique dysimmune process.

Written informed consent obtained from the parents.

1)Aeschlimann et al. Arthritis Res Ther 2017;19:255.

2)Ghosn et al. J Am Acad Dermatol 2008;59:S84-7.

**Disclosure of Interest**

None Declared

### B61 GIANT CORONARY, ABDOMINAL AND ILIAC ANEURYSMS IN INFANT OF 3 MONTHS OF AGE WITH KAWASAKI DISEASE, A CASE REPORT AND LITERATURE REVIEW.

#### Merari E. Gomez Cortes, Edith A. Benitez Vazquez, Velka A. Cortez Lopez, Maria T. Braña Ruiz, Luis Aparicio Vera, Sofia Osorio Sagrero, Enrique Faugier Fuentes, Rocio Maldonado Velazquez

##### Rheumatology, Hospital Infantil de Mexico Federico Gomez, Mexico City, Mexico

###### **Correspondence:** Merari E. Gomez Cortes

**Introduction:** Kawasaki disease is one of the most frequent vasculitis and the main cause of acquired heart disease in childhood; the cause is still unknown and affects mainly children under 5 years old. Timely treatment with intravenous immunoglobulin (IVIG) has succeeded in decreasing the incidence of coronary aneurysms from 25% to approximately 4%, with the risk of developing future thrombosis, stenosis, myocardial ischemia or involution of aneurysms.

**Objectives:** Presentation of the case and treatment initiated in the presence of giant coronary aneurysms as well as systemic aneurysms in a 3-month-old girl.

**Methods:** Description of the clinical case of a patient outside the age range for Kawasaki disease with formation of giant aneurysms of the coronary arteries, iliac arteries and abdominal artery.

**Results:** We present the case of a female patient of 3 months of age, with no relevant history, who comes to our institution for 16 days with fever as well as irritability, treated with antipyretic and multiple antibiotic schemes without clinical response.

At hospital admission, an approach was made to rule out an infectious process, requesting an echocardiogram, which reported: right coronary artery with a 6 x 11mm aneurysm (*z* 11.7) before the bifurcation and right coronary artery with a 4 x 6mm saccular aneurysm (*z* 10.2). Physical examination with fever, conjunctival non-exudative erythema, cleft lip, generalized maculopapular rash and BCGitis, which integrates the definitive diagnosis. Subsequently, management was started with intravenous immunoglobulin 2 g/kg, methylprednisolone bolus 30 mg/kg, acetyl salicylic acid 80 mg/kg and enoxaparin 1 mg/kg/ per dose. The control echocardiogram reported a decrease in the right coronary aneurysm, which was now medium but an increase in the giant aneurysm of the left coronary artery, added to the increase of inflammatory markers, we decided a second dose of IVIG followed by 7 days with full dose of prednisone (2 mg/kg).

Angiotomography was performed three days later reporting fusiform aneurysms in the abdominal artery and common iliac arteries but without fever and normalization of C-reactive protein (CRP), so decided decrease of acetyl salicylic acid to 4 mg/kg, continue with anticoagulation and hospital discharge.

**Conclusion:** The incidence of systemic aneurysms is not well described with series that report between 0.6 to 2.5%, developing giant aneurysms up to 10%, and an age range of 1 to 20 months with an average of 6 months as the case of our patient. The sites mainly affected are brachial artery, common iliac artery, internal iliac artery, subclavian artery, axillary artery, lateral thoracic artery, femoral artery, and renal artery. Informed consent to publish had been obtained from the parent.

**Disclosure of Interest**

None Declared

### B62 REPORT OF A CHILD DIAGNOSED WITH WEGENER’S DISEASE WHO PRESENTED WITH PURPURA AND ARTHRITIS

#### Shabnam Hajiani, Reza Shiari, Mohsen Jari

##### SBMU, tehran, Iran, Islamic Republic Of

###### **Correspondence:** Shabnam Hajiani

**Introduction:**

**Background: Granulomatosis with polyangiitis (GPA) or Wegener's granulomatosis (WG) is a chronic systemic vasculitis involving small to medium sized arteries characterized by granulomatous inflammation of the upper and lower airways, necrotizing and pauci-immune glomerulonephritis and vasculitis that affects many other vital organs. Clinical manifestations are inflammation of upper and lower respiratory tract and renal involvement.**

**Objectives:** Patient is an 11-year-old boy complaining of fever, pain and swelling of the ankles and ecchymotic skin lesions on the legs and buttock, and purpuric lesions resembling erythema multiform on the elbows and dorsal side of forearms.One day after admission, petechiae presented on the surface of his mouth and patient had hemoptysis for several times. Microscopic hematuria was detected in urine analysis. The next day due to massive hemoptysis, evidence of pulmonary hemorrhage in CT scan of the lungs and patient’s respiratory distress, he was candidate to be admitted in the ICU. According to the presentation and physical examination of the patient and C-ANCA titer which has been reported more than 300, the diagnosis of AAV (Wegener) was made with Printo’s criteria.

**Methods:**

case report

**Results:** case report

**Conclusion:** This case was an 11-year-old boy presenting with purpuric rashes on the limbs and arthritis who has been diagnosed with WG by the result of C-ANCA titer and ruling other diagnosis out such as infectious diseases, immune disorders, neoplastic disorders, Anti GBM disease, ANCA positive ulcerative colitis and other autoimmune diseases. Patient has been treated with methylprednisolone pulse for three days with 30mg/Kg/day dosage and after that cyclophosphamide pulse and 2mg/Kg/day of prednisolone as maintenance therapy. Informed consent for publication had been obtained.

**Disclosure of Interest**

S. Hajiani Employee of: i"m fellow ship now and any affilation was not publicate by me , R. Shiari: None Declared, M. Jari: None Declared

### B63 APPENDICITIS AS A RARE PRESENTATION OF KAWASAKI DISEASE

#### Shima Salehi^1^, Shirin Sayyahfar^2^, Samira Mehralizadeh^3^, Mohammad R. goudarzi^4^

##### ^1^Pediatric Rheumatology; ^2^Pediatric infectious speciality; ^3^Pediatric Cardiology, Ali-asghar Children's Hospital,Iran University of Medical Siences; ^4^Pediatric cardiology, Hazrate Rasool General Hosputal, Iran University of Medical Siences, Tehran, Iran, Islamic Republic Of

###### **Correspondence:** Shima Salehi

**Introduction:** We present a 4 years old girl with prolonged fever who was diagnosed as perforated appendicitis at the same time with coronary artery dilation as a complication of Kawasaki disorder

**Objectives:** Atypical presentations of the kawasaki disease can pose a diagnostic dilemma. The prevalence of incomplete or atypical disease that do not meet the classical presentation of the disease is relatively high(13.8 percent in Japan). It is important to know other significant, though not diagnostic, findings that raise a suspicion of the disease in absence of the classical criteria.

**Methods:** Our patient is a 4 years old girl with no prior positive medical history who presented to our emergency department with prolonged fever and a febrile seizure on the first day of her feve.Three days after the fever began she started having generalized colicky abdominal pain .Her initial lab results showed a WBC count of 19500 per mm3 with 73% Neutrophils, a hemoglobin of 9.2 with normal MCV and MCHC, normal platelet count and an elevated ESR and CRP at 81 and 116 respectively. Serum Albumin and liver function tests were normal.

**Results:** To diagnose the source of her prolonged fever an echocardiogram and abdominal ultrasound was ordered. Abdominal ultrasound showed a collection in the right lower quadrant compatible with a diagnosis of perforated appendicitis. Echocardiogram showed dilation of LAD with a Z score of 4(small aneurysm).CT Angiography of abdominal aorta and femoral arteries revealed no dilation or narrowing.

**Conclusion:** upto now just two cases similar to ours has been previously described. Our case provides the third example of this extremely rare presentation of the disease.In every person with prolonged fever and abdominal pain vasculitides like kawasaki disease must be ruled out. Informed consent to publish had been obtained.

**Disclosure of Interest**

None Declared

### B64 CLINICAL MANIFESTATIONS, THERAPEUTIC APPROACH, COMPLICATIONS AND OUTCOME AT CHILDREN WITH KAWASAKI DISEASE

#### Barbara Stanimirovic^1^, Dario Djukic^2^, Olivera Ljuboja^3^

##### ^1^Pediatric rheumatology; ^2^Pediatric Cardiology; ^3^Pediatric Pulomology, University Clinical center Republic of Srpska, Banjaluka, Bosnia and Herzegovina

###### **Correspondence:** Barbara Stanimirovic

**Introduction:** Kawasaki disease is an acute vasculitis characterized by fever, bilateral non purulent conjunctivitis, erythema of the mouth, rash and cervical lymphadenopathy. In 15 to 20% of untreated children, ectasia or coronary artery aneurysms may develop. The most difficult complications are ischemic heart disease and sudden death.

**Objectives:** The aim of this study is to describe clinical manifestations, laboratory investigations, therapeutic approach, complications and outcome in children with Kawasaki disease.

**Methods:** Medical records of all children with Kawasaki disease during 8 years period (between 2009 and 2017) at the Pediatric Clinic Banja Luka were retrospectively reviewed. 20 children, 12 girls and 8 boys were analyzed.

**Results:** The youngest child was 4 months old, and the oldest child was 7 years old. The most common clinical manifestations were: fever 20/20(100%), rush 15/20 (75%), conjunctivitis 12/20 (60%), lips and buccal mucosa hyperaemia 13/20(65%), gastrointestinal 9/20(45%), cervical lymphadenopathy 8/20 (40%) and renal involvement 8/20 (40%). In 8/20 ( 40%) of patients had coronary ivovement. Coronary aneurysm occurred in 5/20( 25 %) of children. 15/20 (75% ) of children had elevated parameters of inflammation. All 20 children were treated with i.v. immunoglobulins (IG) with acetyl salicylic acid. In 2 children, a second dose of IG and methylprednisolone were administered. Two patients required administration of Anti-TNF alpfa monoclonal antybodies-infliximab, given as a single dose, with favourable outcome. During follow-up period 4 children ( 20%) had persistent aneurysmal enlargement of the coronary arteries. During the study period no death was recorded.

**Conclusion:** In eight children 8/20 (40%) who developed coronary involvement , the therapy was started late, after the seventh day of the onset of the disease. The most complicated patient was 4 months old infant who received therapy at the latest from all patients (Day 13). There was no significant difference in clinical manifestations, laboratory results, therapeutic approach and outcome in children with Kawasaki disease compared to other studies.

**Disclosure of Interest**

None Declared

